# Noncovalent Interactions in the Oxazaborolidine-Catalyzed Enantioselective Mukaiyama Aldol

**DOI:** 10.1021/acs.joc.2c01039

**Published:** 2022-07-18

**Authors:** Elliot H. E. Farrar, Matthew N. Grayson

**Affiliations:** Department of Chemistry, University of Bath, Claverton Down, Bath BA2 7AY, U.K.

## Abstract

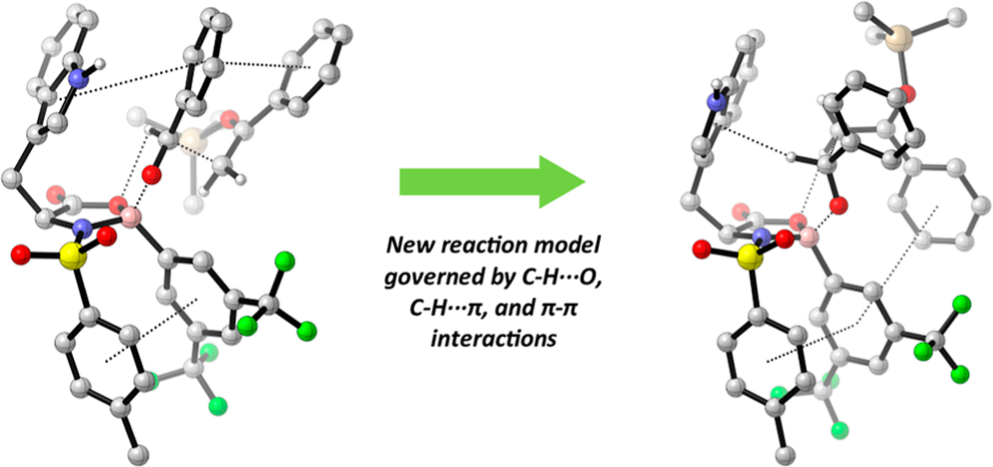

Current models for oxazaborolidine-catalyzed transition-state structures are determined by C–H···O–B and C–H···O=S formyl hydrogen bonding between the electrophile and catalyst. However, selectivity in the oxazaborolidine-catalyzed Mukaiyama aldol cannot be fully rationalized using these models. Combined density functional theory and noncovalent interaction analyses reveal a new reaction model relying on C–H···O, C–H···π, and π–π interactions between the nucleophile, electrophile, and catalyst to induce selectivity.

## Introduction

**Figure 1:**
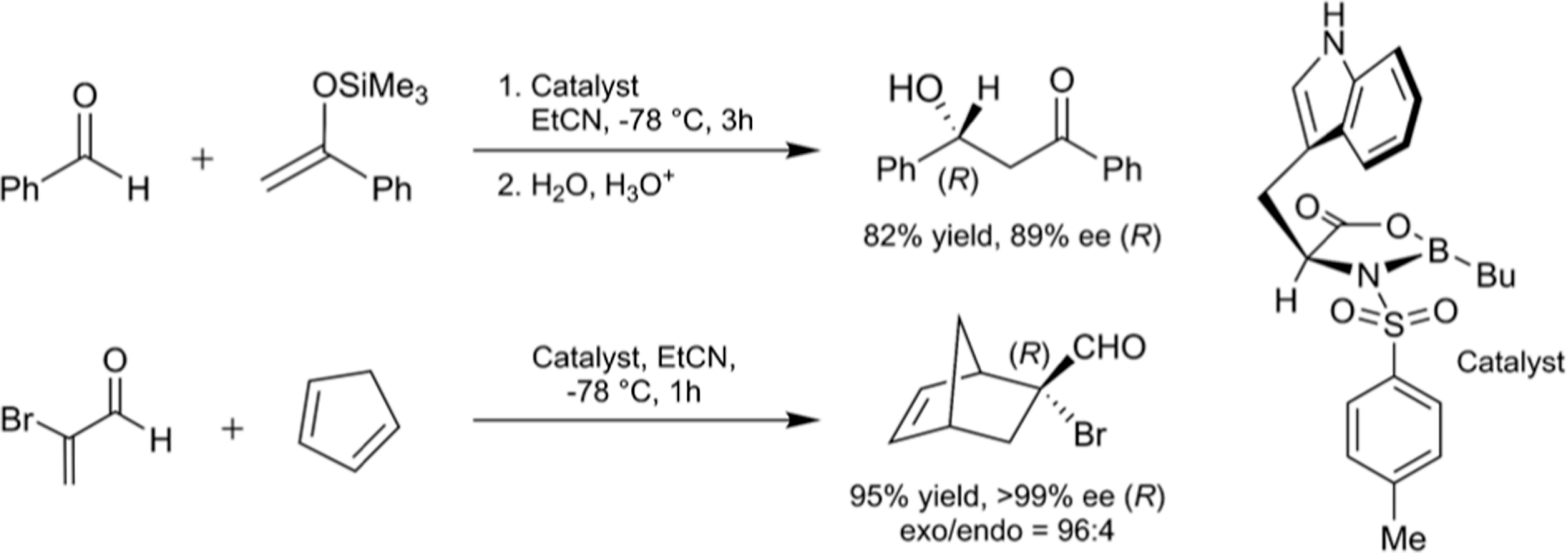
Selective NTOB-catalyzed Mukaiyama aldol and Diels–Alder reactions.

The Mukaiyama aldol and Diels–Alder reactions are two of the most synthetically useful C–C bond-forming methods in modern chemistry, facilitating the addition of structural and stereochemical complexity to chemical systems.^1,2^*N*-Sulfonylated oxazaborolidinones have long been a popular catalyst in these two important reaction classes; original valine-derived variations have been used in both aldol^3−7^ and Diels–Alder^8−14^ reactions to achieve high enantioselectivities and diastereoselectivities. Similar results have also been obtained with *N*-sulfonylated tryptophan-derived oxazaborolidinone (NTOB) catalysts in aldol^15,16^ and Diels–Alder^17−19^ reactions (Figure 1), finding use in a variety of natural product syntheses.^20−23^

**Figure 2:**
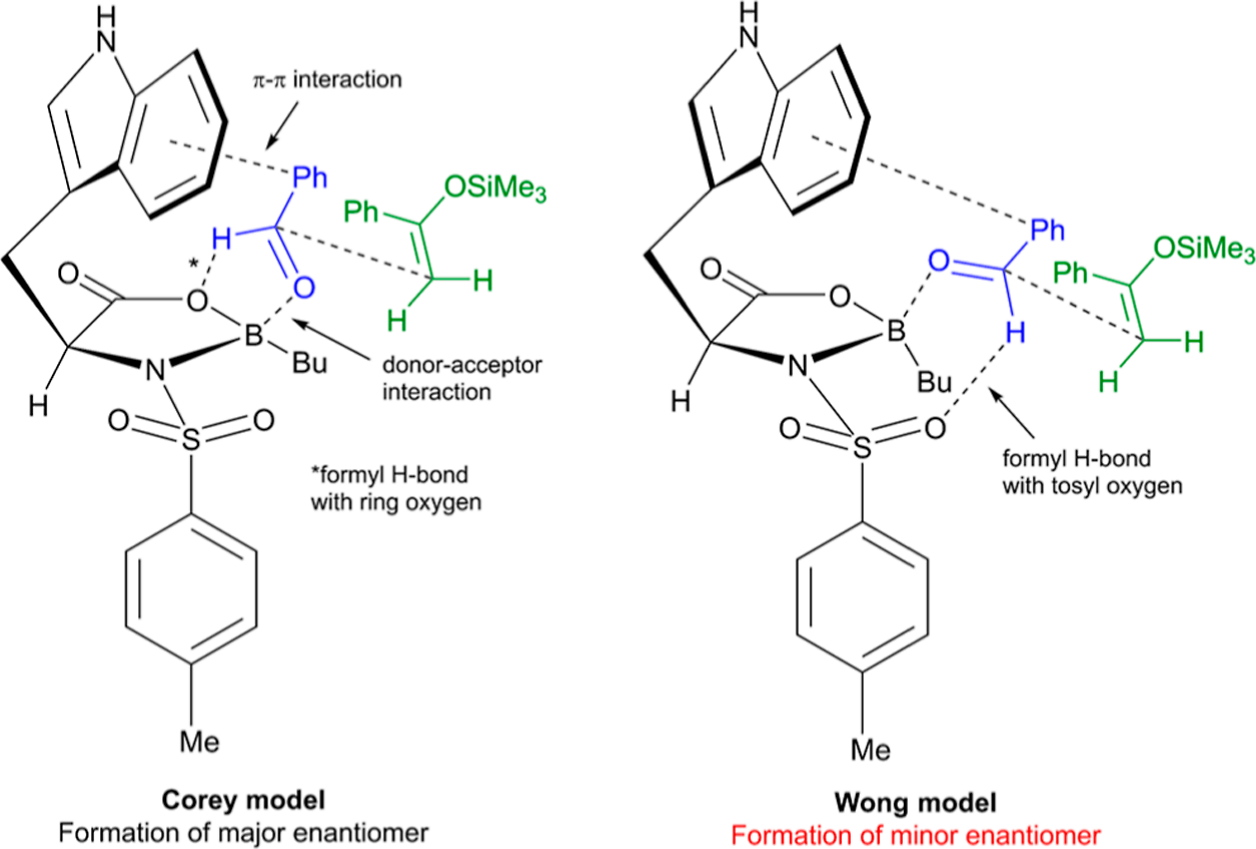
Corey and Wong models applied to the NTOB-catalyzed Mukaiyama aldol reaction.

Selectivity in the NTOB-catalyzed Mukaiyama aldol was first rationalized by Corey^15,18^ (Figure 2) on the basis of three major interactions: a donor–acceptor interaction between the carbonyl oxygen of the aldehyde and electron-deficient boron of the catalyst,^9^ a stabilizing nonclassical C–H···O–B hydrogen bonding interaction between the formyl hydrogen of the aldehyde and the ring oxygen of the oxazaborolidinone catalyst,^24−26^ and an attractive π–π interaction between the aldehyde and electron-rich indole. Previous density functional theory (DFT) studies have found nonclassical hydrogen bonding interactions^27^ to be vital to inducing selectivity in a variety of organocatalyzed reaction types.^28−31^ Together, these interactions result in a rigid transition-state (TS) structure complex where one face of the aldehyde is blocked by the steric bulk of the catalyst, resulting in preferential nucleophilic attack from the opposite face of the aldehyde and consequently high enantioselectivity.

In 2005, Wong proposed a similar model except with the C–H···O interaction forming with an S=O oxygen of the *N*-sulfonyl group, rather than the ring oxygen (Figure 2).^32^ DFT and *ab initio* calculations found this to be the most electron-rich region of the NTOB and thus the superior hydrogen bond acceptor. As a result of this alteration, the opposite face of the aldehyde is left exposed to nucleophilic attack, corresponding to a prediction of inverted enantioselectivity. For the Diels–Alder reaction, this is accounted for by a preference of the enal nucleophile to adopt an *s*-trans conformation, conserving the correct overall sense of selectivity. However, no such caveat can be made for the Mukaiyama aldol, for which the Wong model appears to predict the incorrect product enantiomer. Accordingly, Corey’s model continues to be implicated in NTOB-catalyzed Mukaiyama aldol reactions in the literature.^16,21,33^

**Figure 3:**
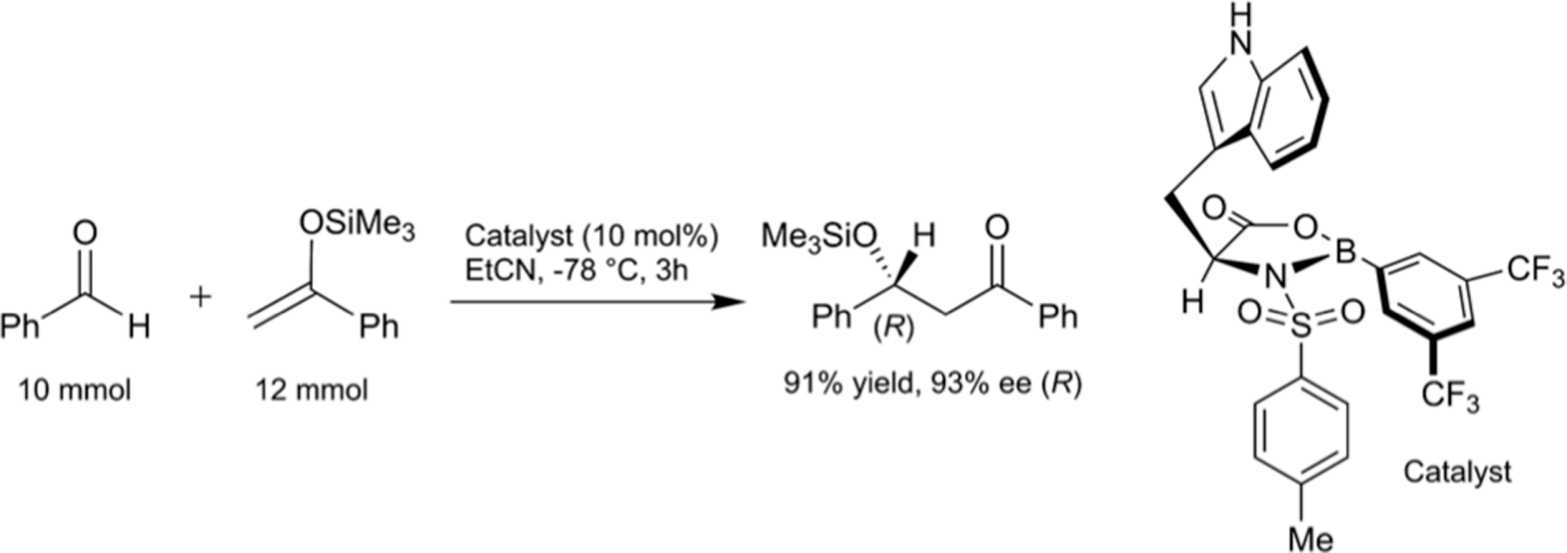
Chosen reaction conditions for computational analysis.

Herein, we report the results of a thorough computational analysis of the full TS complex for an NTOB-catalyzed Mukaiyama aldol using a trimethylsilyl enol ether derived from acetophenone (Figure 3).^16^ Conformational searching was performed using Schrödinger’s MacroModel (version 11.6)^34,35^ and subsequent DFT and natural bond orbital (NBO) analyses using Gaussian 16 (Revision A.03).^36^ Computed structures were illustrated with CYLView.^37^ Noncovalent interaction (NCI) analyses were performed using the NCIPLOT^38^ program and illustrated using VMD.^39^ Full details of the conformational searching and computational methods are provided in the Supporting Information. These analyses reveal a new reaction model, distinct from the Corey and Wong models, which involves a series of noncovalent C–H···O, C–H···π, and π–π interactions (defined in the Supporting Information). Selectivity through this model is validated on several sets of experimental conditions.

## Results and Discussion

**Figure 4:**
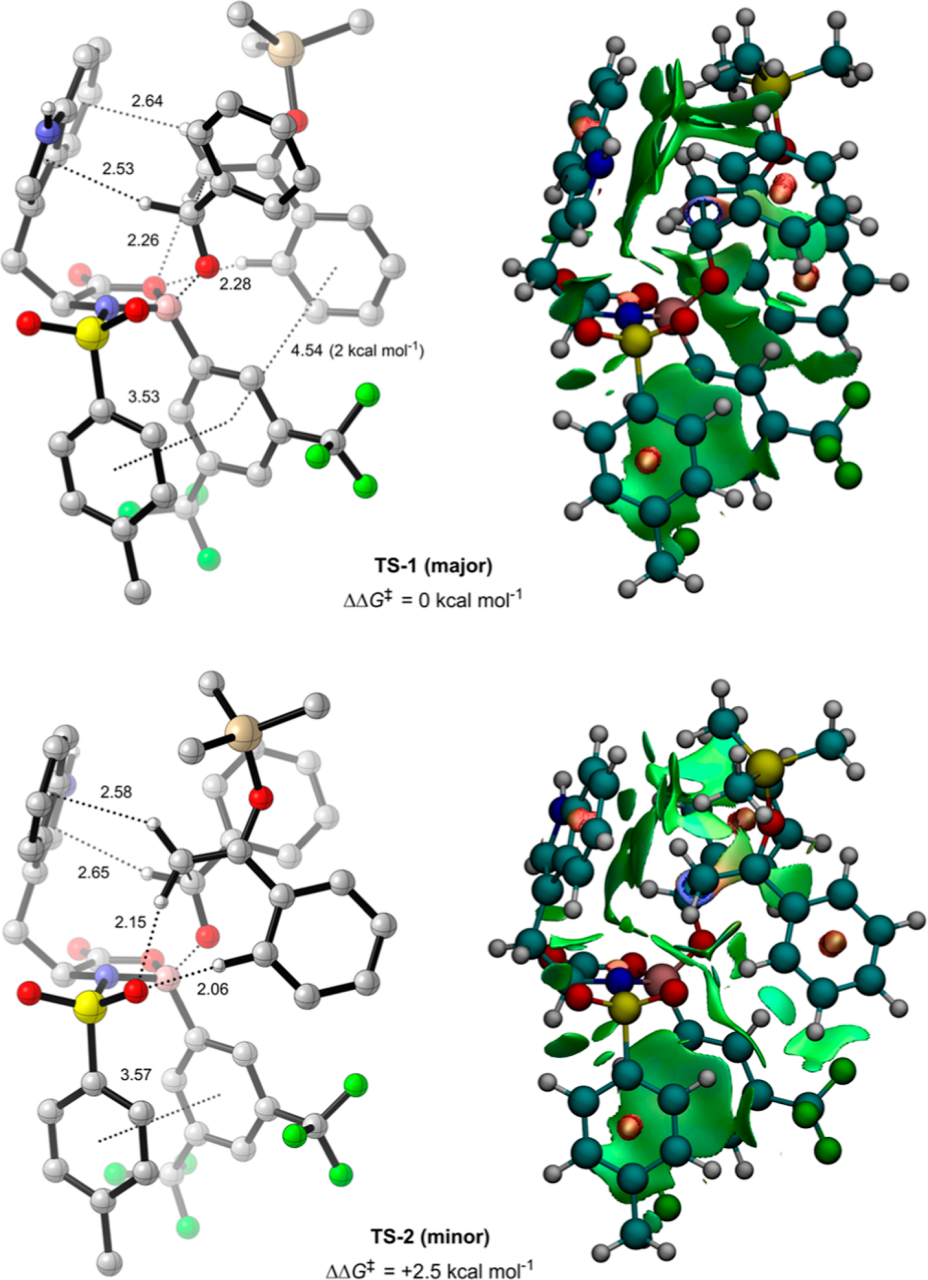
Lowest free energy major (**TS-1**) and minor (**TS-2**) TSs for the NTOB-catalyzed Mukaiyama aldol (B3LYP-D3(BJ)/def2-TZVPP/IEFPCM(propanonitrile)//B3LYP-D3(BJ)/6-31G(d,p)) with NCI distances in angstroms (Å) and key NBO interaction strengths. π-interactions measured from the relevant ring centroid. Green, red, and blue NCI surfaces represent weak, strong repulsive, and strong attractive NCIs, respectively.

A total of 328 unique TSs were obtained for the NTOB-catalyzed Mukaiyama aldol, of which 47 selected low-energy conformers were reoptimized at a higher level of theory (full details in the Supporting Information). **TS-1**, the lowest-energy major TS, is found to be 2.5 kcal mol^–1^ lower in energy than **TS-2**, the lowest-energy minor TS (Figure 4). Thus, based on a Boltzmann weighting at 195.15 K over all conformers within 3 kcal mol^–1^ of **TS-1**, a computed *ee* of >99% is predicted, in good agreement with the experimental *ee* of 93%. These results were validated at several levels of DFT (Table S3).

**Figure 5:**
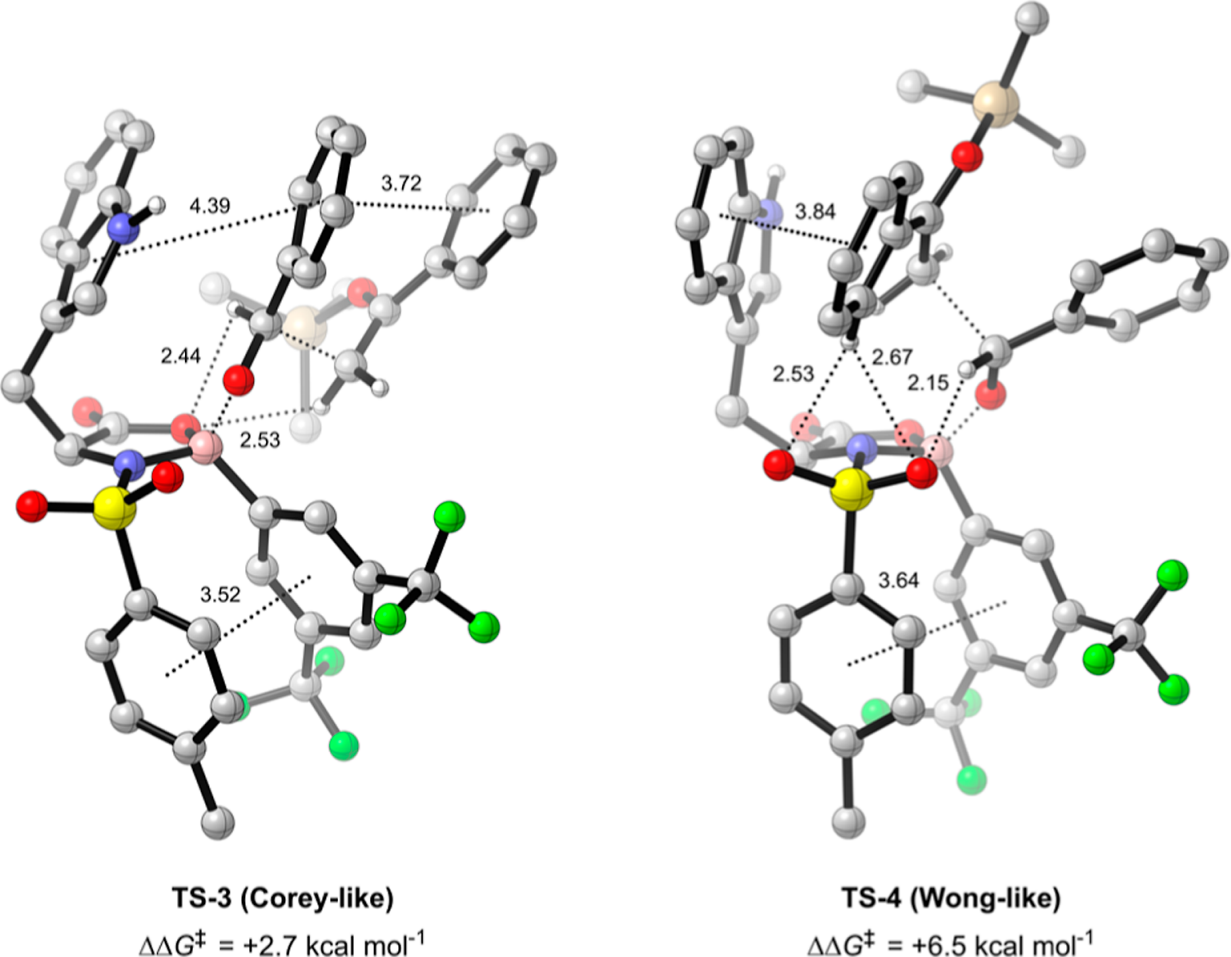
Lowest free energy Corey (**TS-3**) and Wong-type (**TS-4**) TSs, relative to **TS-1**, for the NTOB-catalyzed Mukaiyama aldol (B3LYP-D3(BJ)/def2-TZVPP/IEFPCM(propanonitrile)//B3LYP-D3(BJ)/6-31G(d,p)) with NCI distances in angstroms (Å). π-interactions measured from the relevant ring centroid. Full NCI analysis of **TS-3** and **TS-4** provided in Figure S10.

Like the Corey and Wong models, the aldehyde binds the catalyst at two points in **TS-1** and **TS-2**. However, while a donor–acceptor interaction is present between the carbonyl oxygen of the aldehyde and the boron of the catalyst, the formyl hydrogen is found to interact with the five-membered ring of the electron-rich indole via a C–H···π interaction,^40^ rather than forming a C–H···O interaction with either the ring oxygen or an *N*-sulfonyl oxygen. Instead, the ring oxygen in **TS-1** and an *N*-sulfonyl oxygen in **TS-2** are involved in nonclassical C–H···O hydrogen bonding with the vinyl and ortho-phenyl hydrogens of the silyl enol ether nucleophile. Enal-catalyst vinyl interactions were considered briefly by Corey in the oxazaborolidinium-catalyzed Diels–Alder.^41^ Additionally, a weak C–H···π interaction is present between a vinyl hydrogen of the silyl enol ether and the six-membered ring of the indole, fixing the indole into one of two conformations, depending on which face the silyl enol ether is bound; analogous structures to **TS-1** and **TS-2** with the indoles in their opposite conformations are both higher in energy than their respective counterparts (Figure S9). Thus, several important noncovalent C–H···O, C–H···π, and π–π interactions between the nucleophile, electrophile, and catalyst contribute to the overall stability of **TS-1** and **TS-2**, lowering their free energy compared to the Corey and Wong models. Accordingly, **TS-3** and **TS-4**, the lowest-energy major TSs located representing Corey and Wong-like binding, respectively, are found to be 2.7 and 6.5 kcal mol^–1^ higher in energy than **TS-1** (Figure 5). Although some nucleophile–catalyst interactions are present in **TS-3** and **TS-4**, they are fewer and longer than in **TS-1** and **TS-2**, contributing to their higher relative energies. Additionally, the nucleophilic binding in **TS-4** results in steric clashing between the silyl enol ether and indole, raising the energy of this TS further.

Since **TS-1** and **TS-2** share the same mode of electrophilic binding to the catalyst, but with distinct nucleophilic bindings, differences between the two TSs are most likely a consequence of the positioning of the nucleophile and any subsequent interactions that occur as a result. Thus, the origins of selectivity are much less apparent than in the Corey or Wong models, where one face of the aldehyde is blocked by the steric bulk of the indole in only the major TS. Indeed, no H–H contacts between the substrate and catalyst within 90% of the van der Waals radii are found in **TS-1** or **TS-2**, confirming that steric factors are not a major factor in selectivity.

As evidenced from **TS-1** and **TS-2**, nucleophilic attack is preferred when the silyl enol ether binds the backside of the catalyst–aldehyde complex. NCI analyses on **TS-1** and **TS-2** (Figure 4) reveal the importance of a further class of interactions occurring in both systems, π–π interactions.^42^ Such interactions are common in many organic and biological systems^43,44^ and have been rationalized on the basis of direct through-space interactions between the polarized substituents and closest region of the complementary aromatic system (Figure S3b).^45−47^ Indeed, the formation of attractive π–π interactions between the aromatic aldehyde and the electron-rich indole was an important element of Corey’s original model.^25^ However, in **TS-1** and **TS-2**, the aldehyde is positioned perpendicular to the indole, removing the possibility of such interactions between these components. Nonetheless, both **TS-1** and **TS-2** possess face-centered π–π interactions between the *N*-sulfonyl aromatic and the electron-deficient boron substituent of the catalyst. Additionally, in **TS-1**, there is a significant interaction of approximately 2 kcal mol^–1^ (calculated by NBO analysis) between the electron-deficient aromatic boron substituent and the electron-rich phenyl of the silyl enol ether. This interaction is geometrically impossible in **TS-2** due to the positioning of the silyl enol ether. This arrangement of overlapping aromatics results in significant stabilization of **TS-1**, lowering its free energy with respect to **TS-2** and inducing selectivity in the reaction. These findings are in line with previous quantum chemical analyses on the oxazaborolidinium-catalyzed cycloadditions of maleimides, which found that NCIs between the substrate and aromatic catalyst were vital to inducing selectivity and that the importance of nonclassical C–H···O hydrogen bonding had been overstated by the previous models.^48^

**Figure 6:**
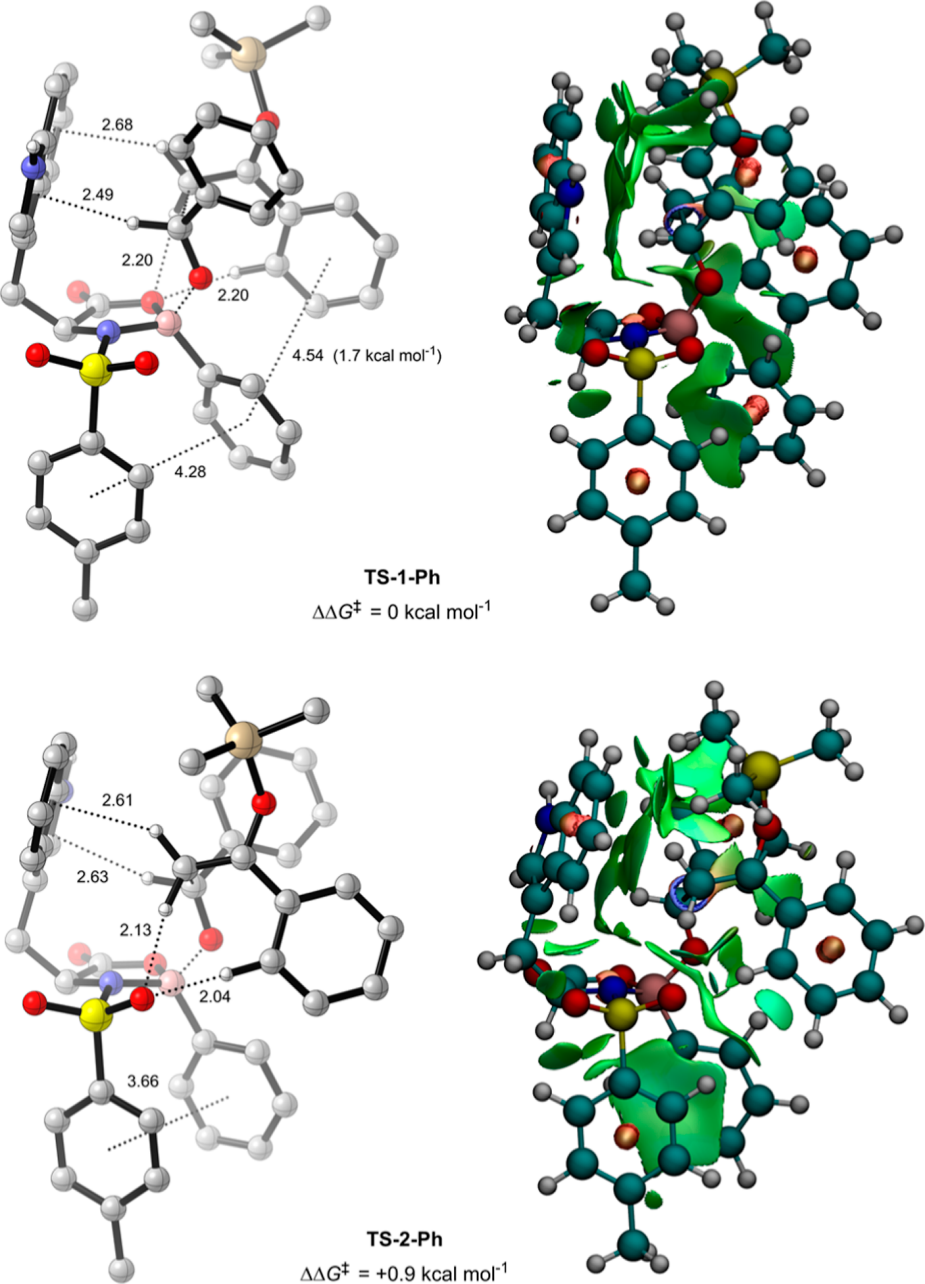
Reoptimized phenyl-substituted conformations of **TS-1** (**TS-1-Ph**) and **TS-2** (**TS-2-Ph**) (B3LYP-D3(BJ)/def2-TZVPP/IEFPCM(propanonitrile)//B3LYP-D3(BJ)/6-31G(d,p)) with NCI distances in angstroms (Å) and key NBO interaction strengths. π interactions measured from the relevant ring centroid. Green, red, and blue NCI surfaces represent weak, strong repulsive, and strong attractive NCIs, respectively.

To further assess the impact of these nucleophile–catalyst π–π interactions on selectivity, additional calculations were performed with alternative boron substituents. First, **TS-1** and **TS-2** were reoptimized with the boron substituent replaced by a nonsubstituted phenyl group (Figure 6). Consequently, the free energy difference between the structures dropped to 0.9 kcal mol^–1^, corresponding to a computed *ee* of 81% based on a Boltzmann weighting at 195.15 K between **TS-1-Ph** and **TS-2-Ph**, in excellent agreement with the experimental value of 79%.^16^ Importantly, this trend makes chemical sense with respect to our reaction model and is supported by both NCI and NBO analyses on **TS-1-Ph** and **TS-2-Ph**; the removal of the electron-withdrawing CF_3_ groups from the boron substituent weakens the π–π interactions between itself and the electron-rich phenyl of the silyl enol ether in **TS-1-Ph**, which are now calculated at 1.7 kcal mol^–1^ (compared to 2 kcal mol^–1^ in **TS-1**), while the same interaction remains impossible in **TS-2-Ph**. As a result, **TS-1-Ph** increases in free energy relative to **TS-2-Ph**, and selectivity becomes poorer.

**Figure 7:**
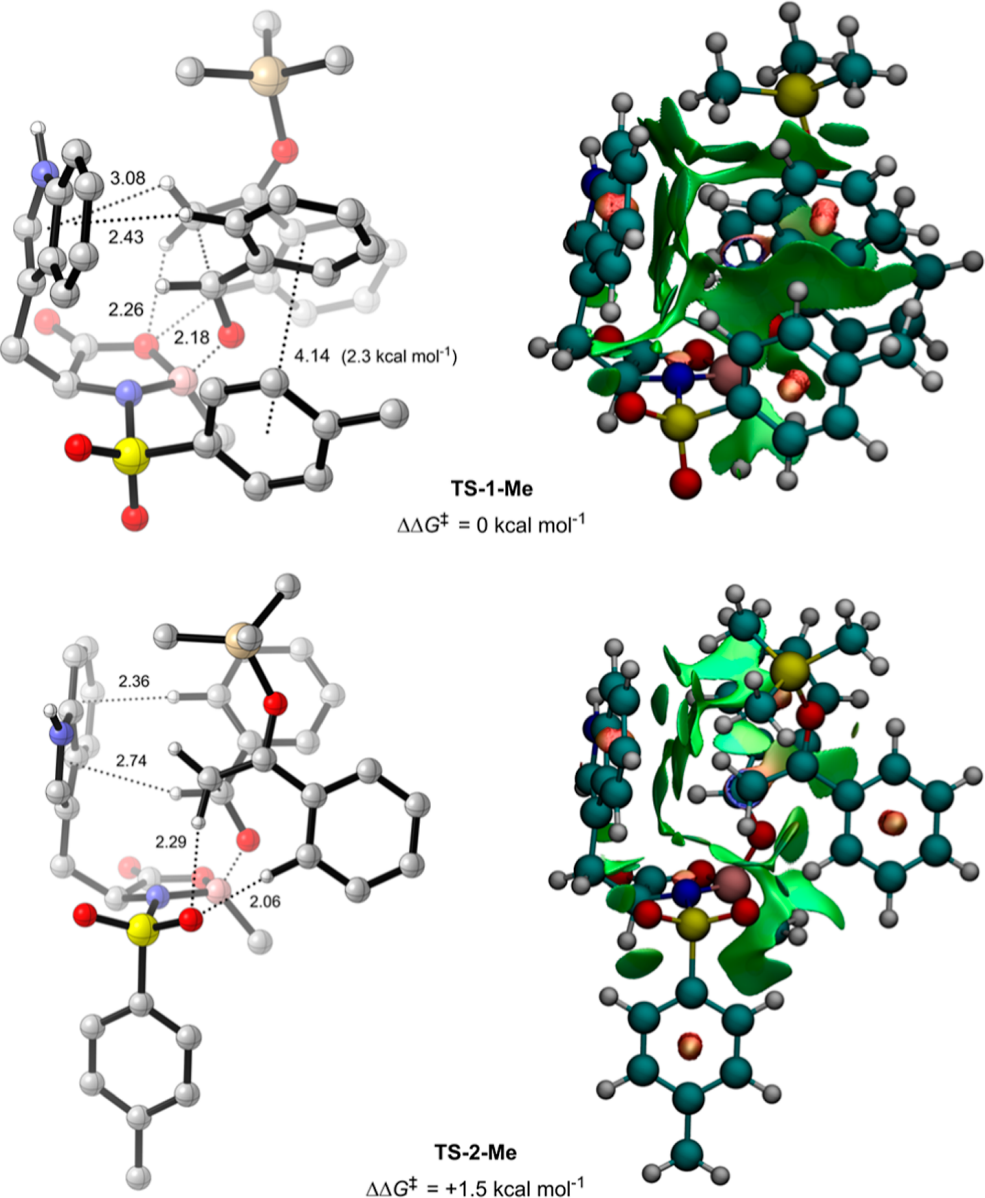
Lowest free energy major (**TS-1-Me**) and minor (**TS-1-Me**) TSs for the methyl-substituted NTOB-catalyzed Mukaiyama aldol (B3LYP-D3(BJ)/def2-TZVPP/IEFPCM(propanonitrile)//B3LYP-D3(BJ)/6-31G(d,p)) with NCI distances in angstroms (Å) and key NBO interaction strengths. π-interactions measured from the relevant ring centroid. Green, red, and blue NCI surfaces represent weak, strong repulsive, and strong attractive NCIs, respectively.

The aromatic boron substituent was then replaced with a methyl group, as an approximation for the *n*-butyl used in the experimental work, and a new conformational analysis was performed. A total of 74 unique TSs were obtained, of which 25 selected low-energy conformers were reoptimized at a higher level of theory (full details in the Supporting Information). In contrast to previous systems, no π–π interactions could form between the nonaromatic boron substituent and either the silyl enol ether or *N*-sulfonyl group. Nevertheless, selectivity is preserved in both experiment and DFT; **TS-1-Me** (Figure 7), the lowest-energy major TS, is found to be 1.5 kcal mol^–1^ lower in energy than **TS-2-Me**, the lowest-energy minor TS, corresponding to a computed *ee* of 93% based on a Boltzmann weighting at 195.15 K over all conformers within 3 kcal mol^–1^ of **TS-1-Me**, in excellent agreement with the experimental *ee* of 82% (R = *n*-butyl).^16^ In lieu of π–π interactions between the silyl enol ether and boron substituent, the *N*-sulfonyl group in **TS-1-Me** is able to direct itself toward the top face of the oxazaborolidinone ring, allowing a π–π interaction with the phenyl of the aldehyde of approximately 2.3 kcal mol^–1^ (calculated by NBO analysis). This kind of interaction is not geometrically possible in **TS-2-Me**, where the alternative binding of the silyl enol ether sterically screens the *N*-sulfonyl group from occupying any position on the top face of the ring, resulting in its higher free energy. With the exclusion of π–π interactions, **TS-2-Me** otherwise shares the reaction model as **TS-2**. Thus, selectivity is preserved, despite the lack of an aromatic boron substituent.

## Conclusions

In conclusion, our computational analyses reveal a new reaction model for the NTOB-catalyzed Mukaiyama aldol, which matches experimental selectivity and is validated on systems with less polarized and nonaromatic boron substituents. While previous models focused on interactions between the catalyst and electrophile, nucleophile–catalyst interactions are found to be vital in stabilizing the TS complex and inducing selectivity. A variety of nonclassical C–H···O, C–H··· π, and π–π interactions are important in the model, while traditional formyl C–H···O interactions, such as those in the Corey and Wong models, are absent. Selectivity is rationalized by the presence of π–π interactions in the major TSs that are not geometrically possible in the minor TS due to the direction of nucleophilic binding.

## Supporting Information Available

Full computational methods, interaction definitions, additional reactant state and TS analyses, complete list of all energies, frequencies, and molecular geometries (in Cartesian coordinates) for all computed structures (generated by EsiGen software)^49^ (PDF)
