# Supplementary material for: Noncovalent Interactions in the Oxazaborolidine-Catalyzed Enantioselective Mukaiyama Aldol
Source: J Org Chem. 2022 Jul 18;87(15):10054–61. doi: 10.1021/acs.joc.2c01039 (PMC9361351; doi:10.1021/acs.joc.2c01039)
Supplement: Supplementary file 1 — jo2c01039_si_001.pdf [file jo2c01039_si_001.pdf]

Noncovalent Interactions in the Oxazaborolidine-Catalyzed Enantioselective Mukaiyama Aldol

Supporting Information

*Elliot H. E. Farrar and Matthew N. Grayson**

Department of Chemistry, University of Bath, Claverton Down, Bath, BA2 7AY, United Kingdom

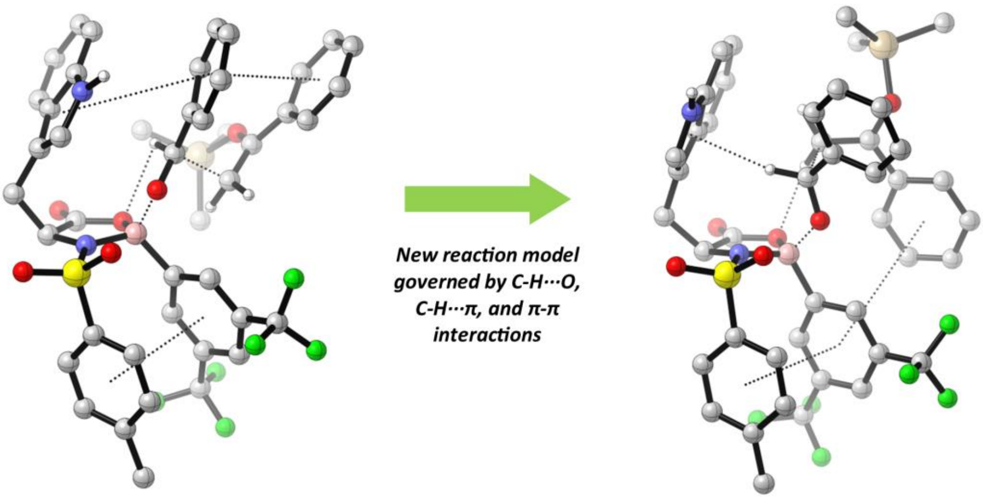

Table of Contents

1. **Computational methods** S2
2. **Interaction definitions** S4
3. **Analysis of ground state structures** S6
4. **Analysis of NTOB-aldehyde complex** S7
5. **Analysis of transition-state complex** S9
6. **Geometries and energies for all computed structures** S12
7. **References** S517

1. Computational methods

Conformational searches were carried out on the isolated nucleophile (acetophenone-derived trimethylsilyl enol ether), electrophile (benzaldehyde), an *N*-sulfonylated tryptophan-derived oxazaborolidinone (NTOB) catalyst, the NTOB-aldehyde complex, and two Mukaiyama aldol transition-state structure (TS) complexes with Me and 3,5-$(CF_3)_2C_6H_3$ boron substituents (Figure S1) using the conformational search tool within Schrödinger's MacroModel (version 11.6)[1,2] with the OPLS2005 force field.[3] A mixed Monte Carlo Multiple Minimum (MCMM) / low-mode sampling approach was used to explore the possible conformations of each molecule or complex.[4–6] Conformational searching of the NTOB-aldehyde complex and both Mukaiyama aldol TS complexes were performed with the C=O of the electrophile bound to the boron of the NTOB catalyst. Many unique conformers were obtained for each molecule or complex (Table S1), among which several distinct binding modes were represented (see sections 4 and 5). Thus, a thorough sampling of the conformational space was performed for each reaction.

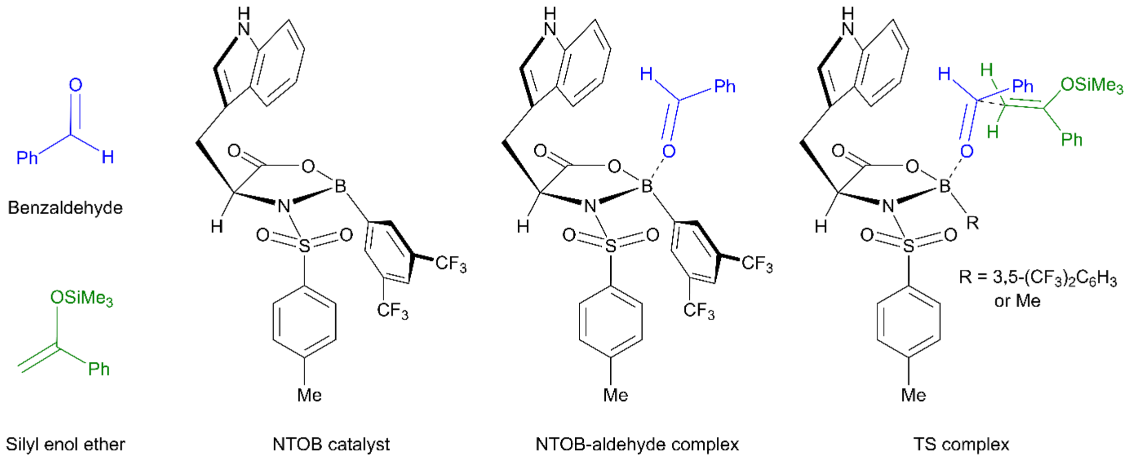

Figure S1. Conformational searches were performed for six distinct chemical species. Constraints indicated by dashed lines.

Species	B3LYP/6-31G(d) Conformers	Selected Conformers for Reoptimization	B3LYP-D3(BJ)/6-31G(d,p)) Conformers
Benzaldehyde	1	All Conformers	1
Silyl enol ether	3	All Conformers	3
NTOB catalyst	14	All Conformers	14
NTOB-aldehyde complex	124	Conformers within 6 kcal mol^{-1} of lowest	29
TS complex (R = 3,5-$(CF_3)_2C_6H_3$)	328	Conformers within 5 kcal mol^{-1} of lowest, plus the lowest Wong-like major and minor TS	47
TS complex (R = Me)	74	Conformers within 5 kcal mol^{-1} of lowest	25

Table S1. Selected low-energy conformers were reoptimized at a higher level of theory after optimization with DFT.

Conformations provided by these searches were subsequently optimized by DFT[7,8] calculations carried out using Gaussian16 (Revision A.03)[9] with the B3LYP density functional[10,11] and split-valence polarized 6-31G(d) basis set.[12] In order to apply polarization of the hydrogen atoms and better account for dispersion interactions in the system, select low-energy conformations for each species (Table S1) were reoptimized using the B3LYP density functional,[10,11] dispersion-corrected with the D3 version of Grimme's dispersion[13] with Becke-Johnson damping (D3(BJ)),[14–16] and a split-valence polarized 6-31G(d,p) basis set. No qualitative differences were observed between the lowest energy major and minor TS conformers derived at the two levels of theory. Single point energy (SPE) calculations were used to correct the Gibbs free energy derived from the original B3LYP calculations.[17] These were performed with an ultrafine integration grid using the B3LYP-D3(BJ) method and the polarized triple-ζ valence quality (def2-TZVPP) basis set.[18] The integral equation formalism version of the polarisable continuum model (IEFPCM)[19] (propanonitrile) was also used was used to incorporate the effect of solvent. Additionally, for the lowest energy conformers of the Mukaiyama aldol TS complex (R = 3,5-$(CF_3)_2C_6H_3$), SPEs were recalculated with several other methods, including the ωB97X-D[20] and M06-2X[21] functionals, the 6-311G(d,p)[22] and cc-pVTZ[23] basis sets, and the solvent model based on density (SMD)[24] (Table S3). All temperature (195.15 K) and concentration-corrected (1 mol/l) quasiharmonic (Grimme approximation[25]) free energies were calculated with GoodVibes[26] with a vibrational scaling factor of 0.977.[27] Computed *ee*s were generated based on Boltzmann weightings at 195.15 K over all

conformers within 3 kcal mol^{-1} of either **TS-1** or **TS-1-Me** (as appropriate) or over both conformers for the phenyl-substituted system (**TS-1-Ph** and **TS-1-Ph**). Computed structures were illustrated with CYLView.[28] Noncovalent interaction (NCI) analyses were performed using the NCIPLOT[29] program and illustrated using VMD.[30]

Natural bond orbital (NBO) analyses were performed using Gaussian16 (Revision A.03)[9] with an ultrafine integration grid using the B3LYP-D3(BJ) method and the 6-31G(d,p) basis set. A full list of approximated interaction strengths are given below:

- **TS-1** silyl enol ether π-π interaction with boron substituent (3,5-$(CF_3)_2C_6H_3$): 2 kcal mol^{-1}
- **TS-1-Ph** silyl enol ether π-π interaction with boron substituent (Ph): 1.7 kcal mol^{-1}
- **TS-1-Me** benzaldehyde π-π interaction with *N*-sulfonyl substituent: 2.3 kcal mol^{-1}

2. Interaction definitions

Nonclassical C-H···O hydrogen bonding

In this study, nonclassical C-H···O hydrogen bonds[31] are defined as any interaction between a carbon-bound hydrogen and oxygen within the sum of the O-H Van der Waals radii threshold (2.72Å), without consideration of directionality.

π interactions (π···π and C-H···π).

Many varieties of π interaction are possible in NTOB-catalyzed reactions, including both parallel π-π and edge-to-face C-H···π arrangements (Figure S2a).[32,33] Such interactions are common in many organic and biological systems.[34,35] Studies of the benzene-benzene dimer indicate that parallel π-π interactions are generally disfavored relative to parallel displaced and edge-to-face arrangements,[36] except where aromatic systems are substituted with electron-withdrawing or electron-donating groups.[37–39] However, whilst derivatives of the long-standing Hunter-Sanders electrostatic model[40–42] (Figure S3a) allow qualitative predictions of most π-π geometries in line with experimental trends, they fail to totally account for the effects of these substitutions on the involved π-systems. More recent reports have rationalized these substituent effects on the basis of direct through-space interactions between the highly polarized substituent and closest region of the complementary aromatic system (Figure S3b),[43–45] allowing extension of these concepts outside of the specific situation of interaction between an electron-rich and an electron-poor aromatic. Indeed, computations have shown that all substituted benzene dimers have more favorable binding interactions than their unsubstituted cases, whether the substituents are electron donating or electron withdrawing.[37,38] These studies on π-π interactions form a strong basis upon which selectivity can be rationalized in the NTOB-catalyzed Mukaiyama aldol reaction, where face-centered interactions can form between different combinations of nonsubstituted, electron-rich, and electron-deficient aromatics.

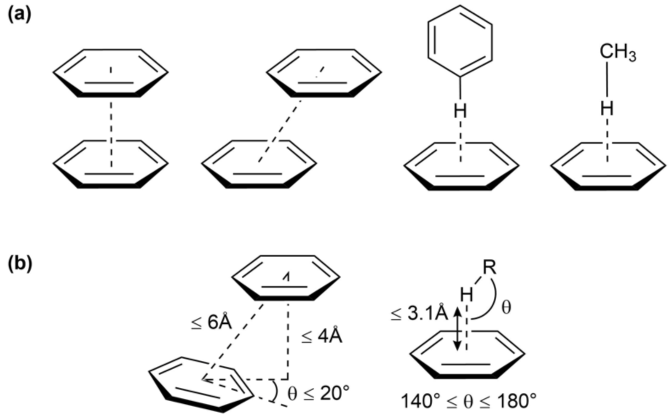

Figure S2. (a) parallel π-π and edge-to-face C-H···π bonding arrangements; (b) criteria for π interaction types.

In this study, π-π or C-H···π interactions are measured from the ring centroid of the relevant π-system. Parallel π-π interactions are defined as any situation where two π-systems lie in parallel planes, no more than 20° out of alignment, with a maximum perpendicular distance of 4Å between the two planes, and a maximum distance of 6Å between the ring centroids (Figure S2b).[46] C-H···π interactions are defined by a threshold of up to 3.1Å between the hydrogen and the ring centroid of the π-system, and within a 140-180° angle of approach between the C-H and π-system (Figure S2b). Although this distance threshold is greater than the sum of the C-H Van der Waal radii (2.9Å), C-H distances of around 3.1 Å have been reported for such interactions.[47]

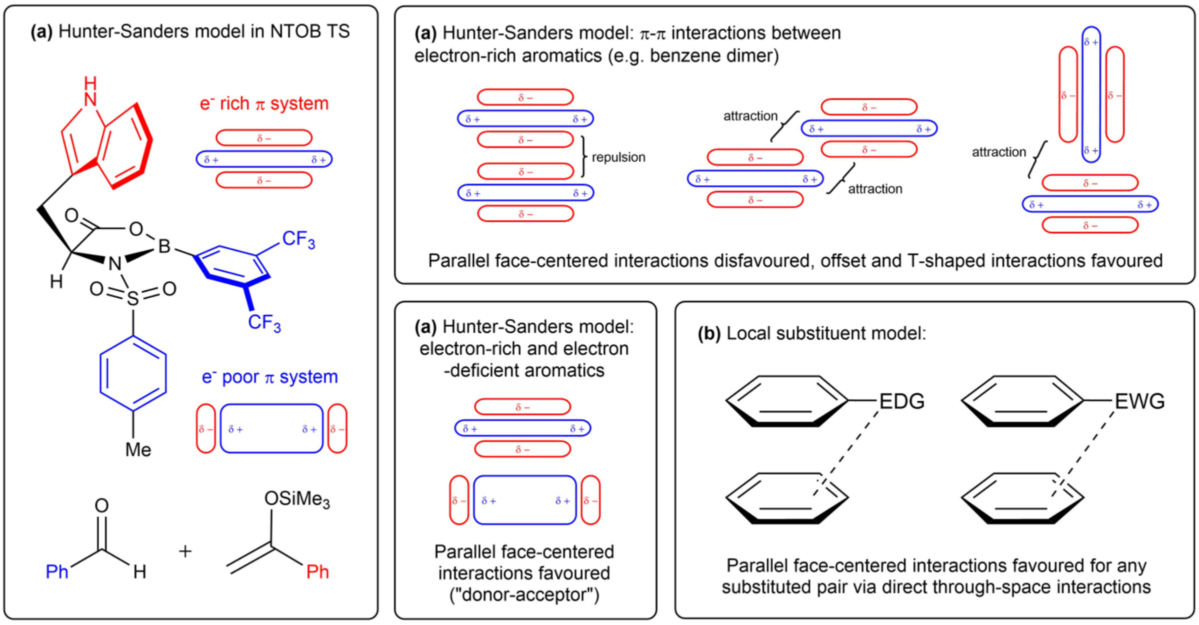

Figure S3. (a) Hunter-Sanders model for aromatic π-π interactions: each atom is treated as a local quadrupole, consisting of the positively charged nucleus and a region of negative π-charge above and below the plane of the ring. The energy of interaction is determined by attraction between the negative π-electrons and the positive nuclei, attractive contact-dependant van der Waals interactions, and repulsion between the negative π-electrons; (b) direct substituent effects allow rationalization of all classes of π-π interaction.

3. Analysis of ground state structures

The lowest energy conformer for the nucleophile (acetophenone-derived trimethylsilyl enol ether), electrophile (benzaldehyde), and NTOB catalyst are shown in Figure S4. In the lowest energy catalyst conformation, the five-membered oxazaborolidinone ring is close to planar, and the *N*-sulfonyl group lies perpendicular to this plane on the bottom face of the ring due to steric shielding of the top face by the indole unit.[48,49] As a result, the *N*-sulfonyl oxygens lie slightly upwards of the plane of the ring, in agreement with previous analyses of these catalyst types.[49–51] An intramolecular stabilizing C-H···O interaction is present between the hydrogen at the 4-position of the indole and the nearby *N*-sulfonyl oxygen.

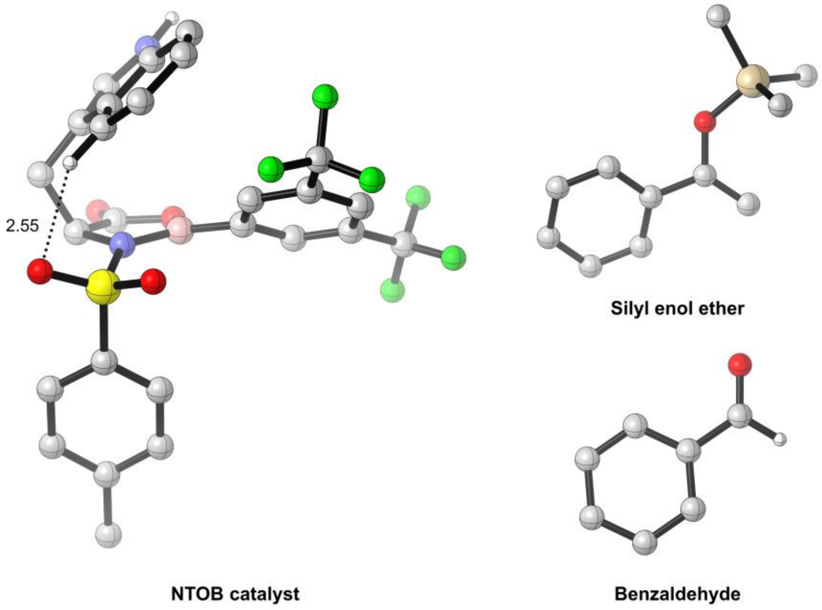

Figure S4. Lowest free energy conformers for the nucleophile (acetophenone-derived trimethylsilyl enol ether), electrophile (benzaldehyde), and NTOB catalyst (B3LYP-D3(BJ)/def2-TZVPP/IEFPCM(propanonitrile)//B3LYP-D3(BJ)/6-31G(d,p)). All distances in angstroms (Å).

4. Analysis of NTOB-aldehyde complex

Among the 124 conformations obtained and optimized with B3LYP/6-31G(d) for the NTOB-aldehyde complex, six distinct binding modes can be defined. These corresponded to each combination of a top or bottom-face binding of the aldehyde, and the presence of either a C-H···O-B, C-H···O=S, or no formyl interaction between the catalyst and aldehyde (Figure S5). The A1 and B1 modes correspond to Corey and Wong-like binding, respectively. Among the 46 selected conformers reoptimized with B3LYP-D3(BJ)/6-31G(d,p), all but the C2 binding mode were represented. The relative energy of the lowest energy conformer for each mode with B3LYP-D3(BJ)/6-31G(d,p) are included in Figure S5. The overall lowest energy conformer, **NTOB-E1,** resembles the Wong model (B1), and is given in Figure S6 along with **NTOB-E2**, the lowest energy conformer representing the Corey model (A1).

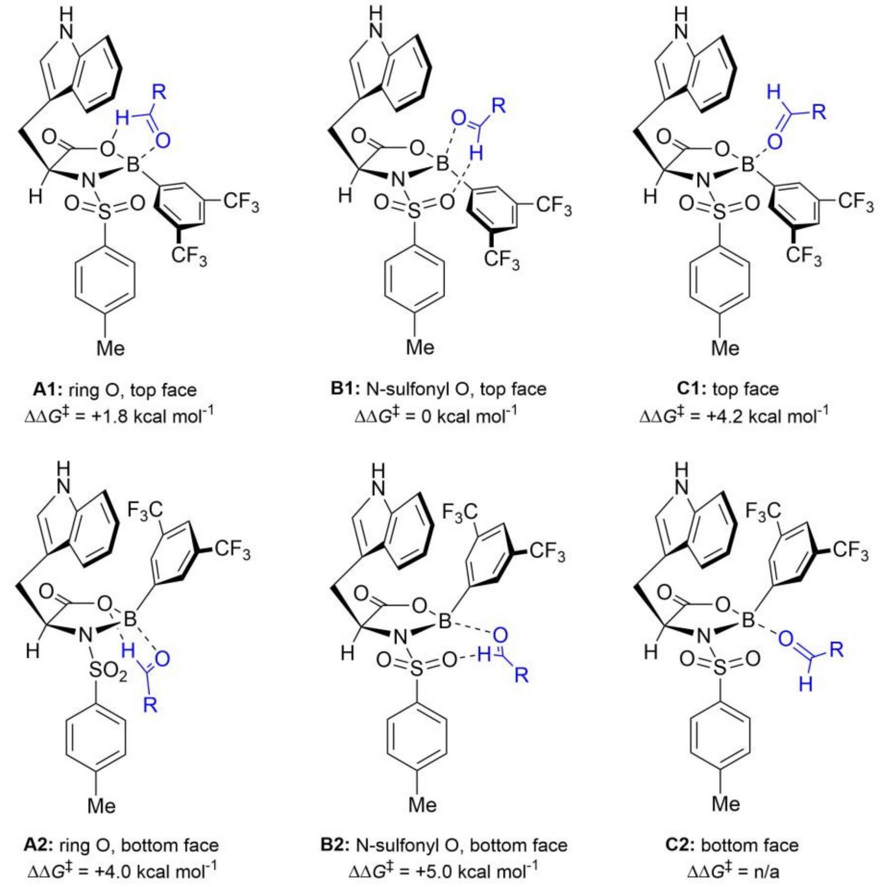

Figure S5. Six possible binding modes for the NTOB-aldehyde complex and relative energies of the lowest energy conformer for each (B3LYP-D3(BJ)/def2-TZVPP/IEFPCM(propanonitrile)//B3LYP-D3(BJ)/6-31G(d,p)).

In both **NTOB-E1** and **NTOB-E2**, the *N*-sulfonyl group is located on the bottom face of the NTOB catalyst due to steric shielding of the top face by the indole unit.[48,49] As a result, the aldehyde binds from the top face, due to steric shielding of the bottom face by the *N*-sulfonyl group[51–53] and the formation of π interactions with the electron rich indole (Figure S7).[54,55] The lowest energy conformer that places the *N*-sulfonyl group on the top face is 3.7 kcal mol^{-1} higher in energy than **NTOB-E1**, and the lowest conformer with electrophilic binding from the bottom face is 4.0 kcal mol^{-1} higher in energy than **NTOB-E1**. Accordingly, supporting NOESY analyses on the binding of amines to oxazaborolidinones has shown that facial selectivity improves as the amine is made more electron deficient, and the roles of both sterics and π-π interactions have been supported by reports of diminished, and even reversed, enantioselectivity in *N*-sulfonylated oxazaborolidinone-catalyzed Diels-Alder and Mukaiyama aldol reactions as the amino substituent is changed from electron rich aryl substituents to increasingly bulky alkyl groups.[56–59] Whilst these studies support the idea of favorable π-π interactions with the electron rich indole, facial selectivity has also been found to persist in the absence of aromatic groups.[55] However, this could also be due to the formation of C-H···π interactions with the aldehyde, in line with our new TS model.

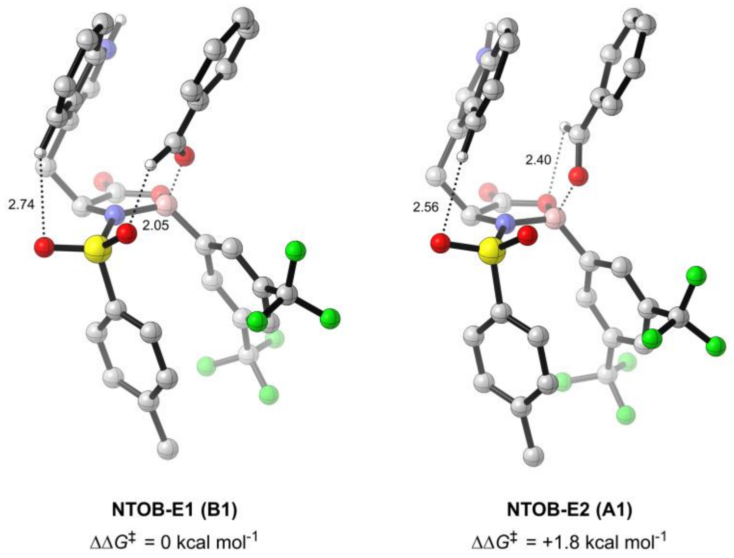

Figure S6. Lowest free energy conformers for the NTOB-aldehyde complex (B3LYP-D3(BJ)/def2-TZVPP/IEFPCM(propanonitrile)//B3LYP-D3(BJ)/6-31G(d,p)). All distances in angstroms (Å).

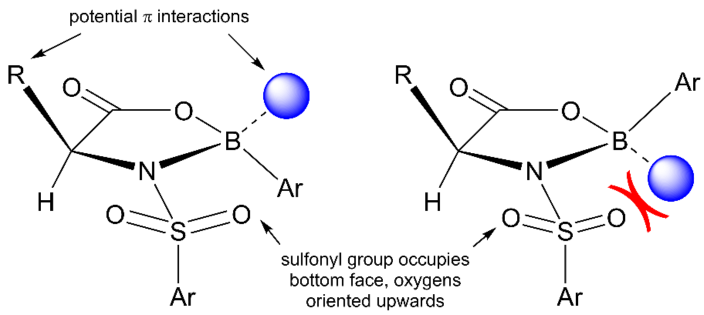

Figure S7 Possible NTOB-aldehyde binding.

All conformers within 4.2 kcal mol^{-1} of **NTOB-E1** possess some form of formyl interaction (A or B-type modes), whilst C-type modes are generally disfavored due to the lack of stabilizing C-H···O-B or C-H···O=S formyl interaction. Comparison of **NTOB-E1** and **NTOB-E2** represents a direct comparison of the Wong and Corey models, respectively. The overall preference for a B1 mode is in agreement with Wong's conclusions that the *N*-sulfonyl oxygens are better hydrogen bond acceptors than the ring oxygen.[50] Thus, if interactions to the nucleophile are neglected, these results predict the opposite sense of enantioselectivity for Mukaiyama aldol reactions, suggesting that interactions between the catalyst and nucleophile are essential in the TS reaction model.

5. Analysis of transition-state complex

Among the 328 conformations obtained and optimized with B3LYP/6-31G(d) for the Mukaiyama aldol TS complex (R = 3,5-$(CF_3)_2C_6H_3$), all six of the binding modes located for the NTOB-aldehyde complex (Figure S5) are represented. In addition, a new mode (D) can be defined involving a C-H···π interaction between a vinyl hydrogen of the silyl enol ether and the six-membered ring of the indole (Figure S8). For this interaction to form, the six-membered ring of the indole must be orientated in the same direction as the silyl enol ether is bound, thus fixing the indole into one of two conformations. Analogous structures to **TS-1** and **TS-2** with the indoles in their opposite conformations are both higher in energy than their respective counterparts (Figure S9). Among the 47 selected conformers reoptimized with B3LYP-D3(BJ)/6-31G(d,p) for the Mukaiyama aldol TS complex (R = 3,5-$(CF_3)_2C_6H_3$), all but the C1 binding mode were represented. **TS-1**, **TS-2**, **TS-1'**, **TS-2'**, **TS-1-Me**, **TS-2-Me**, **TS-1-Ph**, **TS-2-Ph** each correspond to binding mode D, whilst **TS-3** and **TS-4** correspond to A1 (Corey-like) and B1 (Wong-like) modes, respectively. The relative energies of the lowest energy conformer for each mode with B3LYP-D3(BJ)/6-31G(d,p), in addition to the number of conformers belonging to each, are included in Table S2.

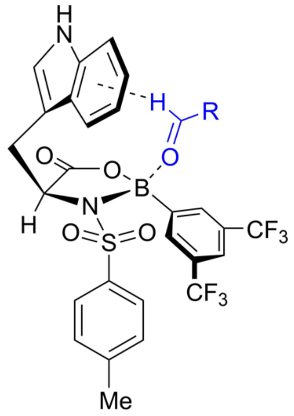

D: indole π-system, top face

Figure S8. New NTOB-aldehyde binding mode.

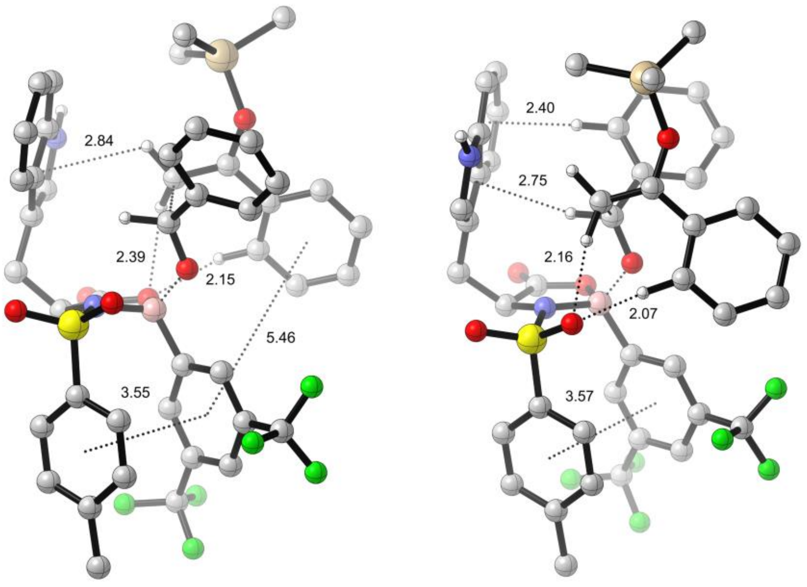

TS-1' (major)
$\Delta\Delta G^{\ddagger}$ = +1.4 kcal mol^{-1}

TS-2' (minor)
$\Delta\Delta G^{\ddagger}$ = +2.7 kcal mol^{-1}

Figure S9. Lowest free energy major (**TS-1'**) and minor (**TS-2'**) TSs for the NTOB-catalyzed Mukaiyama aldol reaction (R = 3,5-$(CF_3)_2C_6H_3$) with indole conformation flipped relative to **TS-1** and **TS-2** (B3LYP-D3(BJ)/def2-TZVPP/IEFPCM(propanonitrile)//B3LYP-D3(BJ)/6-31G(d,p)) with NCI distances in angstroms (Å). π interactions measured from the relevant ring centroid.

Binding Mode	Number	Relative Energy of Lowest / kcal mol^{-1}
A1	5	2.7
A2	4	3.3
B1	2	6.5
B2	3	4.1
C1	0	n/a
C2	8	2.5
D	15	0

Table S2. Number of conformers belonging to each binding mode and relative energy of the lowest energy conformer within the 47 reoptimized conformers of the NTOB-catalyzed Mukaiyama aldol reaction (R = 3,5-$(CF_3)_2C_6H_3$) (B3LYP-D3(BJ)/def2-TZVPP/IEFPCM(propanonitrile)//B3LYP-D3(BJ)/6-31G(d,p)).

Like for the NTOB-aldehyde complex, in the lowest energy major and minor TSs, **TS-1** and **TS-2**, the *N*-sulfonyl group is located on the bottom face of the NTOB catalyst and the aldehyde on the top face.[51–53] The lowest energy conformer with the *N*-sulfonyl located on the top face is 4.1 kcal mol^{-1} higher in energy than **TS-1**, and the lowest conformer with the aldehyde bound on the bottom face is 2.5 kcal mol^{-1} higher in energy **TS-1**.

To validate the energy difference between **TS-1** and **TS-2**, SPEs were recalculated at several levels of theory on the B3LYP-D3(BJ)/6-31G(d,p) structures and the free energy difference and computed *ee*, based on a Boltzmann weighting at 195.15 K between **TS-1** and **TS-2**, calculated (Table S3). In all cases, a strong preference for **TS-1** and a computed *ee* in good agreement with the experiment *ee* of 94% was obtained.

SPE Method	Free Energy Difference / kcal mol^{-1}	Computed *ee*
B3LYP-D3(BJ)/def2-TZVPP/IEFPCM(propanonitrile)	2.5	>99%
B3LYP-D3(BJ)/6-311G(d,p)/IEFPCM(propanonitrile)	2.1	99%
B3LYP-D3(BJ)/cc-PVTZ/IEFPCM(propanonitrile)	4.1	>99%
B3LYP-D3(BJ)/def2-TZVPP/SMD(propanonitrile)	2.6	>99%
ωB97XD/def2-TZVPP/IEFPCM(propanonitrile)	4.0	>99%
ωB97XD/def2-TZVPP/SMD(propanonitrile)	4.1	>99%
M06-2X/def2-TZVPP/IEFPCM(propanonitrile)	3.3	>99%
M06-2X/def2-TZVPP/SMD(propanonitrile)	3.4	>99%

Table S3. Free energy difference and computed *ee* (based on a Boltzmann weighting at 195.15 K) between **TS-1** and **TS-2** with several SPE methods on the B3LYP-D3(BJ)/6-31G(d,p) structures.

NCI analysis of **TS-3** and **TS-4**, the lowest energy major TSs located representing Corey and Wong-like binding, is given in Figure S10. π-π interactions are identified between the indole, aldehyde, and silyl enol ether in **TS-3**, and between the indole and silyl enol ether in **TS-4**, as well as between the *N*-sulfonyl and boron substituent in both **TS-3** and **TS-4**.

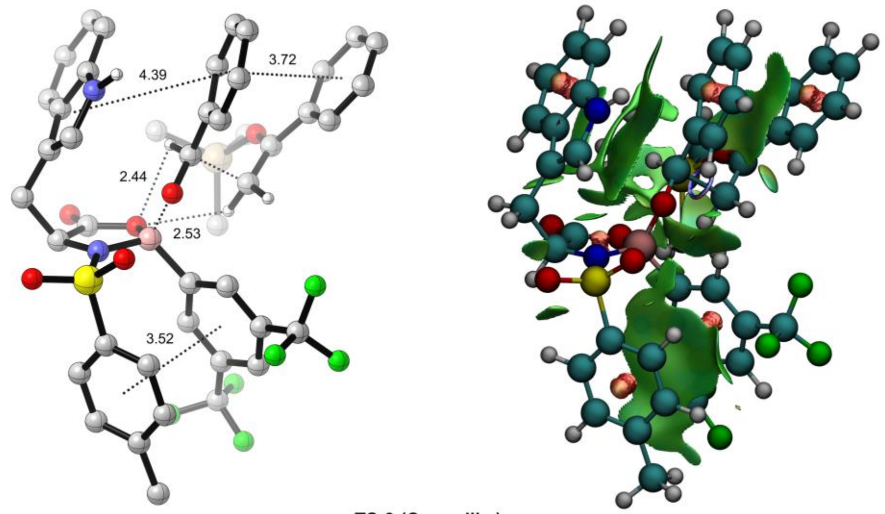

TS-3 (Corey-like)

$\Delta\Delta G^{\ddagger}$ = +2.7 kcal mol^{-1}

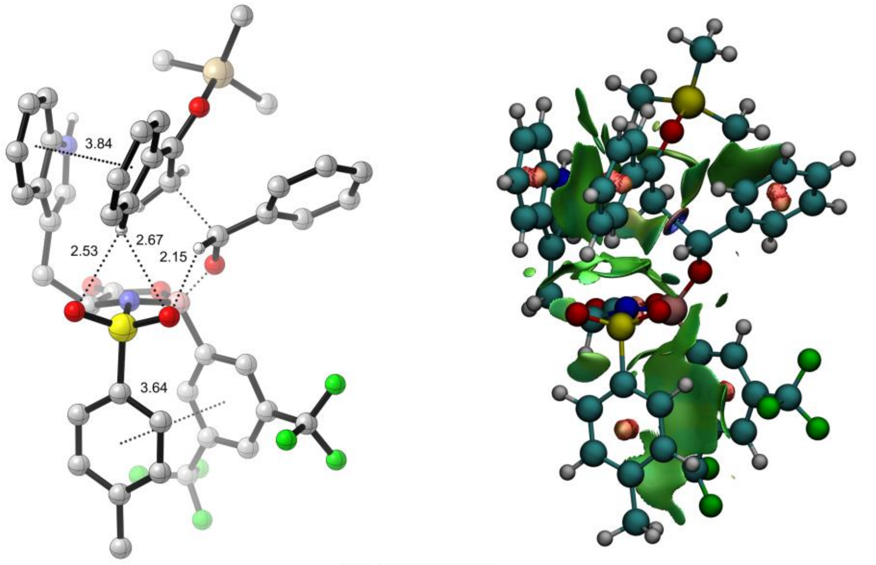

TS-4 (Wong-like)

$\Delta\Delta G^{\ddagger}$ = +6.5 kcal mol^{-1}

Figure S10. Lowest free energy Corey (**TS-3**) and Wong-like (**TS-4**) TSs, with energies relative to **TS-1**, for the NTOB-catalyzed Mukaiyama aldol reaction (R = 3,5-$(CF_3)_2C_6H_3$) (B3LYP-D3(BJ)/def2-TZVPP/IEFPCM(propanonitrile)//B3LYP-D3(BJ)/6-31G(d,p)) with NCI distances in angstroms (Å). π interactions measured from the relevant ring centroid. Green, red, and blue NCI surfaces represent weak, strong repulsive and strong attractive NCIs, respectively.

6. Geometries and energies for all computed structures

Geometries and energies are given for both the B3LYP/6-31G(d)-optimized and B3LYP-D3(BJ)/6-31G(d,p)-optimized structures. All energies in Hartrees, coordinates in Å. Cartesian coordinates generated by ESIgen software.[60] Structures labelled as **TS X** contain the boron substituent R = 3,5-$(CF_3)_2C_6H_3$, whilst those labelled **TS Me X** contain R = Me. Structures provided and discussed in the manuscript or ESI are labelled (**NTOB-E1**, **NTOB-E2**, **TS-1**, **TS-1'**, **TS-2**, **TS-2'**, **TS-3**, **TS-4**, **TS-Me-1**, **TS-Me-2**, **TS-Ph-1**, and **TS-Ph-2**).

Optimization with B3LYP/6-31G(d)

Benzaldehyde

B3LYP/6-31G(d) = -345.573441
B3LYP-D3(BJ)/def2-TZVPP/IEFPCM(propanonitrile) = -345.741194
B3LYP-D3(BJ)/def2-TZVPP/IEFPCM(propanonitrile)//B3LYP-D3(BJ)/6-31G(d) Free Energy (Quasiharmonic) = -345.65007

Frequencies (Top 3 out of 36)

1. 124.9970 cm^{-1}
2. 221.0056 cm^{-1}
3. 240.2080 cm^{-1}

B3LYP/6-31G(d) Molecular Geometry in Cartesian Coordinates

O	-2.847619	-0.395783	0.000189
C	-1.992222	0.468773	-0.000137
C	-0.533953	0.214395	-0.000039
C	0.361111	1.292022	0.000025
C	-0.045225	-1.101239	-0.000041
C	1.735957	1.060667	0.000079
H	-0.025055	2.309403	-0.000066
C	1.326268	-1.331223	-0.000047
H	-0.759104	-1.919350	-0.000081
C	2.216815	-0.250722	0.000042
H	2.430258	1.896231	0.000125
H	1.707967	-2.348499	-0.000198
H	3.288189	-0.433387	0.000016
H	-2.273810	1.545835	-0.000609

Silyl enol ether 1

B3LYP/6-31G(d) = -793.582784
B3LYP-D3(BJ)/def2-TZVPP/IEFPCM(propanonitrile) = -793.881485
B3LYP-D3(BJ)/def2-TZVPP/IEFPCM(propanonitrile)//B3LYP-D3(BJ)/6-31G(d) Free Energy (Quasiharmonic) = -793.668721

Frequencies (Top 3 out of 81)

1. 12.7725 cm^{-1}
2. 31.5113 cm^{-1}
3. 45.1915 cm^{-1}

B3LYP/6-31G(d) Molecular Geometry in Cartesian Coordinates

C	0.122328	2.352467	-0.691487
H	1.099297	2.748359	-0.944235
H	-0.687997	3.058744	-0.557247
C	-0.083302	1.029448	-0.601512
C	-1.393956	0.401376	-0.291831
C	-2.385159	1.086341	0.431623
C	-1.668205	-0.904281	-0.730061
C	-3.616528	0.490250	0.693117
H	-2.180391	2.082663	0.812097
C	-2.902139	-1.499128	-0.468679
H	-0.909027	-1.440872	-1.288691
C	-3.882125	-0.805257	0.242392
H	-4.366994	1.034377	1.260694
H	-3.098121	-2.507770	-0.823386
H	-4.841821	-1.270776	0.450353
O	0.925528	0.133432	-0.873911
Si	2.185797	-0.308683	0.167486
C	1.486319	-0.775015	1.854495
H	2.288320	-1.079849	2.538373
H	0.960395	0.068237	2.317079
H	0.775655	-1.605451	1.777186
C	3.417093	1.108899	0.342425
H	4.258754	0.815533	0.982623
H	3.826085	1.402944	-0.631312
H	2.952536	1.993558	0.792497
C	2.982979	-1.781022	-0.686786
H	3.851458	-2.143468	-0.123407
H	2.277090	-2.613484	-0.785509
H	3.325534	-1.513109	-1.692810

Silyl enol ether 2

B3LYP/6-31G(d) = -793.582784
B3LYP-D3(BJ)/def2-TZVPP/IEFPCM(propanonitrile) = -793.881487
B3LYP-D3(BJ)/def2-TZVPP/IEFPCM(propanonitrile)//B3LYP-D3(BJ)/6-31G(d) Free Energy (Quasiharmonic) = -793.668718

Frequencies (Top 3 out of 81)

1. 12.9119 cm^{-1}
2. 31.5471 cm^{-1}
3. 45.1935 cm^{-1}

B3LYP/6-31G(d) Molecular Geometry in Cartesian Coordinates

C	0.122600	2.352587	-0.691592
H	1.099561	2.748448	-0.944427
H	-0.687678	3.058891	-0.557191
C	-0.083084	1.029571	-0.601737
C	-1.393712	0.401489	-0.291967
C	-2.384852	1.086365	0.431657
C	-1.667982	-0.904133	-0.730289
C	-3.616168	0.490206	0.693255
H	-2.180083	2.082670	0.812176
C	-2.901863	-1.499047	-0.468804
H	-0.908853	-1.440645	-1.289064
C	-3.881781	-0.805273	0.242455
H	-4.366584	1.034262	1.260967
H	-3.097855	-2.507664	-0.823579
H	-4.841436	-1.270841	0.450498
O	0.925697	0.133533	-0.874306
Si	2.185518	-0.308750	0.167555
C	1.485386	-0.773743	1.854674
H	2.287103	-1.078157	2.539071
H	0.959400	0.069917	2.316453
H	0.774625	-1.604134	1.777759
C	3.417624	1.108200	0.341923
H	4.258946	0.814765	0.982534
H	3.827032	1.401331	-0.631919

H	2.953486	1.993440	0.791286
C	2.981980	-1.781982	-0.685834
H	3.850267	-2.144542	-0.122233
H	2.275673	-2.614145	-0.784096
H	3.324681	-1.514800	-1.692003

Silyl enol ether 3

B3LYP/6-31G(d) = -793.58319
B3LYP-D3(BJ)/def2-TZVPP/IEFPCM(propanonitrile) = -793.881382
B3LYP-D3(BJ)/def2-TZVPP/IEFPCM(propanonitrile)//B3LYP-D3(BJ)/6-31G(d) Free Energy (Quasiharmonic) = -793.668684

Frequencies (Top 3 out of 81)

1. 13.2524 cm^{-1}
2. 33.1554 cm^{-1}
3. 77.0438 cm^{-1}

B3LYP/6-31G(d) Molecular Geometry in Cartesian Coordinates

C	0.132291	-2.057991	0.375702
H	1.159621	-2.399387	0.413169
H	-0.634038	-2.796117	0.573794
C	-0.192657	-0.778902	0.120612
C	-1.591124	-0.279542	0.042898
C	-2.667058	-1.145405	-0.216367
C	-1.863757	1.085130	0.228041
C	-3.973761	-0.666065	-0.263141
H	-2.478354	-2.197341	-0.407012
C	-3.172562	1.563960	0.180876
H	-1.040242	1.765403	0.412706
C	-4.234100	0.691536	-0.061389
H	-4.790240	-1.353031	-0.470156
H	-3.362083	2.623457	0.333157
H	-5.253684	1.065500	-0.102237
O	0.723299	0.217080	-0.076368
Si	2.417728	0.228281	-0.058393
C	3.066059	-0.293982	1.633904
H	4.160532	-0.220432	1.663293
H	2.669045	0.358374	2.420436
H	2.794562	-1.323100	1.890679
C	2.840491	2.027873	-0.398219
H	3.926425	2.180314	-0.424471
H	2.433346	2.353393	-1.362128
H	2.430032	2.685312	0.376671
C	3.093952	-0.875622	-1.429624
H	4.188711	-0.812097	-1.468960
H	2.827081	-1.928984	-1.294372
H	2.708068	-0.559772	-2.405894

NTOB catalyst 1

B3LYP/6-31G(d) = -2434.772419
B3LYP-D3(BJ)/def2-TZVPP/IEFPCM(propanonitrile) = -2435.851825
B3LYP-D3(BJ)/def2-TZVPP/IEFPCM(propanonitrile)//B3LYP-D3(BJ)/6-31G(d) Free Energy (Quasiharmonic) = -2435.473104

Frequencies (Top 3 out of 171)

1. 9.1202 cm^{-1}
2. 10.2161 cm^{-1}
3. 10.9307 cm^{-1}

B3LYP/6-31G(d) Molecular Geometry in Cartesian Coordinates

B	0.318598	0.017503	0.911125
O	0.521153	0.191705	2.287008
N	1.508986	0.446744	0.218631
C	2.458165	1.011162	1.203273
S	2.092086	0.010888	-1.318353
C	1.762897	0.721459	2.532860
O	2.180903	0.947944	3.633880
H	3.234840	2.697977	0.113218
H	3.412448	0.471139	1.177787
C	2.727550	2.534557	1.067228
C	1.502907	3.397738	1.181717
C	0.637585	3.850725	0.116160
C	-0.390417	4.630005	0.715153
N	-0.151342	4.644708	2.075172
C	0.981402	3.907729	2.348461
H	1.336483	3.797336	3.364082
C	-1.387066	5.045957	-1.409301
C	0.635903	3.688070	-1.281995
H	-2.186882	5.821581	0.444806
H	-0.715501	5.118032	2.763516
C	-0.373618	4.284029	-2.027218
H	-2.163909	5.497257	-2.020303
H	1.413153	3.108394	-1.771235
C	-1.408571	5.231541	-0.032419
H	-0.384622	4.162132	-3.106705
C	2.982358	-1.516541	-1.027861
O	3.070102	1.032813	-1.697858
O	0.927735	-0.283649	-2.149856
C	4.365160	-3.903026	-0.562920
C	4.371668	-1.491745	-0.908699
C	2.269730	-2.716413	-0.931808
C	2.966368	-3.896717	-0.697699
C	5.051404	-2.687926	-0.674297
H	4.908353	-0.555483	-1.018330
H	1.191346	-2.723182	-1.054296
H	2.417549	-4.832394	-0.625070
H	6.134281	-2.674156	-0.582953
C	5.103422	-5.197716	-0.323357
H	4.674461	-5.749508	0.521018
H	6.162176	-5.023068	-0.111188
H	5.042149	-5.853381	-1.200995
H	3.439254	2.779605	1.864519
C	-1.041893	-0.578227	0.449426
C	-1.718309	-1.425058	1.346314
C	-1.670573	-0.264079	-0.768326
C	-2.963441	-1.966148	1.026124
H	-1.266718	-1.663968	2.303651
C	-2.923335	-0.794464	-1.078346
H	-1.185182	0.398721	-1.472559
C	-3.571694	-1.651769	-0.188805
H	-4.542701	-2.064995	-0.437252
C	-3.561043	-0.492055	-2.410527
C	-3.684011	-2.835246	2.023337
F	-4.911319	-0.499318	-2.327332
F	-3.221285	-1.412615	-3.341394
F	-3.187234	0.712294	-2.885573
F	-2.820776	-3.558250	2.770401
F	-4.524236	-3.702414	1.416894
F	-4.426848	-2.099641	2.880941

NTOB catalyst 2

B3LYP/6-31G(d) = -2434.76848
B3LYP-D3(BJ)/def2-TZVPP/IEFPCM(propanonitrile) = -2435.851224
B3LYP-D3(BJ)/def2-TZVPP/IEFPCM(propanonitrile)//B3LYP-D3(BJ)/6-31G(d) Free Energy (Quasiharmonic) = -2435.471677

Frequencies (Top 3 out of 171)

1. 9.4797 cm^{-1}
2. 14.3015 cm^{-1}
3. 15.3173 cm^{-1}

B3LYP/6-31G(d) Molecular Geometry in Cartesian Coordinates

B	-0.454414	-1.560703	-0.868632
O	-0.566564	-2.936559	-0.666472
N	0.846949	-1.261232	-1.425197
C	1.637556	-2.510718	-1.428274
S	1.150803	-0.072347	-2.627914
C	0.602588	-3.565922	-1.035542
O	0.756677	-4.753350	-0.985296
H	3.480534	-1.691889	-0.653645
H	2.011751	-2.717780	-2.433960
C	2.821004	-2.534698	-0.418795
C	2.426545	-2.524676	1.033337
C	2.183410	-1.392461	1.899341
C	1.801426	-1.910966	3.168718
N	1.824195	-3.286949	3.070783
C	2.194927	-3.647893	1.794164
H	2.257508	-4.689607	1.511224
C	1.549648	0.286474	4.058982
C	2.258109	0.004199	1.742207
H	1.189077	-1.506667	5.212543
H	1.580961	-3.930554	3.807687
C	1.937673	0.826463	2.815471
H	1.301655	0.955027	4.878516
H	2.567681	0.436877	0.796576
C	1.482417	-1.087367	4.253589
H	1.982187	1.905109	2.694803
C	2.121850	1.221278	-1.860773
O	2.003228	-0.730120	-3.622093
O	-0.150125	0.494481	-2.979218
C	3.639003	3.253733	-0.677138
C	1.476588	2.334872	-1.321025
C	3.515332	1.118670	-1.845175
C	4.260998	2.133253	-1.249985
C	2.240389	3.337060	-0.725455
H	0.398314	2.424851	-1.373317
H	4.004272	0.268562	-2.309060
H	5.345299	2.056914	-1.234874
H	1.734019	4.197029	-0.295212
C	4.461252	4.357787	-0.058010
H	5.346059	3.961136	0.450658
H	3.878160	4.932950	0.667645
H	4.814035	5.059099	-0.825561
H	3.385480	-3.447960	-0.639290
C	-1.595249	-0.598063	-0.443313
C	-1.288413	0.622774	0.170213
C	-2.948075	-0.935955	-0.610505
C	-2.296572	1.502614	0.567522
H	-0.253224	0.884617	0.361036
C	-3.955634	-0.057007	-0.214154
H	-3.216866	-1.884136	-1.064689
C	-3.634868	1.171771	0.369301
H	-4.419497	1.858084	0.665237
C	-5.403076	-0.451089	-0.357939
C	-1.898635	2.823555	1.168319
F	-5.591074	-1.309633	-1.381668
F	-5.862804	-1.059524	0.759747
F	-6.193386	0.623727	-0.573084
F	-1.032087	2.665384	2.192830
F	-2.954581	3.522278	1.627911
F	-1.267521	3.609427	0.252987

NTOB catalyst 3

B3LYP/6-31G(d) = -2434.771819
B3LYP-D3(BJ)/def2-TZVPP/IEFPCM(propanonitrile) = -2435.849646
B3LYP-D3(BJ)/def2-TZVPP/IEFPCM(propanonitrile)//B3LYP-D3(BJ)/6-31G(d) Free Energy (Quasiharmonic) = -2435.471058

Frequencies (Top 3 out of 171)

1. 6.9348 cm^{-1}
2. 8.9700 cm^{-1}
3. 11.4386 cm^{-1}

B3LYP/6-31G(d) Molecular Geometry in Cartesian Coordinates

B	-0.218142	0.073495	-0.603804
O	-0.429451	0.671621	-1.853071
N	-1.489462	-0.011583	0.074332
C	-2.523821	0.646007	-0.755033
S	-1.974932	-0.966713	1.391851
C	-1.752482	0.986409	-2.027908
O	-2.193978	1.470586	-3.032204
H	-3.877268	1.569258	0.636190
H	-3.314255	-0.067073	-1.021060
C	-3.186050	1.911444	-0.136599
C	-2.237269	2.921678	0.438260
C	-1.585815	4.024513	-0.233351
C	-0.802398	4.701399	0.742914
N	-0.972552	4.032487	1.939048
C	-1.832771	2.971697	1.751696
H	-2.106387	2.323680	2.573094
C	-0.072968	6.298228	-0.868786
C	-1.596728	4.519783	-1.550829
H	0.550922	6.337021	1.202150
H	-0.548159	4.287686	2.817110
C	-0.843767	5.646843	-1.853522
H	0.506943	7.176710	-1.138140
H	-2.173387	4.021247	-2.324397
C	-0.041598	5.835588	0.441126
H	-0.844319	6.033364	-2.868917
C	-2.633351	-2.446941	0.628865
O	-0.763702	-1.322573	2.128403
O	-3.082586	-0.264708	2.044280
C	-3.652973	-4.758988	-0.570200
C	-4.013355	-2.597760	0.494311
C	-1.751125	-3.438545	0.187513
C	-2.268211	-4.583596	-0.408152
C	-4.511317	-3.754787	-0.106298
H	-4.681852	-1.832622	0.874176
H	-0.681320	-3.320183	0.327113
H	-1.587264	-5.360398	-0.746869
H	-5.586092	-3.879532	-0.208386
C	-4.193627	-6.001160	-1.235836
H	-5.275061	-6.092104	-1.098643
H	-3.722186	-6.905261	-0.834640
H	-3.995320	-5.985763	-2.315180
H	-3.786119	2.353300	-0.941540
C	1.240578	-0.357065	-0.266869
C	1.791305	-0.402198	1.025487
C	2.085052	-0.648691	-1.354749
C	3.131993	-0.738240	1.217879
H	1.170025	-0.190280	1.885177
C	3.418293	-1.007212	-1.157657
H	1.693998	-0.599672	-2.365466
C	3.948743	-1.051272	0.131316
H	4.983237	-1.335034	0.287965
C	4.305883	-1.288388	-2.342422
C	3.721153	-0.713423	2.605102
F	4.893493	-0.161704	-2.802857
F	5.297639	-2.151363	-2.028935
F	3.611488	-1.821036	-3.371453
F	4.226220	0.506365	2.904337
F	4.734195	-1.599586	2.732265
F	2.803087	-1.002909	3.548774

NTOB catalyst 4

B3LYP/6-31G(d) = -2434.771955

B3LYP-D3(BJ)/def2-TZVPP/IEFPCM(propanonitrile) = -2435.849729
B3LYP-D3(BJ)/def2-TZVPP/IEFPCM(propanonitrile)//B3LYP-D3(BJ)/6-31G(d) Free Energy (Quasiharmonic) = -2435.471011

Frequencies (Top 3 out of 171)

1. 7.3347 cm^{-1}
2. 10.2298 cm^{-1}
3. 11.4734 cm^{-1}

B3LYP/6-31G(d) Molecular Geometry in Cartesian Coordinates

B	0.235241	-0.018837	-0.607947
O	0.601749	-0.520087	-1.863761
N	1.434629	0.400555	0.076483
C	2.609466	0.061814	-0.757193
S	1.639042	1.435694	1.407131
C	1.960684	-0.459712	-2.037196
O	2.517891	-0.792052	-3.045664
H	4.164460	-0.473680	0.626473
H	3.175009	0.967143	-1.011333
C	3.592302	-0.982328	-0.151960
C	2.955602	-2.219682	0.409068
C	2.625749	-3.449367	-0.277094
C	2.058743	-4.325848	0.690040
N	2.045137	-3.651210	1.894928
C	2.584466	-2.394439	1.721686
H	2.674984	-1.707288	2.551831
C	1.784539	-6.040151	-0.942598
C	2.766433	-3.906490	-1.600903
H	1.201541	-6.272995	1.127537
H	1.708988	-4.023195	2.769492
C	2.346513	-5.191658	-1.918494
H	1.463890	-7.039567	-1.223560
H	3.183943	-3.260594	-2.367695
C	1.633183	-5.619990	0.373250
H	2.448751	-5.550694	-2.938824
C	1.854345	3.051261	0.665397
O	2.901481	1.062151	2.048978
O	0.379633	1.428605	2.148826
C	2.182284	5.572290	-0.500613
C	0.728409	3.761954	0.236662
C	3.136466	3.584219	0.534348
C	3.288755	4.842444	-0.049842
C	0.902438	5.013816	-0.342731
H	-0.265045	3.346701	0.373084
H	3.993608	3.031824	0.904181
H	4.285316	5.264444	-0.149327
H	0.030230	5.573133	-0.671790
C	2.350916	6.926101	-1.146488
H	2.132055	6.878623	-2.220820
H	3.371874	7.301581	-1.031693
H	1.666501	7.662770	-0.710844
H	4.289573	-1.234149	-0.960467
C	-1.285264	-0.003795	-0.270117
C	-2.178427	0.067592	-1.355816
C	-1.825629	-0.137861	1.020401
C	-3.558654	0.042970	-1.157304
H	-1.790266	0.133075	-2.366747
C	-3.206793	-0.190426	1.213041
H	-1.168716	-0.191622	1.878122
C	-4.079412	-0.090439	0.129498
H	-5.151689	-0.121795	0.285498
C	-3.762986	-0.418504	2.595246
C	-4.490688	0.212398	-2.328721
F	-4.991365	0.128367	2.735231
F	-3.887385	-1.739174	2.865187
F	-2.969744	0.106468	3.550516
F	-3.969060	-0.299387	-3.463075
F	-4.749296	1.519893	-2.568238
F	-5.679920	-0.390830	-2.114486

NTOB catalyst 5

B3LYP/6-31G(d) = -2434.768195
B3LYP-D3(BJ)/def2-TZVPP/IEFPCM(propanonitrile) = -2435.849307
B3LYP-D3(BJ)/def2-TZVPP/IEFPCM(propanonitrile)//B3LYP-D3(BJ)/6-31G(d) Free Energy (Quasiharmonic) = -2435.470324

Frequencies (Top 3 out of 171)

1. 4.4594 cm^{-1}
2. 10.1786 cm^{-1}
3. 12.0340 cm^{-1}

B3LYP/6-31G(d) Molecular Geometry in Cartesian Coordinates

B	-0.239345	-0.991535	-1.216142
O	0.065916	-2.338500	-1.407692
N	0.845803	-0.179468	-1.715332
C	1.939862	-1.081662	-2.134687
S	0.713103	1.375294	-2.423077
C	1.305215	-2.464912	-1.995095
O	1.785829	-3.517517	-2.305321
H	3.677815	0.006922	-1.488945
H	2.188457	-0.909177	-3.185311
C	3.236228	-0.974167	-1.285729
C	3.070225	-1.178951	0.193770
C	3.186101	-2.420503	0.928501
C	2.977384	-2.114959	2.301405
N	2.742701	-0.756906	2.386574
C	2.802306	-0.202289	1.125135
H	2.669909	0.862017	0.987666
C	3.307545	-4.392547	2.916964
C	3.462398	-3.752678	0.568292
H	2.867038	-2.832336	4.349083
H	2.566819	-0.246856	3.238041
C	3.521335	-4.720377	1.562118
H	3.357529	-5.173922	3.670252
H	3.607465	-4.025062	-0.472557
C	3.032066	-3.087577	3.305438
H	3.729962	-5.751773	1.292194
C	1.394983	2.548401	-1.249973
O	1.602870	1.351641	-3.586485
O	-0.717150	1.651245	-2.543663
C	2.449945	4.406385	0.561848
C	0.540376	3.212527	-0.367770
C	2.764335	2.824274	-1.265253
C	3.280682	3.745404	-0.356087
C	1.076105	4.126637	0.537492
H	-0.527523	3.033666	-0.398699
H	3.409215	2.342936	-1.992556
H	4.345958	3.961771	-0.366546
H	0.408291	4.633751	1.229020
C	3.015582	5.425447	1.521149
H	4.049035	5.189791	1.794800
H	2.422126	5.484896	2.438976
H	3.018172	6.426101	1.069443
H	3.930357	-1.717335	-1.695713
C	-1.563761	-0.593431	-0.508177
C	-1.598938	0.454358	0.418559
C	-2.746696	-1.317341	-0.733664
C	-2.781014	0.797642	1.077011
H	-0.691146	1.003335	0.643854
C	-3.930002	-0.971277	-0.082183
H	-2.742798	-2.157835	-1.419934
C	-3.954594	0.091689	0.824475
H	-4.873515	0.354475	1.334948
C	-5.207299	-1.710614	-0.389951
C	-2.751403	1.942338	2.052592
F	-4.970432	-2.982613	-0.771668

F	-6.029086	-1.749911	0.682368
F	-5.893783	-1.115419	-1.390934
F	-2.348318	3.093278	1.449629
F	-1.873639	1.716198	3.057385
F	-3.953534	2.182506	2.607826

NTOB catalyst 6

B3LYP/6-31G(d) = -2434.768226
B3LYP-D3(BJ)/def2-TZVPP/IEFPCM(propanonitrile) = -2435.849197
B3LYP-D3(BJ)/def2-TZVPP/IEFPCM(propanonitrile)//B3LYP-D3(BJ)/6-31G(d) Free Energy (Quasiharmonic) = -2435.470052

Frequencies (Top 3 out of 171)

1. 6.6190 cm^{-1}
2. 10.0852 cm^{-1}
3. 11.8991 cm^{-1}

B3LYP/6-31G(d) Molecular Geometry in Cartesian Coordinates

B	-0.179563	-1.020851	-1.215349
O	0.183666	-2.355116	-1.391421
N	0.867962	-0.167728	-1.724648
C	2.000339	-1.025013	-2.135832
S	0.663770	1.371381	-2.450852
C	1.427283	-2.433298	-1.979367
O	1.952981	-3.467230	-2.278772
H	3.684634	0.150648	-1.504539
H	2.240133	-0.853885	-3.188655
C	3.291607	-0.848861	-1.291009
C	3.138586	-1.048757	0.190570
C	3.313762	-2.277490	0.935138
C	3.094440	-1.970564	2.306080
N	2.797062	-0.624240	2.380823
C	2.827838	-0.077918	1.114709
H	2.646718	0.978107	0.968950
C	3.531332	-4.225195	2.939364
C	3.650552	-3.598421	0.585083
H	3.022455	-2.675229	4.359885
H	2.600362	-0.115732	3.228649
C	3.756697	-4.554035	1.586639
H	3.619319	-4.997130	3.698876
H	3.805278	-3.872569	-0.453912
C	3.196620	-2.931215	3.317910
H	4.012137	-5.576891	1.324579
C	1.232993	2.592488	-1.267294
O	-0.775896	1.559214	-2.620642
O	1.591078	1.391696	-3.584644
C	2.108091	4.521817	0.565123
C	2.583463	2.948072	-1.234561
C	0.308501	3.211193	-0.423469
C	0.754978	4.160896	0.493232
C	3.010031	3.905090	-0.315754
H	3.282624	2.499669	-1.932217
H	-0.744681	2.967558	-0.491464
H	0.032912	4.629303	1.156874
H	4.060296	4.184349	-0.288947
C	2.576340	5.578565	1.536071
H	1.955173	5.598394	2.437170
H	2.522465	6.576471	1.081496
H	3.615595	5.414758	1.838559
H	4.020015	-1.561205	-1.696212
C	-1.518513	-0.666154	-0.510624
C	-2.685584	-1.408223	-0.758174
C	-1.582286	0.369068	0.428695
C	-3.882392	-1.090014	-0.117494
H	-2.662520	-2.231674	-1.464399
C	-2.779625	0.687839	1.072332
H	-0.687160	0.931479	0.671611
C	-3.938002	-0.033975	0.796000
H	-4.871859	0.218976	1.284006
C	-2.791146	1.845083	2.033331
C	-5.116559	-1.921093	-0.360344
F	-3.969982	1.981328	2.668023
F	-1.828677	1.717118	2.975597
F	-2.541391	3.020282	1.393905
F	-6.241788	-1.179078	-0.263596
F	-5.224361	-2.919605	0.545285
F	-5.105166	-2.491963	-1.582263

NTOB catalyst 7

B3LYP/6-31G(d) = -2434.768416
B3LYP-D3(BJ)/def2-TZVPP/IEFPCM(propanonitrile) = -2435.849098
B3LYP-D3(BJ)/def2-TZVPP/IEFPCM(propanonitrile)//B3LYP-D3(BJ)/6-31G(d) Free Energy (Quasiharmonic) = -2435.469459

Frequencies (Top 3 out of 171)

1. 8.0637 cm^{-1}
2. 12.3914 cm^{-1}
3. 15.5712 cm^{-1}

B3LYP/6-31G(d) Molecular Geometry in Cartesian Coordinates

B	-0.044189	0.328940	-1.620249
O	-0.841670	1.368902	-2.110808
N	-0.789968	-0.904392	-1.731711
C	-2.158068	-0.589153	-2.206153
S	-0.275791	-2.530649	-1.735454
C	-2.050678	0.891207	-2.553820
O	-2.874216	1.570984	-3.097372
H	-4.225603	-0.595166	-1.683480
H	-2.385371	-1.161689	-3.108685
C	-3.287424	-0.823102	-1.164186
C	-3.195672	-0.014562	0.100439
C	-3.826167	1.261654	0.365205
C	-3.488669	1.627745	1.696609
N	-2.689295	0.623357	2.205166
C	-2.519922	-0.356492	1.249408
H	-1.940886	-1.241110	1.472496
C	-4.760688	3.634831	1.528054
C	-4.653251	2.121091	-0.381415
H	-3.671614	3.071269	3.310062
H	-2.297027	0.602738	3.133425
C	-5.111443	3.292829	0.205609
H	-5.132884	4.560964	1.957263
H	-4.911172	1.884494	-1.409037
C	-3.944454	2.809352	2.290986
H	-5.746606	3.964103	-0.365455
C	-0.380074	-3.121770	-0.045175
O	-1.276704	-3.254497	-2.520432
O	1.136316	-2.525824	-2.119777
C	-0.557741	-4.102095	2.572610
C	-1.487182	-3.884968	0.332087
C	0.653388	-2.859939	0.858653
C	0.549844	-3.345387	2.160186
C	-1.569239	-4.364721	1.638031
H	-2.260313	-4.111598	-0.393812
H	1.534947	-2.306194	0.556050
H	1.352715	-3.143565	2.864672
H	-2.430062	-4.959759	1.931995
C	-0.636573	-4.655319	3.974872
H	-0.125380	-4.005604	4.692492
H	-0.158265	-5.641892	4.031421
H	-1.674385	-4.777555	4.300681
H	-3.310289	-1.895025	-0.944178
C	1.330061	0.731108	-0.997261
C	2.457509	-0.093603	-0.872227

C	1.419145	2.043726	-0.489015
C	3.611217	0.359640	-0.227714
H	2.443613	-1.091276	-1.294388
C	2.574383	2.494952	0.146636
H	0.575672	2.717085	-0.596171
C	3.677628	1.650376	0.288848
H	4.576336	1.998883	0.783935
C	2.616654	3.877979	0.743981
C	4.759348	-0.601567	-0.071562
F	2.201789	3.869981	2.033232
F	3.868904	4.385079	0.738499
F	1.821418	4.738860	0.077960
F	5.831118	-0.033029	0.517042
F	5.158985	-1.102426	-1.257851
F	4.394853	-1.662413	0.695566

NTOB catalyst 8

B3LYP/6-31G(d) = -2434.766797
B3LYP-D3(BJ)/def2-TZVPP/IEFPCM(propanonitrile) = -2435.848109
B3LYP-D3(BJ)/def2-TZVPP/IEFPCM(propanonitrile)//B3LYP-D3(BJ)/6-31G(d) Free Energy (Quasiharmonic) = -2435.468542

Frequencies (Top 3 out of 171)

1. 8.9101 cm^{-1}
2. 9.6516 cm^{-1}
3. 13.0883 cm^{-1}

B3LYP/6-31G(d) Molecular Geometry in Cartesian Coordinates

B	-0.284592	0.267786	-1.634419
O	-1.001439	1.259318	-2.312000
N	-1.088756	-0.921506	-1.566581
C	-2.320666	-0.717000	-2.370661
S	-0.612208	-2.500582	-1.116713
C	-2.185139	0.750472	-2.783044
O	-2.970049	1.390888	-3.425566
H	-3.875251	-2.090893	-1.818179
H	-2.259155	-1.309195	-3.293198
C	-3.707315	-1.016234	-1.735583
C	-3.939514	-0.602216	-0.311082
C	-4.147705	0.717451	0.248554
C	-4.379827	0.545500	1.643137
N	-4.313155	-0.809355	1.907295
C	-4.066757	-1.488807	0.731458
H	-3.979803	-2.566434	0.719006
C	-4.652294	2.893052	1.951171
C	-4.190815	2.022091	-0.280954
H	-4.804445	1.461482	3.565856
H	-4.510092	-1.241232	2.796706
C	-4.439127	3.090179	0.571999
H	-4.844304	3.749719	2.591243
H	-4.045539	2.195978	-1.342234
C	-4.629049	1.618685	2.504537
H	-4.474783	4.097989	0.167864
C	0.029328	-2.289820	0.543124
O	-1.842578	-3.296509	-1.103570
O	0.503083	-2.922099	-1.973424
C	1.044035	-2.109116	3.144064
C	-0.662028	-1.540864	1.498235
C	1.215111	-2.952446	0.866548
C	1.712745	-2.854561	2.164188
C	-0.143987	-1.452894	2.787387
H	-1.575934	-1.021797	1.234323
H	1.749065	-3.514769	0.108983
H	2.645023	-3.354749	2.411750
H	-0.669689	-0.855787	3.528267
C	1.579574	-2.024748	4.552760
H	1.145966	-2.811982	5.183699
H	1.335892	-1.063950	5.017601
H	2.666543	-2.150255	4.576309
H	-4.424818	-0.523311	-2.404805
C	1.133946	0.618325	-1.094490
C	1.350952	1.922826	-0.611726
C	2.217302	-0.272347	-1.101298
C	2.596538	2.307146	-0.118780
H	0.540867	2.644622	-0.628231
C	3.467277	0.119126	-0.614942
H	2.095827	-1.271329	-1.506193
C	3.662082	1.403843	-0.114893
H	4.633903	1.705157	0.257891
C	4.586117	-0.889318	-0.612408
C	2.788928	3.683806	0.462973
F	5.768458	-0.342269	-0.262615
F	4.336572	-1.895945	0.263108
F	4.740957	-1.462174	-1.824295
F	1.954054	4.586004	-0.092319
F	4.052241	4.128177	0.284790
F	2.553493	3.693049	1.796073

NTOB catalyst 9

B3LYP/6-31G(d) = -2434.770879
B3LYP-D3(BJ)/def2-TZVPP/IEFPCM(propanonitrile) = -2435.847248
B3LYP-D3(BJ)/def2-TZVPP/IEFPCM(propanonitrile)//B3LYP-D3(BJ)/6-31G(d) Free Energy (Quasiharmonic) = -2435.468211

Frequencies (Top 3 out of 171)

1. 10.5127 cm^{-1}
2. 13.5839 cm^{-1}
3. 14.7680 cm^{-1}

B3LYP/6-31G(d) Molecular Geometry in Cartesian Coordinates

B	0.218992	-1.053460	-0.024982
O	-0.337762	-2.107402	0.704456
N	-0.837733	-0.153348	-0.402581
C	-2.136843	-0.714449	0.042112
S	-0.772351	1.445557	-1.007587
C	-1.699152	-1.929519	0.858189
O	-2.380251	-2.673453	1.503714
H	-2.605936	-1.998488	-1.648008
H	-2.673497	-0.008765	0.681414
C	-3.064399	-1.143630	-1.136484
C	-4.461480	-1.462332	-0.700438
C	-5.504143	-0.506829	-0.407243
C	-6.651112	-1.247981	-0.011239
N	-6.309691	-2.585288	-0.066609
C	-4.998008	-2.705727	-0.475297
H	-4.537103	-3.679818	-0.562886
C	-7.900585	0.758143	0.295733
C	-5.581096	0.897292	-0.447424
H	-8.723054	-1.212984	0.640869
H	-6.916223	-3.354598	0.170266
C	-6.776046	1.513377	-0.095496
H	-8.820800	1.269838	0.564330
H	-4.720899	1.488624	-0.750007
C	-7.855191	-0.630769	0.341610
H	-6.848748	2.597243	-0.123731
C	0.070267	2.387331	0.260497
O	0.062806	1.437164	-2.209230
O	-2.166766	1.890335	-1.056736
C	1.352070	3.882529	2.247113
C	1.403896	2.759057	0.084388
C	-0.641466	2.768605	1.402612
C	0.005838	3.508651	2.386426
C	2.031762	3.503765	1.082422
H	1.939748	2.486405	-0.817409

H	-1.690755	2.512208	1.506021
H	-0.545603	3.811347	3.272948
H	3.068507	3.800117	0.946517
C	2.049328	4.663555	3.334282
H	1.379339	5.399684	3.790872
H	2.926454	5.192129	2.948958
H	2.392860	3.996310	4.135647
H	-3.073602	-0.311206	-1.845776
C	1.763068	-1.030806	-0.221439
C	2.549148	-1.750674	0.700131
C	2.420963	-0.351948	-1.257841
C	3.939097	-1.762510	0.604515
H	2.065269	-2.310558	1.493352
C	3.815149	-0.359551	-1.345969
H	1.845717	0.186829	-2.002443
C	4.581411	-1.058660	-0.417402
H	5.662444	-1.066589	-0.493113
C	4.464309	0.449242	-2.437630
C	4.763408	-2.483254	1.640070
F	5.798945	0.264294	-2.490555
F	3.958298	0.148567	-3.650658
F	4.250566	1.776897	-2.244103
F	4.097084	-3.526138	2.176430
F	5.101702	-1.660890	2.660593
F	5.916199	-2.956008	1.118069

NTOB catalyst 10

B3LYP/6-31G(d) = -2434.770588
B3LYP-D3(BJ)/def2-TZVPP/IEFPCM(propanonitrile) = -2435.846633
B3LYP-D3(BJ)/def2-TZVPP/IEFPCM(propanonitrile)//B3LYP-D3(BJ)/6-31G(d) Free Energy (Quasiharmonic) = -2435.467918

Frequencies (Top 3 out of 171)

1. 7.7248 cm^{-1}
2. 10.8147 cm^{-1}
3. 13.1452 cm^{-1}

B3LYP/6-31G(d) Molecular Geometry in Cartesian Coordinates

B	-0.628505	-0.929977	-0.203760
O	-0.196798	-1.758984	-1.245096
N	0.537156	-0.396695	0.459301
C	1.755674	-0.956897	-0.175870
S	0.699595	1.009378	1.411295
C	1.178586	-1.764056	-1.337187
O	1.770038	-2.367644	-2.186587
H	2.076449	-2.820969	0.898082
H	2.390462	-0.157573	-0.572106
C	2.602110	-1.867838	0.766263
C	3.997573	-2.083155	0.262247
C	5.122812	-1.203263	0.476139
C	6.232378	-1.767369	-0.211593
N	5.790429	-2.932139	-0.807544
C	4.452794	-3.113217	-0.523356
H	3.915465	-3.961141	-0.923956
C	7.641943	0.020613	0.494949
C	5.302805	-0.002832	1.188190
H	8.333718	-1.614665	-0.740875
H	6.352097	-3.551525	-1.370456
C	6.557377	0.595725	1.188606
H	8.610875	0.511962	0.515909
H	4.476866	0.441321	1.737628
C	7.496050	-1.168463	-0.210840
H	6.709969	1.520602	1.738449
C	0.848982	2.333032	0.215687
O	-0.557110	1.176205	2.137796
O	1.977735	0.888361	2.113596
C	1.072563	4.384441	-1.671874
C	-0.309850	2.925267	-0.295484
C	2.116179	2.757789	-0.188146
C	2.215126	3.780272	-1.131486
C	-0.187278	3.945134	-1.233787
H	-1.285957	2.604670	0.053409
H	3.006839	2.311754	0.241917
H	3.199314	4.117784	-1.445434
H	-1.084877	4.415473	-1.627266
C	1.186816	5.474362	-2.709843
H	0.448031	6.265263	-2.541839
H	1.010490	5.075049	-3.717158
H	2.181615	5.929752	-2.705950
H	2.625370	-1.376429	1.741815
C	-2.165626	-0.768422	-0.012620
C	-2.801527	-0.497462	1.211508
C	-2.975432	-0.989076	-1.143221
C	-4.193699	-0.440935	1.294162
H	-2.210711	-0.333437	2.102928
C	-4.365259	-0.912752	-1.060988
H	-2.513756	-1.236805	-2.093153
C	-4.981258	-0.640502	0.160592
H	-6.062324	-0.604255	0.232082
C	-5.203516	-1.080819	-2.301679
C	-4.852683	-0.109623	2.609219
F	-5.384204	0.100139	-2.938524
F	-4.627044	-1.920914	-3.186758
F	-6.431027	-1.565373	-2.014975
F	-4.134453	-0.561391	3.657343
F	-6.089220	-0.649203	2.696374
F	-4.993695	1.225810	2.768381

NTOB catalyst 11

B3LYP/6-31G(d) = -2434.767945
B3LYP-D3(BJ)/def2-TZVPP/IEFPCM(propanonitrile) = -2435.847707
B3LYP-D3(BJ)/def2-TZVPP/IEFPCM(propanonitrile)//B3LYP-D3(BJ)/6-31G(d) Free Energy (Quasiharmonic) = -2435.467636

Frequencies (Top 3 out of 171)

1. 11.3875 cm^{-1}
2. 11.9116 cm^{-1}
3. 15.8222 cm^{-1}

B3LYP/6-31G(d) Molecular Geometry in Cartesian Coordinates

B	0.165154	-1.726263	0.303872
O	0.002464	-3.002460	-0.244540
N	-0.966941	-1.415193	1.135511
C	-1.873710	-2.593218	1.148000
S	-0.961654	-0.446622	2.562103
C	-1.166526	-3.566556	0.205618
O	-1.551864	-4.647677	-0.143192
H	-3.815206	-1.748380	1.477486
H	-1.890158	-3.046045	2.146796
C	-3.337766	-2.358078	0.707946
C	-3.528316	-1.727356	-0.644883
C	-4.134971	-0.444520	-0.909461
C	-4.131826	-0.255731	-2.319793
N	-3.553733	-1.377316	-2.875481
C	-3.205233	-2.260036	-1.872406
H	-2.764531	-3.216828	-2.116059
C	-5.199517	1.864529	-2.092787
C	-4.705977	0.552710	-0.096326
H	-4.641341	1.019110	-4.003352
H	-3.430695	-1.545608	-3.861866
C	-5.227984	1.694821	-0.692591
H	-5.618475	2.766428	-2.530360
H	-4.743087	0.430475	0.983507
C	-4.654726	0.892750	-2.923833

H	-5.675373	2.466877	-0.072555
C	-0.646204	1.208196	1.977626
O	-2.330262	-0.540485	3.078769
O	0.169387	-0.859504	3.397512
C	-0.146026	3.824515	1.134974
C	0.477385	1.880369	2.462693
C	-1.541197	1.827018	1.102749
C	-1.278819	3.129288	0.684675
C	0.716374	3.184595	2.037357
H	1.149975	1.380046	3.150335
H	-2.419524	1.304880	0.739813
H	-1.967173	3.609618	-0.005655
H	1.595447	3.709483	2.400991
C	0.166991	5.210459	0.629267
H	0.922838	5.156434	-0.163628
H	0.571181	5.846294	1.424046
H	-0.719733	5.699268	0.214656
H	-3.810660	-3.347729	0.745346
C	1.462074	-0.944017	-0.071197
C	1.474323	0.399781	-0.476842
C	2.675849	-1.653003	-0.093847
C	2.661900	1.013506	-0.880733
H	0.553733	0.970661	-0.500517
C	3.864915	-1.032704	-0.476947
H	2.694533	-2.699461	0.192552
C	3.862711	0.303932	-0.874489
H	4.784943	0.783792	-1.182589
C	5.145966	-1.824933	-0.537062
C	2.672259	2.465904	-1.279932
F	6.224037	-1.045063	-0.302076
F	5.158150	-2.825799	0.367397
F	5.322808	-2.386450	-1.754387
F	3.572944	2.710068	-2.255562
F	3.001065	3.270592	-0.237863
F	1.465354	2.880341	-1.724976

NTOB catalyst 12

B3LYP/6-31G(d) = -2434.763104
B3LYP-D3(BJ)/def2-TZVPP/IEFPCM(propanonitrile) = -2435.844797
B3LYP-D3(BJ)/def2-TZVPP/IEFPCM(propanonitrile)//B3LYP-D3(BJ)/6-31G(d) Free Energy (Quasiharmonic) = -2435.46571

Frequencies (Top 3 out of 171)

1. 10.8738 cm^{-1}
2. 12.7228 cm^{-1}
3. 13.8774 cm^{-1}

B3LYP/6-31G(d) Molecular Geometry in Cartesian Coordinates

B	0.864564	-1.687960	-0.221656
O	0.829885	-3.009390	-0.660783
N	-0.475472	-1.244253	0.116108
C	-1.407820	-2.338601	-0.253157
S	-0.786228	-0.330428	1.555166
C	-0.464009	-3.490053	-0.609179
O	-0.756553	-4.616904	-0.888475
H	-2.702979	-3.048692	-1.780932
H	-2.028206	-2.612225	0.602452
C	-2.290918	-2.069258	-1.503047
C	-3.398830	-1.071776	-1.361077
C	-4.607034	-1.194000	-0.577089
C	-5.395829	-0.039792	-0.840275
N	-4.690917	0.740178	-1.737797
C	-3.502626	0.110814	-2.050497
H	-2.812347	0.557885	-2.753028
C	-7.113111	-0.815750	0.618923
C	-5.108530	-2.166915	0.306490
H	-7.237696	1.051075	-0.466197
H	-5.017996	1.600124	-2.149201
C	-6.351424	-1.970085	0.893377
H	-8.081747	-0.689811	1.094408
H	-4.536284	-3.063760	0.527453
C	-6.647915	0.163883	-0.250048
H	-6.745451	-2.715984	1.577625
C	-1.198378	1.343543	1.072472
O	-1.975808	-0.938276	2.153755
O	0.489970	-0.273673	2.271164
C	-1.841489	3.989511	0.431525
C	-2.526526	1.679812	0.812617
C	-0.190196	2.311894	1.047945
C	-0.516471	3.622249	0.712330
C	-2.833832	3.002345	0.494457
H	-3.305223	0.928473	0.874335
H	0.828227	2.049876	1.306436
H	0.273459	4.367889	0.677451
H	-3.869079	3.270367	0.299794
C	-2.185172	5.421943	0.100716
H	-2.106123	6.060102	0.990015
H	-3.206438	5.513280	-0.281468
H	-1.501536	5.832705	-0.650900
H	-1.630800	-1.765753	-2.325194
C	2.210868	-0.914090	-0.233190
C	2.260205	0.452534	-0.533512
C	3.423207	-1.584780	0.003388
C	3.474076	1.142338	-0.558253
H	1.344654	0.988438	-0.760073
C	4.635695	-0.897291	-0.021068
H	3.418271	-2.650784	0.205491
C	4.666795	0.472418	-0.295841
H	5.610765	1.004075	-0.320415
C	5.921484	-1.615956	0.300603
C	3.458664	2.623027	-0.826737
F	6.218656	-1.532109	1.616439
F	5.853966	-2.927006	-0.009799
F	6.966889	-1.087640	-0.373939
F	2.706399	2.925463	-1.908058
F	4.691742	3.124066	-1.030906
F	2.918219	3.305677	0.218803

NTOB catalyst 13

B3LYP/6-31G(d) = -2434.768514
B3LYP-D3(BJ)/def2-TZVPP/IEFPCM(propanonitrile) = -2435.844672
B3LYP-D3(BJ)/def2-TZVPP/IEFPCM(propanonitrile)//B3LYP-D3(BJ)/6-31G(d) Free Energy (Quasiharmonic) = -2435.465424

Frequencies (Top 3 out of 171)

1. 7.7034 cm^{-1}
2. 14.1701 cm^{-1}
3. 15.7130 cm^{-1}

B3LYP/6-31G(d) Molecular Geometry in Cartesian Coordinates

B	-0.411355	-1.116123	-0.559042
O	0.111733	-2.395266	-0.346531
N	0.690454	-0.195275	-0.684726
C	1.968247	-0.935522	-0.515821
S	0.734956	1.432360	-1.209782
C	1.489968	-2.379507	-0.385948
O	2.152943	-3.371390	-0.282514
H	2.234514	-0.822665	1.638867
H	2.591842	-0.827257	-1.406442
C	2.787589	-0.520527	0.741401
C	4.177782	-1.078366	0.756462
C	5.301470	-0.599989	-0.014530
C	6.412265	-1.425650	0.311217
N	5.972291	-2.353650	1.235124

C	4.633838	-2.143855	1.491679
H	4.097980	-2.786001	2.176879
C	7.819971	-0.199167	-1.169908
C	5.477389	0.442283	-0.943113
H	8.514797	-1.877635	0.005102
H	6.529972	-3.093018	1.632870
C	6.732179	0.630810	-1.509781
H	8.788353	-0.026465	-1.631476
H	4.644784	1.083405	-1.220970
C	7.676334	-1.236910	-0.255836
H	6.879472	1.430108	-2.230904
C	0.362496	2.445209	0.222292
O	2.127357	1.675676	-1.590725
O	-0.353065	1.597305	-2.174324
C	-0.189083	4.075555	2.431567
C	1.416926	3.003362	0.948371
C	-0.965770	2.717002	0.562393
C	-1.227389	3.524378	1.666366
C	1.132568	3.810794	2.047398
H	2.441735	2.832891	0.637964
H	-1.785451	2.334294	-0.034294
H	-2.259710	3.741043	1.928694
H	1.952915	4.251625	2.607799
C	-0.489547	4.926638	3.641203
H	-1.408850	5.505412	3.505362
H	0.326277	5.624594	3.853464
H	-0.627153	4.302041	4.533724
H	2.831790	0.571867	0.751013
C	-1.965931	-0.976078	-0.500781
C	-2.731388	0.009916	-1.140370
C	-2.648495	-1.930027	0.281728
C	-4.116183	0.069277	-0.964577
H	-2.247129	0.733750	-1.784998
C	-4.031009	-1.873802	0.447121
H	-2.089078	-2.719687	0.771349
C	-4.773944	-0.863949	-0.168328
H	-5.846826	-0.807161	-0.027038
C	-4.739878	-2.932941	1.251712
C	-4.864940	1.207737	-1.605112
F	-5.131779	-3.966965	0.474704
F	-3.947605	-3.441505	2.219412
F	-5.850433	-2.444160	1.847511
F	-4.588610	1.310694	-2.920575
F	-4.513275	2.391604	-1.038861
F	-6.201241	1.084062	-1.472868

NTOB catalyst 14

B3LYP/6-31G(d) = -2434.764502
B3LYP-D3(BJ)/def2-TZVPP/IEFPCM(propanonitrile) = -2435.843046
B3LYP-D3(BJ)/def2-TZVPP/IEFPCM(propanonitrile)//B3LYP-D3(BJ)/6-31G(d) Free Energy (Quasiharmonic) = -2435.464204

Frequencies (Top 3 out of 171)

1. 7.6356 cm^{-1}
2. 10.6237 cm^{-1}
3. 13.7432 cm^{-1}

B3LYP/6-31G(d) Molecular Geometry in Cartesian Coordinates

B	0.929863	-1.674937	-0.082634
O	0.889416	-2.970914	-0.598957
N	-0.362264	-1.336046	0.471429
C	-1.283174	-2.478582	0.233942
S	-0.565946	-0.322925	1.854647
C	-0.354793	-3.521688	-0.403756
O	-0.642600	-4.634291	-0.740749
H	-2.676518	-3.190931	-1.211405
H	-1.642623	-2.876196	1.186641
C	-2.466616	-2.224445	-0.732712
C	-3.740788	-1.675995	-0.148956
C	-4.746356	-0.961026	-0.901664
C	-5.826942	-0.692878	-0.016194
N	-5.489033	-1.234233	1.206767
C	-4.241088	-1.820021	1.122637
H	-3.793128	-2.262538	1.999574
C	-7.027092	0.434360	-1.740518
C	-4.836748	-0.516151	-2.232991
H	-7.786421	0.199796	0.271248
H	-6.035731	-1.164561	2.050905
C	-5.970644	0.176490	-2.638510
H	-7.902460	0.976419	-2.087396
H	-4.033244	-0.712524	-2.938372
C	-6.970668	0.004086	-0.419885
H	-6.049799	0.523100	-3.665191
C	-1.061287	1.282782	1.233218
O	-1.673492	-0.914072	2.609333
O	0.759005	-0.172574	2.457135
C	-1.797074	3.812416	0.292314
C	-0.155525	2.341830	1.327702
C	-2.336263	1.469564	0.694604
C	-2.690082	2.731683	0.225550
C	-0.528841	3.596004	0.847870
H	0.824768	2.184568	1.761554
H	-3.037218	0.644987	0.636148
H	-3.678050	2.875462	-0.204238
H	0.185432	4.413069	0.901958
C	-2.203202	5.180192	-0.200170
H	-2.896906	5.112377	-1.044435
H	-1.334617	5.766530	-0.515930
H	-2.709568	5.746316	0.592801
H	-2.108841	-1.561235	-1.530871
C	2.242688	-0.855319	-0.220084
C	3.480924	-1.521410	-0.223423
C	2.242098	0.534303	-0.392721
C	4.674086	-0.812445	-0.355851
H	3.511303	-2.601598	-0.125893
C	3.435902	1.244749	-0.529384
H	1.303684	1.075567	-0.433084
C	4.657915	0.576520	-0.504289
H	5.585338	1.125947	-0.614766
C	3.372058	2.742630	-0.666669
C	5.995228	-1.535242	-0.287915
F	4.569991	3.284874	-0.959524
F	2.504064	3.116230	-1.632967
F	2.939332	3.324668	0.482734
F	6.467203	-1.584854	0.977967
F	6.937489	-0.919644	-1.035968
F	5.892875	-2.808022	-0.723884

NTOB-benzaldehyde complex 1

B3LYP/6-31G(d) = -2780.359943
B3LYP-D3(BJ)/def2-TZVPP/IEFPCM(propanonitrile) = -2781.620291
B3LYP-D3(BJ)/def2-TZVPP/IEFPCM(propanonitrile)//B3LYP-D3(BJ)/6-31G(d) Free Energy (Quasiharmonic) = -2781.133221

Frequencies (Top 3 out of 213)

1. 5.6740 cm^{-1}
2. 12.8037 cm^{-1}
3. 15.6463 cm^{-1}

B3LYP/6-31G(d) Molecular Geometry in Cartesian Coordinates

B	0.114468	-0.138739	1.020653
O	0.276307	-0.770995	2.324027
N	0.527619	-1.243254	0.038999
C	0.804778	-2.488456	0.777396

S	0.132825	-1.284883	-1.581787
C	0.692198	-2.044130	2.241777
O	0.939621	-2.741278	3.196888
H	2.163075	-3.548705	-0.511884
H	0.008670	-3.228309	0.606794
C	2.157558	-3.192574	0.519918
C	3.396420	-2.381933	0.792677
C	4.272437	-1.761781	-0.175677
C	5.358433	-1.184085	0.544111
N	5.131960	-1.428077	1.882243
C	3.965018	-2.154582	2.024283
H	3.620061	-2.463296	3.001301
C	6.381278	-0.450039	-1.482387
C	4.258227	-1.654497	-1.580136
H	7.242455	-0.104779	0.472744
H	5.747379	-1.169183	2.637571
C	5.309854	-1.002718	-2.216700
H	7.195023	0.040715	-2.009996
H	3.435550	-2.071401	-2.153834
C	6.417140	-0.528666	-0.093920
H	5.312931	-0.926073	-3.300719
C	-1.601718	-1.717578	-1.739924
O	0.899715	-2.381028	-2.192182
O	0.297600	0.099696	-2.053118
C	-4.323423	-2.374420	-1.853059
C	-2.003625	-3.039543	-1.527967
C	-2.536627	-0.731072	-2.060466
C	-3.886071	-1.068717	-2.117163
C	-3.360210	-3.354834	-1.578513
H	-1.266547	-3.815457	-1.349974
H	-2.212214	0.282734	-2.260237
H	-4.613105	-0.298413	-2.361876
H	-3.674851	-4.380590	-1.403800
C	-5.796585	-2.700630	-1.839699
H	-6.256161	-2.332950	-0.913476
H	-6.321554	-2.224570	-2.675279
H	-5.972848	-3.779237	-1.895054
H	2.147304	-4.075318	1.170054
C	-1.299783	0.610761	0.870204
C	-1.499558	1.746181	0.074564
C	-2.416832	0.092409	1.544179
C	-2.763622	2.326169	-0.063102
H	-0.668014	2.179580	-0.470347
C	-3.682534	0.665937	1.407351
H	-2.297241	-0.769710	2.192434
C	-3.864984	1.789050	0.600167
H	-4.844716	2.240660	0.496579
C	-4.874398	0.026191	2.065785
C	-2.945103	3.468444	-1.022409
F	-5.440502	-0.912286	1.261551
F	-5.842553	0.926751	2.347253
F	-4.548610	-0.596206	3.217302
F	-4.059139	4.187040	-0.764271
F	-3.054818	3.017963	-2.299880
F	-1.896995	4.321425	-1.002473
O	1.203334	1.047650	1.016603
C	2.159657	1.156475	0.213396
C	3.038240	2.299940	0.235059
C	2.837878	3.363354	1.140697
C	4.103502	2.338672	-0.684697
C	3.695985	4.452503	1.116553
H	2.010584	3.313946	1.841060
C	4.959824	3.435612	-0.699020
H	4.255586	1.508676	-1.368713
C	4.754300	4.487888	0.196669
H	3.548314	5.277858	1.806389
H	5.784541	3.470489	-1.403940
H	5.421915	5.345231	0.181376
H	2.324290	0.368708	-0.523313

NTOB-benzaldehyde complex 2

B3LYP/6-31G(d) = -2780.35483
B3LYP-D3(BJ)/def2-TZVPP/IEFPCM(propanonitrile) = -2781.618452
B3LYP-D3(BJ)/def2-TZVPP/IEFPCM(propanonitrile)//B3LYP-D3(BJ)/6-31G(d) Free Energy (Quasiharmonic) = -2781.132536

Frequencies (Top 3 out of 213)

1. 11.0587 cm^{-1}
2. 13.3660 cm^{-1}
3. 14.7861 cm^{-1}

B3LYP/6-31G(d) Molecular Geometry in Cartesian Coordinates

B	0.116060	-0.074510	0.728300
O	0.348810	-0.522060	2.098430
N	0.261610	-1.350000	-0.086910
C	0.540780	-2.492700	0.793600
S	-0.304840	-1.596410	-1.634210
C	0.576470	-1.851400	2.183750
O	0.797620	-2.428330	3.219660
H	1.795600	-3.699090	-0.471150
H	-0.293820	-3.209580	0.777330
C	1.848000	-3.287950	0.539650
C	3.135750	-2.533720	0.731760
C	3.914870	-1.831500	-0.265100
C	5.084830	-1.342600	0.381040
N	4.997680	-1.704030	1.712430
C	3.840270	-2.437910	1.908160
H	3.595720	-2.836190	2.883260
C	5.889010	-0.415430	-1.662160
C	3.747480	-1.587800	-1.641280
H	6.971700	-0.278351	0.206370
H	5.736830	-1.605010	2.390990
C	4.733520	-0.884440	-2.322330
H	6.645260	0.124509	-2.225910
H	2.861580	-1.945450	-2.157380
C	6.080500	-0.636480	-0.303220
H	4.616500	-0.695970	-3.386100
C	-1.994550	-2.186710	-1.470190
O	0.467380	-2.703930	-2.217460
O	-0.333980	-0.279370	-2.276960
C	-4.613880	-3.102870	-1.074970
C	-2.237030	-3.556930	-1.352530
C	-3.048290	-1.269310	-1.419690
C	-4.344540	-1.732590	-1.216330
C	-3.544380	-4.003140	-1.155460
H	-1.416600	-4.261350	-1.438700
H	-2.851930	-0.211170	-1.546530
H	-5.159760	-1.016460	-1.154710
H	-3.734290	-5.069770	-1.064750
C	-6.028310	-3.585210	-0.858790
H	-6.056340	-4.651090	-0.612890
H	-6.639550	-3.434410	-1.757730
H	-6.513330	-3.035370	-0.043970
H	1.816770	-4.131010	1.239640
C	-1.162570	0.883870	0.590520
C	-1.244899	1.942110	-0.326690
C	-2.277390	0.649660	1.409400
C	-2.392349	2.733450	-0.421240
H	-0.409709	2.146730	-0.985710
C	-3.435679	1.423620	1.302090
H	-2.244610	-0.147210	2.145530
C	-3.498359	2.475410	0.388660
H	-4.388219	3.090260	0.317160
C	-4.653060	1.074700	2.113200
C	-2.463589	3.828820	-1.450990
F	-5.449060	0.192880	1.452830
F	-5.415669	2.159090	2.377690
F	-4.334910	0.502510	3.293220

F	-3.304979	4.819720	-1.075980
F	-2.908349	3.367660	-2.642590
F	-1.253579	4.390040	-1.674490
O	1.376850	0.872500	0.317730
C	2.338441	1.134440	1.070610
C	3.420101	1.998940	0.666760
C	3.499991	2.504540	-0.646570
C	4.391741	2.349800	1.623300
C	4.539581	3.358850	-0.986880
H	2.753321	2.207570	-1.375270
C	5.425651	3.211240	1.275610
H	4.322101	1.948670	2.631140
C	5.496371	3.714190	-0.027570
H	4.611801	3.750080	-1.996900
H	6.173461	3.492630	2.010650
H	6.305331	4.386540	-0.300020
H	2.342830	0.706610	2.078910

NTOB-benzaldehyde complex 3

B3LYP/6-31G(d) = -2780.356906
B3LYP-D3(BJ)/def2-TZVPP/IEFPCM(propanonitrile) = -2781.618193
B3LYP-D3(BJ)/def2-TZVPP/IEFPCM(propanonitrile)//B3LYP-D3(BJ)/6-31G(d) Free Energy (Quasiharmonic) = -2781.130951

Frequencies (Top 3 out of 213)

1. 11.9701 cm^{-1}
2. 14.3960 cm^{-1}
3. 16.5099 cm^{-1}

B3LYP/6-31G(d) Molecular Geometry in Cartesian Coordinates

B	0.215285	-0.196971	0.797958
O	0.565042	-0.896494	2.026982
N	0.516594	-1.231208	-0.292657
C	0.914643	-2.507740	0.321825
S	-0.021211	-1.154731	-1.868318
C	1.003406	-2.146350	1.810763
O	1.390460	-2.890887	2.679689
H	1.949635	-3.726536	-1.113960
H	0.106342	-3.250363	0.243061
C	2.206013	-3.195182	-0.195721
C	3.394871	-2.317299	-0.468922
C	4.366774	-1.783616	0.464263
C	5.338860	-1.078861	-0.299940
N	4.949738	-1.148779	-1.627424
C	3.802223	-1.922535	-1.718998
H	3.339346	-2.121843	-2.675356
C	6.579468	-0.545868	1.664369
C	4.527296	-1.851604	1.861759
H	7.182416	0.070160	-0.320293
H	5.540708	-0.915839	-2.411317
C	5.627883	-1.234344	2.443574
H	7.431320	-0.077448	2.149935
H	3.801007	-2.376541	2.474471
C	6.447499	-0.457145	0.283141
H	5.759795	-1.284202	3.520849
C	-1.775536	-1.531738	-1.887248
O	0.654390	-2.223546	-2.618520
O	0.137511	0.255076	-2.263450
C	-4.517051	-2.097757	-1.767472
C	-2.201353	-2.845314	-1.669043
C	-2.700941	-0.508874	-2.099219
C	-4.061373	-0.800878	-2.041146
C	-3.566428	-3.115707	-1.604567
H	-1.478411	-3.648487	-1.570541
H	-2.361195	0.499541	-2.299551
H	-4.778534	0.000928	-2.197972
H	-3.899121	-4.134736	-1.423814
C	-5.991862	-2.378757	-1.617173
H	-6.587747	-1.795618	-2.327477
H	-6.219699	-3.438700	-1.767335
H	-6.324805	-2.106120	-0.607514
H	2.455122	-3.949315	0.561186
C	-1.223172	0.514802	0.870938
C	-1.545319	1.689870	0.179449
C	-2.238332	-0.081954	1.634596
C	-2.832133	2.232599	0.226431
H	-0.794269	2.183084	-0.428225
C	-3.526025	0.456424	1.684698
H	-2.020064	-0.977839	2.207283
C	-3.832556	1.619043	0.977809
H	-4.829643	2.041707	1.017421
C	-4.610578	-0.262642	2.439882
C	-3.161748	3.417079	-0.636771
F	-5.207237	-1.210621	1.669288
F	-5.589018	0.576525	2.846443
F	-4.135993	-0.896357	3.532084
F	-2.137516	4.294957	-0.713011
F	-4.244917	4.092356	-0.196202
F	-3.434585	3.027940	-1.911827
O	1.263754	1.028353	0.721858
C	2.190014	1.144671	-0.112988
C	3.032240	2.316785	-0.142792
C	2.847636	3.374125	0.771662
C	4.040111	2.394321	-1.122003
C	3.665886	4.492301	0.700122
H	2.065600	3.296332	1.519611
C	4.854159	3.520133	-1.188370
H	4.174244	1.567415	-1.813113
C	4.665929	4.564911	-0.279195
H	3.531285	5.310865	1.400491
H	5.631982	3.588043	-1.942719
H	5.302100	5.444241	-0.331873
H	2.352358	0.341137	-0.835121

NTOB-benzaldehyde complex 4

B3LYP/6-31G(d) = -2780.351905
B3LYP-D3(BJ)/def2-TZVPP/IEFPCM(propanonitrile) = -2781.616215
B3LYP-D3(BJ)/def2-TZVPP/IEFPCM(propanonitrile)//B3LYP-D3(BJ)/6-31G(d) Free Energy (Quasiharmonic) = -2781.129154

Frequencies (Top 3 out of 213)

1. 15.7751 cm^{-1}
2. 19.3300 cm^{-1}
3. 21.3324 cm^{-1}

B3LYP/6-31G(d) Molecular Geometry in Cartesian Coordinates

B	-0.387934	0.109061	1.024600
O	-0.334036	-0.371319	2.395142
N	-1.055526	-1.059863	0.284036
C	-1.066054	-2.249382	1.149801
S	-1.860429	-1.049371	-1.146419
C	-0.703155	-1.659184	2.517973
O	-0.702987	-2.267358	3.560449
H	-0.367728	-3.768322	-0.213288
H	-2.073903	-2.679074	1.216875
C	-0.072928	-3.382180	0.766740
C	1.377548	-2.980704	0.756825
C	2.200418	-2.675913	-0.391506
C	3.489258	-2.326201	0.098444
N	3.450394	-2.435990	1.474029
C	2.179723	-2.811346	1.862106
H	1.938467	-2.936562	2.908899
C	4.286557	-1.934981	-2.113497
C	1.977126	-2.652694	-1.780518

H	5.512001	-1.678747	-0.346340
H	4.160833	-2.079761	2.095221
C	3.018067	-2.281972	-2.623317
H	5.076562	-1.641407	-2.799326
H	0.998628	-2.890835	-2.185807
C	4.539624	-1.953753	-0.746756
H	2.850831	-2.248134	-3.696324
C	-3.576406	-1.421241	-0.734528
O	-1.417724	-2.137724	-2.026870
O	-1.787205	0.345204	-1.635843
C	-6.275511	-1.961288	-0.196690
C	-4.254800	-2.376079	-1.492060
C	-4.229425	-0.733738	0.292080
C	-5.567724	-1.010793	0.555524
C	-5.597281	-2.637541	-1.218120
H	-3.728661	-2.908638	-2.276486
H	-3.691556	-0.006472	0.891503
H	-6.071945	-0.485483	1.363252
H	-6.123269	-3.385771	-1.805817
C	-7.734428	-2.232965	0.082055
H	-8.039069	-3.213461	-0.296617
H	-8.373570	-1.481745	-0.400566
H	-7.950345	-2.200392	1.155392
O	-1.490518	1.303886	1.114812
C	-1.560334	2.244701	0.290044
H	-0.778801	2.350491	-0.464389
H	-0.243934	-4.183724	1.495860
C	-2.619287	3.225533	0.327526
C	-3.586850	3.228582	1.352804
C	-2.659308	4.201305	-0.686031
C	-4.580627	4.197247	1.354208
H	-3.531527	2.480584	2.137060
C	-3.663045	5.164233	-0.681765
H	-1.910479	4.188450	-1.473416
C	-4.619738	5.160934	0.337182
H	-5.325776	4.211150	2.143758
H	-3.701696	5.914568	-1.465160
H	-5.401181	5.915779	0.343076
C	1.017035	0.694450	0.508945
C	1.331713	0.913969	-0.843140
C	2.030845	0.927050	1.452087
C	2.597006	1.362358	-1.230909
H	0.592943	0.708530	-1.611222
C	3.306003	1.342915	1.062907
H	1.827805	0.747248	2.502442
C	3.595034	1.573153	-0.281592
H	4.584889	1.892470	-0.586195
C	4.403941	1.437948	2.083481
C	2.880676	1.651469	-2.680254
F	3.943918	1.785387	3.303411
F	5.045287	0.241637	2.240656
F	2.147017	0.883671	-3.509526
F	5.353952	2.331898	1.734738
F	4.182883	1.463046	-2.989875
F	2.592572	2.944359	-2.988399

NTOB-benzaldehyde complex 5

B3LYP/6-31G(d) = -2780.350221
B3LYP-D3(BJ)/def2-TZVPP/IEFPCM(propanonitrile) = -2781.614482
B3LYP-D3(BJ)/def2-TZVPP/IEFPCM(propanonitrile)//B3LYP-D3(BJ)/6-31G(d) Free Energy (Quasiharmonic) = -2781.128216

Frequencies (Top 3 out of 213)

1. 9.7607 cm^{-1}
2. 13.6418 cm^{-1}
3. 13.9951 cm^{-1}

B3LYP/6-31G(d) Molecular Geometry in Cartesian Coordinates

B	-0.422182	0.136130	0.714832
O	-0.498484	0.036375	2.168557
N	-0.933011	-1.223718	0.248698
C	-0.996111	-2.146067	1.393422
S	-1.637476	-1.646898	-1.186462
C	-0.816094	-1.209166	2.589502
O	-0.908010	-1.517462	3.751632
H	-0.120472	-3.933997	0.571634
H	-1.988497	-2.609505	1.477807
C	0.076609	-3.270905	1.418154
C	1.497816	-2.780538	1.382231
C	2.364645	-2.678887	0.230133
C	3.596201	-2.130281	0.681319
N	3.485347	-1.933195	2.043479
C	2.220803	-2.307358	2.452988
H	1.928847	-2.217214	3.490730
C	4.492454	-2.182698	-1.525853
C	2.220864	-2.985958	-1.135600
H	5.590268	-1.434148	0.184621
H	4.139288	-1.400929	2.596688
C	3.282425	-2.734515	-1.995992
H	5.300696	-1.991383	-2.226770
H	1.285977	-3.386941	-1.515000
C	4.666277	-1.872956	-0.182246
H	3.177220	-2.957587	-3.054189
C	-3.408269	-1.736063	-0.857880
O	-1.230769	-3.014348	-1.534734
O	-1.419380	-0.528375	-2.112726
C	-6.159509	-1.870959	-0.321081
C	-3.988858	-2.959188	-0.516125
C	-4.190160	-0.582365	-0.949337
C	-5.554379	-0.657074	-0.680365
C	-5.355782	-3.015595	-0.244197
H	-3.378863	-3.855673	-0.488637
H	-3.733064	0.354154	-1.248858
H	-6.163714	0.240516	-0.758531
H	-5.805548	-3.968644	0.023364
C	-7.645371	-1.947887	-0.062359
H	-7.890195	-2.758842	0.631031
H	-8.197178	-2.136548	-0.992772
H	-8.028916	-1.012381	0.358331
O	-1.532569	1.269698	0.293446
C	-2.087791	2.028269	1.115555
H	-1.894304	1.872173	2.183061
H	-0.110522	-3.844189	2.334559
C	-2.963298	3.099004	0.701331
C	-3.598047	3.873870	1.690618
C	-3.175596	3.377040	-0.665609
C	-4.445115	4.912229	1.318241
H	-3.423300	3.654517	2.740851
C	-4.019910	4.417347	-1.027678
H	-2.664269	2.778753	-1.413398
C	-4.653951	5.181178	-0.037910
H	-4.938680	5.512287	2.076263
H	-4.185669	4.642316	-2.076705
H	-5.312620	5.994951	-0.328348
C	0.967765	0.739324	0.191574
C	1.302394	0.840290	-1.167850
C	1.928418	1.146272	1.129584
C	2.550080	1.324338	-1.568418
H	0.591664	0.513263	-1.919864
C	3.182176	1.614812	0.729805
H	1.702779	1.072769	2.188006
C	3.499797	1.711184	-0.624436
H	4.472984	2.068957	-0.938993
C	4.227636	1.923979	1.761582
C	2.853484	1.456324	-3.036577
F	3.692209	2.372449	2.917970
F	4.962744	0.817171	2.087269
F	2.354478	0.427952	-3.750905

F	5.113629	2.849536	1.336607
F	4.183099	1.514593	-3.278963
F	2.313214	2.589671	-3.554136

NTOB-benzaldehyde complex 6

B3LYP/6-31G(d) = -2780.348411
B3LYP-D3(BJ)/def2-TZVPP/IEFPCM(propanonitrile) = -2781.61375
B3LYP-D3(BJ)/def2-TZVPP/IEFPCM(propanonitrile)//B3LYP-D3(BJ)/6-31G(d) Free Energy (Quasiharmonic) = -2781.127562

Frequencies (Top 3 out of 213)

1. 8.3933 cm^{-1}
2. 13.8340 cm^{-1}
3. 14.0142 cm^{-1}

B3LYP/6-31G(d) Molecular Geometry in Cartesian Coordinates

B	0.176597	-1.120063	-0.647568
O	-0.120155	-2.048310	-1.728509
N	-0.387033	-1.777713	0.593299
C	-1.010180	-3.065976	0.216784
S	0.314410	-1.774782	2.126186
C	-0.853611	-3.102898	-1.304218
O	-1.321482	-3.931493	-2.045869
H	-2.519947	-3.325823	1.717787
H	-0.428249	-3.907233	0.620512
C	-2.485543	-3.279285	0.627272
C	-3.464118	-2.255249	0.121897
C	-4.014365	-1.139983	0.856121
C	-4.933054	-0.475738	-0.003133
N	-4.917508	-1.145782	-1.210677
C	-4.052872	-2.225535	-1.120399
H	-3.924654	-2.903893	-1.952928
C	-5.472368	1.097458	1.706205
C	-3.839339	-0.657408	2.166616
H	-6.368116	1.135532	-0.262878
H	-5.551441	-0.977356	-1.976204
C	-4.568087	0.453336	2.576942
H	-6.033474	1.958507	2.059709
H	-3.144645	-1.148912	2.842374
C	-5.666491	0.643667	0.406398
H	-4.447480	0.828904	3.589556
C	0.637017	-0.038974	2.423284
O	1.605250	-2.480930	2.117681
O	-0.726312	-2.243806	3.056153
C	1.189271	2.649543	2.963771
C	-0.405998	0.888447	2.417665
C	1.942737	0.347381	2.730073
C	2.208744	1.687027	2.997363
C	-0.117973	2.227123	2.682271
H	-1.422400	0.574315	2.204331
H	2.732077	-0.396064	2.736279
H	3.227741	1.994531	3.216056
H	-0.924890	2.956384	2.676214
C	1.510460	4.105413	3.199092
H	0.605008	4.718835	3.236644
H	2.148973	4.487907	2.393609
H	2.055387	4.247793	4.139666
H	-2.756579	-4.271564	0.248025
C	1.646193	-0.489027	-0.731325
C	1.914899	0.884780	-0.675209
C	2.737886	-1.353682	-0.914372
C	3.216068	1.379834	-0.799602
H	1.103168	1.588507	-0.529981
C	4.039144	-0.864898	-1.039036
H	2.572818	-2.424446	-0.969266
C	4.286742	0.507528	-0.984814
H	5.295159	0.888430	-1.100128
C	5.197661	-1.816153	-1.186393
C	3.470230	2.856817	-0.679493
F	5.749594	-2.118733	0.010749
F	6.184065	-1.285179	-1.946868
F	4.827387	-2.980975	-1.758382
F	3.705032	3.228283	0.608097
F	2.415493	3.590987	-1.102165
F	4.548330	3.247640	-1.392771
O	-0.843985	0.186933	-0.930198
C	-1.533198	0.295155	-1.964052
C	-2.408787	1.419696	-2.206434
C	-2.591799	2.429280	-1.241030
C	-3.073070	1.495046	-3.445125
C	-3.427322	3.501729	-1.522459
H	-2.080441	2.349482	-0.287631
C	-3.904797	2.574790	-3.721442
H	-2.924410	0.710482	-4.182872
C	-4.079574	3.575305	-2.759971
H	-3.577221	4.282016	-0.782820
H	-4.413306	2.642063	-4.678299
H	-4.729492	4.419085	-2.975047
H	-1.458025	-0.490870	-2.723678

NTOB-benzaldehyde complex 7

B3LYP/6-31G(d) = -2780.349333
B3LYP-D3(BJ)/def2-TZVPP/IEFPCM(propanonitrile) = -2781.613755
B3LYP-D3(BJ)/def2-TZVPP/IEFPCM(propanonitrile)//B3LYP-D3(BJ)/6-31G(d) Free Energy (Quasiharmonic) = -2781.127303

Frequencies (Top 3 out of 213)

1. 7.5706 cm^{-1}
2. 13.6251 cm^{-1}
3. 15.0882 cm^{-1}

B3LYP/6-31G(d) Molecular Geometry in Cartesian Coordinates

B	0.042877	-0.977121	-0.494263
O	-0.513158	-1.817051	-1.546575
N	-0.379352	-1.661645	0.793041
C	-1.129333	-2.892670	0.471276
S	0.469800	-1.712619	2.242261
C	-1.217764	-2.858598	-1.054572
O	-1.825331	-3.641176	-1.743927
H	-2.344357	-3.367124	2.171149
H	-0.535793	-3.783347	0.725620
C	-2.518956	-3.067716	1.135967
C	-3.426662	-1.871385	1.122930
C	-4.326879	-1.425647	0.082483
C	-5.017803	-0.283074	0.584099
N	-4.534178	-0.039372	1.852614
C	-3.595586	-1.003767	2.173821
H	-3.106287	-1.010295	3.138322
C	-6.297418	-0.106695	-1.421739
C	-4.644293	-1.891327	-1.209666
H	-6.527088	1.243146	0.253655
H	-4.894933	0.652946	2.490493
C	-5.621577	-1.229494	-1.945289
H	-7.067527	0.379472	-2.014966
H	-4.133793	-2.756771	-1.621409
C	-6.003218	0.381602	-0.152882
H	-5.881745	-1.592578	-2.936074
C	0.959229	-0.006292	2.480587
O	1.691985	-2.521892	2.115861
O	-0.501879	-2.081648	3.285783
C	1.771209	2.636208	2.912135
C	-0.004377	0.995904	2.599741
C	2.317831	0.284182	2.610453
C	2.713341	1.601336	2.824375

C	0.410662	2.310815	2.808929
H	-1.058280	0.751880	2.514766
H	3.044733	-0.515698	2.522797
H	3.771803	1.833831	2.902619
H	-0.333937	3.098732	2.899203
C	2.227000	4.064541	3.086226
H	2.870867	4.174123	3.966838
H	1.380867	4.749274	3.199793
H	2.811924	4.384841	2.215595
H	-2.992260	-3.914126	0.623389
C	1.553036	-0.513613	-0.770835
C	2.517253	-1.498242	-1.044763
C	1.975710	0.821186	-0.789173
C	3.841529	-1.161207	-1.326399
H	2.233084	-2.545280	-1.044255
C	3.302236	1.165233	-1.066130
H	1.266814	1.614525	-0.582515
C	4.243683	0.175767	-1.339267
H	5.269363	0.438552	-1.571841
C	3.722815	2.607238	-1.008727
C	4.866622	-2.237793	-1.568997
F	4.796111	2.859434	-1.787208
F	4.063876	2.985154	0.254173
F	2.731637	3.441650	-1.399083
F	5.801350	-1.841491	-2.464774
F	4.309240	-3.372746	-2.039599
F	5.528476	-2.560143	-0.434002
O	-0.828951	0.434198	-0.568082
C	-1.791375	0.626197	-1.342085
C	-2.471867	1.897611	-1.426612
C	-2.065936	3.000846	-0.646422
C	-3.534917	2.028175	-2.339543
C	-2.716230	4.217412	-0.791126
H	-1.247502	2.881929	0.056454
C	-4.180678	3.253193	-2.478425
H	-3.853709	1.166840	-2.918697
C	-3.769488	4.343649	-1.708653
H	-2.407496	5.073812	-0.199234
H	-5.001293	3.358699	-3.181080
H	-4.271055	5.301043	-1.820868
H	-2.113184	-0.191448	-1.994557

NTOB-benzaldehyde complex 8

B3LYP/6-31G(d) = -2780.350343
B3LYP-D3(BJ)/def2-TZVPP/IEFPCM(propanonitrile) = -2781.613835
B3LYP-D3(BJ)/def2-TZVPP/IEFPCM(propanonitrile)//B3LYP-D3(BJ)/6-31G(d) Free Energy (Quasiharmonic) = -2781.127229

Frequencies (Top 3 out of 213)

1. 13.8227 cm^{-1}
2. 15.8164 cm^{-1}
3. 18.3137 cm^{-1}

B3LYP/6-31G(d) Molecular Geometry in Cartesian Coordinates

B	0.417935	0.130375	-0.715120
O	0.489643	0.021603	-2.167896
N	0.925899	-1.227954	-0.242309
C	0.993741	-2.155487	-1.382745
S	1.624920	-1.644794	1.197590
C	0.812916	-1.224629	-2.583042
O	0.908217	-1.537114	-3.743738
H	0.120055	-3.942517	-0.555952
H	1.987617	-2.616467	-1.463136
C	-0.076045	-3.283418	-1.405805
C	-1.498227	-2.795877	-1.376350
C	-2.366381	-2.686608	-0.226071
C	-3.596762	-2.139919	-0.681814
N	-3.484523	-1.952410	-2.045280
C	-2.219902	-2.330251	-2.451380
H	-1.926957	-2.247369	-3.489444
C	-4.493224	-2.173026	1.525501
C	-2.224291	-2.985981	1.141704
H	-5.588775	-1.433905	-0.190253
H	-4.140176	-1.428209	-2.603997
C	-3.285498	-2.726420	1.999494
H	-5.299148	-1.971409	2.225950
H	-1.290615	-3.386839	1.524116
C	-4.666441	-1.873407	0.179866
H	-3.181423	-2.941374	3.059421
C	3.397054	-1.734142	0.876132
O	1.218080	-3.011057	1.549942
O	1.402669	-0.522641	2.118237
C	6.150591	-1.869129	0.351221
C	3.979527	-2.957777	0.539376
C	4.178168	-0.580038	0.968642
C	5.543545	-0.654762	0.705530
C	5.347589	-3.014230	0.273295
H	3.370040	-3.854590	0.511300
H	3.719368	0.356801	1.264483
H	6.152218	0.243207	0.784508
H	5.798836	-3.967670	0.009640
C	7.637557	-1.946105	0.098909
H	7.885402	-2.757386	-0.593036
H	8.185381	-2.134318	1.031760
H	8.022860	-1.010782	-0.320577
O	1.536968	1.263773	-0.304491
C	2.095735	2.009840	-1.135422
H	1.899726	1.843271	-2.200909
H	0.115587	-3.859809	-2.319329
C	2.978476	3.080072	-0.734926
C	3.617154	3.838454	-1.734373
C	3.193945	3.373942	0.628141
C	4.471345	4.875892	-1.375878
H	3.439936	3.607079	-2.781609
C	4.045297	4.413251	0.976387
H	2.679381	2.788665	1.383943
C	4.683330	5.160479	-0.023440
H	4.967996	5.463153	-2.141867
H	4.213540	4.650291	2.022362
H	5.347562	5.973524	0.256163
C	-0.965826	0.747438	-0.191411
C	-1.939734	1.121248	-1.128810
C	-1.277149	0.905855	1.168755
C	-3.184613	1.613337	-0.728162
H	-1.729316	1.010333	-2.187046
C	-2.515087	1.413041	1.569600
H	-0.549213	0.620581	1.920933
C	-3.479376	1.765182	0.625539
H	-4.440127	2.155118	0.940682
C	-2.840032	1.515814	3.035901
C	-4.238337	1.901519	-1.758002
F	-3.335830	0.358421	3.527023
F	-1.748477	1.820682	3.773080
F	-5.123785	2.832146	-1.343546
F	-3.768567	2.472963	3.278723
F	-4.971705	0.787435	-2.060344
F	-3.711263	2.331300	-2.925538

NTOB-benzaldehyde complex 9

B3LYP/6-31G(d) = -2780.351525
B3LYP-D3(BJ)/def2-TZVPP/IEFPCM(propanonitrile) = -2781.613253
B3LYP-D3(BJ)/def2-TZVPP/IEFPCM(propanonitrile)//B3LYP-D3(BJ)/6-31G(d) Free Energy (Quasiharmonic) = -2781.126707

Frequencies (Top 3 out of 213)

1. 11.0592 cm^{-1}
2. 13.4730 cm^{-1}
3. 16.7301 cm^{-1}

B3LYP/6-31G(d) Molecular Geometry in Cartesian Coordinates

B	-0.825143	-0.898429	-1.134329
O	-0.568464	-1.230387	-2.523336
N	-1.133905	0.622956	-1.164079
C	-0.905573	1.108831	-2.540412
S	-2.367568	1.304284	-0.279991
C	-0.671589	-0.173376	-3.345590
O	-0.528535	-0.229192	-4.543339
H	0.155072	2.951201	-2.119031
H	-1.796003	1.609134	-2.936720
C	0.329115	2.043667	-2.706148
C	1.647205	1.419796	-2.324598
C	2.422372	1.660123	-1.129794
C	3.547690	0.790760	-1.174181
N	3.472758	0.086393	-2.357305
C	2.328421	0.456733	-3.034522
H	2.078156	-0.001585	-3.981200
C	4.324776	1.590172	0.933293
C	2.286082	2.522518	-0.028125
H	5.337461	0.047839	-0.200297
H	4.046509	-0.711243	-2.584940
C	3.230675	2.478308	0.989916
H	5.043220	1.573345	1.748379
H	1.450555	3.212748	0.030956
C	4.500123	0.738547	-0.151127
H	3.126670	3.137342	1.847933
C	-1.825176	2.944662	0.205472
O	-2.462812	0.495332	0.955677
O	-3.568583	1.491706	-1.109799
C	-1.074940	5.521790	1.007707
C	-1.002040	3.105509	1.323655
C	-2.286784	4.051508	-0.508144
C	-1.900388	5.330248	-0.107592
C	-0.636723	4.390986	1.714595
H	-0.649284	2.244362	1.880252
H	-2.953015	3.909371	-1.352155
H	-2.255915	6.192769	-0.665621
H	0.002018	4.518282	2.585253
C	-0.685759	6.908762	1.459825
H	-1.281177	7.220426	2.327948
H	-0.844767	7.647963	0.668833
H	0.367285	6.950830	1.758767
O	-2.211719	-1.674647	-0.866370
C	-2.609503	-2.077936	0.250575
H	-1.919451	-2.066708	1.098085
H	0.336404	2.344015	-3.761141
C	-3.938120	-2.602505	0.444745
C	-4.281324	-3.112354	1.711049
C	-4.880986	-2.607053	-0.603314
C	-5.553531	-3.630227	1.926813
H	-3.549537	-3.094681	2.514455
C	-6.149115	-3.123690	-0.378742
H	-4.601575	-2.196112	-1.567530
C	-6.483163	-3.635305	0.882464
H	-5.824274	-4.024700	2.901197
H	-6.883502	-3.127779	-1.178253
H	-7.478354	-4.037225	1.051843
C	0.334855	-1.386357	-0.138921
C	0.481947	-0.875646	1.159026
C	1.331669	-2.258064	-0.604246
C	1.578764	-1.210194	1.956530
H	-0.260288	-0.184945	1.544809
C	2.438935	-2.581289	0.183701
H	1.257802	-2.657898	-1.610316
C	2.569752	-2.061059	1.472172
H	3.434852	-2.300747	2.078797
C	3.551236	-3.405151	-0.400432
C	1.669696	-0.644493	3.346647
F	4.257129	-4.060926	0.544706
F	3.102011	-4.319654	-1.284766
F	1.258021	0.645358	3.392465
F	4.449839	-2.627673	-1.077244
F	2.925917	-0.685730	3.837953
F	0.883227	-1.328002	4.216268

NTOB-benzaldehyde complex 10

B3LYP/6-31G(d) = -2780.350429
B3LYP-D3(BJ)/def2-TZVPP/IEFPCM(propanonitrile) = -2781.612977
B3LYP-D3(BJ)/def2-TZVPP/IEFPCM(propanonitrile)//B3LYP-D3(BJ)/6-31G(d) Free Energy (Quasiharmonic) = -2781.126692

Frequencies (Top 3 out of 213)

1. 12.2098 cm^{-1}
2. 13.9932 cm^{-1}
3. 14.8045 cm^{-1}

B3LYP/6-31G(d) Molecular Geometry in Cartesian Coordinates

B	0.395311	0.132665	-0.491610
O	0.307467	0.063456	-1.945132
N	1.041376	-1.196534	-0.123103
C	1.020642	-2.096939	-1.285802
S	1.848342	-1.626983	1.252879
C	0.649065	-1.152406	-2.429915
O	0.645779	-1.431109	-3.602744
H	0.497306	-4.037914	-0.517644
H	2.023365	-2.488916	-1.504086
C	0.040697	-3.307153	-1.190168
C	-1.335587	-2.966560	-0.700126
C	-2.465609	-2.477309	-1.457020
C	-3.519557	-2.246549	-0.528016
N	-3.054115	-2.606570	0.721959
C	-1.739096	-3.010153	0.612575
H	-1.171902	-3.301236	1.485791
C	-4.955211	-1.472881	-2.268152
C	-2.693746	-2.203518	-2.818384
H	-5.556136	-1.573589	-0.192093
H	-3.500736	-2.368698	1.595530
C	-3.930123	-1.703387	-3.208267
H	-5.909553	-1.076168	-2.603700
H	-1.907748	-2.358882	-3.552136
C	-4.765902	-1.746477	-0.918277
H	-4.111081	-1.480487	-4.256374
C	3.589267	-1.679286	0.792906
O	1.487483	-3.002996	1.621971
O	1.671724	-0.523553	2.206873
C	6.292892	-1.754632	0.048488
C	4.341850	-0.502296	0.795794
C	4.174884	-2.894395	0.433975
C	5.518519	-2.921477	0.059471
C	5.682678	-0.547879	0.424399
H	3.881569	0.430152	1.103736
H	3.588241	-3.806024	0.469844
H	5.972997	-3.868385	-0.221395
H	6.270266	0.367380	0.432376
C	7.756050	-1.797355	-0.322784
H	8.069009	-0.877854	-0.828790
H	8.385243	-1.906938	0.570432
H	7.977665	-2.641731	-0.983089
O	1.485003	1.324870	-0.175964
C	1.894502	2.127183	-1.040840
H	1.578566	1.980939	-2.080215
H	0.010741	-3.756806	-2.190511
C	2.760915	3.234472	-0.711490

C	3.132068	3.494732	0.624601
C	3.225200	4.061739	-1.751628
C	3.963388	4.570015	0.905214
H	2.750632	2.854628	1.414184
C	4.060508	5.135270	-1.461177
H	2.928783	3.855424	-2.776905
C	4.427292	5.386559	-0.135591
H	4.250840	4.781502	1.930519
H	4.422795	5.776053	-2.258917
H	5.076700	6.227523	0.091296
C	-0.962400	0.651723	0.188389
C	-1.902860	1.322200	-0.608459
C	-1.302100	0.412394	1.528671
C	-3.131177	1.744211	-0.093099
H	-1.692352	1.486491	-1.660563
C	-2.542523	0.805778	2.036821
H	-0.596693	-0.092498	2.179425
C	-3.462525	1.481578	1.233773
H	-4.423070	1.786579	1.632084
C	-2.937990	0.400193	3.427790
C	-4.081589	2.508403	-0.973794
F	-1.886990	0.331100	4.266498
F	-3.843777	1.241397	3.972282
F	-3.734170	3.818834	-1.057069
F	-3.519332	-0.839606	3.434897
F	-5.350467	2.471137	-0.509130
F	-4.095316	2.032634	-2.236157

NTOB-benzaldehyde complex 11

B3LYP/6-31G(d) = -2780.349388
B3LYP-D3(BJ)/def2-TZVPP/IEFPCM(propanonitrile) = -2781.613344
B3LYP-D3(BJ)/def2-TZVPP/IEFPCM(propanonitrile)//B3LYP-D3(BJ)/6-31G(d) Free Energy (Quasiharmonic) = -2781.12663

Frequencies (Top 3 out of 213)

1. 8.8905 cm^{-1}
2. 14.1433 cm^{-1}
3. 17.3730 cm^{-1}

B3LYP/6-31G(d) Molecular Geometry in Cartesian Coordinates

B	-0.068264	-1.090084	0.227701
O	0.476594	-2.138598	1.076560
N	0.411760	-1.448222	-1.168622
C	1.178541	-2.710805	-1.111137
S	-0.398583	-1.186393	-2.618254
C	1.219666	-3.027957	0.383066
O	1.823112	-3.938801	0.895646
H	2.456526	-2.778678	-2.829223
H	0.612902	-3.528045	-1.582324
C	2.592159	-2.713694	-1.748215
C	3.474005	-1.538325	-1.438769
C	4.325465	-1.318091	-0.290608
C	5.010222	-0.085321	-0.504062
N	4.569946	0.421983	-1.708742
C	3.665080	-0.461932	-2.269701
H	3.215388	-0.266149	-3.233687
C	6.211659	-0.333871	1.542008
C	4.605782	-2.050465	0.880810
H	6.473026	1.351864	0.211029
H	4.941503	1.239413	-2.167132
C	5.541750	-1.552715	1.781107
H	6.949089	0.021194	2.257160
H	4.100205	-2.992046	1.073046
C	5.953285	0.415688	0.399121
H	5.774288	-2.120358	2.678269
C	-0.822376	0.552409	-2.534355
O	-1.653305	-1.950160	-2.682215
O	0.593260	-1.386747	-3.688404
C	-1.543178	3.255395	-2.451644
C	0.165162	1.519605	-2.341546
C	-2.154937	0.916933	-2.729669
C	-2.505157	2.263944	-2.690324
C	-0.204960	2.863397	-2.295499
H	1.200391	1.222932	-2.208715
H	-2.900888	0.145445	-2.885164
H	-3.546126	2.547368	-2.815562
H	0.557799	3.622697	-2.137026
C	-1.956327	4.701184	-2.322413
H	-2.475398	4.859514	-1.368524
H	-2.647423	4.994673	-3.120248
H	-1.094010	5.374551	-2.353689
H	3.066443	-3.646180	-1.418424
C	-1.586756	-0.712874	0.576368
C	-2.080756	0.599790	0.593119
C	-2.478857	-1.741284	0.914953
C	-3.409073	0.875083	0.923415
H	-1.425973	1.425426	0.342293
C	-3.807660	-1.471498	1.251477
H	-2.135209	-2.770544	0.920651
C	-4.281983	-0.160557	1.257287
H	-5.313585	0.048887	1.517724
C	-4.718216	-2.591984	1.679437
C	-3.934810	2.282640	0.864488
F	-4.644688	-2.808952	3.014611
F	-4.405340	-3.756612	1.074710
F	-6.013489	-2.320909	1.399411
F	-4.778578	2.550592	1.885504
F	-4.629365	2.515704	-0.278498
F	-2.943766	3.206712	0.907566
O	0.768337	0.288244	0.655201
C	1.696695	0.314666	1.490858
C	2.333479	1.545385	1.901169
C	1.910109	2.793980	1.399327
C	3.371539	1.478084	2.848953
C	2.518972	3.955439	1.851501
H	1.109894	2.828559	0.667076
C	3.976542	2.648820	3.296603
H	3.704080	0.510562	3.212639
C	3.548470	3.882496	2.801076
H	2.195782	4.921545	1.475590
H	4.778298	2.601672	4.026860
H	4.017763	4.796548	3.154703
H	2.023780	-0.626198	1.944543

NTOB-benzaldehyde complex 12

B3LYP/6-31G(d) = -2780.353394
B3LYP-D3(BJ)/def2-TZVPP/IEFPCM(propanonitrile) = -2781.612789
B3LYP-D3(BJ)/def2-TZVPP/IEFPCM(propanonitrile)//B3LYP-D3(BJ)/6-31G(d) Free Energy (Quasiharmonic) = -2781.126614

Frequencies (Top 3 out of 213)

1. 7.7331 cm^{-1}
2. 15.9814 cm^{-1}
3. 16.3943 cm^{-1}

B3LYP/6-31G(d) Molecular Geometry in Cartesian Coordinates

B	0.271611	0.151186	-0.804003
O	0.024331	-0.347864	-2.140282
N	1.021903	-0.997828	-0.133681
C	1.033805	-2.176115	-1.015769
S	1.872806	-0.956449	1.274360
C	0.450376	-1.612627	-2.317698
O	0.372439	-2.217246	-3.358225
H	0.804735	-3.834192	0.336135

H	2.063469	-2.491756	-1.230070
C	0.251924	-3.422780	-0.514282
C	-1.177482	-3.184380	-0.122270
C	-2.354239	-3.228419	-0.962177
C	-3.469296	-2.925584	-0.131254
N	-2.988735	-2.731273	1.148940
C	-1.615767	-2.870779	1.141764
H	-1.038388	-2.744632	2.047053
C	-4.969100	-3.148627	-1.971076
C	-2.581152	-3.501630	-2.323675
H	-5.615866	-2.641867	0.030764
H	-3.515291	-2.335022	1.912954
C	-3.880910	-3.459202	-2.812147
H	-5.973733	-3.119291	-2.384639
H	-1.749933	-3.716766	-2.988358
C	-4.778635	-2.878672	-0.621023
H	-4.063791	-3.662395	-3.863926
C	3.546030	-1.442365	0.825044
O	1.395527	-1.964604	2.232141
O	1.882708	0.461513	1.692581
C	6.187077	-2.154016	0.213341
C	4.163393	-2.474061	1.530768
C	4.230913	-0.759006	-0.184416
C	5.539794	-1.121474	-0.484936
C	5.478582	-2.821311	1.219347
H	3.613709	-2.994783	2.306984
H	3.739511	0.034266	-0.738711
H	6.069771	-0.597976	-1.277145
H	5.958668	-3.628435	1.766811
C	7.615916	-2.520154	-0.109160
H	8.314363	-1.780364	0.303319
H	7.785238	-2.555797	-1.191072
H	7.884125	-3.495053	0.308795
O	1.382203	1.340508	-1.065106
C	1.556907	2.285510	-0.262205
H	0.870282	2.402380	0.578474
H	0.315288	-4.159584	-1.324467
C	2.619376	3.251396	-0.428540
C	3.467052	3.225552	-1.554430
C	2.785766	4.239161	0.559581
C	4.468560	4.178287	-1.680447
H	3.314271	2.467110	-2.315552
C	3.795967	5.186686	0.429428
H	2.128862	4.248116	1.425174
C	4.633564	5.155093	-0.689015
H	5.121536	4.169786	-2.547874
H	3.931648	5.947045	1.192143
H	5.419695	5.897847	-0.792935
C	-1.022373	0.825139	-0.134009
C	-1.442189	0.567707	1.178590
C	-1.828417	1.669382	-0.916050
C	-2.622879	1.120680	1.684068
H	-0.855105	-0.081964	1.816438
C	-2.996691	2.241475	-0.407932
H	-1.554027	1.865477	-1.948646
C	-3.404253	1.965773	0.897000
H	-4.317450	2.397606	1.289991
C	-3.788246	3.205916	-1.249886
C	-3.095455	0.746862	3.061490
F	-3.733422	2.896138	-2.561773
F	-5.090281	3.242248	-0.890984
F	-2.075697	0.456145	3.892317
F	-3.313461	4.471609	-1.127183
F	-3.899455	-0.359232	3.035757
F	-3.826782	1.726676	3.633510

NTOB-benzaldehyde complex 13

B3LYP/6-31G(d) = -2780.351537

B3LYP-D3(BJ)/def2-TZVPP/IEFPCM(propanonitrile) = -2781.613268

B3LYP-D3(BJ)/def2-TZVPP/IEFPCM(propanonitrile)//B3LYP-D3(BJ)/6-31G(d) Free Energy (Quasiharmonic) = -2781.126481

Frequencies (Top 3 out of 213)

1. 11.6752 cm^{-1}
2. 16.6178 cm^{-1}
3. 18.8160 cm^{-1}

B3LYP/6-31G(d) Molecular Geometry in Cartesian Coordinates

B	0.829833	0.893267	-1.134930
O	0.574941	1.223624	-2.524672
N	1.132884	-0.629302	-1.161774
C	0.903142	-1.116880	-2.537230
S	2.363540	-1.313831	-0.275960
C	0.674025	0.164660	-3.344878
O	0.531207	0.218682	-4.542740
H	-0.164268	-2.954535	-2.112652
H	1.791814	-1.621219	-2.932382
C	-0.334913	-2.047473	-2.701499
C	-1.650822	-1.418099	-2.321481
C	-2.426555	-1.652288	-1.125832
C	-3.549263	-0.779696	-1.172846
N	-3.472225	-0.079127	-2.358097
C	-2.329068	-0.455038	-3.034244
H	-2.077371	-0.000317	-3.982278
C	-4.328549	-1.570185	0.937204
C	-2.292578	-2.511332	-0.021274
H	-5.336795	-0.028503	-0.201199
H	-4.043442	0.719670	-2.588039
C	-3.236925	-2.461178	0.996715
H	-5.046864	-1.548638	1.752290
H	-1.458583	-3.203200	0.040287
C	-4.501488	-0.721515	-0.149921
H	-3.134501	-3.117500	1.856989
C	1.813609	-2.951388	0.210771
O	2.461152	-0.503290	0.958466
O	3.564280	-1.507023	-1.104840
C	1.055528	-5.524752	1.018046
C	0.992633	-3.107776	1.330874
C	2.273192	-4.061330	-0.499857
C	1.884831	-5.338018	-0.095581
C	0.625065	-4.391735	1.725607
H	0.648171	-2.244847	1.889891
H	2.944267	-3.922975	-1.340651
H	2.243840	-6.203054	-0.647497
H	-0.006732	-4.515867	2.601704
C	0.621291	-6.907389	1.441348
H	0.587004	-6.999689	2.532170
H	1.297278	-7.676372	1.054781
H	-0.384903	-7.134513	1.065287
O	2.219240	1.664606	-0.867726
C	2.618058	2.068330	0.248701
H	1.927518	2.061382	1.095864
H	-0.343102	-2.349747	-3.755932
C	3.948665	2.587903	0.442611
C	4.293170	3.098977	1.708071
C	4.892192	2.586398	-0.604862
C	5.567342	3.612112	1.923543
H	3.560825	3.085961	2.511057
C	6.162284	3.098307	-0.380572
H	4.611712	2.174538	-1.568375
C	6.497633	3.611201	0.879769
H	5.839094	4.007510	2.897272
H	6.897187	3.097694	-1.179618
H	7.494358	4.009395	1.048936
C	-0.328766	1.387425	-0.140976
C	-0.478483	0.879579	1.157811
C	-1.321988	2.262091	-0.608397
C	-1.574350	1.219850	1.954178

H	0.260752	0.186534	1.545116
C	-2.428411	2.590972	0.178402
H	-1.246124	2.659776	-1.615170
C	-2.561815	2.073658	1.467765
H	-3.426300	2.317770	2.073511
C	-3.537347	3.417840	-0.407862
C	-1.668245	0.657375	3.345415
F	-4.438460	2.642381	-1.083638
F	-4.241288	4.078033	0.535663
F	-1.258539	-0.632939	3.394830
F	-3.084303	4.328988	-1.293712
F	-2.925213	0.701585	3.834606
F	-0.882240	1.341763	4.214773

NTOB-benzaldehyde complex 14

B3LYP/6-31G(d) = -2780.353406
B3LYP-D3(BJ)/def2-TZVPP/IEFPCM(propanonitrile) = -2781.612781
B3LYP-D3(BJ)/def2-TZVPP/IEFPCM(propanonitrile)//B3LYP-D3(BJ)/6-31G(d) Free Energy (Quasiharmonic) = -2781.126441

Frequencies (Top 3 out of 213)

1. 7.7097 cm^{-1}
2. 15.9839 cm^{-1}
3. 16.3950 cm^{-1}

B3LYP/6-31G(d) Molecular Geometry in Cartesian Coordinates

B	0.272239	0.151468	-0.803671
O	0.025996	-0.348477	-2.139842
N	1.023253	-0.996608	-0.132453
C	1.036224	-2.175398	-1.013862
S	1.873550	-0.953805	1.275860
C	0.452916	-1.613053	-2.316332
O	0.375828	-2.218351	-3.356537
H	0.807936	-3.832851	0.338902
H	2.066166	-2.490453	-1.227628
C	0.255057	-3.422312	-0.511892
C	-1.174602	-3.184664	-0.120377
C	-2.351099	-3.229870	-0.960574
C	-3.466579	-2.927441	-0.130073
N	-2.986494	-2.732256	1.150166
C	-1.613431	-2.870807	1.143405
H	-1.036368	-2.743845	2.048783
C	-4.965746	-3.152308	-1.970191
C	-2.577464	-3.503815	-2.322015
H	-5.613386	-2.645140	0.031262
H	-3.513514	-2.335948	1.913827
C	-3.877122	-3.462488	-2.810845
H	-5.970289	-3.123840	-2.384030
H	-1.745921	-3.718652	-2.986390
C	-4.775820	-2.881644	-0.620205
H	-4.059588	-3.666250	-3.862587
C	3.547147	-1.438637	0.826628
O	1.397209	-1.962272	2.233805
O	1.881372	0.464269	1.693753
C	6.190910	-2.144091	0.219649
C	4.168686	-2.463326	1.539438
C	4.230662	-0.756707	-0.184336
C	5.541931	-1.114762	-0.481084
C	5.485658	-2.805972	1.231961
H	3.622238	-2.978259	2.321783
H	3.738924	0.036348	-0.738655
H	6.073195	-0.588084	-1.270261
H	5.970953	-3.603445	1.788987
C	7.604475	-2.541264	-0.131964
H	8.217708	-1.666944	-0.375651
H	7.620023	-3.202304	-1.008437
H	8.086870	-3.077025	0.691278
O	1.381846	1.341589	-1.064689
C	1.555286	2.287041	-0.262007
H	0.868047	2.403646	0.578200
H	0.319122	-4.159517	-1.321664
C	2.617055	3.253709	-0.428037
C	2.782253	4.241734	0.560028
C	3.465245	3.228353	-1.553552
C	3.791807	5.189988	0.430208
H	2.124975	4.250287	1.425340
C	4.466072	4.181837	-1.679255
H	3.313382	2.469693	-2.314639
C	4.629913	5.158883	-0.687865
H	3.926604	5.950525	1.192901
H	5.119426	4.173733	-2.546403
H	5.415540	5.902205	-0.791525
C	-1.022736	0.824485	-0.134646
C	-1.442671	0.567773	1.178062
C	-1.829461	1.667138	-0.917690
C	-2.624100	1.119931	1.682687
H	-0.855076	-0.080679	1.816676
C	-2.998504	2.238433	-0.410432
H	-1.554976	1.862583	-1.950386
C	-3.406149	1.963479	0.894625
H	-4.319921	2.394693	1.286960
C	-3.790826	3.201251	-1.253522
C	-3.096754	0.746834	3.060278
F	-5.092906	3.236931	-0.894711
F	-3.317081	4.467470	-1.132282
F	-3.900052	-0.359797	3.035163
F	-3.735700	2.889988	-2.565046
F	-3.828874	1.726592	3.631377
F	-2.077033	0.457393	3.891584

NTOB-benzaldehyde complex 15

B3LYP/6-31G(d) = -2780.353406
B3LYP-D3(BJ)/def2-TZVPP/IEFPCM(propanonitrile) = -2781.612781
B3LYP-D3(BJ)/def2-TZVPP/IEFPCM(propanonitrile)//B3LYP-D3(BJ)/6-31G(d) Free Energy (Quasiharmonic) = -2781.126438

Frequencies (Top 3 out of 213)

1. 7.7086 cm^{-1}
2. 15.9865 cm^{-1}
3. 16.4026 cm^{-1}

B3LYP/6-31G(d) Molecular Geometry in Cartesian Coordinates

B	0.272286	0.151546	-0.803681
O	0.026051	-0.348386	-2.139859
N	1.023231	-0.996587	-0.132467
C	1.036219	-2.175352	-1.013906
S	1.873501	-0.953840	1.275862
C	0.452921	-1.612970	-2.316362
O	0.375820	-2.218251	-3.356578
H	0.807987	-3.832830	0.338819
H	2.066170	-2.490381	-1.227684
C	0.255094	-3.422301	-0.511971
C	-1.174581	-3.184734	-0.120450
C	-2.351079	-3.229917	-0.960650
C	-3.466569	-2.927595	-0.130123
N	-2.986493	-2.732504	1.150138
C	-1.613422	-2.870990	1.143357
H	-1.036363	-2.744066	2.048744
C	-4.965727	-3.152341	-1.970260
C	-2.577436	-3.503753	-2.322115
H	-5.613384	-2.645372	0.031239
H	-3.513518	-2.336209	1.913804
C	-3.877094	-3.462416	-2.810941
H	-5.970272	-3.123861	-2.384097

H	-1.745888	-3.718511	-2.986509
C	-4.775811	-2.881795	-0.620250
H	-4.059554	-3.666091	-3.862700
C	3.547090	-1.438696	0.826620
O	1.397115	-1.962318	2.233773
O	1.881369	0.464220	1.693808
C	6.190859	-2.144148	0.219652
C	4.230630	-0.756724	-0.184300
C	4.168616	-2.463412	1.539409
C	5.485592	-2.806051	1.231939
C	5.541904	-1.114775	-0.481042
H	3.738896	0.036357	-0.738590
H	3.622164	-2.978368	2.321737
H	5.970873	-3.603543	1.788952
H	6.073190	-0.588061	-1.270179
C	7.604377	-2.541406	-0.132055
H	8.217439	-1.667211	-0.376590
H	7.619743	-3.203105	-1.008043
H	8.087064	-3.076511	0.691444
O	1.381879	1.341623	-1.064627
C	1.555340	2.287021	-0.261881
H	0.868124	2.403573	0.578352
H	0.319194	-4.159490	-1.321755
C	2.617091	3.253707	-0.427892
C	2.782319	4.241674	0.560228
C	3.465233	3.228432	-1.553445
C	3.791853	5.189948	0.430424
H	2.125079	4.250163	1.425571
C	4.466042	4.181936	-1.679131
H	3.313346	2.469820	-2.314575
C	4.629912	5.158922	-0.687687
H	3.926674	5.950439	1.193160
H	5.119362	4.173893	-2.546306
H	5.415526	5.902261	-0.791333
C	-1.022712	0.824552	-0.134674
C	-1.442732	0.567703	1.177975
C	-1.829355	1.667333	-0.917666
C	-2.624163	1.119859	1.682601
H	-0.855205	-0.080860	1.816540
C	-2.998401	2.238615	-0.410408
H	-1.554803	1.862890	-1.950321
C	-3.406129	1.963530	0.894597
H	-4.319907	2.394737	1.286930
C	-3.790661	3.201562	-1.253407
C	-3.096893	0.746634	3.060129
F	-3.735171	2.890705	-2.565012
F	-5.092834	3.236930	-0.894900
F	-3.829047	1.726340	3.631279
F	-3.317149	4.467821	-1.131666
F	-2.077213	0.457121	3.891466
F	-3.900181	-0.359998	3.034863

NTOB-benzaldehyde complex 16

B3LYP/6-31G(d) = -2780.353406
B3LYP-D3(BJ)/def2-TZVPP/IEFPCM(propanonitrile) = -2781.612776
B3LYP-D3(BJ)/def2-TZVPP/IEFPCM(propanonitrile)//B3LYP-D3(BJ)/6-31G(d) Free Energy (Quasiharmonic) = -2781.126435

Frequencies (Top 3 out of 213)

1. 7.7107 cm^{-1}
2. 15.9859 cm^{-1}
3. 16.3929 cm^{-1}

B3LYP/6-31G(d) Molecular Geometry in Cartesian Coordinates

B	0.272234	0.151414	-0.803699
O	0.025928	-0.348502	-2.139860
N	1.023237	-0.996678	-0.132539
C	1.036104	-2.175482	-1.013937
S	1.873458	-0.953971	1.275828
C	0.452766	-1.613104	-2.316381
O	0.375603	-2.218386	-3.356590
H	0.807822	-3.832903	0.338852
H	2.066023	-2.490582	-1.227752
C	0.254924	-3.422370	-0.511931
C	-1.174729	-3.184727	-0.120376
C	-2.351256	-3.229980	-0.960535
C	-3.466719	-2.927567	-0.130006
N	-2.986596	-2.732343	1.150218
C	-1.613528	-2.870857	1.143414
H	-1.036439	-2.743870	2.048771
C	-4.965935	-3.152488	-1.970077
C	-2.577655	-3.503949	-2.321966
H	-5.613525	-2.645303	0.031390
H	-3.513597	-2.335990	1.913874
C	-3.877328	-3.462655	-2.810759
H	-5.970491	-3.124043	-2.383887
H	-1.746126	-3.718776	-2.986362
C	-4.775975	-2.881805	-0.620099
H	-4.059820	-3.666433	-3.862493
C	3.547091	-1.438741	0.826630
O	1.397081	-1.962516	2.233672
O	1.881261	0.464068	1.693824
C	6.190858	-2.144234	0.219659
C	4.230565	-0.756939	-0.184452
C	4.168686	-2.463315	1.539567
C	5.485660	-2.805971	1.232102
C	5.541833	-1.115015	-0.481196
H	3.738796	0.036023	-0.738876
H	3.622284	-2.978172	2.321995
H	5.970984	-3.603357	1.789230
H	6.073050	-0.588448	-1.270477
C	7.604401	-2.541444	-0.131996
H	8.217477	-1.667203	-0.376335
H	7.619842	-3.202998	-1.008090
H	8.087030	-3.076674	0.691457
O	1.381941	1.341593	-1.064720
C	1.555428	2.286960	-0.261957
H	0.868249	2.403482	0.578311
H	0.318955	-4.159587	-1.321694
C	2.617219	3.253618	-0.427916
C	2.782503	4.241505	0.560275
C	3.465345	3.228388	-1.553481
C	3.792079	5.189744	0.430529
H	2.125272	4.249960	1.425625
C	4.466194	4.181861	-1.679113
H	3.313420	2.469831	-2.314658
C	4.630120	5.158766	-0.687597
H	3.926945	5.950171	1.193320
H	5.119500	4.173856	-2.546297
H	5.415764	5.902079	-0.791199
C	-1.022681	0.824555	-0.134694
C	-1.442739	0.567766	1.177953
C	-1.829226	1.667410	-0.917714
C	-2.624141	1.120023	1.682543
H	-0.855246	-0.080798	1.816548
C	-2.998232	2.238799	-0.410487
H	-1.554627	1.862937	-1.950362
C	-3.406016	1.963752	0.894511
H	-4.319768	2.395035	1.286820
C	-3.790401	3.201831	-1.253476
C	-3.096947	0.746837	3.060057
F	-3.317096	4.468125	-1.131320
F	-3.734542	2.891281	-2.565136
F	-3.900387	-0.359677	3.034751
F	-5.092669	3.236940	-0.895284
F	-3.828974	1.726637	3.631209
F	-2.077321	0.457169	3.891405

NTOB-benzaldehyde complex 17

B3LYP/6-31G(d) = -2780.350473
B3LYP-D3(BJ)/def2-TZVPP/IEFPCM(propanonitrile) = -2781.612277
B3LYP-D3(BJ)/def2-TZVPP/IEFPCM(propanonitrile)//B3LYP-D3(BJ)/6-31G(d) Free Energy (Quasiharmonic) = -2781.126008

Frequencies (Top 3 out of 213)

1. 11.1213 cm^{-1}
2. 12.8066 cm^{-1}
3. 16.7203 cm^{-1}

B3LYP/6-31G(d) Molecular Geometry in Cartesian Coordinates

B	0.391841	0.127871	-0.482849
O	0.299234	0.056144	-1.935438
N	1.033327	-1.201639	-0.112045
C	1.019680	-2.101600	-1.275513
S	1.837419	-1.630744	1.266611
C	0.647297	-1.158084	-2.420164
O	0.648766	-1.435806	-3.593164
H	0.495762	-4.040002	-0.501479
H	2.024631	-2.489570	-1.490724
C	0.043655	-3.314890	-1.183090
C	-1.338731	-2.974402	-0.710950
C	-2.463076	-2.502429	-1.487017
C	-3.526908	-2.259785	-0.572346
N	-3.072454	-2.597652	0.687860
C	-1.755263	-2.999099	0.598140
H	-1.196642	-3.276481	1.481327
C	-4.949711	-1.527309	-2.340212
C	-2.679770	-2.256838	-2.855465
H	-5.567998	-1.585352	-0.268117
H	-3.530150	-2.351845	1.553408
C	-3.915644	-1.772539	-3.266474
H	-5.901844	-1.139173	-2.691332
H	-1.885883	-2.421798	-3.578524
C	-4.771150	-1.771742	-0.983552
H	-4.088532	-1.572517	-4.320605
C	3.579819	-1.680583	0.812271
O	1.476707	-3.007223	1.634076
O	1.655479	-0.527808	2.219873
C	6.286058	-1.751808	0.077209
C	4.167670	-2.894343	0.452285
C	4.331408	-0.503021	0.821031
C	5.673589	-0.546564	0.454186
C	5.512599	-2.919345	0.082423
H	3.581727	-3.806587	0.483809
H	3.869167	0.428111	1.129949
H	6.260412	0.369131	0.466699
H	5.968853	-3.865148	-0.199288
C	7.750549	-1.792501	-0.289004
H	8.065520	-0.869935	-0.788111
H	7.974432	-2.632814	-0.953730
H	8.376446	-1.907833	0.605803
O	1.490977	1.319472	-0.176256
C	1.903935	2.112173	-1.048138
H	1.586931	1.958353	-2.086141
H	0.025540	-3.770296	-2.181080
C	2.775633	3.218801	-0.729648
C	3.244438	4.032729	-1.778200
C	3.147327	3.491585	0.603711
C	4.084967	5.105155	-1.498788
H	2.947510	3.816962	-2.801385
C	3.983764	4.565735	0.873371
H	2.762082	2.862107	1.399940
C	4.452339	5.368794	-0.175790
H	4.450827	5.735541	-2.303159
H	4.271614	4.786796	1.896549
H	5.105792	6.208904	0.042485
C	-0.958303	0.660576	0.201012
C	-1.319317	0.386582	1.527704
C	-1.866893	1.393110	-0.580180
C	-2.549512	0.808330	2.040740
H	-0.636479	-0.159851	2.168524
C	-3.081926	1.842902	-0.060117
H	-1.633526	1.600491	-1.619426
C	-3.435222	1.545118	1.255280
H	-4.380974	1.885187	1.661747
C	-4.054505	2.586690	-0.934793
C	-2.963978	0.379779	3.419485
F	-4.766140	3.496840	-0.226898
F	-3.429666	3.253555	-1.932270
F	-3.567024	-0.849399	3.394520
F	-4.951628	1.759002	-1.514864
F	-3.858599	1.224972	3.975365
F	-1.921102	0.272802	4.264309

NTOB-benzaldehyde complex 18

B3LYP/6-31G(d) = -2780.354283
B3LYP-D3(BJ)/def2-TZVPP/IEFPCM(propanonitrile) = -2781.61277
B3LYP-D3(BJ)/def2-TZVPP/IEFPCM(propanonitrile)//B3LYP-D3(BJ)/6-31G(d) Free Energy (Quasiharmonic) = -2781.125798

Frequencies (Top 3 out of 213)

1. 10.7010 cm^{-1}
2. 17.2606 cm^{-1}
3. 19.9345 cm^{-1}

B3LYP/6-31G(d) Molecular Geometry in Cartesian Coordinates

B	0.917074	-0.924833	-0.587536
O	0.446516	-2.115919	-1.264535
N	0.797456	-1.295789	0.894902
C	0.219347	-2.645722	1.032838
S	1.511595	-0.539326	2.188055
C	0.173257	-3.131160	-0.418309
O	-0.093267	-4.252888	-0.770050
H	-1.119202	-2.355754	2.701277
H	0.890141	-3.295200	1.605079
C	-1.195378	-2.728241	1.674482
C	-2.291998	-2.018122	0.931249
C	-3.179896	-2.583986	-0.062663
C	-4.034083	-1.536838	-0.503718
N	-3.698214	-0.403057	0.210401
C	-2.641936	-0.694075	1.050226
H	-2.224603	0.074128	1.685364
C	-5.148747	-3.003679	-2.013806
C	-3.343382	-3.866660	-0.617925
H	-5.637882	-0.903259	-1.821056
H	-4.008378	0.527840	-0.023573
C	-4.324389	-4.063215	-1.580826
H	-5.898695	-3.187912	-2.778399
H	-2.691749	-4.682622	-0.321458
C	-5.014467	-1.726665	-1.483710
H	-4.452558	-5.048847	-2.019988
C	0.286288	0.418992	3.086135
O	2.465139	0.427240	1.612985
O	1.989944	-1.588065	3.102639
C	-1.611884	1.910554	4.510239
C	-0.032987	1.711656	2.662741
C	-0.307968	-0.126791	4.225910
C	-1.253223	0.620591	4.926012
C	-0.980151	2.443211	3.376506
H	0.462421	2.153259	1.806252
H	-0.012264	-1.112093	4.569689
H	-1.711791	0.197563	5.816297

H	-1.221082	3.452114	3.050893
C	-2.657650	2.702192	5.257936
H	-2.487442	3.779535	5.164732
H	-2.667127	2.448342	6.322850
H	-3.661877	2.494804	4.865120
O	2.507087	-0.840921	-1.046079
C	3.463926	-1.330331	-0.412169
H	3.271183	-1.872076	0.519176
H	-1.427633	-3.797102	1.750401
C	4.829280	-1.232917	-0.871788
C	5.843846	-1.809603	-0.084994
C	5.150646	-0.576516	-2.077148
C	7.169042	-1.732987	-0.500282
H	5.585038	-2.305811	0.846728
C	6.475050	-0.505608	-2.484861
H	4.354188	-0.136881	-2.668374
C	7.481064	-1.082902	-1.697804
H	7.956170	-2.174077	0.103141
H	6.732611	-0.003030	-3.412075
H	8.516283	-1.023134	-2.022319
C	0.298787	0.451910	-1.121535
C	0.913169	1.693215	-0.912622
C	-0.944549	0.445548	-1.775954
C	0.298886	2.886002	-1.309296
H	1.875654	1.738345	-0.413141
C	-1.554534	1.634836	-2.182185
H	-1.440741	-0.499999	-1.967497
C	-0.938589	2.866186	-1.946538
H	-1.411522	3.789490	-2.260580
C	-2.919536	1.617226	-2.812370
C	0.959288	4.189272	-0.959305
F	-3.900580	1.846608	-1.881314
F	-3.062707	2.586484	-3.741721
F	0.842891	4.452223	0.371391
F	-3.211530	0.441253	-3.393413
F	0.418564	5.238560	-1.613878
F	2.282556	4.177081	-1.231437

NTOB-benzaldehyde complex 19

B3LYP/6-31G(d) = -2780.351933
B3LYP-D3(BJ)/def2-TZVPP/IEFPCM(propanonitrile) = -2781.612759
B3LYP-D3(BJ)/def2-TZVPP/IEFPCM(propanonitrile)//B3LYP-D3(BJ)/6-31G(d) Free Energy (Quasiharmonic) = -2781.125287

Frequencies (Top 3 out of 213)

1. 9.1142 cm^{-1}
2. 15.8743 cm^{-1}
3. 23.5315 cm^{-1}

B3LYP/6-31G(d) Molecular Geometry in Cartesian Coordinates

B	0.795229	-0.884476	-1.016783
O	0.602181	-1.759254	-2.164153
N	0.540079	-1.822801	0.185953
C	0.285025	-3.197984	-0.304414
S	1.291082	-1.718303	1.671756
C	0.303847	-3.027737	-1.826516
O	0.072116	-3.900536	-2.627895
H	-0.928819	-4.099714	1.223487
H	1.109632	-3.877997	-0.043111
C	-1.028829	-3.875347	0.159603
C	-2.283192	-3.085503	-0.087005
C	-3.080574	-2.405507	0.905016
C	-4.160497	-1.778659	0.223921
N	-4.033377	-2.089522	-1.114283
C	-2.901827	-2.864546	-1.295885
H	-2.629979	-3.218980	-2.280987
C	-5.014924	-0.907686	2.272110
C	-2.997149	-2.279503	2.304690
H	-5.935360	-0.535174	0.350423
H	-4.568602	-1.670516	-1.859460
C	-3.962258	-1.534421	2.972393
H	-5.752177	-0.328695	2.821843
H	-2.183980	-2.748859	2.851990
C	-5.129188	-1.021647	0.892024
H	-3.908531	-1.432959	4.053173
C	0.925847	-0.054662	2.209945
O	2.767528	-1.812875	1.508965
O	0.658746	-2.696980	2.565430
C	0.373202	2.534835	3.109645
C	1.979936	0.826528	2.455356
C	-0.400206	0.324753	2.433688
C	-0.662993	1.616202	2.879468
C	1.694260	2.115910	2.901712
H	3.001689	0.499389	2.297505
H	-1.213583	-0.370019	2.253487
H	-1.694134	1.917149	3.045844
H	2.510593	2.809218	3.087741
C	0.066966	3.951490	3.530030
H	-0.122983	4.576335	2.648633
H	0.902979	4.398636	4.077326
H	-0.823767	3.998355	4.165041
O	2.355974	-0.434576	-1.049772
C	3.290467	-1.265307	-0.946151
H	3.060457	-2.332343	-0.931383
H	-1.063229	-4.832988	-0.374210
C	4.677860	-0.870397	-0.924421
C	5.656650	-1.870450	-0.776053
C	5.057800	0.480657	-1.052853
C	7.003054	-1.523724	-0.753293
H	5.352211	-2.907683	-0.666427
C	6.403879	0.818232	-1.033535
H	4.291534	1.238981	-1.174305
C	7.373710	-0.182061	-0.883007
H	7.761588	-2.290938	-0.634061
H	6.705610	1.856098	-1.136070
H	8.425846	0.088610	-0.867584
C	-0.016547	0.495345	-1.131912
C	0.506228	1.751099	-0.794764
C	-1.337693	0.455685	-1.601893
C	-0.260787	2.913730	-0.902891
H	1.521211	1.830666	-0.424357
C	-2.107042	1.615217	-1.721987
H	-1.772902	-0.496014	-1.884834
C	-1.573975	2.854599	-1.369288
H	-2.169258	3.756762	-1.458488
C	-3.538736	1.528936	-2.175576
C	0.329703	4.253218	-0.560984
F	-3.931501	2.642830	-2.830963
F	-3.748931	0.477730	-3.009196
F	-0.553597	5.032107	0.115230
F	-4.393182	1.367443	-1.135797
F	0.687614	4.950333	-1.664161
F	1.435053	4.147356	0.212584

NTOB-benzaldehyde complex 20

B3LYP/6-31G(d) = -2780.35171
B3LYP-D3(BJ)/def2-TZVPP/IEFPCM(propanonitrile) = -2781.612306
B3LYP-D3(BJ)/def2-TZVPP/IEFPCM(propanonitrile)//B3LYP-D3(BJ)/6-31G(d) Free Energy (Quasiharmonic) = -2781.125073

Frequencies (Top 3 out of 213)

1. 6.8492 cm^{-1}
2. 17.0467 cm^{-1}
3. 22.7066 cm^{-1}

B3LYP/6-31G(d) Molecular Geometry in Cartesian Coordinates

B	0.690488	-1.005688	-0.935232
O	0.472339	-2.038557	-1.939859
N	0.580598	-1.786633	0.405360
C	0.278085	-3.210138	0.111467
S	1.570345	-1.574272	1.730016
C	0.189590	-3.246640	-1.415228
O	-0.099266	-4.215060	-2.075535
H	-0.821556	-3.862415	1.836653
H	1.113911	-3.864193	0.399020
C	-1.003234	-3.786991	0.762004
C	-2.259310	-3.006038	0.500181
C	-2.922099	-2.109509	1.415472
C	-4.051864	-1.572104	0.739108
N	-4.081392	-2.139529	-0.518321
C	-3.000251	-2.990606	-0.658706
H	-2.845107	-3.537073	-1.579276
C	-4.648365	-0.283079	2.654184
C	-2.680935	-1.723089	2.747101
H	-5.765394	-0.240968	0.802315
H	-4.692268	-1.854743	-1.268537
C	-3.544043	-0.816560	3.352112
H	-5.303172	0.426642	3.152709
H	-1.829214	-2.124446	3.290419
C	-4.917915	-0.653234	1.342179
H	-3.370058	-0.514965	4.381784
C	1.394070	0.157986	2.130136
O	2.994396	-1.792695	1.351047
O	1.044190	-2.402944	2.822444
C	1.174545	2.839435	2.899964
C	0.139447	0.674909	2.467320
C	2.539757	0.952664	2.188048
C	2.419852	2.288188	2.571806
C	0.041496	2.011233	2.841671
H	-0.746226	0.049415	2.425939
H	3.504757	0.522856	1.943572
H	3.309714	2.910817	2.620887
H	-0.934192	2.419981	3.090840
C	1.042795	4.293140	3.281919
H	0.379742	4.420017	4.144885
H	2.013377	4.733912	3.529314
H	0.611643	4.865204	2.452645
O	2.196795	-0.474305	-1.133127
C	3.179732	-1.256815	-1.147774
H	3.005359	-2.334199	-1.136463
H	-1.094513	-4.808755	0.373994
C	4.536873	-0.784756	-1.269522
C	4.825926	0.589508	-1.390394
C	5.578443	-1.730986	-1.273126
C	6.145005	1.003101	-1.514507
H	4.010849	1.305465	-1.391389
C	6.897543	-1.308145	-1.394469
H	5.345023	-2.787070	-1.169300
C	7.177954	0.056051	-1.515864
H	6.376676	2.059312	-1.612030
H	7.704816	-2.033756	-1.393630
H	8.208542	0.386147	-1.612904
C	-0.231376	0.288281	-1.171249
C	0.147096	1.598639	-0.847238
C	-1.502673	0.109623	-1.736615
C	-0.709187	2.680333	-1.071406
H	1.122835	1.789467	-0.414756
C	-2.364680	1.185202	-1.958820
H	-1.822569	-0.886630	-2.016500
C	-1.971810	2.481785	-1.629595
H	-2.630527	3.323124	-1.816319
C	-3.744479	0.953107	-2.509541
C	-0.310469	4.070395	-0.662837
F	-4.679240	0.885577	-1.524435
F	-3.836856	-0.210965	-3.197266
F	-0.892686	4.431874	0.512714
F	-4.134092	1.944963	-3.338994
F	-0.688549	4.995329	-1.573206
F	1.024138	4.197213	-0.487003

NTOB-benzaldehyde complex 21

B3LYP/6-31G(d) = -2780.347452
B3LYP-D3(BJ)/def2-TZVPP/IEFPCM(propanonitrile) = -2781.610513
B3LYP-D3(BJ)/def2-TZVPP/IEFPCM(propanonitrile)//B3LYP-D3(BJ)/6-31G(d) Free Energy (Quasiharmonic) = -2781.124749

Frequencies (Top 3 out of 213)

1. 7.8382 cm^{-1}
2. 10.6253 cm^{-1}
3. 12.3824 cm^{-1}

B3LYP/6-31G(d) Molecular Geometry in Cartesian Coordinates

B	0.668301	-0.256424	0.790491
O	0.461631	-1.294911	1.792125
N	-0.138406	0.922497	1.323550
C	-0.784686	0.545866	2.594329
S	0.169397	2.543370	1.046157
C	-0.310796	-0.886247	2.818934
O	-0.584248	-1.576651	3.770619
H	-2.590799	1.711739	2.686983
H	-0.382916	1.159080	3.409466
C	-2.332616	0.647957	2.677309
C	-3.151647	-0.042372	1.619094
C	-3.538294	-1.437268	1.527602
C	-4.394932	-1.561851	0.394447
N	-4.503604	-0.310632	-0.174224
C	-3.770864	0.592380	0.569781
H	-3.756176	1.638095	0.298890
C	-4.704010	-3.891784	0.801853
C	-3.277546	-2.589421	2.297252
H	-5.642373	-2.839627	-0.840581
H	-5.082138	-0.075743	-0.965413
C	-3.856931	-3.797956	1.925864
H	-5.153438	-4.848078	0.547234
H	-2.630416	-2.535307	3.166289
C	-4.983629	-2.774772	0.021871
H	-3.663147	-4.686028	2.521154
C	-1.292137	3.204690	0.224409
O	1.253226	2.626351	0.065334
O	0.255337	3.225241	2.344472
C	-3.506413	4.318446	-1.088659
C	-1.507656	2.925375	-1.126740
C	-2.159655	4.043292	0.923348
C	-3.263405	4.588035	0.264967
C	-2.611852	3.477872	-1.770708
H	-0.811036	2.292836	-1.666927
H	-1.958200	4.274518	1.963902
H	-3.938596	5.243172	0.810176
H	-2.777236	3.265211	-2.824479
C	-4.675371	4.950256	-1.806880
H	-5.511602	5.138876	-1.125814
H	-5.036125	4.317408	-2.624642
H	-4.389649	5.915132	-2.246003
H	-2.595104	0.257712	3.668670
C	2.190988	-0.185179	0.298100
C	2.576564	0.209178	-0.991751
C	3.205251	-0.589256	1.176805
C	3.914116	0.206431	-1.385530
H	1.826032	0.531538	-1.703710
C	4.549581	-0.584481	0.791065
H	2.948133	-0.919031	2.178178
C	4.912679	-0.188650	-0.493785

H	5.952988	-0.191435	-0.797586
C	5.600437	-1.084651	1.744898
C	4.290682	0.682978	-2.761466
F	5.728548	-2.432840	1.684720
F	5.304276	-0.779436	3.026964
F	6.819070	-0.564996	1.473971
F	5.418368	0.085594	-3.211689
F	4.513841	2.015506	-2.796804
F	3.313864	0.427822	-3.666822
O	-0.127710	-0.786354	-0.574671
C	-0.745140	-1.870862	-0.635575
C	-1.303180	-2.379361	-1.868279
C	-1.108134	-1.710693	-3.094312
C	-2.022308	-3.588556	-1.827073
C	-1.626766	-2.253023	-4.261251
H	-0.541637	-0.785507	-3.110129
C	-2.541996	-4.123501	-3.002145
H	-2.180305	-4.088158	-0.875713
C	-2.341849	-3.458279	-4.214364
H	-1.473203	-1.749272	-5.210779
H	-3.098920	-5.054922	-2.975463
H	-2.741558	-3.880717	-5.132283
H	-0.848875	-2.468145	0.275776

NTOB-benzaldehyde complex 22

B3LYP/6-31G(d) = -2780.355347
B3LYP-D3(BJ)/def2-TZVPP/IEFPCM(propanonitrile) = -2781.611363
B3LYP-D3(BJ)/def2-TZVPP/IEFPCM(propanonitrile)//B3LYP-D3(BJ)/6-31G(d) Free Energy (Quasiharmonic) = -2781.124732

Frequencies (Top 3 out of 213)

1. 10.4484 cm^{-1}
2. 15.9286 cm^{-1}
3. 19.0032 cm^{-1}

B3LYP/6-31G(d) Molecular Geometry in Cartesian Coordinates

B	-1.012040	-0.886707	-0.064659
O	-0.708534	-2.303836	0.012654
N	-0.702305	-0.531800	-1.519087
C	-0.225070	-1.727798	-2.234041
S	-1.296104	0.770050	-2.372189
C	-0.400357	-2.835512	-1.196313
O	-0.273189	-4.017542	-1.392167
H	1.331336	-0.871568	-3.458849
H	-0.866218	-1.945928	-3.094410
C	1.252391	-1.677959	-2.722565
C	2.280147	-1.510135	-1.639152
C	2.992048	-2.558315	-0.939150
C	3.826859	-1.925726	0.022592
N	3.649313	-0.562698	-0.111798
C	2.707378	-0.323476	-1.092396
H	2.417532	0.689203	-1.334565
C	4.644299	-4.030390	0.782960
C	3.012974	-3.962490	-1.030019
H	5.258107	-2.138697	1.639466
H	3.975747	0.121348	0.553653
C	3.837988	-4.680742	-0.174407
H	5.269671	-4.622165	1.446068
H	2.372350	-4.478569	-1.738542
C	4.648937	-2.645715	0.896317
H	3.855599	-5.765643	-0.234133
C	0.073189	1.898261	-2.674624
O	-2.226090	1.480468	-1.481753
O	-1.738892	0.283532	-3.686164
C	2.203581	3.655086	-3.164127
C	0.447143	2.820003	-1.693162
C	0.734341	1.859086	-3.903303
C	1.796224	2.732000	-4.136588
C	1.510522	3.684769	-1.944478
H	-0.090265	2.876971	-0.753478
H	0.405829	1.164846	-4.669310
H	2.311619	2.699048	-5.093350
H	1.799523	4.402637	-1.180561
C	3.332951	4.620004	-3.435491
H	2.953278	5.559880	-3.857700
H	4.049992	4.206902	-4.152158
H	3.874243	4.872329	-2.517752
O	-2.638615	-0.784901	0.112729
C	-3.388700	-1.744137	0.387148
H	-2.938198	-2.723688	0.587178
H	1.426779	-2.614107	-3.266119
C	-4.822822	-1.593446	0.435501
C	-5.609449	-2.688898	0.839850
C	-5.429289	-0.372408	0.071057
C	-6.993005	-2.561985	0.893824
H	-5.130018	-3.626304	1.110356
C	-6.811515	-0.258555	0.122748
H	-4.802732	0.449663	-0.261870
C	-7.589564	-1.348571	0.536303
H	-7.606537	-3.400518	1.208103
H	-7.290236	0.673555	-0.161012
H	-8.671093	-1.251397	0.574720
C	-0.406558	0.005658	1.118709
C	-0.917822	1.267211	1.451234
C	0.731072	-0.439073	1.812875
C	-0.301628	2.068494	2.418321
H	-1.793379	1.642889	0.931622
C	1.342886	0.357397	2.783819
H	1.146921	-1.415040	1.585188
C	0.832029	1.620715	3.091220
H	1.306081	2.239756	3.844017
C	2.607244	-0.099496	3.457349
C	-0.838803	3.451645	2.656777
F	2.691628	0.339269	4.731727
F	2.734759	-1.437796	3.472426
F	-0.323883	4.024611	3.765458
F	3.717434	0.390367	2.819348
F	-2.184054	3.457319	2.782826
F	-0.546139	4.274458	1.614084

NTOB-benzaldehyde complex 23

B3LYP/6-31G(d) = -2780.355347
B3LYP-D3(BJ)/def2-TZVPP/IEFPCM(propanonitrile) = -2781.61136
B3LYP-D3(BJ)/def2-TZVPP/IEFPCM(propanonitrile)//B3LYP-D3(BJ)/6-31G(d) Free Energy (Quasiharmonic) = -2781.124729

Frequencies (Top 3 out of 213)

1. 10.4727 cm^{-1}
2. 15.9310 cm^{-1}
3. 19.0050 cm^{-1}

B3LYP/6-31G(d) Molecular Geometry in Cartesian Coordinates

B	-1.012030	-0.886611	-0.064940
O	-0.708599	-2.303790	0.011570
N	-0.702500	-0.530930	-1.519220
C	-0.225429	-1.726570	-2.234880
S	-1.296410	0.771389	-2.371540
C	-0.400669	-2.834840	-1.197730
O	-0.273658	-4.016770	-1.394250
H	1.330890	-0.869820	-3.459440
H	-0.866699	-1.944201	-3.095290
C	1.251981	-1.676590	-2.723560
C	2.279851	-1.509379	-1.640160
C	2.991631	-2.557949	-0.940620

C	3.826571	-1.925879	0.021350
N	3.649220	-0.562769	-0.112470
C	2.707280	-0.322999	-1.092930
H	2.417590	0.689821	-1.334660
C	4.643732	-4.030968	0.780820
C	3.012362	-3.962099	-1.032080
H	5.257811	-2.139718	1.638120
H	3.975780	0.120942	0.553270
C	3.837312	-4.680809	-0.176790
H	5.269042	-4.623108	1.443670
H	2.371622	-4.477789	-1.740770
C	4.648561	-2.646348	0.894760
H	3.854773	-5.765689	-0.236970
C	0.072889	1.899650	-2.673760
O	-2.226131	1.481439	-1.480530
O	-1.739560	0.285559	-3.685650
C	2.203299	3.656551	-3.162930
C	0.733679	1.861090	-3.902660
C	0.447209	2.820830	-1.691910
C	1.510599	3.685630	-1.943070
C	1.795569	2.734031	-4.135780
H	0.404880	1.167290	-4.668940
H	-0.089911	2.877330	-0.752040
H	1.799888	4.403051	-1.178840
H	2.310679	2.701551	-5.092710
C	3.332648	4.621541	-3.434120
H	3.873978	4.873651	-2.516350
H	2.952948	5.561511	-3.856090
H	4.049658	4.208622	-4.150930
O	-2.638630	-0.784811	0.112640
C	-3.388759	-1.744152	0.386570
H	-2.938299	-2.723851	0.586000
H	1.426221	-2.612480	-3.267610
C	-4.822869	-1.593392	0.435140
C	-5.429260	-0.372082	0.071490
C	-5.609549	-2.689033	0.838850
C	-6.811480	-0.258163	0.123330
H	-4.802660	0.450148	-0.260970
C	-6.993089	-2.562053	0.892980
H	-5.130168	-3.626642	1.108740
C	-7.589579	-1.348383	0.536250
H	-7.290150	0.674157	-0.159820
H	-7.606668	-3.400743	1.206770
H	-8.671099	-1.251154	0.574790
C	-0.406380	0.005070	1.118850
C	-0.917400	1.266549	1.452040
C	0.731260	-0.440170	1.812690
C	-0.300971	2.067270	2.419440
H	-1.792941	1.642619	0.932700
C	1.343310	0.355740	2.783940
H	1.146931	-1.416090	1.584490
C	0.832689	1.619000	3.092000
H	1.306929	2.237600	3.845040
C	2.607670	-0.101659	3.457110
C	-0.837891	3.450399	2.658610
F	2.735011	-1.439989	3.471520
F	2.692260	0.336471	4.731700
F	-0.322772	4.022750	3.767510
F	3.717880	0.388361	2.819230
F	-2.183131	3.456259	2.784780
F	-0.545182	4.273670	1.616280

NTOB-benzaldehyde complex 24

B3LYP/6-31G(d) = -2780.355347
B3LYP-D3(BJ)/def2-TZVPP/IEFPCM(propanonitrile) = -2781.61136
B3LYP-D3(BJ)/def2-TZVPP/IEFPCM(propanonitrile)//B3LYP-D3(BJ)/6-31G(d) Free Energy (Quasiharmonic) = -2781.124727

Frequencies (Top 3 out of 213)

1. 10.4750 cm^{-1}
2. 15.9284 cm^{-1}
3. 18.9996 cm^{-1}

B3LYP/6-31G(d) Molecular Geometry in Cartesian Coordinates

B	-1.011979	-0.886650	-0.065363
O	-0.708496	-2.303833	0.010811
N	-0.702346	-0.530579	-1.519526
C	-0.225090	-1.726003	-2.235438
S	-1.296262	0.771904	-2.371584
C	-0.400368	-2.834547	-1.198593
O	-0.273233	-4.016421	-1.395401
H	1.331317	-0.868793	-3.459565
H	-0.866231	-1.943462	-3.095982
C	1.252380	-1.675766	-2.723916
C	2.280116	-1.508775	-1.640354
C	2.991919	-2.557494	-0.941058
C	3.826694	-1.925633	0.021195
N	3.649230	-0.562500	-0.112231
C	2.707371	-0.322526	-1.092717
H	2.417599	0.690345	-1.334175
C	4.643974	-4.030877	0.780117
C	3.012784	-3.961605	-1.032934
H	5.257785	-2.139826	1.638046
H	3.975658	0.121047	0.553736
C	3.837708	-4.680505	-0.177780
H	5.269264	-4.623159	1.442851
H	2.372171	-4.477148	-1.741857
C	4.648666	-2.646285	0.894474
H	3.855275	-5.765364	-0.238281
C	0.072993	1.900329	-2.673386
O	-2.226123	1.481662	-1.480492
O	-1.739242	0.286365	-3.685857
C	2.203346	3.657474	-3.161905
C	0.447215	2.821225	-1.691236
C	0.733864	1.862153	-3.902249
C	1.795729	2.735218	-4.135044
C	1.510578	3.686152	-1.942068
H	-0.089971	2.877421	-0.751376
H	0.405151	1.168560	-4.668755
H	2.310910	2.703039	-5.091949
H	1.799801	4.403355	-1.177609
C	3.332626	4.622638	-3.432767
H	3.873808	4.874647	-2.514881
H	4.049769	4.209943	-4.149566
H	2.952864	5.562647	-3.854612
O	-2.638592	-0.784973	0.112122
C	-3.388669	-1.744370	0.385976
H	-2.938160	-2.724060	0.585323
H	1.426759	-2.611493	-3.268200
C	-4.822785	-1.593670	0.434576
C	-5.429233	-0.372351	0.071051
C	-5.609416	-2.689378	0.838214
C	-6.811450	-0.258482	0.122937
H	-4.802667	0.449928	-0.261346
C	-6.992963	-2.562449	0.892394
H	-5.129996	-3.626994	1.108012
C	-7.589505	-1.348761	0.535784
H	-7.290159	0.673848	-0.160119
H	-7.606500	-3.401182	1.206131
H	-8.671027	-1.251573	0.574362
C	-0.406475	0.004748	1.118720
C	-0.917575	1.266123	1.452183
C	0.731080	-0.440650	1.812587
C	-0.301301	2.066595	2.419889
H	-1.793064	1.642316	0.932830
C	1.342978	0.355012	2.784139
H	1.146814	-1.416498	1.584176
C	0.832283	1.618166	3.092476

H	1.306397	2.236580	3.845750
C	2.607268	-0.102545	3.457345
C	-0.838299	3.449635	2.659381
F	3.717539	0.387693	2.819755
F	2.734644	-1.440871	3.471382
F	-0.323295	4.021709	3.768488
F	2.691677	0.335218	4.732069
F	-2.183555	3.455393	2.785456
F	-0.545549	4.273197	1.617304

NTOB-benzaldehyde complex 25

B3LYP/6-31G(d) = -2780.355346
B3LYP-D3(BJ)/def2-TZVPP/IEFPCM(propanonitrile) = -2781.61131
B3LYP-D3(BJ)/def2-TZVPP/IEFPCM(propanonitrile)//B3LYP-D3(BJ)/6-31G(d) Free Energy (Quasiharmonic) = -2781.124657

Frequencies (Top 3 out of 213)

1. 10.5964 cm^{-1}
2. 15.9035 cm^{-1}
3. 19.0163 cm^{-1}

B3LYP/6-31G(d) Molecular Geometry in Cartesian Coordinates

B	-1.010889	-0.885970	-0.070492
O	-0.704613	-2.303060	-0.004007
N	-0.700285	-0.519027	-1.521879
C	-0.219094	-1.708284	-2.245361
S	-1.298825	0.786299	-2.366262
C	-0.392780	-2.824521	-1.216515
O	-0.261656	-4.004593	-1.421227
H	1.337070	-0.836688	-3.459689
H	-0.858581	-1.921390	-3.108240
C	1.258913	-1.650322	-2.731282
C	2.284312	-1.490535	-1.644381
C	2.999682	-2.543780	-0.955641
C	3.829760	-1.919052	0.015254
N	3.646446	-0.555261	-0.103158
C	2.704912	-0.308654	-1.082406
H	2.409655	0.705399	-1.312024
C	4.654566	-4.029071	0.752420
C	3.026698	-3.946654	-1.063048
H	5.258540	-2.145045	1.632548
H	3.967095	0.121812	0.572207
C	3.852983	-4.671483	-0.214239
H	5.281036	-4.626006	1.409841
H	2.389571	-4.456997	-1.778857
C	4.653109	-2.645816	0.882135
H	3.875259	-5.755525	-0.286646
C	0.069388	1.916150	-2.667555
O	-2.226322	1.491009	-1.468753
O	-1.746230	0.306360	-3.681114
C	2.192092	3.681756	-3.159615
C	0.447807	2.832094	-1.682301
C	0.719048	1.890412	-3.902688
C	1.775309	2.769524	-4.138273
C	1.505773	3.702843	-1.935912
H	-0.086854	2.884957	-0.740739
H	0.381179	1.206598	-4.673986
H	2.276562	2.752244	-5.102900
H	1.793364	4.421170	-1.171937
C	3.355219	4.611402	-3.410466
H	3.235111	5.557764	-2.873057
H	4.298157	4.161837	-3.071827
H	3.466549	4.835743	-4.476153
O	-2.637549	-0.788451	0.106094
C	-3.386195	-1.750764	0.373651
H	-2.934191	-2.730978	0.566923
H	1.436563	-2.580825	-3.283372
C	-4.820523	-1.602524	0.422894
C	-5.428666	-0.379650	0.067481
C	-5.605649	-2.702032	0.819080
C	-6.811047	-0.268104	0.119910
H	-4.803225	0.445776	-0.259182
C	-6.989382	-2.577442	0.873876
H	-5.124932	-3.640741	1.082698
C	-7.587602	-1.362242	0.525306
H	-7.291040	0.665432	-0.156926
H	-7.601771	-3.419124	1.181903
H	-8.669267	-1.266854	0.564376
C	-0.407997	-0.001925	1.120501
C	-0.918950	1.257933	1.459897
C	0.727255	-0.452304	1.814932
C	-0.304675	2.052211	2.433970
H	-1.792655	1.637922	0.940313
C	1.337170	0.337176	2.792739
H	1.142828	-1.427120	1.581915
C	0.826675	1.598960	3.107058
H	1.299271	2.212640	3.865146
C	2.599442	-0.125108	3.466417
C	-0.841739	3.434047	2.680288
F	3.711556	0.370569	2.836001
F	2.679809	0.302882	4.744667
F	-2.187013	3.439029	2.806307
F	2.727481	-1.463425	3.470458
F	-0.549015	4.262743	1.642356
F	-0.326897	4.000593	3.792309

NTOB-benzaldehyde complex 26

B3LYP/6-31G(d) = -2780.355346
B3LYP-D3(BJ)/def2-TZVPP/IEFPCM(propanonitrile) = -2781.61131
B3LYP-D3(BJ)/def2-TZVPP/IEFPCM(propanonitrile)//B3LYP-D3(BJ)/6-31G(d) Free Energy (Quasiharmonic) = -2781.124656

Frequencies (Top 3 out of 213)

1. 10.5966 cm^{-1}
2. 15.9033 cm^{-1}
3. 19.0173 cm^{-1}

B3LYP/6-31G(d) Molecular Geometry in Cartesian Coordinates

B	-1.010916	-0.885939	-0.070374
O	-0.704677	-2.303037	-0.003907
N	-0.700382	-0.519004	-1.521780
C	-0.219277	-1.708278	-2.245291
S	-1.298941	0.786337	-2.366129
C	-0.392939	-2.824509	-1.216435
O	-0.261872	-4.004586	-1.421156
H	1.336849	-0.836748	-3.459720
H	-0.858823	-1.921361	-3.108131
C	1.258705	-1.650370	-2.731298
C	2.284170	-1.490602	-1.644458
C	2.999535	-2.543861	-0.955734
C	3.829691	-1.919146	0.015102
N	3.646427	-0.555350	-0.103329
C	2.704850	-0.308726	-1.082532
H	2.409627	0.705334	-1.312160
C	4.654458	-4.029181	0.752266
C	3.026493	-3.946739	-1.063114
H	5.258550	-2.145160	1.632324
H	3.967131	0.121722	0.572011
C	3.852797	-4.671583	-0.214335
H	5.280941	-4.626127	1.409665
H	2.389308	-4.457073	-1.778878
C	4.653060	-2.645924	0.881953
H	3.875029	-5.755627	-0.286722
C	0.069292	1.916130	-2.667551

O	-2.226336	1.491096	-1.468551
O	-1.746477	0.306400	-3.680938
C	2.192028	3.681643	-3.159807
C	0.447800	2.832099	-1.682356
C	0.718877	1.890321	-3.902721
C	1.775156	2.769385	-4.138404
C	1.505783	3.702800	-1.936063
H	-0.086802	2.885021	-0.740763
H	0.380940	1.206487	-4.673971
H	2.276353	2.752046	-5.103058
H	1.793447	4.421145	-1.172132
C	3.355152	4.611260	-3.410775
H	3.466421	4.835542	-4.476482
H	3.235079	5.557657	-2.873417
H	4.298104	4.161710	-3.072163
O	-2.637568	-0.788375	0.106295
C	-3.386236	-1.750690	0.373779
H	-2.934254	-2.730931	0.566969
H	1.436292	-2.580885	-3.283387
C	-4.820560	-1.602426	0.423037
C	-5.428683	-0.379516	0.067714
C	-5.605705	-2.701954	0.819131
C	-6.811063	-0.267954	0.120142
H	-4.803228	0.445925	-0.258884
C	-6.989437	-2.577349	0.873925
H	-5.125004	-3.640691	1.082676
C	-7.587637	-1.362114	0.525446
H	-7.291041	0.665610	-0.156627
H	-7.601840	-3.419048	1.181880
H	-8.669301	-1.266713	0.564513
C	-0.407930	-0.001908	1.120579
C	-0.918811	1.257980	1.459971
C	0.727340	-0.452318	1.814959
C	-0.304448	2.052259	2.433987
H	-1.792526	1.637992	0.940421
C	1.337344	0.337163	2.792710
H	1.142861	-1.427157	1.581942
C	0.826920	1.598977	3.107024
H	1.299588	2.212658	3.865067
C	2.599635	-0.125153	3.466329
C	-0.841437	3.434126	2.680292
F	2.680090	0.302866	4.744564
F	3.711733	0.370467	2.835840
F	-0.548740	4.262776	1.642314
F	2.727625	-1.463475	3.470398
F	-0.326503	4.000687	3.792263
F	-2.186703	3.439171	2.806393

NTOB-benzaldehyde complex 27

B3LYP/6-31G(d) = -2780.351535
B3LYP-D3(BJ)/def2-TZVPP/IEFPCM(propanonitrile) = -2781.61182
B3LYP-D3(BJ)/def2-TZVPP/IEFPCM(propanonitrile)//B3LYP-D3(BJ)/6-31G(d) Free Energy (Quasiharmonic) = -2781.124613

Frequencies (Top 3 out of 213)

1. 13.3784 cm^{-1}
2. 17.4813 cm^{-1}
3. 19.4300 cm^{-1}

B3LYP/6-31G(d) Molecular Geometry in Cartesian Coordinates

B	0.947867	-0.913329	-0.747578
O	0.886299	-1.818465	-1.884479
N	0.533822	-1.783220	0.438483
C	0.407971	-3.188661	-0.005197
S	0.996726	-1.544923	2.035884
C	0.637223	-3.095488	-1.514872
O	0.585422	-4.013824	-2.295740
H	-1.001899	-4.057247	1.366202
H	1.209390	-3.817030	0.408881
C	-0.941750	-3.895034	0.288678
C	-2.164680	-3.164457	-0.194079
C	-3.154695	-2.509736	0.627491
C	-4.116149	-1.930149	-0.246169
N	-3.736932	-2.244855	-1.534606
C	-2.563529	-2.977867	-1.497770
H	-2.109064	-3.331369	-2.413323
C	-5.357704	-1.076089	1.600831
C	-3.330286	-2.368717	2.017282
H	-5.927641	-0.752605	-0.460765
H	-4.150624	-1.860675	-2.370348
C	-4.426880	-1.656489	2.488525
H	-6.202454	-0.521140	2.000095
H	-2.609258	-2.795641	2.709036
C	-5.215982	-1.204546	0.224495
H	-4.571627	-1.542267	3.559527
C	0.392742	0.103371	2.389627
O	2.467379	-1.510149	2.171035
O	0.262659	-2.534601	2.837817
C	-0.530497	2.674201	2.998340
C	1.298396	1.079378	2.807502
C	-0.972647	0.381588	2.300763
C	-1.420982	1.664062	2.604640
C	0.830793	2.357869	3.105752
H	2.350471	0.832264	2.894087
H	-1.674069	-0.384531	1.989982
H	-2.482928	1.883074	2.528202
H	1.534986	3.123845	3.420794
C	-1.017759	4.079135	3.258061
H	-0.927597	4.687302	2.349164
H	-0.429971	4.570780	4.040459
H	-2.069606	4.090705	3.560888
O	2.532299	-0.561468	-0.542460
C	3.445074	-0.942154	-1.306403
H	3.166346	-1.417942	-2.254063
H	-0.864052	-4.880476	-0.187157
C	4.838441	-0.786392	-0.973675
C	5.805326	-1.044775	-1.965207
C	5.230726	-0.407617	0.328070
C	7.154743	-0.895165	-1.667920
H	5.489926	-1.351057	-2.959429
C	6.581994	-0.274957	0.617086
H	4.468745	-0.270977	1.088835
C	7.539039	-0.509799	-0.379111
H	7.905929	-1.082963	-2.428662
H	6.897853	0.002401	1.618013
H	8.594542	-0.401742	-0.144867
C	0.212767	0.486134	-1.013755
C	-1.060365	0.449006	-1.602330
C	0.737726	1.744135	-0.691711
C	-1.784600	1.616333	-1.850250
H	-1.493452	-0.508311	-1.870981
C	0.013024	2.915773	-0.927749
H	1.714972	1.820868	-0.228280
C	-1.253036	2.860305	-1.510449
H	-1.815202	3.768951	-1.694598
C	0.608430	4.256091	-0.595887
C	-3.173176	1.529032	-2.422995
F	1.178759	4.841016	-1.675209
F	-0.331618	5.123517	-0.140760
F	-3.309475	0.473093	-3.266453
F	1.565193	4.170977	0.355380
F	-3.507474	2.639068	-3.116567
F	-4.110890	1.371868	-1.459072

NTOB-benzaldehyde complex 28

B3LYP/6-31G(d) = -2780.351535

B3LYP-D3(BJ)/def2-TZVPP/IEFPCM(propanonitrile) = -2781.611818
B3LYP-D3(BJ)/def2-TZVPP/IEFPCM(propanonitrile)//B3LYP-D3(BJ)/6-31G(d) Free Energy (Quasiharmonic) = -2781.124612

Frequencies (Top 3 out of 213)

1. 13.3765 cm^{-1}
2. 17.4831 cm^{-1}
3. 19.4321 cm^{-1}

B3LYP/6-31G(d) Molecular Geometry in Cartesian Coordinates

B	0.947858	-0.913382	-0.747790
O	0.886160	-1.818564	-1.884651
N	0.533651	-1.783149	0.438316
C	0.407732	-3.188609	-0.005290
S	0.996756	-1.544869	2.035675
C	0.636888	-3.095542	-1.514979
O	0.584901	-4.013899	-2.295809
H	-1.001959	-4.057166	1.366312
H	1.209181	-3.816969	0.408750
C	-0.941955	-3.894956	0.288780
C	-2.164933	-3.164360	-0.193807
C	-3.154872	-2.509687	0.627897
C	-4.116423	-1.930073	-0.245638
N	-3.737320	-2.244708	-1.534128
C	-2.563922	-2.977734	-1.497443
H	-2.109551	-3.331217	-2.413052
C	-5.357796	-1.076099	1.601526
C	-3.330316	-2.368717	2.017713
H	-5.927959	-0.752553	-0.459996
H	-4.151131	-1.860536	-2.369815
C	-4.426868	-1.656518	2.489101
H	-6.202515	-0.521183	2.000899
H	-2.609214	-2.795664	2.709373
C	-5.216216	-1.204503	0.225170
H	-4.571504	-1.542339	3.560122
C	0.393010	0.103491	2.389499
O	2.467433	-1.510278	2.170626
O	0.262640	-2.534419	2.837715
C	-0.529918	2.674406	2.998299
C	-0.972403	0.381735	2.301130
C	1.298861	1.079515	2.806911
C	0.831412	2.358051	3.105199
C	-1.420588	1.664253	2.605062
H	-1.673969	-0.384390	1.990686
H	2.350961	0.832376	2.893115
H	1.535751	3.124044	3.419874
H	-2.482554	1.883289	2.529003
C	-1.017022	4.079386	3.258084
H	-0.428896	4.571070	4.040206
H	-2.068741	4.091020	3.561346
H	-0.927194	4.687478	2.349106
O	2.532310	-0.561614	-0.542690
C	3.445074	-0.942496	-1.306557
H	3.166312	-1.418466	-2.254116
H	-0.864343	-4.880400	-0.187064
C	4.838453	-0.786745	-0.973851
C	5.230757	-0.407734	0.327820
C	5.805331	-1.045393	-1.965321
C	6.582031	-0.275105	0.616823
H	4.468787	-0.270898	1.088559
C	7.154757	-0.895813	-1.668053
H	5.489920	-1.351856	-2.959482
C	7.539070	-0.510211	-0.379319
H	6.897899	0.002431	1.617697
H	7.905936	-1.083816	-2.428751
H	8.594578	-0.402177	-0.145088
C	0.212831	0.486127	-1.014010
C	0.737909	1.744133	-0.692182
C	-1.060434	0.449019	-1.602312
C	0.013176	2.915786	-0.928093
H	1.715286	1.820868	-0.229035
C	-1.784701	1.616350	-1.850113
H	-1.493611	-0.508289	-1.870823
C	-1.253030	2.860322	-1.510469
H	-1.815210	3.768973	-1.694550
C	-3.173447	1.529135	-2.422467
C	0.608642	4.256125	-0.596409
F	-4.110923	1.372346	-1.458262
F	-3.310166	0.473067	-3.265694
F	1.565789	4.171024	0.354474
F	-3.507727	2.639098	-3.116158
F	-0.331269	5.123483	-0.140890
F	1.178511	4.841108	-1.675934

NTOB-benzaldehyde complex 29

B3LYP/6-31G(d) = -2780.351535
B3LYP-D3(BJ)/def2-TZVPP/IEFPCM(propanonitrile) = -2781.611817
B3LYP-D3(BJ)/def2-TZVPP/IEFPCM(propanonitrile)//B3LYP-D3(BJ)/6-31G(d) Free Energy (Quasiharmonic) = -2781.124611

Frequencies (Top 3 out of 213)

1. 13.3753 cm^{-1}
2. 17.4908 cm^{-1}
3. 19.4327 cm^{-1}

B3LYP/6-31G(d) Molecular Geometry in Cartesian Coordinates

B	0.947881	-0.913395	-0.747615
O	0.886178	-1.818674	-1.884393
N	0.533889	-1.783109	0.438591
C	0.407959	-3.188608	-0.004903
S	0.996952	-1.544622	2.035921
C	0.637071	-3.095641	-1.514611
O	0.585141	-4.014080	-2.295356
H	-1.001785	-4.056949	1.366774
H	1.209400	-3.816932	0.409195
C	-0.941744	-3.894897	0.289219
C	-2.164717	-3.164383	-0.193518
C	-3.154692	-2.509598	0.628058
C	-4.116236	-1.930142	-0.245590
N	-3.737098	-2.244983	-1.534019
C	-2.563673	-2.977960	-1.497192
H	-2.109268	-3.331571	-2.412733
C	-5.357663	-1.075891	1.601417
C	-3.330160	-2.368396	2.017848
H	-5.927795	-0.752701	-0.460172
H	-4.150859	-1.860907	-2.369777
C	-4.426735	-1.656139	2.489102
H	-6.202399	-0.520925	2.000684
H	-2.609055	-2.795206	2.709591
C	-5.216056	-1.204522	0.225082
H	-4.571385	-1.541779	3.560101
C	0.393008	0.103710	2.389575
O	2.467629	-1.509835	2.170887
O	0.262985	-2.534200	2.838065
C	-0.530131	2.674618	2.998161
C	-0.972379	0.381972	2.300729
C	1.298703	1.079721	2.807360
C	0.831152	2.358249	3.105544
C	-1.420664	1.664482	2.604545
H	-1.673833	-0.384142	1.990010
H	2.350769	0.832586	2.893932
H	1.535380	3.124218	3.420520
H	-2.482603	1.883526	2.528120
C	-1.017338	4.079578	3.257859
H	-0.927079	4.687765	2.348981
H	-0.429583	4.571178	4.040311

H	-2.069203	4.091202	3.560608
O	2.532405	-0.561440	-0.542606
C	3.445056	-0.942306	-1.306592
H	3.166210	-1.418275	-2.254137
H	-0.864135	-4.880423	-0.186454
C	4.838460	-0.786527	-0.974041
C	5.805272	-1.044935	-1.965639
C	5.230825	-0.407704	0.327674
C	7.154709	-0.895276	-1.668458
H	5.489804	-1.351256	-2.959825
C	6.582119	-0.275000	0.616577
H	4.468886	-0.271078	1.088496
C	7.539093	-0.509847	-0.379690
H	7.905844	-1.083081	-2.429247
H	6.898053	0.002397	1.617469
H	8.594610	-0.401750	-0.145532
C	0.212705	0.485991	-1.013892
C	0.737657	1.744054	-0.692094
C	-1.060519	0.448722	-1.602277
C	0.012860	2.915631	-0.928181
H	1.714958	1.820886	-0.228805
C	-1.784847	1.615981	-1.850242
H	-1.493605	-0.508650	-1.870725
C	-1.253283	2.860028	-1.510684
H	-1.815514	3.768623	-1.694873
C	-3.173510	1.528564	-2.422766
C	0.608282	4.256000	-0.596535
F	-4.111089	1.371740	-1.458668
F	-3.507842	2.638408	-3.116622
F	1.564699	4.171038	0.355108
F	-3.309971	0.472383	-3.265893
F	-0.331804	5.123646	-0.141992
F	1.179095	4.840503	-1.675824

NTOB-benzaldehyde complex 30

B3LYP/6-31G(d) = -2780.353008
B3LYP-D3(BJ)/def2-TZVPP/IEFPCM(propanonitrile) = -2781.610811
B3LYP-D3(BJ)/def2-TZVPP/IEFPCM(propanonitrile)//B3LYP-D3(BJ)/6-31G(d) Free Energy (Quasiharmonic) = -2781.124577

Frequencies (Top 3 out of 213)

1. 7.8800 cm^{-1}
2. 12.3089 cm^{-1}
3. 15.1166 cm^{-1}

B3LYP/6-31G(d) Molecular Geometry in Cartesian Coordinates

B	-0.930332	-0.057631	1.068591
O	-0.689366	0.386838	2.423035
N	-0.111448	-1.356933	0.949723
C	0.498459	-1.655079	2.262568
S	-0.542365	-2.626553	-0.041182
C	0.008333	-0.505675	3.148022
O	0.231817	-0.397697	4.328701
H	2.340241	-2.654485	1.739409
H	0.093811	-2.588449	2.670286
C	2.050821	-1.748898	2.282930
C	2.791398	-0.564607	1.727674
C	3.203632	0.636739	2.422729
C	3.864424	1.463502	1.472122
N	3.874477	0.783605	0.270114
C	3.216731	-0.420617	0.429196
H	3.124464	-1.104696	-0.403023
C	4.266537	3.136007	3.121609
C	3.096112	1.089866	3.750854
H	4.890689	3.332179	1.059065
H	4.109708	1.189367	-0.622764
C	3.626877	2.329032	4.084844
H	4.666326	4.103362	3.414027
H	2.581566	0.492176	4.496314
C	4.394696	2.714764	1.803785
H	3.541501	2.688761	5.106639
C	0.917029	-3.118679	-0.966442
O	-1.489207	-2.066844	-1.027696
O	-0.928947	-3.786991	0.774370
C	3.189950	-3.894112	-2.412394
C	1.684078	-4.196510	-0.520976
C	1.255305	-2.438398	-2.138394
C	2.391151	-2.825510	-2.846622
C	2.815807	-4.571797	-1.243389
H	1.386555	-4.739139	0.369939
H	0.636653	-1.624488	-2.497468
H	2.657187	-2.288001	-3.753384
H	3.415346	-5.409121	-0.894991
C	4.400675	-4.326509	-3.204008
H	5.165835	-4.768005	-2.557481
H	4.850503	-3.484884	-3.740474
H	4.129633	-5.082755	-3.952580
O	-2.509608	-0.489505	1.146233
C	-3.286642	-0.544956	0.166291
H	-2.969799	-0.125823	-0.790944
H	2.324105	-1.913914	3.332181
C	-4.611308	-1.110016	0.270273
C	-5.074714	-1.668720	1.478411
C	-5.440858	-1.091274	-0.866242
C	-6.356367	-2.197405	1.540307
H	-4.418393	-1.679172	2.342144
C	-6.723990	-1.622824	-0.796105
H	-5.070286	-0.665206	-1.794838
C	-7.178586	-2.173534	0.405770
H	-6.721138	-2.631312	2.466212
H	-7.367738	-1.612159	-1.670095
H	-8.180647	-2.590045	0.460497
C	-0.753505	1.094864	-0.031859
C	0.252319	1.019383	-1.002166
C	-1.541655	2.256826	-0.015924
C	0.454876	2.043098	-1.931142
H	0.897460	0.149950	-1.018842
C	-1.347388	3.284459	-0.942986
H	-2.321946	2.370090	0.732082
C	-0.347379	3.182957	-1.910915
H	-0.204323	3.973344	-2.637959
C	-2.192742	4.528136	-0.865421
C	1.568937	1.913621	-2.931534
F	-1.708006	5.411167	0.034669
F	-2.257385	5.169564	-2.054091
F	2.785913	2.179058	-2.379343
F	-3.462390	4.243988	-0.490736
F	1.428495	2.753722	-3.975229
F	1.652860	0.655115	-3.432960

NTOB-benzaldehyde complex 31

B3LYP/6-31G(d) = -2780.353142
B3LYP-D3(BJ)/def2-TZVPP/IEFPCM(propanonitrile) = -2781.610857
B3LYP-D3(BJ)/def2-TZVPP/IEFPCM(propanonitrile)//B3LYP-D3(BJ)/6-31G(d) Free Energy (Quasiharmonic) = -2781.124469

Frequencies (Top 3 out of 213)

1. 8.4152 cm^{-1}
2. 14.8691 cm^{-1}
3. 15.9255 cm^{-1}

B3LYP/6-31G(d) Molecular Geometry in Cartesian Coordinates

B	0.915734	-0.071508	-1.078721
O	0.672238	0.364982	-2.435216

N	0.083896	-1.361244	-0.945014
C	-0.539294	-1.660897	-2.251267
S	0.511508	-2.629335	0.049210
C	-0.040790	-0.524561	-3.148890
O	-0.270647	-0.422866	-4.328887
H	-2.390746	-2.630126	-1.705483
H	-0.150103	-2.602522	-2.655050
C	-2.092979	-1.733425	-2.259123
C	-2.812239	-0.533951	-1.708402
C	-3.214286	0.666396	-2.411088
C	-3.854264	1.511410	-1.462281
N	-3.862296	0.843259	-0.253683
C	-3.222923	-0.371513	-0.407402
H	-3.131862	-1.048729	0.430564
C	-4.248712	3.173628	-3.124009
C	-3.113054	1.105328	-3.744448
H	-4.850390	3.398132	-1.057650
H	-4.082255	1.261228	0.637504
C	-3.629578	2.348623	-4.085389
H	-4.637846	4.143630	-3.422005
H	-2.613962	0.493407	-4.488880
C	-4.370137	2.766792	-1.801013
H	-3.548941	2.697366	-5.111372
C	-0.948064	-3.110086	0.980201
O	1.464256	-2.070341	1.030392
O	0.889779	-3.795245	-0.762501
C	-3.220630	-3.869170	2.435437
C	-1.278789	-2.424905	2.151518
C	-1.722531	-4.184779	0.540063
C	-2.854065	-4.551855	1.266998
C	-2.414403	-2.804027	2.864432
H	-0.654100	-1.613847	2.506735
H	-1.430849	-4.731453	-0.350314
H	-3.459387	-5.386692	0.922665
H	-2.674351	-2.263270	3.771046
C	-4.431188	-4.292653	3.232101
H	-4.161181	-5.045150	3.984810
H	-5.199115	-4.735490	2.589809
H	-4.877136	-3.446227	3.764260
O	2.489990	-0.520095	-1.160376
C	3.271104	-0.572100	-0.183431
H	2.962376	-0.139819	0.770671
H	-2.376799	-1.903442	-3.304766
C	4.590191	-1.149884	-0.287228
C	5.425890	-1.123977	0.844672
C	5.042385	-1.727586	-1.490697
C	6.704135	-1.667176	0.774437
H	5.063997	-0.682948	1.769700
C	6.319141	-2.267948	-1.552613
H	4.381320	-1.743395	-2.350721
C	7.147593	-2.236795	-0.422774
H	7.352659	-1.650895	1.644797
H	6.675263	-2.716619	-2.474842
H	8.145796	-2.662473	-0.477510
C	0.756767	1.091025	0.013686
C	-0.236331	1.027662	0.997883
C	1.544806	2.252407	-0.027178
C	-0.429665	2.064585	1.913949
H	-0.882681	0.159830	1.032087
C	1.362231	3.291391	0.889535
H	2.304734	2.363078	-0.796270
C	0.373095	3.203490	1.869806
H	0.229756	4.009614	2.579485
C	2.283060	4.481847	0.860581
C	-1.541665	1.957526	2.919126
F	2.652779	4.803113	-0.397196
F	1.717020	5.576122	1.413685
F	-1.373182	2.786866	3.967462
F	3.425193	4.238505	1.552115
F	-1.656550	0.698563	3.412200
F	-2.754370	2.260071	2.375890

NTOB-benzaldehyde complex 32

B3LYP/6-31G(d) = -2780.353142
B3LYP-D3(BJ)/def2-TZVPP/IEFPCM(propanonitrile) = -2781.610857
B3LYP-D3(BJ)/def2-TZVPP/IEFPCM(propanonitrile)//B3LYP-D3(BJ)/6-31G(d) Free Energy (Quasiharmonic) = -2781.124469

Frequencies (Top 3 out of 213)

1. 8.4126 cm^{-1}
2. 14.8641 cm^{-1}
3. 15.9168 cm^{-1}

B3LYP/6-31G(d) Molecular Geometry in Cartesian Coordinates

B	-0.915715	0.071489	-1.078749
O	-0.672245	-0.365003	-2.435245
N	-0.083921	1.361240	-0.945047
C	0.539224	1.660924	-2.251317
S	-0.511492	2.629311	0.049223
C	0.040728	0.524576	-3.148930
O	0.270557	0.422897	-4.328934
H	2.390666	2.630236	-1.705642
H	0.149992	2.602539	-2.655079
C	2.092906	1.733502	-2.259234
C	2.812238	0.534080	-1.708497
C	3.214297	-0.666287	-2.411143
C	3.854378	-1.511219	-1.462331
N	3.862449	-0.843002	-0.253770
C	3.223017	0.371734	-0.407513
H	3.131988	1.048995	0.430419
C	4.248786	-3.173515	-3.123988
C	3.112995	-1.105300	-3.744471
H	4.850612	-3.397874	-1.057657
H	4.082550	-1.260886	0.637421
C	3.629552	-2.348592	-4.085374
H	4.637943	-4.143517	-3.421954
H	2.613831	-0.493442	-4.488905
C	4.370283	-2.766597	-1.801024
H	3.548865	-2.697395	-5.111333
C	0.948078	3.109967	0.980264
O	-1.464289	2.070340	1.030370
O	-0.889689	3.795263	-0.762462
C	3.220661	3.868873	2.435561
C	1.722609	4.184634	0.540175
C	1.278746	2.424728	2.151561
C	2.414374	2.803756	2.864502
C	2.854152	4.551621	1.267140
H	1.430973	4.731353	-0.350190
H	0.654011	1.613687	2.506735
H	2.674285	2.262939	3.771090
H	3.459528	5.386433	0.922842
C	4.431198	4.292299	3.232287
H	4.877032	3.445870	3.764535
H	4.161189	5.044862	3.984934
H	5.199207	4.735046	2.590034
O	-2.489994	0.520075	-1.160396
C	-3.271088	0.572109	-0.183437
H	-2.962368	0.139796	0.770656
H	2.376679	1.903490	-3.304895
C	-4.590159	1.149930	-0.287205
C	-5.042345	1.727674	-1.490656
C	-5.425851	1.124019	0.844700
C	-6.319088	2.268068	-1.552552
H	-4.381281	1.743493	-2.350681
C	-6.704082	1.667250	0.774486
H	-5.063962	0.682963	1.769717
C	-7.147534	2.236907	-0.422710
H	-6.675204	2.716770	-2.474768

H	-7.352601	1.650966	1.644850
H	-8.145727	2.662610	-0.477430
C	-0.756747	-1.091058	0.013641
C	0.236318	-1.027696	0.997869
C	-1.544781	-2.252445	-0.027252
C	0.429612	-2.064614	1.913953
H	0.882670	-0.159864	1.032093
C	-1.362238	-3.291425	0.889467
H	-2.304682	-2.363114	-0.796371
C	-0.373146	-3.203516	1.869784
H	-0.229839	-4.009633	2.579477
C	-2.283050	-4.481893	0.860465
C	1.541538	-1.957501	2.919206
F	-1.717027	-5.576161	1.413600
F	-3.425225	-4.238567	1.551937
F	2.754345	-2.259604	2.375962
F	-2.652695	-4.803162	-0.397332
F	1.373220	-2.787109	3.967356
F	1.656071	-0.698613	3.412565

NTOB-benzaldehyde complex 33

B3LYP/6-31G(d) = -2780.353142
B3LYP-D3(BJ)/def2-TZVPP/IEFPCM(propanonitrile) = -2781.610856
B3LYP-D3(BJ)/def2-TZVPP/IEFPCM(propanonitrile)//B3LYP-D3(BJ)/6-31G(d) Free Energy (Quasiharmonic) = -2781.124468

Frequencies (Top 3 out of 213)

1. 8.4131 cm^{-1}
2. 14.8675 cm^{-1}
3. 15.9225 cm^{-1}

B3LYP/6-31G(d) Molecular Geometry in Cartesian Coordinates

B	-0.915722	-0.071510	1.078704
O	-0.672236	0.364935	2.435212
N	-0.083894	-1.361242	0.944955
C	0.539275	-1.660953	2.251205
S	-0.511498	-2.629289	-0.049330
C	0.040775	-0.524642	3.148862
O	0.270619	-0.422994	4.328866
H	2.390717	-2.630198	1.705414
H	0.150062	-2.602585	2.654949
C	2.092958	-1.733511	2.259082
C	2.812257	-0.534033	1.708420
C	3.214303	0.666286	2.411157
C	3.854333	1.511316	1.462398
N	3.862392	0.843200	0.253781
C	3.222992	-0.371565	0.407440
H	3.131953	-1.048757	-0.430548
C	4.248748	3.173480	3.124187
C	3.113030	1.105182	3.744526
H	4.850508	3.398031	1.057857
H	4.082416	1.261182	-0.637385
C	3.629564	2.348459	4.085521
H	4.637889	4.143467	3.422224
H	2.613902	0.493249	4.488922
C	4.370216	2.766679	1.801184
H	3.548897	2.697173	5.111511
C	0.948078	-3.110005	-0.980331
O	-1.464237	-2.070252	-1.030496
O	-0.889780	-3.795231	0.762330
C	3.220649	-3.869033	-2.435586
C	1.278825	-2.424760	-2.151604
C	1.722526	-4.184733	-0.540245
C	2.854065	-4.551780	-1.267190
C	2.414443	-2.803852	-2.864526
H	0.654152	-1.613670	-2.506779
H	1.430831	-4.731451	0.350100
H	3.459375	-5.386641	-0.922895
H	2.674411	-2.263040	-3.771101
C	4.431187	-4.292508	-3.232285
H	4.161132	-5.044917	-3.985069
H	5.199085	-4.735456	-2.590039
H	4.877180	-3.446059	-3.764369
O	-2.489992	-0.520108	1.160365
C	-3.271125	-0.572114	0.183436
H	-2.962422	-0.139819	-0.770670
H	2.376758	-1.903574	3.304723
C	-4.590200	-1.149923	0.287250
C	-5.425938	-1.123990	-0.844622
C	-5.042342	-1.727682	1.490711
C	-6.704169	-1.667216	-0.774364
H	-5.064085	-0.682918	-1.769645
C	-6.319084	-2.268074	1.552649
H	-4.381244	-1.743517	2.350710
C	-7.147575	-2.236892	0.422840
H	-7.352724	-1.650914	-1.644701
H	-6.675165	-2.716791	2.474871
H	-8.145768	-2.662592	0.477592
C	-0.756773	1.091052	-0.013668
C	0.236369	1.027747	-0.997825
C	-1.544862	2.252401	0.027180
C	0.429693	2.064688	-1.913872
H	0.882760	0.159943	-1.032009
C	-1.362297	3.291403	-0.889513
H	-2.304825	2.363030	0.796244
C	-0.373120	3.203555	-1.869748
H	-0.229790	4.009692	-2.579415
C	-2.283184	4.481816	-0.860585
C	1.541726	1.957670	-2.919017
F	-1.717172	5.576124	-1.413650
F	-3.425275	4.238429	-1.552172
F	1.373319	2.787105	-3.967289
F	-2.652973	4.803047	0.397180
F	1.656571	0.698741	-3.412195
F	2.754423	2.260110	-2.375709

NTOB-benzaldehyde complex 34

B3LYP/6-31G(d) = -2780.353142
B3LYP-D3(BJ)/def2-TZVPP/IEFPCM(propanonitrile) = -2781.610857
B3LYP-D3(BJ)/def2-TZVPP/IEFPCM(propanonitrile)//B3LYP-D3(BJ)/6-31G(d) Free Energy (Quasiharmonic) = -2781.124467

Frequencies (Top 3 out of 213)

1. 8.4155 cm^{-1}
2. 14.8682 cm^{-1}
3. 15.9221 cm^{-1}

B3LYP/6-31G(d) Molecular Geometry in Cartesian Coordinates

B	-0.915712	-0.071518	1.078704
O	-0.672253	0.364972	2.435203
N	-0.083879	-1.361251	0.945014
C	0.539293	-1.660901	2.251276
S	-0.511462	-2.629340	-0.049227
C	0.040753	-0.524575	3.148893
O	0.270574	-0.422890	4.328898
H	2.390776	-2.630090	1.705523
H	0.150112	-2.602534	2.655048
C	2.092979	-1.733397	2.259160
C	2.812227	-0.533909	1.708453
C	3.214218	0.666459	2.411139
C	3.854216	1.511474	1.462347
N	3.862309	0.843305	0.253758
C	3.222961	-0.371481	0.407468
H	3.131955	-1.048714	-0.430490

C	4.248556	3.173726	3.124066
C	3.112920	1.105408	3.744488
H	4.850316	3.398212	1.057728
H	4.082322	1.261255	-0.637425
C	3.629401	2.348721	4.085432
H	4.637657	4.143741	3.422063
H	2.613812	0.493487	4.488908
C	4.370046	2.766872	1.801081
H	3.548714	2.697477	5.111407
C	0.948127	-3.110071	-0.980200
O	-1.464199	-2.070352	-1.030421
O	-0.889732	-3.795257	0.762473
C	3.220754	-3.869078	-2.435379
C	1.278837	-2.424905	-2.151528
C	1.722647	-4.184709	-0.540015
C	2.854215	-4.551742	-1.266917
C	2.414487	-2.803983	-2.864410
H	0.654122	-1.613876	-2.506767
H	1.430994	-4.731359	0.350387
H	3.459592	-5.386519	-0.922535
H	2.674438	-2.263220	-3.771019
C	4.431253	-4.292632	-3.232093
H	4.876839	-3.446372	-3.764811
H	4.161258	-5.045596	-3.984351
H	5.199438	-4.734958	-2.589763
O	-2.489985	-0.520115	1.160351
C	-3.271099	-0.572138	0.183408
H	-2.962372	-0.139870	-0.770703
H	2.376785	-1.903410	3.304808
C	-4.590182	-1.149930	0.287208
C	-5.425896	-1.124013	-0.844682
C	-5.042358	-1.727655	1.490674
C	-6.704136	-1.667220	-0.774439
H	-5.064018	-0.682968	-1.769708
C	-6.319109	-2.268028	1.552597
H	-4.381279	-1.743478	2.350687
C	-7.147575	-2.236861	0.422770
H	-7.352672	-1.650930	-1.644790
H	-6.675216	-2.716717	2.474823
H	-8.145775	-2.662546	0.477511
C	-0.756753	1.091011	-0.013702
C	0.236341	1.027651	-0.997904
C	-1.544800	2.252388	0.027159
C	0.429660	2.064569	-1.913978
H	0.882702	0.159826	-1.032105
C	-1.362240	3.291368	-0.889561
H	-2.304725	2.363056	0.796256
C	-0.373110	3.203467	-1.869838
H	-0.229782	4.009587	-2.579525
C	-2.283083	4.481814	-0.860611
C	1.541645	1.957497	-2.919171
F	-2.652772	4.803103	0.397167
F	-1.717071	5.576082	-1.413756
F	1.373221	2.786923	-3.967447
F	-3.425231	4.238442	-1.552109
F	1.656426	0.698559	-3.412335
F	2.754377	2.259904	-2.375920

NTOB-benzaldehyde complex 35

B3LYP/6-31G(d) = -2780.353138
B3LYP-D3(BJ)/def2-TZVPP/IEFPCM(propanonitrile) = -2781.610805
B3LYP-D3(BJ)/def2-TZVPP/IEFPCM(propanonitrile)//B3LYP-D3(BJ)/6-31G(d) Free Energy (Quasiharmonic) = -2781.12442

Frequencies (Top 3 out of 213)

1. 8.5396 cm^{-1}
2. 15.1627 cm^{-1}
3. 16.3444 cm^{-1}

B3LYP/6-31G(d) Molecular Geometry in Cartesian Coordinates

B	0.913351	0.070601	1.076661
O	0.666616	-0.366893	2.432217
N	0.078932	1.358589	0.940586
C	-0.549929	1.655644	2.244711
S	0.511719	2.629051	-0.048403
C	-0.051920	0.520066	3.143675
O	-0.286399	0.416777	4.322586
H	-2.401990	2.617673	1.688507
H	-0.164940	2.598077	2.650708
C	-2.103823	1.723336	2.245774
C	-2.816151	0.519650	1.695124
C	-3.221855	-0.677560	2.401013
C	-3.851509	-1.529437	1.451456
N	-3.850224	-0.868269	0.238922
C	-3.214581	0.348644	0.391459
H	-3.116422	1.020822	-0.449758
C	-4.256656	-3.183002	3.119237
C	-3.131002	-1.108660	3.737630
H	-4.839841	-3.420748	1.049063
H	-4.059187	-1.292280	-0.652112
C	-3.647627	-2.351182	4.081210
H	-4.646158	-4.152188	3.419394
H	-2.639323	-0.491478	4.482669
C	-4.367518	-2.784022	1.792914
H	-3.574917	-2.694010	5.109774
C	-0.948294	3.119860	-0.973482
O	1.460495	2.069172	-1.032891
O	0.897396	3.789865	0.767225
C	-3.216304	3.901855	-2.423951
C	-1.285199	2.441655	-2.147079
C	-1.711441	4.201935	-0.531392
C	-2.839118	4.582130	-1.257422
C	-2.417133	2.833916	-2.858958
H	-0.663793	1.630966	-2.509031
H	-1.409717	4.748259	0.355845
H	-3.431104	5.427531	-0.915720
H	-2.677450	2.303890	-3.771739
C	-4.457485	4.300692	-3.185404
H	-4.368229	4.063780	-4.250406
H	-4.659582	5.372364	-3.089302
H	-5.338954	3.767862	-2.804866
O	2.486192	0.522522	1.162562
C	3.269965	0.574560	0.187702
H	2.964407	0.140625	-0.766691
H	-2.393031	1.894850	3.289668
C	4.587667	1.154838	0.294326
C	5.426758	1.128353	-0.835067
C	5.035225	1.735640	1.498051
C	6.703818	1.673945	-0.762039
H	5.068398	0.684972	-1.760346
C	6.310811	2.278416	1.562731
H	4.371506	1.752013	2.356014
C	7.142681	2.246597	0.435424
H	7.354960	1.657213	-1.630433
H	6.663361	2.729525	2.485141
H	8.139949	2.674181	0.492321
C	0.759660	-1.091837	-0.016526
C	-0.224947	-1.025631	-1.008963
C	1.543433	-2.255785	0.031574
C	-0.415190	-2.062900	-1.925152
H	-0.867355	-0.155266	-1.049413
C	1.364524	-3.294912	-0.885767
H	2.296761	-2.368351	0.806855
C	0.383144	-3.204649	-1.873583
H	0.242298	-4.011117	-2.583403
C	2.281041	-4.488481	-0.848795
C	-1.520275	-1.954050	-2.937623
F	2.640809	-4.809382	0.411934

F	1.715078	-5.581531	-1.404453
F	-1.635739	-0.693283	-3.425375
F	3.428783	-4.250002	-1.532660
F	-2.736000	-2.263056	-2.404127
F	-1.342251	-2.778140	-3.988497

NTOB-benzaldehyde complex 36

B3LYP/6-31G(d) = -2780.347189
B3LYP-D3(BJ)/def2-TZVPP/IEFPCM(propanonitrile) = -2781.610058
B3LYP-D3(BJ)/def2-TZVPP/IEFPCM(propanonitrile)//B3LYP-D3(BJ)/6-31G(d) Free Energy (Quasiharmonic) = -2781.124366

Frequencies (Top 3 out of 213)

1. 8.9314 cm^{-1}
2. 11.5504 cm^{-1}
3. 12.5539 cm^{-1}

B3LYP/6-31G(d) Molecular Geometry in Cartesian Coordinates

B	-0.752642	0.058164	0.966782
O	-0.711490	-0.052228	2.419152
N	0.306334	-0.966408	0.486543
C	0.803601	-1.703344	1.666803
S	-0.057689	-1.843551	-0.909439
C	0.113948	-1.013535	2.849605
O	0.303506	-1.281532	4.013101
H	2.812811	-2.202732	1.032949
H	0.455696	-2.745051	1.651802
C	2.339330	-1.702541	1.882186
C	2.981254	-0.355806	2.105223
C	3.876174	0.348767	1.215805
C	4.272543	1.550096	1.873118
N	3.639912	1.566274	3.096526
C	2.879818	0.422067	3.236698
H	2.315957	0.241332	4.140731
C	5.667923	2.165205	0.037634
C	4.409460	0.075763	-0.058047
H	5.453278	3.371801	1.819827
H	3.738419	2.282884	3.798922
C	5.295738	0.980622	-0.633065
H	6.369944	2.848936	-0.431961
H	4.145395	-0.838861	-0.580258
C	5.161088	2.464444	1.297403
H	5.722209	0.766883	-1.609826
C	1.445584	-2.720742	-1.362192
O	-0.290911	-0.819810	-1.940722
O	-1.061505	-2.879410	-0.630212
C	3.715959	-4.139736	-2.192142
C	2.348917	-2.123409	-2.242109
C	1.644500	-4.029358	-0.915626
C	2.782385	-4.722553	-1.322474
C	3.477335	-2.835315	-2.647695
H	2.153866	-1.125155	-2.618796
H	0.906045	-4.501194	-0.276160
H	2.940877	-5.738812	-0.970168
H	4.179322	-2.373896	-3.338128
C	4.924224	-4.915400	-2.659015
H	5.301017	-5.580067	-1.874673
H	4.675783	-5.542870	-3.525062
H	5.737728	-4.248373	-2.961196
H	2.511157	-2.341273	2.756821
C	-2.263384	0.093438	0.414483
C	-2.651028	0.730464	-0.773328
C	-3.273935	-0.502478	1.183428
C	-3.986739	0.767862	-1.177747
H	-1.905319	1.189269	-1.411995
C	-4.610270	-0.479857	0.776664
H	-3.020161	-0.980050	2.123689
C	-4.976166	0.159186	-0.406378
H	-6.013115	0.185667	-0.720152
C	-5.654357	-1.189870	1.595550
C	-4.371715	1.532729	-2.414041
F	-6.883114	-0.647530	1.426441
F	-5.373274	-1.147684	2.917036
F	-5.755125	-2.496750	1.258879
F	-5.500841	1.050984	-2.979783
F	-3.401766	1.504421	-3.353918
F	-4.603682	2.842159	-2.137942
O	-0.196479	1.547951	0.665662
C	0.801605	1.807404	-0.042819
C	1.166546	3.164641	-0.370951
C	2.345875	3.385264	-1.107064
C	0.356494	4.251963	0.019344
C	2.710754	4.683158	-1.450928
H	2.973283	2.543163	-1.384620
C	0.725969	5.541552	-0.333466
H	-0.550747	4.060635	0.582915
C	1.900918	5.755610	-1.068242
H	3.622008	4.859993	-2.013637
H	0.104870	6.383896	-0.044545
H	2.183987	6.768078	-1.343255
H	1.401359	0.974823	-0.419528

NTOB-benzaldehyde complex 37

B3LYP/6-31G(d) = -2780.344064
B3LYP-D3(BJ)/def2-TZVPP/IEFPCM(propanonitrile) = -2781.609768
B3LYP-D3(BJ)/def2-TZVPP/IEFPCM(propanonitrile)//B3LYP-D3(BJ)/6-31G(d) Free Energy (Quasiharmonic) = -2781.124344

Frequencies (Top 3 out of 213)

1. 8.9142 cm^{-1}
2. 15.4727 cm^{-1}
3. 16.9362 cm^{-1}

B3LYP/6-31G(d) Molecular Geometry in Cartesian Coordinates

B	-0.554095	0.173752	0.802423
O	-0.363360	0.418690	2.221575
N	0.411008	-0.997286	0.488664
C	1.101092	-1.372444	1.738276
S	-0.081065	-2.205320	-0.567782
C	0.527828	-0.408667	2.782165
O	0.837516	-0.393787	3.949719
H	3.025075	-2.147926	1.134361
H	0.820052	-2.383562	2.060413
C	2.654091	-1.294317	1.708935
C	3.260815	-0.032635	1.158447
C	3.481097	1.235671	1.823255
C	4.134694	2.090424	0.891191
N	4.289406	1.378177	-0.281459
C	3.776943	0.105245	-0.107918
H	3.841727	-0.628139	-0.900073
C	4.242968	3.853394	2.490902
C	3.217937	1.728434	3.116248
H	5.018121	4.031694	0.478216
H	4.787658	1.699926	-1.096199
C	3.600594	3.025145	3.434030
H	4.530891	4.862659	2.772957
H	2.711641	1.109281	3.849286
C	4.517798	3.398711	1.207027
H	3.399323	3.411138	4.429395
C	1.379448	-3.214840	-0.851682
O	-0.378813	-1.501580	-1.831048
O	-1.081857	-3.090670	0.041130
C	3.615226	-4.832050	-1.344272
C	2.268857	-2.871863	-1.871239

C	1.574113	-4.372415	-0.096177
C	2.694471	-5.164785	-0.340423
C	3.380534	-3.678580	-2.107221
H	2.074227	-1.998127	-2.483876
H	0.847655	-4.652731	0.659230
H	2.850022	-6.063097	0.251810
H	4.071890	-3.416658	-2.904720
C	4.805597	-5.717420	-1.624582
H	5.176547	-6.191154	-0.709922
H	4.539173	-6.521913	-2.322638
H	5.628641	-5.153595	-2.075088
H	2.975799	-1.448675	2.746234
C	-2.111531	0.163889	0.397086
C	-2.598273	0.413675	-0.893821
C	-3.058620	-0.073743	1.404755
C	-3.966262	0.415504	-1.171405
H	-1.912137	0.597224	-1.712628
C	-4.428778	-0.076259	1.132526
H	-2.723035	-0.254441	2.420102
C	-4.892254	0.169105	-0.158403
H	-5.955020	0.172277	-0.370911
C	-5.413918	-0.271630	2.253647
C	-4.444951	0.631532	-2.581042
F	-6.585077	-0.785081	1.812381
F	-5.708656	0.899150	2.868959
F	-4.938523	-1.103492	3.205008
F	-5.667825	1.209882	-2.614991
F	-4.542835	-0.528711	-3.268087
F	-3.603866	1.429968	-3.281703
O	0.066912	1.500398	0.108144
C	0.643044	1.475874	-1.001503
C	1.147176	2.677957	-1.625616
C	1.117648	3.916928	-0.955129
C	1.666729	2.590153	-2.930891
C	1.601313	5.051546	-1.593033
H	0.722609	3.963079	0.054104
C	2.143208	3.732365	-3.565351
H	1.678051	1.629999	-3.440580
C	2.109849	4.959624	-2.895077
H	1.584860	6.009534	-1.082617
H	2.534252	3.673006	-4.576464
H	2.482034	5.852236	-3.390492
H	0.741530	0.517093	-1.520411

NTOB-benzaldehyde complex 38

B3LYP/6-31G(d) = -2780.347299
B3LYP-D3(BJ)/def2-TZVPP/IEFPCM(propanonitrile) = -2781.610046
B3LYP-D3(BJ)/def2-TZVPP/IEFPCM(propanonitrile)//B3LYP-D3(BJ)/6-31G(d) Free Energy (Quasiharmonic) = -2781.124289

Frequencies (Top 3 out of 213)

1. 9.3144 cm^{-1}
2. 11.9422 cm^{-1}
3. 15.1239 cm^{-1}

B3LYP/6-31G(d) Molecular Geometry in Cartesian Coordinates

B	-0.754586	0.074054	0.964134
O	-0.715593	-0.007150	2.418483
N	0.310022	-0.955091	0.506462
C	0.808325	-1.666061	1.702128
S	-0.044907	-1.860786	-0.873485
C	0.112687	-0.956453	2.869561
O	0.300450	-1.200877	4.038523
H	2.821583	-2.167293	1.082644
H	0.465915	-2.709663	1.707267
C	2.343601	-1.652730	1.920685
C	2.977630	-0.298227	2.117916
C	3.870611	0.393391	1.216449
C	4.258229	1.610204	1.850080
N	3.622551	1.647487	3.071440
C	2.868880	0.501876	3.233093
H	2.303895	0.336272	4.139320
C	5.654445	2.196222	0.005744
C	4.408819	0.097686	-0.050250
H	5.428172	3.437533	1.762463
H	3.715062	2.378835	3.759309
C	5.291090	0.995956	-0.641498
H	6.353481	2.874492	-0.476061
H	4.151748	-0.829109	-0.554157
C	5.142719	2.517977	1.257952
H	5.721355	0.764895	-1.612638
C	1.461389	-2.746458	-1.298599
O	-0.271625	-0.858275	-1.927117
O	-1.050419	-2.891016	-0.580202
C	3.737389	-4.181536	-2.084203
C	2.370210	-2.167193	-2.184911
C	1.657648	-4.045496	-0.823753
C	2.798286	-4.746570	-1.208775
C	3.501410	-2.886952	-2.568277
H	2.177413	-1.177034	-2.583473
H	0.915182	-4.504235	-0.179417
H	2.954695	-5.755263	-0.834481
H	4.207744	-2.439716	-3.263585
C	4.948952	-4.966165	-2.526897
H	5.321035	-5.613902	-1.726306
H	4.706337	-5.611905	-3.381080
H	5.764060	-4.305165	-2.837867
H	2.516908	-2.272842	2.808332
C	-2.264134	0.092773	0.407816
C	-2.647194	0.683352	-0.805263
C	-3.278144	-0.469662	1.197127
C	-3.981207	0.704258	-1.216579
H	-1.900110	1.125785	-1.453491
C	-4.613668	-0.457036	0.787577
H	-3.026135	-0.921024	2.150711
C	-4.974494	0.131717	-0.422820
H	-6.010608	0.147458	-0.740247
C	-5.679083	-1.013946	1.692431
C	-4.343433	1.301083	-2.548976
F	-6.762787	-1.432772	0.999530
F	-6.118145	-0.086256	2.578129
F	-5.230853	-2.061803	2.417149
F	-4.253572	0.399300	-3.552228
F	-3.527650	2.331727	-2.877013
F	-5.611514	1.774760	-2.562097
O	-0.204620	1.560165	0.634459
C	0.791892	1.810177	-0.079611
C	1.151163	3.162363	-0.433858
C	2.329435	3.373611	-1.174364
C	0.336895	4.253666	-0.064137
C	2.689095	4.666140	-1.543037
H	2.960141	2.528856	-1.435890
C	0.701222	5.537763	-0.441465
H	-0.569524	4.069358	0.503055
C	1.875130	5.742464	-1.180543
H	3.599492	4.835841	-2.109320
H	0.076908	6.383023	-0.168407
H	2.154127	6.750631	-1.474851
H	1.394933	0.973162	-0.440896

NTOB-benzaldehyde complex 39

B3LYP/6-31G(d) = -2780.347203
B3LYP-D3(BJ)/def2-TZVPP/IEFPCM(propanonitrile) = -2781.61004
B3LYP-D3(BJ)/def2-TZVPP/IEFPCM(propanonitrile)//B3LYP-D3(BJ)/6-31G(d) Free Energy (Quasiharmonic) = -2781.124259

Frequencies (Top 3 out of 213)

1. 8.9287 cm^{-1}
2. 11.5048 cm^{-1}
3. 12.5339 cm^{-1}

B3LYP/6-31G(d) Molecular Geometry in Cartesian Coordinates

B	-0.751952	0.054737	0.966655
O	-0.710241	-0.059921	2.418679
N	0.307403	-0.967829	0.482990
C	0.805484	-1.707933	1.660898
S	-0.056560	-1.841125	-0.915420
C	0.116106	-1.021824	2.846010
O	0.306569	-1.292890	4.008648
H	2.814518	-2.204580	1.024723
H	0.458150	-2.749791	1.643062
C	2.341286	-1.706903	1.875566
C	2.982547	-0.360432	2.101844
C	3.874645	0.348246	1.212879
C	4.271569	1.547459	1.873721
N	3.641825	1.558557	3.098678
C	2.883029	0.413194	3.236379
H	2.321254	0.228668	4.140950
C	5.662165	2.170514	0.037270
C	4.405010	0.080556	-0.063321
H	5.450674	3.370326	1.824401
H	3.741144	2.272799	3.803367
C	5.289279	0.988198	-0.637033
H	6.362513	2.856577	-0.431416
H	4.139861	-0.831987	-0.588638
C	5.158022	2.464655	1.299317
H	5.713454	0.778513	-1.615673
C	1.447158	-2.717641	-1.368467
O	-0.288715	-0.814507	-1.944127
O	-1.061209	-2.877031	-0.639448
C	3.714894	-4.137850	-2.204168
C	1.643107	-4.028578	-0.927886
C	2.349591	-2.120130	-2.249501
C	3.475238	-2.833397	-2.659802
C	2.778431	-4.723395	-1.339702
H	0.900865	-4.503403	-0.295055
H	2.151841	-1.123389	-2.628794
H	4.172465	-2.374432	-3.356643
H	2.930626	-5.744148	-0.997960
C	4.952127	-4.891796	-2.628506
H	5.768244	-4.742529	-1.909158
H	4.764399	-5.968744	-2.687517
H	5.312509	-4.554069	-3.605403
H	2.513874	-2.347832	2.748434
C	-2.262893	0.090777	0.414894
C	-2.651104	0.730053	-0.771524
C	-3.273026	-0.506952	1.182989
C	-3.986956	0.767815	-1.175460
H	-1.905769	1.190363	-1.409549
C	-4.609491	-0.483927	0.776694
H	-3.018790	-0.986304	2.122217
C	-4.975951	0.157322	-0.404982
H	-6.013006	0.184106	-0.718380
C	-5.653112	-1.195854	1.594517
C	-4.372581	1.535018	-2.410108
F	-5.371550	-1.156292	2.915982
F	-5.753696	-2.502064	1.255218
F	-6.882053	-0.653453	1.426963
F	-5.501596	1.053870	-2.976569
F	-3.402830	1.509115	-3.350247
F	-4.605141	2.843761	-2.131259
O	-0.196781	1.545665	0.669962
C	0.798951	1.808183	-0.040707
C	1.162222	3.166851	-0.364803
C	0.352999	4.252273	0.032440
C	2.338884	3.390907	-1.104127
C	0.720640	5.543393	-0.316672
H	-0.552213	4.058336	0.598374
C	2.701958	4.690287	-1.444266
H	2.965711	2.550177	-1.387060
C	1.892939	5.760862	-1.054669
H	0.100129	6.384310	-0.022382
H	3.611147	4.869743	-2.009482
H	2.174562	6.774518	-1.326777
H	1.397747	0.977314	-0.422654

NTOB-benzaldehyde complex 40

B3LYP/6-31G(d) = -2780.351358
B3LYP-D3(BJ)/def2-TZVPP/IEFPCM(propanonitrile) = -2781.611091
B3LYP-D3(BJ)/def2-TZVPP/IEFPCM(propanonitrile)//B3LYP-D3(BJ)/6-31G(d) Free Energy (Quasiharmonic) = -2781.124087

Frequencies (Top 3 out of 213)

1. 13.8364 cm^{-1}
2. 16.1866 cm^{-1}
3. 19.6228 cm^{-1}

B3LYP/6-31G(d) Molecular Geometry in Cartesian Coordinates

B	0.955459	-0.885574	-0.761416
O	0.898383	-1.748679	-1.931740
N	0.502236	-1.788880	0.383007
C	0.372008	-3.177377	-0.109482
S	0.927656	-1.601316	1.997678
C	0.625351	-3.033879	-1.611623
O	0.573026	-3.923513	-2.425151
H	-1.061289	-4.086269	1.210105
H	1.162583	-3.825910	0.294399
C	-0.986096	-3.884446	0.140344
C	-2.198993	-3.128899	-0.329467
C	-3.205314	-2.516180	0.504874
C	-4.149267	-1.891619	-0.356838
N	-3.745516	-2.141689	-1.651952
C	-2.572683	-2.876403	-1.629530
H	-2.101040	-3.183655	-2.552996
C	-5.425307	-1.128950	1.506557
C	-3.407925	-2.445261	1.896370
H	-5.954969	-0.700866	-0.546744
H	-4.139917	-1.711283	-2.474403
C	-4.512629	-1.755510	2.381638
H	-6.276904	-0.592915	1.916954
H	-2.700493	-2.906961	2.579726
C	-5.257013	-1.188037	0.128470
H	-4.677743	-1.694795	3.454034
C	0.297420	0.026758	2.394657
O	2.394339	-1.556321	2.167101
O	0.186773	-2.626268	2.747343
C	-0.678983	2.552137	3.101811
C	-1.067929	0.292178	2.279297
C	1.178649	0.994610	2.879760
C	0.684559	2.250133	3.226727
C	-1.542939	1.552685	2.631975
H	-1.749913	-0.467206	1.914160
H	2.231636	0.757609	2.982378
H	1.368844	3.009188	3.597824
H	-2.605575	1.760621	2.537149
C	-1.194272	3.931075	3.434339
H	-0.989610	4.621887	2.607369
H	-0.706288	4.337910	4.326879
H	-2.274811	3.926101	3.607664
O	2.541763	-0.572315	-0.517444
C	3.463753	-0.953104	-1.270168
H	3.197306	-1.399957	-2.235327

H	-0.908025	-4.851990	-0.370919
C	4.851708	-0.833376	-0.902172
C	5.836383	-1.090322	-1.876426
C	5.220409	-0.490222	0.416322
C	7.181076	-0.974849	-1.544633
H	5.538512	-1.368803	-2.884140
C	6.566861	-0.391334	0.739349
H	4.443353	-0.353562	1.161752
C	7.542314	-0.624728	-0.239183
H	7.946199	-1.161811	-2.291566
H	6.864443	-0.141405	1.753018
H	8.593929	-0.543179	0.021777
C	0.253286	0.537150	-0.992171
C	0.837164	1.778792	-0.717750
C	-1.042627	0.539379	-1.533099
C	0.155364	2.975360	-0.966373
H	1.839433	1.826818	-0.304419
C	-1.725535	1.728725	-1.787797
H	-1.521386	-0.405703	-1.766691
C	-1.130008	2.959078	-1.503647
H	-1.651922	3.885564	-1.711071
C	-3.130557	1.679330	-2.323939
C	0.809816	4.281286	-0.610395
F	-3.473367	2.818627	-2.963093
F	-3.300624	0.659255	-3.205761
F	2.128326	4.281563	-0.914457
F	-4.041964	1.489148	-1.341314
F	0.727208	4.542534	0.720871
F	0.246452	5.329447	-1.248216

NTOB-benzaldehyde complex 41

B3LYP/6-31G(d) = -2780.351358
B3LYP-D3(BJ)/def2-TZVPP/IEFPCM(propanonitrile) = -2781.611091
B3LYP-D3(BJ)/def2-TZVPP/IEFPCM(propanonitrile)//B3LYP-D3(BJ)/6-31G(d) Free Energy (Quasiharmonic) = -2781.124086

Frequencies (Top 3 out of 213)

1. 13.8364 cm^{-1}
2. 16.1871 cm^{-1}
3. 19.6228 cm^{-1}

B3LYP/6-31G(d) Molecular Geometry in Cartesian Coordinates

B	0.955446	-0.885683	-0.761324
O	0.898318	-1.748964	-1.931515
N	0.502270	-1.788814	0.383254
C	0.372027	-3.177388	-0.109018
S	0.927730	-1.600991	1.997885
C	0.625307	-3.034116	-1.611191
O	0.572952	-3.923874	-2.424582
H	-1.061214	-4.086092	1.210762
H	1.162621	-3.825856	0.294930
C	-0.986064	-3.884426	0.140968
C	-2.198984	-3.128957	-0.328907
C	-3.205283	-2.516133	0.505382
C	-4.149275	-1.891706	-0.356385
N	-3.745566	-2.141955	-1.651478
C	-2.572723	-2.876650	-1.628993
H	-2.101110	-3.184028	-2.552433
C	-5.425258	-1.128787	1.506947
C	-3.407846	-2.445017	1.896875
H	-5.954999	-0.701003	-0.546397
H	-4.140002	-1.711670	-2.473976
C	-4.512541	-1.755211	2.382084
H	-6.276847	-0.592704	1.917297
H	-2.700382	-2.906610	2.580271
C	-5.257012	-1.188068	0.128862
H	-4.677617	-1.694344	3.454478
C	0.297479	0.027135	2.394629
O	2.394417	-1.555943	2.167260
O	0.186883	-2.625837	2.747729
C	-0.678941	2.552608	3.101421
C	-1.067881	0.292507	2.279292
C	1.178712	0.995084	2.879530
C	0.684614	2.250652	3.226319
C	-1.542900	1.553062	2.631789
H	-1.749867	-0.466950	1.914312
H	2.231709	0.758122	2.982132
H	1.368901	3.009783	3.597257
H	-2.605545	1.760961	2.536981
C	-1.194243	3.931589	3.433751
H	-0.706247	4.338565	4.326220
H	-2.274778	3.926627	3.607098
H	-0.989605	4.622279	2.606673
O	2.541762	-0.572393	-0.517468
C	3.463719	-0.953290	-1.270177
H	3.197231	-1.400280	-2.235261
H	-0.908006	-4.852045	-0.370154
C	4.851689	-0.833512	-0.902255
C	5.220443	-0.490169	0.416176
C	5.836325	-1.090601	-1.876511
C	6.566909	-0.391239	0.739135
H	4.443418	-0.353399	1.161617
C	7.181032	-0.975085	-1.544787
H	5.538414	-1.369226	-2.884173
C	7.542323	-0.624777	-0.239402
H	6.864532	-0.141165	1.752757
H	7.946125	-1.162156	-2.291724
H	8.593948	-0.543194	0.021505
C	0.253264	0.537007	-0.992262
C	0.837154	1.778691	-0.718049
C	-1.042673	0.539155	-1.533132
C	0.155342	2.975222	-0.966818
H	1.839440	1.826777	-0.304768
C	-1.725593	1.728463	-1.787973
H	-1.521443	-0.405961	-1.766560
C	-1.130055	2.958858	-1.504032
H	-1.651980	3.885313	-1.711571
C	-3.130640	1.678986	-2.324043
C	0.809801	4.281203	-0.611056
F	-4.042001	1.488948	-1.341347
F	-3.300746	0.658779	-3.205705
F	0.727185	4.542677	0.720165
F	-3.473482	2.818187	-2.963350
F	0.246446	5.329259	-1.249057
F	2.128313	4.281421	-0.915111

NTOB-benzaldehyde complex 42

B3LYP/6-31G(d) = -2780.353828
B3LYP-D3(BJ)/def2-TZVPP/IEFPCM(propanonitrile) = -2781.609883
B3LYP-D3(BJ)/def2-TZVPP/IEFPCM(propanonitrile)//B3LYP-D3(BJ)/6-31G(d) Free Energy (Quasiharmonic) = -2781.123993

Frequencies (Top 3 out of 213)

1. 8.6296 cm^{-1}
2. 12.2770 cm^{-1}
3. 14.1894 cm^{-1}

B3LYP/6-31G(d) Molecular Geometry in Cartesian Coordinates

B	-0.380875	0.862294	0.936935
O	0.242905	0.727952	2.237391
N	0.775037	0.655862	-0.041881
C	2.027637	0.412299	0.705660
S	0.644482	0.151832	-1.628283
C	1.572262	0.508043	2.168079

O	2.283051	0.424672	3.138606
H	2.975715	2.354592	0.945196
H	2.397405	-0.605419	0.530581
C	3.178394	1.418704	0.410000
C	4.536563	0.885895	0.766589
C	5.397662	0.107554	-0.093600
C	6.564317	-0.211105	0.655890
N	6.408081	0.352325	1.906497
C	5.189804	0.999800	1.968250
H	4.861971	1.472534	2.882681
C	7.487870	-1.401012	-1.192412
C	5.297902	-0.348724	-1.421266
H	8.500945	-1.193797	0.708199
H	7.061836	0.274329	2.669497
C	6.341020	-1.097447	-1.954376
H	8.286644	-1.987789	-1.638215
H	4.418889	-0.119330	-2.017569
C	7.616240	-0.961072	0.120559
H	6.274692	-1.454868	-2.978630
C	0.307396	-1.608290	-1.628885
O	1.956178	0.341123	-2.261505
O	-0.540965	0.848410	-2.159086
C	-0.228062	-4.351280	-1.456014
C	1.359726	-2.504532	-1.419877
C	-1.001105	-2.067092	-1.801914
C	-1.255791	-3.432396	-1.715295
C	1.080677	-3.867673	-1.329440
H	2.382272	-2.147904	-1.349661
H	-1.802397	-1.365309	-2.000949
H	-2.274623	-3.790440	-1.838246
H	1.895975	-4.565671	-1.157591
C	-0.541312	-5.819049	-1.298289
H	-1.141427	-6.193359	-2.135820
H	-1.121992	-5.987416	-0.382724
H	0.369312	-6.422288	-1.236056
H	3.140870	1.641381	-0.658767
C	-1.751516	0.034875	0.811822
C	-2.834502	0.408928	0.007173
C	-1.866101	-1.169030	1.526410
C	-3.976926	-0.389858	-0.096875
H	-2.796158	1.325770	-0.570703
C	-2.999880	-1.975575	1.418356
H	-1.056807	-1.481781	2.177664
C	-4.066610	-1.589347	0.605860
H	-4.953318	-2.208070	0.528055
C	-3.050284	-3.313744	2.104263
C	-5.067263	0.005657	-1.053529
F	-4.302173	-3.627505	2.507077
F	-2.247069	-3.368410	3.185139
F	-2.654499	-4.310027	1.267261
F	-4.769330	-0.377897	-2.319811
F	-5.250452	1.345171	-1.086593
F	-6.253772	-0.558721	-0.740419
O	-0.823912	2.440702	0.906035
C	-0.692008	3.192658	-0.085625
C	-1.122787	4.570592	-0.067517
C	-0.954021	5.336269	-1.237229
C	-1.697716	5.147884	1.083539
C	-1.356751	6.667459	-1.256651
H	-0.512610	4.879134	-2.118998
C	-2.095880	6.476675	1.055601
H	-1.819057	4.541556	1.975271
C	-1.925504	7.233693	-0.112073
H	-1.230729	7.262466	-2.155708
H	-2.539298	6.930527	1.936548
H	-2.239985	8.273602	-0.127248
H	-0.250400	2.784526	-0.999789

NTOB-benzaldehyde complex 43

B3LYP/6-31G(d) = -2780.353828
B3LYP-D3(BJ)/def2-TZVPP/IEFPCM(propanonitrile) = -2781.609884
B3LYP-D3(BJ)/def2-TZVPP/IEFPCM(propanonitrile)//B3LYP-D3(BJ)/6-31G(d) Free Energy (Quasiharmonic) = -2781.123993

Frequencies (Top 3 out of 213)

1. 8.6221 cm^{-1}
2. 12.2838 cm^{-1}
3. 14.1948 cm^{-1}

B3LYP/6-31G(d) Molecular Geometry in Cartesian Coordinates

B	0.380706	0.862220	-0.936978
O	-0.243067	0.727742	-2.237418
N	-0.775147	0.655566	0.041860
C	-2.027723	0.411824	-0.705651
S	-0.644485	0.151635	1.628286
C	-1.572383	0.507575	-2.168079
O	-2.283171	0.424015	-3.138588
H	-2.975958	2.354046	-0.945173
H	-2.397385	-0.605923	-0.530527
C	-3.178564	1.418129	-0.409998
C	-4.536683	0.885213	-0.766597
C	-5.397725	0.106817	0.093604
C	-6.564340	-0.211969	-0.655895
N	-6.408132	0.351433	-1.906519
C	-5.189913	0.999018	-1.968271
H	-4.862128	1.471775	-2.882708
C	-7.487820	-1.401890	1.192433
C	-5.297942	-0.349411	1.421285
H	-8.500883	-1.194829	-0.708201
H	-7.061880	0.273379	-2.669519
C	-6.341006	-1.098203	1.954404
H	-8.286550	-1.988719	1.638245
H	-4.418954	-0.119930	2.017589
C	-7.616207	-0.962007	-0.120555
H	-6.274662	-1.455588	2.978669
C	-0.306956	-1.608399	1.628948
O	-1.956245	0.340601	2.261474
O	0.540769	0.848537	2.159085
C	0.229164	-4.351251	1.456001
C	-1.359082	-2.504894	1.419982
C	1.001657	-2.066884	1.801932
C	1.256674	-3.432126	1.715278
C	-1.079705	-3.867964	1.329509
H	-2.381712	-2.148500	1.349809
H	1.802785	-1.364911	2.000956
H	2.275599	-3.789922	1.838174
H	-1.894837	-4.566160	1.157676
C	0.542778	-5.818924	1.298122
H	-0.367690	-6.422436	1.236241
H	1.123131	-5.987086	0.382306
H	1.143359	-6.193074	2.135386
H	-3.141066	1.640798	0.658773
C	1.751541	0.035123	-0.811877
C	2.834389	0.409358	-0.007126
C	1.866428	-1.168718	-1.526522
C	3.976960	-0.389209	0.096984
H	2.795804	1.326160	0.570794
C	3.000356	-1.975045	-1.418411
H	1.057246	-1.481599	-2.177853
C	4.066937	-1.588651	-0.605799
H	4.953752	-2.207213	-0.527935
C	3.051068	-3.313184	-2.104354
C	5.067115	0.006470	1.053776
F	2.248119	-3.367912	-3.185424
F	2.655175	-4.309525	-1.267462
F	4.303086	-3.626816	-2.506861
F	4.769025	-0.377022	2.320037
F	5.250174	1.346003	1.086773

F	6.253725	-0.557818	0.740883
O	0.823441	2.440739	-0.906083
C	0.691149	3.192759	0.085471
C	1.121726	4.570758	0.067388
C	1.697078	5.148003	-1.083481
C	0.952344	5.336543	1.236940
C	2.095029	6.476859	-1.055521
H	1.818913	4.541589	-1.975086
C	1.354853	6.667799	1.256382
H	0.510630	4.879439	2.118573
C	1.924020	7.233988	0.111988
H	2.538773	6.930673	-1.936323
H	1.228348	7.262890	2.155316
H	2.238338	8.273947	0.127181
H	0.249357	2.784634	0.999546

NTOB-benzaldehyde complex 44

B3LYP/6-31G(d) = -2780.347472
B3LYP-D3(BJ)/def2-TZVPP/IEFPCM(propanonitrile) = -2781.609775
B3LYP-D3(BJ)/def2-TZVPP/IEFPCM(propanonitrile)//B3LYP-D3(BJ)/6-31G(d) Free Energy (Quasiharmonic) = -2781.123825

Frequencies (Top 3 out of 213)

1. 9.9413 cm^{-1}
2. 11.6532 cm^{-1}
3. 14.8493 cm^{-1}

B3LYP/6-31G(d) Molecular Geometry in Cartesian Coordinates

B	-0.222614	-1.061855	-0.419925
O	0.312593	-2.411895	-0.364790
N	0.159017	-0.546191	-1.792713
C	1.025518	-1.546454	-2.451501
S	-0.796744	0.403665	-2.809898
C	1.042875	-2.711244	-1.463625
O	1.621873	-3.758539	-1.616212
H	2.335205	-0.336472	-3.624981
H	0.563129	-1.909693	-3.375515
C	2.453809	-1.076932	-2.828721
C	3.309889	-0.511599	-1.729068
C	4.177330	-1.222409	-0.812166
C	4.836679	-0.246070	-0.011080
N	4.368076	0.989288	-0.407582
C	3.467491	0.822320	-1.442768
H	2.995534	1.681089	-1.898479
C	6.052757	-1.935312	1.152301
C	4.479181	-2.582498	-0.598489
H	6.273915	0.178431	1.561071
H	4.691880	1.877029	-0.056999
C	5.407194	-2.922701	0.378249
H	6.782708	-2.233897	1.900136
H	3.981320	-3.352231	-1.179039
C	5.776433	-0.585047	0.968291
H	5.645645	-3.969310	0.546241
C	-0.591593	2.089975	-2.220089
O	-2.215507	0.070945	-2.647630
O	-0.164712	0.321900	-4.134894
C	-0.301504	4.739912	-1.351341
C	-1.546590	2.647093	-1.370296
C	0.488742	2.850943	-2.672283
C	0.627670	4.165777	-2.232943
C	-1.387080	3.960877	-0.930112
H	-2.410125	2.067764	-1.068167
H	1.189149	2.424099	-3.382724
H	1.463305	4.762645	-2.591609
H	-2.128966	4.381598	-0.256021
C	-0.159725	6.176356	-0.906694
H	0.890911	6.482132	-0.859983
H	-0.607539	6.337060	0.079392
H	-0.664230	6.855262	-1.606787
H	2.951066	-1.945811	-3.277093
C	-1.691906	-0.915598	0.194078
C	-2.117781	0.216994	0.895502
C	-2.616628	-1.960242	0.033456
C	-3.426848	0.332483	1.373489
H	-1.428518	1.037100	1.066024
C	-3.916089	-1.860977	0.530477
H	-2.319944	-2.861904	-0.492032
C	-4.336261	-0.705480	1.193562
H	-5.352476	-0.619301	1.559442
C	-4.864056	-3.024243	0.404925
C	-3.842141	1.623944	2.017608
F	-6.150278	-2.616002	0.316024
F	-4.788953	-3.842312	1.483049
F	-4.599722	-3.780199	-0.680342
F	-2.937816	2.042522	2.935569
F	-5.037224	1.541094	2.634156
F	-3.938144	2.626439	1.099705
O	0.709122	-0.186710	0.678459
C	1.540799	-0.711758	1.446958
C	2.204968	0.028894	2.497399
C	1.927790	1.392974	2.720700
C	3.114951	-0.653182	3.325710
C	2.552246	2.057150	3.766875
H	1.224325	1.904036	2.071629
C	3.735590	0.019749	4.374110
H	3.335366	-1.699422	3.134361
C	3.452350	1.370045	4.593936
H	2.339447	3.106110	3.950028
H	4.437097	-0.503114	5.016672
H	3.933495	1.893881	5.415440
H	1.755546	-1.780075	1.337424

NTOB-benzaldehyde complex 45

B3LYP/6-31G(d) = -2780.350858
B3LYP-D3(BJ)/def2-TZVPP/IEFPCM(propanonitrile) = -2781.610606
B3LYP-D3(BJ)/def2-TZVPP/IEFPCM(propanonitrile)//B3LYP-D3(BJ)/6-31G(d) Free Energy (Quasiharmonic) = -2781.1238

Frequencies (Top 3 out of 213)

1. 11.1877 cm^{-1}
2. 14.6221 cm^{-1}
3. 16.6844 cm^{-1}

B3LYP/6-31G(d) Molecular Geometry in Cartesian Coordinates

B	0.901685	-0.915468	-0.733779
O	0.810563	-1.820748	-1.867580
N	0.484328	-1.773984	0.458978
C	0.362306	-3.183301	0.023925
S	0.959522	-1.532848	2.053547
C	0.570143	-3.096715	-1.488985
O	0.511955	-4.019138	-2.264699
H	-1.031307	-4.037058	1.420379
H	1.174638	-3.802683	0.430724
C	-0.976407	-3.899729	0.339206
C	-2.211992	-3.202765	-0.160103
C	-3.204419	-2.526956	0.641162
C	-4.187658	-2.009933	-0.247672
N	-3.815125	-2.377590	-1.523044
C	-2.629077	-3.086594	-1.466473
H	-2.177743	-3.475530	-2.368971
C	-5.425784	-1.091241	1.570593
C	-3.368068	-2.322337	2.024390
H	-6.027922	-0.884461	-0.497097
H	-4.268640	-2.072981	-2.370270

C	-4.473496	-1.609267	2.473828
H	-6.277555	-0.534925	1.952703
H	-2.632332	-2.703548	2.727416
C	-5.296466	-1.283955	0.200362
H	-4.609037	-1.446761	3.539799
C	0.363544	0.117025	2.411489
O	2.431065	-1.503832	2.179162
O	0.227860	-2.518315	2.863071
C	-0.546550	2.685679	3.047709
C	-0.998073	0.408097	2.307993
C	1.271679	1.078880	2.856201
C	0.810617	2.356368	3.167988
C	-1.439724	1.689621	2.625435
H	-1.701373	-0.347150	1.975484
H	2.320635	0.821912	2.951774
H	1.516901	3.111501	3.503747
H	-2.498611	1.919259	2.538266
C	-1.025415	4.090251	3.324090
H	-0.903941	4.715457	2.430639
H	-0.453327	4.558814	4.131925
H	-2.084731	4.107591	3.599217
O	2.501887	-0.601630	-0.545053
C	3.398490	-1.003195	-1.316756
H	3.101090	-1.473106	-2.261832
H	-0.885678	-4.895083	-0.113029
C	4.798277	-0.878963	-0.996438
C	5.750542	-1.164008	-1.994655
C	5.209981	-0.503856	0.300342
C	7.105561	-1.045262	-1.708935
H	5.419842	-1.466811	-2.984972
C	6.566370	-0.401830	0.577747
H	4.457652	-0.345100	1.066489
C	7.509430	-0.663653	-0.425028
H	7.845824	-1.253956	-2.474931
H	6.896865	-0.127337	1.574739
H	8.569081	-0.579544	-0.199838
C	0.209533	0.505365	-0.998140
C	0.770277	1.740219	-0.643385
C	-1.049222	0.521229	-1.616926
C	0.091912	2.939189	-0.872883
H	1.738739	1.774723	-0.157360
C	-1.726219	1.718987	-1.861339
H	-1.513748	-0.417121	-1.903310
C	-1.161227	2.936742	-1.486504
H	-1.692995	3.865682	-1.657642
C	-3.045637	1.693827	-2.584606
C	0.719405	4.253541	-0.498894
F	-2.879243	1.576915	-3.923672
F	-3.815095	0.645460	-2.201519
F	1.672360	4.117706	0.449843
F	-3.765364	2.817185	-2.373658
F	-0.200621	5.129716	-0.019768
F	1.304635	4.856650	-1.560233

NTOB-benzaldehyde complex 46

B3LYP/6-31G(d) = -2780.353573
B3LYP-D3(BJ)/def2-TZVPP/IEFPCM(propanonitrile) = -2781.609391
B3LYP-D3(BJ)/def2-TZVPP/IEFPCM(propanonitrile)//B3LYP-D3(BJ)/6-31G(d) Free Energy (Quasiharmonic) = -2781.123713

Frequencies (Top 3 out of 213)

1. 10.0051 cm^{-1}
2. 10.6912 cm^{-1}
3. 15.1070 cm^{-1}

B3LYP/6-31G(d) Molecular Geometry in Cartesian Coordinates

B	-0.473111	0.811546	0.907841
O	0.113482	0.700380	2.228406
N	0.724952	0.671900	-0.030503
C	1.963009	0.491649	0.757024
S	0.679533	0.171031	-1.621432
C	1.454227	0.556132	2.203398
O	2.136381	0.509907	3.196973
H	2.815819	2.469442	1.057315
H	2.389283	-0.505808	0.590204
C	3.070780	1.556678	0.504965
C	4.446691	1.078487	0.872912
C	5.377988	0.411403	-0.007247
C	6.538153	0.108526	0.758781
N	6.310498	0.573772	2.038318
C	5.054958	1.145735	2.101706
H	4.671075	1.534195	3.033727
C	7.586636	-0.896077	-1.132605
C	5.346108	0.042809	-1.365265
H	8.527131	-0.763321	0.811387
H	6.945043	0.482478	2.815951
C	6.447441	-0.606645	-1.911438
H	8.432064	-1.403935	-1.589108
H	4.473459	0.261886	-1.974741
C	7.648468	-0.541432	0.210631
H	6.433612	-0.895510	-2.959109
C	0.558774	-1.616448	-1.631328
O	1.967749	0.517669	-2.236379
O	-0.571031	0.729351	-2.164423
C	0.365469	-4.405503	-1.481989
C	1.721741	-2.381820	-1.508265
C	-0.695506	-2.226709	-1.717917
C	-0.781285	-3.613034	-1.643019
C	1.612736	-3.770358	-1.430429
H	2.696092	-1.904322	-1.491451
H	-1.584859	-1.620332	-1.842622
H	-1.757278	-4.087958	-1.692028
H	2.514888	-4.367727	-1.326903
C	0.240867	-5.905069	-1.362931
H	-0.411750	-6.172648	-0.523172
H	1.213625	-6.378969	-1.201608
H	-0.200895	-6.341455	-2.267246
H	3.041925	1.803262	-0.558590
C	-1.802321	-0.076671	0.743872
C	-1.855335	-1.312106	1.409320
C	-2.915069	0.284388	-0.026907
C	-2.956505	-2.161380	1.284924
H	-1.023661	-1.617807	2.035378
C	-4.030576	-0.550857	-0.136899
H	-2.921917	1.220570	-0.573885
C	-4.056219	-1.782937	0.514622
H	-4.917377	-2.435123	0.424834
C	-5.235927	-0.088426	-0.910459
C	-2.934065	-3.528239	1.913777
F	-4.892142	0.680661	-1.967359
F	-6.066195	0.656213	-0.141014
F	-5.963449	-1.125405	-1.381721
F	-4.168921	-3.933676	2.285325
F	-2.142166	-3.577556	3.003575
F	-2.466297	-4.464540	1.045197
O	-0.988048	2.369128	0.868233
C	-0.884800	3.119910	-0.127560
C	-1.378593	4.476528	-0.121777
C	-1.236562	5.242111	-1.295149
C	-1.988394	5.033468	1.021421
C	-1.700412	6.553002	-1.325969
H	-0.767409	4.800827	-2.170649
C	-2.447620	6.342087	0.982016
H	-2.088347	4.427573	1.916102
C	-2.303636	7.099071	-0.189241
H	-1.595191	7.147828	-2.227808
H	-2.918466	6.780112	1.856706
H	-2.665983	8.123108	-0.213421

H	-0.419070	2.727326	-1.036640

NTOB-benzaldehyde complex 47

B3LYP/6-31G(d) = -2780.344504
B3LYP-D3(BJ)/def2-TZVPP/IEFPCM(propanonitrile) = -2781.608323
B3LYP-D3(BJ)/def2-TZVPP/IEFPCM(propanonitrile)//B3LYP-D3(BJ)/6-31G(d) Free Energy (Quasiharmonic) = -2781.123546

Frequencies (Top 3 out of 213)

1. 8.4765 cm^{-1}
2. 9.8585 cm^{-1}
3. 11.5443 cm^{-1}

B3LYP/6-31G(d) Molecular Geometry in Cartesian Coordinates

B	-0.613006	0.022101	0.784009
O	-0.492971	0.358278	2.199542
N	0.367396	-1.144211	0.595145
C	0.956909	-1.480900	1.906688
S	-0.034242	-2.432540	-0.416402
C	0.316577	-0.484338	2.872178
O	0.519720	-0.420050	4.059744
H	2.946025	-1.994510	1.223122
H	0.651759	-2.489031	2.213908
C	2.505733	-1.383177	2.016098
C	3.100556	0.000929	2.000871
C	3.611634	0.752725	0.876355
C	4.123279	1.981162	1.384580
N	3.907433	1.977430	2.747854
C	3.312196	0.784119	3.111117
H	3.063231	0.581735	4.143261
C	4.806370	2.665303	-0.795498
C	3.710425	0.505124	-0.504798
H	5.114273	3.870575	0.973978
H	4.232979	2.679769	3.393806
C	4.303678	1.457612	-1.323719
H	5.269573	3.388900	-1.460699
H	3.329795	-0.421721	-0.921459
C	4.722697	2.942980	0.563784
H	4.388809	1.269622	-2.390795
C	1.534354	-3.178796	-0.881985
O	-0.602048	-1.837654	-1.630749
O	-0.769395	-3.457427	0.339935
C	3.930966	-4.390471	-1.682689
C	2.232881	-2.669475	-1.979414
C	2.001612	-4.301208	-0.198275
C	3.200259	-4.892532	-0.597921
C	3.425852	-3.274178	-2.367379
H	1.829714	-1.824070	-2.527125
H	1.423605	-4.712453	0.622544
H	3.567508	-5.765261	-0.063640
H	3.970905	-2.879760	-3.221743
C	5.211567	-5.053066	-2.131262
H	5.960285	-4.313068	-2.434238
H	5.033952	-5.706762	-2.995282
H	5.643970	-5.669395	-1.337037
H	2.765577	-1.865514	2.965359
C	-2.143042	0.012419	0.299001
C	-2.544458	0.303811	-1.014230
C	-3.151338	-0.237028	1.240508
C	-3.892699	0.346353	-1.369227
H	-1.796669	0.495839	-1.773787
C	-4.503935	-0.208759	0.886422
H	-2.883841	-0.436897	2.272841
C	-4.883174	0.086659	-0.420655
H	-5.931170	0.131161	-0.692882
C	-5.551272	-0.533139	1.917106
C	-4.284074	0.628801	-2.794023
F	-5.748174	-1.866631	2.032776
F	-6.750278	0.014592	1.610096
F	-5.205260	-0.082646	3.144631
F	-5.496478	1.226439	-2.874545
F	-4.355525	-0.498609	-3.536056
F	-3.394512	1.445113	-3.408858
O	0.037241	1.280307	-0.045799
C	0.452384	2.327601	0.492451
C	0.907295	3.465489	-0.271258
C	0.948933	3.434614	-1.679483
C	1.281228	4.631592	0.422225
C	1.354102	4.563855	-2.377380
H	0.660288	2.526624	-2.197922
C	1.683046	5.759866	-0.283928
H	1.247055	4.643557	1.508511
C	1.717717	5.723872	-1.681109
H	1.386347	4.549315	-3.462463
H	1.966050	6.664066	0.246107
H	2.029511	6.606030	-2.233610
H	0.441708	2.389987	1.585960

NTOB-benzaldehyde complex 48

B3LYP/6-31G(d) = -2780.34863
B3LYP-D3(BJ)/def2-TZVPP/IEFPCM(propanonitrile) = -2781.609646
B3LYP-D3(BJ)/def2-TZVPP/IEFPCM(propanonitrile)//B3LYP-D3(BJ)/6-31G(d) Free Energy (Quasiharmonic) = -2781.123315

Frequencies (Top 3 out of 213)

1. 7.9608 cm^{-1}
2. 13.2725 cm^{-1}
3. 17.3459 cm^{-1}

B3LYP/6-31G(d) Molecular Geometry in Cartesian Coordinates

B	0.083970	-0.218082	-1.103542
O	0.143728	-0.804835	-2.430867
N	-0.539259	-1.310023	-0.237943
C	-1.148979	-2.302383	-1.144861
S	-0.032351	-1.749388	1.295484
C	-0.449157	-2.020028	-2.482053
O	-0.472684	-2.721963	-3.460866
H	-2.937457	-2.922146	-2.120174
H	-0.956163	-3.320451	-0.801668
C	-2.671486	-2.115938	-1.422565
C	-3.628130	-2.066599	-0.263232
C	-4.851779	-1.294261	-0.253475
C	-5.502405	-1.544258	0.988276
N	-4.714714	-2.439054	1.679106
C	-3.595416	-2.746580	0.930875
H	-2.835414	-3.400856	1.328183
C	-7.291432	-0.075608	0.409208
C	-5.468197	-0.425158	-1.171909
H	-7.195393	-1.143775	2.288341
H	-4.886093	-2.771661	2.615304
C	-6.674742	0.176647	-0.833390
H	-8.237272	0.404769	0.644742
H	-5.013426	-0.229020	-2.139859
C	-6.715348	-0.939671	1.334544
H	-7.156956	0.846728	-1.539995
C	1.738949	-2.044016	1.241835
O	-0.658161	-3.045051	1.601316
O	-0.261522	-0.581278	2.159648
C	4.499513	-2.499702	1.132151
C	2.235576	-3.087645	0.457021
C	2.597182	-1.249402	2.001406
C	3.969239	-1.486406	1.941858
C	3.609883	-3.303355	0.403636
H	1.562032	-3.728107	-0.103887

H	2.198082	-0.447555	2.611391
H	4.639402	-0.860302	2.525582
H	3.998821	-4.107233	-0.216154
C	5.991743	-2.689457	1.012512
H	6.245994	-3.705819	0.695607
H	6.399928	-1.995516	0.267066
H	6.500526	-2.488304	1.961239
H	-2.793864	-1.190739	-2.001115
C	1.421668	0.566700	-0.699556
C	1.490055	1.478709	0.360294
C	2.594037	0.345379	-1.442196
C	2.685611	2.124584	0.689360
H	0.614059	1.675811	0.969843
C	3.786388	0.993437	-1.121387
H	2.568586	-0.341398	-2.280470
C	3.840842	1.887808	-0.049992
H	4.767452	2.392605	0.200774
C	5.049651	0.732039	-1.895778
C	2.735193	2.997506	1.911876
F	4.854165	-0.077875	-2.953279
F	5.996614	0.148644	-1.112479
F	5.598061	1.880158	-2.355709
F	2.872364	2.252831	3.039830
F	1.604516	3.722654	2.063012
F	3.772346	3.861872	1.885287
O	-1.039411	0.984588	-1.232207
C	-1.926286	1.186605	-0.369680
C	-2.811627	2.323566	-0.431171
C	-3.836867	2.426686	0.528465
C	-2.662477	3.316314	-1.422617
C	-4.703791	3.513940	0.498515
H	-3.954467	1.647257	1.275642
C	-3.528220	4.399702	-1.441144
H	-1.866127	3.218915	-2.153215
C	-4.546188	4.497422	-0.481525
H	-5.500890	3.593603	1.230646
H	-3.418396	5.171694	-2.196636
H	-5.221814	5.348222	-0.502243
H	-2.021811	0.471733	0.454929

NTOB-benzaldehyde complex 49

B3LYP/6-31G(d) = -2780.348733
B3LYP-D3(BJ)/def2-TZVPP/IEFPCM(propanonitrile) = -2781.609561
B3LYP-D3(BJ)/def2-TZVPP/IEFPCM(propanonitrile)//B3LYP-D3(BJ)/6-31G(d) Free Energy (Quasiharmonic) = -2781.123065

Frequencies (Top 3 out of 213)

1. 11.9736 cm^{-1}
2. 14.1796 cm^{-1}
3. 17.2372 cm^{-1}

B3LYP/6-31G(d) Molecular Geometry in Cartesian Coordinates

B	-0.083448	-0.207960	1.114217
O	-0.138930	-0.788671	2.444337
N	0.533646	-1.305492	0.252131
C	1.142360	-2.297411	1.159982
S	0.041336	-1.739199	-1.286987
C	0.447491	-2.006921	2.497884
O	0.470028	-2.705533	3.479063
H	2.929704	-2.920492	2.135219
H	0.943793	-3.315774	0.820932
C	2.666334	-2.116359	1.434243
C	3.622087	-2.076013	0.273825
C	4.842891	-1.299349	0.254758
C	5.494277	-1.561473	-0.984097
N	4.710018	-2.467527	-1.664052
C	3.592014	-2.770291	-0.912054
H	2.834458	-3.431804	-1.301736
C	7.278309	-0.080034	-0.422487
C	5.456657	-0.417759	1.163027
H	7.185525	-1.170137	-2.289144
H	4.881582	-2.809056	-2.596999
C	6.661074	0.184246	0.817332
H	8.222420	0.400881	-0.663794
H	5.001610	-0.212190	2.128893
C	6.704996	-0.956691	-1.337639
H	7.141176	0.863948	1.516145
C	-1.733359	-2.015975	-1.258177
O	0.658484	-3.040491	-1.586065
O	0.292265	-0.572819	-2.148222
C	-4.498897	-2.451964	-1.204797
C	-2.254321	-3.049105	-0.474787
C	-2.569495	-1.221551	-2.041522
C	-3.944412	-1.448747	-2.010012
C	-3.630622	-3.254890	-0.449496
H	-1.597495	-3.688932	0.106391
H	-2.152041	-0.428032	-2.649879
H	-4.596539	-0.822567	-2.613778
H	-4.038588	-4.050599	0.168596
C	-5.994111	-2.636341	-1.119791
H	-6.486090	-2.376900	-2.062965
H	-6.409551	-1.986164	-0.339704
H	-6.260201	-3.667585	-0.866392
H	2.793124	-1.188976	2.008256
C	-1.423088	0.574791	0.712420
C	-1.500747	1.476448	-0.358651
C	-2.587042	0.365355	1.466186
C	-2.697412	2.117163	-0.684246
H	-0.629193	1.666597	-0.976759
C	-3.785661	1.009342	1.146811
H	-2.552876	-0.306772	2.317397
C	-3.850018	1.887667	0.066642
H	-4.777432	2.389908	-0.181236
C	-5.023162	0.700280	1.945226
C	-2.759820	2.977193	-1.915176
F	-5.987360	1.631325	1.775437
F	-4.762702	0.618914	3.266779
F	-5.562734	-0.493759	1.584503
F	-3.783014	3.857713	-1.876085
F	-2.934207	2.222395	-3.031927
F	-1.622087	3.682869	-2.100185
O	1.040512	0.995002	1.232916
C	1.924247	1.192908	0.366162
C	2.809646	2.330300	0.419022
C	3.829663	2.430079	-0.546498
C	2.665544	3.326703	1.407518
C	4.696333	3.517759	-0.525408
H	3.943225	1.647952	-1.291481
C	3.531121	4.410348	1.417369
H	1.873209	3.231797	2.142800
C	4.543801	4.504807	0.451838
H	5.489310	3.595032	-1.262265
H	3.425245	5.185075	2.170624
H	5.219265	5.355877	0.465706
H	2.016553	0.474920	-0.456053

NTOB-benzaldehyde complex 50

B3LYP/6-31G(d) = -2780.349158
B3LYP-D3(BJ)/def2-TZVPP/IEFPCM(propanonitrile) = -2781.609626
B3LYP-D3(BJ)/def2-TZVPP/IEFPCM(propanonitrile)//B3LYP-D3(BJ)/6-31G(d) Free Energy (Quasiharmonic) = -2781.122965

Frequencies (Top 3 out of 213)

1. 9.2510 cm^{-1}
2. 16.3620 cm^{-1}

3. 17.9681 cm^{-1}

B3LYP/6-31G(d) Molecular Geometry in Cartesian Coordinates

B	0.428989	-1.064281	-0.318315
O	-0.201716	-2.113468	-1.094618
N	0.164986	-1.466042	1.133771
C	-0.448415	-2.813957	1.157283
S	1.138009	-1.094569	2.451135
C	-0.574722	-3.165238	-0.324210
O	-0.949761	-4.218927	-0.773681
H	-1.626367	-2.716985	2.942465
H	0.223525	-3.554196	1.611427
C	-1.818402	-2.920570	1.886354
C	-2.905433	-2.023320	1.368221
C	-3.848446	-2.285964	0.300901
C	-4.673027	-1.133300	0.176861
N	-4.266907	-0.230333	1.140202
C	-3.201092	-0.766411	1.837521
H	-2.751817	-0.223187	2.657128
C	-5.909875	-2.137597	-1.594903
C	-4.093051	-3.383467	-0.546651
H	-6.305080	-0.142583	-0.856889
H	-4.552822	0.736123	1.178795
C	-5.117716	-3.298977	-1.479663
H	-6.698795	-2.100046	-2.341368
H	-3.474556	-4.273141	-0.486835
C	-5.699202	-1.040351	-0.769885
H	-5.309675	-4.140057	-2.140402
C	1.237781	0.694077	2.399418
O	2.509662	-1.612267	2.273644
O	0.398063	-1.509305	3.651362
C	1.431815	3.488638	2.406611
C	0.080999	1.465898	2.522652
C	2.491320	1.298990	2.310320
C	2.579158	2.690198	2.311949
C	0.185702	2.853350	2.522023
H	-0.887475	0.985524	2.595917
H	3.379324	0.680720	2.243724
H	3.554621	3.163348	2.232961
H	-0.715348	3.455788	2.605586
C	1.529963	4.993378	2.341298
H	1.492643	5.331955	1.298344
H	0.703940	5.474469	2.874890
H	2.470902	5.353759	2.769731
O	2.045831	-1.249572	-0.566777
C	2.535879	-2.066915	-1.375767
H	1.858907	-2.586827	-2.063828
H	-2.122044	-3.971566	1.805491
C	3.947692	-2.353132	-1.420581
C	4.811437	-1.893627	-0.403670
C	4.447567	-3.124856	-2.487897
C	6.164421	-2.195462	-0.474926
H	4.392555	-1.350211	0.437112
C	5.806402	-3.409288	-2.556206
H	3.768660	-3.486486	-3.255995
C	6.660568	-2.943257	-1.551208
H	6.837329	-1.859737	0.308053
H	6.201044	-3.995544	-3.380107
H	7.721284	-3.173669	-1.600318
C	0.075526	0.424886	-0.792347
C	-1.278061	0.766385	-0.941020
C	1.024291	1.430310	-1.019502
C	-1.665612	2.067723	-1.271877
H	-2.040291	0.008184	-0.790920
C	0.641220	2.731372	-1.355754
H	2.080510	1.212144	-0.906804
C	-0.708108	3.060405	-1.479041
H	-1.008489	4.071430	-1.728251
C	1.685278	3.780425	-1.620490
C	-3.127784	2.416181	-1.326724
F	1.261009	5.016741	-1.253200
F	2.833484	3.538640	-0.947412
F	-3.637191	2.619407	-0.073888
F	2.009373	3.858496	-2.931679
F	-3.867922	1.437565	-1.882683
F	-3.360665	3.551271	-2.018727

NTOB-benzaldehyde complex 51

B3LYP/6-31G(d) = -2780.349158
B3LYP-D3(BJ)/def2-TZVPP/IEFPCM(propanonitrile) = -2781.609625
B3LYP-D3(BJ)/def2-TZVPP/IEFPCM(propanonitrile)//B3LYP-D3(BJ)/6-31G(d) Free Energy (Quasiharmonic) = -2781.122965

Frequencies (Top 3 out of 213)

1. 9.2501 cm^{-1}
2. 16.3632 cm^{-1}
3. 17.9672 cm^{-1}

B3LYP/6-31G(d) Molecular Geometry in Cartesian Coordinates

B	0.428999	-1.064275	-0.318409
O	-0.201616	-2.113475	-1.094760
N	0.164935	-1.466049	1.133654
C	-0.448372	-2.814005	1.157126
S	1.137883	-1.094527	2.451061
C	-0.574658	-3.165253	-0.324380
O	-0.949694	-4.218931	-0.773879
H	-1.626316	-2.717275	2.942327
H	0.223628	-3.554213	1.611236
C	-1.818348	-2.920752	1.886195
C	-2.905406	-2.023476	1.368168
C	-3.848457	-2.286050	0.300866
C	-4.673051	-1.133383	0.176940
N	-4.266894	-0.230478	1.140325
C	-3.201052	-0.766603	1.837569
H	-2.751752	-0.223446	2.657207
C	-5.909979	-2.137573	-1.594829
C	-4.093089	-3.383495	-0.546752
H	-6.305170	-0.142615	-0.856657
H	-4.552811	0.735974	1.178988
C	-5.117799	-3.298951	-1.479710
H	-6.698938	-2.099980	-2.341250
H	-3.474580	-4.273166	-0.487027
C	-5.699276	-1.040381	-0.769746
H	-5.309781	-4.139987	-2.140499
C	1.237649	0.694121	2.399331
O	2.509547	-1.612216	2.273637
O	0.397877	-1.509241	3.651259
C	1.431684	3.488683	2.406637
C	2.491196	1.299039	2.310353
C	0.080859	1.465940	2.522496
C	0.185563	2.853394	2.521928
C	2.579035	2.690245	2.312035
H	3.379205	0.680767	2.243833
H	-0.887622	0.985567	2.595665
H	-0.715493	3.455829	2.605446
H	3.554507	3.163397	2.233158
C	1.529837	4.993422	2.341349
H	0.703715	5.474520	2.874777
H	2.470703	5.353802	2.769941
H	1.492707	5.331980	1.298378
O	2.045885	-1.249542	-0.566803
C	2.536015	-2.066963	-1.375657
H	1.859110	-2.586992	-2.063696
H	-2.121954	-3.971749	1.805212
C	3.947847	-2.353128	-1.420366
C	4.811546	-1.893401	-0.403519
C	4.447783	-3.125030	-2.487523

C	6.164544	-2.195198	-0.474676
H	4.392626	-1.349827	0.437139
C	5.806631	-3.409430	-2.555736
H	3.768913	-3.486828	-3.255575
C	6.660750	-2.943181	-1.550801
H	6.837417	-1.859296	0.308258
H	6.201317	-3.995827	-3.379515
H	7.721475	-3.173564	-1.599835
C	0.075546	0.424892	-0.792434
C	-1.278044	0.766407	-0.941037
C	1.024313	1.430305	-1.019631
C	-1.665595	2.067751	-1.271879
H	-2.040278	0.008219	-0.790890
C	0.641243	2.731368	-1.355872
H	2.080534	1.212127	-0.906975
C	-0.708089	3.060418	-1.479097
H	-1.008469	4.071446	-1.728295
C	1.685298	3.780413	-1.620642
C	-3.127769	2.416215	-1.326651
F	2.009215	3.858613	-2.931866
F	2.833585	3.538524	-0.947742
F	-3.360666	3.551373	-2.018539
F	1.261115	5.016708	-1.253169
F	-3.637139	2.619319	-0.073784
F	-3.867914	1.437652	-1.882689

NTOB-benzaldehyde complex 52

B3LYP/6-31G(d) = -2780.351328
B3LYP-D3(BJ)/def2-TZVPP/IEFPCM(propanonitrile) = -2781.609223
B3LYP-D3(BJ)/def2-TZVPP/IEFPCM(propanonitrile)//B3LYP-D3(BJ)/6-31G(d) Free Energy (Quasiharmonic) = -2781.122766

Frequencies (Top 3 out of 213)

1. 10.7928 cm^{-1}
2. 13.2251 cm^{-1}
3. 17.2891 cm^{-1}

B3LYP/6-31G(d) Molecular Geometry in Cartesian Coordinates

B	-1.306470	-0.275033	-0.866192
O	-1.433034	-0.775707	-2.228356
N	-0.812369	1.168230	-1.057013
C	-0.604051	1.426013	-2.493851
S	-1.168401	2.448427	-0.045325
C	-1.143118	0.160654	-3.158555
O	-1.261433	-0.021798	-4.345843
H	1.241787	2.555507	-2.441139
H	-1.208139	2.278599	-2.822656
C	0.867003	1.661434	-2.949320
C	1.813492	0.508654	-2.730175
C	2.906043	0.438047	-1.787542
C	3.497021	-0.849154	-1.919845
N	2.814464	-1.509617	-2.920020
C	1.804169	-0.697061	-3.395465
H	1.153339	-1.039933	-4.187593
C	5.093140	-0.358129	-0.220475
C	3.459344	1.337188	-0.859083
H	4.994473	-2.265228	-1.237381
H	2.920171	-2.487660	-3.141350
C	4.543070	0.933762	-0.089935
H	5.934118	-0.647827	0.403835
H	3.047067	2.334391	-0.741083
C	4.579234	-1.266339	-1.137597
H	4.973672	1.623903	0.630776
C	0.378143	3.167480	0.521716
O	-1.817111	1.881840	1.147581
O	-1.847820	3.487206	-0.835159
C	2.705487	4.386841	1.495009
C	1.054671	2.596717	1.602502
C	0.844045	4.340284	-0.074713
C	2.007505	4.936612	0.410987
C	2.211004	3.210198	2.077299
H	0.683634	1.695209	2.076916
H	0.287088	4.787759	-0.890917
H	2.370342	5.850976	-0.052216
H	2.737196	2.765719	2.918262
C	3.939011	5.062063	2.045660
H	3.680897	5.736808	2.872708
H	4.443997	5.661009	1.281103
H	4.655704	4.331741	2.435641
O	-2.822784	-0.212038	-0.294640
C	-3.840054	-0.623654	-0.890403
H	-3.717726	-1.119195	-1.860644
H	0.806644	1.910504	-4.016261
C	-5.159248	-0.452498	-0.334096
C	-6.260000	-1.023592	-1.001190
C	-5.346082	0.288183	0.852830
C	-7.538870	-0.869374	-0.478334
H	-6.102719	-1.584662	-1.918936
C	-6.628068	0.441165	1.362144
H	-4.484195	0.742146	1.332811
C	-7.719154	-0.139011	0.700906
H	-8.392556	-1.309868	-0.983829
H	-6.786005	1.014077	2.270614
H	-8.719518	-0.015261	1.106621
C	-0.509031	-1.242657	0.127432
C	0.276534	-2.282067	-0.397137
C	-0.484205	-1.047410	1.513285
C	1.080658	-3.069535	0.425392
H	0.274485	-2.458711	-1.465988
C	0.326068	-1.833242	2.340606
H	-1.084128	-0.255975	1.952364
C	1.116218	-2.845663	1.804653
H	1.753474	-3.444211	2.445822
C	0.359800	-1.519356	3.810060
C	1.930253	-4.172205	-0.143222
F	0.861766	-0.276964	4.036558
F	-0.878424	-1.534224	4.354768
F	1.523853	-5.391114	0.281886
F	1.119614	-2.387189	4.511077
F	3.226772	-4.049430	0.226753
F	1.907114	-4.202402	-1.499713

NTOB-benzaldehyde complex 53

B3LYP/6-31G(d) = -2780.349103
B3LYP-D3(BJ)/def2-TZVPP/IEFPCM(propanonitrile) = -2781.6096
B3LYP-D3(BJ)/def2-TZVPP/IEFPCM(propanonitrile)//B3LYP-D3(BJ)/6-31G(d) Free Energy (Quasiharmonic) = -2781.122678

Frequencies (Top 3 out of 213)

1. 7.5155 cm^{-1}
2. 12.5548 cm^{-1}
3. 22.1494 cm^{-1}

B3LYP/6-31G(d) Molecular Geometry in Cartesian Coordinates

B	0.309675	-1.020366	-0.542376
O	-0.436059	-1.968898	-1.346888
N	0.163031	-1.578793	0.895290
C	-0.605531	-2.848509	0.846598
S	1.325392	-1.521167	2.092727
C	-0.885919	-3.031576	-0.645519
O	-1.427835	-3.986735	-1.143537
H	-1.605645	-2.728980	2.732363
H	0.006133	-3.699856	1.173953
C	-1.907723	-2.871142	1.691354

C	-2.964626	-1.878584	1.300324
C	-4.066654	-2.081430	0.383340
C	-4.808478	-0.869196	0.344178
N	-4.198779	0.015039	1.213373
C	-3.087675	-0.591797	1.765729
H	-2.471701	-0.058186	2.475673
C	-6.353343	-1.799722	-1.212708
C	-4.505828	-3.167637	-0.396499
H	-6.489424	0.231310	-0.478936
H	-4.392151	1.004254	1.254970
C	-5.641232	-3.017025	-1.181122
H	-7.233293	-1.711395	-1.844290
H	-3.947786	-4.098497	-0.406369
C	-5.947435	-0.710088	-0.452935
H	-5.983864	-3.848211	-1.791459
C	1.659801	0.223970	2.283361
O	2.584024	-2.158928	1.625038
O	0.722844	-2.044518	3.324546
C	2.200514	2.947514	2.631278
C	2.918101	0.723622	1.946937
C	0.678495	1.056802	2.825275
C	0.954753	2.410044	2.991578
C	3.177675	2.081881	2.121808
H	3.675511	0.051540	1.558937
H	-0.282473	0.647199	3.116611
H	0.191317	3.063324	3.406260
H	4.153741	2.477443	1.853308
C	2.467569	4.427732	2.751684
H	1.869628	4.881704	3.548314
H	3.523719	4.630271	2.957104
H	2.211481	4.935355	1.813539
O	1.864328	-1.152945	-1.030014
C	2.489897	-2.237394	-0.964320
H	1.965864	-3.141372	-0.646653
H	-2.305885	-3.889601	1.607986
C	3.871194	-2.362081	-1.362158
C	4.585103	-1.267062	-1.889137
C	4.498273	-3.613717	-1.219419
C	5.910513	-1.431281	-2.266565
H	4.084701	-0.311162	-2.002668
C	5.827975	-3.769086	-1.595597
H	3.941394	-4.449120	-0.804005
C	6.530311	-2.679480	-2.118613
H	6.466456	-0.594352	-2.677915
H	6.317389	-4.731360	-1.483015
H	7.568361	-2.801616	-2.415267
C	-0.051866	0.518660	-0.820247
C	-1.399394	0.892840	-0.942465
C	0.908781	1.534743	-0.925237
C	-1.768357	2.228888	-1.131103
H	-2.171559	0.132154	-0.896617
C	0.545509	2.869774	-1.114573
H	1.960667	1.290654	-0.843861
C	-0.798475	3.227189	-1.214024
H	-1.085242	4.262914	-1.358408
C	1.593875	3.937563	-1.256592
C	-3.221256	2.614258	-1.177201
F	2.800417	3.543529	-0.786285
F	1.254243	5.065369	-0.579295
F	-3.427543	3.749143	-1.877875
F	1.776007	4.306406	-2.544962
F	-3.713766	2.851999	0.079385
F	-3.995741	1.653167	-1.713142

NTOB-benzaldehyde complex 54

B3LYP/6-31G(d) = -2780.351487

B3LYP-D3(BJ)/def2-TZVPP/IEFPCM(propanonitrile) = -2781.608601

B3LYP-D3(BJ)/def2-TZVPP/IEFPCM(propanonitrile)//B3LYP-D3(BJ)/6-31G(d) Free Energy (Quasiharmonic) = -2781.12233

Frequencies (Top 3 out of 213)

1. 7.6554 cm^{-1}
2. 12.4371 cm^{-1}
3. 15.3171 cm^{-1}

B3LYP/6-31G(d) Molecular Geometry in Cartesian Coordinates

B	-1.298210	-0.228440	-0.848890
O	-1.460190	-0.712220	-2.213230
N	-0.733510	1.191390	-1.030750
C	-0.517740	1.444580	-2.468200
S	-1.065710	2.485890	-0.027800
C	-1.113140	0.208140	-3.139400
O	-1.227430	0.031950	-4.327900
H	1.366020	2.509270	-2.435080
H	-1.086010	2.322960	-2.793860
C	0.961440	1.616550	-2.922150
C	1.862010	0.436670	-2.661220
C	2.958990	0.366810	-1.724160
C	3.493600	-0.949250	-1.795590
N	2.775290	-1.628910	-2.756210
C	1.796410	-0.798860	-3.265620
H	1.127290	-1.152180	-4.037750
C	5.127200	-0.440330	-0.137110
C	3.558590	1.287610	-0.847180
H	4.939900	-2.389450	-1.058660
H	2.839820	-2.620850	-2.925070
C	4.632220	0.878400	-0.066940
H	5.962820	-0.732820	0.493170
H	3.188850	2.305970	-0.777200
C	4.565930	-1.371030	-1.002410
H	5.098580	1.584570	0.615170
C	0.497730	3.213670	0.477950
O	-1.677070	1.933670	1.191500
O	-1.773200	3.513970	-0.807590
C	2.849070	4.453430	1.363410
C	0.942700	4.377960	-0.150120
C	1.204440	2.664090	1.550500
C	2.370620	3.289510	1.983580
C	2.117250	4.985560	0.293160
H	0.358070	4.814240	-0.952910
H	0.846230	1.772530	2.052950
H	2.916860	2.865910	2.822520
H	2.460400	5.896850	-0.190540
C	4.128800	5.106050	1.829330
H	5.003610	4.633940	1.362900
H	4.154770	6.169180	1.569770
H	4.251990	5.016250	2.913770
O	-2.803970	-0.094440	-0.263420
C	-3.846100	-0.453930	-0.850120
H	-3.758130	-0.946870	-1.825390
H	0.915240	1.839130	-3.995740
C	-5.149110	-0.227250	-0.276190
C	-5.286810	0.506600	0.921680
C	-6.283340	-0.737580	-0.936460
C	-6.554110	0.713840	1.448200
H	-4.398720	0.912370	1.397040
C	-7.547330	-0.529180	-0.396530
H	-6.163480	-1.294290	-1.862470
C	-7.679090	0.194420	0.793260
H	-6.674150	1.282070	2.365360
H	-8.426760	-0.922480	-0.896780
H	-8.667710	0.360470	1.212430
C	-0.547200	-1.252240	0.125920
C	-0.406310	-1.017970	1.501170
C	0.060170	-2.401060	-0.399530
C	0.344990	-1.875500	2.308530
H	-0.876260	-0.144250	1.942560
C	0.806380	-3.264740	0.406930

H	-0.042790	-2.617220	-1.457650
C	0.960200	-3.004170	1.768520
H	1.545080	-3.666020	2.395240
C	1.482390	-4.450880	-0.221750
C	0.477090	-1.547050	3.769710
F	1.861890	-5.371970	0.687620
F	2.608030	-4.095400	-0.905970
F	0.939590	-0.283450	3.951880
F	0.681230	-5.069010	-1.119080
F	1.323030	-2.378150	4.414670
F	-0.714250	-1.611950	4.408910

NTOB-benzaldehyde complex 55

B3LYP/6-31G(d) = -2780.345826
B3LYP-D3(BJ)/def2-TZVPP/IEFPCM(propanonitrile) = -2781.607477
B3LYP-D3(BJ)/def2-TZVPP/IEFPCM(propanonitrile)//B3LYP-D3(BJ)/6-31G(d) Free Energy (Quasiharmonic) = -2781.121951

Frequencies (Top 3 out of 213)

1. 10.9095 cm^{-1}
2. 13.2624 cm^{-1}
3. 14.4678 cm^{-1}

B3LYP/6-31G(d) Molecular Geometry in Cartesian Coordinates

B	-0.297241	-0.029330	-0.360389
O	-0.687917	-0.057431	-1.762815
N	-0.309046	-1.491039	0.029994
C	-0.790792	-2.322080	-1.080181
S	0.463846	-2.189990	1.329575
C	-0.972938	-1.302992	-2.207192
O	-1.334236	-1.545939	-3.331493
H	-1.949196	-3.779726	-0.000788
H	-0.016652	-3.037453	-1.393456
C	-2.106306	-3.118925	-0.856620
C	-3.352011	-2.295460	-0.675099
C	-3.918359	-1.780905	0.551281
C	-5.104144	-1.071691	0.203835
N	-5.241474	-1.155657	-1.166583
C	-4.193826	-1.892902	-1.684365
H	-4.114662	-2.068618	-2.748219
C	-5.502997	-0.513193	2.489354
C	-3.541668	-1.847078	1.905959
H	-6.815992	0.088173	0.876064
H	-6.006895	-0.779508	-1.703716
C	-4.332126	-1.211738	2.856705
H	-6.105483	-0.037174	3.258533
H	-2.642317	-2.380580	2.198817
C	-5.906440	-0.436081	1.159947
H	-4.047907	-1.257264	3.904285
C	2.069658	-2.713919	0.714708
O	-0.272840	-3.413212	1.680780
O	0.670961	-1.127595	2.316490
C	4.537535	-3.484130	-0.367452
C	2.238262	-4.015113	0.238423
C	3.125177	-1.797770	0.676785
C	4.344239	-2.185420	0.129576
C	3.471376	-4.389349	-0.297394
H	1.421533	-4.725730	0.307386
H	2.991425	-0.799866	1.077433
H	5.154870	-1.462948	0.078453
H	3.605032	-5.403303	-0.666357
C	5.873023	-3.894413	-0.940451
H	6.268797	-3.127793	-1.616012
H	5.799801	-4.833619	-1.497310
H	6.615972	-4.037881	-0.145166
H	-2.220830	-3.752258	-1.744034
C	0.945879	0.937247	-0.070169
C	1.122533	1.649185	1.126259
C	1.938712	1.066117	-1.052729
C	2.240322	2.461239	1.328355
H	0.387070	1.560139	1.916989
C	3.072920	1.856039	-0.843248
H	1.829096	0.543210	-1.997616
C	3.227255	2.564561	0.347079
H	4.095077	3.194756	0.503938
C	4.169785	1.878310	-1.871732
C	2.418430	3.183167	2.637788
F	3.693015	1.743642	-3.127982
F	5.048908	0.860810	-1.677270
F	4.886429	3.023178	-1.828417
F	1.232349	3.516175	3.194262
F	3.128832	4.325425	2.487309
F	3.080334	2.427375	3.542285
O	-1.545831	0.607495	0.528350
C	-2.581195	1.295153	0.476045
C	-3.049770	2.201095	-0.556801
C	-4.278569	2.843671	-0.293201
C	-2.343759	2.501181	-1.740599
C	-4.796018	3.766905	-1.193421
H	-4.819184	2.602384	0.618211
C	-2.868519	3.427979	-2.633400
H	-1.413707	1.994338	-1.961318
C	-4.087685	4.059857	-2.362804
H	-5.741385	4.259757	-0.987752
H	-2.328177	3.659456	-3.546169
H	-4.487015	4.784156	-3.067648
H	-3.218764	1.223431	1.366683

NTOB-benzaldehyde complex 56

B3LYP/6-31G(d) = -2780.351844
B3LYP-D3(BJ)/def2-TZVPP/IEFPCM(propanonitrile) = -2781.607607
B3LYP-D3(BJ)/def2-TZVPP/IEFPCM(propanonitrile)//B3LYP-D3(BJ)/6-31G(d) Free Energy (Quasiharmonic) = -2781.121817

Frequencies (Top 3 out of 213)

1. 8.6672 cm^{-1}
2. 11.0968 cm^{-1}
3. 13.6906 cm^{-1}

B3LYP/6-31G(d) Molecular Geometry in Cartesian Coordinates

B	0.506761	0.875518	-0.912656
O	-0.214442	0.853793	-2.171819
N	-0.607506	0.919449	0.138171
C	-1.925381	0.815385	-0.527137
S	-0.441609	0.485741	1.742537
C	-1.549179	0.900829	-2.012371
O	-2.329962	0.989930	-2.928239
H	-3.191588	2.509814	-1.004954
H	-2.353084	-0.185112	-0.373859
C	-2.992117	1.884469	-0.126791
C	-4.287351	1.316265	0.381424
C	-5.353306	0.729569	-0.399374
C	-6.382887	0.356652	0.509202
N	-5.950956	0.697296	1.775766
C	-4.695860	1.270807	1.692010
H	-4.171910	1.594962	2.579696
C	-7.730489	-0.464262	-1.277364
C	-5.537508	0.485230	-1.772918
H	-8.352205	-0.516002	0.794727
H	-6.472577	0.564077	2.627645
C	-6.721581	-0.107043	-2.195433
H	-8.645239	-0.925511	-1.640339
H	-4.757010	0.742599	-2.483015
C	-7.575455	-0.239210	0.085940

H	-6.874190	-0.302337	-3.253571
C	-0.491077	-1.302091	1.833980
O	-1.611331	0.995661	2.468177
O	0.910648	0.934265	2.121264
C	-0.574156	-4.099926	1.785786
C	-1.725756	-1.953740	1.762763
C	0.699453	-2.027991	1.928370
C	0.646248	-3.418162	1.905527
C	-1.754799	-3.347815	1.733413
H	-2.647838	-1.381606	1.745417
H	1.647384	-1.510400	2.017314
H	1.571411	-3.985270	1.967039
H	-2.712497	-3.857491	1.666021
C	-0.597778	-5.606099	1.694290
H	-0.033787	-6.066601	2.513890
H	-1.619290	-5.996817	1.722969
H	-0.135033	-5.937446	0.756314
H	-2.548521	2.523040	0.639734
C	1.679760	-0.219575	-0.845085
C	2.880320	-0.054325	-0.143731
C	1.478660	-1.447657	-1.496280
C	3.833638	-1.074439	-0.078114
H	3.082787	0.872211	0.381735
C	2.421918	-2.473975	-1.424740
H	0.571127	-1.606775	-2.069231
C	3.609777	-2.292968	-0.715473
H	4.349526	-3.083860	-0.667046
C	2.130416	-3.818336	-2.034283
C	5.059377	-0.884005	0.771511
F	1.258176	-3.740046	-3.058786
F	1.588677	-4.665736	-1.118723
F	3.251326	-4.417840	-2.494503
F	4.797438	-1.151977	2.074972
F	5.518172	0.387103	0.721029
F	6.072371	-1.696251	0.399124
O	1.263220	2.320846	-0.961284
C	1.317024	3.140674	-0.017476
C	2.017793	4.397314	-0.133173
C	2.656492	4.776565	-1.332006
C	2.049901	5.248157	0.988484
C	3.316842	5.994931	-1.398754
H	2.619612	4.107848	-2.185778
C	2.715256	6.467161	0.913089
H	1.557003	4.943243	1.907940
C	3.345797	6.837271	-0.278457
H	3.810851	6.296430	-2.317264
H	2.745205	7.126643	1.774716
H	3.865148	7.789803	-0.337438
H	0.831452	2.886680	0.928696

NTOB-benzaldehyde complex 57

B3LYP/6-31G(d) = -2780.344994
B3LYP-D3(BJ)/def2-TZVPP/IEFPCM(propanonitrile) = -2781.607712
B3LYP-D3(BJ)/def2-TZVPP/IEFPCM(propanonitrile)//B3LYP-D3(BJ)/6-31G(d) Free Energy (Quasiharmonic) = -2781.12179

Frequencies (Top 3 out of 213)

1. 10.8145 cm^{-1}
2. 13.0529 cm^{-1}
3. 15.8056 cm^{-1}

B3LYP/6-31G(d) Molecular Geometry in Cartesian Coordinates

B	0.359872	-1.230086	0.204808
O	0.080491	-2.631909	-0.065642
N	-0.154363	-0.990475	1.612418
C	-0.809769	-2.230153	2.090053
S	0.634063	-0.088897	2.812234
C	-0.589591	-3.222893	0.951411
O	-0.966207	-4.368527	0.926148
H	-2.413618	-1.289576	3.158445
H	-0.295117	-2.615292	2.976259
C	-2.313232	-2.125348	2.459983
C	-3.306004	-2.007760	1.332979
C	-3.908547	-0.816422	0.777342
C	-4.856837	-1.235246	-0.199609
N	-4.805411	-2.613341	-0.249696
C	-3.890294	-3.068486	0.681017
H	-3.710688	-4.126709	0.808919
C	-5.483708	1.022450	-0.644020
C	-3.757699	0.560866	1.016897
H	-6.374527	-0.673127	-1.648850
H	-5.418087	-3.202058	-0.792356
C	-4.542755	1.463344	0.310332
H	-6.088704	1.751414	-1.176346
H	-3.029017	0.914988	1.738266
C	-5.650236	-0.330688	-0.913937
H	-4.433870	2.528402	0.497652
C	0.239948	1.629128	2.463463
O	2.089947	-0.224617	2.691217
O	-0.034898	-0.446570	4.071956
C	-0.342511	4.330225	1.993866
C	1.150949	2.420776	1.764727
C	-0.942210	2.177950	2.965444
C	-1.226242	3.520175	2.723602
C	0.846731	3.759836	1.521447
H	2.090154	2.000665	1.427489
H	-1.613266	1.565860	3.558827
H	-2.144363	3.949454	3.117921
H	1.554241	4.364654	0.960193
C	-0.645904	5.791613	1.763150
H	-1.723606	5.972468	1.690716
H	-0.174339	6.158937	0.845908
H	-0.268639	6.406074	2.591272
H	-2.541718	-3.033144	3.029941
C	1.799869	-0.753065	-0.305507
C	2.044789	0.521449	-0.827855
C	2.883547	-1.644215	-0.244268
C	3.326564	0.914121	-1.226817
H	1.231543	1.233724	-0.917920
C	4.158691	-1.266060	-0.664123
H	2.730867	-2.646685	0.141331
C	4.393127	0.023358	-1.147071
H	5.388060	0.324218	-1.453168
C	5.284854	-2.265479	-0.653939
C	3.540937	2.334408	-1.665455
F	5.128607	-3.197625	0.308243
F	6.482911	-1.670026	-0.456298
F	5.369505	-2.927670	-1.833882
F	3.519320	3.188792	-0.604347
F	2.567180	2.755352	-2.507195
F	4.722311	2.515775	-2.288599
O	-0.677201	-0.376773	-0.801888
C	-1.361640	-0.922060	-1.691421
C	-2.143155	-0.176189	-2.650509
C	-2.225942	1.229339	-2.600774
C	-2.796522	-0.887911	-3.674367
C	-2.948273	1.906917	-3.573445
H	-1.727957	1.761304	-1.797691
C	-3.516317	-0.202008	-4.645964
H	-2.726624	-1.972313	-3.702333
C	-3.588877	1.193466	-4.594329
H	-3.016945	2.989967	-3.542968
H	-4.016179	-0.745871	-5.441572
H	-4.149235	1.730631	-5.354693
H	-1.342349	-2.015326	-1.763535

NTOB-benzaldehyde complex 58

B3LYP/6-31G(d) = -2780.343087
B3LYP-D3(BJ)/def2-TZVPP/IEFPCM(propanonitrile) = -2781.606527
B3LYP-D3(BJ)/def2-TZVPP/IEFPCM(propanonitrile)//B3LYP-D3(BJ)/6-31G(d) Free Energy (Quasiharmonic) = -2781.121746

Frequencies (Top 3 out of 213)

1. 9.3017 cm^{-1}
2. 11.1415 cm^{-1}
3. 15.0449 cm^{-1}

B3LYP/6-31G(d) Molecular Geometry in Cartesian Coordinates

B	0.050337	0.328245	1.042501
O	0.063014	0.390815	2.491003
N	0.819884	-0.909226	0.696257
C	1.520070	-1.384657	1.899753
S	0.502628	-1.960417	-0.580405
C	0.859101	-0.576683	3.022674
O	1.045352	-0.706176	4.205511
H	3.345212	-1.302903	2.995488
H	1.361063	-2.454207	2.059960
C	3.052347	-1.098655	1.958444
C	3.930686	-1.870392	1.020781
C	4.340296	-1.499834	-0.312645
C	5.206764	-2.523200	-0.786173
N	5.309672	-3.465121	0.216586
C	4.543883	-3.067625	1.294180
H	4.500145	-3.674984	2.188563
C	5.522260	-1.376322	-2.851678
C	4.069396	-0.405591	-1.151979
H	6.462715	-3.269510	-2.393357
H	5.830702	-4.325677	0.159051
C	4.661751	-0.352113	-2.406993
H	5.966608	-1.309661	-3.841128
H	3.381599	0.372327	-0.832813
C	5.804664	-2.475435	-2.049385
H	4.448756	0.484520	-3.067178
C	-1.206540	-2.496550	-0.393258
O	1.348026	-3.137390	-0.363199
O	0.571201	-1.172174	-1.814656
C	-3.874659	-3.296636	-0.081618
C	-1.572650	-3.270603	0.710426
C	-2.147445	-2.152619	-1.364091
C	-3.470997	-2.557582	-1.201470
C	-2.901737	-3.658335	0.861557
H	-0.829747	-3.576144	1.440549
H	-1.848026	-1.564132	-2.223479
H	-4.204961	-2.282590	-1.955119
H	-3.188935	-4.251492	1.726269
C	-5.325867	-3.659364	0.119201
H	-5.439673	-4.510528	0.797973
H	-5.812846	-3.910538	-0.829269
H	-5.871159	-2.811936	0.553738
H	3.202504	-0.022851	1.800159
C	-1.329934	0.745318	0.367258
C	-1.432454	1.149521	-0.972618
C	-2.506715	0.705827	1.128878
C	-2.668234	1.476803	-1.533490
H	-0.544634	1.180759	-1.594442
C	-3.744386	1.033522	0.568483
H	-2.456174	0.415978	2.173666
C	-3.833039	1.419209	-0.768169
H	-4.790673	1.677216	-1.204406
C	-4.990898	0.906036	1.401131
C	-2.745416	1.798300	-3.000379
F	-5.418077	-0.381221	1.468199
F	-6.015400	1.631237	0.900973
F	-4.789464	1.310393	2.674191
F	-2.745025	0.667694	-3.750777
F	-1.692777	2.538681	-3.412210
F	-3.869179	2.478837	-3.317688
O	1.090588	1.645399	0.565682
C	1.400282	2.564950	1.346764
C	2.224622	3.689024	0.957404
C	2.715728	3.816646	-0.357733
C	2.530793	4.663878	1.925370
C	3.504255	4.909195	-0.690866
H	2.468243	3.057432	-1.092408
C	3.321980	5.755669	1.583777
H	2.147416	4.556427	2.936971
C	3.806617	5.875605	0.277992
H	3.887289	5.014774	-1.701145
H	3.561897	6.509633	2.326981
H	4.425037	6.728023	0.010809
H	1.033744	2.517730	2.380537

NTOB-benzaldehyde complex 59

B3LYP/6-31G(d) = -2780.351259
B3LYP-D3(BJ)/def2-TZVPP/IEFPCM(propanonitrile) = -2781.606768
B3LYP-D3(BJ)/def2-TZVPP/IEFPCM(propanonitrile)//B3LYP-D3(BJ)/6-31G(d) Free Energy (Quasiharmonic) = -2781.121659

Frequencies (Top 3 out of 213)

1. 9.4808 cm^{-1}
2. 12.1950 cm^{-1}
3. 12.8893 cm^{-1}

B3LYP/6-31G(d) Molecular Geometry in Cartesian Coordinates

B	-0.546458	0.844985	0.572276
O	-0.024358	1.023829	1.917606
N	0.680706	0.533692	-0.250424
C	1.889009	0.549149	0.591974
S	0.702401	-0.209338	-1.746771
C	1.325203	0.893810	1.974906
O	1.957245	1.076096	2.985182
H	2.709529	2.566684	0.553579
H	2.343651	-0.449224	0.643541
C	2.987110	1.575305	0.174639
C	4.364749	1.193338	0.637366
C	5.343652	0.460036	-0.131565
C	6.489223	0.291865	0.694987
N	6.207904	0.896444	1.903634
C	4.933235	1.427862	1.865084
H	4.509934	1.912402	2.732842
C	7.626220	-0.890857	-1.035167
C	5.364204	-0.067320	-1.436445
H	8.503291	-0.495756	0.902835
H	6.823393	0.924589	2.701153
C	6.501993	-0.736729	-1.872370
H	8.501101	-1.418252	-1.405980
H	4.503068	0.046216	-2.089588
C	7.636256	-0.378194	0.257353
H	6.529115	-1.147612	-2.878133
C	0.741503	-1.975136	-1.422437
O	1.975160	0.131352	-2.395550
O	-0.575654	0.121240	-2.383750
C	0.797101	-4.701142	-0.777674
C	1.969689	-2.616896	-1.245145
C	-0.458570	-2.682480	-1.304695
C	-0.421732	-4.034652	-0.977667
C	1.985430	-3.974435	-0.923380
H	2.898023	-2.069693	-1.372713
H	-1.402979	-2.178587	-1.474366
H	-1.355999	-4.578430	-0.864349
H	2.940401	-4.474896	-0.784083
C	0.817955	-6.171410	-0.434206

H	1.806873	-6.489329	-0.090094
H	0.557390	-6.784508	-1.306695
H	0.091811	-6.405387	0.352478
H	2.968453	1.633234	-0.915154
C	-1.885710	-0.028066	0.513182
C	-2.921182	0.192331	-0.407222
C	-2.035652	-1.092676	1.414418
C	-4.060124	-0.616072	-0.424889
H	-2.835834	0.995794	-1.129029
C	-3.161979	-1.919070	1.383632
H	-1.264285	-1.282523	2.153960
C	-4.185023	-1.681913	0.466510
H	-5.069084	-2.308941	0.453761
C	-3.238540	-3.119485	2.286577
C	-5.130681	-0.378180	-1.455997
F	-2.589536	-2.922578	3.453517
F	-2.675356	-4.210329	1.704847
F	-4.514845	-3.453705	2.580722
F	-6.343293	-0.800508	-1.029968
F	-4.870339	-1.033914	-2.609749
F	-5.246687	0.933070	-1.767287
O	-1.027682	2.366652	0.076339
C	-1.067593	3.358203	0.831985
C	-1.451285	4.671974	0.369932
C	-1.771906	4.903870	-0.983754
C	-1.498266	5.726572	1.301486
C	-2.137107	6.179488	-1.390236
H	-1.722569	4.080339	-1.688854
C	-1.867186	7.001316	0.885747
H	-1.246903	5.537291	2.342051
C	-2.185507	7.224454	-0.457349
H	-2.384093	6.368184	-2.430362
H	-1.906745	7.818207	1.599478
H	-2.472485	8.220649	-0.782443
H	-0.795571	3.222219	1.885816

NTOB-benzaldehyde complex 60

B3LYP/6-31G(d) = -2780.348437
B3LYP-D3(BJ)/def2-TZVPP/IEFPCM(propanonitrile) = -2781.606428
B3LYP-D3(BJ)/def2-TZVPP/IEFPCM(propanonitrile)//B3LYP-D3(BJ)/6-31G(d) Free Energy (Quasiharmonic) = -2781.121433

Frequencies (Top 3 out of 213)

1. 7.1265 cm^{-1}
2. 10.0545 cm^{-1}
3. 11.5826 cm^{-1}

B3LYP/6-31G(d) Molecular Geometry in Cartesian Coordinates

B	-0.848911	-0.019433	0.892223
O	-0.491918	0.318768	2.259119
N	0.453316	0.199955	0.125040
C	1.457466	0.823836	1.001504
S	0.816941	-0.335584	-1.396196
C	0.773449	0.757706	2.373926
O	1.285116	1.089685	3.415708
H	1.743706	2.894963	1.564823
H	2.366342	0.213318	1.058622
C	1.846305	2.300011	0.649103
C	3.237636	2.469849	0.116110
C	4.462253	2.304179	0.863728
C	5.539158	2.572235	-0.025875
N	4.984402	2.878260	-1.253736
C	3.604160	2.823067	-1.158417
H	2.980743	3.017337	-2.019715
C	7.126526	2.162993	1.705152
C	4.745982	1.959438	2.197883
H	7.689122	2.718292	-0.309221
H	5.497279	3.162263	-2.073610
C	6.073615	1.892319	2.603467
H	8.154189	2.105195	2.054036
H	3.937846	1.750121	2.894722
C	6.875953	2.506167	0.380669
H	6.307604	1.629594	3.631584
C	2.214175	-1.450549	-1.211922
O	1.262659	0.779471	-2.239763
O	-0.349899	-1.140058	-1.817510
C	4.385577	-3.194387	-0.893234
C	3.517081	-0.950876	-1.278996
C	1.982808	-2.811443	-0.999787
C	3.067555	-3.670943	-0.844271
C	4.589572	-1.825196	-1.110934
H	3.686032	0.104418	-1.460847
H	0.968305	-3.192935	-0.976926
H	2.888121	-4.731609	-0.685126
H	5.602427	-1.432436	-1.149190
C	5.556968	-4.135685	-0.745840
H	5.926663	-4.460069	-1.727624
H	5.281336	-5.035719	-0.186952
H	6.392943	-3.654843	-0.227314
O	-1.091426	-1.626166	0.969004
C	-1.806851	-2.242245	0.145911
H	-2.410043	-1.674971	-0.565416
H	1.114086	2.678436	-0.069344
C	-1.888450	-3.685191	0.132452
C	-1.170715	-4.468563	1.059474
C	-2.715722	-4.301320	-0.824979
C	-1.287051	-5.851289	1.022315
H	-0.541047	-3.975639	1.792999
C	-2.824653	-5.687735	-0.857076
H	-3.260235	-3.688279	-1.538166
C	-2.112226	-6.458749	0.065939
H	-0.741572	-6.462136	1.735188
H	-3.459882	-6.167671	-1.594924
H	-2.199558	-7.541497	0.042499
C	-2.211574	0.681911	0.411423
C	-2.328481	1.400232	-0.787081
C	-3.338709	0.634392	1.250036
C	-3.523163	2.038959	-1.136173
H	-1.481382	1.471943	-1.461451
C	-4.535447	1.262225	0.900985
H	-3.276701	0.115193	2.202540
C	-4.633962	1.970108	-0.297598
H	-5.559709	2.463132	-0.570046
C	-5.743081	1.121434	1.788108
C	-3.589586	2.858350	-2.398325
F	-5.405578	1.045647	3.093195
F	-6.600531	2.155514	1.646735
F	-2.854905	2.312834	-3.390381
F	-6.439452	-0.007043	1.499465
F	-3.118390	4.111990	-2.203148
F	-4.858123	2.981898	-2.852621

NTOB-benzaldehyde complex 61

B3LYP/6-31G(d) = -2780.351257
B3LYP-D3(BJ)/def2-TZVPP/IEFPCM(propanonitrile) = -2781.606567
B3LYP-D3(BJ)/def2-TZVPP/IEFPCM(propanonitrile)//B3LYP-D3(BJ)/6-31G(d) Free Energy (Quasiharmonic) = -2781.121429

Frequencies (Top 3 out of 213)

1. 9.8024 cm^{-1}
2. 12.3271 cm^{-1}
3. 13.9027 cm^{-1}

B3LYP/6-31G(d) Molecular Geometry in Cartesian Coordinates

B	0.563519	0.815024	-0.561393
O	0.055217	0.994845	-1.911971
N	-0.673886	0.521436	0.251357
C	-1.875720	0.557004	-0.599438
S	-0.720884	-0.217555	1.748880
C	-1.295777	0.886510	-1.979327
O	-1.917097	1.074968	-2.995147
H	-2.660560	2.589081	-0.576003
H	-2.348639	-0.432856	-0.650883
C	-2.957320	1.604885	-0.192841
C	-4.339756	1.244908	-0.659077
C	-5.338385	0.543373	0.114071
C	-6.482259	0.387221	-0.717173
N	-6.181012	0.968113	-1.932595
C	-4.895747	1.473387	-1.893686
H	-4.457008	1.936315	-2.765571
C	-7.654156	-0.746341	1.022606
C	-5.377755	0.035342	1.426168
H	-8.511145	-0.361082	-0.926310
H	-6.791019	0.997641	-2.734271
C	-6.531909	-0.603763	1.864524
H	-8.542115	-1.249902	1.395455
H	-4.518176	0.139877	2.082866
C	-7.645703	-0.252328	-0.277160
H	-6.573609	-0.999523	2.875838
C	-0.809357	-1.981697	1.425259
O	-1.985345	0.159204	2.394009
O	0.564066	0.077663	2.389010
C	-0.940875	-4.706300	0.785414
C	-2.055244	-2.592570	1.263476
C	0.371009	-2.718773	1.292275
C	0.296749	-4.069976	0.967583
C	-2.108781	-3.949772	0.944344
H	-2.967740	-2.021479	1.400048
H	1.329749	-2.237890	1.447429
H	1.215894	-4.636084	0.841456
H	-3.077630	-4.426261	0.817261
C	-1.003600	-6.176786	0.448012
H	-0.777458	-6.793381	1.327716
H	-0.272955	-6.438494	-0.325482
H	-1.996810	-6.464491	0.089833
H	-2.942345	1.668754	0.896672
C	1.894582	-0.069528	-0.489215
C	2.033952	-1.148168	-1.375378
C	2.932820	0.154240	0.427346
C	3.150592	-1.987077	-1.330575
H	1.259771	-1.342308	-2.110865
C	4.064648	-0.663915	0.455204
H	2.849891	0.960259	1.146694
C	4.177243	-1.745601	-0.418624
H	5.048699	-2.389360	-0.387457
C	5.198261	-0.343077	1.392022
C	3.209934	-3.206982	-2.208243
F	6.073113	0.528276	0.830489
F	4.765209	0.224989	2.537101
F	5.906543	-1.445323	1.725336
F	4.481203	-3.563543	-2.497653
F	2.634160	-4.279001	-1.603640
F	2.561089	-3.025686	-3.377723
O	1.056165	2.336003	-0.067407
C	1.115340	3.322584	-0.828244
C	1.506649	4.635321	-0.369416
C	1.576242	5.683462	-1.306803
C	1.812483	4.872553	0.986756
C	1.953017	6.956986	-0.894340
H	1.336162	5.490156	-2.349287
C	2.185624	6.146918	1.389941
H	1.745658	4.054101	1.696334
C	2.256606	7.185395	0.451262
H	2.010055	7.768863	-1.612591
H	2.421316	6.339697	2.431939
H	2.549719	8.180640	0.773777
H	0.854784	3.183164	-1.884533

NTOB-benzaldehyde complex 62

B3LYP/6-31G(d) = -2780.348556
B3LYP-D3(BJ)/def2-TZVPP/IEFPCM(propanonitrile) = -2781.606483
B3LYP-D3(BJ)/def2-TZVPP/IEFPCM(propanonitrile)//B3LYP-D3(BJ)/6-31G(d) Free Energy (Quasiharmonic) = -2781.121374

Frequencies (Top 3 out of 213)

1. 7.5292 cm^{-1}
2. 10.6553 cm^{-1}
3. 12.7498 cm^{-1}

B3LYP/6-31G(d) Molecular Geometry in Cartesian Coordinates

B	-0.847255	0.011926	-0.896046
O	-0.489694	-0.343100	-2.258560
N	0.452532	-0.205708	-0.124220
C	1.455254	-0.843590	-0.992219
S	0.816491	0.344834	1.391621
C	0.773700	-0.789374	-2.366405
O	1.285419	-1.135367	-3.403601
H	1.732177	-2.922286	-1.531919
H	2.366922	-0.237761	-1.054536
C	1.836858	-2.317515	-0.622888
C	3.226935	-2.487895	-0.086773
C	4.453016	-2.336347	-0.834984
C	5.527816	-2.599408	0.058657
N	4.970464	-2.889083	1.289308
C	3.590586	-2.828466	1.192020
H	2.965398	-3.010050	2.054809
C	7.118710	-2.216813	-1.675228
C	4.739604	-2.007844	-2.172618
H	7.676816	-2.752152	0.345786
H	5.481251	-3.165895	2.112937
C	6.067922	-1.951354	-2.577577
H	8.146964	-2.167626	-2.023692
H	3.933102	-1.802562	-2.872543
C	6.865294	-2.544029	-0.347255
H	6.304110	-1.701192	-3.608321
C	2.218647	1.451831	1.197044
O	1.256330	-0.762769	2.247977
O	-0.347445	1.158747	1.802792
C	4.397760	3.183006	0.862320
C	3.519345	0.947117	1.269317
C	1.993311	2.811640	0.971892
C	3.081865	3.664852	0.808480
C	4.595693	1.815070	1.093163
H	3.683657	-0.107106	1.461381
H	0.980503	3.197349	0.945157
H	2.907119	4.724728	0.639191
H	5.606805	1.418262	1.135511
C	5.573320	4.117680	0.706211
H	5.944599	4.449372	1.684950
H	5.301631	5.013783	0.139138
H	6.407070	3.628382	0.192026
O	-1.082063	1.618788	-0.990987
C	-1.794585	2.247588	-0.175003
H	-2.400200	1.691366	0.542931
H	1.102342	-2.684261	0.099262
C	-1.869259	3.690983	-0.177924
C	-1.149562	4.460305	-1.115119
C	-2.691623	4.321894	0.774112
C	-1.259211	5.843912	-1.093437
H	-0.523761	3.956056	-1.844242
C	-2.793728	5.709104	0.790768
H	-3.237370	3.719592	1.495476

C	-2.079500	6.466147	-0.142373
H	-0.712259	6.444000	-1.814272
H	-3.424981	6.200454	1.524509
H	-2.161544	7.549504	-0.131024
C	-2.213804	-0.676288	-0.407805
C	-2.333473	-1.379988	0.799069
C	-3.341734	-0.631066	-1.245271
C	-3.533026	-2.002672	1.159348
H	-1.481665	-1.461872	1.466206
C	-4.541076	-1.250328	-0.889198
H	-3.281539	-0.112078	-2.197982
C	-4.643637	-1.939494	0.319819
H	-5.572508	-2.422413	0.599847
C	-5.708940	-1.226711	-1.838807
C	-3.645149	-2.693807	2.492771
F	-5.648575	-2.235387	-2.736721
F	-6.889067	-1.347732	-1.189202
F	-3.975870	-1.823616	3.476724
F	-5.754325	-0.074106	-2.546555
F	-2.483504	-3.275746	2.857247
F	-4.597275	-3.654329	2.484338

NTOB-benzaldehyde complex 63

B3LYP/6-31G(d) = -2780.348573
B3LYP-D3(BJ)/def2-TZVPP/IEFPCM(propanonitrile) = -2781.606477
B3LYP-D3(BJ)/def2-TZVPP/IEFPCM(propanonitrile)//B3LYP-D3(BJ)/6-31G(d) Free Energy (Quasiharmonic) = -2781.12135

Frequencies (Top 3 out of 213)

1. 7.5342 cm^{-1}
2. 10.7116 cm^{-1}
3. 12.8320 cm^{-1}

B3LYP/6-31G(d) Molecular Geometry in Cartesian Coordinates

B	-0.842653	0.003974	-0.904328
O	-0.480227	-0.335375	-2.269467
N	0.457883	-0.209261	-0.132678
C	1.467486	-0.830061	-1.005010
S	0.813423	0.328547	1.389676
C	0.787403	-0.768926	-2.379664
O	1.303736	-1.100392	-3.419318
H	1.764909	-2.900455	-1.564854
H	2.373933	-0.215758	-1.060185
C	1.861432	-2.304108	-0.649374
C	3.251459	-2.467438	-0.110974
C	4.478157	-2.297572	-0.854218
C	5.552838	-2.560078	0.039725
N	4.994761	-2.867037	1.265847
C	3.614667	-2.817640	1.165338
H	2.988809	-3.013398	2.024527
C	7.144947	-2.146204	-1.685843
C	4.765436	-1.953241	-2.187717
H	7.702322	-2.696756	0.331280
H	5.505767	-3.147490	2.088108
C	6.094283	-1.881030	-2.588420
H	8.173659	-2.084482	-2.030943
H	3.959040	-1.748152	-2.887823
C	6.890856	-2.488872	-0.361899
H	6.331018	-1.618536	-3.615967
C	2.206473	1.449460	1.209907
O	1.260700	-0.784101	2.235617
O	-0.358477	1.128102	1.806236
C	4.371349	3.202654	0.898147
C	3.511306	0.955424	1.281205
C	1.969927	2.809351	0.997054
C	3.051450	3.673524	0.844992
C	4.580544	1.834356	1.116529
H	3.684274	-0.099106	1.463689
H	0.953871	3.186474	0.971003
H	2.867940	4.733407	0.685294
H	5.594953	1.445962	1.158053
C	5.539137	4.148992	0.754475
H	5.904263	4.475006	1.737428
H	5.261422	5.047809	0.194660
H	6.378846	3.671745	0.238666
O	-1.091382	1.609740	-0.984676
C	-1.811285	2.224587	-0.164473
H	-2.413756	1.656381	0.546690
H	1.128188	-2.684907	0.066777
C	-1.898657	3.667201	-0.153962
C	-1.181988	4.451780	-1.080797
C	-2.730527	4.281679	0.800572
C	-1.303959	5.834083	-1.046374
H	-0.548698	3.960071	-1.812018
C	-2.845062	5.667710	0.829934
H	-3.274145	3.667709	1.513647
C	-2.133691	6.439915	-0.092896
H	-0.759351	6.445860	-1.759116
H	-3.483835	6.146402	1.565526
H	-2.225411	7.522346	-0.071588
C	-2.203981	-0.699795	-0.423787
C	-3.332983	-0.653023	-1.259676
C	-2.315482	-1.423688	0.771897
C	-4.525577	-1.290023	-0.912385
H	-3.274275	-0.131826	-2.211349
C	-3.507041	-2.067427	1.121459
H	-1.461400	-1.509369	1.435692
C	-4.619397	-2.001193	0.284486
H	-5.538255	-2.510577	0.550157
C	-3.605682	-2.792921	2.437696
C	-5.731335	-1.161487	-1.804059
F	-2.437650	-3.375729	2.780097
F	-3.935581	-1.949976	3.445213
F	-5.390509	-1.093708	-3.108790
F	-4.551134	-3.759591	2.411176
F	-6.433267	-0.033894	-1.525831
F	-6.584516	-2.198282	-1.657551

NTOB-benzaldehyde complex 64

B3LYP/6-31G(d) = -2780.346538
B3LYP-D3(BJ)/def2-TZVPP/IEFPCM(propanonitrile) = -2781.607044
B3LYP-D3(BJ)/def2-TZVPP/IEFPCM(propanonitrile)//B3LYP-D3(BJ)/6-31G(d) Free Energy (Quasiharmonic) = -2781.121258

Frequencies (Top 3 out of 213)

1. 9.2368 cm^{-1}
2. 12.0604 cm^{-1}
3. 14.2540 cm^{-1}

B3LYP/6-31G(d) Molecular Geometry in Cartesian Coordinates

B	0.324144	-0.056515	0.216105
O	0.940492	0.230645	1.501094
N	0.307865	-1.568592	0.166071
C	1.033505	-2.129963	1.312439
S	-0.529239	-2.537644	-0.894405
C	1.387055	-0.880405	2.123320
O	1.982749	-0.869503	3.172394
H	1.961714	-3.966046	0.686432
H	0.368135	-2.740058	1.940040
C	2.301448	-2.983054	1.016701
C	3.256377	-2.426151	-0.000386
C	4.360640	-1.514783	0.199689
C	4.980962	-1.320005	-1.070043
N	4.264080	-2.059085	-1.989564

C	3.244762	-2.728987	-1.340034
H	2.563571	-3.369985	-1.881744
C	6.630749	0.109924	-0.106453
C	4.904400	-0.859398	1.321341
H	6.584486	-0.397962	-2.211062
H	4.506308	-2.180260	-2.960791
C	6.028285	-0.058430	1.157921
H	7.517906	0.731096	-0.198817
H	4.443246	-0.970303	2.298292
C	6.114195	-0.515565	-1.237527
H	6.454465	0.446564	2.020386
C	-2.139668	-2.842810	-0.157011
O	0.157207	-3.837268	-0.945631
O	-0.740098	-1.753105	-2.115016
C	-4.635693	-3.302791	1.032123
C	-2.278581	-3.861868	0.788818
C	-3.236127	-2.062635	-0.531597
C	-4.471826	-2.299031	0.066847
C	-3.521668	-4.078761	1.380130
H	-1.431242	-4.492054	1.037605
H	-3.126794	-1.295676	-1.290060
H	-5.326491	-1.693792	-0.225641
H	-3.629670	-4.870642	2.117339
C	-5.987683	-3.563820	1.652204
H	-6.550705	-2.634702	1.790251
H	-5.894398	-4.053595	2.626694
H	-6.594058	-4.218908	1.012700
H	2.801405	-3.114809	1.983972
C	-0.976887	0.828222	-0.079666
C	-1.459816	1.096236	-1.369980
C	-1.696540	1.356915	1.003682
C	-2.627961	1.836918	-1.564401
H	-0.938254	0.694766	-2.230923
C	-2.854311	2.116000	0.810129
H	-1.349079	1.172704	2.015646
C	-3.331895	2.355157	-0.477300
H	-4.236515	2.931332	-0.631582
C	-3.553805	2.724275	1.995623
C	-3.176043	1.988360	-2.956860
F	-3.495685	1.921964	3.080953
F	-4.858335	2.972269	1.742619
F	-2.992130	3.905021	2.353142
F	-3.863752	0.881771	-3.332944
F	-2.196492	2.168602	-3.869245
F	-4.026072	3.034171	-3.061846
O	1.445064	0.367811	-0.945977
C	2.428542	1.131691	-1.028670
C	2.780107	2.297679	-0.244668
C	1.907774	2.944018	0.656652
C	4.068719	2.827944	-0.470325
C	2.329015	4.090534	1.316469
H	0.912158	2.557467	0.822913
C	4.488099	3.965023	0.209158
H	4.740757	2.322006	-1.157481
C	3.616203	4.596921	1.099833
H	1.655987	4.593162	2.004141
H	5.485617	4.360787	0.045285
H	3.937763	5.491613	1.626039
H	3.122054	0.888948	-1.843205

NTOB-benzaldehyde complex 65

B3LYP/6-31G(d) = -2780.35374
B3LYP-D3(BJ)/def2-TZVPP/IEFPCM(propanonitrile) = -2781.606603
B3LYP-D3(BJ)/def2-TZVPP/IEFPCM(propanonitrile)//B3LYP-D3(BJ)/6-31G(d) Free Energy (Quasiharmonic) = -2781.121241

Frequencies (Top 3 out of 213)

1. 8.8403 cm^{-1}
2. 10.1346 cm^{-1}
3. 14.3499 cm^{-1}

B3LYP/6-31G(d) Molecular Geometry in Cartesian Coordinates

B	-0.418292	0.810625	0.870213
O	0.149049	0.720198	2.199531
N	0.780071	0.569638	-0.047474
C	1.999689	0.355682	0.763536
S	0.752912	0.073031	-1.638968
C	1.474919	0.474912	2.199365
O	2.135648	0.394830	3.204926
H	2.915488	2.310094	1.034066
H	2.389147	-0.659909	0.623488
C	3.152288	1.374291	0.512936
C	4.498546	0.857523	0.933342
C	5.415463	0.104280	0.109303
C	6.546249	-0.207536	0.914632
N	6.316055	0.335753	2.162628
C	5.086424	0.964295	2.169125
H	4.703867	1.419029	3.071296
C	7.584010	-1.357067	-0.898425
C	5.392908	-0.334278	-1.227898
H	8.492658	-1.161689	1.055437
H	6.930524	0.257470	2.957605
C	6.474100	-1.059695	-1.715537
H	8.414251	-1.925238	-1.309459
H	4.543318	-0.108535	-1.866705
C	7.636365	-0.934013	0.425282
H	6.467427	-1.402806	-2.746800
C	0.493676	-1.700757	-1.689252
O	2.086443	0.318605	-2.203494
O	-0.426109	0.728030	-2.233205
C	0.117118	-4.475936	-1.738977
C	-0.779835	-2.218028	-1.934844
C	1.587751	-2.550422	-1.492174
C	1.387574	-3.928490	-1.509428
C	-0.955332	-3.600276	-1.956321
H	-1.616280	-1.555179	-2.120530
H	2.583861	-2.142983	-1.353446
H	2.236179	-4.589405	-1.351484
H	-1.946283	-4.003677	-2.148973
C	-0.081992	-5.971760	-1.789173
H	-1.100028	-6.250263	-1.498820
H	0.616725	-6.491318	-1.125435
H	0.085365	-6.356153	-2.804118
H	3.161342	1.592402	-0.556949
C	-1.811195	0.019377	0.744238
C	-2.123100	-0.938642	1.722898
C	-2.750435	0.222251	-0.276112
C	-3.307886	-1.676650	1.671234
H	-1.429238	-1.108449	2.539125
C	-3.928237	-0.526717	-0.341441
H	-2.555903	0.947264	-1.058380
C	-4.216942	-1.481342	0.631887
H	-5.130248	-2.062250	0.580673
C	-4.830928	-0.358422	-1.530995
C	-3.634882	-2.649685	2.772667
F	-4.953502	0.937774	-1.894723
F	-6.073456	-0.837616	-1.309007
F	-4.340727	-1.019147	-2.612772
F	-4.435200	-3.650645	2.337651
F	-4.284708	-2.046513	3.795607
F	-2.524575	-3.215727	3.292237
O	-0.846588	2.402012	0.773786
C	-0.684226	3.128402	-0.230899
C	-1.115302	4.506886	-0.261291
C	-0.904596	5.245428	-1.441327
C	-1.731327	5.110112	0.854396
C	-1.306072	6.575672	-1.505978
H	-0.431506	4.768657	-2.295885

C	-2.128382	6.437639	0.781492
H	-1.885093	4.524192	1.754724
C	-1.915830	7.167836	-0.396210
H	-1.147427	7.149856	-2.413398
H	-2.603593	6.911092	1.635150
H	-2.229522	8.206873	-0.446738
H	-0.214596	2.699809	-1.121694

NTOB-benzaldehyde complex 66

B3LYP/6-31G(d) = -2780.342223
B3LYP-D3(BJ)/def2-TZVPP/IEFPCM(propanonitrile) = -2781.606693
B3LYP-D3(BJ)/def2-TZVPP/IEFPCM(propanonitrile)//B3LYP-D3(BJ)/6-31G(d) Free Energy (Quasiharmonic) = -2781.120894

Frequencies (Top 3 out of 213)

1. 8.2400 cm^{-1}
2. 9.7758 cm^{-1}
3. 14.0231 cm^{-1}

B3LYP/6-31G(d) Molecular Geometry in Cartesian Coordinates

B	-0.915268	0.017521	0.896588
O	-0.734936	0.104021	2.339214
N	0.205405	-0.930922	0.468328
C	0.738300	-1.627416	1.653704
S	0.318948	-1.568246	-1.058183
C	0.088433	-0.852502	2.812002
O	0.292700	-1.056079	3.981609
H	2.681615	-2.335565	1.040145
H	0.356988	-2.657542	1.688782
C	2.268007	-1.702655	1.833133
C	3.043394	-0.412738	1.896705
C	4.474889	-0.316667	1.718005
C	4.842396	1.041880	1.918013
N	3.681087	1.739048	2.182929
C	2.611925	0.855480	2.196405
H	1.620008	1.221397	2.416343
C	7.140072	0.534023	1.529166
C	5.481116	-1.249621	1.413504
H	6.428853	2.524411	1.988958
H	3.640474	2.688157	2.520444
C	6.800121	-0.818945	1.322705
H	8.180074	0.841033	1.459035
H	5.234156	-2.296225	1.256615
C	6.166912	1.481695	1.826921
H	7.585839	-1.536439	1.101110
C	2.050120	-1.501772	-1.518920
O	-0.405881	-0.606621	-1.915311
O	-0.066598	-2.987951	-1.067221
C	4.713215	-1.403919	-2.374849
C	2.687499	-2.676024	-1.917622
C	2.716960	-0.274338	-1.559793
C	4.039814	-0.235270	-1.984226
C	4.015743	-2.617112	-2.341941
H	2.145240	-3.614913	-1.894257
H	2.212618	0.633530	-1.248001
H	4.567345	0.714957	-1.999366
H	4.516409	-3.531491	-2.650145
C	6.163661	-1.345451	-2.786993
H	6.344128	-0.537776	-3.505676
H	6.491437	-2.284952	-3.242244
H	6.800496	-1.152412	-1.914353
H	2.412470	-2.259952	2.769292
C	-2.468874	-0.199681	0.528453
C	-3.395413	0.791258	0.895189
C	-2.981680	-1.379270	-0.032527
C	-4.767110	0.628012	0.690872
H	-3.046637	1.710713	1.358019
C	-4.354845	-1.550167	-0.232135
H	-2.314293	-2.187011	-0.314193
C	-5.255881	-0.547498	0.122465
H	-6.319298	-0.681329	-0.037009
C	-4.862785	-2.803620	-0.896052
C	-5.721278	1.700697	1.141608
F	-4.844612	-2.693794	-2.245186
F	-6.140794	-3.075605	-0.543588
F	-4.115722	-3.880377	-0.577497
F	-5.194579	2.938209	0.974770
F	-6.030820	1.584112	2.452357
F	-6.885651	1.663729	0.456059
O	-0.452630	1.476160	0.336952
C	-0.830495	1.911765	-0.775760
C	-0.258867	3.097165	-1.371303
C	-0.780699	3.543723	-2.599872
C	0.794568	3.799604	-0.751044
C	-0.260357	4.685666	-3.200016
H	-1.586291	2.989437	-3.073850
C	1.309948	4.936373	-1.357692
H	1.192317	3.433737	0.189882
C	0.781579	5.379263	-2.578192
H	-0.659996	5.034167	-4.147122
H	2.122330	5.483366	-0.888952
H	1.189008	6.270566	-3.047269
H	-1.621513	1.383254	-1.310643

NTOB-benzaldehyde complex 67

B3LYP/6-31G(d) = -2780.346746
B3LYP-D3(BJ)/def2-TZVPP/IEFPCM(propanonitrile) = -2781.606151
B3LYP-D3(BJ)/def2-TZVPP/IEFPCM(propanonitrile)//B3LYP-D3(BJ)/6-31G(d) Free Energy (Quasiharmonic) = -2781.120884

Frequencies (Top 3 out of 213)

1. 7.1894 cm^{-1}
2. 11.0359 cm^{-1}
3. 14.4130 cm^{-1}

B3LYP/6-31G(d) Molecular Geometry in Cartesian Coordinates

B	0.919783	0.453059	1.044292
O	0.749746	0.414229	2.487561
N	-0.488288	0.134854	0.512750
C	-1.316659	-0.355982	1.630543
S	-1.101685	0.523138	-0.977048
C	-0.503587	0.089029	2.856132
O	-0.896035	0.075628	3.996174
H	-1.868866	-2.073721	2.762033
H	-2.296783	0.130769	1.647407
C	-1.489791	-1.902604	1.745714
C	-2.385241	-2.560247	0.743148
C	-3.824839	-2.483539	0.673110
C	-4.232476	-3.257205	-0.450989
N	-3.090236	-3.789612	-1.011361
C	-1.989828	-3.362641	-0.297216
H	-0.993252	-3.654056	-0.600372
C	-6.525541	-2.744034	-0.036640
C	-4.811677	-1.849372	1.449330
H	-5.867331	-3.987387	-1.680098
H	-3.052960	-4.336045	-1.857425
C	-6.147697	-1.982765	1.088548
H	-7.577519	-2.831362	-0.294777
H	-4.536073	-1.265616	2.324295
C	-5.575217	-3.393476	-0.817674
H	-6.916114	-1.498804	1.685609
C	-2.553588	1.524587	-0.642917
O	-1.553622	-0.658194	-1.716133
O	-0.078972	1.384999	-1.617236

C	-4.820763	3.086175	-0.132571
C	-2.399079	2.851313	-0.229923
C	-3.822170	0.970603	-0.811660
C	-4.945593	1.756689	-0.552135
C	-3.530689	3.620815	0.018778
H	-1.407261	3.277075	-0.118760
H	-3.924856	-0.057237	-1.139677
H	-5.933366	1.321013	-0.676140
H	-3.414253	4.655459	0.332771
C	-6.039379	3.924104	0.172169
H	-6.219911	3.975913	1.253936
H	-6.938587	3.506713	-0.291014
H	-5.918108	4.952841	-0.184656
O	1.237179	1.998072	0.777006
C	1.772761	2.400282	-0.284828
H	2.214223	1.674672	-0.969131
H	-0.491901	-2.355057	1.704469
C	1.891235	3.807203	-0.591506
C	1.436265	4.793179	0.307056
C	2.484088	4.180541	-1.811323
C	1.576376	6.135138	-0.019870
H	0.991702	4.486908	1.248528
C	2.616267	5.527221	-2.133838
H	2.824895	3.412017	-2.499771
C	2.163835	6.500620	-1.238519
H	1.233682	6.901300	0.668946
H	3.068412	5.820026	-3.076264
H	2.270001	7.552458	-1.489274
C	2.134968	-0.456211	0.512046
C	3.427874	-0.329292	1.049586
C	1.927227	-1.471117	-0.430826
C	4.465336	-1.176723	0.657958
H	3.624726	0.426242	1.805476
C	2.959278	-2.334647	-0.813268
H	0.939521	-1.606594	-0.861354
C	4.235178	-2.190954	-0.275361
H	5.033811	-2.863898	-0.563476
C	2.660969	-3.414336	-1.818187
C	5.853018	-0.967290	1.202600
F	3.681775	-4.286379	-1.956171
F	1.565527	-4.133890	-1.460878
F	5.834389	-0.480728	2.461973
F	2.400983	-2.902330	-3.041419
F	6.549684	-0.078401	0.451036
F	6.570123	-2.112056	1.217294

NTOB-benzaldehyde complex 68

B3LYP/6-31G(d) = -2780.34473
B3LYP-D3(BJ)/def2-TZVPP/IEFPCM(propanonitrile) = -2781.605329
B3LYP-D3(BJ)/def2-TZVPP/IEFPCM(propanonitrile)//B3LYP-D3(BJ)/6-31G(d) Free Energy (Quasiharmonic) = -2781.120845

Frequencies (Top 3 out of 213)

1. 7.6926 cm^{-1}
2. 12.8355 cm^{-1}
3. 13.4414 cm^{-1}

B3LYP/6-31G(d) Molecular Geometry in Cartesian Coordinates

B	-0.049848	0.538863	0.848468
O	0.178380	0.608071	2.275152
N	1.017111	-0.364199	0.322110
C	2.041760	-0.542818	1.363546
S	0.901342	-1.423964	-0.979454
C	1.343350	-0.011433	2.619137
O	1.762605	-0.052728	3.746848
H	3.880867	0.271082	2.107134
H	2.309382	-1.595005	1.490115
C	3.344582	0.291082	1.150324
C	4.238345	-0.179061	0.046615
C	5.281666	-1.169864	0.147238
C	5.835378	-1.330119	-1.152625
N	5.163518	-0.462825	-1.989862
C	4.200373	0.212716	-1.267865
H	3.536535	0.911265	-1.758536
C	7.377107	-2.961692	-0.348921
C	5.808455	-1.930290	1.205381
H	7.291715	-2.330070	-2.415990
H	5.270482	-0.419081	-2.991023
C	6.847123	-2.816955	0.949475
H	8.187607	-3.665082	-0.519844
H	5.408615	-1.829797	2.211718
C	6.881927	-2.219402	-1.415145
H	7.258711	-3.411385	1.760758
C	-0.532969	-2.461274	-0.661318
O	2.080584	-2.290983	-0.919621
O	0.602341	-0.620791	-2.172410
C	-2.772194	-4.062257	-0.141333
C	-0.521991	-3.330671	0.432221
C	-1.629374	-2.411401	-1.521217
C	-2.738060	-3.213475	-1.255220
C	-1.641138	-4.118461	0.687019
H	0.351372	-3.398351	1.073469
H	-1.619800	-1.742576	-2.373777
H	-3.596439	-3.166126	-1.920906
H	-1.637946	-4.787704	1.543784
C	-4.009971	-4.863258	0.181101
H	-3.772108	-5.756158	0.768108
H	-4.532928	-5.179799	-0.727523
H	-4.709637	-4.256296	0.769769
H	3.049815	1.332888	0.976069
C	-1.575850	0.487275	0.402902
C	-2.006202	0.858180	-0.880188
C	-2.545875	0.049051	1.315507
C	-3.351408	0.771049	-1.242180
H	-1.281324	1.192355	-1.614557
C	-3.893304	-0.038462	0.954209
H	-2.246022	-0.226087	2.321873
C	-4.304298	0.320366	-0.328480
H	-5.348665	0.256460	-0.609719
C	-4.889391	-0.582532	1.942000
C	-3.750644	1.071400	-2.660226
F	-4.648391	-0.139243	3.194880
F	-4.847282	-1.938676	1.997966
F	-6.158586	-0.243647	1.626884
F	-5.066023	1.358083	-2.773514
F	-3.507997	0.010958	-3.472874
F	-3.061645	2.116134	-3.170066
O	0.411873	2.151785	0.289156
C	0.516904	3.116103	1.068403
C	0.849426	4.455744	0.627987
C	1.061144	4.744216	-0.735090
C	0.957429	5.474532	1.592405
C	1.376260	6.040039	-1.119931
H	0.972725	3.945425	-1.464351
C	1.274586	6.770765	1.199152
H	0.792966	5.241813	2.641532
C	1.482873	7.050406	-0.154486
H	1.539793	6.271471	-2.167998
H	1.359823	7.560136	1.939432
H	1.730143	8.062689	-0.462045
H	0.354619	2.943902	2.140840

NTOB-benzaldehyde complex 69

B3LYP/6-31G(d) = -2780.348415
B3LYP-D3(BJ)/def2-TZVPP/IEFPCM(propanonitrile) = -2781.607131

B3LYP-D3(BJ)/def2-TZVPP/IEFPCM(propanonitrile)//B3LYP-D3(BJ)/6-31G(d) Free Energy (Quasiharmonic) = -2781.120721

Frequencies (Top 3 out of 213)

1. 6.0838 cm^{-1}
2. 12.2628 cm^{-1}
3. 13.2410 cm^{-1}

B3LYP/6-31G(d) Molecular Geometry in Cartesian Coordinates

B	-0.971355	0.733382	-0.589800
O	-0.768528	1.592138	-1.744030
N	-0.535529	1.610574	0.588218
C	-0.121129	2.944384	0.092874
S	-0.971050	1.441544	2.194945
C	-0.397985	2.842035	-1.409092
O	-0.301725	3.753631	-2.194091
H	1.438515	3.459627	1.470897
H	-0.771126	3.724547	0.503774
C	1.338166	3.391046	0.382858
C	2.449842	2.562850	-0.199023
C	3.075243	2.706879	-1.497875
C	4.103737	1.727643	-1.578598
N	4.112011	1.041250	-0.381347
C	3.115475	1.539861	0.433068
H	2.973788	1.135200	1.425340
C	4.703422	2.453102	-3.767656
C	2.885905	3.575459	-2.588861
H	5.696368	0.824138	-2.746824
H	4.633517	0.199605	-0.192010
C	3.698752	3.440666	-3.706702
H	5.318900	2.369974	-4.659474
H	2.097400	4.320381	-2.567918
C	4.919963	1.583969	-2.705760
H	3.553864	4.103455	-4.555481
C	0.202405	0.340512	2.984227
O	-2.269836	0.739867	2.236812
O	-0.833316	2.765488	2.816875
C	2.078079	-1.393448	4.129900
C	-0.035091	-1.036448	2.974991
C	1.336944	0.866801	3.602306
C	2.266781	-0.005542	4.169987
C	0.903791	-1.890490	3.544010
H	-0.941744	-1.426273	2.526672
H	1.481033	1.941161	3.648923
H	3.154068	0.400618	4.649160
H	0.731646	-2.962946	3.518424
C	3.111124	-2.345470	4.680502
H	3.522136	-2.963416	3.872845
H	3.939259	-1.813160	5.157678
H	2.674494	-3.027167	5.419947
O	-2.612853	0.499386	-0.587531
C	-3.429240	1.304811	-0.095856
H	-3.070833	2.257742	0.303185
H	1.406943	4.417915	0.003087
C	-4.852024	1.056744	-0.070177
C	-5.397431	-0.138982	-0.578561
C	-5.693897	2.042164	0.477581
C	-6.770585	-0.335942	-0.540677
H	-4.734252	-0.891725	-0.991249
C	-7.069134	1.837583	0.512355
H	-5.262757	2.956636	0.876341
C	-7.604022	0.650971	0.002840
H	-7.198967	-1.254114	-0.930690
H	-7.722561	2.594423	0.934837
H	-8.678291	0.490590	0.030164
C	-0.410457	-0.761025	-0.751477
C	-1.093489	-1.731556	-1.497526
C	0.834636	-1.125821	-0.215918
C	-0.564964	-3.011130	-1.695085
H	-2.050859	-1.489544	-1.946635
C	1.364940	-2.403593	-0.402184
H	1.401856	-0.393089	0.342981
C	0.666709	-3.357629	-1.145114
H	1.079795	-4.347758	-1.302439
C	2.666477	-2.804337	0.235010
C	-1.366848	-4.032647	-2.454950
F	3.452971	-3.502220	-0.612325
F	2.472266	-3.600461	1.320038
F	-2.310113	-4.608852	-1.668642
F	3.389665	-1.741422	0.670289
F	-0.595843	-5.030892	-2.937924
F	-2.022336	-3.481241	-3.500607

NTOB-benzaldehyde complex 70

B3LYP/6-31G(d) = -2780.350049
B3LYP-D3(BJ)/def2-TZVPP/IEFPCM(propanonitrile) = -2781.605518
B3LYP-D3(BJ)/def2-TZVPP/IEFPCM(propanonitrile)//B3LYP-D3(BJ)/6-31G(d) Free Energy (Quasiharmonic) = -2781.120371

Frequencies (Top 3 out of 213)

1. 8.4843 cm^{-1}
2. 11.7804 cm^{-1}
3. 12.0807 cm^{-1}

B3LYP/6-31G(d) Molecular Geometry in Cartesian Coordinates

B	-0.605620	0.854812	0.565367
O	0.007644	1.078446	1.866604
N	0.578030	0.702215	-0.363983
C	1.844362	0.789830	0.383718
S	0.524089	0.072714	-1.908849
C	1.359591	1.108284	1.802743
O	2.058397	1.370618	2.750120
H	3.018660	2.594787	0.670998
H	2.337171	-0.191981	0.427061
C	2.879821	1.849938	-0.121848
C	4.217954	1.284607	-0.503186
C	5.266354	0.846990	0.389960
C	6.353370	0.412658	-0.418983
N	5.970606	0.575656	-1.735672
C	4.691164	1.098694	-1.778997
H	4.197794	1.288599	-2.721415
C	7.641837	-0.121441	1.514267
C	5.391782	0.781647	1.789925
H	8.367346	-0.398294	-0.505436
H	6.539701	0.367276	-2.540858
C	6.575648	0.299654	2.335425
H	8.554938	-0.492930	1.971929
H	4.568782	1.091153	2.428094
C	7.545879	-0.072209	0.127922
H	6.683589	0.242835	3.415342
C	0.804767	-1.690182	-1.724516
O	1.662366	0.616752	-2.659224
O	-0.848841	0.280747	-2.380733
C	1.244441	-4.419714	-1.274332
C	-0.286970	-2.549194	-1.567473
C	2.112277	-2.179370	-1.680112
C	2.319699	-3.540666	-1.454519
C	-0.058926	-3.902828	-1.339349
H	-1.295948	-2.157896	-1.628525
H	2.950999	-1.507291	-1.829535
H	3.336502	-3.923399	-1.415576
H	-0.907219	-4.567175	-1.196398
C	1.471264	-5.892472	-1.030120
H	1.181849	-6.487911	-1.905631
H	0.872209	-6.250296	-0.184967
H	2.522718	-6.106792	-0.815980

H	2.435710	2.357212	-0.980439
C	-1.818547	-0.189109	0.598123
C	-2.977705	-0.067356	-0.181822
C	-1.713993	-1.313053	1.431316
C	-3.992318	-1.026080	-0.128569
H	-3.088786	0.778325	-0.849947
C	-2.714841	-2.287139	1.468993
H	-0.839691	-1.434105	2.063110
C	-3.864679	-2.146167	0.692910
H	-4.651945	-2.890026	0.735402
C	-2.519830	-3.535501	2.285040
C	-5.201202	-0.885878	-1.014163
F	-1.758295	-3.317575	3.377623
F	-1.899676	-4.504340	1.562225
F	-3.694459	-4.057541	2.703387
F	-5.527505	0.410859	-1.218957
F	-6.286673	-1.496646	-0.486415
F	-4.996170	-1.435624	-2.233068
O	-1.306081	2.301396	0.148210
C	-1.378682	3.283239	0.915030
C	-1.949449	4.544726	0.505612
C	-2.425874	4.736809	-0.808048
C	-2.020531	5.589238	1.447113
C	-2.968818	5.963038	-1.164637
H	-2.354462	3.922633	-1.522168
C	-2.567971	6.814122	1.081369
H	-1.647509	5.431187	2.455871
C	-3.040478	6.997743	-0.221737
H	-3.336820	6.121362	-2.173588
H	-2.626887	7.622960	1.802861
H	-3.467037	7.955180	-0.507780
H	-0.988677	3.179011	1.934607

NTOB-benzaldehyde complex 71

B3LYP/6-31G(d) = -2780.349412
B3LYP-D3(BJ)/def2-TZVPP/IEFPCM(propanonitrile) = -2781.605105
B3LYP-D3(BJ)/def2-TZVPP/IEFPCM(propanonitrile)//B3LYP-D3(BJ)/6-31G(d) Free Energy (Quasiharmonic) = -2781.120107

Frequencies (Top 3 out of 213)

1. 7.6085 cm^{-1}
2. 10.4735 cm^{-1}
3. 12.3695 cm^{-1}

B3LYP/6-31G(d) Molecular Geometry in Cartesian Coordinates

B	0.892445	0.048883	0.903054
O	0.546868	-0.256525	2.280197
N	-0.451504	-0.015304	0.187023
C	-1.496054	-0.506290	1.100806
S	-0.823212	0.564460	-1.316694
C	-0.759209	-0.532443	2.448257
O	-1.252940	-0.807294	3.515547
H	-1.385400	-2.680002	1.129405
H	-2.326106	0.206461	1.172729
C	-2.066587	-1.912537	0.741031
C	-3.475034	-2.137760	1.218058
C	-4.636059	-2.288833	0.373235
C	-5.761893	-2.457458	1.227423
N	-5.294037	-2.406758	2.524143
C	-3.925726	-2.213315	2.514094
H	-3.368024	-2.115672	3.434280
C	-7.223715	-2.633681	-0.649341
C	-4.836186	-2.299692	-1.019809
H	-7.905806	-2.762066	1.399514
H	-5.858629	-2.496741	3.354198
C	-6.123655	-2.468561	-1.516101
H	-8.218017	-2.767678	-1.066844
H	-3.995421	-2.182576	-1.699786
C	-7.057963	-2.631985	0.731359
H	-6.287173	-2.480203	-2.590376
C	-2.107073	1.797642	-1.068602
O	-1.412122	-0.495359	-2.145258
O	0.394567	1.257678	-1.785926
C	-4.105794	3.721995	-0.670878
C	-3.450921	1.422239	-1.137057
C	-1.749366	3.123193	-0.812398
C	-2.748780	4.072928	-0.616829
C	-4.437173	2.385637	-0.930998
H	-3.724727	0.395585	-1.354592
H	-0.703701	3.408921	-0.786206
H	-2.471127	5.106409	-0.423474
H	-5.482014	2.089538	-0.977562
C	-5.182856	4.763884	-0.485828
H	-5.479940	5.196705	-1.450285
H	-4.840537	5.587382	0.149318
H	-6.081780	4.333861	-0.032490
O	1.319838	1.619796	0.974346
C	2.057538	2.157399	0.116607
H	2.556683	1.530898	-0.625033
H	-2.037558	-2.001509	-0.347453
C	2.299084	3.582339	0.099161
C	3.136999	4.108415	-0.901663
C	1.723846	4.435042	1.063557
C	3.397156	5.474419	-0.939940
H	3.570584	3.442970	-1.643346
C	1.990649	5.796564	1.019889
H	1.084441	4.010474	1.830680
C	2.825361	6.314329	0.020013
H	4.040947	5.885256	-1.711305
H	1.555701	6.460016	1.761197
H	3.030845	7.380854	-0.008459
C	2.146585	-0.796518	0.365482
C	3.322900	-0.848896	1.132698
C	2.112525	-1.547951	-0.818040
C	4.424810	-1.604512	0.728478
H	3.375462	-0.305937	2.072560
C	3.211453	-2.313089	-1.222327
H	1.219461	-1.547519	-1.434660
C	4.374495	-2.342101	-0.454930
H	5.226338	-2.932915	-0.770625
C	3.116502	-3.161327	-2.463784
C	5.693552	-1.571294	1.537187
F	2.360459	-2.576090	-3.415478
F	4.334734	-3.401525	-3.002294
F	6.477112	-0.520647	1.185049
F	2.559092	-4.365987	-2.201282
F	6.435748	-2.686436	1.364898
F	5.446996	-1.444527	2.858903

NTOB-benzaldehyde complex 72

B3LYP/6-31G(d) = -2780.350047
B3LYP-D3(BJ)/def2-TZVPP/IEFPCM(propanonitrile) = -2781.605339
B3LYP-D3(BJ)/def2-TZVPP/IEFPCM(propanonitrile)//B3LYP-D3(BJ)/6-31G(d) Free Energy (Quasiharmonic) = -2781.120087

Frequencies (Top 3 out of 213)

1. 9.2406 cm^{-1}
2. 12.1446 cm^{-1}
3. 13.8835 cm^{-1}

B3LYP/6-31G(d) Molecular Geometry in Cartesian Coordinates

B	0.620033	0.823881	-0.548321
O	0.023554	1.049087	-1.857068
N	-0.574626	0.681465	0.367142

C	-1.832065	0.792439	-0.392023
S	-0.548050	0.051475	1.912290
C	-1.328527	1.097017	-1.807491
O	-2.014013	1.364496	-2.763130
H	-2.969071	2.618735	-0.696277
H	-2.344497	-0.179140	-0.436388
C	-2.849921	1.875456	0.101280
C	-4.201302	1.338576	0.476672
C	-5.250568	0.913214	-0.421318
C	-6.351869	0.505875	0.382304
N	-5.976539	0.672494	1.700698
C	-4.687967	1.172179	1.750065
H	-4.198644	1.360668	2.694890
C	-7.634666	-0.019997	-1.556952
C	-5.366018	0.838728	-1.821685
H	-8.381304	-0.266640	0.458957
H	-6.556143	0.482322	2.502890
C	-6.554310	0.374395	-2.372842
H	-8.550887	-0.378151	-2.018982
H	-4.532398	1.127771	-2.455683
C	-7.548850	0.038841	-0.170314
H	-6.654730	0.310743	-3.453101
C	-0.877823	-1.702562	1.724305
O	-1.675361	0.626561	2.656153
O	0.827041	0.220393	2.392910
C	-1.392756	-4.419070	1.275047
C	0.189182	-2.588412	1.548295
C	-2.197796	-2.158121	1.696474
C	-2.442735	-3.513268	1.471608
C	-0.075895	-3.935331	1.320356
H	1.208368	-2.222443	1.593780
H	-3.016698	-1.464637	1.857200
H	-3.469397	-3.869951	1.445887
H	0.753362	-4.619604	1.161316
C	-1.661245	-5.885810	1.036899
H	-1.051125	-6.272035	0.212674
H	-1.416978	-6.481608	1.925996
H	-2.713039	-6.067491	0.795685
H	-2.401428	2.378453	0.960164
C	1.829589	-0.224244	-0.567237
C	2.989551	-0.096897	0.210658
C	1.721384	-1.357434	-1.387398
C	4.001889	-1.058642	0.167338
H	3.097830	0.749037	0.879052
C	2.717597	-2.336783	-1.411083
H	0.845781	-1.484164	-2.016291
C	3.868983	-2.189495	-0.638421
H	4.647610	-2.943161	-0.661613
C	2.513580	-3.598329	-2.204220
C	5.270227	-0.846070	0.949271
F	1.753961	-3.394104	-3.300721
F	1.885905	-4.550214	-1.465034
F	3.684318	-4.136479	-2.612695
F	6.174003	-0.123902	0.241367
F	5.869936	-2.014743	1.268951
F	5.050665	-0.173164	2.098889
O	1.324291	2.270628	-0.126878
C	1.414964	3.247176	-0.898473
C	1.987742	4.507780	-0.488780
C	2.446139	4.705759	0.830384
C	2.079626	5.545352	-1.436116
C	2.991966	5.930796	1.186676
H	2.359002	3.896985	1.548895
C	2.629801	6.769102	-1.070636
H	1.720504	5.382825	-2.449195
C	3.084356	6.958550	0.237988
H	3.346224	6.093582	2.199831
H	2.704753	7.572534	-1.796665
H	3.513111	7.915072	0.523799
H	1.039797	3.139089	-1.923224

NTOB-benzaldehyde complex 73

B3LYP/6-31G(d) = -2780.350188
B3LYP-D3(BJ)/def2-TZVPP/IEFPCM(propanonitrile) = -2781.604324
B3LYP-D3(BJ)/def2-TZVPP/IEFPCM(propanonitrile)//B3LYP-D3(BJ)/6-31G(d) Free Energy (Quasiharmonic) = -2781.120037

Frequencies (Top 3 out of 213)

1. 6.7503 cm^{-1}
2. 9.8164 cm^{-1}
3. 12.1990 cm^{-1}

B3LYP/6-31G(d) Molecular Geometry in Cartesian Coordinates

B	-0.530724	0.672624	-0.918864
O	0.042743	0.642914	-2.247026
N	0.651381	0.316538	-0.025662
C	1.895753	0.237102	-0.819631
S	0.710865	0.381138	1.630164
C	1.384735	0.473685	-2.246636
O	2.053760	0.479657	-3.248351
H	2.074931	-1.893735	-1.208579
H	2.583816	1.045992	-0.548737
C	2.658233	-1.112432	-0.707738
C	4.055037	-1.056017	-1.250418
C	5.201918	-0.465259	-0.601374
C	6.312741	-0.636984	-1.473573
N	5.850497	-1.300547	-2.592389
C	4.497935	-1.541178	-2.454255
H	3.942490	-2.028792	-3.243055
C	7.758569	0.461519	0.071567
C	5.398820	0.191043	0.627335
H	8.432523	-0.323048	-1.828743
H	6.401991	-1.538399	-3.401614
C	6.671687	0.646695	0.950237
H	8.741959	0.829037	0.352399
H	4.566552	0.350829	1.308452
C	7.594745	-0.181928	-1.150354
H	6.834639	1.157310	1.895693
C	0.724305	-1.303398	2.249065
O	-0.549581	1.013394	2.056316
O	1.991595	1.000044	2.015700
C	0.710627	-3.892832	3.311841
C	1.732938	-1.689730	3.130837
C	-0.300916	-2.186482	1.897371
C	-0.297123	-3.471658	2.428659
C	1.717823	-2.983192	3.654236
H	2.514014	-0.986648	3.398100
H	-1.088502	-1.875079	1.219545
H	-1.092022	-4.161186	2.155058
H	2.503747	-3.287052	4.340949
C	0.709154	-5.299076	3.861316
H	1.403822	-5.402710	4.700288
H	1.008084	-6.022699	3.091936
H	-0.288668	-5.591096	4.207495
O	-0.939354	2.282585	-0.726272
C	-0.306242	3.122460	-0.055485
H	0.615732	2.822056	0.452800
H	2.695350	-1.385106	0.352222
C	-0.732053	4.497266	0.068692
C	0.045004	5.369875	0.853539
C	-1.896760	4.964480	-0.573417
C	-0.337642	6.699745	0.993942
H	0.936785	4.996170	1.350010
C	-2.270934	6.292780	-0.428651
H	-2.483176	4.275324	-1.172129
C	-1.492486	7.157578	0.353057
H	0.256435	7.377249	1.599191
H	-3.166128	6.662286	-0.919312

H	-1.791542	8.196350	0.462996
C	-1.943033	-0.077149	-0.814283
C	-2.907799	0.188694	0.170766
C	-2.251732	-1.054905	-1.773230
C	-4.126185	-0.495836	0.195657
H	-2.701661	0.923328	0.940652
C	-3.463707	-1.749837	-1.743001
H	-1.541935	-1.264179	-2.566968
C	-4.411312	-1.471656	-0.758971
H	-5.355659	-2.002752	-0.738158
C	-3.724415	-2.843627	-2.743397
C	-5.164268	-0.127672	1.221637
F	-3.217255	-4.029961	-2.326368
F	-5.046645	-3.036430	-2.950297
F	-6.032167	-1.139400	1.450694
F	-3.159874	-2.578628	-3.941051
F	-5.901672	0.938003	0.823571
F	-4.608024	0.205538	2.405960

NTOB-benzaldehyde complex 74

B3LYP/6-31G(d) = -2780.349537
B3LYP-D3(BJ)/def2-TZVPP/IEFPCM(propanonitrile) = -2781.605132
B3LYP-D3(BJ)/def2-TZVPP/IEFPCM(propanonitrile)//B3LYP-D3(BJ)/6-31G(d) Free Energy (Quasiharmonic) = -2781.120035

Frequencies (Top 3 out of 213)

1. 7.7689 cm^{-1}
2. 11.4378 cm^{-1}
3. 12.6429 cm^{-1}

B3LYP/6-31G(d) Molecular Geometry in Cartesian Coordinates

B	0.884935	-0.034119	-0.914357
O	0.534634	0.274135	-2.289619
N	-0.456788	0.026403	-0.193902
C	-1.505188	0.517042	-1.103384
S	-0.822321	-0.556598	1.310091
C	-0.772516	0.547806	-2.453018
O	-1.270049	0.824314	-3.518121
H	-1.399572	2.691018	-1.128222
H	-2.333915	-0.197353	-1.174356
C	-2.077667	1.921294	-0.738904
C	-3.488252	2.144190	-1.210678
C	-4.646715	2.291102	-0.361610
C	-5.775854	2.458679	-1.211631
N	-5.312336	2.411409	-2.510040
C	-3.943563	2.221013	-2.505023
H	-3.388792	2.126273	-3.427284
C	-7.231620	2.628163	0.670450
C	-4.842071	2.298986	1.032134
H	-7.921045	2.758654	-1.375817
H	-5.880027	2.501268	-3.337992
C	-6.128214	2.464003	1.533133
H	-8.224789	2.759103	1.091603
H	-3.998710	2.182512	1.708996
C	-7.070612	2.629338	-0.710812
H	-6.288076	2.473293	2.607980
C	-2.103668	-1.792772	1.064043
O	-1.411374	0.500312	2.142276
O	0.398814	-1.247103	1.774497
C	-4.098527	-3.721766	0.669463
C	-3.448278	-1.420915	1.136724
C	-1.743261	-3.117082	0.805191
C	-2.740767	-4.069136	0.611205
C	-4.432618	-2.386599	0.932188
H	-3.724120	-0.395259	1.356395
H	-0.696948	-3.400096	0.775782
H	-2.460987	-5.101659	0.415809
H	-5.478087	-2.093269	0.982070
C	-5.173387	-4.766230	0.486134
H	-5.465536	-5.202114	1.450720
H	-4.831205	-5.587312	-0.152214
H	-6.075237	-4.337690	0.037236
O	1.314786	-1.604388	-0.990739
C	2.056499	-2.142672	-0.136839
H	2.556619	-1.517108	0.604929
H	-2.044968	2.008364	0.349624
C	2.301089	-3.567113	-0.123734
C	1.724751	-4.418734	-1.088439
C	3.143170	-4.093748	0.873321
C	1.994617	-5.779767	-1.048841
H	1.082095	-3.993711	-1.852591
C	3.406363	-5.459284	0.907518
H	3.577547	-3.429132	1.615290
C	2.833471	-6.298111	-0.052723
H	1.558856	-6.442395	-1.790411
H	4.053355	-5.870571	1.675958
H	3.041325	-7.364258	-0.027434
C	2.139776	0.809930	-0.376633
C	3.313291	0.869796	-1.147429
C	2.107015	1.555985	0.810306
C	4.412541	1.630667	-0.745423
H	3.363566	0.333334	-2.091167
C	3.204831	2.322189	1.214992
H	1.210179	1.563020	1.421326
C	4.364305	2.360995	0.442489
H	5.209650	2.965091	0.750436
C	3.155161	3.068891	2.522409
C	5.673552	1.616674	-1.566867
F	1.914830	3.521853	2.796984
F	3.525668	2.278606	3.558112
F	5.414947	1.508523	-2.887978
F	3.988968	4.133939	2.525733
F	6.465615	0.564832	-1.238330
F	6.411612	2.732960	-1.384894

NTOB-benzaldehyde complex 75

B3LYP/6-31G(d) = -2780.342342
B3LYP-D3(BJ)/def2-TZVPP/IEFPCM(propanonitrile) = -2781.604021
B3LYP-D3(BJ)/def2-TZVPP/IEFPCM(propanonitrile)//B3LYP-D3(BJ)/6-31G(d) Free Energy (Quasiharmonic) = -2781.119853

Frequencies (Top 3 out of 213)

1. 9.4159 cm^{-1}
2. 12.5405 cm^{-1}
3. 14.6715 cm^{-1}

B3LYP/6-31G(d) Molecular Geometry in Cartesian Coordinates

B	-0.004024	0.325151	-1.020317
O	-0.011551	0.449893	-2.462772
N	-0.818961	-0.892741	-0.724795
C	-1.540637	-1.286230	-1.946124
S	-0.565610	-2.004674	0.513635
C	-0.842074	-0.464065	-3.035117
O	-1.027255	-0.540108	-4.222545
H	-3.355307	-1.072524	-3.039778
H	-1.431006	-2.354231	-2.150947
C	-3.057563	-0.923904	-1.994680
C	-3.979328	-1.685861	-1.091472
C	-4.375718	-1.348056	0.254569
C	-5.298882	-2.341209	0.682941
N	-5.446900	-3.234834	-0.357735
C	-4.655191	-2.835644	-1.415868
H	-4.640250	-3.406988	-2.334617
C	-5.562591	-1.264255	2.792883

C	-4.050108	-0.305444	1.138939
H	-6.602245	-3.082927	2.254108
H	-6.016721	-4.065643	-0.338363
C	-4.645210	-0.271277	2.393400
H	-6.008010	-1.213933	3.782814
H	-3.319622	0.446409	0.853976
C	-5.900112	-2.312561	1.945141
H	-4.390511	0.524773	3.088101
C	1.092143	-2.674979	0.294265
O	-1.497411	-3.108468	0.270134
O	-0.563195	-1.252403	1.771752
C	3.650582	-3.776032	-0.025472
C	1.349788	-3.529733	-0.781694
C	2.088356	-2.386143	1.226342
C	3.357884	-2.939142	1.058324
C	2.624237	-4.066541	-0.937446
H	0.558706	-3.792032	-1.476810
H	1.872797	-1.741279	2.070084
H	4.133002	-2.714697	1.786921
H	2.823782	-4.730852	-1.774852
C	5.037804	-4.339558	-0.220439
H	5.586371	-4.390372	0.725419
H	5.623526	-3.712423	-0.905609
H	5.005302	-5.346340	-0.650488
H	-3.154531	0.150920	-1.794860
C	1.375613	0.704830	-0.325376
C	1.490070	0.993171	1.043137
C	2.538307	0.776819	-1.107338
C	2.726489	1.306722	1.611308
H	0.613591	0.939146	1.679080
C	3.772981	1.106125	-0.542195
H	2.479405	0.567017	-2.170421
C	3.875771	1.366913	0.823696
H	4.834270	1.609341	1.267164
C	4.987236	1.233911	-1.422043
C	2.820571	1.485342	3.101396
F	4.963367	0.351728	-2.445006
F	6.133816	1.027880	-0.735123
F	5.077074	2.468415	-1.972826
F	2.840789	0.286628	3.738299
F	1.766223	2.170000	3.595788
F	3.941524	2.143852	3.470102
O	-1.024982	1.673566	-0.496480
C	-1.266251	2.643880	-1.237624
C	-2.048092	3.788383	-0.817304
C	-2.570424	3.878567	0.488593
C	-2.278958	4.821157	-1.744917
C	-3.315291	4.991804	0.852729
H	-2.380296	3.074873	1.192380
C	-3.026886	5.933381	-1.372728
H	-1.871584	4.742520	-2.749791
C	-3.543066	6.016019	-0.076232
H	-3.721778	5.068828	1.856427
H	-3.208612	6.732106	-2.085075
H	-4.127484	6.884431	0.214949
H	-0.870568	2.631197	-2.262068

NTOB-benzaldehyde complex 76

B3LYP/6-31G(d) = -2780.34479
B3LYP-D3(BJ)/def2-TZVPP/IEFPCM(propanonitrile) = -2781.6064
B3LYP-D3(BJ)/def2-TZVPP/IEFPCM(propanonitrile)//B3LYP-D3(BJ)/6-31G(d) Free Energy (Quasiharmonic) = -2781.119616

Frequencies (Top 3 out of 213)

1. 8.3672 cm^{-1}
2. 15.6704 cm^{-1}
3. 20.0587 cm^{-1}

B3LYP/6-31G(d) Molecular Geometry in Cartesian Coordinates

B	0.932777	0.497249	-1.309632
O	1.153878	0.805183	-2.723130
N	0.520149	-1.016466	-1.320944
C	0.079238	-1.299200	-2.711398
S	1.390681	-2.245237	-0.586911
C	0.700027	-0.172933	-3.541442
O	0.746919	-0.117109	-4.745019
H	-1.627886	-1.334093	-3.988261
H	0.456776	-2.262604	-3.060138
C	-1.462913	-1.268865	-2.905595
C	-2.229595	-2.340520	-2.186794
C	-3.011334	-2.189364	-0.983077
C	-3.557710	-3.466012	-0.668906
N	-3.130897	-4.339098	-1.647059
C	-2.334524	-3.660951	-2.548555
H	-1.893519	-4.183627	-3.386568
C	-4.628566	-2.587702	1.273535
C	-3.302436	-1.104572	-0.135913
H	-4.774047	-4.661990	0.676428
H	-3.324320	-5.327849	-1.674284
C	-4.101199	-1.311657	0.982079
H	-5.252567	-2.720103	2.153355
H	-2.894600	-0.119364	-0.342489
C	-4.366615	-3.679220	0.452759
H	-4.311646	-0.480226	1.648260
C	1.025296	-2.000952	1.146729
O	2.859364	-2.050787	-0.751876
O	0.836558	-3.524677	-1.040935
C	0.417496	-1.550866	3.840212
C	2.047345	-1.662071	2.032850
C	-0.290633	-2.159992	1.591081
C	-0.583705	-1.929318	2.930731
C	1.734079	-1.440483	3.374783
H	3.066402	-1.575268	1.672826
H	-1.075762	-2.432931	0.895799
H	-1.612032	-2.024390	3.267886
H	2.525651	-1.169676	4.069328
C	0.066388	-1.229745	5.271947
H	-0.665536	-1.937910	5.675123
H	-0.378552	-0.228757	5.331535
H	0.949413	-1.243618	5.918501
O	2.308331	0.694457	-0.569832
C	3.396277	0.340802	-1.098201
H	3.395347	-0.000817	-2.133502
H	-1.833387	-0.284491	-2.596573
C	4.655919	0.488288	-0.412502
C	5.830397	0.086712	-1.075105
C	4.723479	1.031297	0.886551
C	7.062186	0.223259	-0.444261
H	5.765355	-0.337259	-2.073386
C	5.957380	1.167538	1.507705
H	3.807949	1.339988	1.380139
C	7.123430	0.763592	0.843593
H	7.970876	-0.088583	-0.949495
H	6.019887	1.588699	2.506479
H	8.086189	0.872641	1.335241
C	-0.080590	1.509497	-0.578876
C	-0.702400	2.545882	-1.290103
C	-0.389636	1.387994	0.784208
C	-1.602813	3.418069	-0.669677
H	-0.482150	2.672592	-2.345610
C	-1.293940	2.251496	1.407222
H	0.073653	0.605001	1.374877
C	-1.908769	3.273217	0.682841
H	-2.613532	3.941876	1.162184
C	-1.643327	2.027322	2.852693
C	-2.199082	4.556342	-1.454099
F	-0.539485	1.787557	3.605392
F	-2.279614	3.083659	3.400994

F	-2.440574	4.210462	-2.737022
F	-2.452439	0.949660	3.016270
F	-1.369435	5.626698	-1.486900
F	-3.366544	4.982121	-0.920808

NTOB-benzaldehyde complex 77

B3LYP/6-31G(d) = -2780.342725
B3LYP-D3(BJ)/def2-TZVPP/IEFPCM(propanonitrile) = -2781.604871
B3LYP-D3(BJ)/def2-TZVPP/IEFPCM(propanonitrile)//B3LYP-D3(BJ)/6-31G(d) Free Energy (Quasiharmonic) = -2781.11924

Frequencies (Top 3 out of 213)

1. 8.6113 cm^{-1}
2. 9.5464 cm^{-1}
3. 10.9892 cm^{-1}

B3LYP/6-31G(d) Molecular Geometry in Cartesian Coordinates

B	-0.726876	0.056071	0.908896
O	-0.676230	0.333802	2.336765
N	0.300218	-1.076252	0.735564
C	0.793302	-1.497152	2.059440
S	0.219919	-2.146926	-0.535218
C	0.027779	-0.579195	3.019603
O	0.082650	-0.639421	4.225084
H	2.873970	-1.879456	1.558940
H	0.507589	-2.536639	2.263897
C	2.320435	-1.341075	2.333263
C	2.849280	0.066711	2.464788
C	3.654556	0.811616	1.524439
C	3.939193	2.076994	2.116639
N	3.335007	2.087569	3.354558
C	2.693522	0.884113	3.561455
H	2.153020	0.698847	4.478281
C	5.239222	2.738542	0.227306
C	4.197304	0.532343	0.257565
H	4.933561	4.001596	1.958642
H	3.357359	2.850312	4.013328
C	4.979286	1.490635	-0.376230
H	5.865036	3.463476	-0.286805
H	4.016171	-0.425350	-0.218988
C	4.721966	3.047833	1.481278
H	5.404652	1.274346	-1.352801
C	1.894394	-2.610008	-0.994636
O	-0.327265	-1.358948	-1.655796
O	-0.430880	-3.398738	-0.114872
C	4.441474	-3.422261	-1.836504
C	2.558169	-1.883361	-1.982716
C	2.475252	-3.752647	-0.437358
C	3.746108	-4.141678	-0.852258
C	3.825517	-2.294727	-2.395636
H	2.079532	-1.020235	-2.431789
H	1.926858	-4.336667	0.294086
H	4.200209	-5.027865	-0.415606
H	4.341710	-1.732584	-3.169936
C	5.804660	-3.876482	-2.300102
H	6.311027	-3.099435	-2.880595
H	5.726677	-4.767693	-2.936087
H	6.446782	-4.140433	-1.452472
H	2.497152	-1.879612	3.271943
C	-2.265147	-0.006749	0.423581
C	-3.006564	1.183328	0.342582
C	-2.967103	-1.207231	0.226988
C	-4.372709	1.184611	0.048430
H	-2.520200	2.138477	0.520454
C	-4.333905	-1.211425	-0.061539
H	-2.453799	-2.160010	0.299874
C	-5.046366	-0.016619	-0.159056
H	-6.105742	-0.023160	-0.386448
C	-5.036525	-2.516200	-0.332522
C	-5.122779	2.488083	0.016742
F	-6.356680	-2.444434	-0.040796
F	-4.516403	-3.529289	0.390339
F	-4.944278	-2.870904	-1.635508
F	-6.257448	2.404874	-0.712496
F	-4.371991	3.482146	-0.518717
F	-5.478410	2.897919	1.255619
O	-0.019796	1.334323	0.199996
C	-0.168900	1.587560	-1.017977
C	0.599939	2.602703	-1.694309
C	0.271678	2.906314	-3.030787
C	1.656866	3.277499	-1.049449
C	0.980292	3.889985	-3.710583
H	-0.538822	2.371939	-3.519631
C	2.364870	4.251264	-1.740350
H	1.917345	3.014150	-0.029887
C	2.023852	4.560221	-3.063468
H	0.727538	4.133958	-4.737732
H	3.189911	4.761097	-1.253901
H	2.581264	5.325627	-3.596735
H	-0.904141	1.014349	-1.588972

NTOB-benzaldehyde complex 78

B3LYP/6-31G(d) = -2780.351873
B3LYP-D3(BJ)/def2-TZVPP/IEFPCM(propanonitrile) = -2781.604471
B3LYP-D3(BJ)/def2-TZVPP/IEFPCM(propanonitrile)//B3LYP-D3(BJ)/6-31G(d) Free Energy (Quasiharmonic) = -2781.11909

Frequencies (Top 3 out of 213)

1. 9.3935 cm^{-1}
2. 10.0153 cm^{-1}
3. 14.8572 cm^{-1}

B3LYP/6-31G(d) Molecular Geometry in Cartesian Coordinates

B	-0.528714	0.828991	0.867383
O	0.132281	0.813002	2.159082
N	0.637826	0.858358	-0.126461
C	1.921690	0.743022	0.603480
S	0.568418	0.499704	-1.753462
C	1.473007	0.822496	2.067595
O	2.208935	0.882157	3.022289
H	3.192313	2.401235	1.179495
H	2.350255	-0.259048	0.463681
C	3.015703	1.809898	0.273398
C	4.321593	1.239660	-0.204252
C	5.337467	0.594425	0.597038
C	6.403724	0.245228	-0.277890
N	6.041083	0.656110	-1.545416
C	4.793878	1.250968	-1.493998
H	4.321522	1.630558	-2.388558
C	7.645907	-0.689072	1.529216
C	5.449297	0.281254	1.964421
H	8.368587	-0.653048	-0.510195
H	6.603576	0.558145	-2.375807
C	6.599952	-0.355279	2.414047
H	8.533048	-1.186061	1.912921
H	4.641084	0.523545	2.648551
C	7.562723	-0.395149	0.172736
H	6.697091	-0.603533	3.467597
C	0.688856	-1.277278	-1.951020
O	1.752414	1.083526	-2.395295
O	-0.774941	0.925356	-2.186749
C	0.905203	-4.056360	-2.218996
C	-0.462594	-2.039515	-2.161418
C	1.949675	-1.880314	-1.893719

C	2.044656	-3.264705	-2.018592
C	-0.342466	-3.421081	-2.293702
H	-1.432647	-1.562431	-2.233465
H	2.842131	-1.274739	-1.772753
H	3.022815	-3.736205	-1.966313
H	-1.237605	-4.015403	-2.459429
C	1.020994	-5.552519	-2.385957
H	1.910825	-5.944254	-1.883067
H	1.097721	-5.824603	-3.447073
H	0.144756	-6.069252	-1.980970
H	2.609358	2.480883	-0.485659
C	-1.732971	-0.233098	0.786715
C	-1.759276	-1.282464	1.720070
C	-2.769936	-0.187762	-0.155242
C	-2.761192	-2.255502	1.697764
H	-0.985461	-1.338839	2.478120
C	-3.763668	-1.169416	-0.192329
H	-2.793635	0.600407	-0.898977
C	-3.767904	-2.210631	0.733797
H	-4.538224	-2.972154	0.704226
C	-4.770302	-1.140714	-1.307607
C	-2.789171	-3.328964	2.753123
F	-4.233514	-1.617611	-2.461999
F	-5.197998	0.113997	-1.572315
F	-5.858862	-1.893664	-1.041621
F	-3.472029	-2.931522	3.852230
F	-1.547633	-3.666408	3.162820
F	-3.389804	-4.456667	2.306690
O	-1.273146	2.291125	0.878399
C	-1.294461	3.113474	-0.063161
C	-1.997035	4.371649	0.033234
C	-1.986752	5.228652	-1.083876
C	-2.678305	4.745549	1.209844
C	-2.652006	6.448776	-1.025978
H	-1.461070	4.928045	-1.986476
C	-3.338531	5.964927	1.259343
H	-2.674092	4.071773	2.060419
C	-3.324990	6.813695	0.143704
H	-2.649072	7.113087	-1.884436
H	-3.865344	6.262104	2.160900
H	-3.844368	7.766966	0.189051
H	-0.779431	2.863757	-0.994936

NTOB-benzaldehyde complex 79

B3LYP/6-31G(d) = -2780.351414
B3LYP-D3(BJ)/def2-TZVPP/IEFPCM(propanonitrile) = -2781.604202
B3LYP-D3(BJ)/def2-TZVPP/IEFPCM(propanonitrile)//B3LYP-D3(BJ)/6-31G(d) Free Energy (Quasiharmonic) = -2781.119017

Frequencies (Top 3 out of 213)

1. 9.2725 cm^{-1}
2. 10.3631 cm^{-1}
3. 12.8156 cm^{-1}

B3LYP/6-31G(d) Molecular Geometry in Cartesian Coordinates

B	-0.509391	0.883152	0.551396
O	-0.002183	1.140038	1.889487
N	0.732962	0.516421	-0.232598
C	1.921548	0.523261	0.640512
S	0.781036	-0.172019	-1.751602
C	1.335120	0.942097	1.992690
O	1.946446	1.127595	3.014890
H	2.814391	2.510184	0.579606
H	2.340857	-0.486279	0.741289
C	3.067557	1.501619	0.230461
C	4.414119	1.081252	0.747881
C	5.379827	0.273002	0.039601
C	6.493009	0.089774	0.906349
N	6.206160	0.758153	2.079585
C	4.958562	1.343184	1.980890
H	4.533948	1.882949	2.814729
C	7.622611	-1.216073	-0.738013
C	5.413232	-0.309768	-1.241246
H	8.462188	-0.777163	1.207186
H	6.799943	0.793654	2.893105
C	6.530896	-1.047626	-1.614424
H	8.482439	-1.797545	-1.059978
H	4.578073	-0.183517	-1.925004
C	7.619871	-0.649034	0.531687
H	6.567716	-1.501520	-2.601222
C	0.697109	-1.952014	-1.527614
O	2.099428	0.114112	-2.331920
O	-0.442186	0.262621	-2.434687
C	0.578689	-4.725802	-1.147168
C	1.863902	-2.658058	-1.218333
C	-0.522639	-2.616563	-1.675280
C	-0.569539	-3.995994	-1.485553
C	1.792928	-4.036288	-1.025229
H	2.818531	-2.145144	-1.160219
H	-1.414878	-2.068381	-1.955008
H	-1.516670	-4.515320	-1.609507
H	2.700557	-4.585877	-0.788257
C	0.505303	-6.215013	-0.908917
H	0.236649	-6.432545	0.133364
H	1.466112	-6.701060	-1.106531
H	-0.252641	-6.685636	-1.543861
H	3.086384	1.535418	-0.859746
C	-1.860765	0.021688	0.552785
C	-2.770429	-0.006099	-0.515109
C	-2.171194	-0.747965	1.685307
C	-3.921559	-0.796222	-0.465147
H	-2.565623	0.572633	-1.407919
C	-3.328851	-1.528294	1.742855
H	-1.506575	-0.726869	2.542865
C	-4.210003	-1.563066	0.662807
H	-5.108992	-2.166504	0.703646
C	-3.595731	-2.380455	2.954517
C	-4.801409	-0.873658	-1.681654
F	-4.913105	-2.646940	3.105195
F	-3.169449	-1.787396	4.091314
F	-2.961337	-3.574585	2.875354
F	-4.976804	0.336768	-2.257288
F	-6.026221	-1.370550	-1.401709
F	-4.255301	-1.678569	-2.628373
O	-0.977881	2.359442	-0.050653
C	-1.024112	3.405556	0.626788
C	-1.404074	4.677583	0.057793
C	-1.463048	5.804518	0.899462
C	-1.707731	4.796803	-1.314379
C	-1.827483	7.040290	0.376055
H	-1.224523	5.701485	1.955068
C	-2.068385	6.034410	-1.828317
H	-1.647960	3.917587	-1.947965
C	-2.129051	7.152111	-0.984744
H	-1.876395	7.912920	1.019821
H	-2.301944	6.137109	-2.883490
H	-2.412238	8.117987	-1.393847
H	-0.761539	3.352010	1.690380

NTOB-benzaldehyde complex 80

B3LYP/6-31G(d) = -2780.343135
B3LYP-D3(BJ)/def2-TZVPP/IEFPCM(propanonitrile) = -2781.604482
B3LYP-D3(BJ)/def2-TZVPP/IEFPCM(propanonitrile)//B3LYP-D3(BJ)/6-31G(d) Free Energy (Quasiharmonic) = -2781.118994

Frequencies (Top 3 out of 213)

1. 10.9665 cm-1
2. 13.0815 cm-1
3. 14.3131 cm-1

B3LYP/6-31G(d) Molecular Geometry in Cartesian Coordinates

B	-0.650957	-0.748208	1.097791
O	-0.581602	-0.826456	2.547258
N	0.747752	-0.253202	0.703491
C	1.474795	0.168224	1.914579
S	1.465935	-0.396377	-0.780829
C	0.607628	-0.430596	3.035344
O	0.915819	-0.479845	4.200184
H	1.850658	1.774641	3.248531
H	2.470951	-0.285403	1.958233
C	1.588855	1.696701	2.185637
C	2.566904	2.480022	1.356896
C	2.251183	3.512916	0.399734
C	3.480366	4.011996	-0.107427
N	4.492334	3.302861	0.510360
C	3.937580	2.399958	1.400665
H	4.575710	1.767806	2.004129
C	2.332336	5.559789	-1.510648
C	1.050242	4.064000	-0.077867
H	4.488856	5.406272	-1.434450
H	5.478251	3.483167	0.405791
C	1.099345	5.076789	-1.025717
H	2.340930	6.354284	-2.252052
H	0.090159	3.705126	0.280628
C	3.537984	5.035645	-1.059321
H	0.171044	5.493381	-1.405357
C	2.989280	-1.299179	-0.469631
O	1.844120	0.899008	-1.346563
O	0.565766	-1.264472	-1.576024
C	5.369935	-2.717972	-0.062517
C	4.210192	-0.686549	-0.745541
C	2.938499	-2.612744	0.006805
C	4.124841	-3.309189	0.210027
C	5.391071	-1.402707	-0.539983
H	4.224305	0.333367	-1.113399
H	1.982461	-3.080399	0.220036
H	4.087958	-4.329349	0.584973
H	6.345175	-0.928293	-0.755938
C	6.647794	-3.494596	0.147609
H	6.681262	-3.945121	1.146440
H	7.528657	-2.855470	0.035476
H	6.736446	-4.313186	-0.577980
O	-0.773669	-2.299052	0.676760
C	-1.209498	-2.656432	-0.444595
H	-1.688738	-1.919662	-1.090447
H	0.588873	2.132924	2.082573
C	-1.158667	-4.033460	-0.879775
C	-1.625839	-4.345985	-2.169331
C	-0.667000	-5.051290	-0.038071
C	-1.594556	-5.662551	-2.617536
H	-1.997254	-3.552614	-2.812231
C	-0.644945	-6.363632	-0.490106
H	-0.324367	-4.794603	0.959202
C	-1.105988	-6.667630	-1.778087
H	-1.948486	-5.907416	-3.614082
H	-0.273871	-7.154770	0.154371
H	-1.085410	-7.696470	-2.126780
C	-1.934752	0.053930	0.559686
C	-1.824900	1.141141	-0.318365
C	-3.218754	-0.270487	1.031705
C	-2.949354	1.881535	-0.701633
H	-0.850415	1.430686	-0.700775
C	-4.344210	0.454656	0.638639
H	-3.341393	-1.088969	1.735882
C	-4.214393	1.541086	-0.229468
H	-5.084623	2.114707	-0.525255
C	-5.711656	0.031670	1.103991
C	-2.766291	3.086847	-1.586016
F	-5.678356	-0.522221	2.335083
F	-6.576661	1.068431	1.145614
F	-1.928502	2.834560	-2.611090
F	-6.251742	-0.895575	0.273674
F	-2.236381	4.130249	-0.895000
F	-3.934975	3.518546	-2.109524

NTOB-benzaldehyde complex 81

B3LYP/6-31G(d) = -2780.351483
B3LYP-D3(BJ)/def2-TZVPP/IEFPCM(propanonitrile) = -2781.604105
B3LYP-D3(BJ)/def2-TZVPP/IEFPCM(propanonitrile)//B3LYP-D3(BJ)/6-31G(d) Free Energy (Quasiharmonic) = -2781.118989

Frequencies (Top 3 out of 213)

1. 9.3096 cm-1
2. 10.2382 cm-1
3. 13.2006 cm-1

B3LYP/6-31G(d) Molecular Geometry in Cartesian Coordinates

B	-0.512351	0.877588	0.540704
O	-0.013148	1.148302	1.879171
N	0.735928	0.509604	-0.233515
C	1.919837	0.529052	0.645865
S	0.795730	-0.186671	-1.748404
C	1.324283	0.956871	1.991226
O	1.929396	1.153697	3.015023
H	2.805664	2.518822	0.575490
H	2.342769	-0.477818	0.757561
C	3.064154	1.508871	0.234366
C	4.409760	1.096937	0.761064
C	5.382868	0.289725	0.061800
C	6.492111	0.115408	0.935429
N	6.195891	0.787859	2.103987
C	4.946287	1.366922	1.995817
H	4.514681	1.908580	2.824822
C	7.636411	-1.192973	-0.696737
C	5.425837	-0.298784	-1.216141
H	8.463426	-0.741396	1.250941
H	6.785106	0.829831	2.920517
C	6.548756	-1.033407	-1.579830
H	8.500544	-1.772089	-1.011339
H	4.593865	-0.179374	-1.905002
C	7.624242	-0.620115	0.570299
H	6.592957	-1.491664	-2.564307
C	0.723576	-1.966381	-1.517292
O	2.114689	0.105141	-2.324635
O	-0.427299	0.235688	-2.439475
C	0.623056	-4.738810	-1.122375
C	1.893422	-2.662097	-1.196228
C	-0.491310	-2.639896	-1.664113
C	-0.529818	-4.018280	-1.464586
C	1.830856	-4.039307	-0.993532
H	2.843148	-2.141175	-1.129577
H	-1.387658	-2.098873	-1.944567
H	-1.474620	-4.543636	-1.581149
H	2.739596	-4.579870	-0.740446
C	0.571179	-6.236333	-0.934949
H	1.360655	-6.583576	-0.260716
H	0.706129	-6.757333	-1.892172
H	-0.392652	-6.555452	-0.524851
H	3.088165	1.535439	-0.855907
C	-1.860327	0.010811	0.543610
C	-2.170903	-0.752272	1.680687
C	-2.763371	-0.032925	-0.529183

C	-3.322169	-1.541693	1.737774
H	-1.503950	-0.730430	2.536267
C	-3.906120	-0.835484	-0.480984
H	-2.556934	0.538227	-1.426458
C	-4.195188	-1.594889	0.651681
H	-5.081204	-2.217713	0.686332
C	-4.769833	-0.942534	-1.706669
C	-3.653154	-2.293868	2.998655
F	-5.995109	-1.441482	-1.432252
F	-4.207349	-1.763050	-2.630465
F	-4.944968	0.255040	-2.308268
F	-2.542232	-2.708652	3.645548
F	-4.410416	-3.386926	2.750392
F	-4.349771	-1.525673	3.870355
O	-0.984309	2.346101	-0.076513
C	-1.041450	3.397175	0.592423
C	-1.424401	4.662509	0.010741
C	-1.718666	4.769283	-1.364507
C	-1.495914	5.795601	0.843127
C	-2.082508	6.000778	-1.890726
H	-1.649330	3.885479	-1.990699
C	-1.863475	7.025157	0.307421
H	-1.264615	5.702133	1.901227
C	-2.155658	7.124649	-1.056380
H	-2.308920	6.093936	-2.948340
H	-1.922077	7.902470	0.943968
H	-2.441300	8.085677	-1.475081
H	-0.786363	3.353402	1.658270

NTOB-benzaldehyde complex 82

B3LYP/6-31G(d) = -2780.351514
B3LYP-D3(BJ)/def2-TZVPP/IEFPCM(propanonitrile) = -2781.604138
B3LYP-D3(BJ)/def2-TZVPP/IEFPCM(propanonitrile)//B3LYP-D3(BJ)/6-31G(d) Free Energy (Quasiharmonic) = -2781.118882

Frequencies (Top 3 out of 213)

1. 9.2828 cm^{-1}
2. 10.2183 cm^{-1}
3. 13.2022 cm^{-1}

B3LYP/6-31G(d) Molecular Geometry in Cartesian Coordinates

B	-0.509098	0.878464	0.541498
O	-0.008149	1.145589	1.880024
N	0.737763	0.510193	-0.234806
C	1.922583	0.525750	0.643390
S	0.794367	-0.184582	-1.750528
C	1.329139	0.951811	1.990260
O	1.935613	1.145509	3.013839
H	2.811273	2.514312	0.575059
H	2.343975	-0.482035	0.752594
C	3.068055	1.504430	0.232452
C	4.413418	1.089885	0.757666
C	5.384054	0.280410	0.057567
C	6.493953	0.104170	0.929966
N	6.200488	0.777688	2.098615
C	4.951944	1.359246	1.991675
H	4.522451	1.902132	2.820979
C	7.633680	-1.207159	-0.703021
C	5.424326	-0.308667	-1.220198
H	8.463889	-0.756521	1.243517
H	6.790748	0.818765	2.914435
C	6.545325	-1.045708	-1.584909
H	8.496265	-1.788153	-1.018410
H	4.591814	-0.187797	-1.908140
C	7.624159	-0.633795	0.563807
H	6.587451	-1.504416	-2.569267
C	0.714512	-1.964049	-1.519907
O	2.114456	0.102519	-2.326494
O	-0.427046	0.243564	-2.440895
C	0.603178	-4.737290	-1.132847
C	1.881568	-2.665194	-1.200020
C	-0.501624	-2.633271	-1.674642
C	-0.544999	-4.012540	-1.481496
C	1.814125	-4.042986	-1.003635
H	2.833990	-2.148811	-1.136606
H	-1.393787	-2.089380	-1.962765
H	-1.489281	-4.535535	-1.611675
H	2.722013	-4.588684	-0.758704
C	0.534446	-6.226256	-0.891469
H	-0.239544	-6.696826	-1.506604
H	0.292815	-6.442923	0.157627
H	1.489676	-6.713271	-1.112789
H	3.091314	1.532325	-0.857819
C	-1.858037	0.013204	0.543549
C	-2.762111	-0.026736	-0.528524
C	-2.168081	-0.753019	1.678622
C	-3.905317	-0.828691	-0.481681
H	-2.556084	0.546894	-1.424318
C	-3.319812	-1.541884	1.734406
H	-1.500293	-0.734160	2.533644
C	-4.193858	-1.591295	0.648976
H	-5.080200	-2.213696	0.682562
C	-3.649577	-2.297759	2.993374
C	-4.770114	-0.931458	-1.706961
F	-2.537941	-2.719330	3.634847
F	-4.411735	-3.386989	2.743479
F	-4.340129	-1.530382	3.870551
F	-4.945961	0.268248	-2.304079
F	-5.995035	-1.431637	-1.433325
F	-4.208266	-1.748510	-2.634287
O	-0.979625	2.348789	-0.072155
C	-1.034478	3.398618	0.598931
C	-1.416244	4.665632	0.020159
C	-1.711645	4.775551	-1.354601
C	-1.485436	5.797155	0.854875
C	-2.074310	6.008585	-1.878017
H	-1.644040	3.892917	-1.982632
C	-1.851858	7.028268	0.321973
H	-1.253263	5.701264	1.912566
C	-2.145184	7.130878	-1.041353
H	-2.301547	6.104170	-2.935237
H	-1.908688	7.904386	0.960325
H	-2.429921	8.093125	-1.457864
H	-0.778228	3.352359	1.664393

NTOB-benzaldehyde complex 83

B3LYP/6-31G(d) = -2780.342625
B3LYP-D3(BJ)/def2-TZVPP/IEFPCM(propanonitrile) = -2781.604707
B3LYP-D3(BJ)/def2-TZVPP/IEFPCM(propanonitrile)//B3LYP-D3(BJ)/6-31G(d) Free Energy (Quasiharmonic) = -2781.118821

Frequencies (Top 3 out of 213)

1. 8.5010 cm^{-1}
2. 10.6772 cm^{-1}
3. 12.3222 cm^{-1}

B3LYP/6-31G(d) Molecular Geometry in Cartesian Coordinates

B	0.726947	-0.028281	0.906511
O	0.676144	-0.277847	2.339510
N	-0.301981	1.097936	0.710237
C	-0.794332	1.546366	2.025295
S	-0.225364	2.139796	-0.584373
C	-0.028194	0.648488	3.003803
O	-0.082835	0.733218	4.207805

H	-2.875335	1.917763	1.518063
H	-0.508485	2.589896	2.207880
C	-2.321285	1.396156	2.303403
C	-2.850189	-0.008391	2.465833
C	-3.654373	-0.774431	1.541674
C	-3.939744	-2.026064	2.162058
N	-3.337087	-2.008560	3.400623
C	-2.695681	-0.800788	3.580871
H	-2.156033	-0.594818	4.493773
C	-5.237370	-2.730392	0.286539
C	-4.195299	-0.524169	0.267981
H	-4.933646	-3.953887	2.046363
H	-3.359535	-2.756519	4.076130
C	-4.976507	-1.496570	-0.344950
H	-5.862542	-3.466853	-0.211736
H	-4.012576	0.422038	-0.230369
C	-4.721664	-3.011096	1.547862
H	-5.400162	-1.302703	-1.326964
C	-1.901372	2.589972	-1.051213
O	0.321099	1.327029	-1.687509
O	0.424627	3.401223	-0.192772
C	-4.448997	3.382081	-1.910760
C	-2.564582	1.841731	-2.024471
C	-2.481205	3.745546	-0.521948
C	-3.752003	4.126062	-0.947252
C	-3.830613	2.244098	-2.447690
H	-2.082874	0.972377	-2.457978
H	-1.930463	4.349573	0.191244
H	-4.202835	5.026170	-0.536838
H	-4.343434	1.668632	-3.214534
C	-5.833547	3.792913	-2.350545
H	-5.951012	4.881422	-2.339010
H	-6.597079	3.376108	-1.680518
H	-6.055950	3.436296	-3.361256
H	-2.497331	1.954816	3.230363
C	2.266002	0.022272	0.421611
C	3.006352	-1.171040	0.392308
C	2.969543	1.213030	0.176272
C	4.372089	-1.186377	0.097224
H	2.521526	-2.115738	0.622730
C	4.337664	1.203648	-0.106524
H	2.455047	2.167653	0.197097
C	5.047809	0.004500	-0.157282
H	6.106849	0.000093	-0.386456
C	5.072639	2.504479	-0.300603
C	5.092939	-2.502113	-0.006754
F	4.288378	3.453940	-0.848151
F	6.152282	2.354329	-1.104200
F	5.528374	2.997014	0.875667
F	6.425152	-2.371560	0.168012
F	4.910603	-3.073561	-1.224976
F	4.647484	-3.395302	0.905591
O	0.019856	-1.321689	0.223109
C	0.176742	-1.605538	-0.986968
C	-0.592154	-2.633313	-1.644031
C	-0.255275	-2.971596	-2.969986
C	-1.657028	-3.287079	-0.990599
C	-0.963544	-3.968554	-3.630545
H	0.561602	-2.453506	-3.465703
C	-2.364522	-4.274419	-1.662519
H	-1.923570	-2.997635	0.020290
C	-2.015167	-4.617638	-2.974983
H	-0.704184	-4.239197	-4.649330
H	-3.195433	-4.768328	-1.169659
H	-2.572223	-5.393496	-3.493316
H	0.919872	-1.050980	-1.566177

NTOB-benzaldehyde complex 84

B3LYP/6-31G(d) = -2780.350608

B3LYP-D3(BJ)/def2-TZVPP/IEFPCM(propanonitrile) = -2781.603746
B3LYP-D3(BJ)/def2-TZVPP/IEFPCM(propanonitrile)//B3LYP-D3(BJ)/6-31G(d) Free Energy (Quasiharmonic) = -2781.118593

Frequencies (Top 3 out of 213)

1. 9.2561 cm^{-1}
2. 10.1043 cm^{-1}
3. 14.4346 cm^{-1}

B3LYP/6-31G(d) Molecular Geometry in Cartesian Coordinates

B	-0.467847	0.778686	-0.956509
O	0.159702	0.793147	-2.260327
N	0.680399	0.408585	-0.021443
C	1.942726	0.285597	-0.780157
S	0.690616	0.460257	1.640945
C	1.499693	0.615382	-2.210039
O	2.213709	0.672412	-3.178554
H	1.983545	-1.828059	-1.286924
H	2.673854	1.029544	-0.447422
C	2.603192	-1.121018	-0.723023
C	4.018167	-1.140363	-1.217699
C	5.178341	-0.636402	-0.520526
C	6.304725	-0.867908	-1.358394
N	5.838822	-1.481422	-2.503866
C	4.469485	-1.634408	-2.414758
H	3.911110	-2.075351	-3.228749
C	7.766644	0.106615	0.253467
C	5.376336	-0.012501	0.724987
H	8.451446	-0.690842	-1.637539
H	6.401023	-1.741669	-3.298689
C	6.664944	0.351317	1.098315
H	8.762100	0.402774	0.573337
H	4.533923	0.198256	1.379419
C	7.602243	-0.504888	-0.984654
H	6.828460	0.836290	2.057031
C	0.507704	-1.198407	2.307589
O	-0.518851	1.210111	2.025914
O	2.017433	0.949452	2.050816
C	0.231269	-3.763767	3.401112
C	1.634513	-1.871925	2.783133
C	-0.761229	-1.776607	2.398436
C	-0.886741	-3.052680	2.940390
C	1.487775	-3.149123	3.322046
H	2.606698	-1.392427	2.752018
H	-1.642989	-1.235224	2.078190
H	-1.875743	-3.497804	3.015351
H	2.364987	-3.670941	3.696107
C	0.081782	-5.158605	3.958854
H	0.918450	-5.421031	4.613866
H	0.050907	-5.902122	3.151461
H	-0.846284	-5.263764	4.530723
O	-0.909480	2.370837	-0.735172
C	-0.273694	3.213872	-0.071027
H	0.668357	2.924714	0.404915
H	2.578720	-1.455401	0.318819
C	-0.721570	4.578798	0.082147
C	0.060912	5.457859	0.854005
C	-1.912775	5.030417	-0.521086
C	-0.342364	6.778684	1.019912
H	0.973339	5.096325	1.321044
C	-2.307515	6.349817	-0.351331
H	-2.502934	4.336405	-1.110395
C	-1.523405	7.221189	0.417206
H	0.256048	7.460948	1.615471
H	-3.223094	6.707301	-0.812426
H	-1.838510	8.252921	0.546864
C	-1.866244	0.000099	-0.921354
C	-2.815253	0.148959	0.099863
C	-2.168350	-0.898718	-1.956392

C	-3.997723	-0.596733	0.106287
H	-2.625172	0.842485	0.912506
C	-3.356837	-1.633155	-1.961243
H	-1.470893	-1.013876	-2.779803
C	-4.277841	-1.493299	-0.923191
H	-5.199525	-2.062750	-0.922946
C	-3.615940	-2.626877	-3.061873
C	-4.923575	-0.471528	1.283394
F	-3.035120	-3.822898	-2.800272
F	-4.936946	-2.862768	-3.230125
F	-6.139578	-1.008022	1.047061
F	-3.124307	-2.206239	-4.247326
F	-5.110621	0.816599	1.646503
F	-4.415590	-1.108918	2.372443

NTOB-benzaldehyde complex 85

B3LYP/6-31G(d) = -2780.350884
B3LYP-D3(BJ)/def2-TZVPP/IEFPCM(propanonitrile) = -2781.602737
B3LYP-D3(BJ)/def2-TZVPP/IEFPCM(propanonitrile)//B3LYP-D3(BJ)/6-31G(d) Free Energy (Quasiharmonic) = -2781.118129

Frequencies (Top 3 out of 213)

1. 8.8928 cm^{-1}
2. 10.5128 cm^{-1}
3. 11.8537 cm^{-1}

B3LYP/6-31G(d) Molecular Geometry in Cartesian Coordinates

B	0.569730	0.749697	0.782403
O	0.092678	1.050295	2.109667
N	-0.660211	0.153564	0.089346
C	-1.853054	0.369046	0.943128
S	-0.886585	0.068352	-1.555094
C	-1.242813	0.894144	2.246228
O	-1.834149	1.118736	3.271200
H	-2.146737	-1.601174	1.804412
H	-2.491480	1.151678	0.517805
C	-2.709636	-0.904511	1.172503
C	-4.061437	-0.626398	1.760503
C	-5.209293	-0.094114	1.063916
C	-6.269180	-0.003242	2.008855
N	-5.777293	-0.460768	3.214812
C	-4.454170	-0.824260	3.059331
H	-3.879143	-1.182224	3.901756
C	-7.742211	0.867037	0.348337
C	-5.445392	0.315728	-0.260982
H	-8.337037	0.533043	2.401636
H	-6.286919	-0.484239	4.083831
C	-6.705714	0.790880	-0.604216
H	-8.716922	1.243423	0.049417
H	-4.650859	0.277170	-1.001847
C	-7.538757	0.471841	1.666036
H	-6.897250	1.113949	-1.624065
C	-1.143175	-1.668154	-1.939007
O	0.391199	0.480198	-2.174776
O	-2.119480	0.776333	-1.931190
C	-1.497273	-4.346031	-2.680331
C	-0.293819	-2.647203	-1.415744
C	-2.161587	-2.007687	-2.829612
C	-2.330603	-3.344090	-3.191951
C	-0.477305	-3.974457	-1.789871
H	0.494908	-2.379626	-0.720879
H	-2.811651	-1.234836	-3.224640
H	-3.125719	-3.610020	-3.883881
H	0.183604	-4.736230	-1.383717
C	-1.699615	-5.794685	-3.054444
H	-2.210077	-6.340957	-2.250495
H	-0.743229	-6.299128	-3.230687
H	-2.308778	-5.894220	-3.958021
O	0.736088	2.273968	0.133526
C	1.529370	2.599940	-0.774013
H	2.302439	1.893450	-1.088752
H	-2.832807	-1.398158	0.201944
C	1.484774	3.896420	-1.410327
C	2.435727	4.189219	-2.405349
C	0.515439	4.854336	-1.051097
C	2.420391	5.429393	-3.034720
H	3.174071	3.440501	-2.680325
C	0.506784	6.089579	-1.683537
H	-0.212630	4.608678	-0.285225
C	1.457553	6.375902	-2.672499
H	3.150408	5.659672	-3.804416
H	-0.237412	6.832785	-1.414315
H	1.444539	7.344359	-3.164957
C	2.004378	0.023184	0.805940
C	2.695757	-0.010131	2.032295
C	2.624667	-0.597150	-0.290520
C	3.937343	-0.634370	2.157022
H	2.242318	0.447056	2.905114
C	3.866868	-1.230997	-0.164886
H	2.138266	-0.588155	-1.260590
C	4.532132	-1.254989	1.057022
H	5.490804	-1.750095	1.154168
C	4.446232	-1.920276	-1.370312
C	4.669427	-0.591631	3.472587
F	3.720644	-3.011665	-1.713108
F	4.452326	-1.106331	-2.451952
F	5.422155	0.529194	3.584705
F	5.715955	-2.332623	-1.168589
F	5.509276	-1.642068	3.615769
F	3.822423	-0.601873	4.522948

NTOB-benzaldehyde complex 86

B3LYP/6-31G(d) = -2780.35087
B3LYP-D3(BJ)/def2-TZVPP/IEFPCM(propanonitrile) = -2781.602724
B3LYP-D3(BJ)/def2-TZVPP/IEFPCM(propanonitrile)//B3LYP-D3(BJ)/6-31G(d) Free Energy (Quasiharmonic) = -2781.118106

Frequencies (Top 3 out of 213)

1. 9.0938 cm^{-1}
2. 10.4166 cm^{-1}
3. 11.2521 cm^{-1}

B3LYP/6-31G(d) Molecular Geometry in Cartesian Coordinates

B	-0.566248	0.720816	-0.805733
O	-0.087666	0.985537	-2.140029
N	0.662229	0.142578	-0.095678
C	1.856804	0.338239	-0.952295
S	0.885785	0.102075	1.550880
C	1.247966	0.825969	-2.270513
O	1.840466	1.021653	-3.300745
H	2.160278	-1.652998	-1.760176
H	2.491623	1.134224	-0.546771
C	2.718311	-0.938011	-1.144626
C	4.071633	-0.670887	-1.734334
C	5.216403	-0.121941	-1.045638
C	6.279306	-0.050009	-1.988807
N	5.792035	-0.534208	-3.186213
C	4.468940	-0.896256	-3.027297
H	3.897180	-1.273027	-3.863706
C	7.745572	0.858016	-0.342609
C	5.447448	0.316718	0.270896
H	8.347749	0.480600	-2.386233
H	6.304667	-0.576019	-4.052775
C	6.705989	0.800944	0.607925

H	8.718769	1.242142	-0.048637
H	4.650479	0.292976	1.009752
C	7.547087	0.434181	-1.652139
H	6.893698	1.146194	1.621197
C	1.142721	-1.623711	1.979407
O	-0.392774	0.530242	2.157846
O	2.117587	0.820312	1.910830
C	1.493734	-4.281571	2.790377
C	0.320024	-2.621214	1.449035
C	2.133480	-1.934763	2.910939
C	2.300809	-3.261212	3.308156
C	0.502023	-3.938634	1.857814
H	-0.445163	-2.374660	0.720844
H	2.764515	-1.148155	3.309760
H	3.074226	-3.505120	4.032071
H	-0.137017	-4.715470	1.445225
C	1.696919	-5.719963	3.201818
H	2.262275	-6.271079	2.438882
H	0.740250	-6.237558	3.331680
H	2.254353	-5.793557	4.140643
O	-0.731158	2.263313	-0.197432
C	-1.525462	2.613049	0.700271
H	-2.299508	1.915212	1.031645
H	2.839397	-1.405415	-0.160924
C	-1.480964	3.925274	1.303524
C	-2.433814	4.244062	2.288694
C	-0.509925	4.872868	0.922244
C	-2.418650	5.499816	2.886412
H	-3.173538	3.503261	2.580890
C	-0.501437	6.123749	1.523172
H	0.219607	4.607156	0.164500
C	-1.454096	6.435943	2.502443
H	-3.150141	5.750208	3.648393
H	0.244096	6.859190	1.236860
H	-1.441199	7.416562	2.970221
C	-2.003492	-0.000540	-0.810518
C	-2.623415	-0.597085	0.299513
C	-2.702078	-0.046482	-2.032279
C	-3.876888	-1.211873	0.193133
H	-2.127396	-0.585097	1.264495
C	-3.951091	-0.659151	-2.139824
H	-2.254036	0.401701	-2.912533
C	-4.549946	-1.247292	-1.024473
H	-5.520955	-1.721024	-1.104952
C	-4.632964	-0.741968	-3.480285
C	-4.500644	-1.797061	1.431213
F	-5.977854	-0.820976	-3.355394
F	-4.359823	0.331972	-4.251641
F	-3.620608	-2.562165	2.116606
F	-4.238981	-1.835623	-4.173377
F	-5.574714	-2.566316	1.152179
F	-4.915668	-0.827980	2.283363

NTOB-benzaldehyde complex 87

B3LYP/6-31G(d) = -2780.347603
B3LYP-D3(BJ)/def2-TZVPP/IEFPCM(propanonitrile) = -2781.602513
B3LYP-D3(BJ)/def2-TZVPP/IEFPCM(propanonitrile)//B3LYP-D3(BJ)/6-31G(d) Free Energy (Quasiharmonic) = -2781.117826

Frequencies (Top 3 out of 213)

1. 6.5251 cm^{-1}
2. 10.0331 cm^{-1}
3. 11.9514 cm^{-1}

B3LYP/6-31G(d) Molecular Geometry in Cartesian Coordinates

B	0.760641	0.074025	-0.535546
O	0.381791	0.399906	-1.901835
N	-0.546307	0.219086	0.228281
C	-1.585354	0.790848	-0.646618
S	-0.988187	-0.436126	1.688049
C	-0.926728	0.739023	-2.024918
O	-1.441169	0.995945	-3.084123
H	-1.232800	2.940363	-0.657177
H	-2.483051	0.161890	-0.668049
C	-1.999774	2.251857	-0.282791
C	-3.362105	2.626329	-0.789596
C	-4.597480	2.519416	-0.049373
C	-5.646194	2.937120	-0.915117
N	-5.063995	3.276924	-2.119586
C	-3.697969	3.086502	-2.038884
H	-3.064546	3.269902	-2.894866
C	-7.270958	2.554450	0.788073
C	-4.917200	2.119557	1.261655
H	-7.773531	3.285689	-1.185072
H	-5.554784	3.613278	-2.933024
C	-6.247668	2.139004	1.664930
H	-8.301236	2.561577	1.133738
H	-4.134558	1.807477	1.948820
C	-6.984854	2.961061	-0.510702
H	-6.505703	1.835623	2.676103
C	-2.049534	-1.837365	1.294263
O	-1.830337	0.527201	2.406987
O	0.235639	-0.970383	2.298303
C	-3.685087	-4.026297	0.659868
C	-3.431033	-1.659834	1.183389
C	-1.478212	-3.097659	1.105265
C	-2.296833	-4.179408	0.790019
C	-4.234332	-2.752990	0.860038
H	-3.875085	-0.686120	1.361735
H	-0.408200	-3.224698	1.226158
H	-1.852266	-5.162152	0.650196
H	-5.308512	-2.611633	0.769875
C	-4.569563	-5.210115	0.348646
H	-4.932253	-5.682826	1.271001
H	-4.031224	-5.975879	-0.219456
H	-5.449631	-4.911450	-0.230191
O	1.154413	-1.539436	-0.560292
C	1.302157	-2.190657	-1.614925
H	1.080274	-1.697113	-2.568430
H	-1.977731	2.320100	0.807362
C	1.760565	-3.560430	-1.619030
C	2.140833	-4.205546	-0.423564
C	1.833208	-4.243360	-2.847863
C	2.581675	-5.520807	-0.467112
H	2.095672	-3.656804	0.511897
C	2.271944	-5.562816	-2.881625
H	1.543217	-3.735275	-3.764047
C	2.644555	-6.197736	-1.693030
H	2.882071	-6.023711	0.446921
H	2.327942	-6.095156	-3.825950
H	2.991474	-7.226977	-1.720188
C	2.127153	0.774109	-0.072681
C	2.703989	0.592103	1.195715
C	2.806778	1.599801	-0.981107
C	3.909579	1.210201	1.534249
H	2.205162	-0.038947	1.923219
C	4.010885	2.223517	-0.640975
H	2.394524	1.753727	-1.973266
C	4.570815	2.031434	0.620115
H	5.511319	2.501193	0.882202
C	4.676492	3.138186	-1.633055
C	4.476583	1.023959	2.916794
F	4.113283	4.368965	-1.638505
F	5.991189	3.307965	-1.366927
F	4.251639	-0.222921	3.388017
F	4.579024	2.662215	-2.896512
F	3.929341	1.884048	3.805023
F	5.814710	1.229409	2.945120

NTOB-benzaldehyde complex 88

B3LYP/6-31G(d) = -2780.34768
B3LYP-D3(BJ)/def2-TZVPP/IEFPCM(propanonitrile) = -2781.602525
B3LYP-D3(BJ)/def2-TZVPP/IEFPCM(propanonitrile)//B3LYP-D3(BJ)/6-31G(d) Free Energy (Quasiharmonic) = -2781.117768

Frequencies (Top 3 out of 213)

1. 6.6441 cm^{-1}
2. 9.9894 cm^{-1}
3. 12.2646 cm^{-1}

B3LYP/6-31G(d) Molecular Geometry in Cartesian Coordinates

B	-0.756378	-0.086422	-0.531899
O	-0.379456	-0.412305	-1.898639
N	0.552872	-0.225659	0.228893
C	1.592827	-0.792302	-0.648160
S	0.993649	0.429609	1.688993
C	0.930645	-0.744213	-2.024871
O	1.443897	-0.998461	-3.085299
H	1.251363	-2.943554	-0.658474
H	2.487289	-0.158820	-0.671778
C	2.015469	-2.251223	-0.285256
C	3.378850	-2.618677	-0.794423
C	4.614591	-2.507889	-0.055377
C	5.664129	-2.919256	-0.923155
N	5.082038	-3.259181	-2.127639
C	3.715292	-3.074832	-2.045044
H	3.081702	-3.259183	-2.900695
C	7.289173	-2.532929	0.778941
C	4.934083	-2.109294	1.256095
H	7.792659	-3.257955	-1.196262
H	5.573401	-3.591450	-2.942410
C	6.265085	-2.123734	1.657807
H	8.319871	-2.536228	1.123408
H	4.150902	-1.801973	1.944792
C	7.003350	-2.938158	-0.520325
H	6.522959	-1.821240	2.669285
C	2.049538	1.834984	1.295279
O	1.839930	-0.531245	2.406405
O	-0.231564	0.958807	2.300738
C	3.676335	4.030474	0.661071
C	1.473339	3.093274	1.107696
C	3.431577	1.662685	1.183032
C	4.230507	2.759079	0.859794
C	2.287628	4.178305	0.792540
H	0.402953	3.216189	1.229550
H	3.879412	0.690490	1.360194
H	5.305125	2.621801	0.768560
H	1.839268	5.159477	0.653793
C	4.556086	5.217835	0.349954
H	5.437258	4.922758	-0.229040
H	4.916998	5.691826	1.272350
H	4.014649	5.981565	-0.217946
O	-1.155827	1.526213	-0.557290
C	-1.308425	2.175590	-1.612341
H	-1.087491	1.681492	-2.565787
H	1.995642	-2.319799	0.804904
C	-1.771170	3.543895	-1.617264
C	-2.150729	4.189544	-0.421872
C	-1.849030	4.224737	-2.846937
C	-2.596031	5.503265	-0.466322
H	-2.101655	3.642351	0.514289
C	-2.292208	5.542680	-2.881628
H	-1.559623	3.716217	-3.763061
C	-2.664078	6.178142	-1.693094
H	-2.895987	6.006520	0.447662
H	-2.352286	6.073388	-3.826619
H	-3.014525	7.206167	-1.720963
C	-2.120331	-0.789244	-0.065709
C	-2.787313	-1.636480	-0.963705
C	-2.703671	-0.594407	1.197730
C	-3.986015	-2.267457	-0.618435
H	-2.362388	-1.812448	-1.946753
C	-3.902368	-1.222511	1.542484
H	-2.212404	0.049705	1.918790
C	-4.552296	-2.063026	0.637962
H	-5.482498	-2.548655	0.908243
C	-4.470356	-1.032377	2.924006
C	-4.697962	-3.115508	-1.636858
F	-3.921866	-1.887692	3.816074
F	-4.249018	0.216945	3.390218
F	-5.541049	-4.001963	-1.062644
F	-5.808053	-1.241245	2.952142
F	-3.832294	-3.815754	-2.403137
F	-5.440879	-2.359878	-2.483563

NTOB-benzaldehyde complex 89

B3LYP/6-31G(d) = -2780.34768
B3LYP-D3(BJ)/def2-TZVPP/IEFPCM(propanonitrile) = -2781.602523
B3LYP-D3(BJ)/def2-TZVPP/IEFPCM(propanonitrile)//B3LYP-D3(BJ)/6-31G(d) Free Energy (Quasiharmonic) = -2781.117768

Frequencies (Top 3 out of 213)

1. 6.6414 cm^{-1}
2. 9.9869 cm^{-1}
3. 12.2583 cm^{-1}

B3LYP/6-31G(d) Molecular Geometry in Cartesian Coordinates

B	-0.756363	-0.086447	-0.531846
O	-0.379478	-0.412287	-1.898602
N	0.552909	-0.225697	0.228902
C	1.592876	-0.792216	-0.648219
S	0.993718	0.429535	1.689011
C	0.930623	-0.744157	-2.024895
O	1.443834	-0.998384	-3.085349
H	1.251644	-2.943501	-0.658536
H	2.487270	-0.158638	-0.671878
C	2.015697	-2.251090	-0.285356
C	3.379092	-2.618404	-0.794593
C	4.614838	-2.507663	-0.055547
C	5.664382	-2.918887	-0.923386
N	5.082290	-3.258680	-2.127907
C	3.715537	-3.074393	-2.045274
H	3.081942	-3.258662	-2.900939
C	7.289435	-2.532705	0.778735
C	4.934336	-2.109208	1.255967
H	7.792921	-3.257477	-1.196561
H	5.573653	-3.590847	-2.942718
C	6.265344	-2.123648	1.657661
H	8.320137	-2.536009	1.123188
H	4.151155	-1.801988	1.944709
C	7.003609	-2.937790	-0.520576
H	6.523219	-1.821259	2.669170
C	2.049517	1.834984	1.295325
O	1.840081	-0.531313	2.406335
O	-0.231495	0.958638	2.300837
C	3.676163	4.030600	0.661171
C	3.431551	1.662750	1.182926
C	1.473249	3.093271	1.107929
C	2.287462	4.178365	0.792795
C	4.230405	2.759208	0.859710
H	3.879443	0.690557	1.359958
H	0.402870	3.216122	1.229909

H	1.839050	5.159533	0.654185
H	5.305019	2.621982	0.768351
C	4.555832	5.218041	0.350125
H	5.437208	4.923014	-0.228580
H	4.916414	5.692215	1.272557
H	4.014437	5.981616	-0.218024
O	-1.155896	1.526189	-0.557184
C	-1.308552	2.175569	-1.612225
H	-1.087531	1.681518	-2.565675
H	1.995937	-2.319678	0.804804
C	-1.771482	3.543817	-1.617166
C	-2.151212	4.189423	-0.421806
C	-1.849349	4.224643	-2.846848
C	-2.596689	5.503084	-0.466295
H	-2.102135	3.642244	0.514359
C	-2.292699	5.542528	-2.881579
H	-1.559811	3.716155	-3.762950
C	-2.664738	6.177946	-1.693076
H	-2.896779	6.006302	0.447665
H	-2.352781	6.073221	-3.826577
H	-3.015321	7.205924	-1.720974
C	-2.120288	-0.789327	-0.065654
C	-2.787263	-1.636548	-0.963672
C	-2.703619	-0.594550	1.197797
C	-3.985941	-2.267574	-0.618407
H	-2.362349	-1.812471	-1.946732
C	-3.902293	-1.222702	1.542547
H	-2.212358	0.049547	1.918875
C	-4.552209	-2.063206	0.638008
H	-5.482388	-2.548878	0.908290
C	-4.470279	-1.032589	2.924073
C	-4.697880	-3.115612	-1.636846
F	-4.249087	0.216781	3.390229
F	-5.807949	-1.241620	2.952246
F	-5.440880	-2.359979	-2.483477
F	-3.921660	-1.887798	3.816160
F	-5.540889	-4.002148	-1.062643
F	-3.832204	-3.815763	-2.403201

NTOB-benzaldehyde complex 90

B3LYP/6-31G(d) = -2780.346851
B3LYP-D3(BJ)/def2-TZVPP/IEFPCM(propanonitrile) = -2781.601968
B3LYP-D3(BJ)/def2-TZVPP/IEFPCM(propanonitrile)//B3LYP-D3(BJ)/6-31G(d) Free Energy (Quasiharmonic) = -2781.117337

Frequencies (Top 3 out of 213)

1. 6.7513 cm^{-1}
2. 10.3500 cm^{-1}
3. 12.3540 cm^{-1}

B3LYP/6-31G(d) Molecular Geometry in Cartesian Coordinates

B	0.569598	0.800999	0.895858
O	0.021327	0.941893	2.232215
N	-0.634925	0.321730	0.080969
C	-1.850490	0.285876	0.923038
S	-0.743893	0.290888	-1.572571
C	-1.307244	0.733914	2.285308
O	-1.963478	0.856551	3.291031
H	-2.564984	-1.387162	2.104449
H	-2.577941	1.030422	0.581099
C	-2.572087	-1.099973	1.045945
C	-3.987477	-1.112097	0.544098
C	-5.110881	-0.399397	1.109204
C	-6.250838	-0.719521	0.321272
N	-5.832255	-1.588622	-0.666043
C	-4.476095	-1.819008	-0.525206
H	-3.957103	-2.477958	-1.207506
C	-7.639026	0.659067	1.682601
C	-5.263764	0.466691	2.207848
H	-8.380310	-0.453456	-0.018782
H	-6.420788	-1.993376	-1.376647
C	-6.523701	0.985768	2.480989
H	-8.611053	1.081534	1.923166
H	-4.409628	0.719583	2.830234
C	-7.519487	-0.197801	0.594211
H	-6.654932	1.654474	3.327522
C	-0.822998	-1.427940	-2.079325
O	0.527368	0.848831	-2.069068
O	-2.005807	0.931058	-1.977243
C	-0.873826	-4.067756	-3.010816
C	0.117297	-2.345701	-1.601052
C	-1.779222	-1.805425	-3.020372
C	-1.796257	-3.124257	-3.478836
C	0.080180	-3.656328	-2.065198
H	0.863012	-2.040665	-0.874151
H	-2.497138	-1.075876	-3.378839
H	-2.538277	-3.421826	-4.215713
H	0.806937	-4.372940	-1.690521
C	-0.894911	-5.494674	-3.503846
H	-1.672725	-5.647649	-4.257862
H	-1.081336	-6.195933	-2.681183
H	0.066914	-5.773112	-3.950946
O	0.976067	2.356943	0.503135
C	0.308596	3.132651	-0.212223
H	-0.648402	2.798020	-0.625203
H	-1.976957	-1.847922	0.513927
C	0.740294	4.478020	-0.507561
C	1.948871	4.990401	0.006667
C	-0.077350	5.276246	-1.329885
C	2.325877	6.289689	-0.301124
H	2.565929	4.358233	0.636594
C	0.308701	6.577337	-1.634043
H	-1.002699	4.867007	-1.726905
C	1.507016	7.080468	-1.119225
H	3.254398	6.693873	0.090161
H	-0.316330	7.197478	-2.268857
H	1.808605	8.096876	-1.356974
C	1.970067	0.021825	0.858319
C	2.901837	0.128215	-0.187126
C	2.302275	-0.813381	1.936619
C	4.110258	-0.572666	-0.155062
H	2.682025	0.760475	-1.039398
C	3.506915	-1.521651	1.967303
H	1.620531	-0.894827	2.777101
C	4.420224	-1.404315	0.920917
H	5.363234	-1.937346	0.951037
C	3.796741	-2.453191	3.113566
C	5.061519	-0.468631	-1.316867
F	3.246567	-3.675650	2.911149
F	5.123767	-2.646452	3.286564
F	6.337031	-0.733154	-0.952350
F	3.297885	-1.990249	4.279703
F	5.047346	0.764089	-1.871415
F	4.746701	-1.343673	-2.301410

NTOB-benzaldehyde complex 91

B3LYP/6-31G(d) = -2780.350517
B3LYP-D3(BJ)/def2-TZVPP/IEFPCM(propanonitrile) = -2781.602776
B3LYP-D3(BJ)/def2-TZVPP/IEFPCM(propanonitrile)//B3LYP-D3(BJ)/6-31G(d) Free Energy (Quasiharmonic) = -2781.117315

Frequencies (Top 3 out of 213)

1. 8.9542 cm^{-1}
2. 10.7089 cm^{-1}
3. 13.4055 cm^{-1}

B3LYP/6-31G(d) Molecular Geometry in Cartesian Coordinates

B	-0.600447	0.893927	0.568026
O	-0.022435	1.188775	1.871546
N	0.616965	0.742330	-0.327406
C	1.858766	0.814154	0.466423
S	0.607511	0.226595	-1.912215
C	1.329267	1.179108	1.857660
O	2.000875	1.441560	2.824552
H	3.101418	2.558079	0.804245
H	2.320876	-0.179682	0.552949
C	2.947836	1.835458	-0.005932
C	4.269819	1.217461	-0.361609
C	5.264968	0.713117	0.557192
C	6.361017	0.249536	-0.222555
N	6.035205	0.461727	-1.547545
C	4.782032	1.042064	-1.623876
H	4.333885	1.277615	-2.578349
C	7.551314	-0.387233	1.742307
C	5.335734	0.611819	1.958848
H	8.338792	-0.649802	-0.255969
H	6.623808	0.246915	-2.336855
C	6.475765	0.063968	2.534772
H	8.429277	-0.810301	2.223335
H	4.506241	0.948593	2.574665
C	7.509421	-0.301734	0.354985
H	6.541673	-0.020828	3.616216
C	0.838468	-1.553110	-1.879880
O	1.779807	0.797900	-2.585565
O	-0.741538	0.505000	-2.419461
C	1.211101	-4.328161	-1.784885
C	2.126932	-2.072120	-1.720164
C	-0.263374	-2.400894	-2.014725
C	-0.065804	-3.779361	-1.969054
C	2.300558	-3.453825	-1.668253
H	2.981709	-1.405824	-1.660967
H	-1.254696	-1.991046	-2.170367
H	-0.921406	-4.440470	-2.083193
H	3.301944	-3.858803	-1.544303
C	1.404451	-5.823239	-1.698903
H	1.270059	-6.177186	-0.668149
H	2.410334	-6.117062	-2.015797
H	0.680042	-6.357726	-2.322304
H	2.547530	2.377071	-0.864394
C	-1.788497	-0.180982	0.647387
C	-2.770056	-0.334349	-0.343219
C	-1.865102	-1.025842	1.766510
C	-3.760504	-1.314047	-0.236676
H	-2.745488	0.298106	-1.222555
C	-2.863960	-1.995939	1.882537
H	-1.142465	-0.916085	2.568606
C	-3.815976	-2.152165	0.875613
H	-4.591311	-2.904124	0.961333
C	-2.876518	-2.916700	3.073236
C	-4.715576	-1.502507	-1.381967
F	-4.112825	-3.406475	3.320348
F	-2.455056	-2.294304	4.195909
F	-2.062636	-3.983487	2.888649
F	-5.793628	-2.241203	-1.040524
F	-4.113470	-2.138491	-2.420226
F	-5.167920	-0.322945	-1.863115
O	-1.334973	2.281694	0.068720
C	-1.471948	3.297936	0.779779
C	-2.088192	4.505217	0.283232
C	-2.527950	4.596709	-1.053996
C	-2.240375	5.597195	1.158786
C	-3.115983	5.772157	-1.499199
H	-2.391757	3.746486	-1.714918
C	-2.832893	6.770185	0.704086
H	-1.894785	5.516218	2.186291
C	-3.268970	6.854568	-0.621834
H	-3.455988	5.853963	-2.526909
H	-2.954894	7.615477	1.374061
H	-3.730497	7.771728	-0.977227
H	-1.106137	3.267184	1.813060

NTOB-benzaldehyde complex 92

B3LYP/6-31G(d) = -2780.3506
B3LYP-D3(BJ)/def2-TZVPP/IEFPCM(propanonitrile) = -2781.602757
B3LYP-D3(BJ)/def2-TZVPP/IEFPCM(propanonitrile)//B3LYP-D3(BJ)/6-31G(d) Free Energy (Quasiharmonic) = -2781.117309

Frequencies (Top 3 out of 213)

1. 9.0544 cm^{-1}
2. 10.8480 cm^{-1}
3. 14.2577 cm^{-1}

B3LYP/6-31G(d) Molecular Geometry in Cartesian Coordinates

B	-0.598611	0.889952	0.557348
O	-0.028407	1.193520	1.862383
N	0.624313	0.732836	-0.329712
C	1.861381	0.811260	0.470925
S	0.624502	0.210858	-1.912375
C	1.323297	1.184615	1.856634
O	1.989062	1.453543	2.825794
H	3.101588	2.557208	0.806327
H	2.323977	-0.181481	0.566687
C	2.952273	1.830725	-0.001183
C	4.276267	1.211550	-0.347194
C	5.266350	0.711206	0.579248
C	6.366872	0.244700	-0.192400
N	6.048493	0.451430	-1.520066
C	4.795638	1.031175	-1.605828
H	4.352839	1.262742	-2.563763
C	7.546128	-0.383813	1.781755
C	5.329204	0.615709	1.981687
H	8.344985	-0.654340	-0.210940
H	6.641649	0.233690	-2.305155
C	6.466049	0.070427	2.566284
H	8.421436	-0.804723	2.269468
H	4.496253	0.955004	2.591436
C	7.512086	-0.303981	0.393876
H	6.525875	-0.009891	3.648420
C	0.860019	-1.568229	-1.873863
O	1.798861	0.782258	-2.582083
O	-0.722531	0.483687	-2.427904
C	1.239103	-4.341871	-1.765752
C	2.148532	-2.083266	-1.701959
C	-0.239289	-2.419246	-2.008704
C	-0.038861	-3.797023	-1.953881
C	2.324988	-3.464204	-1.641004
H	3.000635	-1.414436	-1.633235
H	-1.231805	-2.012595	-2.164977
H	-0.893527	-4.460572	-2.061047
H	3.325490	-3.865974	-1.500361
C	1.444372	-5.837632	-1.732943
H	0.577909	-6.351376	-1.303739
H	2.327126	-6.108324	-1.144745
H	1.590862	-6.237081	-2.745364
H	2.556050	2.368102	-0.864181
C	-1.787257	-0.184215	0.637355
C	-2.762436	-0.345268	-0.358189
C	-1.866732	-1.024871	1.759534
C	-3.747228	-1.331169	-0.255059
H	-2.733836	0.281898	-1.241149
C	-2.862224	-1.998436	1.873695
H	-1.140239	-0.919036	2.558538

C	-3.806176	-2.164424	0.860601
H	-4.569616	-2.929421	0.938000
C	-2.952066	-2.835227	3.121518
C	-4.685273	-1.539054	-1.410917
F	-3.538329	-4.031344	2.885962
F	-3.685599	-2.222825	4.081741
F	-1.735269	-3.078127	3.655240
F	-5.768262	-2.272128	-1.072772
F	-4.068563	-2.192867	-2.429631
F	-5.130222	-0.367878	-1.918240
O	-1.330636	2.273940	0.044712
C	-1.475332	3.293394	0.749656
C	-2.089545	4.497089	0.242088
C	-2.518514	4.581149	-1.099133
C	-2.250875	5.593082	1.110966
C	-3.104998	5.753297	-1.554938
H	-2.375448	3.727888	-1.754668
C	-2.841767	6.762714	0.645633
H	-1.913631	5.517787	2.141669
C	-3.267112	6.839741	-0.684214
H	-3.436763	5.829400	-2.585776
H	-2.970820	7.611058	1.310406
H	-3.727395	7.754274	-1.047897
H	-1.118065	3.268359	1.786068

NTOB-benzaldehyde complex 93

B3LYP/6-31G(d) = -2780.351338
B3LYP-D3(BJ)/def2-TZVPP/IEFPCM(propanonitrile) = -2781.602168
B3LYP-D3(BJ)/def2-TZVPP/IEFPCM(propanonitrile)//B3LYP-D3(BJ)/6-31G(d) Free Energy (Quasiharmonic) = -2781.117114

Frequencies (Top 3 out of 213)

1. 9.4954 cm^{-1}
2. 10.8950 cm^{-1}
3. 12.6594 cm^{-1}

B3LYP/6-31G(d) Molecular Geometry in Cartesian Coordinates

B	0.461783	-0.850742	-0.640245
O	-0.140064	-1.193993	-1.913252
N	-0.681210	-0.209624	0.135149
C	-1.939731	-0.332647	-0.627374
S	-0.744068	0.034890	1.787129
C	-1.485689	-0.998536	-1.927379
O	-2.180149	-1.301081	-2.863213
H	-2.046847	1.596393	-1.622350
H	-2.643008	-0.999292	-0.117004
C	-2.650999	1.020459	-0.911611
C	-4.058371	0.874086	-1.406562
C	-5.208040	0.474289	-0.628474
C	-6.330283	0.483589	-1.502907
N	-5.872786	0.870611	-2.746590
C	-4.511877	1.096526	-2.681497
H	-3.960426	1.389967	-3.564037
C	-7.775767	-0.203316	0.264227
C	-5.399071	0.109405	0.717043
H	-8.464112	0.164033	-1.753864
H	-6.434615	0.945604	-3.579735
C	-6.677699	-0.224561	1.148074
H	-8.763250	-0.469778	0.631549
H	-4.559025	0.072769	1.406264
C	-7.617836	0.150063	-1.071436
H	-6.835661	-0.510976	2.184412
C	-0.593086	1.803787	2.084514
O	0.455388	-0.590211	2.363625
O	-2.084641	-0.362659	2.242616
C	-0.370565	4.550988	2.599191
C	0.665358	2.411149	2.062790
C	-1.735549	2.545557	2.388109
C	-1.616120	3.912055	2.639677
C	0.764484	3.776315	2.316281
H	1.560195	1.828976	1.876984
H	-2.699966	2.053172	2.448102
H	-2.506684	4.487190	2.880636
H	1.745328	4.245346	2.303968
C	-0.248921	6.035920	2.844013
H	0.656360	6.275225	3.412324
H	-1.110717	6.423626	3.396239
H	-0.190995	6.587002	1.896033
O	0.771804	-2.295866	0.103569
C	0.651894	-3.416670	-0.430468
H	0.368410	-3.468935	-1.488910
H	-2.649234	1.589493	0.023648
C	0.867775	-4.637185	0.309828
C	1.146055	-4.597666	1.692541
C	0.786557	-5.868836	-0.366966
C	1.345741	-5.786745	2.379884
H	1.183707	-3.636292	2.196030
C	0.992997	-7.054138	0.330157
H	0.565104	-5.885779	-1.431151
C	1.272206	-7.010084	1.699818
H	1.555631	-5.769322	3.444859
H	0.934910	-8.007893	-0.184954
H	1.430068	-7.936808	2.244678
C	1.894855	-0.149791	-0.768287
C	2.794669	-0.034595	0.301538
C	2.282076	0.398724	-2.001053
C	4.013339	0.633784	0.153655
H	2.533552	-0.457082	1.266376
C	3.507440	1.051950	-2.155318
H	1.620936	0.303422	-2.856517
C	4.379588	1.181521	-1.074389
H	5.329318	1.689738	-1.190108
C	3.860294	1.671720	-3.480905
C	4.888922	0.809583	1.362928
F	3.324926	2.908855	-3.612488
F	5.196827	1.804756	-3.637993
F	4.357702	1.719722	2.221288
F	3.401391	0.936628	-4.517918
F	6.128227	1.242648	1.047565
F	5.025216	-0.340492	2.059452

NTOB-benzaldehyde complex 94

B3LYP/6-31G(d) = -2780.351338
B3LYP-D3(BJ)/def2-TZVPP/IEFPCM(propanonitrile) = -2781.602168
B3LYP-D3(BJ)/def2-TZVPP/IEFPCM(propanonitrile)//B3LYP-D3(BJ)/6-31G(d) Free Energy (Quasiharmonic) = -2781.117113

Frequencies (Top 3 out of 213)

1. 9.4955 cm^{-1}
2. 10.8955 cm^{-1}
3. 12.6438 cm^{-1}

B3LYP/6-31G(d) Molecular Geometry in Cartesian Coordinates

B	0.461768	-0.850772	-0.640255
O	-0.140100	-1.194091	-1.913232
N	-0.681248	-0.209743	0.135184
C	-1.939775	-0.332808	-0.627324
S	-0.744081	0.034765	1.787167
C	-1.485743	-0.998748	-1.927306
O	-2.180222	-1.301400	-2.863093
H	-2.046922	1.596170	-1.622423
H	-2.643041	-0.999434	-0.116917
C	-2.651056	1.020279	-0.911636
C	-4.058436	0.873864	-1.406549

C	-5.208085	0.474100	-0.628413
C	-6.330348	0.483342	-1.502820
N	-5.872880	0.870290	-2.746536
C	-4.511973	1.096227	-2.681486
H	-3.960543	1.389625	-3.564054
C	-7.775788	-0.203471	0.264388
C	-5.399083	0.109289	0.717129
H	-8.464181	0.163760	-1.753708
H	-6.434735	0.945261	-3.579666
C	-6.677699	-0.224661	1.148210
H	-8.763261	-0.469917	0.631748
H	-4.559021	0.072692	1.406333
C	-7.617890	0.149834	-1.071299
H	-6.835635	-0.511021	2.184567
C	-0.593122	1.803661	2.084577
O	0.455395	-0.590321	2.363641
O	-2.084639	-0.362809	2.242674
C	-0.370624	4.550864	2.599276
C	-1.735626	2.545463	2.387985
C	0.665332	2.410990	2.063080
C	0.764447	3.776166	2.316581
C	-1.616208	3.911948	2.639574
H	-2.700061	2.053099	2.447846
H	1.560194	1.828798	1.877463
H	1.745306	4.245166	2.304450
H	-2.506797	4.487097	2.880413
C	-0.249073	6.035821	2.843983
H	-1.109746	6.423075	3.398280
H	-0.193710	6.587024	1.895914
H	0.657443	6.275500	3.410146
O	0.771964	-2.295867	0.103544
C	0.652166	-3.416682	-0.430492
H	0.368630	-3.468977	-1.488918
H	-2.649277	1.589371	0.023587
C	0.868248	-4.637176	0.309783
C	0.787176	-5.868830	-0.367023
C	1.146570	-4.597636	1.692486
C	0.993808	-7.054113	0.330077
H	0.565685	-5.885790	-1.431199
C	1.346447	-5.786696	2.379808
H	1.184098	-3.636264	2.195989
C	1.273061	-7.010037	1.699730
H	0.935837	-8.007869	-0.185044
H	1.556369	-5.769258	3.444776
H	1.431072	-7.936746	2.244572
C	1.894779	-0.149708	-0.768345
C	2.281920	0.398872	-2.001105
C	2.794615	-0.034472	0.301460
C	3.507223	1.052215	-2.155381
H	1.620767	0.303533	-2.856555
C	4.013213	0.634032	0.153570
H	2.533560	-0.457018	1.266289
C	4.379380	1.181846	-1.074467
H	5.329054	1.690164	-1.190193
C	4.888784	0.809920	1.362838
C	3.859985	1.672021	-3.480976
F	4.357523	1.720084	2.221148
F	5.025114	-0.340113	2.059423
F	3.324273	2.908996	-3.612655
F	6.128074	1.243018	1.047464
F	3.401342	0.936746	-4.517976
F	5.196487	1.805420	-3.638000

NTOB-benzaldehyde complex 95

B3LYP/6-31G(d) = -2780.351446

B3LYP-D3(BJ)/def2-TZVPP/IEFPCM(propanonitrile) = -2781.6022

B3LYP-D3(BJ)/def2-TZVPP/IEFPCM(propanonitrile)//B3LYP-D3(BJ)/6-31G(d) Free Energy (Quasiharmonic) = -2781.117012

Frequencies (Top 3 out of 213)

1. 9.9016 cm^{-1}
2. 10.8220 cm^{-1}
3. 14.2917 cm^{-1}

B3LYP/6-31G(d) Molecular Geometry in Cartesian Coordinates

B	0.460827	0.849480	0.628619
O	-0.136518	1.207649	1.899711
N	-0.686513	0.204859	-0.137335
C	-1.942541	0.339647	0.627214
S	-0.753903	-0.056873	-1.786474
C	-1.482858	1.017641	1.918989
O	-2.173958	1.332748	2.853176
H	-2.052604	-1.578198	1.643288
H	-2.645316	1.002921	0.111821
C	-2.656923	-1.008305	0.927830
C	-4.062608	-0.852553	1.424700
C	-5.213375	-0.459348	0.644882
C	-6.333254	-0.455115	1.522375
N	-5.873342	-0.828072	2.769467
C	-4.513187	-1.058201	2.703492
H	-3.960023	-1.342057	3.588091
C	-7.781794	0.213891	-0.249116
C	-5.407125	-0.110364	-0.704457
H	-8.465580	-0.127062	1.775147
H	-6.433053	-0.891267	3.605013
C	-6.686074	0.221583	-1.136095
H	-8.769593	0.478387	-0.617007
H	-4.568839	-0.084139	-1.396288
C	-7.621131	-0.123593	1.090327
H	-6.846116	0.495802	-2.175408
C	-0.605494	-1.828923	-2.066170
O	0.444756	0.560730	-2.372592
O	-2.095310	0.337330	-2.242375
C	-0.386606	-4.581441	-2.553681
C	0.652852	-2.436398	-2.045860
C	-1.749933	-2.573536	-2.355205
C	-1.632298	-3.942577	-2.593247
C	0.750253	-3.804176	-2.285802
H	1.548920	-1.852621	-1.871796
H	-2.714571	-2.081547	-2.414928
H	-2.524404	-4.519908	-2.823016
H	1.731219	-4.272993	-2.274897
C	-0.267042	-6.068915	-2.783692
H	0.639238	-6.315442	-3.347239
H	-1.128167	-6.460453	-3.334292
H	-0.212771	-6.610862	-1.830219
O	0.774582	2.285539	-0.130239
C	0.661181	3.412078	0.393095
H	0.381649	3.475995	1.451939
H	-2.659116	-1.587425	-0.001209
C	0.879387	4.624221	-0.360100
C	1.152067	4.569743	-1.743431
C	0.806133	5.862878	0.304720
C	1.354175	5.751048	-2.443346
H	1.183555	3.603257	-2.237471
C	1.014989	7.040275	-0.404963
H	0.588971	5.891359	1.369540
C	1.288617	6.981388	-1.775195
H	1.559784	5.722137	-3.508906
H	0.963091	7.999345	0.100853
H	1.448354	7.901956	-2.329856
C	1.891602	0.144515	0.759853
C	2.274169	-0.404793	1.993845
C	2.789958	0.019592	-0.309915
C	3.493809	-1.068019	2.149197
H	1.607671	-0.314841	2.845575
C	4.001368	-0.662076	-0.161727
H	2.530894	0.439383	-1.276442

C	4.363461	-1.209576	1.067534
H	5.302045	-1.738857	1.180413
C	4.867883	-0.862882	-1.373553
C	3.897105	-1.585850	3.503697
F	4.330267	-1.789916	-2.209995
F	4.999910	0.272875	-2.093630
F	2.831634	-2.004441	4.221466
F	6.109035	-1.290898	-1.058409
F	4.513691	-0.630643	4.240173
F	4.758024	-2.624858	3.411975

NTOB-benzaldehyde complex 96

B3LYP/6-31G(d) = -2780.346734
B3LYP-D3(BJ)/def2-TZVPP/IEFPCM(propanonitrile) = -2781.601477
B3LYP-D3(BJ)/def2-TZVPP/IEFPCM(propanonitrile)//B3LYP-D3(BJ)/6-31G(d) Free Energy (Quasiharmonic) = -2781.117004

Frequencies (Top 3 out of 213)

1. 5.4949 cm^{-1}
2. 8.6363 cm^{-1}
3. 13.6343 cm^{-1}

B3LYP/6-31G(d) Molecular Geometry in Cartesian Coordinates

B	0.441119	0.787650	0.809219
O	-0.517563	0.991842	1.872946
N	-0.409950	0.917210	-0.454221
C	-1.816760	1.196193	-0.099652
S	0.114102	1.071932	-2.023659
C	-1.747285	1.338658	1.424846
O	-2.649292	1.673744	2.147612
H	-2.583595	-0.830213	0.054867
H	-2.151144	2.142579	-0.535900
C	-2.812117	0.076687	-0.517394
C	-4.263203	0.441941	-0.372910
C	-5.180619	0.041514	0.666978
C	-6.446204	0.608458	0.353913
N	-6.293765	1.323319	-0.817113
C	-4.986066	1.215320	-1.249021
H	-4.668760	1.701788	-2.162199
C	-7.428394	-0.380084	2.287222
C	-5.063186	-0.746208	1.825146
H	-8.536344	0.850792	0.895672
H	-7.020726	1.840567	-1.285453
C	-6.182606	-0.951602	2.619974
H	-8.286252	-0.556671	2.930469
H	-4.106452	-1.179248	2.103344
C	-7.577340	0.407582	1.152379
H	-6.099035	-1.556719	3.518523
C	-0.093005	-0.484983	-2.896646
O	1.564122	1.324421	-1.937234
O	-0.763949	2.054327	-2.680051
C	-0.408708	-2.886114	-4.306865
C	-1.165298	-0.632867	-3.778385
C	0.842191	-1.509997	-2.731542
C	0.674080	-2.699641	-3.434660
C	-1.316412	-1.831868	-4.473157
H	-1.854438	0.190386	-3.931801
H	1.702974	-1.379915	-2.087048
H	1.406135	-3.493372	-3.308530
H	-2.148948	-1.945050	-5.163029
C	-0.593386	-4.193785	-5.038145
H	-1.204892	-4.068470	-5.937218
H	-1.095901	-4.932003	-4.399314
H	0.368145	-4.625170	-5.335968
O	1.459116	2.094815	0.963117
C	1.388373	3.151027	0.303384
H	0.589067	3.272336	-0.434238
H	-2.602834	-0.165891	-1.563445
C	2.314734	4.244016	0.488756
C	2.161638	5.394621	-0.307036
C	3.352696	4.170172	1.439496
C	3.037383	6.464159	-0.152681
H	1.362819	5.436781	-1.042874
C	4.221383	5.242026	1.588065
H	3.455078	3.273490	2.041725
C	4.063215	6.385922	0.793648
H	2.925633	7.353475	-0.764996
H	5.023476	5.194916	2.318203
H	4.747357	7.221368	0.914271
C	1.411803	-0.464255	1.040160
C	2.608593	-0.659195	0.336330
C	1.051384	-1.439991	1.982708
C	3.391574	-1.799666	0.537784
H	2.929656	0.079196	-0.391384
C	1.840843	-2.572315	2.198630
H	0.147839	-1.304035	2.568292
C	3.014348	-2.764732	1.469709
H	3.627484	-3.642861	1.633171
C	1.390084	-3.621614	3.179556
C	4.606072	-2.002301	-0.323724
F	2.427972	-4.343554	3.660107
F	0.747498	-3.080702	4.236834
F	5.343698	-0.875345	-0.433439
F	0.532493	-4.501516	2.608611
F	4.252236	-2.355261	-1.589258
F	5.417228	-2.975459	0.142070

NTOB-benzaldehyde complex 97

B3LYP/6-31G(d) = -2780.339494
B3LYP-D3(BJ)/def2-TZVPP/IEFPCM(propanonitrile) = -2781.602407
B3LYP-D3(BJ)/def2-TZVPP/IEFPCM(propanonitrile)//B3LYP-D3(BJ)/6-31G(d) Free Energy (Quasiharmonic) = -2781.116995

Frequencies (Top 3 out of 213)

1. 8.9424 cm^{-1}
2. 12.8453 cm^{-1}
3. 15.5515 cm^{-1}

B3LYP/6-31G(d) Molecular Geometry in Cartesian Coordinates

B	0.310863	-0.495079	0.807998
O	0.638148	-0.616845	2.224157
N	0.471407	1.000133	0.522624
C	0.545538	1.705871	1.816166
S	1.096478	1.701568	-0.853119
C	0.893225	0.585860	2.799534
O	1.272380	0.713756	3.935805
H	-0.592581	2.660529	3.345518
H	1.340660	2.456575	1.827370
C	-0.783957	2.358180	2.308333
C	-1.306037	3.519317	1.517974
C	-2.267677	3.479338	0.443255
C	-2.471282	4.816615	0.009241
N	-1.669987	5.624077	0.791437
C	-0.977385	4.840732	1.693977
H	-0.302195	5.295325	2.407223
C	-4.039185	4.097831	-1.636422
C	-2.979603	2.447927	-0.191651
H	-3.495268	6.171433	-1.345607
H	-1.593655	6.625459	0.711009
C	-3.854658	2.762321	-1.223146
H	-4.728252	4.315189	-2.448219
H	-2.840071	1.414432	0.111521
C	-3.353727	5.141815	-1.026142
H	-4.391226	1.964432	-1.727486

C	2.887514	1.780754	-0.634383
O	0.653539	3.093471	-0.917565
O	0.832420	0.753328	-1.944024
C	5.670877	1.919849	-0.283275
C	3.453673	2.886117	0.008057
C	3.701990	0.762670	-1.131179
C	5.082599	0.838405	-0.953370
C	4.834622	2.943082	0.185225
H	2.819849	3.705603	0.329508
H	3.255417	-0.059352	-1.678258
H	5.715748	0.050188	-1.354359
H	5.271722	3.805794	0.682366
C	7.164115	1.982596	-0.066562
H	7.701143	1.358900	-0.788310
H	7.432512	1.628604	0.937748
H	7.539387	3.007462	-0.157812
O	1.471420	-1.336034	0.024281
C	2.259134	-2.117211	0.600002
H	2.258504	-2.144704	1.695452
H	-1.545629	1.569595	2.351382
C	3.149475	-2.984299	-0.132920
C	4.051950	-3.789600	0.587945
C	3.112052	-3.043164	-1.542415
C	4.917413	-4.638769	-0.093228
H	4.067811	-3.741174	1.673744
C	3.976969	-3.896306	-2.213257
H	2.396841	-2.427698	-2.079247
C	4.878224	-4.690015	-1.490025
H	5.616627	-5.260857	0.456634
H	3.952414	-3.952515	-3.297119
H	5.551449	-5.356870	-2.021542
C	-1.035664	-1.256109	0.383148
C	-1.644694	-1.064543	-0.869569
C	-1.645649	-2.146391	1.277255
C	-2.820005	-1.739106	-1.204902
H	-1.193958	-0.383098	-1.584249
C	-2.818547	-2.829155	0.938209
H	-1.210026	-2.299705	2.260159
C	-3.412258	-2.628723	-0.305288
H	-4.323086	-3.153221	-0.570235
C	-3.400353	-3.826757	1.902475
C	-3.504173	-1.469152	-2.518451
F	-3.293019	-3.409458	3.183405
F	-4.707306	-4.068740	1.661334
F	-4.104600	-2.580861	-3.006497
F	-2.759633	-5.020341	1.829394
F	-2.649436	-1.023747	-3.459302
F	-4.479605	-0.533605	-2.389961

NTOB-benzaldehyde complex 98

B3LYP/6-31G(d) = -2780.348233
B3LYP-D3(BJ)/def2-TZVPP/IEFPCM(propanonitrile) = -2781.601899
B3LYP-D3(BJ)/def2-TZVPP/IEFPCM(propanonitrile)//B3LYP-D3(BJ)/6-31G(d) Free Energy (Quasiharmonic) = -2781.116925

Frequencies (Top 3 out of 213)

1. 7.3729 cm^{-1}
2. 10.2079 cm^{-1}
3. 11.3097 cm^{-1}

B3LYP/6-31G(d) Molecular Geometry in Cartesian Coordinates

B	0.699425	-0.887809	-0.694611
O	0.292566	-1.446548	-1.965069
N	-0.596940	-0.259708	-0.143575
C	-1.725659	-0.616470	-1.037480
S	-0.907545	-0.094432	1.483258
C	-1.033296	-1.374562	-2.174041
O	-1.582654	-1.823333	-3.150712
H	-2.470017	0.572068	-2.691023
H	-2.403692	-1.313605	-0.532916
C	-2.548541	0.592275	-1.597219
C	-3.999453	0.599962	-1.210358
C	-5.007992	-0.353848	-1.613738
C	-6.236505	0.060965	-1.029735
N	-5.978562	1.212872	-0.313796
C	-4.637086	1.529787	-0.428665
H	-4.236413	2.404885	0.063871
C	-7.382819	-1.780809	-2.016640
C	-4.991535	-1.507758	-2.418883
H	-8.361150	-0.310609	-0.766204
H	-6.660068	1.744348	0.204011
C	-6.177055	-2.207305	-2.610172
H	-8.292918	-2.350262	-2.185994
H	-4.066557	-1.839577	-2.882727
C	-7.430516	-0.641948	-1.220459
H	-6.177764	-3.099535	-3.230584
C	-1.476326	1.592645	1.717521
O	0.403728	-0.208968	2.159713
O	-1.999334	-0.982666	1.903107
C	-2.297012	4.215792	2.260639
C	-0.775368	2.665225	1.158337
C	-2.577221	1.811832	2.543782
C	-2.978137	3.122137	2.809557
C	-1.194194	3.964550	1.427732
H	0.080930	2.488965	0.515804
H	-3.112700	0.965311	2.959159
H	-3.835851	3.295094	3.455036
H	-0.653332	4.798962	0.988109
C	-2.718412	5.633002	2.567386
H	-3.709109	5.666408	3.030868
H	-2.745543	6.247845	1.660711
H	-2.013090	6.110972	3.259330
O	0.960808	-2.246233	0.191618
C	1.758365	-2.342202	1.150190
H	2.464983	-1.530069	1.339929
H	-2.065889	1.515460	-1.262920
C	1.804696	-3.509151	1.998281
C	0.913020	-4.584765	1.811573
C	2.765611	-3.554643	3.025619
C	0.991707	-5.690929	2.645749
H	0.174180	-4.528122	1.019289
C	2.838360	-4.667096	3.856869
H	3.442661	-2.716086	3.166038
C	1.952741	-5.731681	3.664946
H	0.307476	-6.522943	2.510941
H	3.576652	-4.707087	4.651653
H	2.008280	-6.600218	4.315455
C	2.062255	-0.043623	-0.814503
C	2.524619	0.876955	0.138724
C	2.844988	-0.213579	-1.971813
C	3.706629	1.600222	-0.058027
H	1.960919	1.032486	1.052527
C	4.026481	0.503061	-2.167796
H	2.516483	-0.911926	-2.734332
C	4.466335	1.418609	-1.210257
H	5.382373	1.977119	-1.361195
C	4.801073	0.337142	-3.448905
C	4.159116	2.550206	1.017286
F	4.351151	1.169607	-4.415839
F	6.115792	0.608091	-3.278829
F	5.146037	3.370506	0.597978
F	4.705792	-0.919082	-3.935099
F	4.626105	1.888232	2.103437
F	3.140359	3.329970	1.450064

NTOB-benzaldehyde complex 99

B3LYP/6-31G(d) = -2780.346478
B3LYP-D3(BJ)/def2-TZVPP/IEFPCM(propanonitrile) = -2781.60146
B3LYP-D3(BJ)/def2-TZVPP/IEFPCM(propanonitrile)//B3LYP-D3(BJ)/6-31G(d) Free Energy (Quasiharmonic) = -2781.116755

Frequencies (Top 3 out of 213)

1. 5.5508 cm^{-1}
2. 9.9266 cm^{-1}
3. 12.6643 cm^{-1}

B3LYP/6-31G(d) Molecular Geometry in Cartesian Coordinates

B	0.793894	-0.134914	0.549375
O	0.385442	-0.546241	1.884980
N	-0.465637	-0.345658	-0.275256
C	-1.520279	-0.973517	0.539126
S	-0.812524	0.239614	-1.791206
C	-0.892770	-0.986506	1.933067
O	-1.429155	-1.353361	2.949064
H	-1.783098	-3.093925	0.942978
H	-2.410486	-0.333833	0.593907
C	-1.956234	-2.414909	0.099117
C	-3.388291	-2.528232	-0.334870
C	-4.558166	-2.454709	0.509921
C	-5.693954	-2.636859	-0.327515
N	-5.225916	-2.803819	-1.616194
C	-3.843637	-2.742686	-1.612175
H	-3.279855	-2.837549	-2.529311
C	-7.161469	-2.431138	1.539993
C	-4.752995	-2.255442	1.889109
H	-7.857297	-2.772831	-0.480249
H	-5.795283	-2.991825	-2.426296
C	-6.050203	-2.245787	2.388024
H	-8.163163	-2.421060	1.961539
H	-3.901140	-2.106313	2.547542
C	-7.000046	-2.628351	0.172802
H	-6.214090	-2.094497	3.451654
C	-2.073522	1.497197	-1.531762
O	-1.430586	-0.820262	-2.594816
O	0.399542	0.923014	-2.260951
C	-4.020656	3.467501	-1.098775
C	-3.425827	1.148046	-1.550983
C	-1.682510	2.819454	-1.308852
C	-2.655908	3.792291	-1.095477
C	-4.386098	2.134297	-1.326969
H	-3.719162	0.121818	-1.744561
H	-0.629085	3.076751	-1.322615
H	-2.352832	4.823485	-0.928988
H	-5.438174	1.860386	-1.333123
C	-5.070977	4.533272	-0.894881
H	-4.690678	5.361490	-0.287972
H	-5.960793	4.129207	-0.401232
H	-5.394945	4.954956	-1.855646
O	1.048426	1.506379	0.655596
C	1.091891	2.123432	1.739524
H	0.884675	1.571244	2.663811
H	-1.292767	-2.724778	-0.711470
C	1.410678	3.530308	1.816442
C	1.377862	4.160979	3.074543
C	1.759416	4.262996	0.662700
C	1.681967	5.514236	3.178650
H	1.112709	3.586084	3.958198
C	2.065566	5.611817	0.776383
H	1.795211	3.755465	-0.296109
C	2.024702	6.235571	2.031125
H	1.656479	6.006323	4.145840
H	2.340807	6.182772	-0.105026
H	2.266385	7.291641	2.113208
C	2.234483	-0.703275	0.131308
C	2.936277	-0.292008	-1.014360
C	2.850935	-1.652986	0.960781
C	4.197177	-0.811919	-1.314095
H	2.487125	0.432458	-1.683467
C	4.111095	-2.177530	0.659229
H	2.340597	-1.990871	1.856811
C	4.793704	-1.758590	-0.480783
H	5.772355	-2.160398	-0.715490
C	4.761233	-3.144843	1.610830
C	4.895615	-0.385037	-2.577598
F	5.396891	-2.500314	2.620524
F	3.859006	-3.970214	2.185616
F	4.500354	-1.115594	-3.644441
F	5.685917	-3.917094	0.997701
F	6.239109	-0.524876	-2.483131
F	4.647011	0.910562	-2.875582

NTOB-benzaldehyde complex 100

B3LYP/6-31G(d) = -2780.350929
B3LYP-D3(BJ)/def2-TZVPP/IEFPCM(propanonitrile) = -2781.601672
B3LYP-D3(BJ)/def2-TZVPP/IEFPCM(propanonitrile)//B3LYP-D3(BJ)/6-31G(d) Free Energy (Quasiharmonic) = -2781.116439

Frequencies (Top 3 out of 213)

1. 7.3786 cm^{-1}
2. 9.6567 cm^{-1}
3. 14.1412 cm^{-1}

B3LYP/6-31G(d) Molecular Geometry in Cartesian Coordinates

B	-0.963824	-0.581549	1.003622
O	-0.592917	-0.647031	2.400537
N	0.387563	-0.648315	0.269570
C	1.473389	-0.839217	1.259680
S	0.584481	-1.330202	-1.240075
C	0.715553	-0.889396	2.592347
O	1.199952	-1.078331	3.682519
H	2.096292	1.185886	1.734855
H	1.976309	-1.800110	1.104548
C	2.526549	0.303698	1.239734
C	3.875033	-0.046942	1.812839
C	5.145454	0.361902	1.258770
C	6.164473	-0.168106	2.099080
N	5.532182	-0.860532	3.109413
C	4.163148	-0.782666	2.937467
H	3.482245	-1.247925	3.634970
C	7.867421	0.794640	0.734418
C	5.525587	1.124815	0.140327
H	8.288062	-0.376343	2.503786
H	5.991776	-1.347390	3.862675
C	6.876311	1.333030	-0.111585
H	8.915936	0.975428	0.513380
H	4.774135	1.549434	-0.520265
C	7.525096	0.037774	1.849213
H	7.177619	1.924098	-0.972607
C	1.636304	-0.216118	-2.175806
O	-0.754346	-1.304444	-1.867215
O	1.302272	-2.608498	-1.133539
C	3.284398	1.473241	-3.683418
C	2.923231	-0.632478	-2.515033
C	1.154548	1.032160	-2.584216
C	1.982372	1.864493	-3.330266
C	3.738632	0.217315	-3.263226
H	3.275090	-1.606686	-2.194556
H	0.150242	1.354251	-2.331858
H	1.610193	2.835461	-3.647700
H	4.744440	-0.103855	-3.520713
C	4.158335	2.379225	-4.517132
H	4.070745	3.423716	-4.198621

H	3.868458	2.338779	-5.575300
H	5.211767	2.090296	-4.453027
O	-1.678058	-2.042998	0.782741
C	-2.562587	-2.295835	-0.064592
H	-3.032532	-1.471600	-0.607565
H	2.662634	0.591644	0.193473
C	-3.013080	-3.641163	-0.328630
C	-2.435784	-4.747445	0.326933
C	-4.048688	-3.831257	-1.262603
C	-2.898708	-6.025483	0.047935
H	-1.632458	-4.581341	1.036747
C	-4.507462	-5.115231	-1.535933
H	-4.480632	-2.971961	-1.768882
C	-3.932573	-6.208063	-0.880428
H	-2.458300	-6.883811	0.545849
H	-5.305020	-5.267943	-2.256287
H	-4.289467	-7.211554	-1.095426
C	-2.013133	0.596386	0.703673
C	-2.290051	1.097298	-0.576570
C	-2.672787	1.206397	1.785972
C	-3.168837	2.167943	-0.769270
H	-1.816160	0.643672	-1.440264
C	-3.557781	2.269527	1.596183
H	-2.480492	0.849697	2.792558
C	-3.809823	2.762144	0.314367
H	-4.491527	3.591126	0.166366
C	-4.195397	2.934867	2.787569
C	-3.420193	2.644290	-2.173212
F	-4.437528	2.055627	3.783919
F	-3.404193	3.904431	3.301449
F	-4.147676	1.748617	-2.882777
F	-5.372969	3.518542	2.466326
F	-2.257887	2.816535	-2.851990
F	-4.085199	3.817344	-2.210683

NTOB-benzaldehyde complex 101

B3LYP/6-31G(d) = -2780.350929
B3LYP-D3(BJ)/def2-TZVPP/IEFPCM(propanonitrile) = -2781.601672
B3LYP-D3(BJ)/def2-TZVPP/IEFPCM(propanonitrile)//B3LYP-D3(BJ)/6-31G(d) Free Energy (Quasiharmonic) = -2781.116438

Frequencies (Top 3 out of 213)

1. 7.3818 cm^{-1}
2. 9.6599 cm^{-1}
3. 14.1513 cm^{-1}

B3LYP/6-31G(d) Molecular Geometry in Cartesian Coordinates

B	0.963898	-0.581424	-1.003753
O	0.592963	-0.646622	-2.400674
N	-0.387500	-0.648222	-0.269683
C	-1.473334	-0.838946	-1.259826
S	-0.584440	-1.330409	1.239831
C	-0.715517	-0.888891	-2.592511
O	-1.199945	-1.077591	-3.682713
H	-2.096248	1.186244	-1.734610
H	-1.976252	-1.799870	-1.104868
C	-2.526506	0.303956	-1.239667
C	-3.874980	-0.046575	-1.812863
C	-5.145410	0.362195	-1.258759
C	-6.164416	-0.167654	-2.099187
N	-5.532107	-0.859924	-3.109616
C	-4.163076	-0.782101	-2.937625
H	-3.482161	-1.247238	-3.635198
C	-7.867387	0.794893	-0.734413
C	-5.525563	1.124926	-0.140198
H	-8.287996	-0.375784	-2.503991
H	-5.991688	-1.346624	-3.862988
C	-6.876291	1.333123	0.111710
H	-8.915905	0.975665	-0.513376
H	-4.774122	1.549422	0.520485
C	-7.525043	0.038209	-1.849325
H	-7.177614	1.924055	0.972820
C	-1.636299	-0.216515	2.175747
O	0.754368	-1.304762	1.867015
O	-1.302218	-2.608693	1.133036
C	-3.284461	1.472542	3.683628
C	-1.154581	1.031701	2.584386
C	-2.923225	-0.632964	2.514878
C	-3.738657	0.216676	3.263210
C	-1.982437	1.863882	3.330572
H	-0.150275	1.353860	2.332118
H	-3.275054	-1.607125	2.194227
H	-4.744460	-0.104568	3.520627
H	-1.610279	2.834796	3.648196
C	-4.158488	2.378388	4.517398
H	-3.868684	2.337852	5.575557
H	-5.211897	2.089395	4.453179
H	-4.070943	3.422921	4.199000
O	1.678071	-2.042874	-0.783091
C	2.562495	-2.295866	0.064313
H	3.032424	-1.471723	0.607436
H	-2.662608	0.591692	-0.193351
C	3.012915	-3.641247	0.328227
C	4.048442	-3.831479	1.262260
C	2.435645	-4.747437	-0.327515
C	4.507168	-5.115497	1.535469
H	4.480361	-2.972255	1.768683
C	2.898523	-6.025520	-0.048642
H	1.632378	-4.581230	-1.037373
C	3.932311	-6.208236	0.879781
H	5.304663	-5.268314	2.255870
H	2.458139	-6.883777	-0.546698
H	4.289169	-7.211760	1.094681
C	2.013200	0.596481	-0.703621
C	2.672945	1.206602	-1.785804
C	2.290028	1.097261	0.576691
C	3.557942	2.269695	-1.595842
H	2.480724	0.850013	-2.792442
C	3.168817	2.167877	0.769570
H	1.816057	0.643563	1.440302
C	3.809897	2.762180	-0.313954
H	4.491605	3.591132	-0.165814
C	3.420066	2.644026	2.173598
C	4.195635	2.935155	-2.787120
F	2.257680	2.816585	2.852164
F	4.147107	1.748047	2.883230
F	3.404445	3.904739	-3.300987
F	4.085446	3.816858	2.211305
F	4.437870	2.056005	-3.783525
F	5.373164	3.518836	-2.465734

NTOB-benzaldehyde complex 102

B3LYP/6-31G(d) = -2780.350962
B3LYP-D3(BJ)/def2-TZVPP/IEFPCM(propanonitrile) = -2781.601546
B3LYP-D3(BJ)/def2-TZVPP/IEFPCM(propanonitrile)//B3LYP-D3(BJ)/6-31G(d) Free Energy (Quasiharmonic) = -2781.116382

Frequencies (Top 3 out of 213)

1. 6.9492 cm^{-1}
2. 9.3931 cm^{-1}
3. 12.1968 cm^{-1}

B3LYP/6-31G(d) Molecular Geometry in Cartesian Coordinates

B	0.955497	-0.622828	-0.973618

O	0.587583	-0.751144	-2.366866
N	-0.397575	-0.653385	-0.241092
C	-1.481216	-0.893338	-1.222901
S	-0.599986	-1.252510	1.302338
C	-0.720426	-1.002500	-2.550335
O	-1.202150	-1.240499	-3.632040
H	-2.110050	1.105856	-1.790386
H	-1.981183	-1.847702	-1.024083
C	-2.538707	0.245144	-1.257523
C	-3.884356	-0.135745	-1.817885
C	-5.157642	0.289676	-1.283154
C	-6.172729	-0.279104	-2.102603
N	-5.535459	-1.009624	-3.082527
C	-4.167119	-0.918572	-2.911656
H	-3.482889	-1.409535	-3.587930
C	-7.882537	0.731013	-0.781456
C	-5.543316	1.096047	-0.197570
H	-8.294596	-0.513567	-2.501935
H	-5.991501	-1.529740	-3.815442
C	-6.895424	1.308153	0.043388
H	-8.932266	0.915825	-0.569699
H	-4.795112	1.550813	0.446460
C	-7.534727	-0.069370	-1.863682
H	-7.200969	1.932437	0.879099
C	-1.637498	-0.078819	2.179852
O	0.739821	-1.209486	1.926406
O	-1.332587	-2.526113	1.263076
C	-3.262019	1.704112	3.602955
C	-1.146521	1.189440	2.508062
C	-2.921928	-0.468712	2.557483
C	-3.725554	0.427505	3.263262
C	-1.962626	2.068103	3.212579
H	-0.144189	1.492036	2.225608
H	-3.281088	-1.458532	2.298916
H	-4.729353	0.126363	3.551067
H	-1.583168	3.054536	3.467574
C	-4.123070	2.662349	4.390380
H	-5.175911	2.365101	4.369749
H	-4.048661	3.682329	3.997518
H	-3.809967	2.697784	5.442047
O	1.664973	-2.075662	-0.685204
C	2.548472	-2.293705	0.172642
H	3.020837	-1.447979	0.679217
H	-2.679305	0.579192	-0.225689
C	2.994893	-3.627575	0.495553
C	4.028697	-3.779774	1.438410
C	2.415820	-4.759691	-0.112634
C	4.483780	-5.051909	1.767620
H	4.462203	-2.900507	1.907706
C	2.875094	-6.025646	0.221918
H	1.614231	-4.622400	-0.830534
C	3.907102	-6.170573	1.158956
H	5.279953	-5.175367	2.495075
H	2.433373	-6.903551	-0.239356
H	4.261199	-7.164732	1.417516
C	2.008532	0.564222	-0.726113
C	2.293197	1.116402	0.530960
C	2.658784	1.132999	-1.836556
C	3.166012	2.199940	0.673893
H	1.826166	0.697304	1.415810
C	3.541201	2.205552	-1.695590
H	2.450506	0.744676	-2.828059
C	3.797246	2.752685	-0.436593
H	4.464863	3.599012	-0.328419
C	4.261929	2.747365	-2.901971
C	3.399420	2.748615	2.054231
F	5.412393	2.070571	-3.136211
F	3.516007	2.651220	-4.022223
F	4.039210	1.854257	2.844729
F	4.598792	4.047632	-2.746116
F	2.227285	3.037205	2.675175
F	4.137724	3.877251	2.046612

NTOB-benzaldehyde complex 103

B3LYP/6-31G(d) = -2780.350962
B3LYP-D3(BJ)/def2-TZVPP/IEFPCM(propanonitrile) = -2781.601546
B3LYP-D3(BJ)/def2-TZVPP/IEFPCM(propanonitrile)//B3LYP-D3(BJ)/6-31G(d) Free Energy (Quasiharmonic) = -2781.116382

Frequencies (Top 3 out of 213)

1. 6.9462 cm^{-1}
2. 9.3888 cm^{-1}
3. 12.1895 cm^{-1}

B3LYP/6-31G(d) Molecular Geometry in Cartesian Coordinates

B	-0.955465	0.622749	-0.973604
O	-0.587559	0.751098	-2.366845
N	0.397598	0.653290	-0.241068
C	1.481231	0.893326	-1.222864
S	0.599977	1.252430	1.302375
C	0.720443	1.002502	-2.550299
O	1.202166	1.240561	-3.631990
H	2.110133	-1.105852	-1.790326
H	1.981159	1.847705	-1.024019
C	2.538764	-0.245113	-1.257484
C	3.884397	0.135800	-1.817865
C	5.157688	-0.289711	-1.283221
C	6.172763	0.279110	-2.102656
N	5.535482	1.009729	-3.082499
C	4.167145	0.918713	-2.911578
H	3.482909	1.409747	-3.587794
C	7.882584	-0.731183	-0.781662
C	5.543372	-1.096196	-0.197726
H	8.294623	0.513543	-2.502041
H	5.991515	1.529929	-3.815360
C	6.895482	-1.308370	0.043162
H	8.932315	-0.916049	-0.569961
H	4.795177	-1.550996	0.446290
C	7.534763	0.069312	-1.863802
H	7.201037	-1.932743	0.878803
C	1.637588	0.078828	2.179893
O	-0.739823	1.209320	1.926445
O	1.332501	2.526077	1.263091
C	3.262310	-1.703956	3.602962
C	1.146627	-1.189362	2.508388
C	2.922111	0.468720	2.557220
C	3.725835	-0.427422	3.262978
C	1.962833	-2.067950	3.212884
H	0.144229	-1.491969	2.226185
H	3.281265	1.458488	2.298442
H	4.729703	-0.126278	3.550542
H	1.583381	-3.054330	3.468102
C	4.123430	-2.662082	4.390443
H	5.176317	-2.365014	4.369445
H	4.048737	-3.682176	3.997940
H	3.810597	-2.697113	5.442205
O	-1.664942	2.075598	-0.685170
C	-2.548444	2.293635	0.172674
H	-3.020793	1.447903	0.679253
H	2.679382	-0.579138	-0.225643
C	-2.994895	3.627493	0.495592
C	-4.028671	3.779650	1.438485
C	-2.415889	4.759633	-0.112614
C	-4.483788	5.051767	1.767719
H	-4.462128	2.900365	1.907793
C	-2.875197	6.025569	0.221961
H	-1.614319	4.622371	-0.830539
C	-3.907173	6.170454	1.159039

H	-5.279937	5.175192	2.495206
H	-2.433527	6.903492	-0.239326
H	-4.261296	7.164599	1.417618
C	-2.008531	-0.564279	-0.726113
C	-2.658866	-1.132967	-1.836549
C	-2.293151	-1.116520	0.530944
C	-3.541335	-2.205482	-1.695591
H	-2.450613	-0.744613	-2.828046
C	-3.166011	-2.200019	0.673871
H	-1.826044	-0.697493	1.415783
C	-3.797350	-2.752660	-0.436613
H	-4.465003	-3.598959	-0.328452
C	-3.399551	-2.748645	2.054209
C	-4.262090	-2.747198	-2.901999
F	-2.227457	-3.036691	2.675484
F	-4.039921	-1.854485	2.844469
F	-3.515925	-2.651553	-4.022137
F	-4.137391	-3.877585	2.046512
F	-5.412216	-2.069944	-3.136562
F	-4.599532	-4.047295	-2.745997

NTOB-benzaldehyde complex 104

B3LYP/6-31G(d) = -2780.337499
B3LYP-D3(BJ)/def2-TZVPP/IEFPCM(propanonitrile) = -2781.600974
B3LYP-D3(BJ)/def2-TZVPP/IEFPCM(propanonitrile)//B3LYP-D3(BJ)/6-31G(d) Free Energy (Quasiharmonic) = -2781.116199

Frequencies (Top 3 out of 213)

1. 8.5902 cm^{-1}
2. 9.2345 cm^{-1}
3. 14.6121 cm^{-1}

B3LYP/6-31G(d) Molecular Geometry in Cartesian Coordinates

B	0.448188	0.181466	-0.833300
O	0.492434	1.348371	-1.692859
N	-0.536854	-0.736799	-1.511738
C	-1.167263	-0.036563	-2.644779
S	-0.532553	-2.414771	-1.509501
C	-0.376170	1.266533	-2.726844
O	-0.501067	2.123940	-3.565472
H	-3.203147	-0.674450	-2.282635
H	-0.999006	-0.608241	-3.562652
C	-2.696283	0.257465	-2.551985
C	-3.133771	1.377763	-1.643447
C	-3.612080	1.312000	-0.281250
C	-3.911808	2.644437	0.127515
N	-3.633012	3.468445	-0.941579
C	-3.173136	2.706693	-1.996468
H	-2.884285	3.171286	-2.928432
C	-4.577840	1.894226	2.293459
C	-3.820164	0.267244	0.637602
H	-4.624735	3.973019	1.692269
H	-3.754948	4.468938	-0.959789
C	-4.294352	0.565596	1.909863
H	-4.956837	2.094802	3.291993
H	-3.606638	-0.759474	0.361179
C	-4.393565	2.949767	1.406193
H	-4.455820	-0.239650	2.621338
C	-1.951057	-2.939114	-0.534000
O	0.655090	-2.855243	-0.776200
O	-0.789807	-2.853778	-2.887174
C	-4.136283	-3.821183	0.985295
C	-1.844481	-3.003757	0.856302
C	-3.128555	-3.325703	-1.176473
C	-4.212889	-3.756478	-0.413144
C	-2.936497	-3.439721	1.604204
H	-0.912218	-2.735785	1.341714
H	-3.180627	-3.307271	-2.259904
H	-5.129956	-4.057877	-0.913625
H	-2.852250	-3.495126	2.686898
C	-5.302918	-4.327493	1.799729
H	-6.259606	-4.031959	1.356336
H	-5.295565	-5.424027	1.855855
H	-5.271073	-3.948533	2.826229
H	-3.009846	0.494286	-3.575038
C	1.879457	-0.244428	-0.268199
C	2.086254	-0.802206	1.001828
C	3.010594	-0.019023	-1.065110
C	3.364223	-1.128317	1.454643
H	1.239839	-0.986293	1.654633
C	4.294106	-0.346670	-0.617263
H	2.893559	0.420109	-2.050944
C	4.479246	-0.903703	0.645763
H	5.473424	-1.154233	0.996726
C	5.486712	-0.022066	-1.475734
C	3.538452	-1.774603	2.802219
F	5.219256	-0.176208	-2.790159
F	6.551416	-0.800165	-1.181413
F	5.883581	1.264506	-1.307544
F	3.438305	-3.120751	2.736249
F	2.598182	-1.356540	3.684010
F	4.746970	-1.496837	3.342720
O	-0.421362	0.653982	0.582287
C	-0.673122	1.659508	1.270211
C	-0.023419	2.957878	1.301143
C	-0.602898	3.911667	2.165246
C	1.142043	3.287973	0.578624
C	-0.038368	5.174261	2.298581
H	-1.502611	3.650912	2.716598
C	1.703980	4.550509	0.724406
H	1.591355	2.567277	-0.090097
C	1.117308	5.491320	1.578351
H	-0.488599	5.906884	2.961555
H	2.602434	4.805274	0.170855
H	1.565507	6.475577	1.684139
H	-1.503811	1.534953	1.978228

NTOB-benzaldehyde complex 105

B3LYP/6-31G(d) = -2780.343939
B3LYP-D3(BJ)/def2-TZVPP/IEFPCM(propanonitrile) = -2781.601765
B3LYP-D3(BJ)/def2-TZVPP/IEFPCM(propanonitrile)//B3LYP-D3(BJ)/6-31G(d) Free Energy (Quasiharmonic) = -2781.116198

Frequencies (Top 3 out of 213)

1. 8.8949 cm^{-1}
2. 11.4283 cm^{-1}
3. 15.3770 cm^{-1}

B3LYP/6-31G(d) Molecular Geometry in Cartesian Coordinates

B	-1.009286	0.163027	-0.954436
O	-0.652539	0.940814	-2.126257
N	0.285423	0.146640	-0.115190
C	1.306876	0.975380	-0.798277
S	0.177347	0.216657	1.564342
C	0.583411	1.457197	-2.064214
O	1.045387	2.183426	-2.912183
H	2.376180	-0.462935	-2.017157
H	1.571623	1.857329	-0.202493
C	2.593297	0.180175	-1.151180
C	3.832816	1.003851	-1.384314
C	5.174866	0.598443	-1.034726
C	6.051141	1.649394	-1.426458
N	5.268882	2.637097	-1.984712
C	3.943940	2.244458	-1.965187

H	3.168910	2.865122	-2.390066
C	7.948580	0.436860	-0.638304
C	5.726599	-0.549164	-0.438591
H	8.086475	2.398871	-1.537547
H	5.606541	3.505349	-2.369460
C	7.102079	-0.621086	-0.246614
H	9.020033	0.352367	-0.478374
H	5.087735	-1.374169	-0.134124
C	7.434743	1.584257	-1.231738
H	7.536822	-1.511108	0.201334
C	1.843855	-0.072297	2.163246
O	-0.637590	-0.960581	1.928931
O	-0.210509	1.555346	2.025201
C	4.425615	-0.513023	3.155396
C	2.655109	1.018179	2.484258
C	2.291749	-1.379308	2.362587
C	3.577633	-1.589469	2.856599
C	3.939122	0.789175	2.971508
H	2.274226	2.027316	2.370226
H	1.632125	-2.215356	2.156564
H	3.927255	-2.605951	3.019878
H	4.574520	1.636550	3.215293
C	5.835677	-0.744119	3.639287
H	6.121691	-0.014676	4.404536
H	5.958602	-1.747161	4.059872
H	6.543137	-0.639912	2.806462
H	2.783853	-0.499603	-0.315822
C	-2.449174	0.579328	-0.366738
C	-3.242739	-0.237529	0.451576
C	-2.972934	1.831391	-0.723518
C	-4.495722	0.179289	0.904262
H	-2.885067	-1.211323	0.765582
C	-4.224658	2.255083	-0.270616
H	-2.398217	2.485719	-1.369854
C	-4.995521	1.430910	0.546628
H	-5.969090	1.755373	0.895123
C	-4.776642	3.578363	-0.728685
C	-5.290726	-0.704954	1.826119
F	-5.425203	3.465496	-1.913195
F	-3.803750	4.498493	-0.903074
F	-5.664976	4.087858	0.154786
F	-4.988902	-0.486424	3.125266
F	-5.058916	-2.018145	1.586686
F	-6.622168	-0.502452	1.694217
O	-1.222677	-1.338181	-1.557287
C	-0.953310	-2.376403	-0.913269
C	-1.223290	-3.694764	-1.437293
C	-1.783121	-3.874373	-2.719063
C	-0.917199	-4.810394	-0.634788
C	-2.028611	-5.158301	-3.184493
H	-2.014340	-3.002436	-3.322163
C	-1.167789	-6.093874	-1.108220
H	-0.492609	-4.660060	0.354407
C	-1.721489	-6.264720	-2.380494
H	-2.459621	-5.306452	-4.169829
H	-0.937054	-6.957593	-0.492569
H	-1.917680	-7.267309	-2.750349
H	-0.513721	-2.273506	0.085022

NTOB-benzaldehyde complex 106

B3LYP/6-31G(d) = -2780.347302
B3LYP-D3(BJ)/def2-TZVPP/IEFPCM(propanonitrile) = -2781.599966
B3LYP-D3(BJ)/def2-TZVPP/IEFPCM(propanonitrile)//B3LYP-D3(BJ)/6-31G(d) Free Energy (Quasiharmonic) = -2781.116102

Frequencies (Top 3 out of 213)

1. 3.1724 cm^{-1}
2. 9.6976 cm^{-1}
3. 11.0712 cm^{-1}

B3LYP/6-31G(d) Molecular Geometry in Cartesian Coordinates

B	-0.428681	0.655552	-0.747674
O	0.351288	0.785699	-1.952891
N	0.628457	0.427173	0.338280
C	1.972881	0.689314	-0.228726
S	0.409861	0.706541	1.962624
C	1.681525	0.854867	-1.722541
O	2.499908	1.001769	-2.592928
H	2.782145	-1.301195	-0.565445
H	2.368849	1.636591	0.153890
C	3.013146	-0.428086	0.055509
C	4.445729	-0.007327	-0.129136
C	5.325608	-0.271073	-1.242745
C	6.589999	0.296165	-0.923284
N	6.471199	0.883629	0.320374
C	5.186859	0.693371	0.791819
H	4.893817	1.073927	1.761576
C	7.512489	-0.458653	-2.986997
C	5.176909	-0.934418	-2.473227
H	8.648913	0.652296	-1.519263
H	7.205727	1.368601	0.810869
C	6.266634	-1.025053	-3.328465
H	8.346201	-0.543581	-3.678922
H	4.216189	-1.350738	-2.760136
C	7.690752	0.211087	-1.782782
H	6.158310	-1.532666	-4.283107
C	0.761181	-0.849561	2.789842
O	-1.027895	0.997214	2.148946
O	1.401487	1.679387	2.444295
C	1.219740	-3.227484	4.197679
C	0.243292	-2.052093	2.300636
C	1.500346	-0.818741	3.972370
C	1.723434	-2.008066	4.666287
C	0.476696	-3.228008	3.006416
H	-0.325212	-2.071554	1.377091
H	1.896920	0.123405	4.334705
H	2.300385	-1.985695	5.587410
H	0.075314	-4.163968	2.625747
C	1.484823	-4.516796	4.937230
H	1.821530	-4.329805	5.961477
H	2.263810	-5.105404	4.435329
H	0.586856	-5.142819	4.982833
O	-0.953937	2.222649	-0.522292
C	-2.000863	2.564668	0.065480
H	-2.733062	1.801213	0.342841
H	2.869166	-0.745774	1.093067
C	-2.300806	3.945210	0.369940
C	-3.515262	4.241866	1.015700
C	-1.406107	4.979861	0.030752
C	-3.834402	5.561272	1.318851
H	-4.195047	3.435724	1.279100
C	-1.732050	6.293886	0.335766
H	-0.473112	4.731127	-0.463896
C	-2.943584	6.583188	0.978042
H	-4.769045	5.795413	1.818861
H	-1.048480	7.097215	0.078660
H	-3.192435	7.613930	1.215197
C	-1.704249	-0.301882	-0.948424
C	-2.039453	-0.670407	-2.265478
C	-2.513592	-0.810201	0.080401
C	-3.123266	-1.505436	-2.539113
H	-1.435721	-0.298934	-3.086463
C	-3.601612	-1.648134	-0.192782
H	-2.299080	-0.551954	1.112331
C	-3.915172	-2.001216	-1.501847
H	-4.759042	-2.647219	-1.712360
C	-4.448559	-2.126471	0.955243
C	-3.407717	-1.926040	-3.957286

F	-3.689056	-2.615374	1.962383
F	-5.185447	-1.117977	1.482082
F	-3.074889	-0.964248	-4.844250
F	-5.310814	-3.098853	0.589510
F	-4.716936	-2.213712	-4.141902
F	-2.711166	-3.035120	-4.297536

NTOB-benzaldehyde complex 107

B3LYP/6-31G(d) = -2780.343894
B3LYP-D3(BJ)/def2-TZVPP/IEFPCM(propanonitrile) = -2781.60072
B3LYP-D3(BJ)/def2-TZVPP/IEFPCM(propanonitrile)//B3LYP-D3(BJ)/6-31G(d) Free Energy (Quasiharmonic) = -2781.115734

Frequencies (Top 3 out of 213)

1. 9.1703 cm^{-1}
2. 10.8101 cm^{-1}
3. 13.5836 cm^{-1}

B3LYP/6-31G(d) Molecular Geometry in Cartesian Coordinates

B	0.791760	0.047660	-0.932170
O	0.429970	-0.344910	-2.284210
N	-0.573970	0.268289	-0.275350
C	-1.583400	-0.504491	-1.040530
S	-0.793560	0.728719	1.292910
C	-0.882300	-0.622831	-2.406490
O	-1.407210	-0.914571	-3.452310
H	-2.972650	0.941239	-1.868920
H	-1.678370	-1.513211	-0.613970
C	-2.999140	0.105249	-1.161680
C	-4.036600	-0.905661	-1.561440
C	-4.741300	-1.795091	-0.668030
C	-5.631529	-2.570001	-1.462190
N	-5.464929	-2.162191	-2.770880
C	-4.502030	-1.173461	-2.823670
H	-4.195940	-0.748741	-3.768540
C	-6.420719	-3.729242	0.465820
C	-4.703529	-2.015741	0.720770
H	-7.152759	-4.119082	-1.532580
H	-5.932339	-2.558441	-3.570950
C	-5.541329	-2.977891	1.272040
H	-7.062699	-4.475342	0.926460
H	-4.016810	-1.456641	1.351640
C	-6.477859	-3.536552	-0.910240
H	-5.515899	-3.160641	2.342970
C	-1.870620	2.168519	1.246100
O	0.530880	1.201020	1.749370
O	-1.499380	-0.307411	2.061940
C	-3.467871	4.469709	1.314070
C	-1.639100	3.197329	0.329960
C	-2.883540	2.276529	2.198420
C	-3.675440	3.424739	2.222610
C	-2.440351	4.334439	0.367010
H	-0.859270	3.095639	-0.417890
H	-3.053700	1.464539	2.897000
H	-4.471920	3.504879	2.958090
H	-2.271381	5.129669	-0.355310
C	-4.314111	5.719539	1.358920
H	-5.268241	5.539509	1.863660
H	-4.527241	6.095729	0.352510
H	-3.800911	6.523049	1.903730
H	-3.283150	0.524529	-0.192230
C	1.901580	-0.935520	-0.303280
C	3.122180	-1.104750	-0.979000
C	1.686720	-1.719440	0.840040
C	4.094631	-1.998070	-0.525260
H	3.319640	-0.535580	-1.883640
C	2.654731	-2.620250	1.294590
H	0.751990	-1.645510	1.386060
C	3.865631	-2.761890	0.618950
H	4.615881	-3.458080	0.975000
C	2.417071	-3.397860	2.562810
C	5.366891	-2.179859	-1.308320
F	3.111591	-4.559050	2.577220
F	1.113981	-3.700970	2.731290
F	2.805281	-2.697170	3.654640
F	6.379661	-2.625639	-0.531420
F	5.774350	-1.018769	-1.874590
F	5.218531	-3.072009	-2.313090
O	1.443180	1.517870	-1.116090
C	2.241620	2.012310	-0.284960
C	2.726180	3.366920	-0.403080
C	3.583829	3.858680	0.598980
C	2.366449	4.184990	-1.493890
C	4.073429	5.157910	0.515130
H	3.851140	3.220670	1.437050
C	2.862899	5.478410	-1.572330
H	1.712369	3.785680	-2.262290
C	3.713349	5.963390	-0.568990
H	4.732049	5.543260	1.287080
H	2.595669	6.113950	-2.411060
H	4.098549	6.977100	-0.636210
H	2.587840	1.397450	0.547230

NTOB-benzaldehyde complex 108

B3LYP/6-31G(d) = -2780.347863
B3LYP-D3(BJ)/def2-TZVPP/IEFPCM(propanonitrile) = -2781.599422
B3LYP-D3(BJ)/def2-TZVPP/IEFPCM(propanonitrile)//B3LYP-D3(BJ)/6-31G(d) Free Energy (Quasiharmonic) = -2781.115107

Frequencies (Top 3 out of 213)

1. 4.3645 cm^{-1}
2. 9.3133 cm^{-1}
3. 12.1586 cm^{-1}

B3LYP/6-31G(d) Molecular Geometry in Cartesian Coordinates

B	0.403221	0.878540	0.430939
O	-0.486873	1.315806	1.487314
N	-0.544756	0.442764	-0.679136
C	-1.936768	0.766609	-0.310006
S	-0.158571	0.236680	-2.292061
C	-1.787835	1.358690	1.092428
O	-2.669321	1.792758	1.786884
H	-2.646181	-1.114586	0.521111
H	-2.347490	1.534642	-0.973664
C	-2.899741	-0.454540	-0.316457
C	-4.359294	-0.094879	-0.287237
C	-5.273501	-0.161959	0.827460
C	-6.548830	0.250153	0.352020
N	-6.404299	0.559512	-0.985408
C	-5.092415	0.346566	-1.362478
H	-4.776902	0.527467	-2.381754
C	-7.520738	-0.089826	2.501576
C	-5.146074	-0.535131	2.176665
H	-8.646077	0.609505	0.791674
H	-7.139133	0.884495	-1.593525
C	-6.265179	-0.498779	2.997376
H	-8.378256	-0.069885	3.168823
H	-4.181393	-0.834305	2.576504
C	-7.679637	0.291287	1.174848
H	-6.174035	-0.783189	4.042131
C	-0.215494	-1.527711	-2.644355
O	1.242146	0.651382	-2.459423
O	-1.224882	0.864648	-3.086284
C	-0.303890	-4.264742	-3.246691

C	0.856009	-2.349328	-2.284253
C	-1.315005	-2.051265	-3.325583
C	-1.352440	-3.414206	-3.618675
C	0.800700	-3.707334	-2.584982
H	1.733174	-1.937934	-1.798908
H	-2.116774	-1.392037	-3.639902
H	-2.207658	-3.819753	-4.153628
H	1.637803	-4.343544	-2.308119
C	-0.360594	-5.744866	-3.539390
H	0.622903	-6.134908	-3.822834
H	-1.062140	-5.967442	-4.349507
H	-0.689555	-6.307607	-2.655793
O	1.150558	2.253610	-0.107881
C	1.072492	3.370128	0.442690
H	0.504027	3.458746	1.376665
H	-2.684925	-1.024227	-1.225150
C	1.702136	4.540423	-0.121728
C	1.635221	5.757324	0.582702
C	2.367739	4.468367	-1.363726
C	2.238783	6.893792	0.055585
H	1.114243	5.800663	1.535862
C	2.962927	5.609541	-1.883076
H	2.389210	3.522561	-1.896801
C	2.900727	6.816925	-1.173932
H	2.194349	7.835316	0.594029
H	3.474283	5.567539	-2.839792
H	3.369747	7.705943	-1.586594
C	1.600726	-0.056515	0.933601
C	2.741668	-0.325631	0.163381
C	1.515689	-0.661468	2.197483
C	3.736770	-1.193279	0.622412
H	2.846941	0.134999	-0.813611
C	2.518027	-1.514961	2.665399
H	0.658672	-0.453338	2.830143
C	3.633540	-1.794024	1.875673
H	4.410840	-2.457862	2.234530
C	2.362594	-2.184308	4.004842
C	4.885466	-1.517939	-0.291299
F	1.618991	-3.312566	3.916113
F	3.555118	-2.541679	4.533196
F	5.887951	-2.161622	0.343794
F	1.746949	-1.380874	4.899974
F	5.409391	-0.408477	-0.858016
F	4.484161	-2.319790	-1.312880

NTOB-benzaldehyde complex 109

B3LYP/6-31G(d) = -2780.343209
B3LYP-D3(BJ)/def2-TZVPP/IEFPCM(propanonitrile) = -2781.599693
B3LYP-D3(BJ)/def2-TZVPP/IEFPCM(propanonitrile)//B3LYP-D3(BJ)/6-31G(d) Free Energy (Quasiharmonic) = -2781.115048

Frequencies (Top 3 out of 213)

1. 8.5029 cm^{-1}
2. 10.5290 cm^{-1}
3. 11.5662 cm^{-1}

B3LYP/6-31G(d) Molecular Geometry in Cartesian Coordinates

B	0.974277	0.032207	-0.665186
O	0.686893	-0.059296	-2.088829
N	-0.298386	-0.443909	0.005995
C	-1.309765	-0.755556	-1.026206
S	-0.279714	-1.393098	1.399599
C	-0.552267	-0.535831	-2.339701
O	-0.974512	-0.712184	-3.456500
H	-2.309174	1.144388	-1.361004
H	-1.613679	-1.807612	-0.972572
C	-2.566392	0.156387	-0.949175
C	-3.813292	-0.389688	-1.592253
C	-5.158783	-0.147370	-1.125056
C	-6.042742	-0.833632	-2.003791
N	-5.261867	-1.455393	-2.954462
C	-3.929261	-1.183227	-2.708224
H	-3.153602	-1.553916	-3.362143
C	-7.940521	-0.079328	-0.770865
C	-5.705395	0.585382	-0.056678
H	-8.090161	-1.344427	-2.518574
H	-5.603583	-2.020463	-3.715882
C	-7.085296	0.614217	0.110352
H	-9.015340	-0.038210	-0.616500
H	-5.058769	1.125327	0.630157
C	-7.431471	-0.811698	-1.837251
H	-7.516467	1.187823	0.926964
C	-1.878352	-1.097572	2.160497
O	0.744563	-0.811772	2.273211
O	-0.241224	-2.816551	1.036240
C	-4.361270	-0.647472	3.371910
C	-2.891498	-2.049132	2.029201
C	-2.077552	0.063158	2.909490
C	-3.317885	0.279863	3.507585
C	-4.124713	-1.815120	2.631994
H	-2.705056	-2.959783	1.470453
H	-1.267430	0.774792	3.030368
H	-3.477208	1.180689	4.095219
H	-4.918032	-2.550475	2.524831
C	-5.715534	-0.398046	3.990190
H	-6.049973	-1.259398	4.579896
H	-6.468552	-0.227372	3.210466
H	-5.704258	0.477558	4.646453
H	-2.768000	0.324552	0.112040
C	2.457660	-0.437629	-0.299476
C	3.171588	0.030124	0.815163
C	3.114708	-1.325901	-1.162478
C	4.484389	-0.374576	1.056413
H	2.696070	0.709926	1.511356
C	4.427451	-1.741277	-0.918710
H	2.599532	-1.699529	-2.041280
C	5.121283	-1.265799	0.191211
H	6.141205	-1.580701	0.378839
C	5.118505	-2.644079	-1.904223
C	5.204442	0.109196	2.285901
F	5.669843	-1.939834	-2.923790
F	4.265162	-3.529943	-2.461988
F	6.120104	-3.348311	-1.331166
F	6.543424	0.168803	2.093113
F	4.998822	-0.700066	3.347930
F	4.797375	1.348007	2.652183
O	1.018309	1.699690	-0.349813
C	1.072842	2.555672	-1.254416
C	1.151345	3.974940	-0.988073
C	1.191264	4.471329	0.330770
C	1.193686	4.861786	-2.080303
C	1.273018	5.840039	0.544768
H	1.160851	3.772612	1.160403
C	1.273899	6.232438	-1.857462
H	1.163803	4.469234	-3.093587
C	1.313716	6.717925	-0.547165
H	1.307349	6.231001	1.556997
H	1.306779	6.920619	-2.696272
H	1.378271	7.788364	-0.372798
H	1.059581	2.214499	-2.296861

NTOB-benzaldehyde complex 110

B3LYP/6-31G(d) = -2780.342228
B3LYP-D3(BJ)/def2-TZVPP/IEFPCM(propanonitrile) = -2781.602016
B3LYP-D3(BJ)/def2-TZVPP/IEFPCM(propanonitrile)//B3LYP-D3(BJ)/6-31G(d) Free Energy (Quasiharmonic) = -2781.114984

Frequencies (Top 3 out of 213)

1. 11.8158 cm^{-1}
2. 14.7955 cm^{-1}
3. 22.1200 cm^{-1}

B3LYP/6-31G(d) Molecular Geometry in Cartesian Coordinates

B	1.223921	0.084598	-0.698451
O	1.775631	-0.810648	-1.696885
N	1.420819	-0.678052	0.617254
C	2.189230	-1.918471	0.357026
S	1.646685	0.001191	2.130098
C	2.340015	-1.913118	-1.162993
O	2.878101	-2.770585	-1.821317
H	1.588378	-3.261448	1.926581
H	3.200728	-1.861187	0.783093
C	1.548350	-3.246943	0.835959
C	0.140388	-3.484919	0.365498
C	-1.058541	-3.413984	1.166114
C	-2.158581	-3.688652	0.307340
N	-1.643114	-3.939795	-0.947608
C	-0.266499	-3.806965	-0.908817
H	0.326346	-3.972273	-1.798290
C	-3.697826	-3.429558	2.108984
C	-1.308941	-3.159585	2.528060
H	-4.309150	-3.884560	0.084571
H	-2.189534	-4.023142	-1.791313
C	-2.621833	-3.170599	2.984467
H	-4.713569	-3.426781	2.495255
H	-0.488882	-2.943816	3.207391
C	-3.481919	-3.692229	0.761813
H	-2.825901	-2.976973	4.034245
C	0.221706	1.061251	2.324733
O	2.853457	0.866438	2.139539
O	1.606683	-1.090520	3.111144
C	-1.998419	2.723938	2.683487
C	-1.058764	0.502598	2.333938
C	0.412583	2.429109	2.523739
C	-0.699583	3.250471	2.699013
C	-2.155889	1.339960	2.511756
H	-1.198965	-0.563999	2.195027
H	1.417795	2.835239	2.543044
H	-0.556749	4.318097	2.845027
H	-3.153780	0.909108	2.510529
C	-3.200529	3.628182	2.805053
H	-4.040938	3.117187	3.285896
H	-3.534801	3.950707	1.811138
H	-2.969872	4.529060	3.382801
O	2.157520	1.454652	-0.607527
C	3.321906	1.771447	-0.275361
H	3.409516	2.652151	0.369713
H	2.217456	-4.037037	0.473339
C	4.568920	1.175157	-0.706003
C	5.730273	1.543210	0.005283
C	4.666961	0.301321	-1.809005
C	6.963552	1.014227	-0.352785
H	5.646010	2.218743	0.852197
C	5.907880	-0.210434	-2.168976
H	3.780097	0.046194	-2.376331
C	7.050496	0.137749	-1.439886
H	7.854752	1.281653	0.206500
H	5.985356	-0.883522	-3.016924
H	8.015897	-0.271399	-1.724849
C	-0.220258	0.648902	-1.103783
C	-0.625860	1.983840	-0.980355
C	-1.155668	-0.263345	-1.615518
C	-1.915996	2.387926	-1.333433
H	0.064661	2.720604	-0.588550
C	-2.442776	0.136406	-1.981108
H	-0.873839	-1.303522	-1.735120
C	-2.834173	1.467287	-1.838146
H	-3.834625	1.780817	-2.114970
C	-3.436463	-0.880506	-2.472291
C	-2.319693	3.831302	-1.212246
F	-4.340375	-0.340134	-3.318114
F	-2.834759	-1.905480	-3.130338
F	-1.552064	4.506416	-0.326392
F	-4.137808	-1.439097	-1.456541
F	-3.607128	3.957490	-0.797958
F	-2.230345	4.488409	-2.391304

NTOB-benzaldehyde complex 111

B3LYP/6-31G(d) = -2780.343507
B3LYP-D3(BJ)/def2-TZVPP/IEFPCM(propanonitrile) = -2781.599079
B3LYP-D3(BJ)/def2-TZVPP/IEFPCM(propanonitrile)//B3LYP-D3(BJ)/6-31G(d) Free Energy (Quasiharmonic) = -2781.114685

Frequencies (Top 3 out of 213)

1. 6.9264 cm^{-1}
2. 10.5045 cm^{-1}
3. 15.0853 cm^{-1}

B3LYP/6-31G(d) Molecular Geometry in Cartesian Coordinates

B	-0.367869	-0.802586	0.666755
O	0.189352	-1.644669	1.703040
N	0.644111	-0.826872	-0.435177
C	1.861594	-1.539113	0.013205
S	0.345725	-0.699384	-2.087472
C	1.434108	-2.081134	1.384880
O	2.080158	-2.782220	2.123748
H	2.965682	0.104815	0.903135
H	2.091570	-2.384242	-0.643725
C	3.107404	-0.620217	0.086342
C	4.448982	-1.297495	0.181854
C	5.672036	-0.754316	-0.364020
C	6.714494	-1.676135	-0.070008
N	6.141897	-2.721980	0.622064
C	4.787102	-2.491939	0.770884
H	4.159653	-3.189560	1.304701
C	8.320430	-0.289920	-1.159863
C	5.989857	0.417364	-1.072419
H	8.821869	-2.176736	-0.228437
H	6.625549	-3.544017	0.947520
C	7.304666	0.639257	-1.463372
H	9.339977	-0.090997	-1.478889
H	5.216986	1.142188	-1.316614
C	8.039425	-1.458669	-0.461116
H	7.557075	1.541478	-2.014122
C	-0.100616	1.028679	-2.311704
O	-0.826948	-1.494707	-2.463322
O	1.638264	-0.937162	-2.741382
C	-0.801346	3.712862	-2.710910
C	0.862688	2.025892	-2.143345
C	-1.403162	1.353522	-2.689565
C	-1.746862	2.692676	-2.875775
C	0.506196	3.357521	-2.341800
H	1.878900	1.764175	-1.867111
H	-2.137622	0.568005	-2.822954
H	-2.769781	2.940886	-3.145702
H	1.256932	4.134660	-2.217711
C	-1.171315	5.156415	-2.956020
H	-0.533764	5.839180	-2.384569
H	-2.214030	5.354256	-2.686570
H	-1.055750	5.415374	-4.016739
H	3.093119	-0.043107	-0.842980
C	-1.938272	-0.953380	0.442337

C	-2.772291	0.110744	0.080274
C	-2.530339	-2.216906	0.596827
C	-4.134874	-0.082485	-0.164070
H	-2.360816	1.108147	-0.028055
C	-3.894723	-2.409858	0.376612
H	-1.918067	-3.063809	0.888987
C	-4.705716	-1.344411	-0.017978
H	-5.761502	-1.497646	-0.207703
C	-4.513721	-3.760682	0.620796
C	-4.954392	1.075340	-0.660232
F	-5.592329	-3.969255	-0.167071
F	-4.936139	-3.887017	1.902450
F	-3.642246	-4.765505	0.394717
F	-6.280241	0.865804	-0.530841
F	-4.721245	1.320898	-1.978345
F	-4.657103	2.221313	-0.003296
O	-0.226852	0.802099	1.322835
C	0.026800	1.017665	2.522722
C	0.177211	2.350130	3.067904
C	0.430282	2.488105	4.445341
C	0.075013	3.491540	2.246764
C	0.574322	3.755523	5.000315
H	0.510558	1.601343	5.069014
C	0.221865	4.752324	2.808225
H	-0.109869	3.366291	1.184477
C	0.469319	4.883547	4.181590
H	0.768235	3.867400	6.062463
H	0.145749	5.637637	2.184373
H	0.582700	5.873675	4.614278
H	0.147266	0.158084	3.194580

NTOB-benzaldehyde complex 112

B3LYP/6-31G(d) = -2780.339958
B3LYP-D3(BJ)/def2-TZVPP/IEFPCM(propanonitrile) = -2781.600687
B3LYP-D3(BJ)/def2-TZVPP/IEFPCM(propanonitrile)//B3LYP-D3(BJ)/6-31G(d) Free Energy (Quasiharmonic) = -2781.114681

Frequencies (Top 3 out of 213)

1. 11.4742 cm^{-1}
2. 13.0669 cm^{-1}
3. 14.7373 cm^{-1}

B3LYP/6-31G(d) Molecular Geometry in Cartesian Coordinates

B	0.811639	-1.276409	-0.822581
O	1.258436	-1.035617	-2.180548
N	-0.616640	-1.772952	-0.985449
C	-0.948422	-1.845130	-2.420421
S	-1.382145	-2.811171	0.082951
C	0.363656	-1.463067	-3.101589
O	0.581698	-1.470664	-4.287662
H	-2.991646	-1.130578	-2.368073
H	-1.211626	-2.869737	-2.703736
C	-2.074533	-0.888431	-2.914247
C	-1.779363	0.586337	-2.804245
C	-2.345868	1.534601	-1.872489
C	-1.751175	2.799089	-2.140242
N	-0.884029	2.623665	-3.197425
C	-0.904401	1.300936	-3.591957
H	-0.288851	0.963345	-4.413753
C	-3.016701	3.831411	-0.405145
C	-3.311922	1.451799	-0.854175
H	-1.583444	4.900774	-1.623325
H	-0.273700	3.332328	-3.573158
C	-3.637518	2.594316	-0.135005
H	-3.287284	4.705951	0.180473
H	-3.800628	0.509027	-0.631344
C	-2.066775	3.950804	-1.411743
H	-4.382009	2.534587	0.654390
C	-2.896756	-2.022114	0.644178
O	-0.516545	-2.912743	1.266039
O	-1.789297	-4.014715	-0.656364
C	-5.280495	-0.910883	1.610964
C	-2.854937	-1.129125	1.717198
C	-4.112215	-2.375598	0.055247
C	-5.292979	-1.811882	0.536761
C	-4.045773	-0.581053	2.188644
H	-1.912180	-0.875125	2.189253
H	-4.129281	-3.100176	-0.751883
H	-6.240004	-2.087036	0.078754
H	-4.014150	0.113180	3.024588
C	-6.567954	-0.339761	2.155990
H	-6.990843	-0.994076	2.929912
H	-7.323869	-0.233911	1.371030
H	-6.409702	0.642792	2.612168
O	1.705794	-2.617902	-0.339728
C	2.872547	-2.911637	-0.028040
H	2.980616	-3.886069	0.469619
H	-2.253010	-1.160833	-3.961777
C	4.100906	-2.166683	-0.216409
C	5.233125	-2.644953	0.477462
C	4.215274	-1.034140	-1.050548
C	6.449300	-1.981895	0.374934
H	5.143103	-3.527757	1.105580
C	5.440598	-0.387746	-1.158450
H	3.362889	-0.702761	-1.630392
C	6.550136	-0.851711	-0.442615
H	7.315739	-2.342434	0.920554
H	5.535952	0.479272	-1.804736
H	7.501919	-0.335142	-0.531163
C	1.127917	-0.094855	0.209539
C	1.063533	-0.241659	1.602074
C	1.385574	1.190171	-0.293818
C	1.222572	0.855256	2.453350
H	0.851309	-1.217448	2.027002
C	1.564022	2.283973	0.556097
H	1.423580	1.342376	-1.367497
C	1.477493	2.125164	1.939349
H	1.595674	2.974344	2.601345
C	1.905361	3.628539	-0.025119
C	1.047081	0.642107	3.931244
F	1.586048	4.642762	0.805894
F	3.233033	3.736193	-0.284732
F	-0.240845	0.338404	4.233989
F	1.269405	3.853224	-1.200941
F	1.378662	1.731888	4.656047
F	1.799663	-0.387922	4.381681

NTOB-benzaldehyde complex 113

B3LYP/6-31G(d) = -2780.342772
B3LYP-D3(BJ)/def2-TZVPP/IEFPCM(propanonitrile) = -2781.597667
B3LYP-D3(BJ)/def2-TZVPP/IEFPCM(propanonitrile)//B3LYP-D3(BJ)/6-31G(d) Free Energy (Quasiharmonic) = -2781.114606

Frequencies (Top 3 out of 213)

1. 3.1186 cm^{-1}
2. 6.0842 cm^{-1}
3. 9.7791 cm^{-1}

B3LYP/6-31G(d) Molecular Geometry in Cartesian Coordinates

B	-0.913912	-0.521814	0.547846
O	-0.640987	-1.299226	1.739997
N	0.324747	-0.680472	-0.289625
C	1.337272	-1.466674	0.447479
S	0.412789	-0.633860	-1.965648

C	0.588672	-1.864765	1.722366
O	1.002963	-2.550223	2.624630
H	2.429055	0.020095	1.599457
H	1.595368	-2.370778	-0.114894
C	2.637578	-0.680656	0.776600
C	3.853680	-1.520092	1.068093
C	5.201761	-1.185114	0.669610
C	6.052278	-2.225579	1.136976
N	5.250187	-3.138458	1.787920
C	3.936405	-2.712875	1.745987
H	3.149572	-3.282198	2.218141
C	7.969812	-1.144603	0.219060
C	5.775790	-0.111121	-0.033555
H	8.066099	-3.025744	1.282341
H	5.564520	-3.996701	2.212674
C	7.148086	-0.100354	-0.252236
H	9.039849	-1.109091	0.033157
H	5.156275	0.701414	-0.404505
C	7.433657	-2.219887	0.918322
H	7.599589	0.726441	-0.794414
C	1.679740	0.584795	-2.346626
O	-0.859492	-0.086342	-2.440335
O	0.911735	-1.928268	-2.444775
C	3.608900	2.495338	-3.035346
C	1.448184	1.933655	-2.062411
C	2.855128	0.179477	-2.976113
C	3.812493	1.138152	-3.312650
C	2.413519	2.875419	-2.402508
H	0.522681	2.236687	-1.583155
H	3.011855	-0.871088	-3.194786
H	4.731705	0.822196	-3.799118
H	2.237546	3.925611	-2.181207
C	4.632692	3.532521	-3.431173
H	4.722373	4.318234	-2.673047
H	4.351619	4.021947	-4.372983
H	5.620807	3.085288	-3.576693
H	2.857348	-0.053709	-0.091442
C	-2.429533	-0.626030	0.062916
C	-3.103850	0.390083	-0.632582
C	-3.162033	-1.771084	0.403076
C	-4.449542	0.265813	-0.973324
H	-2.574927	1.291829	-0.915927
C	-4.509399	-1.906434	0.050531
H	-2.684321	-2.565260	0.967747
C	-5.162012	-0.887600	-0.637304
H	-6.211017	-0.980207	-0.892758
C	-5.240215	-3.176564	0.393727
C	-5.132272	1.359651	-1.748960
F	-4.976522	-4.162711	-0.493914
F	-6.581500	-3.003748	0.404179
F	-4.884010	-3.644767	1.612039
F	-6.453067	1.430501	-1.460716
F	-5.033383	1.174790	-3.083593
F	-4.598229	2.575606	-1.481144
O	-0.836882	1.136473	1.118178
C	-0.839245	1.415641	2.331359
C	-0.824208	2.773460	2.835537
C	-0.797102	2.969870	4.228710
C	-0.843587	3.881351	1.965058
C	-0.785736	4.260609	4.747713
H	-0.784554	2.109313	4.892830
C	-0.834971	5.166187	2.489754
H	-0.871046	3.712472	0.893632
C	-0.805015	5.354772	3.878267
H	-0.763831	4.416532	5.821735
H	-0.853860	6.025089	1.825919
H	-0.798711	6.363022	4.282954
H	-0.853427	0.591409	3.056093

NTOB-benzaldehyde complex 114

B3LYP/6-31G(d) = -2780.342773
B3LYP-D3(BJ)/def2-TZVPP/IEFPCM(propanonitrile) = -2781.598577
B3LYP-D3(BJ)/def2-TZVPP/IEFPCM(propanonitrile)//B3LYP-D3(BJ)/6-31G(d) Free Energy (Quasiharmonic) = -2781.114416

Frequencies (Top 3 out of 213)

1. 6.6890 cm^{-1}
2. 10.4443 cm^{-1}
3. 15.0199 cm^{-1}

B3LYP/6-31G(d) Molecular Geometry in Cartesian Coordinates

B	-0.090349	-0.534855	0.599620
O	0.773022	-1.113619	1.600966
N	0.772011	-0.381458	-0.619165
C	2.155618	-0.803612	-0.308620
S	0.272966	-0.460443	-2.223661
C	2.058917	-1.215831	1.162144
O	2.967822	-1.553467	1.874798
H	3.323598	0.919388	0.361441
H	2.434718	-1.685293	-0.895015
C	3.240596	0.293093	-0.535049
C	4.589581	-0.239695	-0.929981
C	5.747797	-0.484090	-0.103823
C	6.779406	-0.960759	-0.960675
N	6.258194	-1.010656	-2.237944
C	4.947940	-0.577110	-2.213378
H	4.353285	-0.542485	-3.116327
C	8.297965	-1.129412	0.869088
C	6.022922	-0.346615	1.268338
H	8.829944	-1.649022	-1.162352
H	6.751592	-1.322751	-3.059426
C	7.289682	-0.665654	1.739357
H	9.278622	-1.374152	1.268406
H	5.245513	-0.022631	1.952996
C	8.056174	-1.285889	-0.490386
H	7.508256	-0.567112	2.799369
C	-0.636554	1.072248	-2.459577
O	-0.674377	-1.560045	-2.432411
O	1.504279	-0.399898	-3.025485
C	-2.061058	3.448080	-2.881519
C	-2.007336	1.019739	-2.710486
C	0.037142	2.295254	-2.427873
C	-0.678267	3.471748	-2.637254
C	-2.711236	2.207651	-2.908932
H	-2.514599	0.062436	-2.734731
H	1.106601	2.326793	-2.246088
H	-0.154382	4.424728	-2.619591
H	-3.783626	2.162095	-3.078319
C	-2.823851	4.725748	-3.139367
H	-2.334900	5.588545	-2.675051
H	-3.848146	4.665053	-2.757036
H	-2.891421	4.930772	-4.215904
H	2.866301	0.931026	-1.338885
C	-1.580028	-1.100981	0.574256
C	-1.795926	-2.458272	0.861330
C	-2.699670	-0.321265	0.261848
C	-3.074902	-3.014545	0.815510
H	-0.953797	-3.091539	1.120810
C	-3.979357	-0.879223	0.192040
H	-2.582100	0.736996	0.056559
C	-4.175979	-2.230109	0.467831
H	-5.166608	-2.665803	0.413440
C	-5.123466	-0.016071	-0.259697
C	-3.284224	-4.455677	1.198809
F	-6.325587	-0.551180	0.035454
F	-5.098804	0.177407	-1.606786
F	-5.077475	1.215867	0.300150
F	-4.342516	-4.998128	0.555443

F	-3.526269	-4.584742	2.525980
F	-2.204499	-5.215502	0.921484
O	-0.313464	1.103995	1.147901
C	0.010038	1.472277	2.292310
C	-0.140551	2.836942	2.752380
C	0.225786	3.143512	4.075984
C	-0.641261	3.844285	1.902861
C	0.088159	4.444127	4.550170
H	0.613810	2.359897	4.721848
C	-0.773922	5.139705	2.383333
H	-0.908992	3.592381	0.881496
C	-0.411460	5.438092	3.703988
H	0.368048	4.685310	5.570855
H	-1.158117	5.922841	1.736861
H	-0.518557	6.454195	4.073474
H	0.433851	0.729118	2.979977

NTOB-benzaldehyde complex 115

B3LYP/6-31G(d) = -2780.343209
B3LYP-D3(BJ)/def2-TZVPP/IEFPCM(propanonitrile) = -2781.599402
B3LYP-D3(BJ)/def2-TZVPP/IEFPCM(propanonitrile)//B3LYP-D3(BJ)/6-31G(d) Free Energy (Quasiharmonic) = -2781.114286

Frequencies (Top 3 out of 213)

1. 5.3670 cm^{-1}
2. 9.4438 cm^{-1}
3. 15.0133 cm^{-1}

B3LYP/6-31G(d) Molecular Geometry in Cartesian Coordinates

B	0.926655	-0.042752	-0.919034
O	0.633404	-0.546407	-2.251483
N	-0.461975	0.020388	-0.276417
C	-1.381461	-0.867688	-1.031460
S	-0.698663	0.421695	1.311777
C	-0.616256	-1.021695	-2.360386
O	-1.056348	-1.496104	-3.380727
H	-2.803191	0.432546	-2.023128
H	-1.438551	-1.852081	-0.546212
C	-2.823233	-0.342901	-1.246007
C	-3.847482	-1.407222	-1.551662
C	-5.079836	-1.607406	-0.823167
C	-5.762651	-2.695063	-1.436140
N	-4.975831	-3.123325	-2.484096
C	-3.832147	-2.352246	-2.550320
H	-3.079692	-2.527166	-3.304137
C	-7.563633	-2.515516	0.116798
C	-5.679528	-0.976468	0.281413
H	-7.503133	-3.993601	-1.461341
H	-5.185793	-3.896240	-3.095951
C	-6.908785	-1.433568	0.740033
H	-8.523949	-2.851819	0.498317
H	-5.189730	-0.140210	0.774198
C	-6.999930	-3.159490	-0.978510
H	-7.376283	-0.951781	1.594847
C	-1.996171	1.665683	1.336080
O	0.547875	1.103319	1.720331
O	-1.190794	-0.725037	2.087246
C	-3.941012	3.673306	1.525780
C	-1.926608	2.776423	0.491899
C	-3.014555	1.550507	2.282144
C	-3.978417	2.555738	2.369248
C	-2.898977	3.766975	0.589683
H	-1.132467	2.853788	-0.243751
H	-3.047650	0.682698	2.931955
H	-4.772834	2.467324	3.106071
H	-2.851545	4.628416	-0.072225
C	-5.009127	4.737829	1.599583
H	-5.810500	4.539883	0.875581
H	-4.603918	5.729507	1.372103
H	-5.468547	4.776436	2.592257
H	-3.138230	0.158994	-0.329379
C	2.166317	-0.834660	-0.261210
C	3.412060	-0.805084	-0.910719
C	2.053854	-1.654517	0.871886
C	4.503573	-1.538937	-0.441381
H	3.537884	-0.204670	-1.807980
C	3.141516	-2.396864	1.341223
H	1.109100	-1.732373	1.399780
C	4.373883	-2.340441	0.692049
H	5.216489	-2.913922	1.060004
C	3.001967	-3.216199	2.597820
C	5.803841	-1.509353	-1.198329
F	1.759196	-3.721814	2.734005
F	3.252546	-2.476569	3.703656
F	3.869290	-4.254860	2.618112
F	6.857327	-1.812607	-0.407444
F	6.040696	-0.287839	-1.734537
F	5.812307	-2.391606	-2.222698
O	1.366732	1.493761	-1.174424
C	2.083468	2.137151	-0.371519
C	2.365082	3.540936	-0.556113
C	1.882164	4.246901	-1.677404
C	3.148021	4.197973	0.411881
C	2.183457	5.593952	-1.819694
H	1.287358	3.721819	-2.417823
C	3.442356	5.549292	0.263461
H	3.511477	3.644898	1.273805
C	2.961029	6.243052	-0.850509
H	1.820034	6.144722	-2.681869
H	4.043040	6.061534	1.008425
H	3.193517	7.297886	-0.967882
H	2.511204	1.619595	0.488573

NTOB-benzaldehyde complex 116

B3LYP/6-31G(d) = -2780.34725
B3LYP-D3(BJ)/def2-TZVPP/IEFPCM(propanonitrile) = -2781.598225
B3LYP-D3(BJ)/def2-TZVPP/IEFPCM(propanonitrile)//B3LYP-D3(BJ)/6-31G(d) Free Energy (Quasiharmonic) = -2781.113786

Frequencies (Top 3 out of 213)

1. 8.5169 cm^{-1}
2. 8.9835 cm^{-1}
3. 10.8238 cm^{-1}

B3LYP/6-31G(d) Molecular Geometry in Cartesian Coordinates

B	-0.504302	0.674772	0.703055
O	-0.058187	0.910502	2.056997
N	0.735609	0.101175	0.031829
C	1.897531	0.190560	0.944176
S	0.909961	-0.421658	-1.525702
C	1.229158	0.559843	2.270182
O	1.756779	0.602980	3.352864
H	2.533420	2.269786	0.875775
H	2.402311	-0.776960	1.034592
C	2.948381	1.294686	0.591598
C	4.285990	1.070062	1.235329
C	5.406402	0.381764	0.637529
C	6.460608	0.382478	1.592461
N	5.991395	1.041347	2.711183
C	4.688305	1.444129	2.493715
H	4.130940	1.948015	3.269944
C	7.887357	-0.798913	0.091697
C	5.621421	-0.229734	-0.610754
H	8.499695	-0.190831	2.075766

H	6.503372	1.176447	3.568679
C	6.856151	-0.812347	-0.870109
H	8.842027	-1.263145	-0.141140
H	4.830429	-0.249272	-1.356006
C	7.704828	-0.201456	1.333989
H	7.033712	-1.288258	-1.831006
C	0.949469	-2.217464	-1.442758
O	2.211131	-0.012546	-2.072474
O	-0.310585	0.016148	-2.231921
C	0.994089	-5.019915	-1.434874
C	0.104143	-2.919730	-0.580040
C	1.819644	-2.897131	-2.296894
C	1.834706	-4.290797	-2.285761
C	0.135276	-4.311571	-0.581179
H	-0.561146	-2.390325	0.093005
H	2.483612	-2.337454	-2.946129
H	2.518006	-4.819602	-2.945505
H	-0.516170	-4.857166	0.097007
C	0.997286	-6.529645	-1.444483
H	0.865745	-6.936967	-0.436352
H	0.176701	-6.919387	-2.061200
H	1.931801	-6.926315	-1.853226
H	3.062316	1.303035	-0.493073
C	-1.994279	0.052640	0.689352
C	-2.661290	-0.528314	-0.403193
C	-2.711585	0.128388	1.900908
C	-3.974845	-1.000303	-0.291376
H	-2.152630	-0.625442	-1.355548
C	-4.018750	-0.345389	2.013828
H	-2.233039	0.565215	2.770078
C	-4.664315	-0.911760	0.913939
H	-5.681763	-1.274826	0.996923
C	-4.720451	-0.299933	3.345817
C	-4.650646	-1.555481	-1.516512
F	-4.460781	-1.403918	4.084229
F	-6.064184	-0.231920	3.202529
F	-4.336940	0.765019	4.081899
F	-5.004027	-0.567630	-2.376244
F	-5.776809	-2.238138	-1.215766
F	-3.839228	-2.390955	-2.200763
O	-0.609466	2.238388	0.077900
C	-1.286505	2.561526	-0.919222
C	-1.206641	3.873803	-1.520573
C	-0.347156	4.864956	-1.005388
C	-2.010793	4.146950	-2.642756
C	-0.300185	6.112624	-1.610970
H	0.267159	4.634896	-0.141162
C	-1.957557	5.399842	-3.244638
H	-2.665246	3.373499	-3.036196
C	-1.104013	6.378754	-2.727917
H	0.359694	6.881788	-1.221528
H	-2.573928	5.614921	-4.111898
H	-1.061648	7.357200	-3.198429
H	-1.972305	1.831512	-1.359115

NTOB-benzaldehyde complex 117

B3LYP/6-31G(d) = -2780.340346
B3LYP-D3(BJ)/def2-TZVPP/IEFPCM(propanonitrile) = -2781.598259
B3LYP-D3(BJ)/def2-TZVPP/IEFPCM(propanonitrile)//B3LYP-D3(BJ)/6-31G(d) Free Energy (Quasiharmonic) = -2781.113637

Frequencies (Top 3 out of 213)

1. 5.8095 cm^{-1}
2. 9.4486 cm^{-1}
3. 14.7142 cm^{-1}

B3LYP/6-31G(d) Molecular Geometry in Cartesian Coordinates

B	0.651651	0.141680	0.727255
O	0.053083	0.791322	1.879563
N	-0.561276	-0.126419	-0.169972
C	-1.639268	0.816117	0.216494
S	-0.474508	-0.820346	-1.664529
C	-1.223217	1.163679	1.656566
O	-1.919795	1.692670	2.484797
H	-3.290774	-0.386450	0.959183
H	-1.567235	1.730035	-0.389902
C	-3.092839	0.291658	0.122063
C	-4.131920	1.380419	0.060670
C	-4.995313	1.870928	1.108766
C	-5.835084	2.862986	0.530878
N	-5.484575	2.970628	-0.799927
C	-4.468485	2.077208	-1.075443
H	-4.060059	1.998744	-2.074632
C	-6.948498	3.212522	2.608689
C	-5.151906	1.568730	2.473008
H	-7.443735	4.297691	0.803921
H	-5.899551	3.603558	-1.465240
C	-6.124328	2.235712	3.205595
H	-7.698092	3.720193	3.209916
H	-4.501095	0.844028	2.951080
C	-6.813523	3.541581	1.265722
H	-6.247760	2.011286	4.261695
C	-1.651240	-2.180954	-1.654167
O	0.872860	-1.423821	-1.737118
O	-0.912737	0.114188	-2.712113
C	-3.374321	-4.388588	-1.751443
C	-2.434428	-2.394820	-2.788247
C	-1.707730	-3.061423	-0.571304
C	-2.568590	-4.153483	-0.626293
C	-3.289142	-3.496406	-2.827516
H	-2.375763	-1.701334	-3.620163
H	-1.100160	-2.881306	0.309661
H	-2.620267	-4.835029	0.219515
H	-3.901626	-3.662490	-3.710244
C	-4.329180	-5.557786	-1.785025
H	-5.277904	-5.304353	-1.293797
H	-3.916688	-6.427923	-1.263245
H	-4.562562	-5.855371	-2.812130
H	-3.175477	-0.308643	-0.787744
C	1.951633	0.938198	0.206268
C	3.024623	1.144158	1.090132
C	2.035832	1.543694	-1.056303
C	4.141343	1.899137	0.725874
H	2.982635	0.729123	2.093838
C	3.146462	2.310517	-1.421470
H	1.224069	1.437821	-1.768471
C	4.208877	2.487432	-0.536700
H	5.065481	3.088235	-0.818598
C	3.222684	2.904247	-2.803826
C	5.297911	2.029370	1.679520
F	4.014471	4.000781	-2.837210
F	2.007107	3.266912	-3.263889
F	3.735856	2.024035	-3.695332
F	6.141367	0.970194	1.580957
F	4.889275	2.078158	2.966239
F	6.032607	3.136962	1.441866
O	1.118121	-1.302835	1.290060
C	2.026535	-1.979229	0.751673
C	2.356697	-3.310397	1.201608
C	3.362328	-4.016855	0.515062
C	1.704464	-3.896996	2.305825
C	3.709740	-5.299934	0.924570
H	3.855932	-3.556136	-0.336393
C	2.059874	-5.175890	2.710293
H	0.938669	-3.333611	2.829164
C	3.059506	-5.875471	2.019925
H	4.482200	-5.850309	0.396769
H	1.566840	-5.633864	3.562318

H	3.333230	-6.876466	2.341778
H	2.589117	-1.545704	-0.076758

NTOB-benzaldehyde complex 118

B3LYP/6-31G(d) = -2780.340296
B3LYP-D3(BJ)/def2-TZVPP/IEFPCM(propanonitrile) = -2781.59826
B3LYP-D3(BJ)/def2-TZVPP/IEFPCM(propanonitrile)//B3LYP-D3(BJ)/6-31G(d) Free Energy (Quasiharmonic) = -2781.113622

Frequencies (Top 3 out of 213)

1. 6.0321 cm^{-1}
2. 9.3580 cm^{-1}
3. 14.0773 cm^{-1}

B3LYP/6-31G(d) Molecular Geometry in Cartesian Coordinates

B	0.658762	-0.132672	-0.720844
O	0.066492	-0.797221	-1.867978
N	-0.556920	0.131155	0.174003
C	-1.625839	-0.824760	-0.204971
S	-0.477782	0.839207	1.662293
C	-1.206388	-1.179553	-1.642314
O	-1.897935	-1.721207	-2.466544
H	-3.288384	0.357305	-0.955837
H	-1.544941	-1.733153	0.408576
C	-3.084388	-0.313742	-0.114493
C	-4.112967	-1.412081	-0.046980
C	-4.971696	-1.916461	-1.092306
C	-5.802046	-2.913226	-0.508951
N	-5.450549	-3.010231	0.822411
C	-4.442933	-2.105763	1.092962
H	-4.035357	-2.017926	2.091719
C	-6.912104	-3.284650	-2.584757
C	-5.131143	-1.623230	-2.458165
H	-7.397048	-4.364564	-0.774057
H	-5.859392	-3.643527	1.491165
C	-6.097217	-2.303364	-3.187044
H	-7.656855	-3.802678	-3.183161
H	-4.487228	-0.895018	-2.940241
C	-6.774022	-3.605059	-1.240017
H	-6.222760	-2.085911	-4.244353
C	-1.668371	2.187543	1.639479
O	0.863304	1.457158	1.729874
O	-0.906660	-0.090284	2.718179
C	-3.413936	4.378237	1.716669
C	-1.733737	3.057509	0.548665
C	-2.453837	2.403676	2.771553
C	-3.319759	3.496782	2.800809
C	-2.605722	4.141171	0.593716
H	-1.124282	2.875596	-0.330627
H	-2.388213	1.718384	3.609716
H	-3.934010	3.664602	3.681979
H	-2.664281	4.814433	-0.258255
C	-4.380781	5.537802	1.739581
H	-5.327091	5.269859	1.251399
H	-3.977566	6.407074	1.209187
H	-4.616716	5.842841	2.763914
H	-3.173334	0.291253	0.791611
C	1.965720	-0.913363	-0.193283
C	3.041653	-1.115212	-1.074533
C	2.056642	-1.504807	1.075445
C	4.167260	-1.853014	-0.702477
H	3.002100	-0.696909	-2.076924
C	3.177910	-2.251376	1.449976
H	1.244555	-1.399393	1.787287
C	4.242029	-2.425916	0.566799
H	5.112008	-3.001460	0.860327
C	3.267371	-2.818344	2.842632
C	5.274480	-2.080138	-1.696207
F	2.057813	-3.183579	3.316085
F	3.777526	-1.917248	3.714893
F	4.069560	-3.906790	2.891703
F	6.457553	-2.322743	-1.088269
F	5.448666	-1.005882	-2.502764
F	5.021426	-3.135322	-2.502191
O	1.111521	1.311262	-1.295594
C	2.012220	2.001681	-0.762190
C	2.328752	3.332360	-1.223365
C	1.673044	3.901384	-2.334670
C	3.324203	4.056535	-0.540500
C	2.015034	5.180511	-2.749877
H	0.915259	3.324515	-2.854963
C	3.658029	5.339776	-0.960650
H	3.820320	3.609406	0.316728
C	3.004518	5.897796	-2.063110
H	1.519316	5.624972	-3.607482
H	4.422429	5.903870	-0.435597
H	3.267699	6.898899	-2.393309
H	2.578202	1.581298	0.070665

NTOB-benzaldehyde complex 119

B3LYP/6-31G(d) = -2780.345057
B3LYP-D3(BJ)/def2-TZVPP/IEFPCM(propanonitrile) = -2781.597757
B3LYP-D3(BJ)/def2-TZVPP/IEFPCM(propanonitrile)//B3LYP-D3(BJ)/6-31G(d) Free Energy (Quasiharmonic) = -2781.113292

Frequencies (Top 3 out of 213)

1. 7.4463 cm^{-1}
2. 8.9562 cm^{-1}
3. 13.1457 cm^{-1}

B3LYP/6-31G(d) Molecular Geometry in Cartesian Coordinates

B	0.035905	-0.575280	-0.814286
O	-0.660591	-0.397112	-2.068480
N	-0.863266	0.112318	0.194264
C	-2.209402	0.318960	-0.405235
S	-0.538459	0.359762	1.806501
C	-1.920048	0.047433	-1.892209
O	-2.695769	0.222881	-2.798744
H	-2.875067	2.240848	-1.164588
H	-2.904110	-0.455235	-0.055616
C	-2.898692	1.707998	-0.207559
C	-4.314213	1.634141	0.294437
C	-5.519063	1.395771	-0.468042
C	-6.608162	1.441940	0.447073
N	-6.078906	1.686878	1.698619
C	-4.706363	1.800885	1.600062
H	-4.097378	1.977792	2.474991
C	-8.168885	1.028419	-1.306419
C	-5.787480	1.150935	-1.827590
H	-8.753596	1.303013	0.758908
H	-6.609628	1.781064	2.550087
C	-7.105332	0.971995	-2.230439
H	-9.188567	0.884471	-1.654029
H	-4.972185	1.081494	-2.540644
C	-7.935324	1.261861	0.043985
H	-7.322662	0.780504	-3.277969
C	0.694999	1.665696	1.875886
O	0.119788	-0.850971	2.343013
O	-1.777704	0.820799	2.445517
C	2.582710	3.722060	2.126297
C	0.544178	2.837605	1.130231
C	1.774309	1.510106	2.745404
C	2.709609	2.538056	2.861532
C	1.484933	3.853740	1.261149

H	-0.285285	2.952366	0.441242
H	1.888975	0.590601	3.307312
H	3.557491	2.408165	3.528574
H	1.369697	4.762555	0.675621
C	3.611812	4.821516	2.232345
H	3.138085	5.807308	2.299316
H	4.263446	4.835841	1.349123
H	4.249832	4.690005	3.111435
O	-0.057597	-2.257884	-0.661058
C	-0.510034	-2.926564	0.290740
H	-0.868956	-2.406616	1.184019
H	-2.310664	2.289342	0.503172
C	-0.550663	-4.372809	0.264766
C	-0.099620	-5.100003	-0.855195
C	-1.057943	-5.047451	1.390986
C	-0.159794	-6.486405	-0.840412
H	0.286804	-4.561271	-1.714219
C	-1.113813	-6.437469	1.398406
H	-1.399253	-4.476167	2.250417
C	-0.665768	-7.152759	0.284172
H	0.184126	-7.055052	-1.698972
H	-1.502862	-6.963271	2.264759
H	-0.710153	-8.238383	0.289468
C	1.608941	-0.303765	-0.880437
C	2.127480	0.412135	-1.970945
C	2.515094	-0.742716	0.094693
C	3.492089	0.691712	-2.073307
H	1.456567	0.741674	-2.757608
C	3.879920	-0.459208	-0.001609
H	2.157751	-1.303530	0.952390
C	4.378107	0.260498	-1.085605
H	5.437973	0.469826	-1.167304
C	4.792793	-0.901685	1.108572
C	4.004413	1.513224	-3.226024
F	4.546140	-2.178672	1.481022
F	6.096531	-0.824000	0.766392
F	5.300199	1.241864	-3.502875
F	4.630391	-0.140520	2.221336
F	3.931458	2.840635	-2.962171
F	3.294270	1.299770	-4.354027

NTOB-benzaldehyde complex 120

B3LYP/6-31G(d) = -2780.346303
B3LYP-D3(BJ)/def2-TZVPP/IEFPCM(propanonitrile) = -2781.597321
B3LYP-D3(BJ)/def2-TZVPP/IEFPCM(propanonitrile)//B3LYP-D3(BJ)/6-31G(d) Free Energy (Quasiharmonic) = -2781.113173

Frequencies (Top 3 out of 213)

1. 5.9627 cm^{-1}
2. 7.3268 cm^{-1}
3. 13.1535 cm^{-1}

B3LYP/6-31G(d) Molecular Geometry in Cartesian Coordinates

B	0.051863	-0.622152	-0.634129
O	-0.764126	-0.696238	-1.828038
N	-0.789863	0.185008	0.331819
C	-2.193229	0.199903	-0.144322
S	-0.446497	0.376037	1.959068
C	-2.036696	-0.285612	-1.591264
O	-2.897997	-0.305216	-2.432852
H	-2.841229	2.079189	-1.041239
H	-2.799062	-0.539617	0.394775
C	-2.933156	1.571859	-0.074492
C	-4.378620	1.477067	0.330516
C	-5.550978	1.362589	-0.506016
C	-6.680432	1.335428	0.360065
N	-6.204966	1.415575	1.653734
C	-4.827557	1.503363	1.629212
H	-4.254233	1.569673	2.543572
C	-8.169676	1.180155	-1.494652
C	-5.764814	1.285680	-1.894402
H	-8.840704	1.227836	0.561850
H	-6.773120	1.428668	2.485976
C	-7.066157	1.198362	-2.372459
H	-9.175579	1.110154	-1.900249
H	-4.921840	1.266756	-2.576936
C	-7.992069	1.245563	-0.117751
H	-7.239513	1.135987	-3.443545
C	0.720171	1.744148	2.013212
O	0.275180	-0.802091	2.470159
O	-1.700181	0.779427	2.612824
C	2.510254	3.889782	2.234072
C	0.454641	2.939004	1.339370
C	1.864833	1.610956	2.798128
C	2.751489	2.682916	2.899978
C	1.347368	3.999345	1.455001
H	-0.429101	3.036757	0.718540
H	2.062531	0.676043	3.309507
H	3.649686	2.571486	3.501383
H	1.141457	4.927503	0.927278
C	3.486117	5.038493	2.322956
H	4.100472	5.103797	1.415431
H	4.165888	4.921962	3.172514
H	2.966942	5.997311	2.430721
O	0.066212	-2.185988	-0.031133
C	-0.343487	-3.193400	-0.640167
H	-0.700198	-3.078910	-1.671685
H	-2.411921	2.183001	0.663827
C	-0.368961	-4.498911	-0.022584
C	-0.770585	-5.608825	-0.789451
C	-0.005841	-4.658247	1.331469
C	-0.797709	-6.873349	-0.211528
H	-1.054404	-5.471335	-1.829829
C	-0.041356	-5.923941	1.899971
H	0.279653	-3.783122	1.907709
C	-0.432861	-7.027527	1.129692
H	-1.102934	-7.734942	-0.797057
H	0.231345	-6.058730	2.942112
H	-0.457346	-8.015350	1.581598
C	1.593921	-0.290557	-0.876327
C	2.594895	-0.607618	0.053167
C	1.984358	0.360868	-2.056325
C	3.930548	-0.266877	-0.176966
H	2.329348	-1.116921	0.974039
C	3.320026	0.694361	-2.292419
H	1.234928	0.611068	-2.800672
C	4.302329	0.386214	-1.350404
H	5.337540	0.651282	-1.528768
C	3.712549	1.335275	-3.596545
C	4.950339	-0.573125	0.885447
F	4.841117	2.071342	-3.477911
F	3.948019	0.410004	-4.557175
F	4.816772	-1.834475	1.356438
F	2.744401	2.150696	-4.068738
F	4.821063	0.251018	1.955812
F	6.217428	-0.440658	0.437469

NTOB-benzaldehyde complex 121

B3LYP/6-31G(d) = -2780.338058
B3LYP-D3(BJ)/def2-TZVPP/IEFPCM(propanonitrile) = -2781.598673
B3LYP-D3(BJ)/def2-TZVPP/IEFPCM(propanonitrile)//B3LYP-D3(BJ)/6-31G(d) Free Energy (Quasiharmonic) = -2781.113099

Frequencies (Top 3 out of 213)

1. 13.4980 cm^{-1}

2. 14.6929 cm^{-1}
3. 15.8197 cm^{-1}

B3LYP/6-31G(d) Molecular Geometry in Cartesian Coordinates

B	-0.075334	-0.578499	-0.862811
O	0.312841	-1.848665	-1.439377
N	0.510249	0.461676	-1.783575
C	1.377668	-0.210906	-2.773498
S	-0.237948	1.891397	-2.290104
C	1.143482	-1.694968	-2.498930
O	1.623480	-2.616852	-3.109794
H	2.968051	1.233716	-2.729867
H	1.030388	0.006364	-3.787943
C	2.892652	0.142207	-2.733604
C	3.719482	-0.465844	-1.630821
C	4.067034	0.081821	-0.339354
C	4.889879	-0.877088	0.320979
N	5.030281	-1.948158	-0.535014
C	4.333170	-1.694671	-1.699174
H	4.307872	-2.422860	-2.497443
C	5.084115	0.524390	2.243839
C	3.766407	1.280249	0.332883
H	6.037967	-1.412075	2.086609
H	5.572169	-2.778485	-0.353203
C	4.271546	1.489942	1.610860
H	5.472663	0.720367	3.239596
H	3.127271	2.024374	-0.130766
C	5.404004	-0.670317	1.606792
H	4.039876	2.414679	2.132423
C	0.029035	3.081287	-0.970689
O	-1.687610	1.694044	-2.394050
O	0.534502	2.345635	-3.455347
C	0.420450	4.985504	1.048267
C	1.219424	3.810632	-0.931259
C	-0.980434	3.315926	-0.037467
C	-0.772371	4.254563	0.972463
C	1.407370	4.752496	0.078063
H	1.974503	3.657541	-1.695403
H	-1.920011	2.782077	-0.107124
H	-1.557148	4.418249	1.706517
H	2.332309	5.323670	0.107596
C	0.622780	6.028643	2.121352
H	1.680492	6.135803	2.383828
H	0.067782	5.779661	3.031615
H	0.270149	7.012282	1.783931
H	3.299976	-0.189877	-3.695301
C	-1.566972	-0.534258	-0.296239
C	-2.572832	-1.264583	-0.949633
C	-1.938500	0.206681	0.831468
C	-3.894611	-1.238634	-0.502873
H	-2.322291	-1.856898	-1.823746
C	-3.265939	0.256884	1.266796
H	-1.189512	0.770868	1.377484
C	-4.253827	-0.466006	0.603149
H	-5.283690	-0.428583	0.937651
C	-3.612855	1.160539	2.416213
C	-4.933892	-2.093573	-1.178191
F	-2.734970	1.033349	3.438599
F	-4.846044	0.927253	2.908106
F	-3.576089	2.469617	2.044109
F	-4.998211	-3.327089	-0.617437
F	-4.669453	-2.273677	-2.488475
F	-6.170202	-1.556612	-1.079323
O	0.889253	-0.387215	0.551641
C	1.480295	-1.050462	1.422197
C	1.355227	-2.453271	1.777285
C	0.414920	-3.342755	1.217000
C	2.225257	-2.905479	2.792505
C	0.358521	-4.655037	1.670493
H	-0.248185	-3.009819	0.430845
C	2.166336	-4.221462	3.234607
H	2.949551	-2.215025	3.217060
C	1.229924	-5.094847	2.673461
H	-0.365158	-5.340909	1.241013
H	2.839585	-4.567002	4.013248
H	1.176943	-6.123536	3.019609
H	2.193092	-0.475849	2.029182

NTOB-benzaldehyde complex 122

B3LYP/6-31G(d) = -2780.339497
B3LYP-D3(BJ)/def2-TZVPP/IEFPCM(propanonitrile) = -2781.596563
B3LYP-D3(BJ)/def2-TZVPP/IEFPCM(propanonitrile)//B3LYP-D3(BJ)/6-31G(d) Free Energy (Quasiharmonic) = -2781.112147

Frequencies (Top 3 out of 213)

1. 5.0502 cm^{-1}
2. 9.5225 cm^{-1}
3. 13.4217 cm^{-1}

B3LYP/6-31G(d) Molecular Geometry in Cartesian Coordinates

B	-0.820600	-0.057514	1.007606
O	-0.343009	-0.666603	2.234391
N	0.501987	0.417046	0.332947
C	1.609786	-0.379142	0.939897
S	0.576891	0.741340	-1.302846
C	0.994679	-0.798939	2.279908
O	1.592075	-1.192358	3.250465
H	2.868941	1.069001	1.947126
H	1.778404	-1.286777	0.343290
C	2.964050	0.344172	1.130835
C	4.104557	-0.599536	1.390247
C	4.811891	-1.380080	0.401759
C	5.822528	-2.110297	1.087367
N	5.724045	-1.780091	2.424243
C	4.687248	-0.884784	2.598351
H	4.421436	-0.532862	3.584700
C	6.557325	-3.107003	-0.950174
C	4.688129	-1.541154	-0.990201
H	7.468822	-3.521849	0.967601
H	6.278544	-2.176096	3.166728
C	5.558692	-2.400085	-1.650340
H	7.222294	-3.772662	-1.493870
H	3.909535	-1.019532	-1.540921
C	6.702435	-2.972602	0.426265
H	5.465549	-2.536532	-2.724411
C	1.769317	2.078758	-1.482919
O	-0.738960	1.320653	-1.648574
O	1.095575	-0.399457	-2.069149
C	3.524315	4.218032	-1.937466
C	1.708242	3.210827	-0.666056
C	2.690026	2.006640	-2.527508
C	3.561101	3.074812	-2.744445
C	2.586607	4.265395	-0.893265
H	1.005475	3.251715	0.159932
H	2.725161	1.120484	-3.151689
H	4.284742	3.013214	-3.553187
H	2.550505	5.138716	-0.246257
C	4.454975	5.379889	-2.189697
H	3.952433	6.168515	-2.765217
H	5.336791	5.070338	-2.758860
H	4.796259	5.830970	-1.251721
H	3.184795	0.919103	0.227738
C	-1.905904	-0.995050	0.262312
C	-2.874877	-0.546454	-0.647391
C	-1.907903	-2.364894	0.570030
C	-3.792754	-1.421294	-1.230949
H	-2.917480	0.498361	-0.930035

C	-2.818762	-3.247368	-0.017001
H	-1.192772	-2.752684	1.287260
C	-3.769523	-2.780979	-0.921783
H	-4.481775	-3.461597	-1.373480
C	-2.823142	-4.696561	0.390177
C	-4.780117	-0.904277	-2.241716
F	-3.579364	-4.901992	1.495592
F	-1.581947	-5.143691	0.677847
F	-3.327596	-5.491816	-0.580561
F	-4.282881	-0.933657	-3.498395
F	-5.131236	0.379937	-1.990637
F	-5.918309	-1.635851	-2.254117
O	-1.643839	1.213686	1.562859
C	-1.894042	2.283675	0.963984
C	-2.707410	3.316158	1.561969
C	-3.243146	3.171578	2.858566
C	-2.961327	4.482689	0.814910
C	-4.021848	4.187767	3.393448
H	-3.037379	2.264629	3.417761
C	-3.744019	5.495777	1.358457
H	-2.548123	4.580465	-0.185734
C	-4.271317	5.346407	2.644617
H	-4.439373	4.085695	4.390382
H	-3.946400	6.395755	0.786299
H	-4.883477	6.137439	3.068927
H	-1.494002	2.427447	-0.045802

NTOB-benzaldehyde complex 123

B3LYP/6-31G(d) = -2780.336671
B3LYP-D3(BJ)/def2-TZVPP/IEFPCM(propanonitrile) = -2781.596534
B3LYP-D3(BJ)/def2-TZVPP/IEFPCM(propanonitrile)//B3LYP-D3(BJ)/6-31G(d) Free Energy (Quasiharmonic) = -2781.112135

Frequencies (Top 3 out of 213)

1. 8.3842 cm^{-1}
2. 10.3279 cm^{-1}
3. 14.1610 cm^{-1}

B3LYP/6-31G(d) Molecular Geometry in Cartesian Coordinates

B	-0.912978	-1.192114	0.026151
O	-1.005592	-2.057055	1.180158
N	0.559299	-1.182373	-0.308171
C	1.264581	-2.196386	0.504144
S	1.168133	-0.719901	-1.797971
C	0.156906	-2.703164	1.436071
O	0.280026	-3.530388	2.301681
H	2.488702	-2.363906	2.240168
H	1.580770	-3.040903	-0.119992
C	2.450394	-1.700028	1.365673
C	3.806336	-1.657305	0.714049
C	4.905420	-0.827448	1.148656
C	6.023378	-1.135878	0.324873
N	5.614417	-2.112573	-0.558146
C	4.287433	-2.422050	-0.318726
H	3.780874	-3.141256	-0.944982
C	7.373897	0.462952	1.468616
C	5.053841	0.148391	2.149310
H	8.104502	-0.751903	-0.165552
H	6.179573	-2.520358	-1.286043
C	6.280503	0.784866	2.299234
H	8.323000	0.972402	1.612428
H	4.221328	0.403230	2.800127
C	7.260600	-0.500572	0.472222
H	6.404651	1.537241	3.073599
C	2.321076	0.607053	-1.444661
O	0.040099	-0.146564	-2.544652
O	1.927658	-1.835528	-2.381685
C	4.095801	2.723191	-0.994592
C	3.557479	0.616450	-2.089132
C	1.944649	1.652996	-0.600423
C	2.835876	2.699990	-0.379682
C	4.433851	1.674564	-1.860153
H	3.829215	-0.205283	-2.741818
H	0.973762	1.644831	-0.115516
H	2.550504	3.510501	0.286415
H	5.404230	1.678085	-2.350172
C	5.083028	3.825176	-0.696878
H	5.695919	4.063899	-1.572640
H	5.765420	3.518513	0.106686
H	4.579309	4.741340	-0.372102
O	-1.635249	-2.115410	-1.247214
C	-2.748330	-2.542471	-1.596621
H	-2.801397	-2.894253	-2.637844
H	2.191043	-0.704205	1.746533
C	-3.985230	-2.643595	-0.846256
C	-4.078432	-2.465030	0.550467
C	-5.141838	-2.957024	-1.591775
C	-5.316038	-2.578855	1.171988
H	-3.185000	-2.277974	1.132931
C	-6.376544	-3.054743	-0.962859
H	-5.062423	-3.107794	-2.665569
C	-6.461521	-2.863433	0.419779
H	-5.391119	-2.448941	2.247161
H	-7.267086	-3.282990	-1.540169
H	-7.424549	-2.945676	0.916162
C	-1.792864	0.140491	0.149070
C	-2.275490	0.891395	-0.935611
C	-2.103157	0.599537	1.439487
C	-3.036700	2.044973	-0.735064
H	-2.039065	0.581635	-1.946234
C	-2.860628	1.756898	1.642056
H	-1.751571	0.043707	2.303212
C	-3.335568	2.486249	0.554311
H	-3.928784	3.380091	0.707139
C	-3.223501	2.168374	3.043279
C	-3.494066	2.850477	-1.922480
F	-3.541391	3.479037	3.122534
F	-4.293785	1.475242	3.504355
F	-2.553955	3.736452	-2.321061
F	-2.212425	1.941450	3.910831
F	-4.612049	3.561888	-1.646817
F	-3.769666	2.063366	-2.986451

NTOB-benzaldehyde complex 124

B3LYP/6-31G(d) = -2780.340973
B3LYP-D3(BJ)/def2-TZVPP/IEFPCM(propanonitrile) = -2781.596417
B3LYP-D3(BJ)/def2-TZVPP/IEFPCM(propanonitrile)//B3LYP-D3(BJ)/6-31G(d) Free Energy (Quasiharmonic) = -2781.11128

Frequencies (Top 3 out of 213)

1. 4.9383 cm^{-1}
2. 8.4089 cm^{-1}
3. 11.4718 cm^{-1}

B3LYP/6-31G(d) Molecular Geometry in Cartesian Coordinates

B	0.986101	-0.428951	-0.676470
O	0.678017	-1.482783	-1.625526
N	-0.327902	-0.278219	0.097993
C	-1.271798	-1.345918	-0.294848
S	-0.396820	0.441083	1.598810
C	-0.474938	-2.115155	-1.356851
O	-0.829518	-3.123572	-1.918398
H	-2.386794	-0.492641	-1.946108
H	-1.491724	-2.015086	0.546137

C	-2.597132	-0.821596	-0.917725
C	-3.755382	-1.783348	-0.881792
C	-5.145169	-1.401352	-0.782093
C	-5.915277	-2.598069	-0.781224
N	-5.026934	-3.647343	-0.877718
C	-3.736809	-3.155846	-0.946910
H	-2.892846	-3.818197	-1.070963
C	-7.945726	-1.356090	-0.618150
C	-5.817237	-0.168676	-0.705668
H	-7.881475	-3.516510	-0.694263
H	-5.274384	-4.623529	-0.918219
C	-7.205332	-0.155888	-0.625904
H	-9.029864	-1.315916	-0.556360
H	-5.260073	0.764476	-0.714990
C	-7.311391	-2.590818	-0.695208
H	-7.733059	0.793570	-0.580983
C	-2.068781	1.048502	1.813169
O	0.485075	1.622864	1.477266
O	-0.162664	-0.531708	2.674052
C	-4.664013	2.016847	2.208003
C	-2.975333	0.300183	2.567507
C	-2.429770	2.284429	1.274924
C	-3.725147	2.758283	1.476252
C	-4.265508	0.787728	2.754252
H	-2.664116	-0.640981	3.007426
H	-1.702005	2.868137	0.721491
H	-4.010657	3.722579	1.063269
H	-4.976283	0.204182	3.333538
C	-6.077340	2.511399	2.395888
H	-6.193922	3.542287	2.048033
H	-6.377225	2.472123	3.449367
H	-6.780277	1.882799	1.834852
H	-2.883375	0.080928	-0.373476
C	2.468103	-0.649633	-0.062131
C	3.322886	-1.485018	-0.809577
C	3.004196	-0.087553	1.109362
C	4.636230	-1.740994	-0.414735
H	2.950602	-1.945404	-1.717818
C	4.321774	-0.343664	1.506596
H	2.401668	0.567925	1.725742
C	5.147611	-1.170319	0.750629
H	6.170626	-1.357096	1.054622
C	4.828970	0.261972	2.788675
C	5.490892	-2.679747	-1.224279
F	6.180993	0.272031	2.844375
F	4.393480	-0.414722	3.873424
F	4.412507	1.542169	2.932892
F	6.809436	-2.412499	-1.077172
F	5.209171	-2.606010	-2.544203
F	5.308934	-3.968534	-0.855725
O	1.033100	0.929291	-1.622771
C	1.602093	1.986364	-1.278589
C	1.490339	3.207329	-2.044716
C	2.161260	4.353944	-1.579913
C	0.734734	3.260154	-3.233595
C	2.077914	5.543367	-2.296609
H	2.737823	4.303225	-0.659953
C	0.657829	4.450130	-3.943576
H	0.225617	2.365343	-3.576494
C	1.328243	5.588691	-3.475533
H	2.592603	6.430875	-1.941979
H	0.079854	4.499583	-4.861347
H	1.264394	6.517224	-4.036157
H	2.208661	1.993967	-0.368983

TS 1

B3LYP/6-31G(d) = -3573.93751
B3LYP-D3(BJ)/def2-TZVPP/IEFPCM(propanonitrile) = -3575.516454
B3LYP-D3(BJ)/def2-TZVPP/IEFPCM(propanonitrile)//B3LYP-D3(BJ)/6-31G(d) Free Energy (Quasiharmonic) = -3574.799308

Frequencies (Top 3 out of 300)

1. -267.6425 cm^{-1}
2. 8.4590 cm^{-1}
3. 15.1413 cm^{-1}

B3LYP/6-31G(d) Molecular Geometry in Cartesian Coordinates

B	0.667763	-0.354105	-0.259153
O	0.222358	-0.627094	-1.662744
N	0.831376	-1.810794	0.267101
C	0.767397	-2.776023	-0.837406
S	1.592149	-2.204633	1.692259
C	0.277156	-1.917532	-2.007748
O	-0.018720	-2.339757	-3.103673
H	0.435660	-4.720470	0.016255
H	1.775211	-3.127630	-1.115249
C	-0.102220	-4.048211	-0.655132
C	-1.502838	-3.873233	-0.134878
C	-2.720682	-3.623500	-0.878389
C	-3.796806	-3.648894	0.055824
N	-3.248028	-3.876466	1.303169
C	-1.878311	-4.026542	1.177840
H	-1.252772	-4.215660	2.038650
C	-5.387230	-3.250427	-1.678885
C	-3.014747	-3.400258	-2.237307
H	-5.938213	-3.510625	0.398995
H	-3.769647	-4.049090	2.148727
C	-4.338572	-3.215429	-2.621397
H	-6.413258	-3.118780	-2.012273
H	-2.214303	-3.360251	-2.969359
C	-5.130311	-3.466824	-0.327662
H	-4.572122	-3.049927	-3.669769
C	3.350798	-2.343708	1.344193
O	1.149970	-3.552734	2.088445
O	1.396068	-1.060081	2.587921
C	6.067623	-2.501445	0.668068
C	4.189088	-1.252093	1.585217
C	3.863093	-3.526728	0.806326
C	5.215108	-3.594230	0.468144
C	5.536402	-1.340166	1.249258
H	3.782492	-0.351060	2.029748
H	3.218834	-4.390201	0.678339
H	5.612609	-4.512629	0.043052
H	6.185465	-0.486483	1.425934
C	7.517822	-2.550091	0.252353
H	7.812972	-3.553994	-0.067854
H	8.180587	-2.246065	1.071108
H	7.697552	-1.861608	-0.582600
H	-0.127435	-4.523573	-1.643100
C	1.953206	0.627822	-0.272988
C	3.007089	0.402748	-1.173379
C	2.056633	1.748577	0.561073
C	4.116227	1.249069	-1.231580
H	2.967676	-0.445573	-1.849798
C	3.148793	2.619516	0.486457
H	1.270271	1.947210	1.279787
C	4.189223	2.373586	-0.407297
H	5.038691	3.044924	-0.463991
C	3.151555	3.878110	1.308201
C	5.276626	0.933309	-2.133733
F	2.574620	3.700936	2.516327
F	2.460331	4.874018	0.691974
F	4.401086	4.349910	1.520121
F	6.284921	0.320932	-1.456048
F	5.811672	2.051594	-2.677424
F	4.934208	0.113287	-3.148526
O	-0.427399	0.399306	0.470528

C	-1.613899	-0.097494	0.696478
C	-2.356169	0.446579	1.855951
C	-3.384067	-0.312439	2.433279
C	-2.009452	1.682819	2.422855
C	-4.053890	0.155474	3.563037
H	-3.640460	-1.277643	2.004548
C	-2.685465	2.149808	3.547234
H	-1.200836	2.258655	1.987040
C	-3.708168	1.388826	4.120308
H	-4.837640	-0.446135	4.015847
H	-2.405529	3.103325	3.986257
H	-4.226037	1.750843	5.004389
H	-1.745557	-1.164794	0.504378
C	-2.756916	0.311748	-0.981919
H	-3.570562	-0.398982	-0.872469
H	-1.959796	-0.001504	-1.643539
C	-3.062152	1.669738	-0.883431
C	-2.096244	2.744657	-1.126420
C	-2.368904	4.045472	-0.650464
C	-0.880154	2.506006	-1.798512
C	-1.440277	5.065335	-0.816579
H	-3.301964	4.232335	-0.132230
C	0.039947	3.537039	-1.968486
H	-0.646583	1.523735	-2.190496
C	-0.229567	4.812684	-1.470581
H	-1.653431	6.057788	-0.429770
H	0.976118	3.336550	-2.479914
H	0.502144	5.606822	-1.587093
O	-4.233028	2.075294	-0.399107
Si	-5.868513	1.616820	-0.720770
C	-6.801440	3.209900	-0.384601
H	-7.879720	3.069001	-0.527156
H	-6.644117	3.548533	0.645864
H	-6.477275	4.012851	-1.055947
C	-6.416437	0.255193	0.447096
H	-7.502752	0.119426	0.365019
H	-5.945265	-0.705555	0.213213
H	-6.185537	0.506133	1.487414
C	-5.979979	1.089101	-2.520943
H	-5.576699	1.855523	-3.192591
H	-5.444710	0.152700	-2.711403
H	-7.030350	0.929248	-2.795089

TS 2

B3LYP/6-31G(d) = -3573.937758
B3LYP-D3(BJ)/def2-TZVPP/IEFPCM(propanonitrile) = -3575.515631
B3LYP-D3(BJ)/def2-TZVPP/IEFPCM(propanonitrile)//B3LYP-D3(BJ)/6-31G(d) Free Energy (Quasiharmonic) = -3574.798794

Frequencies (Top 3 out of 300)

1. -257.9388 cm^{-1}
2. 5.8845 cm^{-1}
3. 9.7574 cm^{-1}

B3LYP/6-31G(d) Molecular Geometry in Cartesian Coordinates

B	-0.512700	-0.298592	0.252121
O	-0.036643	-0.588790	1.638885
N	-0.702951	-1.755183	-0.286737
C	-0.633720	-2.725955	0.813917
S	-1.490300	-2.126750	-1.704807
C	-0.088547	-1.878981	1.968156
O	0.251655	-2.313900	3.049686
H	-0.387945	-4.686105	-0.033007
H	-1.640823	-3.048998	1.125540
C	0.197824	-4.021160	0.603964
C	1.573768	-3.875230	0.013638
C	2.830891	-3.654106	0.698090
C	3.856162	-3.659524	-0.290896
N	3.241759	-3.854507	-1.513942
C	1.879103	-4.005096	-1.319580
H	1.209197	-4.176377	-2.150164
C	5.535044	-3.298807	1.365702
C	3.196412	-3.469386	2.045176
H	5.975237	-3.497271	-0.744548
H	3.719653	-4.036330	-2.383228
C	4.538543	-3.294249	2.363265
H	6.576216	-3.165259	1.647191
H	2.435625	-3.449629	2.819288
C	5.207546	-3.480769	0.025630
H	4.827791	-3.154710	3.401471
C	-3.261620	-2.053022	-1.403203
O	-1.189315	-3.530853	-2.033068
O	-1.154246	-1.051546	-2.645883
C	-5.996825	-1.859703	-0.814644
C	-3.892464	-3.079802	-0.694798
C	-3.993482	-0.958279	-1.866382
C	-5.351764	-0.872304	-1.572176
C	-5.250722	-2.971683	-0.400661
H	-3.336162	-3.963189	-0.399043
H	-3.504120	-0.185640	-2.446212
H	-5.914320	-0.013329	-1.929696
H	-5.739308	-3.765700	0.158837
C	-7.448162	-1.708061	-0.429810
H	-8.035390	-1.261313	-1.239474
H	-7.900696	-2.670148	-0.169282
H	-7.539197	-1.049735	0.443629
H	0.262118	-4.487077	1.594520
C	-1.809263	0.664588	0.277045
C	-2.062196	1.595259	-0.740367
C	-2.759187	0.567628	1.305150
C	-3.217941	2.380491	-0.740668
C	-3.914775	1.353710	1.313573
H	-2.596980	-0.127729	2.123693
C	-4.153369	2.266602	0.286812
H	-5.046792	2.879858	0.290343
C	-4.943322	1.160790	2.393022
C	-3.505793	3.266709	-1.918426
F	-4.381036	0.851831	3.582111
F	-5.798244	0.146664	2.096144
F	-5.703009	2.264153	2.578015
F	-2.386157	3.834980	-2.418642
F	-4.366025	4.265104	-1.616733
F	-4.069435	2.560020	-2.934635
O	0.563055	0.456788	-0.492311
C	1.665455	-0.104375	-0.912154
C	2.326394	0.503030	-2.087575
C	1.991876	1.794522	-2.520663
C	3.267292	-0.245336	-2.810705
C	2.589666	2.324851	-3.661109
H	1.251139	2.362325	-1.967942
C	3.862599	0.288304	-3.951914
H	3.512171	-1.253322	-2.484118
C	3.526092	1.575160	-4.378146
H	2.319027	3.321876	-3.997152
H	4.580889	-0.301550	-4.515005
H	3.985470	1.989594	-5.271459
H	1.712368	-1.193912	-0.852228
C	3.054713	-0.006882	0.614239
H	2.453496	-0.547430	1.333774
H	3.847730	-0.579398	0.149050
C	3.241518	1.355421	0.840467
C	4.244392	2.174921	0.139977
C	4.115487	3.577686	0.133323
C	5.346912	1.593334	-0.515707
C	5.051813	4.371567	-0.520053
H	3.266458	4.029080	0.632932
C	6.285004	2.392336	-1.161700
H	5.487062	0.518476	-0.501494

C	6.139144	3.781522	-1.169670
H	4.934310	5.451438	-0.524594
H	7.134485	1.930700	-1.656547
H	6.873383	4.402277	-1.675794
O	2.403239	2.024784	1.613841
Si	2.010544	2.030355	3.305140
C	0.208964	2.521196	3.377501
H	-0.419501	1.752054	2.920263
H	-0.109565	2.647836	4.419673
H	0.029765	3.468133	2.856027
C	3.121711	3.371586	4.025653
H	2.942905	4.342792	3.550202
H	2.931528	3.488973	5.099943
H	4.183837	3.128415	3.904018
H	-1.355741	1.690004	-1.557814
C	2.391235	0.373513	4.094029
H	1.675704	-0.406348	3.813800
H	2.346285	0.490010	5.185107
H	3.400487	0.021768	3.849111

TS 3

B3LYP/6-31G(d) = -3573.936588
B3LYP-D3(BJ)/def2-TZVPP/IEFPCM(propanonitrile) = -3575.516096
B3LYP-D3(BJ)/def2-TZVPP/IEFPCM(propanonitrile)//B3LYP-D3(BJ)/6-31G(d) Free Energy (Quasiharmonic) = -3574.79868

Frequencies (Top 3 out of 300)

1. -276.8178 cm^{-1}
2. 9.7091 cm^{-1}
3. 12.7212 cm^{-1}

B3LYP/6-31G(d) Molecular Geometry in Cartesian Coordinates

B	0.643000	-0.209690	-0.545613
O	0.289423	-0.264360	-1.998951
N	0.692843	-1.737788	-0.216599
C	0.731872	-2.524872	-1.458841
S	1.337528	-2.357328	1.190202
C	0.372725	-1.489291	-2.529161
O	0.175286	-1.736462	-3.698347
H	0.098303	-4.474036	-0.795827
H	1.756058	-2.866895	-1.678155
C	-0.190994	-3.765532	-1.573892
C	-1.677864	-3.527651	-1.524559
C	-2.575470	-3.728796	-0.406460
C	-3.897370	-3.477678	-0.873775
N	-3.797131	-3.109609	-2.202096
C	-2.468916	-3.168950	-2.590015
H	-2.177128	-2.942467	-3.605895
C	-4.812824	-4.015993	1.262735
C	-2.394618	-4.120018	0.934263
H	-6.025636	-3.437439	-0.434793
H	-4.574522	-2.998749	-2.834569
C	-3.511779	-4.260535	1.750263
H	-5.666265	-4.146334	1.923117
H	-1.395910	-4.296982	1.321446
C	-5.023291	-3.618128	-0.054192
H	-3.381152	-4.566026	2.784764
C	3.130303	-2.353617	1.043568
O	0.939365	-3.772543	1.279793
O	0.975453	-1.409343	2.250109
C	5.911480	-2.239252	0.699491
C	3.872076	-1.381544	1.717073
C	3.770119	-3.307906	0.247105
C	5.151914	-3.238379	0.075730
C	5.252566	-1.333879	1.542813
H	3.373416	-0.673156	2.366460
H	3.200435	-4.109015	-0.211907
H	5.648034	-3.973712	-0.553058
H	5.822054	-0.567983	2.063572
C	7.394198	-2.115422	0.447145
H	7.822229	-3.053136	0.078692
H	7.934994	-1.825004	1.354464
H	7.583739	-1.342839	-0.309216
H	0.063736	-4.216751	-2.539851
C	1.981962	0.667078	-0.320230
C	2.998047	0.694114	-1.287293
C	2.197711	1.402240	0.854068
C	4.184827	1.404286	-1.084962
H	2.864692	0.159729	-2.223299
C	3.382470	2.112894	1.063026
H	1.436701	1.399014	1.626854
C	4.386214	2.118872	0.095503
H	5.303693	2.673015	0.255450
C	3.621606	2.773996	2.389926
C	5.283327	1.335747	-2.108895
F	4.540023	3.763931	2.316650
F	4.085652	1.879598	3.304153
F	2.493872	3.306611	2.909623
F	6.078612	0.250020	-1.920115
F	6.092686	2.418854	-2.062922
F	4.801450	1.244257	-3.367909
O	-0.451468	0.500036	0.219519
C	-1.628256	-0.029664	0.433517
C	-2.364074	0.419454	1.635971
C	-3.362394	-0.406456	2.174445
C	-2.042050	1.626381	2.275512
C	-4.029120	-0.028629	3.339808
H	-3.590399	-1.359425	1.704120
C	-2.712689	2.000213	3.437180
H	-1.253830	2.249604	1.866634
C	-3.707563	1.175574	3.971663
H	-4.784148	-0.684690	3.764178
H	-2.451210	2.929730	3.935267
H	-4.219161	1.463805	4.886199
H	-1.742136	-1.085422	0.178519
C	-2.801770	0.471684	-1.169744
H	-3.603304	-0.246031	-1.038236
H	-2.051332	0.199050	-1.900099
C	-3.110492	1.822041	-0.992896
C	-2.201427	2.926097	-1.316698
C	-2.513263	4.234090	-0.887365
C	-1.013097	2.714483	-2.045944
C	-1.662874	5.293833	-1.175979
H	-3.418363	4.400984	-0.315839
C	-0.166786	3.782833	-2.330357
H	-0.738094	1.725754	-2.391160
C	-0.486595	5.071305	-1.899489
H	-1.912069	6.294536	-0.834592
H	0.747526	3.602759	-2.887389
H	0.178155	5.900871	-2.124415
O	-4.217478	2.184364	-0.354304
Si	-5.901229	1.836550	-0.419247
C	-6.551896	2.761011	-1.922265
H	-6.344940	3.834700	-1.853370
H	-6.098687	2.391911	-2.849847
H	-7.637955	2.635228	-2.013098
C	-6.535155	2.542486	1.194776
H	-7.617592	2.392659	1.288028
H	-6.049873	2.057392	2.048599
H	-6.339355	3.618361	1.263053
C	-6.260047	-0.002269	-0.569698
H	-5.924356	-0.415010	-1.527473
H	-5.798818	-0.586024	0.233344
H	-7.345538	-0.156689	-0.510615

TS 4

B3LYP/6-31G(d) = -3573.938129
B3LYP-D3(BJ)/def2-TZVPP/IEFPCM(propanonitrile) = -3575.515735
B3LYP-D3(BJ)/def2-TZVPP/IEFPCM(propanonitrile)//B3LYP-D3(BJ)/6-31G(d) Free Energy (Quasiharmonic) = -3574.798122

Frequencies (Top 3 out of 300)

1. -266.6025 cm^{-1}
2. 10.7022 cm^{-1}
3. 12.5435 cm^{-1}

B3LYP/6-31G(d) Molecular Geometry in Cartesian Coordinates

B	-0.635996	0.321000	-0.363098
O	-0.144053	0.593865	-1.747813
N	-0.745237	1.779019	0.186615
C	-0.642782	2.750006	-0.911364
S	-1.507092	2.182676	1.610509
C	-0.144877	1.890065	-2.077047
O	0.197341	2.314006	-3.158538
H	-0.298107	4.690295	-0.053867
H	-1.637376	3.118817	-1.211696
C	0.247314	4.003905	-0.703761
C	1.624440	3.795346	-0.135275
C	2.861468	3.528476	-0.839831
C	3.905104	3.517716	0.130536
N	3.318645	3.735380	1.362674
C	1.957460	3.923359	1.191264
H	1.307818	4.121405	2.032015
C	5.545828	3.120182	-1.557030
C	3.196424	3.319852	-2.191264
H	6.031293	3.338949	0.541980
H	3.816698	3.905756	2.222902
C	4.528989	3.117339	-2.534220
H	6.580189	2.977139	-1.858419
H	2.420157	3.305527	-2.949940
C	5.247389	3.319227	-0.211522
H	4.794673	2.963355	-3.576637
C	-3.279471	2.208009	1.307720
O	-1.127175	3.565050	1.948652
O	-1.230062	1.083677	2.543103
C	-6.015481	2.150909	0.694220
C	-3.853472	3.268583	0.600681
C	-4.068675	1.149471	1.760644
C	-5.426727	1.130701	1.454099
C	-5.212840	3.228205	0.294386
H	-3.252158	4.124958	0.313724
H	-3.623212	0.351756	2.341587
H	-6.032668	0.297715	1.802034
H	-5.657103	4.047668	-0.265378
C	-7.467084	2.068432	0.289814
H	-7.871173	3.051355	0.027429
H	-7.576572	1.418317	-0.587836
H	-8.085304	1.645990	1.089390
H	0.318616	4.473610	-1.692069
C	-1.975927	-0.580431	-0.395646
C	-2.290892	-1.479390	0.633175
C	-2.898718	-0.460319	-1.445620
C	-3.483147	-2.208034	0.625636
C	-4.091601	-1.188690	-1.460371
H	-2.685092	0.206562	-2.275742
C	-4.393457	-2.068094	-0.420904
H	-5.316340	-2.636142	-0.429334
C	-5.092776	-0.967196	-2.559848
C	-3.837060	-3.055025	1.814069
F	-4.499332	-0.683290	-3.739808
F	-5.920153	0.074690	-2.282757
F	-5.884558	-2.046824	-2.753908
F	-4.735496	-4.019625	1.512821
F	-4.391588	-2.301603	2.802202
F	-2.756940	-3.662314	2.352505
O	0.399080	-0.498205	0.382669
C	1.560841	-0.025251	0.745895
C	2.207361	-0.663100	1.913446
C	1.834429	-1.948694	2.335247
C	3.163311	0.054294	2.646251
C	2.415417	-2.505617	3.471566
H	1.077811	-2.490515	1.778181
C	3.737449	-0.504054	3.787652
H	3.436251	1.058092	2.329959
C	3.367316	-1.786285	4.200065
H	2.116451	-3.497645	3.798414
H	4.463356	0.064810	4.362560
H	3.810212	-2.219323	5.092902
H	1.702851	1.053638	0.650209
C	2.845939	-0.304134	-0.858976
H	2.115861	0.063641	-1.567725
H	3.638356	0.394796	-0.609693
C	3.157546	-1.663662	-0.855094
C	2.252287	-2.720108	-1.318506
C	2.550788	-4.068554	-1.026709
C	1.082901	-2.423872	-2.048573
C	1.701808	-5.085521	-1.444908
H	3.444664	-4.300057	-0.459861
C	0.239387	-3.450193	-2.465689
H	0.827313	-1.402013	-2.299275
C	0.543105	-4.779161	-2.166295
H	1.939219	-6.118753	-1.207854
H	-0.659356	-3.206136	-3.023706
H	-0.119624	-5.575592	-2.493695
O	4.276793	-2.104816	-0.286447
Si	5.935872	-1.639213	-0.415558
C	6.830226	-3.250196	-0.059777
H	6.580407	-4.019154	-0.799300
H	7.917326	-3.106560	-0.080889
H	6.565457	-3.636867	0.931017
C	6.362447	-0.336179	0.864622
H	6.024842	-0.632436	1.862968
H	7.451471	-0.202446	0.901244
H	5.918697	0.636097	0.625134
H	-1.604106	-1.593045	1.464905
C	6.226447	-1.027630	-2.168164
H	5.897879	-1.762470	-2.911952
H	7.297937	-0.852651	-2.327271
H	5.706396	-0.084108	-2.366364

TS 5

B3LYP/6-31G(d) = -3573.937255
B3LYP-D3(BJ)/def2-TZVPP/IEFPCM(propanonitrile) = -3575.514722
B3LYP-D3(BJ)/def2-TZVPP/IEFPCM(propanonitrile)//B3LYP-D3(BJ)/6-31G(d) Free Energy (Quasiharmonic) = -3574.797424

Frequencies (Top 3 out of 300)

1. -274.7444 cm^{-1}
2. 6.7367 cm^{-1}
3. 8.0565 cm^{-1}

B3LYP/6-31G(d) Molecular Geometry in Cartesian Coordinates

B	0.570270	0.157251	0.446530
O	0.202540	0.246441	1.894760
N	0.655000	1.681051	0.091510
C	0.721180	2.483731	1.324100
S	1.312600	2.261591	-1.326210
C	0.323940	1.472211	2.404170
O	0.122550	1.742951	3.570390
H	0.144800	4.437121	0.621200
H	1.757410	2.791101	1.538420
C	-0.155250	3.761041	1.423830

C	-1.649660	3.574251	1.414360
C	-2.563170	3.698161	0.299280
C	-3.881080	3.508561	0.808470
N	-3.763430	3.271981	2.162530
C	-2.428680	3.325871	2.519890
H	-2.122440	3.175551	3.545390
C	-4.825820	3.838701	-1.357630
C	-2.402130	3.969141	-1.073210
H	-6.015890	3.433641	0.405250
H	-4.530940	3.160181	2.806390
C	-3.531480	4.037931	-1.883100
H	-5.687970	3.906941	-2.015860
H	-1.409360	4.116621	-1.487930
C	-5.018370	3.570591	-0.005740
H	-3.415830	4.256071	-2.941430
C	3.104550	2.191621	-1.200840
O	0.963600	3.688891	-1.430610
O	0.901800	1.314571	-2.370280
C	5.880960	1.966230	-0.881180
C	3.799150	1.184631	-1.873390
C	3.789440	3.125671	-0.418270
C	5.168980	3.000961	-0.259180
C	5.177900	1.081741	-1.711310
H	3.264650	0.490291	-2.509380
H	3.256790	3.951851	0.040890
H	5.700620	3.719620	0.359730
H	5.709630	0.286690	-2.228050
C	7.358090	1.777870	-0.635490
H	7.885340	1.485760	-1.550290
H	7.824100	2.689150	-0.247580
H	7.516730	0.980770	0.102320
H	0.135370	4.228251	2.371740
C	1.906140	-0.736619	0.259520
C	2.935790	-0.702639	1.215520
C	2.111010	-1.536149	-0.871920
C	4.121750	-1.416009	1.038970
C	3.299110	-2.251429	-1.055900
H	1.341340	-1.580849	-1.635040
C	4.312450	-2.196879	-0.102900
H	5.236600	-2.745579	-0.248460
C	3.532100	-2.974849	-2.351080
C	5.221160	-1.368929	2.063320
F	4.455290	-3.955949	-2.236810
F	3.986550	-2.122529	-3.309770
F	2.403240	-3.535549	-2.836780
F	4.997720	-0.450859	3.025460
F	6.418330	-1.069080	1.492260
F	5.387560	-2.563319	2.679670
O	-0.522670	-0.558579	-0.302380
C	-1.599940	0.030881	-0.756450
C	-2.242440	-0.573919	-1.943250
C	-3.084490	0.213131	-2.742270
C	-1.979820	-1.902359	-2.310680
C	-3.652800	-0.322099	-3.897670
H	-3.271990	1.249361	-2.471190
C	-2.551710	-2.432319	-3.464010
H	-1.313320	-2.499739	-1.697310
C	-3.389520	-1.644439	-4.259320
H	-4.291780	0.297381	-4.520980
H	-2.337190	-3.458289	-3.750200
H	-3.827760	-2.058889	-5.163290
H	-1.610230	1.120951	-0.717090
C	-2.970860	-0.002269	0.759560
H	-3.655180	0.748991	0.385590
H	-2.271310	0.346121	1.509330
C	-3.416640	-1.320389	0.861910
C	-4.582720	-1.849239	0.133220
C	-5.495680	-1.000269	-0.523830
C	-4.813240	-3.238939	0.105140
C	-6.599540	-1.528809	-1.185790
H	-5.356920	0.074781	-0.511980
C	-5.915540	-3.762199	-0.561280
H	-4.112090	-3.897549	0.603370
C	-6.812810	-2.908789	-1.208610
H	-7.295930	-0.861209	-1.684700
H	-6.075600	-4.836449	-0.578220
H	-7.676240	-3.317279	-1.726510
O	-2.724860	-2.222369	1.534810
Si	-2.109420	-2.455399	3.141270
C	-2.312680	-0.897709	4.166660
H	-3.304700	-0.449069	4.034420
H	-2.220840	-1.167769	5.226980
H	-1.551330	-0.140559	3.954140
C	-0.342670	-3.030299	2.945440
H	0.059930	-3.345479	3.916370
H	-0.273660	-3.884789	2.262930
H	0.289230	-2.225289	2.561330
H	2.817490	-0.107339	2.115190
C	-3.205140	-3.830829	3.817260
H	-4.257720	-3.527679	3.862540
H	-2.895110	-4.102639	4.834070
H	-3.141550	-4.735079	3.200930

TS 6

B3LYP/6-31G(d) = -3573.937436
B3LYP-D3(BJ)/def2-TZVPP/IEFPCM(propanonitrile) = -3575.514662
B3LYP-D3(BJ)/def2-TZVPP/IEFPCM(propanonitrile)//B3LYP-D3(BJ)/6-31G(d) Free Energy (Quasiharmonic) = -3574.797175

Frequencies (Top 3 out of 300)

1. -274.6897 cm^{-1}
2. 6.6702 cm^{-1}
3. 14.0415 cm^{-1}

B3LYP/6-31G(d) Molecular Geometry in Cartesian Coordinates

B	0.571994	0.144267	0.444977
O	0.204704	0.237247	1.892872
N	0.667993	1.666909	0.088620
C	0.738264	2.470430	1.320264
S	1.332522	2.241586	-1.328211
C	0.334028	1.462754	2.401181
O	0.134116	1.735893	3.567080
H	0.173575	4.425692	0.612872
H	1.776402	2.771338	1.534713
C	-0.129855	3.753645	1.417669
C	-1.625546	3.576840	1.410309
C	-2.539752	3.705749	0.296335
C	-3.858232	3.526018	0.807620
N	-3.740329	3.290264	2.161799
C	-2.404751	3.335308	2.517240
H	-2.098156	3.184168	3.542503
C	-4.803775	3.860422	-1.357456
C	-2.378767	3.973843	-1.076731
H	-5.994097	3.465649	0.407595
H	-4.507687	3.184564	2.806861
C	-3.508817	4.049814	-1.885020
H	-5.666385	3.934052	-2.014514
H	-1.385525	4.113212	-1.493103
C	-4.996237	3.595150	-0.005002
H	-3.393159	4.265847	-2.943789
C	3.124041	2.180400	-1.192137
O	0.979150	3.667053	-1.442952
O	0.930709	1.287389	-2.369254
C	5.902729	1.984616	-0.870520
C	3.800328	3.128320	-0.418407
C	3.827908	1.172154	-1.852833
C	5.207805	1.084155	-1.689790
C	5.180695	3.018367	-0.258318

H	3.260203	3.955039	0.030917
H	3.300916	0.467333	-2.483453
H	5.747325	0.290161	-2.200145
H	5.705669	3.749131	0.352054
C	7.383409	1.820643	-0.629824
H	7.902822	1.476465	-1.530687
H	7.847011	2.757122	-0.303610
H	7.555847	1.072885	0.154939
H	0.164840	4.221560	2.363975
C	1.900280	-0.760869	0.258179
C	2.107981	-1.544596	-0.885856
C	2.916536	-0.761344	1.226005
C	3.284646	-2.276451	-1.065699
C	4.094041	-1.494230	1.053848
H	2.790944	-0.188629	2.140229
C	4.287222	-2.257126	-0.097234
H	5.197583	-2.828928	-0.233946
C	5.192169	-1.399702	2.076272
C	3.519130	-2.989396	-2.366436
F	6.002488	-0.333015	1.844319
F	5.986538	-2.494236	2.075404
F	4.710380	-1.249466	3.329366
F	4.000682	-2.134904	-3.309886
F	2.385946	-3.523279	-2.871971
F	4.422491	-3.988963	-2.253042
O	-0.526406	-0.563736	-0.303986
C	-1.599456	0.033363	-0.758113
C	-2.245495	-0.566550	-1.945582
C	-1.988427	-1.895357	-2.315575
C	-3.085028	0.225256	-2.742594
C	-2.563427	-2.421024	-3.469337
H	-1.323267	-2.496157	-1.704116
C	-3.656483	-0.305708	-3.898395
H	-3.268168	1.261776	-2.469631
C	-3.398839	-1.628496	-4.262514
H	-2.353077	-3.447290	-3.757577
H	-4.293502	0.317399	-4.520098
H	-3.839502	-2.039638	-5.166817
H	-1.602168	1.123525	-0.718522
C	-2.971035	0.009436	0.756482
H	-3.651535	0.763293	0.380741
H	-2.270642	0.355720	1.506467
C	-3.422995	-1.306493	0.860626
C	-4.590676	-1.831237	0.131604
C	-5.500996	-0.978951	-0.524792
C	-4.825484	-3.220203	0.102536
C	-6.606544	-1.503568	-1.187070
H	-5.358964	0.095655	-0.511910
C	-5.929366	-3.739547	-0.564310
H	-4.126306	-3.881325	0.600213
C	-6.824031	-2.882877	-1.210949
H	-7.300933	-0.833451	-1.685374
H	-6.092718	-4.813292	-0.582139
H	-7.688728	-3.288310	-1.729141
O	-2.735629	-2.210316	1.535521
Si	-2.127035	-2.445065	3.144282
C	-2.320055	-0.883634	4.165894
H	-2.232297	-1.152074	5.226974
H	-1.552404	-0.132945	3.953127
H	-3.308289	-0.427545	4.030796
C	-0.364633	-3.034825	2.953947
H	0.033719	-3.349540	3.926771
H	-0.301289	-3.892313	2.274632
H	0.274411	-2.236292	2.568140
H	1.346644	-1.564862	-1.658309
C	-3.235803	-3.809915	3.820543
H	-4.285998	-3.498084	3.862247
H	-3.177985	-4.716150	3.206564
H	-2.930761	-4.081759	4.838854

TS 7

B3LYP/6-31G(d) = -3573.936987
B3LYP-D3(BJ)/def2-TZVPP/IEFPCM(propanonitrile) = -3575.514548
B3LYP-D3(BJ)/def2-TZVPP/IEFPCM(propanonitrile)//B3LYP-D3(BJ)/6-31G(d) Free Energy (Quasiharmonic) = -3574.796733

Frequencies (Top 3 out of 300)

1. -276.7906 cm^{-1}
2. 11.0938 cm^{-1}
3. 14.1677 cm^{-1}

B3LYP/6-31G(d) Molecular Geometry in Cartesian Coordinates

B	0.672295	-0.162344	-0.553458
O	0.293119	-0.289787	-1.995464
N	0.674510	-1.671267	-0.136313
C	0.711499	-2.522887	-1.336468
S	1.296415	-2.228186	1.306774
C	0.371093	-1.540338	-2.461838
O	0.182661	-1.847314	-3.618184
H	0.036912	-4.419049	-0.567074
H	1.733362	-2.887311	-1.528695
C	-0.224448	-3.757775	-1.395146
C	-1.708954	-3.502109	-1.401809
C	-2.634233	-3.583357	-0.292435
C	-3.942301	-3.368930	-0.814982
N	-3.806801	-3.137273	-2.170012
C	-2.470521	-3.242260	-2.516283
H	-2.151538	-3.114587	-3.541086
C	-4.913610	-3.678920	1.342914
C	-2.489472	-3.840746	1.084605
H	-6.080385	-3.272416	-0.434849
H	-4.567093	-3.068483	-2.828727
C	-3.627098	-3.885149	1.884261
H	-5.783819	-3.735779	1.991481
H	-1.502632	-3.998461	1.509521
C	-5.088460	-3.416504	-0.012907
H	-3.525074	-4.093960	2.945834
C	3.089360	-2.269175	1.183723
O	0.864499	-3.627105	1.470329
O	0.940205	-1.216375	2.309402
C	5.875520	-2.229398	0.870362
C	3.718210	-3.278724	0.448892
C	3.842438	-1.275920	1.811831
C	5.225660	-1.265313	1.653315
C	5.102960	-3.246467	0.292683
H	3.137322	-4.091727	0.026229
H	3.350454	-0.522298	2.413556
H	5.803354	-0.481527	2.136895
H	5.591364	-4.025083	-0.288178
C	7.362963	-2.149724	0.628709
H	7.573085	-1.450125	-0.190609
H	7.780896	-3.122294	0.349691
H	7.896861	-1.788160	1.514116
O	-0.377262	0.639227	0.180821
C	-1.499592	0.137179	0.627479
H	-1.594744	-0.949361	0.591887
H	0.047737	-4.272180	-2.323889
C	-2.107241	0.800327	1.802335
C	-2.949483	0.059915	2.643656
C	-1.799965	2.131656	2.124402
C	-3.477418	0.644248	3.794895
H	-3.166559	-0.979743	2.410561
C	-2.335977	2.712665	3.270512
H	-1.127070	2.691299	1.483411
C	-3.175699	1.971438	4.107686
H	-4.112001	0.058469	4.454316
H	-2.088513	3.740728	3.520233
H	-3.582424	2.424025	5.008134

C	2.050754	0.670994	-0.404165
C	3.058600	0.577303	-1.376043
C	2.310139	1.483613	0.708455
C	4.277916	1.245452	-1.235535
H	2.893232	-0.018959	-2.268534
C	3.528433	2.152083	0.856277
H	1.555421	1.578253	1.481880
C	4.522387	2.037840	-0.114271
H	5.465515	2.559849	-0.002508
C	3.811957	2.897611	2.128457
C	5.363323	1.050884	-2.257358
F	2.715334	3.515290	2.618758
F	4.248708	2.051758	3.101639
F	4.867240	0.868652	-3.500423
F	4.771702	3.837559	1.974785
F	6.122821	-0.042193	-1.980701
F	6.210200	2.104493	-2.309077
C	-2.848599	0.282973	-0.911560
H	-2.128726	-0.050434	-1.647584
H	-3.571053	-0.466922	-0.605127
C	-3.276009	1.612069	-0.940212
O	-4.403585	1.976120	-0.336261
Si	-6.012681	1.348817	-0.320966
C	-7.052874	2.902311	-0.159700
H	-6.898772	3.579088	-1.007544
H	-8.120596	2.654891	-0.118574
H	-6.803882	3.448666	0.757232
C	-6.306390	0.476743	-1.962860
H	-5.657591	-0.396649	-2.088519
H	-7.346088	0.131062	-2.019613
H	-6.134582	1.149469	-2.810875
C	-6.265394	0.212510	1.146749
H	-5.932746	0.687869	2.075201
H	-7.334053	-0.017250	1.250166
H	-5.730604	-0.737767	1.044798
C	-2.490904	2.725455	-1.482255
C	-1.318477	2.507110	-2.234742
C	-2.912787	4.051911	-1.245905
C	-0.593517	3.588479	-2.728928
H	-0.963804	1.505165	-2.440943
C	-2.181454	5.123934	-1.741270
H	-3.808275	4.224519	-0.661054
C	-1.019114	4.895224	-2.485359
H	0.308275	3.404264	-3.304809
H	-2.513553	6.139836	-1.546924
H	-0.448620	5.734581	-2.873684

TS 8

B3LYP/6-31G(d) = -3573.933545
B3LYP-D3(BJ)/def2-TZVPP/IEFPCM(propanonitrile) = -3575.512502
B3LYP-D3(BJ)/def2-TZVPP/IEFPCM(propanonitrile)//B3LYP-D3(BJ)/6-31G(d) Free Energy (Quasiharmonic) = -3574.796295

Frequencies (Top 3 out of 300)

1. -253.7253 cm^{-1}
2. 5.7959 cm^{-1}
3. 8.7551 cm^{-1}

B3LYP/6-31G(d) Molecular Geometry in Cartesian Coordinates

B	-0.113528	-0.311606	0.000260
O	0.489096	-0.231830	1.346738
N	0.065028	-1.825619	-0.317839
C	0.537927	-2.551476	0.866435
S	-0.684511	-2.625653	-1.560324
C	0.857653	-1.418685	1.848942
O	1.362405	-1.568086	2.938301
H	1.382103	-4.391289	0.143418
H	-0.278103	-3.136716	1.322275
C	1.738059	-3.518322	0.695043
C	2.961437	-2.971882	0.014098
C	4.144690	-2.405519	0.623417
C	5.063061	-2.117887	-0.428410
N	4.458368	-2.495576	-1.608560
C	3.199738	-2.995994	-1.339880
H	2.553438	-3.348776	-2.131062
C	6.678581	-1.305277	1.129409
C	4.530838	-2.130990	1.948618
H	7.015397	-1.359722	-1.007833
H	4.834710	-2.347826	-2.531965
C	5.789705	-1.590016	2.187067
H	7.656778	-0.885636	1.349044
H	3.843561	-2.320997	2.767243
C	6.326828	-1.564435	-0.191378
H	6.101427	-1.389704	3.209158
C	-2.233158	-3.270951	-0.908062
O	0.126847	-3.801879	-1.916714
O	-1.001508	-1.611415	-2.574574
C	-4.609358	-4.200632	0.257469
C	-2.246684	-4.502788	-0.250134
C	-3.402730	-2.513730	-1.019081
C	-4.576459	-2.981603	-0.436723
C	-3.432935	-4.956136	0.328884
H	-1.347007	-5.107842	-0.212876
H	-3.387240	-1.574035	-1.558334
H	-5.481562	-2.384284	-0.511316
H	-3.441778	-5.913653	0.843922
C	-5.886936	-4.670925	0.910380
H	-5.752445	-5.634565	1.411040
H	-6.693526	-4.782950	0.175456
H	-6.230762	-3.945918	1.658320
H	1.990753	-3.852736	1.708011
C	-1.612903	0.290949	-0.016087
C	-2.155524	0.961433	-1.121486
C	-2.446045	0.127444	1.101661
C	-3.461303	1.463076	-1.107038
H	-1.557888	1.078643	-2.017301
C	-3.763689	0.592633	1.109778
H	-2.063727	-0.372573	1.986083
C	-4.278894	1.275673	0.007274
H	-5.294760	1.653422	0.017201
C	-4.666010	0.291450	2.274371
C	-3.954540	2.278101	-2.269430
F	-5.595908	1.257288	2.459002
F	-3.983296	0.160206	3.431392
F	-5.347547	-0.868785	2.087500
F	-3.486867	1.822259	-3.449654
F	-3.549455	3.577497	-2.170857
F	-5.302818	2.303511	-2.345872
O	0.713547	0.526644	-0.982289
C	2.007491	0.610507	-0.946950
C	2.706461	0.743780	-2.247429
C	1.966363	0.761814	-3.440608
C	4.109769	0.793081	-2.306651
C	2.620984	0.849402	-4.668245
H	0.888182	0.659433	-3.395197
C	4.758403	0.884446	-3.535012
H	4.693600	0.745403	-1.391473
C	4.014640	0.919803	-4.719410
H	2.040903	0.851016	-5.586636
H	5.843713	0.928200	-3.570915
H	4.521941	0.991512	-5.677739
H	2.530696	0.079611	-0.150873
C	2.575017	2.515709	-0.118983
H	2.319751	3.088744	-1.001491
H	3.628878	2.303798	0.001945
C	1.801672	2.702081	1.025884
C	2.181200	2.242355	2.369038
C	1.302198	2.457020	3.447935

C	3.407836	1.596246	2.614717
C	1.642953	2.045489	4.730686
H	0.348675	2.936165	3.263110
C	3.741978	1.182783	3.898912
H	4.104459	1.395147	1.808297
C	2.863594	1.407285	4.960809
H	0.951351	2.212462	5.551387
H	4.685445	0.672920	4.064860
H	3.126209	1.078472	5.962433
O	0.600939	3.256572	0.951443
Si	-0.112438	4.478230	-0.050891
C	-0.103278	3.978012	-1.865403
H	-1.032477	4.300483	-2.348090
H	0.736914	4.420675	-2.412528
H	-0.044352	2.891037	-1.970528
C	0.901050	6.038035	0.238278
H	0.490952	6.870356	-0.347306
H	0.890753	6.334985	1.292877
H	1.946108	5.906438	-0.065289
C	-1.837369	4.611952	0.661626
H	-2.355846	3.649038	0.622878
H	-1.809714	4.943101	1.705746
H	-2.436751	5.332099	0.092352

TS 9

B3LYP/6-31G(d) = -3573.936609
B3LYP-D3(BJ)/def2-TZVPP/IEFPCM(propanonitrile) = -3575.513979
B3LYP-D3(BJ)/def2-TZVPP/IEFPCM(propanonitrile)//B3LYP-D3(BJ)/6-31G(d) Free Energy (Quasiharmonic) = -3574.796224

Frequencies (Top 3 out of 300)

1. -261.2703 cm^{-1}
2. 10.0357 cm^{-1}
3. 14.2348 cm^{-1}

B3LYP/6-31G(d) Molecular Geometry in Cartesian Coordinates

B	-0.745428	-0.597225	0.767806
O	-0.567097	-1.563574	1.880907
N	-0.672963	-1.572474	-0.467960
C	-0.763886	-2.969813	-0.009074
S	-1.161307	-1.194078	-2.005042
C	-0.567877	-2.840642	1.506585
O	-0.452215	-3.780594	2.262628
H	-0.198270	-4.266531	-1.628613
H	-1.787300	-3.357619	-0.144619
C	0.183733	-4.024542	-0.634709
C	1.645838	-3.691522	-0.758254
C	2.702294	-3.865691	0.218535
C	3.928277	-3.525319	-0.422618
N	3.620328	-3.132548	-1.711868
C	2.255531	-3.259507	-1.910275
H	1.802617	-3.016777	-2.861027
C	5.168737	-4.044301	1.548790
C	2.740035	-4.293720	1.559563
H	6.091792	-3.368422	-0.289795
H	4.294790	-2.965655	-2.442394
C	3.967369	-4.376946	2.207083
H	6.113179	-4.131376	2.079598
H	1.820153	-4.539964	2.080481
C	5.165968	-3.613070	0.225427
H	4.002788	-4.706008	3.241921
C	-2.945254	-1.380952	-2.113709
O	-0.591766	-2.183645	-2.934165
O	-0.854697	0.236219	-2.190255
C	-5.729307	-1.710809	-2.162189
C	-3.489205	-2.652411	-2.315091
C	-3.776070	-0.266006	-1.976443
C	-5.156579	-0.439875	-2.005597
C	-4.875010	-2.807537	-2.331751
H	-2.838417	-3.503814	-2.483340
H	-3.344611	0.719671	-1.848226
H	-5.802648	0.426664	-1.891289
H	-5.297824	-3.797831	-2.481627
C	-7.227652	-1.884784	-2.119617
H	-7.593127	-1.766342	-1.092050
H	-7.529712	-2.875700	-2.472294
H	-7.737019	-1.133701	-2.734043
O	0.419674	0.379757	0.828458
C	1.652634	-0.049824	0.738828
H	1.786226	-1.087405	0.425915
H	0.058584	-4.918295	-0.011411
C	2.634674	0.452968	1.721845
C	3.801502	-0.287829	1.964955
C	2.384846	1.618720	2.462936
C	4.708482	0.134841	2.936322
H	3.981491	-1.211550	1.421166
C	3.297619	2.039852	3.426351
H	1.464590	2.166949	2.292441
C	4.461067	1.301405	3.664463
H	5.595708	-0.459323	3.137097
H	3.095723	2.937428	4.004421
H	5.163981	1.625984	4.427153
C	-2.095489	0.263706	0.977621
C	-3.255975	-0.363144	1.464142
C	-2.182699	1.630707	0.692539
C	-4.450642	0.335922	1.633224
H	-3.229294	-1.416252	1.724802
C	-3.379777	2.339012	0.858554
H	-1.303221	2.155157	0.336575
C	-4.522201	1.697030	1.327758
H	-5.447622	2.245080	1.463769
C	-3.439929	3.783555	0.453011
C	-5.703074	-0.365788	2.079158
F	-4.530433	4.416674	0.934756
F	-2.354430	4.478146	0.865602
F	-5.457396	-1.572129	2.626792
F	-3.481567	3.914710	-0.903964
F	-6.553794	-0.570519	1.035601
F	-6.392230	0.362860	2.987616
C	2.344072	0.544282	-1.128278
H	1.386513	0.387417	-1.610260
H	3.082052	-0.237159	-1.273819
C	2.815832	1.844044	-0.955231
O	4.083213	2.057780	-0.606011
Si	5.633503	1.721808	-1.258324
C	5.763562	2.700766	-2.858370
H	5.019440	2.372138	-3.593432
H	6.754210	2.571439	-3.311641
H	5.612157	3.772095	-2.686033
C	6.790312	2.347973	0.074964
H	6.588972	1.847423	1.028329
H	7.837127	2.161555	-0.193384
H	6.671798	3.426042	0.230805
C	5.878398	-0.114172	-1.584369
H	5.695032	-0.717995	-0.689470
H	5.224200	-0.478131	-2.384343
H	6.914741	-0.289650	-1.901138
C	1.973058	3.043711	-0.961652
C	0.659104	3.015967	-1.472450
C	2.484735	4.256699	-0.454403
C	-0.114704	4.173374	-1.469281
H	0.238802	2.101297	-1.873649
C	1.702208	5.405368	-0.450165
H	3.488922	4.277668	-0.048055
C	0.399721	5.366205	-0.956112
H	-1.130735	4.138059	-1.847095
H	2.103416	6.330592	-0.046161
H	-0.215671	6.261401	-0.946506

TS 10

B3LYP/6-31G(d) = -3573.936914
B3LYP-D3(BJ)/def2-TZVPP/IEFPCM(propanonitrile) = -3575.512668
B3LYP-D3(BJ)/def2-TZVPP/IEFPCM(propanonitrile)//B3LYP-D3(BJ)/6-31G(d) Free Energy (Quasiharmonic) = -3574.796041

Frequencies (Top 3 out of 300)

1. -274.5961 cm^{-1}
2. 5.5567 cm^{-1}
3. 12.5727 cm^{-1}

B3LYP/6-31G(d) Molecular Geometry in Cartesian Coordinates

B	-0.593220	0.085590	-0.447590
O	-0.214560	0.037430	-1.894760
N	-0.705700	1.635730	-0.248650
C	-0.769060	2.312650	-1.554130
S	-1.359840	2.354810	1.102620
C	-0.359950	1.202050	-2.526920
O	-0.167450	1.354820	-3.715680
H	-0.193299	4.324670	-1.034760
H	-1.806460	2.591380	-1.800410
C	0.100811	3.580050	-1.776860
C	1.597140	3.404530	-1.768710
C	2.526191	3.650479	-0.686840
C	3.838190	3.421379	-1.195290
N	3.702560	3.046119	-2.515750
C	2.362410	3.051269	-2.855410
H	2.042600	2.794150	-3.855180
C	4.813451	3.981759	0.909060
C	2.384101	4.059949	0.653140
H	5.979600	3.406649	-0.822250
H	4.461330	2.873229	-3.156470
C	3.525351	4.222519	1.432430
H	5.685031	4.125339	1.542280
H	1.396451	4.237930	1.067770
C	4.987401	3.577039	-0.410910
H	3.423921	4.547869	2.464410
C	-3.151700	2.399620	0.942530
O	-0.928539	3.763420	1.116460
O	-1.030500	1.465880	2.223630
C	-5.945390	2.467471	0.696640
C	-3.931050	1.478691	1.644790
C	-3.754470	3.377601	0.145440
C	-5.142640	3.398961	0.023430
C	-5.317440	1.519671	1.516420
H	-3.461320	0.751860	2.294920
H	-3.149659	4.134750	-0.342190
H	-5.610549	4.161191	-0.594960
H	-5.917410	0.800821	2.068990
C	-7.446650	2.477091	0.535130
H	-7.950560	2.165591	1.456140
H	-7.815470	3.471591	0.263810
H	-7.760270	1.784171	-0.256920
H	-0.202899	3.959390	-2.759290
C	-1.906130	-0.822000	-0.182600
C	-2.841520	-1.058290	-1.201480
C	-2.170540	-1.391090	1.071810
C	-3.994730	-1.816249	-0.978460
C	-3.331890	-2.132230	1.306150
H	-1.470220	-1.229690	1.884410
C	-4.252150	-2.353339	0.282170
H	-5.152081	-2.929069	0.463470
C	-3.637051	-2.596510	2.701420
C	-4.934450	-2.103209	-2.116960
F	-4.529651	-3.611439	2.726500
F	-4.171570	-1.589799	3.446140
F	-2.531561	-3.013350	3.356090
F	-4.989250	-1.079979	-2.998900
F	-6.197220	-2.333379	-1.687360
F	-4.556291	-3.200209	-2.817610
O	0.507620	-0.529090	0.378170
C	1.574220	0.120660	0.770650
C	2.215340	-0.342670	2.020420
C	3.055240	0.531269	2.726000
C	1.952150	-1.620380	2.536930
C	3.621500	0.132189	3.936090
H	3.243220	1.529619	2.338220
C	2.522220	-2.014511	3.744340
H	1.284640	-2.282720	1.995730
C	3.358290	-1.140461	4.445880
H	4.258990	0.819289	4.485710
H	2.307089	-3.000940	4.145870
H	3.794960	-1.448591	5.392120
H	1.571950	1.200180	0.612290
C	2.956680	-0.056621	-0.723480
H	3.630560	0.737289	-0.426570
H	2.259310	0.202930	-1.510480
C	3.416750	-1.373221	-0.683340
C	4.582610	-1.809661	0.104340
C	5.485960	-0.886331	0.667670
C	4.823089	-3.186011	0.286200
C	6.590340	-1.330171	1.388420
H	5.339870	0.179719	0.535930
C	5.925639	-3.624051	1.011070
H	4.129379	-3.900961	-0.139510
C	6.813369	-2.697531	1.564220
H	7.279520	-0.606311	1.813390
H	6.093359	-4.688621	1.147100
H	7.677089	-3.039761	2.127680
O	2.738470	-2.348021	-1.261610
Si	2.146759	-2.758300	-2.841060
C	2.323300	-1.308810	-4.019100
H	3.304080	-0.826431	-3.928400
H	2.244930	-1.687440	-5.046780
H	1.544050	-0.551440	-3.888170
C	0.391899	-3.350190	-2.598230
H	-0.002631	-3.757040	-3.537810
H	0.337859	-4.142620	-1.843330
H	-0.257531	-2.528110	-2.286230
H	-2.671450	-0.649740	-2.193020
C	3.279869	-4.168561	-3.366980
H	4.325489	-3.846471	-3.436530
H	2.984689	-4.549201	-4.352830
H	3.232099	-5.007011	-2.662540

TS 11

B3LYP/6-31G(d) = -3573.93818
B3LYP-D3(BJ)/def2-TZVPP/IEFPCM(propanonitrile) = -3575.51364
B3LYP-D3(BJ)/def2-TZVPP/IEFPCM(propanonitrile)//B3LYP-D3(BJ)/6-31G(d) Free Energy (Quasiharmonic) = -3574.795692

Frequencies (Top 3 out of 300)

1. -271.4965 cm^{-1}
2. 10.1935 cm^{-1}
3. 12.8505 cm^{-1}

B3LYP/6-31G(d) Molecular Geometry in Cartesian Coordinates

B	-0.378446	-0.502150	0.149197
O	0.014686	-0.605585	1.591662
N	-0.644378	-2.001244	-0.169962
C	-0.632802	-2.818438	1.050300
S	-1.268142	-2.565211	-1.598405
C	-0.153222	-1.820102	2.110309

O	0.054758	-2.092179	3.275439
H	-0.287551	-4.860503	0.466070
H	-1.652651	-3.119659	1.340620
C	0.229721	-4.111385	1.068175
C	1.650731	-4.000568	0.588618
C	2.837279	-3.657086	1.344291
C	3.947962	-3.769713	0.456966
N	3.449171	-4.155255	-0.770334
C	2.077731	-4.291130	-0.685550
H	1.486956	-4.576524	-1.544060
C	5.467046	-3.134998	2.185140
C	3.080011	-3.277546	2.678884
H	6.097032	-3.617770	0.171360
H	3.994107	-4.312324	-1.603633
C	4.385544	-3.020227	3.082473
H	6.476641	-2.933153	2.533159
H	2.254158	-3.173428	3.375641
C	5.263226	-3.512253	0.861727
H	4.578477	-2.728060	4.111306
C	-3.060868	-2.537755	-1.461862
O	-0.898461	-3.984200	-1.737405
O	-0.892200	-1.589359	-2.627558
C	-5.844052	-2.409334	-1.147505
C	-3.775600	-1.487394	-2.042432
C	-3.725039	-3.545695	-0.759047
C	-5.109193	-3.469588	-0.601647
C	-5.157423	-1.434287	-1.886545
H	-3.245038	-0.727834	-2.605424
H	-3.171235	-4.389886	-0.361587
H	-5.625967	-4.248779	-0.046747
H	-5.712042	-0.611203	-2.329310
C	-7.333435	-2.292542	-0.932633
H	-7.557711	-1.417656	-0.310494
H	-7.867139	-2.161690	-1.881449
H	-7.740625	-3.177280	-0.433823
H	0.209092	-4.455618	2.109269
C	-1.585031	0.571437	0.013876
C	-2.850807	0.325053	0.573693
C	-1.372196	1.843835	-0.526749
C	-3.847690	1.302157	0.595635
C	-2.354561	2.839103	-0.481182
H	-0.408833	2.074916	-0.966298
C	-3.601361	2.577088	0.078424
H	-4.362790	3.347542	0.121212
C	-1.995923	4.218053	-0.951131
C	-5.214835	1.000717	1.144818
F	-1.123643	4.820282	-0.085536
F	-3.063677	5.034977	-1.052815
F	-1.375077	4.206080	-2.151989
F	-6.127681	0.822179	0.150999
F	-5.684097	2.016259	1.906965
F	-5.237170	-0.116518	1.899798
O	0.772364	0.057369	-0.640165
C	1.989817	-0.417026	-0.601438
C	2.803112	-0.234591	-1.829319
C	4.076049	-0.821396	-1.930546
C	2.282726	0.470046	-2.924363
C	4.819010	-0.692271	-3.100857
H	4.476669	-1.385407	-1.091709
C	3.029694	0.595917	-4.094686
H	1.280839	0.877554	-2.859803
C	4.298391	0.021038	-4.185383
H	5.801885	-1.150895	-3.171776
H	2.614182	1.134819	-4.941459
H	4.876798	0.117933	-5.100317
H	2.140588	-1.357796	-0.068176
C	3.063788	0.543616	0.867543
H	4.057961	0.278849	0.532680
H	2.694216	-0.014672	1.719661
C	2.640213	1.870371	0.745490
C	3.189387	2.822790	-0.231420
C	2.418787	3.937124	-0.621919
C	4.468931	2.649455	-0.793521
C	2.914085	4.841556	-1.555608
H	1.426025	4.072173	-0.208433
C	4.963027	3.564909	-1.717250
H	5.087786	1.810372	-0.496699
C	4.187203	4.660916	-2.102504
H	2.303619	5.686917	-1.859256
H	5.954885	3.423638	-2.136387
H	4.574285	5.372525	-2.826688
O	1.572400	2.287856	1.396828
Si	1.173002	2.505582	3.076942
C	2.091197	4.080343	3.555630
H	1.793922	4.925712	2.925126
H	1.874033	4.347988	4.597291
H	3.177682	3.960716	3.467633
C	1.740026	1.066875	4.136660
H	1.217121	0.135900	3.894648
H	2.820021	0.893851	4.063633
H	1.524813	1.307480	5.186644
H	-3.068940	-0.645216	1.008742
C	-0.678130	2.754289	3.069648
H	-1.024011	3.065919	4.062902
H	-0.969996	3.526072	2.349735
H	-1.193776	1.827220	2.803149

TS 12

B3LYP/6-31G(d) = -3573.937565
B3LYP-D3(BJ)/def2-TZVPP/IEFPCM(propanonitrile) = -3575.513112
B3LYP-D3(BJ)/def2-TZVPP/IEFPCM(propanonitrile)//B3LYP-D3(BJ)/6-31G(d) Free Energy (Quasiharmonic) = -3574.795328

Frequencies (Top 3 out of 300)

1. -241.6699 cm^{-1}
2. 10.1606 cm^{-1}
3. 15.6767 cm^{-1}

B3LYP/6-31G(d) Molecular Geometry in Cartesian Coordinates

B	-0.844971	-0.486405	0.983355
O	-0.812633	-1.286245	2.234691
N	-0.689207	-1.628926	-0.087115
C	-0.895749	-2.942787	0.550203
S	-1.032015	-1.463138	-1.701718
C	-0.870601	-2.602315	2.045506
O	-0.896334	-3.426096	2.934188
H	0.041076	-4.297298	-0.840881
H	-1.905108	-3.325347	0.327969
C	0.095740	-4.086781	0.228811
C	1.533979	-3.894569	0.635117
C	2.667896	-3.657079	-0.230213
C	3.836222	-3.677779	0.585508
N	3.419310	-3.888142	1.884224
C	2.044441	-4.036550	1.904765
H	1.520578	-4.232102	2.829621
C	5.235686	-3.342579	-1.318050
C	2.813631	-3.456708	-1.616047
H	6.004618	-3.566312	0.693350
H	4.023857	-4.016746	2.680783
C	4.091632	-3.304939	-2.143751
H	6.221662	-3.238494	-1.763177
H	1.937864	-3.410698	-2.256178
C	5.123503	-3.523680	0.057454
H	4.214617	-3.166200	-3.214704
C	-2.799421	-1.670164	-1.949459
O	-0.386779	-2.568269	-2.428862
O	-0.698304	-0.069566	-2.047118
C	-5.569742	-2.009902	-2.211439

C	-3.332240	-2.957496	-2.056369
C	-3.630628	-0.548594	-2.013357
C	-5.003874	-0.727719	-2.147167
C	-4.712429	-3.116675	-2.180438
H	-2.674782	-3.820169	-2.068994
H	-3.203829	0.445902	-1.958415
H	-5.651276	0.144299	-2.186315
H	-5.127819	-4.118478	-2.255943
C	-7.067383	-2.179239	-2.286343
H	-7.504262	-1.547576	-3.068490
H	-7.347160	-3.217034	-2.491404
H	-7.529725	-1.885298	-1.335954
H	-0.308616	-4.960436	0.753360
C	-2.177730	0.426967	0.945022
C	-2.190410	1.751388	0.493356
C	-3.400110	-0.108300	1.387794
C	-3.371855	2.503722	0.461888
C	-4.580427	0.633658	1.357237
H	-3.435888	-1.122212	1.773004
C	-4.574943	1.949980	0.890619
H	-5.488470	2.533402	0.874357
C	-5.892282	0.018821	1.758808
C	-3.344088	3.895347	-0.102709
F	-6.641508	-0.306572	0.668967
F	-6.645274	0.872262	2.490622
F	-5.739259	-1.109529	2.479347
F	-3.273145	3.877208	-1.464057
F	-2.269477	4.599059	0.322259
F	-4.444844	4.609076	0.215889
O	0.345196	0.462242	1.016144
C	1.572433	0.018235	1.069473
C	2.497630	0.674811	2.015027
C	2.172755	1.909266	2.598926
C	3.685876	0.026082	2.383415
C	3.028131	2.483231	3.536161
H	1.239420	2.391241	2.329638
C	4.535043	0.601553	3.326795
H	3.931267	-0.936079	1.940721
C	4.209582	1.832304	3.902486
H	2.767224	3.434175	3.992289
H	5.445956	0.086693	3.620352
H	4.869517	2.278117	4.641863
H	1.714226	-1.054382	0.921920
C	2.396034	0.303948	-0.847507
H	1.444921	0.126515	-1.334516
H	3.094744	-0.526834	-0.856607
C	2.928077	1.589107	-0.835153
C	2.145137	2.815596	-1.022621
C	0.851170	2.783536	-1.581748
C	2.699994	4.058065	-0.651635
C	0.139257	3.966734	-1.760277
H	0.403098	1.844905	-1.886616
C	1.977875	5.233257	-0.826019
H	3.690398	4.083376	-0.212841
C	0.695382	5.190413	-1.379888
H	-0.860442	3.931263	-2.179227
H	2.411658	6.183075	-0.525970
H	0.128154	6.107431	-1.512747
O	4.203768	1.783694	-0.500997
Si	5.711495	1.344383	-1.206381
C	6.618926	2.977989	-1.406143
H	7.619131	2.819427	-1.827919
H	6.078872	3.657721	-2.074902
H	6.742693	3.483374	-0.441287
C	5.389683	0.522434	-2.864143
H	6.343537	0.343277	-3.376323
H	4.885586	-0.443632	-2.756683
H	4.779116	1.155132	-3.518635
H	-1.264021	2.208345	0.164306
C	6.609097	0.221117	-0.002900
H	6.128522	-0.760616	0.065732
H	7.644514	0.065169	-0.331436
H	6.636874	0.661576	0.999585

TS 13

B3LYP/6-31G(d) = -3573.936691
B3LYP-D3(BJ)/def2-TZVPP/IEFPCM(propanonitrile) = -3575.512181
B3LYP-D3(BJ)/def2-TZVPP/IEFPCM(propanonitrile)//B3LYP-D3(BJ)/6-31G(d) Free Energy (Quasiharmonic) = -3574.795165

Frequencies (Top 3 out of 300)

1. -277.3009 cm^{-1}
2. 8.0729 cm^{-1}
3. 12.9390 cm^{-1}

B3LYP/6-31G(d) Molecular Geometry in Cartesian Coordinates

B	-0.688609	0.134324	-0.542956
O	-0.295188	0.144012	-1.987335
N	-0.709478	1.673878	-0.259692
C	-0.737062	2.421366	-1.526973
S	-1.313241	2.360907	1.130685
C	-0.398939	1.345928	-2.563886
O	-0.228550	1.549839	-3.745385
H	-0.031010	4.363233	-0.908364
H	-1.755964	2.776245	-1.750286
C	0.210443	3.639695	-1.689170
C	1.692959	3.369809	-1.699792
C	2.642062	3.544577	-0.621512
C	3.937401	3.271185	-1.148807
N	3.771725	2.918344	-2.473574
C	2.429737	3.002474	-2.801013
H	2.088866	2.784278	-3.803183
C	4.957641	3.772383	0.949697
C	2.529340	3.929828	0.728494
H	6.082460	3.190942	-0.806419
H	4.517246	2.775349	-3.137180
C	3.684542	4.039450	1.496050
H	5.842038	3.881581	1.571817
H	1.552950	4.131410	1.158726
C	5.101048	3.382724	-0.378876
H	3.606905	4.346730	2.535547
C	-3.097835	2.535163	0.976265
O	-0.787418	3.734238	1.219430
O	-1.046723	1.393611	2.202386
C	-5.880425	2.798336	0.734303
C	-3.632815	3.595585	0.238298
C	-3.938268	1.627233	1.622368
C	-5.318623	1.764901	1.496329
C	-5.016174	3.714287	0.117969
H	-2.978057	4.338904	-0.203985
H	-3.519763	0.834971	2.229150
H	-5.965100	1.053591	2.004369
H	-5.431266	4.540666	-0.454034
C	-7.377147	2.915209	0.572500
H	-7.728856	2.308496	-0.272558
H	-7.681259	3.949322	0.380321
H	-7.905179	2.564302	1.465319
H	-0.071443	4.087601	-2.648985
C	-2.046607	-0.721656	-0.342420
C	-2.941981	-0.917779	-1.404211
C	-2.382353	-1.299006	0.891287
C	-4.126528	-1.642548	-1.241488
H	-2.713234	-0.506131	-2.382530
C	-3.575814	-2.004810	1.065461
H	-1.710812	-1.174082	1.734218
C	-4.456767	-2.184853	-0.000433
H	-5.380695	-2.734747	0.133473
C	-3.952159	-2.480538	2.439239

C	-5.016686	-1.890596	-2.427608
F	-4.466980	-1.464538	3.186544
F	-2.891269	-2.959852	3.124106
F	-4.890346	-3.453377	2.412664
F	-5.039405	-0.836316	-3.273300
F	-6.294993	-2.142699	-2.062249
F	-4.600895	-2.959379	-3.152058
O	0.362431	-0.584795	0.271358
C	1.495061	-0.047488	0.644774
C	2.105702	-0.575925	1.884775
C	1.771607	-1.850154	2.369877
C	2.977742	0.239427	2.619784
C	2.311645	-2.301845	3.570988
H	1.073380	-2.463970	1.810945
C	3.509490	-0.214782	3.826626
H	3.215616	1.237692	2.260419
C	3.181823	-1.486599	4.301463
H	2.043352	-3.285351	3.946837
H	4.167590	0.430317	4.402364
H	3.591786	-1.837279	5.244885
H	1.604436	1.026049	0.479916
C	2.825288	-0.392954	-0.878141
H	3.568239	0.369161	-0.664418
H	2.104092	-0.127522	-1.640230
C	3.221052	-1.727119	-0.759058
C	2.402060	-2.874299	-1.161578
C	1.226098	-2.712833	-1.922895
C	2.791767	-4.175475	-0.776088
C	0.464580	-3.823218	-2.278261
H	0.897628	-1.732289	-2.243128
C	2.025103	-5.276795	-1.134765
H	3.690195	-4.303858	-0.184369
C	0.858448	-5.103817	-1.887286
H	-0.441330	-3.682286	-2.859857
H	2.332527	-6.272053	-0.826422
H	0.259616	-5.966183	-2.167380
O	4.346846	-2.047994	-0.127778
Si	5.969244	-1.460172	-0.198139
C	6.257698	-0.143051	1.102597
H	5.741186	0.796672	0.879568
H	5.925000	-0.483320	2.088554
H	7.331754	0.075790	1.166709
C	6.272776	-0.821316	-1.942271
H	7.320565	-0.514194	-2.049306
H	6.076212	-1.594889	-2.693327
H	5.646441	0.045380	-2.179400
C	6.973829	-3.002060	0.168315
H	6.715826	-3.414077	1.150629
H	6.799800	-3.782646	-0.580672
H	8.047268	-2.777163	0.174563

TS 14

B3LYP/6-31G(d) = -3573.939014
B3LYP-D3(BJ)/def2-TZVPP/IEFPCM(propanonitrile) = -3575.512563
B3LYP-D3(BJ)/def2-TZVPP/IEFPCM(propanonitrile)//B3LYP-D3(BJ)/6-31G(d) Free Energy (Quasiharmonic) = -3574.794877

Frequencies (Top 3 out of 300)

1. -267.6406 cm^{-1}
2. 12.6497 cm^{-1}
3. 13.8223 cm^{-1}

B3LYP/6-31G(d) Molecular Geometry in Cartesian Coordinates

B	-0.684663	0.344426	-0.343155
O	-0.155425	0.425038	-1.738607
N	-0.847250	1.864864	-0.023490
C	-0.706809	2.670249	-1.244569
S	-1.632995	2.484096	1.302922
C	-0.186160	1.654448	-2.263656
O	0.154766	1.915953	-3.395594
H	-0.347255	4.705122	-0.644779
H	-1.690557	3.000916	-1.616182
C	0.193819	3.934315	-1.196700
C	1.568494	3.793580	-0.601301
C	2.805392	3.424191	-1.258995
C	3.846667	3.540215	-0.292882
N	3.259153	3.932892	0.894460
C	1.899965	4.101143	0.696151
H	1.249164	4.413098	1.500491
C	5.488528	2.895551	-1.900981
C	3.143100	3.027604	-2.567213
H	5.969552	3.399309	0.149385
H	3.755114	4.204841	1.729326
C	4.474596	2.767167	-2.872756
H	6.522184	2.701184	-2.174771
H	2.369656	2.914619	-3.320314
C	5.187999	3.282968	-0.597813
H	4.741725	2.467228	-3.882514
C	-3.382286	2.638787	0.904549
O	-1.152845	3.859841	1.517349
O	-1.499088	1.479537	2.364298
C	-6.105458	2.874831	0.268207
C	-3.829197	3.734125	0.159049
C	-4.286412	1.678102	1.361122
C	-5.636394	1.803706	1.040832
C	-5.182201	3.839848	-0.157955
H	-3.134258	4.512789	-0.136973
H	-3.944288	0.854544	1.974400
H	-6.334749	1.054100	1.404937
H	-5.528193	4.694179	-0.734900
C	-7.565100	2.980495	-0.103968
H	-7.862819	4.020670	-0.271925
H	-7.775769	2.426959	-1.028736
H	-8.208932	2.563126	0.677282
H	0.271166	4.270164	-2.237755
C	-1.964505	-0.642316	-0.288893
C	-2.440772	-1.183615	0.915564
C	-2.606601	-1.047173	-1.466978
C	-3.531854	-2.055904	0.941922
H	-1.961805	-0.903088	1.847640
C	-3.668574	-1.957250	-1.449374
H	-2.257840	-0.668043	-2.422827
C	-4.149483	-2.459664	-0.241976
H	-4.981245	-3.153431	-0.223486
C	-4.220074	-2.457678	-2.753272
C	-4.077959	-2.497458	2.269743
F	-5.427847	-3.046683	-2.614956
F	-3.394444	-3.387440	-3.311599
F	-4.351382	-1.470223	-3.665255
F	-4.891105	-3.572179	2.165592
F	-3.098541	-2.814529	3.144496
F	-4.810268	-1.508937	2.852894
O	0.350507	-0.324002	0.543791
C	1.534251	0.171663	0.783450
C	2.192895	-0.265006	2.034711
C	3.212531	0.520111	2.590942
C	1.768226	-1.423131	2.704201
C	3.799532	0.154060	3.801559
H	3.527054	1.427068	2.080894
C	2.361968	-1.788892	3.909461
H	0.960229	-2.011014	2.282660
C	3.378069	-1.003069	4.460924
H	4.576876	0.776507	4.236882
H	2.023019	-2.681519	4.427791
H	3.831561	-1.285484	5.407212
H	1.705194	1.209248	0.486838
C	2.767309	-0.433743	-0.769354
H	3.583838	0.273855	-0.662834

H	2.028600	-0.173760	-1.515968
C	3.047514	-1.780533	-0.536532
C	2.111042	-2.878849	-0.794678
C	2.388652	-4.163591	-0.279533
C	0.934672	-2.687610	-1.549008
C	1.513892	-5.218232	-0.507143
H	3.287050	-4.313849	0.307195
C	0.063349	-3.750744	-1.774762
H	0.698193	-1.717499	-1.967583
C	0.349608	-5.014488	-1.255537
H	1.735224	-6.200711	-0.099758
H	-0.844777	-3.589601	-2.347642
H	-0.333226	-5.840876	-1.433049
O	4.172776	-2.144035	0.075346
Si	5.835034	-1.752602	-0.186571
C	6.338609	-0.266075	0.841053
H	7.430697	-0.157031	0.815089
H	5.905018	0.664969	0.460268
H	6.036013	-0.383944	1.886502
C	6.072434	-1.448155	-2.025633
H	7.140384	-1.329024	-2.247519
H	5.701990	-2.287290	-2.625425
H	5.563471	-0.537990	-2.360992
C	6.709710	-3.304893	0.402647
H	7.797951	-3.195084	0.321822
H	6.474052	-3.515023	1.452224
H	6.417044	-4.179029	-0.189650

TS 15

B3LYP/6-31G(d) = -3573.934452
B3LYP-D3(BJ)/def2-TZVPP/IEFPCM(propanonitrile) = -3575.511738
B3LYP-D3(BJ)/def2-TZVPP/IEFPCM(propanonitrile)//B3LYP-D3(BJ)/6-31G(d) Free Energy (Quasiharmonic) = -3574.794328

Frequencies (Top 3 out of 300)

1. -272.9091 cm^{-1}
2. 11.6153 cm^{-1}
3. 12.6542 cm^{-1}

B3LYP/6-31G(d) Molecular Geometry in Cartesian Coordinates

B	0.943029	-0.333327	-0.991349
O	0.911939	-1.204973	-2.188866
N	0.789644	-1.402493	0.155698
C	0.928162	-2.759297	-0.408135
S	1.177833	-1.144511	1.743823
C	0.871950	-2.507658	-1.920070
O	0.832045	-3.383841	-2.755599
H	0.226437	-4.173516	1.050194
H	1.939412	-3.153100	-0.213703
C	-0.061468	-3.862010	0.043375
C	-1.531928	-3.546169	0.044769
C	-2.496242	-3.691286	-1.025403
C	-3.776656	-3.383655	-0.481410
N	-3.587072	-3.048238	0.845481
C	-2.244204	-3.160288	1.153762
H	-1.881619	-2.958487	2.150069
C	-4.833883	-3.831652	-2.571720
C	-2.412295	-4.069415	-2.378597
H	-5.920628	-3.226481	-0.799930
H	-4.309129	-2.796385	1.502805
C	-3.576919	-4.134841	-3.133686
H	-5.726585	-3.899364	-3.188162
H	-1.447317	-4.287149	-2.825731
C	-4.951188	-3.451203	-1.239295
H	-3.519356	-4.423547	-4.179668
C	2.946640	-1.367701	1.960979
O	0.527358	-2.181119	2.565147
O	0.874926	0.275594	2.018097
C	5.718609	-1.741505	2.140158
C	3.794942	-0.257040	1.960292
C	3.463874	-2.659143	2.093497
C	4.844990	-2.835385	2.176807
C	5.169231	-0.453627	2.053230
H	3.382221	0.742185	1.887821
H	2.795696	-3.511546	2.154716
H	5.248349	-3.840501	2.271088
H	5.829815	0.409263	2.041074
C	7.215501	-1.930919	2.167161
H	7.648659	-1.665056	1.194928
H	7.488109	-2.967796	2.386041
H	7.688130	-1.288245	2.919022
O	-0.241768	0.606049	-1.103117
C	-1.479014	0.341983	-0.759375
H	-1.642965	-0.572045	-0.183056
H	0.137865	-4.706940	-0.626781
C	-2.523452	0.692017	-1.753272
C	-3.810905	0.148115	-1.638886
C	-2.211338	1.502540	-2.857451
C	-4.771871	0.405892	-2.615818
H	-4.046313	-0.508860	-0.807097
C	-3.177990	1.769085	-3.823900
H	-1.201857	1.886215	-2.959314
C	-4.460334	1.222231	-3.705478
H	-5.755118	-0.048327	-2.535261
H	-2.926665	2.385526	-4.682615
H	-5.206864	1.416251	-4.471008
C	2.264534	0.596536	-0.994525
C	3.481529	0.105397	-1.494072
C	2.261661	1.906096	-0.499519
C	4.644280	0.878141	-1.478926
H	3.524635	-0.895453	-1.911686
C	3.420738	2.688533	-0.484428
H	1.339329	2.326699	-0.114562
C	4.622026	2.179020	-0.972635
H	5.520582	2.785644	-0.969623
C	3.381675	4.051622	0.145557
C	5.956120	0.299741	-1.931412
F	3.494766	3.975294	1.496220
F	4.384321	4.848961	-0.285424
F	5.801056	-0.753980	-2.757410
F	2.217639	4.695661	-0.106190
F	6.697226	-0.134742	-0.874523
F	6.717416	1.214755	-2.574840
C	-1.733676	1.646849	0.752208
H	-1.541380	2.534352	0.160390
H	-0.885980	1.272432	1.315251
C	-3.010955	1.523665	1.308706
O	-4.041563	2.200818	0.825557
Si	-4.383035	3.837558	0.375728
C	-6.024339	3.672056	-0.510784
H	-6.391365	4.653386	-0.834718
H	-6.787456	3.227464	0.137709
H	-5.922734	3.039250	-1.399132
C	-4.518151	4.775162	1.999095
H	-3.567183	4.774084	2.544495
H	-4.790483	5.821933	1.815872
H	-5.281811	4.340048	2.653123
C	-3.067012	4.588095	-0.732792
H	-3.422218	5.565933	-1.084025
H	-2.117851	4.758608	-0.213330
H	-2.874371	3.970057	-1.615700
C	-3.358879	0.546178	2.353079
C	-4.712744	0.243877	2.606006
C	-2.363205	-0.080086	3.128642
C	-5.059087	-0.666378	3.599883
H	-5.481661	0.724004	2.011736
C	-2.717717	-0.982653	4.128356
H	-1.312810	0.132238	2.965093

C	-4.061728	-1.280389	4.366549
H	-6.105897	-0.890937	3.785584
H	-1.935261	-1.456608	4.713001
H	-4.333346	-1.984047	5.148831

TS 16

B3LYP/6-31G(d) = -3573.932349
B3LYP-D3(BJ)/def2-TZVPP/IEFPCM(propanonitrile) = -3575.511771
B3LYP-D3(BJ)/def2-TZVPP/IEFPCM(propanonitrile)//B3LYP-D3(BJ)/6-31G(d) Free Energy (Quasiharmonic) = -3574.794324

Frequencies (Top 3 out of 300)

1. -283.1791 cm^{-1}
2. 10.6920 cm^{-1}
3. 14.2838 cm^{-1}

B3LYP/6-31G(d) Molecular Geometry in Cartesian Coordinates

B	-0.136270	-0.783960	-0.397180
O	0.093810	-1.298400	-1.774660
N	-0.825599	-2.014310	0.270590
C	-0.823379	-3.170000	-0.649070
S	-0.738189	-2.392530	1.893440
C	-0.261989	-2.580600	-1.944200
O	-0.141629	-3.176100	-2.991370
H	-2.453629	-4.404490	0.012290
H	-0.112629	-3.944790	-0.320090
C	-2.175619	-3.883700	-0.905700
C	-3.304249	-3.000641	-1.358380
C	-4.441749	-2.582551	-0.573840
C	-5.252650	-1.762871	-1.406070
N	-4.644170	-1.709041	-2.643620
C	-3.470259	-2.442091	-2.604190
H	-2.845669	-2.529011	-3.482980
C	-6.827910	-1.442771	0.354470
C	-4.861259	-2.834071	0.746300
H	-7.038900	-0.544391	-1.601880
H	-4.898280	-1.072981	-3.383730
C	-6.046729	-2.263701	1.195350
H	-7.747890	-1.007941	0.736320
H	-4.256769	-3.449721	1.406120
C	-6.442990	-1.181961	-0.954810
H	-6.380329	-2.452141	2.212530
C	-1.262120	-0.874510	2.684390
O	-1.722289	-3.453650	2.161400
O	0.659641	-2.652850	2.305520
C	-2.078520	1.487480	3.940730
C	-0.346050	-0.151510	3.448260
C	-2.588890	-0.454950	2.560970
C	-2.983830	0.722509	3.189260
C	-0.761990	1.025380	4.070140
H	0.672680	-0.510170	3.544880
H	-3.298220	-1.032511	1.977430
H	-4.013370	1.056779	3.087990
H	-0.050450	1.597110	4.661020
C	-2.503480	2.798090	4.558180
H	-2.370400	3.619430	3.842790
H	-3.559210	2.782349	4.848370
H	-1.907760	3.036720	5.445400
H	-1.959149	-4.646900	-1.663360
C	-0.969590	0.601230	-0.438010
C	-2.065070	0.729770	-1.304750
C	-0.622560	1.726790	0.321550
C	-2.778740	1.927799	-1.413090
C	-1.338300	2.922550	0.228990
H	0.219210	1.670790	1.000680
C	-2.421790	3.034410	-0.642560
H	-2.973500	3.964419	-0.722790
C	-0.900220	4.128830	1.008210
C	-3.967760	2.018739	-2.329090
F	-1.954620	4.860480	1.448010
F	-0.163610	3.804920	2.095200
F	-0.138940	4.968810	0.258700
F	-4.182610	3.283359	-2.759070
F	-3.818990	1.244439	-3.436700
F	-5.112110	1.612279	-1.731080
O	1.224320	-0.428830	0.221600
C	2.224160	-1.265670	0.296370
C	3.244300	-1.019110	1.341710
C	3.982061	-2.099710	1.846150
C	3.452880	0.264120	1.868540
C	4.918841	-1.901430	2.859310
H	3.789911	-3.101720	1.471200
C	4.391730	0.460010	2.879730
H	2.870000	1.096630	1.488900
C	5.128120	-0.619690	3.375860
H	5.469491	-2.748090	3.259550
H	4.543830	1.455390	3.287980
H	5.850860	-0.465679	4.172570
H	2.007801	-2.317500	0.104740
C	3.244840	-1.178700	-1.492570
H	2.343270	-1.271850	-2.084170
H	3.847901	-2.076800	-1.415840
C	3.921220	0.043390	-1.479120
C	3.279090	1.333740	-1.744230
C	3.939000	2.528560	-1.383500
C	1.999300	1.412860	-2.330150
C	3.326060	3.759340	-1.580620
H	4.920890	2.472860	-0.928650
C	1.396530	2.651400	-2.531310
H	1.466260	0.516700	-2.623850
C	2.049750	3.824370	-2.150110
H	3.836640	4.670760	-1.283150
H	0.404800	2.695800	-2.970090
H	1.564470	4.785990	-2.288700
O	5.174920	0.124550	-1.050880
Si	6.656210	-0.726439	-1.288860
C	7.201510	-0.323169	-3.041350
H	6.484660	-0.696979	-3.781790
H	7.305860	0.757151	-3.190530
H	8.171490	-0.786939	-3.259270
C	6.483451	-2.582619	-1.045360
H	7.483791	-3.034809	-1.055510
H	6.023701	-2.822469	-0.081070
H	5.903571	-3.066199	-1.838960
H	-2.360280	-0.116010	-1.916480
C	7.762120	0.031791	0.018820
H	7.868820	1.112641	-0.125330
H	7.349400	-0.136009	1.019870
H	8.765050	-0.410659	-0.011960

TS 17

B3LYP/6-31G(d) = -3573.932349
B3LYP-D3(BJ)/def2-TZVPP/IEFPCM(propanonitrile) = -3575.511767
B3LYP-D3(BJ)/def2-TZVPP/IEFPCM(propanonitrile)//B3LYP-D3(BJ)/6-31G(d) Free Energy (Quasiharmonic) = -3574.794323

Frequencies (Top 3 out of 300)

1. -283.1024 cm^{-1}
2. 10.6870 cm^{-1}
3. 14.2757 cm^{-1}

B3LYP/6-31G(d) Molecular Geometry in Cartesian Coordinates

B	-0.136212	-0.783860	-0.396985
O	0.093988	-1.298510	-1.774358

N	-0.825500	-2.014152	0.270944
C	-0.823123	-3.169996	-0.648525
S	-0.738173	-2.392117	1.893870
C	-0.261697	-2.580770	-1.943713
O	-0.141206	-3.176430	-2.990771
H	-2.453317	-4.404510	0.012934
H	-0.112334	-3.944679	-0.319361
C	-2.175281	-3.883856	-0.905131
C	-3.303957	-3.000966	-1.358039
C	-4.441556	-2.582862	-0.573657
C	-5.252458	-1.763372	-1.406069
N	-4.643879	-1.709663	-2.643573
C	-3.469904	-2.442603	-2.603937
H	-2.845232	-2.529588	-3.482661
C	-6.827896	-1.443170	0.354289
C	-4.861161	-2.834234	0.746489
H	-7.038799	-0.545074	-1.602198
H	-4.897977	-1.073718	-3.383795
C	-6.046718	-2.263914	1.195351
H	-7.747950	-1.008363	0.736003
H	-4.256672	-3.449744	1.406438
C	-6.442889	-1.182506	-0.954990
H	-6.380390	-2.452239	2.212535
C	-1.262344	-0.874048	2.684573
O	-1.722173	-3.453311	2.161899
O	0.659656	-2.652208	2.306089
C	-2.079120	1.488006	3.940529
C	-2.589163	-0.454694	2.561032
C	-0.346400	-0.150805	3.448373
C	-0.762523	1.026113	4.070061
C	-2.984290	0.722798	3.189144
H	-3.298389	-1.032439	1.977555
H	0.672373	-0.509317	3.545089
H	-0.051089	1.598026	4.660879
H	-4.013874	1.056915	3.087790
C	-2.504281	2.798647	4.557790
H	-1.909120	3.037110	5.445438
H	-3.560204	2.783054	4.847272
H	-2.370578	3.620013	3.842555
O	1.224326	-0.428548	0.221836
C	2.224161	-1.265334	0.296932
H	2.007854	-2.317227	0.105596
H	-1.958693	-4.647164	-1.662638
C	3.244313	-1.018372	1.342146
C	3.982191	-2.098747	1.846886
C	3.452794	0.265043	1.868574
C	4.918990	-1.900069	2.859947
H	3.790119	-3.100889	1.472250
C	4.391667	0.461327	2.879665
H	2.869818	1.097381	1.488694
C	5.128174	-0.618154	3.376092
H	5.469737	-2.746560	3.260426
H	4.543693	1.456852	3.287607
H	5.850930	-0.463835	4.172732
C	-0.969631	0.601256	-0.438078
C	-2.065061	0.729572	-1.304913
C	-0.622782	1.726949	0.321375
C	-2.778854	1.927499	-1.413454
H	-2.360129	-0.116323	-1.916564
C	-1.338676	2.922615	0.228630
H	0.218933	1.671128	1.000587
C	-2.422113	3.034234	-0.643017
H	-2.973929	3.964169	-0.723390
C	-0.900845	4.129022	1.007785
C	-3.967814	2.018188	-2.329560
F	-1.955391	4.860628	1.447315
F	-0.164410	3.805284	2.094956
F	-5.112168	1.611664	-1.731599
F	-0.139496	4.968985	0.258350
F	-3.818862	1.243774	-3.437065
F	-4.182780	3.282730	-2.759711
C	3.245003	-1.179048	-1.492151
H	2.343422	-1.272365	-2.083705
H	3.847981	-2.077155	-1.415013
C	3.921433	0.042992	-1.479104
O	5.175128	0.124227	-1.050814
Si	6.656394	-0.726871	-1.288452
C	6.483505	-2.583018	-1.044744
H	5.903611	-3.066656	-1.838298
H	7.483823	-3.035275	-1.054828
H	6.023735	-2.822741	-0.080435
C	7.202004	-0.323882	-3.040905
H	7.306015	0.756445	-3.190333
H	8.172201	-0.787363	-3.258450
H	6.485473	-0.698133	-3.781429
C	7.762148	0.031458	0.019312
H	7.349197	-0.136110	1.020304
H	8.765019	-0.411128	-0.011187
H	7.869010	1.112271	-0.125016
C	3.279409	1.333298	-1.744711
C	3.939372	2.528196	-1.384355
C	1.999672	1.412294	-2.330761
C	3.326529	3.758952	-1.581959
H	4.921223	2.472594	-0.929416
C	1.396997	2.650806	-2.532410
H	1.466589	0.516067	-2.624172
C	2.050269	3.823870	-2.151576
H	3.837154	4.670437	-1.284778
H	0.405307	2.695118	-2.971284
H	1.565061	4.785472	-2.290535

TS 18

B3LYP/6-31G(d) = -3573.934213
B3LYP-D3(BJ)/def2-TZVPP/IEFPCM(propanonitrile) = -3575.511442
B3LYP-D3(BJ)/def2-TZVPP/IEFPCM(propanonitrile)//B3LYP-D3(BJ)/6-31G(d) Free Energy (Quasiharmonic) = -3574.794068

Frequencies (Top 3 out of 300)

1. -273.2728 cm^{-1}
2. 11.9245 cm^{-1}
3. 13.1164 cm^{-1}

B3LYP/6-31G(d) Molecular Geometry in Cartesian Coordinates

B	0.959906	-0.280276	-0.974086
O	0.946347	-1.085757	-2.218242
N	0.804804	-1.412690	0.109431
C	0.950096	-2.736295	-0.526325
S	1.173527	-1.243270	1.712985
C	0.910333	-2.401350	-2.022565
O	0.884214	-3.230347	-2.905509
H	0.230980	-4.226103	0.845890
H	1.959054	-3.140477	-0.340960
C	-0.043580	-3.862494	-0.147184
C	-1.514973	-3.550811	-0.149849
C	-2.463718	-3.642176	-1.239782
C	-3.752904	-3.368947	-0.698322
N	-3.583219	-3.103669	0.647137
C	-2.244302	-3.227686	0.968087
H	-1.896775	-3.078614	1.978934
C	-4.779029	-3.709585	-2.823996
C	-2.359248	-3.947624	-2.609797
H	-5.892910	-3.203079	-1.038215
H	-4.315779	-2.893562	1.307470
C	-3.513046	-3.977655	-3.383518
H	-5.662782	-3.747801	-3.455664
H	-1.387147	-4.137213	-3.054288
C	-4.916471	-3.400636	-1.475135
H	-3.439686	-4.210204	-4.442422

C	2.934900	-1.509136	1.939087
O	0.496496	-2.309465	2.473025
O	0.889187	0.165586	2.055465
C	5.697275	-1.938800	2.133814
C	3.804797	-0.415666	1.960759
C	3.425779	-2.812377	2.051812
C	4.802886	-3.016223	2.143535
C	5.173973	-0.638967	2.062151
H	3.408977	0.590929	1.896490
H	2.740263	-3.652254	2.089993
H	5.186490	-4.030380	2.222103
H	5.851414	0.210510	2.063574
C	7.190284	-2.155016	2.176312
H	7.653211	-1.596128	2.998371
H	7.651805	-1.803244	1.245483
H	7.442321	-3.212392	2.301285
O	-0.231412	0.654398	-1.045356
C	-1.469368	0.369518	-0.721153
H	-1.633847	-0.577056	-0.200134
H	0.167372	-4.670859	-0.857683
C	-2.510018	0.774625	-1.698491
C	-2.193789	1.647539	-2.752809
C	-3.797554	0.224504	-1.621403
C	-3.156332	1.968774	-3.706676
H	-1.184225	2.037038	-2.828237
C	-4.754376	0.537523	-2.586219
H	-4.036042	-0.479288	-0.829766
C	-4.438738	1.415332	-3.625731
H	-2.901580	2.633795	-4.527281
H	-5.737708	0.079074	-2.536290
H	-5.182027	1.652642	-4.382163
C	2.272801	0.662681	-0.926372
C	3.508114	0.186361	-1.394652
C	2.240923	1.974736	-0.438266
C	4.659926	0.974403	-1.355033
H	3.575791	-0.815975	-1.805461
C	3.386897	2.776796	-0.407836
H	1.305976	2.380752	-0.068191
C	4.606261	2.280384	-0.864046
H	5.496639	2.898442	-0.841504
C	3.281335	4.201395	0.060558
C	5.990367	0.411110	-1.771224
F	2.824822	5.021296	-0.917931
F	2.421668	4.328469	1.098862
F	5.869298	-0.653969	-2.588231
F	4.473168	4.698449	0.462217
F	6.716384	-0.000506	-0.694338
F	6.751428	1.330557	-2.408376
C	-1.732195	1.583892	0.859389
H	-1.541258	2.504993	0.320795
H	-0.884824	1.182717	1.404422
C	-3.011311	1.426062	1.403696
O	-4.041864	2.126795	0.955669
Si	-4.386803	3.786438	0.601775
C	-6.022542	3.669267	-0.302479
H	-6.392071	4.667387	-0.566819
H	-6.787166	3.182245	0.312918
H	-5.913524	3.093392	-1.227877
C	-4.534413	4.625213	2.277295
H	-3.587054	4.593216	2.828015
H	-4.807605	5.680601	2.154872
H	-5.301511	4.150724	2.899127
C	-3.067030	4.605710	-0.452198
H	-3.423728	5.601421	-0.747171
H	-2.121844	4.748498	0.082631
H	-2.866630	4.041861	-1.368989
C	-3.360302	0.389827	2.389163
C	-4.714182	0.068393	2.617603
C	-2.365842	-0.275803	3.132708
C	-5.061394	-0.898633	3.555935
H	-5.482188	0.578776	2.047867
C	-2.721231	-1.235777	4.077063
H	-1.315706	-0.050053	2.987194
C	-4.065080	-1.551828	4.290984
H	-6.108113	-1.137596	3.723275
H	-1.939367	-1.739833	4.636706
H	-4.337523	-2.300545	5.029945

TS 19

B3LYP/6-31G(d) = -3573.935489
B3LYP-D3(BJ)/def2-TZVPP/IEFPCM(propanonitrile) = -3575.511006
B3LYP-D3(BJ)/def2-TZVPP/IEFPCM(propanonitrile)//B3LYP-D3(BJ)/6-31G(d) Free Energy (Quasiharmonic) = -3574.79391

Frequencies (Top 3 out of 300)

1. -275.0135 cm^{-1}
2. 11.3020 cm^{-1}
3. 13.3134 cm^{-1}

B3LYP/6-31G(d) Molecular Geometry in Cartesian Coordinates

B	-0.042850	0.296660	0.013860
O	0.597290	0.117200	-1.317360
N	0.161251	1.828610	0.224300
C	0.669221	2.463140	-0.997150
S	-0.593589	2.725390	1.396600
C	0.994830	1.258379	-1.884490
O	1.535960	1.316939	-2.970080
H	1.526422	4.329569	-0.366680
H	-0.128209	3.024030	-1.513320
C	1.879731	3.424429	-0.865640
C	3.085731	2.895129	-0.142060
C	4.290031	2.338228	-0.716100
C	5.176821	2.051148	0.362020
N	4.534621	2.423678	1.524200
C	3.282391	2.918688	1.218830
H	2.613621	3.272669	1.990470
C	6.843440	1.255277	-1.149720
C	4.719741	2.074848	-2.030120
H	7.114340	1.300787	0.996990
H	4.881671	2.271418	2.458300
C	5.986770	1.539707	-2.233220
H	7.829160	0.839337	-1.340930
H	4.059441	2.270558	-2.869870
C	6.450290	1.506527	0.160740
H	6.326010	1.335697	-3.245400
C	-2.117479	3.355131	0.675210
O	0.237651	3.906070	1.686850
O	-0.947929	1.789270	2.471010
C	-4.455068	4.246632	-0.593080
C	-2.091398	4.529701	-0.080160
C	-3.307909	2.639961	0.833370
C	-4.462359	3.089322	0.200160
C	-3.258588	4.964011	-0.710160
H	-1.176338	5.107850	-0.153700
H	-3.324279	1.746841	1.446710
H	-5.383899	2.524022	0.312700
H	-3.236528	5.876151	-1.301540
C	-5.711168	4.690292	-1.303610
H	-5.552348	5.617922	-1.861890
H	-6.532588	4.858533	-0.596500
H	-6.047109	3.922472	-2.011480
H	2.149931	3.701199	-1.891250
C	-1.559600	-0.256090	0.024010
C	-2.140221	-0.843029	1.159460
C	-2.368380	-0.123579	-1.112400
C	-3.464911	-1.281859	1.155890
H	-1.548771	-0.953840	2.058930
C	-3.703220	-0.542689	-1.116880

H	-1.956640	0.311381	-2.018000
C	-4.259411	-1.129928	0.017180
H	-5.288731	-1.472478	0.011890
C	-4.571320	-0.290388	-2.317660
C	-4.082511	-1.894058	2.381560
F	-3.863140	-0.271289	-3.467650
F	-5.208260	0.906792	-2.231780
F	-5.537381	-1.229118	-2.453080
F	-4.734651	-3.050318	2.087710
F	-5.000481	-1.074678	2.946050
F	-3.171941	-2.187129	3.336010
O	0.739580	-0.486751	1.057880
C	2.022840	-0.696481	0.984560
C	2.754589	-0.760381	2.274930
C	2.052930	-0.656471	3.486010
C	4.154389	-0.878902	2.295230
C	2.743070	-0.688991	4.697570
H	0.979370	-0.505631	3.461980
C	4.838109	-0.913712	3.507690
H	4.707069	-0.930082	1.360730
C	4.132759	-0.825052	4.712930
H	2.194380	-0.596431	5.630610
H	5.920679	-1.009133	3.515090
H	4.667319	-0.852662	5.658650
H	2.554520	-0.247591	0.145580
C	2.451599	-2.627391	0.273770
H	2.583129	-3.107501	1.233260
H	3.374919	-2.398682	-0.245130
C	1.372349	-3.068601	-0.505190
C	0.167258	-3.669380	0.085150
C	0.000668	-3.771710	1.480950
C	-0.851442	-4.159500	-0.755580
C	-1.146892	-4.348490	2.014060
H	0.753648	-3.380921	2.155750
C	-1.993652	-4.740719	-0.217600
H	-0.736742	-4.071340	-1.829130
C	-2.146412	-4.835769	1.168110
H	-1.272512	-4.401930	3.090790
H	-2.772322	-5.110869	-0.878020
H	-3.048402	-5.269619	1.588440
O	1.319579	-2.907581	-1.807100
Si	2.376029	-2.705391	-3.172260
C	2.896118	-4.466131	-3.592640
H	3.460598	-4.928562	-2.774300
H	2.029108	-5.101701	-3.804650
H	3.539238	-4.472252	-4.481360
C	1.264439	-1.935681	-4.460400
H	1.754919	-1.940731	-5.441800
H	0.315599	-2.474810	-4.555610
H	1.055109	-0.894761	-4.191700
C	3.865039	-1.624142	-2.815500
H	3.573130	-0.626062	-2.475620
H	4.572379	-2.055532	-2.099230
H	4.412259	-1.485452	-3.757660

TS 20

B3LYP/6-31G(d) = -3573.932825
B3LYP-D3(BJ)/def2-TZVPP/IEFPCM(propanonitrile) = -3575.510558
B3LYP-D3(BJ)/def2-TZVPP/IEFPCM(propanonitrile)//B3LYP-D3(BJ)/6-31G(d) Free Energy (Quasiharmonic) = -3574.793409

Frequencies (Top 3 out of 300)

1. -268.7470 cm^{-1}
2. 5.4684 cm^{-1}
3. 10.9086 cm^{-1}

B3LYP/6-31G(d) Molecular Geometry in Cartesian Coordinates

B	-0.598746	-0.736815	0.797488
O	-0.438317	-1.814175	1.807492
N	-0.640124	-1.594171	-0.524993
C	-0.750107	-3.025809	-0.198154
S	-1.211068	-1.056190	-1.982271
C	-0.485763	-3.048863	1.312420
O	-0.360741	-4.060155	1.968298
H	-0.299043	-4.173923	-1.959644
H	-1.788755	-3.374915	-0.322603
C	0.139661	-4.034527	-0.969306
C	1.600100	-3.715164	-1.139939
C	2.702360	-4.006050	-0.245663
C	3.899351	-3.610237	-0.909985
N	3.531626	-3.075822	-2.131000
C	2.156674	-3.167345	-2.269441
H	1.659145	-2.818042	-3.162959
C	5.230056	-4.355363	0.924609
C	2.801147	-4.577824	1.037494
H	6.068783	-3.484094	-0.871476
H	4.168260	-2.840110	-2.876670
C	4.058649	-4.745927	1.605581
H	6.198846	-4.509316	1.392640
H	1.904745	-4.867155	1.577221
C	5.166547	-3.779771	-0.340898
H	4.142196	-5.186833	2.595039
C	-3.001256	-1.220226	-1.995071
O	-0.713899	-1.949945	-3.042469
O	-0.887280	0.380771	-2.041648
C	-5.784626	-1.536740	-1.869031
C	-3.574342	-2.451686	-2.323176
C	-3.806415	-0.133615	-1.642775
C	-5.186507	-0.299461	-1.586862
C	-4.959651	-2.601035	-2.252727
H	-2.947444	-3.273681	-2.651817
H	-3.355658	0.824481	-1.413205
H	-5.810537	0.542272	-1.298936
H	-5.404471	-3.561782	-2.499760
C	-7.278242	-1.707224	-1.735875
H	-7.821596	-0.950834	-2.314589
H	-7.581948	-1.592969	-0.687990
H	-7.604477	-2.694276	-2.077283
O	0.621997	0.160916	0.890335
C	1.829266	-0.328728	0.761451
H	1.903200	-1.355237	0.395086
H	0.027015	-4.981375	-0.427460
C	2.834454	0.046326	1.781138
C	3.977855	-0.750085	1.946189
C	2.623046	1.142209	2.631682
C	4.898090	-0.451910	2.950008
H	4.134201	-1.617019	1.309576
C	3.548004	1.437919	3.629216
H	1.719981	1.732624	2.517691
C	4.687621	0.643503	3.789717
H	5.771162	-1.084665	3.082819
H	3.375468	2.281495	4.291954
H	5.402397	0.871738	4.575906
C	-1.862940	0.201089	1.152777
C	-1.842403	1.588214	0.956070
C	-3.034145	-0.353927	1.691342
C	-2.947642	2.386519	1.269359
H	-0.946873	2.050126	0.556040
C	-4.143549	0.436017	2.000062
H	-3.083104	-1.420712	1.885552
C	-4.107301	1.815392	1.791476
H	-4.961060	2.433747	2.045061
C	-5.418749	-0.190873	2.491200
C	-2.916258	3.858446	0.970337
F	-5.218763	-1.399702	3.053013
F	-6.045348	0.585825	3.404742
F	-3.284360	4.115256	-0.311730
F	-6.307071	-0.372544	1.474635

F	-1.677005	4.386757	1.124825
F	-3.753555	4.564085	1.762195
C	2.551532	0.316065	-1.029075
H	1.676973	0.016394	-1.595173
H	3.400745	-0.355314	-1.073419
C	2.786826	1.687100	-0.917656
O	1.797276	2.541033	-1.140079
Si	1.184116	3.256247	-2.599634
C	1.343245	2.017855	-3.999629
H	1.018709	2.485042	-4.938237
H	0.709242	1.144796	-3.819603
H	2.377456	1.682885	-4.142722
C	-0.574036	3.737112	-2.204919
H	-0.638361	4.329060	-1.286584
H	-0.997486	4.338238	-3.019234
H	-1.191158	2.843005	-2.082231
C	2.260119	4.769250	-2.928986
H	2.180016	5.507231	-2.122582
H	3.318765	4.509825	-3.043924
H	1.938439	5.260279	-3.856347
C	4.043146	2.268967	-0.420257
C	4.056393	3.599113	0.045360
C	5.245544	1.534718	-0.400717
C	5.231272	4.172648	0.518430
H	3.131613	4.163515	0.047900
C	6.420657	2.115661	0.064843
H	5.269818	0.515567	-0.769314
C	6.417160	3.433561	0.527767
H	5.223262	5.195564	0.883584
H	7.341515	1.539910	0.065960
H	7.336306	3.883624	0.892605

TS 21

B3LYP/6-31G(d) = -3573.928323
B3LYP-D3(BJ)/def2-TZVPP/IEFPCM(propanonitrile) = -3575.510674
B3LYP-D3(BJ)/def2-TZVPP/IEFPCM(propanonitrile)//B3LYP-D3(BJ)/6-31G(d) Free Energy (Quasiharmonic) = -3574.793294

Frequencies (Top 3 out of 300)

1. -290.5539 cm^{-1}
2. 14.4685 cm^{-1}
3. 14.8348 cm^{-1}

B3LYP/6-31G(d) Molecular Geometry in Cartesian Coordinates

B	-0.735111	-0.352822	0.830467
O	-0.633749	-0.747301	2.246347
N	-1.740090	-1.419579	0.296031
C	-1.963646	-2.464831	1.302683
S	-2.650139	-1.365323	-1.062829
C	-1.276725	-1.881597	2.542926
O	-1.286873	-2.389302	3.642116
H	-1.923308	-4.243087	0.084894
H	-3.033984	-2.568328	1.529841
C	-1.412071	-3.881582	0.981205
C	0.079781	-3.982354	0.805862
C	0.822774	-4.079593	-0.430224
C	2.200637	-4.171521	-0.087044
N	2.281692	-4.146211	1.291194
C	1.010418	-4.017759	1.817770
H	0.858715	-3.966049	2.887151
C	2.824448	-4.273161	-2.385452
C	0.463627	-4.091682	-1.790638
H	4.258377	-4.315514	-0.763331
H	3.134059	-4.044279	1.820032
C	1.464542	-4.188381	-2.749999
H	3.583241	-4.340224	-3.160736
H	-0.576743	-3.994890	-2.084511
C	3.210995	-4.266809	-1.050020
H	1.196741	-4.188975	-3.803288
C	-4.357200	-1.194421	-0.500110
O	-2.612855	-2.642679	-1.792922
O	-2.259958	-0.130066	-1.772901
C	-7.033693	-0.896492	0.283126
C	-4.685396	-0.291898	0.513212
C	-5.349029	-1.953774	-1.122357
C	-6.677303	-1.798237	-0.728070
C	-6.016522	-0.152693	0.898859
H	-3.905066	0.282236	1.000862
H	-5.072775	-2.663512	-1.894255
H	-7.448646	-2.394450	-1.209856
H	-6.270441	0.542840	1.695482
C	-8.477360	-0.715894	0.688615
H	-8.567876	-0.468078	1.751555
H	-9.063415	-1.621064	0.499490
H	-8.946623	0.100342	0.123095
O	-1.423736	1.033038	0.880556
C	-1.293170	1.980867	-0.001355
H	-0.637468	1.793078	-0.849874
H	-1.734010	-4.515937	1.816165
C	-2.487663	2.823376	-0.276090
C	-2.690943	3.315675	-1.572223
C	-3.418976	3.121899	0.729017
C	-3.809852	4.096770	-1.859327
H	-1.991054	3.055418	-2.361332
C	-4.531408	3.908473	0.441641
H	-3.264789	2.732478	1.731269
C	-4.728488	4.399361	-0.852701
H	-3.968631	4.459957	-2.870701
H	-5.247812	4.138337	1.225556
H	-5.598859	5.010181	-1.075859
C	0.719778	-0.355322	0.111524
C	0.907999	-0.270901	-1.278605
C	1.864111	-0.581956	0.894928
C	2.179084	-0.386959	-1.851360
H	0.047370	-0.163946	-1.931885
C	3.129590	-0.727158	0.322857
H	1.752034	-0.688861	1.967348
C	3.298753	-0.620983	-1.057135
H	4.274327	-0.760643	-1.508158
C	4.330374	-1.002423	1.180762
C	2.352555	-0.231237	-3.333809
F	4.005812	-1.590409	2.357929
F	5.218421	-1.819505	0.564860
F	1.266165	-0.605492	-4.031112
F	5.015668	0.131477	1.494929
F	3.409169	-0.923549	-3.807921
F	2.585673	1.082871	-3.664012
C	-0.133615	3.523775	0.788140
H	-0.777933	3.730450	1.632831
H	-0.256430	4.206808	-0.043773
C	1.167195	3.089648	1.068206
O	2.138069	3.173266	0.171255
Si	2.479657	4.210083	-1.176991
C	2.507572	5.964158	-0.496050
H	2.757289	6.676116	-1.292269
H	1.538961	6.262677	-0.079606
H	3.259968	6.071536	0.293384
C	4.163790	3.613758	-1.731547
H	4.088478	2.612650	-2.168114
H	4.575068	4.280973	-2.498929
H	4.876914	3.577392	-0.900986
C	1.216046	4.006815	-2.552179
H	0.206650	4.331783	-2.280376
H	1.532378	4.615282	-3.409771
H	1.175749	2.968276	-2.895557
C	1.553486	2.472251	2.340793
C	0.589371	2.101025	3.299282
C	2.915477	2.239160	2.618740

C	0.979922	1.516653	4.499380
H	-0.466791	2.220032	3.091877
C	3.300219	1.667759	3.825265
H	3.659917	2.488712	1.873402
C	2.333893	1.307523	4.769697
H	0.225071	1.205723	5.214513
H	4.351985	1.482475	4.019604
H	2.635103	0.850581	5.708232

TS 22

B3LYP/6-31G(d) = -3573.932824
B3LYP-D3(BJ)/def2-TZVPP/IEFPCM(propanonitrile) = -3575.510261
B3LYP-D3(BJ)/def2-TZVPP/IEFPCM(propanonitrile)//B3LYP-D3(BJ)/6-31G(d) Free Energy (Quasiharmonic) = -3574.793252

Frequencies (Top 3 out of 300)

1. -270.7316 cm^{-1}
2. 4.0862 cm^{-1}
3. 9.5775 cm^{-1}

B3LYP/6-31G(d) Molecular Geometry in Cartesian Coordinates

B	0.642585	-0.729530	-0.802453
O	0.490902	-1.810055	-1.810394
N	0.660585	-1.583285	0.524077
C	0.774723	-3.015998	0.203144
S	1.203068	-1.041526	1.990612
C	0.533651	-3.043080	-1.311322
O	0.419682	-4.056432	-1.966190
H	0.297692	-4.165735	1.957105
H	1.810879	-3.365881	0.344885
C	-0.128175	-4.023149	0.961725
C	-1.589599	-3.699867	1.115935
C	-2.680647	-3.966051	0.200153
C	-3.884567	-3.573894	0.854194
N	-3.531279	-3.064876	2.090623
C	-2.158985	-3.170173	2.247299
H	-1.672252	-2.839047	3.153538
C	-5.193783	-4.274739	-1.013069
C	-2.765086	-4.514018	-1.094479
H	-6.052229	-3.431890	0.787264
H	-4.177074	-2.847197	2.834024
C	-4.015473	-4.661891	-1.683865
H	-6.156739	-4.412700	-1.497791
H	-1.863432	-4.802527	-1.625840
C	-5.144383	-3.723285	0.263835
H	-4.087688	-5.084742	-2.682054
C	2.994683	-1.181689	2.033084
O	0.701207	-1.943544	3.041311
O	0.860231	0.391803	2.046929
C	5.783883	-1.461344	1.959798
C	3.792191	-0.082324	1.704124
C	3.577284	-2.407990	2.363997
C	4.965402	-2.538975	2.319660
C	5.175260	-0.229923	1.674531
H	3.333443	0.871408	1.472178
H	2.955020	-3.240134	2.675408
H	5.417806	-3.495542	2.569119
H	5.793861	0.622145	1.405790
C	7.281635	-1.612804	1.853539
H	7.599702	-1.514102	0.808350
H	7.616196	-2.588636	2.218391
H	7.805071	-0.837895	2.425870
O	-0.568958	0.176391	-0.915103
C	-1.783020	-0.306366	-0.818551
H	-1.869792	-1.347138	-0.499301
H	-0.011525	-4.969249	0.419276
C	-2.768213	0.118020	-1.839091
C	-3.910111	-0.665735	-2.061850
C	-2.539254	1.253155	-2.631575
C	-4.812142	-0.316096	-3.066050
H	-4.079647	-1.561531	-1.470171
C	-3.445815	1.599548	-3.629654
H	-1.637471	1.835140	-2.473018
C	-4.584441	0.817446	-3.848238
H	-5.685372	-0.937606	-3.243523
H	-3.259519	2.473419	-4.248002
H	-5.284991	1.085790	-4.634551
C	1.919317	0.197462	-1.143221
C	3.101982	-0.370733	-1.642628
C	1.898425	1.587843	-0.975306
C	4.219962	0.410489	-1.940632
H	3.153528	-1.441285	-1.813974
C	3.013155	2.377884	-1.276895
H	0.994343	2.060176	-0.608306
C	4.182897	1.794179	-1.759802
H	5.044310	2.405343	-2.004766
C	2.974392	3.855399	-1.007460
C	5.505027	-0.226002	-2.392023
F	3.272517	4.134168	0.288115
F	3.857334	4.542280	-1.765129
F	6.134313	0.516933	-3.331452
F	1.748110	4.385779	-1.239354
F	5.321450	-1.459246	-2.903769
F	6.384621	-0.355365	-1.359850
C	-2.500453	0.272585	0.990035
H	-1.580328	0.052361	1.519177
H	-3.285140	-0.469932	1.072899
C	-2.863621	1.617111	0.897359
O	-1.945990	2.558233	1.067807
Si	-1.278406	3.314968	2.481615
C	-2.332288	4.848035	2.785043
H	-1.976728	5.375552	3.679341
H	-3.387914	4.600883	2.945504
H	-2.273879	5.550719	1.945697
C	-1.407427	2.122000	3.923232
H	-0.774930	1.244342	3.760253
H	-1.064707	2.619860	4.839294
H	-2.437901	1.790530	4.097924
C	0.473396	3.765949	2.027648
H	1.080389	2.862331	1.924816
H	0.522320	4.324524	1.087567
H	0.921256	4.392199	2.809350
C	-4.194705	2.083120	0.477758
C	-5.320484	1.235849	0.504341
C	-4.362564	3.415082	0.049293
C	-6.571226	1.710327	0.122622
H	-5.225745	0.210376	0.843112
C	-5.612746	3.882259	-0.340639
H	-3.498118	4.066837	0.010816
C	-6.721290	3.032423	-0.302446
H	-7.431235	1.047949	0.157133
H	-5.723982	4.908861	-0.677494
H	-7.699244	3.399337	-0.601574

TS 23

B3LYP/6-31G(d) = -3573.928336
B3LYP-D3(BJ)/def2-TZVPP/IEFPCM(propanonitrile) = -3575.510686
B3LYP-D3(BJ)/def2-TZVPP/IEFPCM(propanonitrile)//B3LYP-D3(BJ)/6-31G(d) Free Energy (Quasiharmonic) = -3574.793229

Frequencies (Top 3 out of 300)

1. -290.5582 cm^{-1}
2. 14.4845 cm^{-1}
3. 14.9164 cm^{-1}

B3LYP/6-31G(d) Molecular Geometry in Cartesian Coordinates

B	-0.736440	-0.351100	0.829600
O	-0.636870	-0.746250	2.245440
N	-1.742890	-1.415939	0.294050
C	-1.968821	-2.461249	1.300110
S	-2.651540	-1.359839	-1.065690
C	-1.281961	-1.879559	2.541110
O	-1.293911	-2.387619	3.640120
H	-1.930372	-4.239109	0.081680
H	-3.039481	-2.563159	1.526420
C	-1.419252	-3.878769	0.978530
C	0.072568	-3.981930	0.804320
C	0.816348	-4.080090	-0.431210
C	2.193788	-4.174371	-0.087000
N	2.273828	-4.149511	1.291310
C	1.002358	-4.019101	1.816940
H	0.849928	-3.967380	2.886220
C	2.819198	-4.276461	-2.384950
C	0.458218	-4.091260	-1.791910
H	4.251808	-4.321572	-0.761730
H	3.125948	-4.049142	1.820840
C	1.459718	-4.189351	-2.750520
H	3.578478	-4.344572	-3.159660
H	-0.581762	-3.992670	-2.086560
C	3.204728	-4.271092	-1.049220
H	1.192728	-4.189211	-3.804020
C	-4.358600	-1.187338	-0.503600
O	-2.615721	-2.637019	-1.796180
O	-2.258550	-0.125069	-1.775070
C	-7.035780	-0.881817	0.274000
C	-4.686210	-0.284658	0.509760
C	-5.352041	-1.939147	-1.132450
C	-6.680971	-1.777877	-0.742810
C	-6.018030	-0.139827	0.890890
H	-3.905770	0.290792	0.995700
H	-5.077441	-2.642378	-1.910880
H	-7.454541	-2.362696	-1.234900
H	-6.272549	0.561413	1.682310
C	-8.472650	-0.741186	0.717620
H	-9.167930	-0.983196	-0.092750
H	-8.696010	-1.419016	1.552290
H	-8.689460	0.276364	1.059860
H	-1.742882	-4.512909	1.813000
C	0.719020	-0.355910	0.111810
C	0.908520	-0.271671	-1.278150
C	1.862340	-0.584541	0.896120
C	2.179870	-0.389831	-1.849890
C	3.128040	-0.731802	0.325070
H	1.749220	-0.691401	1.968440
C	3.298500	-0.625782	-1.054780
H	4.274200	-0.767052	-1.505020
C	4.327660	-1.009262	1.183880
C	2.354750	-0.234421	-3.332210
F	5.214529	-1.828233	0.568750
F	5.014980	0.123327	1.498310
F	4.001110	-1.596412	2.360910
F	1.268450	-0.607371	-4.030360
F	2.589781	1.079359	-3.662330
F	3.410870	-0.928102	-3.805410
O	-1.422639	1.035921	0.879660
C	-1.290129	1.983671	-0.002070
C	-2.483148	2.828151	-0.277150
C	-3.414048	3.128522	0.727790
C	-2.685518	3.320331	-1.573460
C	-4.525198	3.916772	0.440040
H	-3.260568	2.739222	1.730200
C	-3.803198	4.103052	-1.860940
H	-1.986008	3.058631	-2.362430
C	-4.721437	4.407452	-0.854510
H	-5.241258	4.148133	1.223830
H	-3.961377	4.466052	-2.872470
H	-5.590887	5.019453	-1.078000
H	-0.634409	1.794900	-0.850350
C	-0.128378	3.524550	0.788140
H	-0.772718	3.732170	1.632580
H	-0.249738	4.207870	-0.043750
C	1.171602	3.088309	1.068750
C	1.556292	2.470169	2.341470
C	0.591081	2.100190	3.299340
C	2.917781	2.235139	2.620190
C	0.980101	1.515119	4.499600
H	-0.464789	2.220690	3.091310
C	3.301011	1.663068	3.826890
H	3.663022	2.483758	1.875330
C	2.333621	1.304069	4.770700
H	0.224391	1.205180	5.214250
H	4.352401	1.476298	4.021840
H	2.633641	0.846609	5.709370
O	2.143042	3.170479	0.172280
Si	2.486632	4.206769	-1.175880
C	2.516663	5.960849	-0.495040
H	3.268893	6.067248	0.294690
H	2.767684	6.672409	-1.291210
H	1.548303	6.260749	-0.079010
C	1.223312	4.005129	-2.551580
H	1.181792	2.966629	-2.894940
H	1.540793	4.613149	-3.409060
H	0.214232	4.331420	-2.280200
H	0.048600	-0.163200	-1.932120
C	4.170212	3.608158	-1.729650
H	4.582682	4.274718	-2.496950
H	4.882932	3.570988	-0.898780
H	4.093782	2.607088	-2.166100

TS 24

B3LYP/6-31G(d) = -3573.929704
B3LYP-D3(BJ)/def2-TZVPP/IEFPCM(propanonitrile) = -3575.510458
B3LYP-D3(BJ)/def2-TZVPP/IEFPCM(propanonitrile)//B3LYP-D3(BJ)/6-31G(d) Free Energy (Quasiharmonic) = -3574.793194

Frequencies (Top 3 out of 300)

1. -288.6168 cm^{-1}
2. 13.4164 cm^{-1}
3. 15.2414 cm^{-1}

B3LYP/6-31G(d) Molecular Geometry in Cartesian Coordinates

B	0.694281	-0.347940	-0.642664
O	0.473547	-0.755274	-2.043839
N	1.692314	-1.451962	-0.171152
C	1.796113	-2.518811	-1.174626
S	2.640898	-1.453878	1.161401
C	1.030843	-1.930836	-2.364737
O	0.938504	-2.451604	-3.452229
H	1.975729	-4.346354	-0.054374
H	2.837676	-2.650349	-1.500942
C	1.260723	-3.922237	-0.764929
C	-0.115776	-3.951184	-0.166134
C	-1.386623	-4.061634	-0.845984
C	-2.400391	-4.025066	0.153732
N	-1.766624	-3.919514	1.376441
C	-0.402317	-3.853549	1.173574
H	0.279589	-3.734486	2.003390
C	-4.110456	-4.202147	-1.499659
C	-1.771849	-4.180283	-2.193950
H	-4.521348	-4.055256	0.619342
H	-2.217904	-3.693721	2.250287
C	-3.124545	-4.248151	-2.506136

H	-5.160512	-4.250347	-1.775567
H	-1.022819	-4.189764	-2.980509
C	-3.761953	-4.093003	-0.157635
H	-3.430455	-4.331629	-3.545729
C	4.335366	-1.423248	0.544827
O	2.527785	-2.708978	1.923826
O	2.363298	-0.183291	1.864084
C	6.999227	-1.337561	-0.325542
C	5.284159	-2.255365	1.139953
C	4.699241	-0.552105	-0.484031
C	6.023661	-0.517907	-0.912496
C	6.606998	-2.205474	0.701598
H	4.978962	-2.937734	1.925492
H	3.950933	0.079946	-0.949708
H	6.304866	0.154402	-1.719712
H	7.344946	-2.858305	1.161653
C	8.438044	-1.271362	-0.779915
H	8.974317	-0.457752	-0.273369
H	8.510601	-1.085740	-1.856940
H	8.971815	-2.201031	-0.558507
O	1.432171	0.999700	-0.764729
C	1.477011	1.938305	0.135347
H	0.927826	1.777825	1.062269
H	1.304962	-4.535528	-1.673318
C	2.755091	2.690888	0.256414
C	3.572973	2.917643	-0.859862
C	3.149029	3.174089	1.511223
C	4.763731	3.626684	-0.721545
H	3.268500	2.531863	-1.828522
C	4.345282	3.877031	1.648037
H	2.535134	2.966287	2.383681
C	5.151440	4.109805	0.531919
H	5.392136	3.802420	-1.590417
H	4.652377	4.233967	2.626986
H	6.082208	4.660035	0.638552
C	-0.721396	-0.274548	0.148613
C	-0.853381	-0.297349	1.547235
C	-1.908714	-0.311728	-0.601629
C	-2.110545	-0.354958	2.157701
H	0.034636	-0.300480	2.171465
C	-3.165827	-0.353708	0.003693
H	-1.844643	-0.350138	-1.682421
C	-3.275785	-0.378910	1.392546
H	-4.248249	-0.430782	1.869643
C	-4.414349	-0.333042	-0.831665
C	-2.222475	-0.507067	3.646347
F	-5.411675	-1.056868	-0.279449
F	-4.905436	0.938267	-0.970239
F	-3.321643	0.110660	4.142109
F	-4.212890	-0.795411	-2.081748
F	-1.153592	-0.017215	4.303981
F	-2.333516	-1.820574	4.003614
C	0.353737	3.567696	-0.507135
H	0.746502	4.280097	0.205801
H	0.789617	3.636424	-1.497134
C	-1.011308	3.269499	-0.422244
O	-1.686224	2.795308	-1.450523
Si	-1.574619	2.983857	-3.181333
C	-0.045047	2.150929	-3.872389
H	0.078631	1.133019	-3.487619
H	-0.141275	2.087087	-4.964308
H	0.874427	2.707616	-3.659389
C	-3.154710	2.172878	-3.770759
H	-3.248369	2.272377	-4.859155
H	-3.182543	1.105727	-3.530073
H	-4.036302	2.634510	-3.312936
C	-1.577797	4.841365	-3.491684
H	-0.708562	5.339224	-3.047647
H	-1.554902	5.041054	-4.570161
H	-2.480399	5.312518	-3.086474
C	-1.783077	3.390301	0.826063
C	-1.172024	3.765442	2.040425
C	-3.166639	3.132725	0.817048
C	-1.921958	3.872116	3.205733
H	-0.106648	3.964680	2.083655
C	-3.914183	3.250500	1.985163
H	-3.641500	2.816713	-0.102135
C	-3.296288	3.615518	3.182218
H	-1.435014	4.151125	4.135392
H	-4.979833	3.042560	1.961477
H	-3.879466	3.696315	4.095050

TS 25

B3LYP/6-31G(d) = -3573.935258
B3LYP-D3(BJ)/def2-TZVPP/IEFPCM(propanonitrile) = -3575.510382
B3LYP-D3(BJ)/def2-TZVPP/IEFPCM(propanonitrile)//B3LYP-D3(BJ)/6-31G(d) Free Energy (Quasiharmonic) = -3574.793188

Frequencies (Top 3 out of 300)

1. -273.6944 cm^{-1}
2. 7.6042 cm^{-1}
3. 12.2268 cm^{-1}

B3LYP/6-31G(d) Molecular Geometry in Cartesian Coordinates

B	1.022765	-0.401961	-1.158512
O	1.267056	-1.130807	-2.424740
N	0.913062	-1.593451	-0.141975
C	1.223890	-2.866878	-0.821527
S	1.172576	-1.499978	1.489235
C	1.374138	-2.450120	-2.288930
O	1.572881	-3.224065	-3.199949
H	0.136343	-4.272203	0.395290
H	2.209001	-3.237271	-0.497193
C	0.231284	-4.044831	-0.669035
C	-1.135358	-3.891415	-1.283323
C	-2.391411	-3.694727	-0.597461
C	-3.423137	-3.743032	-1.579332
N	-2.811457	-3.943600	-2.797814
C	-1.445623	-4.034246	-2.615992
H	-0.783176	-4.197417	-3.454082
C	-5.100515	-3.431187	0.087611
C	-2.750415	-3.495075	0.748782
H	-5.549131	-3.672806	-2.016312
H	-3.280956	-3.993786	-3.688278
C	-4.095948	-3.363714	1.076231
H	-6.144043	-3.337773	0.377418
H	-1.983513	-3.434101	1.515888
C	-4.777398	-3.619985	-1.252174
H	-4.380874	-3.206981	2.112618
C	2.920666	-1.749465	1.817039
O	0.464885	-2.613034	2.145494
O	0.846326	-0.115075	1.884271
C	5.664668	-2.146847	2.221467
C	3.781319	-0.648261	1.850272
C	3.410397	-3.043308	2.009335
C	4.778692	-3.231105	2.207452
C	5.142366	-0.855573	2.050108
H	3.384491	0.352464	1.727957
H	2.727266	-3.885699	2.027234
H	5.161533	-4.237995	2.353934
H	5.812961	-0.001092	2.056016
C	7.147365	-2.347664	2.422971
H	7.720738	-1.850999	1.631543
H	7.479516	-1.921155	3.377947
H	7.413851	-3.408888	2.420741
H	0.737706	-4.898019	-1.135983
C	2.172274	0.720489	-0.949099
C	1.900715	1.989647	-0.424697

C	3.497513	0.468191	-1.338319
C	2.893124	2.963646	-0.286735
C	4.501322	1.429491	-1.191377
H	3.753451	-0.488840	-1.781696
C	4.205846	2.688410	-0.665325
H	4.978404	3.442980	-0.572163
C	5.926048	1.097901	-1.540070
C	2.539005	4.294057	0.316414
F	6.610438	0.636785	-0.457184
F	6.604588	2.180307	-1.984819
F	6.015355	0.143585	-2.488460
F	1.298889	4.704152	-0.058886
F	3.405295	5.267111	-0.040754
F	2.531905	4.251509	1.670121
O	-0.292679	0.338570	-1.346000
C	-1.471870	0.089497	-0.841382
C	-2.628595	0.390137	-1.722697
C	-2.448676	1.128743	-2.903552
C	-3.900507	-0.110728	-1.408288
C	-3.529763	1.372129	-3.748348
H	-1.454072	1.479192	-3.158001
C	-4.977428	0.128684	-2.260780
H	-4.043275	-0.707702	-0.511926
C	-4.797291	0.874889	-3.428270
H	-3.381406	1.936183	-4.665232
H	-5.954182	-0.279191	-2.016503
H	-5.637172	1.056607	-4.093887
H	-1.564712	-0.787565	-0.197420
C	-1.593497	1.460875	0.651100
H	-1.345580	2.306090	0.021226
H	-0.767038	1.035093	1.207839
C	-2.855038	1.493119	1.253802
C	-3.280327	0.586993	2.329570
C	-4.631435	0.562901	2.732452
C	-2.356490	-0.248938	2.990049
C	-5.044753	-0.269776	3.766026
H	-5.346657	1.197564	2.222593
C	-2.778126	-1.078210	4.024924
H	-1.308129	-0.249651	2.715060
C	-4.118888	-1.090931	4.417469
H	-6.088758	-0.279774	4.066274
H	-2.052427	-1.715843	4.520479
H	-4.442371	-1.737356	5.228961
O	-3.807436	2.292997	0.793553
Si	-3.910598	3.941195	0.274518
C	-3.860283	4.958467	1.854173
H	-3.978481	6.025446	1.628273
H	-4.660645	4.670974	2.544848
H	-2.904291	4.837641	2.376899
C	-5.582622	3.981171	-0.570003
H	-5.811141	4.989810	-0.934756
H	-5.599731	3.299605	-1.427665
H	-6.384320	3.683052	0.114732
H	0.889778	2.236957	-0.128110
C	-2.537388	4.452715	-0.901908
H	-2.462632	3.775784	-1.759431
H	-1.553132	4.520687	-0.425746
H	-2.774676	5.451508	-1.292252

TS 26

B3LYP/6-31G(d) = -3573.929408
B3LYP-D3(BJ)/def2-TZVPP/IEFPCM(propanonitrile) = -3575.51009
B3LYP-D3(BJ)/def2-TZVPP/IEFPCM(propanonitrile)//B3LYP-D3(BJ)/6-31G(d) Free Energy (Quasiharmonic) = -3574.793153

Frequencies (Top 3 out of 300)

1. -298.8058 cm^{-1}
2. 8.6655 cm^{-1}
3. 15.0213 cm^{-1}

B3LYP/6-31G(d) Molecular Geometry in Cartesian Coordinates

B	-0.372856	0.891697	0.724895
O	-0.364966	0.950859	2.203232
N	-1.079980	2.224350	0.365078
C	-1.596441	2.874526	1.574745
S	-0.997998	3.052094	-1.052602
C	-1.016168	2.008143	2.699975
O	-1.152565	2.229871	3.882294
H	-3.506419	3.608847	0.895252
H	-1.182898	3.886957	1.689372
C	-3.141543	2.969932	1.703798
C	-3.865831	1.651320	1.694840
C	-4.534685	1.015141	0.582492
C	-5.058495	-0.219096	1.058375
N	-4.739035	-0.311885	2.398075
C	-4.008933	0.802129	2.766930
H	-3.643760	0.913248	3.778813
C	-5.933017	-0.743497	-1.096617
C	-4.735056	1.358407	-0.767290
H	-6.133240	-2.052116	0.617900
H	-4.832302	-1.149928	2.950578
C	-5.428285	0.477871	-1.589649
H	-6.463396	-1.413431	-1.768221
H	-4.327010	2.282253	-1.165347
C	-5.756195	-1.107814	0.233484
H	-5.577114	0.729361	-2.636319
C	0.160522	4.413162	-0.815234
O	-2.293462	3.695930	-1.321544
O	-0.400431	2.136103	-2.039445
C	1.964762	6.528229	-0.434996
C	-0.305622	5.649978	-0.362950
C	1.515075	4.226094	-1.093374
C	2.404290	5.281161	-0.902138
C	0.599131	6.693211	-0.168901
H	-1.366557	5.797192	-0.192065
H	1.859558	3.270318	-1.469235
H	3.458498	5.133743	-1.126039
H	0.234679	7.654469	0.185761
C	2.931720	7.675504	-0.260565
H	2.607041	8.356718	0.532868
H	3.014232	8.266015	-1.182836
H	3.937168	7.319140	-0.012551
H	-3.328059	3.495886	2.648009
C	-1.128657	-0.460808	0.223455
C	-1.478704	-1.441418	1.168977
C	-1.540382	-0.679801	-1.103905
C	-2.199892	-2.585117	0.812576
H	-1.202695	-1.291039	2.207445
C	-2.258731	-1.825252	-1.463079
H	-1.327208	0.071749	-1.857899
C	-2.593343	-2.786180	-0.509596
H	-3.166330	-3.663045	-0.788482
C	-2.649188	-2.053742	-2.897129
C	-2.634326	-3.566106	1.863598
F	-3.788607	-2.771084	-3.007933
F	-1.687428	-2.766561	-3.562626
F	-2.813998	-0.908715	-3.579409
F	-2.682151	-4.831718	1.389053
F	-3.885734	-3.293329	2.334675
F	-1.814732	-3.567073	2.937052
O	1.103958	0.976981	0.331200
C	1.781864	0.121797	-0.387535
C	3.114399	0.592119	-0.855792
C	3.784391	1.626863	-0.186001
C	3.695056	0.023881	-1.999007
C	5.019326	2.077802	-0.650752
H	3.317261	2.082189	0.680771
C	4.929062	0.478580	-2.462511

H	3.171182	-0.761815	-2.536552
C	5.595672	1.503484	-1.787724
H	5.528934	2.884436	-0.130314
H	5.363649	0.040575	-3.356575
H	6.554799	1.861228	-2.152137
H	1.220735	-0.478850	-1.108407
C	2.110262	-1.386012	0.866569
H	2.441974	-0.816621	1.728294
H	1.070421	-1.686935	0.911679
C	3.021229	-2.297221	0.310120
C	2.674542	-3.234351	-0.764207
C	1.359410	-3.323777	-1.267678
C	3.674081	-4.054679	-1.327786
C	1.058857	-4.197712	-2.307627
H	0.563691	-2.713797	-0.856147
C	3.365495	-4.934558	-2.358487
H	4.687895	-3.985645	-0.951808
C	2.059392	-5.006196	-2.852789
H	0.047092	-4.230638	-2.696266
H	4.143937	-5.562100	-2.782798
H	1.822112	-5.689206	-3.663655
O	4.304701	-2.271052	0.629856
Si	5.159962	-2.033627	2.125837
C	6.896480	-2.555409	1.652077
H	6.925149	-3.596345	1.311484
H	7.290898	-1.925468	0.846805
H	7.576033	-2.465602	2.508065
C	4.365759	-3.183859	3.380101
H	4.400454	-4.227409	3.047752
H	4.896054	-3.121682	4.338311
H	3.318218	-2.923748	3.568470
C	5.119898	-0.241849	2.680356
H	5.835512	-0.107687	3.501990
H	5.410099	0.434516	1.870160
H	4.138479	0.073308	3.050304

TS 27

B3LYP/6-31G(d) = -3573.929722
B3LYP-D3(BJ)/def2-TZVPP/IEFPCM(propanonitrile) = -3575.510473
B3LYP-D3(BJ)/def2-TZVPP/IEFPCM(propanonitrile)//B3LYP-D3(BJ)/6-31G(d) Free Energy (Quasiharmonic) = -3574.793129

Frequencies (Top 3 out of 300)

1. -288.5951 cm^{-1}
2. 13.4584 cm^{-1}
3. 15.2178 cm^{-1}

B3LYP/6-31G(d) Molecular Geometry in Cartesian Coordinates

B	0.695250	-0.346830	-0.641820
O	0.475980	-0.754950	-2.043020
N	1.694410	-1.449430	-0.169260
C	1.800080	-2.516550	-1.172260
S	2.641880	-1.449850	1.164090
C	1.034960	-1.929910	-2.363120
O	0.944100	-2.451170	-3.450510
H	1.981069	-4.343430	-0.051180
H	2.842000	-2.647000	-1.497830
C	1.266050	-3.920440	-0.762380
C	-0.110810	-3.950790	-0.164500
C	-1.381080	-4.062950	-0.845170
C	-2.395550	-4.027230	0.153860
N	-1.762730	-3.920540	1.376960
C	-0.398370	-3.853040	1.174980
H	0.282850	-3.732900	2.005210
C	-4.104311	-4.206860	-1.500620
C	-1.765261	-4.182500	-2.193350
H	-4.516780	-4.059730	0.618060
H	-2.214850	-3.694950	2.250420
C	-3.117671	-4.252060	-2.506420
H	-5.154121	-4.256380	-1.777210
H	-1.015701	-4.191360	-2.979410
C	-3.756830	-4.096860	-0.158400
H	-3.422791	-4.336230	-3.546190
C	4.336490	-1.417930	0.548100
O	2.529920	-2.704980	1.926660
O	2.361850	-0.179580	1.866390
C	7.001940	-1.324730	-0.316350
C	4.699940	-0.546570	-0.480570
C	5.287610	-2.243000	1.149620
C	6.611630	-2.187550	0.715990
C	6.025800	-0.506820	-0.904420
H	3.951790	0.086830	-0.944680
H	4.984530	-2.919230	1.941280
H	7.352840	-2.829580	1.185960
H	6.308330	0.171200	-1.706370
C	8.430180	-1.297140	-0.806560
H	9.128520	-1.613421	-0.025050
H	8.567040	-1.974050	-1.660370
H	8.721320	-0.294761	-1.138360
H	1.311609	-4.534030	-1.670510
C	-0.721070	-0.274930	0.148440
C	-0.854040	-0.297510	1.546970
C	-1.907810	-0.313640	-0.602650
C	-2.111580	-0.356370	2.156540
C	-3.165310	-0.356870	0.001780
H	-1.842920	-0.352260	-1.683390
C	-3.276240	-0.381830	1.390560
H	-4.248990	-0.434660	1.866970
C	-4.413250	-0.337810	-0.834480
C	-2.224390	-0.508180	3.645150
F	-4.210410	-0.800420	-2.084260
F	-5.410220	-1.062480	-0.282730
F	-4.905560	0.932940	-0.973870
F	-1.156600	-0.016800	4.303410
F	-3.324690	0.108300	4.139950
F	-2.334040	-1.821730	4.002730
O	1.431470	1.001700	-0.763930
C	1.474960	1.940530	0.135980
C	2.752120	2.694640	0.257160
C	3.569720	2.922540	-0.859100
C	3.145560	3.177990	1.512060
C	4.759710	3.632840	-0.720650
H	3.265650	2.536640	-1.827850
C	4.341100	3.882120	1.649010
H	2.531930	2.969290	2.384490
C	5.146980	4.116020	0.532930
H	5.387850	3.809530	-1.589520
H	4.647900	4.239070	2.628040
H	6.077210	4.667110	0.639710
H	0.925760	1.779560	1.062800
C	0.349850	3.568540	-0.506960
H	0.741380	4.281370	0.206230
H	0.786160	3.637870	-1.496730
C	-1.014850	3.268630	-0.422800
C	-1.787450	3.388490	0.825080
C	-1.177440	3.764040	2.039850
C	-3.170760	3.129600	0.815240
C	-1.928140	3.869820	3.204750
H	-0.112270	3.964250	2.083720
C	-3.919080	3.246520	1.982950
H	-3.644790	2.813250	-0.104260
C	-3.302210	3.611940	3.180410
H	-1.441980	4.149120	4.134730
H	-4.984530	3.037600	1.958630
H	-3.885980	3.692050	4.092920
O	-1.688630	2.793600	-1.451440
Si	-1.576110	2.982020	-3.182200
C	-3.154850	2.169120	-3.772590

H	-4.037280	2.629480	-3.315100
H	-3.248090	2.268770	-4.861010
H	-3.181410	1.101880	-3.532180
C	-0.045130	2.150750	-3.872140
H	-0.140590	2.086620	-4.964110
H	0.873570	2.708540	-3.658660
H	0.079510	1.133050	-3.487130
H	0.033520	-0.299450	2.171840
C	-1.581230	4.839480	-3.492860
H	-2.484690	5.309640	-3.088390
H	-0.712910	5.338430	-3.048250
H	-1.557770	5.039020	-4.571350

TS 28

B3LYP/6-31G(d) = -3573.933461
B3LYP-D3(BJ)/def2-TZVPP/IEFPCM(propanonitrile) = -3575.509343
B3LYP-D3(BJ)/def2-TZVPP/IEFPCM(propanonitrile)//B3LYP-D3(BJ)/6-31G(d) Free Energy (Quasiharmonic) = -3574.793108

Frequencies (Top 3 out of 300)

1. -270.1663 cm^{-1}
2. 6.6339 cm^{-1}
3. 11.3833 cm^{-1}

B3LYP/6-31G(d) Molecular Geometry in Cartesian Coordinates

B	0.225499	-0.335389	0.060610
O	-0.456141	0.354631	-1.068230
N	0.225680	-1.802119	-0.461590
C	-0.232720	-1.870159	-1.853350
S	1.083110	-3.039619	0.228600
C	-0.696451	-0.434849	-2.119130
O	-1.208701	-0.041599	-3.146690
H	-0.882470	-3.892539	-2.174760
H	0.607810	-2.059469	-2.541440
C	-1.339930	-2.901309	-2.201370
C	-2.563910	-2.897590	-1.329780
C	-3.812230	-2.200290	-1.546380
C	-4.691910	-2.575530	-0.489770
N	-4.003470	-3.456500	0.317720
C	-2.728980	-3.635900	-0.181820
H	-2.019940	-4.288460	0.307500
C	-6.434891	-1.212790	-1.384020
C	-4.283211	-1.311920	-2.531210
H	-6.658860	-2.395650	0.414740
H	-4.335340	-3.825030	1.195370
C	-5.583611	-0.830010	-2.440850
H	-7.447771	-0.820690	-1.342650
H	-3.628341	-0.994190	-3.336950
C	-6.000990	-2.089590	-0.395310
H	-5.953081	-0.141710	-3.196240
C	2.708340	-3.055639	-0.543440
O	0.442450	-4.312559	-0.142350
O	1.263770	-2.680389	1.641100
C	5.204110	-2.944118	-1.818120
C	2.880500	-3.671779	-1.785250
C	3.780420	-2.418949	0.087570
C	5.015510	-2.370688	-0.551830
C	4.124190	-3.606729	-2.414350
H	2.060210	-4.216579	-2.240780
H	3.642940	-1.973399	1.066110
H	5.847550	-1.870298	-0.062600
H	4.256280	-4.081609	-3.383510
C	6.536200	-2.825568	-2.518330
H	6.721990	-1.785438	-2.814730
H	6.574670	-3.443808	-3.420460
H	7.362180	-3.127888	-1.863960
H	-1.617100	-2.696589	-3.242340
C	1.662179	0.335071	0.374080
C	2.142599	0.534351	1.676340
C	2.496829	0.732311	-0.680090
C	3.398849	1.100161	1.913890
H	1.526389	0.240311	2.518170
C	3.751079	1.305891	-0.451600
H	2.164589	0.601361	-1.706150
C	4.213799	1.491801	0.850600
H	5.182109	1.942162	1.034070
C	4.622349	1.668112	-1.621040
C	3.904869	1.220401	3.325130
F	5.279709	0.589732	-2.116780
F	5.563039	2.582902	-1.297190
F	3.897809	2.180301	-2.645770
F	4.841619	2.189082	3.450160
F	4.475609	0.066902	3.747790
F	2.911559	1.506261	4.196950
O	-0.603301	-0.218209	1.318960
C	-1.901211	-0.143919	1.370250
C	-2.555411	-0.805910	2.530830
C	-3.955871	-0.877560	2.608580
C	-1.774661	-1.413579	3.524930
C	-4.566310	-1.530240	3.676270
H	-4.564701	-0.435750	1.823470
C	-2.391370	-2.068660	4.590550
H	-0.694761	-1.406379	3.428530
C	-3.784210	-2.122400	4.673940
H	-5.650620	-1.579280	3.731600
H	-1.781120	-2.546349	5.351780
H	-4.260920	-2.631040	5.507660
H	-2.450671	-0.174930	0.427910
C	-2.347841	1.822110	1.829070
H	-3.386011	1.692260	2.106760
H	-1.631941	1.900721	2.640700
C	-2.095061	2.556850	0.669670
C	-3.153261	2.805610	-0.333960
C	-4.412731	3.274780	0.081220
C	-2.920761	2.564180	-1.699900
C	-5.410241	3.530480	-0.855960
H	-4.593421	3.473160	1.133760
C	-3.933311	2.799770	-2.627760
H	-1.983711	2.125910	-2.024000
C	-5.171551	3.294190	-2.212190
H	-6.372601	3.913650	-0.528800
H	-3.753501	2.581690	-3.676200
H	-5.953611	3.485870	-2.941760
O	-0.861311	2.947411	0.436450
Si	-0.090471	4.312011	-0.318150
C	-1.333672	5.722731	-0.407060
H	-1.801602	5.916151	0.565150
H	-0.811522	6.640381	-0.706810
H	-2.128322	5.538810	-1.136380
C	1.296388	4.696771	0.882890
H	1.918458	5.518231	0.506540
H	0.907798	4.992991	1.863780
H	1.943559	3.824991	1.026890
C	0.571279	3.856521	-2.010820
H	1.371759	3.113621	-1.950840
H	-0.202731	3.470571	-2.680270
H	0.992968	4.757851	-2.475450

TS 29

B3LYP/6-31G(d) = -3573.931483
B3LYP-D3(BJ)/def2-TZVPP/IEFPCM(propanonitrile) = -3575.508708
B3LYP-D3(BJ)/def2-TZVPP/IEFPCM(propanonitrile)//B3LYP-D3(BJ)/6-31G(d) Free Energy (Quasiharmonic) = -3574.792886

Frequencies (Top 3 out of 300)

1. -283.2225 cm^{-1}
2. 9.0746 cm^{-1}
3. 9.7394 cm^{-1}

B3LYP/6-31G(d) Molecular Geometry in Cartesian Coordinates

B	0.616070	0.442610	0.223140
O	0.245630	0.232110	1.655200
N	1.222430	1.865190	0.277030
C	1.194390	2.401940	1.641810
S	2.225809	2.524590	-0.864970
C	0.514360	1.284710	2.435860
O	0.234650	1.332380	3.615710
H	0.989849	4.513700	1.294600
H	2.214139	2.510120	2.044620
C	0.464759	3.751460	1.874250
C	-1.006551	3.788010	1.561910
C	-1.644711	4.268570	0.355990
C	-3.049481	4.186360	0.559970
N	-3.249341	3.650920	1.818550
C	-2.020981	3.437890	2.421110
H	-1.957791	3.051730	3.429130
C	-3.470581	5.112580	-1.594640
C	-1.165611	4.780330	-0.864020
H	-5.044651	4.534650	-0.226900
H	-4.140661	3.577060	2.282990
C	-2.081441	5.197350	-1.821730
H	-4.160551	5.449180	-2.364200
H	-0.098061	4.835860	-1.053120
C	-3.973961	4.604200	-0.403080
H	-1.721561	5.594310	-2.767220
C	3.908370	2.059430	-0.422040
O	2.168679	3.989530	-0.731650
O	1.901770	1.869020	-2.136270
C	6.471170	1.245851	0.377230
C	4.609649	2.808970	0.525320
C	4.490270	0.929850	-1.004000
C	5.761590	0.533111	-0.600980
C	5.882140	2.393881	0.920610
H	4.175299	3.718330	0.926980
H	3.950990	0.378070	-1.765250
H	6.206690	-0.354159	-1.043710
H	6.425459	2.974881	1.661960
C	7.832920	0.772211	0.825870
H	8.254600	1.427431	1.594230
H	8.540380	0.737121	-0.011770
H	7.773450	-0.241689	1.240370
H	0.616869	3.982720	2.935360
C	1.555620	-0.771310	-0.294870
C	2.471650	-1.386960	0.572540
C	1.501220	-1.251270	-1.611660
C	3.300070	-2.428350	0.146290
H	2.543180	-1.050720	1.602360
C	2.319300	-2.300290	-2.042230
H	0.816120	-0.789880	-2.314040
C	3.226120	-2.895980	-1.166250
H	3.854760	-3.714800	-1.496450
C	2.257160	-2.745240	-3.477590
C	4.330420	-3.005640	1.076720
F	2.731270	-4.001820	-3.643450
F	2.984800	-1.940400	-4.284960
F	0.988640	-2.730070	-3.952750
F	5.523340	-2.365909	0.963810
F	4.570610	-4.312450	0.819680
F	3.960130	-2.910540	2.372650
O	-0.643210	0.448410	-0.619640
C	-1.844540	0.169310	-0.165900
C	-2.963850	0.536500	-1.076060
C	-4.269800	0.633570	-0.574800
C	-2.721190	0.825600	-2.426360
C	-5.320150	1.004829	-1.411050
H	-4.459300	0.428350	0.476430
C	-3.774750	1.193300	-3.261550
H	-1.702820	0.794430	-2.798610
C	-5.074840	1.280230	-2.758340
H	-6.327940	1.083289	-1.012350
H	-3.577400	1.427650	-4.303840
H	-5.892890	1.573379	-3.410880
H	-2.022850	0.373780	0.892920
C	-1.813880	-1.765710	0.005550
H	-0.950310	-1.794530	0.660010
H	-1.589090	-2.033700	-1.020140
C	-3.005560	-2.274250	0.556540
C	-4.152360	-2.723670	-0.243430
C	-4.127550	-2.696160	-1.652780
C	-5.306250	-3.211931	0.403530
C	-5.221990	-3.141250	-2.386160
H	-3.263450	-2.312660	-2.181420
C	-6.395040	-3.660831	-0.334540
H	-5.333490	-3.235261	1.486400
C	-6.356460	-3.626781	-1.731470
H	-5.189320	-3.108410	-3.470890
H	-7.275600	-4.037821	0.177560
H	-7.207720	-3.978311	-2.307860
O	-3.208400	-2.277370	1.858000
Si	-2.329450	-2.467900	3.358040
C	-2.452060	-0.834580	4.260980
H	-1.689700	-0.119350	3.931930
H	-3.444890	-0.385150	4.142010
H	-2.296180	-1.002110	5.334580
C	-0.567520	-3.024470	3.044880
H	-0.136950	-3.359800	3.997450
H	-0.507240	-3.867250	2.346970
H	0.059580	-2.209090	2.672540
C	-3.331640	-3.814040	4.205520
H	-4.374510	-3.508320	4.348470
H	-3.327800	-4.745790	3.628370
H	-2.913880	-4.035810	5.195280

TS 30

B3LYP/6-31G(d) = -3573.927078
B3LYP-D3(BJ)/def2-TZVPP/IEFPCM(propanonitrile) = -3575.509238
B3LYP-D3(BJ)/def2-TZVPP/IEFPCM(propanonitrile)//B3LYP-D3(BJ)/6-31G(d) Free Energy (Quasiharmonic) = -3574.792797

Frequencies (Top 3 out of 300)

1. -293.1496 cm^{-1}
2. 10.6690 cm^{-1}
3. 12.9510 cm^{-1}

B3LYP/6-31G(d) Molecular Geometry in Cartesian Coordinates

B	0.396200	0.966959	-0.774964
O	0.759218	1.206642	-2.184780
N	0.726654	2.352041	-0.140064
C	1.443500	3.198986	-1.100105
S	0.225260	2.946651	1.298054
C	1.337573	2.391931	-2.400050
O	1.732245	2.775428	-3.478172
H	2.896333	4.300259	0.044590
H	0.910304	4.145849	-1.266392
C	2.919779	3.552483	-0.752949
C	3.785295	2.402368	-0.325351
C	4.509415	1.460884	-1.150423
C	5.160016	0.549214	-0.270785
N	4.862895	0.939768	1.020300
C	4.018407	2.032396	0.976527
H	3.630116	2.474110	1.883414
C	6.067024	-0.665751	-2.112144

C	4.668982	1.288535	-2.537962
H	6.425242	-1.200952	-0.045875
H	5.024875	0.380554	1.844266
C	5.440086	0.229538	-3.002540
H	6.660827	-1.486072	-2.506779
H	4.172869	1.956613	-3.235693
C	5.939345	-0.514934	-0.735506
H	5.558965	0.084083	-4.072990
C	-0.916197	4.287193	0.906163
O	1.329836	3.579615	2.039574
O	-0.530040	1.858463	1.952676
C	-2.735116	6.356468	0.387682
C	-0.763298	5.517582	1.544899
C	-1.967565	4.075785	0.011068
C	-2.866459	5.108264	-0.240257
C	-1.674013	6.541283	1.282392
H	0.061085	5.662115	2.234315
H	-2.075190	3.116670	-0.483504
H	-3.686327	4.942141	-0.935313
H	-1.555029	7.500334	1.780943
C	-3.699596	7.477879	0.082031
H	-4.722966	7.105223	-0.037142
H	-3.429819	7.986829	-0.852877
H	-3.702597	8.231765	0.875622
O	-1.114612	0.741937	-0.812600
C	-1.832860	-0.010773	-0.007438
H	-1.375348	-0.285807	0.943789
H	3.327742	4.042037	-1.645997
C	-3.271459	0.368805	0.087267
C	-3.923077	1.000112	-0.981473
C	-3.978666	0.111603	1.268985
C	-5.263518	1.365462	-0.867531
H	-3.366321	1.212722	-1.888923
C	-5.318874	0.480207	1.381234
H	-3.469184	-0.349947	2.110863
C	-5.964684	1.105379	0.312549
H	-5.761305	1.856573	-1.699376
H	-5.854202	0.293051	2.308004
H	-7.007703	1.396572	0.402101
C	1.232213	-0.303012	-0.198978
C	1.544683	-0.486289	1.158859
C	1.742748	-1.249397	-1.103247
C	2.322587	-1.566739	1.587177
H	1.180156	0.224784	1.892520
C	2.508510	-2.338920	-0.676064
H	1.562421	-1.115200	-2.165132
C	2.805749	-2.505782	0.674557
H	3.408085	-3.343669	1.007242
C	2.950165	-3.374378	-1.671461
C	2.701079	-1.703800	3.033442
F	3.257818	-2.843218	-2.868090
F	4.020843	-4.077748	-1.242865
F	2.689872	-3.000050	3.437381
F	1.955812	-4.284741	-1.895345
F	1.876653	-1.024576	3.854124
F	3.963116	-1.246403	3.276725
C	-1.746388	-1.788983	-0.816982
H	-1.694589	-1.461604	-1.848019
H	-0.781195	-2.079152	-0.414655
C	-2.847295	-2.597006	-0.468671
O	-2.877538	-3.286173	0.653580
Si	-1.722148	-4.084082	1.692902
C	-2.826406	-5.277245	2.627765
H	-2.242264	-5.845313	3.361980
H	-3.304208	-5.996984	1.953880
H	-3.615266	-4.746877	3.173142
C	-0.499331	-4.996815	0.602187
H	0.161918	-5.600796	1.236673
H	-1.022100	-5.686631	-0.070873
H	0.140903	-4.353558	-0.008234
C	-0.958189	-2.820040	2.843047
H	-1.709705	-2.383605	3.510865
H	-0.194181	-3.296788	3.469711
H	-0.460052	-2.004810	2.311261
C	-4.093290	-2.652266	-1.244428
C	-4.173635	-2.138573	-2.554750
C	-5.237373	-3.255595	-0.683074
C	-5.359077	-2.228272	-3.276717
H	-3.307422	-1.687223	-3.024225
C	-6.421858	-3.334517	-1.405410
H	-5.183942	-3.649750	0.324555
C	-6.486078	-2.822765	-2.704606
H	-5.402108	-1.837070	-4.288726
H	-7.297141	-3.795739	-0.957424
H	-7.410628	-2.891162	-3.271087

TS 31

B3LYP/6-31G(d) = -3573.934951
B3LYP-D3(BJ)/def2-TZVPP/IEFPCM(propanonitrile) = -3575.509805
B3LYP-D3(BJ)/def2-TZVPP/IEFPCM(propanonitrile)//B3LYP-D3(BJ)/6-31G(d) Free Energy (Quasiharmonic) = -3574.792617

Frequencies (Top 3 out of 300)

1. -272.3338 cm^{-1}
2. 8.2946 cm^{-1}
3. 14.0401 cm^{-1}

B3LYP/6-31G(d) Molecular Geometry in Cartesian Coordinates

B	0.990391	-0.473680	-1.164004
O	1.232979	-1.248592	-2.402229
N	0.868268	-1.626060	-0.105096
C	1.161930	-2.926702	-0.738641
S	1.131199	-1.474090	1.520674
C	1.317755	-2.564313	-2.219983
O	1.502785	-3.372614	-3.103491
H	0.063831	-4.279832	0.526296
H	2.142057	-3.298986	-0.401562
C	0.152061	-4.083213	-0.544679
C	-1.217844	-3.920969	-1.149257
C	-2.463764	-3.693051	-0.454409
C	-3.505833	-3.742058	-1.424918
N	-2.909819	-3.973524	-2.645751
C	-1.543667	-4.081734	-2.476190
H	-0.892709	-4.270996	-3.317839
C	-5.161283	-3.375425	0.252450
C	-2.806123	-3.465119	0.891655
H	-5.634515	-3.644662	-1.842527
H	-3.388830	-4.026788	-3.530933
C	-4.145973	-3.307196	1.229817
H	-6.200225	-3.260295	0.550810
H	-2.030715	-3.402888	1.649984
C	-4.854626	-3.592024	-1.086768
H	-4.418248	-3.128200	2.266036
C	2.879548	-1.713199	1.854210
O	0.423182	-2.560955	2.218753
O	0.806023	-0.074832	1.863660
C	5.625160	-2.101313	2.257086
C	3.740860	-0.612006	1.854102
C	3.369335	-3.001535	2.080829
C	4.738407	-3.184791	2.277869
C	5.102568	-0.814854	2.053646
H	3.344373	0.385481	1.706763
H	2.685937	-3.842714	2.125667
H	5.121234	-4.187492	2.450672
H	5.773811	0.039003	2.033654
C	7.109192	-2.297506	2.452964
H	7.452766	-1.827553	3.383096
H	7.675196	-1.840358	1.632804

H	7.372713	-3.358523	2.497409
H	0.639589	-4.957222	-0.992974
C	2.148401	0.645386	-0.985223
C	1.894768	1.916605	-0.456067
C	3.466173	0.382331	-1.391173
C	2.897394	2.880299	-0.325309
H	0.891311	2.172715	-0.144367
C	4.480415	1.334779	-1.254335
H	3.708032	-0.576775	-1.837900
C	4.203379	2.594270	-0.720540
H	4.984545	3.340748	-0.632714
C	5.898156	0.989096	-1.617962
C	2.568346	4.207872	0.297902
F	6.586916	2.067559	-2.056600
F	5.967636	0.044018	-2.577154
F	6.584341	0.507651	-0.545341
F	3.401004	5.188607	-0.114011
F	2.647267	4.167499	1.648940
F	1.302608	4.606131	0.000008
O	-0.321705	0.264739	-1.384318
C	-1.499595	0.057203	-0.858034
C	-2.657656	0.290725	-1.759587
C	-3.948076	-0.099685	-1.369820
C	-2.457871	0.836247	-3.037123
C	-5.022685	0.065268	-2.241125
H	-4.107101	-0.556744	-0.397222
C	-3.535624	0.998837	-3.905563
H	-1.452138	1.102062	-3.343588
C	-4.820868	0.619225	-3.508528
H	-6.015003	-0.253068	-1.934499
H	-3.371140	1.412853	-4.896713
H	-5.659812	0.742489	-4.188599
H	-1.592547	-0.771629	-0.152833
C	-1.595781	1.519721	0.545934
H	-1.336469	2.330179	-0.126980
H	-0.768836	1.102362	1.109084
C	-2.837949	1.623652	1.182268
C	-3.276016	0.762120	2.288381
C	-4.614102	0.818223	2.730987
C	-2.377320	-0.107314	2.939855
C	-5.038832	0.027997	3.792695
H	-5.308906	1.482913	2.231186
C	-2.809779	-0.891268	4.005164
H	-1.338099	-0.164879	2.637939
C	-4.137390	-0.827025	4.435065
H	-6.072451	0.078665	4.123191
H	-2.102385	-1.553601	4.494796
H	-4.469619	-1.439062	5.269379
O	-3.758124	2.476038	0.749326
Si	-3.657472	4.104829	0.155192
C	-5.381298	4.748863	0.520581
H	-5.596617	4.730526	1.594733
H	-5.491894	5.783734	0.174908
H	-6.143052	4.145975	0.013297
C	-3.304062	4.125190	-1.685868
H	-4.004454	3.490325	-2.238032
H	-3.408229	5.150814	-2.063187
H	-2.290799	3.787531	-1.925767
C	-2.339125	5.007502	1.146036
H	-2.481993	4.869821	2.224005
H	-1.319322	4.695677	0.896961
H	-2.408270	6.083572	0.941501

TS 32

B3LYP/6-31G(d) = -3573.932107
B3LYP-D3(BJ)/def2-TZVPP/IEFPCM(propanonitrile) = -3575.509577
B3LYP-D3(BJ)/def2-TZVPP/IEFPCM(propanonitrile)//B3LYP-D3(BJ)/6-31G(d) Free Energy (Quasiharmonic) = -3574.792466

Frequencies (Top 3 out of 300)

1. -269.4662 cm^{-1}
2. 13.4381 cm^{-1}
3. 14.3346 cm^{-1}

B3LYP/6-31G(d) Molecular Geometry in Cartesian Coordinates

B	-0.458680	0.041000	-0.407850
O	-1.280629	-1.185011	-0.573730
N	-0.087340	0.367770	-1.896780
C	-0.703800	-0.631900	-2.796890
S	-0.023851	1.892430	-2.575170
C	-1.422079	-1.577371	-1.839610
O	-2.053929	-2.560391	-2.177380
H	0.623670	-0.694950	-4.481420
H	-1.480460	-0.176131	-3.429290
C	0.251701	-1.406130	-3.740820
C	1.399171	-2.098630	-3.063260
C	2.767941	-1.645989	-3.009440
C	3.499521	-2.596819	-2.246540
N	2.616701	-3.592229	-1.880510
C	1.357461	-3.281360	-2.363150
H	0.519482	-3.940930	-2.181420
C	5.511121	-1.348038	-2.525710
C	3.448510	-0.538169	-3.547710
H	5.404911	-3.184758	-1.387500
H	2.818942	-4.323409	-1.216490
C	4.809960	-0.401028	-3.302160
H	6.574171	-1.210157	-2.346490
H	2.912770	0.201691	-4.136640
C	4.867771	-2.456688	-1.988890
H	5.346810	0.448902	-3.715620
C	1.117179	2.788160	-1.526960
O	0.565679	1.749400	-3.917080
O	-1.337411	2.568959	-2.485860
C	2.915748	4.300461	-0.005910
C	0.673238	3.945130	-0.887870
C	2.451499	2.381381	-1.434770
C	3.335609	3.136501	-0.670610
C	1.577048	4.691851	-0.130920
H	-0.361952	4.249960	-0.990420
H	2.790739	1.483461	-1.940350
H	4.369669	2.813542	-0.583160
H	1.235358	5.595380	0.368350
C	3.884388	5.089941	0.840550
H	4.827218	5.265062	0.309790
H	3.469648	6.061591	1.126340
H	4.124598	4.540012	1.757470
H	-0.378299	-2.131440	-4.270240
C	0.769160	-0.217970	0.611260
C	1.368230	0.798330	1.370860
C	1.263501	-1.517190	0.787600
C	2.417920	0.529531	2.253940
C	2.302471	-1.796559	1.679860
H	0.811861	-2.332940	0.236300
C	2.892130	-0.772479	2.418360
H	3.688950	-0.984619	3.122460
C	2.794121	-3.206349	1.839640
C	3.082159	1.643831	3.011350
F	3.274731	-3.442699	3.081300
F	1.816732	-4.118979	1.608730
F	3.801051	-3.511679	0.975540
F	3.449110	1.262321	4.258010
F	4.216749	2.070252	2.395040
F	2.285329	2.728971	3.142940
O	-1.324340	1.128659	0.243200
C	-2.556601	1.361079	-0.098710
C	-3.089351	2.718509	0.150480
C	-4.196471	3.172598	-0.579850
C	-2.495301	3.562199	1.101480

C	-4.704562	4.451358	-0.363770
H	-4.640671	2.531898	-1.337580
C	-3.009072	4.838289	1.319130
H	-1.627811	3.212939	1.652000
C	-4.115022	5.284998	0.589610
H	-5.553302	4.801088	-0.944460
H	-2.544312	5.488789	2.054920
H	-4.511423	6.282298	0.759020
H	-2.950000	0.835549	-0.967790
C	-3.891340	0.357958	1.234560
H	-3.705770	1.035918	2.055750
H	-4.808030	0.555528	0.689630
C	-3.505410	-0.975632	1.395430
C	-2.477629	-1.410351	2.353530
C	-1.927200	-0.529781	3.304640
C	-2.021599	-2.743071	2.322170
C	-0.956130	-0.971181	4.197950
H	-2.253540	0.502569	3.356040
C	-1.048219	-3.178541	3.213190
H	-2.422999	-3.416601	1.575330
C	-0.513439	-2.294760	4.154880
H	-0.538980	-0.279550	4.923730
H	-0.688458	-4.201610	3.162150
H	0.253111	-2.634520	4.845700
O	-3.998799	-1.948182	0.654410
Si	-5.325229	-2.202942	-0.423890
C	-5.235400	-1.051362	-1.905890
H	-5.319070	0.009008	-1.644310
H	-4.301129	-1.212012	-2.453200
H	-6.061589	-1.281613	-2.590950
C	-6.893819	-1.927433	0.581640
H	-7.778009	-2.145663	-0.030080
H	-6.924709	-2.588083	1.455460
H	-6.987630	-0.895413	0.938120
H	1.009099	1.817600	1.282740
C	-5.080208	-3.984322	-0.932380
H	-4.096778	-4.086592	-1.403930
H	-5.127948	-4.661702	-0.072430
H	-5.842768	-4.300763	-1.654110

TS 33

B3LYP/6-31G(d) = -3573.932767
B3LYP-D3(BJ)/def2-TZVPP/IEFPCM(propanonitrile) = -3575.509718
B3LYP-D3(BJ)/def2-TZVPP/IEFPCM(propanonitrile)//B3LYP-D3(BJ)/6-31G(d) Free Energy (Quasiharmonic) = -3574.792394

Frequencies (Top 3 out of 300)

1. -266.8119 cm^{-1}
2. 11.5945 cm^{-1}
3. 15.6614 cm^{-1}

B3LYP/6-31G(d) Molecular Geometry in Cartesian Coordinates

B	-0.454720	-0.089760	-0.381960
O	-1.189571	-1.358820	-0.151110
N	-0.019730	-0.255050	-1.873190
C	-0.619421	-1.477690	-2.443890
S	0.160730	0.998780	-2.962250
C	-1.305101	-2.119460	-1.239300
O	-1.892121	-3.184700	-1.263750
H	0.677079	-2.017450	-4.068950
H	-1.413431	-1.237150	-3.168460
C	0.336739	-2.480180	-3.140880
C	1.516909	-2.925291	-2.324240
C	2.889349	-2.513271	-2.498100
C	3.661149	-3.179111	-1.506830
N	2.796999	-3.976291	-0.783760
C	1.511529	-3.808731	-1.270410
H	0.684089	-4.354250	-0.837040
C	5.659329	-2.144232	-2.293750
C	3.541989	-1.657391	-3.405410
H	5.612469	-3.504142	-0.612230
H	3.026309	-4.434711	0.084740
C	4.916549	-1.482681	-3.294680
H	6.732339	-1.983552	-2.230250
H	2.974959	-1.129851	-4.167240
C	5.043829	-2.999852	-1.388420
H	5.430919	-0.824792	-3.990360
C	1.326720	2.081309	-2.141650
O	-1.105100	1.744000	-3.130730
O	0.780610	0.442680	-4.176450
C	3.155631	3.802709	-0.909210
C	0.928811	3.372830	-1.796800
C	2.632840	1.643269	-1.909550
C	3.533690	2.506999	-1.293840
C	1.847231	4.223539	-1.181680
H	-0.084499	3.695230	-2.009900
H	2.938000	0.642249	-2.195540
H	4.548220	2.165619	-1.103540
H	1.541361	5.229779	-0.905400
C	4.125771	4.703439	-0.183360
H	3.874671	5.760089	-0.321850
H	5.153581	4.547648	-0.527730
H	4.104861	4.498819	0.894430
H	-0.289331	-3.339030	-3.412600
C	0.722560	0.119390	0.702690
C	0.993690	1.352670	1.309500
C	1.511209	-0.971221	1.096540
C	2.007410	1.495799	2.260010
H	0.409240	2.222070	1.033230
C	2.522779	-0.838451	2.052130
H	1.326729	-1.945391	0.657500
C	2.780720	0.399459	2.641020
H	3.563570	0.505959	3.383800
C	3.374259	-2.020691	2.422350
C	2.217170	2.814769	2.944820
F	3.819889	-1.946441	3.697790
F	4.470979	-2.133751	1.635580
F	2.699349	-3.195151	2.306830
F	1.567450	2.875249	4.140770
F	1.765541	3.858249	2.212290
F	3.524090	3.051749	3.211390
O	-1.432750	1.085430	-0.174350
C	-2.636900	1.107050	-0.657400
C	-3.246270	2.423541	-0.942950
C	-2.757119	3.593531	-0.342550
C	-4.322380	2.505901	-1.837820
C	-3.341529	4.823641	-0.632730
H	-1.913429	3.529920	0.336850
C	-4.901589	3.738171	-2.130440
H	-4.687990	1.604751	-2.324300
C	-4.415259	4.899181	-1.524970
H	-2.956599	5.727021	-0.167790
H	-5.725199	3.794551	-2.836370
H	-4.866429	5.860921	-1.752660
H	-2.939810	0.283711	-1.304350
C	-4.044390	0.541671	0.862610
H	-3.968410	1.477401	1.398710
H	-4.915230	0.461341	0.221640
C	-3.605310	-0.614259	1.512730
C	-2.635520	-0.598950	2.617230
C	-2.239131	-1.813960	3.209920
C	-2.077100	0.601320	3.097590
C	-1.324001	-1.823870	4.255420
H	-2.648121	-2.742030	2.830210
C	-1.150740	0.586430	4.135770
H	-2.346210	1.551210	2.650890
C	-0.774940	-0.625050	4.719470
H	-1.026311	-2.768230	4.701930

H	-0.697660	1.514890	4.468120
H	-0.049350	-0.635350	5.527990
O	-3.992831	-1.821169	1.147220
Si	-5.239121	-2.534429	0.185720
C	-4.854611	-4.356919	0.336440
H	-4.910972	-4.699289	1.375820
H	-5.551562	-4.961509	-0.256180
H	-3.840351	-4.536349	-0.036220
C	-6.874031	-2.049958	0.985780
H	-6.914641	-2.374058	2.031860
H	-7.045581	-0.967788	0.963400
H	-7.709631	-2.526658	0.458420
C	-5.143241	-1.963609	-1.602260
H	-5.911581	-2.485499	-2.187270
H	-4.170281	-2.226339	-2.029570
H	-5.314311	-0.889299	-1.731160

TS 34

B3LYP/6-31G(d) = -3573.929139
B3LYP-D3(BJ)/def2-TZVPP/IEFPCM(propanonitrile) = -3575.510283
B3LYP-D3(BJ)/def2-TZVPP/IEFPCM(propanonitrile)//B3LYP-D3(BJ)/6-31G(d) Free Energy (Quasiharmonic) = -3574.792384

Frequencies (Top 3 out of 300)

1. -291.7679 cm^{-1}
2. 11.8035 cm^{-1}
3. 16.2752 cm^{-1}

B3LYP/6-31G(d) Molecular Geometry in Cartesian Coordinates

B	-0.726702	-0.461295	0.640004
O	-0.510004	-1.077160	1.957106
N	-1.772877	-1.440391	0.020129
C	-1.903044	-2.643707	0.851271
S	-2.748818	-1.201974	-1.269993
C	-1.102596	-2.268193	2.103568
O	-1.015372	-2.955110	3.095780
H	-2.162970	-4.276617	-0.525251
H	-2.944555	-2.790264	1.170971
C	-1.420102	-3.982978	0.221577
C	-0.059558	-3.955326	-0.412639
C	1.223877	-4.191901	0.208527
C	2.214432	-4.033048	-0.802474
N	1.554212	-3.733293	-1.978099
C	0.196819	-3.669156	-1.731247
H	-0.501612	-3.415521	-2.515917
C	3.957625	-4.489337	0.760018
C	1.637580	-4.513740	1.514198
H	4.322355	-4.043836	-1.325171
H	1.991219	-3.412998	-2.828983
C	2.994983	-4.657809	1.775971
H	5.012293	-4.601233	0.996966
H	0.907332	-4.619405	2.311271
C	3.580540	-4.177896	-0.541951
H	3.323137	-4.898200	2.783844
C	-4.426245	-1.175854	-0.607051
O	-2.420309	0.140296	-1.794470
O	-2.719348	-2.340211	-2.204582
C	-7.062488	-1.087551	0.344334
C	-5.434839	-1.845303	-1.301224
C	-4.717036	-0.467121	0.560203
C	-6.028376	-0.431258	1.027914
C	-6.743189	-1.794879	-0.822014
H	-5.186572	-2.406514	-2.195252
H	-3.922411	0.036449	1.100027
H	-6.253020	0.111720	1.943064
H	-7.527653	-2.321632	-1.360176
C	-8.485691	-1.017902	0.845020
H	-9.068328	-1.885664	0.519544
H	-8.994098	-0.121978	0.464157
H	-8.524773	-0.972739	1.938596
O	-1.415892	0.890802	0.952430
C	-1.368081	1.949902	0.196743
H	-0.783297	1.886919	-0.719202
H	-1.460732	-4.725035	1.028380
C	-2.586741	2.798954	0.132304
C	-2.866199	3.502844	-1.046763
C	-3.464579	2.902152	1.221132
C	-4.007467	4.298388	-1.136623
H	-2.205500	3.398896	-1.903255
C	-4.600166	3.703100	1.131612
H	-3.251038	2.350221	2.131879
C	-4.873535	4.404362	-0.046644
H	-4.224888	4.827520	-2.060086
H	-5.275240	3.780145	1.979516
H	-5.761777	5.026211	-0.115763
C	0.679197	-0.309730	-0.160630
C	1.887440	-0.491869	0.537440
C	0.771001	-0.087540	-1.544019
C	3.124378	-0.436536	-0.107550
H	1.854739	-0.704001	1.599310
C	2.010203	-0.039794	-2.193670
H	-0.135423	0.029660	-2.130351
C	3.194655	-0.216404	-1.482102
H	4.153555	-0.188676	-1.987914
C	2.068612	0.116075	-3.683509
C	4.412455	-0.564662	0.655799
F	1.043858	0.857977	-4.162670
F	3.217079	0.717655	-4.088208
F	5.013896	0.657225	0.815153
F	2.017527	-1.081375	-4.324551
F	5.314532	-1.332654	0.004789
F	4.249104	-1.074540	1.888617
C	-0.154447	3.411468	1.062076
H	-0.268653	4.141670	0.269608
H	-0.776378	3.592677	1.929067
C	1.134921	2.922248	1.300916
C	1.505266	2.218989	2.533191
C	0.526282	1.755943	3.435117
C	2.865010	1.993117	2.826982
C	0.901011	1.083280	4.593333
H	-0.526360	1.871609	3.208984
C	3.232605	1.333057	3.992843
H	3.621832	2.315154	2.123793
C	2.252134	0.876867	4.879198
H	0.136879	0.702237	5.263415
H	4.283335	1.157169	4.202023
H	2.540417	0.348444	5.783517
O	2.102482	3.022856	0.403852
Si	2.482719	4.084956	-0.912562
C	4.100566	3.393697	-1.559849
H	3.925069	2.651884	-2.345712
H	4.723261	4.190838	-1.983481
H	4.668550	2.902840	-0.762581
C	1.160778	4.058364	-2.250357
H	0.204616	4.490643	-1.937125
H	1.520361	4.654490	-3.099841
H	0.980048	3.045691	-2.626735
C	2.666584	5.795270	-0.150314
H	1.742370	6.132560	0.332534
H	2.924169	6.531590	-0.921453
H	3.463032	5.809677	0.602247

TS 35

B3LYP/6-31G(d) = -3573.929101
B3LYP-D3(BJ)/def2-TZVPP/IEFPCM(propanonitrile) = -3575.509279

B3LYP-D3(BJ)/def2-TZVPP/IEFPCM(propanonitrile)//B3LYP-D3(BJ)/6-31G(d) Free Energy (Quasiharmonic) = -3574.792358

Frequencies (Top 3 out of 300)

1. -291.8565 cm^{-1}
2. 6.9378 cm^{-1}
3. 14.6440 cm^{-1}

B3LYP/6-31G(d) Molecular Geometry in Cartesian Coordinates

B	-0.728719	-0.456270	0.544780
O	-0.479484	-1.180610	1.799557
N	-1.843656	-1.337440	-0.091621
C	-2.038229	-2.568228	0.683907
S	-2.789361	-0.987929	-1.381652
C	-1.190061	-2.305947	1.932180
O	-1.154450	-3.023814	2.905739
H	-2.364675	-4.086341	-0.807267
H	-3.082649	-2.665639	1.011473
C	-1.639416	-3.902606	-0.009351
C	-0.247753	-3.957076	-0.570883
C	0.968764	-4.366078	0.094884
C	2.027040	-4.236505	-0.849120
N	1.468821	-3.777491	-2.024720
C	0.110116	-3.605793	-1.849832
H	-0.514873	-3.232768	-2.648948
C	3.622369	-4.997908	0.750748
C	1.273196	-4.829890	1.388093
H	4.150718	-4.422250	-1.266494
H	1.977485	-3.544188	-2.862871
C	2.590721	-5.141901	1.700841
H	4.644406	-5.242061	1.028310
H	0.492708	-4.915386	2.138149
C	3.355090	-4.544539	-0.536104
H	2.834087	-5.495561	2.699384
C	-4.477311	-1.017746	-0.750692
O	-2.733192	-2.039639	-2.412162
O	-2.457958	0.397528	-1.773299
C	-7.126582	-1.004213	0.164350
C	-4.817727	-0.257416	0.371038
C	-5.441748	-1.770810	-1.418552
C	-6.758384	-1.758411	-0.955900
C	-6.134393	-0.258264	0.820546
H	-4.056459	0.317533	0.887054
H	-5.155079	-2.359434	-2.282967
H	-7.509968	-2.349271	-1.473815
H	-6.398719	0.328959	1.697085
C	-8.555940	-0.977627	0.651506
H	-9.141924	-1.794889	0.219923
H	-8.608757	-1.061919	1.742864
H	-9.049669	-0.036379	0.376123
H	-1.782158	-4.686495	0.744542
C	0.639837	-0.276471	-0.312402
C	0.687088	0.198942	-1.633904
C	1.862234	-0.660891	0.269111
C	1.897428	0.307247	-2.329677
C	3.068778	-0.560726	-0.425330
H	1.863846	-1.057919	1.277033
C	3.095971	-0.077276	-1.733411
H	4.033667	0.003099	-2.272558
C	4.377991	-0.904641	0.227129
C	1.902862	0.798378	-3.748445
F	5.166558	-1.648494	-0.583438
F	5.097619	0.226588	0.508827
F	4.236433	-1.569173	1.386020
F	3.080936	1.396441	-4.071372
F	1.718530	-0.196585	-4.644533
F	0.926148	1.712069	-3.972343
O	-1.353635	0.888198	1.007303
C	-1.242225	2.035937	0.406499
C	-2.411528	2.952538	0.475670
C	-3.284158	2.937568	1.573760
C	-2.651303	3.838731	-0.582846
C	-4.376349	3.800780	1.611675
H	-3.100241	2.244256	2.389405
C	-3.749645	4.696893	-0.545987
H	-1.992797	3.833841	-1.447543
C	-4.611055	4.683153	0.552654
H	-5.047401	3.785994	2.466182
H	-3.937280	5.369182	-1.378368
H	-5.465613	5.353429	0.582464
H	-0.668793	2.068053	-0.518701
C	0.061591	3.276165	1.449162
H	-0.529579	3.347351	2.353054
H	-0.028801	4.130167	0.788323
C	1.329440	2.691833	1.563692
C	1.700130	1.782844	2.651737
C	0.735164	1.271837	3.542768
C	3.047957	1.401991	2.815130
C	1.110081	0.404602	4.563070
H	-0.313410	1.507902	3.410965
C	3.417533	0.545815	3.844440
H	3.790818	1.759672	2.113957
C	2.449942	0.045623	4.721569
H	0.352442	-0.009697	5.220480
H	4.456976	0.251109	3.950878
H	2.737988	-0.635637	5.517187
O	2.271924	2.886959	0.654664
Si	2.710031	4.158055	-0.437966
C	1.363142	4.499720	-1.705465
H	0.454234	4.935612	-1.277223
H	1.752552	5.221295	-2.436161
H	1.089900	3.597994	-2.263764
C	4.252132	3.487389	-1.264966
H	4.822507	2.847737	-0.582990
H	4.904903	4.305431	-1.592829
H	3.997140	2.889033	-2.145409
H	-0.234152	0.474502	-2.137906
C	3.046711	5.662727	0.640157
H	3.346267	6.517375	0.021291
H	3.856594	5.467339	1.352004
H	2.161906	5.963863	1.212564

TS 36

B3LYP/6-31G(d) = -3573.929154
B3LYP-D3(BJ)/def2-TZVPP/IEFPCM(propanonitrile) = -3575.510289
B3LYP-D3(BJ)/def2-TZVPP/IEFPCM(propanonitrile)//B3LYP-D3(BJ)/6-31G(d) Free Energy (Quasiharmonic) = -3574.79233

Frequencies (Top 3 out of 300)

1. -291.7912 cm^{-1}
2. 11.8538 cm^{-1}
3. 16.2877 cm^{-1}

B3LYP/6-31G(d) Molecular Geometry in Cartesian Coordinates

B	-0.728010	-0.459571	0.639340
O	-0.513129	-1.076231	1.956400
N	-1.775509	-1.436722	0.018560
C	-1.908048	-2.640122	0.849210
S	-2.750219	-1.196362	-1.272150
C	-1.107739	-2.266321	2.102100
O	-1.022278	-2.953721	3.094150
H	-2.169928	-4.272142	-0.527980
H	-2.949968	-2.785102	1.168280
C	-1.426958	-3.979971	0.219320
C	-0.066058	-3.954361	-0.414210
C	1.216692	-4.193120	0.207530

C	2.207982	-4.035669	-0.802970
N	1.548822	-3.734650	-1.978860
C	0.191422	-3.668340	-1.732640
H	-0.506228	-3.413411	-2.517590
C	3.949693	-4.495008	0.760290
C	1.629253	-4.515850	1.513340
H	4.316142	-4.049748	-1.324650
H	1.986752	-3.414649	-2.829390
C	2.986293	-4.662119	1.775750
H	5.004063	-4.608618	0.997730
H	0.898453	-4.620480	2.310050
C	3.573732	-4.182748	-0.541810
H	3.313583	-4.903199	2.783740
C	-4.427709	-1.168533	-0.609530
O	-2.722279	-2.334492	-2.206940
O	-2.418900	0.145348	-1.796260
C	-7.065079	-1.071675	0.337610
C	-4.717940	-0.459323	0.557460
C	-5.438419	-1.829874	-1.308610
C	-6.747679	-1.773014	-0.833000
C	-6.030420	-0.417064	1.021700
H	-3.923380	0.046057	1.095700
H	-5.192179	-2.383913	-2.207670
H	-7.535069	-2.287165	-1.379060
H	-6.256080	0.132746	1.932500
C	-8.478959	-1.047695	0.868080
H	-9.211409	-1.162666	0.062500
H	-8.646629	-1.866365	1.580570
H	-8.694760	-0.111455	1.393740
H	-1.469187	-4.722221	1.025870
C	0.678640	-0.310210	-0.160400
C	0.771670	-0.088210	-1.543740
C	1.886160	-0.494269	0.538420
C	2.011340	-0.042489	-2.192620
C	3.123580	-0.440929	-0.105800
H	1.852470	-0.706339	1.600280
C	3.195070	-0.220969	-1.480320
H	4.154330	-0.194808	-1.985530
C	4.410990	-0.571138	0.658330
C	2.070920	0.113121	-3.682440
F	5.312141	-1.340767	0.007960
F	5.014430	0.649742	0.817850
F	4.246051	-1.080568	1.891120
F	2.018351	-1.084349	-4.323380
F	1.047630	0.856550	-4.162340
F	3.220580	0.712821	-4.086510
O	-1.415001	0.893599	0.951780
C	-1.365211	1.952719	0.196220
C	-2.582492	2.803718	0.131510
C	-3.460132	2.908727	1.220340
C	-2.860892	3.507538	-1.047840
C	-4.594502	3.711347	1.130490
H	-3.247391	2.356888	2.131340
C	-4.000992	4.304717	-1.138020
H	-2.200392	3.402188	-1.904310
C	-4.866893	4.412457	-0.048090
H	-5.269412	3.789846	1.978400
H	-4.217703	4.833707	-2.061730
H	-5.754273	5.035486	-0.117510
H	-0.780281	1.888839	-0.719560
C	-0.149482	3.412219	1.062040
H	-0.771422	3.594419	1.928810
H	-0.262252	4.142619	0.269540
C	1.139018	2.920980	1.301370
C	1.507769	2.217160	2.533820
C	0.527669	1.755380	3.435180
C	2.867039	1.989501	2.828360
C	0.900859	1.082200	4.593590
H	-0.524691	1.872419	3.208440
C	3.233119	1.328931	3.994420
H	3.624679	2.310572	2.125610
C	2.251550	0.874021	4.880210
H	0.135850	0.702150	5.263240
H	4.283499	1.151672	4.204190
H	2.538640	0.345211	5.784690
O	2.107118	3.020081	0.404720
Si	2.489458	4.081521	-0.911630
C	2.675467	5.791631	-0.149450
H	3.471577	5.804941	0.603480
H	2.934496	6.527501	-0.920540
H	1.751516	6.130320	0.332920
C	1.168068	4.056700	-2.249990
H	0.986088	3.044270	-2.626410
H	1.528827	4.652310	-3.099340
H	0.212357	4.490300	-1.937180
H	-0.134200	0.030439	-2.130630
C	4.106608	3.387842	-1.558080
H	4.673488	2.896252	-0.760480
H	3.930428	2.646202	-2.343950
H	4.730678	4.184032	-1.981480

TS 37

B3LYP/6-31G(d) = -3573.928138
B3LYP-D3(BJ)/def2-TZVPP/IEFPCM(propanonitrile) = -3575.509836
B3LYP-D3(BJ)/def2-TZVPP/IEFPCM(propanonitrile)//B3LYP-D3(BJ)/6-31G(d) Free Energy (Quasiharmonic) = -3574.792306

Frequencies (Top 3 out of 300)

1. -293.9000 cm^{-1}
2. 10.4032 cm^{-1}
3. 17.2645 cm^{-1}

B3LYP/6-31G(d) Molecular Geometry in Cartesian Coordinates

B	-0.730160	-0.382979	0.860271
O	-0.646668	-0.770994	2.279164
N	-1.737408	-1.445960	0.319014
C	-1.970093	-2.490038	1.325640
S	-2.661743	-1.376926	-1.028990
C	-1.279327	-1.913965	2.567520
O	-1.282404	-2.430714	3.662237
H	-1.968807	-4.274332	0.117375
H	-3.041228	-2.582913	1.554421
C	-1.434408	-3.911168	0.999442
C	0.051755	-4.018307	0.788318
C	0.765449	-4.093591	-0.466623
C	2.150378	-4.191356	-0.156461
N	2.263855	-4.194020	1.219059
C	1.005653	-4.073951	1.777592
H	0.878147	-4.042340	2.851059
C	2.723442	-4.247592	-2.469733
C	0.376154	-4.081682	-1.818829
H	4.191921	-4.324255	-0.879871
H	3.133145	-4.098501	1.720502
C	1.355634	-4.157416	-2.801995
H	3.464883	-4.297727	-3.262858
H	-0.670416	-3.983055	-2.088065
C	3.139144	-4.266978	-1.143341
H	1.064188	-4.137643	-3.848775
C	-4.359036	-1.154862	-0.452286
O	-2.242309	-0.161316	-1.756561
O	-2.670446	-2.661141	-1.747391
C	-7.021242	-0.786415	0.351421
C	-4.650574	-0.283700	0.598415
C	-5.381336	-1.849446	-1.101723
C	-6.701532	-1.658614	-0.697902
C	-5.975255	-0.109534	0.994064
H	-3.848437	0.239493	1.107453
H	-5.134599	-2.539508	-1.900980

H	-7.496072	-2.204560	-1.201196
H	-6.200372	0.560455	1.820609
C	-8.456665	-0.568725	0.767811
H	-8.531207	-0.298651	1.826361
H	-8.915786	0.244848	0.190235
H	-9.062741	-1.465521	0.602272
H	-1.739710	-4.539140	1.845353
C	0.735170	-0.396179	0.163935
C	1.869755	-0.584470	0.972947
C	0.945047	-0.345833	-1.223979
C	3.147698	-0.713023	0.427379
C	2.228729	-0.450696	-1.772030
H	0.093977	-0.268125	-1.893859
C	3.338824	-0.635917	-0.952574
H	4.328594	-0.745648	-1.381610
C	2.424115	-0.327078	-3.254794
C	4.333028	-1.014236	1.297463
F	1.362133	-0.751657	-3.960432
F	3.509635	-0.998193	-3.692797
F	2.623329	0.984872	-3.615965
F	5.412024	-0.266917	0.960856
F	4.732045	-2.315699	1.184207
F	4.092818	-0.804747	2.608985
O	-1.407089	1.009439	0.895277
C	-1.292779	1.933401	-0.015468
C	-2.495890	2.762618	-0.296890
C	-3.405971	3.099020	0.715421
C	-2.727282	3.204532	-1.606417
C	-4.524723	3.874466	0.422149
H	-3.230858	2.748114	1.728412
C	-3.852450	3.974503	-1.899030
H	-2.045775	2.911506	-2.399923
C	-4.749403	4.316015	-0.885297
H	-5.224437	4.133914	1.211872
H	-4.033198	4.298335	-2.920049
H	-5.624488	4.918404	-1.112944
H	-0.649279	1.722617	-0.867675
C	-0.137241	3.505431	0.709920
H	-0.765807	3.724728	1.563542
H	-0.292326	4.161061	-0.138489
C	1.181841	3.120752	0.979215
C	1.607574	2.570393	2.270034
C	2.980911	2.492766	2.576924
C	0.667985	2.130215	3.223127
C	3.398824	2.009067	3.810333
H	3.708253	2.809991	1.838908
C	1.094736	1.624613	4.446886
H	-0.389980	2.129244	2.991266
C	2.457395	1.573496	4.748024
H	4.459320	1.954573	4.036786
H	0.362577	1.255708	5.158157
H	2.786796	1.183245	5.706931
O	2.137726	3.209008	0.067903
Si	2.421086	4.208752	-1.322970
C	2.385960	5.986491	-0.706556
H	3.141407	6.153314	0.069536
H	1.410489	6.262385	-0.290614
H	2.599314	6.677224	-1.531551
C	1.148985	3.903882	-2.670602
H	1.141554	2.851325	-2.970561
H	0.132332	4.203483	-2.396749
H	1.431404	4.487680	-3.556676
H	1.742785	-0.656920	2.046494
C	4.122305	3.660745	-1.874818
H	4.847477	3.695245	-1.054629
H	4.085904	2.637745	-2.262719
H	4.493360	4.308275	-2.678696

TS 38

B3LYP/6-31G(d) = -3573.936724
B3LYP-D3(BJ)/def2-TZVPP/IEFPCM(propanonitrile) = -3575.510098
B3LYP-D3(BJ)/def2-TZVPP/IEFPCM(propanonitrile)//B3LYP-D3(BJ)/6-31G(d) Free Energy (Quasiharmonic) = -3574.792303

Frequencies (Top 3 out of 300)

1. -283.8713 cm^{-1}
2. 13.6291 cm^{-1}
3. 15.1328 cm^{-1}

B3LYP/6-31G(d) Molecular Geometry in Cartesian Coordinates

B	0.059037	0.423298	-0.116027
O	0.715720	-0.052272	-1.364548
N	0.323802	1.956424	-0.228217
C	0.884482	2.302998	-1.539524
S	-0.279210	3.114597	0.784918
C	1.103803	0.930755	-2.179870
O	1.572544	0.740640	-3.283234
H	1.946887	4.151846	-1.242451
H	0.145264	2.831950	-2.162340
C	2.193244	3.140727	-1.572726
C	3.343046	2.615750	-0.759600
C	4.428085	1.764495	-1.196104
C	5.301815	1.593544	-0.083212
N	4.767748	2.311777	0.965830
C	3.591725	2.910664	0.560079
H	3.007726	3.518991	1.235928
C	6.767074	0.220637	-1.373733
C	4.761466	1.143331	-2.414832
H	7.125956	0.713963	0.702852
H	5.120190	2.314708	1.910306
C	5.921400	0.380718	-2.490906
H	7.669047	-0.378740	-1.465809
H	4.108328	1.244648	-3.276202
C	6.469293	0.824870	-0.156826
H	6.184265	-0.101204	-3.428871
C	-1.785292	3.763491	0.039742
O	0.664333	4.244706	0.823048
O	-0.655178	2.428557	2.028555
C	-4.120641	4.763780	-1.152279
C	-1.695030	4.756037	-0.940426
C	-3.031349	3.283675	0.449004
C	-4.184936	3.788114	-0.147733
C	-2.859558	5.243179	-1.531501
H	-0.728009	5.167346	-1.209933
H	-3.100595	2.543478	1.236763
H	-5.153700	3.419257	0.180751
H	-2.787040	6.018732	-2.290288
C	-5.377234	5.276086	-1.814964
H	-6.219548	5.296166	-1.115388
H	-5.668962	4.632859	-2.655914
H	-5.238907	6.287416	-2.210912
H	2.476396	3.200671	-2.630248
C	-1.468962	-0.107552	-0.053992
C	-2.234352	-0.106310	1.122516
C	-2.069598	-0.647245	-1.199556
C	-3.546242	-0.588225	1.140061
H	-1.803326	0.292610	2.034098
C	-3.365463	-1.170926	-1.176371
H	-1.507197	-0.687435	-2.126409
C	-4.122388	-1.133670	-0.007266
H	-5.131367	-1.526814	0.012784
C	-3.885709	-1.858169	-2.404852
C	-4.336056	-0.479423	2.412622
F	-5.210270	-2.111820	-2.342125
F	-3.266923	-3.062644	-2.591997
F	-3.660309	-1.143422	-3.528103
F	-3.649747	-0.956660	3.478289
F	-5.507012	-1.153673	2.358626

F	-4.638834	0.813265	2.700291
O	0.778807	-0.150941	1.088231
C	2.016565	-0.563160	1.063975
C	2.758494	-0.457071	2.347636
C	2.103573	0.000789	3.501262
C	4.127369	-0.767589	2.406824
C	2.807951	0.129617	4.698739
H	1.060887	0.291065	3.434042
C	4.824974	-0.638542	3.604934
H	4.646353	-1.097156	1.510426
C	4.164800	-0.194633	4.756288
H	2.297089	0.494817	5.585308
H	5.883295	-0.882726	3.643228
H	4.710190	-0.094668	5.690916
H	2.587413	-0.362034	0.157556
C	2.149748	-2.611845	0.733856
H	2.978314	-2.615174	0.035279
H	2.410242	-2.921654	1.736346
C	0.910322	-3.062189	0.247319
C	-0.221678	-3.363160	1.134922
C	-1.413198	-3.890444	0.598470
C	-0.146445	-3.131936	2.523644
C	-2.485498	-4.194614	1.429638
H	-1.498121	-4.035298	-0.471660
C	-1.226747	-3.427226	3.347263
H	0.745007	-2.699058	2.962852
C	-2.396849	-3.964084	2.804786
H	-3.398096	-4.599486	1.002347
H	-1.160513	-3.230283	4.413011
H	-3.240543	-4.189893	3.450570
O	0.643727	-3.158067	-1.033152
Si	1.453140	-3.416216	-2.553569
C	3.144564	-2.612667	-2.660416
H	3.091517	-1.529247	-2.517456
H	3.882871	-3.033901	-1.969745
H	3.526820	-2.779348	-3.676743
C	0.268056	-2.705517	-3.811927
H	0.382989	-1.617513	-3.859581
H	0.483758	-3.110983	-4.808379
H	-0.773753	-2.941487	-3.570270
C	1.584617	-5.292595	-2.648288
H	2.048433	-5.595641	-3.595128
H	2.198621	-5.698298	-1.835530
H	0.597992	-5.766096	-2.594087

TS 39

B3LYP/6-31G(d) = -3573.924417
B3LYP-D3(BJ)/def2-TZVPP/IEFPCM(propanonitrile) = -3575.508822
B3LYP-D3(BJ)/def2-TZVPP/IEFPCM(propanonitrile)//B3LYP-D3(BJ)/6-31G(d) Free Energy (Quasiharmonic) = -3574.792278

Frequencies (Top 3 out of 300)

1. -274.4467 cm^{-1}
2. 8.1628 cm^{-1}
3. 12.9497 cm^{-1}

B3LYP/6-31G(d) Molecular Geometry in Cartesian Coordinates

B	0.338716	0.537503	-0.591525
O	0.303029	0.278429	-2.047882
N	0.896330	1.996533	-0.564017
C	1.352371	2.394672	-1.900952
S	0.781493	3.106864	0.635720
C	0.848119	1.247178	-2.784594
O	0.957657	1.208905	-3.991054
H	3.009526	2.918762	-3.129248
H	0.850165	3.315040	-2.232301
C	2.881956	2.593492	-2.089530
C	3.730704	1.379633	-1.821781
C	4.501766	1.082934	-0.635476
C	5.137394	-0.171562	-0.850487
N	4.783616	-0.600492	-2.114689
C	3.923847	0.323247	-2.680157
H	3.512567	0.162719	-3.667305
C	6.138138	-0.097440	1.312629
C	4.710076	1.745118	0.588289
H	6.432333	-1.732852	-0.074609
H	4.922237	-1.537673	-2.459958
C	5.520123	1.148210	1.547133
H	6.761505	-0.539781	2.085179
H	4.217107	2.690521	0.791557
C	5.957778	-0.772062	0.110243
H	5.673745	1.644997	2.501205
C	-0.428720	4.336751	0.109637
O	2.044552	3.850853	0.764800
O	0.219519	2.400922	1.803232
C	-2.307711	6.242298	-0.736221
C	-1.780397	4.155302	0.404483
C	-0.003299	5.467863	-0.591469
C	-0.944364	6.405854	-1.014532
C	-2.706760	5.105597	-0.018760
H	-2.097805	3.289419	0.972137
H	1.054077	5.619744	-0.779228
H	-0.610882	7.284000	-1.562541
H	-3.758812	4.961912	0.217105
C	-3.316089	7.282093	-1.164766
H	-3.447342	8.048384	-0.389061
H	-4.299026	6.835793	-1.349448
H	-2.998913	7.796035	-2.078152
O	-1.123671	0.479093	-0.172060
C	-1.586298	0.105737	1.000472
H	-0.871965	0.105782	1.826412
H	3.195349	3.421218	-1.447772
C	-2.933248	0.639636	1.360782
C	-3.792013	1.146444	0.375131
C	-3.342024	0.656272	2.701515
C	-5.039998	1.660233	0.728643
H	-3.455772	1.159817	-0.656543
C	-4.589765	1.168143	3.053642
H	-2.672018	0.284239	3.473297
C	-5.443745	1.669123	2.066931
H	-5.693656	2.064944	-0.039832
H	-4.889587	1.190621	4.097654
H	-6.412623	2.077336	2.341956
C	1.267954	-0.570998	0.152516
C	1.764967	-0.443819	1.463086
C	1.641510	-1.722363	-0.555512
C	2.571973	-1.430143	2.036488
H	1.543787	0.452504	2.034586
C	2.447555	-2.713318	0.015613
H	1.308782	-1.832257	-1.582428
C	2.916979	-2.576880	1.319788
H	3.552651	-3.333586	1.763130
C	2.812154	-3.908208	-0.812888
C	3.040462	-1.282195	3.458778
F	3.580618	-3.581695	-1.886713
F	3.484392	-4.847900	-0.120265
F	3.216177	0.005022	3.811492
F	1.703414	-4.517042	-1.330430
F	4.206009	-1.932011	3.680439
F	2.136147	-1.806256	4.331141
C	-1.780123	-1.821490	1.086035
H	-0.758266	-2.073770	0.826167
H	-2.039048	-1.940293	2.134775
C	-2.781108	-2.194976	0.171693
O	-4.030230	-2.126140	0.583532
Si	-5.513050	-3.011724	0.488061
C	-5.960341	-3.294849	2.286820
H	-6.932731	-3.794345	2.376403

H	-6.019618	-2.342442	2.825282
H	-5.216310	-3.920862	2.792290
C	-6.758794	-1.891117	-0.355373
H	-6.576762	-1.797001	-1.431282
H	-7.772957	-2.288829	-0.226039
H	-6.733743	-0.887636	0.083672
C	-5.226159	-4.624324	-0.430317
H	-6.142688	-5.227126	-0.400042
H	-4.964099	-4.466795	-1.480975
H	-4.429136	-5.218001	0.031934
C	-2.501889	-2.471021	-1.239678
C	-3.389222	-2.028519	-2.241207
C	-1.334159	-3.161363	-1.613667
C	-3.102952	-2.259590	-3.581799
H	-4.269443	-1.458882	-1.962559
C	-1.064693	-3.409227	-2.955905
H	-0.647808	-3.526675	-0.859010
C	-1.943220	-2.954030	-3.940912
H	-3.774341	-1.887550	-4.349982
H	-0.161362	-3.947347	-3.224629
H	-1.721590	-3.131150	-4.989305

TS 40

B3LYP/6-31G(d) = -3573.934795
B3LYP-D3(BJ)/def2-TZVPP/IEFPCM(propanonitrile) = -3575.508788
B3LYP-D3(BJ)/def2-TZVPP/IEFPCM(propanonitrile)//B3LYP-D3(BJ)/6-31G(d) Free Energy (Quasiharmonic) = -3574.792249

Frequencies (Top 3 out of 300)

1. -260.5763 cm^{-1}
2. 10.8847 cm^{-1}
3. 11.3239 cm^{-1}

B3LYP/6-31G(d) Molecular Geometry in Cartesian Coordinates

B	0.371116	-0.543378	-0.243156
O	1.131512	0.584508	-0.829508
N	-0.325388	-1.124690	-1.512672
C	0.058638	-0.364943	-2.710829
S	-0.923408	-2.662916	-1.669890
C	1.080359	0.629419	-2.163716
O	1.765883	1.366565	-2.844539
H	-1.796795	-0.365479	-3.811576
H	0.559082	-1.014995	-3.437848
C	-1.085057	0.385650	-3.454541
C	-1.790529	1.445968	-2.657360
C	-1.482140	2.859554	-2.617252
C	-2.391340	3.460291	-1.703388
N	-3.226725	2.463422	-1.240061
C	-2.846121	1.258747	-1.797841
H	-3.369663	0.348851	-1.540965
C	-1.418569	5.606860	-2.056406
C	-0.537447	3.678141	-3.263484
H	-3.055263	5.255925	-0.682640
H	-3.841246	2.561501	-0.446369
C	-0.517590	5.037937	-2.980755
H	-1.370132	6.672568	-1.848606
H	0.182957	3.247415	-3.952081
C	-2.365590	4.826797	-1.404253
H	0.211049	5.676604	-3.473284
C	-2.713700	-2.541034	-1.844153
O	-0.424511	-3.228829	-2.933362
O	-0.688662	-3.352047	-0.393133
C	-5.501029	-2.376684	-2.119737
C	-3.291026	-2.625946	-3.111585
C	-3.513744	-2.402520	-0.706684
C	-4.896902	-2.318488	-0.854383
C	-4.677095	-2.540365	-3.240799
H	-2.658843	-2.782055	-3.979215
H	-3.064624	-2.384258	0.280394
H	-5.520182	-2.219454	0.031334
H	-5.125164	-2.612413	-4.228922
C	-6.999110	-2.255994	-2.267868
H	-7.355548	-2.768391	-3.167280
H	-7.522839	-2.679285	-1.404324
H	-7.303819	-1.204088	-2.349703
H	-0.628702	0.830321	-4.346915
C	-0.596854	-0.111275	0.967717
C	-1.082675	-1.049066	1.889349
C	-1.003994	1.221823	1.130756
C	-1.943786	-0.675555	2.926638
C	-1.861768	1.598626	2.168320
H	-0.635127	1.978016	0.446078
C	-2.340261	0.651112	3.075419
H	-2.998354	0.942661	3.885766
C	-2.304605	3.026404	2.310809
C	-2.372292	-1.714290	3.921980
F	-2.472283	3.379052	3.606779
F	-3.514146	3.250406	1.706587
F	-1.437286	3.898493	1.763268
F	-1.363717	-2.031293	4.777100
F	-2.738772	-2.868085	3.317098
F	-3.414035	-1.308395	4.681455
O	1.395221	-1.537521	0.343839
C	2.543843	-1.779060	-0.205653
C	3.093126	-3.145998	-0.078700
C	2.494676	-4.077942	0.783099
C	4.191654	-3.534018	-0.862764
C	3.003505	-5.371567	0.868611
H	1.615486	-3.787510	1.345871
C	4.694766	-4.828577	-0.776450
H	4.640973	-2.822476	-1.551916
C	4.104820	-5.748173	0.095719
H	2.530222	-6.092764	1.528724
H	5.539156	-5.124156	-1.392630
H	4.495265	-6.759972	0.162117
H	2.791147	-1.241946	-1.122630
C	4.087509	-0.750718	0.862691
H	4.067351	-1.407007	1.722002
H	4.894934	-0.941943	0.165888
C	3.682306	0.573233	1.042351
C	2.811321	1.003574	2.145244
C	2.493437	2.368785	2.289167
C	2.270810	0.082289	3.063833
C	1.673816	2.797640	3.326088
H	2.887546	3.079762	1.573418
C	1.441744	0.514482	4.094482
H	2.470215	-0.977729	2.962566
C	1.145106	1.872179	4.230986
H	1.429872	3.851731	3.419752
H	1.000880	-0.212812	4.769552
H	0.490484	2.207869	5.030367
O	4.002493	1.533465	0.196738
Si	5.120895	1.829638	-1.087408
C	6.812205	1.953001	-0.267987
H	7.582066	2.189376	-1.012916
H	6.830324	2.743030	0.491106
H	7.101404	1.013968	0.218251
C	4.501981	3.449627	-1.781594
H	3.483443	3.308361	-2.159708
H	4.486834	4.239396	-1.022330
H	5.131905	3.791400	-2.611788
H	-0.793435	-2.090309	1.788665
C	5.088508	0.479737	-2.394647
H	5.444572	-0.492660	-2.037671
H	4.079386	0.372501	-2.805354
H	5.745605	0.779221	-3.221746

TS 41

B3LYP/6-31G(d) = -3573.934992
B3LYP-D3(BJ)/def2-TZVPP/IEFPCM(propanonitrile) = -3575.509316
B3LYP-D3(BJ)/def2-TZVPP/IEFPCM(propanonitrile)//B3LYP-D3(BJ)/6-31G(d) Free Energy (Quasiharmonic) = -3574.792165

Frequencies (Top 3 out of 300)

1. -268.9578 cm^{-1}
2. 11.8317 cm^{-1}
3. 15.3743 cm^{-1}

B3LYP/6-31G(d) Molecular Geometry in Cartesian Coordinates

B	-0.017651	0.455700	0.187790
O	-0.619861	0.137740	1.500120
N	-0.272421	1.994260	0.120620
C	-0.776672	2.496830	1.404990
S	0.277938	3.025931	-1.044940
C	-0.957531	1.213280	2.219620
O	-1.358201	1.168010	3.361130
H	-1.867332	4.299770	0.954190
H	-0.013792	3.099830	1.922340
C	-2.089012	3.329120	1.402290
C	-3.273862	2.723279	0.702830
C	-4.293961	1.852349	1.247710
C	-5.241161	1.615419	0.209050
N	-4.813561	2.315399	-0.899220
C	-3.629382	2.960819	-0.603430
H	-3.112942	3.555049	-1.343820
C	-6.551331	0.226108	1.641670
C	-4.511831	1.255329	2.504500
H	-7.083731	0.647828	-0.413370
H	-5.224201	2.258079	-1.818150
C	-5.631101	0.450479	2.686840
H	-7.420191	-0.402872	1.817410
H	-3.801011	1.405839	3.310650
C	-6.369541	0.807198	0.391060
H	-5.806651	-0.009772	3.655960
C	1.813158	3.756251	-0.448480
O	-0.670172	4.145040	-1.174440
O	0.603489	2.201111	-2.218190
C	4.194168	4.883772	0.520040
C	1.761698	4.858041	0.410470
C	3.042458	3.228401	-0.848620
C	4.218918	3.796052	-0.363710
C	2.948607	5.408281	0.891140
H	0.805848	5.300601	0.669980
H	3.081839	2.400581	-1.545700
H	5.173678	3.387252	-0.685800
H	2.905707	6.268211	1.555370
C	5.475517	5.467322	1.066130
H	5.788428	4.943922	1.979480
H	5.358177	6.525562	1.321460
H	6.295118	5.379383	0.345270
H	-2.323232	3.501660	2.459700
C	1.498549	-0.111009	0.121330
C	2.233819	-0.202439	-1.069950
C	2.113430	-0.598029	1.284250
C	3.526990	-0.734878	-1.092180
H	1.794529	0.165291	-1.991100
C	3.392310	-1.162558	1.261810
H	1.572040	-0.561869	2.223840
C	4.117110	-1.227588	0.072390
H	5.113990	-1.650908	0.052460
C	3.938780	-1.771028	2.522020
C	4.284180	-0.724488	-2.388510
F	3.720560	-0.990768	3.601960
F	5.266390	-2.007748	2.451700
F	3.344300	-2.969848	2.792650
F	3.540000	-1.219078	-3.410590
F	5.420800	-1.451848	-2.335960
F	4.636569	0.535632	-2.750990
O	-0.785371	-0.244710	-0.929150
C	-2.041500	-0.576110	-0.859890
C	-2.813441	-0.487911	-2.128020
C	-4.184570	-0.792071	-2.160750
C	-2.178361	-0.040950	-3.297950
C	-4.903070	-0.671431	-3.347460
H	-4.692580	-1.107571	-1.253700
C	-2.904221	0.081629	-4.482960
H	-1.134961	0.250760	-3.253130
C	-4.262651	-0.239631	-4.514170
H	-5.962520	-0.913412	-3.364320
H	-2.407951	0.439890	-5.380470
H	-4.824281	-0.146371	-5.439880
H	-2.573131	-0.329000	0.060250
C	-2.250820	-2.631940	-0.505080
H	-3.244200	-2.602331	-0.075140
H	-2.216930	-2.964890	-1.534980
C	-1.188930	-3.017170	0.321270
C	-1.197330	-2.925670	1.784380
C	-0.020860	-3.217680	2.502410
C	-2.345320	-2.524210	2.496080
C	0.007680	-3.104640	3.887510
H	0.875381	-3.501819	1.966170
C	-2.310860	-2.411790	3.880100
H	-3.263390	-2.272331	1.976400
C	-1.136310	-2.701050	4.580030
H	0.931640	-3.310989	4.419320
H	-3.197090	-2.081781	4.412910
H	-1.111330	-2.600650	5.661470
O	-0.035280	-3.399600	-0.199990
Si	0.440101	-4.201389	-1.663240
C	2.181711	-4.758229	-1.259300
H	2.195941	-5.454999	-0.413900
H	2.636471	-5.263929	-2.119790
H	2.814791	-3.901879	-1.002850
C	-0.723999	-5.665200	-1.887610
H	-0.382619	-6.283360	-2.727290
H	-0.737429	-6.300460	-0.994550
H	-1.754799	-5.363200	-2.101210
C	0.408210	-3.038229	-3.133780
H	-0.543600	-2.510980	-3.252010
H	1.203380	-2.290099	-3.062810
H	0.582231	-3.617569	-4.050220

TS 42

B3LYP/6-31G(d) = -3573.924435
B3LYP-D3(BJ)/def2-TZVPP/IEFPCM(propanonitrile) = -3575.50882
B3LYP-D3(BJ)/def2-TZVPP/IEFPCM(propanonitrile)//B3LYP-D3(BJ)/6-31G(d) Free Energy (Quasiharmonic) = -3574.792128

Frequencies (Top 3 out of 300)

1. -274.4625 cm^{-1}
2. 8.3005 cm^{-1}
3. 13.0087 cm^{-1}

B3LYP/6-31G(d) Molecular Geometry in Cartesian Coordinates

B	0.339220	0.539120	-0.590420
O	0.303290	0.282970	-2.047300
N	0.897472	1.997859	-0.560030
C	1.353382	2.398549	-1.896250
S	0.782333	3.106199	0.641530
C	0.848551	1.253049	-2.782130
O	0.957751	1.217259	-3.988700
H	3.196673	3.423677	-1.441350

H	0.851473	3.319799	-2.225610
C	2.882992	2.597267	-2.084650
C	3.731371	1.382647	-1.819270
C	4.502371	1.083516	-0.633550
C	5.137610	-0.170775	-0.850890
N	4.783639	-0.597264	-2.115870
C	3.924140	0.327786	-2.679600
H	3.512780	0.169207	-3.667030
C	6.138470	-0.100896	1.312310
C	4.710901	1.743396	0.591420
H	6.432148	-1.733846	-0.077930
H	4.921959	-1.533844	-2.462890
C	5.520811	1.144495	1.549140
H	6.761739	-0.544836	2.084020
H	4.218212	2.688566	0.796440
C	5.957869	-0.773265	0.108700
H	5.674621	1.639485	2.504110
C	-0.428096	4.335900	0.115470
O	2.045313	3.850038	0.772050
O	0.220222	2.398350	1.807820
C	-2.306524	6.244222	-0.725660
C	-0.002025	5.469970	-0.580500
C	-1.779446	4.155662	0.412450
C	-2.705215	5.108572	-0.006350
C	-0.942434	6.410711	-0.998810
H	1.056055	5.625429	-0.761220
H	-2.096037	3.292582	0.984830
H	-3.755996	4.969573	0.237710
H	-0.607514	7.294880	-1.536100
C	-3.321694	7.254913	-1.204600
H	-3.691894	6.998713	-2.206460
H	-4.189814	7.298404	-0.538410
H	-2.889843	8.259263	-1.266060
H	3.010553	2.924487	-3.123760
C	1.268199	-0.571091	0.151350
C	1.641188	-1.721312	-0.558820
C	1.765540	-0.446432	1.462040
C	2.447028	-2.713552	0.010350
C	2.572299	-1.434062	2.033520
H	1.544810	0.448949	2.035200
C	2.916768	-2.579623	1.314670
H	3.552287	-3.337343	1.756510
C	3.041109	-1.288903	3.455990
C	2.811026	-3.907073	-0.820380
F	4.206738	-1.939104	3.676080
F	3.216830	-0.002393	3.811220
F	2.137018	-1.814772	4.327510
F	3.483506	-4.848043	-0.129740
F	1.701926	-4.514872	-1.338370
F	3.578957	-3.578793	-1.894050
O	-1.123110	0.480311	-0.170920
C	-1.585560	0.105771	1.001340
C	-2.932229	0.639703	1.362640
C	-3.340659	0.654743	2.703500
C	-3.791009	1.148173	0.377880
C	-4.588109	1.166674	3.056600
H	-2.670620	0.281452	3.474630
C	-5.038689	1.662014	0.732350
H	-3.454969	1.162803	-0.653850
C	-5.442109	1.669305	2.070750
H	-4.887659	1.187924	4.100720
H	-5.692308	2.068135	-0.035410
H	-6.410748	2.077566	2.346540
H	-0.871000	0.104731	1.827080
C	-1.779802	-1.821418	1.084780
H	-0.758182	-2.073669	0.823980
H	-2.038052	-1.941348	2.133560
C	-2.781502	-2.193698	0.170730
C	-2.503392	-2.468358	-1.241130
C	-3.391712	-2.025197	-2.241500
C	-1.335833	-3.158099	-1.616760
C	-3.106602	-2.255087	-3.582540
H	-4.271831	-1.456026	-1.961570
C	-1.067503	-3.404749	-2.959450
H	-0.648733	-3.523939	-0.863040
C	-1.947033	-2.948948	-3.943290
H	-3.778812	-1.882617	-4.349810
H	-0.164284	-3.942430	-3.229430
H	-1.726323	-3.125178	-4.992020
O	-4.030322	-2.124986	0.583510
Si	-5.513353	-3.010275	0.488950
C	-6.759762	-1.888624	-0.352120
H	-6.733851	-0.885494	0.087680
H	-6.578962	-1.793724	-1.428170
H	-7.773902	-2.286103	-0.221900
C	-5.958733	-3.294995	2.287930
H	-6.017312	-2.343065	2.827320
H	-6.931093	-3.794454	2.378110
H	-5.214234	-3.921555	2.792020
H	1.308188	-1.829261	-1.585870
C	-5.227694	-4.622065	-0.431230
H	-4.966574	-4.463586	-1.481990
H	-4.430375	-5.216356	0.029710
H	-6.144325	-5.224695	-0.400680

TS 43

B3LYP/6-31G(d) = -3573.933665
B3LYP-D3(BJ)/def2-TZVPP/IEFPCM(propanonitrile) = -3575.509762
B3LYP-D3(BJ)/def2-TZVPP/IEFPCM(propanonitrile)//B3LYP-D3(BJ)/6-31G(d) Free Energy (Quasiharmonic) = -3574.792041

Frequencies (Top 3 out of 300)

1. -266.3607 cm^{-1}
2. 9.9138 cm^{-1}
3. 12.2022 cm^{-1}

B3LYP/6-31G(d) Molecular Geometry in Cartesian Coordinates

B	-0.116587	0.572701	0.027610
O	-0.433210	0.268117	1.454816
N	-0.395325	2.095052	0.001667
C	-0.696444	2.616941	1.341985
S	-0.127851	3.113599	-1.275117
C	-0.623246	1.355247	2.203185
O	-0.709565	1.332625	3.415156
H	-1.969000	4.329723	1.046566
H	0.098661	3.293233	1.694094
C	-2.045136	3.367889	1.558209
C	-3.287842	2.658404	1.104921
C	-4.093298	1.703507	1.834643
C	-5.162738	1.319292	0.977052
N	-5.024784	2.031966	-0.196718
C	-3.890465	2.815771	-0.120548
H	-3.582285	3.436027	-0.950531
C	-6.041339	-0.150014	2.636153
C	-4.030825	1.143830	3.124883
H	-6.932858	0.100141	0.679992
H	-5.530635	1.845241	-1.048967
C	-5.001682	0.227782	3.511007
H	-6.781753	-0.874494	2.965336
H	-3.223993	1.409919	3.800840
C	-6.136051	0.390420	1.359418
H	-4.959363	-0.209250	4.505474
C	1.426336	3.962780	-0.939346
O	-1.156183	4.166474	-1.274688
O	0.079228	2.266916	-2.453203
C	3.854254	5.272057	-0.430080
C	1.432771	5.147545	-0.199129
C	2.617110	3.432976	-1.439959

C	3.819046	4.088503	-1.181501
C	2.645139	5.787785	0.055843
H	0.497421	5.575587	0.145519
H	2.591662	2.526915	-2.034920
H	4.744960	3.673176	-1.571810
H	2.649317	6.710373	0.631594
C	5.159216	5.992119	-0.184340
H	5.992697	5.288259	-0.085957
H	5.117507	6.601044	0.724634
H	5.400737	6.665749	-1.017230
H	-2.090461	3.572780	2.635242
C	-0.920671	-0.372022	-1.000881
C	-2.210722	-0.052377	-1.448814
C	-0.393987	-1.599989	-1.417403
C	-2.939734	-0.922007	-2.265217
H	-2.649111	0.895315	-1.157436
C	-1.126138	-2.487038	-2.211550
H	0.605792	-1.880403	-1.109252
C	-2.407715	-2.155159	-2.645203
H	-2.975266	-2.834886	-3.269173
C	-0.536275	-3.832367	-2.510019
C	-4.334514	-0.552383	-2.687212
F	-0.527538	-4.627672	-1.394723
F	0.757163	-3.752910	-2.907253
F	-1.210761	-4.509403	-3.459742
F	-5.243313	-0.778785	-1.702497
F	-4.749567	-1.252199	-3.765272
F	-4.443344	0.764832	-2.999140
O	1.375033	0.216915	-0.162795
C	2.311446	0.463078	0.707416
C	3.705489	0.456463	0.200717
C	4.730602	1.036001	0.965360
C	4.006501	-0.079301	-1.060150
C	6.035048	1.077337	0.479981
H	4.499960	1.469351	1.936401
C	5.312920	-0.033898	-1.545061
H	3.207723	-0.506487	-1.656424
C	6.328974	0.540797	-0.777893
H	6.820053	1.535124	1.075517
H	5.536933	-0.445611	-2.525020
H	7.345901	0.575917	-1.159231
H	2.100325	1.210419	1.477512
C	2.377142	-0.956639	2.235830
H	1.582134	-0.559964	2.855750
H	3.385221	-0.737063	2.562696
C	2.135782	-2.194207	1.625037
C	3.167793	-2.977451	0.924682
C	4.541557	-2.776060	1.162958
C	2.776564	-3.960550	-0.007315
C	5.493033	-3.536703	0.490913
H	4.871280	-2.038293	1.885652
C	3.733527	-4.705312	-0.688715
H	1.723821	-4.120996	-0.204847
C	5.092800	-4.499744	-0.438763
H	6.548211	-3.377566	0.692685
H	3.415958	-5.444678	-1.417883
H	5.838392	-5.088939	-0.965648
O	0.911810	-2.661573	1.514585
Si	-0.366205	-3.113421	2.618484
C	-0.290578	-2.104113	4.196689
H	0.676079	-2.198496	4.705538
H	-0.500956	-1.042237	4.029686
H	-1.054376	-2.490038	4.885159
C	0.009590	-4.925755	2.970695
H	0.986249	-5.055747	3.451768
H	-0.747717	-5.348771	3.642648
H	0.005247	-5.519111	2.049537
C	-1.953804	-2.915608	1.659670
H	-2.788506	-3.332128	2.237310
H	-2.171360	-1.862677	1.462016
H	-1.905687	-3.446316	0.703347

TS 44

B3LYP/6-31G(d) = -3573.934005
B3LYP-D3(BJ)/def2-TZVPP/IEFPCM(propanonitrile) = -3575.508527
B3LYP-D3(BJ)/def2-TZVPP/IEFPCM(propanonitrile)//B3LYP-D3(BJ)/6-31G(d) Free Energy (Quasiharmonic) = -3574.792

Frequencies (Top 3 out of 300)

1. -263.4920 cm^{-1}
2. 5.2564 cm^{-1}
3. 12.0694 cm^{-1}

B3LYP/6-31G(d) Molecular Geometry in Cartesian Coordinates

B	0.252830	-0.325406	0.084006
O	-0.422224	0.244127	-1.114941
N	0.234417	-1.839982	-0.273430
C	-0.214174	-2.055031	-1.653645
S	1.043816	-3.011097	0.572560
C	-0.660816	-0.651634	-2.077306
O	-1.158373	-0.368818	-3.147210
H	-0.878136	-4.095431	-1.768551
H	0.630662	-2.325142	-2.309009
C	-1.326503	-3.108840	-1.903658
C	-2.563382	-3.009745	-1.055648
C	-3.804231	-2.332162	-1.361390
C	-4.705147	-2.601774	-0.290367
N	-4.035850	-3.403755	0.610346
C	-2.753761	-3.634875	0.154168
H	-2.056917	-4.239408	0.716860
C	-6.425681	-1.327639	-1.343889
C	-4.253567	-1.542003	-2.435839
H	-6.687711	-2.330997	0.556056
H	-4.386612	-3.690214	1.510915
C	-5.553718	-1.051305	-2.416708
H	-7.437501	-0.930989	-1.359462
H	-3.582939	-1.304594	-3.256107
C	-6.013525	-2.105507	-0.266987
H	-5.906274	-0.438349	-3.241732
C	2.674018	-3.180608	-0.167662
O	0.365772	-4.299854	0.353635
O	1.225895	-2.486134	1.931922
C	5.193479	-3.331567	-1.388605
C	3.760225	-2.510110	0.400416
C	2.840501	-3.955469	-1.317701
C	4.096704	-4.019839	-1.921754
C	5.007765	-2.593892	-0.209750
H	3.619657	-1.935745	1.308982
H	2.005720	-4.520283	-1.719329
H	4.225323	-4.617403	-2.820966
H	5.850146	-2.063805	0.226757
C	6.542202	-3.356579	-2.066044
H	6.556080	-4.049257	-2.913067
H	7.335351	-3.655568	-1.370485
H	6.799708	-2.358110	-2.440733
H	-1.588276	-3.011746	-2.964051
C	1.694684	0.370303	0.315925
C	2.092740	0.908201	1.546296
C	2.603218	0.477076	-0.750141
C	3.335295	1.532166	1.705438
H	1.422221	0.838890	2.395284
C	3.848697	1.091100	-0.597989
H	2.338643	0.079161	-1.725401
C	4.222744	1.628002	0.635043
H	5.183859	2.114520	0.756824
C	4.825015	1.136664	-1.740992
C	3.676632	2.185445	3.015361
F	5.732641	0.127451	-1.673287

F	5.537687	2.287476	-1.747159
F	4.214358	1.032925	-2.940495
F	5.010847	2.289110	3.204652
F	3.167093	1.513922	4.069511
F	3.176220	3.449257	3.083151
O	-0.575166	-0.058336	1.321577
C	-1.873992	-0.050982	1.376080
C	-2.490355	-0.602293	2.610761
C	-3.885840	-0.727619	2.713059
C	-1.675733	-1.053470	3.659950
C	-4.458294	-1.276719	3.857135
H	-4.519393	-0.407514	1.889366
C	-2.254780	-1.605942	4.801961
H	-0.598136	-1.009801	3.549995
C	-3.642981	-1.711697	4.907820
H	-5.538880	-1.366460	3.931837
H	-1.618218	-1.963786	5.606143
H	-4.090282	-2.139709	5.800989
H	-2.425005	-0.206821	0.447111
C	-2.443517	1.944422	1.623005
H	-1.775797	2.128574	2.457975
H	-3.486634	1.778462	1.859101
C	-2.159871	2.579657	0.415418
C	-3.167489	2.693044	-0.661224
C	-4.473995	3.115157	-0.356152
C	-2.836826	2.374668	-1.990511
C	-5.422934	3.246867	-1.366546
H	-4.731268	3.374773	0.666687
C	-3.799308	2.485945	-2.991438
H	-1.857458	1.974438	-2.225461
C	-5.086598	2.933609	-2.685911
H	-6.423431	3.594320	-1.125204
H	-3.541872	2.207213	-4.008840
H	-5.830386	3.029125	-3.472265
O	-0.929769	2.999513	0.209611
Si	-0.156294	4.318632	-0.615427
C	-1.444806	5.590578	-1.130617
H	-0.926559	6.494934	-1.475133
H	-2.084982	5.241173	-1.945727
H	-2.089316	5.883886	-0.293931
C	0.788270	3.634036	-2.079647
H	1.374850	4.436132	-2.545717
H	1.481169	2.844797	-1.773509
H	0.123521	3.218470	-2.843468
C	0.975047	5.030134	0.700358
H	0.395605	5.476404	1.517180
H	1.633352	4.272592	1.136981
H	1.607876	5.819284	0.275138

TS 45

B3LYP/6-31G(d) = -3573.936509
B3LYP-D3(BJ)/def2-TZVPP/IEFPCM(propanonitrile) = -3575.508014
B3LYP-D3(BJ)/def2-TZVPP/IEFPCM(propanonitrile)//B3LYP-D3(BJ)/6-31G(d) Free Energy (Quasiharmonic) = -3574.791944

Frequencies (Top 3 out of 300)

1. -282.0879 cm^{-1}
2. 8.1749 cm^{-1}
3. 10.2139 cm^{-1}

B3LYP/6-31G(d) Molecular Geometry in Cartesian Coordinates

B	0.729746	0.408094	0.053226
O	0.138321	0.528131	1.414859
N	1.318455	1.828130	-0.138195
C	1.119911	2.656321	1.054781
S	2.303541	2.300363	-1.377169
C	0.341975	1.722146	1.985063
O	-0.052933	2.011821	3.094006
H	1.022373	4.676795	0.323433
H	2.078862	2.879262	1.549023
C	0.366255	4.004602	0.879918
C	-0.971921	3.926815	0.200997
C	-2.278068	3.848404	0.816567
C	-3.239405	3.843944	-0.235322
N	-2.541611	3.915453	-1.422192
C	-1.186775	3.957633	-1.156170
H	-0.460081	4.019826	-1.953807
C	-5.035867	3.739054	1.332670
C	-2.735557	3.799377	2.146489
H	-5.332699	3.791151	-0.810824
H	-2.948282	3.860783	-2.342936
C	-4.103120	3.746134	2.390952
H	-6.099135	3.702483	1.555600
H	-2.025896	3.783193	2.968073
C	-4.616624	3.787866	0.007363
H	-4.462643	3.713160	3.416200
C	3.999974	2.156962	-0.791610
O	2.082251	3.733796	-1.631517
O	2.121244	1.320715	-2.455122
C	6.626371	1.923956	0.165966
C	4.567693	3.203950	-0.060893
C	4.737799	1.003789	-1.070489
C	6.041903	0.899058	-0.591926
C	5.871808	3.077818	0.415565
H	4.007154	4.118565	0.101331
H	4.304993	0.212008	-1.671279
H	6.618867	0.005424	-0.817847
H	6.314056	3.895568	0.979669
C	8.028866	1.780499	0.707454
H	8.488641	2.756119	0.895384
H	8.671904	1.228808	0.013390
H	8.029530	1.229496	1.657377
H	0.253656	4.415455	1.890017
C	1.735748	-0.858555	-0.014029
C	2.355349	-1.341593	1.148548
C	2.034585	-1.517242	-1.217040
C	3.234662	-2.428344	1.116371
H	2.152038	-0.862497	2.101508
C	2.930251	-2.589073	-1.258404
H	1.584077	-1.166733	-2.138448
C	3.533893	-3.057273	-0.091179
H	4.224333	-3.891658	-0.123177
C	3.308155	-3.175727	-2.589236
C	3.809820	-2.957197	2.401122
F	3.840146	-4.414021	-2.474718
F	4.230910	-2.408631	-3.221663
F	2.246091	-3.269793	-3.421989
F	4.080194	-1.966485	3.280240
F	4.956696	-3.645842	2.202364
F	2.949984	-3.802301	3.023650
O	-0.373527	0.171056	-0.962188
C	-1.647639	0.086422	-0.676766
C	-2.568072	0.232402	-1.834914
C	-3.919586	0.540280	-1.621350
C	-2.084183	0.118373	-3.147207
C	-4.777108	0.727179	-2.704629
H	-4.293851	0.658822	-0.608047
C	-2.945777	0.301025	-4.227510
H	-1.028783	-0.076687	-3.305586
C	-4.293195	0.603330	-4.009508
H	-5.821089	0.972155	-2.529691
H	-2.562696	0.219639	-5.241034
H	-4.961927	0.749066	-4.853772
H	-1.979875	0.552796	0.253360
C	-1.810293	-1.778636	-0.013546
H	-1.054524	-1.661416	0.753773
H	-1.463630	-2.290685	-0.902865
C	-3.110376	-2.053847	0.438331

C	-4.174925	-2.607023	-0.413182
C	-3.932079	-2.994665	-1.746316
C	-5.472194	-2.772734	0.112649
C	-4.954156	-3.528299	-2.523942
H	-2.949017	-2.873559	-2.184947
C	-6.489935	-3.309028	-0.668022
H	-5.667526	-2.474996	1.135828
C	-6.234386	-3.688785	-1.988453
H	-4.750816	-3.819830	-3.549949
H	-7.484108	-3.431477	-0.248002
H	-7.029647	-4.109459	-2.597610
O	-3.497588	-1.718980	1.652117
Si	-2.870546	-1.630213	3.282523
C	-2.964426	0.162207	3.799619
H	-2.077564	0.727655	3.492809
H	-3.849764	0.660419	3.389167
H	-3.028808	0.222270	4.893947
C	-1.139757	-2.346979	3.374436
H	-0.878979	-2.485781	4.431750
H	-1.055589	-3.325524	2.888485
H	-0.394993	-1.671917	2.942394
C	-4.101235	-2.704702	4.214082
H	-5.121200	-2.313073	4.126086
H	-4.101100	-3.736251	3.843635
H	-3.850394	-2.735478	5.281566

TS 46

B3LYP/6-31G(d) = -3573.931244
B3LYP-D3(BJ)/def2-TZVPP/IEFPCM(propanonitrile) = -3575.509271
B3LYP-D3(BJ)/def2-TZVPP/IEFPCM(propanonitrile)//B3LYP-D3(BJ)/6-31G(d) Free Energy (Quasiharmonic) = -3574.791895

Frequencies (Top 3 out of 300)

1. -278.1001 cm^{-1}
2. 9.3849 cm^{-1}
3. 10.8861 cm^{-1}

B3LYP/6-31G(d) Molecular Geometry in Cartesian Coordinates

B	-0.717090	-0.188720	0.489730
O	-0.549259	0.008500	1.952950
N	-1.634660	-1.441690	0.466910
C	-1.764591	-2.017490	1.810350
S	-2.615381	-1.956339	-0.751120
C	-1.075680	-0.971810	2.690340
O	-0.991430	-1.022140	3.900260
H	-1.678972	-4.131360	1.402400
H	-2.818761	-2.062739	2.121760
C	-1.142942	-3.423470	2.039580
C	0.338888	-3.527331	1.801070
C	1.016158	-4.015641	0.620550
C	2.413048	-3.929752	0.873220
N	2.569628	-3.431022	2.151070
C	1.324769	-3.178281	2.694370
H	1.229299	-2.773881	3.692860
C	2.915358	-4.794202	-1.290140
C	0.584168	-4.511671	-0.623700
H	4.435638	-4.222443	0.141950
H	3.447069	-3.128453	2.543790
C	1.535038	-4.892312	-1.562630
H	3.633278	-5.096983	-2.048080
H	-0.474782	-4.568060	-0.854570
C	3.373418	-4.311633	-0.069830
H	1.210687	-5.264161	-2.530690
C	-4.302710	-1.506768	-0.303000
O	-2.614772	-3.427009	-0.792520
O	-2.253580	-1.173109	-1.942820
C	-6.928250	-0.801297	0.392800
C	-5.070411	-2.373898	0.478890
C	-4.838940	-0.301718	-0.756950
C	-6.143269	0.041223	-0.407260
C	-6.370471	-2.010737	0.828360
H	-4.663471	-3.332138	0.783410
H	-4.243139	0.343972	-1.390660
H	-6.562449	0.977113	-0.770420
H	-6.965221	-2.685447	1.439640
C	-8.351670	-0.435346	0.741390
H	-9.053900	-0.824245	-0.008020
H	-8.647360	-0.852086	1.709870
H	-8.488829	0.650394	0.782120
H	-1.373462	-3.679970	3.080890
C	0.725980	-0.357441	-0.231340
C	0.882510	-0.714791	-1.580190
C	1.898760	-0.222112	0.530620
C	2.150180	-0.909172	-2.141470
C	3.163610	-0.422493	-0.024020
H	1.815951	0.034078	1.579030
C	3.299700	-0.763173	-1.371140
H	4.278440	-0.920163	-1.808550
C	4.391670	-0.326483	0.831930
C	2.251240	-1.256742	-3.601600
F	4.193611	0.422377	1.948700
F	4.828830	-1.540423	1.267310
F	5.435801	0.228696	0.168920
F	3.505370	-1.614353	-3.960950
F	1.427939	-2.268731	-3.939330
F	1.906730	-0.199412	-4.383910
O	-1.519399	1.030370	0.011420
C	-1.122398	1.926270	-0.842290
C	-2.172978	2.654571	-1.600340
C	-3.431508	2.913611	-1.037900
C	-1.893348	3.094160	-2.902130
C	-4.392337	3.607692	-1.769530
H	-3.647568	2.560001	-0.034750
C	-2.861227	3.780121	-3.635100
H	-0.925228	2.877270	-3.347350
C	-4.109987	4.042632	-3.068390
H	-5.364387	3.810302	-1.328130
H	-2.643117	4.102311	-4.649260
H	-4.863307	4.579952	-3.637670
H	-0.198818	1.724999	-1.387890
C	-0.418557	3.581740	0.208710
H	-0.565197	4.313070	-0.573520
H	-1.186367	3.572660	0.972570
C	0.899342	3.329399	0.609040
C	2.059073	3.539818	-0.277730
C	1.926843	4.142318	-1.545870
C	3.338402	3.131017	0.143360
C	3.039123	4.329148	-2.359090
H	0.957393	4.471379	-1.904010
C	4.447232	3.311007	-0.677820
H	3.448622	2.646987	1.104690
C	4.302493	3.913667	-1.928650
H	2.921123	4.796888	-3.332140
H	5.419892	2.962776	-0.345330
H	5.168213	4.055106	-2.569550
O	1.180482	2.790999	1.776880
Si	0.628422	2.993469	3.420760
C	1.437141	1.583558	4.342580
H	1.552722	1.844418	5.402130
H	0.825451	0.677389	4.279660
H	2.433091	1.365628	3.940890
C	-1.243948	2.981140	3.563660
H	-1.669138	2.126560	3.029860
H	-1.511148	2.871250	4.622640
H	-1.709957	3.904760	3.202600
H	0.001540	-0.875301	-2.195530
C	1.328663	4.673079	3.910180
H	2.422243	4.687458	3.842500

H	0.940634	5.477849	3.274420
H	1.055763	4.911589	4.945750

TS 47

B3LYP/6-31G(d) = -3573.92462
B3LYP-D3(BJ)/def2-TZVPP/IEFPCM(propanonitrile) = -3575.508381
B3LYP-D3(BJ)/def2-TZVPP/IEFPCM(propanonitrile)//B3LYP-D3(BJ)/6-31G(d) Free Energy (Quasiharmonic) = -3574.791877

Frequencies (Top 3 out of 300)

1. -278.1449 cm^{-1}
2. 9.2213 cm^{-1}
3. 13.3336 cm^{-1}

B3LYP/6-31G(d) Molecular Geometry in Cartesian Coordinates

B	0.379087	0.550753	-0.661344
O	0.388998	0.330222	-2.122956
N	0.963746	1.998685	-0.578909
C	1.482301	2.417044	-1.887754
S	0.845168	3.078870	0.641754
C	0.981410	1.304408	-2.816593
O	1.130273	1.292656	-4.019022
H	3.330975	3.378811	-1.334105
H	1.019398	3.360123	-2.211237
C	3.022557	2.578312	-2.011724
C	3.827928	1.333827	-1.749919
C	4.555238	0.986654	-0.549880
C	5.160351	-0.280900	-0.776859
N	4.830597	-0.669710	-2.060316
C	4.015467	0.292465	-2.627827
H	3.629026	0.167019	-3.629958
C	6.100258	-0.286107	1.414681
C	4.747182	1.613883	0.694776
H	6.387595	-1.896764	-0.002514
H	4.954100	-1.601325	-2.425973
C	5.511787	0.971414	1.661465
H	6.688129	-0.764301	2.193631
H	4.276530	2.568725	0.906730
C	5.935301	-0.927139	0.191862
H	5.652255	1.441312	2.631066
C	-0.293112	4.357571	0.065896
O	2.121170	3.776794	0.869066
O	0.213161	2.365842	1.770379
C	-2.078843	6.368164	-0.730524
C	-1.500902	4.009443	-0.542322
C	0.035023	5.697226	0.275655
C	-0.858965	6.690802	-0.122906
C	-2.380689	5.013837	-0.937301
H	-1.739713	2.965660	-0.714933
H	0.984687	5.948354	0.734916
H	-0.599676	7.734896	0.035908
H	-3.317971	4.742062	-1.417572
C	-3.054148	7.447291	-1.137699
H	-3.578727	7.185811	-2.063169
H	-3.818307	7.601286	-0.364020
H	-2.549526	8.406248	-1.292857
H	3.198838	2.931501	-3.035053
C	1.253605	-0.595359	0.089534
C	1.719633	-0.502451	1.414443
C	1.611667	-1.745991	-0.627236
C	2.482528	-1.520267	1.992668
C	2.376584	-2.766930	-0.051980
H	1.302488	-1.830810	-1.664162
C	2.815014	-2.664441	1.265844
H	3.419205	-3.444830	1.712149
C	2.725406	-3.959987	-0.889641
C	2.916468	-1.410276	3.429291
F	3.475145	-3.629495	-1.974666
F	3.407104	-4.903349	-0.211553
F	1.606460	-4.565516	-1.389559
F	3.100741	-0.134050	3.815720
F	1.982764	-1.940927	4.266162
F	4.066602	-2.081875	3.665742
O	-1.103176	0.510665	-0.298677
C	-1.604401	0.217361	0.883903
C	-2.948462	0.793673	1.177993
C	-3.349512	0.969481	2.509006
C	-3.812781	1.181102	0.144261
C	-4.592968	1.525962	2.804339
H	-2.674506	0.691189	3.314857
C	-5.055227	1.740821	0.440236
H	-3.491913	1.055513	-0.885341
C	-5.449749	1.912267	1.770188
H	-4.886347	1.673890	3.839925
H	-5.713056	2.053099	-0.366845
H	-6.414314	2.357046	1.999935
H	-0.911127	0.260785	1.724810
C	-1.821088	-1.700135	1.079828
H	-0.805510	-1.975624	0.820015
H	-2.062727	-1.752067	2.138154
C	-2.842724	-2.120172	0.210632
C	-2.603165	-2.481540	-1.188874
C	-3.542302	-2.137844	-2.182401
C	-1.428364	-3.160455	-1.562639
C	-3.300556	-2.453191	-3.514572
H	-4.431445	-1.580679	-1.907133
C	-1.201370	-3.491111	-2.894992
H	-0.702927	-3.455145	-0.813960
C	-2.132192	-3.133903	-3.872401
H	-4.015004	-2.159562	-4.277979
H	-0.292013	-4.019554	-3.162024
H	-1.945954	-3.378679	-4.914114
O	-4.082774	-2.018746	0.644447
Si	-5.550880	-2.927896	0.706059
C	-6.853548	-1.892740	-0.160122
H	-6.819800	-0.856525	0.194197
H	-6.725974	-1.884770	-1.248015
H	-7.854753	-2.287774	0.051645
C	-5.903998	-3.073182	2.541897
H	-5.956379	-2.082083	3.006111
H	-6.860591	-3.579701	2.719401
H	-5.123911	-3.644822	3.057385
H	1.509245	0.390582	1.994996
C	-5.283295	-4.604943	-0.095606
H	-5.067833	-4.527454	-1.165752
H	-4.459679	-5.153004	0.376330
H	-6.189635	-5.212614	0.020659

TS 48

B3LYP/6-31G(d) = -3573.925387
B3LYP-D3(BJ)/def2-TZVPP/IEFPCM(propanonitrile) = -3575.507842
B3LYP-D3(BJ)/def2-TZVPP/IEFPCM(propanonitrile)//B3LYP-D3(BJ)/6-31G(d) Free Energy (Quasiharmonic) = -3574.791852

Frequencies (Top 3 out of 300)

1. -277.5339 cm^{-1}
2. 10.2594 cm^{-1}
3. 13.0360 cm^{-1}

B3LYP/6-31G(d) Molecular Geometry in Cartesian Coordinates

B	0.423294	0.626516	-0.478013
O	0.421850	0.549212	-1.963323
N	0.886560	2.098881	-0.273021
C	1.210412	2.742883	-1.551431

S	0.828754	2.982799	1.117614
C	0.792369	1.684623	-2.574759
O	0.801273	1.830746	-3.776159
H	2.953351	3.897373	-1.020249
H	0.578154	3.628766	-1.716018
C	2.684107	3.178067	-1.797396
C	3.702989	2.073364	-1.863107
C	4.589111	1.612671	-0.817337
C	5.385543	0.572183	-1.370552
N	5.010325	0.427131	-2.691006
C	3.993644	1.317943	-2.975433
H	3.551161	1.356159	-3.961376
C	6.524711	0.266330	0.699564
C	4.791395	1.976595	0.526938
H	6.933119	-0.920895	-1.063256
H	5.321202	-0.311713	-3.301606
C	5.755502	1.303435	1.266700
H	7.264072	-0.246869	1.309015
H	4.186225	2.754440	0.981598
C	6.350183	-0.114785	-0.625382
H	5.914197	1.573155	2.307480
C	-0.530590	4.153293	0.920451
O	2.035067	3.818569	1.225551
O	0.459100	2.058822	2.196282
C	-2.651496	5.967645	0.622464
C	-0.303592	5.394743	0.319110
C	-1.799647	3.820057	1.394667
C	-2.847988	4.726708	1.243502
C	-1.363576	6.287405	0.169845
H	0.698634	5.668766	0.007465
H	-1.951994	2.871900	1.896988
H	-3.833444	4.466393	1.622185
H	-1.183088	7.255251	-0.292304
C	-3.797005	6.934876	0.437300
H	-4.597939	6.750863	1.160637
H	-4.234160	6.844266	-0.566263
H	-3.467659	7.973154	0.552380
H	2.667266	3.718466	-2.751823
C	1.314989	-0.545024	0.183385
C	1.174913	-0.929398	1.525697
C	2.251358	-1.245400	-0.591392
C	1.939540	-1.965067	2.069142
H	0.476005	-0.397473	2.160563
C	3.020872	-2.279260	-0.050472
H	2.384164	-0.978089	-1.633969
C	2.872308	-2.645119	1.286615
H	3.474950	-3.440105	1.712494
C	3.941868	-3.087627	-0.916065
C	1.698895	-2.423778	3.479178
F	3.362854	-4.272829	-1.282202
F	4.272867	-2.463626	-2.069080
F	5.091417	-3.413924	-0.287998
F	0.790745	-3.444192	3.519630
F	1.210771	-1.448395	4.269428
F	2.822549	-2.900137	4.062108
O	-1.017980	0.392602	0.010489
C	-2.076005	0.715299	-0.687081
C	-3.346933	0.880159	0.069115
C	-3.457154	0.419232	1.389140
C	-4.438898	1.525624	-0.528087
C	-4.642750	0.608012	2.099085
H	-2.599026	-0.054195	1.854274
C	-5.623699	1.710963	0.181809
H	-4.355418	1.894255	-1.548063
C	-5.728710	1.251379	1.498224
H	-4.714893	0.264347	3.127375
H	-6.460306	2.222831	-0.285937
H	-6.648356	1.404967	2.056507
H	-1.930461	1.435211	-1.498312
C	-2.430180	-0.704148	-2.038557
H	-3.125824	-0.192569	-2.698275
H	-1.401428	-0.745520	-2.377135
C	-2.957164	-1.788440	-1.326135
C	-2.123725	-2.759797	-0.616129
C	-0.877983	-3.158020	-1.137006
C	-2.546849	-3.279490	0.624998
C	-0.084394	-4.066711	-0.443429
H	-0.542006	-2.771232	-2.093153
C	-1.735481	-4.161315	1.328548
H	-3.482795	-2.939370	1.054761
C	-0.506835	-4.560267	0.792682
H	0.878095	-4.359696	-0.848364
H	-2.046991	-4.525611	2.302791
H	0.129969	-5.238567	1.351593
O	-4.267292	-1.813975	-1.149005
Si	-5.583146	-2.926010	-1.240404
C	-6.713712	-2.174777	-2.536110
H	-7.626545	-2.771633	-2.653188
H	-7.011494	-1.158813	-2.253834
H	-6.223297	-2.121627	-3.514771
C	-6.396852	-2.929591	0.450476
H	-7.388379	-3.395476	0.394255
H	-6.527643	-1.906282	0.820031
H	-5.813661	-3.485149	1.192720
C	-4.954110	-4.616472	-1.763523
H	-5.806143	-5.292654	-1.908789
H	-4.293255	-5.066659	-1.016551
H	-4.408316	-4.569573	-2.712943

TS 49

B3LYP/6-31G(d) = -3573.931263
B3LYP-D3(BJ)/def2-TZVPP/IEFPCM(propanonitrile) = -3575.509286
B3LYP-D3(BJ)/def2-TZVPP/IEFPCM(propanonitrile)//B3LYP-D3(BJ)/6-31G(d) Free Energy (Quasiharmonic) = -3574.79181

Frequencies (Top 3 out of 300)

1. -278.1865 cm^{-1}
2. 9.8151 cm^{-1}
3. 10.9598 cm^{-1}

B3LYP/6-31G(d) Molecular Geometry in Cartesian Coordinates

B	-0.717610	-0.190340	0.487940
O	-0.552620	0.007310	1.951450
N	-1.632910	-1.444950	0.463690
C	-1.764170	-2.020830	1.806960
S	-2.611440	-1.960750	-0.755600
C	-1.078600	-0.973850	2.688030
O	-0.996410	-1.023860	3.898110
H	-1.674380	-4.134570	1.399400
H	-2.818810	-2.067910	2.116540
C	-1.140510	-3.425710	2.037340
C	0.341840	-3.527050	1.801080
C	1.021660	-4.014120	0.621520
C	2.418040	-3.925820	0.876220
N	2.571900	-3.426900	2.154330
C	1.325800	-3.176320	2.695820
H	1.228190	-2.772120	3.694180
C	2.924960	-4.789220	-1.286480
C	0.592320	-4.510780	-0.623390
H	4.442180	-4.214960	0.147850
H	3.448210	-3.122660	2.548260
C	1.545200	-4.889690	-1.560980
H	3.644490	-5.090670	-2.043420
H	-0.466200	-4.568970	-0.855790
C	3.380420	-4.305960	-0.065470
H	1.222900	-5.262000	-2.529550
C	-4.299280	-1.512741	-0.307690
O	-2.608780	-3.431400	-0.797250

O	-2.249340	-1.176880	-1.946770
C	-6.927900	-0.812811	0.382610
C	-4.838110	-0.310211	-0.765170
C	-5.067720	-2.382681	0.470400
C	-6.370220	-2.023571	0.814840
C	-6.144780	0.028819	-0.420220
H	-4.245050	0.332949	-1.404010
H	-4.661840	-3.343131	0.769280
H	-6.967990	-2.704001	1.416710
H	-6.568400	0.959109	-0.792380
C	-8.332130	-0.421961	0.778390
H	-8.795400	0.225589	0.026590
H	-8.336310	0.127319	1.729520
H	-8.971360	-1.301231	0.909730
H	-1.372210	-3.682510	3.078310
C	0.726940	-0.356800	-0.230660
C	1.898230	-0.218830	0.533100
C	0.886270	-0.714890	-1.578990
C	3.164320	-0.417390	-0.019330
H	1.813270	0.037920	1.581210
C	2.155190	-0.907460	-2.138070
H	0.006590	-0.877450	-2.195630
C	3.303200	-0.758830	-1.365970
H	4.282920	-0.914490	-1.801650
C	2.259410	-1.255700	-3.597810
C	4.390810	-0.318780	0.838580
F	1.917660	-0.198390	-4.381380
F	1.436100	-2.267150	-3.937110
F	3.514110	-1.614450	-3.954010
F	5.435070	0.237820	0.176990
F	4.189620	0.430330	1.954620
F	4.829330	-1.531750	1.275320
O	-1.521210	1.027230	0.008140
C	-1.124601	1.923250	-0.845670
C	-2.175351	2.648950	-1.605980
C	-1.894121	3.088970	-2.907300
C	-3.435611	2.905140	-1.046150
C	-2.862091	3.772580	-3.642320
H	-0.924611	2.874230	-3.350530
C	-4.396581	3.596859	-1.779830
H	-3.652941	2.551040	-0.043460
C	-4.112601	4.032279	-3.078170
H	-2.642681	4.095110	-4.656090
H	-5.370021	3.797159	-1.340440
H	-4.866011	4.567779	-3.649060
H	-0.199821	1.723280	-1.389710
C	-0.425661	3.580550	0.205270
H	-1.194821	3.570560	0.967760
H	-0.572191	4.311220	-0.577600
C	0.891979	3.330800	0.608100
C	2.052939	3.542980	-0.276630
C	3.332259	3.136760	0.147010
C	1.921929	4.144700	-1.545270
C	4.442259	3.318490	-0.672180
H	3.441609	2.653340	1.108750
C	3.035369	4.333270	-2.356500
H	0.952539	4.471800	-1.905340
C	4.298719	3.920340	-1.923540
H	5.414959	2.972220	-0.337750
H	2.918299	4.800370	-3.329970
H	5.165349	4.063130	-2.562900
O	1.171959	2.793380	1.776670
Si	0.616609	2.995380	3.419470
C	1.426539	1.587500	4.343330
H	1.539809	1.849090	5.402950
H	0.816790	0.680070	4.279810
H	2.423609	1.371350	3.943450
C	-1.256001	2.979230	3.558980
H	-1.723241	3.901830	3.196940
H	-1.678401	2.123690	3.024490
H	-1.524901	2.868910	4.617490
C	1.312499	4.676620	3.909460
H	1.037199	4.915030	4.944430
H	2.406169	4.693210	3.843800
H	0.924009	5.480320	3.272630

TS 50

B3LYP/6-31G(d) = -3573.928089
B3LYP-D3(BJ)/def2-TZVPP/IEFPCM(propanonitrile) = -3575.508867
B3LYP-D3(BJ)/def2-TZVPP/IEFPCM(propanonitrile)//B3LYP-D3(BJ)/6-31G(d) Free Energy (Quasiharmonic) = -3574.791783

Frequencies (Top 3 out of 300)

1. -286.7847 cm^{-1}
2. 11.2112 cm^{-1}
3. 13.0681 cm^{-1}

B3LYP/6-31G(d) Molecular Geometry in Cartesian Coordinates

B	0.188258	0.407173	-0.391340
O	-0.003120	-0.152063	-1.755546
N	0.518775	1.888126	-0.730767
C	0.693017	2.065233	-2.177602
S	0.391130	3.202441	0.248669
C	0.231580	0.716551	-2.734198
O	0.113834	0.449441	-3.914707
H	2.406346	3.369998	-2.262001
H	0.014785	2.836615	-2.569708
C	2.130034	2.394820	-2.671201
C	3.174627	1.368790	-2.326038
C	4.126110	1.407783	-1.238567
C	4.893774	0.212650	-1.310380
N	4.442938	-0.501774	-2.402829
C	3.399614	0.187215	-2.992350
H	2.895001	-0.214050	-3.860823
C	6.145695	0.835193	0.620319
C	4.399053	2.326059	-0.208011
H	6.470147	-1.013460	-0.459939
H	4.667674	-1.468063	-2.583090
C	5.400775	2.029598	0.708526
H	6.916546	0.629805	1.358315
H	3.813610	3.235274	-0.112579
C	5.904001	-0.087920	-0.390722
H	5.608257	2.725237	1.516954
C	-1.115086	4.076090	-0.219745
O	1.484132	4.142292	-0.046799
O	0.192523	2.690443	1.614666
C	-3.461680	5.430155	-0.950244
C	-1.066744	5.067054	-1.203251
C	-2.319019	3.765782	0.413612
C	-3.479546	4.442484	0.045155
C	-2.239293	5.728907	-1.566355
H	-0.117388	5.333026	-1.655495
H	-2.336940	3.016577	1.195823
H	-4.414901	4.203903	0.546738
H	-2.200071	6.498634	-2.333590
C	-4.719092	6.181174	-1.319876
H	-5.612312	5.563488	-1.177999
H	-4.696692	6.512365	-2.363357
H	-4.841252	7.077176	-0.696746
O	-1.192542	0.310559	0.259783
C	-1.449491	-0.247543	1.413504
H	-0.604555	-0.371998	2.095372
H	2.052414	2.505475	-3.759903
C	-2.728193	0.130371	2.073479
C	-2.863505	-0.040156	3.458573
C	-3.794292	0.670027	1.339874
C	-4.045034	0.322796	4.102480
H	-2.032791	-0.441341	4.035139

C	-4.976030	1.030209	1.985685
H	-3.675500	0.822254	0.272337
C	-5.105290	0.856340	3.366145
H	-4.134431	0.199198	5.178058
H	-5.797685	1.449785	1.411366
H	-6.025911	1.142437	3.867382
C	1.340890	-0.418679	0.401310
C	1.966806	0.044291	1.573246
C	1.791606	-1.643125	-0.116779
C	2.981436	-0.688181	2.195337
H	1.675119	1.003313	1.989977
C	2.809884	-2.376505	0.500835
H	1.351638	-2.019027	-1.033609
C	3.411324	-1.904318	1.665681
H	4.208009	-2.462195	2.143047
C	3.305999	-3.638522	-0.141233
C	3.592171	-0.182818	3.474511
F	2.300532	-4.353871	-0.706660
F	4.199946	-3.392717	-1.141912
F	3.625176	1.161893	3.531331
F	3.931137	-4.454426	0.733536
F	4.857293	-0.631618	3.645073
F	2.884932	-0.603171	4.558014
C	-1.563971	-2.229842	1.196942
H	-1.848161	-2.509730	2.205102
H	-0.520124	-2.377976	0.938738
C	-2.489485	-2.468796	0.170266
O	-2.081790	-2.297824	-1.060807
Si	-2.106482	-3.008401	-2.647602
C	-3.136237	-4.583007	-2.548585
H	-3.090304	-5.100994	-3.514937
H	-2.745325	-5.273422	-1.791748
H	-4.190163	-4.394717	-2.321776
C	-2.819814	-1.725559	-3.810198
H	-2.137011	-0.872256	-3.886827
H	-2.915578	-2.160638	-4.813567
H	-3.810902	-1.364407	-3.514610
C	-0.324083	-3.380251	-3.075871
H	-0.275780	-3.973897	-3.997778
H	0.210134	-2.440723	-3.248407
H	0.195890	-3.939213	-2.290662
C	-3.918687	-2.723146	0.426200
C	-4.335449	-3.408819	1.582057
C	-4.892614	-2.265824	-0.481285
C	-5.688163	-3.637660	1.816958
H	-3.598059	-3.793050	2.279054
C	-6.244574	-2.484443	-0.236137
H	-4.582757	-1.709103	-1.358626
C	-6.645253	-3.174120	0.911263
H	-5.995983	-4.181387	2.705187
H	-6.986985	-2.113671	-0.936903
H	-7.700697	-3.350655	1.098541

TS 51

B3LYP/6-31G(d) = -3573.924635
B3LYP-D3(BJ)/def2-TZVPP/IEFPCM(propanonitrile) = -3575.508386
B3LYP-D3(BJ)/def2-TZVPP/IEFPCM(propanonitrile)//B3LYP-D3(BJ)/6-31G(d) Free Energy (Quasiharmonic) = -3574.791776

Frequencies (Top 3 out of 300)

1. -278.1634 cm^{-1}
2. 9.2065 cm^{-1}
3. 13.3529 cm^{-1}

B3LYP/6-31G(d) Molecular Geometry in Cartesian Coordinates

B	0.379238	0.551194	-0.660411
O	0.388827	0.332413	-2.122312
N	0.965415	1.998438	-0.576310
C	1.484410	2.417738	-1.884684
S	0.848453	3.077193	0.645783
C	0.982153	1.306816	-2.814820
O	1.130787	1.296453	-4.017300
H	3.334237	3.376679	-1.329998
H	1.022590	3.361734	-2.207000
C	3.024845	2.577377	-2.008584
C	3.828841	1.331656	-1.748436
C	4.555972	0.982200	-0.548951
C	5.159630	-0.285748	-0.777593
N	4.829224	-0.672612	-2.061472
C	4.015066	0.291165	-2.627655
H	3.628311	0.167373	-3.629872
C	6.099895	-0.294705	1.413780
C	4.748819	1.607681	0.696445
H	6.385199	-1.903933	-0.005446
H	4.951588	-1.603927	-2.428277
C	5.512866	0.963165	1.662213
H	6.687357	-0.774515	2.192043
H	4.279259	2.562781	0.909656
C	5.934021	-0.934044	0.190199
H	5.654011	1.431707	2.632372
C	-0.287870	4.357945	0.070699
O	0.216075	2.363401	1.773713
O	2.125292	3.773441	0.873645
C	-2.073683	6.371358	-0.718085
C	0.038524	5.696838	0.289041
C	-1.496128	4.012004	-0.537468
C	-2.377185	5.017852	-0.926424
C	-0.856515	6.691636	-0.103381
H	0.984689	5.945990	0.756542
H	-1.737902	2.968557	-0.707998
H	-3.318370	4.747569	-1.399823
H	-0.601823	7.734805	0.068629
C	-3.022225	7.454951	-1.173658
H	-4.066751	7.136854	-1.086209
H	-2.849132	7.714392	-2.226670
H	-2.895520	8.371045	-0.587718
O	-1.103026	0.512171	-0.297755
C	-1.604616	0.218511	0.884588
H	-0.911372	0.260720	1.725578
H	3.201431	2.931641	-3.031493
C	-2.948155	0.795921	1.178877
C	-3.349113	0.971557	2.509935
C	-3.812017	1.184651	0.145238
C	-4.592018	1.529196	2.805418
H	-2.674430	0.692272	3.315712
C	-5.053923	1.745479	0.441377
H	-3.491195	1.059196	-0.884397
C	-5.448342	1.916797	1.771381
H	-4.885289	1.677061	3.841040
H	-5.711422	2.058671	-0.365623
H	-6.412437	2.362513	2.001269
C	1.252599	-0.596760	0.088995
C	1.718902	-0.505960	1.413955
C	1.609302	-1.746922	-0.629208
C	2.480786	-1.525294	1.990837
H	1.509551	0.386591	1.995621
C	2.373201	-2.769377	-0.055292
H	1.299895	-1.830153	-1.666196
C	2.811938	-2.668948	1.262587
H	3.415367	-3.450516	1.707858
C	2.720543	-3.961835	-0.894419
C	2.915072	-1.417508	3.427524
F	3.469925	-3.630804	-1.979527
F	3.401843	-4.906488	-0.217732
F	1.981009	-1.948312	4.263891
F	1.600804	-4.565902	-1.394335
F	3.100638	-0.141943	3.815510
F	4.064606	-2.090503	3.662935

C	-1.823067	-1.698967	1.079175
H	-2.064382	-1.751425	2.137550
H	-0.807834	-1.975206	0.818817
C	-2.845413	-2.117402	0.210052
C	-2.606747	-2.477929	-1.189829
C	-3.546067	-2.132784	-2.182683
C	-1.432641	-3.157479	-1.564623
C	-3.305173	-2.447360	-3.515193
H	-4.434654	-1.575132	-1.906608
C	-1.206505	-3.487342	-2.897319
H	-0.707093	-3.453273	-0.816487
C	-2.137499	-3.128716	-3.874045
H	-4.019750	-2.152637	-4.278059
H	-0.297682	-4.016301	-3.165142
H	-1.951931	-3.372899	-4.916017
O	-4.085206	-2.015149	0.644405
Si	-5.554117	-2.922965	0.706151
C	-5.906283	-3.069559	2.542067
H	-6.863221	-3.575389	2.719681
H	-5.126404	-3.642350	3.056591
H	-5.957520	-2.078829	3.007197
C	-6.856252	-1.885751	-0.158359
H	-6.729304	-1.876979	-1.246321
H	-7.857740	-2.279929	0.053660
H	-6.821211	-0.849872	0.196819
C	-5.288656	-4.599573	-0.097147
H	-6.195517	-5.206459	0.019155
H	-5.073784	-4.521367	-1.167359
H	-4.465283	-5.148851	0.373800

TS 52

B3LYP/6-31G(d) = -3573.928099
B3LYP-D3(BJ)/def2-TZVPP/IEFPCM(propanonitrile) = -3575.508863
B3LYP-D3(BJ)/def2-TZVPP/IEFPCM(propanonitrile)//B3LYP-D3(BJ)/6-31G(d) Free Energy (Quasiharmonic) = -3574.791765

Frequencies (Top 3 out of 300)

1. -286.8096 cm^{-1}
2. 11.1610 cm^{-1}
3. 13.0282 cm^{-1}

B3LYP/6-31G(d) Molecular Geometry in Cartesian Coordinates

B	0.188689	0.408306	-0.390357
O	-0.003840	-0.148655	-1.755357
N	0.519826	1.889614	-0.727608
C	0.692998	2.069028	-2.174282
S	0.392294	3.202510	0.253759
C	0.230465	0.721488	-2.732748
O	0.111535	0.456422	-3.913606
H	2.406688	3.373293	-2.257879
H	0.014848	2.841409	-2.564578
C	2.129780	2.398823	-2.668368
C	3.174199	1.371903	-2.325388
C	4.126243	1.408904	-1.238346
C	4.893478	0.213637	-1.312396
N	4.441837	-0.498964	-2.405707
C	3.398433	0.191277	-2.993626
H	2.893239	-0.208486	-3.862456
C	6.146572	0.832806	0.618624
C	4.399976	2.325496	-0.206505
H	6.469901	-1.014277	-0.464653
H	4.666128	-1.465062	-2.587536
C	5.402066	2.027303	0.709068
H	6.917731	0.626036	1.355912
H	3.814874	3.234755	-0.109374
C	5.904078	-0.088671	-0.393718
H	5.610175	2.721621	1.518468
C	-1.114638	4.075142	-0.214374
O	1.484996	4.142976	-0.040793
O	0.194286	2.688622	1.619119
C	-3.461219	5.431882	-0.940425
C	-2.317612	3.766399	0.421469
C	-1.066125	5.069805	-1.194193
C	-2.238084	5.734898	-1.553134
C	-3.477660	4.446157	0.056914
H	-2.333704	3.021386	1.207720
H	-0.115776	5.341084	-1.641165
H	-2.197222	6.512716	-2.312047
H	-4.410673	4.214068	0.565766
C	-4.729908	6.141722	-1.350456
H	-5.441815	6.202428	-0.520530
H	-4.525652	7.159853	-1.697790
H	-5.231194	5.612902	-2.172392
H	2.051499	2.511158	-3.756851
C	1.341476	-0.419253	0.400254
C	1.968244	0.041724	1.572523
C	1.791435	-1.643069	-0.119959
C	2.982951	-0.692066	2.192925
C	2.809815	-2.377749	0.495946
H	1.350794	-2.017422	-1.037100
C	3.412091	-1.907551	1.661163
H	4.208848	-2.466441	2.137222
C	3.305133	-3.638947	-0.148345
C	3.594594	-0.188904	3.472535
F	3.930512	-4.456396	0.724802
F	2.299095	-4.353093	-0.714278
F	4.198571	-3.391904	-1.149176
F	4.859719	-0.638262	3.641579
F	3.627930	1.155703	3.531508
F	2.887933	-0.610863	4.555787
O	-1.191679	0.311397	0.261519
C	-1.448023	-0.248041	1.414774
C	-2.725943	0.129811	2.076328
C	-2.860418	-0.043075	3.461219
C	-3.792077	0.671737	1.344475
C	-4.041172	0.319729	4.106617
H	-2.029637	-0.445992	4.036483
C	-4.973040	1.031816	1.991765
H	-3.673857	0.825920	0.277164
C	-5.101486	0.855528	3.371991
H	-4.129911	0.194280	5.182035
H	-5.794657	1.453295	1.418787
H	-6.021497	1.141529	3.874405
H	-0.602600	-0.373691	2.095823
C	-1.563630	-2.229691	1.195749
H	-1.847048	-2.510888	2.203763
H	-0.520102	-2.378091	0.936398
C	-2.490195	-2.466807	0.169572
C	-3.919286	-2.720781	0.426416
C	-4.893775	-2.261793	-0.479627
C	-4.335388	-3.407742	1.581749
C	-6.245627	-2.480049	-0.233585
H	-4.584389	-1.704086	-1.356508
C	-5.688011	-3.636211	1.817528
H	-3.597590	-3.793243	2.277611
C	-6.645654	-3.171015	0.913269
H	-6.988471	-2.107987	-0.933208
H	-5.995332	-4.180929	2.705321
H	-7.701023	-3.347254	1.101241
O	-2.083527	-2.294281	-1.061626
Si	-2.109550	-3.002824	-2.649283
C	-3.140111	-4.576982	-2.551670
H	-3.095152	-5.093704	-3.518744
H	-2.749048	-5.268626	-1.796035
H	-4.193770	-4.388410	-2.323853
C	-0.327647	-3.375037	-3.079263
H	-0.280277	-3.967394	-4.002046
H	0.206970	-2.435555	-3.250805

H	0.192551	-3.935398	-2.295202
H	1.677158	1.000226	1.990880
C	-2.822970	-1.718036	-3.809697
H	-3.813646	-1.356753	-3.512881
H	-2.139725	-0.865024	-3.885659
H	-2.919704	-2.151718	-4.813577

TS 53

B3LYP/6-31G(d) = -3573.933565
B3LYP-D3(BJ)/def2-TZVPP/IEFPCM(propanonitrile) = -3575.508344
B3LYP-D3(BJ)/def2-TZVPP/IEFPCM(propanonitrile)//B3LYP-D3(BJ)/6-31G(d) Free Energy (Quasiharmonic) = -3574.791754

Frequencies (Top 3 out of 300)

1. -267.7265 cm^{-1}
2. 11.0235 cm^{-1}
3. 12.0894 cm^{-1}

B3LYP/6-31G(d) Molecular Geometry in Cartesian Coordinates

B	0.267613	-0.347854	0.103993
O	-0.421835	0.343826	-1.019877
N	0.293673	-1.805817	-0.436462
C	-0.170470	-1.868906	-1.826768
S	1.161105	-3.039929	0.246699
C	-0.652375	-0.436704	-2.077231
O	-1.171721	-0.036060	-3.099768
H	-0.804139	-3.897470	-2.139715
H	0.669106	-2.044619	-2.519645
C	-1.268457	-2.909615	-2.175138
C	-2.496536	-2.904182	-1.309541
C	-3.760308	-2.249834	-1.564348
C	-4.636990	-2.597852	-0.496132
N	-3.931597	-3.422850	0.354630
C	-2.650413	-3.594727	-0.130460
H	-1.930931	-4.211856	0.388681
C	-6.410245	-1.330095	-1.466830
C	-4.248234	-1.424914	-2.594723
H	-6.613912	-2.426440	0.388042
H	-4.261495	-3.763689	1.244157
C	-5.562615	-0.977322	-2.537091
H	-7.433924	-0.965224	-1.451178
H	-3.596746	-1.129798	-3.411996
C	-5.959141	-2.143307	-0.432725
H	-5.947447	-0.341715	-3.330213
C	2.777225	-3.048904	-0.543397
O	0.521064	-4.315641	-0.115883
O	1.353756	-2.677838	1.656573
C	5.257535	-2.925723	-1.845939
C	2.944286	-3.683459	-1.776380
C	3.846499	-2.385392	0.064521
C	5.073988	-2.331222	-0.588494
C	4.180728	-3.612910	-2.419373
H	2.126044	-4.245822	-2.213903
H	3.711796	-1.924297	1.036273
H	5.903215	-1.808308	-0.118658
H	4.309328	-4.102584	-3.381613
C	6.582333	-2.804982	-2.559625
H	6.606508	-3.410140	-3.471035
H	7.413521	-3.123196	-1.919274
H	6.772850	-1.761917	-2.842124
H	-1.542223	-2.713922	-3.218603
C	1.689064	0.347495	0.434715
C	2.135962	0.582388	1.742977
C	2.538013	0.743307	-0.608498
C	3.370793	1.186500	1.997377
H	1.508501	0.288887	2.576642
C	3.771641	1.353806	-0.363676
H	2.232713	0.584071	-1.638735
C	4.198704	1.578999	0.944743
H	5.149453	2.060577	1.140482
C	4.661501	1.716680	-1.518804
C	3.838106	1.358034	3.416980
F	3.948610	2.178128	-2.576372
F	5.369095	0.650624	-1.969981
F	5.560907	2.671557	-1.194197
F	4.732698	2.366251	3.539515
F	4.443376	0.239505	3.881862
F	2.813305	1.625122	4.258081
O	-0.572459	-0.256747	1.357286
C	-1.866129	-0.130972	1.401901
C	-2.550649	-0.766585	2.559218
C	-1.799720	-1.383162	3.570313
C	-3.953430	-0.803864	2.615778
C	-2.447921	-2.014283	4.631752
H	-0.718311	-1.399558	3.491566
C	-4.595483	-1.433062	3.679091
H	-4.538692	-0.352047	1.818399
C	-3.842846	-2.035191	4.693427
H	-1.861017	-2.498438	5.407131
H	-5.681328	-1.456354	3.718000
H	-4.344151	-2.525544	5.523640
H	-2.412174	-0.144658	0.457518
C	-2.230166	1.860151	1.852201
H	-1.441059	1.950591	2.591665
H	-3.239501	1.755517	2.228773
C	-2.080755	2.558654	0.652271
C	-3.229917	2.762552	-0.259767
C	-3.133142	2.439501	-1.624798
C	-4.439938	3.268631	0.247662
C	-4.230141	2.633320	-2.462172
H	-2.232440	1.979405	-2.016709
C	-5.523010	3.481186	-0.601567
H	-4.515773	3.528264	1.299685
C	-5.419243	3.164608	-1.958228
H	-4.154242	2.355973	-3.509183
H	-6.446549	3.893608	-0.205314
H	-6.267488	3.322582	-2.618545
O	-0.879261	2.953532	0.301285
Si	-0.171287	4.202000	-0.685981
C	-1.445560	5.559332	-0.965694
H	-0.936275	6.437925	-1.382579
H	-2.231309	5.265072	-1.667492
H	-1.924217	5.872529	-0.030798
C	0.469326	3.488115	-2.298264
H	0.570104	4.298739	-3.031835
H	1.458156	3.038552	-2.166921
H	-0.186869	2.725420	-2.726878
C	1.227544	4.807945	0.406147
H	0.853050	5.253514	1.334637
H	1.899326	3.984889	0.672885
H	1.821531	5.568809	-0.115386

TS 54

B3LYP/6-31G(d) = -3573.931139
B3LYP-D3(BJ)/def2-TZVPP/IEFPCM(propanonitrile) = -3575.509344
B3LYP-D3(BJ)/def2-TZVPP/IEFPCM(propanonitrile)//B3LYP-D3(BJ)/6-31G(d) Free Energy (Quasiharmonic) = -3574.791609

Frequencies (Top 3 out of 300)

1. -280.4282 cm^{-1}
2. 9.4728 cm^{-1}
3. 16.4479 cm^{-1}

B3LYP/6-31G(d) Molecular Geometry in Cartesian Coordinates

B	0.706672	-0.140213	-0.523283
O	0.573477	0.120219	-1.981980
N	1.691612	-1.343642	-0.538458
C	1.843429	-1.878561	-1.897241
S	2.705519	-1.821436	0.657953
C	1.123510	-0.826947	-2.747557
O	1.043015	-0.841824	-3.958390
H	1.841591	-3.998673	-1.538687
H	2.899474	-1.882405	-2.203791
C	1.276944	-3.299810	-2.161490
C	-0.199504	-3.467029	-1.929148
C	-0.855269	-4.014313	-0.763104
C	-2.255046	-3.973747	-1.011782
N	-2.433094	-3.445993	-2.275144
C	-1.199355	-3.128753	-2.810728
H	-1.121287	-2.690391	-3.796352
C	-2.719313	-4.909460	1.130676
C	-0.401489	-4.525867	0.466741
H	-4.263389	-4.360108	-0.285184
H	-3.320635	-3.151453	-2.651435
C	-1.335450	-4.964519	1.397683
H	-3.423501	-5.253164	1.883769
H	0.659635	-4.548765	0.695002
C	-3.197871	-4.414912	-0.077213
H	-0.995338	-5.347561	2.355871
C	4.357717	-1.283855	0.150820
O	2.793922	-3.290295	0.708492
O	2.314718	-1.082723	1.869267
C	6.962496	-0.477014	-0.525129
C	4.553942	-0.113704	-0.583535
C	5.451083	-2.058876	0.545789
C	6.739878	-1.647738	0.214009
C	5.850417	0.278612	-0.918504
H	3.700536	0.478446	-0.896078
H	5.284732	-2.977862	1.097313
H	7.588096	-2.251996	0.527531
H	5.999376	1.185019	-1.501146
C	8.362377	-0.063409	-0.913174
H	9.074285	-0.249466	-0.101536
H	8.713592	-0.628603	-1.786576
H	8.410629	0.999524	-1.170642
H	1.520705	-3.524943	-3.206993
C	-0.744044	-0.418034	0.143621
C	-0.909289	-0.901771	1.452638
C	-1.906109	-0.239402	-0.622684
C	-2.178191	-1.179103	1.971443
C	-3.174235	-0.519806	-0.109175
H	-1.813595	0.112682	-1.642551
C	-3.320137	-0.988334	1.196783
H	-4.300886	-1.214530	1.599037
C	-4.390177	-0.382133	-0.975625
C	-2.314557	-1.649243	3.394202
F	-4.190821	0.456005	-2.026278
F	-4.791719	-1.568695	-1.510979
F	-5.457563	0.096636	-0.290109
F	-3.464287	-2.333525	3.596452
F	-1.295081	-2.444820	3.769707
F	-2.331560	-0.601000	4.260984
O	1.427329	1.079617	0.058850
C	0.958771	1.871919	0.982681
C	1.916073	2.458861	1.953627
C	3.281271	2.584508	1.664447
C	1.433288	2.878697	3.203281
C	4.147396	3.126382	2.610805
H	3.660117	2.236950	0.710606
C	2.302654	3.417942	4.148508
H	0.378096	2.761319	3.439222
C	3.661664	3.547020	3.851792
H	5.206452	3.211200	2.384235
H	1.923513	3.725725	5.118873
H	4.341810	3.963949	4.589381
H	-0.018297	1.621339	1.397566
C	0.392784	3.629194	0.048986
H	0.506051	4.294523	0.892990
H	1.206274	3.655773	-0.666020
C	-0.903082	3.421304	-0.439707
C	-2.109047	3.593337	0.392196
C	-2.067114	4.251937	1.638389
C	-3.340747	3.082894	-0.059475
C	-3.220102	4.388759	2.403906
H	-1.139576	4.673118	2.010668
C	-4.488596	3.208629	0.717030
H	-3.382374	2.562948	-1.007275
C	-4.433111	3.864022	1.948439
H	-3.173386	4.905032	3.358298
H	-5.419938	2.776457	0.365122
H	-5.329613	3.964546	2.553752
O	-1.122370	2.932389	-1.642596
Si	-0.514233	3.233967	-3.249823
C	-1.301385	1.895784	-4.289497
H	-1.362399	2.220831	-5.335795
H	-0.711737	0.973786	-4.253218
H	-2.319581	1.675598	-3.948906
C	1.362172	3.207224	-3.318075
H	1.675347	3.167577	-4.369306
H	1.821928	4.096141	-2.871990
H	1.751364	2.311636	-2.825727
H	-0.032982	-1.090915	2.066367
C	-1.178534	4.949599	-3.656265
H	-2.273609	4.973187	-3.625875
H	-0.805105	5.707790	-2.957615
H	-0.866541	5.249516	-4.664455

TS 55

B3LYP/6-31G(d) = -3573.931239
B3LYP-D3(BJ)/def2-TZVPP/IEFPCM(propanonitrile) = -3575.507875
B3LYP-D3(BJ)/def2-TZVPP/IEFPCM(propanonitrile)//B3LYP-D3(BJ)/6-31G(d) Free Energy (Quasiharmonic) = -3574.79157

Frequencies (Top 3 out of 300)

1. -276.6190 cm^{-1}
2. 8.1600 cm^{-1}
3. 10.9016 cm^{-1}

B3LYP/6-31G(d) Molecular Geometry in Cartesian Coordinates

B	0.454882	0.384553	-0.293289
O	0.046588	-0.180412	-1.615193
N	0.772054	1.851846	-0.677332
C	0.780605	2.008912	-2.138027
S	0.655848	3.196497	0.265862
C	0.208954	0.677892	-2.628697
O	-0.070409	0.415828	-3.780355
H	2.473234	3.295141	-2.453201
H	0.081406	2.794791	-2.458548
C	2.158001	2.304408	-2.791466
C	3.226026	1.287874	-2.501529
C	4.243864	1.352881	-1.477946
C	5.005608	0.156395	-1.569578
N	4.489810	-0.582934	-2.616304
C	3.408951	0.091087	-3.153050
H	2.852439	-0.330308	-3.979666
C	6.369256	0.820146	0.268708
C	4.577437	2.294925	-0.487703
H	6.622693	-1.057067	-0.781463
H	4.698702	-1.556397	-2.776974
C	5.632444	2.018450	0.373294
H	7.181509	0.629585	0.965130
H	4.000035	3.208238	-0.379528

C	6.067546	-0.125920	-0.703927
H	5.889259	2.732590	1.150917
C	-0.969159	3.920642	-0.056759
O	1.621412	4.196139	-0.215188
O	0.664884	2.753596	1.666425
C	-3.492129	5.041473	-0.578718
C	-2.090168	3.464707	0.641917
C	-1.095510	4.939570	-1.002335
C	-2.353689	5.485431	-1.261574
C	-3.337300	4.025920	0.377876
H	-1.975928	2.690123	1.390859
H	-0.211963	5.313663	-1.508110
H	-2.447550	6.277693	-2.000455
H	-4.207590	3.681275	0.933120
C	-4.844373	5.664188	-0.833510
H	-5.051332	6.466317	-0.112669
H	-5.650379	4.928338	-0.738827
H	-4.901213	6.103331	-1.834601
H	1.972822	2.370013	-3.870890
C	1.642496	-0.488594	0.369694
C	2.071561	-1.685757	-0.218940
C	2.293144	-0.079789	1.546290
C	3.108413	-2.444334	0.335121
H	1.607181	-2.024844	-1.138665
C	3.327983	-0.834370	2.101625
H	2.004523	0.854996	2.016752
C	3.742767	-2.023718	1.501829
H	4.554717	-2.600846	1.928759
C	3.983402	-0.384026	3.378812
C	3.605668	-3.666429	-0.377878
F	3.329721	-0.860779	4.470233
F	4.007165	0.958109	3.495439
F	5.260767	-0.823834	3.474452
F	4.604497	-3.373284	-1.267322
F	2.634843	-4.276011	-1.096285
F	4.120817	-4.585174	0.467248
O	-0.758894	0.351768	0.633469
C	-1.522160	-0.700495	0.762233
C	-2.149953	-0.891910	2.092565
C	-2.014097	0.091506	3.085681
C	-2.803136	-2.095988	2.398982
C	-2.544932	-0.123954	4.356540
H	-1.446544	0.990202	2.867169
C	-3.325154	-2.309643	3.671816
H	-2.893861	-2.869622	1.640100
C	-3.203106	-1.320204	4.651406
H	-2.424887	0.635373	5.124074
H	-3.821651	-3.247941	3.902079
H	-3.606890	-1.488531	5.646022
H	-1.197575	-1.616961	0.262101
C	-2.979957	-0.307325	-0.591440
H	-3.233325	0.678606	-0.221602
H	-2.283331	-0.305799	-1.421592
C	-3.988436	-1.278748	-0.613177
C	-5.202112	-1.218563	0.215717
C	-5.433200	-0.158358	1.115052
C	-6.170699	-2.236976	0.109105
C	-6.594232	-0.121129	1.879791
H	-4.701160	0.631576	1.232809
C	-7.332371	-2.192281	0.871811
H	-5.999587	-3.056657	-0.578435
C	-7.547979	-1.134828	1.759667
H	-6.754494	0.699294	2.572890
H	-8.071232	-2.982573	0.775556
H	-8.455692	-1.100735	2.355714
O	-3.874716	-2.383855	-1.328171
Si	-3.021896	-2.888261	-2.771011
C	-3.054132	-1.498709	-4.030531
H	-2.203734	-0.815402	-3.928201
H	-2.995515	-1.935564	-5.035883
H	-3.984889	-0.921331	-3.983617
C	-1.289407	-3.407216	-2.286920
H	-0.813476	-3.923447	-3.130488
H	-0.668448	-2.538303	-2.046812
H	-1.286133	-4.098792	-1.436690
C	-4.071877	-4.352417	-3.298762
H	-4.125782	-5.111570	-2.510184
H	-3.644330	-4.829546	-4.188969
H	-5.094871	-4.046485	-3.545885

TS 56

B3LYP/6-31G(d) = -3573.929277
B3LYP-D3(BJ)/def2-TZVPP/IEFPCM(propanonitrile) = -3575.508756
B3LYP-D3(BJ)/def2-TZVPP/IEFPCM(propanonitrile)//B3LYP-D3(BJ)/6-31G(d) Free Energy (Quasiharmonic) = -3574.791557

Frequencies (Top 3 out of 300)

1. -290.0545 cm^{-1}
2. 10.3484 cm^{-1}
3. 14.7773 cm^{-1}

B3LYP/6-31G(d) Molecular Geometry in Cartesian Coordinates

B	0.675338	-0.506095	-0.575188
O	0.411160	-1.267732	-1.804174
N	1.772039	-1.388359	0.092205
C	1.951891	-2.643034	-0.648329
S	2.702200	-1.023885	1.389128
C	1.108318	-2.405030	-1.904564
O	1.064825	-3.149043	-2.857703
H	2.246410	-4.113452	0.896411
H	2.995567	-2.761888	-0.971004
C	1.536283	-3.952813	0.079974
C	0.133362	-3.989358	0.614601
C	-1.063335	-4.444081	-0.057397
C	-2.143382	-4.285384	0.856744
N	-1.616688	-3.764111	2.020962
C	-0.256437	-3.584185	1.868199
H	0.346881	-3.170001	2.663695
C	-3.692068	-5.149329	-0.736452
C	-1.332871	-4.973386	-1.333034
H	-4.272545	-4.482677	1.238009
H	-2.146277	-3.504633	2.838169
C	-2.638368	-5.321754	-1.657211
H	-4.704056	-5.423338	-1.022902
H	-0.535279	-5.081108	-2.061908
C	-3.459646	-4.629491	0.531722
H	-2.855030	-5.726746	-2.642329
C	4.400151	-1.137607	0.796558
O	2.587303	-2.028635	2.460728
O	2.412907	0.387367	1.719810
C	7.067968	-1.248473	-0.055781
C	5.324487	-1.890577	1.519015
C	4.789872	-0.438925	-0.349082
C	6.115386	-0.501360	-0.767057
C	6.650698	-1.940348	1.087270
H	4.999798	-2.430971	2.401300
H	4.060293	0.136519	-0.908547
H	6.417544	0.038197	-1.661756
H	7.370971	-2.531129	1.647973
C	8.507731	-1.288856	-0.510203
H	8.581626	-1.431948	-1.594207
H	9.059344	-2.098889	-0.023291
H	9.023356	-0.349013	-0.272848
O	1.333574	0.802428	-1.094296
C	1.323237	1.965530	-0.515152
H	0.789631	2.058583	0.429080
H	1.690784	-4.760580	-0.645787
C	2.550454	2.796042	-0.653538

C	2.917157	3.659043	0.387513
C	3.355965	2.715279	-1.799135
C	4.072282	4.433555	0.283857
H	2.315161	3.696738	1.291320
C	4.504922	3.495467	-1.903348
H	3.076268	2.035347	-2.598724
C	4.864603	4.358042	-0.863090
H	4.358016	5.087932	1.102523
H	5.122569	3.431202	-2.795052
H	5.762889	4.963732	-0.944796
C	-0.688032	-0.265812	0.274595
C	-1.910710	-0.693379	-0.275261
C	-0.733786	0.295206	1.562458
C	-3.115679	-0.555392	0.415417
H	-1.914309	-1.155982	-1.254918
C	-1.943723	0.452807	2.249105
H	0.187790	0.594970	2.050934
C	-3.143179	0.026147	1.682914
H	-4.080324	0.142297	2.217273
C	-1.955722	1.031545	3.634250
C	-4.420937	-0.980993	-0.198122
F	-0.914371	1.873684	3.847198
F	-3.090897	1.743535	3.871576
F	-5.170100	-1.702960	0.670312
F	-1.892366	0.083240	4.595023
F	-4.267761	-1.713506	-1.313863
F	-5.183239	0.100523	-0.537099
C	0.073487	3.266684	-1.555832
H	0.604993	3.208050	-2.496891
H	0.298647	4.151015	-0.971470
C	-1.252207	2.815870	-1.542964
O	-2.103202	3.182721	-0.595357
Si	-2.271595	4.562506	0.439275
C	-2.356039	6.066425	-0.688366
H	-3.196680	5.990869	-1.387261
H	-2.498119	6.978061	-0.095105
H	-1.440949	6.195932	-1.276946
C	-3.889553	4.226058	1.317043
H	-3.795698	3.382396	2.008484
H	-4.196297	5.102075	1.901852
H	-4.693220	3.999612	0.607904
C	-0.855050	4.685565	1.668082
H	0.108408	4.917325	1.202031
H	-1.074528	5.495890	2.375811
H	-0.747346	3.767627	2.255168
C	-1.793085	1.875033	-2.526941
C	-0.955616	1.215366	-3.449093
C	-3.177866	1.610788	-2.553526
C	-1.490658	0.318660	-4.367632
H	0.117210	1.359510	-3.419415
C	-3.707496	0.724440	-3.482474
H	-3.823955	2.084623	-1.825457
C	-2.865790	0.077692	-4.393033
H	-0.830232	-0.209553	-5.047776
H	-4.772900	0.517352	-3.479131
H	-3.279474	-0.626674	-5.109151

TS 57

B3LYP/6-31G(d) = -3573.92999
B3LYP-D3(BJ)/def2-TZVPP/IEFPCM(propanonitrile) = -3575.508243
B3LYP-D3(BJ)/def2-TZVPP/IEFPCM(propanonitrile)//B3LYP-D3(BJ)/6-31G(d) Free Energy (Quasiharmonic) = -3574.791549

Frequencies (Top 3 out of 300)

1. -288.4926 cm^{-1}
2. 6.8563 cm^{-1}
3. 11.4756 cm^{-1}

B3LYP/6-31G(d) Molecular Geometry in Cartesian Coordinates

B	0.441178	0.544360	-0.373375
O	0.217996	0.428095	-1.841725
N	0.922627	2.010062	-0.263386
C	1.080370	2.611342	-1.594109
S	0.922356	2.979673	1.068410
C	0.473763	1.553442	-2.515319
O	0.262499	1.688287	-3.702647
H	2.907793	3.728916	-1.371161
H	0.476099	3.523959	-1.695086
C	2.533926	2.944903	-2.034493
C	3.476390	1.773381	-2.044439
C	4.419548	1.393392	-1.017463
C	5.066899	0.208038	-1.461000
N	4.555365	-0.099046	-2.706405
C	3.590436	0.832252	-3.040617
H	3.056172	0.760180	-3.978543
C	6.360901	0.101492	0.537189
C	4.774890	1.931430	0.233429
H	6.501375	-1.367350	-1.048086
H	4.705993	-0.973899	-3.184097
C	5.738150	1.281493	0.994576
H	7.103535	-0.387299	1.162333
H	4.281584	2.822786	0.608623
C	6.035181	-0.451079	-0.695623
H	6.009546	1.680664	1.967995
C	-0.557713	4.006698	0.981526
O	2.048963	3.920872	0.973088
O	0.769478	2.102975	2.237977
C	-2.860887	5.604717	0.859797
C	-0.506048	5.250609	0.349191
C	-1.741247	3.561015	1.572491
C	-2.879207	4.361515	1.509774
C	-1.657425	6.035943	0.287655
H	0.433464	5.610865	-0.055876
H	-1.755155	2.610054	2.091746
H	-3.795804	4.020943	1.986748
H	-1.613800	7.006899	-0.200095
C	-4.109204	6.450758	0.772861
H	-4.722382	6.164032	-0.092203
H	-3.867226	7.512639	0.661755
H	-4.734117	6.336629	1.665201
H	2.450562	3.376608	-3.039747
C	1.458849	-0.618078	0.124129
C	1.977061	-0.685488	1.429852
C	1.887103	-1.601834	-0.781873
C	2.873016	-1.690184	1.806569
H	1.686030	0.067127	2.156019
C	2.790699	-2.601886	-0.410821
H	1.523962	-1.574077	-1.803645
C	3.288878	-2.654652	0.889793
H	3.982962	-3.432426	1.185475
C	3.278946	-3.585734	-1.434066
C	3.440444	-1.695422	3.200921
F	4.377635	-3.130849	-2.105348
F	2.345734	-3.849599	-2.377638
F	3.639137	-4.765424	-0.882225
F	3.861404	-2.931572	3.568165
F	2.526086	-1.298837	4.115778
F	4.501783	-0.869745	3.322242
O	-0.960411	0.428156	0.254738
C	-1.408245	-0.668726	0.809490
C	-2.597319	-0.539664	1.691993
C	-3.564376	0.453751	1.475921
C	-2.733736	-1.405818	2.785302
C	-4.652281	0.568010	2.338776
H	-3.444915	1.137463	0.642359
C	-3.821193	-1.285327	3.652009
H	-1.968248	-2.154533	2.976531
C	-4.784939	-0.300173	3.427060

H	-5.397418	1.340053	2.166693
H	-3.905588	-1.947497	4.509209
H	-5.629272	-0.200329	4.103626
H	-0.648958	-1.366975	1.169783
C	-1.896370	-1.957921	-0.657234
H	-1.694055	-2.893843	-0.147426
H	-1.119315	-1.640157	-1.340642
C	-3.232383	-1.739516	-1.032937
C	-3.670584	-0.703186	-1.969798
C	-5.042736	-0.384164	-2.072340
C	-2.748830	-0.019477	-2.788352
C	-5.471342	0.593725	-2.960536
H	-5.755775	-0.900075	-1.440206
C	-3.185377	0.960031	-3.675729
H	-1.690213	-0.236008	-2.740697
C	-4.544289	1.267996	-3.764385
H	-6.528337	0.835335	-3.028040
H	-2.450602	1.476014	-4.285701
H	-4.884392	2.031587	-4.458820
O	-4.213607	-2.395201	-0.432300
Si	-4.579035	-4.020128	0.025124
C	-6.123754	-3.799301	1.060278
H	-5.923391	-3.154034	1.922811
H	-6.932581	-3.343181	0.478828
H	-6.484483	-4.764285	1.435974
C	-3.200531	-4.831986	1.012755
H	-2.904773	-4.226228	1.875237
H	-2.308506	-5.041516	0.412583
H	-3.562683	-5.794536	1.396925
C	-4.889088	-4.940741	-1.582692
H	-5.689871	-4.472691	-2.165632
H	-3.990164	-4.970307	-2.209474
H	-5.183503	-5.978060	-1.381033

TS 58

B3LYP/6-31G(d) = -3573.932114
B3LYP-D3(BJ)/def2-TZVPP/IEFPCM(propanonitrile) = -3575.508823
B3LYP-D3(BJ)/def2-TZVPP/IEFPCM(propanonitrile)//B3LYP-D3(BJ)/6-31G(d) Free Energy (Quasiharmonic) = -3574.791511

Frequencies (Top 3 out of 300)

1. -278.2647 cm^{-1}
2. 8.9594 cm^{-1}
3. 12.8480 cm^{-1}

B3LYP/6-31G(d) Molecular Geometry in Cartesian Coordinates

B	-0.002361	-0.382346	-0.709505
O	-0.187784	-1.840008	-0.964820
N	0.813555	0.031974	-1.978661
C	0.899918	-1.092751	-2.931517
S	0.846581	1.557465	-2.654753
C	0.261833	-2.243539	-2.153910
O	0.158753	-3.385662	-2.556274
H	2.642680	-0.697515	-4.125241
H	0.267993	-0.921520	-3.817671
C	2.302818	-1.492375	-3.459636
C	3.346615	-1.766753	-2.413749
C	4.481238	-0.935123	-2.088214
C	5.203260	-1.589422	-1.052682
N	4.547922	-2.773661	-0.782906
C	3.429052	-2.867330	-1.593059
H	2.782707	-3.733018	-1.539868
C	6.817925	0.163430	-0.998377
C	4.965681	0.290972	-2.582574
H	6.894462	-1.558553	0.308407
H	4.727853	-3.361833	0.016264
C	6.125727	0.825630	-2.034512
H	7.719959	0.609459	-0.587757
H	4.428644	0.817436	-3.366091
C	6.367633	-1.050427	-0.494454
H	6.508418	1.771534	-2.408909
C	1.297274	2.605595	-1.276010
O	1.919887	1.577447	-3.661698
O	-0.501723	1.972627	-3.103179
C	2.007173	4.280576	0.848350
C	0.369583	3.525967	-0.788077
C	2.584538	2.524866	-0.739543
C	2.926524	3.362200	0.318237
C	0.731904	4.355374	0.272639
H	-0.616252	3.582911	-1.236149
H	3.304747	1.817650	-1.137282
H	3.925332	3.297653	0.742654
H	0.010563	5.069562	0.662099
C	2.370818	5.137395	2.037074
H	3.439671	5.374914	2.049388
H	1.810658	6.078258	2.040944
H	2.138718	4.612840	2.972343
H	2.133099	-2.384997	-4.074528
C	0.704480	-0.134834	0.723351
C	0.260700	0.829427	1.638817
C	1.800691	-0.921398	1.107839
C	0.890413	1.014146	2.872142
C	2.418632	-0.761837	2.352039
H	2.174149	-1.680046	0.427910
C	1.971417	0.213937	3.242151
H	2.454314	0.347174	4.203659
C	3.604538	-1.610399	2.718254
C	0.375357	2.040433	3.840877
F	3.744674	-1.740222	4.056891
F	3.507988	-2.867487	2.206951
F	4.768529	-1.099602	2.252813
F	-0.443325	1.497834	4.775506
F	1.384229	2.643361	4.521278
F	-0.328802	3.020050	3.228900
O	-1.380112	0.262723	-0.610132
C	-2.259784	0.278929	-1.579274
C	-3.270441	1.363441	-1.548911
C	-3.918981	1.730696	-2.736934
C	-3.556025	2.058776	-0.365118
C	-4.839802	2.776176	-2.742434
H	-3.673703	1.217535	-3.663615
C	-4.477316	3.104175	-0.373178
H	-3.044788	1.780695	0.550318
C	-5.122249	3.463720	-1.559365
H	-5.323974	3.065006	-3.671086
H	-4.689396	3.642197	0.546570
H	-5.835473	4.283526	-1.564106
H	-1.900972	0.024572	-2.577039
C	-3.351142	-1.440864	-1.555151
H	-4.017283	-1.201645	-2.375466
H	-2.542370	-2.122316	-1.790681
C	-3.895182	-1.545121	-0.269757
C	-5.163546	-0.920762	0.138156
C	-5.441720	-0.749163	1.509051
C	-6.119898	-0.495820	-0.804805
C	-6.631756	-0.159783	1.921119
H	-4.706224	-1.067912	2.238141
C	-7.312110	0.086477	-0.387618
H	-5.943079	-0.631212	-1.865420
C	-7.570528	0.258985	0.974403
H	-6.828155	-0.024865	2.980802
H	-8.042103	0.404315	-1.126028
H	-8.502377	0.715152	1.296963
O	-3.199998	-2.102686	0.701493
Si	-2.630033	-3.708534	1.061588
C	-4.018077	-4.453222	2.095863
H	-4.203102	-3.869078	3.004528
H	-4.958799	-4.511372	1.535825

H	-3.756298	-5.472741	2.405853
C	-1.079877	-3.455751	2.070869
H	-0.707123	-4.419676	2.439186
H	-0.295896	-2.992025	1.466596
H	-1.266467	-2.815675	2.940333
H	-0.579304	1.462003	1.378763
C	-2.379974	-4.699505	-0.508538
H	-3.280389	-4.711742	-1.134490
H	-1.537638	-4.343700	-1.111772
H	-2.172006	-5.741160	-0.229184

TS 59

B3LYP/6-31G(d) = -3573.927358
B3LYP-D3(BJ)/def2-TZVPP/IEFPCM(propanonitrile) = -3575.508049
B3LYP-D3(BJ)/def2-TZVPP/IEFPCM(propanonitrile)//B3LYP-D3(BJ)/6-31G(d) Free Energy (Quasiharmonic) = -3574.791503

Frequencies (Top 3 out of 300)

1. -277.5849 cm^{-1}
2. 11.1354 cm^{-1}
3. 12.8403 cm^{-1}

B3LYP/6-31G(d) Molecular Geometry in Cartesian Coordinates

B	0.963300	-0.284166	-1.007094
O	0.879312	-1.255520	-2.127727
N	0.774828	-1.254933	0.223494
C	0.881779	-2.655281	-0.222147
S	1.149637	-0.861923	1.783873
C	0.825820	-2.529456	-1.750649
O	0.769412	-3.473604	-2.508262
H	0.109349	-3.890435	1.359816
H	1.881149	-3.059585	0.008834
C	-0.141720	-3.684684	0.316557
C	-1.602145	-3.334969	0.231427
C	-2.539889	-3.625863	-0.832374
C	-3.824509	-3.198895	-0.390159
N	-3.662256	-2.657172	0.870219
C	-2.331964	-2.750552	1.237523
H	-1.986424	-2.400504	2.199296
C	-4.832812	-3.945845	-2.417936
C	-2.427224	-4.217367	-2.104565
H	-5.951943	-3.032109	-0.805781
H	-4.395176	-2.274124	1.445520
C	-3.569941	-4.370453	-2.879764
H	-5.707952	-4.085569	-3.047216
H	-1.455621	-4.523380	-2.479253
C	-4.977952	-3.353157	-1.168400
H	-3.490178	-4.822122	-3.864750
C	2.902891	-1.149292	2.053311
O	0.439018	-1.789487	2.687258
O	0.910423	0.587735	1.915239
C	5.652412	-1.634203	2.303129
C	3.356523	-2.444681	2.315836
C	3.803838	-0.085150	1.957052
C	5.166328	-0.336398	2.085684
C	4.726882	-2.676952	2.433873
H	2.647741	-3.255189	2.448489
H	3.440572	0.920728	1.783082
H	5.867662	0.489353	1.999085
H	5.080675	-3.686204	2.629299
C	7.138261	-1.890647	2.366194
H	7.359062	-2.911320	2.693172
H	7.636847	-1.197210	3.053409
H	7.590086	-1.747373	1.376728
O	-0.159578	0.704709	-1.161247
C	-1.435347	0.436119	-0.935320
H	-1.645323	-0.509385	-0.427772
H	0.053275	-4.601631	-0.251870
C	-2.365589	0.791779	-2.041664
C	-1.997166	1.741259	-3.007549
C	-3.596922	0.132164	-2.158169
C	-2.853980	2.029789	-4.067118
H	-1.025900	2.219651	-2.933455
C	-4.446190	0.414353	-3.226995
H	-3.873427	-0.629124	-1.434336
C	-4.080330	1.367795	-4.179101
H	-2.557848	2.758106	-4.817294
H	-5.384664	-0.124209	-3.322390
H	-4.740679	1.583805	-5.014820
C	2.335333	0.567235	-1.087504
C	3.523527	-0.026249	-1.542777
C	2.404285	1.909044	-0.693021
C	4.727762	0.679880	-1.579035
H	3.512417	-1.056752	-1.883602
C	3.604954	2.625058	-0.731509
H	1.505860	2.404228	-0.341851
C	4.777131	2.014522	-1.172801
H	5.708180	2.568669	-1.209522
C	3.641182	4.030612	-0.203579
C	6.005817	-0.005533	-1.974290
F	2.521212	4.722628	-0.517950
F	3.735888	4.047017	1.151149
F	6.831839	0.818243	-2.660187
F	4.694753	4.733808	-0.676029
F	5.794694	-1.096846	-2.737082
F	6.704924	-0.419969	-0.881295
C	-1.814355	1.644935	0.521768
H	-1.560896	2.547276	-0.023426
H	-1.033826	1.289289	1.190988
C	-3.136214	1.543206	0.993457
O	-3.425669	0.672810	1.954347
Si	-3.312801	0.753516	3.686843
C	-4.192382	2.336180	4.199491
H	-4.199955	2.421362	5.293230
H	-5.231740	2.365741	3.854809
H	-3.683523	3.225019	3.808342
C	-4.257875	-0.776692	4.226267
H	-3.759351	-1.696060	3.898932
H	-4.317640	-0.816286	5.320719
H	-5.284941	-0.781762	3.842621
C	-1.536041	0.731439	4.257538
H	-1.010551	-0.177462	3.948268
H	-0.959357	1.579857	3.875129
H	-1.514284	0.782651	5.354514
C	-4.279117	2.237096	0.393275
C	-5.591894	1.818064	0.697224
C	-4.105166	3.321458	-0.491835
C	-6.689119	2.456442	0.132703
H	-5.734162	0.973138	1.360607
C	-5.206521	3.960262	-1.050530
H	-3.111266	3.675162	-0.736994
C	-6.499370	3.530424	-0.742200
H	-7.693277	2.116238	0.368379
H	-5.056146	4.795547	-1.727563
H	-7.357372	4.031218	-1.181888

TS 60

B3LYP/6-31G(d) = -3573.927461
B3LYP-D3(BJ)/def2-TZVPP/IEFPCM(propanonitrile) = -3575.507498
B3LYP-D3(BJ)/def2-TZVPP/IEFPCM(propanonitrile)//B3LYP-D3(BJ)/6-31G(d) Free Energy (Quasiharmonic) = -3574.791355

Frequencies (Top 3 out of 300)

1. -269.4250 cm^{-1}
2. 9.2883 cm^{-1}

3. 10.9799 cm-1

B3LYP/6-31G(d) Molecular Geometry in Cartesian Coordinates

B	0.405068	0.421744	-0.348703
O	0.273001	0.150832	-1.807206
N	0.594358	1.966503	-0.372204
C	0.708477	2.474167	-1.746222
S	0.598566	3.005890	0.910603
C	0.361550	1.241900	-2.582457
O	0.196426	1.230212	-3.781501
H	2.315470	3.902733	-1.547723
H	-0.059051	3.237304	-1.944097
C	2.073222	3.066504	-2.207612
C	3.231053	2.108650	-2.282506
C	4.298788	1.921060	-1.325258
C	5.173112	0.935624	-1.860833
N	4.665263	0.560600	-3.088197
C	3.497637	1.256777	-3.329694
H	2.939641	1.100704	-4.242743
C	6.594640	1.077553	0.045723
C	4.606691	2.489117	-0.074864
H	6.962136	-0.269149	-1.608363
H	5.013665	-0.200058	-3.649904
C	5.748294	2.064280	0.592749
H	7.475484	0.761242	0.598407
H	3.948315	3.228997	0.368661
C	6.318598	0.498641	-1.186827
H	5.990227	2.492522	1.561852
C	-0.897384	4.003415	0.754999
O	1.706005	3.965098	0.781134
O	0.450780	2.188182	2.119720
C	-3.220830	5.565520	0.551146
C	-0.891358	5.158988	-0.032354
C	-2.045396	3.631998	1.455574
C	-3.196062	4.413038	1.347831
C	-2.051988	5.922989	-0.136491
H	0.020785	5.470186	-0.530218
H	-2.024684	2.754351	2.092175
H	-4.086655	4.124027	1.900647
H	-2.044887	6.820750	-0.750347
C	-4.459201	6.426324	0.465155
H	-4.387251	7.288765	1.140807
H	-5.357699	5.867005	0.745265
H	-4.604788	6.820972	-0.546434
H	1.877817	3.482817	-3.203654
C	1.535144	-0.501277	0.336389
C	1.617995	-0.678683	1.726505
C	2.455839	-1.201677	-0.455527
C	2.577851	-1.517982	2.297352
H	0.939434	-0.133744	2.372734
C	3.411564	-2.050825	0.111433
H	2.427709	-1.083852	-1.533791
C	3.479752	-2.214841	1.493901
H	4.226330	-2.863236	1.937412
C	4.300010	-2.877449	-0.770546
C	2.589518	-1.731662	3.785875
F	5.470345	-3.196942	-0.180477
F	4.591277	-2.270237	-1.944835
F	3.703548	-4.062932	-1.102649
F	3.801210	-2.134246	4.234764
F	1.702270	-2.693093	4.157034
F	2.254524	-0.616726	4.466532
O	-0.922473	-0.006542	0.317796
C	-2.097768	0.340296	-0.119780
C	-3.223866	0.263557	0.842471
C	-3.102392	-0.482006	2.024508
C	-4.408787	0.971178	0.591186
C	-4.154919	-0.518491	2.937239
H	-2.174196	-1.006400	2.224045
C	-5.459659	0.933465	1.506957
H	-4.495129	1.573227	-0.310462
C	-5.335012	0.185240	2.681485
H	-4.050245	-1.088888	3.855789
H	-6.368323	1.496875	1.312304
H	-6.149638	0.161890	3.400186
H	-2.139935	1.175162	-0.826227
C	-2.637653	-0.919978	-1.659655
H	-1.673853	-0.845229	-2.146591
H	-3.431984	-0.330655	-2.105245
C	-3.013950	-2.125870	-1.062823
C	-2.073629	-3.143171	-0.592280
C	-2.538645	-4.198175	0.222956
C	-0.708025	-3.103509	-0.943552
C	-1.664736	-5.180008	0.670142
H	-3.583521	-4.224139	0.508385
C	0.160730	-4.095590	-0.497572
H	-0.321425	-2.306804	-1.567605
C	-0.314382	-5.132535	0.307662
H	-2.031526	-5.982373	1.303844
H	1.209647	-4.055284	-0.770666
H	0.368403	-5.902457	0.656014
O	-4.287249	-2.349550	-0.750018
Si	-5.789407	-2.359792	-1.591008
C	-5.693987	-3.780238	-2.817556
H	-5.487936	-4.731659	-2.314954
H	-6.641623	-3.886188	-3.359926
H	-4.905821	-3.616698	-3.561844
C	-7.020505	-2.659335	-0.211650
H	-8.046776	-2.674181	-0.597706
H	-6.836600	-3.617708	0.286405
H	-6.954356	-1.869547	0.544834
C	-6.130073	-0.735014	-2.476014
H	-6.076351	0.119300	-1.793206
H	-7.145907	-0.761197	-2.891333
H	-5.444966	-0.555065	-3.311638

TS 61

B3LYP/6-31G(d) = -3573.92929
B3LYP-D3(BJ)/def2-TZVPP/IEFPCM(propanonitrile) = -3575.508757
B3LYP-D3(BJ)/def2-TZVPP/IEFPCM(propanonitrile)//B3LYP-D3(BJ)/6-31G(d) Free Energy (Quasiharmonic) = -3574.791349

Frequencies (Top 3 out of 300)

1. -290.0342 cm-1
2. 10.5063 cm-1
3. 14.9300 cm-1

B3LYP/6-31G(d) Molecular Geometry in Cartesian Coordinates

B	0.676192	-0.505384	-0.574646
O	0.413545	-1.267475	-1.803701
N	1.773466	-1.386431	0.093510
C	1.954878	-2.641096	-0.646668
S	2.702887	-1.020650	1.390571
C	1.111718	-2.404196	-1.903382
O	1.069415	-3.148527	-2.856337
H	2.250402	-4.110896	0.898422
H	2.998810	-2.758973	-0.968851
C	1.540304	-3.951108	0.081796
C	0.137316	-3.988795	0.616147
C	-1.058886	-4.444511	-0.056050
C	-2.139231	-4.286628	0.857880
N	-1.613175	-3.764865	2.022167
C	-0.253041	-3.583851	1.869642
H	0.349798	-3.169136	2.665226
C	-3.686927	-5.151873	-0.735577
C	-1.327758	-4.974087	-1.331715
H	-4.268306	-4.485637	1.238756

H	-2.143128	-3.505697	2.839237
C	-2.632918	-5.323498	-1.656130
H	-4.698643	-5.426692	-1.022208
H	-0.529940	-5.081213	-2.060433
C	-3.455156	-4.631806	0.532624
H	-2.849075	-5.728693	-2.641275
C	4.400887	-1.132126	0.797610
O	2.589744	-2.025909	2.461901
O	2.410947	0.389944	1.721719
C	7.070087	-1.233952	-0.051412
C	4.788812	-0.435204	-0.349274
C	5.328787	-1.875806	1.525822
C	6.655775	-1.919369	1.097108
C	6.115864	-0.491447	-0.764468
H	4.058459	0.139352	-0.908652
H	5.006822	-2.409146	2.413389
H	7.379927	-2.497975	1.665572
H	6.418172	0.052068	-1.656653
C	8.500552	-1.310573	-0.529772
H	9.178901	-1.599775	0.279058
H	8.609317	-2.053724	-1.330822
H	8.840737	-0.350079	-0.932217
H	1.695682	-4.758884	-0.643772
C	-0.687994	-0.266489	0.274235
C	-0.735169	0.294297	1.562150
C	-1.909894	-0.695120	-0.276527
C	-1.945726	0.450644	2.247985
C	-3.115473	-0.558356	0.413333
H	-1.912388	-1.157603	-1.256244
C	-3.144391	0.022954	1.680900
H	-4.082012	0.138126	2.214636
C	-4.419916	-0.985076	-0.201160
C	-1.959222	1.029068	3.633245
F	-5.169070	-1.707743	0.666690
F	-5.182932	0.095788	-0.540624
F	-4.265295	-1.717387	-1.316839
F	-0.918782	1.872082	3.847133
F	-3.095207	1.739972	3.869940
F	-1.895698	0.080579	4.593841
O	1.333364	0.803751	-1.093486
C	1.321731	1.966785	-0.514195
C	2.548256	2.798415	-0.651839
C	3.354184	2.718936	-1.797235
C	2.913922	3.661155	0.389793
C	4.502527	3.500125	-1.900667
H	3.075294	2.039231	-2.597302
C	4.068465	4.436638	0.286931
H	2.311636	3.697833	1.293446
C	4.861201	4.362397	-0.859807
H	5.120471	3.436888	-2.792241
H	4.353456	5.090746	1.106066
H	5.759051	4.968818	-0.940863
H	0.787533	2.059243	0.429756
C	0.071325	3.267008	-1.555425
H	0.603414	3.208954	-2.496188
H	0.295414	4.151434	-0.970797
C	-1.253945	2.814976	-1.543329
C	-1.793400	1.873762	-2.527740
C	-0.954790	1.214928	-3.449450
C	-3.177929	1.608320	-2.555212
C	-1.488488	0.317874	-4.368432
H	0.117892	1.360003	-3.419094
C	-3.706219	0.721622	-3.484594
H	-3.824878	2.081512	-1.827489
C	-2.863396	0.075717	-4.394714
H	-0.827187	-0.209664	-5.048251
H	-4.771444	0.513612	-3.481932
H	-3.276030	-0.628912	-5.111180
O	-2.105816	3.180916	-0.596153
Si	-2.275847	4.560366	0.438673
C	-2.360793	6.064465	-0.688690
H	-3.200986	5.988422	-1.388071
H	-2.503900	6.975860	-0.095305
H	-1.445474	6.194803	-1.276729
C	-0.860106	4.684213	1.668322
H	-0.752080	3.766241	2.255295
H	-1.080579	5.494242	2.376079
H	0.103461	4.916754	1.202887
H	0.185788	0.594862	2.051296
C	-3.894073	4.222525	1.315418
H	-4.697148	3.995618	0.605756
H	-3.800002	3.378801	2.006752
H	-4.201820	5.098199	1.900212

TS 62

B3LYP/6-31G(d) = -3573.929807
B3LYP-D3(BJ)/def2-TZVPP/IEFPCM(propanonitrile) = -3575.507806
B3LYP-D3(BJ)/def2-TZVPP/IEFPCM(propanonitrile)//B3LYP-D3(BJ)/6-31G(d) Free Energy (Quasiharmonic) = -3574.791246

Frequencies (Top 3 out of 300)

1. -255.9099 cm^{-1}
2. 10.1490 cm^{-1}
3. 11.2590 cm^{-1}

B3LYP/6-31G(d) Molecular Geometry in Cartesian Coordinates

B	0.949803	0.011882	-1.173752
O	0.917159	-0.527858	-2.548355
N	0.628946	-1.281653	-0.329446
C	0.778260	-2.465062	-1.204259
S	0.961798	-1.449934	1.292345
C	0.799385	-1.850321	-2.608988
O	0.740157	-2.490074	-3.637006
H	-0.163433	-4.050065	-0.093661
H	1.775639	-2.913206	-1.058864
C	-0.234363	-3.626880	-1.097559
C	-1.681526	-3.353968	-1.417351
C	-2.804801	-3.541765	-0.525801
C	-3.989801	-3.307786	-1.277703
N	-3.595447	-2.961141	-2.555771
C	-2.214404	-3.022826	-2.641016
H	-1.708683	-2.834749	-3.577189
C	-5.354505	-3.820184	0.610022
C	-2.926254	-3.912319	0.826352
H	-6.161666	-3.273095	-1.321479
H	-4.212595	-2.863319	-3.347119
C	-4.194411	-4.048813	1.378034
H	-6.332407	-3.941810	1.068276
H	-2.039081	-4.075195	1.430910
C	-5.268259	-3.443992	-0.725853
H	-4.297001	-4.335537	2.421106
C	2.673863	-1.951754	1.497206
O	0.137129	-2.556517	1.812559
O	0.814348	-0.106868	1.880919
C	5.365905	-2.712177	1.673119
C	3.646485	-0.989524	1.783584
C	3.025239	-3.295493	1.342901
C	4.368179	-3.663414	1.426926
C	4.979427	-1.378204	1.871241
H	3.360210	0.044079	1.937056
H	2.258472	-4.046188	1.183685
H	4.642832	-4.707350	1.298194
H	5.736864	-0.628197	2.083180
C	6.825325	-3.094748	1.704189
H	7.321694	-2.711158	2.603171
H	6.959594	-4.180386	1.678492
H	7.347950	-2.668696	0.838689
H	0.150079	-4.384719	-1.791748

C	2.366645	0.754473	-0.896148
C	2.499809	1.878498	-0.071095
C	3.539757	0.258008	-1.488597
C	3.744682	2.467084	0.173548
C	4.786642	0.837809	-1.247123
H	3.483551	-0.593206	-2.159312
C	4.898356	1.949799	-0.411497
H	5.863066	2.409668	-0.229574
C	6.037414	0.225728	-1.814244
C	3.845487	3.605576	1.149515
F	6.642176	-0.592872	-0.910100
F	6.953534	1.163499	-2.148162
F	5.797338	-0.518149	-2.912811
F	3.907876	3.151982	2.427511
F	2.777051	4.430474	1.083212
F	4.950920	4.357985	0.948679
O	-0.145140	1.059498	-1.143158
C	-0.923619	1.429807	-0.165843
C	-1.373671	2.843015	-0.205919
C	-1.941491	3.432555	0.935217
C	-1.156234	3.627778	-1.349555
C	-2.293977	4.780712	0.928744
H	-2.091736	2.837680	1.831753
C	-1.510244	4.975326	-1.352079
H	-0.689753	3.174129	-2.217331
C	-2.082034	5.554269	-0.215259
H	-2.722040	5.230314	1.820245
H	-1.327557	5.578911	-2.236939
H	-2.348706	6.607704	-0.216447
H	-0.732019	1.003212	0.819289
C	-2.605604	0.260529	-0.467235
H	-2.066666	-0.659877	-0.280350
H	-2.748592	0.494594	-1.516345
C	-3.593488	0.663043	0.430447
C	-3.608769	0.299401	1.854032
C	-4.527161	0.920929	2.726312
C	-2.709017	-0.648851	2.379373
C	-4.535713	0.608026	4.080644
H	-5.221289	1.652371	2.329665
C	-2.720789	-0.954683	3.736512
H	-2.003364	-1.167445	1.744386
C	-3.630515	-0.328572	4.590527
H	-5.246044	1.096017	4.742178
H	-2.010747	-1.682348	4.116431
H	-3.636601	-0.568577	5.650360
O	-4.522877	1.548812	0.082101
Si	-5.441910	1.819406	-1.356060
C	-4.424857	2.663513	-2.689141
H	-5.087746	2.959720	-3.512736
H	-3.935559	3.566722	-2.311361
H	-3.649616	2.014976	-3.110856
C	-6.805397	2.949428	-0.738483
H	-7.487517	3.220128	-1.553431
H	-7.397982	2.468995	0.047954
H	-6.391138	3.877871	-0.329413
H	1.625345	2.311342	0.402079
C	-6.088118	0.150063	-1.926626
H	-5.270335	-0.523719	-2.204378
H	-6.671242	-0.343059	-1.141283
H	-6.737032	0.274444	-2.802532

TS 63

B3LYP/6-31G(d) = -3573.927553
B3LYP-D3(BJ)/def2-TZVPP/IEFPCM(propanonitrile) = -3575.507485
B3LYP-D3(BJ)/def2-TZVPP/IEFPCM(propanonitrile)//B3LYP-D3(BJ)/6-31G(d) Free Energy (Quasiharmonic) = -3574.791228

Frequencies (Top 3 out of 300)

1. -269.9816 cm^{-1}
2. 9.8221 cm^{-1}
3. 12.0561 cm^{-1}

B3LYP/6-31G(d) Molecular Geometry in Cartesian Coordinates

B	0.401890	0.411110	-0.354490
O	0.267840	0.129080	-1.810630
N	0.596950	1.954690	-0.390170
C	0.706470	2.452220	-1.768270
S	0.611459	3.002330	0.885890
C	0.354150	1.214470	-2.594070
O	0.183670	1.194270	-3.792260
H	2.317309	3.878890	-1.587650
H	-0.060291	3.215470	-1.968780
C	2.070619	3.037980	-2.239830
C	3.226240	2.077181	-2.311860
C	4.298540	1.896601	-1.358330
C	5.168980	0.905451	-1.889570
N	4.654060	0.519691	-3.110680
C	3.486360	1.215621	-3.352770
H	2.923780	1.052391	-4.261750
C	6.600580	1.062881	0.008160
C	4.613360	2.475181	-0.114470
H	6.958310	-0.298559	-1.635990
H	5.000250	-0.244549	-3.668880
C	5.757890	2.055031	0.551000
H	7.483980	0.750621	0.559060
H	3.957949	3.219451	0.326040
C	6.317660	0.473451	-1.217800
H	6.004820	2.490961	1.515370
C	-0.885211	3.999600	0.735730
O	1.718109	3.960210	0.741170
O	0.473050	2.192540	2.101420
C	-3.208971	5.562269	0.540240
C	-0.884811	5.149580	-0.059810
C	-2.027661	3.634070	1.448380
C	-3.178531	4.415389	1.344750
C	-2.045631	5.913840	-0.159680
H	0.023439	5.456520	-0.567340
H	-2.002400	2.760840	2.090870
H	-4.064751	4.131029	1.906920
H	-2.042901	6.807290	-0.779840
C	-4.447261	6.423629	0.458670
H	-4.369161	7.290279	1.128230
H	-5.343961	5.866929	0.749540
H	-4.600611	6.811989	-0.554200
H	1.871889	3.446750	-3.238350
C	1.528370	-0.509580	0.338880
C	1.597200	-0.687850	1.729600
C	2.455460	-1.210730	-0.444480
C	2.549160	-1.529420	2.309800
H	0.903410	-0.153810	2.368210
C	3.402060	-2.064289	0.131530
H	2.436170	-1.094810	-1.523180
C	3.456880	-2.228559	1.514540
H	4.187841	-2.890669	1.963780
C	4.288411	-2.901529	-0.742450
C	2.621530	-1.654749	3.806930
F	4.595781	-2.298259	-1.914490
F	5.450281	-3.233439	-0.142470
F	3.682051	-4.080949	-1.078770
F	1.398020	-1.568970	4.379960
F	3.382430	-0.687049	4.364910
F	3.157521	-2.840439	4.189560
O	-0.926860	-0.007260	0.316200
C	-2.101760	0.340690	-0.121660
C	-3.226300	0.273189	0.843280
C	-3.104490	-0.465221	2.029780
C	-4.409690	0.982569	0.589910
C	-4.155350	-0.492991	2.944750

H	-2.177310	-0.990750	2.231140
C	-5.458920	0.953469	1.507880
H	-4.496080	1.579339	-0.315240
C	-5.334060	0.212279	2.686830
H	-4.050350	-1.057701	3.866780
H	-6.366370	1.518229	1.311470
H	-6.147330	0.195699	3.407250
H	-2.142420	1.172010	-0.832420
C	-2.648250	-0.925711	-1.652590
H	-3.442290	-0.336731	-2.099160
H	-1.685790	-0.856470	-2.143000
C	-3.026260	-2.128041	-1.049430
C	-2.087389	-3.145920	-0.577050
C	-0.721959	-3.109520	-0.929350
C	-2.553849	-4.198390	0.240620
C	0.145221	-4.102420	-0.482010
H	-0.334129	-2.314770	-1.555120
C	-1.681609	-5.181310	0.688750
H	-3.598569	-4.221821	0.526830
C	-0.331419	-5.137180	0.325150
H	1.194041	-4.064570	-0.755790
H	-2.049699	-5.982080	1.323750
H	0.350041	-5.908180	0.673820
O	-4.299289	-2.347151	-0.732740
Si	-5.803839	-2.356901	-1.569700
C	-6.141600	-0.736041	-2.462890
H	-6.083900	0.121909	-1.785000
H	-5.457870	-0.562781	-3.301050
H	-7.158440	-0.761601	-2.875790
C	-7.032169	-2.644951	-0.185460
H	-6.961710	-1.851081	0.566340
H	-8.059479	-2.658922	-0.568790
H	-6.849829	-3.601041	0.317540
C	-5.716029	-3.784371	-2.788640
H	-6.665369	-3.890211	-3.328060
H	-5.511779	-4.733721	-2.281410
H	-4.929229	-3.627421	-3.535790

TS 64

B3LYP/6-31G(d) = -3573.927443
B3LYP-D3(BJ)/def2-TZVPP/IEFPCM(propanonitrile) = -3575.507865
B3LYP-D3(BJ)/def2-TZVPP/IEFPCM(propanonitrile)//B3LYP-D3(BJ)/6-31G(d) Free Energy (Quasiharmonic) = -3574.79118

Frequencies (Top 3 out of 300)

1. -266.9263 cm^{-1}
2. 8.4110 cm^{-1}
3. 12.5215 cm^{-1}

B3LYP/6-31G(d) Molecular Geometry in Cartesian Coordinates

B	0.343307	0.430660	-0.230383
O	0.276551	-0.030571	-1.649692
N	0.274579	1.978803	-0.453623
C	0.551436	2.309811	-1.860140
S	0.078172	3.173381	0.667043
C	0.397223	0.953766	-2.550638
O	0.370446	0.781037	-3.748213
H	1.884792	4.004066	-1.837173
H	-0.233225	2.963203	-2.267969
C	1.921964	2.973643	-2.199737
C	3.141244	2.294733	-1.647305
C	3.916734	1.228948	-2.240321
C	4.959539	0.911634	-1.323800
N	4.830088	1.763627	-0.244313
C	3.728157	2.571212	-0.436241
H	3.428250	3.291162	0.311404
C	5.779033	-0.800539	-2.767933
C	3.833796	0.506542	-3.444813
H	6.675533	-0.333722	-0.855362
H	5.301146	1.659222	0.642354
C	4.761541	-0.497555	-3.695437
H	6.484916	-1.596857	-2.988286
H	3.039591	0.713010	-4.156756
C	5.893849	-0.098418	-1.573057
H	4.699371	-1.065550	-4.620044
C	-1.431714	4.042273	0.197849
O	1.146844	4.179809	0.559749
O	-0.184426	2.506693	1.950655
C	-3.786069	5.405036	-0.497941
C	-1.392336	5.046856	-0.774059
C	-2.629095	3.730399	0.842601
C	-3.794609	4.411135	0.489558
C	-2.567354	5.709942	-1.121693
H	-0.448213	5.325070	-1.230194
H	-2.636417	2.981419	1.626732
H	-4.724083	4.174122	1.002341
H	-2.534086	6.488400	-1.880374
C	-5.044400	6.159808	-0.856284
H	-5.941008	5.626704	-0.524394
H	-5.123112	6.317995	-1.937603
H	-5.057876	7.150586	-0.383619
O	-0.884071	-0.127447	0.491253
C	-2.111171	0.149558	0.152083
H	-2.254975	0.996183	-0.526018
H	1.962778	3.016468	-3.295436
C	-3.147209	-0.009540	1.201771
C	-2.868482	-0.726551	2.374325
C	-4.403247	0.595964	1.045142
C	-3.836478	-0.837840	3.370499
H	-1.884593	-1.164796	2.500867
C	-5.368369	0.485977	2.045481
H	-4.612203	1.176925	0.149763
C	-5.087147	-0.235478	3.209531
H	-3.610151	-1.384550	4.281570
H	-6.332610	0.972430	1.924484
H	-5.834927	-0.314626	3.993916
C	1.625161	-0.202702	0.525485
C	2.129681	0.276973	1.744122
C	2.290745	-1.292836	-0.054109
C	3.256954	-0.299651	2.338079
H	1.648715	1.118814	2.231238
C	3.383061	-1.906512	0.563426
H	1.965442	-1.655391	-1.021022
C	3.882343	-1.406581	1.763866
H	4.748527	-1.857914	2.235053
C	4.003497	-3.135780	-0.035144
C	3.887770	0.351695	3.533593
F	5.342301	-3.175722	0.141598
F	3.508633	-4.272725	0.535580
F	2.998369	1.001410	4.308074
F	3.763289	-3.248806	-1.361465
F	4.817962	1.278318	3.148199
F	4.543717	-0.533182	4.319380
C	-2.679219	-1.112925	-1.359990
H	-1.763969	-0.970177	-1.920371
H	-3.546356	-0.574964	-1.728856
C	-2.919202	-2.351249	-0.757525
O	-4.134859	-2.654771	-0.313354
Si	-5.702555	-2.811493	-1.007093
C	-6.768054	-3.201963	0.482677
H	-7.821395	-3.304935	0.195654
H	-6.696720	-2.404542	1.230541
H	-6.458331	-4.138614	0.959276
C	-6.258118	-1.230456	-1.861421
H	-5.660282	-0.997366	-2.749405
H	-7.297731	-1.347581	-2.193698
H	-6.223598	-0.370242	-1.184827
C	-5.597815	-4.232441	-2.232047

H	-5.258822	-5.154921	-1.747935
H	-6.580143	-4.429836	-2.678694
H	-4.903632	-4.008975	-3.050625
C	-1.874832	-3.316696	-0.414351
C	-0.599896	-3.263083	-1.014063
C	-2.148547	-4.338616	0.520495
C	0.368481	-4.207388	-0.686577
H	-0.362729	-2.490168	-1.735573
C	-1.170423	-5.266617	0.854496
H	-3.124156	-4.376681	0.990815
C	0.090004	-5.203683	0.250911
H	1.345905	-4.161087	-1.153258
H	-1.385880	-6.038237	1.588073
H	0.859219	-5.924002	0.513838

TS 65

B3LYP/6-31G(d) = -3573.928191
B3LYP-D3(BJ)/def2-TZVPP/IEFPCM(propanonitrile) = -3575.509002
B3LYP-D3(BJ)/def2-TZVPP/IEFPCM(propanonitrile)//B3LYP-D3(BJ)/6-31G(d) Free Energy (Quasiharmonic) = -3574.791153

Frequencies (Top 3 out of 300)

1. -294.7569 cm^{-1}
2. 11.7766 cm^{-1}
3. 17.1861 cm^{-1}

B3LYP/6-31G(d) Molecular Geometry in Cartesian Coordinates

B	-0.733223	-0.386769	0.849800
O	-0.647896	-0.796306	2.262237
N	-1.755223	-1.428107	0.298069
C	-2.010914	-2.475609	1.295469
S	-2.661360	-1.337080	-1.061454
C	-1.313432	-1.922102	2.543414
O	-1.335080	-2.442838	3.636082
H	-2.028635	-4.251047	0.073007
H	-3.084434	-2.551118	1.518892
C	-1.497286	-3.903678	0.963241
C	-0.011100	-4.034738	0.765794
C	0.709476	-4.135871	-0.483084
C	2.090518	-4.249826	-0.162984
N	2.195069	-4.235786	1.213204
C	0.935182	-4.093510	1.762499
H	0.801349	-4.049499	2.834722
C	2.675378	-4.357210	-2.470834
C	0.327933	-4.140838	-1.837795
H	4.134129	-4.426009	-0.872275
H	3.062784	-4.159184	1.720386
C	1.311178	-4.251728	-2.812927
H	3.419921	-4.434471	-3.258907
H	-0.715248	-4.028934	-2.114785
C	3.083888	-4.357194	-1.142632
H	1.026850	-4.245925	-3.861782
C	-4.364392	-1.122671	-0.500518
O	-2.232302	-0.108960	-1.762082
O	-2.661694	-2.608366	-1.802199
C	-7.035013	-0.754479	0.273465
C	-4.668727	-0.241184	0.538253
C	-5.378225	-1.822959	-1.156290
C	-6.703325	-1.630499	-0.768502
C	-5.997544	-0.065004	0.917342
H	-3.873499	0.294045	1.045617
H	-5.122231	-2.512137	-1.953393
H	-7.492421	-2.175158	-1.281604
H	-6.233591	0.619738	1.728637
C	-8.469207	-0.581484	0.714274
H	-9.166682	-0.764514	-0.109613
H	-8.722475	-1.285489	1.518171
H	-8.652443	0.427846	1.097859
H	-1.822599	-4.532426	1.801163
C	0.729188	-0.397207	0.147168
C	0.942383	-0.251709	-1.234141
C	1.860800	-0.648262	0.941963
C	2.228111	-0.306975	-1.783908
C	3.140019	-0.743206	0.391403
H	1.731955	-0.785826	2.008840
C	3.335854	-0.558933	-0.977162
H	4.330565	-0.613135	-1.405962
C	4.321724	-1.098394	1.246912
C	2.427930	-0.145808	-3.264205
F	4.087018	-0.928521	2.565657
F	5.414072	-0.360696	0.933709
F	4.694032	-2.402296	1.090587
F	3.593058	0.507219	-3.539074
F	1.433129	0.580270	-3.835850
F	2.484162	-1.318458	-3.923006
O	-1.395454	1.013630	0.914219
C	-1.278633	1.958518	0.025721
C	-2.474531	2.810000	-0.218729
C	-3.366726	3.132348	0.814021
C	-2.718021	3.286371	-1.513818
C	-4.479824	3.927862	0.554973
H	-3.182334	2.754276	1.815583
C	-3.837860	4.076286	-1.772263
H	-2.050491	3.005128	-2.323418
C	-4.716858	4.403483	-0.738363
H	-5.165428	4.176709	1.360361
H	-4.028465	4.427010	-2.782548
H	-5.587665	5.021344	-0.939360
H	-0.651771	1.760451	-0.841204
C	-0.088011	3.492570	0.767345
H	-0.705397	3.711002	1.629359
H	-0.235920	4.165648	-0.068833
C	1.226013	3.079960	1.022021
C	1.649081	2.499134	2.300200
C	0.707080	2.055886	3.249390
C	3.022524	2.393132	2.598315
C	1.131407	1.519775	4.460944
H	-0.351793	2.076118	3.022730
C	3.438410	1.878811	3.819910
H	3.751364	2.712691	1.862784
C	2.494445	1.440679	4.753939
H	0.396727	1.148757	5.168497
H	4.498954	1.802642	4.039804
H	2.822085	1.026637	5.703436
O	2.178954	3.168008	0.107682
Si	2.468075	4.175264	-1.276083
C	2.447247	5.949770	-0.649964
H	3.205549	6.106437	0.125467
H	2.664653	6.643196	-1.471642
H	1.474922	6.231382	-0.230619
C	1.189908	3.891383	-2.622776
H	1.487548	4.462565	-3.512124
H	1.140299	2.838914	-2.919985
H	0.183744	4.224992	-2.348109
H	0.093764	-0.114109	-1.896944
C	4.166352	3.624330	-1.834159
H	4.137995	2.603410	-2.228352
H	4.536917	4.278562	-2.632973
H	4.891501	3.655824	-1.013613

TS 66

B3LYP/6-31G(d) = -3573.931799
B3LYP-D3(BJ)/def2-TZVPP/IEFPCM(propanonitrile) = -3575.507744
B3LYP-D3(BJ)/def2-TZVPP/IEFPCM(propanonitrile)//B3LYP-D3(BJ)/6-31G(d) Free Energy (Quasiharmonic) = -3574.791115

Frequencies (Top 3 out of 300)

1. -277.3238 cm^{-1}
2. 9.1348 cm^{-1}
3. 14.1891 cm^{-1}

B3LYP/6-31G(d) Molecular Geometry in Cartesian Coordinates

B	0.346510	0.585650	-0.419170
O	0.052220	0.192300	-1.826370
N	0.781280	2.057960	-0.601850
C	0.960760	2.372980	-2.024660
S	0.723530	3.277540	0.498270
C	0.396230	1.132240	-2.724020
O	0.282220	0.992380	-3.919750
H	2.739220	3.582580	-1.994160
H	0.333820	3.227250	-2.319760
C	2.410280	2.668950	-2.496660
C	3.403520	1.562709	-2.270520
C	4.336910	1.423249	-1.175380
C	5.064540	0.221939	-1.395150
N	4.605569	-0.326251	-2.576650
C	3.600390	0.473449	-3.086810
H	3.100530	0.211740	-4.009750
C	6.306280	0.540949	0.613620
C	4.624890	2.188659	-0.029950
H	6.578199	-1.165341	-0.690720
H	4.837949	-1.249931	-2.906200
C	5.602420	1.740179	0.849170
H	7.059260	0.214279	1.326090
H	4.070780	3.098739	0.176520
C	6.048499	-0.233861	-0.510680
H	5.822640	2.317109	1.743250
C	-0.697469	4.297430	0.030650
O	1.889741	4.160740	0.339840
O	0.440930	2.668710	1.807030
C	-2.894059	5.925301	-0.607200
C	-0.511989	5.664940	-0.170140
C	-1.970010	3.731621	-0.087190
C	-3.053549	4.545351	-0.406410
C	-1.608949	6.467081	-0.487700
H	0.483641	6.085020	-0.080820
H	-2.099470	2.664421	0.058210
H	-4.043310	4.103961	-0.504550
H	-1.460229	7.532331	-0.647710
C	-4.082549	6.801971	-0.923770
H	-4.799009	6.288121	-1.574090
H	-3.776529	7.727761	-1.421070
H	-4.619929	7.084411	-0.008610
H	2.333500	2.894900	-3.567310
C	1.406039	-0.441330	0.247650
C	1.933939	-1.502880	-0.501960
C	1.843689	-0.311770	1.576140
C	2.850529	-2.404390	0.046180
H	1.635109	-1.616920	-1.537840
C	2.765849	-1.206700	2.125650
H	1.468430	0.507010	2.183120
C	3.272579	-2.262011	1.367370
H	3.978809	-2.960321	1.801600
C	3.266809	-0.996321	3.529420
C	3.436439	-3.496501	-0.799500
F	4.313199	-0.142391	3.569140
F	3.687429	-2.156201	4.092980
F	2.309799	-0.485110	4.335520
F	4.634909	-3.148061	-1.348140
F	2.635759	-3.828230	-1.847110
F	3.660988	-4.627391	-0.096130
O	-0.971750	0.557810	0.366030
C	-1.595741	-0.552069	0.652760
C	-2.456941	-0.535029	1.859960
C	-2.616490	0.650901	2.595190
C	-3.038391	-1.724339	2.330950
C	-3.369710	0.646021	3.767910
H	-2.105180	1.550601	2.270870
C	-3.786311	-1.723689	3.506100
H	-2.890201	-2.653089	1.787240
C	-3.960861	-0.535689	4.222570
H	-3.477370	1.563441	4.339480
H	-4.222031	-2.650099	3.869780
H	-4.538601	-0.536609	5.142920
H	-1.053651	-1.484399	0.471750
C	-2.828661	-0.777119	-0.962670
H	-2.007011	-0.634149	-1.653760
H	-3.456300	0.095551	-0.817740
C	-3.442451	-2.031219	-0.906860
C	-2.742081	-3.285879	-1.214500
C	-1.404011	-3.288639	-1.663010
C	-3.400902	-4.519079	-1.032100
C	-0.744832	-4.490760	-1.901520
H	-0.872821	-2.357900	-1.831230
C	-2.740282	-5.715829	-1.285610
H	-4.427742	-4.523829	-0.685910
C	-1.410002	-5.705099	-1.714760
H	0.293608	-4.476360	-2.214220
H	-3.259392	-6.659099	-1.142020
H	-0.891562	-6.641300	-1.901870
O	-4.670411	-2.186469	-0.429310
Si	-6.105381	-1.210398	-0.477800
C	-6.360721	-0.706468	-2.269900
H	-6.446371	-1.582548	-2.922510
H	-7.285591	-0.125268	-2.370970
H	-5.539801	-0.086648	-2.646810
C	-7.407571	-2.417238	0.122950
H	-7.469021	-3.297478	-0.526510
H	-7.187911	-2.761958	1.139640
H	-8.397081	-1.944758	0.139100
C	-5.975190	0.269212	0.669560
H	-5.661680	-0.027278	1.675750
H	-6.961480	0.744172	0.752970
H	-5.275430	1.031932	0.312900

TS 67

B3LYP/6-31G(d) = -3573.933512
B3LYP-D3(BJ)/def2-TZVPP/IEFPCM(propanonitrile) = -3575.507413
B3LYP-D3(BJ)/def2-TZVPP/IEFPCM(propanonitrile)//B3LYP-D3(BJ)/6-31G(d) Free Energy (Quasiharmonic) = -3574.791113

Frequencies (Top 3 out of 300)

1. -263.9626 cm^{-1}
2. 10.6261 cm^{-1}
3. 13.8680 cm^{-1}

B3LYP/6-31G(d) Molecular Geometry in Cartesian Coordinates

B	0.266210	-0.313576	0.031513
O	-0.468177	0.440243	-1.020185
N	0.292841	-1.735172	-0.606592
C	-0.190873	-1.703922	-1.991613
S	1.167613	-3.011720	-0.019540
C	-0.699944	-0.266447	-2.131090
O	-1.235525	0.198381	-3.115512
H	-0.798563	-3.712651	-2.456428
H	0.641327	-1.811317	-2.706894
C	-1.277665	-2.732672	-2.405615
C	-2.494145	-2.819524	-1.527634
C	-3.763149	-2.145924	-1.693739
C	-4.624172	-2.610728	-0.657615
N	-3.905652	-3.521020	0.089021
C	-2.630462	-3.633081	-0.427588

H	-1.900442	-4.295624	0.014989
C	-6.411919	-1.247366	-1.456981
C	-4.266791	-1.213922	-2.620155
H	-6.588524	-2.542778	0.267880
H	-4.220368	-3.953110	0.943704
C	-5.580087	-0.777232	-2.493794
H	-7.435199	-0.887964	-1.385991
H	-3.627596	-0.828956	-3.408972
C	-5.945619	-2.169087	-0.525653
H	-5.975536	-0.057000	-3.205016
C	2.782739	-2.989096	-0.816033
O	0.522757	-4.260014	-0.460485
O	1.374739	-2.748922	1.410656
C	5.287398	-2.935426	-2.079557
C	2.930031	-3.544418	-2.090280
C	3.879257	-2.431072	-0.154749
C	5.118966	-2.410873	-0.790354
C	4.176441	-3.508186	-2.713240
H	2.088494	-4.031219	-2.571811
H	3.768013	-2.037812	0.848916
H	5.973323	-1.984636	-0.269959
H	4.289986	-3.944775	-3.702667
C	6.628972	-2.873909	-2.770135
H	7.452598	-2.972417	-2.055091
H	6.761296	-1.914267	-3.287378
H	6.731749	-3.665409	-3.519536
H	-1.568777	-2.457752	-3.426373
C	1.687904	0.379948	0.361269
C	2.380402	1.086889	-0.631946
C	2.296464	0.293688	1.622337
C	3.616109	1.691423	-0.381020
H	1.949847	1.179929	-1.624182
C	3.546773	0.866545	1.870503
H	1.795910	-0.248005	2.416442
C	4.214543	1.578122	0.873257
H	5.178229	2.032685	1.069811
C	4.213107	0.628166	3.196303
C	4.256411	2.533022	-1.448106
F	3.332779	0.655656	4.221095
F	5.172432	1.543549	3.462787
F	4.812739	-0.589370	3.230381
F	3.722493	3.787040	-1.479510
F	4.079873	2.013609	-2.682267
F	5.584652	2.686397	-1.257072
O	-0.531886	-0.326976	1.316790
C	-1.829070	-0.301774	1.404341
C	-2.436426	-1.081619	2.514860
C	-3.832133	-1.210918	2.606829
C	-1.617493	-1.739724	3.443800
C	-4.400188	-1.971881	3.625020
H	-4.469506	-0.726371	1.871001
C	-2.191708	-2.503340	4.459750
H	-0.540502	-1.682654	3.334205
C	-3.580010	-2.615678	4.558201
H	-5.480972	-2.064656	3.692533
H	-1.551647	-3.019144	5.169882
H	-4.023621	-3.209007	5.353300
H	-2.401839	-0.260811	0.476565
C	-2.328851	1.610535	2.060446
H	-1.573437	1.643871	2.838609
H	-3.345110	1.411206	2.374819
C	-2.163201	2.451599	0.959899
C	-3.281821	2.739991	0.033473
C	-3.116673	2.621092	-1.358114
C	-4.531693	3.125650	0.550758
C	-4.185906	2.893940	-2.209207
H	-2.183198	2.254176	-1.769279
C	-5.587549	3.418399	-0.308560
H	-4.660651	3.229979	1.624189
C	-5.415872	3.303536	-1.690280
H	-4.056059	2.772198	-3.280386
H	-6.542899	3.736222	0.099256
H	-6.242687	3.524143	-2.359813
O	-0.960713	2.916728	0.708750
Si	-0.266402	4.314683	-0.066802
C	1.250581	4.623783	0.987551
H	0.977303	4.836583	2.027120
H	1.920195	3.758334	0.981352
H	1.815482	5.481924	0.603204
C	-1.492733	5.735272	0.079610
H	-1.014726	6.656839	-0.277130
H	-2.394390	5.576421	-0.519795
H	-1.797150	5.905859	1.118725
C	0.163391	3.913542	-1.852048
H	0.113184	2.836074	-2.034088
H	-0.517354	4.406010	-2.554842
H	1.185983	4.237648	-2.075003

TS 68

B3LYP/6-31G(d) = -3573.926115
B3LYP-D3(BJ)/def2-TZVPP/IEFPCM(propanonitrile) = -3575.507541
B3LYP-D3(BJ)/def2-TZVPP/IEFPCM(propanonitrile)//B3LYP-D3(BJ)/6-31G(d) Free Energy (Quasiharmonic) = -3574.791068

Frequencies (Top 3 out of 300)

1. -274.2905 cm^{-1}
2. 7.2757 cm^{-1}
3. 12.0479 cm^{-1}

B3LYP/6-31G(d) Molecular Geometry in Cartesian Coordinates

B	0.310594	0.627777	-0.417320
O	0.483949	0.321628	-1.852021
N	0.638655	2.156244	-0.401763
C	1.188229	2.575944	-1.695802
S	0.309019	3.255533	0.765592
C	0.963749	1.337634	-2.571110
O	1.199442	1.286138	-3.758218
H	2.725665	4.020053	-1.251713
H	0.591880	3.389720	-2.133264
C	2.678311	3.029722	-1.713029
C	3.647282	2.115594	-1.020151
C	4.334038	0.960708	-1.553788
C	5.122231	0.418788	-0.498438
N	4.936529	1.223532	0.608397
C	4.033086	2.219847	0.293789
H	3.716121	2.939553	1.035155
C	5.906635	-1.334903	-1.912604
C	4.358992	0.325159	-2.808946
H	6.503895	-1.122740	0.156655
H	5.204794	0.984037	1.551247
C	5.139958	-0.812713	-2.974262
H	6.504992	-2.228102	-2.072389
H	3.752833	0.702368	-3.627400
C	5.910921	-0.724274	-0.663027
H	5.157712	-1.313958	-3.938598
C	-0.936025	4.371320	0.091248
O	1.481130	4.105469	1.034688
O	-0.311485	2.506501	1.876520
C	-2.867117	6.098055	-0.986638
C	-0.537201	5.557851	-0.527626
C	-2.289343	4.042947	0.186951
C	-3.241455	4.905416	-0.350270
C	-1.503351	6.407390	-1.066399
H	0.514844	5.818936	-0.564850
H	-2.591121	3.131570	0.688560
H	-4.295188	4.647857	-0.270302
H	-1.190504	7.329682	-1.550162
C	-3.910149	7.040436	-1.539322

H	-3.508461	7.649051	-2.355916
H	-4.269235	7.730492	-0.764072
H	-4.782256	6.496423	-1.917741
H	2.941037	3.150870	-2.771188
C	1.290142	-0.323140	0.469531
C	1.717359	-0.042001	1.778336
C	1.793321	-1.492878	-0.122938
C	2.597925	-0.893874	2.455121
H	1.374164	0.861553	2.272756
C	2.641592	-2.366043	0.563738
H	1.530673	-1.709409	-1.152118
C	3.055143	-2.071052	1.861084
H	3.728094	-2.733846	2.392602
C	3.052416	-3.655193	-0.086863
C	3.142192	-0.491820	3.795840
F	3.248286	-3.528861	-1.415195
F	2.076062	-4.609285	0.056524
F	4.172547	-4.179318	0.449261
F	3.488077	-1.559095	4.551376
F	2.268646	0.245010	4.509938
F	4.273032	0.265044	3.668195
O	-1.168000	0.366918	-0.160588
C	-1.711940	0.022185	0.984563
C	-3.141864	0.413819	1.160666
C	-3.700507	0.464651	2.445300
C	-3.931682	0.759711	0.055005
C	-5.027710	0.850627	2.624199
H	-3.086843	0.219674	3.309210
C	-5.259558	1.148309	0.235047
H	-3.482984	0.749566	-0.933029
C	-5.812185	1.190675	1.518271
H	-5.445513	0.901319	3.625817
H	-5.860402	1.429650	-0.626043
H	-6.844273	1.500895	1.658032
H	-1.097137	0.162645	1.875903
C	-1.706321	-1.910910	1.210297
H	-2.096896	-1.975157	2.222248
H	-0.637782	-2.064290	1.108810
C	-2.523389	-2.464870	0.209130
C	-2.009754	-2.839498	-1.111364
C	-0.760764	-3.476023	-1.230240
C	-2.747852	-2.548306	-2.275653
C	-0.262644	-3.817075	-2.484235
H	-0.187941	-3.726891	-0.345480
C	-2.235390	-2.872366	-3.526835
H	-3.691806	-2.019809	-2.193520
C	-0.994764	-3.509715	-3.632834
H	0.705320	-4.302821	-2.556512
H	-2.792661	-2.614470	-4.422461
H	-0.595715	-3.756609	-4.612308
O	-3.820677	-2.491683	0.434673
Si	-5.185970	-3.524272	0.174607
C	-4.610674	-5.171394	-0.519460
H	-3.851888	-5.638101	0.119091
H	-4.194224	-5.079305	-1.527079
H	-5.463666	-5.859994	-0.570720
C	-6.358075	-2.608938	-0.969011
H	-6.500048	-1.576739	-0.630362
H	-5.999687	-2.582826	-2.003507
H	-7.339787	-3.098582	-0.972323
C	-5.913313	-3.699195	1.894050
H	-5.219853	-4.207491	2.573540
H	-6.140456	-2.715530	2.319888
H	-6.843362	-4.280126	1.870498

TS 69

B3LYP/6-31G(d) = -3573.926115
B3LYP-D3(BJ)/def2-TZVPP/IEFPCM(propanonitrile) = -3575.507542
B3LYP-D3(BJ)/def2-TZVPP/IEFPCM(propanonitrile)//B3LYP-D3(BJ)/6-31G(d) Free Energy (Quasiharmonic) = -3574.791065

Frequencies (Top 3 out of 300)

1. -274.2917 cm^{-1}
2. 7.3011 cm^{-1}
3. 12.1215 cm^{-1}

B3LYP/6-31G(d) Molecular Geometry in Cartesian Coordinates

B	0.310563	0.627793	-0.417302
O	0.483904	0.321583	-1.851995
N	0.638402	2.156308	-0.401844
C	1.187854	2.576012	-1.695935
S	0.308584	3.255620	0.765442
C	0.963535	1.337613	-2.571158
O	1.199203	1.286079	-3.758271
H	2.725080	4.020407	-1.252033
H	0.591354	3.389657	-2.133433
C	2.677863	3.030024	-1.713252
C	3.646990	2.116112	-1.020308
C	4.333900	0.961272	-1.553844
C	5.122196	0.419573	-0.498459
N	4.936404	1.224400	0.608301
C	4.032808	2.220552	0.293614
H	3.715755	2.940286	1.034914
C	5.906816	-1.334151	-1.912465
C	4.358913	0.325598	-2.808939
H	6.504097	-1.121690	0.156757
H	5.204719	0.985030	1.551168
C	5.140037	-0.812180	-2.974157
H	6.505297	-2.227280	-2.072173
H	3.752681	0.702639	-3.627416
C	5.911046	-0.723393	-0.662951
H	5.157839	-1.313522	-3.938442
C	-0.936908	4.370969	0.091212
O	-0.311539	2.506520	1.876536
O	1.480491	4.105930	1.034239
C	-2.868725	6.096971	-0.986558
C	-2.290159	4.042716	0.188137
C	-0.538495	5.557060	-0.528795
C	-1.504999	6.406222	-1.067503
C	-3.242638	4.904828	-0.349037
H	-2.591590	3.131732	0.690667
H	0.513520	5.818140	-0.566911
H	-1.192472	7.328165	-1.552141
H	-4.296319	4.647365	-0.268105
C	-3.912085	7.038960	-1.539290
H	-4.785019	6.494769	-1.915535
H	-3.511271	7.645987	-2.357502
H	-4.269667	7.730522	-0.764692
O	-1.167981	0.366754	-0.160497
C	-1.711816	0.021930	0.984673
H	-1.096967	0.162405	1.875981
H	2.940541	3.151105	-2.771430
C	-3.141765	0.413416	1.160886
C	-3.700344	0.464078	2.445556
C	-3.931664	0.759360	0.055301
C	-5.027564	0.849944	2.624564
H	-3.086618	0.219058	3.309409
C	-5.259560	1.147836	0.235452
H	-3.483012	0.749365	-0.932754
C	-5.812122	1.190041	1.518709
H	-5.445318	0.900508	3.626209
H	-5.860468	1.429219	-0.625580
H	-6.844226	1.500169	1.658554
C	1.290285	-0.322940	0.469556
C	1.717515	-0.041689	1.778332
C	1.793599	-1.492636	-0.122882
C	2.598215	-0.893421	2.455121

H	1.374219	0.861842	2.272724
C	2.642005	-2.365664	0.563799
H	1.530951	-1.709241	-1.152045
C	3.055562	-2.070567	1.861120
H	3.728615	-2.733254	2.392643
C	3.052962	-3.654789	-0.086765
C	3.142467	-0.491243	3.795810
F	4.173187	-4.178747	0.449323
F	2.076738	-4.608997	0.056710
F	2.268828	0.245470	4.509917
F	3.248749	-3.528487	-1.415114
F	4.273182	0.265798	3.668106
F	3.488546	-1.558441	4.551365
C	-1.705982	-1.911190	1.210321
H	-2.096526	-1.975547	2.222277
H	-0.637426	-2.064430	1.108795
C	-2.522999	-2.465161	0.209123
C	-2.009332	-2.839569	-1.111421
C	-2.747468	-2.548293	-2.275665
C	-0.760271	-3.475935	-1.230393
C	-2.234968	-2.872101	-3.526896
H	-3.691486	-2.019921	-2.193450
C	-0.262113	-3.816739	-2.484440
H	-0.187418	-3.726871	-0.345669
C	-0.994265	-3.509284	-3.632993
H	-2.792270	-2.614134	-4.422483
H	0.705908	-4.302361	-2.556794
H	-0.595183	-3.755973	-4.612504
O	-3.820284	-2.492168	0.434676
Si	-5.185408	-3.524955	0.174504
C	-5.912543	-3.700481	1.893975
H	-6.842487	-4.281576	1.870352
H	-5.218923	-4.208841	2.573255
H	-6.139827	-2.716979	2.320113
C	-6.357776	-2.609509	-0.968750
H	-5.999450	-2.582997	-2.003257
H	-7.339389	-3.099353	-0.972162
H	-6.499938	-1.577445	-0.629770
C	-4.609914	-5.171804	-0.520049
H	-5.462792	-5.860542	-0.571344
H	-4.193630	-5.079399	-1.527706
H	-3.850949	-5.638530	0.118276

TS 70

B3LYP/6-31G(d) = -3573.926131
B3LYP-D3(BJ)/def2-TZVPP/IEFPCM(propanonitrile) = -3575.50753
B3LYP-D3(BJ)/def2-TZVPP/IEFPCM(propanonitrile)//B3LYP-D3(BJ)/6-31G(d) Free Energy (Quasiharmonic) = -3574.790961

Frequencies (Top 3 out of 300)

1. -274.3173 cm^{-1}
2. 7.5311 cm^{-1}
3. 12.8253 cm^{-1}

B3LYP/6-31G(d) Molecular Geometry in Cartesian Coordinates

B	0.311498	0.628814	-0.416154
O	0.484201	0.324971	-1.851439
N	0.640676	2.157023	-0.398071
C	1.189953	2.578564	-1.691639
S	0.311201	3.254899	0.770695
C	0.964335	1.341902	-2.568967
O	1.199434	1.292337	-3.756287
H	2.728265	4.021171	-1.245906
H	0.593925	3.393441	-2.127489
C	2.680276	3.031543	-1.708651
C	3.648859	2.115861	-1.017316
C	4.334667	0.961227	-1.552693
C	5.122766	0.417341	-0.498288
N	4.937918	1.220716	0.609686
C	4.035067	2.218069	0.296665
H	3.718803	2.937002	1.039086
C	5.905543	-1.335002	-1.915025
C	4.358822	0.327346	-2.808711
H	6.503544	-1.126025	0.154343
H	5.206238	0.979714	1.552136
C	5.138933	-0.810857	-2.975768
H	6.503230	-2.228404	-2.076177
H	3.752721	0.706090	-3.626500
C	5.910613	-0.726049	-0.664634
H	5.156064	-1.310826	-3.940777
C	-0.934244	4.370391	0.096577
O	1.483315	4.104685	1.040165
O	-0.308750	2.504555	1.881029
C	-2.866210	6.099462	-0.976245
C	-2.287711	4.044856	0.198031
C	-0.535859	5.558156	-0.520482
C	-1.502486	6.410089	-1.054301
C	-3.240421	4.909736	-0.334603
H	-2.589496	3.137651	0.707145
H	0.515731	5.821645	-0.553323
H	-1.190361	7.336821	-1.529972
H	-4.294614	4.658007	-0.243506
C	-3.905811	7.013986	-1.579706
H	-4.131696	6.725828	-2.615193
H	-3.562761	8.053529	-1.599669
H	-4.846385	6.976952	-1.019832
O	-1.167150	0.368474	-0.159397
C	-1.710993	0.023036	0.985596
H	-1.095757	0.162054	1.876857
H	2.942797	3.154072	-2.766702
C	-3.140507	0.415703	1.162641
C	-3.698697	0.465329	2.447530
C	-3.930299	0.763933	0.057714
C	-5.025454	0.852402	2.627374
H	-3.085013	0.218561	3.310917
C	-5.257730	1.153594	0.238689
H	-3.481872	0.754814	-0.930447
C	-5.809923	1.194747	1.522141
H	-5.442894	0.902155	3.629191
H	-5.858515	1.436841	-0.621812
H	-6.841659	1.505807	1.662645
C	1.290690	-0.324211	0.468785
C	1.792692	-1.493454	-0.125649
C	1.718646	-0.045369	1.777836
C	2.640591	-2.368289	0.559358
H	1.529428	-1.708256	-1.155039
C	2.598786	-0.898964	2.453001
H	1.376356	0.857714	2.273745
C	3.054872	-2.075585	1.856992
H	3.727515	-2.739704	2.387241
C	3.143757	-0.499442	3.794196
C	3.050211	-3.656728	-0.093406
F	3.489079	-1.568174	4.547922
F	4.275116	0.256802	3.667446
F	3.245903	-3.528403	-1.421569
F	2.270955	0.236887	4.509717
F	2.073089	-4.610244	0.048634
F	4.170013	-4.182652	0.441634
C	-1.706771	-1.910298	1.209341
H	-0.638424	-2.064378	1.106925
H	-2.096649	-1.975248	2.221514
C	-2.525022	-2.462613	0.208243
O	-3.822163	-2.488624	0.434725
Si	-5.188464	-3.520018	0.175357
C	-4.615260	-5.166676	-0.521538
H	-3.855915	-5.634692	0.115390
H	-5.468794	-5.854627	-0.572470
H	-4.200126	-5.073630	-1.529611

C	-5.913621	-3.696643	1.895550
H	-6.139514	-2.713373	2.322960
H	-6.844103	-4.276900	1.872497
H	-5.219610	-4.206300	2.573458
C	-6.361388	-2.602361	-0.965545
H	-6.004280	-2.575129	-2.000457
H	-7.343459	-3.091287	-0.968276
H	-6.502171	-1.570504	-0.625360
C	-2.012720	-2.836332	-1.113027
C	-0.764103	-3.473274	-1.233576
C	-2.751776	-2.543910	-2.276400
C	-0.267269	-3.813506	-2.488304
H	-0.190580	-3.725137	-0.349554
C	-2.240611	-2.867190	-3.528314
H	-3.695458	-2.015128	-2.192956
C	-1.000334	-3.504945	-3.635977
H	0.700413	-4.299625	-2.561853
H	-2.798637	-2.608410	-4.423217
H	-0.602307	-3.751249	-4.616016

TS 71

B3LYP/6-31G(d) = -3573.931924
B3LYP-D3(BJ)/def2-TZVPP/IEFPCM(propanonitrile) = -3575.50815
B3LYP-D3(BJ)/def2-TZVPP/IEFPCM(propanonitrile)//B3LYP-D3(BJ)/6-31G(d) Free Energy (Quasiharmonic) = -3574.790882

Frequencies (Top 3 out of 300)

1. -269.3007 cm^{-1}
2. 14.6951 cm^{-1}
3. 15.6271 cm^{-1}

B3LYP/6-31G(d) Molecular Geometry in Cartesian Coordinates

B	-0.458502	0.044247	-0.398657
O	-1.309284	-1.152478	-0.618374
N	-0.047349	0.407181	-1.870105
C	-0.674903	-0.545842	-2.812343
S	0.074704	1.948646	-2.502189
C	-1.425516	-1.508458	-1.897509
O	-2.063160	-2.472127	-2.277154
H	0.647375	-0.573838	-4.500745
H	-1.436945	-0.050815	-3.433482
C	0.270775	-1.301858	-3.779490
C	1.415366	-2.025379	-3.129941
C	2.796357	-1.607505	-3.103381
C	3.523350	-2.592830	-2.379990
N	2.625769	-3.573564	-2.010663
C	1.362091	-3.217785	-2.448336
H	0.513055	-3.860173	-2.256703
C	5.558790	-1.388334	-2.685639
C	3.491007	-0.505110	-3.634374
H	5.438765	-3.253757	-1.596773
H	2.811933	-4.302238	-1.339258
C	4.861324	-0.406190	-3.420054
H	6.629570	-1.281983	-2.533106
H	2.959396	0.261853	-4.191395
C	4.901313	-2.493755	-2.158389
H	5.408017	0.440589	-3.826907
C	1.248847	2.770884	-1.431454
O	-1.212041	2.672085	-2.393453
O	0.659663	1.823770	-3.847814
C	3.108654	4.174283	0.119452
C	2.567678	2.312849	-1.357784
C	0.850369	3.925372	-0.758806
C	1.784578	4.617360	0.013258
C	3.482462	3.014177	-0.578864
H	2.871254	1.418159	-1.891066
H	-0.173138	4.271270	-0.847991
H	1.478727	5.518706	0.538973
H	4.504693	2.652065	-0.507151
C	4.110162	4.905217	0.980024
H	4.337607	4.321178	1.878890
H	5.054570	5.062954	0.446613
H	3.731458	5.881936	1.296814
H	-0.368396	-2.005123	-4.327470
C	0.737987	-0.282130	0.639556
C	1.381041	0.704107	1.402277
C	1.159718	-1.606745	0.827165
C	2.410732	0.385464	2.292814
C	2.179941	-1.933954	1.724110
H	0.675329	-2.399432	0.269983
C	2.818941	-0.937308	2.460555
H	3.612889	-1.186004	3.156433
C	2.543812	-3.367397	1.983242
C	3.129922	1.466588	3.048470
F	2.189496	-4.192703	0.963777
F	1.925669	-3.856275	3.092244
F	3.869709	-3.531182	2.184665
F	3.470658	1.070742	4.298133
F	2.390095	2.592027	3.172653
F	4.287986	1.830162	2.436139
O	-1.305471	1.138202	0.267917
C	-2.514365	1.436082	-0.105808
C	-2.997887	2.805711	0.174121
C	-4.071421	3.325233	-0.562727
C	-2.390576	3.596136	1.161751
C	-4.533467	4.615898	-0.316887
H	-4.525272	2.725482	-1.347799
C	-2.858354	4.884598	1.408833
H	-1.548121	3.196559	1.716914
C	-3.931143	5.396535	0.672811
H	-5.356099	5.016315	-0.902401
H	-2.383214	5.493813	2.172813
H	-4.291593	6.403234	0.865330
H	-2.900921	0.956683	-1.004029
C	-3.944478	0.449782	1.145142
H	-3.787978	1.112235	1.984622
H	-4.827264	0.674600	0.556659
C	-3.593727	-0.893055	1.306921
C	-2.611594	-1.354519	2.298484
C	-2.038551	-0.477949	3.240071
C	-2.218947	-2.707798	2.307865
C	-1.105018	-0.942102	4.161412
H	-2.313359	0.570271	3.258262
C	-1.282615	-3.165293	3.226576
H	-2.640152	-3.380780	1.571357
C	-0.721793	-2.284377	4.155869
H	-0.667712	-0.252523	4.877257
H	-0.970088	-4.204828	3.209372
H	0.022158	-2.642274	4.860993
O	-4.078051	-1.848552	0.537727
Si	-5.373229	-2.059405	-0.587428
C	-5.150369	-3.839126	-1.111618
H	-5.241935	-4.526679	-0.263365
H	-5.894842	-4.128930	-1.862822
H	-4.154130	-3.956980	-1.551655
C	-5.207770	-0.892141	-2.050614
H	-4.264699	-1.075945	-2.575006
H	-6.022137	-1.087461	-2.760257
H	-5.264661	0.167202	-1.777903
H	1.076419	1.740381	1.307811
C	-6.968211	-1.760645	0.368808
H	-7.043849	-2.431861	1.231789
H	-7.049969	-0.731509	0.736413
H	-7.836063	-1.949861	-0.275114

TS 72

B3LYP/6-31G(d) = -3573.9322
B3LYP-D3(BJ)/def2-TZVPP/IEFPCM(propanonitrile) = -3575.508385
B3LYP-D3(BJ)/def2-TZVPP/IEFPCM(propanonitrile)//B3LYP-D3(BJ)/6-31G(d) Free Energy (Quasiharmonic) = -3574.79088

Frequencies (Top 3 out of 300)

1. -277.5299 cm^{-1}
2. 11.1581 cm^{-1}
3. 12.4881 cm^{-1}

B3LYP/6-31G(d) Molecular Geometry in Cartesian Coordinates

B	-0.175235	-0.101940	-0.936958
O	0.099716	0.848130	-2.047410
N	-0.823170	-1.298188	-1.713766
C	-0.851735	-1.005382	-3.165389
S	-0.636945	-2.921040	-1.374281
C	-0.249316	0.393821	-3.260333
O	-0.097885	1.027703	-4.280471
H	-2.570691	-2.095497	-3.851151
H	-0.183019	-1.679040	-3.721646
C	-2.234726	-1.056660	-3.863712
C	-3.281731	-0.159043	-3.269258
C	-4.364858	-0.547621	-2.399173
C	-5.083599	0.633065	-2.064749
N	-4.480229	1.680018	-2.729922
C	-3.392052	1.201480	-3.437102
H	-2.778700	1.870460	-4.025450
C	-6.602823	-0.612386	-0.713660
C	-4.808076	-1.780142	-1.884876
H	-6.718677	1.534752	-0.957022
H	-4.678735	2.654494	-2.565848
C	-5.919922	-1.800523	-1.050339
H	-7.464792	-0.660125	-0.053401
H	-4.281834	-2.698480	-2.132046
C	-6.195826	0.617876	-1.216024
H	-6.273302	-2.747508	-0.650361
C	-1.068701	-3.066312	0.356243
O	-1.624731	-3.657694	-2.179817
O	0.779692	-3.334500	-1.510136
C	-1.764833	-3.480765	3.035430
C	-2.375370	-2.800339	0.776058
C	-0.110969	-3.546692	1.248805
C	-0.467161	-3.748817	2.582646
C	-2.708973	-3.003422	2.111420
H	-3.115266	-2.429561	0.074532
H	0.890845	-3.762279	0.895325
H	0.276225	-4.124409	3.281530
H	-3.720200	-2.784236	2.444282
C	-2.141603	-3.675047	4.483957
H	-2.265463	-2.705392	4.978848
H	-3.091244	-4.213760	4.580026
H	-1.375344	-4.237277	5.026640
O	1.153114	-0.450670	-0.255872
C	2.206270	-0.893364	-0.888618
H	2.060392	-1.259222	-1.904854
H	-2.046298	-0.784924	-4.909537
C	3.209113	-1.632416	-0.088548
C	4.010075	-2.598092	-0.715653
C	3.332659	-1.422551	1.293082
C	4.926656	-3.341176	0.027122
H	3.881562	-2.794102	-1.776920
C	4.251786	-2.164637	2.032083
H	2.696334	-0.689029	1.777360
C	5.051796	-3.122920	1.402126
H	5.527695	-4.102730	-0.461804
H	4.339081	-2.001981	3.102812
H	5.759523	-3.708144	1.983145
C	-1.052934	0.615117	0.219790
C	-1.837147	1.735200	-0.081953
C	-1.016986	0.208628	1.564368
C	-2.540397	2.431807	0.908854
H	-1.877197	2.092745	-1.105832
C	-1.738055	0.881411	2.552142
H	-0.404570	-0.638565	1.851193
C	-2.501265	2.006670	2.233200
H	-3.035277	2.548791	3.004619
C	-1.735035	0.390650	3.971984
C	-3.289995	3.676427	0.531284
F	-2.838080	-0.355183	4.250102
F	-1.735191	1.411523	4.861780
F	-2.494853	4.540908	-0.154747
F	-0.664386	-0.385717	4.252094
F	-3.769932	4.342911	1.601297
F	-4.352779	3.424853	-0.279700
C	3.236269	0.724095	-1.666526
H	2.361467	1.147711	-2.142035
H	3.910482	0.182763	-2.320803
C	3.809497	1.399812	-0.587861
O	5.034640	1.095269	-0.172610
Si	6.595984	0.908542	-0.878126
C	7.150349	2.637835	-1.362745
H	8.166410	2.615449	-1.775741
H	7.154094	3.314632	-0.501219
H	6.494364	3.071064	-2.126875
C	7.591391	0.188240	0.535514
H	8.631916	0.017919	0.234007
H	7.599781	0.859288	1.401573
H	7.170804	-0.771221	0.856322
C	6.591381	-0.237511	-2.368504
H	6.146796	-1.209435	-2.130650
H	6.064273	0.186048	-3.230353
H	7.628563	-0.416497	-2.680745
C	3.096239	2.362893	0.257816
C	1.826858	2.861731	-0.100925
C	3.693678	2.818143	1.452844
C	1.179838	3.788235	0.712587
H	1.337140	2.530439	-1.008263
C	3.041020	3.740650	2.260589
H	4.663985	2.430657	1.739866
C	1.783416	4.229639	1.891422
H	0.197985	4.154309	0.428872
H	3.508763	4.078887	3.180828
H	1.274833	4.951757	2.524225

TS 73

B3LYP/6-31G(d) = -3573.931171
B3LYP-D3(BJ)/def2-TZVPP/IEFPCM(propanonitrile) = -3575.5072
B3LYP-D3(BJ)/def2-TZVPP/IEFPCM(propanonitrile)//B3LYP-D3(BJ)/6-31G(d) Free Energy (Quasiharmonic) = -3574.790588

Frequencies (Top 3 out of 300)

1. -278.9143 cm^{-1}
2. 6.3254 cm^{-1}
3. 10.8211 cm^{-1}

B3LYP/6-31G(d) Molecular Geometry in Cartesian Coordinates

B	0.055806	-0.187520	0.223132
O	-0.139939	0.028192	1.688032
N	0.795151	-1.562099	0.236619
C	0.866859	-2.109791	1.602752
S	0.827173	-2.610073	-1.053695
C	0.181563	-1.030481	2.438375
O	-0.075897	-1.123999	3.619553
H	2.636746	-3.336364	1.592107
H	0.257170	-3.019072	1.681458
C	2.268102	-2.481543	2.168958

C	3.316760	-1.405590	2.199798
C	3.521458	-0.378413	3.201077
C	4.648815	0.385842	2.789640
N	5.123940	-0.175154	1.621175
C	4.310471	-1.233454	1.266377
H	4.518936	-1.804266	0.373119
C	4.468931	1.793497	4.703971
C	2.884352	-0.038946	4.409665
H	5.980169	2.055466	3.176951
H	5.787812	0.262814	1.001308
C	3.363488	1.037623	5.145175
H	4.813410	2.635929	5.298100
H	2.016815	-0.595074	4.749147
C	5.126813	1.477502	3.521820
H	2.872942	1.308321	6.076471
C	2.548882	-2.859739	-1.512106
O	0.192886	-1.918544	-2.182398
O	0.319137	-3.919692	-0.602218
C	5.220253	-3.246829	-2.266779
C	3.223000	-1.853366	-2.207979
C	3.185094	-4.061477	-1.203041
C	4.516301	-4.245072	-1.580022
C	4.552607	-2.049697	-2.571354
H	2.712979	-0.929834	-2.458396
H	2.636612	-4.841118	-0.685316
H	5.013642	-5.181572	-1.339153
H	5.080648	-1.257959	-3.096787
C	6.651208	-3.463301	-2.698823
H	7.156081	-4.197844	-2.063274
H	7.225458	-2.531297	-2.666141
H	6.697840	-3.836047	-3.730672
H	2.077911	-2.853009	3.183951
C	0.807547	1.048746	-0.489706
C	2.205513	1.152662	-0.466676
C	0.104726	2.089587	-1.113365
C	2.871497	2.233070	-1.055831
H	2.783077	0.367326	0.009635
C	0.761100	3.188420	-1.674357
H	-0.976447	2.042732	-1.166536
C	2.153248	3.266063	-1.657196
H	2.665401	4.105062	-2.112291
C	-0.051712	4.316237	-2.243937
C	4.372382	2.284414	-1.014111
F	0.660146	5.091273	-3.091492
F	-0.518860	5.139513	-1.264984
F	-1.138473	3.873604	-2.916406
F	4.886111	3.187488	-1.875986
F	4.928531	1.078856	-1.325470
F	4.847081	2.602133	0.216658
O	-1.349537	-0.236443	-0.396757
C	-2.189906	-1.210310	-0.188271
C	-3.185831	-1.499690	-1.246145
C	-3.752339	-2.780639	-1.318842
C	-3.528577	-0.537516	-2.206839
C	-4.656534	-3.094656	-2.332660
H	-3.453075	-3.541953	-0.602066
C	-4.435454	-0.852501	-3.215928
H	-3.060604	0.440061	-2.170120
C	-5.003840	-2.128159	-3.280362
H	-5.073057	-4.096175	-2.395242
H	-4.689117	-0.106257	-3.963683
H	-5.700284	-2.374002	-4.077511
H	-1.799749	-2.098511	0.317032
C	-3.281786	-0.752781	1.493241
H	-3.691241	-1.728778	1.729548
H	-2.423623	-0.456165	2.081851
C	-4.189108	0.231328	1.089763
C	-3.840541	1.642702	0.910484
C	-2.625507	2.170054	1.396018
C	-4.726505	2.495700	0.217163
C	-2.306421	3.507005	1.174500
H	-1.919355	1.545250	1.929542
C	-4.397325	3.827430	-0.000934
H	-5.657455	2.093217	-0.163982
C	-3.183167	4.336050	0.472092
H	-1.360882	3.897065	1.537324
H	-5.080393	4.469669	-0.549419
H	-2.915507	5.371283	0.283826
O	-5.406526	-0.098762	0.676838
Si	-6.711163	-1.080796	1.229171
C	-7.856958	-1.092605	-0.252168
H	-8.181649	-0.079503	-0.514120
H	-7.355204	-1.522286	-1.126270
H	-8.754049	-1.689629	-0.048656
C	-7.434063	-0.182407	2.713183
H	-7.749208	0.833979	2.452549
H	-8.311432	-0.717077	3.097665
H	-6.708545	-0.109453	3.531824
C	-6.172385	-2.820867	1.695395
H	-5.631022	-3.310113	0.879207
H	-5.546919	-2.847453	2.594294
H	-7.065854	-3.423111	1.905863

TS 74

B3LYP/6-31G(d) = -3573.929814
B3LYP-D3(BJ)/def2-TZVPP/IEFPCM(propanonitrile) = -3575.507076
B3LYP-D3(BJ)/def2-TZVPP/IEFPCM(propanonitrile)//B3LYP-D3(BJ)/6-31G(d) Free Energy (Quasiharmonic) = -3574.790481

Frequencies (Top 3 out of 300)

1. -275.8516 cm^{-1}
2. 3.0049 cm^{-1}
3. 11.6844 cm^{-1}

B3LYP/6-31G(d) Molecular Geometry in Cartesian Coordinates

B	-0.890373	0.613658	-0.948013
O	-0.822273	1.851438	-1.762863
N	-0.709319	1.212674	0.500772
C	-0.812897	2.683067	0.450033
S	-1.101519	0.424073	1.897941
C	-0.763059	2.975872	-1.055144
O	-0.710977	4.089568	-1.529322
H	-0.046382	3.439452	2.309333
H	-1.812331	3.009052	0.782231
C	0.206887	3.529576	1.250463
C	1.673154	3.237882	1.080465
C	2.602940	3.820345	0.134998
C	3.899140	3.333244	0.463996
N	3.751480	2.469198	1.533319
C	2.419450	2.436734	1.909736
H	2.092315	1.847243	2.753510
C	4.888295	4.619271	-1.282687
C	2.473742	4.719602	-0.939899
H	6.031955	3.348855	0.045691
H	4.506444	2.044261	2.048217
C	3.613285	5.108187	-1.633314
H	5.760459	4.948075	-1.841779
H	1.493709	5.083091	-1.232093
C	5.048994	3.723265	-0.231544
H	3.521368	5.800229	-2.465935
C	-2.859749	0.616770	2.212480
O	-0.411005	1.084848	3.022167
O	-0.851463	-1.009955	1.649464
C	-5.614981	0.997278	2.558665
C	-3.326830	1.790940	2.809122
C	-3.750115	-0.388507	1.825096
C	-5.115590	-0.190836	2.004564
C	-4.700016	1.973194	2.972755

H	-2.626293	2.540443	3.161625
H	-3.376580	-1.307879	1.390042
H	-5.808786	-0.967373	1.692241
H	-5.064317	2.890241	3.428999
C	-7.103471	1.216088	2.676198
H	-7.335675	2.114806	3.255616
H	-7.598020	0.363722	3.156446
H	-7.549476	1.330709	1.680449
O	0.253677	-0.272884	-1.373135
C	1.507155	-0.145477	-0.989946
H	1.691824	0.524271	-0.146065
H	0.003041	4.565775	0.955302
C	2.516423	-0.119913	-2.078195
C	3.769917	0.466102	-1.853388
C	2.204880	-0.617265	-3.354081
C	4.696287	0.562368	-2.890496
H	4.004325	0.878344	-0.876507
C	3.138939	-0.533578	-4.383194
H	1.219422	-1.035588	-3.531137
C	4.385883	0.057181	-4.154202
H	5.652207	1.046228	-2.712954
H	2.888419	-0.910656	-5.371026
H	5.106709	0.135929	-4.963699
C	-2.249128	-0.203102	-1.262949
C	-3.445353	0.477815	-1.540826
C	-2.299470	-1.602264	-1.258114
C	-4.639094	-0.206243	-1.778693
H	-3.448004	1.562437	-1.581525
C	-3.490045	-2.295470	-1.499188
H	-1.394201	-2.165174	-1.059267
C	-4.670044	-1.601858	-1.759215
H	-5.593004	-2.136334	-1.953700
C	-3.506455	-3.793083	-1.384440
C	-5.926388	0.544191	-1.977913
F	-3.603154	-4.187567	-0.088524
F	-4.548611	-4.350555	-2.040246
F	-6.731303	-0.061821	-2.881056
F	-2.375689	-4.353952	-1.872686
F	-5.729271	1.811034	-2.393834
F	-6.641797	0.617344	-0.821023
C	1.772985	-1.867191	-0.046340
H	1.619799	-2.480651	-0.926400
H	0.907212	-1.757304	0.599796
C	3.017109	-1.955602	0.596722
O	3.142797	-1.535972	1.849955
Si	2.618422	-2.183274	3.379226
C	4.110053	-3.132834	4.029691
H	3.875422	-3.583000	5.002513
H	4.978532	-2.479118	4.171885
H	4.405692	-3.944156	3.354570
C	2.238321	-0.697460	4.446943
H	1.368315	-0.145358	4.075661
H	2.017324	-1.024284	5.471074
H	3.093036	-0.012692	4.502002
C	1.155781	-3.325174	3.126298
H	0.271635	-2.780779	2.781012
H	1.374426	-4.127821	2.412256
H	0.908095	-3.800841	4.084174
C	4.261214	-2.369422	-0.063116
C	4.260962	-2.953709	-1.346618
C	5.495321	-2.190815	0.596199
C	5.453851	-3.342735	-1.945997
H	3.330853	-3.107777	-1.879587
C	6.685262	-2.578669	-0.008165
H	5.504420	-1.735457	1.579127
C	6.668035	-3.156778	-1.280858
H	5.435929	-3.791653	-2.934410
H	7.628119	-2.430150	0.510286
H	7.598106	-3.462467	-1.751983

TS 75

B3LYP/6-31G(d) = -3573.932618
B3LYP-D3(BJ)/def2-TZVPP/IEFPCM(propanonitrile) = -3575.507761
B3LYP-D3(BJ)/def2-TZVPP/IEFPCM(propanonitrile)//B3LYP-D3(BJ)/6-31G(d) Free Energy (Quasiharmonic) = -3574.790459

Frequencies (Top 3 out of 300)

1. -268.8871 cm^{-1}
2. 11.1991 cm^{-1}
3. 15.3586 cm^{-1}

B3LYP/6-31G(d) Molecular Geometry in Cartesian Coordinates

B	-0.503746	-0.235022	-0.020088
O	-0.067662	-0.813042	1.275351
N	-1.327493	-1.420686	-0.593002
C	-1.327229	-2.572600	0.314661
S	-2.191271	-1.438529	-1.998391
C	-0.578398	-2.024942	1.528405
O	-0.451572	-2.596144	2.591201
H	-1.257755	-4.244058	-1.043674
H	-2.349112	-2.820239	0.638760
C	-0.670426	-3.889984	-0.192625
C	0.778048	-3.781808	-0.569498
C	1.925174	-4.077291	0.259230
C	3.084315	-3.806250	-0.520732
N	2.651974	-3.390951	-1.764276
C	1.272113	-3.360441	-1.780894
H	0.728347	-3.043292	-2.659649
C	4.512607	-4.439058	1.280295
C	2.092797	-4.549406	1.574600
H	5.249533	-3.734015	-0.629266
H	3.246562	-2.978006	-2.466017
C	3.379233	-4.728076	2.068948
H	5.505785	-4.580548	1.698494
H	1.228420	-4.749251	2.201082
C	4.380936	-3.972670	-0.022206
H	3.517244	-5.092349	3.083800
C	-3.892075	-1.767077	-1.498393
O	-1.794027	-2.581276	-2.838522
O	-2.133111	-0.076309	-2.544037
C	-6.539421	-2.259826	-0.715578
C	-4.654775	-0.734893	-0.946064
C	-4.438793	-3.038392	-1.671872
C	-5.755947	-3.275936	-1.275984
C	-5.967062	-0.987653	-0.557837
H	-4.226100	0.254635	-0.833935
H	-3.841780	-3.821708	-2.126173
H	-6.181541	-4.267440	-1.410879
H	-6.558777	-0.182485	-0.128267
C	-7.974311	-2.513669	-0.318143
H	-8.235534	-1.981353	0.603104
H	-8.164373	-3.579976	-0.160278
H	-8.666251	-2.169092	-1.098051
H	-0.797735	-4.619224	0.616352
C	0.723888	0.331264	-0.899359
C	0.541782	1.317967	-1.881961
C	2.028353	-0.138867	-0.694548
C	1.615887	1.821402	-2.617495
H	-0.453962	1.696499	-2.074629
C	3.108498	0.360416	-1.432706
H	2.210134	-0.897635	0.058861
C	2.909203	1.345631	-2.398449
H	3.747235	1.751033	-2.954545
C	4.493340	-0.176982	-1.213558
C	1.395964	2.873397	-3.668620
F	5.445803	0.754447	-1.451710
F	4.778899	-1.222723	-2.047168
F	4.681898	-0.631396	0.044072

F	0.249989	3.564625	-3.474563
F	1.336126	2.350926	-4.914197
F	2.407605	3.782673	-3.683563
O	-1.425056	0.969985	0.291390
C	-1.958525	1.178959	1.458920
C	-3.213383	1.972232	1.483360
C	-3.613114	2.714561	0.360717
C	-4.027037	1.958154	2.626643
C	-4.804831	3.438023	0.390826
H	-2.997085	2.693087	-0.532401
C	-5.218094	2.679894	2.651627
H	-3.727536	1.374168	3.494109
C	-5.607300	3.425434	1.534831
H	-5.111969	4.005388	-0.483278
H	-5.845363	2.657917	3.538357
H	-6.536227	3.988602	1.554382
H	-1.890455	0.369861	2.186171
C	-0.797518	2.394162	2.710532
H	-1.359459	3.313305	2.616702
H	-0.974460	1.853028	3.632596
C	0.527406	2.428290	2.243522
C	0.969446	3.366896	1.201279
C	2.340018	3.476151	0.896272
C	0.051452	4.171220	0.496760
C	2.778085	4.367897	-0.075966
H	3.050206	2.850956	1.423727
C	0.493882	5.050339	-0.485430
H	-1.011784	4.094470	0.694455
C	1.857319	5.154558	-0.772543
H	3.837598	4.439917	-0.302692
H	-0.225352	5.647951	-1.037015
H	2.198711	5.833535	-1.547921
O	1.445689	1.586318	2.649037
Si	1.870911	0.595769	4.018579
C	2.821989	1.797008	5.114808
H	2.191802	2.630060	5.448217
H	3.192051	1.283881	6.011026
H	3.687920	2.218119	4.591829
C	0.394573	-0.122020	4.928002
H	-0.280582	0.630454	5.350029
H	-0.163332	-0.814407	4.289005
H	0.782282	-0.710371	5.770987
C	2.954478	-0.738776	3.289581
H	2.346704	-1.531977	2.843243
H	3.574334	-1.194794	4.071586
H	3.624324	-0.349034	2.515984

TS 76

B3LYP/6-31G(d) = -3573.931401
B3LYP-D3(BJ)/def2-TZVPP/IEFPCM(propanonitrile) = -3575.506943
B3LYP-D3(BJ)/def2-TZVPP/IEFPCM(propanonitrile)//B3LYP-D3(BJ)/6-31G(d) Free Energy (Quasiharmonic) = -3574.790433

Frequencies (Top 3 out of 300)

1. -276.1406 cm^{-1}
2. 7.3316 cm^{-1}
3. 13.8734 cm^{-1}

B3LYP/6-31G(d) Molecular Geometry in Cartesian Coordinates

B	0.455777	0.396306	-0.286300
O	0.060997	-0.154726	-1.617471
N	0.779453	1.866274	-0.652578
C	0.785070	2.044542	-2.110802
S	0.669000	3.197151	0.310985
C	0.218368	0.718456	-2.619291
O	-0.060680	0.470703	-3.774124
H	2.474827	3.339187	-2.409226
H	0.082067	2.832351	-2.418380
C	2.159470	2.354285	-2.764103
C	3.230202	1.334345	-2.497843
C	4.249033	1.380070	-1.474396
C	5.013187	0.187791	-1.591655
N	4.497612	-0.530292	-2.653086
C	3.415884	0.152810	-3.176257
H	2.860702	-0.251344	-4.012309
C	6.378048	0.817156	0.257173
C	4.582825	2.303418	-0.466472
H	6.631009	-1.040186	-0.827450
H	4.720038	-1.494765	-2.845242
C	5.640440	2.012565	0.385683
H	7.191054	0.614697	0.949249
H	4.003541	3.212995	-0.339224
C	6.076373	-0.110511	-0.732401
H	5.897837	2.711290	1.176930
C	-0.946287	3.940459	-0.018168
O	1.648014	4.195653	-0.144212
O	0.661274	2.731585	1.704113
C	-3.451789	5.097502	-0.545702
C	-2.081068	3.476813	0.652718
C	-1.049695	4.987548	-0.935194
C	-2.298838	5.553502	-1.195368
C	-3.318939	4.057540	0.387819
H	-1.983825	2.685317	1.386143
H	-0.154658	5.370930	-1.412996
H	-2.373550	6.372558	-1.906652
H	-4.198637	3.711162	0.926834
C	-4.804106	5.699664	-0.845320
H	-5.342981	5.108132	-1.597644
H	-4.711488	6.717497	-1.237108
H	-5.434284	5.736647	0.049937
H	1.969262	2.439278	-3.841294
C	1.628481	-0.490723	0.385281
C	2.113607	-1.642007	-0.249370
C	2.196884	-0.150512	1.624735
C	3.123893	-2.423483	0.321491
H	1.708085	-1.929547	-1.213271
C	3.203681	-0.929324	2.198552
H	1.853308	0.740514	2.140928
C	3.674892	-2.072508	1.551846
H	4.451878	-2.678546	2.003024
C	3.835148	-0.502386	3.496105
C	3.667416	-3.605482	-0.425606
F	4.322761	-1.561110	4.190142
F	2.955989	0.132643	4.302627
F	4.872151	0.343419	3.301535
F	4.151061	-4.560099	0.398077
F	4.702399	-3.262877	-1.252713
F	2.736438	-4.187095	-1.216962
O	-0.769852	0.358583	0.627082
C	-1.526649	-0.698979	0.747746
C	-2.170251	-0.895223	2.069593
C	-2.052174	0.087252	3.065916
C	-2.820548	-2.103419	2.366145
C	-2.597531	-0.133148	4.329768
H	-1.486762	0.989348	2.856319
C	-3.357235	-2.321931	3.631993
H	-2.897646	-2.876303	1.605015
C	-3.252817	-1.333392	4.614566
H	-2.491059	0.625587	5.099874
H	-3.851532	-3.263239	3.854572
H	-3.668101	-1.505476	5.603789
H	-1.190188	-1.612988	0.251010
C	-2.972706	-0.315494	-0.625698
H	-3.236784	0.668421	-0.258025
H	-2.264948	-0.309398	-1.446319
C	-3.973417	-1.294115	-0.659032
C	-5.198578	-1.241559	0.153536
C	-5.449403	-0.181830	1.048076

C	-6.158257	-2.267085	0.035457
C	-6.620829	-0.151939	1.797132
H	-4.724908	0.613735	1.174292
C	-7.330362	-2.229761	0.782465
H	-5.972089	-3.086390	-0.648607
C	-7.565523	-1.172685	1.665778
H	-6.796379	0.668383	2.486633
H	-8.062154	-3.025495	0.677419
H	-8.481402	-1.144305	2.249506
O	-3.842294	-2.399616	-1.370589
Si	-2.960967	-2.902757	-2.796531
C	-2.984716	-1.519798	-4.063271
H	-2.146432	-0.823722	-3.947433
H	-2.899509	-1.960919	-5.064884
H	-3.924250	-0.955349	-4.038646
C	-1.232679	-3.405166	-2.280467
H	-0.737299	-3.919589	-3.113891
H	-0.622751	-2.531113	-2.031006
H	-1.239056	-4.094815	-1.428668
C	-3.989134	-4.378563	-3.334775
H	-4.049762	-5.134471	-2.543571
H	-3.542617	-4.856112	-4.215390
H	-5.010432	-4.082706	-3.600478

TS 77

B3LYP/6-31G(d) = -3573.931862
B3LYP-D3(BJ)/def2-TZVPP/IEFPCM(propanonitrile) = -3575.507876
B3LYP-D3(BJ)/def2-TZVPP/IEFPCM(propanonitrile)//B3LYP-D3(BJ)/6-31G(d) Free Energy (Quasiharmonic) = -3574.790405

Frequencies (Top 3 out of 300)

1. -281.4187 cm^{-1}
2. 9.3179 cm^{-1}
3. 11.6484 cm^{-1}

B3LYP/6-31G(d) Molecular Geometry in Cartesian Coordinates

B	-0.654272	-0.830242	0.771286
O	-0.534039	-1.876909	1.818481
N	-0.675904	-1.726651	-0.525014
C	-0.799929	-3.147281	-0.156990
S	-1.196846	-1.232606	-2.016829
C	-0.570976	-3.125240	1.359588
O	-0.463700	-4.117084	2.047823
H	-0.318412	-4.359212	-1.866259
H	-1.836860	-3.496357	-0.294711
C	0.103540	-4.182723	-0.874896
C	1.566442	-3.867787	-1.032189
C	2.654112	-4.139746	-0.114117
C	3.862309	-3.767650	-0.770182
N	3.515346	-3.253978	-2.007395
C	2.141495	-3.349704	-2.166104
H	1.658818	-3.023107	-3.076253
C	5.161148	-4.477562	1.100500
C	2.729590	-4.676979	1.185060
H	6.032060	-3.658427	-0.704822
H	4.163709	-3.071808	-2.758180
C	3.977487	-4.839648	1.775167
H	6.121960	-4.626885	1.586108
H	1.822987	-4.944243	1.719313
C	5.120139	-3.934034	-0.180010
H	4.043728	-5.253349	2.777598
C	-2.992470	-1.301754	-2.056766
O	-0.733450	-2.204962	-3.021430
O	-0.802017	0.182595	-2.137324
C	-5.791747	-1.449542	-1.992662
C	-3.627911	-2.519716	-2.312119
C	-3.740862	-0.148391	-1.808470
C	-5.129617	-0.230630	-1.782693
C	-5.020717	-2.584664	-2.272196
H	-3.042570	-3.397651	-2.563719
H	-3.236601	0.795358	-1.637313
H	-5.710914	0.664452	-1.577719
H	-5.514859	-3.534066	-2.463045
C	-7.295551	-1.527341	-1.891940
H	-7.672775	-2.500689	-2.220080
H	-7.614356	-1.372558	-0.853871
H	-7.779268	-0.752738	-2.498471
H	-0.016643	-5.108571	-0.299481
C	-1.911797	0.138548	1.072591
C	-1.826647	1.527235	0.918583
C	-3.128586	-0.380115	1.544296
C	-2.906837	2.365428	1.218157
C	-4.214662	0.448105	1.831491
H	-3.231986	-1.448946	1.702556
C	-4.110126	1.831983	1.673796
H	-4.945203	2.479039	1.917171
C	-5.535520	-0.130067	2.257129
C	-2.767950	3.844695	1.006364
F	-6.120341	0.609038	3.228149
F	-5.427010	-1.392333	2.715920
F	-6.420652	-0.161278	1.221930
F	-3.707945	4.557845	1.659496
F	-2.869019	4.181314	-0.309243
F	-1.556993	4.303394	1.418055
O	0.568186	0.057504	0.875847
C	1.783559	-0.389843	0.671820
C	2.817706	0.008617	1.659968
C	4.023593	-0.703556	1.730040
C	2.574748	1.051747	2.566246
C	4.976775	-0.371618	2.691396
H	4.205718	-1.533109	1.051920
C	3.531150	1.379444	3.524607
H	1.624289	1.573204	2.527723
C	4.734853	0.671599	3.587630
H	5.901160	-0.939509	2.748744
H	3.332408	2.179060	4.233235
H	5.475335	0.923739	4.342142
H	1.868291	-1.413552	0.298599
C	2.350502	0.304405	-1.113888
H	1.400906	0.095031	-1.596807
H	3.160035	-0.391203	-1.306380
C	2.681777	1.651717	-0.954398
C	4.051241	2.118008	-0.678787
C	5.172370	1.419201	-1.165010
C	4.256152	3.280328	0.089014
C	6.459283	1.877567	-0.900024
H	5.034223	0.536970	-1.781126
C	5.544700	3.726234	0.365145
H	3.398011	3.805156	0.492953
C	6.648850	3.029251	-0.132347
H	7.315623	1.338575	-1.294730
H	5.689634	4.614617	0.972934
H	7.654541	3.382680	0.077429
O	1.710206	2.548371	-0.902483
Si	1.232934	3.875169	-1.919389
C	1.055926	5.356308	-0.781017
H	2.003422	5.647401	-0.314598
H	0.690495	6.219054	-1.352494
H	0.324871	5.157025	0.009515
C	-0.396904	3.409755	-2.707590
H	-0.615308	4.086499	-3.543887
H	-0.390007	2.381630	-3.081519
H	-1.218710	3.491409	-1.989729
H	-0.894331	1.958802	0.571330
C	2.585980	4.115665	-3.206583
H	3.551408	4.377089	-2.761485
H	2.300490	4.928793	-3.885596
H	2.724861	3.215912	-3.817542

TS 78

B3LYP/6-31G(d) = -3573.926507
B3LYP-D3(BJ)/def2-TZVPP/IEFPCM(propanonitrile) = -3575.507188
B3LYP-D3(BJ)/def2-TZVPP/IEFPCM(propanonitrile)//B3LYP-D3(BJ)/6-31G(d) Free Energy (Quasiharmonic) = -3574.790277

Frequencies (Top 3 out of 300)

1. -276.7613 cm^{-1}
2. 9.8618 cm^{-1}
3. 14.5453 cm^{-1}

B3LYP/6-31G(d) Molecular Geometry in Cartesian Coordinates

B	0.347467	0.637290	-0.476266
O	0.551896	0.359939	-1.911400
N	0.687750	2.164035	-0.422581
C	1.277043	2.603494	-1.693783
S	0.351231	3.242326	0.756685
C	1.062885	1.384401	-2.597966
O	1.328387	1.352411	-3.779149
H	2.809217	4.024470	-1.175003
H	0.700977	3.432290	-2.129352
C	2.770081	3.044593	-1.658549
C	3.711353	2.107444	-0.958271
C	4.397257	0.953060	-1.494334
C	5.153459	0.384669	-0.429590
N	4.950953	1.173702	0.685570
C	4.066624	2.185593	0.366064
H	3.741504	2.897619	1.111318
C	5.950555	-1.355729	-1.853021
C	4.445102	0.337650	-2.758860
H	6.500660	-1.183925	0.233037
H	5.193392	0.915131	1.630332
C	5.216048	-0.807054	-2.924015
H	6.541871	-2.253555	-2.013113
H	3.863954	0.735588	-3.585575
C	5.932217	-0.765242	-0.593927
H	5.250923	-1.292921	-3.895696
C	-0.862833	4.374553	0.049844
O	1.516466	4.081016	1.087309
O	-0.294107	2.479873	1.845964
C	-2.788162	6.152170	-0.947557
C	-0.711175	5.745773	0.257414
C	-1.964872	3.879379	-0.650193
C	-2.916503	4.768818	-1.142180
C	-1.674876	6.622732	-0.239700
H	0.154916	6.111352	0.797913
H	-2.069935	2.811855	-0.810885
H	-3.775070	4.382563	-1.686758
H	-1.556752	7.691418	-0.076782
C	-3.809480	7.109724	-1.514401
H	-4.816409	6.678799	-1.497404
H	-3.832262	8.049763	-0.953768
H	-3.580541	7.358014	-2.559375
H	3.064109	3.186602	-2.705830
C	1.292385	-0.342743	0.415064
C	1.697424	-0.087572	1.736179
C	1.789114	-1.511354	-0.185380
C	2.550963	-0.962722	2.417699
C	2.614232	-2.404797	0.503702
H	1.541192	-1.710498	-1.221988
C	3.005341	-2.135743	1.813642
H	3.659232	-2.815572	2.347384
C	3.026245	-3.687111	-0.159614
C	3.068413	-0.591965	3.777983
F	4.128177	-4.232338	0.393007
F	2.036837	-4.633671	-0.054037
F	3.253107	-3.540695	-1.480096
F	2.187045	0.142932	4.484559
F	3.382445	-1.677292	4.521568
F	4.211388	0.152447	3.692247
O	-1.143638	0.386680	-0.263173
C	-1.724644	0.101986	0.883258
C	-3.151994	0.518210	1.004450
C	-3.713364	0.708624	2.274093
C	-3.937729	0.746581	-0.134087
C	-5.038231	1.121076	2.405990
H	-3.102074	0.555821	3.160370
C	-5.262002	1.163343	-0.001717
H	-3.492981	0.613283	-1.115526
C	-5.816730	1.348167	1.267848
H	-5.458097	1.281934	3.395043
H	-5.859627	1.353728	-0.889592
H	-6.846239	1.680743	1.370105
H	-1.131842	0.280492	1.781323
C	-1.738742	-1.818229	1.202560
H	-0.672489	-1.985045	1.099741
H	-2.119280	-1.822864	2.220419
C	-2.572594	-2.420408	0.244612
C	-2.084502	-2.873003	-1.061290
C	-2.857726	-2.674827	-2.222584
C	-0.829137	-3.498666	-1.171088
C	-2.373752	-3.078060	-3.462099
H	-3.809374	-2.158837	-2.150931
C	-0.358653	-3.918710	-2.411751
H	-0.229983	-3.682796	-0.287695
C	-1.126044	-3.703223	-3.558208
H	-2.960322	-2.893817	-4.357278
H	0.612940	-4.398052	-2.475441
H	-0.750234	-4.014272	-4.528615
O	-3.866848	-2.426178	0.489694
Si	-5.237612	-3.473106	0.357861
C	-6.443047	-2.649997	-0.820669
H	-6.574886	-1.593757	-0.561297
H	-6.114655	-2.707205	-1.863907
H	-7.424465	-3.136001	-0.756780
C	-5.915120	-3.510406	2.105829
H	-6.129245	-2.495407	2.458720
H	-6.845841	-4.088655	2.155148
H	-5.202657	-3.965248	2.803315
H	1.357093	0.813191	2.237547
C	-4.678789	-5.169702	-0.221250
H	-4.283328	-5.154450	-1.241469
H	-3.906768	-5.588155	0.434681
H	-5.532503	-5.859010	-0.203017

TS 79

B3LYP/6-31G(d) = -3573.931651
B3LYP-D3(BJ)/def2-TZVPP/IEFPCM(propanonitrile) = -3575.506266
B3LYP-D3(BJ)/def2-TZVPP/IEFPCM(propanonitrile)//B3LYP-D3(BJ)/6-31G(d) Free Energy (Quasiharmonic) = -3574.790212

Frequencies (Top 3 out of 300)

1. -282.2578 cm^{-1}
2. 8.8186 cm^{-1}
3. 10.4695 cm^{-1}

B3LYP/6-31G(d) Molecular Geometry in Cartesian Coordinates

B	0.481240	0.662587	-0.333224
O	0.275950	0.333637	-1.766925
N	1.252940	2.001002	-0.434552
C	1.581553	2.308812	-1.831685
S	1.356503	3.188455	0.705521
C	0.854815	1.207439	-2.605341

O	0.808029	1.104223	-3.810920
H	3.548702	3.164211	-1.659535
H	1.142079	3.268872	-2.141774
C	3.089073	2.336533	-2.205481
C	3.839717	1.060205	-1.944183
C	4.675892	0.748262	-0.807255
C	5.172326	-0.569621	-1.000312
N	4.674618	-1.023426	-2.205855
C	3.865843	-0.047895	-2.758487
H	3.373732	-0.210829	-3.707896
C	6.374158	-0.476610	1.054814
C	5.055986	1.452696	0.350183
H	6.367869	-2.213328	-0.236972
H	4.742226	-1.975168	-2.530364
C	5.900494	0.835425	1.263986
H	7.027358	-0.933714	1.793724
H	4.673171	2.451117	0.536523
C	6.015851	-1.197849	-0.077426
H	6.194612	1.368476	2.164232
C	0.127366	4.433916	0.263029
O	2.654548	3.867978	0.574002
O	0.946613	2.592177	1.983158
C	-1.788836	6.357152	-0.446737
C	0.509712	5.551798	-0.480732
C	-1.200040	4.271723	0.665195
C	-2.145329	5.229365	0.308404
C	-0.450743	6.500504	-0.833886
H	1.550489	5.683347	-0.756128
H	-1.481003	3.409923	1.258046
H	-3.177805	5.100407	0.625465
H	-0.151194	7.370398	-1.413690
C	-2.817148	7.405135	-0.801677
H	-2.957117	8.116277	0.023355
H	-3.794024	6.954682	-1.008931
H	-2.515034	7.982013	-1.681658
H	3.125504	2.586679	-3.272956
C	1.192735	-0.564832	0.448723
C	1.312075	-0.598077	1.848186
C	1.677381	-1.672341	-0.261302
C	1.878080	-1.696140	2.501832
H	0.979188	0.256289	2.429035
C	2.244757	-2.772894	0.388817
H	1.609705	-1.674996	-1.344085
C	2.345808	-2.794205	1.778611
H	2.779012	-3.645772	2.289570
C	2.765491	-3.923880	-0.421911
C	1.905668	-1.729181	4.005552
F	2.811276	-5.073845	0.283827
F	4.026183	-3.712044	-0.891361
F	1.997218	-4.156540	-1.518697
F	0.712917	-2.147585	4.510571
F	2.144633	-0.515748	4.539862
F	2.843864	-2.578709	4.482360
O	-0.891373	0.872654	0.314513
C	-2.025490	0.494627	-0.216065
C	-3.245666	0.966468	0.487785
C	-3.197663	1.323608	1.843926
C	-4.451955	1.089802	-0.215787
C	-4.341837	1.801233	2.481251
H	-2.256327	1.243541	2.378914
C	-5.593655	1.569316	0.424042
H	-4.491096	0.821913	-1.269186
C	-5.540745	1.925124	1.773949
H	-4.295932	2.083264	3.529448
H	-6.521663	1.672084	-0.131412
H	-6.429964	2.301959	2.271855
H	-2.080675	0.484468	-1.307349
C	-1.979917	-1.496609	-0.067999
H	-1.679308	-1.554945	0.970771
H	-1.159581	-1.626714	-0.765533
C	-3.212622	-2.057383	-0.427999
C	-4.302620	-2.321981	0.521356
C	-4.121190	-2.191530	1.913159
C	-5.561219	-2.736596	0.039730
C	-5.166326	-2.463300	2.789642
H	-3.163937	-1.886831	2.318676
C	-6.602461	-3.006141	0.920047
H	-5.708075	-2.839526	-1.028738
C	-6.408711	-2.870028	2.297533
H	-5.009137	-2.361570	3.859187
H	-7.566810	-3.323410	0.533863
H	-7.221790	-3.084625	2.985462
O	-3.521419	-2.288193	-1.692315
Si	-2.662648	-2.760919	-3.138568
C	-4.037248	-3.581702	-4.119239
H	-4.859089	-2.883381	-4.314782
H	-3.662653	-3.929804	-5.089489
H	-4.448172	-4.449131	-3.590594
C	-2.019227	-1.238531	-4.013436
H	-1.674548	-1.520635	-5.016925
H	-2.812660	-0.493176	-4.146817
H	-1.174483	-0.757265	-3.510710
C	-1.336645	-4.000922	-2.660838
H	-0.496492	-3.573644	-2.105786
H	-0.923088	-4.445610	-3.575171
H	-1.756623	-4.819155	-2.064076

TS 80

B3LYP/6-31G(d) = -3573.933897
B3LYP-D3(BJ)/def2-TZVPP/IEFPCM(propanonitrile) = -3575.507668
B3LYP-D3(BJ)/def2-TZVPP/IEFPCM(propanonitrile)//B3LYP-D3(BJ)/6-31G(d) Free Energy (Quasiharmonic) = -3574.790176

Frequencies (Top 3 out of 300)

1. -274.5734 cm^{-1}
2. 7.4877 cm^{-1}
3. 13.5943 cm^{-1}

B3LYP/6-31G(d) Molecular Geometry in Cartesian Coordinates

B	-0.064249	0.453560	-0.440530
O	-0.242760	-0.316940	-1.711090
N	0.998611	1.503950	-0.891500
C	1.282011	1.378340	-2.331460
S	1.185061	2.987800	-0.161420
C	0.374251	0.227380	-2.758080
O	0.212061	-0.142040	-3.905500
H	3.317111	2.050599	-2.559300
H	0.951351	2.276400	-2.867980
C	2.759751	1.129059	-2.757400
C	3.471791	-0.030631	-2.120330
C	3.407350	-1.434621	-2.471640
C	4.256530	-2.130411	-1.565930
N	4.834700	-1.190401	-0.737150
C	4.346191	0.059459	-1.064200
H	4.678291	0.933369	-0.522280
C	3.742410	-4.219091	-2.591960
C	2.739530	-2.173301	-3.467220
H	5.066440	-4.026991	-0.890840
H	5.336520	-1.403081	0.111240
C	2.913980	-3.550781	-3.516780
H	3.851300	-5.298811	-2.652450
H	2.083910	-1.676010	-4.174450
C	4.425550	-3.518271	-1.605970
H	2.400010	-4.126760	-4.282000
C	2.896311	3.104259	0.387110
O	0.364261	2.974670	1.055010
O	1.003671	4.038840	-1.178820
C	5.549751	3.301769	1.271670

C	3.286981	2.444839	1.554810
C	3.808341	3.875009	-0.333390
C	5.128051	3.965269	0.111420
C	4.608711	2.540469	1.982890
H	2.564391	1.865989	2.119150
H	3.479291	4.405129	-1.220840
H	5.839501	4.566079	-0.450150
H	4.913421	2.013709	2.883810
C	6.971271	3.430109	1.765820
H	7.663451	3.648519	0.946250
H	7.307951	2.513749	2.262140
H	7.061301	4.245489	2.495820
H	2.732271	1.011139	-3.848220
C	0.308130	-0.495480	0.809200
C	1.631650	-0.781300	1.166480
C	-0.699540	-1.121150	1.556560
C	1.935340	-1.634390	2.234180
H	2.437880	-0.322690	0.604660
C	-0.405950	-2.001140	2.599670
H	-1.735830	-0.918990	1.315200
C	0.918340	-2.259260	2.955510
H	1.150650	-2.923310	3.779790
C	-1.527850	-2.732170	3.277750
C	3.372570	-1.909481	2.574540
F	-1.883790	-3.852220	2.589000
F	-2.652510	-1.977910	3.367810
F	-1.210450	-3.132360	4.526810
F	3.528330	-2.365191	3.835610
F	4.145900	-0.795721	2.453700
F	3.931290	-2.837511	1.753590
O	-1.422809	1.051560	-0.103100
C	-2.120729	1.788390	-0.923750
C	-3.022209	2.795930	-0.314780
C	-3.252309	2.803080	1.068030
C	-3.604009	3.790541	-1.117000
C	-4.065189	3.783241	1.633880
H	-2.762249	2.059980	1.686070
C	-4.414139	4.769291	-0.548380
H	-3.404439	3.807231	-2.186610
C	-4.650179	4.763641	0.829760
H	-4.231599	3.789421	2.707360
H	-4.849959	5.542801	-1.174530
H	-5.277169	5.531011	1.275810
H	-1.623239	2.120130	-1.840500
C	-3.364329	0.612061	-2.044980
H	-2.643579	0.243800	-2.765880
H	-4.028749	1.389681	-2.399670
C	-3.862790	-0.320409	-1.125540
C	-4.992199	-0.051479	-0.222570
C	-5.118800	-0.789929	0.971830
C	-5.956049	0.931751	-0.519140
C	-6.172810	-0.540959	1.844480
H	-4.372290	-1.534789	1.221350
C	-7.015829	1.165941	0.351030
H	-5.891589	1.502281	-1.438680
C	-7.125959	0.432991	1.535050
H	-6.248890	-1.105819	2.768920
H	-7.756609	1.920911	0.105000
H	-7.952499	0.620541	2.214930
O	-3.216870	-1.445300	-0.899620
Si	-2.801440	-2.842360	-1.866600
C	-1.304710	-3.596970	-1.047050
H	-0.428270	-2.953870	-1.164880
H	-1.467690	-3.770690	0.021380
H	-1.081560	-4.564600	-1.514360
C	-2.525330	-2.374260	-3.660720
H	-1.639600	-1.744840	-3.800940
H	-2.369410	-3.298750	-4.233050
H	-3.393650	-1.869319	-4.100110
C	-4.321530	-3.942439	-1.693920
H	-4.174900	-4.883299	-2.239100
H	-4.516470	-4.193339	-0.645230
H	-5.221050	-3.463909	-2.098980

TS 81

B3LYP/6-31G(d) = -3573.932905
B3LYP-D3(BJ)/def2-TZVPP/IEFPCM(propanonitrile) = -3575.506692
B3LYP-D3(BJ)/def2-TZVPP/IEFPCM(propanonitrile)//B3LYP-D3(BJ)/6-31G(d) Free Energy (Quasiharmonic) = -3574.790127

Frequencies (Top 3 out of 300)

1. -270.7823 cm^{-1}
2. 9.4865 cm^{-1}
3. 10.9720 cm^{-1}

B3LYP/6-31G(d) Molecular Geometry in Cartesian Coordinates

B	0.327864	-0.303472	0.087606
O	-0.400106	0.457599	-0.964835
N	0.365187	-1.719303	-0.561402
C	-0.107488	-1.677257	-1.950125
S	1.279222	-2.979882	0.005873
C	-0.645519	-0.248469	-2.070655
O	-1.215086	0.210637	-3.040363
H	-0.671521	-3.695392	-2.423136
H	0.732859	-1.755594	-2.660013
C	-1.167775	-2.723412	-2.384048
C	-2.396926	-2.818467	-1.525367
C	-3.694175	-2.230259	-1.770403
C	-4.554356	-2.653175	-0.716036
N	-3.807213	-3.459121	0.117198
C	-2.516923	-3.548946	-0.366508
H	-1.769698	-4.146355	0.136122
C	-6.394345	-1.472120	-1.671457
C	-4.224277	-1.414266	-2.786595
H	-6.542080	-2.615010	0.160027
H	-4.121555	-3.844402	0.994162
C	-5.563907	-1.048707	-2.729832
H	-7.438247	-1.169958	-1.655462
H	-3.587378	-1.064781	-3.594018
C	-5.900896	-2.277331	-0.650755
H	-5.983803	-0.426857	-3.516435
C	2.900814	-2.845631	-0.761485
O	0.688943	-4.235606	-0.486670
O	1.448288	-2.754088	1.447098
C	5.375676	-2.457804	-2.022272
C	3.083097	-3.276040	-2.078337
C	3.956530	-2.269034	-0.050901
C	5.182157	-2.084488	-0.684393
C	4.315486	-3.073474	-2.699519
H	2.279616	-3.783406	-2.602155
H	3.815367	-1.971317	0.981597
H	6.001840	-1.631876	-0.131523
H	4.454168	-3.400211	-3.727267
C	6.686757	-2.171607	-2.713267
H	6.770984	-2.711721	-3.661373
H	7.541760	-2.448231	-2.085928
H	6.775539	-1.099018	-2.929353
H	-1.446701	-2.455937	-3.409791
C	1.749613	0.377980	0.438346
C	2.277762	0.392997	1.738240
C	2.533897	0.947852	-0.572926
C	3.534861	0.937370	2.010556
H	1.702606	-0.041603	2.547683
C	3.790497	1.501961	-0.306362
H	2.163264	0.966688	-1.594624
C	4.303022	1.497927	0.989003
H	5.274862	1.927082	1.200321
C	4.585232	2.076785	-1.443836

C	4.099942	0.833432	3.399512
F	3.850653	2.964876	-2.161741
F	4.982912	1.118743	-2.317520
F	5.697362	2.720812	-1.029837
F	3.148607	0.984888	4.348270
F	5.055735	1.761592	3.635166
F	4.670715	-0.378595	3.609951
O	-0.481660	-0.327603	1.362700
C	-1.777775	-0.231737	1.443499
C	-2.428598	-0.995806	2.543172
C	-1.648389	-1.696778	3.473186
C	-3.829229	-1.068507	2.616879
C	-2.265879	-2.447454	4.473779
H	-0.568517	-1.683013	3.376106
C	-4.440588	-1.816945	3.619368
H	-4.436678	-0.547559	1.880273
C	-3.658665	-2.504338	4.554486
H	-1.656105	-2.996411	5.185744
H	-5.524955	-1.866829	3.672509
H	-4.135755	-3.088152	5.337124
H	-2.340420	-0.180933	0.510109
C	-2.154039	1.688134	2.077939
H	-1.291434	1.773625	2.731171
H	-3.109542	1.516945	2.556881
C	-2.175214	2.472184	0.920820
C	-3.428874	2.641777	0.148221
C	-3.474864	2.334795	-1.223253
C	-4.595305	3.078576	0.801868
C	-4.672472	2.476800	-1.922969
H	-2.601180	1.934131	-1.727156
C	-5.779416	3.242458	0.087371
H	-4.560628	3.319648	1.860526
C	-5.819256	2.940861	-1.276266
H	-4.707891	2.208013	-2.973895
H	-6.670233	3.602092	0.594486
H	-6.746430	3.057130	-1.830502
O	-1.048503	2.973422	0.475159
Si	-0.580767	4.325675	-0.526947
C	1.197143	4.615554	-0.016822
H	1.310444	4.604762	1.072513
H	1.870032	3.861807	-0.436778
H	1.532014	5.595848	-0.379339
C	-1.683656	5.765417	-0.022436
H	-1.387201	6.666230	-0.574469
H	-2.739735	5.576610	-0.241111
H	-1.590160	5.988824	1.046650
C	-0.717857	3.928748	-2.355210
H	-1.725574	4.104697	-2.744073
H	-0.025472	4.574155	-2.910968
H	-0.452233	2.888203	-2.568530

TS 82

B3LYP/6-31G(d) = -3573.92498
B3LYP-D3(BJ)/def2-TZVPP/IEFPCM(propanonitrile) = -3575.506791
B3LYP-D3(BJ)/def2-TZVPP/IEFPCM(propanonitrile)//B3LYP-D3(BJ)/6-31G(d) Free Energy (Quasiharmonic) = -3574.790115

Frequencies (Top 3 out of 300)

1. -278.1675 cm^{-1}
2. 9.8346 cm^{-1}
3. 13.7747 cm^{-1}

B3LYP/6-31G(d) Molecular Geometry in Cartesian Coordinates

B	-0.581952	0.987771	1.006944
O	-0.947868	1.297253	2.404008
N	-1.185502	2.220533	0.259237
C	-2.009240	3.020990	1.173529
S	-0.775075	2.814570	-1.208931
C	-1.720428	2.378695	2.536507
O	-2.159966	2.778855	3.591632
H	-3.691541	3.510498	-0.082917
H	-1.658505	4.062105	1.211580
C	-3.536819	3.043566	0.893550
C	-4.220092	1.703911	0.946482
C	-4.614733	0.867580	-0.164101
C	-5.216520	-0.300132	0.382695
N	-5.204421	-0.163991	1.756159
C	-4.588424	1.028584	2.086342
H	-4.459041	1.315261	3.120963
C	-5.582761	-1.198779	-1.795140
C	-4.511249	0.982775	-1.562418
H	-6.147800	-2.227386	0.025467
H	-5.392711	-0.912660	2.404772
C	-4.993437	-0.048565	-2.360089
H	-5.943259	-1.990412	-2.446686
H	-4.031744	1.848851	-2.007856
C	-5.702983	-1.339561	-0.417001
H	-4.906766	0.026532	-3.440764
C	0.152506	4.332673	-0.895899
O	-1.966519	3.239507	-1.962190
O	0.138271	1.827056	-1.819666
C	1.629184	6.684050	-0.502358
C	1.204571	4.343606	0.023714
C	-0.172208	5.481891	-1.616111
C	0.568051	6.647466	-1.414529
C	1.930805	5.515482	0.214252
H	1.441345	3.449517	0.590109
H	-1.000427	5.457090	-2.315573
H	0.310927	7.544443	-1.972919
H	2.746103	5.523930	0.934151
C	2.439963	7.942027	-0.298069
H	3.425938	7.859240	-0.774158
H	2.611995	8.139133	0.766263
H	1.939620	8.815628	-0.727261
O	0.940323	1.042411	1.000369
C	1.754880	0.365687	0.210389
H	1.322591	-0.045178	-0.703393
H	-3.964671	3.714822	1.648050
C	3.092101	1.006627	0.018708
C	3.724058	1.687828	1.068100
C	3.710985	0.945897	-1.235827
C	4.963172	2.292285	0.864919
H	3.230227	1.751918	2.033355
C	4.949035	1.558227	-1.438275
H	3.205359	0.449503	-2.059517
C	5.579010	2.227979	-0.388382
H	5.447205	2.819568	1.682712
H	5.412653	1.524665	-2.420499
H	6.541130	2.707522	-0.547415
C	-1.196930	-0.451116	0.565821
C	-1.328660	-0.864801	-0.771376
C	-1.702436	-1.313885	1.553611
C	-1.929679	-2.083338	-1.100451
H	-0.988000	-0.212566	-1.569522
C	-2.319969	-2.524668	1.225738
H	-1.636084	-1.015705	2.594672
C	-2.435124	-2.920463	-0.106324
H	-2.925190	-3.851861	-0.365934
C	-2.948188	-3.368280	2.297251
C	-1.996019	-2.526280	-2.533560
F	-2.902537	-4.687731	1.999727
F	-2.353693	-3.204385	3.498134
F	-0.895499	-3.283055	-2.862193
F	-4.268003	-3.069069	2.476445
F	-3.066302	-3.309133	-2.784599
F	-2.019417	-1.500994	-3.400385
C	2.029487	-1.280875	1.111583
H	2.219517	-0.921638	2.117501

H	1.029148	-1.674129	0.952168
C	3.075718	-1.977779	0.476634
O	2.837519	-2.519456	-0.702594
Si	2.636284	-4.118539	-1.356256
C	4.176560	-5.131900	-0.982335
H	5.074143	-4.725506	-1.459949
H	4.033611	-6.151890	-1.361822
H	4.372623	-5.208690	0.092931
C	1.130464	-4.865444	-0.529593
H	1.213421	-4.855689	0.563446
H	1.015315	-5.911892	-0.839234
H	0.217619	-4.333704	-0.812218
C	2.417613	-3.796531	-3.184288
H	3.258969	-3.229321	-3.597965
H	2.355259	-4.745076	-3.731684
H	1.494583	-3.241985	-3.377503
C	4.461743	-1.966484	0.955939
C	4.750965	-1.848843	2.330369
C	5.527558	-2.072352	0.040562
C	6.069208	-1.850267	2.774001
H	3.942985	-1.792484	3.052091
C	6.843695	-2.054145	0.487937
H	5.310579	-2.128683	-1.019779
C	7.117134	-1.948681	1.854709
H	6.280287	-1.776276	3.836559
H	7.658476	-2.117049	-0.227274
H	8.146119	-1.942323	2.203115

TS 83

B3LYP/6-31G(d) = -3573.932695
B3LYP-D3(BJ)/def2-TZVPP/IEFPCM(propanonitrile) = -3575.507019
B3LYP-D3(BJ)/def2-TZVPP/IEFPCM(propanonitrile)//B3LYP-D3(BJ)/6-31G(d) Free Energy (Quasiharmonic) = -3574.790105

Frequencies (Top 3 out of 300)

1. -274.9375 cm^{-1}
2. 10.5434 cm^{-1}
3. 13.4733 cm^{-1}

B3LYP/6-31G(d) Molecular Geometry in Cartesian Coordinates

B	0.317023	0.711506	-0.238590
O	0.169254	0.191867	-1.628155
N	0.593769	2.207302	-0.509065
C	0.872224	2.435108	-1.932115
S	0.418517	3.478890	0.513458
C	0.494969	1.094742	-2.569487
O	0.479612	0.864089	-3.756195
H	2.422350	3.921647	-1.996695
H	0.187649	3.186606	-2.351784
C	2.322402	2.874423	-2.293719
C	3.415422	2.066862	-1.657708
C	4.041056	0.860918	-2.149004
C	4.991666	0.454315	-1.169435
N	4.953384	1.384506	-0.149126
C	3.990944	2.331285	-0.438864
H	3.775487	3.135850	0.250117
C	5.597887	-1.448917	-2.473492
C	3.891711	0.080694	-3.310245
H	6.492355	-0.990744	-0.557486
H	5.366970	1.263594	0.763726
C	4.667377	-1.062848	-3.459838
H	6.186803	-2.350978	-2.616751
H	3.162455	0.355778	-4.067279
C	5.774723	-0.694673	-1.318565
H	4.553564	-1.674738	-4.350986
C	-1.029861	4.379022	-0.084359
O	1.545021	4.417117	0.372508
O	0.103428	2.925421	1.840313
C	-3.290122	5.817854	-0.916648
C	-0.929192	5.749246	-0.321729
C	-2.248720	3.716858	-0.255796
C	-3.365215	4.438101	-0.669184
C	-2.058596	6.457787	-0.733988
H	0.024850	6.245276	-0.181397
H	-2.311797	2.649330	-0.071023
H	-4.314829	3.924074	-0.802712
H	-1.978682	7.526738	-0.916679
C	-4.501740	6.583122	-1.393693
H	-5.420160	6.214244	-0.923940
H	-4.630152	6.482739	-2.479708
H	-4.413143	7.651555	-1.173107
H	2.389060	2.837829	-3.388019
C	1.435420	-0.148197	0.560672
C	1.891604	-1.364336	0.032108
C	2.015649	0.282132	1.766015
C	2.858664	-2.138591	0.680360
H	1.513501	-1.699823	-0.926141
C	3.018273	-0.464108	2.390598
H	1.694188	1.220027	2.208688
C	3.436693	-1.688370	1.863445
H	4.207327	-2.267892	2.358672
C	3.746348	0.105630	3.573726
C	3.239298	-3.477394	0.114771
F	4.233128	-0.855216	4.391635
F	2.976761	0.922225	4.317170
F	4.825452	0.845712	3.173901
F	3.193438	-3.495508	-1.238890
F	2.390261	-4.455577	0.532928
F	4.480674	-3.859770	0.483360
O	-1.044659	0.585843	0.458140
C	-1.584847	-0.568052	0.740951
C	-2.525099	-0.595851	1.886789
C	-2.868552	0.595077	2.546900
C	-3.004628	-1.822054	2.377006
C	-3.699960	0.555763	3.664614
H	-2.439261	1.532034	2.209073
C	-3.831341	-1.855437	3.497949
H	-2.714776	-2.750075	1.892273
C	-4.187717	-0.665140	4.138986
H	-3.951097	1.478924	4.179151
H	-4.187065	-2.808742	3.878791
H	-4.827243	-0.691774	5.017129
H	-0.952683	-1.452686	0.625115
C	-2.696320	-0.954878	-0.936482
H	-1.858449	-0.723446	-1.581794
H	-3.432399	-0.164089	-0.840225
C	-3.152942	-2.274636	-0.883515
C	-2.291417	-3.437740	-1.134747
C	-2.768398	-4.735368	-0.856608
C	-0.975784	-3.285726	-1.623094
C	-1.949536	-5.842810	-1.046187
H	-3.778206	-4.859050	-0.483234
C	-0.158595	-4.398114	-1.800262
H	-0.586636	-2.303912	-1.870896
C	-0.641755	-5.676780	-1.510878
H	-2.327156	-6.836774	-0.824119
H	0.860526	-4.264949	-2.147532
H	0.001376	-6.541632	-1.646285
O	-4.371333	-2.571170	-0.448100
Si	-5.919491	-1.806027	-0.613306
C	-7.075542	-3.142209	0.012186
H	-6.862169	-3.391229	1.057779
H	-8.119350	-2.810621	-0.043736
H	-6.984792	-4.059998	-0.579190
C	-6.051547	-0.255449	0.437061
H	-5.753849	-0.441718	1.473993
H	-5.446718	0.575016	0.058574
H	-7.096908	0.080121	0.444957

C	-6.150573	-1.446157	-2.442942
H	-5.399157	-0.745189	-2.823017
H	-7.136874	-1.000018	-2.620358
H	-6.087500	-2.362114	-3.041036

TS 84

B3LYP/6-31G(d) = -3573.931636
B3LYP-D3(BJ)/def2-TZVPP/IEFPCM(propanonitrile) = -3575.505945
B3LYP-D3(BJ)/def2-TZVPP/IEFPCM(propanonitrile)//B3LYP-D3(BJ)/6-31G(d) Free Energy (Quasiharmonic) = -3574.790036

Frequencies (Top 3 out of 300)

1. -278.0976 cm^{-1}
2. 7.0274 cm^{-1}
3. 10.2338 cm^{-1}

B3LYP/6-31G(d) Molecular Geometry in Cartesian Coordinates

B	0.464814	0.599625	-0.238991
O	0.200830	0.130797	-1.626253
N	1.117926	1.976736	-0.507709
C	1.289294	2.206329	-1.947565
S	1.218570	3.252430	0.535432
C	0.596688	0.994937	-2.570133
O	0.429042	0.809280	-3.756944
H	3.181246	3.228200	-2.026917
H	0.741250	3.101318	-2.278253
C	2.749133	2.326015	-2.466804
C	3.627436	1.138207	-2.188062
C	4.582571	0.984855	-1.114241
C	5.172400	-0.300220	-1.257460
N	4.613107	-0.886607	-2.375741
C	3.678445	-0.025334	-2.919815
H	3.124295	-0.300935	-3.807081
C	6.536560	0.052017	0.663575
C	4.999509	1.807590	-0.051355
H	6.568217	-1.777121	-0.492832
H	4.735013	-1.852484	-2.636542
C	5.970852	1.334538	0.821288
H	7.288843	-0.291169	1.369140
H	4.549516	2.784098	0.097290
C	6.144880	-0.783458	-0.375016
H	6.294974	1.959218	1.649381
C	-0.174676	4.336507	0.162038
O	2.417081	4.039426	0.207494
O	1.012286	2.716916	1.885898
C	-2.351980	6.001692	-0.437665
C	-1.411191	4.106460	0.768443
C	-0.010427	5.397864	-0.730457
C	-1.099966	6.217172	-1.027448
C	-2.487596	4.935768	0.464551
H	-1.518936	3.293817	1.476499
H	0.965146	5.590636	-1.163590
H	-0.970560	7.043238	-1.722813
H	-3.448908	4.753520	0.939594
C	-3.517877	6.914750	-0.735554
H	-3.411530	7.395859	-1.713227
H	-3.595511	7.713114	0.014500
H	-4.467572	6.368927	-0.725963
H	2.667407	2.494043	-3.547858
C	1.292395	-0.503329	0.607339
C	1.411229	-0.440844	2.006607
C	1.873574	-1.605536	-0.032997
C	2.069133	-1.441911	2.724650
H	1.004714	0.414753	2.535321
C	2.527234	-2.614740	0.682381
H	1.815107	-1.682441	-1.113182
C	2.627534	-2.541300	2.069285
H	3.129260	-3.320460	2.630274
C	3.117476	-3.775352	-0.065675
C	2.101326	-1.379217	4.227229
F	3.415182	-4.815630	0.740923
F	4.267065	-3.450561	-0.718830
F	2.268578	-4.237935	-1.020263
F	2.206212	-0.116009	4.684946
F	3.128808	-2.089252	4.746753
F	0.963775	-1.894511	4.767709
O	-0.890664	0.760479	0.471714
C	-2.031246	0.367692	-0.028125
C	-3.240929	0.779839	0.725752
C	-3.166087	1.078101	2.095210
C	-4.467164	0.906319	0.058079
C	-4.303121	1.501040	2.780985
H	-2.210908	0.992487	2.603973
C	-5.601820	1.333586	0.746084
H	-4.527281	0.682432	-1.004411
C	-5.521998	1.630654	2.108525
H	-4.237167	1.736435	3.839567
H	-6.545717	1.438999	0.218733
H	-6.405960	1.965287	2.644513
H	-2.122570	0.363848	-1.116857
C	-1.936256	-1.645649	0.085728
H	-1.768714	-1.739801	1.151554
H	-1.038758	-1.715133	-0.516372
C	-3.104535	-2.188682	-0.458935
C	-4.302796	-2.521817	0.327303
C	-4.302126	-2.471828	1.735788
C	-5.483314	-2.917421	-0.333259
C	-5.446198	-2.800862	2.454865
H	-3.409878	-2.179740	2.276332
C	-6.624071	-3.246786	0.390074
H	-5.492136	-2.959350	-1.415798
C	-6.609778	-3.188781	1.786182
H	-5.428385	-2.758231	3.539742
H	-7.526104	-3.549491	-0.133898
H	-7.500561	-3.448716	2.351291
O	-3.251870	-2.315242	-1.765487
Si	-2.335186	-2.762315	-3.181633
C	-2.415056	-1.290594	-4.335103
H	-1.662290	-0.534511	-4.083884
H	-2.211109	-1.619259	-5.362319
H	-3.407833	-0.825956	-4.332602
C	-0.578548	-3.242613	-2.742844
H	-0.127798	-3.738274	-3.612575
H	0.025584	-2.358557	-2.520502
H	-0.506207	-3.938290	-1.900118
C	-3.315667	-4.227834	-3.834221
H	-3.333926	-5.054041	-3.114255
H	-2.867375	-4.605963	-4.761263
H	-4.352431	-3.950918	-4.057436

TS 85

B3LYP/6-31G(d) = -3573.934168
B3LYP-D3(BJ)/def2-TZVPP/IEFPCM(propanonitrile) = -3575.507374
B3LYP-D3(BJ)/def2-TZVPP/IEFPCM(propanonitrile)//B3LYP-D3(BJ)/6-31G(d) Free Energy (Quasiharmonic) = -3574.789995

Frequencies (Top 3 out of 300)

1. -276.3282 cm^{-1}
2. 8.3265 cm^{-1}
3. 10.6833 cm^{-1}

B3LYP/6-31G(d) Molecular Geometry in Cartesian Coordinates

B	-0.092172	0.503261	-0.471156
O	-0.194152	-0.284070	-1.737705

N	0.978783	1.560139	-0.878321
C	1.309931	1.448684	-2.308766
S	1.146527	3.031327	-0.119531
C	0.448747	0.272423	-2.764604
O	0.338906	-0.106779	-3.914291
H	3.334182	2.171547	-2.445408
H	0.971271	2.339507	-2.852544
C	2.806076	1.243986	-2.690290
C	3.522752	0.078046	-2.068957
C	3.529230	-1.306672	-2.495516
C	4.355709	-2.020641	-1.583064
N	4.853148	-1.107349	-0.675789
C	4.336483	0.140980	-0.963357
H	4.608794	0.994938	-0.360207
C	3.975949	-4.066604	-2.744741
C	2.942180	-2.012693	-3.562922
H	5.201285	-3.921797	-0.966686
H	5.317752	-1.346110	0.186748
C	3.171598	-3.378227	-3.676129
H	4.129301	-5.136802	-2.856622
H	2.303305	-1.500759	-4.274866
C	4.579308	-3.397443	-1.687258
H	2.719749	-3.929232	-4.496909
C	2.817779	3.098447	0.548405
O	1.061784	4.095984	-1.134224
O	0.245296	3.024451	1.039081
C	5.408534	3.214176	1.614614
C	3.120162	2.384575	1.710360
C	3.786653	3.882650	-0.076695
C	5.074674	3.932812	0.458818
C	4.411487	2.439159	2.228394
H	2.353343	1.794421	2.199736
H	3.525520	4.452505	-0.962082
H	5.830514	4.544324	-0.028287
H	4.648605	1.868401	3.122827
C	6.795599	3.297139	2.206732
H	7.087813	2.352905	2.678444
H	7.543101	3.544424	1.446082
H	6.846723	4.074783	2.980371
H	2.818819	1.159751	-3.784333
C	0.230813	-0.425581	0.807858
C	-0.796784	-0.947577	1.603436
C	1.539514	-0.809594	1.132801
C	-0.540707	-1.835156	2.651387
C	1.808024	-1.664388	2.207805
H	2.362234	-0.426696	0.538125
C	0.767210	-2.197343	2.969585
H	0.970834	-2.871708	3.792389
C	3.231350	-2.037880	2.511907
C	-1.710007	-2.434669	3.375386
F	4.061173	-0.958642	2.451129
F	3.731242	-2.937754	1.627448
F	3.375568	-2.576906	3.741054
F	-2.596239	-1.496981	3.786407
F	-1.350482	-3.154451	4.456785
F	-2.415804	-3.275437	2.559315
O	-1.471688	1.096139	-0.214074
C	-2.174767	1.728695	-1.111837
C	-3.096964	2.778953	-0.618657
C	-3.713589	3.655133	-1.527206
C	-3.308331	2.950692	0.756693
C	-4.540619	4.676394	-1.069622
H	-3.529742	3.544650	-2.594075
C	-4.137711	3.974579	1.211330
H	-2.788983	2.303056	1.452967
C	-4.758036	4.834410	0.303161
H	-5.004586	5.356657	-1.778329
H	-4.288936	4.108951	2.278594
H	-5.398197	5.635914	0.661905
H	-1.681954	1.961582	-2.060295
C	-3.409848	0.419170	-2.119179
H	-4.137727	1.137954	-2.471869
H	-2.705561	0.061546	-2.861356
C	-3.803295	-0.502165	-1.139472
C	-4.886308	-0.261391	-0.173482
C	-5.867703	0.725800	-0.390141
C	-4.946101	-1.031714	1.006421
C	-6.879185	0.931534	0.542739
H	-5.852598	1.327324	-1.291745
C	-5.950798	-0.810150	1.942046
H	-4.190108	-1.785264	1.191833
C	-6.922474	0.167274	1.711213
H	-7.632837	1.691383	0.358606
H	-5.971141	-1.398204	2.854741
H	-7.710598	0.334070	2.440369
O	-3.091014	-1.585703	-0.918209
Si	-2.572417	-2.966553	-1.853248
C	-4.036574	-4.144225	-1.710072
H	-4.945901	-3.721097	-2.153242
H	-3.822470	-5.084633	-2.233261
H	-4.253122	-4.387426	-0.663863
C	-1.071560	-3.616871	-0.954598
H	-0.780206	-4.589741	-1.369771
H	-0.224516	-2.933694	-1.061253
H	-1.277051	-3.751369	0.112487
H	-1.821253	-0.663978	1.394254
C	-2.258213	-2.524928	-3.647885
H	-2.009821	-3.449905	-4.185867
H	-3.144511	-2.101310	-4.134798
H	-1.417890	-1.833618	-3.774037

TS 86

B3LYP/6-31G(d) = -3573.930517
B3LYP-D3(BJ)/def2-TZVPP/IEFPCM(propanonitrile) = -3575.506048
B3LYP-D3(BJ)/def2-TZVPP/IEFPCM(propanonitrile)//B3LYP-D3(BJ)/6-31G(d) Free Energy (Quasiharmonic) = -3574.789959

Frequencies (Top 3 out of 300)

1. -265.3095 cm^{-1}
2. 7.4978 cm^{-1}
3. 10.3703 cm^{-1}

B3LYP/6-31G(d) Molecular Geometry in Cartesian Coordinates

B	-0.552781	0.369490	-0.021660
O	-1.346041	-0.265060	-1.107530
N	0.247070	1.452060	-0.828730
C	-0.060780	1.346650	-2.263430
S	0.703990	2.928080	-0.215780
C	-1.156411	0.283870	-2.305830
O	-1.772071	-0.039159	-3.305340
H	1.883990	1.705530	-3.141520
H	-0.482210	2.288300	-2.636000
C	1.104580	0.940970	-3.214820
C	1.702159	-0.424150	-2.989430
C	3.027279	-0.730741	-2.502500
C	3.136969	-2.147191	-2.434940
N	1.948459	-2.670850	-2.900060
C	1.089689	-1.635550	-3.216660
H	0.098939	-1.842860	-3.595900
C	5.365549	-1.995211	-1.605400
C	4.139119	0.046239	-2.133820
H	4.338998	-3.876161	-1.906540
H	1.672479	-3.636150	-2.807140
C	5.292269	-0.589641	-1.692160
H	6.277739	-2.462721	-1.244270
H	4.099140	1.129489	-2.188710
C	4.290509	-2.793001	-1.977460
H	6.153529	0.005739	-1.400300

C	2.468520	3.139210	-0.503780
O	0.537940	2.855290	1.243110
O	0.032080	3.990590	-0.985600
C	5.209580	3.608549	-0.858750
C	3.386530	2.571229	0.383090
C	2.901000	3.946919	-1.556480
C	4.266940	4.170709	-1.730110
C	4.745710	2.812219	0.199000
H	3.049130	1.963459	1.215150
H	2.171740	4.415950	-2.208380
H	4.603230	4.806149	-2.545980
H	5.458510	2.375409	0.894030
C	6.688480	3.840249	-1.060320
H	7.219160	3.893889	-0.103810
H	7.140710	3.022499	-1.637100
H	6.878220	4.769019	-1.608000
H	0.691630	1.012960	-4.229060
C	0.352889	-0.675200	0.797980
C	0.509499	-1.998280	0.358960
C	1.069059	-0.287900	1.937220
C	1.358559	-2.889390	1.015990
C	1.936669	-1.172380	2.588120
H	0.967850	0.728070	2.307780
C	2.090209	-2.479160	2.133550
H	2.769019	-3.160930	2.633250
C	2.720169	-0.666170	3.765410
C	1.480458	-4.315900	0.565680
F	3.499899	-1.618471	4.322810
F	3.537049	0.361279	3.415420
F	1.910129	-0.190550	4.741250
F	2.769578	-4.710720	0.453500
F	0.895578	-5.176540	1.441750
F	0.890958	-4.543000	-0.637290
O	-1.549210	0.989690	0.966870
C	-2.615490	1.626171	0.588290
C	-3.074440	2.752971	1.429140
C	-2.523160	2.963081	2.702230
C	-4.039770	3.645151	0.936450
C	-2.948180	4.041761	3.473490
H	-1.742420	2.299161	3.054380
C	-4.458810	4.724311	1.708920
H	-4.448530	3.501411	-0.061300
C	-3.917950	4.920091	2.982860
H	-2.510810	4.207381	4.453910
H	-5.198299	5.416801	1.316940
H	-4.242449	5.763671	3.585940
H	-2.793180	1.721531	-0.484360
C	-4.322351	0.384501	0.836170
H	-4.353771	0.477811	1.912970
H	-5.037520	1.006621	0.310540
C	-4.038781	-0.874879	0.301660
C	-3.320511	-1.917849	1.046000
C	-3.095871	-3.173199	0.445810
C	-2.842701	-1.698129	2.353060
C	-2.421332	-4.174759	1.133210
H	-3.446781	-3.342549	-0.564910
C	-2.168961	-2.705039	3.037090
H	-2.975841	-0.736919	2.834550
C	-1.955322	-3.944279	2.431190
H	-2.241512	-5.132839	0.654630
H	-1.796161	-2.515949	4.039190
H	-1.411882	-4.722430	2.957920
O	-4.344481	-1.203179	-0.939150
Si	-5.358471	-0.624959	-2.214520
C	-4.908641	-1.772189	-3.619520
H	-3.845101	-1.646649	-3.850340
H	-5.091601	-2.821819	-3.363800
H	-5.485441	-1.539339	-4.522660
C	-4.986830	1.161811	-2.660990
H	-5.596230	1.441901	-3.530230
H	-5.217430	1.875881	-1.863220
H	-3.936780	1.269341	-2.951140
H	-0.036341	-2.331830	-0.514900
C	-7.128551	-0.836988	-1.607540
H	-7.338551	-1.881528	-1.351330
H	-7.336971	-0.226208	-0.721550
H	-7.838031	-0.537138	-2.388700

TS 87

B3LYP/6-31G(d) = -3573.929048
B3LYP-D3(BJ)/def2-TZVPP/IEFPCM(propanonitrile) = -3575.507288
B3LYP-D3(BJ)/def2-TZVPP/IEFPCM(propanonitrile)//B3LYP-D3(BJ)/6-31G(d) Free Energy (Quasiharmonic) = -3574.789946

Frequencies (Top 3 out of 300)

1. -274.0839 cm^{-1}
2. 10.0208 cm^{-1}
3. 13.6639 cm^{-1}

B3LYP/6-31G(d) Molecular Geometry in Cartesian Coordinates

B	-0.495881	0.275967	-0.240170
O	-0.330554	0.069148	-1.705235
N	-1.212221	1.659958	-0.232002
C	-1.310239	2.218682	-1.585647
S	-2.034154	2.367693	1.011747
C	-0.784129	1.078054	-2.454443
O	-0.760727	1.071625	-3.668945
H	-0.917874	4.304327	-1.211745
H	-2.361020	2.387794	-1.866446
C	-0.536134	3.534616	-1.887009
C	0.963405	3.460138	-1.800489
C	1.812620	3.833786	-0.691502
C	3.156841	3.617685	-1.101525
N	3.119688	3.151591	-2.400186
C	1.804186	3.048059	-2.808453
H	1.559125	2.701734	-3.803225
C	3.979184	4.364115	1.006069
C	1.571931	4.334123	0.601772
H	5.267545	3.684678	-0.595134
H	3.915954	2.809880	-2.914475
C	2.654470	4.594853	1.432129
H	4.803222	4.572362	1.683632
H	0.555627	4.492047	0.948694
C	4.249002	3.871466	-0.264738
H	2.478651	4.975857	2.434603
C	-3.786539	2.231898	0.606520
O	-1.757674	3.813011	1.025506
O	-1.790658	1.548633	2.205259
C	-6.509436	2.000584	-0.031638
C	-4.414499	3.239664	-0.129898
C	-4.508550	1.119819	1.041486
C	-5.859535	1.010556	0.719281
C	-5.766170	3.112959	-0.448787
H	-3.856140	4.122181	-0.423260
H	-4.016630	0.361213	1.638107
H	-6.417806	0.142001	1.060631
H	-6.253262	3.897860	-1.022711
C	-7.982210	1.890225	-0.348657
H	-8.231578	2.403944	-1.282965
H	-8.591349	2.343765	0.444585
H	-8.295697	0.844891	-0.440260
H	-0.831756	3.816558	-2.905046
C	0.908836	0.155262	0.545371
C	2.111634	0.107342	-0.168254
C	0.990405	0.070950	1.946154
C	3.345088	-0.035114	0.476707
H	2.089244	0.178389	-1.249865
C	2.220038	-0.057417	2.594786

H	0.082596	0.133030	2.535624
C	3.409180	-0.116728	1.864653
H	4.362426	-0.215544	2.370261
C	2.271839	-0.214954	4.088949
C	4.584792	-0.188847	-0.352089
F	3.416556	0.282097	4.613219
F	2.225313	-1.526969	4.456277
F	1.242187	0.393010	4.709266
F	5.719122	-0.106561	0.371225
F	4.672078	0.727967	-1.345886
F	4.615318	-1.408037	-0.980306
O	-1.406948	-0.843609	0.293201
C	-2.161525	-1.583483	-0.469426
C	-3.334231	-2.219471	0.183909
C	-3.404353	-2.325200	1.581972
C	-4.392541	-2.704192	-0.598908
C	-4.516004	-2.915492	2.182008
H	-2.596875	-1.917990	2.181670
C	-5.503192	-3.290307	0.004420
H	-4.347358	-2.613600	-1.681975
C	-5.565388	-3.401165	1.396602
H	-4.567882	-2.988610	3.264757
H	-6.321788	-3.656287	-0.609012
H	-6.430917	-3.859151	1.867353
H	-2.311570	-1.241200	-1.493757
C	-1.232213	-3.310658	-1.165417
H	-1.754957	-4.031599	-0.552665
H	-1.577050	-3.268904	-2.192054
C	0.158435	-3.224189	-0.967939
C	0.796662	-3.552664	0.312689
C	0.037044	-3.947803	1.433438
C	2.198566	-3.480232	0.435777
C	0.662123	-4.257823	2.635277
H	-1.044289	-3.998450	1.377271
C	2.817530	-3.799438	1.638699
H	2.791481	-3.144326	-0.405038
C	2.053471	-4.185462	2.742242
H	0.064221	-4.548588	3.493859
H	3.898007	-3.729756	1.720410
H	2.538209	-4.417508	3.685753
O	0.970937	-2.768120	-1.891359
Si	1.109909	-2.697766	-3.624151
C	-0.503892	-2.346577	-4.514901
H	-0.897716	-1.362650	-4.239620
H	-0.286013	-2.314200	-5.591297
H	-1.272836	-3.113048	-4.369965
C	2.349610	-1.326449	-3.903107
H	2.742145	-1.369615	-4.926672
H	1.864443	-0.353941	-3.768990
H	3.195039	-1.394430	-3.210833
C	1.783946	-4.398512	-4.069301
H	2.730444	-4.600012	-3.555551
H	1.968144	-4.466760	-5.148560
H	1.080184	-5.196383	-3.804042

TS 88

B3LYP/6-31G(d) = -3573.924999
B3LYP-D3(BJ)/def2-TZVPP/IEFPCM(propanonitrile) = -3575.506789
B3LYP-D3(BJ)/def2-TZVPP/IEFPCM(propanonitrile)//B3LYP-D3(BJ)/6-31G(d) Free Energy (Quasiharmonic) = -3574.789882

Frequencies (Top 3 out of 300)

1. -278.1864 cm^{-1}
2. 9.8150 cm^{-1}
3. 13.9663 cm^{-1}

B3LYP/6-31G(d) Molecular Geometry in Cartesian Coordinates

B	-0.582363	0.987597	1.006522
O	-0.948518	1.297798	2.403388
N	-1.186270	2.219760	0.258050
C	-2.010410	3.020418	1.171820
S	-0.776043	2.813000	-1.210462
C	-1.721433	2.379049	2.535191
O	-2.161136	2.779750	3.590052
H	-3.692799	3.508597	-0.084997
H	-1.660086	4.061682	1.209278
C	-3.537974	3.042220	0.891716
C	-4.220686	1.702306	0.945280
C	-4.614889	0.865269	-0.164924
C	-5.216196	-0.302441	0.382396
N	-5.204253	-0.165637	1.755800
C	-4.588778	1.027360	2.085442
H	-4.459572	1.314582	3.119936
C	-5.581895	-1.202292	-1.795034
C	-4.511353	0.979842	-1.563288
H	-6.146636	-2.230263	0.026023
H	-5.392066	-0.914141	2.404744
C	-4.993028	-0.052096	-2.360495
H	-5.941995	-1.994398	-2.446223
H	-4.032199	1.845919	-2.009110
C	-5.702157	-1.342462	-0.416835
H	-4.906306	0.022512	-3.441199
C	0.151321	4.331329	-0.897738
O	0.136699	1.824863	-1.821102
O	-1.967535	3.238034	-1.963635
C	1.632000	6.681112	-0.510100
C	1.201000	4.343635	0.023773
C	-0.166717	5.477632	-1.626584
C	0.576519	6.641397	-1.429611
C	1.930879	5.514625	0.209823
H	1.437947	3.449947	0.590746
H	-0.988682	5.449538	-2.333275
H	0.328468	7.534112	-1.998996
H	2.748849	5.522452	0.926574
C	2.411091	7.953633	-0.275251
H	2.350561	8.625848	-1.137176
H	3.468729	7.746019	-0.079115
H	2.021911	8.500271	0.594077
O	0.939857	1.042831	1.000104
C	1.754834	0.366614	0.210103
H	1.322787	-0.044502	-0.703675
H	-3.966158	3.713677	1.645851
C	3.091634	1.008409	0.018453
C	3.723191	1.689905	1.067897
C	3.710469	0.948271	-1.236135
C	4.961877	2.295234	0.864706
H	3.229392	1.753527	2.033200
C	4.948059	1.561519	-1.438598
H	3.205107	0.451676	-2.059864
C	5.577648	2.231558	-0.388661
H	5.445624	2.822694	1.682558
H	5.411590	1.528499	-2.420877
H	6.539388	2.711842	-0.547737
C	-1.196742	-0.451810	0.566274
C	-1.328406	-0.866380	-0.770658
C	-1.701727	-1.314223	1.554641
C	-1.928851	-2.085418	-1.098923
H	-0.988157	-0.214458	-1.569230
C	-2.318697	-2.525511	1.227571
H	-1.635424	-1.015378	2.595515
C	-2.433777	-2.922191	-0.104234
H	-2.923406	-3.853991	-0.363223
C	-2.946453	-3.368716	2.299673
C	-1.995173	-2.529225	-2.531767
F	-2.900500	-4.688316	2.002878
F	-2.351771	-3.203981	3.500348
F	-0.894319	-3.285622	-2.860138
F	-4.266321	-3.069764	2.478967

F	-2.019230	-1.504454	-3.399183
F	-3.065094	-3.312774	-2.782192
C	2.030406	-1.279841	1.111328
H	1.030328	-1.673724	0.951842
H	2.220139	-0.920449	2.117245
C	3.077129	-1.976026	0.476425
C	4.463150	-1.963687	0.955713
C	4.752317	-1.845623	2.330119
C	5.529021	-2.068954	0.040332
C	6.070568	-1.846038	2.773724
H	3.944314	-1.789719	3.051850
C	6.845154	-2.049744	0.487678
H	5.312057	-2.125622	-1.019994
C	7.118545	-1.943858	1.854426
H	6.281617	-1.771723	3.836265
H	7.659964	-2.112185	-0.227541
H	8.147532	-1.936709	2.202810
O	2.839336	-2.517959	-0.702778
Si	2.638890	-4.117291	-1.356051
C	2.418970	-3.795795	-3.184021
H	3.259577	-3.227840	-3.598198
H	1.495315	-3.242169	-3.376875
H	2.357269	-4.744490	-3.731231
C	4.180155	-5.129415	-0.982845
H	4.376877	-5.205922	0.092320
H	4.037815	-6.149562	-1.362142
H	5.077148	-4.722356	-1.461007
C	1.134127	-4.865204	-0.528353
H	1.217754	-4.855209	0.564634
H	0.220694	-4.334226	-0.810513
H	1.019597	-5.911792	-0.837752

TS 89

B3LYP/6-31G(d) = -3573.930258
B3LYP-D3(BJ)/def2-TZVPP/IEFPCM(propanonitrile) = -3575.507065
B3LYP-D3(BJ)/def2-TZVPP/IEFPCM(propanonitrile)//B3LYP-D3(BJ)/6-31G(d) Free Energy (Quasiharmonic) = -3574.789877

Frequencies (Top 3 out of 300)

1. -286.8420 cm^{-1}
2. 8.3793 cm^{-1}
3. 14.0047 cm^{-1}

B3LYP/6-31G(d) Molecular Geometry in Cartesian Coordinates

B	0.322554	0.862267	-0.351222
O	0.282320	0.640871	-1.817453
N	0.733067	2.353443	-0.277138
C	1.107720	2.851526	-1.605393
S	0.556381	3.393028	0.983205
C	0.719557	1.689343	-2.526321
O	0.797405	1.708745	-3.733758
H	2.724698	4.239511	-1.304009
H	0.482562	3.706915	-1.900786
C	2.596181	3.271314	-1.794234
C	3.609044	2.302741	-1.256771
C	4.224184	1.182423	-1.930357
C	5.090853	0.553476	-0.991219
N	5.013616	1.273976	0.184612
C	4.107810	2.304391	0.022972
H	3.876550	2.976144	0.837579
C	5.722796	-1.091691	-2.598958
C	4.127109	0.643610	-3.226425
H	6.499125	-1.047573	-0.579664
H	5.364197	0.966235	1.079532
C	4.873437	-0.484354	-3.546307
H	6.290581	-1.975430	-2.877785
H	3.457582	1.088621	-3.957292
C	5.844241	-0.580497	-1.311180
H	4.798542	-0.911397	-4.542972
C	-0.896254	4.404318	0.641949
O	0.246107	2.582434	2.171206
O	1.688009	4.334359	1.001762
C	-3.163884	5.970403	0.112524
C	-2.138811	4.014417	1.143288
C	-0.770334	5.577082	-0.106597
C	-1.903775	6.344114	-0.373354
C	-3.259788	4.798188	0.876124
H	-2.213542	3.117498	1.747245
H	0.207492	5.897293	-0.450065
H	-1.804017	7.255248	-0.958652
H	-4.225824	4.500203	1.278297
C	-4.378181	6.831162	-0.145166
H	-5.301484	6.243113	-0.115021
H	-4.320649	7.324118	-1.121426
H	-4.470143	7.621041	0.612177
O	-1.122835	0.735569	0.134204
C	-1.654253	-0.354031	0.624030
H	-0.979651	-1.048176	1.133723
H	2.733409	3.427963	-2.871085
C	-2.994642	-0.195478	1.247190
C	-3.406030	-1.083215	2.251715
C	-3.844568	0.854027	0.866691
C	-4.647793	-0.925404	2.866417
H	-2.745044	-1.886905	2.564992
C	-5.087493	1.006417	1.479612
H	-3.508547	1.557693	0.112409
C	-5.492650	0.117154	2.479987
H	-4.950300	-1.609297	3.654385
H	-5.736880	1.826755	1.185531
H	-6.457075	0.244306	2.964110
C	1.329173	-0.218509	0.334733
C	1.803693	-1.302794	-0.422240
C	1.835556	-0.084412	1.639966
C	2.714144	-2.227935	0.100503
H	1.493106	-1.401093	-1.457951
C	2.777260	-0.983019	2.148743
H	1.510498	0.750894	2.252223
C	3.213227	-2.070681	1.390577
H	3.940046	-2.767749	1.791509
C	3.425335	-0.715529	3.477311
C	3.124845	-3.417953	-0.717883
F	4.543435	0.055014	3.331174
F	2.614731	-0.061318	4.329903
F	3.163532	-3.157172	-2.035752
F	3.828956	-1.853264	4.089361
F	2.232499	-4.450976	-0.562988
F	4.328207	-3.907056	-0.353777
C	-1.890759	-1.521332	-0.983646
H	-2.275364	-0.767669	-1.659754
H	-0.836926	-1.729883	-1.115446
C	-2.735482	-2.587424	-0.647449
C	-2.287672	-3.780338	0.084173
C	-0.921284	-4.109211	0.190902
C	-3.239166	-4.622757	0.696339
C	-0.520930	-5.242572	0.891572
H	-0.161681	-3.504743	-0.289005
C	-2.832423	-5.749081	1.402321
H	-4.290840	-4.372770	0.621749
C	-1.473166	-6.062104	1.501811
H	0.535728	-5.480148	0.948833
H	-3.574333	-6.385472	1.876103
H	-1.157924	-6.945725	2.049748
O	-4.042164	-2.507989	-0.824821
Si	-5.128427	-1.957699	-2.059940
C	-4.685872	-0.262932	-2.736265
H	-5.496907	0.070052	-3.397357
H	-4.583470	0.482880	-1.941749
H	-3.766163	-0.263038	-3.330739

C	-5.048464	-3.268900	-3.402183
H	-5.299530	-4.261139	-3.011565
H	-5.756301	-3.036483	-4.207389
H	-4.049449	-3.324943	-3.849932
C	-6.765658	-1.933978	-1.150473
H	-7.024510	-2.926654	-0.765811
H	-7.575743	-1.611328	-1.815479
H	-6.729442	-1.241613	-0.302094

TS 90

B3LYP/6-31G(d) = -3573.933225
B3LYP-D3(BJ)/def2-TZVPP/IEFPCM(propanonitrile) = -3575.506509
B3LYP-D3(BJ)/def2-TZVPP/IEFPCM(propanonitrile)//B3LYP-D3(BJ)/6-31G(d) Free Energy (Quasiharmonic) = -3574.789877

Frequencies (Top 3 out of 300)

1. -284.6378 cm^{-1}
2. 9.2102 cm^{-1}
3. 11.9579 cm^{-1}

B3LYP/6-31G(d) Molecular Geometry in Cartesian Coordinates

B	-0.476679	0.591579	0.238163
O	-0.105637	0.343696	1.657409
N	-1.107913	2.003195	0.344508
C	-1.224662	2.423604	1.747301
S	-1.275960	3.125061	-0.853489
C	-0.476332	1.321525	2.496613
O	-0.245943	1.298441	3.686445
H	-3.133041	3.417190	1.776168
H	-0.684808	3.365161	1.927392
C	-2.664058	2.586922	2.310021
C	-3.526851	1.358785	2.224944
C	-4.524926	1.054719	1.224904
C	-5.073442	-0.214790	1.552677
N	-4.451662	-0.645701	2.708075
C	-3.515323	0.294158	3.095664
H	-2.913235	0.140181	3.981141
C	-6.526301	-0.134593	-0.333777
C	-5.009377	1.729781	0.089230
H	-6.457281	-1.806589	1.039420
H	-4.526016	-1.579324	3.080717
C	-6.003644	1.130727	-0.673497
H	-7.297479	-0.579609	-0.957300
H	-4.592785	2.689654	-0.199426
C	-6.068194	-0.825611	0.781178
H	-6.379688	1.640420	-1.556591
C	0.111224	4.266612	-0.682950
O	-2.474872	3.933805	-0.585994
O	-1.111504	2.417890	-2.129299
C	2.278561	6.026677	-0.398827
C	-0.040292	5.438679	0.060775
C	1.328062	3.974084	-1.302230
C	2.398789	4.852372	-1.157367
C	1.043524	6.306362	0.199598
H	-1.004724	5.679180	0.494986
H	1.422003	3.079520	-1.905974
H	3.342021	4.625683	-1.649250
H	0.921629	7.222044	0.773387
C	3.455065	6.957778	-0.224297
H	3.129140	7.977781	0.003179
H	4.076108	6.992517	-1.125906
H	4.100336	6.629314	0.601605
H	-2.541509	2.896272	3.355528
C	-1.376577	-0.617534	-0.348439
C	-1.802902	-1.659972	0.486486
C	-1.732899	-0.695401	-1.705622
C	-2.555137	-2.733359	-0.002850
H	-1.549227	-1.632963	1.540893
C	-2.478121	-1.768525	-2.199122
H	-1.444718	0.107142	-2.376741
C	-2.895792	-2.796326	-1.352182
H	-3.476109	-3.625990	-1.738320
C	-2.768886	-1.859217	-3.672269
C	-3.030202	-3.800606	0.940378
F	-3.870059	-2.601962	-3.930512
F	-1.741748	-2.447345	-4.343194
F	-2.953181	-0.648595	-4.234097
F	-2.137193	-4.037863	1.930422
F	-3.259097	-4.975949	0.314693
F	-4.198754	-3.467774	1.561984
O	0.811996	0.611228	-0.600167
C	2.008184	0.388465	-0.127482
C	3.127122	0.636854	-1.073347
C	4.434558	0.797943	-0.590080
C	2.879660	0.761125	-2.449506
C	5.478880	1.083239	-1.468746
H	4.631021	0.714080	0.475783
C	3.925913	1.047262	-3.324825
H	1.863357	0.652842	-2.814216
C	5.226891	1.207860	-2.837761
H	6.486221	1.217646	-1.084585
H	3.725145	1.152107	-4.387349
H	6.039837	1.436517	-3.521590
H	2.190267	0.663078	0.914282
C	2.036055	-1.590403	0.266032
H	1.888881	-1.978259	-0.736368
H	1.137855	-1.550838	0.868480
C	3.233794	-1.913459	0.919444
C	3.500065	-1.584070	2.325152
C	4.761110	-1.872820	2.889199
C	2.521321	-0.962222	3.129641
C	5.029364	-1.549578	4.214067
H	5.519691	-2.346583	2.277121
C	2.800954	-0.632399	4.452262
H	1.540242	-0.721345	2.738346
C	4.052003	-0.926824	4.998576
H	6.002815	-1.779221	4.638034
H	2.032961	-0.138124	5.039114
H	4.268423	-0.671667	6.032404
O	4.267149	-2.420336	0.262801
Si	4.414314	-3.571213	-1.031089
C	3.941597	-2.818959	-2.682748
H	4.460156	-1.871006	-2.858871
H	4.229162	-3.511155	-3.484894
H	2.866465	-2.637047	-2.781529
C	3.333302	-5.038613	-0.576852
H	2.273131	-4.768642	-0.519443
H	3.430302	-5.826316	-1.334102
H	3.624583	-5.467960	0.388336
C	6.244064	-3.974993	-0.971644
H	6.851672	-3.080056	-1.147031
H	6.532001	-4.394249	-0.001217
H	6.504488	-4.709732	-1.742975

TS 91

B3LYP/6-31G(d) = -3573.930272
B3LYP-D3(BJ)/def2-TZVPP/IEFPCM(propanonitrile) = -3575.507073
B3LYP-D3(BJ)/def2-TZVPP/IEFPCM(propanonitrile)//B3LYP-D3(BJ)/6-31G(d) Free Energy (Quasiharmonic) = -3574.789833

Frequencies (Top 3 out of 300)

1. -286.9005 cm^{-1}
2. 8.3890 cm^{-1}
3. 14.0114 cm^{-1}

B3LYP/6-31G(d) Molecular Geometry in Cartesian Coordinates

B	0.322985	0.863138	-0.349782
O	0.282324	0.644488	-1.816419
N	0.734772	2.353819	-0.273014
C	1.109417	2.854159	-1.600399
S	0.558423	3.391355	0.989058
C	0.720139	1.693990	-2.523417
O	0.797625	1.715634	-3.730845
H	2.727376	4.240471	-1.296772
H	0.484825	3.710617	-1.893942
C	2.598124	3.273270	-1.788772
C	3.610430	2.303027	-1.253289
C	4.224688	1.183533	-1.929039
C	5.091104	0.552280	-0.991215
N	5.014549	1.270664	0.185953
C	4.109418	2.301989	0.026368
H	3.878742	2.972391	0.842253
C	5.721644	-1.090357	-2.602089
C	4.127002	0.647158	-3.226071
H	6.498372	-1.050474	-0.582862
H	5.365167	0.961105	1.080230
C	4.872513	-0.480717	-3.548163
H	6.288786	-1.973963	-2.882643
H	3.457634	1.093958	-3.955994
C	5.843671	-0.581611	-1.313397
H	4.797147	-0.905883	-4.545594
C	-0.893301	4.403661	0.646780
O	1.690933	4.331557	1.010047
O	0.246625	2.579007	2.175451
C	-3.158514	5.974591	0.120703
C	-2.136505	4.014792	1.147490
C	-0.764648	5.580520	-0.094704
C	-1.896452	6.351510	-0.357433
C	-3.255777	4.802116	0.883961
H	-2.211596	3.119889	1.754389
H	0.215043	5.904453	-0.429236
H	-1.793211	7.269814	-0.930724
H	-4.220796	4.508704	1.291832
C	-4.382108	6.805684	-0.185439
H	-4.834039	6.507822	-1.141216
H	-5.148993	6.690894	0.587764
H	-4.135058	7.869672	-0.262938
H	2.735260	3.431851	-2.865353
C	1.328998	-0.219500	0.334083
C	1.835626	-0.087936	1.639479
C	1.802805	-1.302718	-0.424850
C	2.776896	-0.987946	2.146571
C	2.712849	-2.229244	0.096152
H	1.491986	-1.399049	-1.460678
C	3.212179	-2.074509	1.386437
H	3.938685	-2.772659	1.786052
C	3.122840	-3.418047	-0.724354
C	3.425288	-0.723163	3.475530
F	4.326104	-3.908246	-0.361405
F	3.161256	-3.155067	-2.041790
F	2.230119	-4.450971	-0.570938
F	3.828143	-1.862227	4.085611
F	2.615232	-0.069814	4.329296
F	4.543928	0.046811	3.330579
O	-1.122362	0.736690	0.135690
C	-1.654292	-0.353062	0.624719
C	-2.994253	-0.194135	1.248769
C	-3.406046	-1.082923	2.252200
C	-3.843313	0.856777	0.870257
C	-4.647365	-0.924783	2.867712
H	-2.745710	-1.887719	2.564012
C	-5.085789	1.009548	1.483994
H	-3.506880	1.561295	0.116965
C	-5.491370	0.119189	2.483221
H	-4.950174	-1.609524	3.654829
H	-5.734412	1.831056	1.191490
H	-6.455427	0.246597	2.968012
H	-0.979896	-1.048049	1.133538
C	-1.892350	-1.518392	-0.983714
H	-0.838689	-1.727115	-1.116687
H	-2.277342	-0.763968	-1.658762
C	-2.737073	-2.584724	-0.648112
C	-2.288987	-3.778626	0.081695
C	-3.240203	-4.621417	0.693800
C	-0.922615	-4.108120	0.186710
C	-2.833187	-5.748697	1.398088
H	-4.291859	-4.370964	0.620524
C	-0.522001	-5.242447	0.885669
H	-0.163262	-3.503368	-0.293234
C	-1.473946	-6.062328	1.495890
H	-3.574866	-6.385362	1.871863
H	0.534621	-5.480486	0.941613
H	-1.158494	-6.946695	2.042500
O	-4.043871	-2.504730	-0.824280
Si	-5.131264	-1.952649	-2.057563
C	-4.689213	-0.256917	-2.731851
H	-5.500810	0.077025	-3.391772
H	-4.586137	0.487657	-1.936258
H	-3.769993	-0.256202	-3.327081
C	-6.767630	-1.930018	-1.146533
H	-7.578323	-1.606444	-1.810349
H	-7.026188	-2.923185	-0.762942
H	-6.730577	-1.238772	-0.297277
H	1.511099	0.746475	2.253246
C	-5.052654	-3.261910	-3.401771
H	-4.054055	-3.317417	-3.850513
H	-5.303463	-4.254683	-3.012347
H	-5.761211	-3.028274	-4.205991

TS 92

B3LYP/6-31G(d) = -3573.927089
B3LYP-D3(BJ)/def2-TZVPP/IEFPCM(propanonitrile) = -3575.506512
B3LYP-D3(BJ)/def2-TZVPP/IEFPCM(propanonitrile)//B3LYP-D3(BJ)/6-31G(d) Free Energy (Quasiharmonic) = -3574.789687

Frequencies (Top 3 out of 300)

1. -288.3018 cm^{-1}
2. 6.7328 cm^{-1}
3. 14.5980 cm^{-1}

B3LYP/6-31G(d) Molecular Geometry in Cartesian Coordinates

B	0.515981	0.914350	-0.834058
O	0.977882	1.224241	-2.198056
N	0.871093	2.237128	-0.086863
C	1.657668	3.123019	-0.957686
S	0.349031	2.755440	1.372263
C	1.595855	2.403112	-2.309709
O	2.053179	2.842928	-3.340083
H	3.082415	4.084698	0.338360
H	1.156571	4.092889	-1.085706
C	3.123334	3.411179	-0.522036
C	3.953616	2.209121	-0.174749
C	4.731986	1.368252	-1.057242
C	5.331699	0.359499	-0.251037
N	4.951351	0.596601	1.055280
C	4.109343	1.691413	1.087914
H	3.673822	2.033563	2.016210
C	6.383991	-0.614265	-2.156617
C	4.987552	1.366367	-2.440930
H	6.601388	-1.399155	-0.151477
H	5.076597	-0.048691	1.820441
C	5.807086	0.378909	-2.974220

H	7.017666	-1.375304	-2.604644
H	4.528385	2.109819	-3.085571
C	6.155244	-0.636652	-0.785381
H	6.003215	0.366592	-4.043142
C	-0.723464	4.166578	1.039584
O	-0.471658	1.659007	1.931190
O	1.450207	3.283217	2.195599
C	-2.449679	6.336413	0.623925
C	-1.688458	4.099940	0.031893
C	-0.609814	5.303461	1.840416
C	-1.474237	6.377036	1.628444
C	-2.542032	5.181745	-0.167512
H	-1.765133	3.217210	-0.593657
H	0.150887	5.339757	2.612396
H	-1.385696	7.262759	2.253048
H	-3.293288	5.127308	-0.951814
C	-3.359272	7.516293	0.376126
H	-4.357555	7.193859	0.061102
H	-2.960952	8.160740	-0.418809
H	-3.468060	8.134322	1.273109
O	-1.003019	0.759035	-1.015808
C	-1.897376	0.217610	-0.226328
H	-1.619465	0.075703	0.816312
H	3.574242	3.970248	-1.351161
C	-3.294144	0.674332	-0.457780
C	-4.226711	0.612201	0.587179
C	-3.681291	1.205906	-1.696759
C	-5.527731	1.074258	0.396470
H	-3.923552	0.223344	1.555985
C	-4.984248	1.664578	-1.884979
H	-2.948190	1.272217	-2.494662
C	-5.909929	1.598924	-0.840515
H	-6.239860	1.036044	1.216076
H	-5.276500	2.078469	-2.846311
H	-6.923500	1.962003	-0.987335
C	1.234367	-0.444312	-0.312456
C	1.600333	-0.688442	1.020611
C	1.569115	-1.430108	-1.256638
C	2.265999	-1.862383	1.389812
H	1.365934	0.044454	1.783811
C	2.216182	-2.612751	-0.888483
H	1.335436	-1.261155	-2.303068
C	2.578621	-2.833828	0.438752
H	3.087840	-3.747328	0.725250
C	2.427230	-3.704532	-1.899245
C	2.692210	-2.083456	2.811863
F	3.506336	-4.466020	-1.614915
F	1.355528	-4.547852	-1.923531
F	4.010744	-1.794515	3.006697
F	2.575120	-3.231635	-3.150976
F	2.536672	-3.379652	3.188272
F	1.998423	-1.329513	3.688705
C	-1.856671	-1.733995	-0.693477
H	-0.914916	-1.936465	-0.197283
H	-1.762067	-1.602459	-1.764022
C	-2.985804	-2.423654	-0.226965
C	-4.216582	-2.604291	-1.011075
C	-4.287433	-2.251337	-2.373696
C	-5.356556	-3.164229	-0.399010
C	-5.459557	-2.451132	-3.095476
H	-3.427032	-1.830384	-2.879894
C	-6.525979	-3.360068	-1.124072
H	-5.310775	-3.438170	0.648044
C	-6.581544	-3.004379	-2.474526
H	-5.495769	-2.178142	-4.145851
H	-7.396241	-3.790929	-0.637540
H	-7.494545	-3.161819	-3.042012
O	-3.061750	-2.884832	1.008172
Si	-1.947402	-3.368352	2.262301
C	-0.608066	-4.439314	1.494192
H	0.242935	-4.512830	2.181876
H	-0.986276	-5.452467	1.315515
H	-0.216257	-4.060328	0.545442
C	-1.331382	-1.838212	3.149604
H	-2.163747	-1.291732	3.608271
H	-0.646738	-2.131114	3.955195
H	-0.781403	-1.138967	2.514380
C	-3.063973	-4.378213	3.381909
H	-2.496531	-4.766192	4.236774
H	-3.887497	-3.774239	3.779337
H	-3.494786	-5.235749	2.853037

TS 93

B3LYP/6-31G(d) = -3573.924712
B3LYP-D3(BJ)/def2-TZVPP/IEFPCM(propanonitrile) = -3575.506343
B3LYP-D3(BJ)/def2-TZVPP/IEFPCM(propanonitrile)//B3LYP-D3(BJ)/6-31G(d) Free Energy (Quasiharmonic) = -3574.789539

Frequencies (Top 3 out of 300)

1. -281.6889 cm^{-1}
2. 7.6265 cm^{-1}
3. 15.0181 cm^{-1}

B3LYP/6-31G(d) Molecular Geometry in Cartesian Coordinates

B	0.597430	1.079199	-0.855751
O	1.142830	1.395359	-2.189001
N	0.993591	2.363599	-0.058121
C	1.909241	3.193339	-0.849681
S	0.372181	2.919319	1.347059
C	1.879081	2.509599	-2.222231
O	2.452591	2.922779	-3.204981
H	3.319451	4.036728	0.540559
H	1.505271	4.206279	-0.988661
C	3.359891	3.342808	-0.303441
C	4.026031	2.070878	0.135779
C	4.740560	1.101998	-0.665241
C	5.160650	0.062258	0.212709
N	4.741110	0.402758	1.483949
C	4.039260	1.591578	1.422539
H	3.585881	2.017988	2.306259
C	6.154810	-1.136323	-1.594231
C	5.058150	0.999288	-2.032301
H	6.175879	-1.842273	0.451339
H	4.723790	-0.224552	2.273819
C	5.755690	-0.116042	-2.481471
H	6.695229	-1.998163	-1.976751
H	4.736741	1.766578	-2.730491
C	5.867050	-1.058122	-0.235711
H	5.994440	-0.207683	-3.537731
C	-0.604779	4.373900	0.915089
O	1.428631	3.412199	2.247819
O	-0.541839	1.869440	1.844179
C	-2.178268	6.619070	0.329079
C	-0.473648	5.529910	1.684929
C	-1.512889	4.324160	-0.145171
C	-2.290468	5.443730	-0.428801
C	-1.262088	6.641510	1.388129
H	0.241362	5.550489	2.499939
H	-1.603719	3.422970	-0.742071
H	-2.998018	5.403941	-1.253751
H	-1.159868	7.542360	1.988679
C	-3.003887	7.837501	-0.010221
H	-2.531097	8.424760	-0.808691
H	-4.003288	7.559471	-0.362291
H	-3.119437	8.497601	0.855449
H	3.931531	3.832818	-1.101301
C	1.230600	-0.317691	-0.317061
C	1.293710	-0.680731	1.037799

C	1.788070	-1.213571	-1.247051
C	1.897489	-1.877051	1.440359
C	2.378979	-2.414741	-0.847971
H	1.781090	-0.951221	-2.299881
C	2.450899	-2.749101	0.502559
H	2.924169	-3.672861	0.815409
C	2.863979	-3.399591	-1.873771
C	2.002189	-2.209521	2.898979
F	3.897069	-4.144362	-1.420891
F	1.872398	-4.280101	-2.202431
F	3.251449	-2.811122	-3.019201
F	2.024319	-3.550121	3.113519
F	3.135909	-1.714892	3.466919
F	0.966539	-1.714241	3.615909
O	-0.911980	1.015520	-1.063691
C	-1.763680	0.306060	-0.345941
C	-3.153630	0.852931	-0.323501
C	-3.896600	0.794231	0.861819
C	-3.713620	1.451031	-1.460921
C	-5.186190	1.326201	0.908869
H	-3.448170	0.366561	1.754459
C	-5.004409	1.973701	-1.413331
H	-3.124780	1.516231	-2.371361
C	-5.744029	1.911461	-0.228591
H	-5.748350	1.295661	1.838269
H	-5.432659	2.436211	-2.298631
H	-6.747299	2.327282	-0.190861
H	-1.405900	-0.059690	0.617479
C	-1.849681	-1.384100	-1.232491
H	-2.105390	-1.063340	-2.236891
H	-0.806111	-1.645400	-1.083901
C	-2.782571	-2.196120	-0.562261
C	-4.174471	-2.356429	-0.998951
C	-5.200781	-2.532449	-0.050271
C	-4.507041	-2.328029	-2.367701
C	-6.521951	-2.671658	-0.460481
H	-4.955191	-2.518619	1.005569
C	-5.828101	-2.487349	-2.773661
H	-3.725811	-2.218329	-3.112501
C	-6.837431	-2.655418	-1.822131
H	-7.308311	-2.787518	0.279539
H	-6.071021	-2.481948	-3.831971
H	-7.869281	-2.772268	-2.141221
O	-2.438131	-2.691090	0.611109
Si	-2.144541	-4.240400	1.342399
C	-2.058541	-3.819780	3.163369
H	-1.880922	-4.726840	3.754249
H	-1.236121	-3.125890	3.368089
H	-2.987871	-3.365209	3.524509
C	-0.519272	-4.898240	0.677049
H	-0.471872	-5.984290	0.827109
H	-0.374832	-4.708480	-0.392501
H	0.326918	-4.452541	1.207279
H	0.873470	-0.019491	1.789199
C	-3.557392	-5.409019	0.923189
H	-4.522752	-5.069519	1.311219
H	-3.662612	-5.557979	-0.157391
H	-3.347352	-6.390339	1.367839

TS 94

B3LYP/6-31G(d) = -3573.931825
B3LYP-D3(BJ)/def2-TZVPP/IEFPCM(propanonitrile) = -3575.506028
B3LYP-D3(BJ)/def2-TZVPP/IEFPCM(propanonitrile)//B3LYP-D3(BJ)/6-31G(d) Free Energy (Quasiharmonic) = -3574.789431

Frequencies (Top 3 out of 300)

1. -275.9130 cm^{-1}
2. 9.0206 cm^{-1}
3. 13.5492 cm^{-1}

B3LYP/6-31G(d) Molecular Geometry in Cartesian Coordinates

B	0.474600	0.529850	-0.119920
O	0.219150	-0.169410	-1.414180
N	0.673210	1.987270	-0.603040
C	0.776280	2.036940	-2.066540
S	0.455130	3.382170	0.239420
C	0.369800	0.623200	-2.481670
O	0.180090	0.256970	-3.623000
H	2.248120	3.536811	-2.507630
H	0.023030	2.713220	-2.496330
C	2.161510	2.456031	-2.645650
C	3.341600	1.775721	-2.019840
C	3.935400	0.506141	-2.368380
C	4.991370	0.271861	-1.442300
N	5.046280	1.361851	-0.595350
C	4.037280	2.242191	-0.931580
H	3.878570	3.149481	-0.365200
C	5.487401	-1.827129	-2.461290
C	3.679000	-0.458769	-3.359610
H	6.571781	-1.050109	-0.758150
H	5.544820	1.387311	0.282240
C	4.452891	-1.612569	-3.394810
H	6.071481	-2.742139	-2.510590
H	2.872070	-0.314099	-4.073040
C	5.773391	-0.886819	-1.477590
H	4.256621	-2.367129	-4.152150
C	-1.177301	4.003200	-0.220400
O	1.409689	4.391550	-0.245420
O	0.405870	3.029150	1.665230
C	-3.714431	4.961049	-0.950310
C	-2.317370	3.503680	0.415390
C	-1.291801	4.986100	-1.204020
C	-2.556931	5.453730	-1.563940
C	-3.571401	3.985909	0.049670
H	-2.212480	2.761750	1.198500
H	-0.396241	5.396830	-1.657590
H	-2.641811	6.223730	-2.326960
H	-4.457630	3.612499	0.559000
C	-5.082311	5.455359	-1.357330
H	-5.760351	5.512039	-0.498810
H	-5.544071	4.781949	-2.091840
H	-5.029791	6.448589	-1.814180
H	2.116180	2.266031	-3.725360
C	1.686870	-0.182390	0.681550
C	2.405950	0.469011	1.697060
C	2.072921	-1.492749	0.364350
C	3.473590	-0.158289	2.343430
H	2.135720	1.482181	1.976860
C	3.118241	-2.136709	1.034510
H	1.577271	-2.017260	-0.445770
C	3.831831	-1.470139	2.026140
H	4.657981	-1.955289	2.532730
C	3.445701	-3.565379	0.701850
C	4.321660	0.618811	3.307910
F	3.294121	-3.830999	-0.613420
F	2.631551	-4.430069	1.364250
F	4.713241	-3.893359	1.039700
F	5.340120	1.267431	2.659660
F	4.912670	-0.172609	4.230740
F	3.628480	1.566351	3.966630
O	-0.795850	0.444310	0.725460
C	-1.489840	-0.654230	0.858700
C	-2.200149	-0.822940	2.150040
C	-2.223810	0.223900	3.085540
C	-2.775629	-2.058990	2.483190
C	-2.834870	0.037209	4.324470
H	-1.716130	1.154110	2.851950
C	-3.378019	-2.243211	3.724830

H	-2.742329	-2.879810	1.770620
C	-3.415129	-1.192301	4.645430
H	-2.839230	0.847149	5.048490
H	-3.813129	-3.206001	3.977050
H	-3.882119	-1.337121	5.615810
H	-1.066329	-1.568360	0.434510
C	-2.869970	-0.444061	-0.613690
H	-3.216290	0.539709	-0.321650
H	-2.118770	-0.438800	-1.394690
C	-3.805009	-1.486671	-0.643300
C	-5.073529	-1.470501	0.101710
C	-5.439400	-0.382221	0.918970
C	-5.958329	-2.562441	-0.004820
C	-6.649350	-0.389121	1.604640
H	-4.774380	0.465029	1.035740
C	-7.169299	-2.562171	0.678410
H	-5.683279	-3.404031	-0.629240
C	-7.518799	-1.476091	1.485370
H	-6.913810	0.454289	2.235440
H	-7.842179	-3.409462	0.583210
H	-8.464989	-1.476642	2.019340
O	-3.566029	-2.617171	-1.282340
Si	-2.597139	-3.134940	-2.647010
C	-0.860979	-3.489670	-2.045720
H	-0.853599	-4.120880	-1.149650
H	-0.304929	-4.023870	-2.826620
H	-0.316159	-2.562750	-1.840030
C	-3.506819	-4.701631	-3.139360
H	-2.997579	-5.192461	-3.977590
H	-3.546589	-5.418391	-2.311250
H	-4.534799	-4.489141	-3.454480
C	-2.664709	-1.821160	-3.984090
H	-3.639959	-1.322041	-4.024310
H	-2.507449	-2.303330	-4.957590
H	-1.878849	-1.066290	-3.870800

TS 95

B3LYP/6-31G(d) = -3573.92809
B3LYP-D3(BJ)/def2-TZVPP/IEFPCM(propanonitrile) = -3575.506169
B3LYP-D3(BJ)/def2-TZVPP/IEFPCM(propanonitrile)//B3LYP-D3(BJ)/6-31G(d) Free Energy (Quasiharmonic) = -3574.789404

Frequencies (Top 3 out of 300)

1. -295.8834 cm^{-1}
2. 8.4862 cm^{-1}
3. 14.3531 cm^{-1}

B3LYP/6-31G(d) Molecular Geometry in Cartesian Coordinates

B	-0.228472	-0.624447	-1.161699
O	-0.204134	-0.471164	-2.629341
N	-1.476059	-1.558613	-0.936372
C	-2.101217	-1.827806	-2.244257
S	-1.365069	-2.812803	0.148446
C	-1.166698	-1.151330	-3.254719
O	-1.311864	-1.181834	-4.457117
H	-4.192228	-1.744897	-1.683470
H	-2.116351	-2.901633	-2.466584
C	-3.538618	-1.256869	-2.413459
C	-3.642008	0.241213	-2.287044
C	-4.131661	1.002217	-1.161312
C	-3.979948	2.380026	-1.483427
N	-3.455158	2.445751	-2.756898
C	-3.240235	1.163806	-3.225401
H	-2.806928	0.999444	-4.202128
C	-4.856275	3.034007	0.636169
C	-4.684356	0.663690	0.085970
H	-4.183531	4.447547	-0.860095
H	-3.088635	3.283848	-3.180994
C	-5.037510	1.675856	0.970360
H	-5.133087	3.803036	1.352285
H	-4.826806	-0.376906	0.359555
C	-4.330450	3.403488	-0.596611
H	-5.457811	1.419628	1.939505
C	-3.047344	-3.150671	0.691493
O	-0.637662	-2.278657	1.316171
O	-0.894151	-4.052893	-0.497063
C	-5.629578	-3.745607	1.606554
C	-3.599894	-2.402175	1.733700
C	-3.758910	-4.204060	0.116725
C	-5.045935	-4.489047	0.572765
C	-4.882849	-2.706606	2.182923
H	-3.029985	-1.600886	2.191582
H	-3.296847	-4.804607	-0.659481
H	-5.599773	-5.311015	0.125647
H	-5.310848	-2.129962	2.999619
C	-7.031486	-4.042632	2.083016
H	-7.119180	-3.924484	3.168511
H	-7.335186	-5.062306	1.825817
H	-7.756517	-3.358011	1.623336
H	-3.874749	-1.577628	-3.406948
C	-0.373389	0.819035	-0.437143
C	-0.807488	0.953119	0.892843
C	-0.187118	2.001562	-1.171381
C	-1.057716	2.207606	1.455229
H	-0.980734	0.058768	1.482631
C	-0.434538	3.259663	-0.611078
H	0.120320	1.932657	-2.210166
C	-0.874472	3.371240	0.707000
H	-1.090898	4.342601	1.136393
C	-0.325989	4.493865	-1.460097
C	-1.508785	2.314797	2.884654
F	0.611496	4.371602	-2.424372
F	-0.020204	5.590774	-0.732384
F	-1.498472	4.778939	-2.100185
F	-0.447708	2.450895	3.735192
F	-2.186356	1.223477	3.294858
F	-2.298100	3.390234	3.092955
O	1.045102	-1.386289	-0.851133
C	1.913700	-1.171660	0.106055
C	2.739583	-2.353696	0.484142
C	2.627324	-3.550105	-0.238991
C	3.579631	-2.305552	1.607522
C	3.358000	-4.672355	0.149208
H	1.934282	-3.601348	-1.070191
C	4.309506	-3.428304	1.991549
H	3.649321	-1.393658	2.194562
C	4.204974	-4.613864	1.258466
H	3.250942	-5.599754	-0.406430
H	4.948710	-3.382576	2.869011
H	4.767592	-5.492592	1.562515
H	1.585667	-0.551558	0.944206
C	3.088881	0.174645	-0.697150
H	3.307598	-0.340261	-1.626535
H	2.318301	0.931618	-0.789251
C	4.185462	0.488263	0.126777
C	4.091289	1.354236	1.308579
C	2.866478	1.932453	1.702478
C	5.241466	1.605629	2.085485
C	2.794664	2.726110	2.842889
H	1.962077	1.759861	1.130884
C	5.166702	2.411754	3.215186
H	6.184727	1.159985	1.792698
C	3.943714	2.970513	3.598825
H	1.836942	3.135084	3.145300
H	6.060581	2.600731	3.802619
H	3.885580	3.592467	4.487710
O	5.361076	-0.088366	-0.039152
Si	6.248912	-0.674597	-1.418678

C	7.929658	-1.009874	-0.660258
H	8.364167	-0.103187	-0.224870
H	7.859646	-1.765383	0.130314
H	8.628851	-1.384720	-1.417374
C	6.283667	0.733297	-2.662695
H	6.724760	1.639189	-2.231927
H	6.888686	0.450348	-3.532904
H	5.282626	0.986184	-3.028941
C	5.496324	-2.238345	-2.125194
H	5.331172	-2.994775	-1.351535
H	4.542318	-2.067972	-2.634488
H	6.190247	-2.660256	-2.864200

TS 96

B3LYP/6-31G(d) = -3573.929195
B3LYP-D3(BJ)/def2-TZVPP/IEFPCM(propanonitrile) = -3575.505818
B3LYP-D3(BJ)/def2-TZVPP/IEFPCM(propanonitrile)//B3LYP-D3(BJ)/6-31G(d) Free Energy (Quasiharmonic) = -3574.789345

Frequencies (Top 3 out of 300)

1. -269.4868 cm^{-1}
2. 7.3481 cm^{-1}
3. 11.2606 cm^{-1}

B3LYP/6-31G(d) Molecular Geometry in Cartesian Coordinates

B	0.613344	0.675113	-0.539715
O	0.551703	0.510430	-2.008885
N	1.565350	1.887928	-0.401066
C	2.162751	2.221437	-1.700190
S	1.742367	2.909916	0.873188
C	1.374813	1.337067	-2.672859
O	1.490772	1.350854	-3.876773
H	4.202462	2.627165	-1.139009
H	1.963787	3.269333	-1.966701
C	3.689549	1.976777	-1.852402
C	4.136277	0.551062	-1.675891
C	4.723931	-0.055359	-0.502554
C	4.977095	-1.419130	-0.817014
N	4.578604	-1.619625	-2.123368
C	4.063221	-0.440205	-2.626524
H	3.684486	-0.389651	-3.638291
C	5.859238	-1.816321	1.360729
C	5.060248	0.416128	0.779993
H	5.704666	-3.354383	-0.155720
H	4.507199	-2.520471	-2.569268
C	5.624315	-0.465907	1.693344
H	6.295716	-2.481821	2.101170
H	4.855363	1.445007	1.058256
C	5.537458	-2.312051	0.102859
H	5.880920	-0.112444	2.688524
C	1.010224	4.475931	0.343103
O	3.164929	3.213793	1.100051
O	0.933897	2.366780	1.976129
C	-0.132563	6.935694	-0.377172
C	1.735339	5.654454	0.518848
C	-0.279171	4.511701	-0.192950
C	-0.838573	5.735377	-0.550753
C	1.159620	6.873197	0.158417
H	2.739418	5.606273	0.925401
H	-0.831179	3.589973	-0.338078
H	-1.839905	5.758959	-0.974889
H	1.728618	7.790299	0.291680
C	-0.760301	8.259894	-0.743203
H	-1.413408	8.623479	0.061441
H	-1.375347	8.176474	-1.645916
H	-0.001076	9.028487	-0.919394
H	3.946190	2.329455	-2.858822
C	1.061084	-0.707144	0.179890
C	1.196385	-0.832170	1.573546
C	1.303371	-1.852433	-0.592866
C	1.547928	-2.050581	2.160417
H	1.038759	0.039759	2.201771
C	1.651513	-3.075027	-0.007189
H	1.218852	-1.783536	-1.672643
C	1.775451	-3.182796	1.376672
H	2.045103	-4.125648	1.837156
C	1.920262	-4.262784	-0.883955
C	1.602475	-2.162660	3.660040
F	1.783003	-5.433769	-0.228063
F	3.175367	-4.260467	-1.408969
F	1.072413	-4.300235	-1.949973
F	2.082309	-1.046504	4.239704
F	2.369510	-3.199505	4.067851
F	0.362574	-2.371101	4.181689
O	-0.785781	1.073851	-0.050271
C	-1.895108	0.646593	-0.598183
C	-3.098095	1.479288	-0.327904
C	-3.123966	2.353712	0.771377
C	-4.195056	1.431615	-1.199532
C	-4.235649	3.165835	0.989072
H	-2.260260	2.402628	1.427750
C	-5.302893	2.251470	-0.981514
H	-4.172450	0.766827	-2.059572
C	-5.326555	3.117180	0.114484
H	-4.246057	3.847592	1.834941
H	-6.141077	2.221494	-1.672167
H	-6.186556	3.760228	0.280975
H	-1.812772	0.237964	-1.608721
C	-2.266574	-1.084515	0.318781
H	-1.294086	-1.508589	0.103937
H	-2.376356	-0.709246	1.329765
C	-3.381844	-1.690020	-0.274381
C	-3.311479	-2.448622	-1.531884
C	-4.483002	-2.666127	-2.285922
C	-2.093278	-2.982189	-1.999210
C	-4.430800	-3.378481	-3.478852
H	-5.423289	-2.260672	-1.930323
C	-2.050493	-3.706435	-3.185979
H	-1.179009	-2.864837	-1.429919
C	-3.215521	-3.901062	-3.931635
H	-5.337920	-3.529563	-4.056893
H	-1.104759	-4.121700	-3.518858
H	-3.178403	-4.462997	-4.860667
O	-4.605874	-1.479755	0.179101
Si	-5.398405	-1.482311	1.722999
C	-7.057530	-0.716292	1.312746
H	-7.583717	-1.289213	0.541169
H	-7.699543	-0.684128	2.201218
H	-6.933803	0.309729	0.949359
C	-5.524284	-3.293748	2.204006
H	-6.054301	-3.876913	1.442974
H	-4.533922	-3.742363	2.344128
H	-6.069758	-3.401694	3.149639
C	-4.475935	-0.492370	3.023349
H	-3.534278	-0.960675	3.329394
H	-5.106179	-0.422564	3.919802
H	-4.263752	0.528669	2.690107

TS 97

B3LYP/6-31G(d) = -3573.932476
B3LYP-D3(BJ)/def2-TZVPP/IEFPCM(propanonitrile) = -3575.505712
B3LYP-D3(BJ)/def2-TZVPP/IEFPCM(propanonitrile)//B3LYP-D3(BJ)/6-31G(d) Free Energy (Quasiharmonic) = -3574.789201

Frequencies (Top 3 out of 300)

1. -281.5479 cm^{-1}
2. 11.3610 cm^{-1}
3. 13.2194 cm^{-1}

B3LYP/6-31G(d) Molecular Geometry in Cartesian Coordinates

B	-0.729530	-0.340229	0.044494
O	-0.216102	-0.990944	1.275337
N	-1.671174	-1.441890	-0.497171
C	-1.673574	-2.620773	0.375741
S	-2.755979	-1.314996	-1.730916
C	-0.792927	-2.166694	1.538388
O	-0.614157	-2.790910	2.563933
H	-1.799911	-4.218243	-1.062544
H	-2.679489	-2.818342	0.773691
C	-1.136039	-3.946284	-0.237917
C	0.288670	-3.893613	-0.708671
C	1.468159	-4.301603	0.020457
C	2.590236	-4.035434	-0.813319
N	2.104484	-3.509217	-1.993430
C	0.727930	-3.415494	-1.919676
H	0.150060	-3.017197	-2.742021
C	4.087718	-4.870526	0.843901
C	1.689015	-4.873092	1.287241
H	4.746720	-4.075169	-1.057317
H	2.674695	-3.116724	-2.726202
C	2.990716	-5.153927	1.683979
H	5.095001	-5.094992	1.185017
H	0.854402	-5.069409	1.953774
C	3.903455	-4.306588	-0.413053
H	3.169848	-5.595006	2.661129
C	-4.387903	-1.388401	-0.970543
O	-2.660390	-2.497566	-2.601706
O	-2.599429	0.031673	-2.298876
C	-6.925643	-1.482879	0.218563
C	-5.088221	-2.594089	-0.930726
C	-4.943208	-0.224819	-0.431865
C	-6.203135	-0.280740	0.156675
C	-6.348413	-2.633402	-0.331941
H	-4.658070	-3.481675	-1.382252
H	-4.395626	0.709247	-0.488402
H	-6.638583	0.626745	0.569426
H	-6.893680	-3.573625	-0.300394
C	-8.304040	-1.522353	0.834855
H	-8.591136	-2.541900	1.110573
H	-9.060709	-1.146199	0.133673
H	-8.359491	-0.898894	1.734183
H	-1.248158	-4.710867	0.539875
C	0.472574	0.150403	-0.926209
C	0.273382	0.969979	-2.050475
C	1.789852	-0.261087	-0.665357
C	1.339027	1.376488	-2.860922
H	-0.732735	1.278818	-2.312096
C	2.855578	0.139232	-1.475644
H	1.983700	-0.900482	0.187833
C	2.639255	0.966099	-2.579205
H	3.467435	1.296208	-3.195393
C	4.250435	-0.332860	-1.184771
C	1.064050	2.238938	-4.062998
F	4.433379	-0.638438	0.121212
F	5.179435	0.604555	-1.502260
F	4.576916	-1.444536	-1.897266
F	0.132328	3.184337	-3.796159
F	0.601765	1.519275	-5.108714
F	2.176214	2.884011	-4.490085
O	-1.595330	0.826470	0.555783
C	-1.261429	2.081712	0.601491
C	-2.376415	3.045576	0.799589
C	-3.532116	2.677808	1.504326
C	-2.276254	4.336163	0.260215
C	-4.568157	3.594060	1.670559
H	-3.608971	1.673191	1.907663
C	-3.318468	5.248165	0.422865
H	-1.390489	4.616133	-0.305084
C	-4.463779	4.880292	1.132039
H	-5.460085	3.306212	2.220378
H	-3.240430	6.241088	-0.010830
H	-5.275431	5.591042	1.260148
H	-0.457942	2.417685	-0.055741
C	-0.220623	2.464247	2.362975
H	-0.755123	1.786565	3.019016
H	-0.521595	3.500762	2.428066
C	1.142699	2.210841	2.147267
C	2.020378	3.135114	1.401864
C	3.275255	2.681315	0.952043
C	1.638848	4.461398	1.114433
C	4.113030	3.519587	0.223079
H	3.570487	1.659337	1.152612
C	2.487800	5.301536	0.401087
H	0.686392	4.848984	1.459986
C	3.724675	4.832495	-0.050893
H	5.060596	3.137233	-0.143908
H	2.184796	6.324208	0.196058
H	4.380284	5.488495	-0.616644
O	1.704352	1.075509	2.491903
Si	1.840117	0.118279	3.945113
C	2.952921	1.174801	5.041402
H	3.917665	1.374131	4.561573
H	2.490816	2.139633	5.282868
H	3.152450	0.660039	5.989479
C	2.673254	-1.451187	3.376157
H	3.456777	-1.250961	2.637464
H	3.139891	-1.961406	4.228102
H	1.946946	-2.138168	2.932070
C	0.187611	-0.185011	4.777713
H	0.372147	-0.736773	5.709325
H	-0.340267	0.736048	5.048125
H	-0.453638	-0.811910	4.151651

TS 98

B3LYP/6-31G(d) = -3573.929979
B3LYP-D3(BJ)/def2-TZVPP/IEFPCM(propanonitrile) = -3575.506019
B3LYP-D3(BJ)/def2-TZVPP/IEFPCM(propanonitrile)//B3LYP-D3(BJ)/6-31G(d) Free Energy (Quasiharmonic) = -3574.789075

Frequencies (Top 3 out of 300)

1. -275.3617 cm^{-1}
2. 10.6002 cm^{-1}
3. 10.9031 cm^{-1}

B3LYP/6-31G(d) Molecular Geometry in Cartesian Coordinates

B	0.292250	-0.186270	-0.816160
O	0.315310	-1.661830	-0.957380
N	0.769560	0.264750	-2.242210
C	1.017920	-0.928870	-3.087720
S	0.224719	1.577540	-3.116540
C	0.591161	-2.092370	-2.200490
O	0.495741	-3.248200	-2.543770
H	2.700560	-0.202969	-4.185980
H	0.368400	-0.936370	-3.972300
C	2.478480	-1.092169	-3.590180
C	3.510770	-1.297039	-2.519720
C	3.993961	-2.556928	-1.996270
C	4.950701	-2.254208	-0.988590
N	5.060480	-0.878848	-0.927440
C	4.180230	-0.315508	-1.831120
H	4.103130	0.758772	-1.922340
C	5.317391	-4.569878	-0.562380

C	3.719031	-3.907199	-2.282060
H	6.319481	-2.989818	0.526400
H	5.527920	-0.378908	-0.187510
C	4.383542	-4.895788	-1.568110
H	5.812152	-5.366828	-0.013410
H	2.976161	-4.170379	-3.028600
C	5.611851	-3.246918	-0.256600
H	4.173472	-5.941058	-1.778880
C	0.514299	2.983250	-2.046710
O	1.091199	1.703540	-4.298750
O	-1.235571	1.503640	-3.349330
C	0.956588	5.280380	-0.506920
C	-0.574291	3.689240	-1.536190
C	1.821839	3.418531	-1.816950
C	2.033158	4.556621	-1.045270
C	-0.343862	4.831120	-0.768330
H	-1.581241	3.348950	-1.749530
H	2.656949	2.879131	-2.251370
H	3.049348	4.896491	-0.861520
H	-1.190322	5.385010	-0.370100
C	1.201598	6.497541	0.350860
H	1.469888	6.195341	1.369540
H	2.026297	7.103491	-0.040090
H	0.311377	7.131330	0.411950
H	2.478261	-1.948379	-4.275740
C	1.167210	0.305661	0.454620
C	0.920409	1.525390	1.105960
C	2.175600	-0.508749	0.989430
C	1.649439	1.917701	2.230350
C	2.894080	-0.130809	2.131300
H	2.386270	-1.467829	0.526970
C	2.639559	1.087551	2.757000
H	3.182999	1.374531	3.650170
C	3.948760	-1.041949	2.692860
C	1.407179	3.250261	2.880200
F	4.159100	-0.828518	4.011370
F	3.625761	-2.344039	2.541560
F	5.161360	-0.868988	2.087600
F	1.456449	3.172431	4.230850
F	2.349818	4.163311	2.519040
F	0.209049	3.781730	2.551550
O	-1.144060	0.241250	-0.481060
C	-2.193710	-0.115811	-1.170790
C	-3.405310	0.723119	-1.029620
C	-3.625571	1.490309	0.124190
C	-4.319300	0.793299	-2.091250
C	-4.748781	2.310248	0.213300
H	-2.906311	1.446029	0.935580
C	-5.440601	1.616788	-2.000260
H	-4.126110	0.233859	-3.002820
C	-5.659191	2.374378	-0.846030
H	-4.911401	2.906318	1.107080
H	-6.132061	1.682578	-2.835600
H	-6.528281	3.023228	-0.778230
H	-2.022250	-0.523011	-2.167110
C	-2.793179	-2.005081	-0.562350
H	-1.815189	-2.447841	-0.698630
H	-3.510179	-2.198411	-1.352420
C	-3.300879	-1.873951	0.731010
C	-2.478530	-1.848161	1.945380
C	-1.113889	-2.202530	1.919000
C	-3.063140	-1.481321	3.176580
C	-0.362059	-2.190610	3.090820
H	-0.631619	-2.482510	0.990700
C	-2.305570	-1.468741	4.340880
H	-4.108840	-1.198521	3.202140
C	-0.953500	-1.825720	4.301510
H	0.689871	-2.456410	3.051920
H	-2.765590	-1.178901	5.281330
H	-0.363010	-1.816830	5.213540
O	-4.592020	-1.623902	0.927110
Si	-6.091869	-2.309302	0.426520
C	-6.217909	-3.968782	1.299990
H	-5.421549	-4.651852	0.982010
H	-7.176079	-4.452212	1.072650
H	-6.149179	-3.857022	2.387690
C	-6.208959	-2.521992	-1.437820
H	-7.229049	-2.835482	-1.695580
H	-6.009190	-1.583882	-1.965960
H	-5.529139	-3.290202	-1.822190
H	0.135119	2.177500	0.741980
C	-7.334960	-1.055312	1.051770
H	-7.271060	-0.936642	2.139090
H	-7.160410	-0.075082	0.594560
H	-8.358480	-1.364883	0.808490

TS 99

B3LYP/6-31G(d) = -3573.926956
B3LYP-D3(BJ)/def2-TZVPP/IEFPCM(propanonitrile) = -3575.505839
B3LYP-D3(BJ)/def2-TZVPP/IEFPCM(propanonitrile)//B3LYP-D3(BJ)/6-31G(d) Free Energy (Quasiharmonic) = -3574.789032

Frequencies (Top 3 out of 300)

1. -275.4414 cm^{-1}
2. 10.1109 cm^{-1}
3. 13.8943 cm^{-1}

B3LYP/6-31G(d) Molecular Geometry in Cartesian Coordinates

B	-0.549920	-0.342270	0.326590
O	-0.391510	-0.402080	1.795640
N	-1.327160	-1.665290	0.063050
C	-1.465390	-2.449930	1.295570
S	-2.138740	-2.114930	-1.299250
C	-0.913600	-1.500690	2.360720
O	-0.923100	-1.704480	3.554050
H	-1.124490	-4.445100	0.550000
H	-2.525530	-2.629320	1.528910
C	-0.740170	-3.825300	1.364080
C	0.763500	-3.790770	1.327860
C	1.635600	-4.003149	0.193950
C	2.972800	-3.902229	0.668110
N	2.908600	-3.658239	2.025200
C	1.584440	-3.578969	2.410930
H	1.318640	-3.381649	3.440320
C	3.837650	-4.304639	-1.515260
C	1.421000	-4.269289	-1.171140
H	5.095630	-3.944949	0.208850
H	3.697480	-3.429119	2.608560
C	2.520740	-4.418729	-2.006830
H	4.675860	-4.418929	-2.197770
H	0.411850	-4.331700	-1.565890
C	4.082440	-4.043779	-0.172330
H	2.364680	-4.617609	-3.063800
C	-3.890650	-2.082880	-0.868670
O	-1.851320	-3.523560	-1.615070
O	-1.902730	-1.067980	-2.302460
C	-6.612380	-2.019980	-0.187070
C	-4.610800	-0.894510	-0.997200
C	-4.520490	-3.246180	-0.421040
C	-5.872800	-3.205060	-0.081260
C	-5.961200	-0.870280	-0.657980
H	-4.119750	-0.008110	-1.379180
H	-3.963050	-4.175280	-0.371050
H	-6.363020	-4.113400	0.261090
H	-6.519140	0.056940	-0.766830
C	-8.069770	-1.976850	0.207520
H	-8.539480	-2.961540	0.116290
H	-8.632890	-1.272590	-0.414180

H	-8.187380	-1.654850	1.251010
H	-1.073240	-4.279660	2.305260
C	0.863400	-0.158820	-0.431070
C	0.947730	0.204881	-1.784080
C	2.067110	-0.347879	0.260880
C	2.183560	0.391501	-2.408730
H	0.035790	0.332140	-2.356140
C	3.305920	-0.169579	-0.362420
H	2.036950	-0.635899	1.305340
C	3.373660	0.203451	-1.703310
H	4.332550	0.348521	-2.189020
C	4.578410	-0.277939	0.425830
C	2.253590	0.781561	-3.857520
F	4.452030	-1.047069	1.532480
F	4.997650	0.947631	0.870770
F	5.598790	-0.779449	-0.300770
F	2.480020	-0.266979	-4.674750
F	3.262570	1.669491	-4.080920
F	1.111800	1.375921	-4.282970
O	-1.416670	0.892620	-0.013650
C	-2.004730	1.630320	0.885160
C	-3.243540	2.336370	0.453490
C	-3.511710	2.544390	-0.908250
C	-4.160650	2.787270	1.412880
C	-4.678230	3.200590	-1.298050
H	-2.812360	2.165100	-1.646470
C	-5.328820	3.438790	1.020230
H	-3.965350	2.615270	2.469300
C	-5.587590	3.651230	-0.336210
H	-4.882270	3.354000	-2.354120
H	-6.038430	3.775770	1.770680
H	-6.496590	4.161220	-0.643040
H	-2.007640	1.241750	1.905170
C	-0.939890	3.290590	1.407140
H	-1.449420	3.468890	2.347020
H	-1.255380	3.953020	0.609860
C	0.442480	3.028070	1.470830
C	1.117720	2.434471	2.628270
C	2.499670	2.168701	2.563110
C	0.420180	2.112520	3.809720
C	3.161370	1.597561	3.644040
H	3.041520	2.384641	1.652010
C	1.085240	1.545431	4.889080
H	-0.646930	2.288120	3.886180
C	2.457080	1.287781	4.810100
H	4.220990	1.377221	3.562070
H	0.532090	1.288880	5.787060
H	2.972340	0.837501	5.654100
O	1.217820	3.249651	0.427910
Si	1.231250	4.433661	-0.850260
C	0.935590	6.099331	-0.021710
H	1.682180	6.292121	0.757250
H	1.020090	6.901781	-0.764960
H	-0.056490	6.179260	0.435330
C	2.968950	4.305361	-1.532020
H	3.160300	5.135331	-2.223880
H	3.718150	4.358351	-0.734420
H	3.122610	3.374001	-2.085490
C	-0.058630	4.053730	-2.156780
H	0.143700	3.099570	-2.652710
H	-0.020510	4.836640	-2.925910
H	-1.080650	4.029250	-1.765300

TS 100

B3LYP/6-31G(d) = -3573.934606
B3LYP-D3(BJ)/def2-TZVPP/IEFPCM(propanonitrile) = -3575.505945
B3LYP-D3(BJ)/def2-TZVPP/IEFPCM(propanonitrile)//B3LYP-D3(BJ)/6-31G(d) Free Energy (Quasiharmonic) = -3574.78881

Frequencies (Top 3 out of 300)

1. -265.8025 cm^{-1}
2. 12.8417 cm^{-1}
3. 14.9717 cm^{-1}

B3LYP/6-31G(d) Molecular Geometry in Cartesian Coordinates

B	0.240846	-0.308303	0.042269
O	-0.530163	0.431846	-0.992351
N	0.224218	-1.744136	-0.564469
C	-0.301548	-1.730040	-1.934209
S	1.099343	-3.022411	0.019203
C	-0.819229	-0.294972	-2.074067
O	-1.407661	0.139676	-3.042579
H	-0.928974	-3.747220	-2.335272
H	0.509325	-1.845288	-2.672661
C	-1.399605	-2.762376	-2.303931
C	-2.605027	-2.814097	-1.407839
C	-3.878780	-2.154845	-1.592926
C	-4.728453	-2.574335	-0.528152
N	-3.998002	-3.444214	0.253843
C	-2.727586	-3.576889	-0.270475
H	-1.992110	-4.219038	0.192754
C	-6.534102	-1.266979	-1.379973
C	-4.396369	-1.274198	-2.560402
H	-6.686041	-2.474375	0.409358
H	-4.305408	-3.845899	1.125825
C	-5.713960	-0.843594	-2.447246
H	-7.561045	-0.916102	-1.321305
H	-3.767147	-0.926502	-3.373936
C	-6.051947	-2.135730	-0.406617
H	-6.124868	-0.174240	-3.198764
C	2.683040	-3.046439	-0.837843
O	0.416656	-4.268757	-0.367419
O	1.363638	-2.732339	1.434386
C	5.138552	-3.064270	-2.195208
C	2.771500	-3.627462	-2.106065
C	3.814034	-2.497999	-0.228754
C	5.028906	-2.513351	-0.910687
C	3.993893	-3.626692	-2.775927
H	1.903445	-4.106289	-2.546805
H	3.748449	-2.084236	0.770566
H	5.909957	-2.094017	-0.430785
H	4.061706	-4.083129	-3.760586
C	6.454016	-3.041303	-2.936558
H	6.587168	-2.091823	-3.472027
H	6.511538	-3.844941	-3.677834
H	7.301845	-3.148026	-2.251583
H	-1.704405	-2.512444	-3.326864
C	1.680591	0.374238	0.308556
C	2.337821	1.073692	-0.714139
C	2.342724	0.276698	1.540772
C	3.597238	1.648131	-0.520903
H	1.858514	1.182250	-1.682027
C	3.613167	0.827083	1.732980
H	1.870037	-0.258826	2.355895
C	4.249876	1.521065	0.704947
H	5.233065	1.951236	0.855427
C	4.331271	0.578201	3.028653
C	4.222354	2.467607	-1.613544
F	3.498281	0.633752	4.091974
F	5.324584	1.470202	3.247893
F	4.901394	-0.654143	3.046049
F	3.823992	3.770899	-1.558597
F	3.889307	2.021183	-2.843855
F	5.571731	2.480650	-1.536968
O	-0.514489	-0.291005	1.357414
C	-1.808261	-0.257875	1.466819
C	-2.393516	-0.986969	2.619866
C	-3.786329	-1.133045	2.733040

C	-1.556480	-1.584946	3.573819
C	-4.332944	-1.849355	3.794310
H	-4.438210	-0.696822	1.979917
C	-2.108754	-2.303001	4.633745
H	-0.481642	-1.515999	3.451285
C	-3.494267	-2.431183	4.751422
H	-5.411461	-1.954634	3.877745
H	-1.454359	-2.771552	5.363272
H	-3.921324	-2.989014	5.580534
H	-2.399514	-0.231298	0.549651
C	-2.380107	1.690627	2.011277
H	-1.724837	1.775401	2.869490
H	-3.414704	1.442263	2.213531
C	-2.147282	2.506968	0.904457
C	-0.927672	3.313737	0.742382
C	-0.383794	3.551708	-0.534136
C	-0.287054	3.863188	1.869507
C	0.774405	4.308838	-0.673900
H	-0.829151	3.077110	-1.399250
C	0.865289	4.629868	1.722529
H	-0.710181	3.714535	2.857632
C	1.401687	4.849160	0.451969
H	1.212272	4.452375	-1.656288
H	1.346284	5.053081	2.599518
H	2.314835	5.425293	0.336390
O	-3.043492	2.465441	-0.065161
Si	-3.831198	3.506828	-1.201445
C	-5.611835	2.926537	-1.112523
H	-6.031221	3.073431	-0.110529
H	-6.237853	3.482265	-1.821797
H	-5.687616	1.861279	-1.358208
C	-3.655058	5.281859	-0.600455
H	-4.236515	5.946375	-1.252280
H	-4.037417	5.405672	0.419452
H	-2.616049	5.625814	-0.618441
C	-3.092017	3.234322	-2.904073
H	-2.278189	3.936632	-3.116356
H	-2.696690	2.216925	-3.012480
H	-3.861802	3.381434	-3.671884

TS 101

B3LYP/6-31G(d) = -3573.930676
B3LYP-D3(BJ)/def2-TZVPP/IEFPCM(propanonitrile) = -3575.505431
B3LYP-D3(BJ)/def2-TZVPP/IEFPCM(propanonitrile)//B3LYP-D3(BJ)/6-31G(d) Free Energy (Quasiharmonic) = -3574.788779

Frequencies (Top 3 out of 300)

1. -274.7587 cm^{-1}
2. 9.0697 cm^{-1}
3. 11.9528 cm^{-1}

B3LYP/6-31G(d) Molecular Geometry in Cartesian Coordinates

B	0.073290	-0.075770	-0.553491
O	0.436660	0.438280	-1.904251
N	-0.676049	-1.401030	-0.936731
C	-0.788369	-1.520240	-2.400931
S	-0.670499	-2.786700	-0.023401
C	0.045000	-0.348130	-2.915081
O	0.308760	-0.129660	-4.077131
H	-2.800269	-2.290680	-2.616311
H	-0.319969	-2.449830	-2.746041
C	-2.218459	-1.452970	-3.012971
C	-2.967270	-0.162890	-2.801381
C	-4.146430	0.040920	-1.992751
C	-4.481570	1.420859	-2.073061
N	-3.570890	2.012320	-2.922681
C	-2.661400	1.063280	-3.346991
H	-1.861930	1.333640	-4.022521
C	-6.350440	1.112119	-0.626831
C	-4.966490	-0.805101	-1.226011
H	-5.788540	3.035359	-1.445511
H	-3.467890	3.007360	-3.047301
C	-6.055810	-0.265161	-0.554471
H	-7.203920	1.506519	-0.081641
H	-4.753599	-1.867211	-1.156371
C	-5.569000	1.972809	-1.387831
H	-6.691870	-0.913731	0.042369
C	-2.368159	-3.331230	0.214269
O	-0.164599	-2.415210	1.306129
O	0.002141	-3.855750	-0.788821
C	-4.958999	-4.288801	0.688769
C	-3.136719	-2.764620	1.234059
C	-2.873779	-4.371860	-0.566411
C	-4.167369	-4.836480	-0.330001
C	-4.422719	-3.248271	1.461769
H	-2.738749	-1.964190	1.847839
H	-2.250479	-4.822660	-1.331261
H	-4.561889	-5.646841	-0.938381
H	-5.020339	-2.807261	2.255589
C	-6.341629	-4.827361	0.970469
H	-6.310249	-5.604921	1.745349
H	-7.013209	-4.040181	1.328839
H	-6.787739	-5.275171	0.076629
H	-2.084609	-1.645670	-4.085061
C	-0.763310	0.994470	0.308959
C	-1.135570	2.233340	-0.225381
C	-1.149230	0.724920	1.630179
C	-1.861920	3.166560	0.521899
C	-1.903010	1.639180	2.369979
H	-0.860310	-0.219480	2.082659
C	-2.261780	2.872070	1.824709
H	-2.835860	3.585270	2.403149
C	-2.296860	1.276690	3.774299
C	-2.215261	4.483370	-0.104441
F	-2.856650	0.041450	3.830499
F	-1.229530	1.248870	4.607689
F	-3.189230	2.142900	4.303229
F	-2.625001	5.398340	0.797039
F	-1.157111	5.020970	-0.769941
F	-3.212571	4.376770	-1.027901
O	1.379890	-0.288379	0.211539
C	2.289351	-1.151109	-0.147401
C	3.173471	-1.690029	0.909759
C	3.350211	-1.007419	2.121979
C	3.799081	-2.929869	0.715089
C	4.153481	-1.555989	3.118499
H	2.834360	-0.066189	2.278539
C	4.599331	-3.478419	1.716619
H	3.624251	-3.481329	-0.205841
C	4.782011	-2.788949	2.918349
H	4.279221	-1.028749	4.060079
H	5.062171	-4.450119	1.568149
H	5.396901	-3.219888	3.703949
H	2.013371	-1.869199	-0.924851
C	3.536880	-0.208239	-1.492061
H	2.723290	0.215781	-2.065971
H	4.019801	-1.071869	-1.935491
C	4.341620	0.643511	-0.731541
C	3.909070	1.947041	-0.219301
C	2.692160	2.533411	-0.625431
C	4.720280	2.634571	0.709039
C	2.301160	3.767371	-0.111341
H	2.048220	2.033481	-1.338181
C	4.325039	3.866261	1.214459
H	5.650080	2.183592	1.034259
C	3.114219	4.435361	0.805849
H	1.354189	4.200841	-0.417341
H	4.956139	4.383371	1.931659

H	2.804919	5.397711	1.204399
O	5.526990	0.239242	-0.290131
Si	6.925000	-0.539508	-0.929241
C	7.767060	0.738522	-2.020471
H	8.704440	0.340102	-2.427951
H	7.133900	1.022462	-2.869231
H	8.006090	1.649502	-1.460731
C	6.520011	-2.092798	-1.908661
H	7.457661	-2.603178	-2.164881
H	5.913301	-2.793478	-1.325981
H	6.001121	-1.881018	-2.849841
H	-0.850160	2.475800	-1.244021
C	7.901141	-0.941608	0.617599
H	8.839911	-1.450188	0.367259
H	8.151140	-0.034928	1.179619
H	7.325141	-1.599158	1.278109

TS 102

B3LYP/6-31G(d) = -3573.926797
B3LYP-D3(BJ)/def2-TZVPP/IEFPCM(propanonitrile) = -3575.50502
B3LYP-D3(BJ)/def2-TZVPP/IEFPCM(propanonitrile)//B3LYP-D3(BJ)/6-31G(d) Free Energy (Quasiharmonic) = -3574.788701

Frequencies (Top 3 out of 300)

1. -284.9101 cm^{-1}
2. 6.4491 cm^{-1}
3. 15.2170 cm^{-1}

B3LYP/6-31G(d) Molecular Geometry in Cartesian Coordinates

B	0.309500	0.490658	-1.021337
O	-0.000342	-0.174743	-2.294449
N	1.878016	0.614188	-1.086336
C	2.355141	-0.018651	-2.328311
S	2.657686	1.967389	-0.533729
C	1.068020	-0.416237	-3.055777
O	1.021442	-0.905244	-4.163283
H	4.255002	-0.901497	-1.790106
H	2.880433	0.701365	-2.967803
C	3.275608	-1.260409	-2.122321
C	2.762019	-2.284095	-1.149273
C	1.834775	-3.369562	-1.386188
C	1.625345	-4.021914	-0.137927
N	2.410872	-3.380137	0.799420
C	3.066512	-2.328834	0.188289
H	3.721291	-1.687033	0.760696
C	0.102456	-5.562335	-1.134778
C	1.159362	-3.855007	-2.521294
H	0.613752	-5.593540	0.967626
H	2.327666	-3.487379	1.799327
C	0.303363	-4.941053	-2.384147
H	-0.578486	-6.405956	-1.059057
H	1.283356	-3.368930	-3.484158
C	0.763475	-5.114255	0.003271
H	-0.227724	-5.316241	-3.255026
C	4.275412	1.421293	0.041092
O	1.912197	2.406129	0.661622
O	2.922761	2.923614	-1.624195
C	6.806263	0.626252	0.953802
C	5.383590	1.542899	-0.797612
C	4.419758	0.933432	1.342353
C	5.680427	0.540189	1.786953
C	6.637545	1.139897	-0.338617
H	5.263743	1.969197	-1.787957
H	3.561790	0.882355	2.004454
H	5.794663	0.169668	2.803046
H	7.500556	1.237136	-0.992854
C	8.161073	0.166045	1.436485
H	8.290993	0.355600	2.507271
H	8.970811	0.671072	0.900232
H	8.288461	-0.913357	1.279884
O	-0.318089	1.885125	-1.154919
C	-0.930289	2.563558	-0.228883
H	-1.064734	2.077327	0.739111
H	3.420427	-1.701170	-3.116292
C	-0.699806	4.031139	-0.199773
C	-0.089505	4.679667	-1.282507
C	-1.070370	4.775152	0.930086
C	0.133660	6.053858	-1.234508
H	0.242538	4.094847	-2.132601
C	-0.843978	6.147899	0.976226
H	-1.520606	4.273171	1.784010
C	-0.246707	6.791667	-0.111012
H	0.619779	6.547767	-2.070962
H	-1.123533	6.713930	1.860473
H	-0.066869	7.862728	-0.076144
C	-0.237056	-0.380500	0.229344
C	0.341536	-0.309545	1.506875
C	-1.228814	-1.351742	0.031242
C	-0.039886	-1.180767	2.529475
H	1.116899	0.424761	1.693117
C	-1.644842	-2.199284	1.064443
H	-1.656616	-1.476443	-0.956930
C	-1.048241	-2.125791	2.321136
H	-1.343851	-2.803397	3.114487
C	-2.759312	-3.176145	0.818067
C	0.720128	-1.205104	3.822510
F	-3.984833	-2.575582	0.943972
F	-2.726293	-3.696714	-0.422030
F	1.395285	-0.060103	4.055061
F	-2.751623	-4.201276	1.697498
F	1.649908	-2.208713	3.829626
F	-0.080614	-1.430834	4.888220
C	-2.952461	2.653230	-0.683580
H	-3.257273	3.283027	0.143547
H	-2.812538	3.186755	-1.614840
C	-3.547708	1.386375	-0.758899
C	-3.532862	0.549833	-1.960861
C	-2.822579	0.935789	-3.115475
C	-4.231541	-0.677077	-1.968427
C	-2.810818	0.118732	-4.240564
H	-2.241506	1.849585	-3.123836
C	-4.225461	-1.481441	-3.100348
H	-4.749749	-0.999222	-1.073873
C	-3.515731	-1.085566	-4.239186
H	-2.228648	0.409422	-5.108870
H	-4.760248	-2.426547	-3.091676
H	-3.499586	-1.723520	-5.118265
O	-4.136917	0.829277	0.288752
Si	-4.689865	1.311707	1.853254
C	-5.816753	-0.099935	2.344039
H	-5.290179	-1.057419	2.291423
H	-6.181835	0.034507	3.369502
H	-6.689999	-0.160046	1.684493
C	-3.211086	1.456311	3.000822
H	-3.554314	1.597568	4.033518
H	-2.597573	0.549146	2.976551
H	-2.565077	2.305796	2.753980
C	-5.651353	2.925228	1.710324
H	-6.194511	3.101664	2.647519
H	-5.020462	3.801637	1.530256
H	-6.395547	2.874434	0.907158

TS 103

B3LYP/6-31G(d) = -3573.923207
B3LYP-D3(BJ)/def2-TZVPP/IEFPCM(propanonitrile) = -3575.504281

B3LYP-D3(BJ)/def2-TZVPP/IEFPCM(propanonitrile)//B3LYP-D3(BJ)/6-31G(d) Free Energy (Quasiharmonic) = -3574.78865

Frequencies (Top 3 out of 300)

1. -280.9899 cm^{-1}
2. 7.8050 cm^{-1}
3. 10.2704 cm^{-1}

B3LYP/6-31G(d) Molecular Geometry in Cartesian Coordinates

B	-0.068024	0.189978	-0.556255
O	-0.447738	-0.397378	-1.860949
N	0.990484	1.278551	-0.980514
C	1.193098	1.195968	-2.438891
S	0.926398	2.786230	-0.288222
C	0.160159	0.162605	-2.902778
O	-0.042794	-0.146867	-4.059131
H	3.338245	1.479836	-2.531237
H	0.962440	2.148733	-2.930332
C	2.613895	0.734851	-2.875615
C	3.007779	-0.637557	-2.393106
C	3.901274	-0.970609	-1.308002
C	3.911468	-2.388188	-1.188011
N	3.091060	-2.888292	-2.177909
C	2.539162	-1.834749	-2.882247
H	1.841602	-2.015956	-3.688076
C	5.413274	-2.281146	0.661236
C	4.694370	-0.218466	-0.424287
H	4.629085	-4.139134	-0.127424
H	2.762850	-3.840970	-2.216900
C	5.436500	-0.875524	0.549619
H	5.997223	-2.766158	1.438837
H	4.719710	0.864391	-0.494715
C	4.654902	-3.055564	-0.209218
H	6.043123	-0.298678	1.242830
C	2.594461	3.452469	-0.405568
O	0.652500	2.567680	1.144763
O	0.069556	3.707138	-1.060289
C	5.165101	4.564146	-0.530711
C	2.907415	4.357829	-1.419358
C	3.542963	3.109528	0.561504
C	4.816870	3.668170	0.492060
C	4.191044	4.901273	-1.478779
H	2.145267	4.647667	-2.134775
H	3.283047	2.423500	1.360522
H	5.553329	3.408035	1.248842
H	4.434457	5.608174	-2.268324
C	6.559835	5.138088	-0.608540
H	6.589626	6.037236	-1.231937
H	7.260568	4.412792	-1.042672
H	6.941063	5.399725	0.384710
H	2.619072	0.768078	-3.972035
C	0.524146	-0.943065	0.435306
C	1.286671	-0.639057	1.574449
C	0.347794	-2.300141	0.131253
C	1.847596	-1.644075	2.367061
H	1.464142	0.401430	1.828466
C	0.897510	-3.310109	0.926281
H	-0.207783	-2.567016	-0.760935
C	1.655115	-2.989413	2.052299
H	2.099398	-3.767302	2.661799
C	0.733370	-4.739977	0.505790
C	2.645003	-1.264268	3.584249
F	1.647897	-5.110776	-0.437826
F	-0.485785	-4.969012	-0.049502
F	0.874642	-5.605308	1.530788
F	1.843356	-1.031752	4.655579
F	3.361730	-0.135242	3.387964
F	3.510874	-2.235394	3.951947
O	-1.341229	0.845767	-0.067107
C	-1.829064	0.830898	1.151441
C	-2.702573	1.983173	1.513389
C	-3.066545	2.929583	0.545786
C	-3.131500	2.155108	2.837909
C	-3.859461	4.021644	0.899774
H	-2.678157	2.828824	-0.461128
C	-3.926691	3.242872	3.188301
H	-2.830136	1.440929	3.601377
C	-4.298643	4.177054	2.216401
H	-4.115350	4.764556	0.149061
H	-4.244573	3.371228	4.219389
H	-4.910217	5.032605	2.490605
H	-1.149753	0.509992	1.945777
C	-2.942849	-0.758426	1.398564
H	-3.305438	-0.593823	2.408559
H	-2.166691	-1.508841	1.292087
C	-3.901362	-0.692255	0.373689
C	-3.652988	-1.262854	-0.953206
C	-3.949637	-0.521273	-2.112777
C	-3.055417	-2.530407	-1.081066
C	-3.634603	-1.029759	-3.367512
H	-4.359492	0.478622	-2.018488
C	-2.774276	-3.047340	-2.341423
H	-2.824396	-3.116179	-0.197065
C	-3.049507	-2.293347	-3.484047
H	-3.819270	-0.433058	-4.255280
H	-2.317254	-4.028747	-2.426667
H	-2.794958	-2.681459	-4.465563
O	-4.955888	0.064814	0.584393
Si	-6.628873	0.192823	0.162619
C	-6.817068	1.716680	-0.914836
H	-6.271382	2.562439	-0.481958
H	-6.450614	1.560471	-1.934809
H	-7.874834	1.999622	-0.983888
C	-7.451325	0.437166	1.830033
H	-7.318912	-0.438165	2.475975
H	-7.023425	1.303686	2.346426
H	-8.528638	0.608986	1.716546
C	-7.196686	-1.387682	-0.678738
H	-6.722365	-1.537216	-1.653315
H	-6.990730	-2.270564	-0.062695
H	-8.281886	-1.344194	-0.836538

TS 104

B3LYP/6-31G(d) = -3573.930672
B3LYP-D3(BJ)/def2-TZVPP/IEFPCM(propanonitrile) = -3575.506023
B3LYP-D3(BJ)/def2-TZVPP/IEFPCM(propanonitrile)//B3LYP-D3(BJ)/6-31G(d) Free Energy (Quasiharmonic) = -3574.788589

Frequencies (Top 3 out of 300)

1. -237.6365 cm^{-1}
2. 13.3179 cm^{-1}
3. 15.5485 cm^{-1}

B3LYP/6-31G(d) Molecular Geometry in Cartesian Coordinates

B	-0.618273	0.931546	1.002630
O	-0.978632	1.476458	2.325288
N	-0.758648	2.197529	0.109429
C	-1.384824	3.294157	0.868326
S	-0.100260	2.509652	-1.349326
C	-1.382099	2.750348	2.300541
O	-1.734407	3.374285	3.275730
H	-2.781819	4.091204	-0.568978
H	-0.759241	4.196940	0.844971
C	-2.825647	3.692348	0.447806
C	-3.840517	2.584972	0.534407
C	-4.368222	1.789015	-0.549661

C	-5.279448	0.856703	0.019691
N	-5.315988	1.094345	1.379205
C	-4.436449	2.116407	1.681845
H	-4.301429	2.442115	2.704201
C	-5.733913	-0.089654	-2.120758
C	-4.156423	1.764516	-1.940203
H	-6.652142	-0.795149	-0.291073
H	-5.713571	0.461591	2.056887
C	-4.837745	0.827432	-2.708125
H	-6.244441	-0.815212	-2.747988
H	-3.450046	2.448349	-2.401039
C	-5.967830	-0.086244	-0.750358
H	-4.672862	0.793952	-3.781813
C	1.292645	3.622971	-1.050122
O	-1.024261	3.266105	-2.206311
O	0.441254	1.229288	-1.857602
C	3.496507	5.323301	-0.693028
C	2.134445	3.443697	0.049917
C	1.537462	4.643645	-1.970887
C	2.637265	5.480236	-1.789114
C	3.226381	4.293782	0.219573
H	1.930609	2.657010	0.769237
H	0.864787	4.779352	-2.810698
H	2.826353	6.273810	-2.508194
H	3.875379	4.161417	1.082469
C	4.660615	6.262003	-0.481854
H	5.084476	6.595886	-1.434809
H	4.347237	7.160294	0.066390
H	5.458791	5.788623	0.099284
H	-3.106406	4.519743	1.110912
C	-1.538647	-0.349108	0.639527
C	-2.275676	-0.961135	1.665722
C	-1.707745	-0.856446	-0.660536
C	-3.153420	-2.018245	1.407543
C	-2.564274	-1.930087	-0.916891
H	-1.187934	-0.387518	-1.488128
C	-3.298400	-2.515462	0.114202
H	-3.981997	-3.331837	-0.090182
C	-2.693135	-2.497560	-2.302720
C	-4.022149	-2.555859	2.507678
F	-1.949235	-3.629860	-2.447236
F	-3.964985	-2.844581	-2.598207
F	-2.272534	-1.639241	-3.259248
F	-5.200440	-1.869311	2.601685
F	-4.358340	-3.850152	2.311416
F	-3.434758	-2.467578	3.719300
O	0.879104	0.547735	1.201070
C	1.417980	-0.622995	1.045092
C	2.246707	-1.151652	2.148429
C	2.643325	-2.499327	2.145476
C	2.566392	-0.339458	3.249364
C	3.352824	-3.024503	3.223875
H	2.379499	-3.137627	1.306822
C	3.282568	-0.866919	4.321039
H	2.216365	0.686998	3.265377
C	3.679607	-2.208083	4.309975
H	3.640661	-4.072033	3.221951
H	3.515439	-0.237778	5.175525
H	4.227927	-2.619521	5.152997
H	0.863948	-1.356988	0.456082
C	2.853250	-0.241532	-0.483201
H	2.107137	0.255484	-1.090950
H	3.483921	0.407617	0.112418
C	3.377603	-1.459206	-0.895542
C	2.702664	-2.364789	-1.838072
C	3.182938	-3.678269	-2.013318
C	1.575352	-1.947034	-2.576342
C	2.548704	-4.550217	-2.892058
H	4.048315	-4.005037	-1.448547
C	0.945940	-2.825845	-3.452173
H	1.192002	-0.937076	-2.480860
C	1.427660	-4.127700	-3.611044
H	2.925595	-5.561863	-3.013623
H	0.068468	-2.496347	-3.996964
H	0.928134	-4.811023	-4.292017
O	4.484575	-1.958626	-0.343020
Si	6.035815	-1.312293	0.043705
C	6.787343	-0.739305	-1.580903
H	7.798564	-0.345398	-1.420994
H	6.858007	-1.560997	-2.302151
H	6.191380	0.058785	-2.038680
C	6.912547	-2.801456	0.770296
H	7.945685	-2.553185	1.041441
H	6.403321	-3.149039	1.676115
H	6.944470	-3.634452	0.059346
H	-2.184561	-0.583526	2.678597
C	5.951705	0.094046	1.289056
H	5.568190	1.023270	0.853585
H	5.331735	-0.161473	2.154776
H	6.964973	0.303067	1.656605

TS 105

B3LYP/6-31G(d) = -3573.923504
B3LYP-D3(BJ)/def2-TZVPP/IEFPCM(propanonitrile) = -3575.504588
B3LYP-D3(BJ)/def2-TZVPP/IEFPCM(propanonitrile)//B3LYP-D3(BJ)/6-31G(d) Free Energy (Quasiharmonic) = -3574.788586

Frequencies (Top 3 out of 300)

1. -276.7095 cm^{-1}
2. 8.9482 cm^{-1}
3. 12.0242 cm^{-1}

B3LYP/6-31G(d) Molecular Geometry in Cartesian Coordinates

B	0.313718	0.618711	-0.482989
O	0.341338	0.503984	-1.964618
N	0.665664	2.128746	-0.312570
C	0.980779	2.754224	-1.602394
S	0.533492	3.047468	1.050505
C	0.652791	1.642469	-2.601318
O	0.676871	1.755773	-3.806260
H	2.633403	4.036084	-1.072206
H	0.297741	3.592554	-1.807796
C	2.428478	3.277512	-1.831452
C	3.521064	2.243467	-1.842390
C	4.427907	1.892499	-0.771967
C	5.302945	0.890624	-1.275350
N	4.953142	0.664492	-2.591537
C	3.876450	1.465128	-2.919648
H	3.442423	1.430059	-3.909467
C	6.442296	0.756214	0.812797
C	4.590600	2.326197	0.556920
H	6.951484	-0.471657	-0.894778
H	5.312810	-0.083970	-3.162423
C	5.594308	1.757024	1.330555
H	7.211247	0.324239	1.448075
H	3.924969	3.074446	0.974947
C	6.308257	0.308139	-0.495757
H	5.722966	2.080827	2.360028
C	-0.864031	4.157838	0.783244
O	1.700727	3.935217	1.171215
O	0.169236	2.138929	2.144387
C	-3.043196	5.880115	0.377929
C	-0.670749	5.383466	0.139222
C	-2.130062	3.794033	1.243057
C	-3.207810	4.653994	1.036597
C	-1.759931	6.228694	-0.066055
H	0.325731	5.681270	-0.168996
H	-2.259853	2.856750	1.771322

H	-4.192249	4.366862	1.398169
H	-1.607232	7.181039	-0.568659
C	-4.208884	6.821732	0.186567
H	-4.261257	7.555655	1.001758
H	-5.162034	6.282916	0.172643
H	-4.121565	7.384118	-0.749276
H	2.396420	3.787244	-2.802285
C	1.276538	-0.462444	0.228381
C	1.158711	-0.787625	1.587245
C	2.272327	-1.125019	-0.506672
C	2.001152	-1.729898	2.186864
H	0.420309	-0.275483	2.193939
C	3.121244	-2.060718	0.090590
H	2.391998	-0.899554	-1.560426
C	2.990517	-2.371679	1.444824
H	3.652523	-3.091186	1.913425
C	4.138724	-2.812857	-0.718427
C	1.781200	-2.105037	3.624357
F	3.723999	-4.085530	-0.975341
F	5.323436	-2.925361	-0.078119
F	4.383500	-2.244582	-1.922113
F	1.447185	-1.047719	4.389563
F	2.866875	-2.686553	4.180594
F	0.758926	-3.003954	3.748554
O	-1.111503	0.297586	-0.003029
C	-2.181139	0.573510	-0.700794
C	-3.454883	0.697215	0.062587
C	-4.580658	1.276511	-0.538979
C	-3.527079	0.276959	1.398339
C	-5.763600	1.431584	0.181295
H	-4.525305	1.621407	-1.569352
C	-4.710268	0.438350	2.118540
H	-2.642160	-0.140904	1.866407
C	-5.831055	1.011774	1.512520
H	-6.628937	1.887563	-0.291844
H	-4.754186	0.124830	3.157963
H	-6.750795	1.138844	2.077022
H	-2.065555	1.301594	-1.510155
C	-2.458921	-0.852542	-2.044312
H	-3.246637	-0.403259	-2.640430
H	-1.454050	-0.786173	-2.450159
C	-2.791890	-2.018056	-1.340959
C	-4.187242	-2.424663	-1.095372
C	-4.548975	-3.002446	0.135838
C	-5.178080	-2.229250	-2.074893
C	-5.868620	-3.369494	0.380620
H	-3.797586	-3.124707	0.908403
C	-6.492985	-2.617623	-1.833685
H	-4.909063	-1.809181	-3.038875
C	-6.841518	-3.184209	-0.605372
H	-6.140485	-3.796747	1.341381
H	-7.245513	-2.480865	-2.604687
H	-7.869441	-3.480550	-0.416316
O	-1.823900	-2.681884	-0.752267
Si	-1.298125	-4.335145	-0.590096
C	-2.578363	-5.474030	-1.370376
H	-2.790655	-5.198639	-2.410043
H	-2.173953	-6.494397	-1.382315
H	-3.527110	-5.498125	-0.826588
C	0.311909	-4.426008	-1.536078
H	0.692046	-5.455435	-1.535830
H	0.177016	-4.123689	-2.581082
H	1.084749	-3.789248	-1.097841
C	-1.096279	-4.638314	1.246249
H	-0.449804	-3.894428	1.722255
H	-0.636669	-5.621894	1.406979
H	-2.057401	-4.639599	1.773095

TS 106

B3LYP/6-31G(d) = -3573.929572
B3LYP-D3(BJ)/def2-TZVPP/IEFPCM(propanonitrile) = -3575.50524
B3LYP-D3(BJ)/def2-TZVPP/IEFPCM(propanonitrile)//B3LYP-D3(BJ)/6-31G(d) Free Energy (Quasiharmonic) = -3574.788558

Frequencies (Top 3 out of 300)

1. -289.7653 cm^{-1}
2. 3.7974 cm^{-1}
3. 10.6641 cm^{-1}

B3LYP/6-31G(d) Molecular Geometry in Cartesian Coordinates

B	0.338275	0.778835	-0.364833
O	0.306194	0.551884	-1.831428
N	0.700167	2.284367	-0.302722
C	1.119419	2.770782	-1.622422
S	0.457375	3.327212	0.936446
C	0.778811	1.588762	-2.539073
O	0.906981	1.587520	-3.741809
H	2.682268	4.197949	-1.260620
H	0.490727	3.611382	-1.950396
C	2.600880	3.226989	-1.756367
C	3.624706	2.288653	-1.187623
C	4.317310	1.209620	-1.853014
C	5.177388	0.607636	-0.890546
N	5.019741	1.303285	0.292185
C	4.073375	2.292928	0.110653
H	3.781317	2.941414	0.924640
C	5.957448	-0.970518	-2.500111
C	4.297997	0.690945	-3.160532
H	6.652324	-0.924265	-0.451201
H	5.351303	0.998676	1.195368
C	5.114379	-0.390436	-3.469831
H	6.582086	-1.817387	-2.771839
H	3.635032	1.115104	-3.909652
C	6.001353	-0.479161	-1.199832
H	5.100734	-0.801714	-4.475811
C	-0.916529	4.384544	0.419642
O	1.601747	4.243121	1.087083
O	0.028917	2.529650	2.096916
C	-3.067611	6.057348	-0.261794
C	-1.970069	3.883656	-0.346796
C	-0.923941	5.715298	0.843559
C	-1.997822	6.536783	0.507331
C	-3.034092	4.721218	-0.682344
H	-1.949853	2.852672	-0.684341
H	-0.089197	6.097308	1.421186
H	-2.001272	7.571553	0.842180
H	-3.848957	4.331362	-1.288400
C	-4.206619	6.968679	-0.653645
H	-4.464090	7.659241	0.156780
H	-3.941368	7.579452	-1.526741
H	-5.105172	6.399930	-0.914034
H	2.772285	3.392434	-2.826965
C	1.365204	-0.270680	0.330562
C	1.851687	-0.115219	1.641381
C	1.857982	-1.356885	-0.407766
C	2.795521	-0.997571	2.171669
C	2.771105	-2.267690	0.137200
H	1.553806	-1.473876	-1.443943
C	3.251975	-2.090617	1.430298
H	3.976568	-2.778318	1.850005
C	3.205381	-3.445760	-0.686284
C	3.419448	-0.713781	3.508302
F	2.196221	-4.370556	-0.791580
F	3.522339	-3.106093	-1.947620
F	4.260056	-4.095851	-0.152857
F	4.538294	0.059523	3.374189
F	2.592498	-0.052570	4.339212
F	3.816513	-1.842915	4.139300

O	-1.091015	0.618439	0.151290
C	-1.599810	-0.496502	0.607226
C	-2.813991	-0.368427	1.454812
C	-3.496689	0.853543	1.555383
C	-3.243864	-1.458601	2.228091
C	-4.601697	0.971906	2.397015
H	-3.131600	1.713196	1.006252
C	-4.346504	-1.336208	3.070870
H	-2.704491	-2.400692	2.180588
C	-5.032798	-0.121676	3.152587
H	-5.113863	1.926500	2.479091
H	-4.662454	-2.183896	3.672352
H	-5.886989	-0.022749	3.816938
H	-0.892040	-1.266193	0.926821
C	-2.103191	-1.455145	-1.086238
H	-1.087248	-1.649054	-1.407051
H	-2.558523	-0.606928	-1.581799
C	-2.930769	-2.548039	-0.798150
C	-2.438993	-3.856664	-0.342922
C	-3.361244	-4.834787	0.084835
C	-1.063069	-4.164150	-0.312403
C	-2.920955	-6.075322	0.530772
H	-4.419638	-4.604509	0.069521
C	-0.628221	-5.407053	0.137819
H	-0.320525	-3.447533	-0.643460
C	-1.553650	-6.364249	0.559928
H	-3.642940	-6.817894	0.858154
H	0.434456	-5.620024	0.153721
H	-1.209988	-7.334006	0.908966
O	-4.245341	-2.420961	-0.771882
Si	-5.480742	-1.553781	-1.625456
C	-6.947450	-1.750412	-0.477377
H	-7.838031	-1.266761	-0.896326
H	-7.188072	-2.806073	-0.309963
H	-6.741627	-1.291896	0.496372
C	-5.683004	-2.466883	-3.254115
H	-6.491461	-2.020368	-3.846053
H	-4.769046	-2.418730	-3.857422
H	-5.927825	-3.522972	-3.096340
H	1.502568	0.719132	2.242606
C	-5.068570	0.257827	-1.901440
H	-4.773938	0.753350	-0.970716
H	-4.279569	0.411860	-2.645163
H	-5.968508	0.763983	-2.275151

TS 107

B3LYP/6-31G(d) = -3573.929778
B3LYP-D3(BJ)/def2-TZVPP/IEFPCM(propanonitrile) = -3575.505131
B3LYP-D3(BJ)/def2-TZVPP/IEFPCM(propanonitrile)//B3LYP-D3(BJ)/6-31G(d) Free Energy (Quasiharmonic) = -3574.788516

Frequencies (Top 3 out of 300)

1. -254.4489 cm^{-1}
2. 7.8395 cm^{-1}
3. 14.2008 cm^{-1}

B3LYP/6-31G(d) Molecular Geometry in Cartesian Coordinates

B	0.289070	-0.391810	0.173490
O	1.132861	0.077058	-0.955880
N	-0.648933	-1.434688	-0.551930
C	-0.410802	-1.373209	-2.004030
S	-1.060646	-2.899677	0.111200
C	0.795450	-0.443351	-2.133310
O	1.383430	-0.221763	-3.174960
H	-2.362543	-1.663455	-2.879190
H	-0.101405	-2.351249	-2.394610
C	-1.608891	-0.869856	-2.866250
C	-2.236769	0.415735	-2.411700
C	-1.844696	1.769414	-2.736440
C	-2.710194	2.640336	-2.015450
N	-3.600346	1.848818	-1.316250
C	-3.293398	0.522517	-1.542070
H	-3.865530	-0.259081	-1.062760
C	-1.596090	4.558604	-2.888970
C	-0.852004	2.334022	-3.558240
H	-3.250689	4.679777	-1.501500
H	-4.207155	2.165039	-0.574400
C	-0.738742	3.717252	-3.626760
H	-1.474917	5.636554	-2.953760
H	-0.167086	1.696951	-4.109950
C	-2.594791	4.032566	-2.077400
H	0.030259	4.161060	-4.253690
C	-2.719967	-3.277524	-0.474820
O	-1.154625	-2.698917	1.564740
O	-0.183548	-3.960919	-0.424750
C	-5.308578	-3.911708	-1.346910
C	-3.825045	-2.748161	0.198020
C	-2.892948	-4.140043	-1.557040
C	-4.184019	-4.446521	-1.988020
C	-5.106816	-3.067429	-0.243810
H	-3.685044	-2.112232	1.065980
H	-2.025609	-4.579555	-2.038130
H	-4.318260	-5.121000	-2.830180
H	-5.967105	-2.662547	0.283970
C	-6.703679	-4.224455	-1.832660
H	-6.726931	-5.148915	-2.418150
H	-7.403939	-4.332154	-0.997350
H	-7.086127	-3.419654	-2.474300
H	-1.229501	-0.778927	-3.891670
C	-0.495168	0.815711	0.894930
C	-0.117955	2.152931	0.708150
C	-1.656128	0.572544	1.642240
C	-0.865073	3.204602	1.249770
C	-2.427266	1.619466	2.149710
H	-1.971070	-0.451245	1.809850
C	-2.031673	2.945365	1.966850
H	-2.633121	3.759986	2.356860
C	-3.771727	1.341058	2.750760
C	-0.433290	4.633501	1.074120
F	-3.900769	0.070819	3.189070
F	-4.064275	2.162019	3.782650
F	-4.765686	1.528611	1.825430
F	-1.485048	5.449924	0.821510
F	0.157541	5.122040	2.200740
F	0.453191	4.794909	0.071430
O	1.212678	-1.054912	1.199240
C	2.097916	-1.949234	0.873780
C	2.385674	-3.010715	1.859600
C	1.924434	-2.900854	3.180270
C	3.093292	-4.156596	1.465290
C	2.188412	-3.916944	4.094810
H	1.334806	-2.035702	3.461820
C	3.351129	-5.171927	2.381390
H	3.422731	-4.258587	0.433980
C	2.905400	-5.049946	3.700580
H	1.821712	-3.833074	5.113930
H	3.889698	-6.061368	2.067000
H	3.104788	-5.843156	4.415940
H	2.172756	-2.227574	-0.177830
C	4.074918	-1.100238	0.848140
H	4.208298	-1.135509	1.920120
H	4.555266	-1.906809	0.306430
C	4.027151	0.156682	0.243720
C	3.662053	1.377993	0.974950
C	3.736706	2.626932	0.326940
C	3.245563	1.336583	2.320250
C	3.419769	3.796423	1.008220
H	4.036516	2.662892	-0.713390

C	2.927956	2.510234	2.996120
H	3.147771	0.389564	2.837500
C	3.016939	3.742694	2.345670
H	3.469471	4.750973	0.493560
H	2.598266	2.462205	4.029680
H	2.751890	4.655174	2.869810
O	4.263061	0.339031	-1.041200
Si	5.024359	-0.521420	-2.334200
C	4.774362	0.639020	-3.776720
H	3.699432	0.786722	-3.927480
H	5.241194	1.614589	-3.600850
H	5.197581	0.223479	-4.699160
C	4.205506	-2.182869	-2.650310
H	4.692015	-2.659860	-3.511230
H	4.278904	-2.882699	-1.810860
H	3.151416	-2.040106	-2.907700
H	0.753196	2.381689	0.105140
C	6.830079	-0.721524	-1.835420
H	7.301021	0.250455	-1.649990
H	6.945268	-1.328025	-0.929760
H	7.393228	-1.215765	-2.636740

TS 108

B3LYP/6-31G(d) = -3573.928862
B3LYP-D3(BJ)/def2-TZVPP/IEFPCM(propanonitrile) = -3575.505068
B3LYP-D3(BJ)/def2-TZVPP/IEFPCM(propanonitrile)//B3LYP-D3(BJ)/6-31G(d) Free Energy (Quasiharmonic) = -3574.788488

Frequencies (Top 3 out of 300)

1. -274.4535 cm^{-1}
2. 10.1607 cm^{-1}
3. 11.2212 cm^{-1}

B3LYP/6-31G(d) Molecular Geometry in Cartesian Coordinates

B	0.036036	0.104774	-0.336917
O	-0.276070	-0.509601	-1.658174
N	0.705281	1.460627	-0.790058
C	0.907369	1.449152	-2.250083
S	0.519290	2.917768	-0.025841
C	0.160733	0.196460	-2.708874
O	-0.025589	-0.119892	-3.862281
H	2.826884	2.402395	-2.531715
H	0.414007	2.309771	-2.718881
C	2.388863	1.420223	-2.734880
C	3.248066	0.347355	-2.130286
C	3.426007	-1.015273	-2.581482
C	4.299208	-1.652602	-1.655020
N	4.651562	-0.710601	-0.707990
C	4.002177	0.473934	-0.990070
H	4.136074	1.334177	-0.349798
C	4.173619	-3.702946	-2.864640
C	2.939475	-1.759648	-3.672011
H	5.329675	-3.466142	-1.052200
H	5.111073	-0.904392	0.169334
C	3.317801	-3.090224	-3.802633
H	4.442930	-4.748484	-2.988130
H	2.256732	-1.308297	-4.385652
C	4.676674	-2.993123	-1.780540
H	2.941073	-3.673887	-4.638475
C	2.117867	3.745809	-0.019543
O	0.180992	2.620224	1.373507
O	-0.376277	3.784525	-0.822991
C	4.583580	5.082599	0.025357
C	3.029799	3.480232	1.005611
C	2.411208	4.690780	-1.003518
C	3.641266	5.347330	-0.977093
C	4.252736	4.147972	1.018216
H	2.781609	2.778357	1.794529
H	1.673534	4.921344	-1.764670
H	3.867256	6.085236	-1.742987
H	4.958773	3.946739	1.820353
C	5.925303	5.775060	0.033276
H	6.276117	5.957695	1.054461
H	5.884328	6.735466	-0.490341
H	6.686507	5.161740	-0.466889
H	2.346889	1.315032	-3.826031
C	0.960329	-0.861784	0.562542
C	1.055978	-2.233469	0.288565
C	1.738136	-0.363346	1.614485
C	1.864440	-3.078767	1.052563
H	0.507968	-2.646870	-0.549699
C	2.597804	-1.190991	2.340709
H	1.677779	0.689986	1.862816
C	2.653466	-2.560214	2.078987
H	3.304575	-3.207467	2.657921
C	3.575111	-0.592096	3.306835
C	1.933644	-4.554203	0.774855
F	4.786344	-0.382727	2.702387
F	3.818764	-1.389811	4.368519
F	3.178231	0.609047	3.780133
F	1.637575	-5.281009	1.881593
F	3.169605	-4.943535	0.385861
F	1.070431	-4.943819	-0.194182
O	-1.283801	0.264949	0.407785
C	-2.225999	1.091358	0.037613
C	-3.149826	1.576449	1.087166
C	-3.788040	2.813256	0.915958
C	-3.350741	0.850211	2.270043
C	-4.628222	3.313639	1.910466
H	-3.586868	3.401036	0.023572
C	-4.194227	1.350572	3.258584
H	-2.824235	-0.087707	2.410567
C	-4.837585	2.579393	3.080679
H	-5.101190	4.283323	1.781743
H	-4.341197	0.788614	4.176790
H	-5.484958	2.972130	3.860219
H	-1.966195	1.831817	-0.721920
C	-3.394095	0.128019	-1.359262
H	-2.549963	-0.318161	-1.868737
H	-3.833184	0.988554	-1.851649
C	-4.262373	-0.695954	-0.639972
C	-3.890248	-1.999956	-0.082813
C	-4.769966	-2.652276	0.807304
C	-2.668872	-2.623028	-0.413610
C	-4.437772	-3.887276	1.349147
H	-5.703468	-2.171592	1.074014
C	-2.343337	-3.861457	0.134179
H	-1.974257	-2.148708	-1.096589
C	-3.223776	-4.494924	1.013371
H	-5.121601	-4.377187	2.036498
H	-1.396706	-4.329013	-0.116179
H	-2.963616	-5.460817	1.437517
O	-5.466445	-0.265226	-0.283997
Si	-6.797154	0.546854	-1.019306
C	-7.877035	0.955702	0.455386
H	-7.338721	1.596229	1.162779
H	-8.785078	1.484666	0.141928
H	-8.184815	0.049442	0.988679
C	-6.291236	2.100251	-1.950510
H	-7.197778	2.636676	-2.259986
H	-5.706488	2.779539	-1.321957
H	-5.717974	1.886371	-2.859082
C	-7.580204	-0.705394	-2.181682
H	-6.889994	-0.997837	-2.981656
H	-8.474705	-0.283698	-2.656560
H	-7.881028	-1.614349	-1.649141

TS 109

B3LYP/6-31G(d) = -3573.927581
B3LYP-D3(BJ)/def2-TZVPP/IEFPCM(propanonitrile) = -3575.505362
B3LYP-D3(BJ)/def2-TZVPP/IEFPCM(propanonitrile)//B3LYP-D3(BJ)/6-31G(d) Free Energy (Quasiharmonic) = -3574.788287

Frequencies (Top 3 out of 300)

1. -273.2627 cm^{-1}
2. 10.3613 cm^{-1}
3. 15.0620 cm^{-1}

B3LYP/6-31G(d) Molecular Geometry in Cartesian Coordinates

B	0.028609	0.700000	-0.500880
O	-0.533811	0.449580	-1.840100
N	1.540119	1.007362	-0.826490
C	1.766199	0.808882	-2.268890
S	2.393598	2.197513	-0.047380
C	0.370259	0.521281	-2.827380
O	0.108440	0.371460	-3.998960
H	3.767980	0.003884	-2.400600
H	2.116828	1.732633	-2.746420
C	2.756080	-0.337647	-2.640200
C	2.499732	-1.655637	-1.966270
C	1.598393	-2.713898	-2.368010
C	1.680084	-3.732638	-1.376350
N	2.606874	-3.321217	-0.438750
C	3.072372	-2.069366	-0.789510
H	3.798082	-1.563406	-0.168790
C	0.065745	-5.058640	-2.525790
C	0.732133	-2.905459	-3.460320
H	0.992086	-5.666359	-0.667240
H	2.737204	-3.734497	0.472600
C	-0.022356	-4.070530	-3.527300
H	-0.542584	-5.955890	-2.603100
H	0.634472	-2.141129	-4.225230
C	0.920415	-4.905069	-1.440050
H	-0.699005	-4.221650	-4.364310
C	4.103778	1.633905	0.025770
O	1.907898	2.228662	1.344240
O	2.412216	3.438553	-0.842880
C	6.781129	0.808988	0.171950
C	4.513149	0.793685	1.065210
C	5.019578	2.083936	-0.924750
C	6.347798	1.662737	-0.849870
C	5.843740	0.386537	1.127750
H	3.804309	0.473864	1.821750
H	4.695327	2.767925	-1.701890
H	7.059688	2.011108	-1.594190
H	6.162960	-0.265233	1.937860
C	8.225150	0.377469	0.267680
H	8.752419	0.528930	-0.679240
H	8.309441	-0.680871	0.538150
H	8.757929	0.951140	1.037440
H	2.707220	-0.439517	-3.731310
C	-0.177269	-0.572870	0.471870
C	-1.107958	-1.570971	0.155710
C	0.646311	-0.795729	1.585890
C	-1.237977	-2.728121	0.932040
H	-1.724518	-1.458332	-0.728780
C	0.561072	-1.974809	2.329460
H	1.379050	-0.044408	1.857640
C	-0.394137	-2.946510	2.018370
H	-0.466806	-3.857830	2.601710
C	1.583902	-2.277688	3.383340
C	-2.324976	-3.716222	0.620810
F	2.557083	-3.109387	2.897670
F	1.054933	-2.909209	4.454730
F	2.218571	-1.174317	3.832460
F	-3.510366	-3.351213	1.205150
F	-2.579146	-3.814002	-0.699950
F	-2.047025	-4.955432	1.078670
O	-0.657192	1.949040	0.077470
C	-1.371423	2.780159	-0.628190
C	-1.198025	4.230789	-0.375770
C	-1.754026	5.160028	-1.270300
C	-0.448595	4.686760	0.719710
C	-1.579457	6.525219	-1.066340
H	-2.313245	4.809528	-2.135460
C	-0.279237	6.055430	0.920360
H	0.031206	3.966380	1.372470
C	-0.846758	6.975569	0.036160
H	-2.005208	7.237208	-1.767810
H	0.313423	6.403881	1.761380
H	-0.707239	8.041380	0.195590
H	-1.579633	2.490359	-1.659890
C	-3.375703	2.654487	-0.059570
H	-3.800904	3.246476	-0.859600
H	-3.274993	3.179077	0.882180
C	-3.687431	1.293276	-0.007130
C	-4.137351	0.507186	-1.165790
C	-4.292021	1.086856	-2.440800
C	-4.417409	-0.864024	-1.008610
C	-4.705390	0.316915	-3.521450
H	-4.077942	2.138196	-2.599150
C	-4.826038	-1.631875	-2.094160
H	-4.293119	-1.325574	-0.037620
C	-4.971939	-1.044735	-3.352490
H	-4.808481	0.775675	-4.500160
H	-5.011777	-2.692485	-1.956580
H	-5.284818	-1.645695	-4.201640
O	-3.489371	0.591737	1.092490
Si	-3.449151	0.959157	2.794260
C	-3.166969	-0.718213	3.573560
H	-2.118469	-1.020452	3.483840
H	-3.417799	-0.689013	4.641150
H	-3.774778	-1.495844	3.100570
C	-2.065072	2.152088	3.222320
H	-1.884722	2.111918	4.304480
H	-1.134762	1.875809	2.715830
H	-2.291283	3.193028	2.967590
C	-5.149802	1.661095	3.193750
H	-5.359843	2.579255	2.633560
H	-5.217382	1.904385	4.261290
H	-5.941861	0.938764	2.966360

TS 110

B3LYP/6-31G(d) = -3573.931424
B3LYP-D3(BJ)/def2-TZVPP/IEFPCM(propanonitrile) = -3575.504844
B3LYP-D3(BJ)/def2-TZVPP/IEFPCM(propanonitrile)//B3LYP-D3(BJ)/6-31G(d) Free Energy (Quasiharmonic) = -3574.788267

Frequencies (Top 3 out of 300)

1. -274.9953 cm^{-1}
2. 8.9502 cm^{-1}
3. 10.5048 cm^{-1}

B3LYP/6-31G(d) Molecular Geometry in Cartesian Coordinates

B	0.409361	0.638350	-0.059770
O	0.175081	-0.073370	-1.347450
N	0.752351	2.062060	-0.574260
C	0.929571	2.061780	-2.031480
S	0.698541	3.492570	0.241780
C	0.442131	0.669350	-2.428190
O	0.298971	0.272790	-3.565800
H	2.549811	3.417081	-2.429010

H	0.253691	2.781780	-2.515830
C	2.370121	2.346981	-2.556940
C	3.457691	1.568601	-1.879080
C	3.974831	0.265161	-2.225100
C	4.960761	-0.064559	-1.251540
N	5.048031	1.001551	-0.377560
C	4.128551	1.962581	-0.747090
H	4.011961	2.871771	-0.173960
C	5.371601	-2.164529	-2.305420
C	3.707921	-0.654849	-3.255360
H	6.407751	-1.509669	-0.523170
H	5.511171	0.982061	0.519300
C	4.406041	-1.856289	-3.285200
H	5.895661	-3.115389	-2.350660
H	2.952231	-0.436829	-4.005240
C	5.663131	-1.273199	-1.279040
H	4.203541	-2.574829	-4.075410
C	-0.822429	4.312820	-0.269530
O	1.792791	4.357761	-0.228110
O	0.566941	3.169660	1.669000
C	-3.201729	5.554620	-1.084670
C	-2.014919	4.031890	0.400610
C	-0.802279	5.217030	-1.333360
C	-1.991360	5.825600	-1.735700
C	-3.192309	4.651120	-0.011300
H	-2.011589	3.347750	1.241070
H	0.137441	5.456410	-1.819430
H	-1.974850	6.529600	-2.564390
H	-4.119339	4.430800	0.512990
C	-4.478490	6.245400	-1.502260
H	-4.447990	6.543190	-2.555380
H	-4.646300	7.155280	-0.910700
H	-5.350730	5.599210	-1.356000
H	2.349571	2.143671	-3.634810
C	1.508901	-0.143230	0.837780
C	2.254931	0.472671	1.855960
C	1.760671	-1.502929	0.604150
C	3.210421	-0.239239	2.586400
H	2.090281	1.521741	2.077960
C	2.677891	-2.232459	1.366250
H	1.242471	-2.012950	-0.199590
C	3.420111	-1.601009	2.360190
H	4.143291	-2.154539	2.947710
C	2.898882	-3.688409	1.064840
C	4.098381	0.487921	3.554320
F	3.402762	-4.356629	2.128440
F	3.755412	-3.880699	0.038610
F	1.738672	-4.309270	0.720050
F	3.486041	1.532541	4.142170
F	5.203531	0.993921	2.922630
F	4.564811	-0.317999	4.534520
O	-0.908049	0.634770	0.727850
C	-2.044749	0.186850	0.267190
C	-3.249699	0.573260	1.041960
C	-3.138699	0.975870	2.382190
C	-4.506729	0.579100	0.420790
C	-4.272469	1.377930	3.085780
H	-2.158229	0.994730	2.847600
C	-5.637819	0.986640	1.126280
H	-4.594769	0.276010	-0.619890
C	-5.522989	1.384460	2.460420
H	-4.179399	1.696440	4.120320
H	-6.606569	0.997000	0.634770
H	-6.404299	1.703670	3.010060
H	-2.164279	0.144710	-0.817940
C	-1.865649	-1.815160	0.450400
H	-1.691229	-1.856980	1.518390
H	-0.972619	-1.858930	-0.158320
C	-2.998959	-2.450680	-0.070120
C	-4.186839	-2.799250	0.724730
C	-5.329689	-3.315630	0.081590
C	-4.212489	-2.645040	2.125440
C	-6.459308	-3.662760	0.814150
H	-5.318099	-3.436900	-0.994930
C	-5.345319	-2.991890	2.853650
H	-3.350549	-2.253110	2.651790
C	-6.471178	-3.501650	2.202170
H	-7.332078	-4.059791	0.303800
H	-5.348779	-2.866900	3.932300
H	-7.353128	-3.775241	2.774650
O	-3.120429	-2.659190	-1.368040
Si	-2.177679	-3.237600	-2.721710
C	-3.029518	-4.863330	-3.133630
H	-4.088888	-4.715070	-3.372610
H	-2.555818	-5.331460	-4.005340
H	-2.966408	-5.574720	-2.302100
C	-2.409689	-1.953410	-4.061850
H	-3.445099	-1.597590	-4.111450
H	-1.738989	-1.099680	-3.913490
H	-2.164559	-2.391140	-5.037920
C	-0.378608	-3.496060	-2.271060
H	-0.225568	-4.045500	-1.335670
H	0.155151	-2.542350	-2.215430
H	0.092222	-4.083670	-3.070220

TS 111

B3LYP/6-31G(d) = -3573.915936
B3LYP-D3(BJ)/def2-TZVPP/IEFPCM(propanonitrile) = -3575.504479
B3LYP-D3(BJ)/def2-TZVPP/IEFPCM(propanonitrile)//B3LYP-D3(BJ)/6-31G(d) Free Energy (Quasiharmonic) = -3574.788257

Frequencies (Top 3 out of 300)

1. -287.1775 cm^{-1}
2. 8.3012 cm^{-1}
3. 10.1678 cm^{-1}

B3LYP/6-31G(d) Molecular Geometry in Cartesian Coordinates

B	0.249782	0.459697	-0.138978
O	-0.150082	0.252604	-1.551259
N	0.540664	1.986571	-0.127807
C	0.422942	2.548446	-1.479799
S	0.552731	2.988411	1.175495
C	-0.063622	1.353649	-2.304892
O	-0.316083	1.386836	-3.489617
H	1.984152	4.032337	-1.525683
H	-0.362705	3.316882	-1.528607
C	1.713747	3.145313	-2.103059
C	2.877024	2.196173	-2.180418
C	3.996819	2.103976	-1.271759
C	4.829798	1.050930	-1.738620
N	4.253923	0.550138	-2.889552
C	3.074904	1.228064	-3.136755
H	2.461049	0.975187	-3.990664
C	6.354638	1.386073	0.061408
C	4.377978	2.806285	-0.113659
H	6.614336	-0.148620	-1.443485
H	4.531080	-0.310641	-3.334980
C	5.550329	2.442680	0.536758
H	7.260744	1.119752	0.599598
H	3.751891	3.602168	0.277837
C	6.005650	0.674977	-1.080382
H	5.849317	2.973840	1.436544
C	-1.131864	3.597253	1.436931
O	1.329866	4.189822	0.835047
O	0.878078	2.184121	2.358823
C	-3.734272	4.564525	1.886295
C	-1.588172	4.718697	0.740477
C	-1.955900	2.968796	2.372793

C	-3.245560	3.453390	2.590891
C	-2.885144	5.185751	0.960012
H	-0.921205	5.239825	0.061935
H	-1.568195	2.128536	2.937778
H	-3.878051	2.975096	3.336148
H	-3.234090	6.063023	0.419735
C	-5.116679	5.109369	2.158294
H	-5.544137	5.583858	1.268784
H	-5.802631	4.321507	2.487018
H	-5.090930	5.868936	2.950796
H	1.427839	3.479800	-3.107923
C	1.462879	-0.530455	0.276280
C	1.941596	-0.642470	1.590653
C	2.084431	-1.324529	-0.698553
C	2.983513	-1.516067	1.914886
C	3.132248	-2.193313	-0.380960
H	1.738895	-1.266947	-1.724488
C	3.587531	-2.299263	0.932446
H	4.394385	-2.976438	1.186414
C	3.791354	-2.996712	-1.464088
C	3.402240	-1.666633	3.351111
F	4.328087	-4.146169	-0.997831
F	2.927875	-3.327311	-2.452914
F	4.813963	-2.322509	-2.069221
F	2.578204	-2.518610	4.020376
F	4.652691	-2.169031	3.471483
F	3.367832	-0.496999	4.020609
O	-0.974498	0.237509	0.771829
C	-1.535632	-0.816639	1.294586
C	-1.303268	-2.213520	0.844774
C	-1.099528	-2.567793	-0.498969
C	-1.275437	-3.214901	1.830376
C	-0.855556	-3.897527	-0.838426
H	-1.104341	-1.800989	-1.265774
C	-1.029333	-4.542883	1.487021
H	-1.416218	-2.944742	2.874769
C	-0.819216	-4.886194	0.148859
H	-0.677610	-4.160031	-1.877378
H	-0.984928	-5.303395	2.261403
H	-0.615749	-5.918678	-0.121866
H	-1.663553	-0.740408	2.380119
C	-3.541165	-0.509329	1.135553
H	-3.494018	0.516274	1.480406
H	-3.883199	-1.228966	1.871187
C	-3.908313	-0.742462	-0.195248
C	-3.777794	0.244976	-1.265655
C	-3.862407	-0.169542	-2.612213
C	-3.587273	1.614335	-0.986457
C	-3.747697	0.752766	-3.643050
H	-3.994388	-1.222049	-2.833004
C	-3.494287	2.534701	-2.025479
H	-3.530960	1.971204	0.034750
C	-3.566300	2.107878	-3.352974
H	-3.786557	0.417939	-4.674935
H	-3.356827	3.585925	-1.792903
H	-3.471192	2.827031	-4.160967
O	-4.270016	-1.955403	-0.591138
Si	-5.352432	-3.195630	-0.074847
C	-7.080423	-2.516774	-0.369368
H	-7.837705	-3.267921	-0.113037
H	-7.276315	-1.629804	0.244562
H	-7.227600	-2.237921	-1.418550
C	-5.108443	-3.663766	1.729393
H	-5.711387	-4.554684	1.948395
H	-4.063818	-3.911371	1.945099
H	-5.432227	-2.878973	2.422051
H	1.515409	-0.017743	2.368367
C	-4.916467	-4.613435	-1.216314
H	-5.051546	-4.336382	-2.267645
H	-3.872498	-4.915166	-1.078053
H	-5.550376	-5.485480	-1.015617

TS 112

B3LYP/6-31G(d) = -3573.934077
B3LYP-D3(BJ)/def2-TZVPP/IEFPCM(propanonitrile) = -3575.505961
B3LYP-D3(BJ)/def2-TZVPP/IEFPCM(propanonitrile)//B3LYP-D3(BJ)/6-31G(d) Free Energy (Quasiharmonic) = -3574.788199

Frequencies (Top 3 out of 300)

1. -285.1686 cm^{-1}
2. 8.8071 cm^{-1}
3. 12.4699 cm^{-1}

B3LYP/6-31G(d) Molecular Geometry in Cartesian Coordinates

B	0.182240	-0.535570	-0.115420
O	-0.321350	-1.839320	0.404700
N	0.737970	-0.959910	-1.520200
C	0.531969	-2.410160	-1.726150
S	0.564210	-0.069900	-2.922460
C	-0.108351	-2.869950	-0.420890
O	-0.413961	-4.014180	-0.151680
H	2.097919	-2.969161	-3.080200
H	-0.212011	-2.599840	-2.514790
C	1.783099	-3.255461	-2.074430
C	2.932879	-3.132201	-1.116210
C	4.135629	-2.359041	-1.308710
C	4.934189	-2.519471	-0.143210
N	4.254949	-3.372911	0.702330
C	3.051059	-3.726081	0.118290
H	2.367689	-4.393321	0.626270
C	6.625890	-1.103272	-1.047030
C	4.621330	-1.560861	-2.361010
H	6.762949	-2.012582	0.911140
H	4.502819	-3.550901	1.663400
C	5.858810	-0.943152	-2.220550
H	7.587330	-0.603142	-0.964760
H	4.030730	-1.422841	-3.262910
C	6.176150	-1.892042	0.005000
H	6.245340	-0.326432	-3.027890
C	1.285111	1.515920	-2.508570
O	1.385650	-0.720600	-3.957470
O	-0.862790	0.158790	-3.241920
C	2.417591	4.026069	-2.005690
C	0.500501	2.661550	-2.634370
C	2.634261	1.602939	-2.153350
C	3.185511	2.855009	-1.898840
C	1.073631	3.908420	-2.381340
H	-0.538849	2.568150	-2.927960
H	3.239750	0.707169	-2.063680
H	4.229771	2.924359	-1.605500
H	0.465632	4.804690	-2.479350
C	3.026302	5.371909	-1.695000
H	3.186832	5.477119	-0.615990
H	4.001032	5.491199	-2.181730
H	2.379662	6.191589	-2.023510
H	1.433549	-4.294090	-2.124240
C	1.239080	0.117660	0.921230
C	1.429701	1.501170	1.048880
C	2.003520	-0.709071	1.757630
C	2.346871	2.033069	1.960430
C	2.919820	-0.185511	2.672950
H	1.874790	-1.783181	1.701770
C	3.098180	1.193279	2.782060
H	3.796671	1.605809	3.501980
C	3.755930	-1.099511	3.523310
C	2.578901	3.515609	2.030860
F	3.945070	-0.606391	4.767700
F	3.204839	-2.331191	3.657850

F	4.995700	-1.297122	2.999030
F	2.812871	3.934859	3.296190
F	3.662081	3.892229	1.298930
F	1.530431	4.228040	1.558690
O	-0.975470	0.452280	-0.206240
C	-2.189060	0.133251	-0.579990
C	-3.046260	1.244561	-1.055260
C	-4.162450	0.968271	-1.857430
C	-2.733759	2.577421	-0.744100
C	-4.951339	2.008092	-2.348320
H	-4.396810	-0.060209	-2.118330
C	-3.528379	3.613921	-1.229900
H	-1.857239	2.788381	-0.140760
C	-4.638559	3.332431	-2.033000
H	-5.801259	1.784882	-2.987040
H	-3.275378	4.643821	-0.993150
H	-5.249179	4.143212	-2.420970
H	-2.321540	-0.820929	-1.091850
C	-3.037650	-0.466409	1.164280
H	-2.249060	-1.178119	1.370550
H	-2.966260	0.472001	1.701440
C	-4.318000	-0.958539	0.887670
C	-4.569501	-2.297689	0.338720
C	-5.842901	-2.617328	-0.177860
C	-3.556141	-3.277659	0.303390
C	-6.087701	-3.873418	-0.720620
H	-6.625560	-1.867978	-0.158650
C	-3.806641	-4.531049	-0.246150
H	-2.568291	-3.086289	0.703580
C	-5.070441	-4.832778	-0.758520
H	-7.070831	-4.105558	-1.120120
H	-3.002301	-5.258909	-0.274580
H	-5.264072	-5.812088	-1.187560
O	-5.384290	-0.174168	0.946990
Si	-6.027110	1.009822	2.032060
C	-7.419969	1.753992	1.024820
H	-7.030929	2.236772	0.121580
H	-8.143960	0.991592	0.716620
H	-7.959009	2.511412	1.606510
C	-4.770099	2.312201	2.533520
H	-5.290549	3.107352	3.083480
H	-3.990319	1.920561	3.195580
H	-4.289019	2.772671	1.664410
H	0.854101	2.181260	0.431010
C	-6.634540	0.040482	3.522950
H	-7.370350	-0.718828	3.236180
H	-5.809340	-0.467498	4.035610
H	-7.110200	0.710732	4.249520

TS 113

B3LYP/6-31G(d) = -3573.927279
B3LYP-D3(BJ)/def2-TZVPP/IEFPCM(propanonitrile) = -3575.505399
B3LYP-D3(BJ)/def2-TZVPP/IEFPCM(propanonitrile)//B3LYP-D3(BJ)/6-31G(d) Free Energy (Quasiharmonic) = -3574.788185

Frequencies (Top 3 out of 300)

1. -301.5413 cm^{-1}
2. 10.0251 cm^{-1}
3. 12.3218 cm^{-1}

B3LYP/6-31G(d) Molecular Geometry in Cartesian Coordinates

B	-0.115810	0.689447	1.158990
O	0.118693	0.222529	2.535556
N	-1.657287	1.009391	1.182367
C	-2.190225	0.707236	2.523392
S	-2.280282	2.310482	0.364221
C	-0.950014	0.311125	3.327942
O	-0.942858	0.049023	4.511549
H	-4.103506	-0.167234	1.997875
H	-2.629559	1.599685	2.984382
C	-3.227832	-0.452517	2.589166
C	-2.717547	-1.798463	2.139544
C	-3.065340	-2.512725	0.933128
C	-2.303874	-3.714910	0.916461
N	-1.559776	-3.741409	2.077567
C	-1.801118	-2.586179	2.797265
H	-1.296855	-2.409759	3.736882
C	-3.254720	-4.358332	-1.173505
C	-3.952516	-2.267441	-0.129038
H	-1.773063	-5.541625	-0.130179
H	-0.802396	-4.381269	2.261667
C	-4.039214	-3.186655	-1.167351
H	-3.338464	-5.055014	-2.003436
H	-4.560731	-1.368629	-0.142602
C	-2.379735	-4.639379	-0.130527
H	-4.721043	-2.999999	-1.993185
C	-3.899718	1.817314	-0.247889
O	-2.519058	3.445351	1.273192
O	-1.426318	2.493292	-0.825684
C	-6.429490	1.152503	-1.257492
C	-5.045554	2.196184	0.452700
C	-4.000222	1.124260	-1.456555
C	-5.261739	0.802225	-1.951985
C	-6.300086	1.854953	-0.052060
H	-4.950678	2.773252	1.366374
H	-3.107052	0.851594	-2.008043
H	-5.340717	0.271090	-2.897609
H	-7.192705	2.152936	0.492701
C	-7.789158	0.768410	-1.791121
H	-8.048958	-0.259098	-1.503960
H	-7.818154	0.815699	-2.885010
H	-8.573398	1.424906	-1.400872
O	0.698710	1.986058	1.082903
C	1.339886	2.471459	0.056072
H	1.295024	1.906993	-0.875846
H	-3.554140	-0.499332	3.635556
C	1.341313	3.952672	-0.085572
C	1.692882	4.537178	-1.310822
C	0.967119	4.772471	0.987902
C	1.680721	5.921568	-1.459703
H	1.953663	3.903674	-2.155552
C	0.958534	6.157296	0.836938
H	0.652288	4.314439	1.918922
C	1.319647	6.735299	-0.382543
H	1.941778	6.365546	-2.416352
H	0.655374	6.786700	1.668829
H	1.307123	7.815689	-0.497999
C	0.292940	-0.435860	0.070071
C	0.893306	-1.631552	0.492292
C	-0.011319	-0.324626	-1.296396
C	1.162880	-2.672750	-0.399941
H	1.125212	-1.757995	1.543258
C	0.265779	-1.360000	-2.193754
H	-0.503054	0.572757	-1.658478
C	0.848067	-2.547266	-1.752645
H	1.035087	-3.361405	-2.443322
C	-0.003443	-1.164890	-3.656542
C	1.850139	-3.916436	0.081155
F	1.073982	-0.595486	-4.281651
F	-0.241569	-2.325554	-4.301691
F	1.557454	-4.207523	1.374447
F	-1.050662	-0.344961	-3.887142
F	3.209409	-3.800882	0.022269
F	1.530068	-5.003070	-0.653310
C	3.341633	2.217501	0.308368
H	3.465783	2.868552	1.164399
H	3.658109	2.657088	-0.630001
C	3.633667	0.856960	0.493432

O	3.872408	0.055003	-0.531552
Si	4.597520	0.169156	-2.099862
C	4.376279	-1.559758	-2.786421
H	3.396411	-1.652994	-3.266030
H	5.141558	-1.782973	-3.539521
H	4.437933	-2.318442	-1.999252
C	3.735800	1.424557	-3.202267
H	3.852902	2.462098	-2.873166
H	4.171326	1.354271	-4.208039
H	2.668888	1.198954	-3.303904
C	6.393742	0.630877	-1.787356
H	6.897652	-0.123566	-1.172927
H	6.940466	0.709189	-2.735001
H	6.482381	1.595678	-1.274584
C	3.620541	0.199115	1.801439
C	4.057670	-1.137671	1.916536
C	3.180902	0.872917	2.958792
C	4.070894	-1.769583	3.153160
H	4.362517	-1.676337	1.028474
C	3.183319	0.229473	4.191483
H	2.795135	1.882604	2.893603
C	3.635126	-1.087121	4.294096
H	4.407825	-2.799413	3.227021
H	2.811718	0.749242	5.068630
H	3.635755	-1.585970	5.259249

TS 114

B3LYP/6-31G(d) = -3573.931443
B3LYP-D3(BJ)/def2-TZVPP/IEFPCM(propanonitrile) = -3575.504702
B3LYP-D3(BJ)/def2-TZVPP/IEFPCM(propanonitrile)//B3LYP-D3(BJ)/6-31G(d) Free Energy (Quasiharmonic) = -3574.788153

Frequencies (Top 3 out of 300)

1. -251.6214 cm^{-1}
2. 9.9410 cm^{-1}
3. 11.3717 cm^{-1}

B3LYP/6-31G(d) Molecular Geometry in Cartesian Coordinates

B	-0.714357	0.830388	0.778237
O	-1.278957	1.245822	2.072567
N	-0.878496	2.126867	-0.057889
C	-1.615537	3.148378	0.705902
S	-0.167698	2.518252	-1.473274
C	-1.750755	2.496830	2.086257
O	-2.228111	3.039321	3.055995
H	-2.805515	4.188996	-0.754574
H	-1.008428	4.055674	0.833651
C	-2.994994	3.582883	0.135269
C	-3.936482	2.464693	-0.206109
C	-4.899631	1.811338	0.652060
C	-5.543025	0.810642	-0.129314
N	-5.008346	0.874269	-1.401204
C	-4.033369	1.852324	-1.431730
H	-3.476636	2.055937	-2.335911
C	-6.899077	0.161967	1.721615
C	-5.288657	1.974152	1.994339
H	-7.016530	-0.781628	-0.222202
H	-5.133165	0.174941	-2.117312
C	-6.280441	1.151117	2.513281
H	-7.668378	-0.469490	2.158394
H	-4.800609	2.713462	2.622396
C	-6.539794	-0.021018	0.391282
H	-6.581416	1.265317	3.551332
C	1.110796	3.731692	-1.080799
O	-1.100234	3.207995	-2.378085
O	0.493550	1.290121	-1.968094
C	3.149477	5.590643	-0.572594
C	1.914119	3.584944	0.052426
C	1.312087	4.796793	-1.960608
C	2.330512	5.712912	-1.703341
C	2.924089	4.514039	0.297126
H	1.741369	2.761484	0.738072
H	0.669524	4.903571	-2.827710
H	2.486478	6.541492	-2.390105
H	3.543071	4.406671	1.185134
C	4.222295	6.612561	-0.280136
H	5.033574	6.184246	0.317578
H	3.813847	7.460637	0.285212
H	4.653992	7.015877	-1.202162
O	0.793021	0.596092	1.121051
C	1.450929	-0.521489	1.030062
H	0.977213	-1.335006	0.477161
H	-3.434198	4.243975	0.892290
C	2.290136	-0.916510	2.183662
C	2.797143	-2.223826	2.266898
C	2.514148	-0.020792	3.241871
C	3.522067	-2.626074	3.387311
H	2.605613	-2.929443	1.463385
C	3.245850	-0.424685	4.356111
H	2.080613	0.972092	3.192293
C	3.753766	-1.725647	4.430493
H	3.894420	-3.644581	3.452110
H	3.404543	0.268945	5.177011
H	4.313802	-2.041439	5.306390
C	-1.430960	-0.528126	0.269291
C	-1.782243	-0.786905	-1.063837
C	-1.785861	-1.498420	1.221783
C	-2.456151	-1.957538	-1.425500
H	-1.522949	-0.071080	-1.834212
C	-2.441951	-2.679550	0.862552
H	-1.555482	-1.324319	2.268920
C	-2.786636	-2.915555	-0.466566
H	-3.293522	-3.830650	-0.749556
C	-2.819928	-3.674870	1.926410
C	-2.881499	-2.167507	-2.849605
F	-1.857725	-3.781303	2.873195
F	-3.958556	-3.329464	2.564362
F	-2.927554	-3.475322	-3.185857
F	-3.014981	-4.913119	1.413296
F	-2.053429	-1.556276	-3.731452
F	-4.128067	-1.669101	-3.096384
C	2.862852	-0.108066	-0.472055
H	2.107109	0.335266	-1.109476
H	3.464919	0.593568	0.093454
C	3.450027	-1.312694	-0.843220
O	4.586612	-1.730360	-0.287444
Si	6.094376	-0.980590	0.094891
C	6.807420	-0.379414	-1.536659
H	7.790789	0.081182	-1.381338
H	6.161320	0.372956	-2.003728
H	6.932133	-1.203133	-2.248208
C	5.921052	0.429014	1.326531
H	5.313172	0.145971	2.192141
H	6.919096	0.699020	1.696098
H	5.487001	1.330974	0.881454
C	7.061072	-2.405070	0.836505
H	7.143550	-3.242901	0.135398
H	8.077248	-2.090563	1.102963
H	6.575505	-2.772586	1.747443
C	2.813119	-2.289903	-1.739896
C	3.387057	-3.567203	-1.903179
C	1.630634	-1.979130	-2.444085
C	2.794865	-4.506164	-2.740656
H	4.293588	-3.812603	-1.362446
C	1.044168	-2.924798	-3.280246
H	1.169676	-1.001355	-2.354642
C	1.621871	-4.187952	-3.430408
H	3.245892	-5.487861	-2.854485

H	0.129453	-2.673553	-3.806006
H	1.158280	-4.922474	-4.083132

TS 115

B3LYP/6-31G(d) = -3573.931693
B3LYP-D3(BJ)/def2-TZVPP/IEFPCM(propanonitrile) = -3575.504713
B3LYP-D3(BJ)/def2-TZVPP/IEFPCM(propanonitrile)//B3LYP-D3(BJ)/6-31G(d) Free Energy (Quasiharmonic) = -3574.788124

Frequencies (Top 3 out of 300)

1. -251.2341 cm^{-1}
2. 10.9798 cm^{-1}
3. 12.2212 cm^{-1}

B3LYP/6-31G(d) Molecular Geometry in Cartesian Coordinates

B	-0.719297	0.838096	0.782343
O	-1.280236	1.251030	2.079063
N	-0.883557	2.137041	-0.049918
C	-1.621767	3.155523	0.716643
S	-0.175594	2.532350	-1.465498
C	-1.752268	2.501859	2.096384
O	-2.227201	3.042414	3.068413
H	-2.819202	4.194712	-0.738958
H	-1.017345	4.064634	0.843923
C	-3.004298	3.585717	0.149830
C	-3.941201	2.464048	-0.192809
C	-4.896063	1.799710	0.666237
C	-5.536288	0.798848	-0.117649
N	-5.008050	0.872979	-1.391573
C	-4.039673	1.857523	-1.421199
H	-3.487686	2.068986	-2.326490
C	-6.878862	0.130249	1.736089
C	-5.279915	1.952286	2.011229
H	-6.999199	-0.803014	-0.212646
H	-5.131901	0.176839	-2.110943
C	-6.263356	1.119329	2.530315
H	-7.642080	-0.508634	2.172821
H	-4.794306	2.691624	2.641124
C	-6.524666	-0.042767	0.402998
H	-6.560256	1.225839	3.570355
C	1.103834	3.744715	-1.072614
O	-1.110212	3.224037	-2.366687
O	0.485881	1.306031	-1.964609
C	3.139170	5.606306	-0.560184
C	1.910862	3.593738	0.057019
C	1.296308	4.818366	-1.944421
C	2.311024	5.737144	-1.683706
C	2.917872	4.525672	0.305050
H	1.739013	2.769156	0.741552
H	0.645085	4.932897	-2.804024
H	2.454983	6.576452	-2.360048
H	3.535348	4.418696	1.194058
C	4.246906	6.598739	-0.297502
H	4.541669	6.601879	0.756683
H	5.140899	6.358241	-0.888270
H	3.945548	7.616106	-0.568881
H	-3.444875	4.243076	0.909317
C	-1.438439	-0.518597	0.271684
C	-1.779293	-0.780654	-1.063345
C	-1.810772	-1.481048	1.225956
C	-2.462288	-1.946238	-1.425357
C	-2.474397	-2.657219	0.866155
H	-1.602621	-1.296800	2.275735
C	-2.809732	-2.896450	-0.465133
H	-3.335283	-3.801801	-0.746246
C	-2.775176	-3.698746	1.909302
C	-2.886937	-2.155133	-2.849774
F	-1.722627	-4.540020	2.084841
F	-3.039002	-3.152886	3.113592
F	-3.832499	-4.469679	1.568268
F	-4.134306	-1.658554	-3.096778
F	-2.059321	-1.542272	-3.730677
F	-2.931365	-3.462848	-3.186967
O	0.788416	0.600710	1.121120
C	1.442650	-0.518689	1.026454
C	2.281138	-0.920431	2.178074
C	2.509621	-0.028849	3.238866
C	2.782435	-2.230234	2.256995
C	3.240191	-0.439310	4.351429
H	2.080364	0.966056	3.192526
C	3.506196	-2.639020	3.375819
H	2.587070	-2.932674	1.451618
C	3.742435	-1.742743	4.421546
H	3.402380	0.251097	5.174363
H	3.873972	-3.659381	3.437361
H	4.301542	-2.063636	5.296184
H	0.966410	-1.328830	0.470738
C	2.855558	-0.103109	-0.475425
H	2.100277	0.342735	-1.111613
H	3.457813	0.596280	0.092648
C	3.441455	-1.307514	-0.849008
C	2.804619	-2.281466	-1.749261
C	3.376549	-3.559418	-1.914314
C	1.624154	-1.966947	-2.455113
C	2.784328	-4.495401	-2.755093
H	4.281431	-3.807777	-1.372183
C	1.037562	-2.909656	-3.294485
H	1.164893	-0.988510	-2.364248
C	1.613267	-4.173519	-3.446372
H	3.233713	-5.477704	-2.870124
H	0.124142	-2.655758	-3.821201
H	1.149536	-4.905768	-4.101543
O	4.576567	-1.728314	-0.292490
Si	6.085706	-0.983126	0.092638
C	7.047200	-2.410496	0.835409
H	8.063738	-2.098890	1.103889
H	6.558883	-2.777060	1.745255
H	7.128664	-3.248259	0.134102
C	5.914422	0.426745	1.324249
H	6.912677	0.694386	1.694961
H	5.482943	1.329818	0.878919
H	5.304950	0.144946	2.189150
H	-1.509489	-0.069279	-1.834296
C	6.803233	-0.383490	-1.537574
H	6.926755	-1.207367	-2.249147
H	6.160007	0.370805	-2.005516
H	7.787657	0.074254	-1.380509

TS 116

B3LYP/6-31G(d) = -3573.931818
B3LYP-D3(BJ)/def2-TZVPP/IEFPCM(propanonitrile) = -3575.504074
B3LYP-D3(BJ)/def2-TZVPP/IEFPCM(propanonitrile)//B3LYP-D3(BJ)/6-31G(d) Free Energy (Quasiharmonic) = -3574.788056

Frequencies (Top 3 out of 300)

1. -274.2244 cm^{-1}
2. 3.0592 cm^{-1}
3. 11.4780 cm^{-1}

B3LYP/6-31G(d) Molecular Geometry in Cartesian Coordinates

B	0.445828	0.594312	-0.104170
O	0.188337	-0.061546	-1.420414
N	0.744976	2.043217	-0.549234
C	0.876963	2.123987	-2.009378

S	0.653690	3.425513	0.335897
C	0.388202	0.749623	-2.465634
O	0.185921	0.423333	-3.616735
H	2.467434	3.522347	-2.368141
H	0.179644	2.862408	-2.430917
C	2.297839	2.458942	-2.555586
C	3.412752	1.657970	-1.952896
C	3.918498	0.370639	-2.369274
C	4.938948	0.004997	-1.445551
N	5.058094	1.036405	-0.534433
C	4.123517	2.008180	-0.831089
H	4.024582	2.891950	-0.216160
C	5.308720	-2.053955	-2.591400
C	3.614357	-0.509350	-3.423819
H	6.409369	-1.466276	-0.824354
H	5.550667	0.980062	0.345013
C	4.309447	-1.708670	-3.523909
H	5.829134	-3.002668	-2.690934
H	2.831979	-0.264083	-4.137014
C	5.638524	-1.201638	-1.543688
H	4.077369	-2.397069	-4.332563
C	-0.866834	4.249421	-0.185395
O	1.744874	4.334600	-0.051151
O	0.491207	3.028392	1.741833
C	-3.225138	5.549083	-0.976070
C	-0.793202	5.416412	-0.944527
C	-2.107368	3.723711	0.188082
C	-3.271879	4.373772	-0.208493
C	-1.971494	6.054557	-1.337294
H	0.177880	5.819788	-1.209832
H	-2.149466	2.816241	0.780131
H	-4.238051	3.968786	0.085731
H	-1.911580	6.963544	-1.930944
C	-4.497039	6.259016	-1.375299
H	-4.900987	6.845313	-0.539391
H	-5.275362	5.549208	-1.677292
H	-4.326492	6.949170	-2.207329
H	2.251252	2.321590	-3.643147
C	1.576425	-0.217581	0.722707
C	2.360324	0.370304	1.728319
C	1.805590	-1.573834	0.448785
C	3.338101	-0.363008	2.405650
H	2.205413	1.412653	1.986410
C	2.750193	-2.323767	1.156290
H	1.247876	-2.062086	-0.342460
C	3.533436	-1.718917	2.135233
H	4.276452	-2.289689	2.679991
C	2.960255	-3.770876	0.807551
C	4.258939	0.333852	3.365214
F	3.489481	-4.470065	1.838632
F	3.793621	-3.931646	-0.243689
F	1.792722	-4.379549	0.471816
F	5.348125	0.852372	2.714805
F	4.750427	-0.499864	4.308508
F	3.669998	1.365361	3.998147
O	-0.864674	0.566497	0.692054
C	-1.554534	-0.529043	0.868301
C	-2.315613	-0.627043	2.136617
C	-2.405907	0.479838	2.996259
C	-2.873739	-1.853715	2.529256
C	-3.065789	0.359705	4.217999
H	-1.911280	1.406046	2.722566
C	-3.524241	-1.970880	3.754805
H	-2.787466	-2.719461	1.876975
C	-3.628216	-0.861521	4.598151
H	-3.121817	1.215924	4.884288
H	-3.945033	-2.926450	4.054246
H	-4.133031	-0.953424	5.555981
H	-1.109092	-1.463369	0.516934
C	-2.889343	-0.422221	-0.663948
H	-3.309056	0.540817	-0.400915
H	-2.105720	-0.383350	-1.411301
C	-3.751297	-1.524873	-0.706547
C	-5.045371	-1.582798	-0.008447
C	-5.840561	-2.742108	-0.110656
C	-5.525282	-0.500735	0.756416
C	-7.074735	-2.813154	0.525818
H	-5.477397	-3.579739	-0.694017
C	-6.758652	-0.577981	1.394384
H	-4.931677	0.398489	0.869266
C	-7.538068	-1.731665	1.279937
H	-7.677154	-3.712363	0.434944
H	-7.111578	0.262826	1.983978
H	-8.502787	-1.787580	1.776517
O	-3.408695	-2.647465	-1.312088
Si	-2.458983	-3.108944	-2.710227
C	-0.686921	-3.390602	-2.184290
H	-0.598126	-4.005126	-1.281340
H	-0.149225	-3.911360	-2.987101
H	-0.175788	-2.436946	-2.020220
C	-3.316036	-4.706764	-3.198864
H	-2.812136	-5.163612	-4.059255
H	-3.298234	-5.435706	-2.380609
H	-4.361710	-4.535632	-3.479079
C	-2.622808	-1.779019	-4.023553
H	-3.625432	-1.336285	-4.045583
H	-2.447912	-2.234221	-5.007010
H	-1.880287	-0.982307	-3.903690

TS 117

B3LYP/6-31G(d) = -3573.935563
B3LYP-D3(BJ)/def2-TZVPP/IEFPCM(propanonitrile) = -3575.505815
B3LYP-D3(BJ)/def2-TZVPP/IEFPCM(propanonitrile)//B3LYP-D3(BJ)/6-31G(d) Free Energy (Quasiharmonic) = -3574.788013

Frequencies (Top 3 out of 300)

1. -279.7081 cm^{-1}
2. 11.2108 cm^{-1}
3. 13.7161 cm^{-1}

B3LYP/6-31G(d) Molecular Geometry in Cartesian Coordinates

B	-0.136780	-0.393751	-0.328910
O	0.248960	-1.613291	-1.081590
N	-0.366950	-0.975761	1.109530
C	0.031739	-2.397891	1.144780
S	-0.117740	-0.132081	2.524470
C	0.401759	-2.689621	-0.309390
O	0.814719	-3.756691	-0.721930
H	-1.106511	-3.248730	2.752310
H	0.949069	-2.546421	1.733740
C	-1.005681	-3.419010	1.678760
C	-2.357201	-3.383040	1.023860
C	-3.579131	-2.880180	1.604160
C	-4.606011	-3.037790	0.633270
N	-4.034651	-3.634780	-0.472110
C	-2.684001	-3.826320	-0.236140
H	-2.056271	-4.289240	-0.985890
C	-6.218791	-2.090300	2.111270
C	-3.906941	-2.325070	2.855620
H	-6.692901	-2.753000	0.107510
H	-4.474411	-3.712200	-1.376570
C	-5.219821	-1.937180	3.096060
H	-7.235481	-1.773050	2.327670
H	-3.140671	-2.187420	3.613730
C	-5.926991	-2.641750	0.869720
H	-5.484150	-1.507410	4.058700
C	-1.214220	1.272260	2.353210
O	1.262360	0.405389	2.626540

O	-0.569150	-0.991830	3.631550
C	-2.926670	3.475680	2.141690
C	-2.589370	1.063730	2.223780
C	-0.686330	2.561900	2.410500
C	-1.546180	3.654120	2.301510
C	-3.431650	2.166480	2.118860
H	-2.994580	0.058270	2.192170
H	0.381620	2.696749	2.540590
H	-1.137620	4.661100	2.334850
H	-4.501080	2.005330	2.008700
C	-3.845220	4.658670	1.954090
H	-3.999770	4.854380	0.885780
H	-3.427309	5.567650	2.398780
H	-4.828580	4.478730	2.401210
H	-0.538921	-4.403410	1.547300
C	-1.389980	0.350730	-1.027750
C	-2.444130	-0.406270	-1.558920
C	-1.477080	1.744340	-1.152510
C	-3.539490	0.198020	-2.181870
H	-2.406790	-1.487810	-1.492300
C	-2.579420	2.356820	-1.754390
H	-0.679300	2.366290	-0.763250
C	-3.618440	1.587200	-2.277720
H	-4.470770	2.060190	-2.752880
C	-2.640850	3.852320	-1.890400
C	-4.671620	-0.642390	-2.703250
F	-3.899940	4.323020	-1.690000
F	-2.269530	4.271130	-3.123380
F	-1.839020	4.484490	-1.003500
F	-5.624880	-0.860730	-1.764430
F	-4.253021	-1.869860	-3.111770
F	-5.291930	-0.065290	-3.756360
O	1.026640	0.588139	-0.331020
C	2.178520	0.405179	-0.918460
C	3.023120	1.612079	-1.073350
C	2.849690	2.723229	-0.235360
C	3.979710	1.662379	-2.098100
C	3.621100	3.867659	-0.426200
H	2.104350	2.676979	0.551280
C	4.748630	2.808879	-2.286270
H	4.108060	0.807799	-2.757990
C	4.570350	3.913829	-1.450140
H	3.479250	4.727639	0.222390
H	5.478530	2.844819	-3.090170
H	5.166380	4.809899	-1.599720
H	2.198600	-0.300411	-1.750690
C	3.150730	-0.905561	0.314280
H	2.680640	-0.545241	1.221990
H	2.713739	-1.803991	-0.104140
C	4.537130	-0.789321	0.188610
C	5.347580	0.143539	0.979890
C	6.711880	0.320209	0.673600
C	4.792100	0.855869	2.063910
C	7.498070	1.184418	1.426250
H	7.140270	-0.225402	-0.158840
C	5.588380	1.715469	2.814770
H	3.748630	0.735269	2.334940
C	6.938660	1.883678	2.499820
H	8.547910	1.313478	1.178910
H	5.152080	2.251999	3.652070
H	7.555320	2.554758	3.091610
O	5.208360	-1.464871	-0.733490
Si	4.916799	-2.948161	-1.604230
C	4.412949	-4.277851	-0.378950
H	3.349789	-4.226071	-0.122310
H	4.586379	-5.263091	-0.830420
H	5.004719	-4.230901	0.542320
C	3.621479	-2.677691	-2.936660
H	3.668529	-3.512141	-3.648640
H	2.601899	-2.662081	-2.536070
H	3.798569	-1.758611	-3.507450
C	6.613049	-3.260491	-2.343450
H	6.926609	-2.431892	-2.988610
H	6.607369	-4.172181	-2.952880
H	7.371889	-3.385862	-1.562990

TS 118

B3LYP/6-31G(d) = -3573.930351
B3LYP-D3(BJ)/def2-TZVPP/IEFPCM(propanonitrile) = -3575.504279
B3LYP-D3(BJ)/def2-TZVPP/IEFPCM(propanonitrile)//B3LYP-D3(BJ)/6-31G(d) Free Energy (Quasiharmonic) = -3574.787852

Frequencies (Top 3 out of 300)

1. -274.3941 cm^{-1}
2. 9.2674 cm^{-1}
3. 10.5908 cm^{-1}

B3LYP/6-31G(d) Molecular Geometry in Cartesian Coordinates

B	0.094279	-0.048150	-0.530640
O	0.449859	0.426470	-1.897520
N	-0.690430	-1.363530	-0.871330
C	-0.812540	-1.525610	-2.330420
S	-0.703760	-2.721810	0.082530
C	0.015499	-0.367780	-2.885210
O	0.244129	-0.166950	-4.057460
H	-2.799280	-2.350850	-2.533090
H	-0.338160	-2.461390	-2.651660
C	-2.244910	-1.497140	-2.934740
C	-3.039441	-0.234080	-2.722290
C	-4.309331	-0.115150	-2.043690
C	-4.688721	1.255200	-2.090860
N	-3.712101	1.924680	-2.797410
C	-2.723131	1.030700	-3.161860
H	-1.872401	1.366580	-3.737700
C	-6.700451	0.799649	-0.895170
C	-5.175930	-1.030370	-1.421370
H	-6.138211	2.781219	-1.557180
H	-3.608801	2.927600	-2.825040
C	-6.357080	-0.567331	-0.855340
H	-7.629421	1.132289	-0.439640
H	-4.927490	-2.086630	-1.378610
C	-5.872701	1.727889	-1.515230
H	-7.031230	-1.269961	-0.372200
C	-2.409250	-3.235230	0.331030
O	-0.195710	-2.316150	1.401300
O	-0.041380	-3.821630	-0.647900
C	-5.002680	-4.156320	0.856340
C	-3.198170	-2.573130	1.276040
C	-2.896640	-4.350230	-0.350820
C	-4.192130	-4.798200	-0.088600
C	-4.485350	-3.037820	1.528160
H	-2.814370	-1.714700	1.816260
H	-2.258240	-4.868840	-1.057920
H	-4.572010	-5.668820	-0.617830
H	-5.099460	-2.521330	2.261490
C	-6.389270	-4.667181	1.168560
H	-7.102230	-3.843491	1.285400
H	-6.397120	-5.235871	2.107770
H	-6.760490	-5.329221	0.380040
H	-2.110610	-1.691680	-4.006900
C	-0.707881	1.066030	0.309330
C	-1.103371	0.842980	1.636050
C	-1.050601	2.297950	-0.260660
C	-1.842251	1.793450	2.345490
H	-0.837641	-0.094610	2.116480
C	-1.761121	3.266210	0.455600
H	-0.762441	2.503760	-1.286600
C	-2.173201	3.017450	1.764500

H	-2.741201	3.755520	2.317100
C	-2.015551	4.599160	-0.183000
C	-2.277781	1.460310	3.744830
F	-0.881781	5.350870	-0.246820
F	-2.933101	5.333300	0.476100
F	-2.446931	4.483220	-1.470500
F	-1.221541	1.249720	4.564470
F	-3.017211	0.321760	3.775230
F	-3.028101	2.435710	4.302920
O	1.404059	-0.273029	0.224690
C	2.290500	-1.161519	-0.129380
C	3.175280	-1.701609	0.926230
C	3.772040	-2.957589	0.744750
C	3.381240	-1.003049	2.124730
C	4.573530	-3.506159	1.745270
H	3.573540	-3.520499	-0.164340
C	4.185750	-1.551819	3.120090
H	2.886709	-0.049029	2.272110
C	4.786110	-2.800709	2.932700
H	5.013920	-4.489789	1.607530
H	4.334420	-1.011959	4.051110
H	5.402180	-3.231259	3.717580
H	1.990140	-1.886179	-0.891390
C	3.539379	-0.264019	-1.506900
H	3.988340	-1.142969	-1.955880
H	2.724939	0.179141	-2.064890
C	4.383069	0.567801	-0.767200
C	3.995299	1.882011	-0.246530
C	2.781709	2.496491	-0.620090
C	4.845999	2.549441	0.660860
C	2.431659	3.737231	-0.092970
H	2.109719	2.012921	-1.318150
C	4.492149	3.788691	1.178270
H	5.773389	2.076691	0.960950
C	3.283499	4.384821	0.803500
H	1.486669	4.193681	-0.369720
H	5.153619	4.290251	1.878950
H	3.005509	5.352521	1.211970
O	5.567539	0.134281	-0.352020
Si	6.924920	-0.693099	-1.017400
C	7.930090	-1.101559	0.509100
H	8.219149	-0.194778	1.051880
H	7.353770	-1.734199	1.193270
H	8.847850	-1.639098	0.241970
C	7.772929	0.544711	-2.149480
H	8.054389	1.455561	-1.609620
H	8.686289	0.113132	-2.577240
H	7.124629	0.835941	-2.984220
C	6.450410	-2.248259	-1.962170
H	5.836460	-2.921089	-1.354790
H	5.916740	-2.035639	-2.894850
H	7.366130	-2.790929	-2.231010

TS 119

B3LYP/6-31G(d) = -3573.930918
B3LYP-D3(BJ)/def2-TZVPP/IEFPCM(propanonitrile) = -3575.504632
B3LYP-D3(BJ)/def2-TZVPP/IEFPCM(propanonitrile)//B3LYP-D3(BJ)/6-31G(d) Free Energy (Quasiharmonic) = -3574.7878

Frequencies (Top 3 out of 300)

1. -283.4790 cm^{-1}
2. 12.0708 cm^{-1}
3. 12.7490 cm^{-1}

B3LYP/6-31G(d) Molecular Geometry in Cartesian Coordinates

B	0.333220	-0.057110	-0.025540
O	-0.309100	0.893080	-0.985330
N	0.148700	-1.397290	-0.815150
C	-0.228000	-1.128860	-2.210900
S	0.873941	-2.837770	-0.412520
C	-0.540000	0.368500	-2.186930
O	-0.956760	1.014790	-3.129370
H	-1.155239	-2.988390	-2.780680
H	0.633440	-1.265970	-2.884940
C	-1.409949	-1.927980	-2.824970
C	-2.778620	-1.702601	-2.236720
C	-3.525269	-2.563601	-1.344270
C	-4.809169	-1.972201	-1.163130
N	-4.829760	-0.808381	-1.904470
C	-3.616740	-0.657611	-2.547560
H	-3.436160	0.186379	-3.196130
C	-5.503589	-3.777861	0.232320
C	-3.247599	-3.785681	-0.703190
H	-6.783129	-2.102241	-0.261880
H	-5.588840	-0.146141	-1.943180
C	-4.237319	-4.378281	0.071860
H	-6.258239	-4.271961	0.839050
H	-2.268079	-4.243041	-0.801210
C	-5.805489	-2.564381	-0.377540
H	-4.031109	-5.321831	0.569530
C	2.495141	-2.897910	-1.198100
O	0.100231	-3.927100	-1.030800
O	1.083061	-2.802570	1.039100
C	5.017941	-2.989609	-2.425860
C	2.600941	-3.263910	-2.543450
C	3.639961	-2.609719	-0.452540
C	4.887761	-2.658189	-1.070170
C	3.856851	-3.299759	-3.147080
H	1.716421	-3.555050	-3.099960
H	3.559021	-2.372159	0.600510
H	5.775401	-2.439689	-0.481350
H	3.936661	-3.588189	-4.192540
C	6.372731	-3.003059	-3.092800
H	6.391021	-3.681439	-3.952000
H	7.158921	-3.313409	-2.396480
H	6.640351	-2.003199	-3.459840
H	-1.425050	-1.639870	-3.882920
C	1.828220	0.441680	0.341850
C	2.509430	1.352160	-0.477210
C	2.493380	0.008190	1.499760
C	3.790520	1.815471	-0.157820
H	2.032270	1.722890	-1.379440
C	3.785980	0.440351	1.805510
H	2.001670	-0.700500	2.156770
C	4.443980	1.355711	0.983560
H	5.440390	1.703101	1.228590
C	4.496040	-0.159509	2.985620
C	4.417760	2.876791	-1.015380
F	3.676980	-0.315559	4.048760
F	5.548110	0.586301	3.391260
F	4.983970	-1.394159	2.688680
F	3.887139	4.102531	-0.751580
F	4.216010	2.651771	-2.332960
F	5.750420	2.980681	-0.821270
O	-0.412150	-0.082320	1.280410
C	-1.692290	-0.317760	1.401400
C	-2.108150	-1.149601	2.559080
C	-3.364820	-1.771481	2.549580
C	-1.245260	-1.340580	3.648630
C	-3.755309	-2.572071	3.620720
H	-4.025260	-1.652031	1.695000
C	-1.642429	-2.137480	4.718540
H	-0.264670	-0.877840	3.636570
C	-2.898469	-2.751671	4.708940
H	-4.722179	-3.066201	3.595680
H	-0.967789	-2.290440	5.556200
H	-3.202839	-3.378271	5.543080
H	-2.265430	-0.460661	0.482920

C	-2.547900	1.429419	2.008050
H	-1.887510	1.550859	2.859930
H	-3.543530	1.059839	2.220820
C	-2.424210	2.370519	0.981130
C	-3.511530	2.606299	0.003210
C	-4.842690	2.692499	0.452520
C	-3.239740	2.749219	-1.368890
C	-5.876940	2.940989	-0.446860
H	-5.060570	2.600869	1.512160
C	-4.281530	2.986299	-2.264910
H	-2.232730	2.611859	-1.744650
C	-5.598040	3.091219	-1.808680
H	-6.897290	3.028268	-0.084330
H	-4.059750	3.080999	-3.324160
H	-6.403831	3.291669	-2.509920
O	-1.279370	2.998480	0.862170
Si	-0.673761	4.542200	0.334900
C	0.917089	4.715430	1.306480
H	0.774899	4.458470	2.361620
H	1.709469	4.078140	0.901350
H	1.273959	5.752110	1.257850
C	-1.956971	5.816799	0.857270
H	-2.918521	5.664399	0.355450
H	-2.129371	5.795929	1.939520
H	-1.605311	6.823360	0.598290
C	-0.352191	4.570260	-1.514090
H	-0.016251	3.593620	-1.877400
H	-1.237151	4.859160	-2.089910
H	0.440849	5.297790	-1.728330

TS 120

B3LYP/6-31G(d) = -3573.929814
B3LYP-D3(BJ)/def2-TZVPP/IEFPCM(propanonitrile) = -3575.504865
B3LYP-D3(BJ)/def2-TZVPP/IEFPCM(propanonitrile)//B3LYP-D3(BJ)/6-31G(d) Free Energy (Quasiharmonic) = -3574.787729

Frequencies (Top 3 out of 300)

1. -266.6979 cm^{-1}
2. 6.3420 cm^{-1}
3. 13.6976 cm^{-1}

B3LYP/6-31G(d) Molecular Geometry in Cartesian Coordinates

B	0.427789	0.815586	-0.423702
O	0.581870	0.615213	-1.881011
N	0.984622	2.252085	-0.247784
C	1.653446	2.693338	-1.477790
S	0.753588	3.293573	0.997917
C	1.277201	1.595132	-2.479832
O	1.563090	1.603342	-3.654501
H	3.352704	3.846162	-0.842201
H	1.209671	3.626808	-1.853091
C	3.192237	2.915827	-1.393559
C	3.974979	1.810926	-0.745683
C	4.550871	0.638496	-1.363521
C	5.173306	-0.117117	-0.328837
N	4.995603	0.579137	0.850617
C	4.258351	1.718837	0.595114
H	3.978017	2.392442	1.392462
C	5.847829	-1.792421	-1.887374
C	4.598079	0.146687	-2.680847
H	6.289589	-1.894639	0.228392
H	5.161742	0.211152	1.775463
C	5.242438	-1.059673	-2.928584
H	6.340262	-2.734950	-2.111714
H	4.113427	0.689721	-3.487368
C	5.823463	-1.330417	-0.575637
H	5.276562	-1.450272	-3.942297
C	-0.279708	4.616491	0.329898
O	2.016048	3.949972	1.381970
O	-0.011494	2.570216	2.027770
C	-1.916620	6.695203	-0.604435
C	0.040794	5.940265	0.633421
C	-1.405558	4.319565	-0.440844
C	-2.212010	5.356956	-0.903316
C	-0.778589	6.967284	0.165967
H	0.929046	6.153421	1.217821
H	-1.637953	3.289019	-0.685618
H	-3.082789	5.123054	-1.511549
H	-0.523798	7.998674	0.398351
C	-2.812331	7.811669	-1.086862
H	-3.282881	7.563521	-2.044117
H	-2.255735	8.745897	-1.213772
H	-3.619590	8.008807	-0.368626
H	3.530275	3.085344	-2.423164
C	1.188625	-0.373701	0.380940
C	1.486168	-0.306875	1.752726
C	1.642546	-1.503777	-0.318907
C	2.222104	-1.314759	2.382455
H	1.159315	0.554868	2.327105
C	2.344287	-2.532607	0.318706
H	1.474192	-1.564227	-1.389200
C	2.645611	-2.441779	1.675426
H	3.211729	-3.223029	2.169839
C	2.734870	-3.762461	-0.449995
C	2.672869	-1.137490	3.804171
F	2.942599	-3.518922	-1.757189
F	1.741340	-4.708674	-0.396213
F	3.841118	-4.353873	0.045154
F	1.822208	-0.387982	4.530685
F	3.887549	-0.515561	3.859957
F	2.824927	-2.319413	4.445784
O	-1.068687	0.839283	-0.103126
C	-1.952176	0.065940	-0.679987
C	-3.343012	0.594027	-0.697139
C	-3.744091	1.568214	0.232049
C	-4.245817	0.160775	-1.677956
C	-5.032163	2.097330	0.174147
H	-3.031299	1.915459	0.973961
C	-5.531958	0.698709	-1.736914
H	-3.933877	-0.581917	-2.408150
C	-5.928185	1.665435	-0.809936
H	-5.333690	2.858184	0.888752
H	-6.219274	0.370942	-2.511847
H	-6.927290	2.089859	-0.860260
H	-1.630834	-0.455677	-1.585553
C	-2.032850	-1.538882	0.505806
H	-0.974732	-1.757862	0.430914
H	-2.326224	-1.034970	1.419769
C	-2.929072	-2.463408	-0.046657
C	-2.563701	-3.384290	-1.131935
C	-1.227881	-3.787079	-1.324054
C	-3.560050	-3.892381	-1.990457
C	-0.895681	-4.663059	-2.352450
H	-0.447557	-3.454836	-0.650884
C	-3.219730	-4.754159	-3.027343
H	-4.590949	-3.591039	-1.842230
C	-1.888170	-5.140699	-3.211415
H	0.137982	-4.971611	-2.468249
H	-3.992147	-5.128133	-3.693216
H	-1.626921	-5.819171	-4.018675
O	-4.215411	-2.446869	0.257886
Si	-5.172619	-2.403006	1.703035
C	-4.613026	-1.074418	2.904919
H	-4.573759	-0.086828	2.434228
H	-5.336630	-1.019324	3.728873
H	-3.634782	-1.285314	3.350147
C	-6.887575	-2.060968	1.033675
H	-6.920557	-1.094109	0.519830

H	-7.201067	-2.834149	0.323515
H	-7.622794	-2.036329	1.847000
C	-5.006267	-4.117359	2.452803
H	-5.628313	-4.204564	3.352190
H	-3.971935	-4.329532	2.747552
H	-5.322524	-4.895826	1.749870

TS 121

B3LYP/6-31G(d) = -3573.928351
B3LYP-D3(BJ)/def2-TZVPP/IEFPCM(propanonitrile) = -3575.504277
B3LYP-D3(BJ)/def2-TZVPP/IEFPCM(propanonitrile)//B3LYP-D3(BJ)/6-31G(d) Free Energy (Quasiharmonic) = -3574.787359

Frequencies (Top 3 out of 300)

1. -286.2572 cm^{-1}
2. 11.0379 cm^{-1}
3. 13.2787 cm^{-1}

B3LYP/6-31G(d) Molecular Geometry in Cartesian Coordinates

B	0.009479	-0.280285	-0.370266
O	0.394131	0.489606	-1.578809
N	-1.060583	-1.275273	-0.950570
C	-1.234561	-1.011102	-2.389582
S	-1.113647	-2.848590	-0.424770
C	-0.183633	0.056001	-2.694145
O	0.080587	0.472400	-3.805381
H	-3.339502	-1.365655	-2.730719
H	-0.987036	-1.893284	-2.992167
C	-2.655159	-0.513842	-2.798412
C	-3.189107	0.625165	-1.977232
C	-2.931001	2.041744	-2.121884
C	-3.613103	2.701442	-1.060302
N	-4.279036	1.734488	-0.333315
C	-3.996870	0.495703	-0.875065
H	-4.407346	-0.401472	-0.432732
C	-2.815529	4.826222	-1.792216
C	-2.196829	2.820013	-3.036413
H	-4.083235	4.569238	-0.058212
H	-4.678315	1.869937	0.583546
C	-2.145705	4.197933	-2.861998
H	-2.750087	5.904982	-1.678344
H	-1.657876	2.347975	-3.852099
C	-3.561031	4.087542	-0.881098
H	-1.576605	4.804703	-3.561600
C	-2.832935	-3.360472	-0.598083
O	-0.824036	-2.805607	1.019508
O	-0.337691	-3.740967	-1.305729
C	-5.501370	-4.188683	-0.854787
C	-3.229222	-4.080315	-1.725274
C	-3.747749	-3.073509	0.418581
C	-5.071044	-3.487561	0.282180
C	-4.558978	-4.483855	-1.848193
H	-2.496873	-4.337187	-2.483286
H	-3.423569	-2.554911	1.314768
H	-5.780571	-3.272516	1.077857
H	-4.865666	-5.046708	-2.726520
C	-6.945682	-4.603187	-1.005014
H	-7.549723	-3.783181	-1.415622
H	-7.386297	-4.878193	-0.040760
H	-7.049742	-5.456362	-1.682857
H	-2.592601	-0.240232	-3.859040
C	-0.538832	0.709304	0.786383
C	-0.044339	2.015949	0.895425
C	-1.566019	0.344260	1.669300
C	-0.531560	2.914031	1.851435
H	0.729212	2.345926	0.211036
C	-2.082485	1.245566	2.602504
H	-1.968302	-0.659658	1.618283
C	-1.562184	2.537921	2.709040
H	-1.950251	3.232687	3.445115
C	-3.272049	0.862823	3.433339
C	0.036209	4.304831	1.899803
F	-3.248968	1.424895	4.660577
F	-3.386394	-0.472125	3.596719
F	-4.441270	1.276932	2.851292
F	-0.429788	5.083322	0.900410
F	1.392385	4.288925	1.774794
F	-0.245636	4.935442	3.061780
O	1.269060	-1.036589	0.007938
C	1.769122	-1.174156	1.209702
C	2.542354	-2.418542	1.471304
C	2.845745	-3.305600	0.430043
C	2.930136	-2.736060	2.781778
C	3.539572	-4.484570	0.699144
H	2.493319	-3.085602	-0.571047
C	3.623896	-3.913454	3.047211
H	2.672119	-2.065192	3.598963
C	3.935514	-4.788724	2.002863
H	3.757488	-5.175004	-0.110891
H	3.911250	-4.154740	4.066963
H	4.471434	-5.711430	2.208886
H	1.138899	-0.844694	2.040000
C	3.054983	0.309675	1.555943
H	2.333729	1.105846	1.712306
H	3.548617	-0.064259	2.445016
C	3.814088	0.397389	0.378888
C	5.065510	-0.354972	0.185530
C	5.360940	-0.919287	-1.069237
C	5.964325	-0.547071	1.250249
C	6.527502	-1.655602	-1.252278
H	4.651580	-0.810324	-1.881773
C	7.138019	-1.270123	1.057807
H	5.759381	-0.100583	2.217902
C	7.420759	-1.827318	-0.191630
H	6.737100	-2.101846	-2.220005
H	7.833165	-1.398557	1.882240
H	8.334421	-2.396623	-0.337782
O	3.280386	1.034534	-0.630326
Si	3.684902	2.159396	-1.901028
C	3.478148	1.310737	-3.555083
H	3.716443	2.030981	-4.349358
H	4.140445	0.449138	-3.691105
H	2.439887	0.991169	-3.702334
C	5.451695	2.730462	-1.581773
H	5.710589	3.518932	-2.299685
H	5.564483	3.154845	-0.577151
H	6.184623	1.925301	-1.694906
C	2.459530	3.558938	-1.697036
H	2.460787	3.974830	-0.683494
H	2.699611	4.373596	-2.392146
H	1.445780	3.213503	-1.926047

TS 122

B3LYP/6-31G(d) = -3573.930507
B3LYP-D3(BJ)/def2-TZVPP/IEFPCM(propanonitrile) = -3575.503369
B3LYP-D3(BJ)/def2-TZVPP/IEFPCM(propanonitrile)//B3LYP-D3(BJ)/6-31G(d) Free Energy (Quasiharmonic) = -3574.787327

Frequencies (Top 3 out of 300)

1. -273.8673 cm^{-1}
2. 8.2793 cm^{-1}
3. 9.0162 cm^{-1}

B3LYP/6-31G(d) Molecular Geometry in Cartesian Coordinates

B	-0.187782	-0.462712	-0.346432
O	0.298037	0.142629	-1.617411
N	-1.257519	-1.483566	-0.849975
C	-1.428589	-1.317668	-2.305197
S	-1.378196	-3.019074	-0.221586
C	-0.335794	-0.324713	-2.703112
O	-0.069669	0.010483	-3.834952
H	-3.540705	-1.634342	-2.624092
H	-1.215073	-2.253258	-2.836529
C	-2.832912	-0.809516	-2.753860
C	-3.335991	0.404415	-2.026331
C	-3.066849	1.797743	-2.306999
C	-3.716927	2.561835	-1.296364
N	-4.372797	1.673567	-0.466601
C	-4.117760	0.386970	-0.898287
H	-4.525703	-0.459555	-0.364116
C	-2.908277	4.597620	-2.238580
C	-2.341678	2.476875	-3.303632
H	-4.133131	4.521260	-0.457337
H	-4.752982	1.896290	0.441467
C	-2.270145	3.864051	-3.259493
H	-2.825829	5.681123	-2.225371
H	-1.823383	1.923030	-4.080668
C	-3.643017	3.957543	-1.246912
H	-1.706705	4.395091	-4.022359
C	-3.121850	-3.454643	-0.352578
O	-1.078064	-2.919095	1.216384
O	-0.652864	-3.993633	-1.056448
C	-5.827579	-4.174462	-0.524937
C	-3.560130	-4.249491	-1.411229
C	-4.013642	-3.036606	0.638960
C	-5.355661	-3.397612	0.544722
C	-4.908598	-4.598965	-1.492759
H	-2.846767	-4.604714	-2.147362
H	-3.656728	-2.455482	1.482900
H	-6.048048	-3.080007	1.320954
H	-5.248730	-5.220548	-2.317525
C	-7.290889	-4.532072	-0.631059
H	-7.865075	-3.710970	-1.080354
H	-7.727417	-4.730664	0.353723
H	-7.443143	-5.417759	-1.255919
O	0.970945	-1.228241	0.272236
C	2.086168	-0.671007	0.655713
H	2.100912	0.421515	0.718193
H	-2.761726	-0.620260	-3.832099
C	2.838991	-1.387288	1.714100
C	3.914935	-0.760481	2.363578
C	2.426576	-2.665218	2.125154
C	4.578379	-1.406954	3.404656
H	4.219708	0.239524	2.066597
C	3.097953	-3.307639	3.163803
H	1.560407	-3.119410	1.655192
C	4.174543	-2.685020	3.802262
H	5.400994	-0.910925	3.912409
H	2.769052	-4.291340	3.486799
H	4.688084	-3.186969	4.618006
C	-0.732816	0.685379	0.652652
C	-1.620442	0.384871	1.696520
C	-0.404185	2.034018	0.459368
C	-2.182589	1.392532	2.481861
H	-1.889655	-0.649974	1.878722
C	-0.925490	3.043701	1.274569
H	0.234251	2.312552	-0.370656
C	-1.828978	2.730160	2.286974
H	-2.266066	3.511335	2.899653
C	-0.505564	4.473238	1.074783
C	-3.286255	1.067391	3.444164
F	0.504799	4.821412	1.912383
F	-0.062680	4.710646	-0.181773
F	-3.275805	-0.221967	3.839272
F	-1.518399	5.337213	1.315156
F	-4.511546	1.282726	2.869499
F	-3.254155	1.837266	4.553313
C	3.231826	-0.764756	-1.019934
H	3.360644	-1.839837	-1.082451
H	2.435807	-0.365253	-1.634407
C	4.371509	0.021627	-0.826924
C	4.347512	1.491387	-0.856432
C	3.190123	2.200032	-1.245694
C	5.489535	2.217918	-0.461731
C	3.175855	3.591569	-1.216542
H	2.301337	1.673922	-1.578122
C	5.470130	3.608171	-0.444501
H	6.381151	1.679327	-0.162760
C	4.312460	4.298169	-0.815176
H	2.270681	4.123143	-1.492549
H	6.355797	4.155845	-0.135313
H	4.295333	5.384132	-0.791345
O	5.528205	-0.506864	-0.450924
Si	6.336398	-1.995882	-0.837913
C	5.528121	-3.471290	-0.008570
H	4.553170	-3.727818	-0.435292
H	6.178272	-4.347370	-0.132707
H	5.391188	-3.307680	1.065193
C	6.330817	-2.137594	-2.711403
H	6.814933	-1.273471	-3.180087
H	6.877239	-3.035930	-3.024044
H	5.315212	-2.214354	-3.115055
C	8.050148	-1.684961	-0.143593
H	8.704106	-2.546695	-0.323577
H	8.517841	-0.808622	-0.605837
H	8.012956	-1.517239	0.938689

TS 123

B3LYP/6-31G(d) = -3573.927366
B3LYP-D3(BJ)/def2-TZVPP/IEFPCM(propanonitrile) = -3575.503578
B3LYP-D3(BJ)/def2-TZVPP/IEFPCM(propanonitrile)//B3LYP-D3(BJ)/6-31G(d) Free Energy (Quasiharmonic) = -3574.787242

Frequencies (Top 3 out of 300)

1. -292.8083 cm^{-1}
2. 6.8393 cm^{-1}
3. 15.0527 cm^{-1}

B3LYP/6-31G(d) Molecular Geometry in Cartesian Coordinates

B	0.163972	0.608572	-0.970403
O	-0.201316	0.047382	-2.285524
N	1.727851	0.769845	-1.114522
C	2.145028	0.279039	-2.438953
S	2.528901	2.062269	-0.459132
C	0.828629	-0.089725	-3.125452
O	0.723985	-0.471110	-4.269996
H	4.096552	-0.584764	-2.090263
H	2.606370	1.078625	-3.031892
C	3.115198	-0.941223	-2.419231
C	2.682447	-2.091700	-1.554912
C	1.807370	-3.192702	-1.894928
C	1.664339	-3.991910	-0.725020
N	2.438159	-3.415609	0.263137
C	3.024375	-2.269673	-0.237644
H	3.660553	-1.663000	0.390950
C	0.199600	-5.491223	-1.861566
C	1.130214	-3.583636	-3.064875
H	0.755117	-5.725376	0.215965
H	2.402715	-3.648397	1.244205
C	0.337063	-4.724362	-3.036406
H	-0.435549	-6.373038	-1.868387
H	1.203790	-2.985489	-3.967947

C	0.863314	-5.137862	-0.692031
H	-0.194669	-5.028385	-3.934233
C	4.155316	1.467919	0.039190
O	1.809381	2.395258	0.786192
O	2.777395	3.109089	-1.465905
C	6.700454	0.593872	0.832750
C	5.250238	1.670889	-0.800661
C	4.319076	0.853050	1.283633
C	5.585951	0.419531	1.667944
C	6.511195	1.225901	-0.402563
H	5.114188	2.185271	-1.746060
H	3.470740	0.726748	1.947909
H	5.713698	-0.056824	2.637331
H	7.362731	1.380593	-1.060711
C	8.071861	0.141160	1.274169
H	8.523965	0.867339	1.962591
H	8.026527	-0.818245	1.800977
H	8.750493	0.029652	0.422812
H	3.227823	-1.257949	-3.463277
C	-0.272068	-0.407161	0.213921
C	0.336050	-0.403171	1.478635
C	-1.155809	-1.461373	-0.063947
C	0.093477	-1.418408	2.407231
C	-1.430316	-2.460367	0.874172
H	-1.597762	-1.529704	-1.050311
C	-0.800380	-2.450085	2.117607
H	-0.980956	-3.242632	2.836338
C	-2.408892	-3.556598	0.559262
C	0.907019	-1.502280	3.664513
F	-2.601931	-3.720279	-0.763875
F	-2.022619	-4.746494	1.070249
F	-3.641960	-3.301846	1.097821
F	1.489087	-0.329174	3.992801
F	0.178443	-1.908181	4.728510
F	1.919740	-2.411119	3.530301
O	-0.502881	1.980085	-0.968087
C	-0.981519	2.652384	0.042989
C	-0.717449	4.118380	0.057781
C	-0.323543	4.787308	-1.107996
C	-0.848328	4.834365	1.256564
C	-0.075014	6.157913	-1.073133
H	-0.182827	4.221002	-2.021805
C	-0.597607	6.203387	1.288645
H	-1.123202	4.310582	2.169778
C	-0.216231	6.869499	0.120432
H	0.242530	6.669461	-1.977297
H	-0.687209	6.748922	2.223987
H	-0.016871	7.937390	0.144592
H	-0.972918	2.164212	1.019888
C	-2.990846	2.761035	-0.186102
H	-3.187318	3.573550	0.499713
H	-2.994553	3.048358	-1.231771
C	-3.523680	1.505442	0.140010
C	-3.805648	1.085853	1.523022
C	-3.722604	1.984422	2.607067
C	-4.169288	-0.249017	1.779952
C	-3.994172	1.555667	3.901387
H	-3.453860	3.022678	2.445894
C	-4.429688	-0.676027	3.078016
H	-4.223371	-0.952168	0.960790
C	-4.346382	0.223939	4.141881
H	-3.929747	2.260050	4.725539
H	-4.685289	-1.716398	3.253342
H	-4.551587	-0.108479	5.155653
O	-3.702402	0.573530	-0.773135
Si	-4.283494	0.575347	-2.422222
C	-3.029477	1.279996	-3.616831
H	-2.067463	0.765419	-3.533982
H	-3.402871	1.143966	-4.640474
H	-2.855312	2.351888	-3.472250
C	-5.874691	1.582060	-2.382229
H	-6.348407	1.572162	-3.371675
H	-6.593885	1.169311	-1.665769
H	-5.695150	2.630044	-2.116787
H	1.036740	0.386981	1.724250
C	-4.609371	-1.246735	-2.707202
H	-3.726507	-1.855626	-2.485392
H	-5.429257	-1.607586	-2.075873
H	-4.884852	-1.430496	-3.752601

TS 124

B3LYP/6-31G(d) = -3573.931566
B3LYP-D3(BJ)/def2-TZVPP/IEFPCM(propanonitrile) = -3575.503996
B3LYP-D3(BJ)/def2-TZVPP/IEFPCM(propanonitrile)//B3LYP-D3(BJ)/6-31G(d) Free Energy (Quasiharmonic) = -3574.787225

Frequencies (Top 3 out of 300)

1. -279.1936 cm^{-1}
2. 6.3272 cm^{-1}
3. 12.0314 cm^{-1}

B3LYP/6-31G(d) Molecular Geometry in Cartesian Coordinates

B	-0.130324	0.743910	-0.712475
O	-0.628283	0.454639	-2.077320
N	1.349199	1.194964	-0.999363
C	1.608516	1.117900	-2.448467
S	2.015822	2.463201	-0.153395
C	0.262482	0.695357	-3.037815
O	0.031746	0.551639	-4.221962
H	3.660497	0.425675	-2.463377
H	1.858534	2.104797	-2.857729
C	2.704853	0.104737	-2.889027
C	2.435280	-1.332696	-2.525813
C	3.099312	-2.120348	-1.513709
C	2.481505	-3.401466	-1.499933
N	1.514484	-3.397481	-2.482869
C	1.484109	-2.154688	-3.085824
H	0.782774	-1.946815	-3.881663
C	3.915367	-4.139713	0.255548
C	4.164688	-1.880545	-0.628757
H	2.364225	-5.376828	-0.609641
H	0.822940	-4.120093	-2.608087
C	4.562020	-2.886468	0.242878
H	4.242357	-4.903892	0.955749
H	4.670477	-0.920515	-0.622398
C	2.868830	-4.414614	-0.616419
H	5.382908	-2.705643	0.932138
C	3.775880	2.121070	-0.004790
O	1.909421	3.719886	-0.919815
O	1.461585	2.389628	1.209473
C	6.527063	1.692437	0.314138
C	4.675578	2.723590	-0.885010
C	4.231572	1.320039	1.044921
C	5.601309	1.116607	1.197325
C	6.042522	2.498704	-0.724538
H	4.306962	3.380200	-1.665954
H	3.526743	0.872493	1.737332
H	5.957865	0.500921	2.019656
H	6.744159	2.970194	-1.408436
C	8.006974	1.436410	0.472751
H	8.603209	2.233867	0.017875
H	8.289229	1.359271	1.528163
H	8.297190	0.494009	-0.010359
O	-0.944162	1.954739	-0.258440
C	-1.649926	2.093005	0.827231
H	-1.687704	1.241125	1.509906
H	2.790595	0.214600	-3.977266
C	-1.653043	3.432744	1.468974

C	-1.157227	4.556153	0.792659
C	-2.127434	3.567625	2.782945
C	-1.151813	5.798238	1.423789
H	-0.740545	4.439985	-0.201427
C	-2.118362	4.810127	3.410324
H	-2.488788	2.691817	3.318537
C	-1.635867	5.930398	2.727517
H	-0.753676	6.663039	0.900808
H	-2.476527	4.904544	4.431666
H	-1.624867	6.900708	3.216569
C	-0.241301	-0.552725	0.244502
C	-0.819182	-1.737853	-0.234238
C	0.318974	-0.590193	1.530950
C	-0.832805	-2.907722	0.529978
H	-1.250943	-1.745736	-1.228697
C	0.308691	-1.758260	2.298880
H	0.795856	0.301802	1.926426
C	-0.266974	-2.927409	1.805601
H	-0.268734	-3.833779	2.399506
C	0.899728	-1.724867	3.681038
C	-1.411448	-4.169427	-0.040637
F	1.164409	-2.961483	4.157897
F	2.055078	-1.021224	3.716605
F	-0.483995	-4.924588	-0.695148
F	0.061871	-1.133224	4.569560
F	-2.394367	-3.924515	-0.945090
F	-1.943392	-4.968368	0.912456
C	-3.640539	2.040338	0.307095
H	-4.051096	2.374280	1.249816
H	-3.584203	2.806135	-0.456936
C	-3.929058	0.731655	-0.098907
O	-3.760313	0.338300	-1.343567
Si	-4.015872	1.079254	-2.907081
C	-3.113183	2.716815	-3.084780
H	-2.972522	2.929357	-4.152033
H	-2.122201	2.672810	-2.625030
H	-3.667195	3.557496	-2.651982
C	-3.420372	-0.215129	-4.113917
H	-3.821458	-0.001509	-5.112990
H	-2.327564	-0.212646	-4.182041
H	-3.760169	-1.216759	-3.827176
C	-5.883349	1.323170	-2.979826
H	-6.168816	1.781725	-3.934801
H	-6.417088	0.369920	-2.895302
H	-6.240480	1.982603	-2.179918
C	-4.314383	-0.336671	0.842809
C	-4.711129	-0.055059	2.165696
C	-4.292747	-1.678010	0.417268
C	-5.069106	-1.083607	3.030753
H	-4.760984	0.968247	2.521675
C	-4.638259	-2.705317	1.289809
H	-3.977045	-1.907323	-0.591861
C	-5.029979	-2.411779	2.597418
H	-5.378828	-0.849443	4.045047
H	-4.581335	-3.735129	0.951614
H	-5.301832	-3.213545	3.278256

TS 125

B3LYP/6-31G(d) = -3573.929568
B3LYP-D3(BJ)/def2-TZVPP/IEFPCM(propanonitrile) = -3575.503527
B3LYP-D3(BJ)/def2-TZVPP/IEFPCM(propanonitrile)//B3LYP-D3(BJ)/6-31G(d) Free Energy (Quasiharmonic) = -3574.787131

Frequencies (Top 3 out of 300)

1. -232.6194 cm^{-1}
2. 6.7737 cm^{-1}
3. 7.8766 cm^{-1}

B3LYP/6-31G(d) Molecular Geometry in Cartesian Coordinates

B	-0.198761	-0.232226	-0.768307
O	-0.434079	-0.701640	-2.149388
N	-0.764976	-1.439812	0.052232
C	-1.096653	-2.563067	-0.846937
S	-0.382495	-1.739513	1.629614
C	-0.791608	-1.984447	-2.232701
O	-0.865314	-2.614297	-3.263991
H	-2.661231	-3.634845	0.187160
H	-0.411474	-3.403834	-0.677221
C	-2.538128	-3.146644	-0.785507
C	-3.685725	-2.203515	-1.023582
C	-4.260119	-1.785580	-2.287453
C	-5.338781	-0.908778	-1.985243
N	-5.435839	-0.830841	-0.610161
C	-4.433154	-1.593796	-0.044677
H	-4.346418	-1.665082	1.029431
C	-5.822292	-0.606550	-4.298665
C	-3.991150	-2.078343	-3.637895
H	-6.930423	0.372310	-2.718156
H	-5.974586	-0.140544	-0.110111
C	-4.772173	-1.489300	-4.623827
H	-6.408473	-0.154749	-5.094643
H	-3.167511	-2.731574	-3.904706
C	-6.120530	-0.305328	-2.975778
H	-4.564571	-1.705425	-5.668424
C	-1.896199	-1.901526	2.584666
O	0.318103	-3.035144	1.727426
O	0.279020	-0.523084	2.140251
C	-4.221605	-2.133909	4.129827
C	-2.330718	-3.163578	2.989390
C	-2.598494	-0.750987	2.951252
C	-3.756458	-0.876045	3.714499
C	-3.492196	-3.270361	3.754736
H	-1.756732	-4.043066	2.717962
H	-2.246517	0.226063	2.640483
H	-4.309040	0.019414	3.987634
H	-3.833541	-4.253508	4.069347
C	-5.459607	-2.254795	4.985989
H	-5.942999	-3.228490	4.857633
H	-5.211943	-2.149966	6.050639
H	-6.190855	-1.475979	4.745702
H	-2.548996	-3.946866	-1.535949
C	-0.855769	1.205169	-0.466766
C	-0.154196	2.414410	-0.589096
C	-2.208149	1.287398	-0.110707
C	-0.772352	3.644818	-0.350324
C	-2.830451	2.514943	0.141953
H	-2.784596	0.372294	-0.023526
C	-2.116821	3.704764	0.022298
H	-2.594455	4.658670	0.214237
C	-4.283017	2.536205	0.524004
C	0.011157	4.925249	-0.419831
F	-4.667121	3.721094	1.043902
F	-5.101847	2.283287	-0.527109
F	-4.571842	1.582215	1.455669
F	0.388717	5.352248	0.820361
F	1.143056	4.804871	-1.145014
F	-0.711108	5.934022	-0.957163
O	1.349268	-0.056547	-0.689065
C	2.174855	-1.055125	-0.772781
C	3.230760	-1.035816	-1.802949
C	3.581609	0.152617	-2.461540
C	3.845437	-2.240984	-2.177713
C	4.538865	0.131915	-3.472572
H	3.083318	1.076283	-2.188860
C	4.797770	-2.259210	-3.194671
H	3.554012	-3.167639	-1.688251
C	5.149460	-1.070512	-3.840738
H	4.799975	1.053145	-3.985664

H	5.252172	-3.199317	-3.494808
H	5.885380	-1.084250	-4.640065
H	1.787268	-2.044246	-0.510534
C	3.254727	-1.127738	1.075638
H	2.356986	-0.955096	1.654087
H	3.599147	-2.154452	1.027371
C	4.199268	-0.118485	0.945457
C	3.907785	1.306448	1.128082
C	4.858385	2.270963	0.732250
C	2.683244	1.738938	1.678284
C	4.586291	3.627023	0.868468
H	5.796297	1.941921	0.300736
C	2.423941	3.099226	1.816148
H	1.930320	1.024671	1.988897
C	3.364876	4.045503	1.405142
H	5.320778	4.360105	0.546908
H	1.472714	3.424697	2.222577
H	3.138167	5.103740	1.492660
O	5.423086	-0.385153	0.483780
Si	6.669244	-1.491057	0.903897
C	7.196958	-1.061884	2.656590
H	7.527276	-0.019977	2.732303
H	6.376102	-1.205933	3.368870
H	8.028086	-1.701558	2.978181
C	7.987365	-1.110734	-0.373069
H	8.860718	-1.761135	-0.243690
H	7.598971	-1.264110	-1.386166
H	8.328961	-0.072397	-0.298112
H	0.892582	2.397047	-0.867897
C	6.117360	-3.286245	0.792128
H	5.387341	-3.556567	1.562735
H	5.686061	-3.515171	-0.187918
H	6.989737	-3.938416	0.929217

TS 126

B3LYP/6-31G(d) = -3573.927393
B3LYP-D3(BJ)/def2-TZVPP/IEFPCM(propanonitrile) = -3575.503587
B3LYP-D3(BJ)/def2-TZVPP/IEFPCM(propanonitrile)//B3LYP-D3(BJ)/6-31G(d) Free Energy (Quasiharmonic) = -3574.787117

Frequencies (Top 3 out of 300)

1. -292.8246 cm^{-1}
2. 6.8641 cm^{-1}
3. 15.0103 cm^{-1}

B3LYP/6-31G(d) Molecular Geometry in Cartesian Coordinates

B	0.162665	0.610267	-0.969478
O	-0.199148	0.047810	-2.285042
N	1.726057	0.777225	-1.111915
C	2.146538	0.287879	-2.435795
S	2.521985	2.072205	-0.455332
C	0.832288	-0.085966	-3.123694
O	0.730400	-0.468214	-4.268190
H	4.100766	-0.568528	-2.084529
H	2.605575	1.089112	-3.028310
C	3.121211	-0.928745	-2.414752
C	2.691396	-2.080771	-1.551009
C	1.822228	-3.185814	-1.893039
C	1.679322	-3.985122	-0.723187
N	2.447650	-3.404902	0.266970
C	3.030143	-2.256553	-0.232615
H	3.661138	-1.646418	0.397845
C	0.224350	-5.491229	-1.863279
C	1.150084	-3.580116	-3.064740
H	0.774835	-5.722061	0.215963
H	2.410327	-3.637237	1.248083
C	0.361814	-4.724257	-3.038053
H	-0.406971	-6.375777	-1.871533
H	1.223508	-2.981904	-3.967786
C	0.883180	-5.134527	-0.692000
H	-0.166109	-5.030908	-3.937231
C	4.149882	1.481975	0.043282
O	2.767639	3.120316	-1.461455
O	1.800206	2.402158	0.789479
C	6.698271	0.619489	0.839456
C	5.245497	1.692351	-0.794491
C	4.315595	0.870151	1.288555
C	5.584824	0.444366	1.675211
C	6.508173	1.255550	-0.394178
H	5.108936	2.212257	-1.736787
H	3.468476	0.746246	1.954849
H	5.715210	-0.023446	2.648293
H	7.361128	1.422265	-1.047594
C	8.062426	0.123736	1.256074
H	8.196440	-0.932124	0.985738
H	8.861574	0.689435	0.766484
H	8.203862	0.201390	2.339266
H	3.236417	-1.245146	-3.458606
C	-0.271048	-0.407121	0.214303
C	0.335405	-0.400886	1.479808
C	-1.150482	-1.464632	-0.064728
C	0.095264	-1.416948	2.408148
C	-1.422512	-2.464630	0.873028
H	-1.590857	-1.534615	-1.051681
C	-0.794364	-2.451939	2.117354
H	-0.973012	-3.245098	2.835889
C	-2.396399	-3.564634	0.556753
C	0.907609	-1.497801	3.666397
F	-3.631040	-3.314930	1.094009
F	-2.587279	-3.728759	-0.766636
F	-2.005933	-4.753123	1.067874
F	1.483396	-0.322112	3.996403
F	0.179738	-1.908269	4.729116
F	1.925038	-2.401475	3.532637
O	-0.509096	1.979373	-0.967691
C	-0.991035	2.649800	0.043063
C	-0.732205	4.116705	0.058371
C	-0.866693	4.831976	1.257183
C	-0.339623	4.787262	-1.106921
C	-0.620825	6.201865	1.289767
H	-1.140531	4.307040	2.170046
C	-0.095961	6.158725	-1.071555
H	-0.196101	4.221651	-2.020725
C	-0.240750	6.869557	0.122032
H	-0.713179	6.746886	2.225140
H	0.220591	6.671580	-1.975327
H	-0.045165	7.938136	0.146594
H	-0.981653	2.161452	1.019864
C	-3.000630	2.751393	-0.187882
H	-3.200673	3.562843	0.498161
H	-3.004255	3.039265	-1.233397
C	-3.529244	1.493720	0.137035
C	-3.811254	1.072439	1.519527
C	-3.732254	1.970676	2.604148
C	-4.170808	-0.263750	1.775354
C	-4.003771	1.540335	3.897951
H	-3.466668	3.009875	2.443821
C	-4.431169	-0.692314	3.072914
H	-4.221760	-0.966620	0.955744
C	-4.351906	0.207340	4.137352
H	-3.942472	2.244470	4.722553
H	-4.683542	-1.733610	3.247416
H	-4.557083	-0.126298	5.150729
O	-3.703537	0.561587	-0.776750
Si	-4.282603	0.561951	-2.426526
C	-5.877777	1.562405	-2.388032
H	-6.350282	1.551022	-3.378041
H	-6.596191	1.146564	-1.672578

H	-5.702666	2.610985	-2.121984
C	-4.600985	-1.261255	-2.712790
H	-4.874508	-1.445586	-3.758603
H	-3.715986	-1.866771	-2.490245
H	-5.420187	-1.625642	-2.082605
H	1.033027	0.391721	1.726257
C	-3.029836	1.272060	-3.619209
H	-3.401133	1.134607	-4.643423
H	-2.860393	2.344652	-3.474225
H	-2.065827	0.761455	-3.534989

TS 127

B3LYP/6-31G(d) = -3573.933842
B3LYP-D3(BJ)/def2-TZVPP/IEFPCM(propanonitrile) = -3575.503433
B3LYP-D3(BJ)/def2-TZVPP/IEFPCM(propanonitrile)//B3LYP-D3(BJ)/6-31G(d) Free Energy (Quasiharmonic) = -3574.78707

Frequencies (Top 3 out of 300)

1. -273.0024 cm^{-1}
2. 9.7657 cm^{-1}
3. 10.5188 cm^{-1}

B3LYP/6-31G(d) Molecular Geometry in Cartesian Coordinates

B	0.359001	0.119930	-0.316580
O	0.789861	-1.280350	-0.549200
N	0.242421	0.134960	1.238830
C	0.576771	-1.182370	1.804250
S	0.150630	1.460970	2.224720
C	0.985642	-1.981029	0.568390
O	1.425952	-3.112659	0.593270
H	-0.832639	-1.309870	3.434170
H	1.443341	-1.103979	2.471440
C	-0.546488	-1.929990	2.579050
C	-1.756048	-2.323011	1.777030
C	-2.000837	-3.599751	1.139430
C	-3.260647	-3.506862	0.487000
N	-3.764898	-2.247482	0.744310
C	-2.849668	-1.539061	1.497140
H	-3.058159	-0.519601	1.787250
C	-3.079286	-5.745011	-0.310190
C	-1.289467	-4.811141	1.059970
H	-4.761787	-4.457942	-0.757940
H	-4.543258	-1.836282	0.251680
C	-1.834866	-5.867911	0.342580
H	-3.473456	-6.586692	-0.873570
H	-0.314117	-4.906620	1.526520
C	-3.807777	-4.563632	-0.248710
H	-1.289086	-6.805181	0.272020
C	-1.523300	1.622609	2.870200
O	0.364900	2.637000	1.365290
O	1.022560	1.221951	3.387130
C	-4.118630	1.910528	3.895930
C	-1.811890	1.174819	4.160370
C	-2.508210	2.241219	2.095650
C	-3.795100	2.376138	2.612520
C	-3.105150	1.318239	4.661420
H	-1.023900	0.744280	4.768810
H	-2.273310	2.633549	1.113520
H	-4.556171	2.865108	2.009170
H	-3.326760	0.972978	5.668340
C	-5.524050	2.036507	4.433650
H	-6.028341	2.922147	4.033390
H	-5.530850	2.104377	5.526340
H	-6.130660	1.163657	4.157750
H	-0.074108	-2.827630	2.994880
C	-0.975089	0.499070	-1.145720
C	-1.845559	-0.492401	-1.628340
C	-1.335400	1.834429	-1.371990
C	-3.027329	-0.159621	-2.296720
H	-1.596338	-1.537911	-1.480830
C	-2.528600	2.171359	-2.020670
H	-0.683000	2.627820	-1.020500
C	-3.381860	1.177378	-2.491690
H	-4.299530	1.436768	-3.007100
C	-2.909381	3.619969	-2.131460
C	-3.976069	-1.226812	-2.764260
F	-3.315051	4.112798	-0.928940
F	-3.924181	3.826708	-2.999110
F	-1.872911	4.390269	-2.529300
F	-3.395928	-2.434812	-2.874160
F	-4.531179	-0.925642	-3.959140
F	-5.029038	-1.377512	-1.896770
O	1.446020	1.046751	-0.886620
C	2.463870	1.570541	-0.274060
C	2.837729	2.955631	-0.655160
C	2.433499	3.497481	-1.883060
C	3.577259	3.741812	0.240220
C	2.775399	4.807111	-2.212760
H	1.839029	2.892011	-2.559490
C	3.914138	5.051792	-0.091220
H	3.862939	3.331982	1.206080
C	3.518358	5.585162	-1.320820
H	2.454728	5.225191	-3.162830
H	4.473888	5.660882	0.612960
H	3.779168	6.607992	-1.578420
H	2.632980	1.299071	0.768960
C	4.233531	0.668742	-0.984650
H	4.023370	0.766192	-2.043620
H	4.898300	1.419222	-0.581180
C	4.295301	-0.615278	-0.438870
C	4.793161	-0.885128	0.925970
C	5.586531	0.047003	1.623580
C	4.473222	-2.107358	1.549490
C	6.042841	-0.234177	2.907100
H	5.870720	0.985893	1.160250
C	4.926062	-2.377438	2.838180
H	3.842182	-2.819128	1.030730
C	5.712102	-1.445917	3.519430
H	6.659521	0.491173	3.429740
H	4.659792	-3.317678	3.312160
H	6.066422	-1.661337	4.523660
O	3.811002	-1.668418	-1.055380
Si	3.535202	-2.171258	-2.699280
C	2.301271	-1.077499	-3.594350
H	1.351751	-1.042879	-3.054730
H	2.652291	-0.050159	-3.737390
H	2.114122	-1.501459	-4.589850
C	2.907923	-3.915268	-2.465850
H	3.707973	-4.584768	-2.129930
H	2.121493	-3.921429	-1.703110
H	2.497613	-4.319359	-3.398850
C	5.232222	-2.096057	-3.516000
H	5.629511	-1.075177	-3.554260
H	5.958052	-2.721307	-2.983920
H	5.171162	-2.463217	-4.548070

TS 128

B3LYP/6-31G(d) = -3573.92915
B3LYP-D3(BJ)/def2-TZVPP/IEFPCM(propanonitrile) = -3575.503703
B3LYP-D3(BJ)/def2-TZVPP/IEFPCM(propanonitrile)//B3LYP-D3(BJ)/6-31G(d) Free Energy (Quasiharmonic) = -3574.78701

Frequencies (Top 3 out of 300)

1. -269.7249 cm^{-1}
2. 9.1878 cm^{-1}

3. 14.1500 cm^{-1}

B3LYP/6-31G(d) Molecular Geometry in Cartesian Coordinates

B	-0.017563	-0.232504	-0.030083
O	0.532064	0.747093	-1.005795
N	0.244152	-1.568062	-0.765439
C	0.707856	-1.320171	-2.136263
S	0.385008	-3.078123	-0.129889
C	0.963885	0.187425	-2.129769
O	1.479464	0.813889	-3.039842
H	-0.387062	-2.795599	-3.255429
H	1.667221	-1.822969	-2.326557
C	-0.269468	-1.709089	-3.281031
C	-1.612335	-1.035118	-3.225894
C	-2.844369	-1.570746	-2.692853
C	-3.835348	-0.562247	-2.836170
N	-3.237123	0.520145	-3.451269
C	-1.900927	0.236959	-3.661571
H	-1.249265	0.966568	-4.123434
C	-5.477857	-1.953257	-1.814562
C	-3.205419	-2.795882	-2.102058
H	-5.886782	0.060108	-2.499685
H	-3.648894	1.438917	-3.505600
C	-4.513888	-2.972816	-1.670921
H	-6.489530	-2.120449	-1.454659
H	-2.465255	-3.576693	-1.957593
C	-5.153144	-0.735289	-2.399091
H	-4.799775	-3.909268	-1.199903
C	2.155619	-3.448359	-0.078325
O	-0.182763	-4.063413	-1.063267
O	-0.096508	-3.012775	1.257873
C	4.878119	-4.116627	0.073563
C	3.007938	-2.702477	0.741211
C	2.648254	-4.522549	-0.818379
C	4.003672	-4.847442	-0.738570
C	4.356666	-3.041310	0.811903
H	2.613893	-1.868127	1.310617
H	1.968314	-5.099230	-1.435582
H	4.383596	-5.688950	-1.312941
H	5.017523	-2.469549	1.460269
C	6.347212	-4.460557	0.145271
H	6.534868	-5.486197	-0.187288
H	6.733349	-4.358643	1.165547
H	6.942223	-3.794675	-0.493918
H	0.247382	-1.457868	-4.215767
C	-1.542578	0.147084	0.353350
C	-2.125602	1.322805	-0.140657
C	-2.335416	-0.666811	1.180665
C	-3.445714	1.670359	0.164811
H	-1.543485	1.974170	-0.783632
C	-3.651625	-0.319249	1.492101
H	-1.920180	-1.587868	1.577647
C	-4.217911	0.850721	0.985059
H	-5.238654	1.118954	1.231334
C	-4.493689	-1.242942	2.330645
C	-4.058284	2.893175	-0.451980
F	-5.454243	-0.567712	3.010340
F	-3.755545	-1.913177	3.242526
F	-5.130311	-2.170639	1.580657
F	-5.040285	3.420708	0.310337
F	-4.627080	2.627259	-1.667759
F	-3.148422	3.869674	-0.673211
O	0.853236	-0.190941	1.235985
C	0.889297	0.836297	2.031084
C	0.904448	0.560334	3.489444
C	0.752934	1.612247	4.407901
C	0.989328	-0.760584	3.956612
C	0.709827	1.352350	5.773853
H	0.655545	2.634517	4.048025
C	0.947394	-1.014710	5.326800
H	1.035417	-1.577229	3.243814
C	0.815556	0.036613	6.236313
H	0.584716	2.170336	6.477635
H	1.000847	-2.039384	5.683148
H	0.778592	-0.167652	7.302823
H	0.344072	1.731398	1.721271
C	2.772953	1.740812	1.867286
H	2.597854	2.460856	2.655545
H	3.360271	0.868395	2.133482
C	2.901952	2.228625	0.567343
C	2.397312	3.559297	0.176124
C	1.789824	3.747654	-1.080591
C	2.492507	4.648228	1.065033
C	1.280774	4.998109	-1.424385
H	1.680175	2.907385	-1.757599
C	1.996990	5.897588	0.704709
H	2.988544	4.524103	2.023034
C	1.384766	6.073439	-0.539231
H	0.795316	5.130497	-2.386880
H	2.090910	6.734268	1.390972
H	0.989801	7.046820	-0.816682
O	3.381980	1.421561	-0.360156
Si	4.706624	1.473173	-1.479175
C	4.739262	-0.248128	-2.207739
H	3.867588	-0.396112	-2.853498
H	5.639374	-0.384826	-2.820049
H	4.733414	-1.022260	-1.433208
C	4.478704	2.770805	-2.813732
H	5.338001	2.727996	-3.496258
H	3.575603	2.563287	-3.396166
H	4.416122	3.790301	-2.420829
C	6.216794	1.844126	-0.415354
H	6.142042	2.825203	0.068724
H	7.124432	1.851877	-1.031361
H	6.353207	1.090590	0.368556

TS 129

B3LYP/6-31G(d) = -3573.926667
B3LYP-D3(BJ)/def2-TZVPP/IEFPCM(propanonitrile) = -3575.50301
B3LYP-D3(BJ)/def2-TZVPP/IEFPCM(propanonitrile)//B3LYP-D3(BJ)/6-31G(d) Free Energy (Quasiharmonic) = -3574.786992

Frequencies (Top 3 out of 300)

1. -212.2599 cm^{-1}
2. 10.0325 cm^{-1}
3. 12.9399 cm^{-1}

B3LYP/6-31G(d) Molecular Geometry in Cartesian Coordinates

B	0.228251	-0.023430	-0.899110
O	0.386971	-0.387590	-2.322370
N	0.751521	1.469970	-0.901980
C	1.324231	1.777270	-2.229510
S	0.075511	2.698320	-0.042170
C	0.953271	0.556410	-3.074770
O	1.160061	0.459930	-4.263520
H	3.061851	3.003929	-1.826460
H	0.831691	2.646000	-2.683750
C	2.864871	2.017379	-2.257490
C	3.696051	0.986909	-1.547260
C	4.151921	-0.298291	-2.032960
C	4.864610	-0.917871	-0.968100
N	4.869381	-0.035471	0.095210
C	4.145231	1.086859	-0.253970
H	4.015771	1.893919	0.453190
C	5.277630	-2.854391	-2.295590
C	4.030090	-0.988841	-3.253340
H	5.961020	-2.650221	-0.252930

H	5.106721	-0.275711	1.046100
C	4.590260	-2.254861	-3.370430
H	5.696380	-3.849831	-2.416130
H	3.481631	-0.551661	-4.081930
C	5.428780	-2.192291	-1.082860
H	4.489980	-2.798731	-4.305820
C	1.370741	3.888610	0.320350
O	-0.355009	2.126050	1.250790
O	-0.927519	3.426080	-0.851170
C	3.354721	5.773089	0.924930
C	1.528041	5.010160	-0.494510
C	2.171701	3.708770	1.450650
C	3.155291	4.650639	1.743350
C	2.521761	5.939709	-0.189540
H	0.867081	5.159430	-1.341520
H	2.010321	2.856190	2.101610
H	3.773251	4.517129	2.627990
H	2.644342	6.814399	-0.823400
C	4.447452	6.768139	1.233230
H	4.224032	7.753179	0.811590
H	5.406102	6.440349	0.809970
H	4.592132	6.883199	2.312560
H	3.136061	2.077379	-3.318840
C	1.008990	-1.046960	0.065510
C	1.488360	-0.683720	1.328360
C	1.267510	-2.356640	-0.367990
C	2.205170	-1.584720	2.122360
H	1.304291	0.320090	1.697010
C	1.970200	-3.265570	0.427040
H	0.942110	-2.660980	-1.357540
C	2.449440	-2.884121	1.679850
H	3.011020	-3.580741	2.290930
C	2.183490	-4.674770	-0.053340
C	2.788220	-1.105291	3.415790
F	1.128630	-5.472360	0.256360
F	3.274730	-5.243891	0.511710
F	2.338740	-4.740080	-1.390920
F	1.921811	-0.324890	4.110610
F	3.899871	-0.333201	3.213850
F	3.165430	-2.110601	4.231620
O	-1.273829	-0.224740	-0.605270
C	-2.243439	0.380050	-1.221230
C	-3.252559	-0.420840	-1.930760
C	-4.071559	0.198360	-2.889030
C	-3.359540	-1.804410	-1.716870
C	-4.984219	-0.554459	-3.624490
H	-3.971239	1.265830	-3.072090
C	-4.277370	-2.551880	-2.449630
H	-2.705380	-2.277950	-0.992790
C	-5.090840	-1.930899	-3.402430
H	-5.599819	-0.073949	-4.379790
H	-4.350880	-3.623600	-2.288220
H	-5.795980	-2.520579	-3.981950
H	-2.050629	1.383430	-1.611190
C	-3.398239	1.361010	0.355570
H	-3.970899	2.031490	-0.274380
H	-2.503879	1.772510	0.802910
C	-4.055759	0.300680	0.956300
C	-3.444789	-0.605210	1.936520
C	-4.144710	-1.755660	2.356640
C	-2.165539	-0.352540	2.473330
C	-3.583900	-2.622370	3.287000
H	-5.121900	-1.962839	1.936800
C	-1.612080	-1.224670	3.407310
H	-1.600919	0.519880	2.169290
C	-2.316810	-2.358030	3.816660
H	-4.131740	-3.507080	3.599360
H	-0.623580	-1.018880	3.805810
H	-1.880170	-3.036240	4.544810
O	-5.284009	-0.052169	0.558680
Si	-6.778109	0.775291	0.392250
C	-7.916929	-0.574489	-0.236700
H	-7.545100	-0.983249	-1.182980
H	-7.991920	-1.400579	0.479130
H	-8.928489	-0.188059	-0.409800
C	-6.685469	2.202091	-0.831350
H	-6.076509	3.036381	-0.466600
H	-6.283779	1.877841	-1.797100
H	-7.696689	2.591331	-1.007590
C	-7.249249	1.394961	2.104140
H	-7.317769	0.572111	2.824330
H	-6.514709	2.113711	2.485950
H	-8.222449	1.900551	2.078810

TS 130

B3LYP/6-31G(d) = -3573.929532
B3LYP-D3(BJ)/def2-TZVPP/IEFPCM(propanonitrile) = -3575.503135
B3LYP-D3(BJ)/def2-TZVPP/IEFPCM(propanonitrile)//B3LYP-D3(BJ)/6-31G(d) Free Energy (Quasiharmonic) = -3574.786979

Frequencies (Top 3 out of 300)

1. -232.9774 cm^{-1}
2. 7.1710 cm^{-1}
3. 9.0473 cm^{-1}

B3LYP/6-31G(d) Molecular Geometry in Cartesian Coordinates

B	0.204130	-0.222476	0.762486
O	0.449092	-0.696845	2.139926
N	0.778677	-1.420760	-0.066963
C	1.119869	-2.546826	0.825395
S	0.383531	-1.718066	-1.641994
C	0.816930	-1.977413	2.215573
O	0.899842	-2.611440	3.243422
H	2.691894	-3.588445	-0.228000
H	0.438717	-3.390484	0.653517
C	2.564657	-3.120670	0.754076
C	3.706097	-2.175932	1.015140
C	4.287803	-1.803247	2.289533
C	5.357127	-0.906727	2.013720
N	5.441265	-0.772710	0.642077
C	4.439728	-1.521155	0.055037
H	4.343463	-1.549214	-1.020381
C	5.857994	-0.694259	4.333520
C	4.031260	-2.151052	3.629169
H	6.947020	0.354300	2.784862
H	5.968245	-0.056851	0.165823
C	4.816134	-1.596204	4.631683
H	6.447892	-0.270727	5.142194
H	3.212923	-2.818750	3.875814
C	6.143283	-0.338252	3.021398
H	4.618166	-1.854559	5.668527
C	1.891696	-1.893106	-2.603426
O	-0.272488	-0.497005	-2.148537
O	-0.327887	-3.008395	-1.736139
C	4.203199	-2.145916	-4.166434
C	2.307317	-3.157839	-3.018544
C	2.603319	-0.748681	-2.972646
C	3.752866	-0.883812	-3.746409
C	3.460702	-3.274505	-3.795095
H	1.721638	-4.031071	-2.751997
H	2.260803	0.231709	-2.661696
H	4.308172	0.007272	-4.028461
H	3.783300	-4.259332	-4.123680
C	5.465149	-2.280395	-4.984849
H	5.485423	-3.224277	-5.538468
H	5.564984	-1.460525	-5.704162
H	6.355201	-2.257898	-4.342315
H	2.580268	-3.935670	1.488222

C	0.847726	1.221830	0.465231
C	0.141212	2.425970	0.607682
C	2.194144	1.315120	0.090857
C	0.749299	3.661788	0.370543
C	2.806248	2.547809	-0.160601
H	2.774141	0.404028	-0.011064
C	2.087963	3.732612	-0.020851
H	2.557260	4.690832	-0.212152
C	4.252936	2.579286	-0.563103
C	-0.039749	4.937476	0.461655
F	4.620227	3.764821	-1.093030
F	5.088934	2.338017	0.478255
F	4.536941	1.623048	-1.493320
F	0.682411	5.943389	1.004526
F	-0.429511	5.376951	-0.770371
F	-1.164813	4.803352	1.194858
O	-1.345326	-0.059184	0.689557
C	-2.164046	-1.064169	0.765731
C	-3.217241	-1.061693	1.798859
C	-3.574136	0.117959	2.469861
C	-3.822918	-2.274484	2.163578
C	-4.528431	0.081148	3.483227
H	-3.082761	1.047510	2.204733
C	-4.772310	-2.308849	3.182875
H	-3.526743	-3.194380	1.664319
C	-5.130041	-1.128810	3.841383
H	-4.794222	0.995623	4.005910
H	-5.219701	-3.254795	3.475096
H	-5.863638	-1.155152	4.642528
H	-1.770863	-2.048062	0.492555
C	-3.247738	-1.125476	-1.080431
H	-2.352944	-0.941858	-1.659916
H	-3.585304	-2.154817	-1.041254
C	-4.197990	-0.123228	-0.938374
C	-3.915383	1.304978	-1.108886
C	-4.871152	2.260155	-0.702990
C	-2.694473	1.749745	-1.657434
C	-4.607728	3.618985	-0.828016
H	-5.806216	1.921580	-0.272676
C	-2.443860	3.112765	-1.784006
H	-1.937648	1.042889	-1.975572
C	-3.389932	4.049615	-1.363295
H	-5.346149	4.344710	-0.498869
H	-1.495471	3.447637	-2.189434
H	-3.169963	5.109943	-1.442163
O	-5.418922	-0.401386	-0.475877
Si	-6.659399	-1.511229	-0.902501
C	-7.195993	-1.068242	-2.649051
H	-7.533034	-0.027724	-2.713395
H	-6.376797	-1.200203	-3.365572
H	-8.024276	-1.709912	-2.973988
C	-7.975465	-1.151941	0.382649
H	-8.845124	-1.806577	0.249794
H	-7.582595	-1.312848	1.392847
H	-8.323882	-0.115108	0.319145
H	-0.901513	2.400558	0.900788
C	-6.095660	-3.303839	-0.810377
H	-5.366364	-3.561759	-1.585897
H	-5.659813	-3.539757	0.165990
H	-6.964297	-3.960180	-0.951275

TS 131

B3LYP/6-31G(d) = -3573.929532
B3LYP-D3(BJ)/def2-TZVPP/IEFPCM(propanonitrile) = -3575.503136
B3LYP-D3(BJ)/def2-TZVPP/IEFPCM(propanonitrile)//B3LYP-D3(BJ)/6-31G(d) Free Energy (Quasiharmonic) = -3574.786961

Frequencies (Top 3 out of 300)

1. -232.9804 cm^{-1}
2. 7.1760 cm^{-1}
3. 9.0733 cm^{-1}

B3LYP/6-31G(d) Molecular Geometry in Cartesian Coordinates

B	0.204121	-0.222330	0.762531
O	0.449076	-0.696469	2.140053
N	0.778670	-1.420756	-0.066714
C	1.119865	-2.546668	0.825836
S	0.383501	-1.718336	-1.641688
C	0.816929	-1.977018	2.215918
O	0.899854	-2.610870	3.243875
H	2.691877	-3.588497	-0.227373
H	0.438714	-3.390358	0.654109
C	2.564654	-3.120523	0.754609
C	3.706098	-2.175731	1.015458
C	4.287814	-1.802763	2.289764
C	5.357135	-0.906304	2.013745
N	5.441263	-0.772590	0.642071
C	4.439721	-1.521164	0.055204
H	4.343446	-1.549459	-1.020206
C	5.858020	-0.693323	4.333494
C	4.031281	-2.150273	3.629479
H	6.947035	0.354893	2.784596
H	5.968234	-0.056829	0.165658
C	4.816162	-1.595202	4.631864
H	6.447923	-0.269613	5.142070
H	3.212948	-2.817918	3.876278
C	6.143298	-0.337607	3.021291
H	4.618202	-1.853328	5.668767
C	1.891656	-1.893624	-2.603091
O	-0.272473	-0.497343	-2.148453
O	-0.327971	-3.008654	-1.735593
C	4.203142	-2.146841	-4.166058
C	2.603288	-0.749312	-2.972577
C	2.307240	-3.158474	-3.017947
C	3.460594	-3.275343	-3.794478
C	3.752836	-0.884648	-3.746337
H	2.260788	0.231161	-2.661871
H	1.721519	-4.031623	-2.751222
H	3.783140	-4.260251	-4.122882
H	4.308145	0.006356	-4.028624
C	5.465201	-2.281674	-4.984246
H	6.354901	-2.264317	-4.341049
H	5.567837	-1.459293	-5.700261
H	5.483160	-3.223464	-5.541517
H	2.580278	-3.935374	1.488921
C	0.847731	1.221920	0.465043
C	0.141253	2.426095	0.607378
C	2.194130	1.315132	0.090584
C	0.749357	3.661869	0.370047
H	-0.901457	2.400746	0.900545
C	2.806252	2.547773	-0.161063
H	2.774100	0.404013	-0.011247
C	2.088001	3.732612	-0.021429
H	2.557310	4.690796	-0.212876
C	4.252924	2.579167	-0.563625
C	-0.039661	4.937584	0.461035
F	4.620191	3.764592	-1.093816
F	4.536901	1.622732	-1.493646
F	5.088962	2.338126	0.477754
F	0.682568	5.943571	1.003676
F	-0.429539	5.376873	-0.771021
F	-1.164655	4.803593	1.194371
O	-1.345331	-0.059044	0.689566
C	-2.164059	-1.064013	0.765892
C	-3.217260	-1.061366	1.799014
C	-3.574158	0.118398	2.469820
C	-3.822941	-2.274095	2.163930
C	-4.528461	0.081755	3.483185

H	-3.082782	1.047905	2.204541
C	-4.772340	-2.308291	3.183226
H	-3.526763	-3.194074	1.664825
C	-5.130075	-1.128143	3.841537
H	-4.794255	0.996316	4.005715
H	-5.219733	-3.254189	3.475600
H	-5.863678	-1.154352	4.642680
H	-1.770878	-2.047952	0.492879
C	-3.247721	-1.125597	-1.080264
H	-3.585264	-2.154941	-1.040958
H	-2.352923	-0.942034	-1.659759
C	-4.197998	-0.123349	-0.938368
C	-3.915406	1.304839	-1.109068
C	-4.871214	2.260057	-0.703360
C	-2.694473	1.749547	-1.657609
C	-4.607805	3.618873	-0.828565
H	-5.806297	1.921528	-0.273050
C	-2.443874	3.112554	-1.784360
H	-1.937618	1.042659	-1.975607
C	-3.389986	4.049448	-1.363837
H	-5.346257	4.344634	-0.499565
H	-1.495468	3.447384	-2.189783
H	-3.170030	5.109768	-1.442846
O	-5.418939	-0.401462	-0.475872
Si	-6.659367	-1.511418	-0.902348
C	-6.095581	-3.303997	-0.809900
H	-6.964199	-3.960384	-0.950696
H	-5.366267	-3.562034	-1.585364
H	-5.659743	-3.539732	0.166515
C	-7.195915	-1.068743	-2.648991
H	-7.533008	-0.028254	-2.713519
H	-6.376684	-1.200779	-3.365458
H	-8.024152	-1.710510	-2.973856
C	-7.975486	-1.151951	0.382698
H	-8.845126	-1.806628	0.249918
H	-7.582648	-1.312687	1.392936
H	-8.323924	-0.115136	0.319015

TS 132

B3LYP/6-31G(d) = -3573.929128
B3LYP-D3(BJ)/def2-TZVPP/IEFPCM(propanonitrile) = -3575.503698
B3LYP-D3(BJ)/def2-TZVPP/IEFPCM(propanonitrile)//B3LYP-D3(BJ)/6-31G(d) Free Energy (Quasiharmonic) = -3574.786926

Frequencies (Top 3 out of 300)

1. -232.3549 cm^{-1}
2. 9.6783 cm^{-1}
3. 12.5639 cm^{-1}

B3LYP/6-31G(d) Molecular Geometry in Cartesian Coordinates

B	-0.001389	0.088962	1.037559
O	0.192583	-0.109852	2.494240
N	0.805474	-1.120553	0.468790
C	1.153893	-2.059674	1.553735
S	0.620579	-1.697503	-1.070704
C	0.738955	-1.288294	2.814727
O	0.896354	-1.688786	3.944242
H	2.796467	-3.244245	0.812882
H	0.520896	-2.956673	1.500197
C	2.610943	-2.565191	1.652524
C	3.713872	-1.542361	1.701608
C	5.070756	-1.769778	1.258245
C	5.801334	-0.571525	1.491572
N	4.936165	0.323334	2.084746
C	3.686281	-0.261230	2.195389
H	2.873581	0.296449	2.637044
C	7.786342	-1.543508	0.594358
C	5.742252	-2.869948	0.697387
H	7.691352	0.485660	1.338634
H	5.103611	1.313715	2.180898
C	7.088531	-2.748890	0.371448
H	8.838638	-1.477666	0.330784
H	5.218371	-3.805647	0.521321
C	7.153124	-0.441111	1.157270
H	7.617537	-3.598881	-0.052202
C	2.248670	-1.944572	-1.780200
O	-0.025990	-0.616331	-1.840639
O	-0.030129	-3.024402	-1.024240
C	4.759852	-2.332547	-2.954216
C	3.050927	-0.838965	-2.074182
C	2.670286	-3.237316	-2.089471
C	3.922378	-3.420106	-2.676879
C	4.299112	-1.041852	-2.652021
H	2.705561	0.166060	-1.862973
H	2.019094	-4.079030	-1.880376
H	4.253817	-4.426599	-2.920509
H	4.928770	-0.181883	-2.864792
C	6.138820	-2.538642	-3.530077
H	6.889962	-2.506235	-2.730287
H	6.394870	-1.755310	-4.251674
H	6.226266	-3.507997	-4.031214
O	-1.541943	0.006871	0.836106
C	-2.224945	-1.095250	0.919855
H	-1.676920	-2.032494	0.789839
H	2.627233	-3.189665	2.556973
C	-3.388914	-1.151238	1.825094
C	-3.871545	-2.401128	2.244365
C	-3.977730	0.019828	2.326173
C	-4.926975	-2.480510	3.150726
H	-3.399412	-3.309827	1.877464
C	-5.036771	-0.063108	3.226472
H	-3.584390	0.982969	2.020284
C	-5.514321	-1.310181	3.640085
H	-5.280249	-3.451169	3.487467
H	-5.483138	0.846585	3.618050
H	-6.331645	-1.370182	4.353564
C	0.460632	1.562044	0.580394
C	-0.405294	2.667164	0.576665
C	1.801739	1.808486	0.263397
C	0.049745	3.950991	0.264714
H	-1.452133	2.524546	0.816749
C	2.267453	3.092086	-0.039947
H	2.501353	0.980188	0.260152
C	1.393933	4.175878	-0.041353
H	1.748491	5.173284	-0.274990
C	3.730891	3.288949	-0.311737
C	-0.904574	5.109796	0.191182
F	3.999546	4.478459	-0.886941
F	4.468953	3.238596	0.835871
F	-1.230689	5.407626	-1.099242
F	4.230168	2.324523	-1.121665
F	-0.372366	6.238556	0.712113
F	-2.064824	4.872057	0.837607
C	-3.052781	-1.470960	-1.015143
H	-3.263132	-2.527618	-0.897349
H	-2.123255	-1.228607	-1.512308
C	-4.127592	-0.596036	-1.095315
C	-4.000762	0.831004	-1.405181
C	-5.109518	1.685251	-1.227903
C	-2.784790	1.375501	-1.868758
C	-5.002279	3.045035	-1.493477
H	-6.041999	1.270930	-0.863272
C	-2.690498	2.737885	-2.137433
H	-1.912931	0.748016	-2.013270
C	-3.790176	3.576542	-1.944103
H	-5.859625	3.694550	-1.340669
H	-1.747975	3.152418	-2.478222
H	-3.695427	4.641532	-2.132495

O	-5.353186	-0.986300	-0.738270
Si	-6.391968	-2.284247	-1.173912
C	-6.783410	-2.065725	-2.999690
H	-5.880051	-2.145933	-3.615656
H	-7.483844	-2.838196	-3.340396
H	-7.238852	-1.089285	-3.198556
C	-5.622357	-3.970634	-0.852631
H	-6.380655	-4.747434	-1.016131
H	-4.784203	-4.190614	-1.522629
H	-5.272420	-4.064564	0.180663
C	-7.876620	-1.997448	-0.066545
H	-8.638850	-2.770328	-0.222103
H	-7.581926	-2.020959	0.988580
H	-8.340891	-1.024853	-0.263930

TS 133

B3LYP/6-31G(d) = -3573.928602
B3LYP-D3(BJ)/def2-TZVPP/IEFPCM(propanonitrile) = -3575.50293
B3LYP-D3(BJ)/def2-TZVPP/IEFPCM(propanonitrile)//B3LYP-D3(BJ)/6-31G(d) Free Energy (Quasiharmonic) = -3574.786874

Frequencies (Top 3 out of 300)

1. -285.6917 cm^{-1}
2. 10.4800 cm^{-1}
3. 11.6453 cm^{-1}

B3LYP/6-31G(d) Molecular Geometry in Cartesian Coordinates

B	-0.249016	-0.373187	-0.981865
O	-0.137896	0.650583	-2.029086
N	-1.748661	-0.814746	-1.124914
C	-2.353061	-0.116662	-2.273859
S	-2.259120	-2.352762	-0.779188
C	-1.199749	0.716127	-2.834925
O	-1.245901	1.373137	-3.851769
H	-4.379727	0.169387	-1.580721
H	-2.667950	-0.829629	-3.044678
C	-3.559709	0.804204	-1.932150
C	-3.285086	1.890587	-0.929317
C	-2.839107	3.242989	-1.187969
C	-2.715806	3.894223	0.071397
N	-3.071754	2.977795	1.039315
C	-3.410578	1.783044	0.434069
H	-3.724903	0.936399	1.028102
C	-2.029829	5.921335	-0.977904
C	-2.545943	3.973743	-2.354317
H	-2.213416	5.701802	1.166493
H	-3.069020	3.145936	2.032778
C	-2.149492	5.300034	-2.238148
H	-1.711999	6.959115	-0.919942
H	-2.601997	3.497140	-3.327738
C	-2.308666	5.226996	0.193113
H	-1.919755	5.869983	-3.134709
C	-3.810854	-2.182788	0.121606
O	-2.582291	-3.088140	-2.013905
O	-1.288200	-2.915101	0.176987
C	-6.227551	-1.956128	1.527763
C	-5.018963	-2.399505	-0.539423
C	-3.791385	-1.871339	1.484624
C	-4.996461	-1.757862	2.173379
C	-6.217493	-2.278700	0.165648
H	-5.013590	-2.672147	-1.589414
H	-2.848553	-1.733987	2.003800
H	-4.981955	-1.520224	3.234758
H	-7.159014	-2.445247	-0.352075
C	-7.523948	-1.854529	2.295818
H	-7.516206	-1.001838	2.983901
H	-7.696386	-2.755090	2.899759
H	-8.380126	-1.740360	1.623810
O	0.652201	-1.519931	-1.476381
C	1.522093	-2.212748	-0.801705
H	1.655058	-1.962836	0.252430
H	-3.887346	1.244043	-2.881791
C	1.640337	-3.654994	-1.144806
C	1.086001	-4.152874	-2.332389
C	2.284175	-4.534371	-0.262373
C	1.188504	-5.508772	-2.634087
H	0.549059	-3.477754	-2.989451
C	2.383136	-5.890011	-0.565298
H	2.687752	-4.158666	0.675398
C	1.840625	-6.378806	-1.756562
H	0.745451	-5.890374	-3.549581
H	2.872950	-6.566000	0.130079
H	1.914434	-7.436747	-1.993270
C	0.163964	0.221289	0.466249
C	0.616859	1.546805	0.565281
C	0.035555	-0.496205	1.666807
C	0.938626	2.123509	1.797333
H	0.699690	2.141635	-0.337489
C	0.339760	0.083337	2.903388
H	-0.334471	-1.516595	1.636410
C	0.801776	1.396446	2.980277
H	1.033169	1.847150	3.938066
C	0.200640	-0.747938	4.145902
C	1.495462	3.518212	1.835927
F	0.197300	-0.002122	5.272261
F	-0.941272	-1.476400	4.141511
F	0.918786	4.330139	0.930545
F	1.220823	-1.639313	4.270017
F	2.835633	3.524056	1.559125
F	1.358437	4.095181	3.051022
C	3.421094	-1.649759	-1.344167
H	3.355741	-2.000902	-2.365597
H	3.976563	-2.309185	-0.686477
C	3.576992	-0.270404	-1.141509
O	4.026826	0.209866	0.009880
Si	5.075099	-0.339916	1.269317
C	6.631337	-1.016790	0.455669
H	7.094440	-0.266519	-0.195262
H	7.366922	-1.286606	1.223749
H	6.441331	-1.912398	-0.145675
C	5.407424	1.236155	2.224133
H	4.478662	1.783370	2.414396
H	5.882948	1.021344	3.188452
H	6.072407	1.903391	1.664008
C	4.227924	-1.642252	2.325168
H	4.065912	-2.579670	1.781774
H	4.867788	-1.877296	3.185768
H	3.263498	-1.307877	2.720776
C	3.170340	0.746250	-2.109992
C	3.241245	2.112551	-1.761777
C	2.692277	0.396260	-3.389814
C	2.839383	3.089115	-2.663789
H	3.578154	2.393015	-0.771221
C	2.289539	1.378334	-4.285549
H	2.611884	-0.643709	-3.682488
C	2.359185	2.725060	-3.925418
H	2.881787	4.135816	-2.377545
H	1.896912	1.094200	-5.256323
H	2.027706	3.489330	-4.622147

TS 134

B3LYP/6-31G(d) = -3573.923819
B3LYP-D3(BJ)/def2-TZVPP/IEFPCM(propanonitrile) = -3575.502433
B3LYP-D3(BJ)/def2-TZVPP/IEFPCM(propanonitrile)//B3LYP-D3(BJ)/6-31G(d) Free Energy (Quasiharmonic) = -3574.78682

Frequencies (Top 3 out of 300)

1. -280.5881 cm^{-1}
2. 8.5430 cm^{-1}
3. 11.0814 cm^{-1}

B3LYP/6-31G(d) Molecular Geometry in Cartesian Coordinates

B	0.054994	0.218758	-0.413208
O	-0.244321	-0.515909	-1.660605
N	1.074936	1.308488	-0.910423
C	1.328618	1.103273	-2.347277
S	0.982292	2.858898	-0.333092
C	0.372612	-0.029973	-2.734267
O	0.213581	-0.444945	-3.863564
H	3.411778	1.628010	-2.592258
H	1.042545	1.984202	-2.934895
C	2.799173	0.727373	-2.702622
C	3.387820	-0.383285	-1.879331
C	3.273993	-1.811852	-2.079089
C	3.956228	-2.441389	-0.999430
N	4.483607	-1.441947	-0.205306
C	4.115073	-0.216866	-0.727323
H	4.413573	0.698824	-0.236172
C	3.410999	-4.604185	-1.842781
C	2.667487	-2.622140	-3.056695
H	4.551567	-4.291038	-0.030856
H	4.834660	-1.571548	0.732031
C	2.741608	-4.003881	-2.928640
H	3.445643	-5.687820	-1.767270
H	2.124831	-2.172692	-3.882753
C	4.030066	-3.831651	-0.866964
H	2.270116	-4.636390	-3.676463
C	2.654912	3.520150	-0.441631
O	0.154589	3.717924	-1.201372
O	0.662447	2.742909	1.101722
C	5.248143	4.580202	-0.588934
C	3.559617	3.283153	0.596304
C	3.021325	4.303285	-1.536163
C	4.314484	4.822092	-1.604848
C	4.845722	3.812654	0.514819
H	3.251000	2.712550	1.466002
H	2.292870	4.518591	-2.310706
H	4.598299	5.433932	-2.457768
H	5.546834	3.636331	1.327330
C	6.655202	5.120738	-0.681303
H	6.705024	6.000973	-1.330140
H	7.042551	5.401280	0.304004
H	7.339150	4.368728	-1.096711
O	-1.260599	0.870347	-0.033250
C	-1.803950	0.923958	1.159308
H	-1.168725	0.624006	1.997558
H	2.802100	0.470167	-3.769010
C	-2.674420	2.102626	1.428346
C	-3.153883	2.344544	2.724352
C	-2.984896	3.005612	0.402218
C	-3.945722	3.458493	2.989253
H	-2.895079	1.663776	3.532661
C	-3.774713	4.123899	0.670622
H	-2.558675	2.851616	-0.582416
C	-4.263979	4.349096	1.959151
H	-4.303528	3.640687	3.998925
H	-3.989003	4.832529	-0.124901
H	-4.873272	5.224832	2.166531
C	0.630381	-0.796382	0.708296
C	0.282696	-2.152049	0.658152
C	1.526874	-0.407542	1.716126
C	0.762050	-3.074650	1.594032
H	-0.360274	-2.499603	-0.143975
C	2.054605	-1.334251	2.618284
H	1.820084	0.633712	1.785937
C	1.661371	-2.675466	2.578130
H	2.051341	-3.389171	3.294467
C	3.132376	-0.915743	3.574701
C	0.277150	-4.493045	1.504795
F	3.086738	0.400114	3.868863
F	4.377167	-1.147011	3.051538
F	0.593238	-5.069203	0.326346
F	3.085717	-1.597714	4.739503
F	-1.084405	-4.547788	1.600116
F	0.769698	-5.277446	2.486520
C	-2.977880	-0.638482	1.432881
H	-2.219040	-1.413432	1.418978
H	-3.395423	-0.403182	2.406892
C	-3.866833	-0.603809	0.346990
O	-4.913761	0.187094	0.450924
Si	-6.547094	0.321301	-0.103732
C	-7.483618	0.682869	1.480352
H	-8.545812	0.865881	1.277246
H	-7.075850	1.572070	1.973995
H	-7.417495	-0.153177	2.185770
C	-6.622745	1.779884	-1.281222
H	-7.666318	2.075009	-1.447264
H	-6.093901	2.641728	-0.859363
H	-6.184062	1.555628	-2.259073
C	-7.089637	-1.295954	-0.889863
H	-6.550717	-1.511899	-1.817219
H	-6.947688	-2.144128	-0.210287
H	-8.159657	-1.242004	-1.127794
C	-3.547145	-1.245753	-0.931055
C	-2.988349	-2.537405	-0.959631
C	-3.729555	-0.546040	-2.139298
C	-2.628015	-3.114682	-2.173067
H	-2.846812	-3.091617	-0.036631
C	-3.336328	-1.117096	-3.344084
H	-4.109566	0.469708	-2.117506
C	-2.785591	-2.400853	-3.362917
H	-2.200182	-4.112532	-2.187489
H	-3.430088	-0.553035	-4.266751
H	-2.463747	-2.836452	-4.303655

TS 135

B3LYP/6-31G(d) = -3573.930186
B3LYP-D3(BJ)/def2-TZVPP/IEFPCM(propanonitrile) = -3575.503324
B3LYP-D3(BJ)/def2-TZVPP/IEFPCM(propanonitrile)//B3LYP-D3(BJ)/6-31G(d) Free Energy (Quasiharmonic) = -3574.786746

Frequencies (Top 3 out of 300)

1. -259.4728 cm^{-1}
2. 8.3944 cm^{-1}
3. 12.3267 cm^{-1}

B3LYP/6-31G(d) Molecular Geometry in Cartesian Coordinates

B	-0.220092	-0.656215	-0.365854
O	-0.489237	-1.941464	-1.035609
N	-0.546381	-1.032088	1.119202
C	-0.912734	-2.454935	1.235390
S	-0.192913	-0.159837	2.467951
C	-0.734647	-2.958273	-0.199013
O	-0.810186	-4.117721	-0.534250
H	-2.427109	-2.383344	2.771904
H	-0.199836	-2.980019	1.881921
C	-2.339139	-2.787280	1.758111
C	-3.492338	-2.335361	0.905621
C	-4.178195	-3.106625	-0.110366
C	-5.203852	-2.278948	-0.643294
N	-5.157748	-1.077306	0.036362
C	-4.118077	-1.112522	0.945249

H	-3.913517	-0.253980	1.567969
C	-5.895001	-4.000916	-2.138325
C	-4.034969	-4.412532	-0.614328
H	-6.828558	-2.049703	-2.064695
H	-5.627186	-0.234480	-0.259326
C	-4.893096	-4.845506	-1.616539
H	-6.543824	-4.366296	-2.930021
H	-3.242750	-5.058229	-0.249302
C	-6.063235	-2.707147	-1.660956
H	-4.784108	-5.849889	-2.016996
C	-1.692239	0.591845	3.121560
O	0.303906	-1.077173	3.506502
O	0.647304	0.974164	2.033163
C	-4.011471	1.773229	4.164304
C	-2.192269	1.760503	2.540415
C	-2.320214	0.022315	4.229667
C	-3.474719	0.614597	4.740703
C	-3.346666	2.337950	3.064602
H	-1.676512	2.222650	1.705808
H	-1.892980	-0.859049	4.695624
H	-3.960622	0.172506	5.607013
H	-3.732833	3.250818	2.617409
C	-5.278472	2.393248	4.703373
H	-5.274954	3.481625	4.583035
H	-6.160423	2.010105	4.173108
H	-5.414583	2.167715	5.765913
O	1.277273	-0.340194	-0.634275
C	2.204633	-1.232716	-0.435590
H	1.936259	-2.102207	0.171168
H	-2.366082	-3.878586	1.860414
C	3.155413	-1.511230	-1.534021
C	3.318507	-0.603878	-2.591717
C	3.853958	-2.727416	-1.552342
C	4.179700	-0.908878	-3.643036
H	2.748325	0.318572	-2.588226
C	4.710056	-3.032085	-2.609483
H	3.699024	-3.448140	-0.752748
C	4.878601	-2.119442	-3.653952
H	4.296081	-0.207051	-4.464136
H	5.229819	-3.986045	-2.627885
H	5.540024	-2.358200	-4.482332
C	-1.038319	0.578525	-0.996513
C	-0.560662	1.892574	-0.938288
C	-2.292180	0.376433	-1.598817
C	-1.308861	2.969044	-1.430264
H	0.411068	2.082942	-0.497476
C	-3.038485	1.446750	-2.098640
H	-2.684022	-0.631507	-1.684018
C	-2.553968	2.754914	-2.013404
H	-3.132537	3.585039	-2.401299
C	-4.406998	1.223466	-2.677263
C	-0.741736	4.353472	-1.307467
F	-4.585490	-0.027855	-3.136430
F	-4.679867	2.077230	-3.688012
F	-0.443931	4.652021	-0.011962
F	-5.387027	1.435276	-1.739182
F	-1.586623	5.308883	-1.747939
F	0.416689	4.494227	-1.997300
C	3.366872	-0.536387	1.166855
H	2.529688	-0.022177	1.622234
H	3.640297	-1.485021	1.616293
C	4.394884	0.211114	0.595157
O	5.568228	-0.350326	0.312973
Si	6.702064	-1.314927	1.179366
C	7.224562	-0.304380	2.674923
H	7.985124	-0.840184	3.256052
H	6.377688	-0.108437	3.342841
H	7.648447	0.661310	2.378024
C	5.994857	-2.977889	1.702584
H	6.806136	-3.598667	2.104640
H	5.235480	-2.893436	2.487410
H	5.558141	-3.518278	0.856453
C	8.073275	-1.535366	-0.078721
H	8.507371	-0.572495	-0.369902
H	8.880370	-2.158950	0.323973
H	7.693170	-2.020122	-0.984993
C	4.244012	1.587368	0.111761
C	5.239788	2.150684	-0.713343
C	3.117701	2.364637	0.452794
C	5.107507	3.449646	-1.190020
H	6.101277	1.552629	-0.985713
C	2.994806	3.665848	-0.027376
H	2.343991	1.963021	1.095995
C	3.984226	4.209577	-0.849929
H	5.876893	3.870105	-1.831493
H	2.118839	4.250648	0.232423
H	3.879038	5.223479	-1.226038

TS 136

B3LYP/6-31G(d) = -3573.928895
B3LYP-D3(BJ)/def2-TZVPP/IEFPCM(propanonitrile) = -3575.503286
B3LYP-D3(BJ)/def2-TZVPP/IEFPCM(propanonitrile)//B3LYP-D3(BJ)/6-31G(d) Free Energy (Quasiharmonic) = -3574.786712

Frequencies (Top 3 out of 300)

1. -288.6162 cm^{-1}
2. 6.3333 cm^{-1}
3. 12.5712 cm^{-1}

B3LYP/6-31G(d) Molecular Geometry in Cartesian Coordinates

B	-0.085878	0.695328	-0.416560
O	-0.650243	0.521267	-1.780257
N	1.398084	1.127247	-0.741217
C	1.584236	1.158207	-2.204431
S	2.110223	2.351333	0.140240
C	0.202556	0.820086	-2.761571
O	-0.097766	0.797279	-3.938973
H	3.607381	0.402869	-2.332155
H	1.839224	2.168817	-2.548239
C	2.636825	0.165308	-2.778210
C	2.320838	-1.295349	-2.593593
C	2.883984	-2.207935	-1.626064
C	2.263443	-3.471968	-1.825354
N	1.388483	-3.334303	-2.882454
C	1.418455	-2.027913	-3.330389
H	0.795807	-1.713278	-4.156242
C	3.528863	-4.439099	-0.052294
C	3.860050	-2.090775	-0.621220
H	2.062769	-5.540970	-1.201001
H	0.714065	-4.027654	-3.165623
C	4.173901	-3.201434	0.151255
H	3.789062	-5.287784	0.574694
H	4.359037	-1.142853	-0.446946
C	2.566580	-4.591199	-1.042870
H	4.925276	-3.117076	0.932203
C	3.880167	2.032812	0.099593
O	1.684300	2.175905	1.537862
O	1.911599	3.651867	-0.528289
C	6.650180	1.608181	0.118314
C	4.438567	1.164347	1.040606
C	4.688163	2.708562	-0.814798
C	6.065368	2.486042	-0.803405
C	5.816387	0.960650	1.043349
H	3.800549	0.671989	1.766679
H	4.243710	3.416679	-1.506147
H	6.696349	3.014012	-1.514290
H	6.253776	0.291651	1.780776
C	8.138640	1.353296	0.113929

H	8.681798	2.155488	-0.395443
H	8.375435	0.414756	-0.404400
H	8.533204	1.267915	1.132298
H	2.714641	0.398555	-3.847283
C	-0.231524	-0.640569	0.471332
C	0.125226	-0.679550	1.829528
C	-0.727227	-1.820384	-0.093481
C	-0.011113	-1.848388	2.581613
H	0.527482	0.213973	2.296605
C	-0.876202	-2.990528	0.658776
H	-1.006133	-1.827529	-1.141572
C	-0.517375	-3.014823	2.003568
H	-0.623332	-3.919713	2.589403
C	-1.483409	-4.194479	0.003786
C	0.321382	-1.830003	4.047153
F	-0.810463	-4.581557	-1.111060
F	-2.762387	-3.943742	-0.411027
F	-1.545737	-5.266205	0.819120
F	-0.714350	-1.357349	4.793120
F	1.381000	-1.039463	4.320958
F	0.602815	-3.064729	4.524789
O	-0.856354	1.817329	0.271432
C	-1.565345	2.715150	-0.362172
C	-1.640846	4.059705	0.261844
C	-1.119135	4.278089	1.545779
C	-2.207814	5.130597	-0.447538
C	-1.184059	5.550036	2.113376
H	-0.624389	3.461715	2.059634
C	-2.267983	6.398255	0.123020
H	-2.595230	4.969011	-1.451248
C	-1.761914	6.608617	1.409747
H	-0.766396	5.717838	3.102139
H	-2.701217	7.223699	-0.434987
H	-1.806134	7.598988	1.854754
H	-1.514788	2.698801	-1.451209
C	-3.566864	2.239870	-0.398222
H	-3.918496	2.901682	0.380989
H	-3.801019	2.574921	-1.401995
C	-3.692454	0.862518	-0.146338
C	-3.695517	0.309854	1.212199
C	-3.923193	-1.066744	1.410951
C	-3.464328	1.129756	2.336180
C	-3.936035	-1.599759	2.694421
H	-4.062392	-1.713859	0.553878
C	-3.465644	0.587062	3.615769
H	-3.254880	2.186313	2.214664
C	-3.704677	-0.777005	3.799735
H	-4.109062	-2.662861	2.832639
H	-3.268204	1.225867	4.471242
H	-3.695095	-1.199298	4.800063
O	-3.716869	-0.031619	-1.108014
Si	-4.130931	-0.171269	-2.790320
C	-3.639664	1.307143	-3.836589
H	-2.552971	1.442991	-3.842899
H	-3.939899	1.092103	-4.871419
H	-4.130762	2.244027	-3.552736
C	-3.203124	-1.705504	-3.324515
H	-2.146271	-1.460053	-3.473838
H	-3.272652	-2.504435	-2.578939
H	-3.599713	-2.086679	-4.273551
C	-5.998262	-0.408131	-2.763284
H	-6.281954	-1.278945	-2.161890
H	-6.379852	-0.564903	-3.779707
H	-6.511679	0.468023	-2.349872

TS 137

B3LYP/6-31G(d) = -3573.930185
B3LYP-D3(BJ)/def2-TZVPP/IEFPCM(propanonitrile) = -3575.503241
B3LYP-D3(BJ)/def2-TZVPP/IEFPCM(propanonitrile)//B3LYP-D3(BJ)/6-31G(d) Free Energy (Quasiharmonic) = -3574.786708

Frequencies (Top 3 out of 300)

1. -264.8927 cm^{-1}
2. 8.5658 cm^{-1}
3. 11.5855 cm^{-1}

B3LYP/6-31G(d) Molecular Geometry in Cartesian Coordinates

B	-0.219670	-0.647726	-0.389345
O	-0.489119	-1.921045	-1.081366
N	-0.537809	-1.052360	1.090426
C	-0.897892	-2.478530	1.182088
S	-0.181582	-0.203358	2.452865
C	-0.726689	-2.954016	-0.262593
O	-0.800026	-4.107309	-0.619064
H	-2.400550	-2.444496	2.730910
H	-0.178617	-3.012896	1.813794
C	-2.319085	-2.827061	1.708308
C	-3.480940	-2.362262	0.874760
C	-4.169432	-3.112991	-0.154791
C	-5.204675	-2.279432	-0.659401
N	-5.161098	-1.094613	0.049208
C	-4.114655	-1.145146	0.949400
H	-3.912130	-0.301280	1.592436
C	-5.896444	-3.968947	-2.190745
C	-4.021845	-4.405283	-0.691631
H	-6.842058	-2.026433	-2.062106
H	-5.639988	-0.248347	-0.220193
C	-4.885050	-4.819333	-1.697471
H	-6.549146	-4.319217	-2.986076
H	-3.222719	-5.054481	-0.348579
C	-6.069247	-2.688376	-1.680588
H	-4.772776	-5.812937	-2.123125
C	-1.678065	0.539699	3.122525
O	0.658883	0.937288	2.035865
O	0.316235	-1.138355	3.475097
C	-3.997504	1.702935	4.184574
C	-2.306177	-0.048726	4.220511
C	-2.180991	1.715228	2.557871
C	-3.336735	2.282310	3.089975
C	-3.462462	0.533248	4.739619
H	-1.882057	-0.940947	4.668283
H	-1.670550	2.186209	1.724967
H	-3.729500	3.196346	2.650896
H	-3.952925	0.072223	5.593414
C	-5.231838	2.346322	4.770124
H	-4.962475	3.118203	5.503276
H	-5.860832	1.612752	5.284356
H	-5.836416	2.831625	3.996703
O	1.273458	-0.319853	-0.659792
C	2.207772	-1.214304	-0.500877
H	1.944528	-2.113180	0.063754
H	-2.340936	-3.920369	1.788314
C	3.153818	-1.438639	-1.616670
C	3.307718	-0.483227	-2.632466
C	3.855925	-2.650255	-1.694934
C	4.164080	-0.736534	-3.701437
H	2.734501	0.436012	-2.583906
C	4.707039	-2.902868	-2.769583
H	3.708202	-3.407176	-0.928302
C	4.866900	-1.942708	-3.771773
H	4.273520	0.002828	-4.489916
H	5.230408	-3.852835	-2.834415
H	5.524856	-2.140613	-4.613583
C	-1.044674	0.597339	-0.990927
C	-0.560914	1.908695	-0.922986
C	-2.308340	0.408179	-1.576643
C	-1.311611	2.994542	-1.389597

H	0.418566	2.089291	-0.495505
C	-3.057708	1.488078	-2.050876
H	-2.705651	-0.596950	-1.669688
C	-2.566479	2.793109	-1.956142
H	-3.147200	3.630478	-2.324711
C	-4.435874	1.277258	-2.611125
C	-0.735660	4.374492	-1.257894
F	-4.622421	0.035692	-3.093274
F	-5.399978	1.469308	-1.652750
F	-0.423929	4.658154	0.037669
F	-4.725045	2.151762	-3.599368
F	-1.579033	5.339081	-1.680757
F	0.417303	4.515394	-1.956828
C	3.360003	-0.579124	1.123444
H	3.623072	-1.539800	1.554060
H	2.523829	-0.070091	1.586650
C	4.402653	0.177623	0.589146
C	4.265315	1.564184	0.133476
C	5.282044	2.144310	-0.653764
C	3.130933	2.334856	0.463193
C	5.162079	3.453277	-1.105345
H	6.150245	1.551661	-0.916488
C	3.020369	3.646165	0.007960
H	2.342442	1.920721	1.080001
C	4.030490	4.206450	-0.777510
H	5.947614	3.887120	-1.717649
H	2.138709	4.226337	0.258970
H	3.935400	5.228444	-1.133945
O	5.581984	-0.379435	0.323723
Si	6.662017	-1.383502	1.218259
C	8.189818	-1.383692	0.132695
H	8.980885	-2.005619	0.568501
H	7.961179	-1.781519	-0.862479
H	8.590757	-0.371964	0.006368
C	6.933133	-0.539342	2.874774
H	7.334120	0.472358	2.747370
H	7.646436	-1.108048	3.483803
H	6.000980	-0.461804	3.446103
C	6.001560	-3.130454	1.439656
H	6.789082	-3.757670	1.877230
H	5.137905	-3.183058	2.111305
H	5.719685	-3.577819	0.481085

TS 138

B3LYP/6-31G(d) = -3573.926341
B3LYP-D3(BJ)/def2-TZVPP/IEFPCM(propanonitrile) = -3575.502534
B3LYP-D3(BJ)/def2-TZVPP/IEFPCM(propanonitrile)//B3LYP-D3(BJ)/6-31G(d) Free Energy (Quasiharmonic) = -3574.786694

Frequencies (Top 3 out of 300)

1. -247.1187 cm^{-1}
2. 7.5812 cm^{-1}
3. 9.8930 cm^{-1}

B3LYP/6-31G(d) Molecular Geometry in Cartesian Coordinates

B	0.462023	-0.653723	-0.221444
O	0.379728	0.614592	-0.998552
N	-0.484863	-1.565838	-1.055637
C	-0.846132	-0.909536	-2.323284
S	-0.407493	-3.230985	-1.013387
C	-0.255661	0.490413	-2.164798
O	-0.338115	1.388549	-2.978729
H	-2.707727	-1.877475	-2.794234
H	-0.337024	-1.399922	-3.163625
C	-2.352938	-0.847548	-2.685136
C	-3.254905	-0.085313	-1.752768
C	-3.725012	1.274306	-1.912123
C	-4.620172	1.542195	-0.838357
N	-4.678149	0.400088	-0.066012
C	-3.863471	-0.569688	-0.619369
H	-3.795721	-1.547532	-0.165717
C	-5.043469	3.738387	-1.664810
C	-3.501407	2.280287	-2.869689
H	-5.966267	2.950081	0.126179
H	-5.219760	0.285727	0.776120
C	-4.163500	3.494868	-2.739839
H	-5.547576	4.698443	-1.591867
H	-2.804428	2.112814	-3.684724
C	-5.281315	2.767864	-0.698562
H	-4.003183	4.274003	-3.480629
C	-2.048037	-3.802327	-0.529778
O	0.482007	-3.597057	0.093531
O	-0.180185	-3.737033	-2.376470
C	-4.564665	-4.759195	0.257062
C	-2.880148	-4.411299	-1.467958
C	-2.449791	-3.678306	0.802501
C	-3.703270	-4.150793	1.184323
C	-4.133351	-4.879352	-1.069986
H	-2.538091	-4.526262	-2.491069
H	-1.784878	-3.220353	1.527263
H	-4.016344	-4.053648	2.221502
H	-4.782364	-5.352562	-1.802971
C	-5.905891	-5.303133	0.689082
H	-6.327933	-4.722608	1.516380
H	-5.816283	-6.341701	1.034281
H	-6.626474	-5.295620	-0.135147
H	-2.403859	-0.398807	-3.683746
C	2.003139	-1.096791	-0.042396
C	2.797518	-1.320198	-1.176173
C	2.632786	-1.198782	1.203791
C	4.155891	-1.628353	-1.073732
H	2.354693	-1.263829	-2.167037
C	3.991050	-1.508747	1.315917
H	2.053087	-1.044677	2.107124
C	4.765365	-1.724584	0.176658
H	5.817765	-1.968772	0.260517
C	4.645039	-1.514101	2.667854
C	4.981520	-1.757310	-2.322681
F	5.729467	-2.318010	2.713166
F	3.801982	-1.912980	3.646093
F	5.072740	-0.268122	3.015104
F	4.301899	-2.346071	-3.328163
F	6.112548	-2.468325	-2.121420
F	5.367453	-0.534186	-2.780939
O	-0.119401	-0.421447	1.172367
C	-0.923757	0.545301	1.486905
C	-1.818764	0.320987	2.648503
C	-2.938557	1.148330	2.830356
C	-1.575814	-0.723013	3.552818
C	-3.802081	0.933386	3.902481
H	-3.129356	1.955047	2.126547
C	-2.442276	-0.934252	4.624296
H	-0.713485	-1.364279	3.402167
C	-3.554733	-0.107797	4.802635
H	-4.666149	1.578125	4.039668
H	-2.248688	-1.744313	5.321893
H	-4.226797	-0.273087	5.640268
H	-1.319294	1.162823	0.680719
C	0.257552	2.097138	2.342489
H	0.936771	1.449018	2.886926
H	-0.543802	2.553066	2.908768
C	0.769203	2.831597	1.273361
C	-0.017899	3.932463	0.669071
C	-0.227471	3.984959	-0.720305
C	-0.595893	4.913905	1.493950
C	-1.004128	5.005583	-1.265809
H	0.157439	3.201207	-1.364534
C	-1.347037	5.945871	0.936635

H	-0.425714	4.884169	2.566439
C	-1.555039	5.991007	-0.444019
H	-1.191434	5.015725	-2.334719
H	-1.770794	6.712661	1.579078
H	-2.153050	6.788103	-0.876919
O	1.916536	2.471162	0.750585
Si	3.239314	3.182446	-0.130267
C	4.723204	2.701479	0.912347
H	4.746106	3.264422	1.852955
H	4.718316	1.636213	1.164207
H	5.655013	2.916342	0.374333
C	3.055558	5.056051	-0.172003
H	4.003311	5.487540	-0.520075
H	2.265706	5.393527	-0.848870
H	2.852489	5.473846	0.820617
C	3.283450	2.441865	-1.849669
H	3.685371	1.424214	-1.843830
H	2.294385	2.409371	-2.317448
H	3.938962	3.051523	-2.485282

TS 139

B3LYP/6-31G(d) = -3573.926432
B3LYP-D3(BJ)/def2-TZVPP/IEFPCM(propanonitrile) = -3575.503651
B3LYP-D3(BJ)/def2-TZVPP/IEFPCM(propanonitrile)//B3LYP-D3(BJ)/6-31G(d) Free Energy (Quasiharmonic) = -3574.78663

Frequencies (Top 3 out of 300)

1. -265.7266 cm^{-1}
2. 10.0634 cm^{-1}
3. 14.5620 cm^{-1}

B3LYP/6-31G(d) Molecular Geometry in Cartesian Coordinates

B	0.314825	-0.413946	-0.390327
O	-0.581698	0.554199	-1.077090
N	0.177379	-1.643395	-1.322437
C	-0.603776	-1.318819	-2.527921
S	0.806772	-3.175972	-1.163711
C	-1.033441	0.122952	-2.259293
O	-1.704457	0.810376	-3.002006
H	-1.451727	-3.210580	-3.071280
H	0.037933	-1.329347	-3.420053
C	-1.832743	-2.207337	-2.862483
C	-2.944286	-2.265705	-1.850495
C	-4.203383	-1.555346	-1.896102
C	-4.982536	-2.004046	-0.791627
N	-4.220756	-2.932584	-0.112137
C	-3.004564	-3.087705	-0.748940
H	-2.258995	-3.773764	-0.370885
C	-6.802077	-0.602043	-1.435131
C	-4.760775	-0.607049	-2.772742
H	-6.859474	-1.895313	0.298348
H	-4.493218	-3.417005	0.728656
C	-6.050413	-0.144971	-2.537646
H	-7.809259	-0.224336	-1.279409
H	-4.181339	-0.231226	-3.610684
C	-6.277825	-1.534435	-0.546732
H	-6.492444	0.579300	-3.217382
C	2.448305	-2.962432	-0.473006
O	0.942179	-3.722679	-2.524808
O	0.046120	-3.947391	-0.161923
C	5.003225	-2.605587	0.614329
C	3.499538	-2.563643	-1.299721
C	2.664269	-3.227643	0.879296
C	3.939527	-3.048932	1.412437
C	4.766280	-2.383727	-0.750463
H	3.327061	-2.392187	-2.357001
H	1.840638	-3.577338	1.491821
H	4.110613	-3.249408	2.467536
H	5.576923	-2.044314	-1.389731
C	6.358275	-2.329477	1.220235
H	6.580007	-3.018056	2.042582
H	6.394185	-1.310547	1.627999
H	7.157501	-2.415228	0.477103
H	-2.225952	-1.819348	-3.809228
C	1.796972	0.225384	-0.238587
C	2.419033	0.461992	0.993813
C	2.505189	0.602286	-1.389202
C	3.690434	1.036627	1.075239
H	1.908576	0.181124	1.907976
C	3.782407	1.164022	-1.318500
H	2.063451	0.441908	-2.369254
C	4.386023	1.387139	-0.081430
H	5.378099	1.819961	-0.022002
C	4.550165	1.421509	-2.584283
C	4.299832	1.338974	2.413829
F	5.153538	0.289169	-3.032894
F	5.525748	2.341560	-2.415599
F	3.751637	1.854732	-3.584294
F	3.792607	0.567226	3.399235
F	4.091209	2.629711	2.781259
F	5.644447	1.154343	2.411555
O	-0.194847	-0.706189	1.010496
C	-1.428625	-0.572415	1.405161
C	-1.882317	-1.463838	2.503389
C	-3.251136	-1.554401	2.804826
C	-0.964065	-2.236880	3.225945
C	-3.692052	-2.402900	3.817269
H	-3.965760	-0.961246	2.239006
C	-1.409285	-3.086396	4.237712
H	0.088727	-2.179065	2.973370
C	-2.770476	-3.170001	4.537738
H	-4.752290	-2.466857	4.047359
H	-0.693128	-3.689258	4.788820
H	-3.114390	-3.832696	5.327093
H	-2.174813	-0.325546	0.649743
C	-1.528440	1.238246	2.437334
H	-0.562207	1.153011	2.924352
H	-2.398046	1.016671	3.042170
C	-1.683579	2.226396	1.463204
C	-3.034858	2.607612	0.987788
C	-3.357245	2.563366	-0.380329
C	-4.022367	2.968180	1.922803
C	-4.649939	2.880109	-0.795972
H	-2.625825	2.228256	-1.108201
C	-5.301801	3.312034	1.493725
H	-3.773915	3.006881	2.979678
C	-5.617883	3.264384	0.133585
H	-4.901700	2.802439	-1.848527
H	-6.052327	3.611094	2.219985
H	-6.621018	3.515910	-0.199230
O	-0.609914	2.739153	0.912030
Si	-0.178740	4.234050	0.123779
C	1.688624	4.224085	0.247011
H	2.038387	4.011565	1.262775
H	2.119971	3.467411	-0.416197
H	2.092248	5.199510	-0.052652
C	-0.948743	5.607760	1.154789
H	-0.666120	6.584519	0.742554
H	-2.042793	5.555844	1.159716
H	-0.599768	5.574266	2.193297
C	-0.746909	4.282078	-1.663093
H	-0.137522	5.013523	-2.209702
H	-0.628869	3.311082	-2.155334
H	-1.795006	4.581954	-1.758577

TS 140

B3LYP/6-31G(d) = -3573.933077
B3LYP-D3(BJ)/def2-TZVPP/IEFPCM(propanonitrile) = -3575.502958
B3LYP-D3(BJ)/def2-TZVPP/IEFPCM(propanonitrile)//B3LYP-D3(BJ)/6-31G(d) Free Energy (Quasiharmonic) = -3574.7866

Frequencies (Top 3 out of 300)

1. -266.7539 cm^{-1}
2. 9.1290 cm^{-1}
3. 11.4355 cm^{-1}

B3LYP/6-31G(d) Molecular Geometry in Cartesian Coordinates

B	0.206299	-0.395348	1.098532
O	0.593003	0.147564	2.419903
N	0.773902	0.716968	0.153341
C	1.587469	1.684680	0.916586
S	1.050057	0.530640	-1.472042
C	1.332105	1.258806	2.369548
O	1.715267	1.859730	3.348525
H	0.385042	3.438448	1.366069
H	2.658452	1.550825	0.708702
C	1.227847	3.185658	0.709647
C	2.381231	4.120339	0.952465
C	3.199083	4.740679	-0.063821
C	4.179401	5.526746	0.604835
N	3.958332	5.380547	1.959036
C	2.886945	4.531406	2.161441
H	2.576582	4.251275	3.157306
C	5.118089	6.218180	-1.475763
C	3.201849	4.707145	-1.471078
H	5.881055	6.867164	0.442041
H	4.512731	5.796537	2.690697
C	4.159394	5.442998	-2.160077
H	5.853032	6.783371	-2.042830
H	2.472141	4.107220	-2.008647
C	5.140883	6.271015	-0.086253
H	4.171338	5.422041	-3.246668
C	2.663265	-0.219830	-1.698127
O	1.112513	1.863323	-2.092616
O	0.037057	-0.434476	-1.939842
C	5.189035	-1.412104	-1.903780
C	3.808311	0.580828	-1.664941
C	2.761819	-1.603077	-1.872686
C	4.020524	-2.185289	-1.977832
C	5.061539	-0.024816	-1.762428
H	3.725795	1.659834	-1.583867
H	1.863375	-2.207056	-1.923313
H	4.098934	-3.262292	-2.098330
H	5.953633	0.595259	-1.726608
C	6.543125	-2.076745	-1.955588
H	6.653205	-2.693028	-2.855988
H	7.354125	-1.342363	-1.946763
H	6.675020	-2.739576	-1.091539
H	0.882027	3.299791	-0.318900
C	0.729500	-1.911505	0.927245
C	-0.036322	-2.921297	0.334761
C	2.007888	-2.267579	1.389720
C	0.454422	-4.224263	0.187848
C	2.504664	-3.563522	1.246329
H	2.628139	-1.523578	1.879178
C	1.729235	-4.554620	0.639922
H	2.107801	-5.564966	0.535718
C	3.904515	-3.903792	1.677620
C	-0.393788	-5.247226	-0.512015
F	3.973476	-5.133080	2.239036
F	4.401593	-3.021559	2.566991
F	4.762159	-3.922395	0.619924
F	-1.678502	-5.221223	-0.084989
F	0.061614	-6.506491	-0.345396
F	-0.439165	-5.022038	-1.855484
O	-1.327730	-0.453728	1.059743
C	-2.044139	0.628092	1.191567
C	-3.274470	0.540827	2.008642
C	-3.822303	1.709186	2.558301
C	-3.865312	-0.699488	2.293397
C	-4.948426	1.642039	3.376902
H	-3.347261	2.667830	2.364359
C	-4.994208	-0.762164	3.107125
H	-3.418269	-1.602630	1.893087
C	-5.539438	0.405563	3.648054
H	-5.354987	2.549586	3.814722
H	-5.443063	-1.725870	3.330875
H	-6.413962	0.350720	4.290605
H	-1.497512	1.570925	1.268480
C	-2.622797	1.182213	-0.729847
H	-1.756471	0.720469	-1.185606
H	-2.579140	2.262794	-0.637389
C	-3.879945	0.617699	-0.948529
C	-4.099208	-0.790793	-1.287657
C	-5.394484	-1.339764	-1.181400
C	-3.038587	-1.614032	-1.721089
C	-5.619672	-2.676167	-1.488912
H	-6.208781	-0.710584	-0.842001
C	-3.274953	-2.951453	-2.029445
H	-2.037385	-1.214513	-1.836967
C	-4.560406	-3.485334	-1.911374
H	-6.619538	-3.090601	-1.395729
H	-2.451255	-3.578996	-2.352366
H	-4.736172	-4.530829	-2.149251
O	-4.990244	1.314994	-0.720516
Si	-5.503621	2.899940	-1.175874
C	-5.079965	3.111150	-2.993540
H	-5.421819	4.089009	-3.354214
H	-4.000085	3.054312	-3.170830
H	-5.561831	2.342085	-3.607600
C	-4.700047	4.229968	-0.117022
H	-5.150956	5.201586	-0.356886
H	-4.864055	4.044731	0.949474
H	-3.622118	4.324400	-0.287526
H	-1.036874	-2.692334	-0.013518
C	-7.349398	2.825819	-0.857869
H	-7.830151	2.057834	-1.473798
H	-7.558092	2.594167	0.192712
H	-7.825120	3.786508	-1.088877

TS 141

B3LYP/6-31G(d) = -3573.927368
B3LYP-D3(BJ)/def2-TZVPP/IEFPCM(propanonitrile) = -3575.503964
B3LYP-D3(BJ)/def2-TZVPP/IEFPCM(propanonitrile)//B3LYP-D3(BJ)/6-31G(d) Free Energy (Quasiharmonic) = -3574.786536

Frequencies (Top 3 out of 300)

1. -245.3298 cm^{-1}
2. 9.1131 cm^{-1}
3. 17.7746 cm^{-1}

B3LYP/6-31G(d) Molecular Geometry in Cartesian Coordinates

B	0.656579	-0.587383	-0.898159
O	0.801224	-0.965937	-2.321361
N	1.331830	-1.782355	-0.185220
C	1.645448	-2.842635	-1.157213
S	2.012898	-1.840494	1.305006
C	1.347021	-2.172771	-2.502852
O	1.541195	-2.679420	-3.584523
H	1.092341	-4.615765	-0.061755
H	2.715607	-3.092809	-1.136555
C	0.838974	-4.162733	-1.023817

C	-0.652178	-4.011347	-1.154004
C	-1.628685	-3.981867	-0.089926
C	-2.903678	-3.803711	-0.695227
N	-2.706944	-3.750281	-2.060670
C	-1.354490	-3.854710	-2.325842
H	-0.987352	-3.814344	-3.342235
C	-3.975876	-3.811525	1.434140
C	-1.552600	-4.079095	1.311334
H	-5.045254	-3.569717	-0.433581
H	-3.398390	-3.434119	-2.723423
C	-2.722834	-3.991809	2.056144
H	-4.871258	-3.739818	2.045751
H	-0.590871	-4.190652	1.803213
C	-4.083261	-3.716249	0.051120
H	-2.673125	-4.053918	3.139853
C	3.798280	-1.700660	1.072153
O	1.803135	-3.168360	1.900800
O	1.571754	-0.632679	2.027019
C	6.576223	-1.492283	0.723511
C	4.375500	-0.452056	0.826106
C	4.595035	-2.842613	1.155737
C	5.975028	-2.730446	0.978351
C	5.754208	-0.356006	0.657869
H	3.746158	0.428997	0.776809
H	4.136758	-3.799353	1.380991
H	6.593845	-3.621733	1.048552
H	6.204095	0.618682	0.481501
C	8.066786	-1.380671	0.508071
H	8.313686	-1.408892	-0.561635
H	8.460770	-0.439689	0.906956
H	8.604007	-2.204827	0.987921
O	1.564116	0.669617	-0.743421
C	1.268439	1.764663	-1.371974
H	0.224355	1.911998	-1.661744
H	1.224263	-4.824499	-1.809138
C	2.276696	2.351747	-2.285044
C	3.608345	1.912321	-2.270658
C	1.877317	3.317671	-3.223674
C	4.527379	2.446391	-3.171255
H	3.904446	1.137063	-1.572679
C	2.797633	3.845917	-4.124119
H	0.838813	3.640009	-3.254151
C	4.127328	3.414966	-4.095242
H	5.555947	2.096944	-3.161745
H	2.478749	4.583577	-4.854987
H	4.845752	3.823918	-4.800205
C	-0.900412	-0.308374	-0.553556
C	-1.830392	-0.179372	-1.597496
C	-1.406084	-0.305882	0.757140
C	-3.201729	-0.059229	-1.349340
H	-1.480341	-0.211784	-2.624078
C	-2.774961	-0.184910	1.008595
H	-0.722505	-0.433465	1.588427
C	-3.683837	-0.060603	-0.042478
H	-4.747422	0.010233	0.153677
C	-3.282854	-0.164787	2.422127
C	-4.173697	-0.038878	-2.492521
F	-3.310654	1.113227	2.929806
F	-4.547855	-0.624816	2.523567
F	-5.300005	0.649165	-2.196449
F	-2.514873	-0.880079	3.263499
F	-4.580889	-1.296640	-2.837641
F	-3.646653	0.508279	-3.608697
C	1.287992	3.352887	0.070607
H	1.441850	4.144419	-0.647942
H	2.174045	3.013155	0.592762
C	0.067290	3.294141	0.739455
O	-0.075932	2.588686	1.848088
Si	0.698900	2.647094	3.405167
C	0.254741	4.349096	4.080732
H	0.676053	4.482504	5.084859
H	-0.830688	4.479027	4.154945
H	0.648396	5.155026	3.449772
C	-0.084263	1.256371	4.369480
H	0.217379	1.316014	5.422881
H	-1.176333	1.292727	4.326203
H	0.244437	0.289157	3.976560
C	2.560507	2.441820	3.270947
H	2.984416	2.417755	4.283552
H	2.801860	1.488418	2.791165
H	3.053657	3.259007	2.733234
C	-1.171749	3.896685	0.208636
C	-2.411606	3.535709	0.767869
C	-1.151675	4.834984	-0.843832
C	-3.593552	4.086160	0.279581
H	-2.442062	2.806114	1.566026
C	-2.334910	5.387511	-1.321677
H	-0.211290	5.155600	-1.279499
C	-3.560832	5.012434	-0.763943
H	-4.542277	3.785588	0.714241
H	-2.302095	6.115724	-2.126960
H	-4.484140	5.442675	-1.141610

TS 142

B3LYP/6-31G(d) = -3573.928319
B3LYP-D3(BJ)/def2-TZVPP/IEFPCM(propanonitrile) = -3575.503076
B3LYP-D3(BJ)/def2-TZVPP/IEFPCM(propanonitrile)//B3LYP-D3(BJ)/6-31G(d) Free Energy (Quasiharmonic) = -3574.786525

Frequencies (Top 3 out of 300)

1. -249.4505 cm^{-1}
2. 8.4855 cm^{-1}
3. 9.3112 cm^{-1}

B3LYP/6-31G(d) Molecular Geometry in Cartesian Coordinates

B	0.034770	1.027548	-0.282543
O	0.320391	2.208921	-1.125490
N	0.941952	1.327000	0.961439
C	1.521866	2.682203	0.855179
S	0.737534	0.654004	2.452351
C	0.997748	3.170540	-0.498485
O	1.185080	4.283153	-0.938228
H	3.379907	2.550911	1.951522
H	1.107017	3.336478	1.631844
C	3.070858	2.814142	0.934062
C	3.887507	2.030727	-0.057956
C	4.240269	2.387668	-1.418421
C	5.037305	1.328212	-1.934446
N	5.190745	0.397086	-0.926509
C	4.486543	0.818122	0.185046
H	4.490828	0.223931	1.086702
C	5.273723	2.442057	-4.027347
C	3.986677	3.499694	-2.243720
H	6.145775	0.505685	-3.609701
H	5.542541	-0.538953	-1.056871
C	4.504725	3.515024	-3.532284
H	5.656462	2.481095	-5.043885
H	3.371558	4.318824	-1.887601
C	5.551287	1.335120	-3.235324
H	4.306183	4.367486	-4.176403
C	2.280629	-0.116770	2.963342
O	-0.228813	-0.450435	2.289879
O	0.452109	1.710953	3.442034
C	4.643692	-1.362955	3.813138
C	3.049705	0.473780	3.965738
C	2.670026	-1.327646	2.385310
C	3.847546	-1.937669	2.809433
C	4.226908	-0.150963	4.379348

H	2.719806	1.402555	4.418592
H	2.069609	-1.782369	1.605584
H	4.154571	-2.872705	2.347432
H	4.828270	0.310622	5.158697
C	5.901369	-2.050242	4.288693
H	6.614263	-1.335209	4.711335
H	6.398203	-2.586348	3.473275
H	5.674307	-2.787304	5.070374
H	3.268291	3.887144	0.819158
C	0.288581	-0.362707	-1.054455
C	1.570010	-0.923706	-1.136176
C	-0.746059	-1.049318	-1.702321
C	1.802211	-2.132203	-1.801181
H	2.399750	-0.409802	-0.661350
C	-0.521330	-2.252725	-2.376754
H	-1.751710	-0.646529	-1.665500
C	0.756100	-2.810611	-2.427475
H	0.931047	-3.749732	-2.938001
C	-1.696020	-2.964355	-2.981709
C	3.198878	-2.683723	-1.837820
F	-2.469301	-2.137895	-3.721010
F	-1.337763	-3.999356	-3.767561
F	-2.524053	-3.477571	-2.017189
F	3.242187	-3.968715	-2.248111
F	3.787196	-2.639828	-0.607172
F	4.015191	-1.978568	-2.659477
O	-1.480298	1.103174	-0.031440
C	-2.112598	1.835772	0.836374
C	-3.101340	2.818828	0.337204
C	-3.610174	3.797836	1.207788
C	-3.477451	2.835850	-1.013817
C	-4.485939	4.771351	0.737067
H	-3.302040	3.802948	2.251408
C	-4.355166	3.812045	-1.481689
H	-3.052773	2.101308	-1.688482
C	-4.862714	4.777612	-0.610087
H	-4.864501	5.532511	1.413545
H	-4.633849	3.825904	-2.531464
H	-5.540427	5.542241	-0.979958
H	-1.559651	2.145051	1.729184
C	-3.282486	0.588908	2.044168
H	-2.481768	0.267456	2.698962
H	-3.949292	1.350651	2.425945
C	-3.777307	-0.353448	1.142757
C	-4.966884	-0.150376	0.300906
C	-5.142605	-0.942346	-0.852612
C	-5.940931	0.817408	0.615036
C	-6.255600	-0.761254	-1.667362
H	-4.392237	-1.680628	-1.111317
C	-7.057772	0.984726	-0.197513
H	-5.837313	1.430698	1.503072
C	-7.217590	0.198293	-1.340917
H	-6.372044	-1.369081	-2.559897
H	-7.804499	1.729394	0.061952
H	-8.089495	0.332598	-1.975263
O	-3.080870	-1.447195	0.882911
Si	-2.724477	-2.867907	1.813705
C	-1.092488	-3.524014	1.189806
H	-1.147688	-3.788423	0.130658
H	-0.311076	-2.771990	1.325839
H	-0.813944	-4.423764	1.753004
C	-4.141288	-4.045761	1.425010
H	-4.175580	-4.273729	0.353713
H	-4.012126	-4.993384	1.963011
H	-5.113959	-3.630862	1.714044
C	-2.680396	-2.442989	3.642981
H	-2.564141	-3.368090	4.222275
H	-3.599791	-1.955957	3.987198
H	-1.831129	-1.792335	3.872340

TS 143

B3LYP/6-31G(d) = -3573.922244
B3LYP-D3(BJ)/def2-TZVPP/IEFPCM(propanonitrile) = -3575.502816
B3LYP-D3(BJ)/def2-TZVPP/IEFPCM(propanonitrile)//B3LYP-D3(BJ)/6-31G(d) Free Energy (Quasiharmonic) = -3574.786458

Frequencies (Top 3 out of 300)

1. -282.0716 cm^{-1}
2. 9.2412 cm^{-1}
3. 11.0291 cm^{-1}

B3LYP/6-31G(d) Molecular Geometry in Cartesian Coordinates

B	0.807935	0.882468	-0.840686
O	1.351872	1.088500	-2.191586
N	1.380241	2.124353	-0.090416
C	2.371923	2.812588	-0.926733
S	0.891988	2.769165	1.329776
C	2.212742	2.107989	-2.279168
O	2.794179	2.427982	-3.290683
H	3.917045	3.486881	0.411784
H	2.095678	3.865381	-1.079686
C	3.845627	2.786584	-0.425090
C	4.366501	1.446213	0.006475
C	4.933867	0.393231	-0.806428
C	5.254793	-0.683032	0.069207
N	4.920330	-0.285489	1.349009
C	4.364862	0.978024	1.297548
H	3.995972	1.461337	2.191119
C	6.039697	-2.004938	-1.754354
C	5.194390	0.243973	-2.181313
H	6.039822	-2.694930	0.296563
H	4.858916	-0.896816	2.148886
C	5.739130	-0.949778	-2.639527
H	6.459686	-2.928294	-2.144293
H	4.945882	1.039246	-2.877827
C	5.806325	-1.883727	-0.388775
H	5.931864	-1.076610	-3.701498
C	0.152411	4.361458	0.915085
O	-0.167594	1.876530	1.846295
O	2.029481	3.084122	2.210456
C	-1.048702	6.832359	0.354626
C	-0.766471	4.462495	-0.132409
C	0.481197	5.479444	1.681489
C	-0.121505	6.704772	1.396083
C	-1.356331	5.693374	-0.405397
H	-1.003946	3.591427	-0.733640
H	1.208133	5.384373	2.480477
H	0.139878	7.577389	1.989864
H	-2.065516	5.772841	-1.226110
C	-1.713940	8.156273	0.061505
H	-1.802474	8.330255	-1.016598
H	-1.154246	8.990985	0.494882
H	-2.729111	8.189284	0.478988
H	4.447025	3.198053	-1.245217
C	1.248458	-0.576172	-0.277779
C	1.338126	-0.903109	1.084156
C	1.643985	-1.564500	-1.198666
C	1.798720	-2.158628	1.501941
C	2.094350	-2.819734	-0.784487
H	1.631035	-1.331246	-2.258261
C	2.176275	-3.126691	0.573215
H	2.538286	-4.094831	0.898244
C	2.453969	-3.866048	-1.804574
C	1.948978	-2.432469	2.969252
F	1.364687	-4.597003	-2.163561
F	3.366233	-4.744500	-1.329192
F	2.951426	-3.330069	-2.934730
F	0.905646	-1.938628	3.686019

F	3.068119	-1.858617	3.489950
F	2.027228	-3.752492	3.247886
O	-0.712899	1.030105	-1.027203
C	-1.647080	0.519260	-0.273035
C	-2.944215	1.242223	-0.277138
C	-3.348519	1.995122	-1.389530
C	-3.730607	1.247014	0.883185
C	-4.520727	2.746684	-1.336724
H	-2.726972	2.000254	-2.279678
C	-4.900268	2.006137	0.935742
H	-3.403119	0.689983	1.757129
C	-5.297783	2.756542	-0.173544
H	-4.824807	3.333650	-2.199178
H	-5.488178	2.029049	1.849206
H	-6.203662	3.354972	-0.129158
H	-1.343176	0.109210	0.688176
C	-2.023603	-1.273967	-1.167655
H	-1.036246	-1.674122	-0.969930
H	-2.171654	-0.861234	-2.160147
C	-3.115266	-1.980977	-0.647214
C	-3.015114	-2.860422	0.530771
C	-1.979420	-2.689107	1.470176
C	-3.921002	-3.921667	0.718242
C	-1.876332	-3.527953	2.576103
H	-1.249034	-1.899122	1.345506
C	-3.805940	-4.769693	1.815258
H	-4.686807	-4.113841	-0.022605
C	-2.790276	-4.568739	2.753351
H	-1.075134	-3.367487	3.289434
H	-4.503318	-5.594031	1.933442
H	-2.704209	-5.228226	3.612218
O	-4.272897	-1.830795	-1.261428
Si	-5.995690	-1.859055	-1.185459
C	-6.598334	-1.606110	0.574613
H	-6.232291	-0.655938	0.977512
H	-6.289804	-2.406926	1.253075
H	-7.695289	-1.567464	0.577606
C	-6.559307	-3.494089	-1.929717
H	-6.036273	-3.695383	-2.871531
H	-6.419040	-4.359827	-1.274413
H	-7.630903	-3.431358	-2.158415
H	1.058282	-0.167257	1.832171
C	-6.508014	-0.453010	-2.310660
H	-6.138700	0.510304	-1.945936
H	-6.120382	-0.602522	-3.324711
H	-7.601635	-0.394637	-2.376948

TS 144

B3LYP/6-31G(d) = -3573.927364
B3LYP-D3(BJ)/def2-TZVPP/IEFPCM(propanonitrile) = -3575.503241
B3LYP-D3(BJ)/def2-TZVPP/IEFPCM(propanonitrile)//B3LYP-D3(BJ)/6-31G(d) Free Energy (Quasiharmonic) = -3574.786442

Frequencies (Top 3 out of 300)

1. -302.7970 cm^{-1}
2. 2.8315 cm^{-1}
3. 10.9961 cm^{-1}

B3LYP/6-31G(d) Molecular Geometry in Cartesian Coordinates

B	-0.003324	0.591865	-1.245959
O	-0.087311	0.018716	-2.598878
N	1.456419	1.180529	-1.238296
C	2.073475	0.969020	-2.560675
S	1.840506	2.559543	-0.402667
C	0.967186	0.289068	-3.369431
O	1.050992	-0.022681	-4.537784
H	4.134100	0.580512	-2.014914
H	2.310199	1.926814	-3.039229
C	3.348150	0.077067	-2.586238
C	3.169990	-1.330419	-2.074716
C	3.783607	-1.916621	-0.905753
C	3.308974	-3.254316	-0.801684
N	2.476428	-3.475452	-1.878632
C	2.388167	-2.317522	-2.628876
H	1.777568	-2.289663	-3.520122
C	4.593330	-3.618163	1.174326
C	4.701736	-1.454664	0.053012
H	3.313505	-5.126736	0.297735
H	1.855092	-4.263939	-1.975341
C	5.095509	-2.304047	1.079630
H	4.916662	-4.258027	1.991208
H	5.098882	-0.445818	-0.003627
C	3.696615	-4.111266	0.233613
H	5.803574	-1.951111	1.825095
C	3.442415	2.300650	0.377787
O	0.864238	2.655045	0.701230
O	2.017765	3.695482	-1.322993
C	5.921611	2.028957	1.658779
C	4.586951	2.844005	-0.207898
C	3.518427	1.628173	1.599376
C	4.754440	1.499699	2.228678
C	5.817966	2.695982	0.430352
H	4.504666	3.392986	-1.139888
H	2.625835	1.217995	2.058573
H	4.813079	0.976434	3.179872
H	6.709613	3.117926	-0.027326
C	7.250235	1.912917	2.367761
H	8.084548	1.912255	1.658801
H	7.308467	0.995814	2.963195
H	7.405329	2.756468	3.053681
O	-1.040220	1.724477	-1.296293
C	-1.750321	2.228887	-0.325910
H	-1.633191	1.793211	0.666057
H	3.678126	0.066882	-3.632784
C	-1.965558	3.701997	-0.362098
C	-1.788872	4.419990	-1.552572
C	-2.322806	4.384699	0.808930
C	-1.981847	5.799323	-1.570555
H	-1.473898	3.892925	-2.446712
C	-2.511669	5.764545	0.789268
H	-2.422644	3.836871	1.742691
C	-2.348257	6.473983	-0.403173
H	-1.833379	6.351485	-2.494268
H	-2.773807	6.287836	1.704620
H	-2.492931	7.550772	-0.419313
C	-0.264492	-0.534339	-0.111381
C	-0.552877	-1.851623	-0.503320
C	-0.115716	-0.302563	1.265251
C	-0.670646	-2.888433	0.426051
H	-0.657966	-2.070905	-1.559474
C	-0.248926	-1.334135	2.200568
H	0.148733	0.691681	1.610745
C	-0.522755	-2.637691	1.790238
H	-0.593809	-3.442009	2.513126
C	-0.141190	-1.018839	3.662960
C	-1.002739	-4.277737	-0.037912
F	-1.339839	-0.587877	4.167350
F	0.214772	-2.088534	4.403007
F	-0.555727	-5.226094	0.812795
F	0.746896	-0.032493	3.914584
F	-0.474414	-4.555413	-1.259078
F	-2.344523	-4.470463	-0.158567
C	-3.690652	1.671529	-0.549310
H	-4.100034	2.222793	0.289296
H	-3.859637	2.142666	-1.509361
C	-3.799251	0.273028	-0.494520
C	-3.637243	-0.597208	-1.660000
C	-3.873247	-1.982763	-1.536569

C	-3.246478	-0.082425	-2.912915
C	-3.741025	-2.820247	-2.636266
H	-4.138702	-2.392458	-0.570222
C	-3.102639	-0.928507	-4.007166
H	-3.010729	0.968008	-3.027202
C	-3.358375	-2.294500	-3.875108
H	-3.914302	-3.885861	-2.522946
H	-2.768161	-0.523601	-4.956790
H	-3.245691	-2.952121	-4.732639
O	-3.990769	-0.360376	0.652935
Si	-4.792150	-0.012440	2.149408
C	-6.572042	0.404032	1.706179
H	-7.153652	0.604548	2.614292
H	-6.640152	1.292982	1.068876
H	-7.055251	-0.425660	1.178132
C	-4.622543	-1.625147	3.083064
H	-4.967615	-2.478308	2.489168
H	-5.211753	-1.598988	4.007880
H	-3.577349	-1.797070	3.360515
C	-3.955071	1.384195	3.085993
H	-4.419946	1.468984	4.077111
H	-2.893455	1.171429	3.248215
H	-4.052557	2.359735	2.599340

TS 145

B3LYP/6-31G(d) = -3573.926346
B3LYP-D3(BJ)/def2-TZVPP/IEFPCM(propanonitrile) = -3575.503653
B3LYP-D3(BJ)/def2-TZVPP/IEFPCM(propanonitrile)//B3LYP-D3(BJ)/6-31G(d) Free Energy (Quasiharmonic) = -3574.78636

Frequencies (Top 3 out of 300)

1. -257.4815 cm^{-1}
2. 13.5825 cm^{-1}
3. 16.4921 cm^{-1}

B3LYP/6-31G(d) Molecular Geometry in Cartesian Coordinates

B	0.810370	-0.399290	-0.566190
O	0.825470	-0.436100	-2.044190
N	1.836199	-1.500290	-0.212160
C	2.234059	-2.231741	-1.425390
S	2.626309	-1.764931	1.203640
C	1.602189	-1.402920	-2.547260
O	1.768729	-1.600200	-3.728720
H	2.446609	-4.293631	-0.836210
H	3.321939	-2.190341	-1.574900
C	1.800189	-3.726040	-1.510200
C	0.364139	-3.997260	-1.168370
C	-0.797231	-3.915780	-2.025960
C	-1.930661	-4.226970	-1.222410
N	-1.470361	-4.511640	0.048290
C	-0.099431	-4.344600	0.076930
H	0.460279	-4.485880	0.991100
C	-3.399431	-3.905839	-3.073530
C	-0.997441	-3.610220	-3.384840
H	-4.085401	-4.457919	-1.096680
H	-2.052741	-4.570059	0.870410
C	-2.291301	-3.607179	-3.892220
H	-4.399611	-3.887569	-3.498240
H	-0.155591	-3.354230	-4.021440
C	-3.233561	-4.223699	-1.730330
H	-2.454611	-3.363909	-4.938890
C	4.307229	-1.137921	1.017540
O	2.777419	-3.212661	1.418400
O	1.971590	-0.931180	2.225620
C	6.931420	-0.169202	0.765300
C	5.293239	-1.942731	0.440110
C	4.626350	0.133949	1.494330
C	5.932250	0.606879	1.368850
C	6.590679	-1.450642	0.310230
H	5.054629	-2.954601	0.130730
H	3.861500	0.725819	1.985050
H	6.183660	1.590099	1.760620
H	7.355499	-2.080712	-0.137590
C	8.337500	0.358148	0.604800
H	8.539190	1.181368	1.297610
H	8.503940	0.735458	-0.412930
H	9.081900	-0.425382	0.783020
H	2.030289	-4.046680	-2.533840
C	-0.709400	-0.554450	-0.037490
C	-1.052841	-1.151830	1.186280
C	-1.766260	-0.146500	-0.865680
C	-2.386601	-1.323349	1.561390
H	-0.268731	-1.484270	1.855470
C	-3.103180	-0.281299	-0.476610
H	-1.546800	0.258700	-1.848100
C	-3.423810	-0.876469	0.740240
H	-4.458500	-0.990939	1.044530
C	-4.205490	0.151461	-1.399160
C	-2.735351	-2.057879	2.823100
F	-3.818290	1.175571	-2.209560
F	-5.292370	0.595651	-0.712880
F	-4.636010	-0.836829	-2.206890
F	-1.716941	-2.112910	3.702490
F	-3.093111	-3.354509	2.565900
F	-3.794651	-1.504809	3.460780
O	1.434320	0.989950	-0.191750
C	1.084200	2.044680	-0.861830
C	2.153930	2.936240	-1.370360
C	1.879761	3.809080	-2.434850
C	3.452410	2.878769	-0.844220
C	2.883381	4.621469	-2.956390
H	0.884211	3.829450	-2.871350
C	4.452541	3.696589	-1.363910
H	3.673300	2.173089	-0.051670
C	4.171111	4.572199	-2.416190
H	2.665181	5.284009	-3.789070
H	5.457981	3.640989	-0.956110
H	4.955281	5.204689	-2.822960
H	0.192740	1.973850	-1.487200
C	0.208961	3.493040	0.460300
H	1.090961	3.564540	1.083770
H	0.119041	4.267290	-0.292010
C	-0.970450	3.048940	1.063310
C	-1.004210	2.277610	2.309170
C	0.161730	1.694300	2.841710
C	-2.222950	2.122781	3.000630
C	0.108680	0.980940	4.034360
H	1.097120	1.740770	2.298260
C	-2.265950	1.424881	4.201450
H	-3.126710	2.552571	2.585270
C	-1.100450	0.852830	4.721040
H	1.006240	0.496530	4.403040
H	-3.210000	1.309851	4.725530
H	-1.139690	0.291630	5.650170
O	-2.163269	3.285721	0.531530
Si	-2.861739	4.493221	-0.488360
C	-2.368689	6.174731	0.199570
H	-1.287869	6.348320	0.161370
H	-2.850759	6.971131	-0.381060
H	-2.689899	6.286061	1.241460
C	-2.281859	4.262421	-2.260030
H	-1.229539	4.532720	-2.397190
H	-2.424979	3.228161	-2.588630
H	-2.872069	4.903261	-2.927180
C	-4.696149	4.170351	-0.306810
H	-4.947030	3.141881	-0.584130
H	-5.273059	4.843191	-0.952980
H	-5.028419	4.334141	0.724650

TS 146

B3LYP/6-31G(d) = -3573.922312
B3LYP-D3(BJ)/def2-TZVPP/IEFPCM(propanonitrile) = -3575.503251
B3LYP-D3(BJ)/def2-TZVPP/IEFPCM(propanonitrile)//B3LYP-D3(BJ)/6-31G(d) Free Energy (Quasiharmonic) = -3574.786285

Frequencies (Top 3 out of 300)

1. -288.9090 cm^{-1}
2. 10.8669 cm^{-1}
3. 12.7340 cm^{-1}

B3LYP/6-31G(d) Molecular Geometry in Cartesian Coordinates

B	0.752862	0.816261	-0.959029
O	1.041639	0.936117	-2.399727
N	1.644354	1.946685	-0.372775
C	2.530505	2.490605	-1.408597
S	1.499744	2.690896	1.079180
C	1.993263	1.831583	-2.685895
O	2.397599	2.062942	-3.803008
H	4.382670	2.686356	-0.322190
H	2.399233	3.577231	-1.508541
C	4.049316	2.206617	-1.246457
C	4.436964	0.752338	-1.249397
C	4.772641	-0.075907	-0.113682
C	5.062703	-1.376231	-0.613499
N	4.928544	-1.327849	-1.986266
C	4.536549	-0.055957	-2.357820
H	4.358903	0.185351	-3.396816
C	5.478759	-2.190956	1.587932
C	4.849755	0.147067	1.273345
H	5.617243	-3.429639	-0.183847
H	4.899981	-2.137864	-2.585954
C	5.200292	-0.909044	2.106219
H	5.743511	-2.997004	2.267335
H	4.607139	1.120607	1.687790
C	5.413941	-2.441807	0.221806
H	5.250196	-0.748950	3.179779
C	0.914827	4.361728	0.722419
O	0.441196	1.968165	1.811554
O	2.813714	2.872091	1.717299
C	-0.022212	6.964468	0.261205
C	-0.190561	4.562481	-0.107078
C	1.555864	5.444616	1.324529
C	1.081317	6.735545	1.092427
C	-0.648828	5.857869	-0.331563
H	-0.678291	3.713519	-0.573322
H	2.415082	5.268323	1.962043
H	1.580777	7.578842	1.563546
H	-1.508573	6.014423	-0.979241
C	-0.508466	8.367276	-0.016197
H	-1.597806	8.400099	-0.127052
H	-0.227539	9.055285	0.787626
H	-0.076179	8.757957	-0.947039
H	4.536873	2.728810	-2.078759
C	1.084432	-0.682940	-0.426308
C	1.315277	-1.710223	-1.358401
C	1.255662	-1.004597	0.931493
C	1.701398	-2.992307	-0.957642
C	1.630990	-2.290686	1.335326
H	1.127932	-0.232358	1.684114
C	1.857427	-3.293804	0.395391
H	2.168255	-4.283453	0.708593
C	1.755905	-2.597508	2.801199
C	2.044919	-4.033508	-1.984136
F	2.253418	-1.568028	3.506450
F	2.529387	-3.677704	3.039722
F	0.530420	-2.876254	3.352069
F	1.764611	-5.284983	-1.554601
F	3.375609	-4.029083	-2.289240
F	1.386556	-3.847352	-3.147647
O	-0.728031	1.210116	-0.835280
C	-1.617985	0.645405	-0.065581
C	-2.795504	1.478040	0.289504
C	-3.258214	2.485941	-0.568682
C	-3.400575	1.307861	1.542373
C	-4.301410	3.319753	-0.170895
H	-2.783279	2.618113	-1.535968
C	-4.442381	2.146377	1.940381
H	-3.028489	0.544346	2.220870
C	-4.893776	3.154523	1.085319
H	-4.647881	4.105858	-0.836310
H	-4.885075	2.026950	2.925368
H	-5.697284	3.815052	1.399413
H	-1.245490	0.005933	0.734816
C	-2.311049	-0.841638	-1.240558
H	-1.374348	-1.387438	-1.257158
H	-2.494350	-0.204294	-2.099324
C	-3.436429	-1.534220	-0.768462
C	-3.345482	-2.684812	0.145475
C	-2.224855	-2.857676	0.981756
C	-4.352913	-3.668632	0.159671
C	-2.135751	-3.958994	1.828504
H	-1.420304	-2.131391	0.984792
C	-4.251918	-4.778114	0.992646
H	-5.191293	-3.589985	-0.520900
C	-3.148375	-4.920562	1.837396
H	-1.270776	-4.059270	2.473979
H	-5.029564	-5.536305	0.976185
H	-3.072334	-5.783828	2.492409
O	-4.614550	-1.117597	-1.189739
Si	-6.306911	-0.976397	-0.869831
C	-7.182112	-2.314965	-1.863885
H	-7.116427	-3.316602	-1.427139
H	-6.789452	-2.364364	-2.885926
H	-8.247840	-2.062996	-1.936468
C	-6.745968	0.701786	-1.573652
H	-6.208279	1.506392	-1.063293
H	-6.501991	0.754288	-2.640765
H	-7.821564	0.890830	-1.467965
H	1.220330	-1.490434	-2.416800
C	-6.648077	-1.083372	0.972714
H	-6.376695	-2.052434	1.401908
H	-6.103041	-0.303318	1.514808
H	-7.719456	-0.925143	1.151179

TS 147

B3LYP/6-31G(d) = -3573.931543
B3LYP-D3(BJ)/def2-TZVPP/IEFPCM(propanonitrile) = -3575.502537
B3LYP-D3(BJ)/def2-TZVPP/IEFPCM(propanonitrile)//B3LYP-D3(BJ)/6-31G(d) Free Energy (Quasiharmonic) = -3574.786235

Frequencies (Top 3 out of 300)

1. -263.8254 cm^{-1}
2. 8.4793 cm^{-1}
3. 10.8288 cm^{-1}

B3LYP/6-31G(d) Molecular Geometry in Cartesian Coordinates

B	-0.182550	0.257673	1.013687
O	-0.631147	-0.378029	2.273382
N	-0.747953	-0.757645	-0.038009
C	-1.645061	-1.733256	0.614015
S	-0.914145	-0.444804	-1.657443
C	-1.391298	-1.461233	2.103785

O	-1.816922	-2.141804	3.010502
H	-1.110567	-3.757007	1.170643
H	-2.699156	-1.482476	0.425266
C	-1.414954	-3.236761	0.254590
C	-2.609743	-3.937471	-0.328297
C	-3.781291	-4.396514	0.383019
C	-4.632971	-5.023492	-0.568548
N	-4.004147	-4.938781	-1.795107
C	-2.792830	-4.286944	-1.643851
H	-2.149692	-4.102591	-2.492538
C	-6.236222	-5.520263	1.123963
C	-4.188711	-4.337764	1.728593
H	-6.500155	-6.066868	-0.953210
H	-4.362901	-5.303418	-2.663302
C	-5.409289	-4.899789	2.083121
H	-7.185004	-5.950444	1.434019
H	-3.561248	-3.847155	2.467575
C	-5.861635	-5.590059	-0.213366
H	-5.736680	-4.859099	3.118612
C	-2.481352	0.387761	-1.914029
O	-0.980969	-1.725073	-2.378022
O	0.159120	0.511393	-1.991693
C	-4.943539	1.700914	-2.151416
C	-3.660331	-0.361052	-1.960037
C	-2.512984	1.780872	-2.022222
C	-3.740652	2.423298	-2.144667
C	-4.881592	0.304144	-2.072768
H	-3.623762	-1.444908	-1.919017
H	-1.587958	2.345479	-2.006154
H	-3.768043	3.507360	-2.215211
H	-5.801154	-0.275205	-2.095165
C	-6.265395	2.426522	-2.214267
H	-6.421981	3.009441	-1.298277
H	-7.103487	1.731093	-2.319988
H	-6.298148	3.129426	-3.055151
H	-0.583957	-3.285930	-0.452262
C	-0.659818	1.796340	0.939684
C	-1.952301	2.150003	1.363430
C	0.156949	2.826576	0.461675
C	-2.413042	3.464919	1.290880
C	-0.297595	4.148707	0.386809
H	1.168361	2.597823	0.146159
C	-1.586412	4.477426	0.798131
H	-1.937879	5.501653	0.748880
C	0.605157	5.197200	-0.197070
C	-3.826008	3.808907	1.673535
F	0.169867	6.452532	0.038330
F	0.709466	5.066657	-1.550146
F	1.867325	5.109001	0.285222
F	-4.622194	3.935390	0.575806
F	-3.896702	4.991655	2.327167
F	-4.392396	2.873722	2.461018
O	1.352002	0.271194	1.036683
C	2.027496	-0.841251	1.124717
C	3.204101	-0.861870	2.021249
C	3.810956	0.330858	2.442970
C	3.679599	-2.088446	2.507752
C	4.885077	0.289682	3.328959
H	3.417631	1.277880	2.090504
C	4.750688	-2.125387	3.398953
H	3.189626	-3.011608	2.207821
C	5.358740	-0.935492	3.806574
H	5.346038	1.216482	3.658834
H	5.099810	-3.078212	3.787316
H	6.189794	-0.962084	4.506077
H	1.449866	-1.768056	1.089865
C	2.714871	-1.256638	-0.795479
H	1.884908	-0.744278	-1.265299
H	2.651530	-2.340285	-0.791407
C	3.991010	-0.699914	-0.886880
C	4.251375	0.726459	-1.097149
C	3.234240	1.600909	-1.534313
C	5.543437	1.240987	-0.859717
C	3.509612	2.953519	-1.719361
H	2.237410	1.231194	-1.746367
C	5.807327	2.592849	-1.044101
H	6.323365	0.571986	-0.515851
C	4.790838	3.452278	-1.472394
H	2.718623	3.620016	-2.045880
H	6.803642	2.979926	-0.849983
H	4.996447	4.509747	-1.613803
O	5.074385	-1.433281	-0.642485
Si	5.601289	-2.984171	-1.188324
C	7.421343	-2.966105	-0.741262
H	7.901738	-3.912525	-1.017120
H	7.951451	-2.158635	-1.258231
H	7.559328	-2.823522	0.336500
C	5.300986	-3.040999	-3.041894
H	5.654627	-3.992425	-3.457658
H	4.236435	-2.950443	-3.285584
H	5.832921	-2.233438	-3.557264
H	-2.613322	1.388547	1.764977
C	4.712372	-4.382336	-0.299131
H	3.647565	-4.444568	-0.548647
H	5.167925	-5.338698	-0.586809
H	4.804828	-4.286678	0.787521

TS 148

B3LYP/6-31G(d) = -3573.932043
B3LYP-D3(BJ)/def2-TZVPP/IEFPCM(propanonitrile) = -3575.502099
B3LYP-D3(BJ)/def2-TZVPP/IEFPCM(propanonitrile)//B3LYP-D3(BJ)/6-31G(d) Free Energy (Quasiharmonic) = -3574.786133

Frequencies (Top 3 out of 300)

1. -290.8125 cm^{-1}
2. 7.3649 cm^{-1}
3. 10.0928 cm^{-1}

B3LYP/6-31G(d) Molecular Geometry in Cartesian Coordinates

B	0.169580	-0.794740	0.931660
O	0.156500	-0.240510	2.305480
N	0.340180	0.521770	0.084500
C	0.766170	1.623670	0.980520
S	0.829480	0.533310	-1.511210
C	0.449070	1.056400	2.372560
O	0.443910	1.699740	3.400020
H	-0.870969	3.009190	1.313770
H	1.855500	1.767140	0.925850
C	0.080211	3.002360	0.767050
C	0.931831	4.171340	1.184540
C	1.610811	5.085560	0.296050
C	2.304881	6.026780	1.107840
N	2.053131	5.688390	2.421370
C	1.236921	4.573950	2.462100
H	0.947061	4.123670	3.400020
C	3.142381	7.151350	-0.821750
C	1.703431	5.201450	-1.103740
H	3.592342	7.773769	1.200760
H	2.428991	6.159480	3.229210
C	2.466951	6.228620	-1.646670
H	3.730512	7.944419	-1.275810
H	1.192711	4.491880	-1.749950
C	3.070361	7.063350	0.564280
H	2.546551	6.324360	-2.726330
C	2.618580	0.419490	-1.579230
O	0.466911	1.844820	-2.084600
O	0.265680	-0.689610	-2.106010
C	5.411410	0.204279	-1.556110

C	3.217950	-0.813420	-1.852390
C	3.396510	1.556260	-1.340340
C	4.786040	1.435899	-1.325100
C	4.606030	-0.908411	-1.840840
H	2.605950	-1.680420	-2.070520
H	2.932441	2.525250	-1.189360
H	5.391121	2.317749	-1.130710
H	5.072460	-1.868541	-2.046140
C	6.911540	0.060499	-1.477630
H	7.305770	-0.507961	-2.327730
H	7.410340	1.034269	-1.457480
H	7.193080	-0.481381	-0.565970
H	-0.154069	3.098250	-0.293690
C	1.301540	-1.942460	0.787580
C	1.141150	-3.061620	-0.039380
C	2.513450	-1.833290	1.488060
C	2.151039	-4.018560	-0.180230
C	3.527470	-2.783291	1.350140
H	2.671420	-0.997010	2.161750
C	3.352819	-3.885460	0.512100
H	4.134129	-4.629731	0.408900
C	4.851610	-2.589851	2.035070
C	1.969919	-5.141430	-1.162070
F	5.751220	-1.981961	1.214940
F	5.408589	-3.766531	2.403880
F	4.753960	-1.822701	3.140420
F	2.248419	-4.735300	-2.427620
F	0.701299	-5.607390	-1.176090
F	2.782979	-6.190160	-0.901530
O	-1.173420	-1.469750	0.739930
C	-2.119400	-1.194709	-0.117360
C	-3.139940	-2.260569	-0.295450
C	-3.988400	-2.240289	-1.413280
C	-3.220340	-3.329659	0.609950
C	-4.908540	-3.266889	-1.616980
H	-3.914960	-1.428539	-2.131850
C	-4.141111	-4.355259	0.402740
H	-2.542350	-3.354329	1.456210
C	-4.989061	-4.324949	-0.708050
H	-5.553270	-3.247739	-2.491130
H	-4.188721	-5.185979	1.101490
H	-5.700721	-5.129629	-0.871820
H	-1.828990	-0.657310	-1.020660
C	-3.043270	0.393941	0.716450
H	-2.169270	1.023871	0.613280
H	-3.191740	-0.015509	1.709480
C	-4.180850	0.735281	-0.024940
C	-4.129030	1.444311	-1.309430
C	-5.320200	1.704321	-2.019010
C	-2.902909	1.862811	-1.865530
C	-5.280709	2.364721	-3.241570
H	-6.266500	1.379741	-1.602540
C	-2.868699	2.514871	-3.093500
H	-1.963010	1.683630	-1.359560
C	-4.056149	2.770341	-3.783030
H	-6.204929	2.561511	-3.777450
H	-1.907319	2.810020	-3.501290
H	-4.029549	3.282081	-4.741150
O	-5.387500	0.315521	0.326300
Si	-6.182050	0.091281	1.853680
C	-5.514640	-1.397999	2.779260
H	-6.182270	-1.622179	3.621470
H	-5.473370	-2.285409	2.139680
H	-4.513970	-1.234229	3.192990
C	-7.956940	-0.186338	1.318370
H	-8.606110	-0.340788	2.188600
H	-8.347030	0.671282	0.759120
H	-8.040590	-1.072608	0.679350
H	0.217720	-3.189700	-0.593470
C	-5.953700	1.687561	2.817730
H	-4.898789	1.887521	3.035930
H	-6.354139	2.548051	2.270070
H	-6.481530	1.628491	3.777520

TS 149

B3LYP/6-31G(d) = -3573.930364
B3LYP-D3(BJ)/def2-TZVPP/IEFPCM(propanonitrile) = -3575.502926
B3LYP-D3(BJ)/def2-TZVPP/IEFPCM(propanonitrile)//B3LYP-D3(BJ)/6-31G(d) Free Energy (Quasiharmonic) = -3574.786096

Frequencies (Top 3 out of 300)

1. -266.0255 cm^{-1}
2. 9.2092 cm^{-1}
3. 15.8349 cm^{-1}

B3LYP/6-31G(d) Molecular Geometry in Cartesian Coordinates

B	0.048880	-0.417850	0.031760
O	0.299140	0.756030	-0.844850
N	0.560931	-1.570910	-0.862240
C	0.848660	-1.086760	-2.217540
S	0.995521	-3.093510	-0.427600
C	0.756150	0.429660	-2.048270
O	1.062859	1.246141	-2.899400
H	0.202701	-2.648410	-3.544820
H	1.883811	-1.316519	-2.508580
C	-0.083759	-1.611350	-3.352180
C	-1.551509	-1.543311	-3.051190
C	-2.461060	-0.441131	-3.262770
C	-3.731060	-0.846312	-2.761610
N	-3.596179	-2.139852	-2.296650
C	-2.283439	-2.537991	-2.449810
H	-1.953228	-3.510441	-2.112210
C	-4.687081	1.263488	-3.331930
C	-2.333280	0.845669	-3.816950
H	-5.808350	-0.332173	-2.395710
H	-4.262909	-2.617402	-1.707810
C	-3.442381	1.682719	-3.844400
H	-5.534721	1.942818	-3.361810
H	-1.374571	1.187879	-4.197210
C	-4.848830	-0.006552	-2.789370
H	-3.350851	2.680929	-4.264990
C	2.799551	-3.139959	-0.520600
O	0.527142	-4.062810	-1.432390
O	0.609771	-3.267690	0.981790
C	5.599651	-3.305137	-0.594450
C	3.565541	-2.257868	0.247050
C	3.415572	-4.098038	-1.324750
C	4.809002	-4.171558	-1.358190
C	4.954681	-2.346178	0.203650
H	3.074691	-1.507959	0.858370
H	2.803262	-4.772759	-1.912770
H	5.287412	-4.917228	-1.988660
H	5.552111	-1.662848	0.803630
C	7.106121	-3.412587	-0.605650
H	7.467152	-3.892657	-1.520690
H	7.464422	-4.012167	0.241700
H	7.580261	-2.428087	-0.528010
H	0.166040	-1.031220	-4.249370
C	-1.498940	-0.422631	0.507710
C	-2.152379	-1.574061	0.976540
C	-2.257190	0.755639	0.424320
C	-3.506729	-1.547231	1.318020
H	-1.600439	-2.504561	1.062290
C	-3.603310	0.793948	0.802560
H	-1.802441	1.655809	0.023470
C	-4.240640	-0.361612	1.245060
H	-5.290280	-0.344572	1.514550
C	-4.353571	2.095828	0.758040

C	-4.217749	-2.826622	1.651680
F	-5.689341	1.913338	0.651490
F	-3.966261	2.873658	-0.275130
F	-4.151131	2.826128	1.886980
F	-5.277439	-2.631212	2.467900
F	-3.413818	-3.734051	2.236180
F	-4.715459	-3.421662	0.522370
O	0.973680	-0.314200	1.255970
C	0.833750	0.580240	2.186810
C	1.051710	0.138501	3.585980
C	1.502920	-1.163599	3.852890
C	0.731840	0.994260	4.653000
C	1.653421	-1.587359	5.172410
H	1.682041	-1.845439	3.028410
C	0.882190	0.565030	5.967720
H	0.352039	1.993440	4.449830
C	1.352140	-0.725989	6.229540
H	1.991821	-2.599549	5.374600
H	0.625869	1.229910	6.787640
H	1.467400	-1.062969	7.256070
H	0.067009	1.345080	2.042180
C	2.425479	1.955651	2.049740
H	2.132929	2.514241	2.928870
H	3.228219	1.237501	2.179220
C	2.328129	2.595841	0.815480
C	1.477748	3.785021	0.614380
C	0.748038	3.943760	-0.580100
C	1.359758	4.757391	1.627310
C	-0.091462	5.044200	-0.739790
H	0.804659	3.184190	-1.351770
C	0.534187	5.863240	1.449760
H	1.943318	4.664551	2.538410
C	-0.198683	6.005010	0.268160
H	-0.670372	5.144960	-1.652980
H	0.463007	6.614900	2.230630
H	-0.852713	6.862250	0.135040
O	2.922179	2.040781	-0.225120
Si	4.103419	2.547772	-1.389080
C	4.512860	0.975602	-2.315260
H	3.663730	0.680722	-2.940370
H	5.376669	1.134452	-2.972760
H	4.749060	0.147662	-1.638350
C	3.458668	3.872112	-2.549180
H	4.247418	4.123162	-3.270910
H	2.595458	3.499331	-3.109310
H	3.171458	4.794731	-2.035350
C	5.548019	3.183912	-0.359690
H	5.265928	4.057622	0.239880
H	6.375818	3.488513	-1.011860
H	5.927759	2.415093	0.322760

TS 150

B3LYP/6-31G(d) = -3573.926694
B3LYP-D3(BJ)/def2-TZVPP/IEFPCM(propanonitrile) = -3575.502275
B3LYP-D3(BJ)/def2-TZVPP/IEFPCM(propanonitrile)//B3LYP-D3(BJ)/6-31G(d) Free Energy (Quasiharmonic) = -3574.786083

Frequencies (Top 3 out of 300)

1. -245.6492 cm^{-1}
2. 12.7149 cm^{-1}
3. 13.7266 cm^{-1}

B3LYP/6-31G(d) Molecular Geometry in Cartesian Coordinates

B	-0.063252	-0.084618	0.832096
O	-0.029470	-0.259384	2.293632
N	-0.657829	1.380812	0.703576
C	-1.009195	1.871063	2.052828
S	-0.140599	2.483799	-0.400281
C	-0.435002	0.801812	2.984762
O	-0.406591	0.889037	4.194311
H	-2.933375	2.788465	1.654056
H	-0.510510	2.822605	2.271287
C	-2.532842	2.031686	2.335849
C	-3.341691	0.763831	2.236817
C	-4.280176	0.397085	1.201967
C	-4.745690	-0.913757	1.499574
N	-4.148492	-1.301378	2.680605
C	-3.298265	-0.301169	3.107599
H	-2.715711	-0.424396	4.009954
C	-6.109304	-0.939844	-0.455962
C	-4.782251	1.040916	0.057724
H	-5.966716	-2.608228	0.917150
H	-4.148415	-2.246539	3.032774
C	-5.684397	0.370232	-0.758886
H	-6.808718	-1.442515	-1.118588
H	-4.466369	2.049380	-0.189687
C	-5.649414	-1.596713	0.679276
H	-6.069605	0.860024	-1.649544
C	-1.529628	3.540809	-0.818989
O	0.209229	1.732206	-1.623327
O	0.889069	3.379194	0.179406
C	-3.629113	5.239060	-1.562545
C	-2.462922	3.107483	-1.765123
C	-1.622047	4.812237	-0.251964
C	-2.674634	5.648718	-0.622876
C	-3.500275	3.961645	-2.130017
H	-2.380447	2.122725	-2.212286
H	-0.865739	5.145817	0.450175
H	-2.746832	6.640275	-0.183025
H	-4.223106	3.629790	-2.871373
C	-4.777862	6.139321	-1.949387
H	-4.579706	7.181355	-1.680075
H	-5.702239	5.836791	-1.440245
H	-4.974196	6.096706	-3.026311
H	-2.599949	2.451147	3.347557
C	-0.956138	-1.212488	0.102638
C	-1.465778	-1.044098	-1.191457
C	-1.310432	-2.386510	0.783278
C	-2.308535	-1.992636	-1.775985
H	-1.218895	-0.145490	-1.747479
C	-2.157264	-3.336500	0.205130
H	-0.940165	-2.545670	1.790910
C	-2.664819	-3.146270	-1.080977
H	-3.333231	-3.873930	-1.525141
C	-2.604546	-4.521511	1.010315
C	-2.816897	-1.749148	-3.168698
F	-2.949914	-5.572807	0.236553
F	-3.707887	-4.234469	1.770454
F	-1.657524	-4.944889	1.874429
F	-3.203313	-0.461160	-3.345095
F	-3.873748	-2.530958	-3.478797
F	-1.862321	-1.989969	-4.103045
O	1.422015	-0.266686	0.468642
C	2.025088	-0.397239	-0.675078
C	2.852106	-1.602832	-0.891333
C	3.239332	-2.418013	0.183515
C	3.199378	-1.972661	-2.200194
C	3.969693	-3.580473	-0.052290
H	2.943746	-2.138308	1.189197
C	3.925792	-3.139099	-2.432799
H	2.875553	-1.356249	-3.035981
C	4.315777	-3.943054	-1.357402
H	4.260103	-4.212943	0.782002
H	4.173499	-3.429871	-3.449980
H	4.874355	-4.857547	-1.537950
H	1.534397	0.026364	-1.551947
C	3.398495	1.211688	-0.776222
H	3.647735	1.172445	-1.830567

H	2.655464	1.948684	-0.501492
C	4.382878	0.845831	0.135520
C	4.239443	0.961705	1.588643
C	5.161233	0.308491	2.434448
C	3.178273	1.688554	2.168303
C	5.017843	0.370287	3.814756
H	5.972292	-0.258833	1.993626
C	3.040447	1.741366	3.553038
H	2.471740	2.226200	1.546398
C	3.953668	1.082338	4.377473
H	5.729251	-0.143708	4.455110
H	2.205580	2.279750	3.989912
H	3.834717	1.120933	5.456653
O	5.472473	0.189507	-0.268603
Si	6.674692	0.430535	-1.471828
C	7.718490	-1.117545	-1.309184
H	8.154952	-1.202046	-0.307779
H	7.111954	-2.012490	-1.487938
H	8.541095	-1.115941	-2.034353
C	7.601077	1.995349	-0.994221
H	6.941813	2.871218	-1.006820
H	8.032323	1.915261	0.009787
H	8.420738	2.189876	-1.696887
C	5.953901	0.581441	-3.203193
H	5.297952	-0.262447	-3.441176
H	5.391454	1.509725	-3.350882
H	6.773385	0.579327	-3.933678

TS 151

B3LYP/6-31G(d) = -3573.928833
B3LYP-D3(BJ)/def2-TZVPP/IEFPCM(propanonitrile) = -3575.501206
B3LYP-D3(BJ)/def2-TZVPP/IEFPCM(propanonitrile)//B3LYP-D3(BJ)/6-31G(d) Free Energy (Quasiharmonic) = -3574.786069

Frequencies (Top 3 out of 300)

1. -283.7807 cm^{-1}
2. 5.8143 cm^{-1}
3. 6.9727 cm^{-1}

B3LYP/6-31G(d) Molecular Geometry in Cartesian Coordinates

B	0.149810	0.092617	0.071187
O	-0.373411	-0.640727	-1.120182
N	0.898841	1.301273	-0.610330
C	0.894526	1.109741	-2.071351
S	0.714437	2.850689	-0.034361
C	-0.010468	-0.102300	-2.283158
O	-0.370736	-0.525806	-3.364717
H	2.817633	1.851196	-2.707118
H	0.413604	1.952688	-2.583599
C	2.297223	0.888325	-2.717125
C	3.149756	-0.153056	-2.052613
C	3.141557	-1.585289	-2.253878
C	4.078739	-2.141231	-1.337494
N	4.650396	-1.091980	-0.644393
C	4.067798	0.087783	-1.061076
H	4.360099	1.028911	-0.616765
C	3.582866	-4.350162	-2.086158
C	2.437403	-2.454072	-3.108000
H	5.012546	-3.923283	-0.520295
H	5.187463	-1.180807	0.205739
C	2.663893	-3.821924	-3.015597
H	3.729393	-5.425362	-2.029525
H	1.708373	-2.063132	-3.811649
C	4.305487	-3.517676	-1.239211
H	2.117654	-4.500188	-3.665907
C	2.195779	3.727327	-0.559938
O	-0.422210	3.520264	-0.699434
O	0.755981	2.768986	1.432737
C	4.499319	5.120690	-1.342707
C	3.362187	3.618784	0.202659
C	2.158308	4.543813	-1.689324
C	3.310783	5.229950	-2.075166
C	4.502112	4.312712	-0.194409
H	3.366247	3.016395	1.105209
H	1.231850	4.653102	-2.243131
H	3.281246	5.868377	-2.954795
H	5.407808	4.235428	0.402647
C	5.751529	5.844416	-1.776898
H	6.293745	6.256426	-0.918763
H	6.438764	5.164440	-2.297359
H	5.522609	6.667047	-2.461486
H	2.115994	0.637354	-3.769822
C	1.076106	-0.853183	0.988638
C	0.975208	-2.249836	0.926383
C	2.058540	-0.315730	1.831583
C	1.818073	-3.079352	1.672568
C	2.922926	-1.139468	2.555964
H	2.156365	0.761745	1.908877
C	2.804575	-2.528731	2.488250
H	3.479584	-3.168550	3.046155
C	4.080268	-0.541611	3.298127
C	1.662111	-4.573640	1.617918
F	3.858251	0.732375	3.679739
F	5.206000	-0.512665	2.513095
F	4.415203	-1.247668	4.399472
F	1.076593	-4.989286	0.475796
F	0.895890	-5.035624	2.639993
F	2.854597	-5.208627	1.717210
O	-1.032735	0.538273	0.905049
C	-2.194098	0.903610	0.415594
C	-2.987407	1.825570	1.261217
C	-2.658414	2.015058	2.611701
C	-4.041907	2.558078	0.696549
C	-3.385178	2.917919	3.384841
H	-1.811325	1.480646	3.027098
C	-4.761179	3.464769	1.470843
H	-4.285387	2.428570	-0.355291
C	-4.437301	3.642046	2.818359
H	-3.118434	3.070471	4.426952
H	-5.567822	4.038041	1.022762
H	-4.995552	4.352792	3.421843
H	-2.249840	1.089125	-0.659706
C	-3.166217	-0.847277	0.342133
H	-3.043306	-1.115193	1.384317
H	-2.444695	-1.295993	-0.330381
C	-4.470394	-0.687260	-0.145442
C	-5.637591	-0.415407	0.706359
C	-5.537138	-0.376856	2.111904
C	-6.897947	-0.197557	0.113706
C	-6.659679	-0.126585	2.893561
H	-4.582620	-0.530532	2.600189
C	-8.017953	0.048292	0.899599
H	-6.983153	-0.221340	-0.966020
C	-7.902518	0.085027	2.291879
H	-6.564256	-0.096108	3.974747
H	-8.982342	0.213213	0.427824
H	-8.778086	0.277036	2.905868
O	-4.710691	-0.643777	-1.443410
Si	-4.296075	-1.511763	-2.899163
C	-3.010272	-2.831779	-2.566517
H	-3.241852	-3.436985	-1.682689
H	-2.985495	-3.512141	-3.427908
H	-2.007701	-2.405697	-2.461952
C	-3.734922	-0.201803	-4.110981
H	-2.705316	0.109971	-3.909337
H	-3.755608	-0.608523	-5.129953
H	-4.390701	0.675968	-4.090953
H	0.253501	-2.704652	0.255963

C	-5.944043	-2.269287	-3.400759
H	-5.837231	-2.828346	-4.338633
H	-6.316741	-2.966678	-2.641588
H	-6.709729	-1.501205	-3.559688

TS 152

B3LYP/6-31G(d) = -3573.925011
B3LYP-D3(BJ)/def2-TZVPP/IEFPCM(propanonitrile) = -3575.502973
B3LYP-D3(BJ)/def2-TZVPP/IEFPCM(propanonitrile)//B3LYP-D3(BJ)/6-31G(d) Free Energy (Quasiharmonic) = -3574.786063

Frequencies (Top 3 out of 300)

1. -276.6528 cm^{-1}
2. 7.1331 cm^{-1}
3. 9.9066 cm^{-1}

B3LYP/6-31G(d) Molecular Geometry in Cartesian Coordinates

B	0.533250	0.168363	0.230364
O	0.743904	1.525200	-0.340045
N	0.728251	0.430128	1.735384
C	0.982204	1.856314	1.990820
S	0.753766	-0.652471	2.986867
C	0.835066	2.465567	0.599967
O	0.822452	3.659737	0.368300
H	2.415702	1.802503	3.589987
H	0.216138	2.288218	2.646957
C	2.372977	2.232159	2.586901
C	3.563560	1.816083	1.771065
C	4.308560	2.625948	0.832541
C	5.341118	1.805405	0.298686
N	5.234611	0.569252	0.903856
C	4.158042	0.576081	1.768180
H	3.882693	-0.317813	2.311119
C	6.128620	3.589842	-1.071620
C	4.212228	3.958870	0.392359
H	7.017301	1.615404	-1.065552
H	5.725264	-0.258700	0.602762
C	5.120752	4.426738	-0.548610
H	6.818493	3.982625	-1.814035
H	3.421660	4.604208	0.764089
C	6.251399	2.269043	-0.657160
H	5.050286	5.454007	-0.896998
C	-0.851706	-1.474835	3.018267
O	0.832951	0.135571	4.227572
O	1.739403	-1.713015	2.726923
C	-3.305479	-2.804139	3.296020
C	-1.966433	-0.778486	3.490179
C	-0.946586	-2.826242	2.686958
C	-2.173386	-3.477811	2.820681
C	-3.183451	-1.443667	3.620204
H	-1.874080	0.264563	3.775073
H	-0.067494	-3.360611	2.343513
H	-2.244998	-4.531719	2.562813
H	-4.049184	-0.904437	3.998676
C	-4.613727	-3.533546	3.494944
H	-4.659878	-3.994445	4.490559
H	-4.741058	-4.337273	2.761826
H	-5.470464	-2.855695	3.413677
H	2.363239	3.322452	2.704109
C	1.464508	-0.952474	-0.473054
C	1.120263	-2.310573	-0.440262
C	2.670897	-0.613461	-1.107141
C	1.946539	-3.295737	-0.986869
H	0.191598	-2.615984	0.031752
C	3.497570	-1.593564	-1.665299
H	2.968660	0.428410	-1.163293
C	3.143076	-2.943232	-1.605539
H	3.786148	-3.702609	-2.035200
C	4.812492	-1.220013	-2.290931
C	1.502256	-4.730616	-0.945239
F	4.836733	0.050973	-2.731451
F	5.123141	-2.021886	-3.334887
F	5.848881	-1.346028	-1.405179
F	0.579547	-4.999325	-1.903525
F	0.922491	-5.043838	0.239243
F	2.524634	-5.592441	-1.133982
O	-0.952806	-0.184471	-0.012930
C	-1.378111	-0.398177	-1.236898
C	-2.531426	-1.323249	-1.405061
C	-3.382301	-1.624414	-0.333177
C	-2.741425	-1.940705	-2.647291
C	-4.431357	-2.524952	-0.509862
H	-3.199236	-1.170268	0.634632
C	-3.788213	-2.844159	-2.819252
H	-2.068233	-1.728573	-3.475694
C	-4.638986	-3.134052	-1.749961
H	-5.083648	-2.759350	0.326647
H	-3.932731	-3.329122	-3.780628
H	-5.453583	-3.841393	-1.880093
H	-0.597053	-0.533704	-1.992381
C	-1.911535	1.292906	-2.090885
H	-0.959409	1.805717	-1.994069
H	-2.135211	0.884633	-3.070526
C	-2.982958	1.849271	-1.373109
C	-4.387489	1.475001	-1.587731
C	-5.309001	1.606138	-0.528956
C	-4.841992	0.979007	-2.825546
C	-6.639827	1.243791	-0.701752
H	-4.958715	1.967779	0.430891
C	-6.177723	0.630882	-2.997561
H	-4.159095	0.893844	-3.663423
C	-7.078057	0.758016	-1.936929
H	-7.336935	1.336665	0.125925
H	-6.518085	0.262193	-3.960538
H	-8.119814	0.481665	-2.073675
O	-2.728452	2.647461	-0.351863
Si	-2.677386	4.392932	-0.256647
C	-1.480630	5.000379	-1.561139
H	-0.474310	4.623705	-1.350768
H	-1.778004	4.695714	-2.571650
H	-1.442336	6.097188	-1.547808
C	-2.142953	4.735420	1.500401
H	-1.082544	4.497327	1.625742
H	-2.282298	5.798101	1.734720
H	-2.729217	4.156523	2.223057
C	-4.421956	5.023018	-0.591169
H	-4.792230	4.726563	-1.578939
H	-5.142835	4.674826	0.156552
H	-4.418611	6.120268	-0.559764

TS 153

B3LYP/6-31G(d) = -3573.929909
B3LYP-D3(BJ)/def2-TZVPP/IEFPCM(propanonitrile) = -3575.502322
B3LYP-D3(BJ)/def2-TZVPP/IEFPCM(propanonitrile)//B3LYP-D3(BJ)/6-31G(d) Free Energy (Quasiharmonic) = -3574.785923

Frequencies (Top 3 out of 300)

1. -273.6805 cm^{-1}
2. 6.4692 cm^{-1}
3. 10.6593 cm^{-1}

B3LYP/6-31G(d) Molecular Geometry in Cartesian Coordinates

B	-0.209053	-0.639765	-0.380817
O	-0.439001	-1.969728	-0.971821

N	-0.463394	-0.950865	1.136597
C	-0.794440	-2.373312	1.332121
S	-0.063449	-0.016602	2.429222
C	-0.631654	-2.947608	-0.076391
O	-0.676344	-4.124949	-0.348853
H	-2.290276	-2.253874	2.885509
H	-0.060372	-2.848997	1.992500
C	-2.207903	-2.703595	1.890788
C	-3.374211	-2.304387	1.030415
C	-4.064600	-3.131380	0.062985
C	-5.095153	-2.335820	-0.507785
N	-5.047605	-1.098880	0.105205
C	-4.001391	-1.081930	1.006962
H	-3.791487	-0.189326	1.578448
C	-5.789539	-4.136330	-1.905316
C	-3.920455	-4.461862	-0.371483
H	-6.725417	-2.184454	-1.932514
H	-5.522805	-0.276920	-0.236055
C	-4.782895	-4.949716	-1.344454
H	-6.441071	-4.544724	-2.673401
H	-3.124769	-5.085738	0.023170
C	-5.958524	-2.819205	-1.496899
H	-4.673486	-5.973785	-1.691381
C	-1.544758	0.712010	3.148765
O	0.714672	1.124102	1.903355
O	0.524979	-0.876448	3.469745
C	-3.837185	1.850981	4.295491
C	-2.097336	1.866032	2.586925
C	-2.106464	0.137685	4.290198
C	-3.247756	0.708066	4.852262
C	-3.237557	2.422524	3.163039
H	-1.634898	2.339415	1.728654
H	-1.637251	-0.729273	4.742588
H	-3.681139	0.261893	5.744017
H	-3.660773	3.325383	2.729356
C	-5.090357	2.446160	4.891613
H	-5.133145	3.529107	4.736028
H	-5.153641	2.252905	5.967386
H	-5.988478	2.014762	4.429944
H	-2.218504	-3.789328	2.043057
C	-1.115675	0.521090	-1.032587
C	-2.305903	0.215008	-1.713567
C	-0.779628	1.874452	-0.907358
C	-3.127175	1.221889	-2.228212
H	-2.590168	-0.823559	-1.845447
C	-1.608582	2.886500	-1.404152
H	0.139167	2.147010	-0.400621
C	-2.788664	2.568297	-2.070303
H	-3.427876	3.349264	-2.466006
C	-1.246860	4.316790	-1.130740
C	-4.427206	0.886332	-2.902977
F	-1.958451	5.189741	-1.873586
F	0.066703	4.566687	-1.367310
F	-1.462483	4.640134	0.173030
F	-4.481436	-0.383216	-3.339877
F	-4.678610	1.697158	-3.954510
F	-5.489741	1.050935	-2.049012
O	1.250934	-0.255201	-0.726118
C	2.229874	-1.114134	-0.640293
C	3.153172	-1.217712	-1.794057
C	3.915098	-2.382391	-1.967659
C	3.224392	-0.195880	-2.752492
C	4.743601	-2.522563	-3.079507
H	3.833094	-3.190539	-1.245180
C	4.058436	-0.337134	-3.859399
H	2.606619	0.686775	-2.630016
C	4.820747	-1.496686	-4.024647
H	5.315042	-3.436392	-3.217449
H	4.104443	0.454090	-4.602486
H	5.461687	-1.607074	-4.895140
H	2.015606	-2.063919	-0.143821
C	3.362711	-0.525449	1.001372
H	3.566902	-1.486524	1.463190
H	2.540096	0.028558	1.435365
C	4.463582	0.187149	0.520243
C	4.394572	1.564983	0.025536
C	5.499455	2.118933	-0.654270
C	3.234991	2.348816	0.202137
C	5.443233	3.416143	-1.149079
H	6.387683	1.515570	-0.798967
C	3.186513	3.646362	-0.300759
H	2.380800	1.956869	0.740923
C	4.285668	4.181571	-0.976558
H	6.298492	3.831045	-1.674938
H	2.283923	4.235297	-0.174467
H	4.241212	5.194592	-1.367279
O	5.644393	-0.401872	0.355574
Si	6.549614	-1.519047	1.315674
C	6.420248	-0.955581	3.102979
H	6.785081	0.070700	3.223797
H	5.390690	-0.991447	3.475360
H	7.027353	-1.602379	3.748277
C	8.280015	-1.327003	0.620671
H	8.649956	-0.303627	0.748055
H	8.981311	-2.001503	1.126543
H	8.303896	-1.562972	-0.449223
C	5.932926	-3.280030	1.086740
H	4.922690	-3.438112	1.479523
H	5.937866	-3.569052	0.030659
H	6.599716	-3.968985	1.621037

TS 154

B3LYP/6-31G(d) = -3573.928277
B3LYP-D3(BJ)/def2-TZVPP/IEFPCM(propanonitrile) = -3575.502005
B3LYP-D3(BJ)/def2-TZVPP/IEFPCM(propanonitrile)//B3LYP-D3(BJ)/6-31G(d) Free Energy (Quasiharmonic) = -3574.785891

Frequencies (Top 3 out of 300)

1. -250.4541 cm^{-1}
2. 6.3370 cm^{-1}
3. 9.6987 cm^{-1}

B3LYP/6-31G(d) Molecular Geometry in Cartesian Coordinates

B	0.060832	0.996835	-0.283321
O	0.382015	2.185130	-1.102080
N	0.986072	1.238231	0.961917
C	1.596388	2.582706	0.887524
S	0.748226	0.547623	2.440613
C	1.085576	3.114924	-0.455147
O	1.301793	4.231083	-0.870882
H	3.453278	2.337810	1.966666
H	1.195552	3.227387	1.679670
C	3.147853	2.673156	0.969707
C	3.942654	1.941500	-0.078444
C	4.347311	2.418419	-1.386080
C	5.102570	1.378539	-1.995341
N	5.179912	0.338719	-1.090132
C	4.469968	0.678517	0.045946
H	4.420520	-0.006929	0.879431
C	5.450333	2.694807	-3.950992
C	4.168525	3.622258	-2.093139
H	6.216852	0.683715	-3.724432
H	5.479068	-0.597966	-1.314823
C	4.720314	3.747236	-3.361465
H	5.862406	2.821680	-4.948720
H	3.580559	4.427105	-1.665571
C	5.651885	1.497244	-3.276404
H	4.579298	4.671167	-3.915946

C	2.287221	-0.211986	2.978801
O	0.427586	1.588904	3.437357
O	-0.205920	-0.561056	2.241265
C	4.638091	-1.446457	3.877941
C	2.704264	-1.411805	2.395896
C	3.018714	0.368878	4.014167
C	4.188867	-0.251456	4.453992
C	3.874518	-2.016707	2.846367
H	2.126394	-1.866406	1.599084
H	2.662301	1.283463	4.475674
H	4.757518	0.199393	5.263504
H	4.198781	-2.947624	2.387558
C	5.919363	-2.099045	4.339036
H	5.833388	-3.190958	4.344398
H	6.753120	-1.843016	3.672082
H	6.195021	-1.773614	5.346951
H	3.372383	3.745721	0.922833
C	0.264040	-0.386734	-1.081556
C	1.519700	-1.002631	-1.154818
C	-0.790121	-1.017810	-1.754128
C	1.709329	-2.210456	-1.833732
C	-0.607894	-2.219671	-2.444115
H	-1.778326	-0.573222	-1.723804
C	0.644426	-2.833051	-2.485368
H	0.785135	-3.772115	-3.006533
C	-1.804249	-2.872416	-3.072639
C	3.080358	-2.822785	-1.847985
F	-2.660958	-3.369147	-2.124663
F	-1.479912	-3.907062	-3.873678
F	-2.536381	-2.002769	-3.803815
F	3.079578	-4.098520	-2.286054
F	3.638181	-2.827924	-0.603797
F	3.948650	-2.135888	-2.634816
O	-1.449695	1.114933	-0.022590
C	-2.058405	1.844994	0.864732
C	-3.029027	2.858808	0.391596
C	-3.507603	3.833559	1.283744
C	-3.417239	2.907855	-0.955166
C	-4.365662	4.834462	0.838318
H	-3.189669	3.813620	2.324237
C	-4.277179	3.911272	-1.397696
H	-3.015592	2.175877	-1.646524
C	-4.754804	4.872601	-0.504726
H	-4.720718	5.591963	1.531456
H	-4.565337	3.949528	-2.444309
H	-5.418666	5.658418	-0.854784
H	-1.491146	2.125782	1.757914
C	-3.240681	0.602166	2.059023
H	-3.880262	1.372172	2.469881
H	-2.440283	0.242880	2.694381
C	-3.776347	-0.305262	1.144741
C	-4.973153	-0.049579	0.327908
C	-5.917177	0.933493	0.683310
C	-5.186666	-0.805638	-0.843173
C	-7.041654	1.150734	-0.106566
H	-5.784365	1.519454	1.585801
C	-6.307018	-0.574703	-1.634922
H	-4.459219	-1.555193	-1.133181
C	-7.238955	0.399692	-1.267783
H	-7.765140	1.906560	0.184546
H	-6.452831	-1.155201	-2.541237
H	-8.116766	0.572930	-1.884298
O	-3.115108	-1.411247	0.848410
Si	-2.790573	-2.865498	1.738804
C	-2.704618	-2.487008	3.576668
H	-3.602594	-1.979571	3.947013
H	-2.609316	-3.429135	4.131843
H	-1.831516	-1.869322	3.807329
C	-1.191923	-3.558158	1.070186
H	-1.278400	-3.805891	0.009065
H	-0.385914	-2.830982	1.198187
H	-0.926978	-4.473481	1.614542
H	2.363846	-0.531386	-0.662262
C	-4.251648	-3.987018	1.346683
H	-5.205154	-3.548957	1.664043
H	-4.312522	-4.186736	0.270936
H	-4.143606	-4.951606	1.858592

TS 155

B3LYP/6-31G(d) = -3573.928835
B3LYP-D3(BJ)/def2-TZVPP/IEFPCM(propanonitrile) = -3575.50249
B3LYP-D3(BJ)/def2-TZVPP/IEFPCM(propanonitrile)//B3LYP-D3(BJ)/6-31G(d) Free Energy (Quasiharmonic) = -3574.785875

Frequencies (Top 3 out of 300)

1. -282.5640 cm^{-1}
2. 8.5749 cm^{-1}
3. 11.0544 cm^{-1}

B3LYP/6-31G(d) Molecular Geometry in Cartesian Coordinates

B	0.071246	-0.314517	0.521931
O	0.361893	-0.259881	1.980544
N	-0.149758	1.197950	0.158818
C	-0.113596	1.981883	1.408897
S	0.571195	1.883906	-1.173977
C	0.337471	0.974907	2.469027
O	0.608156	1.261382	3.621027
H	-1.743049	3.356487	1.058154
H	0.647224	2.767699	1.355320
C	-1.458064	2.638890	1.833849
C	-2.591771	1.683532	2.095655
C	-3.757462	1.459975	1.273393
C	-4.524894	0.438677	1.898144
N	-3.872260	0.090911	3.062421
C	-2.708668	0.827773	3.166630
H	-2.044874	0.689991	4.009109
C	-6.157977	0.523078	0.163828
C	-4.238854	2.025726	0.079778
H	-6.269825	-0.848322	1.835096
H	-4.090860	-0.724932	3.613228
C	-5.428744	1.555778	-0.461382
H	-7.079640	0.168487	-0.289858
H	-3.685924	2.817000	-0.417097
C	-5.717417	-0.047400	1.351825
H	-5.804927	1.985493	-1.386329
C	-0.505277	3.247482	-1.643128
O	0.507548	0.901545	-2.268165
O	1.875834	2.497582	-0.833827
C	-2.138968	5.366723	-2.475871
C	-1.663237	2.978004	-2.378544
C	-0.145851	4.558438	-1.334258
C	-0.968261	5.607898	-1.747085
C	-2.466965	4.037710	-2.789387
H	-1.924213	1.955634	-2.630870
H	0.776138	4.750666	-0.796282
H	-0.688120	6.630718	-1.507150
H	-3.364342	3.830211	-3.367595
C	-3.036013	6.501188	-2.910529
H	-2.541142	7.470534	-2.796296
H	-3.956861	6.526734	-2.313634
H	-3.335557	6.394246	-3.959301
H	-1.233290	3.222304	2.735892
C	-1.178868	-1.284138	0.201671
C	-1.715145	-1.355444	-1.093798
C	-1.782010	-2.070004	1.191820
C	-2.821856	-2.156202	-1.379937
H	-1.265859	-0.762416	-1.885183
C	-2.888809	-2.879110	0.908759

H	-1.392733	-2.036864	2.204540
C	-3.419417	-2.924096	-0.379356
H	-4.283522	-3.539328	-0.600065
C	-3.548674	-3.647878	2.016215
C	-3.342800	-2.216886	-2.789247
F	-4.447364	-2.884970	2.712765
F	-2.657076	-4.094019	2.929722
F	-4.238212	-4.717088	1.565813
F	-2.627791	-3.078753	-3.554009
F	-3.277473	-1.012560	-3.402618
F	-4.630682	-2.627234	-2.841990
O	1.312775	-0.804381	-0.187041
C	1.843885	-1.978507	0.023383
C	2.489614	-2.629430	-1.144781
C	2.637413	-1.927705	-2.350917
C	2.891837	-3.972796	-1.069284
C	3.198818	-2.565339	-3.456961
H	2.274399	-0.907297	-2.418623
C	3.448787	-4.604215	-2.177369
H	2.753702	-4.528140	-0.143360
C	3.608950	-3.897281	-3.373394
H	3.303332	-2.021744	-4.391731
H	3.748135	-5.646750	-2.114052
H	4.039639	-4.390343	-4.240832
H	1.294128	-2.656283	0.684656
C	3.318320	-1.823625	1.402340
H	2.720810	-1.769046	2.306978
H	3.805196	-2.768661	1.194892
C	3.975883	-0.645870	1.030440
C	4.964394	-0.577133	-0.052457
C	4.974341	0.555969	-0.891549
C	5.869549	-1.627042	-0.293672
C	5.879120	0.628442	-1.946804
H	4.234161	1.336265	-0.742189
C	6.783757	-1.535526	-1.338393
H	5.877981	-2.497000	0.355562
C	6.788833	-0.409402	-2.166414
H	5.868129	1.492665	-2.604482
H	7.492160	-2.341029	-1.508280
H	7.499196	-0.344164	-2.986100
O	3.555062	0.498274	1.532337
Si	4.244513	1.549817	2.734517
C	3.330441	3.168096	2.535664
H	3.897294	3.988319	2.992739
H	3.175855	3.401621	1.476625
H	2.354206	3.110302	3.027070
C	6.084559	1.720747	2.370933
H	6.270894	2.180970	1.394859
H	6.545950	2.359304	3.135166
H	6.605951	0.757006	2.391762
C	3.939609	0.725177	4.390726
H	2.862702	0.591600	4.541598
H	4.431347	-0.252179	4.463854
H	4.323136	1.349924	5.207388

TS 156

B3LYP/6-31G(d) = -3573.925761
B3LYP-D3(BJ)/def2-TZVPP/IEFPCM(propanonitrile) = -3575.501764
B3LYP-D3(BJ)/def2-TZVPP/IEFPCM(propanonitrile)//B3LYP-D3(BJ)/6-31G(d) Free Energy (Quasiharmonic) = -3574.785856

Frequencies (Top 3 out of 300)

1. -257.1270 cm^{-1}
2. 6.6590 cm^{-1}
3. 10.0182 cm^{-1}

B3LYP/6-31G(d) Molecular Geometry in Cartesian Coordinates

B	0.468046	-0.685882	-0.234499
O	0.391652	0.567752	-1.038249
N	-0.471127	-1.614272	-1.058112
C	-0.841430	-0.977280	-2.332436
S	-0.401663	-3.277951	-0.987701
C	-0.254176	0.425946	-2.196904
O	-0.348860	1.313734	-3.020729
H	-2.707496	-1.953733	-2.767416
H	-0.336422	-1.478788	-3.168640
C	-2.350483	-0.922035	-2.686211
C	-3.244124	-0.134267	-1.767075
C	-3.715526	1.220537	-1.960269
C	-4.599769	1.519265	-0.885550
N	-4.650987	0.399363	-0.081158
C	-3.841179	-0.585794	-0.614200
H	-3.768499	-1.550190	-0.133258
C	-5.030221	3.691833	-1.769121
C	-3.501012	2.198993	-2.947995
H	-5.935020	2.955006	0.052596
H	-5.177930	0.311868	0.773441
C	-4.161312	3.417395	-2.845939
H	-5.533407	4.653808	-1.718327
H	-2.812371	2.008221	-3.764931
C	-5.258683	2.749046	-0.773645
H	-4.009327	4.174504	-3.611050
C	-2.038320	-3.832092	-0.471611
O	0.501668	-3.630987	0.112051
O	-0.196681	-3.807013	-2.345584
C	-4.549250	-4.759946	0.366575
C	-2.882653	-4.461541	-1.384956
C	-2.425186	-3.672801	0.861348
C	-3.675900	-4.130998	1.268645
C	-4.132867	-4.915187	-0.961537
H	-2.552417	-4.602798	-2.408655
H	-1.751221	-3.198204	1.566757
H	-3.977514	-4.005841	2.306223
H	-4.791439	-5.404451	-1.675210
C	-5.887468	-5.287876	0.826879
H	-6.300423	-4.682442	1.640828
H	-5.796856	-6.316141	1.201421
H	-6.615828	-5.302596	0.009620
H	-2.407508	-0.497935	-3.695156
C	2.008503	-1.121684	-0.031551
C	2.814181	-1.390419	-1.148301
C	2.627938	-1.165075	1.222398
C	4.170562	-1.694605	-1.018376
H	2.381114	-1.375454	-2.144829
C	3.986776	-1.461397	1.361099
H	2.040119	-0.971528	2.112929
C	4.769660	-1.731969	0.241241
H	5.822901	-1.965014	0.344754
C	4.626309	-1.374315	2.716428
C	5.019897	-1.864134	-2.246135
F	5.758355	-2.104767	2.804739
F	3.799079	-1.784786	3.703871
F	4.969171	-0.089129	3.016749
F	4.334646	-2.397755	-3.278043
F	6.097097	-2.648509	-2.020911
F	5.497674	-0.663608	-2.676175
O	-0.124173	-0.428635	1.149585
C	-0.899813	0.566126	1.458110
C	-1.806043	0.367296	2.618636
C	-2.911165	1.216163	2.788370
C	-1.584450	-0.670906	3.534545
C	-3.782210	1.027455	3.859662
H	-3.085456	2.019113	2.076015
C	-2.458191	-0.856086	4.605123
H	-0.732529	-1.328213	3.394067
C	-3.556644	-0.008687	4.771170
H	-4.635063	1.688948	3.987107
H	-2.280880	-1.662067	5.311757

H	-4.234399	-0.153602	5.607993
H	-1.289982	1.177094	0.644100
C	0.314625	2.064859	2.289560
H	1.010651	1.404373	2.796954
H	-0.449367	2.529263	2.899519
C	0.794207	2.817049	1.215428
C	-0.009782	3.937907	0.669547
C	-0.347551	3.981972	-0.694777
C	-0.484276	4.940141	1.535085
C	-1.146062	5.019162	-1.175752
H	-0.039873	3.185377	-1.364302
C	-1.255632	5.988045	1.038468
H	-0.220146	4.910499	2.588433
C	-1.590590	6.026376	-0.317476
H	-1.435812	5.023118	-2.221486
H	-1.598466	6.770744	1.709363
H	-2.205629	6.835469	-0.701595
O	1.916193	2.458729	0.641671
Si	3.176606	3.242335	-0.279273
C	4.632080	2.084543	-0.073052
H	4.709822	1.698954	0.948804
H	4.567885	1.227365	-0.750808
H	5.563370	2.617345	-0.304790
C	3.479920	4.898159	0.564393
H	4.301270	5.423854	0.061417
H	2.600318	5.549632	0.529447
H	3.767246	4.766167	1.613975
C	2.697775	3.440077	-2.082360
H	2.148486	2.569296	-2.455533
H	2.080224	4.326789	-2.255706
H	3.611195	3.543308	-2.682312

TS 157

B3LYP/6-31G(d) = -3573.927785
B3LYP-D3(BJ)/def2-TZVPP/IEFPCM(propanonitrile) = -3575.501684
B3LYP-D3(BJ)/def2-TZVPP/IEFPCM(propanonitrile)//B3LYP-D3(BJ)/6-31G(d) Free Energy (Quasiharmonic) = -3574.785742

Frequencies (Top 3 out of 300)

1. -255.1489 cm^{-1}
2. 7.2788 cm^{-1}
3. 12.7605 cm^{-1}

B3LYP/6-31G(d) Molecular Geometry in Cartesian Coordinates

B	0.216710	0.044930	0.625590
O	0.393910	-0.000950	2.084170
N	0.873230	-1.322340	0.192490
C	1.211380	-2.106770	1.397050
S	0.436300	-2.126390	-1.178440
C	0.777260	-1.189480	2.543480
O	0.799970	-1.508650	3.712660
H	2.935930	-3.259180	0.782830
H	0.605240	-3.019590	1.448220
C	2.701490	-2.526800	1.562100
C	3.720310	-1.421590	1.540160
C	4.189950	-0.617680	2.649510
C	5.154710	0.289020	2.129190
N	5.285190	0.025280	0.779770
C	4.410250	-0.986710	0.434930
H	4.365760	-1.342280	-0.585210
C	5.510511	1.238160	4.286290
C	3.907540	-0.583090	4.027720
H	6.545251	1.912589	2.509380
H	5.732060	0.636969	0.113420
C	4.568420	0.339940	4.828040
H	6.005421	1.953109	4.938380
H	3.161530	-1.246710	4.452520
C	5.816821	1.224529	2.931040
H	4.349540	0.377890	5.891850
C	1.914920	-2.804040	-1.942710
O	-0.413580	-3.292360	-0.841470
O	-0.089530	-1.114330	-2.113590
C	4.199730	-3.860830	-3.174870
C	2.245540	-4.145340	-1.748350
C	2.697470	-1.986640	-2.762460
C	3.834410	-2.519110	-3.365770
C	3.387340	-4.661400	-2.361080
H	1.607970	-4.775160	-1.137300
H	2.417850	-0.953450	-2.932900
H	4.445510	-1.880400	-3.998920
H	3.647519	-5.705710	-2.207230
C	5.418910	-4.432270	-3.858110
H	6.232870	-3.700861	-3.901730
H	5.788380	-5.322921	-3.340400
H	5.189540	-4.724030	-4.891520
H	2.746950	-3.063370	2.517830
C	0.857581	1.367210	-0.042890
C	2.032121	1.305030	-0.802170
C	0.300761	2.639180	0.159990
C	2.617601	2.447790	-1.352420
C	0.879791	3.789220	-0.385710
H	-0.597239	2.744420	0.760540
C	2.042211	3.702150	-1.151140
H	2.489191	4.592660	-1.577370
C	0.204641	5.121090	-0.205020
C	3.901571	2.327370	-2.120470
F	1.073191	6.152110	-0.316980
F	-0.760429	5.323160	-1.137320
F	-0.395409	5.226170	1.000840
F	4.997361	2.361370	-1.306430
F	3.983651	1.155530	-2.801420
F	4.066001	3.323390	-3.014740
O	-1.333500	0.112661	0.513640
C	-2.082440	0.399621	-0.511960
C	-3.101809	1.461191	-0.347640
C	-3.622899	2.095891	-1.485450
C	-3.505829	1.887771	0.927300
C	-4.535899	3.141081	-1.353180
H	-3.289019	1.785661	-2.473190
C	-4.421909	2.929511	1.055600
H	-3.078409	1.409901	1.802780
C	-4.939999	3.556901	-0.081320
H	-4.917549	3.642541	-2.238280
H	-4.723439	3.262701	2.044730
H	-5.642879	4.378901	0.023490
H	-1.628790	0.306731	-1.499870
C	-3.206820	-1.323279	-0.934710
H	-2.352150	-1.985499	-0.895970
H	-3.553970	-1.075539	-1.931620
C	-4.154400	-1.360759	0.084920
C	-3.868300	-1.796999	1.453290
C	-4.790260	-1.512959	2.483140
C	-2.670610	-2.468629	1.775860
C	-4.514240	-1.875449	3.795520
H	-5.707240	-0.988959	2.241200
C	-2.400590	-2.824749	3.094490
H	-1.961370	-2.730219	0.999070
C	-3.315910	-2.526309	4.105350
H	-5.227100	-1.642409	4.581480
H	-1.463710	-3.314079	3.339800
H	-3.093770	-2.797659	5.133500
O	-5.360690	-0.820629	-0.088250
Si	-6.611040	-0.956729	-1.260760
C	-6.034460	-0.514609	-2.995840
H	-6.905940	-0.469799	-3.661940
H	-5.549760	0.466881	-3.019940
H	-5.343310	-1.252879	-3.416710
C	-7.877220	0.275692	-0.636500

H	-8.750270	0.312892	-1.299030
H	-8.229470	0.013142	0.367210
H	-7.446169	1.282002	-0.588920
H	2.503330	0.342660	-0.951770
C	-7.216280	-2.735089	-1.187100
H	-8.053450	-2.887638	-1.879400
H	-6.424930	-3.440259	-1.467080
H	-7.560770	-2.999948	-0.181330

TS 158

B3LYP/6-31G(d) = -3573.928032
B3LYP-D3(BJ)/def2-TZVPP/IEFPCM(propanonitrile) = -3575.503217
B3LYP-D3(BJ)/def2-TZVPP/IEFPCM(propanonitrile)//B3LYP-D3(BJ)/6-31G(d) Free Energy (Quasiharmonic) = -3574.785692

Frequencies (Top 3 out of 300)

1. -296.8855 cm^{-1}
2. 12.3822 cm^{-1}
3. 14.6497 cm^{-1}

B3LYP/6-31G(d) Molecular Geometry in Cartesian Coordinates

B	0.709666	0.775704	-0.685275
O	1.107365	0.790003	-2.107870
N	1.227824	2.144306	-0.192415
C	2.036942	2.786819	-1.239209
S	0.805099	2.975080	1.156573
C	1.821740	1.868917	-2.448407
O	2.250862	2.083522	-3.558504
H	3.639258	3.786012	-0.202455
H	1.626989	3.770616	-1.508430
C	3.550753	2.972583	-0.927203
C	4.250552	1.754273	-0.398503
C	4.883232	0.687744	-1.141751
C	5.380238	-0.246773	-0.189303
N	5.080588	0.245462	1.065732
C	4.385364	1.431472	0.929725
H	4.034011	1.974322	1.796176
C	6.233151	-1.642417	-1.924971
C	5.084678	0.429157	-2.510088
H	6.423928	-2.114863	0.178420
H	5.139263	-0.282251	1.923709
C	5.754537	-0.728716	-2.886308
H	6.751368	-2.540470	-2.251277
H	4.698778	1.112111	-3.261212
C	6.053325	-1.413724	-0.565418
H	5.908936	-0.938536	-3.941501
C	-0.301884	4.300945	0.642309
O	1.985648	3.650203	1.718630
O	0.024317	2.051512	2.001452
C	-2.020381	6.365269	-0.173213
C	-1.681544	4.089641	0.628311
C	0.227333	5.540014	0.274293
C	-0.633294	6.557865	-0.135730
C	-2.528115	5.120554	0.226909
H	-2.080545	3.136387	0.955767
H	1.296155	5.712327	0.340389
H	-0.218708	7.522296	-0.419063
H	-3.604026	4.960132	0.232340
C	-2.943524	7.464126	-0.643176
H	-2.526495	8.454863	-0.435302
H	-3.924011	7.399284	-0.159731
H	-3.109505	7.401820	-1.726959
H	4.010638	3.314883	-1.862275
C	1.286487	-0.566063	0.025528
C	1.636011	-0.654303	1.383141
C	1.407509	-1.739309	-0.735761
C	2.074705	-1.857181	1.946724
H	1.552072	0.223154	2.014047
C	1.758629	-2.961961	-0.156316
H	1.215833	-1.703889	-1.804334
C	2.109496	-3.029671	1.190169
H	2.401690	-3.971244	1.639892
C	1.621101	-4.213877	-0.969947
C	2.561187	-1.880395	3.368344
F	2.036764	-4.062191	-2.240640
F	0.297051	-4.585946	-1.047801
F	2.276894	-5.264638	-0.440145
F	3.889528	-1.567423	3.448877
F	2.428583	-3.099843	3.936351
F	1.913788	-0.994438	4.149613
O	-0.838410	0.782253	-0.668098
C	-1.494244	-0.177275	-1.276837
C	-2.809562	0.168830	-1.874525
C	-3.341079	-0.652953	-2.879353
C	-3.502478	1.326629	-1.497016
C	-4.553507	-0.332716	-3.486911
H	-2.788407	-1.531723	-3.201216
C	-4.716713	1.643011	-2.103538
H	-3.066742	1.984909	-0.755477
C	-5.247906	0.814119	-3.094489
H	-4.948728	-0.967651	-4.275132
H	-5.244663	2.546568	-1.811159
H	-6.191056	1.068619	-3.570219
H	-0.897859	-0.853673	-1.892257
C	-1.776659	-1.639866	0.092560
H	-1.009272	-1.268901	0.756843
H	-1.472188	-2.519856	-0.465194
C	-3.095330	-1.629408	0.571574
C	-3.595595	-0.649683	1.536888
C	-2.739537	0.320480	2.100304
C	-4.950927	-0.676027	1.927441
C	-3.229918	1.230449	3.032375
H	-1.695922	0.391503	1.819365
C	-5.431956	0.235535	2.859360
H	-5.613560	-1.412861	1.488749
C	-4.572523	1.188630	3.416662
H	-2.550524	1.964979	3.453320
H	-6.476960	0.205128	3.154333
H	-4.949966	1.895512	4.150692
O	-4.000131	-2.471674	0.097040
Si	-3.902179	-4.146730	-0.370848
C	-5.699649	-4.666852	-0.257743
H	-5.820799	-5.715740	-0.553755
H	-6.083259	-4.561771	0.763188
H	-6.328805	-4.059415	-0.918151
C	-3.246290	-4.334327	-2.120551
H	-3.394953	-5.373045	-2.443370
H	-3.778817	-3.691557	-2.828675
H	-2.172857	-4.128485	-2.193915
C	-2.803318	-5.030364	0.870261
H	-1.740740	-4.832100	0.695922
H	-2.951028	-6.113835	0.777897
H	-3.043733	-4.752950	1.902723

TS 159

B3LYP/6-31G(d) = -3573.927987
B3LYP-D3(BJ)/def2-TZVPP/IEFPCM(propanonitrile) = -3575.501709
B3LYP-D3(BJ)/def2-TZVPP/IEFPCM(propanonitrile)//B3LYP-D3(BJ)/6-31G(d) Free Energy (Quasiharmonic) = -3574.785681

Frequencies (Top 3 out of 300)

1. -271.6691 cm^{-1}
2. 5.8258 cm^{-1}
3. 11.7823 cm^{-1}

B3LYP/6-31G(d) Molecular Geometry in Cartesian Coordinates

B	0.681570	-0.548821	0.868841
O	0.079416	-0.220148	2.171857
N	-0.317464	0.137857	-0.119418
C	-1.231611	1.019986	0.639797
S	0.030035	0.512216	-1.705736
C	-0.965625	0.604707	2.093506
O	-1.614394	0.966033	3.051168
H	-3.176055	0.083644	0.841561
H	-0.922787	2.071304	0.541829
C	-2.740801	0.919235	0.284424
C	-3.510296	2.188091	0.536266
C	-4.012371	3.089818	-0.474456
C	-4.676226	4.154016	0.199063
N	-4.572539	3.901156	1.551272
C	-3.867346	2.728072	1.747908
H	-3.642105	2.367413	2.740833
C	-5.223506	5.194443	-1.876413
C	-3.962789	3.105555	-1.881326
H	-5.790753	6.012222	0.044518
H	-4.918132	4.498447	2.285820
C	-4.566159	4.154670	-2.565627
H	-5.686283	6.000623	-2.439419
H	-3.446375	2.316935	-2.422900
C	-5.286882	5.208631	-0.487183
H	-4.529785	4.177595	-3.651594
C	0.877397	2.093085	-1.752979
O	-1.246038	0.696081	-2.424010
O	0.965683	-0.526788	-2.166834
C	2.218560	4.550518	-1.663884
C	0.141090	3.278464	-1.672891
C	2.272772	2.121306	-1.834379
C	2.928704	3.347290	-1.791422
C	0.819926	4.496417	-1.624164
H	-0.943726	3.257019	-1.668283
H	2.830718	1.197452	-1.931039
H	4.014138	3.369669	-1.843163
H	0.248479	5.418363	-1.552790
C	2.957576	5.861491	-1.549910
H	2.274414	6.715271	-1.590424
H	3.695614	5.977472	-2.352283
H	3.502883	5.909231	-0.598965
O	0.682856	-2.084608	0.746904
C	0.267226	-2.777671	-0.271146
H	-0.243962	-2.239408	-1.065043
H	-2.807613	0.667751	-0.774662
C	1.112712	-3.923130	-0.696173
C	2.017228	-4.525793	0.191408
C	1.037861	-4.377909	-2.020978
C	2.820723	-5.578428	-0.239100
H	2.095464	-4.148561	1.206108
C	1.846850	-5.428372	-2.449627
H	0.369530	-3.883294	-2.721172
C	2.734474	-6.034754	-1.557791
H	3.523463	-6.037733	0.450334
H	1.793912	-5.765159	-3.481033
H	3.366967	-6.852392	-1.892179
C	2.232929	-0.079275	0.832585
C	3.231070	-0.763063	0.126548
C	2.619483	1.085921	1.515123
C	4.550308	-0.299948	0.088635
H	2.983698	-1.669247	-0.414372
C	3.932644	1.558797	1.475142
H	1.885750	1.635810	2.095576
C	4.910858	0.866085	0.760514
H	5.934166	1.222898	0.736510
C	4.294141	2.863099	2.129344
C	5.560069	-1.021730	-0.758797
F	5.550549	2.847191	2.631801
F	3.457851	3.184873	3.137806
F	6.831719	-0.755297	-0.382322
F	4.251630	3.893252	1.241188
F	5.454875	-0.663778	-2.063293
F	5.398367	-2.363312	-0.711240
C	-1.413956	-3.923714	0.248560
H	-0.901916	-4.568739	0.950899
H	-1.563792	-4.357539	-0.733380
C	-2.433950	-3.112977	0.755191
O	-3.371856	-2.589559	-0.018518
Si	-4.027487	-2.909573	-1.592963
C	-2.759479	-2.572097	-2.931948
H	-3.256229	-2.609563	-3.910372
H	-2.310691	-1.576851	-2.831536
H	-1.954923	-3.315308	-2.952320
C	-4.611318	-4.699624	-1.590445
H	-5.340470	-4.878455	-0.792066
H	-5.100370	-4.934860	-2.543934
H	-3.788853	-5.411375	-1.459631
C	-5.458054	-1.704313	-1.672222
H	-6.181028	-1.884149	-0.868686
H	-5.108490	-0.669745	-1.585986
H	-5.989951	-1.797260	-2.626845
C	-2.528979	-2.731514	2.168153
C	-3.717530	-2.150718	2.656802
C	-1.458247	-2.943750	3.059182
C	-3.832669	-1.805003	3.997152
H	-4.543422	-1.982000	1.975701
C	-1.575795	-2.580528	4.396190
H	-0.519324	-3.346575	2.700178
C	-2.761110	-2.015436	4.869251
H	-4.754055	-1.361085	4.362364
H	-0.734077	-2.723631	5.066621
H	-2.847265	-1.730759	5.913994

TS 160

B3LYP/6-31G(d) = -3573.9278
B3LYP-D3(BJ)/def2-TZVPP/IEFPCM(propanonitrile) = -3575.501662
B3LYP-D3(BJ)/def2-TZVPP/IEFPCM(propanonitrile)//B3LYP-D3(BJ)/6-31G(d) Free Energy (Quasiharmonic) = -3574.785644

Frequencies (Top 3 out of 300)

1. -254.9056 cm^{-1}
2. 7.3109 cm^{-1}
3. 12.7117 cm^{-1}

B3LYP/6-31G(d) Molecular Geometry in Cartesian Coordinates

B	0.216853	0.044729	0.623293
O	0.394840	-0.000556	2.081763
N	0.874098	-1.322176	0.190147
C	1.214339	-2.105606	1.394776
S	0.435692	-2.127734	-1.179438
C	0.780381	-1.188317	2.541282
O	0.804947	-1.506798	3.710587
H	2.940010	-3.254999	0.778272
H	0.609371	-3.019159	1.447016
C	2.705211	-2.523471	1.558216
C	3.721933	-1.416307	1.536235
C	4.193190	-0.614810	2.646622
C	5.154395	0.295615	2.126249
N	5.281324	0.036439	0.775578
C	4.407402	-0.976298	0.430281
H	4.359993	-1.328187	-0.590991
C	5.514387	1.238807	4.285271
C	3.914558	-0.585035	4.025686
H	6.542546	1.921004	2.507506
H	5.723927	0.651588	0.109533
C	4.575733	0.336961	4.826963

H	6.009634	1.952815	4.938144
H	3.170983	-1.251411	4.450470
C	5.816833	1.230038	2.929100
H	4.359743	0.371271	5.891486
C	1.914338	-2.808393	-1.941103
O	-0.089047	-1.116409	-2.115967
O	-0.415726	-3.292115	-0.840730
C	4.195433	-3.872491	-3.174189
C	2.697575	-1.994344	-2.763270
C	2.239576	-4.151510	-1.748815
C	3.377873	-4.671832	-2.364009
C	3.831459	-2.531164	-3.368968
H	2.417093	-0.962333	-2.939557
H	1.596501	-4.780706	-1.142924
H	3.629708	-5.719252	-2.217486
H	4.439045	-1.896958	-4.009873
C	5.443628	-4.434165	-3.811439
H	6.324599	-4.240403	-3.185321
H	5.372015	-5.517945	-3.947277
H	5.634267	-3.979064	-4.788997
H	2.752547	-3.060509	2.513577
C	0.856289	1.367469	-0.045542
C	2.027066	1.305789	-0.810551
C	0.301745	2.639553	0.162933
C	2.611583	2.449259	-1.360312
C	0.879640	3.790269	-0.382509
H	-0.593442	2.744217	0.767774
C	2.038656	3.703777	-1.153201
H	2.484921	4.594873	-1.578979
C	0.206988	5.122553	-0.195600
C	3.892328	2.329315	-2.133738
F	-0.389184	5.224793	1.012410
F	-0.760589	5.328958	-1.124322
F	1.076755	6.152615	-0.307058
F	3.971455	1.158292	-2.816225
F	4.991439	2.362222	-1.323879
F	4.053574	3.326370	-3.027394
O	-1.333425	0.111639	0.511898
C	-2.082630	0.398003	-0.513630
C	-3.101657	1.459926	-0.349996
C	-3.623294	2.093355	-1.488262
C	-3.504856	1.888095	0.924674
C	-4.536000	3.138890	-1.356698
H	-3.290101	1.781825	-2.475826
C	-4.420631	2.930180	1.052274
H	-3.077095	1.411118	1.800480
C	-4.939240	3.556326	-0.085093
H	-4.918102	3.639342	-2.242173
H	-4.721534	3.264605	2.041175
H	-5.641891	4.378590	0.019141
H	-1.629533	0.303803	-1.501688
C	-3.207991	-1.325476	-0.934131
H	-2.353294	-1.987682	-0.895405
H	-3.555907	-1.078477	-1.930962
C	-4.154574	-1.361505	0.086417
C	-3.867319	-1.796311	1.455018
C	-2.669369	-2.467635	1.777293
C	-4.788378	-1.511125	2.485359
C	-2.398231	-2.822329	3.096076
H	-1.960785	-2.730035	1.000183
C	-4.511245	-1.872221	3.797894
H	-5.705551	-0.987367	2.243641
C	-3.312672	-2.522787	4.107402
H	-1.461155	-3.311429	3.341105
H	-5.223437	-1.638322	4.584206
H	-3.089670	-2.793033	5.135650
O	-5.360900	-0.821188	-0.086215
Si	-6.612317	-0.958639	-1.257403
C	-6.037385	-0.518192	-2.993462
H	-6.909490	-0.474002	-3.658784
H	-5.552691	0.463267	-3.018946
H	-5.346641	-1.256868	-3.414280
C	-7.878030	0.274316	-0.633236
H	-8.229282	0.012807	0.371092
H	-7.447043	1.280721	-0.587147
H	-8.751717	0.310727	-1.294965
H	2.496228	0.343241	-0.965039
C	-7.217357	-2.736968	-1.181418
H	-6.426174	-3.442353	-1.461344
H	-7.560974	-3.000837	-0.175087
H	-8.055095	-2.890306	-1.872859

TS 161

B3LYP/6-31G(d) = -3573.929671
B3LYP-D3(BJ)/def2-TZVPP/IEFPCM(propanonitrile) = -3575.502103
B3LYP-D3(BJ)/def2-TZVPP/IEFPCM(propanonitrile)//B3LYP-D3(BJ)/6-31G(d) Free Energy (Quasiharmonic) = -3574.785635

Frequencies (Top 3 out of 300)

1. -285.1555 cm^{-1}
2. 4.3885 cm^{-1}
3. 11.5548 cm^{-1}

B3LYP/6-31G(d) Molecular Geometry in Cartesian Coordinates

B	-0.326942	-0.486315	-0.491946
O	-0.006729	-0.091325	-1.884647
N	-1.535861	-1.461509	-0.679948
C	-1.891440	-1.500603	-2.109632
S	-1.605858	-2.877501	0.189705
C	-0.814317	-0.651378	-2.792701
O	-0.712979	-0.489414	-3.987770
H	-4.041876	-1.702520	-2.099062
H	-1.803036	-2.514857	-2.518492
C	-3.314949	-0.957117	-2.436933
C	-3.636868	0.376456	-1.824983
C	-3.360783	1.695594	-2.349665
C	-3.799028	2.635587	-1.373813
N	-4.338197	1.917429	-0.323911
C	-4.214518	0.568729	-0.594891
H	-4.561296	-0.166161	0.117691
C	-3.089497	4.460225	-2.734820
C	-2.786619	2.177288	-3.540580
H	-3.996047	4.717091	-0.787784
H	-4.556197	2.291245	0.587798
C	-2.657781	3.549237	-3.720429
H	-2.966780	5.527200	-2.900599
H	-2.423457	1.485390	-4.294588
C	-3.667162	4.016333	-1.550735
H	-2.208943	3.929001	-4.634543
C	-3.348892	-3.329686	0.208092
O	-0.910347	-3.982425	-0.500262
O	-1.232687	-2.541112	1.573712
C	-6.055602	-4.058748	0.279551
C	-3.837753	-4.261007	-0.707558
C	-4.188206	-2.779143	1.179690
C	-5.531694	-3.145615	1.207650
C	-5.186995	-4.614100	-0.668900
H	-3.162703	-4.715683	-1.424845
H	-3.783627	-2.092876	1.916346
H	-6.183637	-2.724117	1.969267
H	-5.567864	-5.341249	-1.382005
C	-7.521073	-4.422741	0.299026
H	-8.118651	-3.677416	-0.242456
H	-7.700027	-5.392595	-0.175860
H	-7.909552	-4.467350	1.322139
O	0.845720	-1.306914	0.011304
C	1.941925	-0.853884	0.557474
H	1.904866	0.153669	0.984246

H	-3.387811	-0.915092	-3.530457
C	2.721767	-1.853534	1.333385
C	3.743882	-1.436242	2.199493
C	2.391591	-3.217069	1.256584
C	4.434926	-2.367442	2.973357
H	3.987976	-0.379957	2.280469
C	3.089614	-4.143688	2.029498
H	1.570322	-3.531922	0.621161
C	4.112392	-3.724251	2.885297
H	5.215192	-2.033545	3.651776
H	2.820611	-5.194911	1.976969
H	4.645212	-4.449943	3.494057
C	-0.616524	0.826348	0.409547
C	-0.219811	2.097305	-0.034515
C	-1.331911	0.763010	1.614466
C	-0.482652	3.251206	0.708327
H	0.273149	2.190537	-0.996173
C	-1.654850	1.921800	2.326964
H	-1.654028	-0.204144	1.987365
C	-1.215110	3.172483	1.891934
H	-1.445373	4.066690	2.461764
C	-2.590572	1.841864	3.497749
C	-0.013183	4.598681	0.238522
F	-2.372743	2.819907	4.404390
F	-2.520424	0.661800	4.143942
F	0.807139	4.513135	-0.837444
F	-3.889309	1.982035	3.089771
F	0.692809	5.241970	1.209540
F	-1.030689	5.419130	-0.095473
C	3.035027	-0.342630	-1.033462
H	2.330232	0.387849	-1.411062
H	2.985143	-1.294616	-1.548427
C	4.300919	0.096813	-0.622927
O	5.320133	-0.738363	-0.530634
Si	5.944797	-2.095697	-1.416177
C	6.697430	-1.355057	-2.970267
H	7.172722	-2.137010	-3.575258
H	7.460202	-0.605535	-2.732346
H	5.935205	-0.874959	-3.594933
C	7.224600	-2.767263	-0.224308
H	7.745716	-3.629159	-0.658019
H	7.977901	-2.011950	0.025635
H	6.750248	-3.094974	0.707439
C	4.646124	-3.385641	-1.828254
H	4.067726	-3.683905	-0.947973
H	3.947915	-3.061031	-2.607050
H	5.156734	-4.281168	-2.206722
C	4.570877	1.446078	-0.102619
C	5.828282	1.736078	0.466555
C	3.598554	2.466084	-0.148926
C	6.098635	3.001133	0.975714
H	6.580225	0.957285	0.509997
C	3.872671	3.730020	0.364579
H	2.625582	2.284666	-0.589000
C	5.121975	4.000018	0.928168
H	7.070762	3.209274	1.413392
H	3.106680	4.496663	0.329034
H	5.333935	4.987434	1.328652

TS 162

B3LYP/6-31G(d) = -3573.928631
B3LYP-D3(BJ)/def2-TZVPP/IEFPCM(propanonitrile) = -3575.502423
B3LYP-D3(BJ)/def2-TZVPP/IEFPCM(propanonitrile)//B3LYP-D3(BJ)/6-31G(d) Free Energy (Quasiharmonic) = -3574.785627

Frequencies (Top 3 out of 300)

1. -274.9220 cm^{-1}
2. 9.9118 cm^{-1}
3. 11.6676 cm^{-1}

B3LYP/6-31G(d) Molecular Geometry in Cartesian Coordinates

B	-0.381825	0.623746	-0.675951
O	-0.774405	0.229852	-2.041723
N	0.917803	1.468569	-0.944189
C	1.178684	1.519819	-2.393829
S	1.346557	2.782051	-0.021282
C	0.004650	0.750096	-3.000352
O	-0.189145	0.589710	-4.184651
H	3.341302	1.477603	-2.458061
H	1.129492	2.552772	-2.760346
C	2.517357	0.897930	-2.884922
C	2.717550	-0.565445	-2.581782
C	3.754443	-1.147106	-1.761026
C	3.550472	-2.555252	-1.752900
N	2.463438	-2.813888	-2.561117
C	1.962649	-1.618897	-3.043550
H	1.108326	-1.614410	-3.705396
C	5.439412	-2.879321	-0.334388
C	4.847802	-0.623589	-1.049590
H	4.196540	-4.502776	-1.042170
H	1.969835	-3.692550	-2.598241
C	5.675458	-1.489283	-0.345536
H	6.102055	-3.530978	0.229039
H	5.044759	0.444143	-1.045520
C	4.376355	-3.430564	-1.039918
H	6.521376	-1.089991	0.208113
C	3.115888	2.674138	0.297640
O	0.694023	2.619412	1.290271
O	1.145003	4.024036	-0.788514
C	5.852383	2.642801	0.904764
C	3.584591	1.857607	1.329675
C	3.994526	3.477245	-0.430313
C	5.356207	3.449368	-0.128094
C	4.945666	1.849272	1.623421
H	2.898236	1.245282	1.904097
H	3.608282	4.130178	-1.205723
H	6.040823	4.075589	-0.695279
H	5.310134	1.213317	2.426313
C	7.320668	2.651168	1.259110
H	7.937720	2.951864	0.406320
H	7.658305	1.664185	1.592832
H	7.524518	3.356543	2.075768
H	2.540652	1.073162	-3.968215
C	-0.147877	-0.655196	0.279407
C	-0.280713	-1.954176	-0.230335
C	0.276982	-0.532962	1.610462
C	0.002586	-3.081634	0.546713
H	-0.596776	-2.084643	-1.259086
C	0.576838	-1.656915	2.386550
H	0.405529	0.458570	2.034193
C	0.440001	-2.942699	1.863922
H	0.683938	-3.811875	2.462889
C	1.012521	-1.458886	3.809794
C	-0.225754	-4.450549	-0.023156
F	1.901055	-0.444922	3.931847
F	1.583349	-2.562198	4.338121
F	-0.039693	-1.140239	4.614065
F	-1.532814	-4.830580	0.077691
F	0.072492	-4.519314	-1.349155
F	0.497792	-5.403766	0.597128
O	-1.501097	1.511306	-0.104795
C	-2.471587	2.008844	-0.820369
C	-2.872290	3.412109	-0.543705
C	-3.749629	4.072844	-1.419187
C	-2.340158	4.102858	0.556093
C	-4.105815	5.398462	-1.191147
H	-4.142812	3.549821	-2.288617
C	-2.701664	5.430709	0.779782

H	-1.613928	3.608884	1.192057
C	-3.587012	6.078084	-0.084553
H	-4.778474	5.904937	-1.877681
H	-2.275631	5.966263	1.623377
H	-3.861614	7.114404	0.092770
H	-2.515018	1.696224	-1.865556
C	-4.259294	1.096646	-0.360237
H	-4.820114	1.431283	-1.223663
H	-4.480792	1.639886	0.550039
C	-3.999461	-0.274052	-0.235436
C	-3.961278	-1.211096	-1.366921
C	-4.306680	-0.810919	-2.673169
C	-3.566538	-2.545664	-1.151216
C	-4.257618	-1.716846	-3.725982
H	-4.611474	0.209837	-2.876041
C	-3.513593	-3.448066	-2.208433
H	-3.279595	-2.861800	-0.156852
C	-3.860927	-3.037450	-3.497368
H	-4.520050	-1.391285	-4.727969
H	-3.182707	-4.464867	-2.022638
H	-3.817386	-3.741197	-4.323946
O	-3.659000	-0.796115	0.926748
Si	-3.983789	-0.358286	2.583974
C	-3.351460	-1.838145	3.538728
H	-2.259744	-1.843130	3.604705
H	-3.742144	-1.813397	4.563863
H	-3.676796	-2.779043	3.081611
C	-3.089391	1.222191	3.051199
H	-3.036276	1.297480	4.144777
H	-2.065519	1.224473	2.664710
H	-3.587271	2.125027	2.681770
C	-5.855494	-0.200051	2.721287
H	-6.261772	0.590205	2.080791
H	-6.133441	0.040122	3.755083
H	-6.353309	-1.139416	2.454889

TS 163

B3LYP/6-31G(d) = -3573.925125
B3LYP-D3(BJ)/def2-TZVPP/IEFPCM(propanonitrile) = -3575.502767
B3LYP-D3(BJ)/def2-TZVPP/IEFPCM(propanonitrile)//B3LYP-D3(BJ)/6-31G(d) Free Energy (Quasiharmonic) = -3574.785536

Frequencies (Top 3 out of 300)

1. -275.2168 cm^{-1}
2. 9.4787 cm^{-1}
3. 9.9748 cm^{-1}

B3LYP/6-31G(d) Molecular Geometry in Cartesian Coordinates

B	-0.092532	0.151647	0.878301
O	0.212765	-0.961859	1.821974
N	-0.597107	1.266674	1.874422
C	-0.873156	0.636345	3.187938
S	-0.245502	2.893539	1.937495
C	-0.264181	-0.757826	3.059226
O	-0.189685	-1.571807	3.951844
H	-2.634008	1.642247	3.856844
H	-0.326993	1.143075	3.991282
C	-2.363691	0.607965	3.625674
C	-3.337319	0.029052	2.641447
C	-3.720101	-1.355039	2.471134
C	-4.654884	-1.404363	1.398651
N	-4.846126	-0.108282	0.960807
C	-4.036199	0.735749	1.695271
H	-4.021703	1.794810	1.480841
C	-4.836800	-3.773543	1.596789
C	-3.361360	-2.555874	3.110643
H	-5.923274	-2.621591	0.123097
H	-5.297420	0.155885	0.097889
C	-3.920350	-3.748622	2.667834
H	-5.250598	-4.723033	1.267969
H	-2.636578	-2.552442	3.919424
C	-5.219046	-2.603049	0.950861
H	-3.642198	-4.681853	3.150358
C	-1.047027	3.640344	0.517629
O	1.200771	3.146051	1.759673
O	-0.894910	3.424170	3.146416
C	-2.312810	4.844435	-1.672759
C	-0.338313	3.827460	-0.670273
C	-2.366429	4.081243	0.638918
C	-2.989313	4.675887	-0.455739
C	-0.978258	4.423977	-1.755633
H	0.698056	3.515425	-0.737655
H	-2.881801	3.980288	1.588185
H	-4.016297	5.020590	-0.363023
H	-0.430411	4.565684	-2.684044
C	-3.014220	5.433276	-2.871956
H	-2.317771	5.974451	-3.520917
H	-3.811771	6.122086	-2.575286
H	-3.470239	4.634598	-3.470184
H	-2.393823	0.050905	4.570450
C	-1.152192	-0.350916	-0.247541
C	-1.391262	-1.726213	-0.404458
C	-1.868159	0.510186	-1.089460
C	-2.269320	-2.221203	-1.370283
H	-0.899713	-2.423281	0.262227
C	-2.778923	0.023209	-2.034922
H	-1.737786	1.582471	-1.002544
C	-2.977654	-1.346798	-2.192747
H	-3.680701	-1.723308	-2.926961
C	-3.635787	0.987142	-2.799838
C	-2.441117	-3.700619	-1.566122
F	-4.194150	0.434893	-3.898355
F	-2.951796	2.082657	-3.205865
F	-4.669216	1.448474	-2.033125
F	-1.694405	-4.154158	-2.614217
F	-3.720508	-4.035931	-1.841901
F	-2.053560	-4.418790	-0.488496
O	1.181190	0.503144	0.127278
C	2.309631	0.832185	0.707647
C	3.268408	1.607514	-0.114799
C	3.246062	1.530884	-1.515596
C	4.173708	2.473309	0.515331
C	4.126272	2.303001	-2.271303
H	2.526685	0.877061	-1.998076
C	5.051001	3.246937	-0.243842
H	4.154880	2.572463	1.597274
C	5.031926	3.159929	-1.638740
H	4.101088	2.243350	-3.355994
H	5.733442	3.932426	0.250947
H	5.709098	3.769610	-2.230947
H	2.261202	1.112280	1.757624
C	3.317655	-0.862544	1.266846
H	2.439943	-1.326082	1.699210
H	4.004146	-0.406380	1.971977
C	3.877474	-1.414243	0.113004
C	3.155335	-2.290631	-0.813494
C	3.643513	-2.478170	-2.123852
C	1.993583	-2.979938	-0.412721
C	2.977669	-3.317010	-3.009371
H	4.533997	-1.945421	-2.436683
C	1.343229	-3.832754	-1.299401
H	1.600114	-2.857865	0.589744
C	1.826741	-3.997226	-2.598810
H	3.353048	-3.441362	-4.021159
H	0.452758	-4.362431	-0.980959
H	1.301766	-4.651072	-3.289175
O	5.088337	-1.051671	-0.293612
Si	6.671696	-0.976569	0.383582

C	7.205653	-2.759375	0.645090
H	7.172104	-3.329388	-0.289899
H	8.233255	-2.801534	1.026843
H	6.563787	-3.269895	1.372593
C	7.644250	-0.110200	-0.961955
H	8.692781	0.016450	-0.666760
H	7.626693	-0.680779	-1.897137
H	7.227146	0.882452	-1.164183
C	6.719994	-0.016984	1.999528
H	6.292564	0.984838	1.888665
H	7.765757	0.102803	2.311286
H	6.197952	-0.529993	2.814646

TS 164

B3LYP/6-31G(d) = -3573.924772
B3LYP-D3(BJ)/def2-TZVPP/IEFPCM(propanonitrile) = -3575.502426
B3LYP-D3(BJ)/def2-TZVPP/IEFPCM(propanonitrile)//B3LYP-D3(BJ)/6-31G(d) Free Energy (Quasiharmonic) = -3574.785431

Frequencies (Top 3 out of 300)

1. -233.5369 cm^{-1}
2. 9.9826 cm^{-1}
3. 14.6147 cm^{-1}

B3LYP/6-31G(d) Molecular Geometry in Cartesian Coordinates

B	0.123620	-0.929813	0.616002
O	0.165590	-1.996709	1.631699
N	0.622847	-1.694879	-0.646783
C	0.726062	-3.145678	-0.366336
S	0.542816	-1.196262	-2.226303
C	0.427811	-3.210125	1.135169
O	0.444772	-4.223517	1.797604
H	2.175660	-3.929013	-1.753246
H	-0.065311	-3.706601	-0.884367
C	2.057318	-3.883176	-0.670358
C	3.302224	-3.317328	-0.042085
C	4.444300	-2.776542	-0.742580
C	5.389374	-2.366075	0.238677
N	4.857429	-2.679398	1.471724
C	3.604186	-3.240110	1.298422
H	3.028835	-3.570019	2.152438
C	6.892373	-1.610076	-1.450539
C	4.761421	-2.601981	-2.103336
H	7.311601	-1.450629	0.666107
H	5.226414	-2.365384	2.356394
C	5.978006	-2.024394	-2.442492
H	7.833876	-1.155927	-1.747966
H	4.053726	-2.892679	-2.874741
C	6.611250	-1.773457	-0.099459
H	6.230211	-1.881812	-3.489842
C	1.545455	0.289840	-2.308140
O	1.127413	-2.257221	-3.059798
O	-0.829695	-0.755489	-2.578312
C	3.077554	2.627117	-2.496121
C	1.052946	1.383373	-3.026350
C	2.808662	0.334854	-1.721092
C	3.561674	1.504154	-1.813369
C	1.822358	2.540131	-3.116312
H	0.078885	1.323218	-3.498552
H	3.193891	-0.519162	-1.178950
H	4.531793	1.541161	-1.325068
H	1.437401	3.394939	-3.667660
C	3.858323	3.918080	-2.529435
H	4.905171	3.764176	-2.250584
H	3.832958	4.379572	-3.523068
H	3.424876	4.638938	-1.824206
O	-1.364689	-0.434729	0.581784
C	-2.366588	-1.246680	0.725385
H	-2.208044	-2.297937	0.470229
H	1.886262	-4.908424	-0.318474
C	-3.335895	-1.001834	1.809682
C	-3.384976	0.237272	2.467475
C	-4.169582	-2.046644	2.237947
C	-4.265413	0.425428	3.530078
H	-2.714573	1.029812	2.154313
C	-5.043089	-1.856277	3.306353
H	-4.111390	-3.016010	1.748562
C	-5.096116	-0.617025	3.950474
H	-4.294460	1.383426	4.041501
H	-5.669683	-2.676013	3.646758
H	-5.772769	-0.469161	4.787690
C	0.976669	0.365413	1.056512
C	0.552688	1.684194	0.858456
C	2.234295	0.180758	1.651349
C	1.360513	2.773103	1.205427
H	-0.422715	1.870947	0.423211
C	3.042117	1.261672	2.007381
H	2.592009	-0.827426	1.831014
C	2.613047	2.570812	1.781085
H	3.237766	3.412279	2.055863
C	4.422608	1.013002	2.545481
C	0.888152	4.163632	0.890036
F	5.320390	0.809167	1.547317
F	4.885137	2.048802	3.278888
F	-0.419584	4.340193	1.195193
F	4.475684	-0.091194	3.335063
F	1.004372	4.446161	-0.438691
F	1.587166	5.113086	1.548173
C	-3.529321	-1.090511	-1.088670
H	-2.621955	-0.988395	-1.673351
H	-3.995535	-2.070776	-1.098929
C	-4.358330	0.000315	-0.865302
O	-5.614021	-0.171050	-0.441530
Si	-6.907038	-1.173473	-0.977986
C	-8.386144	-0.422515	-0.104052
H	-9.303506	-0.974958	-0.340404
H	-8.538418	0.621080	-0.400915
H	-8.252552	-0.446673	0.983394
C	-6.999089	-1.006264	-2.847499
H	-7.827348	-1.604550	-3.246464
H	-7.164100	0.034608	-3.147643
H	-6.078917	-1.352743	-3.331460
C	-6.671842	-2.965814	-0.456902
H	-6.475315	-3.044058	0.617390
H	-7.590373	-3.528361	-0.668180
H	-5.856884	-3.465431	-0.992017
C	-3.916467	1.399259	-0.902155
C	-2.648994	1.755916	-1.407321
C	-4.758403	2.410128	-0.393905
C	-2.236462	3.085916	-1.382662
H	-1.989255	1.000456	-1.818033
C	-4.338338	3.735446	-0.375767
H	-5.729750	2.138695	0.001947
C	-3.073148	4.076163	-0.862501
H	-1.248647	3.347977	-1.749343
H	-4.992334	4.503114	0.028242
H	-2.736638	5.108503	-0.832503

TS 165

B3LYP/6-31G(d) = -3573.926408
B3LYP-D3(BJ)/def2-TZVPP/IEFPCM(propanonitrile) = -3575.503341
B3LYP-D3(BJ)/def2-TZVPP/IEFPCM(propanonitrile)//B3LYP-D3(BJ)/6-31G(d) Free Energy (Quasiharmonic) = -3574.785297

Frequencies (Top 3 out of 300)

1. -285.7177 cm^{-1}
2. 13.1701 cm^{-1}
3. 16.1686 cm^{-1}

B3LYP/6-31G(d) Molecular Geometry in Cartesian Coordinates

B	0.198710	-0.212759	-1.081830
O	0.356229	0.875671	-2.068430
N	-0.699890	-1.229470	-1.867310
C	-0.963260	-0.707040	-3.227520
S	-0.566329	-2.893790	-1.813680
C	-0.285661	0.661430	-3.224940
O	-0.316132	1.462970	-4.132810
H	-2.832649	-1.605211	-3.778010
H	-0.452540	-1.308470	-3.994090
C	-2.447600	-0.591281	-3.655120
C	-3.329681	0.186918	-2.719740
C	-4.325090	-0.351662	-1.824160
C	-4.912171	0.744297	-1.132360
N	-4.315002	1.893098	-1.607980
C	-3.357542	1.550338	-2.547390
H	-2.774992	2.314709	-3.043560
C	-6.348870	-0.724264	0.079440
C	-4.787959	-1.652063	-1.550910
H	-6.353172	1.422746	0.344950
H	-4.404033	2.806157	-1.190200
C	-5.791129	-1.825394	-0.603600
H	-7.131590	-0.891584	0.814730
H	-4.355179	-2.507202	-2.063350
C	-5.919221	0.573006	-0.176350
H	-6.155729	-2.825874	-0.385680
C	-0.834828	-3.284790	-0.084230
O	0.791902	-3.353549	-2.162910
O	-1.687608	-3.439801	-2.598690
C	-1.288538	-4.052100	2.571820
C	0.129992	-4.030269	0.592060
C	-2.036239	-2.935281	0.538320
C	-2.249528	-3.315861	1.859880
C	-0.102777	-4.406009	1.916190
H	1.040762	-4.313059	0.075880
H	-2.787779	-2.365451	0.002670
H	-3.178778	-3.033412	2.348450
H	0.647633	-4.987679	2.446490
C	-1.527767	-4.429730	4.013760
H	-2.521107	-4.872371	4.150770
H	-1.475488	-3.542830	4.655650
H	-0.783787	-5.149070	4.370010
H	-2.432611	-0.123251	-4.647110
C	-0.400051	0.380140	0.303450
C	-1.052472	1.620060	0.304750
C	-0.247160	-0.257600	1.544120
C	-1.523172	2.206170	1.484520
H	-1.176582	2.150250	-0.632280
C	-0.723531	0.316500	2.725540
H	0.262290	-1.212989	1.595150
C	-1.364872	1.557390	2.706890
H	-1.721212	2.010699	3.625210
C	-0.568830	-0.392470	4.039450
C	-2.089343	3.595539	1.446460
F	-1.717860	-0.983781	4.448630
F	-0.207801	0.464860	5.029890
F	0.374370	-1.364889	3.997970
F	-1.102374	4.537940	1.458390
F	-2.812493	3.835159	0.322410
F	-2.887113	3.863259	2.500200
O	1.577400	-0.795018	-0.745180
C	2.617160	-0.634888	-1.525400
C	3.620451	-1.732097	-1.556960
C	4.528221	-1.806366	-2.622520
C	3.671611	-2.696737	-0.541000
C	5.474901	-2.827445	-2.673230
H	4.481270	-1.071746	-3.423740
C	4.623032	-3.712876	-0.589960
H	2.945151	-2.654197	0.262530
C	5.527852	-3.779965	-1.653120
H	6.164791	-2.884635	-3.510440
H	4.652363	-4.462846	0.195740
H	6.264583	-4.577585	-1.691610
H	2.425839	-0.150528	-2.484340
C	3.830289	0.840663	-0.878330
H	4.336549	1.028944	-1.818010
H	4.428159	0.277964	-0.170880
C	3.071128	1.895243	-0.332100
C	2.463357	2.972762	-1.113540
C	2.744837	3.142313	-2.484050
C	1.558647	3.856962	-0.492160
C	2.134136	4.156912	-3.208970
H	3.434718	2.477493	-2.992070
C	0.947876	4.869611	-1.222130
H	1.315457	3.723452	0.554010
C	1.233686	5.022251	-2.581150
H	2.348846	4.267152	-4.267170
H	0.233655	5.522991	-0.730830
H	0.750505	5.808691	-3.154130
O	2.776318	1.905753	0.954580
Si	3.639568	1.456593	2.396800
C	2.712788	2.364793	3.746260
H	1.713938	1.948132	3.909490
H	3.260368	2.286613	4.693930
H	2.606177	3.429592	3.511450
C	3.615810	-0.398797	2.669980
H	4.220540	-0.632046	3.556420
H	2.601280	-0.760618	2.862690
H	4.030120	-0.967547	1.831420
C	5.389208	2.121444	2.192610
H	5.936098	1.638495	1.375860
H	5.959568	1.952375	3.114310
H	5.383017	3.200794	2.002200

TS 166

B3LYP/6-31G(d) = -3573.925421
B3LYP-D3(BJ)/def2-TZVPP/IEFPCM(propanonitrile) = -3575.501289
B3LYP-D3(BJ)/def2-TZVPP/IEFPCM(propanonitrile)//B3LYP-D3(BJ)/6-31G(d) Free Energy (Quasiharmonic) = -3574.785293

Frequencies (Top 3 out of 300)

1. -249.4229 cm^{-1}
2. 7.8230 cm^{-1}
3. 9.8282 cm^{-1}

B3LYP/6-31G(d) Molecular Geometry in Cartesian Coordinates

B	0.596834	-0.603241	-0.156827
O	0.382525	0.596244	-1.017591
N	-0.301411	-1.645803	-0.890040
C	-0.725121	-1.122796	-2.200182
S	-0.067925	-3.290163	-0.725769
C	-0.271190	0.334862	-2.149444
O	-0.472449	1.166116	-3.012671
H	-2.499819	-2.286347	-2.559221
H	-0.172839	-1.623842	-3.006932
C	-2.231182	-1.225207	-2.553087
C	-3.193793	-0.450308	-1.694148
C	-3.818050	0.814746	-2.016857
C	-4.719824	1.125400	-0.960759
N	-4.628406	0.103176	-0.038021
C	-3.722246	-0.840272	-0.486702
H	-3.538250	-1.735878	0.088638
C	-5.411146	3.123228	-2.063307

C	-3.724825	1.700001	-3.106027
H	-6.205288	2.491008	-0.153048
H	-5.161015	0.029365	0.814323
C	-4.523126	2.837414	-3.121817
H	-6.025053	4.019050	-2.106195
H	-3.024263	1.503402	-3.911464
C	-5.518004	2.274875	-0.967511
H	-4.466708	3.519764	-3.966189
C	-1.665459	-3.986494	-0.263367
O	0.803539	-3.485950	0.437397
O	0.268544	-3.865790	-2.038415
C	-4.104367	-5.141617	0.501074
C	-2.392731	-4.738502	-1.184700
C	-2.131658	-3.817535	1.042868
C	-3.346720	-4.389289	1.413055
C	-3.608518	-5.304714	-0.798429
H	-1.998438	-4.885803	-2.184624
H	-1.544600	-3.250066	1.757498
H	-3.709883	-4.257866	2.429957
H	-4.176170	-5.889293	-1.518438
C	-5.401923	-5.789113	0.923342
H	-5.920210	-5.193577	1.682492
H	-5.223172	-6.782198	1.356531
H	-6.079204	-5.921965	0.073544
H	-2.320597	-0.881143	-3.589499
C	2.174676	-0.907885	-0.003966
C	2.929078	-1.324739	-1.112777
C	2.870432	-0.690489	1.190029
C	4.309533	-1.516843	-1.031289
H	2.436170	-1.518384	-2.061278
C	4.254568	-0.868600	1.278013
H	2.325127	-0.381841	2.075219
C	4.985513	-1.284720	0.168162
H	6.058603	-1.421672	0.231742
C	4.961392	-0.514371	2.554410
C	5.075128	-2.025742	-2.222682
F	6.165513	-1.114632	2.665320
F	4.238979	-0.846836	3.648306
F	5.187515	0.827500	2.643871
F	4.498754	-1.658252	-3.389352
F	5.159029	-3.374807	-2.232302
F	6.346861	-1.557035	-2.238270
O	0.046480	-0.321439	1.237724
C	-0.868670	0.553518	1.520428
C	-1.710827	0.275919	2.711546
C	-2.909905	0.982565	2.896175
C	-1.335996	-0.706175	3.639676
C	-3.721613	0.710495	3.995289
H	-3.200819	1.742457	2.174774
C	-2.151675	-0.975158	4.738125
H	-0.413551	-1.256102	3.484016
C	-3.343100	-0.268400	4.919599
H	-4.647514	1.262165	4.135017
H	-1.856595	-1.737437	5.453775
H	-3.975328	-0.478546	5.777927
H	-1.359811	1.062785	0.691048
C	0.129216	2.261192	2.267364
H	0.925181	1.724124	2.773139
H	-0.675834	2.637191	2.885156
C	0.458274	3.013485	1.139716
C	-0.512042	3.988082	0.582607
C	-0.924914	3.904881	-0.758844
C	-1.070230	4.969402	1.421512
C	-1.882078	4.795481	-1.244133
H	-0.542995	3.121190	-1.405064
C	-2.002326	5.874038	0.918616
H	-0.749378	5.036863	2.457260
C	-2.412763	5.784452	-0.413911
H	-2.227728	4.695520	-2.267713
H	-2.411511	6.643699	1.567087
H	-3.152830	6.479110	-0.801507
O	1.590976	2.772044	0.528452
Si	2.704285	3.625509	-0.509188
C	2.966890	5.301956	0.306504
H	2.041699	5.887476	0.347488
H	3.350148	5.195004	1.327708
H	3.701199	5.884681	-0.263541
C	2.069992	3.790776	-2.266789
H	2.922226	4.001548	-2.926272
H	1.591754	2.871034	-2.620148
H	1.352182	4.609465	-2.377350
C	4.228914	2.547251	-0.430120
H	4.522783	2.321934	0.599794
H	4.059274	1.593500	-0.940787
H	5.072145	3.046155	-0.924238

TS 167

B3LYP/6-31G(d) = -3573.936979
B3LYP-D3(BJ)/def2-TZVPP/IEFPCM(propanonitrile) = -3575.502388
B3LYP-D3(BJ)/def2-TZVPP/IEFPCM(propanonitrile)//B3LYP-D3(BJ)/6-31G(d) Free Energy (Quasiharmonic) = -3574.785286

Frequencies (Top 3 out of 300)

1. -293.4897 cm^{-1}
2. 8.4128 cm^{-1}
3. 10.2624 cm^{-1}

B3LYP/6-31G(d) Molecular Geometry in Cartesian Coordinates

B	-0.261338	-0.055116	-0.746565
O	-0.056670	1.412314	-0.803628
N	-1.407394	-0.243927	-1.775635
C	-1.663984	1.015843	-2.492461
S	-1.720356	-1.693013	-2.523971
C	-0.686326	1.993106	-1.839285
O	-0.490551	3.137927	-2.180526
H	-3.776078	0.826486	-2.883197
H	-1.399261	0.920641	-3.552362
C	-3.118176	1.563029	-2.410246
C	-3.610697	1.899628	-1.030125
C	-3.567284	3.190009	-0.375425
C	-4.126847	3.021758	0.921509
N	-4.513967	1.700833	1.033430
C	-4.183084	1.033010	-0.130430
H	-4.393053	-0.022136	-0.231575
C	-3.780635	5.324171	1.431233
C	-3.121014	4.469015	-0.756348
H	-4.648489	3.917989	2.828682
H	-4.767732	1.247919	1.898684
C	-3.234386	5.519127	0.145419
H	-3.847850	6.164560	2.117167
H	-2.668047	4.624418	-1.730558
C	-4.232560	4.074530	1.836980
H	-2.888404	6.509327	-0.139488
C	-3.425953	-2.118287	-2.128623
O	-1.666819	-1.507252	-3.982757
O	-0.890177	-2.709205	-1.861403
C	-6.069864	-2.823911	-1.515326
C	-3.726459	-2.645827	-0.869715
C	-4.423778	-1.959301	-3.088966
C	-5.737652	-2.310400	-2.774833
C	-5.042338	-2.991680	-0.572729
H	-2.934882	-2.796662	-0.143075
H	-4.164815	-1.584966	-4.073724
H	-6.514780	-2.190564	-3.525817
H	-5.275864	-3.409860	0.403814
C	-7.496884	-3.178753	-1.170779
H	-7.980222	-2.369039	-0.608108
H	-8.094927	-3.353338	-2.070710

H	-7.546965	-4.079119	-0.548828
H	-3.145730	2.457016	-3.044408
C	-0.532045	-0.571927	0.760977
C	-1.287892	0.197308	1.660734
C	-0.013437	-1.785572	1.233201
C	-1.523995	-0.235586	2.969080
C	-0.213475	-2.203301	2.553373
H	0.556811	-2.414288	0.557788
C	-0.976799	-1.435152	3.430063
H	-1.137317	-1.759148	4.451392
C	0.476028	-3.449749	3.030353
C	-2.416882	0.565999	3.873779
F	1.812494	-3.236109	3.205599
F	0.368414	-4.462967	2.145441
F	-0.002268	-3.889219	4.214098
F	-3.735536	0.240136	3.692905
F	-2.323840	1.892120	3.651829
F	-2.155708	0.349708	5.180714
O	1.023858	-0.727628	-1.228199
C	2.152236	-0.115768	-1.464715
C	3.200213	-0.950470	-2.108153
C	2.991628	-2.328720	-2.280274
C	4.373847	-0.361936	-2.605262
C	3.956000	-3.103926	-2.922467
H	2.056878	-2.766772	-1.946336
C	5.334058	-1.141746	-3.247702
H	4.530303	0.708324	-2.500991
C	5.130023	-2.515743	-3.401056
H	3.783291	-4.167214	-3.063207
H	6.234811	-0.676051	-3.637946
H	5.876493	-3.122748	-3.906266
H	2.097472	0.923579	-1.798129
C	2.788047	0.393827	0.372804
H	1.884578	0.941990	0.607633
H	2.846103	-0.590637	0.821348
C	3.972749	1.132490	0.270172
C	4.004434	2.535944	-0.161978
C	2.817941	3.224467	-0.496541
C	5.238147	3.210186	-0.276025
C	2.870989	4.542166	-0.940805
H	1.846954	2.747024	-0.427088
C	5.280934	4.530856	-0.707431
H	6.153286	2.686020	-0.026033
C	4.098848	5.199394	-1.044003
H	1.945331	5.039899	-1.212468
H	6.236734	5.040922	-0.787365
H	4.137493	6.229339	-1.388079
O	5.158816	0.564559	0.420660
Si	5.773191	-0.742402	1.391298
C	4.834883	-2.353676	1.165608
H	5.461990	-3.168703	1.551370
H	3.887756	-2.395869	1.714409
H	4.633029	-2.569426	0.111134
C	5.691879	-0.129376	3.165337
H	4.658590	0.061815	3.476982
H	6.105776	-0.881504	3.848155
H	6.262635	0.796087	3.299763
H	-1.691222	1.151050	1.336768
C	7.525782	-0.885807	0.742502
H	7.525602	-1.172074	-0.315260
H	8.069347	0.060694	0.836926
H	8.084625	-1.650115	1.295654

TS 168

B3LYP/6-31G(d) = -3573.926323
B3LYP-D3(BJ)/def2-TZVPP/IEFPCM(propanonitrile) = -3575.502876
B3LYP-D3(BJ)/def2-TZVPP/IEFPCM(propanonitrile)//B3LYP-D3(BJ)/6-31G(d) Free Energy (Quasiharmonic) = -3574.78528

Frequencies (Top 3 out of 300)

1. -235.9100 cm^{-1}
2. 11.5623 cm^{-1}
3. 16.6209 cm^{-1}

B3LYP/6-31G(d) Molecular Geometry in Cartesian Coordinates

B	-0.362782	-0.629932	0.784995
O	-0.010306	-1.794824	1.631095
N	0.009267	-1.174157	-0.634532
C	0.236208	-2.630200	-0.565256
S	-0.510863	-0.501667	-2.060044
C	0.313734	-2.890206	0.942879
O	0.602309	-3.954559	1.442467
H	1.208172	-3.214911	-2.391363
H	-0.655628	-3.175363	-0.915989
C	1.443156	-3.233291	-1.324456
C	2.790967	-2.600093	-1.117672
C	3.800868	-2.916803	-0.129980
C	4.941665	-2.113851	-0.425173
N	4.627208	-1.350079	-1.530140
C	3.341638	-1.645553	-1.938104
H	2.899761	-1.153876	-2.792218
C	6.150347	-3.062345	1.401061
C	3.865277	-3.802524	0.962099
H	6.982042	-1.561115	0.081144
H	5.215233	-0.652934	-1.960173
C	5.033613	-3.865551	1.712375
H	7.052277	-3.141259	2.002681
H	3.003405	-4.407444	1.225353
C	6.119385	-2.175044	0.329667
H	5.088486	-4.545514	2.558175
C	-2.136328	-1.177077	-2.433946
O	0.379304	-0.954830	-3.142820
O	-0.666808	0.941125	-1.808182
C	-4.664639	-2.317919	-2.853662
C	-2.238995	-2.436917	-3.029968
C	-3.285683	-0.460602	-2.089051
C	-4.535168	-1.034231	-2.303078
C	-3.500332	-2.998464	-3.230283
H	-1.346087	-2.961341	-3.353343
H	-3.200474	0.530690	-1.660940
H	-5.427676	-0.479557	-2.024743
H	-3.578368	-3.982701	-3.685406
C	-6.027768	-2.946893	-3.008571
H	-5.981338	-3.875846	-3.584969
H	-6.728310	-2.269309	-3.510443
H	-6.452556	-3.181422	-2.024387
O	0.535846	0.533697	1.249182
C	1.829989	0.369870	1.307779
H	2.293479	-0.208835	0.504709
H	1.473264	-4.285418	-1.016962
C	2.472196	0.301911	2.634304
C	3.759658	-0.247639	2.750625
C	1.787277	0.720239	3.787204
C	4.354469	-0.371584	4.003211
H	4.281555	-0.603512	1.866421
C	2.390929	0.602533	5.035204
H	0.779864	1.111027	3.692508
C	3.675340	0.059591	5.145627
H	5.340544	-0.818826	4.085514
H	1.856508	0.920518	5.925989
H	4.139839	-0.039125	6.123065
C	-1.894564	-0.191545	1.053757
C	-2.358513	1.124113	0.946579
C	-2.843358	-1.175172	1.381244
C	-3.705268	1.448112	1.146749
H	-1.661062	1.909098	0.684137
C	-4.191083	-0.862894	1.567186
H	-2.527075	-2.206305	1.500002

C	-4.633112	0.455875	1.454733
H	-5.676705	0.703653	1.609762
C	-5.199619	-1.953391	1.802359
C	-4.162752	2.861168	0.928456
F	-5.762620	-2.364909	0.634290
F	-4.655268	-3.044047	2.378030
F	-5.379513	3.104298	1.458510
F	-6.217984	-1.539946	2.591498
F	-4.243294	3.155729	-0.395824
F	-3.304655	3.767151	1.465116
C	2.781220	2.237242	0.738951
H	3.788153	1.864332	0.873426
H	2.359172	2.777274	1.576986
C	2.386572	2.587218	-0.547579
O	1.276866	3.244784	-0.804172
Si	0.308557	4.470013	-0.056342
C	-0.006401	4.098935	1.761460
H	-0.898282	4.639445	2.098431
H	0.832792	4.417228	2.390922
H	-0.183438	3.033989	1.938131
C	1.301128	6.058814	-0.239400
H	1.517969	6.275109	-1.291283
H	0.736529	6.907544	0.166801
H	2.253989	6.009685	0.300702
C	-1.225005	4.453896	-1.124617
H	-1.020207	4.919168	-2.095533
H	-1.550591	3.426114	-1.313540
H	-2.054259	4.990580	-0.653050
C	3.141239	2.198695	-1.758650
C	2.453011	1.946121	-2.958629
C	4.546323	2.148480	-1.745624
C	3.160962	1.626020	-4.114778
H	1.369079	1.964531	-2.960284
C	5.250322	1.856892	-2.913436
H	5.090157	2.381947	-0.835293
C	4.558005	1.587698	-4.098681
H	2.618885	1.404711	-5.029228
H	6.337321	1.857841	-2.903620
H	5.107025	1.356495	-5.007490

TS 169

B3LYP/6-31G(d) = -3573.926698
B3LYP-D3(BJ)/def2-TZVPP/IEFPCM(propanonitrile) = -3575.501471
B3LYP-D3(BJ)/def2-TZVPP/IEFPCM(propanonitrile)//B3LYP-D3(BJ)/6-31G(d) Free Energy (Quasiharmonic) = -3574.785276

Frequencies (Top 3 out of 300)

1. -248.5255 cm^{-1}
2. 10.3296 cm^{-1}
3. 13.2024 cm^{-1}

B3LYP/6-31G(d) Molecular Geometry in Cartesian Coordinates

B	-0.060523	-0.089886	0.832236
O	-0.035130	-0.256397	2.294568
N	-0.653267	1.374526	0.693783
C	-0.989704	1.883908	2.039564
S	-0.156473	2.461308	-0.435391
C	-0.424226	0.816051	2.978007
O	-0.390570	0.912696	4.186651
H	-2.903464	2.827617	1.650739
H	-0.475867	2.830645	2.243390
C	-2.508002	2.070342	2.334904
C	-3.338601	0.815408	2.252749
C	-4.318434	0.475723	1.247586
C	-4.795616	-0.830485	1.547162
N	-4.165834	-1.240045	2.703344
C	-3.285762	-0.258340	3.112285
H	-2.679247	-0.399608	3.995922
C	-6.234931	-0.806805	-0.353129
C	-4.854184	1.144091	0.133079
H	-6.065593	-2.497327	0.989343
H	-4.173822	-2.187299	3.049502
C	-5.801635	0.501067	-0.653830
H	-6.969146	-1.288189	-0.993602
H	-4.531317	2.150603	-0.113383
C	-5.740630	-1.487931	0.752837
H	-6.216263	1.011251	-1.519630
C	-1.564755	3.475335	-0.894205
O	0.218970	1.687875	-1.637067
O	0.848402	3.393049	0.129538
C	-3.690879	5.107467	-1.706446
C	-1.688961	4.760374	-0.364799
C	-2.479973	2.995162	-1.835127
C	-3.530402	3.817186	-2.235030
C	-2.754657	5.563678	-0.769549
H	-0.946450	5.129770	0.334149
H	-2.373264	1.999967	-2.253061
H	-4.238931	3.448789	-2.972861
H	-2.851465	6.565882	-0.359516
C	-4.853894	5.972630	-2.129264
H	-4.666427	7.029606	-1.916084
H	-5.058915	5.871623	-3.200541
H	-5.770019	5.686441	-1.596203
H	-2.558350	2.499673	3.343534
C	-0.945637	-1.225495	0.103590
C	-1.393326	-1.098412	-1.218374
C	-1.349326	-2.368656	0.808665
C	-2.221906	-2.057155	-1.806549
C	-2.185034	-3.327104	0.227716
H	-1.023827	-2.497715	1.835720
C	-2.629573	-3.178452	-1.086595
H	-3.285817	-3.914331	-1.535515
C	-2.678177	-4.480380	1.052641
C	-2.647841	-1.870204	-3.235262
F	-3.799140	-4.154169	1.769434
F	-1.765195	-4.892582	1.957972
F	-3.016462	-5.548366	0.299197
F	-3.723357	-2.621202	-3.557341
F	-1.660423	-2.206393	-4.103470
F	-2.963666	-0.578508	-3.501981
O	1.428850	-0.268851	0.480701
C	2.042166	-0.412746	-0.656125
C	2.882516	-1.614404	-0.844267
C	3.242542	-2.005488	-2.143407
C	3.270349	-2.405075	0.248463
C	3.982388	-3.168633	-2.348843
H	2.918169	-1.408619	-2.993069
C	4.014136	-3.564239	0.039829
H	2.964464	-2.109564	1.246534
C	4.373073	-3.947952	-1.255732
H	4.239945	-3.476153	-3.358628
H	4.304999	-4.177793	0.887983
H	4.942123	-4.859934	-1.415041
H	1.552671	-0.012238	-1.544327
C	3.395098	1.205083	-0.779956
H	2.643294	1.940209	-0.525157
H	3.651795	1.148463	-1.831731
C	4.379013	0.870197	0.144672
C	4.226436	1.016034	1.594040
C	5.146569	0.385513	2.458634
C	3.159047	1.750667	2.152062
C	4.995360	0.476355	3.836505
H	5.962444	-0.187555	2.034445
C	3.013995	1.833376	3.534556
H	2.453698	2.271927	1.514952
C	3.925363	1.196042	4.377945
H	5.705274	-0.020805	4.491618
H	2.175259	2.378277	3.955540

H	3.800365	1.257564	5.455356
O	5.477388	0.217378	-0.239587
Si	6.685434	0.444434	-1.440077
C	7.598806	2.023247	-0.984128
H	8.422098	2.210323	-1.684570
H	6.934369	2.894676	-1.016803
H	8.023623	1.963664	0.024036
C	5.973908	0.560846	-3.177857
H	5.326593	-0.292333	-3.406129
H	5.404717	1.481847	-3.344161
H	6.797953	0.553788	-3.903167
H	-1.108227	-0.224642	-1.795367
C	7.737876	-1.093759	-1.243725
H	8.169061	-1.157434	-0.238499
H	7.138100	-1.995654	-1.409844
H	8.564665	-1.099765	-1.964099

TS 170

B3LYP/6-31G(d) = -3573.93064
B3LYP-D3(BJ)/def2-TZVPP/IEFPCM(propanonitrile) = -3575.502399
B3LYP-D3(BJ)/def2-TZVPP/IEFPCM(propanonitrile)//B3LYP-D3(BJ)/6-31G(d) Free Energy (Quasiharmonic) = -3574.785209

Frequencies (Top 3 out of 300)

1. -285.2861 cm^{-1}
2. 12.4493 cm^{-1}
3. 13.3571 cm^{-1}

B3LYP/6-31G(d) Molecular Geometry in Cartesian Coordinates

B	0.185331	-0.119933	-0.082470
O	-0.381327	0.755052	-1.159071
N	-0.131404	-1.528281	-0.691522
C	-0.496556	-1.406543	-2.110326
S	0.484231	-2.957935	-0.108068
C	-0.695797	0.101325	-2.274741
O	-1.088015	0.641664	-3.291263
H	-1.557159	-3.250419	-2.450273
H	0.351018	-1.685729	-2.758159
C	-1.731104	-2.188626	-2.634496
C	-3.088603	-1.795322	-2.111847
C	-3.900412	-2.479977	-1.128198
C	-5.151977	-1.802392	-1.067225
N	-5.089035	-0.749756	-1.958129
C	-3.859966	-0.761991	-2.590517
H	-3.619117	-0.033590	-3.351610
C	-5.977018	-3.356075	0.542899
C	-3.705723	-3.614026	-0.317168
H	-7.148498	-1.699260	-0.213060
H	-5.844842	-0.118555	-2.172703
C	-4.743857	-4.039652	0.502816
H	-6.771147	-3.720038	1.189870
H	-2.752675	-4.133574	-0.324717
C	-6.196719	-2.224887	-0.236509
H	-4.602208	-4.914759	1.130999
C	2.085602	-3.248378	-0.883270
O	-0.380268	-4.053002	-0.578071
O	0.714385	-2.752250	1.326008
C	4.576976	-3.704016	-2.094192
C	2.144392	-3.785849	-2.172794
C	3.259449	-2.967404	-0.181232
C	4.491282	-3.197350	-0.790110
C	3.385461	-4.001874	-2.769438
H	1.232116	-4.068351	-2.687362
H	3.211322	-2.594702	0.834152
H	5.401373	-2.983536	-0.234783
H	3.428235	-4.423243	-3.771018
C	5.917733	-3.914580	-2.756409
H	6.684406	-4.197953	-2.027600
H	6.261947	-2.995329	-3.248915
H	5.869565	-4.696001	-3.521822
H	-1.714716	-2.039876	-3.720699
C	1.725346	0.285130	0.206821
C	2.476885	0.990835	-0.743434
C	2.362549	-0.025297	1.417786
C	3.798262	1.377692	-0.498614
H	2.023273	1.260701	-1.692370
C	3.693513	0.328125	1.654652
H	1.816379	-0.575608	2.175770
C	4.421354	1.040786	0.701912
H	5.449449	1.325602	0.891022
C	4.362736	-0.147870	2.912443
C	4.516904	2.227637	-1.506676
F	4.735869	-1.451329	2.806227
F	5.481949	0.554174	3.201079
F	3.547278	-0.067458	3.986637
F	4.214324	1.881000	-2.776393
F	5.859540	2.162222	-1.375616
F	4.182121	3.545041	-1.381623
O	-0.550231	0.094918	1.211363
C	-1.852769	-0.022552	1.313965
C	-2.360391	-0.678128	2.544539
C	-3.669386	-1.180138	2.577898
C	-1.532829	-0.819894	3.669279
C	-4.146370	-1.811574	3.724310
H	-4.305222	-1.102848	1.700154
C	-2.016174	-1.446865	4.814063
H	-0.513282	-0.453656	3.625706
C	-3.324090	-1.940275	4.845940
H	-5.154604	-2.215142	3.733225
H	-1.369057	-1.563206	5.678963
H	-3.696326	-2.434938	5.739173
H	-2.401784	-0.237735	0.394874
C	-2.708655	1.798854	1.596346
H	-3.735598	1.500906	1.408958
H	-2.427502	1.909761	2.636067
C	-2.145068	2.713058	0.698055
C	-0.947498	3.495812	1.051287
C	0.107622	3.649311	0.133250
C	-0.834974	4.075123	2.330052
C	1.252290	4.357554	0.491820
H	0.052866	3.144525	-0.822726
C	0.296719	4.809358	2.670759
H	-1.649572	3.977127	3.041002
C	1.343697	4.946552	1.754871
H	2.085079	4.417738	-0.201726
H	0.366131	5.268700	3.652539
H	2.235407	5.502656	2.030477
O	-2.651850	2.783761	-0.513936
Si	-2.911373	4.002770	-1.724941
C	-1.521140	4.030395	-2.976210
H	-1.232433	3.008053	-3.248092
H	-1.865905	4.539567	-3.885701
H	-0.637259	4.564197	-2.613113
C	-3.139067	5.643593	-0.832368
H	-2.225232	5.967024	-0.323433
H	-3.406261	6.422056	-1.558103
H	-3.943921	5.594776	-0.089775
C	-4.511471	3.412478	-2.509429
H	-4.375385	2.414218	-2.939743
H	-5.324846	3.362719	-1.776785
H	-4.824288	4.085335	-3.316875

TS 171

B3LYP/6-31G(d) = -3573.927853
B3LYP-D3(BJ)/def2-TZVPP/IEFPCM(propanonitrile) = -3575.502437

B3LYP-D3(BJ)/def2-TZVPP/IEFPCM(propanonitrile)//B3LYP-D3(BJ)/6-31G(d) Free Energy (Quasiharmonic) = -3574.785176

Frequencies (Top 3 out of 300)

1. -269.9991 cm^{-1}
2. 10.2305 cm^{-1}
3. 15.5615 cm^{-1}

B3LYP/6-31G(d) Molecular Geometry in Cartesian Coordinates

B	-0.271110	0.239006	-0.652326
O	-0.930498	-1.088901	-0.574614
N	0.295872	0.208334	-2.102998
C	-0.271404	-0.936992	-2.842300
S	0.597551	1.562591	-3.040778
C	-0.983500	-1.727198	-1.748894
O	-1.550040	-2.790977	-1.900715
H	1.059474	-1.230918	-4.497876
H	-1.050803	-0.611004	-3.549487
C	0.723021	-1.822109	-3.643940
C	1.908658	-2.332193	-2.878204
C	2.030997	-3.565475	-2.132091
C	3.347156	-3.599776	-1.591577
N	3.992728	-2.452931	-2.009460
C	3.119371	-1.692975	-2.765682
H	3.427917	-0.751701	-3.199320
C	2.931831	-5.712369	-0.567642
C	1.168934	-4.649056	-1.877285
H	4.813101	-4.658965	-0.390933
H	4.873766	-2.122940	-1.646458
C	1.626364	-5.707193	-1.102122
H	3.258831	-6.552972	0.038847
H	0.155242	-4.644480	-2.265371
C	3.808177	-4.660592	-0.804519
H	0.966870	-6.547320	-0.899965
C	1.512034	2.626492	-1.924818
O	-0.654845	2.253429	-3.401171
O	1.496075	1.151379	-4.134244
C	2.929824	4.301656	-0.186091
C	0.940498	3.827250	-1.508738
C	2.800562	2.269996	-1.520972
C	3.496468	3.105391	-0.653128
C	1.653863	4.656352	-0.642645
H	-0.047766	4.096451	-1.864156
H	3.242314	1.340737	-1.862462
H	4.490637	2.816781	-0.320956
H	1.209200	5.591539	-0.310520
C	3.662380	5.158362	0.818395
H	3.492671	4.784260	1.836614
H	3.319944	6.197704	0.787834
H	4.743554	5.149100	0.643391
H	0.130991	-2.659009	-4.033555
C	0.830867	0.459577	0.508488
C	0.696920	1.437242	1.503998
C	1.993826	-0.324024	0.535518
C	1.683592	1.641567	2.470248
H	-0.183412	2.068744	1.510946
C	2.993852	-0.114125	1.492401
H	2.135975	-1.089232	-0.221819
C	2.847114	0.871724	2.467041
H	3.625129	1.041619	3.201989
C	4.268011	-0.905862	1.407037
C	1.479767	2.660452	3.553856
F	4.042860	-2.232110	1.295984
F	5.070114	-0.720438	2.477109
F	4.998752	-0.549832	0.308089
F	0.992564	2.098570	4.690071
F	0.608752	3.629095	3.195362
F	2.640555	3.274648	3.897326
O	-1.331090	1.331467	-0.460181
C	-2.603822	1.210766	-0.711533
C	-3.337092	2.433552	-1.125915
C	-4.522610	2.304268	-1.863135
C	-2.862565	3.710896	-0.797416
C	-5.220760	3.437438	-2.274960
H	-4.889738	1.314248	-2.124337
C	-3.565633	4.841525	-1.205486
H	-1.938652	3.805566	-0.236844
C	-4.744856	4.708227	-1.944172
H	-6.130526	3.329722	-2.858691
H	-3.192110	5.829926	-0.952633
H	-5.288097	5.592591	-2.265390
H	-2.938083	0.285371	-1.184158
C	-3.545149	0.916749	1.098002
H	-4.540806	1.236785	0.817554
H	-2.946002	1.631965	1.652588
C	-3.392528	-0.437301	1.412536
C	-4.406126	-1.428743	0.977579
C	-5.763769	-1.201954	1.269956
C	-4.037314	-2.567555	0.240073
C	-6.732182	-2.115402	0.861034
H	-6.051660	-0.325270	1.843089
C	-5.018315	-3.461070	-0.189526
H	-3.006275	-2.722271	-0.058872
C	-6.360657	-3.245930	0.128355
H	-7.775373	-1.943462	1.110447
H	-4.726665	-4.321023	-0.785236
H	-7.118120	-3.951742	-0.201306
O	-2.295569	-0.817208	2.018293
Si	-1.764413	-2.137562	3.029674
C	-0.232379	-1.430020	3.839422
H	-0.403616	-0.437689	4.269657
H	0.107439	-2.093573	4.644677
H	0.583581	-1.341049	3.114859
C	-3.152130	-2.433577	4.266259
H	-3.371898	-1.530952	4.847924
H	-4.076828	-2.759392	3.778052
H	-2.857435	-3.218854	4.973575
C	-1.354419	-3.671199	2.032624
H	-0.706049	-4.320452	2.635247
H	-0.812634	-3.423386	1.114078
H	-2.243670	-4.247518	1.760228

TS 172

B3LYP/6-31G(d) = -3573.931029
B3LYP-D3(BJ)/def2-TZVPP/IEFPCM(propanonitrile) = -3575.502159
B3LYP-D3(BJ)/def2-TZVPP/IEFPCM(propanonitrile)//B3LYP-D3(BJ)/6-31G(d) Free Energy (Quasiharmonic) = -3574.785145

Frequencies (Top 3 out of 300)

1. -283.5099 cm^{-1}
2. 9.3388 cm^{-1}
3. 12.0426 cm^{-1}

B3LYP/6-31G(d) Molecular Geometry in Cartesian Coordinates

B	0.205795	0.626315	-0.743952
O	-0.257854	-0.001482	-2.004136
N	1.747271	0.789632	-1.013614
C	2.072988	0.213640	-2.329851
S	2.542700	2.167829	-0.535788
C	0.716163	-0.218887	-2.888063
O	0.535662	-0.706405	-3.984921
H	4.057580	-0.593673	-2.055143
H	2.471638	0.976249	-3.011315
C	3.065726	-0.986820	-2.299097
C	2.704947	-2.087250	-1.342094
C	1.863949	-3.239956	-1.578776

C	1.781481	-3.958492	-0.351997
N	2.557664	-3.285153	0.570635
C	3.087768	-2.158605	-0.025711
H	3.715813	-1.485716	0.540523
C	0.355297	-5.592710	-1.342250
C	1.176441	-3.739598	-2.700275
H	0.966606	-5.654540	0.731645
H	2.564342	-3.451219	1.565833
C	0.433131	-4.906644	-2.571556
H	-0.241326	-6.498265	-1.270791
H	1.206404	-3.205611	-3.645183
C	1.028690	-5.129382	-0.217812
H	-0.104436	-5.296380	-3.432084
C	4.249811	1.672734	-0.242943
O	2.594062	3.166053	-1.621449
O	1.978882	2.538367	0.773563
C	6.916575	0.949712	0.238861
C	5.213110	1.886305	-1.228686
C	4.606495	1.128805	0.993818
C	5.933026	0.771109	1.223866
C	6.536435	1.519025	-0.983265
H	4.928924	2.353757	-2.165512
H	3.859646	1.009390	1.771881
H	6.212640	0.355723	2.189248
H	7.287139	1.687810	-1.751531
C	8.344697	0.527843	0.489434
H	9.043147	1.075577	-0.151135
H	8.635203	0.695424	1.532070
H	8.480622	-0.542018	0.282734
O	-0.426856	2.004671	-0.720054
C	-1.260338	2.493014	0.157174
H	-1.530371	1.847050	0.996309
H	3.120246	-1.367454	-3.326172
C	-1.116160	3.935667	0.490280
C	-0.359238	4.789505	-0.323615
C	-1.725409	4.439448	1.650172
C	-0.228263	6.134162	0.017921
H	0.152540	4.386879	-1.190651
C	-1.590359	5.783630	1.987212
H	-2.293072	3.771224	2.294487
C	-0.845504	6.635448	1.166473
H	0.370072	6.789620	-0.608441
H	-2.054566	6.164985	2.892575
H	-0.735827	7.683956	1.430024
C	-0.147282	-0.321980	0.520864
C	0.580843	-0.263502	1.718310
C	-1.103521	-1.343302	0.402935
C	0.384627	-1.200963	2.736190
H	1.327724	0.512880	1.843812
C	-1.333900	-2.256381	1.435024
H	-1.655882	-1.443344	-0.522772
C	-0.582746	-2.197807	2.608570
H	-0.738516	-2.920814	3.403010
C	-2.361858	-3.344101	1.299155
C	1.314774	-1.236393	3.912770
F	-1.819158	-4.576849	1.389965
F	-3.035129	-3.285836	0.124223
F	2.328019	-2.133770	3.704594
F	-3.298467	-3.270153	2.285721
F	0.698837	-1.624574	5.050730
F	1.905007	-0.047664	4.150770
C	-3.074945	2.573526	-0.752424
H	-3.498982	3.406179	-0.209332
H	-2.710019	2.816231	-1.743387
C	-3.673886	1.318177	-0.566962
O	-3.487583	0.328197	-1.412126
Si	-3.588756	0.130263	-3.149007
C	-5.438588	0.265483	-3.483738
H	-5.640060	0.141068	-4.555070
H	-5.837829	1.242462	-3.186057
H	-6.001026	-0.507018	-2.947456
C	-2.645229	1.463779	-4.072085
H	-2.577712	1.174681	-5.128789
H	-3.136131	2.442488	-4.030451
H	-1.622944	1.556082	-3.695675
C	-2.934629	-1.592518	-3.450786
H	-1.844152	-1.586562	-3.540955
H	-3.216066	-2.277471	-2.643012
H	-3.351823	-1.989744	-4.384838
C	-4.420750	0.965565	0.655678
C	-4.667951	-0.388183	0.952249
C	-4.897957	1.950670	1.544775
C	-5.351088	-0.746687	2.110456
H	-4.299193	-1.154183	0.282596
C	-5.595162	1.587952	2.692288
H	-4.746686	3.003851	1.334793
C	-5.819700	0.238795	2.981267
H	-5.501010	-1.797303	2.335775
H	-5.966522	2.358569	3.361598
H	-6.357831	-0.041269	3.882569

TS 173

B3LYP/6-31G(d) = -3573.930812
B3LYP-D3(BJ)/def2-TZVPP/IEFPCM(propanonitrile) = -3575.501487
B3LYP-D3(BJ)/def2-TZVPP/IEFPCM(propanonitrile)//B3LYP-D3(BJ)/6-31G(d) Free Energy (Quasiharmonic) = -3574.785142

Frequencies (Top 3 out of 300)

1. -268.0396 cm^{-1}
2. 10.7550 cm^{-1}
3. 12.0514 cm^{-1}

B3LYP/6-31G(d) Molecular Geometry in Cartesian Coordinates

B	-0.297464	0.714632	-0.357833
O	-0.949481	-0.443464	-1.021339
N	0.682816	1.176182	-1.477595
C	0.418626	0.455575	-2.731195
S	1.396684	2.673840	-1.523835
C	-0.687016	-0.520311	-2.331520
O	-1.260123	-1.273045	-3.092017
H	2.376182	0.442935	-3.630491
H	0.019391	1.134944	-3.494501
C	1.617799	-0.302779	-3.370895
C	2.224541	-1.404438	-2.546945
C	1.879965	-2.810945	-2.567602
C	2.712613	-3.460817	-1.614330
N	3.538586	-2.499215	-1.067900
C	3.227357	-1.270491	-1.617046
H	3.760310	-0.384690	-1.303852
C	1.720310	-5.572858	-2.100949
C	0.964673	-3.587813	-3.301956
H	3.275854	-5.306562	-0.619994
H	4.103858	-2.625463	-0.242003
C	0.895592	-4.954574	-3.063872
H	1.637791	-6.643611	-1.933370
H	0.303805	-3.119257	-4.024071
C	2.638700	-4.835361	-1.363804
H	0.189184	-5.560533	-3.625317
C	3.171543	2.419655	-1.345513
O	0.976903	3.395762	-0.314643
O	1.191898	3.253226	-2.861052
C	5.936719	2.056277	-1.052096
C	4.005868	2.540070	-2.456025
C	3.701300	2.135579	-0.083886
C	5.075056	1.951630	0.051900
C	5.380391	2.352746	-2.302677
H	3.579668	2.791159	-3.421499
H	3.045013	2.066775	0.777111

H	5.487963	1.729390	1.033234
H	6.030369	2.444896	-3.169514
C	7.428337	1.888071	-0.884815
H	7.664777	1.129093	-0.131291
H	7.895833	2.826089	-0.557071
H	7.906998	1.595495	-1.824782
H	1.246251	-0.709005	-4.319137
C	0.375108	0.341717	1.058895
C	1.137296	-0.828831	1.191046
C	0.236274	1.147929	2.197440
C	1.740256	-1.175330	2.404583
H	1.250637	-1.487577	0.336272
C	0.826181	0.799647	3.416618
H	-0.342399	2.062142	2.130586
C	1.588560	-0.362398	3.528093
H	2.045780	-0.632912	4.472510
C	0.555120	1.628700	4.640386
C	2.572171	-2.421941	2.492436
F	-0.595723	1.243497	5.256008
F	0.415613	2.939559	4.351337
F	1.538750	1.517681	5.562467
F	2.737659	-2.847260	3.763272
F	2.034549	-3.445793	1.790797
F	3.830511	-2.236430	1.987719
O	-1.375412	1.770263	-0.090322
C	-2.483802	1.895750	-0.757169
C	-2.984382	3.276790	-0.967180
C	-2.364021	4.360752	-0.328074
C	-4.043442	3.511142	-1.859681
C	-2.815295	5.657603	-0.568361
H	-1.506159	4.179398	0.309019
C	-4.488532	4.807521	-2.097236
H	-4.510948	2.674290	-2.374622
C	-3.878536	5.883868	-1.444708
H	-2.324575	6.494869	-0.080414
H	-5.303374	4.981868	-2.794279
H	-4.224227	6.897063	-1.631076
H	-2.673696	1.187206	-1.566897
C	-4.057496	1.140282	0.374436
H	-4.844418	1.488910	-0.283608
H	-3.881864	1.726483	1.268288
C	-3.863247	-0.241539	0.430218
C	-3.202825	-0.907233	1.563868
C	-3.383675	-0.415014	2.871233
C	-2.372371	-2.025742	1.364465
C	-2.755524	-1.029301	3.949988
H	-4.038615	0.432994	3.043219
C	-1.740680	-2.632453	2.445941
H	-2.162237	-2.360874	0.356926
C	-1.934560	-2.139858	3.739022
H	-2.897912	-0.637633	4.952270
H	-1.065835	-3.466020	2.275053
H	-1.434905	-2.611863	4.580274
O	-4.213170	-0.932402	-0.638555
Si	-4.844743	-2.477369	-1.102756
C	-3.457501	-3.577821	-1.711790
H	-2.698069	-2.986199	-2.237575
H	-2.966028	-4.129858	-0.903868
H	-3.859839	-4.316037	-2.417298
C	-6.001442	-2.015057	-2.506864
H	-6.780026	-1.317380	-2.178384
H	-5.446572	-1.543598	-3.325839
H	-6.497989	-2.905454	-2.911637
C	-5.782935	-3.191767	0.363362
H	-6.276910	-4.124466	0.062817
H	-5.120844	-3.423622	1.203759
H	-6.560866	-2.507374	0.721192

TS 174

B3LYP/6-31G(d) = -3573.927599
B3LYP-D3(BJ)/def2-TZVPP/IEFPCM(propanonitrile) = -3575.502409
B3LYP-D3(BJ)/def2-TZVPP/IEFPCM(propanonitrile)//B3LYP-D3(BJ)/6-31G(d) Free Energy (Quasiharmonic) = -3574.785032

Frequencies (Top 3 out of 300)

1. -259.7662 cm^{-1}
2. 13.6653 cm^{-1}
3. 16.4419 cm^{-1}

B3LYP/6-31G(d) Molecular Geometry in Cartesian Coordinates

B	-0.365672	-0.925704	0.712385
O	-0.063656	-2.245011	1.318119
N	0.008407	-1.199061	-0.786775
C	0.290487	-2.632481	-0.985299
S	-0.527437	-0.306679	-2.074231
C	0.341924	-3.170711	0.449078
O	0.676037	-4.295838	0.747104
H	1.301392	-2.858236	-2.867934
H	-0.571825	-3.132395	-1.456860
C	1.534167	-3.036056	-1.815472
C	2.843259	-2.369534	-1.494408
C	3.880046	-2.807418	-0.585260
C	4.971016	-1.899701	-0.722678
N	4.598973	-0.955318	-1.657887
C	3.328885	-1.245665	-2.118050
H	2.851307	-0.630655	-2.866181
C	6.254074	-3.117444	0.879246
C	4.003571	-3.879629	0.317958
H	6.987406	-1.346561	-0.127581
H	5.148730	-0.163836	-1.954822
C	5.184367	-4.023193	1.036894
H	7.167977	-3.262771	1.449551
H	3.176405	-4.567551	0.464464
C	6.161451	-2.042435	0.000409
H	5.286372	-4.847810	1.737067
C	-2.145277	-0.922056	-2.560953
O	0.362781	-0.552864	-3.223075
O	-0.704049	1.072163	-1.585728
C	-4.666702	-1.967181	-3.201631
C	-2.240960	-2.030173	-3.406344
C	-3.297029	-0.307146	-2.062251
C	-4.543805	-0.830655	-2.388184
C	-3.500049	-2.546224	-3.715597
H	-1.345283	-2.467851	-3.834164
H	-3.210330	0.567735	-1.428837
H	-5.437866	-0.362519	-1.985829
H	-3.574307	-3.414111	-4.366145
C	-6.029262	-2.547117	-3.494140
H	-6.679549	-1.813455	-3.986090
H	-6.526052	-2.846291	-2.563165
H	-5.963360	-3.426747	-4.141512
O	0.544183	0.092207	1.404841
C	1.842007	-0.011109	1.442006
H	2.341946	-0.380964	0.542392
H	1.624524	-4.120554	-1.681867
C	2.475076	-0.339433	2.737804
C	3.790219	-0.828742	2.767457
C	1.750855	-0.213446	3.934555
C	4.373252	-1.186638	3.980539
H	4.345556	-0.955373	1.842299
C	2.341594	-0.564952	5.144088
H	0.725968	0.140185	3.896412
C	3.653606	-1.049643	5.170014
H	5.383252	-1.585514	3.992035
H	1.777102	-0.473430	6.067791
H	4.109130	-1.330742	6.115783
C	-1.867301	-0.456149	1.070810
C	-2.183709	0.877368	1.356346

C	-2.912182	-1.391388	1.133967
C	-3.488489	1.265663	1.679764
H	-1.394095	1.620563	1.332533
C	-4.218817	-1.009330	1.446053
H	-2.704655	-2.440592	0.948995
C	-4.516962	0.326062	1.723060
H	-5.526195	0.623310	1.983299
C	-5.335491	-2.016132	1.423730
C	-3.775178	2.715492	1.942300
F	-5.988989	-2.011459	0.228789
F	-4.898503	-3.275941	1.621425
F	-2.833604	3.280529	2.741312
F	-6.270617	-1.755228	2.366080
F	-4.971363	2.914496	2.532714
F	-3.780298	3.446498	0.793876
C	2.608341	1.962523	1.323855
H	3.649836	1.669801	1.363248
H	2.148380	2.267341	2.256704
C	2.141732	2.567812	0.162475
O	0.939409	3.109027	0.177419
Si	0.241435	4.623172	-0.306658
C	-0.309625	5.397320	1.312281
H	-0.754053	6.384243	1.131335
H	0.530923	5.533620	2.002637
H	-1.064676	4.781058	1.810642
C	1.562026	5.683179	-1.131671
H	1.867040	5.296406	-2.108576
H	1.158408	6.692560	-1.283394
H	2.460198	5.781701	-0.511031
C	-1.204085	4.269111	-1.437722
H	-0.919922	3.659356	-2.299382
H	-1.997491	3.737450	-0.905247
H	-1.620246	5.216809	-1.803588
C	2.903015	2.545097	-1.105676
C	4.302220	2.692224	-1.098764
C	2.234386	2.383293	-2.332085
C	5.016278	2.690891	-2.295855
H	4.824676	2.847986	-0.159704
C	2.956295	2.360408	-3.524126
H	1.162773	2.215345	-2.335663
C	4.344460	2.521441	-3.511262
H	6.093040	2.837557	-2.282534
H	2.431882	2.204898	-4.462225
H	4.901926	2.517520	-4.444000

TS 175

B3LYP/6-31G(d) = -3573.925523
B3LYP-D3(BJ)/def2-TZVPP/IEFPCM(propanonitrile) = -3575.501737
B3LYP-D3(BJ)/def2-TZVPP/IEFPCM(propanonitrile)//B3LYP-D3(BJ)/6-31G(d) Free Energy (Quasiharmonic) = -3574.785023

Frequencies (Top 3 out of 300)

1. -225.3576 cm^{-1}
2. 8.4933 cm^{-1}
3. 11.1173 cm^{-1}

B3LYP/6-31G(d) Molecular Geometry in Cartesian Coordinates

B	0.002832	-0.352692	-0.876438
O	-0.152143	-0.933934	-2.232983
N	-0.641142	-1.462298	-0.008397
C	-0.890300	-2.680320	-0.807645
S	-0.697017	-1.506784	1.647792
C	-0.532871	-2.216806	-2.227165
O	-0.601526	-2.915670	-3.211612
H	-2.413808	-3.789964	0.219856
H	-0.176881	-3.468302	-0.530506
C	-2.279503	-3.358113	-0.776995
C	-3.491363	-2.540693	-1.136195
C	-4.822283	-2.774803	-0.623899
C	-5.681553	-1.803864	-1.209059
N	-4.914900	-1.049847	-2.071849
C	-3.603888	-1.489986	-2.014238
H	-2.854464	-1.015411	-2.630505
C	-7.553328	-2.655643	0.000535
C	-5.371925	-3.710827	0.270432
H	-7.682276	-0.974019	-1.354131
H	-5.193010	-0.168984	-2.478607
C	-6.726165	-3.641713	0.577498
H	-8.608776	-2.625844	0.257624
H	-4.749267	-4.484465	0.713608
C	-7.043645	-1.727965	-0.901140
H	-7.159119	-4.363539	1.265095
C	-2.221698	-0.740224	2.205595
O	-0.725075	-2.919967	2.052923
O	0.377431	-0.622156	2.146135
C	-4.607210	0.482661	3.015873
C	-3.385681	-1.499630	2.309560
C	-2.224696	0.618066	2.535629
C	-3.413665	1.215719	2.941052
C	-4.570335	-0.882740	2.708442
H	-3.370502	-2.559605	2.088232
H	-1.305902	1.189283	2.478141
H	-3.418018	2.273323	3.194047
H	-5.480024	-1.474369	2.762279
C	-5.902476	1.165510	3.381451
H	-6.284848	1.736965	2.525680
H	-6.672441	0.443092	3.668786
H	-5.767445	1.870781	4.209287
H	-2.180071	-4.209660	-1.464987
C	-0.617861	1.133160	-0.793533
C	0.160598	2.299586	-0.838690
C	-2.009683	1.301412	-0.783185
C	-0.423878	3.569437	-0.874635
C	-2.601836	2.565989	-0.853755
H	-2.648398	0.426927	-0.723089
C	-1.812348	3.712789	-0.897300
H	-2.265709	4.695391	-0.951433
C	-4.099761	2.660792	-0.929511
C	0.439126	4.799104	-0.833135
F	-4.570721	2.164735	-2.112569
F	-4.709616	1.945001	0.041758
F	-4.547722	3.930101	-0.838453
F	-0.124645	5.842353	-1.481491
F	0.655935	5.218157	0.446484
F	1.658131	4.593651	-1.378004
O	1.550684	-0.197556	-0.704871
C	2.351620	-1.216336	-0.715859
C	3.481913	-1.240627	-1.663355
C	4.095184	-2.465395	-1.970950
C	3.909545	-0.071865	-2.312000
C	5.121462	-2.522523	-2.911509
H	3.746795	-3.376989	-1.490465
C	4.940519	-0.131545	-3.246145
H	3.413483	0.867182	-2.093303
C	5.549110	-1.353378	-3.547405
H	5.575444	-3.477526	-3.160825
H	5.261640	0.774592	-3.752015
H	6.343642	-1.397385	-4.287275
H	1.920095	-2.191790	-0.472270
C	3.298347	-1.294910	1.214409
H	2.356452	-1.090546	1.709796
H	3.622522	-2.329884	1.205710
C	4.279609	-0.317126	1.132692
C	4.020904	1.121513	1.251787
C	5.015514	2.044146	0.864923
C	2.785165	1.607457	1.726396
C	4.774512	3.411399	0.932784
H	5.963202	1.673535	0.492589

C	2.556622	2.978747	1.795521
H	2.002971	0.924066	2.033918
C	3.540967	3.883059	1.391414
H	5.543247	4.111434	0.617806
H	1.596749	3.345945	2.142953
H	3.339714	4.949494	1.424104
O	5.526528	-0.631656	0.772070
Si	6.701960	-1.759230	1.319235
C	8.133885	-1.440923	0.152581
H	7.829718	-1.606567	-0.887039
H	8.496290	-0.410338	0.236273
H	8.974882	-2.110452	0.369253
C	6.110517	-3.541085	1.191563
H	6.950376	-4.214395	1.406853
H	5.315405	-3.778081	1.906796
H	5.750953	-3.775595	0.184145
H	1.240591	2.217276	-0.847275
C	7.090519	-1.308736	3.102359
H	7.444663	-0.275348	3.186703
H	6.208067	-1.414128	3.744256
H	7.871446	-1.964968	3.505918

TS 176

B3LYP/6-31G(d) = -3573.931441
B3LYP-D3(BJ)/def2-TZVPP/IEFPCM(propanonitrile) = -3575.501067
B3LYP-D3(BJ)/def2-TZVPP/IEFPCM(propanonitrile)//B3LYP-D3(BJ)/6-31G(d) Free Energy (Quasiharmonic) = -3574.7849

Frequencies (Top 3 out of 300)

1. -258.0049 cm^{-1}
2. 6.5771 cm^{-1}
3. 11.1599 cm^{-1}

B3LYP/6-31G(d) Molecular Geometry in Cartesian Coordinates

B	0.203539	-0.721805	0.330371
O	-0.644995	-0.366176	1.498525
N	-0.343422	0.271096	-0.737811
C	-1.105608	1.346785	-0.070393
S	0.389857	0.526143	-2.214295
C	-1.380639	0.727209	1.306323
O	-2.157786	1.169454	2.129145
H	-3.246461	1.629439	-0.090583
H	-0.464222	2.222324	0.115283
C	-2.413246	1.825653	-0.773096
C	-2.429504	3.280353	-1.149368
C	-2.628218	4.400288	-0.257785
C	-2.600528	5.581502	-1.050614
N	-2.391890	5.187736	-2.357330
C	-2.288562	3.808640	-2.409327
H	-2.104722	3.297628	-3.343457
C	-2.966078	6.938741	0.875197
C	-2.828608	4.515988	1.130078
H	-2.744822	7.748725	-1.119034
H	-2.321093	5.804943	-3.150796
C	-2.995142	5.781347	1.680378
H	-3.098920	7.914109	1.335948
H	-2.839596	3.627589	1.755950
C	-2.768213	6.855873	-0.498852
H	-3.147670	5.884376	2.751525
C	1.770933	1.646017	-1.964725
O	0.935086	-0.783107	-2.604749
O	-0.572438	1.208871	-3.091401
C	3.926364	3.340873	-1.386991
C	1.535112	3.000828	-1.711191
C	3.072484	1.141550	-1.988583
C	4.137613	1.991861	-1.704257
C	2.614363	3.833409	-1.418249
H	0.527421	3.402054	-1.753206
H	3.245796	0.098006	-2.219318
H	5.147632	1.589561	-1.712608
H	2.431524	4.883711	-1.204809
C	5.080118	4.225353	-0.981988
H	5.307506	4.081775	0.082334
H	4.850260	5.285144	-1.131430
H	5.988511	3.988051	-1.546227
O	-0.048490	-2.180597	-0.047086
C	-0.883165	-2.601704	-0.945694
H	-1.503639	-1.856719	-1.444759
H	-2.561933	1.210681	-1.661893
C	-0.483603	-3.779952	-1.747439
C	-1.088936	-3.999475	-2.993732
C	0.514914	-4.656630	-1.297892
C	-0.703800	-5.083167	-3.778945
H	-1.843197	-3.304406	-3.354997
C	0.894967	-5.741289	-2.084213
H	0.994965	-4.469551	-0.342805
C	0.285513	-5.958534	-3.323301
H	-1.165465	-5.239114	-4.749771
H	1.673504	-6.413803	-1.735433
H	0.587978	-6.802599	-3.936899
C	1.772113	-0.637146	0.719598
C	2.739587	-1.456351	0.121011
C	2.225977	0.313588	1.647427
C	4.098322	-1.319002	0.417407
H	2.430606	-2.197515	-0.607941
C	3.583184	0.455955	1.947898
H	1.511231	0.953703	2.155678
C	4.531178	-0.361099	1.332449
H	5.584341	-0.256613	1.565744
C	4.038332	1.546092	2.878293
C	5.103934	-2.130641	-0.347337
F	3.122686	1.818064	3.832769
F	5.195740	1.231008	3.503650
F	4.665832	-3.383732	-0.601356
F	4.262716	2.709757	2.213132
F	6.284433	-2.238257	0.301900
F	5.373643	-1.562748	-1.554558
C	-2.499792	-3.573434	0.065545
H	-1.870409	-4.208420	0.677965
H	-2.913278	-4.048985	-0.812511
C	-3.255363	-2.588411	0.699451
C	-4.333989	-1.832266	0.029158
C	-4.854014	-2.224328	-1.220022
C	-4.874698	-0.695758	0.661128
C	-5.881900	-1.501332	-1.816467
H	-4.471529	-3.104299	-1.726148
C	-5.902303	0.024755	0.058409
H	-4.461303	-0.370626	1.608341
C	-6.409817	-0.375062	-1.179366
H	-6.274859	-1.818796	-2.777973
H	-6.301246	0.905206	0.553430
H	-7.211956	0.188405	-1.647682
O	-3.004169	-2.189583	1.926491
Si	-2.316448	-2.843227	3.381964
C	-3.377840	-4.347692	3.785150
H	-3.300884	-5.126319	3.017417
H	-3.059076	-4.792133	4.736214
H	-4.434529	-4.074820	3.884048
C	-0.512575	-3.323773	3.191970
H	0.091819	-2.465881	2.887469
H	-0.140800	-3.675878	4.163440
H	-0.351078	-4.130354	2.469201
C	-2.558192	-1.431026	4.582513
H	-1.992192	-1.592808	5.507533
H	-2.219044	-0.495607	4.123517
H	-3.614308	-1.311374	4.850014

TS 177

B3LYP/6-31G(d) = -3573.931442
B3LYP-D3(BJ)/def2-TZVPP/IEFPCM(propanonitrile) = -3575.501059
B3LYP-D3(BJ)/def2-TZVPP/IEFPCM(propanonitrile)//B3LYP-D3(BJ)/6-31G(d) Free Energy (Quasiharmonic) = -3574.784894

Frequencies (Top 3 out of 300)

1. -257.9815 cm^{-1}
2. 6.5544 cm^{-1}
3. 11.1560 cm^{-1}

B3LYP/6-31G(d) Molecular Geometry in Cartesian Coordinates

B	0.203512	-0.721731	0.329990
O	-0.645005	-0.365930	1.498113
N	-0.343109	0.271383	-0.738160
C	-1.105175	1.347173	-0.070782
S	0.390369	0.526378	-2.214555
C	-1.380376	0.727637	1.305914
O	-2.157447	1.170038	2.128727
H	-2.561233	1.211386	-1.662532
H	-0.463694	2.222634	0.114920
C	-2.412711	1.826189	-0.773591
C	-2.428881	3.280946	-1.149648
C	-2.627736	4.400785	-0.257977
C	-2.599890	5.582084	-1.050675
N	-2.391018	5.188458	-2.357396
C	-2.287704	3.809368	-2.409525
H	-2.103721	3.298451	-3.343679
C	-2.965781	6.939124	0.875210
C	-2.828369	4.516343	1.129863
H	-2.744155	7.749314	-1.118896
H	-2.320091	5.805750	-3.150784
C	-2.994995	5.781646	1.680265
H	-3.098701	7.914445	1.336039
H	-2.839455	3.627881	1.755642
C	-2.767666	6.856399	-0.498810
H	-3.147711	5.884564	2.751395
C	1.771593	1.646016	-1.964739
O	-0.571714	1.209337	-3.091716
O	0.935426	-0.782933	-2.605024
C	3.927213	3.340507	-1.386630
C	1.535955	3.000857	-1.711195
C	3.073068	1.141340	-1.988421
C	4.138290	1.991470	-1.703910
C	2.615298	3.833256	-1.418065
H	0.528336	3.402249	-1.753347
H	3.246245	0.097776	-2.219170
H	5.148249	1.589013	-1.712133
H	2.432597	4.883580	-1.204619
C	5.081057	4.224784	-0.981440
H	4.851318	5.284627	-1.130688
H	5.308415	4.080980	0.082859
H	5.989434	3.987488	-1.545707
H	-3.246056	1.629854	-0.091269
C	1.772078	-0.637556	0.719335
C	2.739357	-1.456955	0.120700
C	2.226150	0.312928	1.647321
C	4.098112	-1.320034	0.417208
C	3.583376	0.454862	1.947905
H	1.511552	0.953188	2.155599
C	4.531178	-0.362384	1.332412
H	5.584356	-0.258234	1.565797
C	4.038803	1.544714	2.878499
C	5.103541	-2.131867	-0.347572
F	5.195844	1.228977	3.504207
F	3.123004	1.817134	3.832697
F	4.264035	2.708285	2.213460
F	6.283964	-2.239906	0.301732
F	4.665092	-3.384801	-0.601772
F	5.373492	-1.563905	-1.554702
O	-0.048898	-2.180416	-0.047625
C	-0.884092	-2.601184	-0.945917
C	-0.485076	-3.779245	-1.748210
C	-1.091097	-3.998351	-2.994243
C	0.513585	-4.656156	-1.299440
C	-0.706481	-5.081853	-3.779973
H	-1.845499	-3.303118	-3.354896
C	0.893114	-5.740624	-2.086274
H	0.994156	-4.469406	-0.344550
C	0.282987	-5.957448	-3.325105
H	-1.168677	-5.237476	-4.750598
H	1.671766	-6.413317	-1.738099
H	0.585047	-6.801364	-3.939108
H	-1.504721	-1.855978	-1.444469
C	-2.500257	-3.573026	0.065922
H	-2.914036	-4.048531	-0.812020
H	-1.870584	-4.207995	0.678063
C	-3.255682	-2.588096	0.700152
C	-4.334681	-1.832111	0.030274
C	-4.874435	-0.694940	0.661869
C	-4.855992	-2.225001	-1.218107
C	-5.902333	0.025458	0.059508
H	-4.460078	-0.369214	1.608462
C	-5.884198	-1.502139	-1.814167
H	-4.474322	-3.105563	-1.723825
C	-6.411125	-0.375172	-1.177481
H	-6.300519	0.906439	0.554197
H	-6.278192	-1.820261	-2.775032
H	-7.213509	0.188194	-1.645500
O	-3.004024	-2.189174	1.927059
Si	-2.315717	-2.842629	3.382338
C	-2.556779	-1.430207	4.582763
H	-1.990376	-1.591889	5.507553
H	-2.217760	-0.494890	4.123465
H	-3.612764	-1.310418	4.850719
C	-0.511996	-3.323473	3.191663
H	-0.139883	-3.675468	4.163045
H	-0.350924	-4.130219	2.468982
H	0.092426	-2.465741	2.886767
H	2.430210	-2.197922	-0.608381
C	-3.377121	-4.346896	3.786224
H	-3.300568	-5.125666	3.018593
H	-3.058027	-4.791211	4.737236
H	-4.433737	-4.073879	3.885499

TS 178

B3LYP/6-31G(d) = -3573.929798
B3LYP-D3(BJ)/def2-TZVPP/IEFPCM(propanonitrile) = -3575.501866
B3LYP-D3(BJ)/def2-TZVPP/IEFPCM(propanonitrile)//B3LYP-D3(BJ)/6-31G(d) Free Energy (Quasiharmonic) = -3574.784785

Frequencies (Top 3 out of 300)

1. -276.6466 cm^{-1}
2. 10.4903 cm^{-1}
3. 12.5765 cm^{-1}

B3LYP/6-31G(d) Molecular Geometry in Cartesian Coordinates

B	0.439403	0.484427	-0.302021
O	0.942566	0.044232	-1.615497
N	1.242823	1.803424	-0.108468
C	2.093706	2.079268	-1.273887
S	1.089737	2.892013	1.117999
C	1.812579	0.879262	-2.182276
O	2.307995	0.708871	-3.274859
H	3.773858	3.210351	-0.536148

H	1.738988	2.974330	-1.808949
C	3.621576	2.264297	-1.059790
C	4.356947	1.173357	-0.331986
C	5.028989	0.021985	-0.895603
C	5.644400	-0.676715	0.181400
N	5.331323	0.006783	1.340839
C	4.581794	1.127180	1.020923
H	4.243147	1.812600	1.784638
C	6.561041	-2.294303	-1.312664
C	5.197120	-0.473948	-2.201429
H	6.871999	-2.348259	0.827896
H	5.737717	-0.170793	2.246234
C	5.961181	-1.620019	-2.396559
H	7.154531	-3.185849	-1.497557
H	4.720184	0.024192	-3.039647
C	6.408254	-1.832651	-0.009842
H	6.105808	-2.001684	-3.404309
C	0.159626	4.291391	0.465661
O	2.412771	3.433065	1.469829
O	0.270551	2.245338	2.154770
C	-1.272830	6.459942	-0.596307
C	0.834375	5.294149	-0.236668
C	-1.220179	4.373699	0.663037
C	-1.922076	5.455093	0.134032
C	0.115000	6.364359	-0.765480
H	1.913681	5.253715	-0.337448
H	-1.736807	3.618076	1.241954
H	-2.994750	5.521602	0.299948
H	0.643845	7.144901	-1.307347
C	-2.050118	7.608427	-1.194247
H	-1.430492	8.506105	-1.289653
H	-2.415475	7.356083	-2.198708
H	-2.924380	7.861000	-0.585151
H	4.035632	2.380215	-2.068646
C	-1.190795	0.517667	-0.348269
C	-2.029600	0.880310	0.718996
C	-1.830501	0.128802	-1.539999
C	-3.424778	0.869114	0.597770
C	-3.221746	0.101132	-1.659839
H	-1.222475	-0.162164	-2.388672
C	-4.035476	0.476108	-0.590060
H	-5.114817	0.470465	-0.683967
C	-3.847965	-0.416378	-2.923628
C	-4.249497	1.258639	1.790679
F	-3.957984	-1.779382	-2.899897
F	-3.124977	-0.114902	-4.020139
F	-5.097731	0.060054	-3.111790
F	-4.120905	0.353532	2.798785
F	-3.865369	2.452047	2.303726
F	-5.566315	1.347986	1.509166
O	0.981363	-0.525705	0.729913
C	0.320376	-1.185271	1.630178
C	0.990967	-1.403503	2.937385
C	2.386639	-1.328294	3.048282
C	0.217407	-1.659982	4.078623
C	2.993950	-1.522598	4.286341
H	2.978190	-1.100024	2.168008
C	0.829469	-1.845625	5.316458
H	-0.866989	-1.688287	3.999780
C	2.220894	-1.783633	5.421275
H	4.075902	-1.463151	4.371132
H	0.222612	-2.029529	6.198530
H	2.700346	-1.929078	6.385431
H	-0.760871	-1.045057	1.670819
C	0.184433	-3.189821	1.081576
H	1.250488	-3.373930	1.095142
H	-0.342805	-3.547329	1.958812
C	-0.481141	-3.273884	-0.146654
C	0.176702	-3.133510	-1.444383
C	1.579651	-3.039784	-1.554095
C	-0.601941	-3.082132	-2.620781
C	2.183210	-2.892830	-2.796580
H	2.207564	-3.060974	-0.671417
C	0.007849	-2.934422	-3.859746
H	-1.681866	-3.123857	-2.547176
C	1.399687	-2.835422	-3.950519
H	3.261906	-2.792630	-2.857800
H	-0.602829	-2.879489	-4.756300
H	1.872429	-2.702191	-4.919249
O	-1.803170	-3.394822	-0.203740
Si	-3.022790	-4.125234	0.775326
C	-2.443540	-5.866036	1.193989
H	-3.224857	-6.398268	1.750771
H	-2.234880	-6.441790	0.285169
H	-1.538280	-5.872089	1.810885
C	-4.505163	-4.127422	-0.368987
H	-5.426786	-4.360391	0.177309
H	-4.629864	-3.153165	-0.852173
H	-4.388287	-4.873702	-1.163009
H	-1.587044	1.205369	1.654751
C	-3.334523	-3.116258	2.330548
H	-3.536613	-2.060876	2.120109
H	-4.215530	-3.518117	2.847841
H	-2.497697	-3.169154	3.035892

TS 179

B3LYP/6-31G(d) = -3573.926208
B3LYP-D3(BJ)/def2-TZVPP/IEFPCM(propanonitrile) = -3575.502492
B3LYP-D3(BJ)/def2-TZVPP/IEFPCM(propanonitrile)//B3LYP-D3(BJ)/6-31G(d) Free Energy (Quasiharmonic) = -3574.784769

Frequencies (Top 3 out of 300)

1. -289.8352 cm^{-1}
2. 8.1874 cm^{-1}
3. 14.5026 cm^{-1}

B3LYP/6-31G(d) Molecular Geometry in Cartesian Coordinates

B	0.272641	0.297319	-0.929640
O	-0.847258	1.211200	-1.284650
N	0.542830	-0.416771	-2.306920
C	-0.500430	-0.008810	-3.276160
S	2.037990	-0.648532	-3.018650
C	-1.286559	1.065721	-2.538140
O	-2.191318	1.724531	-3.011620
H	-0.738701	-1.861690	-4.299960
H	-0.062699	0.465270	-4.162870
C	-1.404141	-1.160739	-3.787980
C	-2.231401	-1.866959	-2.751780
C	-3.600511	-1.582447	-2.382180
C	-3.973022	-2.521507	-1.380270
N	-2.878992	-3.332428	-1.158240
C	-1.841932	-2.933389	-1.978920
H	-0.890942	-3.445620	-1.945580
C	-6.164281	-1.593085	-1.245850
C	-4.552160	-0.637097	-2.807730
H	-5.508492	-3.259136	-0.032240
H	-2.825663	-4.068108	-0.471580
C	-5.819750	-0.652756	-2.239090
H	-7.162941	-1.577544	-0.817460
H	-4.289649	0.106873	-3.553720
C	-5.246862	-2.537816	-0.802270
H	-6.560729	0.073965	-2.561730
C	2.853509	-1.813213	-1.928190
O	2.846991	0.585857	-3.029020
O	1.791579	-1.321482	-4.304890
C	4.149317	-3.652644	-0.260030
C	3.964699	-1.408524	-1.191700
C	2.400128	-3.134063	-1.872070

C	3.044507	-4.039843	-1.036570
C	4.603228	-2.332154	-0.361610
H	4.317790	-0.386894	-1.276050
H	1.559988	-3.443322	-2.485020
H	2.687696	-5.065293	-0.982120
H	5.468919	-2.019775	0.217430
C	4.804137	-4.634325	0.680350
H	5.791747	-4.288065	1.000490
H	4.921746	-5.619215	0.214850
H	4.186816	-4.766164	1.576640
H	-2.061610	-0.729409	-4.552510
C	-0.128450	-0.661080	0.315280
C	0.801839	-1.489391	0.960820
C	-1.429200	-0.638629	0.839490
C	0.445239	-2.275231	2.062080
H	1.828549	-1.520082	0.613110
C	-1.783151	-1.394409	1.960020
H	-2.176420	-0.008359	0.371700
C	-0.850441	-2.229720	2.575170
H	-1.122912	-2.820490	3.442290
C	-3.134241	-1.219288	2.589990
C	1.457238	-3.202642	2.673820
F	-4.069740	-0.794027	1.718020
F	-3.092500	-0.288038	3.591790
F	-3.590532	-2.359817	3.156360
F	2.699628	-2.664713	2.691300
F	1.558667	-4.370432	1.984420
F	1.147247	-3.534642	3.948220
O	1.451241	1.120508	-0.443290
C	1.686562	2.341028	-0.857840
C	3.090733	2.809257	-0.763450
C	4.002812	2.185856	0.101510
C	3.514814	3.889506	-1.550190
C	5.317213	2.644105	0.178100
H	3.673632	1.337836	0.692400
C	4.830264	4.341105	-1.475340
H	2.815384	4.364247	-2.234230
C	5.733694	3.722265	-0.607330
H	6.021262	2.154804	0.845880
H	5.153705	5.169275	-2.099520
H	6.760214	4.073564	-0.549790
H	1.136523	2.673419	-1.737530
C	0.779064	3.775279	0.319190
H	1.677704	4.218048	0.725530
H	0.304714	4.370319	-0.453050
C	-0.085987	3.168800	1.253230
C	0.396703	2.537399	2.491180
C	1.738653	2.648608	2.908580
C	-0.499838	1.799040	3.288550
C	2.163032	2.042468	4.086290
H	2.457183	3.205337	2.318320
C	-0.068469	1.186180	4.459140
H	-1.528068	1.687401	2.971990
C	1.262682	1.309338	4.864320
H	3.198982	2.140107	4.397580
H	-0.773249	0.603060	5.043590
H	1.599831	0.833068	5.780870
O	-1.369517	3.034181	1.038160
Si	-2.698006	3.881172	0.288840
C	-3.182725	5.132222	1.611150
H	-2.372005	5.840812	1.818350
H	-4.053425	5.713933	1.283970
H	-3.447026	4.637482	2.552350
C	-2.235115	4.737031	-1.314710
H	-1.471915	5.514231	-1.199390
H	-1.920356	4.007991	-2.067710
H	-3.137545	5.228562	-1.702950
C	-3.991847	2.559863	0.031370
H	-3.754228	1.958773	-0.851830
H	-4.972547	3.023564	-0.134500
H	-4.083098	1.889933	0.893110

TS 180

B3LYP/6-31G(d) = -3573.922217
B3LYP-D3(BJ)/def2-TZVPP/IEFPCM(propanonitrile) = -3575.501443
B3LYP-D3(BJ)/def2-TZVPP/IEFPCM(propanonitrile)//B3LYP-D3(BJ)/6-31G(d) Free Energy (Quasiharmonic) = -3574.784769

Frequencies (Top 3 out of 300)

1. -246.0488 cm^{-1}
2. 8.7736 cm^{-1}
3. 12.6235 cm^{-1}

B3LYP/6-31G(d) Molecular Geometry in Cartesian Coordinates

B	1.016651	-0.347215	-1.076233
O	0.995715	-1.187329	-2.285190
N	0.761076	-1.421451	0.053701
C	0.931585	-2.769630	-0.531872
S	1.091367	-1.209201	1.667493
C	0.919052	-2.493023	-2.039687
O	0.893626	-3.352630	-2.892362
H	0.254971	-4.247880	0.873411
H	1.946778	-3.146922	-0.324122
C	-0.036486	-3.908103	-0.122672
C	-1.515907	-3.638871	-0.116658
C	-2.458509	-3.705149	-1.214075
C	-3.754897	-3.485050	-0.667023
N	-3.598504	-3.296073	0.691677
C	-2.258803	-3.392297	1.013803
H	-1.919316	-3.276057	2.032877
C	-4.765090	-3.725780	-2.813128
C	-2.343428	-3.944400	-2.596591
H	-5.893970	-3.323854	-1.010819
H	-4.339372	-3.108088	1.348469
C	-3.492953	-3.951567	-3.377416
H	-5.644651	-3.739811	-3.451650
H	-1.367963	-4.104990	-3.044489
C	-4.913417	-3.489684	-1.451244
H	-3.411031	-4.133599	-4.445626
C	2.859222	-1.342692	1.955513
O	0.463874	-2.308988	2.418099
O	0.705462	0.183606	1.980987
C	5.637411	-1.545024	2.276134
C	3.466090	-2.602348	1.961479
C	3.617453	-0.188296	2.161933
C	4.995431	-0.299544	2.324196
C	4.848839	-2.691971	2.113726
H	2.865740	-3.501688	1.874408
H	3.136432	0.780942	2.194533
H	5.580965	0.603352	2.478471
H	5.322024	-3.670680	2.107234
C	7.140634	-1.637934	2.366686
H	7.468248	-2.645088	2.642746
H	7.590870	-1.394494	1.396014
H	7.544424	-0.932491	3.101140
H	0.189890	-4.722572	-0.821987
C	2.399660	0.498670	-0.994218
C	3.578538	-0.041469	-1.533082
C	2.498707	1.757498	-0.387681
C	4.801146	0.628276	-1.444246
C	3.719609	2.430774	-0.288675
H	1.616653	2.228943	0.032622
C	4.881573	1.870730	-0.815874
H	5.827554	2.394935	-0.747038
C	3.795529	3.707780	0.497238
C	6.054079	-0.026051	-1.958187
F	4.854902	4.469746	0.147788
F	3.922458	3.451536	1.828930

F	2.685808	4.465559	0.356970
F	5.821544	-0.793576	-3.042768
F	6.612011	-0.838732	-1.020739
F	7.000770	0.879433	-2.294143
O	-0.117998	0.644206	-1.280874
C	-0.912305	1.203368	-0.417343
C	-1.441435	2.536489	-0.789769
C	-1.872632	3.410638	0.220023
C	-1.416052	2.977046	-2.121538
C	-2.262272	4.711608	-0.096114
H	-1.873867	3.077950	1.255517
C	-1.816809	4.273832	-2.435186
H	-1.056135	2.304046	-2.892727
C	-2.236843	5.144832	-1.424742
H	-2.567301	5.392301	0.693998
H	-1.786326	4.612727	-3.466938
H	-2.531854	6.161325	-1.670460
H	-0.691725	1.044491	0.636712
C	-2.532188	-0.086898	-0.449204
H	-1.957392	-0.911680	-0.042148
H	-2.627902	-0.059808	-1.529178
C	-3.647640	0.346661	0.263908
C	-3.826636	0.101472	1.708970
C	-5.117502	-0.018406	2.258604
C	-2.715127	-0.081108	2.554407
C	-5.292225	-0.303622	3.610408
H	-5.985907	0.067147	1.616144
C	-2.895151	-0.350474	3.909384
H	-1.704132	-0.008664	2.172306
C	-4.180703	-0.462096	4.442849
H	-6.295869	-0.407479	4.013347
H	-2.021740	-0.480943	4.540927
H	-4.317358	-0.679323	5.498501
O	-4.584981	0.992440	-0.409796
Si	-5.869876	2.131353	-0.415693
C	-7.474196	1.147669	-0.486086
H	-8.298947	1.818147	-0.759284
H	-7.418710	0.367062	-1.253284
H	-7.745343	0.671280	0.462045
C	-5.633209	3.067062	-2.020859
H	-6.431751	3.807010	-2.157482
H	-4.674988	3.595642	-2.042496
H	-5.661020	2.383793	-2.877126
H	3.543249	-0.997988	-2.044960
C	-5.790276	3.269084	1.077695
H	-5.895823	2.735933	2.027305
H	-4.844612	3.820536	1.098398
H	-6.601887	4.005387	1.014023

TS 181

B3LYP/6-31G(d) = -3573.931583
B3LYP-D3(BJ)/def2-TZVPP/IEFPCM(propanonitrile) = -3575.500627
B3LYP-D3(BJ)/def2-TZVPP/IEFPCM(propanonitrile)//B3LYP-D3(BJ)/6-31G(d) Free Energy (Quasiharmonic) = -3574.784711

Frequencies (Top 3 out of 300)

1. -260.4747 cm^{-1}
2. 6.4652 cm^{-1}
3. 9.5922 cm^{-1}

B3LYP/6-31G(d) Molecular Geometry in Cartesian Coordinates

B	0.299924	-0.734257	0.290929
O	-0.498921	-0.601839	1.538831
N	-0.366674	0.358809	-0.595003
C	-1.158249	1.273801	0.253979
S	0.295380	0.892449	-2.034170
C	-1.302803	0.460457	1.547001
O	-2.056302	0.720971	2.464920
H	-3.279417	0.941484	-0.056313
H	-0.579578	2.177081	0.501434
C	-2.542130	1.712938	-0.300606
C	-2.990261	3.058199	0.204262
C	-2.997063	4.287459	-0.554844
C	-3.481347	5.313653	0.304125
N	-3.750552	4.725359	1.522887
C	-3.446840	3.377469	1.460012
H	-3.550578	2.740149	2.325944
C	-3.257516	6.938191	-1.427247
C	-2.638903	4.620663	-1.874816
H	-3.992263	7.410809	0.552128
H	-4.076327	5.207905	2.345485
C	-2.771141	5.938980	-2.295403
H	-3.350441	7.959763	-1.786269
H	-2.255819	3.859779	-2.549993
C	-3.619011	6.640074	-0.117972
H	-2.494951	6.207233	-3.311665
C	1.583259	2.084856	-1.653097
O	-0.745608	1.616894	-2.779113
O	0.931227	-0.293164	-2.626375
C	3.597889	3.887823	-0.914560
C	2.922823	1.701178	-1.747105
C	1.238758	3.378564	-1.249252
C	2.248850	4.264750	-0.876497
C	3.916548	2.604709	-1.381435
H	3.180462	0.710302	-2.099736
H	0.202625	3.701350	-1.245439
H	1.980858	5.267033	-0.551727
H	4.957758	2.299184	-1.450092
C	4.682957	4.821324	-0.436290
H	5.570696	4.769341	-1.076265
H	4.995547	4.546954	0.579573
H	4.340256	5.860474	-0.409885
H	-2.464359	1.744814	-1.387438
C	1.879874	-0.606558	0.617620
C	2.329772	0.237427	1.645157
C	2.857285	-1.284923	-0.123692
C	3.690899	0.414606	1.906520
H	1.609295	0.765141	2.262701
C	4.220028	-1.115042	0.134987
H	2.550664	-1.937794	-0.933380
C	4.648079	-0.262345	1.150949
H	5.704617	-0.132401	1.354142
C	5.225210	-1.772427	-0.766729
C	4.133539	1.398898	2.953449
F	4.840266	-3.012746	-1.143186
F	6.442558	-1.884674	-0.190029
F	5.398345	-1.056951	-1.909657
F	3.254806	1.486158	3.975791
F	4.258056	2.651497	2.441758
F	5.337210	1.076367	3.479572
O	0.113708	-2.136991	-0.284485
C	-0.771820	-2.482502	-1.168442
C	-0.363113	-3.469452	-2.193859
C	-1.069354	-3.532573	-3.404307
C	0.737476	-4.314473	-1.988527
C	-0.681276	-4.429758	-4.396248
H	-1.906967	-2.860122	-3.574024
C	1.120274	-5.212894	-2.981188
H	1.292746	-4.247521	-1.058556
C	0.411443	-5.274456	-4.184451
H	-1.222217	-4.462646	-5.337648
H	1.977894	-5.860132	-2.821186
H	0.716190	-5.972432	-4.959297
H	-1.489479	-1.723340	-1.482717
C	-2.183122	-3.749685	-0.201984
H	-1.442294	-4.391096	0.261058
H	-2.628161	-4.141527	-1.105587
C	-2.972068	-2.950452	0.624803

C	-4.180974	-2.242336	0.154479
C	-4.819253	-2.581778	-1.054271
C	-4.725073	-1.209136	0.942121
C	-5.964694	-1.905981	-1.462642
H	-4.438527	-3.388349	-1.671818
C	-5.868532	-0.533013	0.525006
H	-4.224459	-0.924668	1.860307
C	-6.492179	-0.878913	-0.675614
H	-6.449029	-2.182943	-2.394484
H	-6.266797	0.272993	1.134095
H	-7.384495	-0.350163	-0.998632
O	-2.646145	-2.685527	1.869719
Si	-1.777340	-3.445558	3.168858
C	-2.022643	-2.218628	4.557823
H	-1.361567	-2.438084	5.404580
H	-1.806388	-1.206575	4.197874
H	-3.055693	-2.233315	4.923737
C	0.033638	-3.740347	2.777410
H	0.190635	-4.433848	1.944667
H	0.544593	-2.803543	2.542711
H	0.514130	-4.178567	3.662358
C	-2.677306	-5.076506	3.456301
H	-3.739321	-4.913491	3.671598
H	-2.607241	-5.745564	2.590750
H	-2.241371	-5.603607	4.314086

TS 182

B3LYP/6-31G(d) = -3573.929865
B3LYP-D3(BJ)/def2-TZVPP/IEFPCM(propanonitrile) = -3575.500499
B3LYP-D3(BJ)/def2-TZVPP/IEFPCM(propanonitrile)//B3LYP-D3(BJ)/6-31G(d) Free Energy (Quasiharmonic) = -3574.784643

Frequencies (Top 3 out of 300)

1. -275.5965 cm^{-1}
2. 3.2314 cm^{-1}
3. 9.9143 cm^{-1}

B3LYP/6-31G(d) Molecular Geometry in Cartesian Coordinates

B	0.350604	0.338228	-0.141664
O	0.719808	-0.545976	-1.283584
N	-0.017961	-0.724922	0.937515
C	0.271912	-2.086995	0.453482
S	-0.074882	-0.436255	2.571802
C	0.735899	-1.840277	-0.981101
O	1.096719	-2.714829	-1.745645
H	-1.086745	-3.321279	1.578378
H	1.121123	-2.512848	1.004079
C	-0.863929	-3.148317	0.520261
C	-2.137736	-2.870079	-0.230510
C	-2.445480	-3.174097	-1.614101
C	-3.781994	-2.746597	-1.848402
N	-4.269264	-2.245377	-0.658375
C	-3.275341	-2.307597	0.297857
H	-3.468725	-1.962691	1.302546
C	-3.683458	-3.475828	-4.114992
C	-1.741699	-3.784027	-2.669491
H	-5.426750	-2.530957	-3.248184
H	-5.110548	-1.697354	-0.564477
C	-2.365319	-3.928986	-3.902100
H	-4.139787	-3.595438	-5.094133
H	-0.717139	-4.110183	-2.527670
C	-4.409404	-2.879906	-3.091447
H	-1.825194	-4.394145	-4.722609
C	-1.757520	-0.703774	3.146973
O	0.764495	-1.441605	3.250270
O	0.194147	0.996993	2.755908
C	-4.369542	-1.077259	4.090713
C	-2.061566	-1.835130	3.903596
C	-2.735568	0.254347	2.869348
C	-4.033077	0.056515	3.333726
C	-3.365512	-2.014828	4.367141
H	-1.281655	-2.553357	4.132565
H	-2.483199	1.139444	2.296302
H	-4.797355	0.793146	3.099859
H	-3.604226	-2.896988	4.956270
C	-5.771889	-1.262418	4.619801
H	-6.519276	-0.877224	3.918045
H	-5.993561	-2.317394	4.809877
H	-5.908636	-0.723008	5.566426
H	-0.409793	-4.074710	0.147589
C	-0.803217	1.395574	-0.549927
C	-0.517741	2.720931	-0.909361
C	-2.148946	1.005040	-0.592439
C	-1.522311	3.618809	-1.277205
C	-3.163343	1.901865	-0.946851
H	-2.410054	-0.015042	-0.329666
C	-2.857956	3.217286	-1.292734
H	-3.641802	3.916960	-1.556324
C	-4.588969	1.427674	-0.958673
C	-1.154209	5.013380	-1.701630
F	-4.834215	0.552389	-1.964200
F	-5.474308	2.437492	-1.090089
F	-4.911288	0.767894	0.192225
F	-0.843834	5.074324	-3.019659
F	-0.073296	5.477873	-1.030552
F	-2.162216	5.891677	-1.498228
O	1.586500	1.198820	0.177808
C	2.537013	0.953077	1.027838
C	3.060182	2.092282	1.824298
C	3.696565	1.830899	3.046941
C	2.906225	3.417136	1.394608
C	4.178563	2.879861	3.825716
H	3.785549	0.805167	3.396948
C	3.393305	4.463887	2.174074
H	2.390094	3.622721	0.463099
C	4.033089	4.198766	3.387719
H	4.656202	2.670559	4.778759
H	3.265566	5.489275	1.838656
H	4.407399	5.017946	3.995535
H	2.525851	-0.009653	1.542684
C	4.313993	0.592343	-0.032567
H	4.973475	0.740337	0.810586
H	4.244332	1.435280	-0.710950
C	4.228864	-0.695245	-0.569177
C	4.538768	-1.911315	0.210966
C	5.314981	-1.860770	1.385573
C	4.053260	-3.157942	-0.231106
C	5.595059	-3.022623	2.097331
H	5.723744	-0.919154	1.736585
C	4.329452	-4.314871	0.492715
H	3.432788	-3.201619	-1.118474
C	5.101382	-4.252644	1.654694
H	6.201813	-2.969475	2.996571
H	3.936732	-5.267278	0.148717
H	5.318528	-5.158092	2.214572
O	3.758229	-0.930035	-1.771415
Si	3.684898	-0.101124	-3.301512
C	5.479685	0.294298	-3.719164
H	6.092720	-0.613736	-3.742559
H	5.542049	0.762756	-4.709275
H	5.930061	0.987927	-2.999754
C	2.640630	1.454568	-3.229569
H	2.586098	1.891264	-4.235453
H	1.624207	1.218699	-2.904619
H	3.047523	2.221453	-2.562053
H	0.509347	3.064537	-0.889223
C	2.940603	-1.409083	-4.408143
H	2.645587	-0.990075	-5.377412

H	3.647391	-2.226053	-4.592785
H	2.054355	-1.832303	-3.922627

TS 183

B3LYP/6-31G(d) = -3573.92731
B3LYP-D3(BJ)/def2-TZVPP/IEFPCM(propanonitrile) = -3575.502077
B3LYP-D3(BJ)/def2-TZVPP/IEFPCM(propanonitrile)//B3LYP-D3(BJ)/6-31G(d) Free Energy (Quasiharmonic) = -3574.784444

Frequencies (Top 3 out of 300)

1. -271.0244 cm^{-1}
2. 14.0396 cm^{-1}
3. 19.4106 cm^{-1}

B3LYP/6-31G(d) Molecular Geometry in Cartesian Coordinates

B	0.175932	-0.248530	-0.155341
O	0.546272	0.851846	-1.093589
N	0.478235	-1.489225	-1.034400
C	0.711375	-1.092988	-2.429602
S	0.868888	-3.011227	-0.556540
C	0.801238	0.430121	-2.329352
O	1.065699	1.179765	-3.252247
H	-0.356901	-2.613897	-3.510736
H	1.685272	-1.458610	-2.786516
C	-0.363064	-1.521755	-3.466070
C	-1.752801	-1.015186	-3.195267
C	-2.839436	-1.722202	-2.556327
C	-3.949313	-0.834474	-2.514256
N	-3.558969	0.343346	-3.121182
C	-2.235342	0.234490	-3.505156
H	-1.732461	1.060545	-3.989589
C	-5.280667	-2.465070	-1.396151
C	-2.980533	-3.009515	-2.006358
H	-6.004725	-0.489484	-1.907610
H	-4.069190	1.210878	-3.058162
C	-4.194979	-3.364705	-1.431699
H	-6.213335	-2.770648	-0.929809
H	-2.143713	-3.701310	-2.003419
C	-5.173731	-1.189698	-1.938777
H	-4.309172	-4.350804	-0.990014
C	2.664011	-3.146492	-0.753830
O	0.308922	-3.993850	-1.498987
O	0.548664	-3.114359	0.875152
C	5.444009	-3.451912	-1.001671
C	3.186286	-4.303459	-1.334478
C	3.516224	-2.135904	-0.301469
C	4.894733	-2.294078	-0.432499
C	4.568095	-4.448397	-1.451957
H	2.510100	-5.069579	-1.697557
H	3.104753	-1.232188	0.135456
H	5.554399	-1.498314	-0.093683
H	4.971377	-5.349498	-1.908232
C	6.940198	-3.628808	-1.109752
H	7.342314	-4.139292	-0.224374
H	7.454014	-2.665058	-1.190290
H	7.211082	-4.232613	-1.982191
H	-0.006784	-1.152859	-4.435835
C	-1.347049	-0.057330	0.358671
C	-2.031970	1.134917	0.089840
C	-2.038487	-1.050439	1.074534
C	-3.349474	1.337503	0.512632
H	-1.528317	1.912149	-0.472844
C	-3.355735	-0.853553	1.494421
H	-1.547076	-1.995310	1.286696
C	-4.021180	0.342293	1.219405
H	-5.046600	0.486392	1.539841
C	-4.063525	-1.917958	2.289126
C	-4.066907	2.597121	0.131087
F	-5.397984	-1.916044	2.057520
F	-3.906201	-1.725663	3.625092
F	-3.604915	-3.153739	2.015502
F	-3.224256	3.662197	0.039392
F	-5.034679	2.931599	1.008943
F	-4.674007	2.500419	-1.087528
O	1.153696	-0.220783	1.023323
C	0.926710	0.451735	2.118540
C	1.320276	-0.173613	3.402770
C	2.070392	-1.359484	3.424943
C	0.881822	0.390211	4.612942
C	2.389270	-1.956165	4.643223
H	2.355336	-1.827500	2.490618
C	1.201002	-0.210788	5.826252
H	0.277219	1.294387	4.600716
C	1.963024	-1.383171	5.843423
H	2.958135	-2.881453	4.653752
H	0.849660	0.226506	6.756479
H	2.209921	-1.855688	6.790216
H	0.012593	1.046627	2.141357
C	2.107749	2.171127	2.258888
H	3.000005	1.716176	2.668066
H	1.449421	2.654984	2.972269
C	2.204954	2.768564	0.998656
C	3.373091	2.561252	0.124065
C	3.233778	2.520855	-1.275190
C	4.655724	2.393141	0.684865
C	4.342560	2.299514	-2.087764
H	2.254596	2.593912	-1.729001
C	5.763767	2.193498	-0.132944
H	4.790073	2.456176	1.759779
C	5.609292	2.141209	-1.521549
H	4.205957	2.236879	-3.163003
H	6.748776	2.085056	0.312046
H	6.474163	1.976573	-2.158365
O	1.167597	3.458206	0.573013
Si	0.757743	4.878583	-0.333024
C	-0.186868	4.358084	-1.864698
H	-1.201982	4.049556	-1.592350
H	-0.276075	5.214995	-2.545242
H	0.279111	3.533377	-2.413740
C	-0.356934	5.816500	0.851128
H	-1.251574	5.228440	1.084130
H	0.151232	6.058299	1.791503
H	-0.690333	6.759515	0.400029
C	2.323232	5.850825	-0.711819
H	2.919496	6.030708	0.190334
H	2.043158	6.830526	-1.119598
H	2.963614	5.354331	-1.446607

TS 184

B3LYP/6-31G(d) = -3573.931504
B3LYP-D3(BJ)/def2-TZVPP/IEFPCM(propanonitrile) = -3575.501107
B3LYP-D3(BJ)/def2-TZVPP/IEFPCM(propanonitrile)//B3LYP-D3(BJ)/6-31G(d) Free Energy (Quasiharmonic) = -3574.784411

Frequencies (Top 3 out of 300)

1. -264.4966 cm^{-1}
2. 10.7183 cm^{-1}
3. 12.9486 cm^{-1}

B3LYP/6-31G(d) Molecular Geometry in Cartesian Coordinates

B	0.016660	0.332820	-0.231450
O	-0.057550	-1.092330	0.174380
N	-0.484050	0.252460	-1.688350
C	-0.810450	-1.126080	-2.067450

S	-0.863740	1.509780	-2.678680
C	-0.589959	-1.880030	-0.757010
O	-0.857839	-3.054340	-0.578080
H	-0.194850	-1.168730	-4.127460
H	-1.874480	-1.219710	-2.330180
C	0.017411	-1.750730	-3.226660
C	1.496361	-1.820429	-2.982510
C	2.248581	-2.941209	-2.465220
C	3.606971	-2.527168	-2.377100
N	3.669500	-1.228168	-2.840930
C	2.400640	-0.805399	-3.183950
H	2.226190	0.194191	-3.557090
C	4.257432	-4.655488	-1.522530
C	1.916022	-4.251379	-2.074340
H	5.643761	-3.019138	-1.815830
H	4.472550	-0.626378	-2.742650
C	2.919672	-5.093629	-1.612900
H	5.018812	-5.334548	-1.147660
H	0.884702	-4.590019	-2.110650
C	4.618541	-3.367678	-1.899740
H	2.671152	-6.107099	-1.308060
C	-2.646950	1.380429	-2.934030
O	-0.254320	1.328610	-4.006620
O	-0.598621	2.744280	-1.925970
C	-5.420650	1.302318	-3.346200
C	-3.509940	1.355529	-1.834220
C	-3.152770	1.363849	-4.232790
C	-4.534060	1.323329	-4.429260
C	-4.885420	1.313769	-2.047880
H	-3.102820	1.354109	-0.827880
H	-2.465950	1.379889	-5.072010
H	-4.927150	1.307239	-5.442980
H	-5.558620	1.294438	-1.193360
C	-6.915150	1.291808	-3.564190
H	-7.427490	0.686028	-2.808800
H	-7.173780	0.894948	-4.550940
H	-7.329511	2.306788	-3.500840
H	-0.388419	-2.756980	-3.385520
C	1.485180	0.956491	0.045700
C	2.599620	0.116821	0.204100
C	1.708739	2.341221	0.120490
C	3.878970	0.637842	0.423150
H	2.468980	-0.958959	0.155340
C	2.983999	2.864721	0.351420
H	0.878639	3.023811	-0.019170
C	4.078839	2.016222	0.503810
H	5.066619	2.420342	0.694290
C	3.188388	4.355191	0.384050
C	5.070400	-0.272658	0.518380
F	3.409508	4.862782	-0.850280
F	4.256568	4.700542	1.143450
F	2.113518	5.002251	0.887280
F	5.706460	-0.395548	-0.687860
F	4.745711	-1.518548	0.910230
F	6.002590	0.196672	1.379120
O	-1.031540	1.122980	0.590170
C	-0.908830	1.334010	1.865890
C	-1.274741	2.679930	2.372930
C	-1.898621	3.611560	1.528930
C	-0.935371	3.051470	3.684010
C	-2.192892	4.888840	2.002570
H	-2.104331	3.342410	0.498560
C	-1.229711	4.328550	4.151450
H	-0.427351	2.340500	4.332390
C	-1.866482	5.248030	3.312260
H	-2.664112	5.610659	1.341710
H	-0.955392	4.611770	5.163630
H	-2.093082	6.246750	3.675060
H	-0.081580	0.838160	2.379940
C	-2.389800	0.246459	2.895150
H	-2.118270	0.624340	3.871910
H	-3.242010	0.716149	2.415760
C	-2.174410	-1.105750	2.638290
C	-1.254079	-1.927140	3.446250
C	-0.469209	-2.924090	2.834460
C	-1.130719	-1.707460	4.833000
C	0.426341	-3.668470	3.599130
H	-0.531529	-3.077330	1.762400
C	-0.246599	-2.468800	5.590640
H	-1.755220	-0.966300	5.322790
C	0.538211	-3.447310	4.973510
H	1.044642	-4.418639	3.115000
H	-0.171639	-2.302990	6.661540
H	1.235241	-4.035309	5.564280
O	-2.727179	-1.629631	1.557860
Si	-3.858509	-2.909381	1.266350
C	-3.133608	-4.602611	1.616330
H	-2.831078	-4.732141	2.660240
H	-3.888408	-5.367371	1.389930
H	-2.264808	-4.783961	0.976290
C	-5.311419	-2.540252	2.408680
H	-6.108989	-3.278882	2.261430
H	-5.019099	-2.581482	3.464740
H	-5.736819	-1.548882	2.215420
C	-4.299109	-2.712081	-0.541070
H	-4.589519	-1.684361	-0.784880
H	-5.134809	-3.371992	-0.805110
H	-3.441649	-2.985811	-1.164000

TS 185

B3LYP/6-31G(d) = -3573.93191
B3LYP-D3(BJ)/def2-TZVPP/IEFPCM(propanonitrile) = -3575.500599
B3LYP-D3(BJ)/def2-TZVPP/IEFPCM(propanonitrile)//B3LYP-D3(BJ)/6-31G(d) Free Energy (Quasiharmonic) = -3574.784228

Frequencies (Top 3 out of 300)

1. -288.7426 cm^{-1}
2. 6.3013 cm^{-1}
3. 9.9586 cm^{-1}

B3LYP/6-31G(d) Molecular Geometry in Cartesian Coordinates

B	0.025364	-0.302546	-0.447982
O	0.524567	0.434903	-1.639904
N	-1.012865	-1.274614	-1.102681
C	-1.052025	-1.066798	-2.559960
S	-1.331998	-2.794335	-0.503596
C	0.027046	-0.013080	-2.799424
O	0.386301	0.403169	-3.881485
H	-3.157804	-1.345720	-2.966067
H	-0.757470	-1.982014	-3.087725
C	-2.402134	-0.579527	-3.164752
C	-2.896275	0.762320	-2.687605
C	-4.064856	1.026411	-1.880282
C	-4.123948	2.431195	-1.665708
N	-3.062748	2.992433	-2.345454
C	-2.325455	1.988165	-2.944240
H	-1.447316	2.227913	-3.527145
C	-6.106416	2.209364	-0.362674
C	-5.078597	0.221026	-1.332728
H	-5.135099	4.107896	-0.728655
H	-2.751812	3.945687	-2.238001
C	-6.085364	0.816840	-0.584136
H	-6.901554	2.644404	0.237004
H	-5.075471	-0.853309	-1.487815
C	-5.127826	3.035393	-0.901445
H	-6.869486	0.199162	-0.153903
C	-3.117557	-2.955116	-0.331497
O	-0.797436	-2.840784	0.865921

O	-0.922570	-3.802186	-1.496843
C	-5.869892	-3.356153	0.018755
C	-3.752130	-2.436390	0.800144
C	-3.836802	-3.680246	-1.282015
C	-5.207441	-3.870778	-1.103480
C	-5.119320	-2.642186	0.965112
H	-3.186653	-1.896122	1.551595
H	-3.319207	-4.113558	-2.131229
H	-5.766320	-4.442896	-1.840297
H	-5.611352	-2.244151	1.849140
C	-7.356700	-3.551032	0.200631
H	-7.916491	-2.678523	-0.161731
H	-7.717852	-4.423305	-0.353643
H	-7.616450	-3.686346	1.256033
H	-2.249395	-0.566156	-4.251457
C	-0.570931	0.689408	0.674407
C	-0.829550	2.039581	0.387871
C	-0.910625	0.228423	1.952612
C	-1.424929	2.884285	1.325157
H	-0.580096	2.428864	-0.592049
C	-1.521068	1.068333	2.891018
H	-0.723313	-0.810175	2.208793
C	-1.783205	2.401173	2.586643
H	-2.262778	3.048428	3.312310
C	-1.923215	0.483956	4.215463
C	-1.705770	4.325663	1.007769
F	-2.454009	1.402620	5.052124
F	-0.871876	-0.080767	4.855749
F	-2.848699	-0.497382	4.062789
F	-1.023958	5.164856	1.823970
F	-1.365868	4.659323	-0.264854
F	-3.016557	4.632403	1.155406
O	1.198194	-1.050922	0.165328
C	2.397451	-1.132630	-0.351834
C	3.244342	-2.233373	0.183621
C	4.472824	-2.542721	-0.421920
C	2.781391	-3.019512	1.249861
C	5.236393	-3.611729	0.042775
H	4.823637	-1.958306	-1.268417
C	3.550006	-4.086965	1.711679
H	1.803530	-2.813537	1.670467
C	4.779017	-4.382176	1.116083
H	6.180398	-3.851357	-0.438964
H	3.179579	-4.700635	2.528009
H	5.370972	-5.220321	1.474121
H	2.487502	-0.955615	-1.426864
C	3.218017	0.588672	0.160306
H	3.169686	0.498117	1.240322
H	2.420715	1.180883	-0.271461
C	4.491378	0.727786	-0.419279
C	4.696906	1.033093	-1.839612
C	6.005844	1.106083	-2.362210
C	3.603924	1.231677	-2.710265
C	6.210183	1.370063	-3.711196
H	6.850225	0.950401	-1.700873
C	3.816548	1.481258	-4.062429
H	2.581873	1.184446	-2.353563
C	5.117216	1.554558	-4.565525
H	7.222230	1.426781	-4.101797
H	2.954906	1.603312	-4.711056
H	5.282044	1.751782	-5.621293
O	5.595200	0.453402	0.255640
Si	6.056972	0.594650	1.927471
C	5.226252	-0.707002	2.990178
H	5.677509	-0.688599	3.990947
H	4.151194	-0.543122	3.114576
H	5.364989	-1.712412	2.579845
C	5.624302	2.343916	2.460732
H	6.116057	3.089476	1.825795
H	4.545722	2.532716	2.429072
H	5.956661	2.514764	3.492116
C	7.907424	0.312170	1.828505
H	8.363858	0.380536	2.823306
H	8.133303	-0.682227	1.427403
H	8.394213	1.054595	1.186422

TS 186

B3LYP/6-31G(d) = -3573.922421
B3LYP-D3(BJ)/def2-TZVPP/IEFPCM(propanonitrile) = -3575.499917
B3LYP-D3(BJ)/def2-TZVPP/IEFPCM(propanonitrile)//B3LYP-D3(BJ)/6-31G(d) Free Energy (Quasiharmonic) = -3574.784186

Frequencies (Top 3 out of 300)

1. -224.7117 cm^{-1}
2. 7.5330 cm^{-1}
3. 11.9756 cm^{-1}

B3LYP/6-31G(d) Molecular Geometry in Cartesian Coordinates

B	-0.502831	0.350206	0.627866
O	-0.813926	0.566097	2.058056
N	-0.534557	1.824059	0.106809
C	-1.181107	2.697360	1.110244
S	-0.238272	2.391320	-1.410032
C	-1.157228	1.818834	2.363161
O	-1.417595	2.211434	3.477597
H	-2.549356	3.924528	-0.022584
H	-0.549745	3.573008	1.309502
C	-2.615702	3.230547	0.820914
C	-3.693247	2.217251	0.554160
C	-4.538373	1.540674	1.514916
C	-5.424109	0.706288	0.775782
N	-5.138811	0.889952	-0.562612
C	-4.094810	1.781565	-0.685669
H	-3.703129	2.044010	-1.657053
C	-6.467523	-0.056355	2.779390
C	-4.648210	1.563588	2.917424
H	-7.051284	-0.730754	0.807823
H	-5.487267	0.325913	-1.322620
C	-5.607484	0.768467	3.532458
H	-7.203827	-0.671052	3.290588
H	-3.973817	2.171853	3.512235
C	-6.388011	-0.097785	1.392239
H	-5.692521	0.775451	4.615931
C	0.589750	3.971075	-1.145695
O	0.724607	1.458155	-2.029420
O	-1.459493	2.707351	-2.169754
C	1.876452	6.440888	-0.795609
C	-0.068107	5.150625	-1.494025
C	1.892061	4.011116	-0.641098
C	2.523807	5.239037	-0.468589
C	0.578199	6.374055	-1.315402
H	-1.066749	5.101139	-1.913366
H	2.413919	3.090433	-0.403717
H	3.539062	5.268004	-0.079935
H	0.063662	7.291375	-1.590280
C	2.558788	7.769479	-0.574835
H	2.426307	8.111394	0.460153
H	3.636349	7.702976	-0.758959
H	2.148314	8.544372	-1.229659
H	-2.873043	3.832027	1.701956
C	-1.454448	-0.779673	-0.016921
C	-1.759912	-1.903104	0.771693
C	-1.998889	-0.739072	-1.303664
C	-2.561350	-2.940752	0.295576
C	-2.816174	-1.771755	-1.781812
H	-1.806141	0.108111	-1.953370
C	-3.101440	-2.880521	-0.990326
H	-3.737022	-3.676209	-1.360017

C	-3.405334	-1.642730	-3.154443
C	-2.800480	-4.160428	1.141738
F	-2.456920	-1.379594	-4.087950
F	-4.298894	-0.613564	-3.228801
F	-4.060761	-2.752781	-3.553187
F	-1.846785	-5.105686	0.934239
F	-3.988103	-4.746720	0.863295
F	-2.785214	-3.879652	2.461278
O	0.901808	-0.317656	0.641468
C	1.989577	0.239876	1.056558
C	2.796630	-0.432620	2.090719
C	2.583013	-1.784148	2.403637
C	3.742236	0.305937	2.819496
C	3.310317	-2.383993	3.428412
H	1.833609	-2.341383	1.851842
C	4.464272	-0.296108	3.847644
H	3.891635	1.359900	2.594774
C	4.251231	-1.644315	4.151122
H	3.134383	-3.427356	3.674102
H	5.179091	0.286541	4.421980
H	4.806365	-2.112566	4.959338
H	2.024643	1.333982	1.056742
C	3.321971	0.254733	-0.656814
H	2.562897	0.707410	-1.282653
H	4.089985	0.912181	-0.264077
C	3.648596	-1.084066	-0.818133
C	2.789569	-2.075316	-1.472920
C	1.628567	-1.694028	-2.177590
C	3.126147	-3.444395	-1.403247
C	0.836479	-2.657558	-2.797026
H	1.339915	-0.653045	-2.252229
C	2.327803	-4.399060	-2.020130
H	4.008912	-3.744869	-0.851430
C	1.181803	-4.007736	-2.720647
H	-0.057569	-2.348727	-3.329402
H	2.593977	-5.450233	-1.954017
H	0.558896	-4.755987	-3.203087
O	4.743631	-1.586829	-0.237729
Si	6.415689	-1.203742	-0.256498
C	6.759322	0.541167	0.361837
H	7.842637	0.677063	0.475492
H	6.407820	1.313146	-0.331621
H	6.299679	0.717164	1.340245
C	7.001030	-1.409600	-2.031114
H	6.814840	-2.424457	-2.399556
H	6.492099	-0.710717	-2.705290
H	8.078218	-1.217106	-2.108631
H	-1.378769	-1.956221	1.786554
C	7.137118	-2.482131	0.908439
H	6.690093	-2.393062	1.904917
H	6.953095	-3.500250	0.547909
H	8.221324	-2.354153	1.011557

TS 187

B3LYP/6-31G(d) = -3573.924649
B3LYP-D3(BJ)/def2-TZVPP/IEFPCM(propanonitrile) = -3575.500191
B3LYP-D3(BJ)/def2-TZVPP/IEFPCM(propanonitrile)//B3LYP-D3(BJ)/6-31G(d) Free Energy (Quasiharmonic) = -3574.784175

Frequencies (Top 3 out of 300)

1. -263.6074 cm^{-1}
2. 8.6688 cm^{-1}
3. 11.4000 cm^{-1}

B3LYP/6-31G(d) Molecular Geometry in Cartesian Coordinates

B	0.634961	0.494711	-0.258220
O	0.202848	0.008901	-1.593733
N	1.310219	1.835535	-0.638252
C	1.382534	1.993547	-2.097515
S	1.567812	3.145944	0.331711
C	0.569192	0.805140	-2.608849
O	0.287440	0.577821	-3.765532
H	3.318457	2.890589	-2.377087
H	0.867029	2.908061	-2.426480
C	2.802606	1.993971	-2.729162
C	3.627261	0.770421	-2.442009
C	4.653545	0.620844	-1.435577
C	5.143647	-0.709738	-1.531550
N	4.461838	-1.326171	-2.562319
C	3.543854	-0.438989	-3.091787
H	2.901728	-0.733531	-3.911076
C	6.673042	-0.333156	0.255820
C	5.207343	1.479027	-0.467553
H	6.489255	-2.231943	-0.767328
H	4.486535	-2.316534	-2.748678
C	6.209940	0.994885	0.362813
H	7.452228	-0.683528	0.927838
H	4.837723	2.493459	-0.355373
C	6.146246	-1.204002	-0.689966
H	6.639240	1.647230	1.118571
C	0.185444	4.271722	0.053439
O	2.755349	3.866171	-0.151332
O	1.477519	2.677781	1.719246
C	-1.974847	6.007382	-0.387468
C	0.289938	5.278106	-0.909367
C	-0.978631	4.136101	0.812157
C	-2.046686	5.001564	0.587744
C	-0.790184	6.134155	-1.124509
H	1.218676	5.406677	-1.454766
H	-1.031504	3.374749	1.581320
H	-2.948461	4.898874	1.187022
H	-0.704333	6.922189	-1.869015
C	-3.148588	6.923224	-0.643197
H	-3.830435	6.494102	-1.389874
H	-2.822367	7.896563	-1.023880
H	-3.730948	7.092887	0.268639
H	2.647587	2.106789	-3.809483
C	1.499255	-0.624241	0.522756
C	1.886425	-0.474875	1.865185
C	1.866028	-1.814202	-0.121874
C	2.605277	-1.472639	2.527238
H	1.644775	0.444151	2.388692
C	2.591936	-2.812648	0.536037
H	1.584686	-1.964686	-1.158586
C	2.964400	-2.649349	1.868484
H	3.523915	-3.420243	2.385026
C	3.005220	-4.047476	-0.211642
C	2.931492	-1.311034	3.986983
F	3.206847	-5.102278	0.607412
F	4.171053	-3.874923	-0.901591
F	2.082389	-4.418774	-1.129870
F	3.186992	-0.029183	4.315430
F	4.004874	-2.048636	4.354497
F	1.898135	-1.715453	4.772707
O	-0.635660	0.730479	0.592183
C	-1.843710	0.490328	0.178547
C	-2.943060	0.987440	1.037200
C	-2.745239	1.194227	2.411438
C	-4.172642	1.328665	0.456020
C	-3.765270	1.737049	3.189054
H	-1.784342	0.945619	2.850788
C	-5.187884	1.884677	1.235160
H	-4.321457	1.185851	-0.611887
C	-4.986893	2.087215	2.603111
H	-3.605020	1.899330	4.251103
H	-6.129117	2.171288	0.773940
H	-5.775846	2.523137	3.209864
H	-2.021121	0.517918	-0.897627

C	-1.984623	-1.580289	0.275827
H	-1.914329	-1.664106	1.354995
H	-1.063170	-1.735569	-0.269938
C	-3.168916	-2.046917	-0.301710
C	-3.330683	-2.241712	-1.752667
C	-2.397504	-1.682391	-2.650867
C	-4.394469	-3.000165	-2.279140
C	-2.546400	-1.845454	-4.024839
H	-1.544468	-1.119309	-2.292976
C	-4.530398	-3.175261	-3.652585
H	-5.094280	-3.489006	-1.613691
C	-3.614149	-2.588935	-4.531202
H	-1.816501	-1.384616	-4.683564
H	-5.349084	-3.775604	-4.039161
H	-3.728301	-2.721962	-5.603441
O	-4.177710	-2.279807	0.517728
Si	-5.836678	-2.467160	0.901416
C	-6.226537	-4.305906	0.806957
H	-6.242825	-4.707525	-0.211599
H	-5.498693	-4.887196	1.384365
H	-7.215504	-4.489964	1.245767
C	-6.916211	-1.439730	-0.244621
H	-7.969945	-1.577938	0.029779
H	-6.686477	-0.373958	-0.143936
H	-6.807552	-1.712840	-1.298565
C	-5.957588	-1.877079	2.675279
H	-6.986617	-1.970080	3.044334
H	-5.313578	-2.473525	3.331215
H	-5.658252	-0.828309	2.771374

TS 188

B3LYP/6-31G(d) = -3573.931151
B3LYP-D3(BJ)/def2-TZVPP/IEFPCM(propanonitrile) = -3575.499267
B3LYP-D3(BJ)/def2-TZVPP/IEFPCM(propanonitrile)//B3LYP-D3(BJ)/6-31G(d) Free Energy (Quasiharmonic) = -3574.783854

Frequencies (Top 3 out of 300)

1. -259.6478 cm^{-1}
2. 7.4412 cm^{-1}
3. 10.1419 cm^{-1}

B3LYP/6-31G(d) Molecular Geometry in Cartesian Coordinates

B	0.298209	-0.695673	0.294156
O	-0.575910	-0.482951	1.478284
N	-0.337706	0.305789	-0.714300
C	-1.208640	1.259248	0.002374
S	0.325859	0.686218	-2.193538
C	-1.402631	0.553117	1.350925
O	-2.200778	0.886309	2.204495
H	-3.367331	1.313741	0.022966
H	-0.668794	2.193598	0.220982
C	-2.573060	1.612523	-0.668802
C	-2.753873	3.064524	-1.009296
C	-3.035747	4.138155	-0.083949
C	-3.162263	5.331906	-0.847700
N	-2.962927	4.989870	-2.170445
C	-2.718189	3.630492	-2.260141
H	-2.517826	3.161252	-3.212669
C	-3.595618	6.601715	1.123079
C	-3.195651	4.202246	1.312474
H	-3.536057	7.472659	-0.856794
H	-2.991583	5.626831	-2.950834
C	-3.473261	5.430963	1.899705
H	-3.812788	7.547560	1.612440
H	-3.092406	3.304117	1.915782
C	-3.441933	6.569442	-0.258728
H	-3.596263	5.494536	2.977669
C	1.544094	1.986594	-1.960933
O	-0.736378	1.245882	-3.042497
O	1.037539	-0.527575	-2.622083
C	3.453390	3.999884	-1.562647
C	2.902851	1.669739	-2.007216
C	1.125229	3.303753	-1.748440
C	2.083010	4.295731	-1.545258
C	3.844512	2.676754	-1.809056
H	3.218387	0.653486	-2.205143
H	0.069373	3.554385	-1.763304
H	1.758218	5.319599	-1.376385
H	4.900908	2.422198	-1.845260
C	4.481693	5.074707	-1.303361
H	5.405527	4.888969	-1.861281
H	4.746694	5.113060	-0.238410
H	4.107355	6.065179	-1.582007
H	-2.672781	1.004817	-1.569410
C	1.853722	-0.516743	0.703767
C	2.217744	0.257267	1.816339
C	2.893537	-1.105369	-0.031228
C	3.556243	0.449264	2.171423
H	1.445042	0.705320	2.433417
C	4.233621	-0.903401	0.309212
H	2.653399	-1.713636	-0.896673
C	4.576130	-0.125000	1.414041
H	5.614714	0.021017	1.686021
C	5.305808	-1.459697	-0.583256
C	3.899918	1.328237	3.342859
F	5.471621	-0.687784	-1.692799
F	5.010052	-2.702885	-1.023781
F	6.509340	-1.522534	0.027651
F	3.949423	2.636274	2.991893
F	5.107356	1.021490	3.871293
F	2.987637	1.227394	4.335464
O	0.171156	-2.146659	-0.171348
C	-0.649355	-2.591247	-1.072116
C	-0.163129	-3.670959	-1.960839
C	-0.798432	-3.886826	-3.192850
C	0.944648	-4.454438	-1.605502
C	-0.335274	-4.875907	-4.056691
H	-1.640391	-3.261500	-3.480684
C	1.402802	-5.445119	-2.470378
H	1.446969	-4.265609	-0.662385
C	0.763265	-5.660062	-3.694537
H	-0.822095	-5.028239	-5.015746
H	2.265596	-6.044833	-2.194689
H	1.126526	-6.430120	-4.369455
H	-1.354153	-1.881077	-1.506725
C	-2.126864	-3.774124	-0.083858
H	-1.420163	-4.376791	0.474630
H	-2.517998	-4.239005	-0.977723
C	-2.959311	-2.908945	0.625398
C	-4.128091	-2.231301	0.025874
C	-4.655126	-2.618717	-1.221786
C	-4.748947	-1.178754	0.726100
C	-5.767433	-1.972298	-1.750974
H	-4.212165	-3.437886	-1.778175
C	-5.860552	-0.534172	0.190146
H	-4.333266	-0.855568	1.673061
C	-6.373899	-0.928390	-1.047062
H	-6.164915	-2.285878	-2.711901
H	-6.321247	0.283190	0.737023
H	-7.241863	-0.424470	-1.462941
O	-2.713712	-2.554436	1.866528
Si	-1.920056	-3.206496	3.267862
C	-2.829435	-4.817119	3.630099
H	-2.440944	-5.273019	4.549286
H	-3.902376	-4.645806	3.772378
H	-2.708634	-5.550361	2.824224
C	-2.246841	-1.877155	4.540813
H	-1.634546	-2.025189	5.438212

H	-2.013504	-0.894317	4.116363
H	-3.298941	-1.869916	4.847891
C	-0.088856	-3.515523	3.000670
H	0.116862	-4.276910	2.241014
H	0.428665	-2.597250	2.712913
H	0.346178	-3.870043	3.944610

TS 189

B3LYP/6-31G(d) = -3573.925848
B3LYP-D3(BJ)/def2-TZVPP/IEFPCM(propanonitrile) = -3575.500614
B3LYP-D3(BJ)/def2-TZVPP/IEFPCM(propanonitrile)//B3LYP-D3(BJ)/6-31G(d) Free Energy (Quasiharmonic) = -3574.783782

Frequencies (Top 3 out of 300)

1. -279.9372 cm^{-1}
2. 12.0308 cm^{-1}
3. 14.8806 cm^{-1}

B3LYP/6-31G(d) Molecular Geometry in Cartesian Coordinates

B	0.606492	0.940372	-1.153449
O	1.098517	1.316914	-2.493218
N	0.676391	2.316248	-0.415132
C	1.367040	3.313201	-1.256820
S	-0.076727	2.813399	0.941757
C	1.509694	2.581094	-2.594940
O	1.965396	3.076897	-3.600964
H	2.607325	4.313037	0.198815
H	0.730160	4.193654	-1.418235
C	2.751034	3.814111	-0.763094
C	3.825709	2.766778	-0.656146
C	4.285436	2.087100	0.532753
C	5.317284	1.191676	0.133709
N	5.473007	1.333012	-1.229941
C	4.577796	2.274525	-1.697077
H	4.534207	2.523013	-2.748444
C	5.609299	0.440243	2.377450
C	3.932775	2.146915	1.893692
H	6.767324	-0.317884	0.714372
H	6.114221	0.808263	-1.803594
C	4.595384	1.325755	2.798050
H	6.101862	-0.195313	3.108266
H	3.139717	2.809691	2.226774
C	5.985616	0.363522	1.041386
H	4.321489	1.356502	3.848954
C	-1.412607	3.907652	0.408596
O	0.793810	3.645629	1.784203
O	-0.685927	1.608058	1.550704
C	-3.552366	5.582041	-0.297681
C	-2.089890	3.698160	-0.794678
C	-1.788005	4.950819	1.258739
C	-2.854804	5.773185	0.903624
C	-3.151613	4.534543	-1.137715
H	-1.783602	2.904681	-1.468469
H	-1.238905	5.116987	2.179289
H	-3.145671	6.583373	1.568120
H	-3.671759	4.372459	-2.078609
C	-4.681212	6.504203	-0.692281
H	-4.299972	7.388708	-1.219506
H	-5.231822	6.861791	0.184166
H	-5.390508	6.006999	-1.361748
H	3.051159	4.582769	-1.485509
C	1.466077	-0.298135	-0.565638
C	1.718308	-0.529663	0.796315
C	1.959334	-1.242291	-1.479078
C	2.402231	-1.669404	1.223568
H	1.368348	0.178867	1.538103
C	2.660082	-2.376763	-1.055885
H	1.807833	-1.078851	-2.541719
C	2.880487	-2.601493	0.300922
H	3.422045	-3.479398	0.635032
C	3.100169	-3.394950	-2.068214
C	2.549755	-1.978093	2.686924
F	3.530894	-2.825630	-3.214068
F	2.075456	-4.225258	-2.413962
F	4.094765	-4.185723	-1.608172
F	3.776586	-2.460175	2.987211
F	1.660125	-2.943974	3.062326
F	2.321056	-0.913822	3.477699
O	-0.806366	0.415577	-1.476535
C	-1.910220	0.167911	-0.832975
C	-3.047985	-0.266702	-1.683077
C	-4.363340	-0.132147	-1.212644
C	-2.826768	-0.752633	-2.981069
C	-5.439437	-0.492454	-2.021822
H	-4.542156	0.264710	-0.217226
C	-3.905384	-1.113678	-3.786172
H	-1.809319	-0.819747	-3.351380
C	-5.212919	-0.987870	-3.308113
H	-6.454494	-0.378694	-1.651561
H	-3.726891	-1.482596	-4.792474
H	-6.052430	-1.264890	-3.940002
H	-2.154416	0.799689	0.020566
C	-1.445627	-1.388910	0.436786
H	-0.805427	-1.940299	-0.241878
H	-0.925586	-0.721894	1.111664
C	-2.595826	-2.029819	0.894987
C	-3.478017	-1.489016	1.940578
C	-3.154668	-0.307627	2.640568
C	-4.676622	-2.160992	2.255086
C	-4.010839	0.177270	3.625478
H	-2.243271	0.239589	2.426985
C	-5.525468	-1.668885	3.240394
H	-4.930712	-3.065367	1.715236
C	-5.194846	-0.498807	3.929711
H	-3.747183	1.085937	4.158433
H	-6.445611	-2.197517	3.472842
H	-5.856765	-0.116713	4.702119
O	-3.029952	-3.150847	0.333664
Si	-2.178679	-4.532629	-0.293565
C	-3.574542	-5.773441	-0.472752
H	-4.069256	-5.962889	0.486336
H	-4.332344	-5.416025	-1.179250
H	-3.195836	-6.731724	-0.848267
C	-0.927910	-5.043777	1.011840
H	-1.427505	-5.342801	1.940506
H	-0.348578	-5.904695	0.655411
H	-0.214359	-4.249314	1.257469
C	-1.399558	-4.158573	-1.959925
H	-1.249490	-5.100439	-2.503375
H	-2.053082	-3.525614	-2.569945
H	-0.419821	-3.676384	-1.885974

TS 190

B3LYP/6-31G(d) = -3573.927882
B3LYP-D3(BJ)/def2-TZVPP/IEFPCM(propanonitrile) = -3575.50049
B3LYP-D3(BJ)/def2-TZVPP/IEFPCM(propanonitrile)//B3LYP-D3(BJ)/6-31G(d) Free Energy (Quasiharmonic) = -3574.783774

Frequencies (Top 3 out of 300)

1. -275.2811 cm^{-1}
2. 10.6773 cm^{-1}
3. 12.4570 cm^{-1}

B3LYP/6-31G(d) Molecular Geometry in Cartesian Coordinates

B	0.846620	0.588251	-1.018359
O	1.542340	0.751691	-2.305369
N	1.210580	1.930441	-0.306319
C	2.166129	2.703911	-1.123129
S	0.582460	2.579811	1.050121
C	2.236010	1.885201	-2.416859
O	2.849290	2.224451	-3.403109
H	3.470149	3.660912	0.300321
H	1.747889	3.686221	-1.382759
C	3.578729	2.947022	-0.520719
C	4.308910	1.728092	-0.034949
C	5.165710	0.837082	-0.786719
C	5.622790	-0.162528	0.117321
N	5.087370	0.126702	1.357151
C	4.287280	1.247802	1.251731
H	3.761040	1.639362	2.111091
C	6.885751	-1.220817	-1.605959
C	5.601160	0.787832	-2.123849
H	6.811391	-1.952627	0.429381
H	5.080980	-0.503038	2.145031
C	6.453260	-0.236337	-2.517899
H	7.548991	-2.011787	-1.946489
H	5.252800	1.522982	-2.842759
C	6.477391	-1.197017	-0.277479
H	6.788230	-0.285347	-3.550639
C	-0.334731	4.039860	0.518391
O	-0.386550	1.588010	1.579341
O	1.615519	3.073331	1.974201
C	-1.842872	6.294590	-0.198079
C	-1.054831	4.040360	-0.678909
C	-0.354891	5.155320	1.358101
C	-1.110662	6.269070	0.996931
C	-1.801321	5.164510	-1.027009
H	-1.018521	3.182660	-1.342919
H	0.224469	5.146711	2.274961
H	-1.125892	7.136570	1.652371
H	-2.355341	5.164790	-1.962509
C	-2.626462	7.521070	-0.600559
H	-2.984872	8.073020	0.274471
H	-2.004012	8.209410	-1.187399
H	-3.492072	7.259800	-1.218009
H	4.148769	3.448492	-1.312399
C	1.278681	-0.805039	-0.319759
C	1.654751	-0.951619	1.021271
C	1.246141	-1.969019	-1.104579
C	1.960381	-2.205999	1.561071
C	1.522941	-3.227499	-0.564929
H	0.990571	-1.890909	-2.157669
C	1.883961	-3.356489	0.776121
H	2.097382	-4.330969	1.198871
C	1.323742	-4.447249	-1.418179
C	2.395891	-2.308889	2.995121
F	1.930102	-5.541529	-0.913069
F	1.771992	-4.272479	-2.677029
F	-0.004688	-4.755449	-1.525319
F	2.155361	-3.530339	3.518961
F	1.774561	-1.401309	3.781211
F	3.735261	-2.089018	3.145501
O	-0.623300	0.464450	-1.463419
C	-1.777440	0.496180	-0.852089
C	-2.961410	0.500840	-1.749099
C	-2.870510	0.029830	-3.066709
C	-4.175110	1.033419	-1.288459
C	-3.981630	0.083569	-3.905569
H	-1.921340	-0.353950	-3.425669
C	-5.283320	1.089279	-2.131889
H	-4.243700	1.415839	-0.273019
C	-5.189200	0.611669	-3.441239
H	-3.903600	-0.279391	-4.926619
H	-6.216670	1.513029	-1.771329
H	-6.052070	0.657809	-4.100089
H	-1.852060	1.108540	0.045401
C	-1.828929	-1.207830	0.257401
H	-1.323059	-1.838700	-0.460159
H	-1.191189	-0.805990	1.035681
C	-3.140299	-1.546720	0.591081
C	-3.967589	-2.450381	-0.224269
C	-3.385119	-3.367100	-1.122889
C	-5.369729	-2.423361	-0.094199
C	-4.188218	-4.220631	-1.873369
H	-2.307469	-3.452580	-1.210939
C	-6.166509	-3.271481	-0.855109
H	-5.820919	-1.723571	0.599311
C	-5.578428	-4.171841	-1.747539
H	-3.725448	-4.931281	-2.551629
H	-7.247239	-3.232882	-0.752489
H	-6.201268	-4.839211	-2.336829
O	-3.773919	-0.991881	1.610971
Si	-3.378680	-0.365560	3.188351
C	-3.489580	1.501610	3.082751
H	-3.460350	1.930800	4.092501
H	-2.644050	1.916630	2.524921
H	-4.426070	1.827019	2.614921
C	-1.695899	-0.976100	3.748721
H	-1.552179	-2.043950	3.548361
H	-0.871520	-0.420480	3.292501
H	-1.619529	-0.838300	4.835131
H	1.699860	-0.080709	1.663651
C	-4.759059	-1.085861	4.239641
H	-5.744849	-0.786461	3.865951
H	-4.722799	-2.181041	4.253161
H	-4.676519	-0.737001	5.276341

TS 191

B3LYP/6-31G(d) = -3573.915623
B3LYP-D3(BJ)/def2-TZVPP/IEFPCM(propanonitrile) = -3575.499329
B3LYP-D3(BJ)/def2-TZVPP/IEFPCM(propanonitrile)//B3LYP-D3(BJ)/6-31G(d) Free Energy (Quasiharmonic) = -3574.783444

Frequencies (Top 3 out of 300)

1. -297.0510 cm^{-1}
2. 6.7476 cm^{-1}
3. 13.4453 cm^{-1}

B3LYP/6-31G(d) Molecular Geometry in Cartesian Coordinates

B	0.308250	-0.456268	0.591930
O	0.233547	-1.072469	1.932373
N	0.120953	-1.700561	-0.323195
C	0.180479	-2.934971	0.471134
S	-0.411548	-1.736746	-1.870636
C	0.142961	-2.405018	1.909030
O	0.059384	-3.094609	2.902890
H	1.384196	-4.200363	-0.789187
H	-0.719340	-3.547115	0.319104
C	1.419799	-3.843645	0.244123
C	2.747691	-3.197302	0.528769
C	3.681680	-2.643990	-0.425104
C	4.780412	-2.128385	0.316077
N	4.532582	-2.381171	1.650789
C	3.303753	-3.001581	1.771167
H	2.913950	-3.267729	2.744380
C	5.849601	-1.384250	-1.680007
C	3.694221	-2.521078	-1.826545
H	6.687825	-1.094526	0.296081
H	5.037339	-1.953262	2.411860
C	4.773680	-1.893388	-2.436630
H	6.672985	-0.888684	-2.187355
H	2.857743	-2.879820	-2.418187

C	5.868346	-1.495972	-0.294482
H	4.785025	-1.779862	-3.517234
C	-2.083897	-2.422001	-1.814349
O	0.364945	-2.688649	-2.679918
O	-0.511829	-0.331198	-2.313000
C	-4.703298	-3.427080	-1.844978
C	-2.454239	-3.362665	-2.777325
C	-3.005419	-1.982368	-0.862401
C	-4.303660	-2.489982	-0.881878
C	-3.758022	-3.855293	-2.787099
H	-1.719548	-3.706253	-3.497162
H	-2.701952	-1.272774	-0.099348
H	-5.012023	-2.159876	-0.125985
H	-4.042606	-4.592179	-3.534704
C	-6.118548	-3.954346	-1.876628
H	-6.608552	-3.847375	-0.903472
H	-6.145656	-5.013217	-2.155501
H	-6.727148	-3.411541	-2.612328
O	-0.986792	0.407523	0.536674
C	-1.403600	1.457909	-0.101966
H	-2.135289	1.260008	-0.889091
H	1.272535	-4.715196	0.894015
C	-0.561391	2.656558	-0.342500
C	0.262137	3.215052	0.646922
C	-0.590071	3.231311	-1.622408
C	1.050210	4.323559	0.356104
H	0.292708	2.770883	1.636738
C	0.216153	4.330464	-1.916472
H	-1.202721	2.779730	-2.397834
C	1.033652	4.878968	-0.927622
H	1.690584	4.746864	1.124349
H	0.215509	4.748605	-2.918727
H	1.664455	5.732893	-1.157053
C	1.680041	0.371669	0.406060
C	2.169130	0.793722	-0.841302
C	2.479296	0.631428	1.529282
C	3.386970	1.466244	-0.954269
H	1.594683	0.590006	-1.738604
C	3.708203	1.289178	1.417461
H	2.145018	0.296674	2.505625
C	4.167155	1.719164	0.173713
H	5.121930	2.223679	0.080776
C	4.586286	1.451194	2.622760
C	3.849709	1.964219	-2.295373
F	5.363958	2.554857	2.548879
F	3.878642	1.531905	3.771332
F	5.199891	2.013167	-2.381593
F	5.444235	0.397463	2.777925
F	3.406804	3.229499	-2.537432
F	3.404783	1.199774	-3.311371
C	-2.789391	2.315203	1.207809
H	-2.105031	2.384296	2.043460
H	-2.952735	3.246433	0.678526
C	-3.871783	1.440479	1.327839
O	-4.941961	1.544762	0.544702
Si	-5.625755	2.779540	-0.453963
C	-5.843828	4.332277	0.583924
H	-4.892453	4.763626	0.912438
H	-6.446837	4.129733	1.476368
H	-6.367112	5.099096	-0.000802
C	-4.537550	3.061391	-1.958632
H	-3.583986	3.541887	-1.715559
H	-4.321292	2.118112	-2.473478
H	-5.058728	3.711665	-2.672730
C	-7.271190	2.024259	-0.936867
H	-7.893732	1.822485	-0.058024
H	-7.831638	2.699849	-1.594216
H	-7.128883	1.079802	-1.473804
C	-3.904282	0.292940	2.239396
C	-5.105916	-0.427938	2.413519
C	-2.752267	-0.127215	2.935472
C	-5.148298	-1.529933	3.258443
H	-5.996590	-0.109607	1.884264
C	-2.792613	-1.254863	3.752162
H	-1.808641	0.384196	2.804941
C	-3.990016	-1.951814	3.921524
H	-6.081243	-2.069699	3.394574
H	-1.879576	-1.602086	4.224804
H	-4.020737	-2.827040	4.564389

TS 192

B3LYP/6-31G(d) = -3573.927717
B3LYP-D3(BJ)/def2-TZVPP/IEFPCM(propanonitrile) = -3575.499073
B3LYP-D3(BJ)/def2-TZVPP/IEFPCM(propanonitrile)//B3LYP-D3(BJ)/6-31G(d) Free Energy (Quasiharmonic) = -3574.783417

Frequencies (Top 3 out of 300)

1. -274.8326 cm^{-1}
2. 5.8780 cm^{-1}
3. 10.0643 cm^{-1}

B3LYP/6-31G(d) Molecular Geometry in Cartesian Coordinates

B	0.717740	-0.525170	0.819500
O	0.121940	-0.250420	2.138270
N	-0.310360	0.171570	-0.132910
C	-1.229250	1.015200	0.663790
S	-0.033170	0.562670	-1.726180
C	-0.926080	0.572510	2.101390
O	-1.555260	0.909230	3.080980
H	-3.144890	0.027250	0.895580
H	-0.947799	2.075400	0.582220
C	-2.744300	0.882380	0.341530
C	-3.539629	2.127361	0.630010
C	-4.117109	3.010321	-0.356670
C	-4.781289	4.055111	0.346370
N	-4.605729	3.809021	1.692290
C	-3.856009	2.658841	1.856760
H	-3.574189	2.306571	2.838230
C	-5.458259	5.072741	-1.702070
C	-4.134909	3.024031	-1.764330
H	-5.959199	5.877371	0.242660
H	-4.935649	4.396431	2.441890
C	-4.802269	4.052091	-2.420550
H	-5.972179	5.862621	-2.243370
H	-3.620889	2.250161	-2.329080
C	-5.456189	5.088491	-0.311400
H	-4.818329	4.073131	-3.507050
C	0.737591	2.182430	-1.816890
O	-1.338670	0.689770	-2.403110
O	0.930100	-0.432900	-2.224400
C	1.940311	4.710209	-1.946280
C	-0.056589	3.330940	-1.737760
C	2.120851	2.280119	-1.987450
C	2.707691	3.541629	-2.052630
C	0.552721	4.583080	-1.796790
H	-1.136139	3.253200	-1.662830
H	2.725330	1.387009	-2.088530
H	3.783081	3.616539	-2.193930
H	-0.065439	5.475410	-1.737390
C	2.595471	6.070149	-1.979490
H	2.935541	6.365539	-0.978010
H	3.473151	6.076419	-2.634380
H	1.902072	6.841579	-2.329610
H	-2.830800	0.644540	-0.719040
C	2.265080	-0.039311	0.796900
C	3.214740	-0.534661	-0.108500
C	2.705660	0.925499	1.716520
C	4.528960	-0.058991	-0.122680

C	4.023500	1.391379	1.719490
H	2.010150	1.319059	2.450520
C	4.944970	0.907519	0.791900
H	5.964010	1.275699	0.781190
C	4.466851	2.377449	2.765070
C	5.463020	-0.525461	-1.202300
F	4.861201	1.760149	3.905040
F	3.477111	3.231669	3.108450
F	5.515711	3.123999	2.345280
F	6.761570	-0.311951	-0.891680
F	5.228500	0.130629	-2.370580
F	5.322010	-1.842961	-1.466530
O	0.750930	-2.056010	0.648360
C	0.315510	-2.736140	-0.370280
C	1.183969	-3.837350	-0.862030
C	2.160569	-4.411841	-0.034540
C	1.056279	-4.276870	-2.187870
C	2.982619	-5.423541	-0.524240
H	2.278919	-4.043521	0.979430
C	1.884159	-5.285610	-2.676280
H	0.330029	-3.802720	-2.843140
C	2.843779	-5.865551	-1.843250
H	3.740839	-5.861391	0.118900
H	1.789229	-5.610370	-3.708550
H	3.490749	-6.650901	-2.224150
H	-0.246310	-2.195840	-1.127950
C	-1.297961	-3.946390	0.187730
H	-0.737781	-4.596400	0.847420
H	-1.483021	-4.358880	-0.797430
C	-2.314110	-3.176450	0.762440
C	-2.352420	-2.831170	2.186810
C	-1.243610	-3.056470	3.026890
C	-3.523070	-2.271359	2.739280
C	-1.307260	-2.727430	4.376220
H	-0.318180	-3.444380	2.619680
C	-3.583490	-1.958099	4.091060
H	-4.378050	-2.092439	2.097900
C	-2.475000	-2.182000	4.912100
H	-0.437630	-2.881800	5.007440
H	-4.490970	-1.528749	4.505120
H	-2.518930	-1.923270	5.966180
O	-3.297740	-2.656799	0.044640
Si	-4.021420	-2.963909	-1.502470
C	-2.826100	-2.577810	-2.894300
H	-3.369850	-2.602589	-3.847820
H	-2.387440	-1.577960	-2.794440
H	-2.012770	-3.307780	-2.970200
C	-5.478390	-1.788019	-1.492250
H	-6.053970	-1.877129	-2.421570
H	-6.157690	-1.996299	-0.658050
H	-5.146800	-0.747599	-1.405320
H	2.925410	-1.285351	-0.834970
C	-4.567531	-4.765759	-1.503210
H	-3.725171	-5.461439	-1.421760
H	-5.255291	-4.972619	-0.675420
H	-5.094821	-4.996719	-2.437160

TS 193

B3LYP/6-31G(d) = -3573.930798
B3LYP-D3(BJ)/def2-TZVPP/IEFPCM(propanonitrile) = -3575.500273
B3LYP-D3(BJ)/def2-TZVPP/IEFPCM(propanonitrile)//B3LYP-D3(BJ)/6-31G(d) Free Energy (Quasiharmonic) = -3574.783412

Frequencies (Top 3 out of 300)

1. -271.7654 cm^{-1}
2. 6.7717 cm^{-1}
3. 16.9785 cm^{-1}

B3LYP/6-31G(d) Molecular Geometry in Cartesian Coordinates

B	-0.290909	0.710280	-0.146890
O	-0.902459	-0.382660	-0.953370
N	0.644501	1.359120	-1.213170
C	0.375011	0.809290	-2.550730
S	1.274031	2.891130	-1.071110
C	-0.654579	-0.279880	-2.261970
O	-1.200939	-0.970530	-3.099610
H	2.249481	1.088630	-3.570050
H	-0.102819	1.562320	-3.190630
C	1.584551	0.246170	-3.353330
C	2.365171	-0.875380	-2.724300
C	2.108891	-2.299750	-2.804100
C	3.126831	-2.951060	-2.052200
N	3.973721	-1.972610	-1.573010
C	3.501821	-0.735360	-1.964470
H	4.031671	0.161590	-1.680790
C	2.203641	-5.093530	-2.541360
C	1.139531	-3.092510	-3.447460
H	3.961651	-4.812210	-1.311050
H	4.675951	-2.111250	-0.862570
C	1.196931	-4.474100	-3.310530
H	2.217401	-6.176370	-2.448490
H	0.345761	-2.627550	-4.022290
C	3.181761	-4.340840	-1.903350
H	0.450261	-5.091360	-3.803470
C	3.064631	2.716600	-0.971370
O	0.854281	3.417500	0.234490
O	0.995941	3.632330	-2.312050
C	5.851541	2.461480	-0.795880
C	3.642231	2.230530	0.204280
C	3.861111	3.092910	-2.051850
C	5.247781	2.961030	-1.957150
C	5.025931	2.096970	0.280230
H	3.014331	1.958590	1.045290
H	3.395761	3.492000	-2.946840
H	5.869311	3.254220	-2.799910
H	5.471971	1.700590	1.189180
C	7.353861	2.347820	-0.688420
H	7.821421	2.257240	-1.674110
H	7.648901	1.479540	-0.089480
H	7.781591	3.235390	-0.203550
H	1.172901	-0.078210	-4.316970
C	0.439311	0.184680	1.193100
C	1.576181	-0.632310	1.106030
C	-0.018179	0.502510	2.479430
C	2.235821	-1.095330	2.250600
H	1.963671	-0.896250	0.126910
C	0.617901	0.019620	3.626180
H	-0.882309	1.148240	2.588240
C	1.757481	-0.777660	3.521540
H	2.262021	-1.139030	4.409190
C	0.017091	0.296980	4.974990
C	3.482041	-1.918430	2.089330
F	-0.507959	1.538900	5.054840
F	0.914921	0.169410	5.976500
F	-0.999009	-0.566810	5.251820
F	4.466501	-1.218980	1.450270
F	3.270881	-3.026290	1.339850
F	3.998491	-2.324860	3.267310
O	-1.406449	1.650000	0.293340
C	-2.569729	1.807940	-0.273250
C	-3.140239	3.182510	-0.246260
C	-4.286089	3.479620	-1.001570
C	-2.506979	4.197060	0.485160
C	-4.802649	4.771480	-1.013700
H	-4.768439	2.695880	-1.582460
C	-3.029389	5.490060	0.468460
H	-1.588819	3.973320	1.016270
C	-4.176939	5.779040	-0.272180

H	-5.685859	4.996640	-1.605040
H	-2.529179	6.276680	1.026150
H	-4.577679	6.789040	-0.283760
H	-2.765219	1.244570	-1.188440
C	-3.920729	0.839230	0.907600
H	-3.459719	1.085810	1.858740
H	-4.815559	1.391700	0.650340
C	-3.871779	-0.494030	0.485530
C	-4.749349	-0.963770	-0.614780
C	-4.205119	-1.564980	-1.763500
C	-6.138109	-0.756050	-0.531270
C	-5.047959	-1.955060	-2.804270
H	-3.130899	-1.667270	-1.877590
C	-6.973359	-1.175670	-1.563930
H	-6.560349	-0.292290	0.355730
C	-6.428669	-1.774360	-2.703160
H	-4.616129	-2.391310	-3.700190
H	-8.046929	-1.031860	-1.481630
H	-7.079689	-2.089910	-3.513820
O	-2.962399	-1.281500	1.000100
Si	-2.717849	-2.998850	1.218360
C	-4.373079	-3.683320	1.797050
H	-4.711389	-3.191400	2.716250
H	-5.155599	-3.568380	1.039380
H	-4.276309	-4.754830	2.012260
C	-1.420489	-3.051070	2.564870
H	-1.666169	-2.397280	3.407720
H	-1.316609	-4.074560	2.946900
H	-0.444969	-2.739900	2.176900
C	-2.106589	-3.829750	-0.346720
H	-2.910369	-4.027960	-1.062050
H	-1.339779	-3.231280	-0.849250
H	-1.651259	-4.792100	-0.078920

TS 194

B3LYP/6-31G(d) = -3573.928982
B3LYP-D3(BJ)/def2-TZVPP/IEFPCM(propanonitrile) = -3575.499621
B3LYP-D3(BJ)/def2-TZVPP/IEFPCM(propanonitrile)//B3LYP-D3(BJ)/6-31G(d) Free Energy (Quasiharmonic) = -3574.783393

Frequencies (Top 3 out of 300)

1. -277.7965 cm^{-1}
2. 9.2029 cm^{-1}
3. 10.7373 cm^{-1}

B3LYP/6-31G(d) Molecular Geometry in Cartesian Coordinates

B	0.543290	0.345630	0.217448
O	1.102397	1.320183	-0.760040
N	0.193226	-0.849324	-0.732828
C	0.485454	-0.502386	-2.133759
S	0.096473	-2.443456	-0.293411
C	1.112159	0.885332	-2.016384
O	1.543348	1.531091	-2.953683
H	-1.145362	-1.468129	-3.173760
H	1.240110	-1.184626	-2.544703
C	-0.703813	-0.467391	-3.137727
C	-1.785754	0.554820	-2.888367
C	-3.190756	0.294541	-2.669950
C	-3.834278	1.551438	-2.497029
N	-2.869859	2.525803	-2.643964
C	-1.647039	1.924487	-2.872076
H	-0.765246	2.528404	-3.029892
C	-5.952106	0.497494	-2.210423
C	-3.976724	-0.870545	-2.631607
H	-5.669057	2.640021	-2.101971
H	-2.993837	3.500690	-2.416580
C	-5.342696	-0.758972	-2.406175
H	-7.021932	0.551476	-2.026811
H	-3.525631	-1.847725	-2.774230
C	-5.206744	1.668876	-2.254002
H	-5.955661	-1.656207	-2.378714
C	-1.548267	-3.086469	-0.631458
O	1.025328	-3.216333	-1.140278
O	0.246805	-2.477141	1.171195
C	-4.049917	-4.287061	-1.017723
C	-1.724068	-3.992715	-1.676885
C	-2.604150	-2.773786	0.230072
C	-3.843049	-3.372186	0.026872
C	-2.975425	-4.582308	-1.865470
H	-0.883645	-4.248918	-2.312786
H	-2.461581	-2.089630	1.058847
H	-4.663345	-3.126933	0.696710
H	-3.112142	-5.293307	-2.676696
C	-5.395534	-4.947271	-1.202537
H	-6.205734	-4.208951	-1.194774
H	-5.447021	-5.497187	-2.147228
H	-5.601297	-5.658109	-0.391920
H	-0.242500	-0.299663	-4.119684
C	-0.700149	0.964661	1.038735
C	-1.318743	2.154669	0.625067
C	-1.248773	0.310929	2.149313
C	-2.449155	2.655533	1.271645
C	-2.390676	0.801846	2.793500
H	-0.791276	-0.607209	2.506633
C	-3.000084	1.976705	2.361302
H	-3.884211	2.355591	2.861512
C	-2.980170	-0.000166	3.918810
C	-3.129451	3.903138	0.784302
F	-3.512709	-1.166274	3.465626
F	-3.967469	0.656690	4.566233
F	-2.049802	-0.339254	4.840033
F	-3.354117	4.784181	1.785496
F	-4.339541	3.638687	0.229772
F	-2.408535	4.555296	-0.165037
O	1.627147	0.073776	1.260919
C	2.561985	-0.830221	1.244940
C	2.920029	-1.440385	2.550227
C	3.539734	-2.698074	2.577533
C	2.618120	-0.789508	3.754095
C	3.860391	-3.294378	3.794493
H	3.742129	-3.217908	1.644043
C	2.944162	-1.387197	4.969458
H	2.112082	0.169908	3.724902
C	3.568454	-2.637236	4.992817
H	4.325905	-4.275849	3.809794
H	2.702674	-0.882262	5.900616
H	3.816647	-3.103413	5.942395
H	2.632178	-1.485352	0.375509
C	4.418557	0.090812	0.913176
H	5.005074	-0.766909	1.210389
H	4.334284	0.867104	1.665074
C	4.473285	0.476520	-0.429037
C	4.815525	-0.457681	-1.520531
C	5.476996	-1.676726	-1.272996
C	4.476995	-0.124845	-2.847072
C	5.787523	-2.537196	-2.320656
H	5.772252	-1.949754	-0.265464
C	4.782505	-0.995857	-3.889259
H	3.945414	0.798334	-3.044282
C	5.439180	-2.200889	-3.631394
H	6.303531	-3.470484	-2.115226
H	4.502080	-0.733160	-4.905085
H	5.678922	-2.877037	-4.447333
O	4.118345	1.673600	-0.835087
Si	4.030152	3.277436	-0.168409
C	2.903485	3.388269	1.328466
H	3.293821	2.859007	2.204215
H	2.796754	4.444875	1.607847

H	1.908809	2.994457	1.105915
C	3.390278	4.256314	-1.626822
H	3.059036	5.256465	-1.323536
H	4.162589	4.374390	-2.395388
H	2.545711	3.725602	-2.079736
H	-0.915097	2.691201	-0.224477
C	5.806929	3.707218	0.288054
H	5.863873	4.737448	0.661036
H	6.201454	3.052048	1.073343
H	6.470733	3.631137	-0.580448

TS 195

B3LYP/6-31G(d) = -3573.915654
B3LYP-D3(BJ)/def2-TZVPP/IEFPCM(propanonitrile) = -3575.499403
B3LYP-D3(BJ)/def2-TZVPP/IEFPCM(propanonitrile)//B3LYP-D3(BJ)/6-31G(d) Free Energy (Quasiharmonic) = -3574.783328

Frequencies (Top 3 out of 300)

1. -297.1062 cm^{-1}
2. 6.6457 cm^{-1}
3. 13.7568 cm^{-1}

B3LYP/6-31G(d) Molecular Geometry in Cartesian Coordinates

B	0.308812	-0.456590	0.592041
O	0.234296	-1.077092	1.930418
N	0.116936	-1.697329	-0.327048
C	0.174933	-2.934568	0.463051
S	-0.417042	-1.727006	-1.874047
C	0.140538	-2.409338	1.902777
O	0.056791	-3.102055	2.894425
H	1.374281	-4.198253	-0.803137
H	-0.726345	-3.544315	0.310153
C	1.412061	-3.845031	0.231294
C	2.741689	-3.202399	0.516249
C	3.675512	-2.647835	-0.437067
C	4.776356	-2.137043	0.304312
N	4.529848	-2.393787	1.638516
C	3.299890	-3.012016	1.758523
H	2.910876	-3.280600	2.731382
C	5.844362	-1.388469	-1.690743
C	3.686368	-2.520219	-1.838104
H	6.685948	-1.107199	0.285137
H	5.036510	-1.969420	2.400309
C	4.766303	-1.892751	-2.447574
H	6.668113	-0.892971	-2.197566
H	2.848319	-2.875209	-2.429790
C	5.864788	-1.504892	-0.305629
H	4.776393	-1.775608	-3.527803
C	-2.091210	-2.407632	-1.817628
O	-0.513170	-0.319718	-2.311892
O	0.355465	-2.678798	-2.687256
C	-4.715979	-3.398256	-1.852408
C	-3.009136	-1.970776	-0.860498
C	-2.469391	-3.334327	-2.790638
C	-3.776899	-3.817792	-2.804078
C	-4.310450	-2.469381	-0.883366
H	-2.702084	-1.264754	-0.095580
H	-1.740238	-3.669513	-3.520026
H	-4.069964	-4.538121	-3.564381
H	-5.017932	-2.136374	-0.127828
C	-6.118217	-3.959935	-1.849628
H	-6.830547	-3.260647	-1.399256
H	-6.168432	-4.892769	-1.272340
H	-6.460875	-4.188914	-2.864275
O	-0.983608	0.411625	0.542050
C	-1.398871	1.462839	-0.096279
H	-2.128321	1.266630	-0.885853
H	1.263887	-4.718418	0.878512
C	-0.555946	2.661907	-0.331057
C	0.267863	3.214654	0.661379
C	-0.583472	3.242787	-1.608237
C	1.057341	4.323609	0.376173
H	0.297510	2.765527	1.648990
C	0.224110	4.342392	-1.896700
H	-1.196312	2.795601	-2.386058
C	1.041869	4.885185	-0.904889
H	1.697954	4.742486	1.146643
H	0.224390	4.765344	-2.896933
H	1.673734	5.739483	-1.129984
C	1.682203	0.368942	0.407213
C	2.483298	0.623201	1.530393
C	2.170458	0.794788	-0.839170
C	3.713231	1.279156	1.419451
H	2.149589	0.285520	2.505920
C	3.389338	1.465613	-0.951176
H	1.594555	0.595414	-1.736514
C	4.171391	1.712961	0.176731
H	5.126904	2.216194	0.084470
C	3.851013	1.967822	-2.291063
C	4.593039	1.435184	2.624280
F	3.404987	1.206732	-3.309115
F	3.408119	3.233941	-2.528653
F	5.448909	0.379091	2.774852
F	5.201122	2.016729	-2.378366
F	3.886881	1.513449	3.773937
F	5.372979	2.537430	2.553299
C	-2.789675	2.317457	1.211046
H	-2.954486	3.247387	0.679916
H	-2.106986	2.389559	2.047815
C	-3.869635	1.439716	1.330299
C	-3.900568	0.293839	2.244081
C	-2.747706	-0.124177	2.940006
C	-5.101417	-0.427837	2.420166
C	-2.786456	-1.250776	3.758222
H	-1.804664	0.387873	2.807796
C	-5.142263	-1.528598	3.266764
H	-5.992796	-0.111023	1.891172
C	-3.983101	-1.948546	3.929502
H	-1.872757	-1.596711	4.230503
H	-6.074618	-2.069021	3.404259
H	-4.012519	-2.822944	4.573555
O	-4.938557	1.539055	0.544815
Si	-5.623817	2.768898	-0.458924
C	-5.854004	4.321643	0.576339
H	-4.906115	4.756170	0.910745
H	-6.462065	4.117665	1.465016
H	-6.375921	5.086459	-0.012208
C	-4.530593	3.054662	-1.959202
H	-4.305726	2.111780	-2.471068
H	-5.053006	3.699844	-2.677012
H	-3.581453	3.542223	-1.712940
C	-7.263116	2.004573	-0.948387
H	-7.824554	2.676989	-1.608137
H	-7.113314	1.060939	-1.484748
H	-7.888164	1.799495	-0.072088

TS 196

B3LYP/6-31G(d) = -3573.915654
B3LYP-D3(BJ)/def2-TZVPP/IEFPCM(propanonitrile) = -3575.499404
B3LYP-D3(BJ)/def2-TZVPP/IEFPCM(propanonitrile)//B3LYP-D3(BJ)/6-31G(d) Free Energy (Quasiharmonic) = -3574.783318

Frequencies (Top 3 out of 300)

1. -297.1290 cm^{-1}
2. 6.6240 cm^{-1}

3. 13.7640 cm^{-1}

B3LYP/6-31G(d) Molecular Geometry in Cartesian Coordinates

B	0.308791	-0.456592	0.592049
O	0.234269	-1.077179	1.930389
N	0.116957	-1.697284	-0.327116
C	0.174976	-2.934566	0.462910
S	-0.417007	-1.726885	-1.874124
C	0.140537	-2.409423	1.902667
O	0.056773	-3.102203	2.894270
H	1.374377	-4.198139	-0.803342
H	-0.726283	-3.544330	0.309961
C	1.412127	-3.844989	0.231116
C	2.741739	-3.202355	0.516144
C	3.675575	-2.647710	-0.437113
C	4.776393	-2.136949	0.304326
N	4.529858	-2.393789	1.638506
C	3.299909	-3.012049	1.758443
H	2.910876	-3.280705	2.731276
C	5.844427	-1.388210	-1.690651
C	3.686460	-2.519996	-1.838141
H	6.685959	-1.107057	0.285269
H	5.036505	-1.969478	2.400341
C	4.766396	-1.892462	-2.447542
H	6.668175	-0.892653	-2.197420
H	2.848431	-2.874959	-2.429871
C	5.864825	-1.504732	-0.305545
H	4.776506	-1.775239	-3.527762
C	-2.091064	-2.407765	-1.817730
O	-0.513340	-0.319562	-2.311815
O	0.355638	-2.678471	-2.687441
C	-4.715681	-3.398788	-1.852449
C	-3.009148	-1.970842	-0.860688
C	-2.469043	-3.334642	-2.790551
C	-3.776546	-3.818318	-2.803961
C	-4.310326	-2.469611	-0.883519
H	-2.702211	-1.264629	-0.095899
H	-1.739832	-3.669837	-3.519878
H	-4.069484	-4.538804	-3.564149
H	-5.017906	-2.136527	-0.128093
C	-6.117850	-3.960630	-1.849158
H	-6.832837	-3.254735	-1.413435
H	-6.454704	-4.205003	-2.862098
H	-6.171285	-4.884478	-1.257858
H	1.263956	-4.718426	0.878267
C	1.682180	0.368959	0.407281
C	2.170458	0.794849	-0.839079
C	2.483258	0.623171	1.530483
C	3.389340	1.465675	-0.951039
C	3.713195	1.279127	1.419587
H	2.149534	0.285449	2.505992
C	4.171376	1.712977	0.176890
H	5.126893	2.216207	0.084663
C	4.592999	1.435092	2.624426
C	3.851059	1.967930	-2.290894
F	5.448938	0.379037	2.774886
F	3.886847	1.513202	3.774096
F	5.372872	2.537392	2.553545
F	5.201174	2.016646	-2.378220
F	3.408351	3.234137	-2.528363
F	3.404900	1.206995	-3.309004
O	-0.983628	0.411614	0.542129
C	-1.398928	1.462780	-0.096267
C	-0.556038	2.661866	-0.331083
C	0.267733	3.214680	0.661348
C	-0.583563	3.242697	-1.608284
C	1.057170	4.323658	0.376116
H	0.297384	2.765588	1.648974
C	0.223981	4.342324	-1.896773
H	-1.196365	2.795452	-2.386100
C	1.041700	4.885187	-0.904967
H	1.697752	4.742588	1.146583
H	0.224265	4.765237	-2.897022
H	1.673535	5.739501	-1.130081
H	-2.128359	1.266509	-0.885843
C	-2.789692	2.317372	1.211035
H	-2.107031	2.389371	2.047834
H	-2.954449	3.247362	0.679993
C	-3.869700	1.439672	1.330175
C	-3.900710	0.293701	2.243835
C	-2.747904	-0.124389	2.939812
C	-5.101577	-0.427983	2.419757
C	-2.786728	-1.251063	3.757922
H	-1.804848	0.387666	2.807728
C	-5.142499	-1.528822	3.266250
H	-5.992912	-0.111116	1.890720
C	-3.983393	-1.948839	3.929043
H	-1.873070	-1.597044	4.230250
H	-6.074870	-2.069249	3.403622
H	-4.012870	-2.823291	4.573019
O	-4.938587	1.539143	0.544659
Si	-5.623822	2.769223	-0.458812
C	-5.853875	4.321783	0.576757
H	-6.376024	5.086637	-0.011536
H	-6.461683	4.117610	1.465562
H	-4.905942	4.756354	0.910978
C	-7.263194	2.005100	-0.948346
H	-7.888220	1.799893	-0.072062
H	-7.824615	2.677683	-1.607941
H	-7.113482	1.061564	-1.484903
H	1.594570	0.595512	-1.736440
C	-4.530653	3.055201	-1.959087
H	-3.581385	3.542477	-1.712752
H	-4.306028	2.112421	-2.471249
H	-5.052977	3.700711	-2.676666

TS 197

B3LYP/6-31G(d) = -3573.929587
B3LYP-D3(BJ)/def2-TZVPP/IEFPCM(propanonitrile) = -3575.499287
B3LYP-D3(BJ)/def2-TZVPP/IEFPCM(propanonitrile)//B3LYP-D3(BJ)/6-31G(d) Free Energy (Quasiharmonic) = -3574.783294

Frequencies (Top 3 out of 300)

1. -283.6779 cm^{-1}
2. 6.8349 cm^{-1}
3. 9.4816 cm^{-1}

B3LYP/6-31G(d) Molecular Geometry in Cartesian Coordinates

B	0.045357	-0.331575	-0.534797
O	0.505148	0.610201	-1.589434
N	-1.048985	-1.147757	-1.297398
C	-1.113734	-0.709039	-2.700928
S	-1.394452	-2.737944	-0.941730
C	-0.024273	0.356977	-2.792035
O	0.313480	0.935457	-3.804547
H	-3.228870	-0.900668	-3.103575
H	-0.842912	-1.530707	-3.374916
C	-2.468506	-0.118452	-3.190899
C	-2.941067	1.132196	-2.493807
C	-4.109803	1.275334	-1.656446
C	-4.152986	2.626362	-1.214119
N	-3.079422	3.276776	-1.786425
C	-2.353364	2.375815	-2.543662
H	-1.473029	2.697799	-3.081954
C	-6.151124	2.222111	0.019106
C	-5.137311	0.404430	-1.253263
H	-5.154513	4.141787	-0.025650

H	-2.765341	4.201076	-1.533842
C	-6.144004	0.883818	-0.425410
H	-6.947635	2.564734	0.674328
H	-5.145874	-0.630683	-1.580126
C	-5.157361	3.111415	-0.369771
H	-6.940179	0.214979	-0.109204
C	-3.173137	-2.872071	-0.693124
O	-1.079177	-3.574941	-2.111937
O	-0.793268	-3.031012	0.368075
C	-5.914218	-3.248778	-0.244565
C	-3.727701	-2.508014	0.536788
C	-3.967360	-3.426955	-1.696573
C	-5.332644	-3.604871	-1.468222
C	-5.090569	-2.696541	0.748436
H	-3.102993	-2.098573	1.323488
H	-3.512586	-3.736025	-2.631708
H	-5.951459	-4.040037	-2.249440
H	-5.522368	-2.412406	1.704933
C	-7.383708	-3.482962	0.015318
H	-7.541403	-4.415158	0.573958
H	-7.819651	-2.673113	0.610419
H	-7.949119	-3.561356	-0.918704
H	-2.336409	0.069193	-4.264122
C	-0.469111	0.438316	0.786431
C	-0.809623	1.800243	0.742588
C	-0.654692	-0.229600	2.003824
C	-1.317833	2.464481	1.858933
C	-1.183636	0.427635	3.121109
H	-0.410914	-1.285437	2.071882
C	-1.514228	1.778682	3.060658
H	-1.918553	2.286610	3.929019
C	-1.433495	-0.371677	4.368834
C	-1.697392	3.916970	1.791703
F	-0.332212	-1.058446	4.755106
F	-2.411450	-1.292060	4.176926
F	-1.812395	0.397542	5.413441
F	-1.324378	4.501198	0.622997
F	-1.123295	4.632974	2.787959
F	-3.033146	4.102404	1.909853
O	1.221666	-1.214860	-0.158625
C	2.441798	-1.092091	-0.616978
C	3.301894	-2.293269	-0.448434
C	2.888167	-3.350140	0.376962
C	4.500182	-2.405694	-1.169336
C	3.677288	-4.494134	0.487016
H	1.930186	-3.282689	0.881488
C	5.282115	-3.553165	-1.060070
H	4.813839	-1.597146	-1.825951
C	4.874292	-4.597332	-0.225887
H	3.346405	-5.315143	1.116905
H	6.204051	-3.635930	-1.628803
H	5.481672	-5.494698	-0.143257
H	2.563109	-0.560277	-1.564414
C	3.163109	0.370249	0.485304
H	2.993530	-0.058396	1.465612
H	2.405735	1.084426	0.183760
C	4.485437	0.686283	0.133459
C	5.667029	0.099054	0.780741
C	5.548453	-0.845350	1.820554
C	6.956414	0.492490	0.368344
C	6.683291	-1.376101	2.424180
H	4.573196	-1.182917	2.149828
C	8.087324	-0.036354	0.979681
H	7.056253	1.216673	-0.431313
C	7.954574	-0.972365	2.009098
H	6.574959	-2.107276	3.219504
H	9.074123	0.279808	0.654063
H	8.838859	-1.385578	2.486284
O	4.756584	1.466741	-0.894235
Si	4.074345	2.790487	-1.806006
C	2.612292	3.549609	-0.910055
H	2.816098	3.742262	0.149241
H	2.382603	4.515815	-1.377904
H	1.717554	2.925263	-0.991332
C	3.639374	2.116999	-3.496390
H	3.602141	2.945012	-4.216312
H	4.396567	1.410106	-3.854987
H	2.657226	1.630777	-3.516926
H	-0.691087	2.344827	-0.186420
C	5.528449	3.979154	-1.875353
H	5.265193	4.873668	-2.453076
H	5.830061	4.307962	-0.874278
H	6.398783	3.519251	-2.357382

TS 198

B3LYP/6-31G(d) = -3573.916159
B3LYP-D3(BJ)/def2-TZVPP/IEFPCM(propanonitrile) = -3575.499699
B3LYP-D3(BJ)/def2-TZVPP/IEFPCM(propanonitrile)//B3LYP-D3(BJ)/6-31G(d) Free Energy (Quasiharmonic) = -3574.783242

Frequencies (Top 3 out of 300)

1. -297.7628 cm^{-1}
2. 8.0180 cm^{-1}
3. 16.3972 cm^{-1}

B3LYP/6-31G(d) Molecular Geometry in Cartesian Coordinates

B	0.133432	0.040901	-0.546760
O	-0.053052	-0.246295	-1.986733
N	-0.142042	1.573232	-0.514041
C	-0.226392	2.111173	-1.878210
S	-0.591302	2.491387	0.763728
C	-0.281896	0.839322	-2.729478
O	-0.492435	0.810334	-3.923754
H	0.913246	3.929706	-1.698381
H	-1.169658	2.653335	-2.031096
C	0.932924	3.039978	-2.334173
C	2.301469	2.416617	-2.317848
C	3.334668	2.583615	-1.321452
C	4.440105	1.786505	-1.727601
N	4.099021	1.187225	-2.923923
C	2.809518	1.552806	-3.259599
H	2.345890	1.172770	-4.159947
C	5.681576	2.424552	0.204401
C	3.432491	3.315256	-0.123723
H	6.440882	1.064125	-1.300251
H	4.605801	0.420140	-3.338423
C	4.600423	3.227155	0.624232
H	6.576132	2.368533	0.818797
H	2.595802	3.910906	0.227640
C	5.616834	1.694917	-0.977051
H	4.679416	3.776382	1.558474
C	-2.302126	2.974794	0.443742
O	-0.575232	1.601050	1.942200
O	0.161979	3.753846	0.813593
C	-4.972874	3.747738	0.077512
C	-2.686498	4.288428	0.715069
C	-3.236374	2.041171	-0.011239
C	-4.560204	2.434590	-0.192385
C	-4.017320	4.663741	0.534692
H	-1.944520	5.001150	1.057766
H	-2.926612	1.025169	-0.237636
H	-5.286267	1.707711	-0.546648
H	-4.315589	5.687205	0.749470
C	-6.405983	4.167162	-0.149968
H	-6.572060	4.456133	-1.196335
H	-6.676112	5.026737	0.471843
H	-7.101632	3.351469	0.075210
O	-1.054035	-0.727496	0.094392

C	-1.376698	-1.132624	1.287185
H	-2.126880	-0.523351	1.797472
H	0.672358	3.361904	-3.350195
C	-0.445561	-1.831084	2.205900
C	-0.492426	-1.489589	3.566129
C	0.465126	-2.806319	1.769505
C	0.381296	-2.089972	4.471106
H	-1.180359	-0.717464	3.900264
C	1.323829	-3.415798	2.677629
H	0.506385	-3.072108	0.718358
C	1.288991	-3.053254	4.028187
H	0.361318	-1.798203	5.517033
H	2.032434	-4.163011	2.332669
H	1.973528	-3.518054	4.731823
C	1.592536	-0.430459	-0.041650
C	2.174099	0.004459	1.161316
C	2.367748	-1.264074	-0.861500
C	3.457239	-0.396993	1.535742
H	1.619881	0.668777	1.815598
C	3.659897	-1.655878	-0.497032
H	1.965667	-1.596168	-1.812795
C	4.211210	-1.230525	0.710001
H	5.214977	-1.524996	0.993805
C	4.498538	-2.456198	-1.449031
C	4.024593	0.028968	2.861585
F	5.234526	-1.657957	-2.280641
F	5.388879	-3.248237	-0.810771
F	3.745281	-0.879098	3.837283
F	3.754723	-3.249682	-2.251902
F	3.530010	1.209821	3.279731
F	5.373457	0.140876	2.824215
C	-2.672662	-2.722613	0.913237
H	-1.934049	-3.331794	0.409757
H	-2.909464	-3.027172	1.924804
C	-3.680166	-2.150427	0.138281
C	-4.891636	-1.536339	0.716833
C	-5.026599	-1.322860	2.102397
C	-5.957508	-1.174633	-0.128572
C	-6.190420	-0.765109	2.622433
H	-4.218462	-1.578873	2.779145
C	-7.125888	-0.630375	0.397340
H	-5.861026	-1.333635	-1.196227
C	-7.245444	-0.421632	1.773475
H	-6.274181	-0.597536	3.692074
H	-7.944903	-0.369630	-0.267023
H	-8.156204	0.005758	2.183327
O	-3.569412	-2.022127	-1.168572
Si	-2.937426	-2.969758	-2.492029
C	-1.176411	-3.549673	-2.217993
H	-1.076013	-4.301303	-1.427448
H	-0.818307	-4.010168	-3.148329
H	-0.524459	-2.698522	-2.001921
C	-4.128801	-4.424835	-2.605696
H	-4.125778	-5.024414	-1.687659
H	-5.157408	-4.094255	-2.788825
H	-3.840865	-5.087576	-3.431053
C	-3.069057	-1.808804	-3.949775
H	-2.935066	-2.370618	-4.883022
H	-4.051770	-1.325902	-3.994156
H	-2.298685	-1.029710	-3.923623

TS 199

B3LYP/6-31G(d) = -3573.932667
B3LYP-D3(BJ)/def2-TZVPP/IEFPCM(propanonitrile) = -3575.498578
B3LYP-D3(BJ)/def2-TZVPP/IEFPCM(propanonitrile)//B3LYP-D3(BJ)/6-31G(d) Free Energy (Quasiharmonic) = -3574.783106

Frequencies (Top 3 out of 300)

1. -284.6771 cm^{-1}
2. 4.3275 cm^{-1}
3. 7.8970 cm^{-1}

B3LYP/6-31G(d) Molecular Geometry in Cartesian Coordinates

B	0.040093	0.206283	0.137808
O	-0.022926	-0.713005	-1.035051
N	1.197985	-0.430413	0.940657
C	1.826372	-1.536681	0.202940
S	1.955198	0.305388	2.221309
C	0.894147	-1.688702	-1.003658
O	0.941043	-2.577401	-1.828511
H	1.384889	-3.647761	0.456173
H	2.810361	-1.242588	-0.192802
C	1.982142	-2.889332	0.976220
C	3.395424	-3.382311	1.100445
C	4.191743	-4.012092	0.072220
C	5.451694	-4.332060	0.651442
N	5.413808	-3.907919	1.964620
C	4.180027	-3.339239	2.226767
H	3.950343	-2.925593	3.198225
C	6.211239	-5.273292	-1.403338
C	3.961896	-4.335946	-1.277477
H	7.425507	-5.200983	0.386213
H	6.162218	-4.003077	2.632713
C	4.971599	-4.961889	-1.998952
H	6.980746	-5.763093	-1.994374
H	3.013602	-4.084872	-1.744779
C	6.468898	-4.962522	-0.072525
H	4.806894	-5.214661	-3.043178
C	3.324652	1.241294	1.528732
O	2.540663	-0.736545	3.077131
O	0.987161	1.262705	2.770749
C	5.379505	2.670921	0.267866
C	4.511090	0.588054	1.186104
C	3.167274	2.608271	1.283705
C	4.193599	3.310256	0.659488
C	5.526155	1.308193	0.554357
H	4.642904	-0.463859	1.418670
H	2.250326	3.105622	1.578693
H	4.065704	4.370392	0.456140
H	6.445489	0.798421	0.276558
C	6.452926	3.441293	-0.462652
H	7.339937	2.826084	-0.642118
H	6.080620	3.789729	-1.434100
H	6.764661	4.328822	0.101167
H	1.551450	-2.748827	1.969052
C	0.181431	1.741189	-0.343152
C	-0.490627	2.796307	0.287786
C	1.014843	2.068351	-1.424715
C	-0.339273	4.119612	-0.139430
C	1.181345	3.388223	-1.848392
H	1.545414	1.281257	-1.951651
C	0.500168	4.425449	-1.208907
H	0.614005	5.449556	-1.545257
C	2.152794	3.719075	-2.947369
C	-1.041846	5.220615	0.605332
F	1.734227	4.767776	-3.693715
F	2.354689	2.680558	-3.785388
F	3.370183	4.064802	-2.450877
F	-2.287951	4.852218	0.989645
F	-1.168589	6.343026	-0.139243
F	-0.376997	5.569122	1.731639
O	-1.261456	0.096748	0.925757
C	-2.133598	-0.864085	0.787384
C	-3.050069	-1.075307	1.937830
C	-3.893604	-2.197233	1.977066
C	-3.020701	-0.195095	3.030493
C	-4.706092	-2.427319	3.085515
H	-3.902277	-2.898744	1.147120

C	-3.836338	-0.428225	4.136235
H	-2.327513	0.638839	3.017448
C	-4.683470	-1.539361	4.165115
H	-5.347415	-3.303907	3.112568
H	-3.798947	0.250204	4.983829
H	-5.312107	-1.722603	5.032360
H	-1.799572	-1.771375	0.277519
C	-3.229345	-0.231721	-0.770632
H	-2.360166	-0.104915	-1.403549
H	-3.616816	0.687842	-0.345370
C	-4.157677	-1.214631	-1.144995
C	-3.791063	-2.428826	-1.883489
C	-2.466200	-2.650123	-2.316400
C	-4.768407	-3.410619	-2.152886
C	-2.130087	-3.822474	-2.986107
H	-1.683291	-1.923786	-2.133904
C	-4.427368	-4.573739	-2.832807
H	-5.787298	-3.249682	-1.820447
C	-3.107889	-4.784493	-3.248471
H	-1.098021	-3.972970	-3.285915
H	-5.188489	-5.321622	-3.036323
H	-2.844540	-5.698675	-3.773435
O	-5.414530	-1.174891	-0.729680
Si	-6.535569	0.114095	-0.406591
C	-6.122483	1.008539	1.189014
H	-6.946627	1.689874	1.437951
H	-6.003055	0.310874	2.024242
H	-5.211140	1.611463	1.123739
C	-8.153114	-0.821863	-0.260191
H	-8.982810	-0.133506	-0.059202
H	-8.387264	-1.366508	-1.181573
H	-8.116744	-1.546426	0.561056
H	-1.137751	2.582906	1.130986
C	-6.496475	1.254242	-1.899715
H	-6.738952	0.714532	-2.822065
H	-7.235478	2.055994	-1.779620
H	-5.518133	1.728392	-2.033910

TS 200

B3LYP/6-31G(d) = -3573.925715
B3LYP-D3(BJ)/def2-TZVPP/IEFPCM(propanonitrile) = -3575.499911
B3LYP-D3(BJ)/def2-TZVPP/IEFPCM(propanonitrile)//B3LYP-D3(BJ)/6-31G(d) Free Energy (Quasiharmonic) = -3574.783052

Frequencies (Top 3 out of 300)

1. -268.3316 cm^{-1}
2. 12.0729 cm^{-1}
3. 15.0677 cm^{-1}

B3LYP/6-31G(d) Molecular Geometry in Cartesian Coordinates

B	0.784545	0.652702	-1.287779
O	1.253890	0.885696	-2.665678
N	1.345719	1.914963	-0.558970
C	2.185911	2.706732	-1.479043
S	0.905011	2.563238	0.872189
C	1.991224	1.985667	-2.817479
O	2.471484	2.352637	-3.866570
H	3.787922	3.320302	-0.169496
H	1.789679	3.726108	-1.585282
C	3.695515	2.822538	-1.138340
C	4.473195	1.534542	-1.141041
C	4.888971	0.748835	-0.002336
C	5.613593	-0.368952	-0.503431
N	5.635288	-0.255537	-1.878252
C	4.951402	0.883421	-2.254113
H	4.846536	1.147419	-3.297248
C	5.980270	-1.191766	1.703871
C	4.727550	0.879339	1.389600
H	6.707799	-2.194653	-0.069645
H	6.055584	-0.915248	-2.513609
C	5.272310	-0.088769	2.224600
H	6.385985	-1.936364	2.383167
H	4.166716	1.713015	1.801258
C	6.162667	-1.345079	0.334535
H	5.142896	-0.002249	3.299927
C	-0.080715	4.028107	0.487245
O	2.061724	3.060647	1.630988
O	0.020110	1.571405	1.529953
C	-1.670560	6.293725	0.020771
C	-0.006462	5.119065	1.356549
C	-0.935455	4.060296	-0.617086
C	-1.721438	5.189749	-0.841178
C	-0.803337	6.237337	1.120914
H	0.677133	5.088626	2.198061
H	-0.977448	3.223697	-1.306661
H	-2.380986	5.213521	-1.705174
H	-0.744753	7.084528	1.800142
C	-2.500520	7.526175	-0.248683
H	-3.416725	7.281447	-0.795774
H	-1.942066	8.250838	-0.855873
H	-2.782271	8.031117	0.681210
H	4.105477	3.504822	-1.892783
C	1.233928	-0.808762	-0.768697
C	1.059837	-1.891791	-1.642452
C	1.708204	-1.093137	0.521623
C	1.294646	-3.209110	-1.234346
H	0.722003	-1.705079	-2.658110
C	1.945868	-2.406103	0.933048
H	1.878074	-0.286566	1.223691
C	1.731006	-3.475996	0.061121
H	1.899680	-4.496479	0.387158
C	2.372029	-2.717811	2.341779
C	0.958837	-4.332918	-2.171242
F	3.543626	-3.388777	2.386510
F	1.453669	-3.520648	2.954300
F	2.498377	-1.617500	3.108061
F	1.509972	-5.506880	-1.798036
F	1.352769	-4.080555	-3.437184
F	-0.391364	-4.538284	-2.227091
O	-0.749256	0.626240	-1.478037
C	-1.787002	0.639681	-0.687825
C	-3.099976	0.729506	-1.372398
C	-3.239685	0.357431	-2.717379
C	-4.206481	1.244303	-0.679570
C	-4.471428	0.491526	-3.354328
H	-2.373540	-0.014728	-3.254389
C	-5.435726	1.381091	-1.321607
H	-4.096930	1.548700	0.358338
C	-5.571009	1.002301	-2.659494
H	-4.572671	0.204382	-4.397260
H	-6.285328	1.790190	-0.781745
H	-6.528881	1.110958	-3.160949
H	-1.693421	1.172663	0.256791
C	-1.723627	-1.165329	0.305453
H	-0.951884	-0.834963	0.989360
H	-1.367621	-1.743549	-0.535607
C	-2.970489	-1.493659	0.831132
C	-3.959921	-2.307797	0.105271
C	-5.314829	-2.266365	0.486718
C	-3.578348	-3.152150	-0.956915
C	-6.262732	-3.029482	-0.186175
H	-5.611496	-1.622944	1.306618
C	-4.530721	-3.920664	-1.619665
H	-2.537390	-3.248267	-1.246066
C	-5.873949	-3.858121	-1.241977
H	-7.305925	-2.980504	0.112886
H	-4.220144	-4.576625	-2.427486
H	-6.613867	-4.459552	-1.762976

O	-3.399464	-1.010260	1.985967
Si	-2.719602	-0.473255	3.497719
C	-0.948898	-1.049059	3.712218
H	-0.782413	-2.085764	3.400962
H	-0.241666	-0.407566	3.177622
H	-0.696100	-0.990350	4.779163
C	-3.873454	-1.282933	4.739822
H	-3.817011	-2.375926	4.683614
H	-3.607223	-0.988262	5.762320
H	-4.915812	-0.990399	4.569702
C	-2.874915	1.395272	3.519099
H	-2.667275	1.772497	4.528616
H	-2.149112	1.858224	2.842383
H	-3.884123	1.724808	3.246078

TS 201

B3LYP/6-31G(d) = -3573.92444
B3LYP-D3(BJ)/def2-TZVPP/IEFPCM(propanonitrile) = -3575.499887
B3LYP-D3(BJ)/def2-TZVPP/IEFPCM(propanonitrile)//B3LYP-D3(BJ)/6-31G(d) Free Energy (Quasiharmonic) = -3574.783046

Frequencies (Top 3 out of 300)

1. -274.6958 cm^{-1}
2. 6.2375 cm^{-1}
3. 13.4497 cm^{-1}

B3LYP/6-31G(d) Molecular Geometry in Cartesian Coordinates

B	0.420002	0.084383	0.015385
O	-0.255413	1.019368	-0.935690
N	0.319877	-1.251432	-0.795930
C	0.039621	-0.955779	-2.208619
S	1.134900	-2.643022	-0.374138
C	-0.403835	0.509098	-2.158473
O	-0.835845	1.135218	-3.105726
H	-0.663088	-2.861444	-2.923259
H	0.964516	-0.985358	-2.808632
C	-1.004848	-1.825315	-2.955896
C	-2.436346	-1.740244	-2.495926
C	-3.172766	-2.682310	-1.680288
C	-4.522181	-2.227359	-1.624017
N	-4.590519	-1.065994	-2.366662
C	-3.342314	-0.781896	-2.885840
H	-3.185205	0.088523	-3.506600
C	-5.154652	-4.100737	-0.288037
C	-2.833845	-3.876105	-1.016266
H	-6.549480	-2.567745	-0.913075
H	-5.412476	-0.498122	-2.497314
C	-3.825529	-4.570390	-0.332483
H	-5.908108	-4.671650	0.248608
H	-1.809459	-4.235232	-1.027703
C	-5.520845	-2.921501	-0.930020
H	-3.571606	-5.493151	0.181846
C	2.828745	-2.510647	-0.968669
O	0.533525	-3.760543	-1.120869
O	1.180028	-2.659257	1.091896
C	5.452733	-2.182114	-1.905708
C	3.852448	-2.204822	-0.069278
C	3.111773	-2.707480	-2.323337
C	4.417309	-2.533925	-2.781519
C	5.152029	-2.047072	-0.542922
H	3.632082	-2.094402	0.985342
H	2.328221	-3.020105	-3.005524
H	4.634795	-2.678184	-3.837051
H	5.944391	-1.805175	0.161300
C	6.850992	-1.925929	-2.412585
H	7.607169	-2.330409	-1.730725
H	7.030259	-0.846454	-2.497072
H	7.010502	-2.368270	-3.401026
H	-0.949512	-1.496263	-4.000190
C	1.901337	0.612053	0.393709
C	2.461149	0.417183	1.664117
C	2.706074	1.246384	-0.565453
C	3.765412	0.822253	1.959172
H	1.875322	-0.079971	2.428845
C	4.009576	1.659085	-0.276099
H	2.313891	1.433699	-1.561170
C	4.550394	1.447765	0.991598
H	5.559564	1.768256	1.221969
C	4.858466	2.265890	-1.358416
C	4.359314	0.476069	3.294853
F	5.459455	1.313832	-2.118793
F	5.846366	3.042176	-0.858529
F	4.132250	3.029995	-2.204704
F	5.400938	1.274516	3.619537
F	4.826782	-0.799985	3.304947
F	3.454980	0.564007	4.295557
O	-0.349822	-0.004485	1.303887
C	-1.603949	-0.358441	1.370434
C	-1.987927	-1.295664	2.450110
C	-3.138148	-2.084869	2.300618
C	-1.200443	-1.427153	3.604147
C	-3.499874	-2.988411	3.297414
H	-3.729223	-2.022916	1.390819
C	-1.570092	-2.325560	4.600013
H	-0.297512	-0.834743	3.702861
C	-2.721884	-3.104795	4.451201
H	-4.379816	-3.610420	3.162674
H	-0.954142	-2.428809	5.488803
H	-3.003943	-3.810217	5.228157
H	-2.141072	-0.467618	0.426211
C	-2.599407	1.315821	2.121445
H	-1.768936	1.554287	2.776503
H	-3.440545	0.820388	2.588032
C	-2.906402	2.283108	1.154525
C	-4.228276	2.307375	0.488491
C	-4.977506	1.126349	0.337023
C	-4.806214	3.524699	0.087335
C	-6.259561	1.165617	-0.204751
H	-4.555205	0.171984	0.629855
C	-6.089816	3.561419	-0.451319
H	-4.268449	4.451821	0.240168
C	-6.820976	2.381556	-0.602109
H	-6.824413	0.242677	-0.304307
H	-6.523847	4.513795	-0.741632
H	-7.824756	2.410937	-1.016640
O	-2.014646	3.208244	0.902774
Si	-1.422782	4.432968	-0.182445
C	-2.042938	6.064188	0.537253
H	-1.499827	6.889713	0.059396
H	-3.111140	6.251404	0.383699
H	-1.840935	6.121254	1.612975
C	-1.990776	4.157633	-1.944324
H	-1.554828	4.947944	-2.570632
H	-1.640993	3.195767	-2.336810
H	-3.078009	4.209883	-2.059099
C	0.432541	4.342621	0.016336
H	0.728772	4.504460	1.058803
H	0.812231	3.368098	-0.299238
H	0.918914	5.113488	-0.594444

TS 202

B3LYP/6-31G(d) = -3573.918866
B3LYP-D3(BJ)/def2-TZVPP/IEFPCM(propanonitrile) = -3575.499851
B3LYP-D3(BJ)/def2-TZVPP/IEFPCM(propanonitrile)//B3LYP-D3(BJ)/6-31G(d) Free Energy (Quasiharmonic) = -3574.783034

Frequencies (Top 3 out of 300)

1. -228.7209 cm^{-1}
2. 7.2378 cm^{-1}
3. 15.7699 cm^{-1}

B3LYP/6-31G(d) Molecular Geometry in Cartesian Coordinates

B	-0.324360	0.505159	0.838650
O	-0.371380	0.834579	2.283560
N	-0.323160	1.948089	0.227170
C	-0.769160	2.917679	1.246750
S	-0.092380	2.436039	-1.326000
C	-0.606680	2.121279	2.544040
O	-0.711060	2.590359	3.655280
H	-2.319449	4.011920	0.197810
H	-0.081589	3.772569	1.289240
C	-2.219019	3.481960	1.148410
C	-3.336380	2.483040	1.300940
C	-4.209000	1.959580	0.273090
C	-5.102240	1.051301	0.906580
N	-4.801780	1.049881	2.253500
C	-3.734680	1.895210	2.479860
H	-3.333850	2.021730	3.476100
C	-6.160990	0.549091	-1.168880
C	-4.325730	2.158280	-1.115130
H	-6.732650	-0.365709	0.709490
H	-5.146280	0.368041	2.911880
C	-5.294950	1.453081	-1.818320
H	-6.902680	0.006901	-1.749520
H	-3.646810	2.826450	-1.633690
C	-6.077100	0.337201	0.202190
H	-5.382180	1.592461	-2.892550
C	1.199531	3.695569	-1.253950
O	-1.267830	3.128810	-1.870170
O	0.464310	1.275329	-2.048360
C	3.204551	5.663438	-1.166310
C	0.868161	5.014359	-0.927220
C	2.515831	3.362729	-1.575220
C	3.505711	4.344378	-1.529490
C	1.869861	5.981159	-0.875960
H	-0.166019	5.287159	-0.747050
H	2.748450	2.355568	-1.900470
H	4.527491	4.084758	-1.796940
H	1.606562	7.004859	-0.621110
C	4.283261	6.717108	-1.088020
H	5.169881	6.427578	-1.660840
H	4.600362	6.880078	-0.049500
H	3.930382	7.680318	-1.471910
H	-2.278939	4.233640	1.945530
C	-1.506451	-0.513720	0.429940
C	-2.177001	-1.221010	1.440300
C	-1.911121	-0.749540	-0.892190
C	-3.208611	-2.116900	1.146200
H	-1.897241	-1.054130	2.474980
C	-2.925321	-1.663280	-1.191960
H	-1.438400	-0.209790	-1.705370
C	-3.585501	-2.351180	-0.175610
H	-4.383501	-3.046400	-0.408460
C	-3.259021	-1.961640	-2.625210
C	-3.976571	-2.769750	2.258310
F	-4.523181	-2.409479	-2.780340
F	-2.444251	-2.944810	-3.125860
F	-3.098701	-0.894710	-3.429860
F	-3.228101	-2.948680	3.367570
F	-4.471661	-3.975930	1.902800
F	-5.054441	-2.019739	2.642900
O	0.994100	-0.286341	0.671620
C	2.179600	0.230029	0.765330
C	3.090590	-0.242492	1.821260
C	2.783499	-1.387742	2.572610
C	4.240960	0.501238	2.128980
C	3.620169	-1.780012	3.613810
H	1.876389	-1.937711	2.347450
C	5.073120	0.106498	3.173020
H	4.469690	1.399018	1.559000
C	4.765149	-1.036672	3.915040
H	3.373029	-2.659362	4.201860
H	5.955490	0.692348	3.414120
H	5.410829	-1.341692	4.733930
H	2.288970	1.278709	0.473580
C	3.151660	-0.305222	-1.090120
H	2.246640	-0.089931	-1.643820
H	3.902330	0.475848	-1.083640
C	3.592609	-1.615172	-0.949040
C	4.950419	-1.959302	-0.475330
C	5.189269	-3.224802	0.092310
C	6.026979	-1.060902	-0.596180
C	6.459349	-3.571393	0.541830
H	4.365779	-3.922752	0.185640
C	7.299489	-1.415833	-0.157570
H	5.882260	-0.094412	-1.066900
C	7.518749	-2.669153	0.417860
H	6.624878	-4.548013	0.987690
H	8.122509	-0.716163	-0.271640
H	8.512249	-2.944223	0.761070
O	2.804399	-2.659472	-1.110800
Si	1.465239	-3.215661	-2.072990
C	2.196078	-4.680891	-2.999570
H	3.003188	-4.372562	-3.674080
H	2.598548	-5.433651	-2.312450
H	1.423048	-5.165991	-3.608680
C	0.885089	-1.910151	-3.283030
H	1.656569	-1.682051	-4.027970
H	0.012369	-2.305971	-3.817150
H	0.573639	-0.972241	-2.816720
C	0.186859	-3.766151	-0.824630
H	-0.743452	-4.040401	-1.334190
H	0.537758	-4.635941	-0.257460
H	-0.048511	-2.963601	-0.119520

TS 203

B3LYP/6-31G(d) = -3573.926947
B3LYP-D3(BJ)/def2-TZVPP/IEFPCM(propanonitrile) = -3575.499903
B3LYP-D3(BJ)/def2-TZVPP/IEFPCM(propanonitrile)//B3LYP-D3(BJ)/6-31G(d) Free Energy (Quasiharmonic) = -3574.782906

Frequencies (Top 3 out of 300)

1. -263.4012 cm^{-1}
2. 10.3125 cm^{-1}
3. 15.0774 cm^{-1}

B3LYP/6-31G(d) Molecular Geometry in Cartesian Coordinates

B	0.138794	-0.461771	0.199948
O	0.118971	0.824337	-0.551660
N	0.809936	-1.381845	-0.869400
C	0.852129	-0.716838	-2.179717
S	1.422238	-2.904294	-0.695392
C	0.505214	0.725231	-1.819268
O	0.568548	1.670161	-2.585523
H	0.316687	-2.223781	-3.623383
H	1.873678	-0.713016	-2.586955
C	-0.090032	-1.263415	-3.297603
C	-1.528053	-1.434420	-2.906089
C	-2.594418	-0.460426	-2.960274
C	-3.761440	-1.091108	-2.441069
N	-3.417805	-2.388655	-2.118250
C	-2.076407	-2.577748	-2.377546

H	-1.594609	-3.520827	-2.161879
C	-5.041367	0.897139	-2.757691
C	-2.686383	0.874518	-3.394725
H	-5.863458	-0.922675	-1.926202
H	-3.974699	-3.020554	-1.561604
C	-3.903499	1.538038	-3.289244
H	-5.977895	1.443206	-2.682259
H	-1.812314	1.384904	-3.789426
C	-4.986239	-0.425219	-2.332048
H	-3.983214	2.569186	-3.625233
C	3.194700	-2.762326	-0.989857
O	0.918597	-3.780666	-1.766059
O	1.238486	-3.276893	0.713826
C	5.956631	-2.543167	-1.433566
C	3.699834	-2.873599	-2.288100
C	4.055570	-2.562625	0.090428
C	5.426031	-2.452574	-0.139080
C	5.072415	-2.754439	-2.500598
H	3.028256	-3.079443	-3.114804
H	3.654019	-2.515621	1.096306
H	6.094016	-2.300650	0.705363
H	5.463711	-2.839960	-3.511659
C	7.446117	-2.457550	-1.669558
H	7.903479	-3.455829	-1.665019
H	7.943078	-1.869273	-0.891142
H	7.673721	-2.001800	-2.639026
H	0.007706	-0.564776	-4.138067
C	-1.330391	-0.841162	0.758840
C	-2.279744	0.172451	0.952007
C	-1.735042	-2.155152	1.046689
C	-3.571569	-0.102449	1.415699
H	-2.018072	1.198394	0.717591
C	-3.032672	-2.436620	1.480428
H	-1.031441	-2.971813	0.929041
C	-3.960738	-1.412341	1.676715
H	-4.964957	-1.633689	2.017786
C	-3.480533	-3.862048	1.616808
C	-4.531957	1.032678	1.632756
F	-4.474123	-4.008885	2.521741
F	-2.482944	-4.694550	1.966927
F	-3.979110	-4.335791	0.430050
F	-4.162233	1.812073	2.683481
F	-5.789395	0.606205	1.888954
F	-4.600754	1.854670	0.559149
O	1.025563	-0.256187	1.433592
C	2.160066	0.368416	1.429941
C	3.121153	-0.020358	2.491466
C	2.693301	-0.791107	3.582800
C	4.474214	0.340057	2.387703
C	3.607174	-1.185010	4.558711
H	1.652824	-1.091353	3.639174
C	5.384643	-0.057983	3.362912
H	4.812838	0.923746	1.534387
C	4.951044	-0.818234	4.453823
H	3.271141	-1.786354	5.398551
H	6.430916	0.220780	3.273323
H	5.660957	-1.128563	5.215537
H	2.576633	0.671566	0.466320
C	1.958152	2.415438	2.007714
H	3.033714	2.535755	2.045516
H	1.455553	2.277311	2.957674
C	1.299812	3.113442	0.993250
C	-0.094511	3.567149	1.093998
C	-0.924195	3.632360	-0.042829
C	-0.617102	3.949630	2.345706
C	-2.244629	4.054010	0.079367
H	-0.550899	3.278526	-0.995525
C	-1.934091	4.383935	2.457075
H	0.019017	3.932158	3.224908
C	-2.751820	4.431380	1.325744
H	-2.889798	4.050418	-0.793727
H	-2.325648	4.677590	3.426432
H	-3.788259	4.741387	1.418006
O	1.976099	3.307832	-0.127238
Si	2.424303	4.544003	-1.243179
C	3.221627	5.889332	-0.193239
H	4.063803	5.502124	0.391421
H	3.601682	6.697535	-0.830229
H	2.502257	6.333640	0.504904
C	0.996646	5.226437	-2.248917
H	0.241144	5.731156	-1.639105
H	1.393127	5.959570	-2.964133
H	0.511721	4.427579	-2.818577
C	3.646867	3.651585	-2.346499
H	4.493890	3.251293	-1.778329
H	3.144263	2.819344	-2.851515
H	4.044377	4.323798	-3.116454

TS 204

B3LYP/6-31G(d) = -3573.919019
B3LYP-D3(BJ)/def2-TZVPP/IEFPCM(propanonitrile) = -3575.499663
B3LYP-D3(BJ)/def2-TZVPP/IEFPCM(propanonitrile)//B3LYP-D3(BJ)/6-31G(d) Free Energy (Quasiharmonic) = -3574.782824

Frequencies (Top 3 out of 300)

1. -257.6581 cm^{-1}
2. 9.0814 cm^{-1}
3. 10.9747 cm^{-1}

B3LYP/6-31G(d) Molecular Geometry in Cartesian Coordinates

B	-0.365686	0.596924	0.930585
O	-0.570906	0.908394	2.366653
N	-0.326067	2.057273	0.342373
C	-0.893948	3.004764	1.324847
S	0.057444	2.575962	-1.159915
C	-0.860894	2.184287	2.618034
O	-1.098534	2.632247	3.718229
H	-2.314740	4.107959	0.124057
H	-0.228339	3.867948	1.452103
C	-2.324763	3.565357	1.072415
C	-3.453637	2.568798	1.090129
C	-4.214821	2.067800	-0.032547
C	-5.180534	1.161746	0.488027
N	-5.027194	1.139647	1.859296
C	-3.981661	1.969869	2.210703
H	-3.688824	2.079625	3.245772
C	-6.014306	0.700841	-1.696887
C	-4.177315	2.285274	-1.422272
H	-6.797244	-0.230243	0.095434
H	-5.444806	0.451389	2.466603
C	-5.072232	1.601153	-2.236265
H	-6.695912	0.176703	-2.361799
H	-3.438717	2.952150	-1.854165
C	-6.081697	0.469916	-0.327766
H	-5.040408	1.754327	-3.311672
C	1.346011	3.825545	-0.920584
O	0.662986	1.422297	-1.860425
O	-1.036008	3.291209	-1.834155
C	3.390857	5.745890	-0.726655
C	2.209355	3.815953	0.176140
C	1.489450	4.795893	-1.917179
C	2.509213	5.738281	-1.818030
C	3.221433	4.773242	0.265987
H	2.084545	3.085707	0.968455
H	0.798169	4.812247	-2.752858
H	2.617300	6.487896	-2.598492
H	3.881528	4.767590	1.130050
C	4.469229	6.797023	-0.613955

H	4.048956	7.759886	-0.295650
H	5.231754	6.513744	0.118275
H	4.966326	6.965122	-1.575851
H	-2.473003	4.308781	1.865623
C	-1.516866	-0.401494	0.390192
C	-2.296339	-1.116485	1.313491
C	-1.790628	-0.605867	-0.969209
C	-3.308350	-1.986205	0.900118
C	-2.789919	-1.489915	-1.387463
H	-1.225815	-0.063306	-1.721199
C	-3.560484	-2.184110	-0.457880
H	-4.345687	-2.856166	-0.783743
C	-2.967673	-1.753901	-2.853362
C	-4.189212	-2.656836	1.913183
F	-2.804363	-0.651560	-3.607296
F	-4.174852	-2.280924	-3.148207
F	-2.035918	-2.659911	-3.295737
F	-5.317319	-1.928672	2.174253
F	-3.572415	-2.835489	3.100134
F	-4.621010	-3.868949	1.494207
O	0.956109	-0.175109	0.910491
C	2.144013	0.173075	0.507545
C	3.274210	-0.039228	1.449256
C	3.134220	-0.879031	2.563554
C	4.472702	0.666480	1.263126
C	4.183282	-1.016137	3.470214
H	2.190937	-1.391435	2.720068
C	5.519007	0.531647	2.175156
H	4.572712	1.342620	0.416531
C	5.377114	-0.314515	3.278794
H	4.064900	-1.659730	4.337472
H	6.436928	1.096120	2.034598
H	6.187826	-0.413508	3.995565
H	2.213867	1.071276	-0.104512
C	2.586640	-0.904848	-1.143875
H	1.638609	-0.697140	-1.622833
H	3.421155	-0.318874	-1.514698
C	2.870014	-2.196515	-0.693458
C	1.854311	-3.215365	-0.424037
C	0.611113	-3.201075	-1.085410
C	2.135434	-4.258936	0.483329
C	-0.320874	-4.207298	-0.850284
H	0.377208	-2.430871	-1.810399
C	1.190371	-5.246044	0.732393
H	3.089370	-4.268433	0.998100
C	-0.037475	-5.225123	0.062049
H	-1.263104	-4.188158	-1.385581
H	1.408230	-6.035225	1.446290
H	-0.771052	-6.004330	0.249478
O	4.104446	-2.523748	-0.325736
Si	5.644797	-2.603340	-1.090364
C	6.781555	-3.064674	0.324436
H	7.821663	-3.134268	-0.016109
H	6.735068	-2.314248	1.121178
H	6.505854	-4.032878	0.756827
C	6.128356	-0.959752	-1.865462
H	5.477978	-0.678779	-2.701120
H	6.117692	-0.147946	-1.130787
H	7.149551	-1.034449	-2.261097
H	-2.116304	-0.976229	2.373808
C	5.514547	-3.948665	-2.395875
H	5.213607	-4.905572	-1.955391
H	4.782023	-3.690870	-3.169725
H	6.480475	-4.097817	-2.894080

TS 205

B3LYP/6-31G(d) = -3573.925857
B3LYP-D3(BJ)/def2-TZVPP/IEFPCM(propanonitrile) = -3575.499801
B3LYP-D3(BJ)/def2-TZVPP/IEFPCM(propanonitrile)//B3LYP-D3(BJ)/6-31G(d) Free Energy (Quasiharmonic) = -3574.782791

Frequencies (Top 3 out of 300)

1. -272.3222 cm^{-1}
2. 5.4998 cm^{-1}
3. 12.3175 cm^{-1}

B3LYP/6-31G(d) Molecular Geometry in Cartesian Coordinates

B	0.049864	0.127337	-0.301469
O	0.084120	-0.874983	-1.394388
N	-0.523543	-0.755035	0.869245
C	-0.460537	-2.180334	0.497975
S	-0.584332	-0.334426	2.462590
C	0.016976	-2.121018	-0.953516
O	0.293778	-3.104610	-1.616669
H	-2.060471	-3.024386	1.676128
H	0.306303	-2.695257	1.089136
C	-1.766117	-3.019960	0.621555
C	-2.934970	-2.622895	-0.239864
C	-3.250275	-3.093855	-1.573532
C	-4.461758	-2.460372	-1.966304
N	-4.873392	-1.669993	-0.912086
C	-3.947751	-1.761604	0.108921
H	-4.100817	-1.214128	1.026575
C	-4.434846	-3.580596	-4.069706
C	-2.645288	-3.998754	-2.465084
H	-5.981523	-2.182011	-3.491532
H	-5.587557	-0.959696	-0.964393
C	-3.241555	-4.232984	-3.697330
H	-4.872290	-3.779340	-5.044605
H	-1.708649	-4.480136	-2.204894
C	-5.060609	-2.686328	-3.210128
H	-2.775722	-4.925245	-4.393736
C	-2.294999	-0.334253	3.016461
O	-0.150486	1.073046	2.553360
O	0.112842	-1.364505	3.259242
C	-4.945057	-0.284326	3.925801
C	-2.756153	-1.351422	3.851445
C	-3.133785	0.718411	2.641383
C	-4.451379	0.731147	3.091027
C	-4.078259	-1.320503	4.296683
H	-2.081867	-2.144955	4.155029
H	-2.760552	1.512306	2.004572
H	-5.107429	1.541057	2.782517
H	-4.439483	-2.114172	4.946090
C	-6.366446	-0.240921	4.433901
H	-6.722850	-1.236825	4.715383
H	-6.446553	0.399722	5.322119
H	-7.049094	0.165605	3.680112
H	-1.471512	-4.049354	0.383742
C	-0.847923	1.417046	-0.677098
C	-0.332240	2.702076	-0.891771
C	-2.233001	1.256170	-0.828544
C	-1.160343	3.780203	-1.221295
C	-3.068837	2.330419	-1.146703
H	-2.666193	0.271064	-0.687250
C	-2.538130	3.604681	-1.344406
H	-3.180700	4.439912	-1.594735
C	-4.546333	2.087895	-1.262702
C	-0.564691	5.151842	-1.381598
F	-5.049857	1.510578	-0.130597
F	-4.853748	1.236697	-2.271397
F	-5.250332	3.219798	-1.468150
F	-0.399644	5.772401	-0.187869
F	-1.343616	5.961181	-2.136139
F	0.654730	5.107887	-1.965290
O	1.524754	0.521894	-0.203889
C	2.223126	1.051031	0.770249

C	3.276022	2.026956	0.367857
C	3.844995	2.867985	1.336047
C	3.658307	2.168142	-0.973694
C	4.785979	3.828275	0.971778
H	3.533013	2.780839	2.374708
C	4.597848	3.132250	-1.336383
H	3.191175	1.536185	-1.722058
C	5.166344	3.960636	-0.366336
H	5.212205	4.482118	1.727632
H	4.879219	3.244365	-2.379913
H	5.893303	4.715753	-0.652786
H	1.673629	1.315304	1.675167
C	3.164828	-0.389653	1.757850
H	3.688009	0.228616	2.479122
H	2.292611	-0.913741	2.139152
C	3.953756	-1.069215	0.819844
C	5.336766	-0.657484	0.513401
C	6.202964	-0.230005	1.535409
C	5.798517	-0.660109	-0.815661
C	7.504160	0.164949	1.236432
H	5.866986	-0.242287	2.567390
C	7.093363	-0.245629	-1.112504
H	5.125066	-0.952460	-1.614082
C	7.950755	0.161182	-0.086928
H	8.170248	0.475020	2.036214
H	7.432907	-0.234372	-2.144135
H	8.964123	0.476490	-0.319068
O	3.381825	-2.001021	0.091313
Si	3.803165	-3.560367	-0.585310
C	5.566911	-4.004366	-0.092990
H	5.741392	-5.054151	-0.362672
H	6.328617	-3.400383	-0.593350
H	5.723116	-3.918727	0.988750
C	3.551253	-3.418322	-2.433395
H	4.240029	-2.713066	-2.911140
H	3.705732	-4.398958	-2.901648
H	2.520635	-3.109379	-2.639815
H	0.735528	2.875323	-0.810954
C	2.593863	-4.750625	0.205144
H	2.602371	-4.677607	1.298679
H	1.581561	-4.551932	-0.160114
H	2.854550	-5.782695	-0.062107

TS 206

B3LYP/6-31G(d) = -3573.928977
B3LYP-D3(BJ)/def2-TZVPP/IEFPCM(propanonitrile) = -3575.50004
B3LYP-D3(BJ)/def2-TZVPP/IEFPCM(propanonitrile)//B3LYP-D3(BJ)/6-31G(d) Free Energy (Quasiharmonic) = -3574.782786

Frequencies (Top 3 out of 300)

1. -272.9258 cm^{-1}
2. 5.3354 cm^{-1}
3. 14.4011 cm^{-1}

B3LYP/6-31G(d) Molecular Geometry in Cartesian Coordinates

B	-0.319567	0.232194	-0.441538
O	-1.236118	-0.893694	-0.772109
N	0.260292	0.576216	-1.859005
C	-0.347285	-0.304372	-2.880742
S	0.545032	2.126226	-2.415292
C	-1.213743	-1.249225	-2.055752
O	-1.824800	-2.208376	-2.491526
H	1.101710	-0.351461	-4.461266
H	-1.036582	0.255673	-3.532054
C	0.619404	-1.082136	-3.808831
C	1.655200	-1.918443	-3.113004
C	3.046602	-1.586604	-2.921347
C	3.645741	-2.662920	-2.211291
N	2.664790	-3.611706	-2.004987
C	1.471948	-3.153914	-2.537813
H	0.576056	-3.757298	-2.482057
C	5.761625	-1.564798	-2.231733
C	3.846327	-0.490932	-3.296701
H	5.434162	-3.488137	-1.297402
H	2.756692	-4.416368	-1.404536
C	5.192365	-0.491541	-2.949556
H	6.816394	-1.533883	-1.971409
H	3.411632	0.345767	-3.836730
C	4.998699	-2.662978	-1.853885
H	5.820130	0.348412	-3.235589
C	1.635683	2.818669	-1.175124
O	-0.695962	2.929876	-2.423585
O	1.274267	2.006512	-3.689847
C	3.375746	4.010049	0.666007
C	1.261234	4.003539	-0.542994
C	2.876123	2.227260	-0.919104
C	3.730383	2.823663	0.003490
C	2.135001	4.589857	0.373420
H	0.301029	4.451501	-0.771849
H	3.164419	1.311098	-1.423647
H	4.689389	2.357941	0.215789
H	1.847341	5.514740	0.867826
C	4.302624	4.624221	1.686943
H	4.302779	4.029732	2.607863
H	4.000351	5.644635	1.942513
H	5.335149	4.656569	1.320878
H	-0.020234	-1.712346	-4.438712
C	0.752096	-0.219019	0.685371
C	1.226519	0.644084	1.683621
C	1.225296	-1.538914	0.710586
C	2.126167	0.209393	2.661621
H	0.888335	1.673825	1.705073
C	2.107331	-1.987715	1.697643
H	0.889571	-2.236031	-0.048111
C	2.566406	-1.113865	2.683092
H	3.241569	-1.458575	3.458440
C	2.597901	-3.407439	1.685874
C	2.674127	1.174524	3.674648
F	1.710798	-4.252637	1.091595
F	2.820789	-3.880759	2.931760
F	3.762474	-3.551445	1.004802
F	2.858509	0.593162	4.882726
F	1.868739	2.243577	3.860390
F	3.886814	1.662443	3.297730
O	-1.110133	1.377096	0.172416
C	-2.329917	1.710240	-0.143830
C	-2.715483	3.130489	0.049190
C	-3.754549	3.669157	-0.722216
C	-2.060589	3.938543	0.989006
C	-4.129740	5.001167	-0.562380
H	-4.254162	3.047566	-1.461262
C	-2.442429	5.268537	1.150865
H	-1.250292	3.521417	1.577708
C	-3.476841	5.802650	0.377170
H	-4.924584	5.416041	-1.175677
H	-1.931044	5.891416	1.879637
H	-3.769729	6.841342	0.503047
H	-2.785401	1.208272	-0.998365
C	-3.578091	0.913226	1.305583
H	-4.410838	1.581797	1.130043
H	-2.939867	1.147079	2.151766
C	-3.798675	-0.438387	1.023357
C	-4.892510	-0.854759	0.115365
C	-6.175683	-0.295959	0.261278
C	-4.658004	-1.781349	-0.916047
C	-7.208923	-0.674543	-0.592065
H	-6.369596	0.407888	1.065243
C	-5.691820	-2.135507	-1.781413

H	-3.661027	-2.173122	-1.087107
C	-6.968285	-1.593273	-1.616964
H	-8.200975	-0.253013	-0.457534
H	-5.490975	-2.829707	-2.592076
H	-7.772533	-1.880006	-2.288879
O	-2.966220	-1.323667	1.513072
Si	-2.933678	-3.001582	1.966990
C	-2.085454	-2.930795	3.638606
H	-2.669543	-2.360627	4.369740
H	-1.938868	-3.941047	4.040551
H	-1.099599	-2.460807	3.551554
C	-4.699681	-3.632816	2.127958
H	-5.320848	-2.964213	2.734960
H	-5.191968	-3.766968	1.160324
H	-4.680372	-4.608080	2.631423
C	-1.927275	-3.985205	0.728911
H	-2.191492	-5.047503	0.812716
H	-0.856690	-3.893200	0.937003
H	-2.091055	-3.672560	-0.306660

TS 207

B3LYP/6-31G(d) = -3573.925961
B3LYP-D3(BJ)/def2-TZVPP/IEFPCM(propanonitrile) = -3575.500662
B3LYP-D3(BJ)/def2-TZVPP/IEFPCM(propanonitrile)//B3LYP-D3(BJ)/6-31G(d) Free Energy (Quasiharmonic) = -3574.782773

Frequencies (Top 3 out of 300)

1. -275.6285 cm^{-1}
2. 12.7269 cm^{-1}
3. 13.4342 cm^{-1}

B3LYP/6-31G(d) Molecular Geometry in Cartesian Coordinates

B	0.239203	-0.679566	-0.849980
O	-0.087672	-1.806096	-1.764039
N	0.018830	-1.377458	0.543926
C	-0.189389	-2.824696	0.366787
S	0.614102	-0.825275	1.981798
C	-0.321934	-2.965799	-1.154190
O	-0.586313	-4.003190	-1.721542
H	-1.094500	-3.546360	2.178576
H	0.718513	-3.384538	0.644532
C	-1.363285	-3.499668	1.120563
C	-2.729424	-2.882211	0.998548
C	-3.754772	-3.143974	0.008401
C	-4.903394	-2.389765	0.382651
N	-4.572734	-1.680283	1.523299
C	-3.278839	-2.003287	1.898051
H	-2.828322	-1.570313	2.778849
C	-6.138465	-3.244826	-1.469790
C	-3.823943	-3.950220	-1.143669
H	-6.968003	-1.853363	-0.033382
H	-5.226410	-1.170505	2.097389
C	-5.010616	-3.992851	-1.865925
H	-7.053798	-3.305228	-2.052675
H	-2.953906	-4.510332	-1.470965
C	-6.099530	-2.430325	-0.342358
H	-5.071021	-4.612431	-2.756412
C	2.260609	-1.509605	2.218206
O	-0.209566	-1.370438	3.078160
O	0.766018	0.634360	1.850588
C	4.822356	-2.623901	2.451261
C	3.375663	-0.774814	1.804927
C	2.414254	-2.780146	2.777773
C	3.693078	-3.328250	2.885991
C	4.642944	-1.334799	1.927059
H	3.245058	0.220185	1.395912
H	1.548702	-3.322171	3.143635
H	3.812346	-4.320433	3.314125
H	5.507361	-0.770264	1.589041
C	6.204276	-3.226500	2.527118
H	6.180902	-4.239520	2.939938
H	6.870506	-2.621720	3.154585
H	6.657099	-3.276011	1.529439
O	-0.746668	0.443594	-1.124113
C	-2.032217	0.178972	-1.216100
H	-2.387757	-0.687971	-0.657396
H	-1.387419	-4.529728	0.745321
C	-2.676765	0.416089	-2.528759
C	-3.896114	-0.212657	-2.827848
C	-2.059551	1.231426	-3.490224
C	-4.488749	-0.025358	-4.074290
H	-4.368404	-0.861191	-2.094910
C	-2.661070	1.420181	-4.731663
H	-1.104599	1.690539	-3.258102
C	-3.876491	0.794766	-5.025856
H	-5.422914	-0.529242	-4.305584
H	-2.177082	2.046180	-5.476324
H	-4.339524	0.937863	-5.998580
C	1.710220	-0.098737	-1.185175
C	2.750631	-0.978025	-1.529787
C	2.014809	1.266768	-1.160860
C	4.038118	-0.517790	-1.811253
H	2.554284	-2.043603	-1.592307
C	3.303379	1.736734	-1.441969
H	1.231589	1.978249	-0.925941
C	4.325759	0.848037	-1.765881
H	5.321441	1.210377	-1.993799
C	3.578191	3.210119	-1.346470
C	5.152047	-1.485435	-2.101111
F	2.632987	3.946011	-1.978482
F	3.581420	3.635066	-0.051316
F	4.700631	-2.675872	-2.543610
F	4.773268	3.554968	-1.870241
F	5.897989	-1.737691	-0.989696
F	6.010712	-1.003155	-3.029037
C	-3.204153	1.464644	-0.077735
H	-3.869180	0.717934	0.345130
H	-3.587882	2.035589	-0.911658
C	-2.385989	2.163873	0.814718
O	-2.156893	1.649625	2.002191
Si	-2.027868	2.289511	3.619099
C	-3.348449	3.629395	3.728001
H	-3.384019	4.037485	4.745754
H	-4.345061	3.237504	3.493885
H	-3.142549	4.462961	3.047234
C	-2.433253	0.799191	4.675688
H	-3.444598	0.425366	4.478539
H	-1.718998	-0.007429	4.477412
H	-2.374765	1.055667	5.740680
C	-0.327059	2.961662	4.027179
H	0.449854	2.247047	3.739840
H	-0.120876	3.918180	3.537570
H	-0.264511	3.119370	5.112020
C	-1.688378	3.396642	0.403309
C	-2.358324	4.383321	-0.344046
C	-0.327853	3.571606	0.717784
C	-1.686406	5.534612	-0.745053
H	-3.410984	4.261133	-0.580852
C	0.345631	4.712553	0.284633
H	0.200305	2.783593	1.242998
C	-0.333262	5.697699	-0.436332
H	-2.215890	6.301162	-1.303560
H	1.406023	4.816660	0.487483
H	0.194744	6.587889	-0.766611

TS 208

B3LYP/6-31G(d) = -3573.928309
B3LYP-D3(BJ)/def2-TZVPP/IEFPCM(propanonitrile) = -3575.498845
B3LYP-D3(BJ)/def2-TZVPP/IEFPCM(propanonitrile)//B3LYP-D3(BJ)/6-31G(d) Free Energy (Quasiharmonic) = -3574.782765

Frequencies (Top 3 out of 300)

1. -217.9032 cm^{-1}
2. 3.2667 cm^{-1}
3. 10.8357 cm^{-1}

B3LYP/6-31G(d) Molecular Geometry in Cartesian Coordinates

B	0.015081	-0.274082	-0.992548
O	0.063821	-0.559132	-2.441978
N	0.603776	1.184662	-0.953395
C	0.979419	1.616986	-2.315414
S	0.184149	2.335523	0.148464
C	0.525825	0.446654	-3.191326
O	0.610848	0.415008	-4.397582
H	2.748931	2.817061	-1.996517
H	0.400878	2.501032	-2.612265
C	2.479492	1.926284	-2.572408
C	3.461571	0.823709	-2.266479
C	4.685930	0.960616	-1.511322
C	5.306074	-0.320189	-1.470346
N	4.512846	-1.179433	-2.199658
C	3.409963	-0.491264	-2.665869
H	2.680456	-1.002093	-3.276353
C	7.121547	0.536988	-0.182392
C	5.334205	2.040097	-0.886043
H	6.965890	-1.536689	-0.779192
H	4.588394	-2.186266	-2.199616
C	6.539531	1.820488	-0.230170
H	8.063807	0.395046	0.340213
H	4.900452	3.035850	-0.913077
C	6.514144	-0.548266	-0.803128
H	7.049186	2.652180	0.250232
C	1.667231	3.197263	0.676017
O	-0.323136	1.614452	1.335826
O	-0.690897	3.346633	-0.481509
C	3.890254	4.606125	1.628157
C	2.560131	2.566375	1.547837
C	1.866172	4.518517	0.277450
C	2.980671	5.211562	0.752996
C	3.661491	3.275431	2.014141
H	2.397448	1.542728	1.866617
H	1.147387	4.996603	-0.379020
H	3.137379	6.242457	0.445344
H	4.359874	2.784897	2.686984
C	5.086808	5.359635	2.157633
H	6.013730	4.798632	1.990029
H	5.191064	6.337322	1.677554
H	5.002930	5.525407	3.239144
H	2.533107	2.215623	-3.630822
C	0.818185	-1.382189	-0.140792
C	1.306631	-2.533118	-0.775760
C	1.135833	-1.221826	1.215345
C	2.102218	-3.463526	-0.099622
H	1.074753	-2.696131	-1.823322
C	1.925051	-2.151653	1.898264
H	0.780091	-0.342345	1.741369
C	2.420728	-3.279553	1.245428
H	3.046479	-3.992241	1.769124
C	2.222780	-1.926209	3.352784
C	2.690938	-4.617986	-0.857472
F	3.278925	-2.646675	3.784301
F	1.171110	-2.269011	4.149215
F	2.485144	-0.623693	3.617886
F	3.051667	-5.638332	-0.051565
F	1.844119	-5.108557	-1.785487
F	3.824091	-4.251508	-1.535903
O	-1.484843	-0.402970	-0.623268
C	-2.407104	0.326191	-1.175673
C	-3.510796	-0.333797	-1.891259
C	-3.758658	-1.705944	-1.726925
C	-4.281235	0.405732	-2.802674
C	-4.769289	-2.321864	-2.460068
H	-3.140195	-2.274708	-1.041278
C	-5.286346	-0.216497	-3.539955
H	-4.071894	1.462869	-2.949313
C	-5.534932	-1.581003	-3.365400
H	-4.953160	-3.385351	-2.336271
H	-5.864393	0.356217	-4.259842
H	-6.313938	-2.068828	-3.944966
H	-2.116425	1.316519	-1.538495
C	-3.392700	1.368495	0.463092
H	-3.811956	2.197018	-0.096315
H	-2.443873	1.555605	0.947693
C	-4.259453	0.411153	0.964779
C	-3.850120	-0.698315	1.833277
C	-4.772034	-1.719896	2.143021
C	-2.544349	-0.774516	2.360555
C	-4.399383	-2.783296	2.956445
H	-5.772140	-1.672163	1.728907
C	-2.178527	-1.844108	3.173657
H	-1.817130	-0.002333	2.141852
C	-3.101919	-2.847694	3.473796
H	-5.117979	-3.565227	3.185931
H	-1.168403	-1.897834	3.565679
H	-2.810882	-3.679498	4.109619
O	-5.532416	0.360593	0.558067
Si	-6.797422	1.520143	0.464489
C	-6.996589	2.249915	2.185535
H	-7.817775	2.976924	2.204572
H	-7.218467	1.474397	2.927065
H	-6.087623	2.771305	2.507400
C	-8.259560	0.470940	-0.061703
H	-8.057028	-0.023570	-1.018443
H	-8.481017	-0.306711	0.677666
H	-9.160776	1.083649	-0.183782
C	-6.452470	2.866605	-0.803759
H	-7.357108	3.473416	-0.939776
H	-5.649898	3.546635	-0.498354
H	-6.191054	2.441046	-1.778144

TS 209

B3LYP/6-31G(d) = -3573.923741
B3LYP-D3(BJ)/def2-TZVPP/IEFPCM(propanonitrile) = -3575.498765
B3LYP-D3(BJ)/def2-TZVPP/IEFPCM(propanonitrile)//B3LYP-D3(BJ)/6-31G(d) Free Energy (Quasiharmonic) = -3574.782749

Frequencies (Top 3 out of 300)

1. -256.2099 cm^{-1}
2. 8.0314 cm^{-1}
3. 12.1908 cm^{-1}

B3LYP/6-31G(d) Molecular Geometry in Cartesian Coordinates

B	-0.081967	0.008845	0.940625
O	-0.091151	-0.068922	2.412775
N	-0.719868	1.438679	0.694898
C	-1.155318	1.995426	1.992799
S	-0.163304	2.486780	-0.443531
C	-0.601075	1.003735	3.018120
O	-0.662454	1.147991	4.219483
H	-3.079919	2.823782	1.435920
H	-0.693583	2.971963	2.180710
C	-2.695371	2.124059	2.184608

C	-3.459228	0.825999	2.123338
C	-4.322831	0.361851	1.062716
C	-4.769056	-0.940582	1.421000
N	-4.232895	-1.230572	2.657931
C	-3.437713	-0.177913	3.064768
H	-2.906941	-0.222650	4.005469
C	-6.009277	-1.139814	-0.605584
C	-4.770689	0.911514	-0.150985
H	-5.906580	-2.707079	0.885301
H	-4.226569	-2.150232	3.072143
C	-5.601959	0.159579	-0.972112
H	-6.652225	-1.707468	-1.272853
H	-4.466685	1.910241	-0.447964
C	-5.602420	-1.704425	0.597598
H	-5.943823	0.575825	-1.916222
C	-1.549750	3.492915	-0.982815
O	0.821463	3.433128	0.128760
O	0.250156	1.671009	-1.604304
C	-3.643828	5.106818	-1.909232
C	-2.428008	2.993893	-1.948780
C	-1.693852	4.788819	-0.486296
C	-2.743138	5.583097	-0.948026
C	-3.463245	3.806125	-2.404786
H	-2.304383	1.990475	-2.341603
H	-0.978711	5.173050	0.232897
H	-2.855239	6.593540	-0.562828
H	-4.143063	3.422580	-3.161752
C	-4.790605	5.961671	-2.392907
H	-5.726715	5.682362	-1.891881
H	-4.613675	7.022631	-2.190827
H	-4.952737	5.841752	-3.469600
H	-2.830811	2.601841	3.162950
C	-0.916333	-1.191739	0.258237
C	-1.249737	-2.336483	0.996521
C	-1.395459	-1.115555	-1.056489
C	-2.045983	-3.348122	0.451753
C	-2.184965	-2.126967	-1.608582
H	-1.168340	-0.239275	-1.654696
C	-2.520075	-3.251424	-0.857202
H	-3.149636	-4.027326	-1.275994
C	-2.658294	-1.989589	-3.027977
C	-2.481439	-4.498012	1.312310
F	-3.021228	-0.716102	-3.318327
F	-3.719038	-2.781167	-3.297965
F	-1.686942	-2.319851	-3.917423
F	-1.558737	-4.826233	2.241391
F	-2.750142	-5.609431	0.593916
F	-3.628764	-4.210076	2.003776
O	1.420978	-0.137110	0.637795
C	2.013823	-0.428496	-0.489081
C	2.842062	-1.652734	-0.533083
C	3.187097	-2.206966	-1.775602
C	3.231349	-2.305647	0.646708
C	3.912294	-3.394612	-1.838863
H	2.870413	-1.713561	-2.691193
C	3.959829	-3.491092	0.578911
H	2.932195	-1.890252	1.602901
C	4.302435	-4.037028	-0.661442
H	4.165411	-3.822878	-2.804528
H	4.249093	-3.999187	1.494794
H	4.862684	-4.966845	-0.710185
H	1.483107	-0.171563	-1.405428
C	3.280996	1.207866	-0.823814
H	3.131113	1.297613	-1.892507
H	2.682176	1.889420	-0.234056
C	4.550896	0.854634	-0.369897
C	5.571051	0.233586	-1.236744
C	5.533135	0.376268	-2.636899
C	6.620659	-0.506175	-0.660576
C	6.511688	-0.209801	-3.434425
H	4.756727	0.971327	-3.105402
C	7.589827	-1.100618	-1.461962
H	6.655221	-0.619006	0.416370
C	7.539454	-0.953890	-2.850495
H	6.475826	-0.079970	-4.512202
H	8.387423	-1.678486	-1.003874
H	8.301497	-1.412193	-3.474861
O	4.907774	0.935699	0.897321
Si	4.511380	1.854970	2.324028
C	6.204226	2.053363	3.116906
H	6.884176	2.628695	2.478469
H	6.119418	2.581479	4.074686
H	6.669285	1.081085	3.315704
C	3.381526	0.807893	3.384556
H	3.097193	1.357060	4.290956
H	2.461568	0.527885	2.863416
H	3.885123	-0.112690	3.702801
H	-0.904758	-2.423965	2.021786
C	3.802953	3.523112	1.845599
H	3.770548	4.153218	2.744377
H	4.443240	4.033413	1.116059
H	2.789286	3.478530	1.436259

TS 210

B3LYP/6-31G(d) = -3573.929964
B3LYP-D3(BJ)/def2-TZVPP/IEFPCM(propanonitrile) = -3575.500858
B3LYP-D3(BJ)/def2-TZVPP/IEFPCM(propanonitrile)//B3LYP-D3(BJ)/6-31G(d) Free Energy (Quasiharmonic) = -3574.782665

Frequencies (Top 3 out of 300)

1. -266.8729 cm^{-1}
2. 15.0466 cm^{-1}
3. 18.5647 cm^{-1}

B3LYP/6-31G(d) Molecular Geometry in Cartesian Coordinates

B	0.259190	-0.371719	-0.084790
O	0.237480	0.808521	-0.995400
N	0.825250	-1.455399	-1.035710
C	0.845340	-0.976429	-2.423440
S	1.559710	-2.869419	-0.644720
C	0.511640	0.506181	-2.262150
O	0.509290	1.323571	-3.164360
H	0.330510	-2.671299	-3.638140
H	1.859850	-1.028120	-2.845020
C	-0.108940	-1.693939	-3.423790
C	-1.520380	-1.872579	-2.948480
C	-2.629270	-0.952949	-3.059650
C	-3.744120	-1.564569	-2.418250
N	-3.331430	-2.802909	-1.967780
C	-1.993180	-2.966579	-2.265810
H	-1.460300	-3.858559	-1.966980
C	-5.118960	0.327881	-2.886950
C	-2.795260	0.322221	-3.630480
H	-5.828410	-1.420759	-1.828340
H	-3.835410	-3.375149	-1.306390
C	-4.032570	0.948741	-3.536840
H	-6.073110	0.844621	-2.826700
H	-1.960990	0.818441	-4.118280
C	-4.990630	-0.937449	-2.324840
H	-4.167590	1.936151	-3.970870
C	3.319740	-2.603630	-0.963480
O	1.154559	-3.935339	-1.577460
O	1.367130	-3.071019	0.800000
C	6.075780	-2.283130	-1.387430
C	3.948850	-1.419890	-0.569560
C	4.051870	-3.626380	-1.568540
C	5.421390	-3.461230	-1.771120
C	5.317500	-1.268870	-0.785230

H	3.372300	-0.625830	-0.106390
H	3.545629	-4.533940	-1.878750
H	5.990109	-4.261080	-2.239660
H	5.803410	-0.343360	-0.484420
C	7.552530	-2.098020	-1.645240
H	8.088660	-3.052020	-1.607130
H	8.006680	-1.423210	-0.912030
H	7.729140	-1.665920	-2.639360
H	-0.074540	-1.109059	-4.351370
C	-1.218480	-0.603639	0.540520
C	-2.134080	0.457501	0.555090
C	-1.654610	-1.831739	1.066700
C	-3.422800	0.317051	1.080380
H	-1.837860	1.411251	0.132960
C	-2.953040	-1.985559	1.559470
H	-0.972800	-2.676239	1.087120
C	-3.845710	-0.910279	1.580460
H	-4.847650	-1.031599	1.974790
C	-3.435540	-3.342209	1.987150
C	-4.322060	1.518651	1.119950
F	-3.991071	-4.026249	0.938350
F	-4.397200	-3.270359	2.934540
F	-2.446901	-4.119009	2.465970
F	-5.594050	1.210951	1.444420
F	-4.352440	2.175221	-0.063370
F	-3.890860	2.428621	2.042010
O	1.289270	-0.102169	1.019070
C	0.987350	0.438851	2.166550
C	1.682510	-0.069230	3.371760
C	2.760990	-0.959950	3.260700
C	1.215650	0.295381	4.645910
C	3.369830	-1.460390	4.409810
H	3.085190	-1.284520	2.279490
C	1.825560	-0.208730	5.790320
H	0.364430	0.966311	4.737960
C	2.910010	-1.083660	5.673720
H	4.196040	-2.159530	4.317500
H	1.452560	0.071811	6.771250
H	3.385270	-1.480650	6.566560
H	-0.054160	0.729281	2.308570
C	1.604250	2.443201	2.303670
H	2.616940	2.264360	2.639380
H	0.882410	2.691901	3.074300
C	1.436420	3.074321	1.067210
C	2.552160	3.232240	0.116180
C	3.864490	3.433520	0.590180
C	2.332930	3.175990	-1.272070
C	4.923230	3.576940	-0.301270
H	4.049450	3.514580	1.656400
C	3.399240	3.298420	-2.159200
H	1.344620	2.971191	-1.661460
C	4.694080	3.504790	-1.678560
H	5.926400	3.750550	0.077610
H	3.211490	3.217190	-3.225470
H	5.523270	3.608370	-2.373130
O	0.220150	3.449011	0.734230
Si	-0.633280	4.718911	-0.090900
C	-1.848220	5.313001	1.210430
H	-1.337400	5.707201	2.096570
H	-2.478790	6.114681	0.805720
H	-2.510040	4.500641	1.528990
C	0.574410	6.085251	-0.556410
H	1.250900	5.799071	-1.366820
H	0.003230	6.960751	-0.891100
H	1.183840	6.400791	0.298470
C	-1.514440	3.973231	-1.563900
H	-0.869300	3.351811	-2.193350
H	-1.919480	4.782161	-2.186390
H	-2.359600	3.353291	-1.246500

TS 211

B3LYP/6-31G(d) = -3573.920678

B3LYP-D3(BJ)/def2-TZVPP/IEFPCM(propanonitrile) = -3575.4986

B3LYP-D3(BJ)/def2-TZVPP/IEFPCM(propanonitrile)//B3LYP-D3(BJ)/6-31G(d) Free Energy (Quasiharmonic) = -3574.782498

Frequencies (Top 3 out of 300)

1. -243.7160 cm^{-1}
2. 6.3004 cm^{-1}
3. 12.0261 cm^{-1}

B3LYP/6-31G(d) Molecular Geometry in Cartesian Coordinates

B	-0.259537	-0.590195	-0.754679
O	-0.984373	-1.308371	-1.830317
N	-0.677357	-1.436122	0.496972
C	-1.264651	-2.712526	0.049541
S	0.041221	-1.315597	1.985758
C	-1.557007	-2.445894	-1.431831
O	-2.181173	-3.188172	-2.156219
H	-2.133657	-3.650511	1.773260
H	-0.499835	-3.507899	0.055457
C	-2.486280	-3.284092	0.806648
C	-3.680959	-2.397729	1.031500
C	-4.875161	-2.297192	0.220388
C	-5.791786	-1.464289	0.920756
N	-5.162016	-1.048608	2.078488
C	-3.909096	-1.633397	2.149876
H	-3.261751	-1.473583	3.000621
C	-7.442517	-1.761699	-0.773858
C	-5.270954	-2.849365	-1.011565
H	-7.766975	-0.563062	0.998813
H	-5.607060	-0.550272	2.833207
C	-6.545997	-2.577938	-1.493162
H	-8.436280	-1.575424	-1.173229
H	-4.578958	-3.460566	-1.582366
C	-7.076456	-1.191251	0.440470
H	-6.859900	-3.001749	-2.443479
C	1.573031	-2.256762	1.962339
O	-0.824703	-1.967763	2.982034
O	0.390796	0.106800	2.137812
C	3.955488	-3.720162	1.743410
C	1.533288	-3.654275	1.988196
C	2.793824	-1.584523	1.890700
C	3.972196	-2.319350	1.786566
C	2.721706	-4.373022	1.870819
H	0.590129	-4.174242	2.119026
H	2.816290	-0.502497	1.913786
H	4.919681	-1.789768	1.722416
H	2.689151	-5.459755	1.876276
C	5.229527	-4.499221	1.526226
H	6.081726	-4.028313	2.028070
H	5.463514	-4.544900	0.454594
H	5.142878	-5.527896	1.890377
O	-0.840227	0.823698	-0.743143
C	-1.833838	1.236188	-0.011561
H	-1.834937	0.936000	1.037161
H	-2.790909	-4.159835	0.221252
C	-3.141478	1.456148	-0.660433
C	-4.267155	1.809288	0.103929
C	-3.280074	1.265816	-2.045872
C	-5.502230	1.988678	-0.507646
H	-4.172151	1.926391	1.180436
C	-4.521779	1.441590	-2.652478
H	-2.421344	0.938225	-2.620308
C	-5.630959	1.810464	-1.888930
H	-6.370246	2.252613	0.089252
H	-4.627307	1.273820	-3.720358
H	-6.599415	1.940292	-2.363500

C	1.319495	-0.510924	-1.104159
C	2.165224	0.509037	-0.650883
C	1.915331	-1.549471	-1.841523
C	3.544012	0.481609	-0.897578
H	1.747717	1.324534	-0.072163
C	3.288622	-1.583726	-2.088622
H	1.294920	-2.346730	-2.238001
C	4.116561	-0.565495	-1.614939
H	5.183127	-0.589138	-1.803859
C	3.903136	-2.762211	-2.792737
C	4.419055	1.535165	-0.286780
F	4.273557	-3.729857	-1.912544
F	3.052472	-3.338081	-3.666422
F	5.680017	1.516155	-0.762756
F	5.019360	-2.418311	-3.476354
F	4.504914	1.393073	1.064555
F	3.936582	2.792106	-0.493824
C	-1.497841	3.314679	0.504529
H	-2.196817	3.324860	1.329781
H	-1.886026	3.706037	-0.426189
C	-0.145747	3.509113	0.772322
O	0.706003	3.814674	-0.197737
Si	0.558130	4.568250	-1.754160
C	-0.094360	3.330581	-3.001397
H	0.026526	3.729935	-4.016432
H	-1.155606	3.106260	-2.854871
H	0.453758	2.384851	-2.940910
C	-0.555038	6.078229	-1.575582
H	-0.211227	6.729172	-0.763357
H	-0.516691	6.664047	-2.502781
H	-1.604978	5.831698	-1.389020
C	2.314210	5.093703	-2.137851
H	2.680343	5.822109	-1.405515
H	2.999369	4.241831	-2.131760
H	2.361564	5.562720	-3.128330
C	0.477435	3.326162	2.091726
C	1.879049	3.381261	2.212928
C	-0.292766	3.107277	3.250024
C	2.488565	3.210221	3.451432
H	2.483426	3.540390	1.329528
C	0.319832	2.946602	4.486428
H	-1.374528	3.059173	3.191932
C	1.712401	2.994994	4.591685
H	3.571934	3.236699	3.522165
H	-0.288167	2.775060	5.369717
H	2.188785	2.861130	5.558955

TS 212

B3LYP/6-31G(d) = -3573.917453
B3LYP-D3(BJ)/def2-TZVPP/IEFPCM(propanonitrile) = -3575.498475
B3LYP-D3(BJ)/def2-TZVPP/IEFPCM(propanonitrile)//B3LYP-D3(BJ)/6-31G(d) Free Energy (Quasiharmonic) = -3574.782215

Frequencies (Top 3 out of 300)

1. -295.9442 cm^{-1}
2. 9.2758 cm^{-1}
3. 12.9733 cm^{-1}

B3LYP/6-31G(d) Molecular Geometry in Cartesian Coordinates

B	0.448348	0.444449	-0.167778
O	0.108307	-0.205086	-1.467208
N	0.675807	1.912300	-0.634475
C	0.488328	2.042987	-2.085894
S	0.598199	3.262073	0.306086
C	0.041405	0.642464	-2.497275
O	-0.340361	0.315635	-3.603200
H	1.953024	3.520080	-2.632684
H	-0.338556	2.727453	-2.327199
C	1.726539	2.488371	-2.911868
C	2.943625	1.622966	-2.746106
C	4.078007	1.866093	-1.884956
C	4.972216	0.774355	-2.050921
N	4.414296	-0.075883	-2.985651
C	3.191018	0.430365	-3.384483
H	2.588760	-0.094453	-4.114188
C	6.496706	1.715910	-0.480926
C	4.425675	2.899134	-0.995101
H	6.839442	-0.173914	-1.478490
H	4.746055	-1.008328	-3.176834
C	5.629078	2.814433	-0.307123
H	7.426658	1.675185	0.080333
H	3.751851	3.734500	-0.831429
C	6.180828	0.680845	-1.352188
H	5.903951	3.602910	0.388379
C	-1.126935	3.806447	0.346436
O	1.316968	4.346722	-0.379899
O	0.943196	2.866617	1.674863
C	-3.790444	4.696024	0.443927
C	-1.609639	4.669852	-0.639835
C	-1.958350	3.392287	1.389201
C	-3.278126	3.841085	1.432266
C	-2.936988	5.096213	-0.592524
H	-0.941119	5.030423	-1.414234
H	-1.556451	2.750226	2.164947
H	-3.915400	3.546374	2.263669
H	-3.307705	5.769708	-1.361811
C	-5.205660	5.219190	0.525714
H	-5.633218	5.380772	-0.469590
H	-5.238863	6.182074	1.052359
H	-5.860108	4.530319	1.070492
H	1.399653	2.495112	-3.959323
C	1.668008	-0.310716	0.572862
C	2.435514	-1.258922	-0.115454
C	1.988328	-0.086074	1.921613
C	3.465578	-1.968558	0.510299
H	2.219237	-1.458859	-1.159881
C	3.022294	-0.784456	2.548876
H	1.437309	0.660409	2.482510
C	3.766727	-1.736188	1.849853
H	4.561785	-2.283480	2.341767
C	3.285341	-0.572586	4.014516
C	4.234977	-2.986986	-0.279447
F	4.565861	-0.858187	4.348319
F	2.499981	-1.373074	4.782574
F	3.038307	0.695349	4.401585
F	5.112305	-2.421696	-1.156736
F	3.411867	-3.762447	-1.031057
F	4.958871	-3.814666	0.503149
O	-0.808106	0.422204	0.708079
C	-1.724613	-0.493673	0.922974
C	-1.454717	-1.959912	1.049013
C	-2.072776	-2.610714	2.130596
C	-0.632701	-2.707339	0.191637
C	-1.855776	-3.966601	2.369183
H	-2.704774	-2.042452	2.809487
C	-0.412749	-4.061752	0.435907
H	-0.173595	-2.232764	-0.663874
C	-1.018615	-4.694969	1.522734
H	-2.327412	-4.447734	3.221549
H	0.246299	-4.618837	-0.223919
H	-0.837244	-5.749697	1.710657
H	-2.420122	-0.163006	1.698257
C	-2.994554	-0.176368	-0.560974
H	-3.385253	0.762081	-0.178916
H	-2.259441	-0.062326	-1.343691
C	-3.899038	-1.240073	-0.714401
C	-3.654699	-2.388748	-1.590558
C	-2.678030	-2.345722	-2.609689

C	-4.427432	-3.558876	-1.434111
C	-2.488194	-3.446429	-3.439599
H	-2.071851	-1.462930	-2.783725
C	-4.223315	-4.655637	-2.261525
H	-5.175277	-3.597017	-0.650963
C	-3.253796	-4.601743	-3.267955
H	-1.739758	-3.395430	-4.224387
H	-4.817955	-5.554084	-2.124156
H	-3.098582	-5.458091	-3.918551
O	-4.997311	-1.331907	0.019844
Si	-6.054358	-0.217064	0.820811
C	-6.433374	1.191303	-0.362696
H	-5.571860	1.844079	-0.537891
H	-6.777451	0.814476	-1.332559
H	-7.237023	1.812835	0.051983
C	-5.264087	0.377909	2.417732
H	-4.414339	1.046075	2.242588
H	-6.002690	0.936763	3.006630
H	-4.923337	-0.460545	3.035539
C	-7.556929	-1.283766	1.158750
H	-8.330052	-0.711832	1.685798
H	-7.996131	-1.660171	0.228302
H	-7.299284	-2.146880	1.782895

TS 213

B3LYP/6-31G(d) = -3573.92749
B3LYP-D3(BJ)/def2-TZVPP/IEFPCM(propanonitrile) = -3575.49851
B3LYP-D3(BJ)/def2-TZVPP/IEFPCM(propanonitrile)//B3LYP-D3(BJ)/6-31G(d) Free Energy (Quasiharmonic) = -3574.782024

Frequencies (Top 3 out of 300)

1. -269.6808 cm^{-1}
2. 6.2979 cm^{-1}
3. 9.0426 cm^{-1}

B3LYP/6-31G(d) Molecular Geometry in Cartesian Coordinates

B	0.180226	-0.301271	-0.916699
O	0.832767	-0.852488	-2.129976
N	1.170436	-0.791956	0.189850
C	2.370293	-1.407280	-0.414906
S	1.231738	-0.230262	1.745022
C	1.985974	-1.477280	-1.900213
O	2.644191	-2.031053	-2.753348
H	2.704101	-3.530152	-0.683867
H	3.231991	-0.727874	-0.344302
C	2.790997	-2.808197	0.136550
C	4.183744	-2.872735	0.697517
C	5.421893	-2.897594	-0.048212
C	6.481294	-2.990098	0.896919
N	5.905748	-3.012736	2.151940
C	4.529258	-2.942784	2.024752
H	3.889572	-2.932312	2.895651
C	8.102463	-2.998922	-0.850567
C	5.733622	-2.851588	-1.419641
H	8.622532	-3.114803	1.246953
H	6.405341	-3.088000	3.023772
C	7.068130	-2.903127	-1.804349
H	9.136098	-3.037741	-1.184582
H	4.939918	-2.763133	-2.156362
C	7.824913	-3.042504	0.511361
H	7.322408	-2.866531	-2.860358
C	2.309193	1.203551	1.768113
O	1.858011	-1.252986	2.595840
O	-0.134301	0.227124	2.064414
C	3.995958	3.435292	1.623800
C	3.693593	1.026503	1.831935
C	1.756101	2.482899	1.664383
C	2.601007	3.585632	1.599522
C	4.524512	2.145192	1.754087
H	4.113325	0.032424	1.947571
H	0.679811	2.604420	1.629514
H	2.171904	4.578914	1.501855
H	5.602436	2.008685	1.788854
C	4.890217	4.644156	1.491403
H	4.718582	5.360709	2.304167
H	4.686963	5.168091	0.549616
H	5.948496	4.366764	1.504518
O	-1.186843	-0.972971	-0.812874
C	-1.375361	-2.170433	-0.328999
H	-0.528562	-2.606695	0.210158
H	2.074830	-3.092895	0.910312
C	-2.165946	-3.126073	-1.143632
C	-2.124595	-4.496742	-0.842958
C	-2.900721	-2.686842	-2.254238
C	-2.808954	-5.413684	-1.636908
H	-1.536671	-4.845694	0.003786
C	-3.587604	-3.605945	-3.044602
H	-2.900140	-1.630816	-2.500959
C	-3.545784	-4.968224	-2.737860
H	-2.758473	-6.474084	-1.405941
H	-4.146904	-3.260183	-3.909351
H	-4.075108	-5.683091	-3.361940
C	-0.100748	1.277038	-1.123682
C	-1.371231	1.841865	-0.947534
C	0.916053	2.125721	-1.586437
C	-1.619990	3.188011	-1.229072
H	-2.181695	1.213176	-0.602247
C	0.680420	3.477684	-1.852885
H	1.911077	1.728856	-1.762729
C	-0.594152	4.018024	-1.682599
H	-0.786415	5.059472	-1.917227
C	1.806612	4.378429	-2.280742
C	-2.987053	3.773036	-1.020209
F	1.387544	5.339495	-3.135713
F	2.360021	5.029309	-1.219917
F	-3.345185	4.611363	-2.016029
F	2.805141	3.703483	-2.884169
F	-3.061284	4.497240	0.130868
F	-3.953061	2.821456	-0.929369
C	-2.395728	-2.029681	1.417155
H	-1.620684	-1.529523	1.990322
H	-2.515076	-3.095019	1.574928
C	-3.538432	-1.272946	1.143237
O	-3.479780	0.045879	1.260437
Si	-3.898671	1.073851	2.603691
C	-2.538324	2.332758	2.817861
H	-2.712098	2.899413	3.742128
H	-2.512145	3.048565	1.991519
H	-1.564332	1.840157	2.886205
C	-5.534497	1.883190	2.155700
H	-6.339619	1.152976	2.018688
H	-5.841936	2.575531	2.949630
H	-5.433896	2.463789	1.232015
C	-4.058827	-0.056355	4.099821
H	-4.816422	-0.835136	3.953784
H	-3.106925	-0.544183	4.337114
H	-4.356118	0.530557	4.977545
C	-4.775284	-1.824132	0.573366
C	-5.626590	-0.981643	-0.170800
C	-5.130495	-3.177040	0.738616
C	-6.793141	-1.483288	-0.738160
H	-5.344107	0.054443	-0.319311
C	-6.304884	-3.669799	0.179166
H	-4.502640	-3.837772	1.326038
C	-7.136614	-2.826557	-0.562147
H	-7.433652	-0.828352	-1.321631
H	-6.573751	-4.712051	0.323046
H	-8.051553	-3.215497	-1.000413

TS 214

B3LYP/6-31G(d) = -3573.919305
B3LYP-D3(BJ)/def2-TZVPP/IEFPCM(propanonitrile) = -3575.497695
B3LYP-D3(BJ)/def2-TZVPP/IEFPCM(propanonitrile)//B3LYP-D3(BJ)/6-31G(d) Free Energy (Quasiharmonic) = -3574.782024

Frequencies (Top 3 out of 300)

1. -247.5803 cm^{-1}
2. 4.8110 cm^{-1}
3. 13.1088 cm^{-1}

B3LYP/6-31G(d) Molecular Geometry in Cartesian Coordinates

B	-0.620270	-0.749869	0.702970
O	-0.370660	-2.056619	1.360480
N	-0.311470	-1.104039	-0.787380
C	-0.179130	-2.562579	-0.937220
S	-0.810870	-0.200209	-2.089860
C	-0.087389	-3.046269	0.514060
O	0.171471	-4.182279	0.845950
H	0.671801	-2.946419	-2.869690
H	-1.114999	-2.994969	-1.330450
C	0.954351	-3.121679	-1.829250
C	2.354890	-2.611169	-1.626660
C	3.388351	-3.146449	-0.764930
C	4.584370	-2.424759	-1.041860
N	4.273900	-1.470879	-1.994960
C	2.943140	-1.608829	-2.357350
H	2.496310	-0.972469	-3.108090
C	5.806031	-3.739989	0.530450
C	3.427211	-4.174569	0.195040
H	6.706290	-2.162159	-0.646900
H	4.948480	-0.918659	-2.501240
C	4.631031	-4.459269	0.829490
H	6.734541	-3.994829	1.034800
H	2.522871	-4.719739	0.446600
C	5.798670	-2.711329	-0.407340
H	4.668561	-5.252009	1.571660
C	-2.487380	-0.708980	-2.501060
O	0.020590	-0.564339	-3.250620
O	-0.866770	1.192741	-1.631240
C	-5.104940	-1.582550	-3.003590
C	-3.569760	0.020840	-2.001430
C	-2.700530	-1.845380	-3.285030
C	-4.006170	-2.275590	-3.525670
C	-4.864490	-0.419060	-2.257370
H	-3.392400	0.918830	-1.421290
H	-1.858060	-2.372739	-3.719920
H	-4.171089	-3.165630	-4.127980
H	-5.704810	0.139850	-1.854140
C	-6.517220	-2.072290	-3.213840
H	-6.974990	-2.341980	-2.254070
H	-6.547079	-2.953170	-3.862330
H	-7.147630	-1.297180	-3.666070
O	0.380990	0.243321	1.319000
C	1.669400	0.059781	1.280710
H	2.100520	-0.290899	0.337960
H	0.933681	-4.205899	-1.665950
C	2.351480	-0.359399	2.522460
C	3.604010	-0.989889	2.449370
C	1.732010	-0.183619	3.770680
C	4.229440	-1.435079	3.611420
H	4.069630	-1.163529	1.483520
C	2.366750	-0.619959	4.929070
H	0.749490	0.274681	3.812390
C	3.616800	-1.243429	4.852350
H	5.185240	-1.945829	3.542820
H	1.883180	-0.487839	5.892860
H	4.105200	-1.591829	5.758430
C	-2.082430	-0.186440	1.089530
C	-3.163660	-1.067230	1.248870
C	-2.336500	1.176150	1.283830
C	-4.443480	-0.605790	1.566250
H	-3.007740	-2.135070	1.133050
C	-3.611260	1.644990	1.616290
H	-1.525170	1.883551	1.166970
C	-4.676690	0.757130	1.756340
H	-5.664750	1.116150	2.020740
C	-3.838370	3.122640	1.755430
C	-5.603660	-1.559740	1.632960
F	-3.984150	3.727420	0.547340
F	-2.791880	3.743430	2.361210
F	-5.219599	-2.821600	1.912470
F	-4.944110	3.414430	2.472790
F	-6.277150	-1.607790	0.450720
F	-6.508940	-1.191850	2.569170
C	2.514900	2.030111	1.227170
H	3.495480	1.740181	1.580930
H	1.832080	2.387341	1.986480
C	2.430710	2.552291	-0.060240
O	3.417020	2.329661	-0.937560
Si	5.112010	2.600921	-0.869700
C	5.621260	2.458291	-2.670300
H	6.706460	2.567861	-2.783070
H	5.141700	3.231961	-3.279960
H	5.337970	1.487171	-3.092220
C	5.376030	4.333691	-0.190260
H	4.988890	4.432061	0.830400
H	6.445540	4.575501	-0.160920
H	4.879410	5.087271	-0.811560
C	5.980710	1.310311	0.185940
H	7.065920	1.384201	0.037340
H	5.792420	1.440891	1.257240
H	5.675410	0.293851	-0.084240
C	1.294540	3.306471	-0.589730
C	1.232410	3.600531	-1.966290
C	0.275280	3.793761	0.253270
C	0.184450	4.352781	-2.480940
H	2.000270	3.211901	-2.624030
C	-0.773190	4.543671	-0.266000
H	0.302690	3.600701	1.319970
C	-0.820470	4.824761	-1.633880
H	0.141870	4.559391	-3.546270
H	-1.557590	4.897931	0.393180
H	-1.643220	5.408191	-2.037720

TS 215

B3LYP/6-31G(d) = -3573.927076
B3LYP-D3(BJ)/def2-TZVPP/IEFPCM(propanonitrile) = -3575.498999
B3LYP-D3(BJ)/def2-TZVPP/IEFPCM(propanonitrile)//B3LYP-D3(BJ)/6-31G(d) Free Energy (Quasiharmonic) = -3574.781861

Frequencies (Top 3 out of 300)

1. -297.0414 cm^{-1}
2. 11.5033 cm^{-1}
3. 12.2755 cm^{-1}

B3LYP/6-31G(d) Molecular Geometry in Cartesian Coordinates

B	0.285876	-0.441242	-0.050353
O	-0.198169	0.652682	-0.940272
N	0.764258	-1.467619	-1.111226
C	0.592691	-0.955510	-2.475616
S	1.611862	-2.853877	-0.843247
C	0.090874	0.464719	-2.225293

O	-0.046621	1.322116	-3.078184
H	0.010669	-2.743458	-3.524690
H	1.557356	-0.885992	-2.998735
C	-0.392432	-1.735767	-3.394574
C	-1.805336	-1.795637	-2.891650
C	-2.900182	-0.923019	-3.250105
C	-4.029535	-1.331925	-2.486772
N	-3.635944	-2.412848	-1.724063
C	-2.300267	-2.675782	-1.959965
H	-1.790194	-3.480142	-1.448502
C	-5.377716	0.372316	-3.469257
C	-3.043348	0.154805	-4.142882
H	-6.110939	-1.002866	-1.967466
H	-4.187008	-2.825712	-0.987794
C	-4.276295	0.786888	-4.247132
H	-6.327726	0.891351	-3.566955
H	-2.194458	0.502371	-4.724386
C	-5.269284	-0.689037	-2.577963
H	-4.395311	1.619937	-4.935236
C	3.256515	-2.575551	-1.525670
O	1.029046	-3.955532	-1.626651
O	1.759735	-2.986304	0.613363
C	5.823719	-2.141115	-2.565435
C	3.591230	-3.091203	-2.777546
C	4.190745	-1.850695	-0.781168
C	5.462418	-1.638965	-1.305116
C	4.870018	-2.866096	-3.289854
H	2.863527	-3.676405	-3.329467
H	3.922306	-1.469563	0.197611
H	6.190893	-1.079425	-0.722332
H	5.130934	-3.268183	-4.265912
C	7.217402	-1.929706	-3.108118
H	7.250674	-2.067499	-4.193454
H	7.925734	-2.642086	-2.664841
H	7.587950	-0.923685	-2.881698
H	-0.359671	-1.240313	-4.372211
C	-0.872634	-0.916645	0.981110
C	-0.652703	-1.808911	2.044191
C	-2.184543	-0.441410	0.815845
C	-1.681287	-2.176079	2.919992
H	0.327734	-2.255922	2.170380
C	-3.220006	-0.828439	1.670174
H	-2.398472	0.229495	-0.007472
C	-2.973012	-1.688945	2.741561
H	-3.770130	-1.976531	3.417112
C	-4.628869	-0.380599	1.410529
C	-1.359171	-3.059856	4.094085
F	-4.686838	0.787081	0.726012
F	-5.330850	-0.209199	2.552617
F	-5.323348	-1.293979	0.673063
F	-0.699291	-2.370543	5.061799
F	-0.565057	-4.093140	3.748389
F	-2.469574	-3.574076	4.670021
O	1.510208	0.153735	0.641388
C	1.615694	0.521847	1.894609
C	3.011254	0.745387	2.367369
C	4.024469	1.121692	1.473468
C	3.319480	0.567811	3.723855
C	5.325493	1.317172	1.935235
H	3.782659	1.246651	0.423559
C	4.622261	0.758646	4.181008
H	2.539326	0.264333	4.418542
C	5.627655	1.136895	3.287581
H	6.106020	1.610259	1.238129
H	4.854533	0.604277	5.230961
H	6.643551	1.285129	3.643445
H	0.964399	0.013903	2.607512
C	0.749525	2.290424	2.348671
H	-0.088409	1.976304	2.965479
H	1.598569	2.682832	2.893385
C	0.383174	3.010416	1.191497
C	1.380371	3.724880	0.374506
C	1.308442	3.666087	-1.030789
C	2.435818	4.431419	0.982131
C	2.276349	4.302705	-1.803740
H	0.537616	3.077431	-1.516509
C	3.382278	5.088401	0.200948
H	2.494315	4.497330	2.063968
C	3.306526	5.022141	-1.192820
H	2.225206	4.227205	-2.885841
H	4.179232	5.650269	0.679305
H	4.052309	5.526939	-1.800851
O	-0.856029	2.937426	0.791258
Si	-2.089347	3.940900	0.063163
C	-2.699416	3.082696	-1.476960
H	-1.889764	2.750879	-2.134374
H	-3.304475	2.204398	-1.229956
H	-3.341124	3.769411	-2.044304
C	-1.389919	5.657237	-0.264615
H	-0.679325	5.676771	-1.095605
H	-2.222370	6.325564	-0.520529
H	-0.892779	6.078564	0.616661
C	-3.397299	4.009352	1.408102
H	-3.808627	3.013748	1.601904
H	-3.001020	4.410278	2.347991
H	-4.227833	4.654553	1.094749

TS 216

B3LYP/6-31G(d) = -3573.92302
B3LYP-D3(BJ)/def2-TZVPP/IEFPCM(propanonitrile) = -3575.500021
B3LYP-D3(BJ)/def2-TZVPP/IEFPCM(propanonitrile)//B3LYP-D3(BJ)/6-31G(d) Free Energy (Quasiharmonic) = -3574.78178

Frequencies (Top 3 out of 300)

1. -290.5968 cm^{-1}
2. 12.1456 cm^{-1}
3. 15.5831 cm^{-1}

B3LYP/6-31G(d) Molecular Geometry in Cartesian Coordinates

B	0.054030	1.077670	0.369661
O	-0.137279	1.914110	1.573511
N	-0.818400	1.829230	-0.677449
C	-1.296099	3.111170	-0.114959
S	-0.632000	1.734020	-2.328949
C	-0.845749	3.027960	1.345561
O	-1.093559	3.850980	2.199121
H	-3.061279	3.588091	-1.245989
H	-0.768139	3.966280	-0.560539
C	-2.813089	3.420301	-0.197029
C	-3.732999	2.384681	0.390371
C	-4.664490	1.551711	-0.333149
C	-5.319530	0.719981	0.617741
N	-4.830060	1.060411	1.861191
C	-3.873389	2.051051	1.717471
H	-3.375969	2.464021	2.584301
C	-6.603080	-0.322328	-1.100229
C	-5.018130	1.436171	-1.691069
H	-6.756350	-0.864498	0.988831
H	-4.990020	0.537361	2.708541
C	-5.980790	0.504032	-2.059889
H	-7.348920	-1.045168	-1.420389
H	-4.529229	2.052531	-2.440569
C	-6.282090	-0.225898	0.248601
H	-6.258970	0.406152	-3.105919
C	-0.912850	-0.002250	-2.683479
O	-1.695799	2.546640	-2.939629
O	0.759421	2.020220	-2.746549
C	-1.340341	-2.701180	-3.292609

C	-2.126110	-0.601130	-2.340439
C	0.075770	-0.722230	-3.355229
C	-0.144661	-2.065750	-3.653269
C	-2.329001	-1.944050	-2.647119
H	-2.896040	-0.034479	-1.829949
H	1.002430	-0.230400	-3.628759
H	0.629709	-2.632640	-4.164409
H	-3.271141	-2.411079	-2.371409
C	-1.537541	-4.176190	-3.548369
H	-1.123101	-4.767120	-2.721029
H	-2.598401	-4.431639	-3.636549
H	-1.030511	-4.497800	-4.464449
H	-2.934639	4.377921	0.324531
C	-0.367320	-0.458590	0.675451
C	-1.484900	-0.722600	1.482391
C	0.322219	-1.565850	0.165141
C	-1.899651	-2.027770	1.761211
C	-0.086531	-2.875470	0.438201
H	1.189120	-1.403641	-0.463959
C	-1.201631	-3.118170	1.239711
H	-1.519071	-4.131790	1.455471
C	0.724669	-4.027820	-0.076899
C	-3.142061	-2.263989	2.574441
F	1.776949	-4.320531	0.745171
F	0.001428	-5.164930	-0.188589
F	1.268099	-3.780591	-1.293009
F	-3.339010	-1.290749	3.502181
F	-4.258581	-2.287979	1.807051
F	-3.101551	-3.441719	3.237711
O	1.538960	1.003679	-0.003039
C	2.450570	1.811119	-0.467459
C	2.463571	3.285899	-0.274199
C	2.844701	4.072829	-1.374069
C	2.143231	3.914029	0.940291
C	2.881282	5.461359	-1.268439
H	3.065151	3.592379	-2.322479
C	2.181382	5.302599	1.042021
H	1.856641	3.319249	1.799191
C	2.550322	6.078809	-0.060149
H	3.156872	6.060839	-2.131389
H	1.914302	5.777019	1.981521
H	2.575442	7.161969	0.022041
H	2.873230	1.493279	-1.421319
C	4.194970	1.339519	0.557291
H	4.889410	1.863948	-0.086949
H	3.985930	1.827819	1.500581
C	4.145690	-0.054011	0.518321
C	4.596970	-0.852012	-0.635759
C	4.172659	-2.188641	-0.762359
C	5.430040	-0.307642	-1.633119
C	4.552949	-2.949192	-1.863589
H	3.531659	-2.618611	-0.003699
C	5.818830	-1.077152	-2.725069
H	5.793620	0.711238	-1.551759
C	5.377939	-2.397932	-2.846369
H	4.193409	-3.969521	-1.954939
H	6.467140	-0.646532	-3.482579
H	5.677999	-2.993672	-3.703959
O	3.514730	-0.741821	1.447781
Si	3.293720	-0.646881	3.170691
C	2.568200	0.987919	3.737491
H	2.307090	0.893089	4.800251
H	3.267840	1.827229	3.656261
H	1.648770	1.241559	3.198511
C	5.014680	-0.924962	3.879491
H	4.983060	-0.911742	4.975861
H	5.423819	-1.892662	3.568541
H	5.715710	-0.144282	3.561541
H	-2.042840	0.107630	1.900721
C	2.122649	-2.063031	3.520551
H	2.123989	-2.301881	4.591241
H	1.098349	-1.800511	3.236391
H	2.397569	-2.967531	2.968991

TS 217

B3LYP/6-31G(d) = -3573.929355
B3LYP-D3(BJ)/def2-TZVPP/IEFPCM(propanonitrile) = -3575.498514
B3LYP-D3(BJ)/def2-TZVPP/IEFPCM(propanonitrile)//B3LYP-D3(BJ)/6-31G(d) Free Energy (Quasiharmonic) = -3574.78175

Frequencies (Top 3 out of 300)

1. -273.3281 cm^{-1}
2. 7.8535 cm^{-1}
3. 12.5726 cm^{-1}

B3LYP/6-31G(d) Molecular Geometry in Cartesian Coordinates

B	-0.434610	0.244110	-0.297020
O	0.193980	-1.091410	-0.153400
N	-1.043650	0.123450	-1.711200
C	-0.826610	-1.214550	-2.274300
S	-2.008430	1.214940	-2.483390
C	-0.129100	-1.938480	-1.126050
O	0.137439	-3.125830	-1.109070
H	-0.487460	-0.778580	-4.359450
H	-1.780510	-1.724660	-2.472370
C	0.044810	-1.304420	-3.563520
C	1.436030	-0.759300	-3.416370
C	2.635570	-1.484010	-3.059740
C	3.693630	-0.534160	-2.993580
N	3.159550	0.696080	-3.320030
C	1.805490	0.557230	-3.552660
H	1.191380	1.410240	-3.805900
C	5.255560	-2.231691	-2.388520
C	2.926059	-2.834980	-2.792760
H	5.787020	-0.142301	-2.582210
H	3.639810	1.575150	-3.205070
C	4.227169	-3.194961	-2.463120
H	6.262919	-2.542871	-2.123990
H	2.137939	-3.581100	-2.821790
C	5.002900	-0.890371	-2.651630
H	4.459289	-4.237001	-2.257670
C	-3.662990	0.500901	-2.503250
O	-1.599800	1.320730	-3.893180
O	-2.061320	2.412520	-1.636190
C	-6.239740	-0.608169	-2.508070
C	-4.533600	0.768231	-1.444170
C	-4.069720	-0.300899	-3.570610
C	-5.350340	-0.855029	-3.561200
C	-5.810920	0.213751	-1.454440
H	-4.210650	1.417101	-0.637660
H	-3.400540	-0.465999	-4.408400
H	-5.665890	-1.480509	-4.392870
H	-6.493580	0.430381	-0.635470
C	-7.638390	-1.178299	-2.522150
H	-8.358080	-0.447849	-2.914950
H	-7.971420	-1.450029	-1.514490
H	-7.701700	-2.070509	-3.153190
H	0.078560	-2.366610	-3.833810
C	0.629400	1.434860	-0.018970
C	2.008260	1.173570	-0.065880
C	0.239410	2.751100	0.278920
C	2.951710	2.178220	0.172210
H	2.351720	0.169360	-0.287550
C	1.178940	3.755890	0.528420
H	-0.815060	3.001970	0.304390
C	2.543200	3.477210	0.475560
H	3.272130	4.252230	0.681630
C	0.708341	5.155160	0.819710

C	4.420390	1.882959	0.061540
F	-0.403779	5.157650	1.592130
F	0.402301	5.834060	-0.307540
F	1.648731	5.880250	1.471900
F	4.892870	2.126139	-1.199070
F	4.715420	0.596109	0.338430
F	5.160810	2.654939	0.889030
O	-1.584940	0.280940	0.738200
C	-1.399580	0.583610	1.989810
C	-2.564770	1.139840	2.723730
C	-2.348240	2.001310	3.808130
C	-3.877300	0.836941	2.332070
C	-3.428730	2.559681	4.489400
H	-1.331820	2.250870	4.104390
C	-4.954310	1.390581	3.019500
H	-4.037430	0.174761	1.487450
C	-4.733450	2.252381	4.098400
H	-3.252420	3.238631	5.318750
H	-5.969690	1.154281	2.713200
H	-5.576270	2.686361	4.629110
H	-0.427010	0.999600	2.257980
C	-1.126140	-1.080460	3.233740
H	-2.153060	-1.428950	3.254710
H	-0.820220	-0.461910	4.068790
C	-0.186760	-1.981530	2.718230
C	1.266200	-1.821490	2.905830
C	2.158890	-2.234540	1.897070
C	1.782610	-1.223630	4.072760
C	3.527720	-2.026470	2.043370
H	1.767199	-2.655380	0.980270
C	3.153750	-1.039730	4.222080
H	1.114110	-0.932730	4.877280
C	4.027800	-1.430930	3.203770
H	4.199090	-2.310761	1.238590
H	3.541420	-0.589310	5.131320
H	5.096260	-1.268081	3.313700
O	-0.635531	-2.954440	1.954740
Si	-0.621681	-4.682460	1.908040
C	-1.013811	-5.214500	3.673540
H	-0.232971	-4.896840	4.374850
H	-1.967791	-4.800940	4.019960
H	-1.083271	-6.307610	3.734940
C	1.019999	-5.388110	1.341800
H	1.844249	-5.150310	2.021800
H	0.937489	-6.481800	1.287450
H	1.267879	-5.015240	0.343640
C	-2.001341	-5.088380	0.711140
H	-2.130171	-6.173930	0.620330
H	-2.956381	-4.660339	1.035160
H	-1.758651	-4.689100	-0.278700

TS 218

B3LYP/6-31G(d) = -3573.924234
B3LYP-D3(BJ)/def2-TZVPP/IEFPCM(propanonitrile) = -3575.497373
B3LYP-D3(BJ)/def2-TZVPP/IEFPCM(propanonitrile)//B3LYP-D3(BJ)/6-31G(d) Free Energy (Quasiharmonic) = -3574.781604

Frequencies (Top 3 out of 300)

1. -208.6205 cm^{-1}
2. 10.6257 cm^{-1}
3. 11.2753 cm^{-1}

B3LYP/6-31G(d) Molecular Geometry in Cartesian Coordinates

B	0.072431	1.071125	-0.015010
O	0.747140	2.293905	-0.477598
N	0.558515	0.994570	1.465284
C	1.412084	2.153693	1.785512
S	-0.104138	0.100998	2.685274
C	1.363007	2.977051	0.498113
O	1.834326	4.082008	0.363976
H	2.888751	1.244832	3.076545
H	0.971862	2.742039	2.597920
C	2.889864	1.844780	2.160889
C	3.709498	1.177309	1.091621
C	4.561597	1.822383	0.114835
C	5.114442	0.797259	-0.700523
N	4.642400	-0.408693	-0.220970
C	3.785876	-0.170728	0.836344
H	3.300098	-0.994765	1.338863
C	6.309497	2.395671	-2.001709
C	4.916376	3.158135	-0.149304
H	6.367383	0.266108	-2.390515
H	4.718492	-1.284544	-0.714863
C	5.786187	3.429539	-1.197435
H	6.977802	2.642558	-2.822509
H	4.488955	3.966115	0.435941
C	5.979397	1.066710	-1.766688
H	6.059750	4.459151	-1.412318
C	1.037795	-1.211958	3.151849
O	-1.286060	-0.575134	2.119201
O	-0.249008	0.958363	3.873504
C	2.810722	-3.254078	3.898518
C	1.064247	-2.404084	2.424017
C	1.867634	-1.038567	4.260963
C	2.750287	-2.055344	4.621905
C	1.952311	-3.410485	2.800158
H	0.390544	-2.553102	1.588104
H	1.805902	-0.123730	4.840385
H	3.398414	-1.916270	5.483813
H	1.972272	-4.338603	2.233673
C	3.747442	-4.363249	4.314308
H	4.656883	-3.966984	4.777655
H	3.270588	-5.026956	5.047858
H	4.041733	-4.981344	3.459880
H	3.338906	2.809591	2.424272
C	0.391023	-0.200721	-0.952140
C	-0.349691	-1.388061	-0.881585
C	1.491283	-0.179300	-1.827023
C	0.005777	-2.516931	-1.631144
C	1.840480	-1.299833	-2.585562
H	2.085414	0.724705	-1.907602
C	1.100493	-2.480488	-2.491520
H	1.375112	-3.352285	-3.074205
C	3.059421	-1.286124	-3.465162
C	-0.815787	-3.766136	-1.504232
F	2.859616	-1.967695	-4.616436
F	3.459579	-0.045028	-3.787122
F	4.124256	-1.896764	-2.854996
F	-0.213144	-4.846830	-2.040949
F	-2.026832	-3.650632	-2.121632
F	-1.088968	-4.059912	-0.207128
O	-1.451610	1.390173	-0.165192
C	-2.270147	1.923083	0.681375
C	-2.494761	3.378995	0.684464
C	-3.194249	3.966892	1.753995
C	-1.959636	4.194882	-0.327420
C	-3.367456	5.345664	1.804766
H	-3.584261	3.337844	2.551039
C	-2.134674	5.575314	-0.270073
H	-1.377136	3.740117	-1.120732
C	-2.841962	6.151251	0.788665
H	-3.900186	5.795866	2.637494
H	-1.704787	6.204676	-1.043656
H	-2.972154	7.229144	0.831205
H	-2.432119	1.394625	1.623196
C	-4.303083	1.369764	0.007168
H	-4.810108	1.702508	0.902430
H	-4.316559	2.064793	-0.822467

C	-4.190714	0.014248	-0.256212
C	-4.380166	-1.044654	0.751463
C	-4.016510	-2.368044	0.441609
C	-4.916385	-0.771746	2.024258
C	-4.167161	-3.381919	1.382436
H	-3.602134	-2.590985	-0.532882
C	-5.075160	-1.789756	2.957455
H	-5.219415	0.235032	2.291355
C	-4.697630	-3.097739	2.641717
H	-3.858453	-4.392276	1.131191
H	-5.490290	-1.562636	3.935053
H	-4.815512	-3.889535	3.376332
O	-3.793891	-0.427397	-1.443108
Si	-3.893712	0.181407	-3.060707
C	-5.648000	0.815243	-3.326469
H	-5.781093	1.096605	-4.378701
H	-6.387668	0.039475	-3.097370
H	-5.884587	1.695511	-2.719718
C	-3.564711	-1.348652	-4.090205
H	-4.386678	-2.068276	-4.003187
H	-3.457649	-1.087850	-5.150015
H	-2.649143	-1.854374	-3.769607
H	-1.198656	-1.441152	-0.208694
C	-2.608220	1.515429	-3.353501
H	-2.825974	2.438647	-2.806738
H	-1.611990	1.178886	-3.050556
H	-2.574246	1.762634	-4.422384

TS 219

B3LYP/6-31G(d) = -3573.924825
B3LYP-D3(BJ)/def2-TZVPP/IEFPCM(propanonitrile) = -3575.498007
B3LYP-D3(BJ)/def2-TZVPP/IEFPCM(propanonitrile)//B3LYP-D3(BJ)/6-31G(d) Free Energy (Quasiharmonic) = -3574.781601

Frequencies (Top 3 out of 300)

1. -233.1682 cm^{-1}
2. 7.4774 cm^{-1}
3. 12.4813 cm^{-1}

B3LYP/6-31G(d) Molecular Geometry in Cartesian Coordinates

B	0.025212	-0.147298	-1.051558
O	0.046236	-0.388083	-2.508292
N	0.567154	1.330546	-0.984359
C	0.905955	1.806275	-2.340182
S	0.144698	2.429685	0.167964
C	0.434590	0.659979	-3.238030
O	0.458037	0.679488	-4.448561
H	2.741736	2.905353	-1.976043
H	0.328709	2.704429	-2.591654
C	2.408571	2.095927	-2.632615
C	3.343811	0.919596	-2.508438
C	4.399329	0.742293	-1.538447
C	4.990575	-0.528636	-1.782329
N	4.351858	-1.070984	-2.877696
C	3.361114	-0.206399	-3.300165
H	2.741201	-0.458496	-4.148836
C	6.514082	-0.234357	0.026777
C	4.921724	1.535914	-0.502659
H	6.446344	-2.024207	-1.188292
H	4.438316	-2.029821	-3.178731
C	5.969136	1.044079	0.266043
H	7.324923	-0.596908	0.653027
H	4.514290	2.522062	-0.303441
C	6.034612	-1.036178	-1.001971
H	6.375936	1.652647	1.069762
C	1.610530	3.292840	0.743565
O	-0.740374	3.457319	-0.419527
O	-0.341223	1.654158	1.327227
C	3.806050	4.702121	1.760902
C	2.383327	2.722888	1.758273
C	1.914207	4.556411	0.234430
C	3.015291	5.246739	0.739383
C	3.471222	3.434057	2.258697
H	2.134609	1.745575	2.157576
H	1.282710	4.997462	-0.529422
H	3.255152	6.230113	0.342441
H	4.073221	2.992864	3.049022
C	4.969877	5.474527	2.334840
H	5.776692	4.806023	2.652653
H	5.379997	6.181731	1.606886
H	4.661048	6.053152	3.215603
H	2.428198	2.494339	-3.654905
C	0.877118	-1.236822	-0.228867
C	0.968740	-1.213559	1.167085
C	1.616584	-2.222948	-0.901610
C	1.771772	-2.121477	1.865329
C	2.434910	-3.118844	-0.211826
H	1.566666	-2.271120	-1.982766
C	2.518023	-3.077511	1.182866
H	3.160526	-3.765558	1.720704
C	3.246089	-4.150701	-0.943400
C	1.805215	-2.029918	3.362770
F	4.544308	-4.138786	-0.552924
F	3.241290	-3.963400	-2.289229
F	2.792770	-5.406711	-0.724764
F	2.100432	-0.775475	3.783976
F	2.704546	-2.864506	3.922079
F	0.593406	-2.331721	3.913492
O	-1.459282	-0.334656	-0.643821
C	-2.424270	0.389965	-1.137962
C	-3.510897	-0.276353	-1.877338
C	-4.354748	0.488067	-2.699286
C	-3.671441	-1.669970	-1.827093
C	-5.344756	-0.130393	-3.458241
H	-4.221302	1.565700	-2.753102
C	-4.666382	-2.283309	-2.583945
H	-2.995461	-2.258505	-1.217003
C	-5.504333	-1.516950	-3.399185
H	-5.986096	0.466528	-4.100188
H	-4.779849	-3.363351	-2.550879
H	-6.273203	-2.000658	-3.995453
H	-2.162568	1.395729	-1.478005
C	-3.350292	1.314087	0.567431
H	-3.538074	2.274303	0.102211
H	-2.428462	1.258731	1.130559
C	-4.446922	0.550881	0.949599
C	-5.813731	0.800469	0.447795
C	-6.196571	2.059767	-0.051507
C	-6.768159	-0.233235	0.476043
C	-7.491610	2.273905	-0.514784
H	-5.493161	2.885436	-0.049240
C	-8.057811	-0.019095	0.000292
H	-6.480636	-1.204154	0.861435
C	-8.424325	1.234519	-0.495760
H	-7.774943	3.255242	-0.884347
H	-8.780078	-0.830318	0.017832
H	-9.434274	1.402885	-0.859462
O	-4.354005	-0.523805	1.713048
Si	-3.274129	-1.266474	2.857548
C	-2.187577	0.004363	3.705590
H	-2.784958	0.714574	4.289225
H	-1.548014	0.573200	3.025983
H	-1.524953	-0.526433	4.401489
C	-2.327339	-2.588612	1.930647
H	-2.996614	-3.384495	1.583855
H	-1.805829	-2.169511	1.065423
H	-1.571160	-3.037501	2.584762
H	0.422324	-0.458355	1.720888

C	-4.481992	-2.021476	4.083802
H	-5.082517	-1.253033	4.583608
H	-5.168492	-2.720860	3.593329
H	-3.940132	-2.577092	4.859196

TS 220

B3LYP/6-31G(d) = -3573.920548
B3LYP-D3(BJ)/def2-TZVPP/IEFPCM(propanonitrile) = -3575.49798
B3LYP-D3(BJ)/def2-TZVPP/IEFPCM(propanonitrile)//B3LYP-D3(BJ)/6-31G(d) Free Energy (Quasiharmonic) = -3574.781414

Frequencies (Top 3 out of 300)

1. -299.2835 cm^{-1}
2. 8.9094 cm^{-1}
3. 12.0212 cm^{-1}

B3LYP/6-31G(d) Molecular Geometry in Cartesian Coordinates

B	0.500756	0.364250	-0.096837
O	0.129715	-0.277908	-1.390103
N	0.746100	1.829209	-0.562571
C	0.510204	1.972382	-2.005238
S	0.712754	3.171401	0.393655
C	0.046580	0.576256	-2.412588
O	-0.356555	0.258609	-3.514562
H	1.953098	3.452496	-2.598582
H	-0.321970	2.661047	-2.214907
C	1.722191	2.419422	-2.868507
C	2.946983	1.559250	-2.735225
C	4.106680	1.814974	-1.912369
C	5.001570	0.727161	-2.098692
N	4.418219	-0.133931	-3.007559
C	3.180615	0.363058	-3.372377
H	2.559417	-0.170260	-4.079733
C	6.571378	1.691478	-0.588260
C	4.476958	2.857462	-1.042813
H	6.893740	-0.203703	-1.581791
H	4.752813	-1.063368	-3.208336
C	5.702829	2.785889	-0.394330
H	7.519881	1.661330	-0.058278
H	3.803854	3.690238	-0.863925
C	6.233537	0.647319	-1.440087
H	5.995490	3.581777	0.285268
C	-1.012150	3.697311	0.547907
O	1.376690	4.266746	-0.329111
O	1.145021	2.768165	1.734729
C	-3.680065	4.541825	0.814541
C	-1.763278	3.286277	1.652666
C	-1.574181	4.542573	-0.410233
C	-2.903810	4.948143	-0.277056
C	-3.083400	3.714176	1.780867
H	-1.297503	2.665531	2.409687
H	-0.965038	4.910060	-1.229159
H	-3.335732	5.609843	-1.023946
H	-3.653396	3.425023	2.662202
C	-5.116689	4.986083	0.961582
H	-5.812867	4.157106	0.776182
H	-5.362916	5.785002	0.255682
H	-5.318474	5.356599	1.973357
H	1.364548	2.422377	-3.905846
C	1.715014	-0.414754	0.625445
C	2.486779	-1.337767	-0.092505
C	2.032963	-0.231419	1.980869
C	3.523976	-2.057408	0.510136
H	2.270399	-1.507484	-1.142291
C	3.070993	-0.942696	2.586102
H	1.478085	0.493416	2.565486
C	3.823537	-1.864631	1.856370
H	4.624049	-2.419927	2.330318
C	3.332703	-0.778109	4.058227
C	4.311316	-3.036366	-0.312404
F	4.620675	-1.044130	4.378278
F	2.569491	-1.625533	4.797199
F	3.055618	0.468419	4.491827
F	5.016462	-3.902340	0.446041
F	5.209029	-2.428209	-1.137851
F	3.506879	-3.771853	-1.120456
O	-0.739659	0.372654	0.804648
C	-1.746439	-0.441349	0.997157
C	-1.635956	-1.926641	1.104130
C	-2.453481	-2.542693	2.068924
C	-0.758058	-2.723432	0.353869
C	-2.384036	-3.915770	2.293053
H	-3.132172	-1.936559	2.663931
C	-0.686394	-4.096593	0.585388
H	-0.140004	-2.275577	-0.410720
C	-1.494742	-4.696976	1.552837
H	-3.012314	-4.371224	3.053358
H	0.013662	-4.696045	0.010068
H	-1.428554	-5.766579	1.732202
H	-2.429183	-0.041066	1.752372
C	-2.957315	0.001363	-0.526807
H	-2.274064	-0.171166	-1.349098
H	-3.059382	1.040041	-0.234349
C	-4.099800	-0.810945	-0.487244
C	-5.202231	-0.615304	0.467862
C	-5.274031	0.529439	1.288022
C	-6.213012	-1.590824	0.575680
C	-6.325853	0.688027	2.185114
H	-4.519067	1.305579	1.221413
C	-7.260195	-1.427673	1.476565
H	-6.158866	-2.474848	-0.048345
C	-7.320671	-0.288543	2.283371
H	-6.371149	1.576456	2.808221
H	-8.030485	-2.189712	1.551268
H	-8.140103	-0.160939	2.985127
O	-4.194169	-1.898223	-1.224694
Si	-3.768930	-2.279986	-2.884039
C	-5.075034	-3.567077	-3.286939
H	-5.006183	-4.426010	-2.609579
H	-6.087386	-3.154758	-3.209659
H	-4.944361	-3.940874	-4.309681
C	-2.039627	-2.982463	-2.984177
H	-1.279144	-2.197751	-2.914274
H	-1.854771	-3.730258	-2.206779
H	-1.915755	-3.472801	-3.958820
C	-3.963097	-0.714319	-3.902034
H	-4.874413	-0.160203	-3.649710
H	-3.099464	-0.049905	-3.795697
H	-4.028655	-0.987931	-4.962871

TS 221

B3LYP/6-31G(d) = -3573.922923
B3LYP-D3(BJ)/def2-TZVPP/IEFPCM(propanonitrile) = -3575.499028
B3LYP-D3(BJ)/def2-TZVPP/IEFPCM(propanonitrile)//B3LYP-D3(BJ)/6-31G(d) Free Energy (Quasiharmonic) = -3574.781401

Frequencies (Top 3 out of 300)

1. -304.2809 cm^{-1}
2. 11.6813 cm^{-1}
3. 13.3858 cm^{-1}

B3LYP/6-31G(d) Molecular Geometry in Cartesian Coordinates

B	0.280212	0.875610	-0.834833
O	0.715503	0.674357	-2.225165

N	1.481271	1.677709	-0.259459
C	2.093851	2.412057	-1.390109
S	1.515116	2.305162	1.269604
C	1.640565	1.565901	-2.589612
O	2.045394	1.697280	-3.724394
H	3.786348	3.595883	-0.810096
H	1.606797	3.390642	-1.519450
C	3.617941	2.693085	-1.399857
C	4.548565	1.626027	-0.899360
C	4.951456	0.382399	-1.520561
C	5.886573	-0.239942	-0.643346
N	6.046125	0.595508	0.443470
C	5.243204	1.707321	0.282474
H	5.206874	2.480987	1.036783
C	6.148880	-2.082724	-2.133554
C	4.639170	-0.261040	-2.733737
H	7.199745	-1.924689	-0.248588
H	6.629330	0.410850	1.244146
C	5.237005	-1.481212	-3.024663
H	6.597849	-3.038698	-2.390146
H	3.942980	0.192430	-3.432151
C	6.488306	-1.469221	-0.933414
H	4.998843	-1.983071	-3.958763
C	2.157163	0.975123	2.304726
O	0.136223	2.521970	1.764768
O	2.443495	3.446332	1.295519
C	3.031633	-1.065949	4.017774
C	2.888313	-0.092373	1.787541
C	1.870129	1.044671	3.672415
C	2.311937	0.030568	4.516451
C	3.319607	-1.102719	2.648556
H	3.098944	-0.143564	0.726565
H	1.286089	1.871502	4.062950
H	2.079344	0.081199	5.577603
H	3.875910	-1.942076	2.238228
C	3.438470	-2.197255	4.931026
H	4.256637	-2.786079	4.504138
H	3.757411	-1.829467	5.912601
H	2.593761	-2.878601	5.097761
H	3.843344	2.940001	-2.445453
C	-0.113671	-0.522185	-0.126929
C	-0.535976	-0.654331	1.206950
C	-0.016208	-1.704078	-0.882081
C	-0.848489	-1.903033	1.755415
C	-0.329616	-2.951788	-0.338541
H	0.313027	-1.641715	-1.912735
C	-0.746688	-3.061593	0.987640
H	-0.986360	-4.030422	1.412549
C	-0.296073	-4.191694	-1.188406
C	-1.245166	-2.017901	3.199076
F	-1.545953	-4.538342	-1.612310
F	0.175822	-5.259569	-0.505156
F	0.460200	-4.046224	-2.293076
F	-0.203706	-2.328483	4.003112
F	-2.177603	-2.990730	3.386306
F	-1.778557	-0.866906	3.678299
O	-0.921158	1.859131	-1.008612
C	-1.936232	2.068638	-0.220953
C	-2.353642	3.486800	-0.030522
C	-2.034104	4.462383	-0.984628
C	-3.055616	3.856366	1.124459
C	-2.421075	5.785910	-0.788072
H	-1.468042	4.176755	-1.865575
C	-3.438362	5.181673	1.321369
H	-3.273005	3.109407	1.883867
C	-3.126788	6.148308	0.362593
H	-2.164757	6.538517	-1.528590
H	-3.968475	5.462376	2.227211
H	-3.423270	7.182271	0.516149
H	-2.036007	1.432172	0.658063
C	-3.631804	1.418454	-1.129978
H	-3.594756	2.093371	-1.975688
H	-4.344160	1.710665	-0.366725
C	-3.526739	0.045498	-1.404619
C	-2.878853	-0.489870	-2.601381
C	-2.223445	0.354579	-3.521208
C	-2.890916	-1.880156	-2.843110
C	-1.601209	-0.176950	-4.645603
H	-2.159717	1.420015	-3.341408
C	-2.280005	-2.403204	-3.974896
H	-3.360483	-2.540785	-2.125389
C	-1.635353	-1.552623	-4.879765
H	-1.067729	0.481226	-5.323697
H	-2.282534	-3.476224	-4.137658
H	-1.146348	-1.964936	-5.757943
O	-3.947555	-0.867097	-0.540782
Si	-5.179893	-0.943031	0.674753
C	-5.112840	-2.733976	1.213792
H	-5.940757	-2.958776	1.897508
H	-5.190954	-3.415459	0.359650
H	-4.178878	-2.952673	1.741942
C	-6.797201	-0.497027	-0.175854
H	-7.012926	-1.183177	-1.002571
H	-7.630210	-0.560651	0.534953
H	-6.785965	0.521688	-0.579260
H	-0.603838	0.221888	1.842163
C	-4.803202	0.202585	2.115725
H	-3.811129	0.010186	2.537236
H	-4.873213	1.264342	1.857501
H	-5.533920	0.015444	2.913612

TS 222

B3LYP/6-31G(d) = -3573.925793
B3LYP-D3(BJ)/def2-TZVPP/IEFPCM(propanonitrile) = -3575.498623
B3LYP-D3(BJ)/def2-TZVPP/IEFPCM(propanonitrile)//B3LYP-D3(BJ)/6-31G(d) Free Energy (Quasiharmonic) = -3574.781393

Frequencies (Top 3 out of 300)

1. -255.5930 cm^{-1}
2. 12.9854 cm^{-1}
3. 13.3856 cm^{-1}

B3LYP/6-31G(d) Molecular Geometry in Cartesian Coordinates

B	-0.410490	-1.037540	0.925940
O	-0.278361	-2.340590	1.627150
N	0.036580	-1.442710	-0.521020
C	0.123519	-2.912530	-0.629430
S	-0.299110	-0.570170	-1.886630
C	-0.022041	-3.372200	0.825440
O	0.073388	-4.523380	1.191120
H	1.460609	-3.121351	-2.306030
H	-0.751991	-3.303170	-1.172460
C	1.372829	-3.536251	-1.299140
C	2.690079	-3.418291	-0.579450
C	3.787669	-2.527812	-0.877800
C	4.864259	-2.878202	-0.012190
N	4.426879	-3.921262	0.775630
C	3.127349	-4.238701	0.434040
H	2.597138	-5.031951	0.941260
C	6.251150	-1.185723	-0.962500
C	3.972070	-1.473452	-1.792140
H	6.912709	-2.514903	0.613450
H	4.953038	-4.357172	1.516520
C	5.196520	-0.814512	-1.822720
H	7.199590	-0.657473	-1.018140
H	3.163320	-1.175131	-2.453300
C	6.099699	-2.222732	-0.047190
H	5.344900	0.002848	-2.522230

C	-1.909890	-1.070039	-2.509640
O	0.672050	-0.932290	-2.933610
O	-0.406390	0.835850	-1.456780
C	-4.436971	-1.919988	-3.375640
C	-2.014791	-2.172269	-3.361330
C	-3.050880	-0.366719	-2.112920
C	-4.300390	-0.793839	-2.549790
C	-3.277431	-2.590509	-3.783570
H	-1.120341	-2.680490	-3.705480
H	-2.953350	0.501521	-1.472120
H	-5.187890	-0.257498	-2.226110
H	-3.360171	-3.453429	-4.439640
C	-5.808261	-2.389788	-3.796920
H	-6.333170	-1.621288	-4.377720
H	-6.426561	-2.608908	-2.918040
H	-5.754421	-3.295318	-4.408690
O	0.548220	-0.075370	1.612200
C	1.835270	0.006669	1.446780
H	2.237770	-0.243171	0.462450
H	1.127018	-4.598921	-1.409600
C	2.692470	-0.299701	2.616740
C	4.076530	-0.468312	2.449240
C	2.121510	-0.465061	3.887730
C	4.876730	-0.793802	3.540760
H	4.521560	-0.358392	1.463970
C	2.927710	-0.788001	4.976900
H	1.048260	-0.359371	4.001150
C	4.305300	-0.949462	4.807470
H	5.945790	-0.930292	3.402300
H	2.480310	-0.921621	5.957820
H	4.931610	-1.202282	5.658940
C	-1.897820	-0.449679	1.166830
C	-3.007020	-1.309479	1.114980
C	-2.144320	0.889841	1.492210
C	-4.304030	-0.852649	1.357550
H	-2.861451	-2.362839	0.897460
C	-3.439359	1.350651	1.757760
H	-1.309669	1.579590	1.554260
C	-4.529130	0.484272	1.687470
H	-5.530520	0.837132	1.905880
C	-3.662879	2.797861	2.088980
C	-5.480291	-1.778568	1.214220
F	-4.802919	3.002222	2.781300
F	-2.646549	3.314751	2.823230
F	-6.046631	-1.680508	-0.020600
F	-3.749299	3.570421	0.969510
F	-6.459860	-1.492898	2.102400
F	-5.141811	-3.072448	1.383020
C	2.314131	2.081669	1.375770
H	3.376751	1.919309	1.493810
H	1.750971	2.318899	2.272240
C	1.906461	2.677379	0.184490
O	0.686221	3.164830	0.101810
Si	-0.078108	4.559820	-0.599200
C	1.147272	5.573119	-1.607600
H	0.672473	6.531170	-1.857470
H	1.437232	5.088219	-2.543710
H	2.061892	5.798579	-1.047950
C	-1.510338	3.939570	-1.629510
H	-2.293669	3.520151	-0.992030
H	-1.947488	4.769751	-2.198750
H	-1.196269	3.160640	-2.329610
C	-0.635838	5.547200	0.897300
H	-1.308548	4.966360	1.534740
H	0.218132	5.869610	1.504650
H	-1.174107	6.448610	0.577810
C	2.791531	2.722619	-1.002950
C	2.292441	2.408589	-2.279410
C	4.142341	3.086608	-0.861050
C	3.134731	2.459139	-3.388160
H	1.265851	2.074279	-2.380520
C	4.971031	3.162188	-1.978020
H	4.531461	3.346748	0.118920
C	4.469171	2.847688	-3.243450
H	2.747701	2.187459	-4.365910
H	6.007011	3.467388	-1.861180
H	5.118051	2.900458	-4.113530

TS 223

B3LYP/6-31G(d) = -3573.92926
B3LYP-D3(BJ)/def2-TZVPP/IEFPCM(propanonitrile) = -3575.499231
B3LYP-D3(BJ)/def2-TZVPP/IEFPCM(propanonitrile)//B3LYP-D3(BJ)/6-31G(d) Free Energy (Quasiharmonic) = -3574.781371

Frequencies (Top 3 out of 300)

1. -284.0123 cm^{-1}
2. 11.1643 cm^{-1}
3. 15.8427 cm^{-1}

B3LYP/6-31G(d) Molecular Geometry in Cartesian Coordinates

B	-0.337590	0.272230	-0.504970
O	-1.393411	-0.733329	-0.807200
N	0.271370	0.502980	-1.932710
C	-0.462651	-0.302990	-2.933470
S	0.799801	1.967569	-2.537790
C	-1.466081	-1.082349	-2.090550
O	-2.233522	-1.925708	-2.516050
H	0.983359	-0.635191	-4.484430
H	-1.048450	0.336311	-3.611560
C	0.379339	-1.256480	-3.820200
C	1.253968	-2.221881	-3.072440
C	2.675998	-2.109212	-2.854340
C	3.076957	-3.234052	-2.082790
N	1.952617	-4.005011	-1.870140
C	0.861407	-3.385871	-2.454290
H	-0.121303	-3.834510	-2.400580
C	5.341628	-2.489644	-2.079780
C	3.651439	-1.176013	-3.252100
H	4.684676	-4.289393	-1.074670
H	1.904686	-4.785031	-1.233390
C	4.971019	-1.375754	-2.862860
H	6.380608	-2.614745	-1.786360
H	3.371539	-0.308272	-3.843270
C	4.402307	-3.432533	-1.680240
H	5.733339	-0.662884	-3.166480
C	1.976601	2.538809	-1.315770
O	-0.304448	2.950810	-2.590770
O	1.509991	1.691469	-3.798340
C	3.871832	3.538047	0.485660
C	3.139981	1.805518	-1.064580
C	1.755072	3.768569	-0.697190
C	2.705173	4.257958	0.200000
C	4.072241	2.307907	-0.161970
H	3.308800	0.853558	-1.556750
H	0.853843	4.326949	-0.922560
H	2.536533	5.216938	0.684030
H	4.970431	1.732726	0.047530
C	4.881642	4.052136	1.482780
H	5.903572	3.966996	1.095960
H	4.834582	3.467566	2.408540
H	4.699713	5.101527	1.734790
H	-0.343812	-1.796840	-4.443090
C	0.659409	-0.288950	0.640300
C	1.339250	0.544849	1.541720
C	0.840218	-1.669561	0.795740
C	2.162280	0.023388	2.543010
H	1.221781	1.620149	1.469440
C	1.642868	-2.200621	1.810700

H	0.327088	-2.349300	0.124750
C	2.314739	-1.356112	2.691540
H	2.928748	-1.760892	3.487910
C	1.779657	-3.689941	1.944640
C	2.933190	0.938078	3.452100
F	2.161667	-4.064772	3.183820
F	2.692966	-4.214782	1.083890
F	0.609496	-4.331610	1.675740
F	2.999160	0.459798	4.716940
F	2.397991	2.177578	3.518000
F	4.219340	1.093097	3.036980
O	-0.987609	1.519781	0.084330
C	-2.077959	2.066772	-0.381380
C	-2.233068	3.527772	-0.171020
C	-1.568727	4.177801	0.879140
C	-3.050607	4.268372	-1.035410
C	-1.724226	5.550611	1.059080
H	-0.928478	3.602051	1.539510
C	-3.199026	5.641802	-0.857000
H	-3.548438	3.770093	-1.863540
C	-2.539526	6.285162	0.193250
H	-1.206266	6.049901	1.873380
H	-3.821196	6.211423	-1.541390
H	-2.656315	7.356262	0.333550
H	-2.479849	1.668922	-1.313870
C	-3.744929	1.506923	0.711280
H	-3.490279	2.017903	1.631680
H	-4.444479	2.019893	0.059400
C	-3.858750	0.112743	0.764100
C	-3.382831	-0.664238	1.924450
C	-2.711242	-1.888798	1.756780
C	-3.591001	-0.170517	3.227310
C	-2.245502	-2.591258	2.865280
H	-2.496992	-2.238508	0.756220
C	-3.150401	-0.892178	4.332740
H	-4.127600	0.762053	3.371510
C	-2.471122	-2.099948	4.153280
H	-1.676983	-3.504439	2.719340
H	-3.331581	-0.510378	5.333290
H	-2.110022	-2.652548	5.016240
O	-4.346861	-0.497237	-0.293240
Si	-5.414452	-1.800646	-0.709370
C	-5.816522	-1.413126	-2.495560
H	-4.898412	-1.448156	-3.092080
H	-6.516852	-2.149105	-2.908780
H	-6.265581	-0.420035	-2.606750
C	-4.613883	-3.487737	-0.554850
H	-3.667863	-3.496587	-1.105730
H	-4.434023	-3.789347	0.481290
H	-5.277204	-4.234396	-1.011220
C	-6.893462	-1.614845	0.441340
H	-7.650962	-2.373864	0.210000
H	-7.366271	-0.631505	0.338440
H	-6.607412	-1.746005	1.491370

TS 224

B3LYP/6-31G(d) = -3573.925221
B3LYP-D3(BJ)/def2-TZVPP/IEFPCM(propanonitrile) = -3575.499086
B3LYP-D3(BJ)/def2-TZVPP/IEFPCM(propanonitrile)//B3LYP-D3(BJ)/6-31G(d) Free Energy (Quasiharmonic) = -3574.781368

Frequencies (Top 3 out of 300)

1. -274.8252 cm^{-1}
2. 11.1469 cm^{-1}
3. 13.2812 cm^{-1}

B3LYP/6-31G(d) Molecular Geometry in Cartesian Coordinates

B	0.395780	0.251420	-0.173410
O	1.023600	-0.351480	1.027830
N	-0.184550	1.573700	0.429020
C	0.154490	1.680460	1.856630
S	-0.560200	2.945270	-0.414480
C	1.036340	0.458530	2.090730
O	1.661090	0.239820	3.107670
H	-1.639180	2.584340	2.646940
H	0.755280	2.575030	2.048040
C	-1.037840	1.693990	2.856970
C	-1.903410	0.466220	2.863600
C	-1.771070	-0.691880	3.721410
C	-2.798260	-1.604050	3.353840
N	-3.531950	-1.012030	2.344950
C	-2.975930	0.215500	2.042490
H	-3.399140	0.828490	1.259240
C	-2.075240	-3.170360	4.997640
C	-0.895480	-1.047360	4.763970
H	-3.734640	-3.537630	3.655690
H	-4.196230	-1.501580	1.765150
C	-1.058150	-2.275870	5.391420
H	-2.168960	-4.129160	5.500870
H	-0.085970	-0.383720	5.051900
C	-2.957040	-2.848430	3.973180
H	-0.383210	-2.560180	6.194550
C	-2.328260	3.248290	-0.229230
O	-0.343750	2.673090	-1.843350
O	0.113420	4.093700	0.223960
C	-5.077150	3.742020	0.040130
C	-2.775120	4.193520	0.694600
C	-3.236650	2.563140	-1.040740
C	-4.599770	2.813610	-0.897740
C	-4.143440	4.431220	0.824580
H	-2.054520	4.749700	1.284500
H	-2.888180	1.862500	-1.790840
H	-5.303440	2.286000	-1.537350
H	-4.489080	5.172040	1.541560
C	-6.558960	3.982340	0.204480
H	-7.002280	3.252210	0.894650
H	-7.088340	3.889080	-0.749680
H	-6.761120	4.978670	0.610510
H	-0.597400	1.844240	3.849930
C	-0.705000	-0.706400	-0.870990
C	-1.207420	-0.422440	-2.147950
C	-1.253320	-1.809770	-0.196020
C	-2.226990	-1.189850	-2.722160
H	-0.814460	0.428460	-2.696660
C	-2.258170	-2.589850	-0.774200
H	-0.890380	-2.060990	0.795020
C	-2.757080	-2.282680	-2.042650
H	-3.536510	-2.887190	-2.492120
C	-2.867910	-3.740930	-0.025230
C	-2.785310	-0.771540	-4.052170
F	-2.093460	-4.181910	0.981790
F	-3.128190	-4.792260	-0.834690
F	-4.073960	-3.397910	0.531460
F	-3.568360	-1.720590	-4.610110
F	-1.811260	-0.476120	-4.942020
F	-3.549480	0.348510	-3.934670
O	1.500250	0.620030	-1.162510
C	2.195880	-0.058700	-2.035180
C	2.242540	-1.542280	-2.135640
C	2.387250	-2.086920	-3.424060
C	2.168300	-2.408520	-1.032280
C	2.436910	-3.465220	-3.612920
H	2.442910	-1.422450	-4.283820
C	2.221280	-3.788050	-1.226600
H	2.060590	-2.005130	-0.032800
C	2.353730	-4.320070	-2.510830
H	2.532440	-3.871520	-4.615940
H	2.156230	-4.449600	-0.367500

H	2.389890	-5.396690	-2.653670
H	2.221760	0.425290	-3.016100
C	4.169510	0.445710	-1.821610
H	4.118250	1.389980	-2.350380
H	4.629230	-0.364350	-2.373160
C	4.360220	0.505190	-0.434800
C	4.797810	-0.640470	0.372180
C	4.475770	-0.696690	1.742570
C	5.545650	-1.689860	-0.199530
C	4.878200	-1.784310	2.510970
H	3.873600	0.083140	2.190640
C	5.962440	-2.763040	0.580400
H	5.827430	-1.655230	-1.246460
C	5.625940	-2.814900	1.936120
H	4.595110	-1.828200	3.558110
H	6.549760	-3.558950	0.131840
H	5.945270	-3.658730	2.541830
O	4.006620	1.575580	0.245190
Si	4.136520	3.291130	-0.082240
C	5.993190	3.584270	-0.206130
H	6.503940	3.308010	0.723100
H	6.445530	3.010420	-1.023540
H	6.197580	4.644960	-0.397600
C	3.386940	4.076210	1.438440
H	2.297500	4.127960	1.345040
H	3.644990	3.524760	2.348890
H	3.758910	5.102560	1.549820
C	3.249320	3.804420	-1.654570
H	3.825850	3.594920	-2.563020
H	2.261340	3.341540	-1.743390
H	3.088210	4.889600	-1.616430

TS 225

B3LYP/6-31G(d) = -3573.928283
B3LYP-D3(BJ)/def2-TZVPP/IEFPCM(propanonitrile) = -3575.496131
B3LYP-D3(BJ)/def2-TZVPP/IEFPCM(propanonitrile)//B3LYP-D3(BJ)/6-31G(d) Free Energy (Quasiharmonic) = -3574.781271

Frequencies (Top 3 out of 300)

1. -251.5827 cm^{-1}
2. 5.3444 cm^{-1}
3. 8.1722 cm^{-1}

B3LYP/6-31G(d) Molecular Geometry in Cartesian Coordinates

B	-0.449584	0.096276	0.423427
O	-0.584607	-0.759240	-0.789259
N	0.883645	-0.449711	1.003008
C	1.537265	-1.383481	0.073307
S	1.723732	0.270402	2.241208
C	0.464369	-1.558090	-1.005713
O	0.522871	-2.329260	-1.942742
H	1.415096	-3.550837	0.148318
H	2.406933	-0.918692	-0.415128
C	1.976260	-2.764603	0.667704
C	3.444531	-3.059623	0.555472
C	4.163268	-3.464270	-0.631004
C	5.522202	-3.657230	-0.255191
N	5.614273	-3.375105	1.093113
C	4.366721	-3.017254	1.572291
H	4.224208	-2.739233	2.606731
C	6.101432	-4.271997	-2.484806
C	3.790368	-3.681832	-1.970070
H	7.534465	-4.205318	-0.864908
H	6.454566	-3.429113	1.646780
C	4.761194	-4.082805	-2.880298
H	6.837101	-4.586130	-3.220632
H	2.761904	-3.523176	-2.282816
C	6.500600	-4.061265	-1.169425
H	4.485962	-4.251674	-3.918146
C	2.864223	1.432694	1.481160
O	2.540522	-0.754978	2.906277
O	0.737350	1.045160	3.005769
C	4.552324	3.223920	0.140837
C	2.516517	2.784126	1.402738
C	4.062627	0.971795	0.930536
C	4.893454	1.871161	0.260751
C	3.362390	3.666530	0.738098
H	1.594914	3.129800	1.857079
H	4.346657	-0.070591	1.033729
H	5.821065	1.511794	-0.178061
H	3.086772	4.715591	0.666859
C	5.421609	4.188403	-0.629346
H	6.358874	3.721620	-0.947332
H	5.669044	5.072774	-0.030136
H	4.898497	4.541700	-1.526770
H	1.676435	-2.778323	1.717069
C	-0.531865	1.663208	0.046661
C	0.151967	2.153975	-1.077295
C	-1.253515	2.590707	0.809777
C	0.122556	3.507398	-1.421588
H	0.720925	1.472015	-1.702122
C	-1.291875	3.946744	0.469162
H	-1.795303	2.249401	1.684274
C	-0.603613	4.415294	-0.648710
H	-0.640403	5.464368	-0.919216
C	-2.029266	4.913637	1.354551
C	0.928890	4.017721	-2.583596
F	-1.272827	5.302369	2.408293
F	-3.157986	4.370256	1.865566
F	-2.387907	6.038634	0.693703
F	0.303195	5.032184	-3.225178
F	1.179763	3.055538	-3.497047
F	2.133406	4.503352	-2.183957
O	-1.587623	-0.191198	1.390545
C	-2.062277	-1.373692	1.637792
C	-2.362160	-1.714101	3.049052
C	-2.726872	-3.026807	3.391856
C	-2.212469	-0.749298	4.056053
C	-2.957606	-3.366161	4.720810
H	-2.823861	-3.780860	2.613354
C	-2.444495	-1.095413	5.386375
H	-1.868945	0.244299	3.792376
C	-2.822771	-2.397158	5.720812
H	-3.234036	-4.384175	4.980427
H	-2.314773	-0.349061	6.164724
H	-2.999250	-2.662491	6.759613
H	-1.709065	-2.199926	1.016457
C	-4.020843	-1.356605	0.886014
H	-4.262712	-0.378471	1.287919
H	-4.416016	-2.215475	1.412764
C	-3.837642	-1.475054	-0.489339
C	-3.813428	-2.804348	-1.144536
C	-4.834904	-3.733471	-0.874564
C	-2.756055	-3.168214	-1.997475
C	-4.813357	-4.993225	-1.468029
H	-5.658472	-3.453335	-0.224020
C	-2.729564	-4.441130	-2.566152
H	-1.927492	-2.490082	-2.167503
C	-3.758858	-5.350012	-2.312991
H	-5.617345	-5.696468	-1.270212
H	-1.891988	-4.721304	-3.198055
H	-3.737011	-6.337295	-2.766126
O	-3.566616	-0.387149	-1.173836
Si	-3.783388	0.205232	-2.794599
C	-2.370683	-0.315622	-3.912252
H	-2.321423	0.362703	-4.773551
H	-1.411895	-0.264877	-3.387045
H	-2.493237	-1.333684	-4.294663

C	-3.806010	2.058025	-2.526448
H	-3.967863	2.584417	-3.475155
H	-4.604813	2.353206	-1.837234
H	-2.855611	2.405540	-2.107838
C	-5.445231	-0.443998	-3.393605
H	-5.457260	-1.536324	-3.471971
H	-6.261123	-0.139944	-2.727804
H	-5.663763	-0.038889	-4.389652

TS 226

B3LYP/6-31G(d) = -3573.927723
B3LYP-D3(BJ)/def2-TZVPP/IEFPCM(propanonitrile) = -3575.499395
B3LYP-D3(BJ)/def2-TZVPP/IEFPCM(propanonitrile)//B3LYP-D3(BJ)/6-31G(d) Free Energy (Quasiharmonic) = -3574.781263

Frequencies (Top 3 out of 300)

1. -270.6360 cm^{-1}
2. 10.2579 cm^{-1}
3. 13.6942 cm^{-1}

B3LYP/6-31G(d) Molecular Geometry in Cartesian Coordinates

B	-0.150400	0.008670	-1.205040
O	-0.775570	-1.177170	-1.845420
N	1.061310	0.285500	-2.154980
C	1.066870	-0.710020	-3.251240
S	1.627049	1.786631	-2.636430
C	-0.111920	-1.615510	-2.916680
O	-0.419719	-2.617520	-3.531740
H	3.127150	-0.854189	-3.830330
H	0.833330	-0.241020	-4.218170
C	2.364360	-1.533689	-3.446520
C	2.872850	-2.241869	-2.222120
C	4.042550	-1.896419	-1.450780
C	4.139431	-2.831499	-0.382580
N	3.089571	-3.715279	-0.519250
C	2.327851	-3.347929	-1.614670
H	1.457061	-3.924410	-1.895420
C	6.118650	-1.776068	0.429340
C	5.027780	-0.898928	-1.569290
H	5.222261	-3.501458	1.377460
H	2.800581	-4.387529	0.174140
C	6.052070	-0.847078	-0.630200
H	6.931220	-1.710208	1.148080
H	4.979650	-0.173828	-2.377600
C	5.166691	-2.780058	0.565890
H	6.818550	-0.080998	-0.714060
C	2.113399	2.575511	-1.102380
O	0.574509	2.621520	-3.247480
O	2.838549	1.541521	-3.437650
C	2.975028	3.905541	1.206810
C	1.498428	3.768490	-0.726020
C	3.168299	2.044281	-0.353820
C	3.585889	2.709591	0.793820
C	1.931808	4.422341	0.429350
H	0.698998	4.175180	-1.334680
H	3.647859	1.120111	-0.657310
H	4.396619	2.289732	1.383460
H	1.451528	5.351190	0.727330
C	3.427088	4.596741	2.470170
H	2.952268	5.576041	2.585000
H	3.174188	3.991661	3.348120
H	4.513518	4.743232	2.477490
H	2.138190	-2.254599	-4.241900
C	0.209840	-0.350110	0.343910
C	0.165680	-1.690680	0.758510
C	0.580089	0.594590	1.317360
C	0.483450	-2.074400	2.064830
H	-0.123340	-2.450850	0.041930
C	0.888100	0.219390	2.629210
H	0.632779	1.647280	1.059770
C	0.844950	-1.120490	3.013540
H	1.082420	-1.410900	4.030060
C	1.314909	1.253500	3.634620
C	0.354801	-3.516810	2.461260
F	0.773619	2.467170	3.380230
F	2.660859	1.424871	3.651850
F	0.956369	0.910600	4.894230
F	0.828621	-4.359530	1.506220
F	1.007281	-3.803290	3.605440
F	-0.946249	-3.877960	2.653540
O	-1.148871	1.154120	-1.327110
C	-1.946351	1.648579	-0.425040
C	-2.342292	3.069709	-0.609450
C	-2.186952	3.701319	-1.852540
C	-2.845722	3.794529	0.482860
C	-2.545502	5.041389	-1.995230
H	-1.744792	3.152369	-2.676590
C	-3.200352	5.132779	0.333650
H	-2.945802	3.309859	1.451940
C	-3.056172	5.756929	-0.909150
H	-2.415852	5.530409	-2.956520
H	-3.579642	5.691239	1.184910
H	-3.331563	6.801489	-1.026650
H	-1.803451	1.326909	0.608280
C	-3.833431	0.796339	-0.600430
H	-3.920121	0.798919	-1.680810
H	-4.344051	1.605488	-0.097850
C	-3.795030	-0.438151	0.060640
C	-3.854020	-0.545871	1.532340
C	-3.297670	-1.672671	2.165190
C	-4.446461	0.455758	2.326780
C	-3.313800	-1.783771	3.552330
H	-2.828000	-2.441721	1.565500
C	-4.479181	0.329518	3.712210
H	-4.913511	1.319498	1.864970
C	-3.908050	-0.787081	4.329050
H	-2.850329	-2.645901	4.020870
H	-4.953951	1.100878	4.311670
H	-3.926990	-0.878171	5.411480
O	-3.576290	-1.572351	-0.564480
Si	-4.151980	-2.263542	-2.064280
C	-3.250249	-3.892961	-2.166090
H	-3.273149	-4.423591	-1.207660
H	-3.725879	-4.537131	-2.916500
H	-2.210439	-3.733381	-2.469590
C	-3.834680	-1.142761	-3.537550
H	-4.567220	-0.331912	-3.618920
H	-2.830880	-0.711911	-3.499840
H	-3.900250	-1.738611	-4.456590
C	-6.001299	-2.475062	-1.770190
H	-6.476279	-2.921262	-2.652840
H	-6.200019	-3.132892	-0.916730
H	-6.497620	-1.515742	-1.581160

TS 227

B3LYP/6-31G(d) = -3573.916766
B3LYP-D3(BJ)/def2-TZVPP/IEFPCM(propanonitrile) = -3575.496671
B3LYP-D3(BJ)/def2-TZVPP/IEFPCM(propanonitrile)//B3LYP-D3(BJ)/6-31G(d) Free Energy (Quasiharmonic) = -3574.781151

Frequencies (Top 3 out of 300)

1. -296.1016 cm^{-1}
2. 4.7333 cm^{-1}
3. 9.0386 cm^{-1}

B3LYP/6-31G(d) Molecular Geometry in Cartesian Coordinates

B	0.401561	-0.428409	0.355224
O	0.500102	-1.104093	1.660905
N	0.089142	-1.623301	-0.584177
C	0.221455	-2.896047	0.138074
S	-0.543541	-1.569972	-2.091167
C	0.390297	-2.434098	1.589491
O	0.413309	-3.170073	2.551738
H	1.079569	-4.253717	-1.290824
H	-0.712189	-3.474026	0.094196
C	1.374290	-3.832867	-0.325118
C	2.721008	-3.183240	-0.449111
C	3.735365	-3.022900	0.567658
C	4.817163	-2.316463	-0.030865
N	4.478107	-2.084869	-1.349575
C	3.213247	-2.585876	-1.583705
H	2.743035	-2.481579	-2.551569
C	6.049009	-2.369740	2.010761
C	3.841729	-3.404119	1.917629
H	6.789941	-1.440366	0.202545
H	4.952924	-1.444177	-1.968134
C	4.992751	-3.074842	2.623129
H	6.934832	-2.123160	2.590281
H	3.024455	-3.925427	2.407940
C	5.975987	-1.982690	0.677324
H	5.080116	-3.359595	3.668413
C	-2.200650	-2.273456	-1.955711
O	0.182793	-2.453290	-3.018539
O	-0.676577	-0.139543	-2.435314
C	-4.815467	-3.282915	-1.851838
C	-3.038173	-1.918401	-0.896403
C	-2.653132	-3.127926	-2.962304
C	-3.955774	-3.621253	-2.905662
C	-4.335480	-2.425647	-0.851176
H	-2.670941	-1.270772	-0.106417
H	-1.984815	-3.401172	-3.771554
H	-4.307679	-4.285690	-3.691414
H	-4.979293	-2.157535	-0.017053
C	-6.210645	-3.857005	-1.777632
H	-6.885106	-3.203211	-1.214766
H	-6.208902	-4.833307	-1.274950
H	-6.636207	-4.007904	-2.775353
H	1.401134	-4.660360	0.394649
C	1.728698	0.442086	0.063877
C	2.468818	0.925541	1.153656
C	2.222639	0.723329	-1.218527
C	3.619443	1.698081	0.977223
H	2.140455	0.694178	2.162367
C	3.402954	1.450309	-1.398314
H	1.678916	0.380957	-2.091811
C	4.102449	1.958587	-0.303216
H	5.004754	2.541920	-0.445075
C	3.971970	1.620286	-2.777007
C	4.273856	2.334283	2.169185
F	4.796567	0.578530	-3.110849
F	4.722463	2.738947	-2.891120
F	3.021246	1.668794	-3.729815
F	4.232834	1.548420	3.263350
F	3.641056	3.496914	2.508517
F	5.566114	2.655701	1.942155
O	-0.879346	0.454433	0.517668
C	-1.399793	1.483888	-0.077512
C	-0.642585	2.732004	-0.340140
C	-0.844395	3.386011	-1.565639
C	0.271516	3.263596	0.582021
C	-0.128268	4.543387	-1.869021
H	-1.525905	2.957391	-2.295257
C	0.986751	4.417131	0.277731
H	0.429903	2.763918	1.531414
C	0.786058	5.059807	-0.948520
H	-0.272833	5.032165	-2.828249
H	1.715286	4.796771	0.987585
H	1.348574	5.957990	-1.188041
H	-2.175582	1.263456	-0.814753
C	-2.733962	2.236288	1.358862
H	-2.997430	3.162210	0.861607
H	-1.985940	2.335259	2.134792
C	-3.742701	1.289138	1.545178
C	-3.624999	0.123933	2.426795
C	-4.758981	-0.678972	2.679344
C	-2.394100	-0.234620	3.014151
C	-4.659239	-1.801167	3.491958
H	-5.710017	-0.407540	2.235942
C	-2.293076	-1.381916	3.797571
H	-1.500671	0.343191	2.822180
C	-3.424364	-2.161287	4.043909
H	-5.540771	-2.404878	3.688594
H	-1.323647	-1.679993	4.182988
H	-3.343823	-3.052307	4.660131
O	-4.878743	1.335224	0.853071
Si	-5.727910	2.543098	-0.044834
C	-4.785485	2.958676	-1.615750
H	-4.534867	2.052475	-2.179297
H	-5.410591	3.585255	-2.264626
H	-3.857594	3.508532	-1.426478
C	-5.979002	4.039809	1.065173
H	-5.037741	4.524349	1.345352
H	-6.592606	4.788362	0.548620
H	-6.502192	3.764096	1.987845
C	-7.343709	1.676851	-0.432453
H	-8.003034	2.326240	-1.020814
H	-7.170772	0.764022	-1.013375
H	-7.879575	1.398947	0.482021

TS 228

B3LYP/6-31G(d) = -3573.923339
B3LYP-D3(BJ)/def2-TZVPP/IEFPCM(propanonitrile) = -3575.498053
B3LYP-D3(BJ)/def2-TZVPP/IEFPCM(propanonitrile)//B3LYP-D3(BJ)/6-31G(d) Free Energy (Quasiharmonic) = -3574.781129

Frequencies (Top 3 out of 300)

1. -307.3456 cm^{-1}
2. 8.1915 cm^{-1}
3. 12.9821 cm^{-1}

B3LYP/6-31G(d) Molecular Geometry in Cartesian Coordinates

B	-0.133403	0.929878	0.858557
O	-0.386898	0.900784	2.308253
N	-1.446419	1.579182	0.336274
C	-2.013749	2.389512	1.438938
S	-1.659203	2.062996	-1.235758
C	-1.323542	1.781420	2.667968
O	-1.593857	2.049331	3.819120
H	-3.966978	2.939839	0.748085
H	-1.676018	3.433119	1.363285
C	-3.547301	2.440280	1.623234
C	-4.278348	1.145593	1.870040
C	-5.377184	0.628746	1.085888
C	-5.823577	-0.568851	1.712307
N	-5.032179	-0.759212	2.825018
C	-4.116850	0.272661	2.921414
H	-3.426308	0.318264	3.751045
C	-7.506749	-0.874392	0.050581
C	-6.031400	1.063133	-0.081534
H	-7.206646	-2.244683	1.698046
H	-5.112300	-1.523299	3.477267
C	-7.085791	0.312189	-0.585349

H	-8.335131	-1.440444	-0.367271
H	-5.706979	1.967103	-0.590094
C	-6.881288	-1.331721	1.205067
H	-7.594831	0.640476	-1.487546
C	-2.358295	0.631986	-2.074762
O	-0.345934	2.269610	-1.887611
O	-2.625880	3.171840	-1.257265
C	-3.392712	-1.558171	-3.483427
C	-2.157550	0.538853	-3.456478
C	-3.078390	-0.346143	-1.393850
C	-3.592925	-1.431207	-2.104384
C	-2.675113	-0.551589	-4.147909
H	-1.583884	1.300633	-3.973667
H	-3.221278	-0.269041	-0.323210
H	-4.156644	-2.191544	-1.570051
H	-2.510925	-0.629408	-5.220171
C	-3.898520	-2.765006	-4.236518
H	-4.701361	-3.271255	-3.691404
H	-4.278379	-2.491345	-5.227124
H	-3.090512	-3.493055	-4.388132
H	-3.698182	3.116948	2.474340
C	0.249274	-0.533192	0.289805
C	0.505625	-0.820811	-1.061618
C	0.307547	-1.611171	1.189762
C	0.809405	-2.117811	-1.489348
C	0.610141	-2.907468	0.766774
H	0.110048	-1.427956	2.239611
C	0.862106	-3.171965	-0.579168
H	1.093368	-4.178551	-0.910438
C	0.741867	-4.027296	1.760924
C	1.028700	-2.400756	-2.947989
F	0.093339	-3.775948	2.915987
F	2.046025	-4.256051	2.087799
F	0.267952	-5.196230	1.272469
F	1.969215	-3.366158	-3.133973
F	1.454144	-1.304653	-3.624063
F	-0.090418	-2.832007	-3.569522
O	1.018720	1.974108	0.758028
C	1.932474	2.114635	-0.160556
C	2.256110	3.512335	-0.568160
C	1.989421	4.588163	0.289194
C	2.816391	3.758425	-1.828545
C	2.289030	5.889420	-0.107383
H	1.530121	4.395848	1.253556
C	3.111800	5.061086	-2.225315
H	2.990159	2.930189	-2.511058
C	2.854110	6.129089	-1.362913
H	2.073248	6.719629	0.559459
H	3.531206	5.244144	-3.210698
H	3.082329	7.145390	-1.672120
H	1.959386	1.379145	-0.964338
C	3.736052	1.641146	0.610318
H	3.770821	2.416364	1.365485
H	4.343711	1.854051	-0.262062
C	3.735352	0.311660	1.066033
C	3.266842	-0.084266	2.393623
C	2.676208	0.848020	3.271295
C	3.392606	-1.426722	2.810125
C	2.227734	0.447873	4.525453
H	2.524547	1.874431	2.963084
C	2.956331	-1.816756	4.069163
H	3.811933	-2.157465	2.130270
C	2.375018	-0.879752	4.930642
H	1.740553	1.168424	5.174375
H	3.045680	-2.855988	4.369214
H	2.023568	-1.189164	5.911068
O	4.096112	-0.690123	0.278741
Si	5.175142	-0.886044	-1.064009
C	6.860976	-0.289426	-0.481009
H	7.202994	-0.857187	0.391610
H	7.605630	-0.420660	-1.275655
H	6.853573	0.772069	-0.209234
C	4.579677	0.054910	-2.577063
H	5.219657	-0.210177	-3.428992
H	3.554861	-0.219461	-2.847841
H	4.631554	1.142454	-2.461536
H	0.450620	-0.030885	-1.802395
C	5.122250	-2.732481	-1.364712
H	5.325811	-3.296642	-0.447999
H	4.144557	-3.042936	-1.747859
H	5.875886	-3.019954	-2.108256

TS 229

B3LYP/6-31G(d) = -3573.926077
B3LYP-D3(BJ)/def2-TZVPP/IEFPCM(propanonitrile) = -3575.497273
B3LYP-D3(BJ)/def2-TZVPP/IEFPCM(propanonitrile)//B3LYP-D3(BJ)/6-31G(d) Free Energy (Quasiharmonic) = -3574.780779

Frequencies (Top 3 out of 300)

1. -297.4248 cm^{-1}
2. 5.7151 cm^{-1}
3. 9.7390 cm^{-1}

B3LYP/6-31G(d) Molecular Geometry in Cartesian Coordinates

B	0.115409	0.777911	-0.449551
O	0.301922	1.042395	-1.902455
N	-0.243605	-0.760749	-0.492194
C	-0.263692	-1.255667	-1.882218
S	-0.104294	-1.854039	0.726722
C	0.141107	-0.022720	-2.692725
O	0.293331	-0.010488	-3.893419
H	-1.740290	-2.825747	-1.875715
H	0.522679	-2.007422	-2.027432
C	-1.572999	-1.888953	-2.418144
C	-2.830293	-1.061353	-2.366716
C	-4.162330	-1.566259	-2.121116
C	-5.056633	-0.460322	-2.194448
N	-4.302996	0.651238	-2.503090
C	-2.975560	0.284259	-2.599133
H	-2.236287	1.027097	-2.852667
C	-6.926157	-1.870054	-1.737418
C	-4.693323	-2.843229	-1.869011
H	-7.098025	0.263747	-2.049996
H	-4.617908	1.609746	-2.465737
C	-6.063631	-2.984427	-1.681267
H	-7.993232	-2.010435	-1.586391
H	-4.042990	-3.712405	-1.821153
C	-6.434465	-0.595480	-1.994783
H	-6.481750	-3.971390	-1.499211
C	-1.702451	-2.603656	1.050790
O	0.269309	-1.101956	1.940673
O	0.789674	-2.952382	0.286501
C	-4.142125	-3.808730	1.706030
C	-2.740359	-1.822462	1.567143
C	-1.860739	-3.977483	0.871587
C	-3.081043	-4.569152	1.200974
C	-3.947995	-2.430547	1.888420
H	-2.609851	-0.759152	1.730354
H	-1.034414	-4.568903	0.493136
H	-3.206797	-5.640539	1.064474
H	-4.755145	-1.821925	2.287532
C	-5.475106	-4.439907	2.023767
H	-5.406259	-5.531603	2.061793
H	-6.213495	-4.175656	1.256036
H	-5.866185	-4.088571	2.985246
H	-1.340991	-2.176239	-3.453571
C	-1.013555	1.746754	0.188218
C	-1.402602	1.661196	1.533325

C	-1.668651	2.701566	-0.602091
C	-2.440225	2.446204	2.045946
H	-0.895216	0.961757	2.191544
C	-2.704064	3.492238	-0.094212
H	-1.361848	2.832244	-1.634769
C	-3.107219	3.362665	1.234541
H	-3.913369	3.967801	1.631226
C	-3.435911	4.425915	-1.013791
C	-2.876171	2.228916	3.467645
F	-2.615908	5.012398	-1.910086
F	-4.087425	5.404054	-0.352302
F	-4.387892	3.766743	-1.749113
F	-3.699141	3.203565	3.913294
F	-1.827271	2.164400	4.316249
F	-3.550697	1.054574	3.600056
O	1.423516	1.185850	0.183890
C	2.454062	0.543824	0.680504
C	3.514132	1.429982	1.237827
C	3.611138	2.771629	0.843393
C	4.399872	0.931869	2.204250
C	4.585563	3.596969	1.401694
H	2.902985	3.159469	0.118974
C	5.373651	1.759201	2.760750
H	4.309966	-0.099358	2.534877
C	5.470211	3.093327	2.358141
H	4.649720	4.637699	1.096083
H	6.047470	1.367593	3.517896
H	6.225258	3.740417	2.796319
H	2.234339	-0.338083	1.281386
C	3.178352	-0.508482	-0.836057
H	2.601377	-1.412182	-0.664706
H	2.794173	0.116662	-1.631467
C	4.575650	-0.629191	-0.753854
C	5.507952	0.353191	-1.323150
C	5.055102	1.434260	-2.107038
C	6.892419	0.212050	-1.099680
C	5.961512	2.338334	-2.650848
H	3.997908	1.573173	-2.301418
C	7.793189	1.119733	-1.644144
H	7.246707	-0.613594	-0.494402
C	7.330886	2.184720	-2.422255
H	5.597626	3.162897	-3.256395
H	8.857221	0.998577	-1.462781
H	8.035907	2.891454	-2.851165
O	5.152071	-1.593426	-0.064320
Si	4.763908	-3.219930	0.441357
C	3.833779	-3.151403	2.065750
H	4.371198	-2.565257	2.819897
H	2.822356	-2.749729	1.950874
H	3.729753	-4.170759	2.460061
C	6.483667	-3.940220	0.661937
H	7.040593	-3.947086	-0.281829
H	7.065562	-3.368764	1.394064
H	6.424630	-4.975001	1.020749
C	3.811617	-4.078669	-0.927182
H	2.750830	-3.811457	-0.915860
H	4.227347	-3.858450	-1.917148
H	3.878636	-5.164114	-0.778491

TS 230

B3LYP/6-31G(d) = -3573.918908
B3LYP-D3(BJ)/def2-TZVPP/IEFPCM(propanonitrile) = -3575.496571
B3LYP-D3(BJ)/def2-TZVPP/IEFPCM(propanonitrile)//B3LYP-D3(BJ)/6-31G(d) Free Energy (Quasiharmonic) = -3574.780614

Frequencies (Top 3 out of 300)

1. -224.8941 cm^{-1}
2. 12.4936 cm^{-1}
3. 13.5323 cm^{-1}

B3LYP/6-31G(d) Molecular Geometry in Cartesian Coordinates

B	0.177669	-0.557589	-0.837188
O	-0.062088	-1.296385	-2.096820
N	1.046262	-1.601393	-0.059320
C	0.964189	-2.919046	-0.722431
S	1.782578	-1.449384	1.398138
C	0.368694	-2.559294	-2.084711
O	0.290520	-3.321841	-3.021305
H	0.702924	-4.348040	0.871538
H	1.969825	-3.321077	-0.900531
C	0.145015	-4.042158	-0.018061
C	-1.268858	-3.712979	0.369239
C	-2.463475	-3.779997	-0.445041
C	-3.551748	-3.371531	0.377466
N	-3.040066	-3.101353	1.631351
C	-1.673008	-3.285279	1.610609
H	-1.073018	-3.089043	2.487028
C	-5.090085	-3.644597	-1.424101
C	-2.727221	-4.139216	-1.779852
H	-5.679693	-2.974092	0.547060
H	-3.531013	-2.624869	2.372567
C	-4.031677	-4.066429	-2.253891
H	-6.098200	-3.591333	-1.826582
H	-1.918792	-4.440527	-2.438926
C	-4.865080	-3.296366	-0.096880
H	-4.240294	-4.332194	-3.286841
C	3.460658	-2.060496	1.127333
O	1.837688	0.001855	1.677197
O	1.220901	-2.319986	2.444632
C	6.118001	-2.923158	0.841437
C	4.157473	-1.787604	-0.051753
C	4.078122	-2.763946	2.163669
C	5.398735	-3.183936	2.015679
C	5.475451	-2.219605	-0.186973
H	3.674369	-1.259987	-0.866962
H	3.520693	-2.981552	3.068001
H	5.876559	-3.729796	2.825716
H	6.010018	-2.010537	-1.110491
C	7.534842	-3.418046	0.674269
H	8.106256	-2.778442	-0.006332
H	8.062673	-3.454057	1.632914
H	7.549610	-4.433358	0.256392
H	0.166481	-4.889034	-0.715705
C	-1.232539	-0.077817	-0.204720
C	-1.457332	0.161005	1.159464
C	-2.337659	0.042099	-1.063743
C	-2.730607	0.487946	1.639902
C	-3.604280	0.396517	-0.591266
H	-2.213941	-0.184292	-2.117602
C	-3.812502	0.616102	0.768525
H	-4.798385	0.866018	1.142853
C	-4.739663	0.587290	-1.556870
C	-2.969523	0.589305	3.117474
F	-5.943927	0.382596	-0.977907
F	-4.653182	-0.227535	-2.624078
F	-4.760630	1.862896	-2.043975
F	-1.908314	1.106684	3.781436
F	-3.203455	-0.634193	3.676682
F	-4.041476	1.354248	3.419330
O	0.955508	0.687821	-1.343953
C	2.146397	1.134137	-1.116477
C	3.061913	1.264127	-2.278252
C	2.583150	1.065435	-3.582887
C	4.428984	1.516982	-2.074077
C	3.458542	1.138624	-4.664729
H	1.535086	0.825527	-3.727021
C	5.300287	1.585865	-3.157759
H	4.807812	1.647319	-1.062658

C	4.814593	1.403348	-4.456435
H	3.084363	0.976422	-5.671545
H	6.356968	1.776677	-2.992218
H	5.494567	1.456353	-5.302232
H	2.590360	0.915920	-0.144998
C	2.046590	3.236149	-0.689035
H	3.093918	3.348172	-0.443648
H	1.772547	3.533475	-1.693549
C	1.103407	3.379281	0.324333
C	1.424934	3.300361	1.757995
C	2.739576	3.482025	2.229022
C	0.399339	3.056777	2.688893
C	3.016486	3.417715	3.589752
H	3.546697	3.700454	1.537586
C	0.682915	2.979450	4.048333
H	-0.611267	2.900825	2.334758
C	1.989989	3.162383	4.503019
H	4.033568	3.568710	3.939872
H	-0.117573	2.763780	4.748576
H	2.210261	3.105147	5.565424
O	-0.185298	3.510666	0.049187
Si	-1.030071	4.306783	-1.244543
C	-0.899931	3.330092	-2.842254
H	-1.102234	2.268957	-2.668014
H	-1.645831	3.701216	-3.556595
H	0.083107	3.412715	-3.318464
C	-0.261893	6.020307	-1.394954
H	-0.366591	6.583462	-0.460520
H	0.802970	5.985427	-1.649351
H	-0.769937	6.589519	-2.183268
H	-0.637125	0.079460	1.864791
C	-2.789950	4.403724	-0.617463
H	-3.303442	3.440428	-0.682184
H	-2.821761	4.746343	0.422580
H	-3.360998	5.119696	-1.221964

TS 231

B3LYP/6-31G(d) = -3573.927786
B3LYP-D3(BJ)/def2-TZVPP/IEFPCM(propanonitrile) = -3575.498451
B3LYP-D3(BJ)/def2-TZVPP/IEFPCM(propanonitrile)//B3LYP-D3(BJ)/6-31G(d) Free Energy (Quasiharmonic) = -3574.780612

Frequencies (Top 3 out of 300)

1. -285.9211 cm^{-1}
2. 12.3342 cm^{-1}
3. 12.5235 cm^{-1}

B3LYP/6-31G(d) Molecular Geometry in Cartesian Coordinates

B	-0.192171	-0.074197	0.088000
O	-1.082156	-0.889077	0.956528
N	-0.055857	-0.971227	-1.187332
C	-0.809390	-2.225693	-0.974857
S	-0.162365	-0.414365	-2.758183
C	-1.406273	-2.063564	0.419541
O	-2.102039	-2.891467	0.980044
H	0.237159	-3.655169	-2.178345
H	-1.662683	-2.290010	-1.660611
C	-0.019695	-3.550123	-1.121492
C	1.214645	-3.671674	-0.275901
C	2.577532	-3.493098	-0.713020
C	3.414538	-3.683208	0.420480
N	2.593976	-3.991599	1.486437
C	1.276396	-3.969680	1.064740
H	0.473226	-4.183301	1.757368
C	5.364524	-3.277377	-0.890028
C	3.171214	-3.208051	-1.956459
H	5.428364	-3.698842	1.230976
H	2.890743	-4.028127	2.449401
C	4.555348	-3.103873	-2.032960
H	6.443531	-3.183583	-0.980247
H	2.553736	-3.063596	-2.839443
C	4.807388	-3.569668	0.349141
H	5.025395	-2.886674	-2.988691
C	1.158651	0.790529	-2.858346
O	-1.432405	0.298466	-3.030699
O	0.150856	-1.556490	-3.636207
C	3.219294	2.666386	-3.132294
C	2.477201	0.407016	-2.599704
C	0.858286	2.085902	-3.277500
C	1.891389	3.014754	-3.410884
C	3.493412	1.348317	-2.733564
H	2.704170	-0.604298	-2.279656
H	-0.170748	2.349828	-3.495787
H	1.661423	4.026678	-3.736057
H	4.516359	1.058371	-2.509346
C	4.331143	3.682725	-3.225441
H	3.993690	4.607013	-3.704595
H	5.181463	3.293572	-3.797267
H	4.700395	3.932991	-2.224251
H	-0.730869	-4.349615	-0.877988
C	1.184368	0.281835	0.875018
C	1.644095	-0.546459	1.909729
C	1.954013	1.417053	0.581410
C	2.822417	-0.268356	2.606793
H	1.066402	-1.420656	2.183654
C	3.136853	1.700434	1.271772
H	1.626676	2.102385	-0.192196
C	3.580395	0.859281	2.291238
H	4.488032	1.087351	2.839283
C	3.974420	2.886598	0.884623
C	3.313307	-1.197726	3.679978
F	4.938902	2.552429	-0.015681
F	4.616816	3.425947	1.945953
F	3.244377	3.870880	0.312212
F	4.290788	-2.032958	3.228280
F	2.332382	-1.997881	4.163556
F	3.843578	-0.531380	4.729325
O	-0.864456	1.244603	-0.248200
C	-1.819507	1.778888	0.465275
C	-1.801301	3.264257	0.590119
C	-1.106347	4.059074	-0.331331
C	-2.466257	3.871694	1.665880
C	-1.081538	5.443798	-0.176608
H	-0.579114	3.583565	-1.151501
C	-2.437334	5.256416	1.817981
H	-2.998027	3.255689	2.387698
C	-1.746771	6.045634	0.894679
H	-0.535451	6.055234	-0.889511
H	-2.944601	5.718895	2.660136
H	-1.720843	7.125227	1.014273
H	-2.106132	1.244960	1.373061
C	-3.541069	1.545174	-0.555954
H	-4.007067	2.460910	-0.215869
H	-3.133614	1.539942	-1.563134
C	-4.122938	0.355033	-0.103575
C	-4.872729	0.279158	1.165462
C	-4.694585	-0.836784	2.007729
C	-5.729242	1.321530	1.569736
C	-5.363345	-0.896538	3.228903
H	-3.994544	-1.619592	1.729152
C	-6.409483	1.240374	2.781364
H	-5.889874	2.175081	0.918018
C	-6.225226	0.132741	3.614234
H	-5.204031	-1.748336	3.883603
H	-7.085561	2.038169	3.075124
H	-6.751139	0.075086	4.563238
O	-3.879984	-0.767223	-0.750364
Si	-4.943576	-1.900319	-1.547382

C	-4.055332	-2.338261	-3.137576
H	-3.326734	-3.143849	-2.997526
H	-4.777093	-2.677469	-3.891390
H	-3.517209	-1.472578	-3.538802
C	-5.203393	-3.384210	-0.433386
H	-5.667833	-4.200239	-1.001613
H	-4.243445	-3.739042	-0.042360
H	-5.853014	-3.154344	0.417861
C	-6.538455	-0.958385	-1.884231
H	-7.023272	-0.619156	-0.962141
H	-7.248702	-1.608912	-2.409381
H	-6.361081	-0.083551	-2.520490

TS 232

B3LYP/6-31G(d) = -3573.917381
B3LYP-D3(BJ)/def2-TZVPP/IEFPCM(propanonitrile) = -3575.496748
B3LYP-D3(BJ)/def2-TZVPP/IEFPCM(propanonitrile)//B3LYP-D3(BJ)/6-31G(d) Free Energy (Quasiharmonic) = -3574.780568

Frequencies (Top 3 out of 300)

1. -292.8429 cm^{-1}
2. 9.0081 cm^{-1}
3. 12.6882 cm^{-1}

B3LYP/6-31G(d) Molecular Geometry in Cartesian Coordinates

B	-0.214924	0.195838	0.340346
O	-0.210364	0.186785	1.816974
N	0.225914	1.655863	0.049726
C	0.254385	2.445849	1.287622
S	0.772061	2.276397	-1.362154
C	0.078931	1.370858	2.363253
O	0.198260	1.559405	3.554939
H	-0.491804	4.380443	0.720195
H	1.244455	2.895091	1.445539
C	-0.797246	3.586962	1.408698
C	-2.217389	3.194982	1.125260
C	-3.200094	2.664388	2.042579
C	-4.387043	2.429780	1.291583
N	-4.135321	2.822230	-0.008355
C	-2.828912	3.256901	-0.103069
H	-2.416820	3.581313	-1.048286
C	-5.515381	1.619570	3.230400
C	-3.200776	2.366772	3.417549
H	-6.443425	1.733352	1.278996
H	-4.715680	2.607580	-0.805528
C	-4.353599	1.848988	3.995471
H	-6.400256	1.209901	3.710490
H	-2.304683	2.515860	4.013340
C	-5.548040	1.907645	1.870565
H	-4.360320	1.610281	5.055815
C	2.465338	2.799813	-1.030158
O	0.049832	3.501578	-1.744520
O	0.803360	1.152951	-2.320723
C	5.123407	3.600256	-0.640167
C	2.896615	4.030924	-1.524840
C	3.345641	1.961940	-0.341180
C	4.663824	2.368548	-0.150377
C	4.221475	4.419947	-1.329999
H	2.195424	4.670225	-2.049804
H	2.997670	1.012810	0.055857
H	5.348217	1.716299	0.385439
H	4.557334	5.378669	-1.717949
C	6.547787	4.041688	-0.400328
H	6.650445	4.529752	0.578025
H	6.878637	4.758993	-1.158233
H	7.237608	3.190886	-0.411902
O	0.939918	-0.794749	-0.000038
C	1.336723	-1.425352	-1.065069
H	2.138731	-0.944402	-1.630432
H	-0.695192	3.985505	2.425853
C	0.449558	-2.284380	-1.884554
C	0.622009	-2.274610	-3.277356
C	-0.547770	-3.094092	-1.319087
C	-0.207958	-3.043278	-4.091799
H	1.377551	-1.630060	-3.719029
C	-1.376337	-3.859431	-2.132450
H	-0.678534	-3.113061	-0.243153
C	-1.207091	-3.834843	-3.520980
H	-0.083592	-3.014830	-5.170490
H	-2.163524	-4.456240	-1.681584
H	-1.857428	-4.429234	-4.156856
C	-1.646270	-0.285891	-0.232436
C	-2.422338	-1.168542	0.534280
C	-2.192036	0.147132	-1.450154
C	-3.657715	-1.643269	0.086720
H	-2.056011	-1.497912	1.501692
C	-3.452678	-0.281258	-1.875545
H	-1.628088	0.820096	-2.085827
C	-4.189031	-1.194100	-1.120115
H	-5.155294	-1.545137	-1.463188
C	-4.060663	0.305230	-3.116582
C	-4.359723	-2.723207	0.857484
F	-4.759033	1.450008	-2.837507
F	-3.139068	0.640690	-4.039169
F	-4.195993	-2.599430	2.189910
F	-4.942212	-0.533948	-3.705077
F	-3.869381	-3.953935	0.523508
F	-5.686775	-2.760526	0.608401
C	2.557040	-2.936072	-0.275560
H	2.845852	-3.453423	-1.181545
H	1.763389	-3.404096	0.291348
C	3.528948	-2.226544	0.425929
O	3.338696	-1.814069	1.663242
Si	2.593494	-2.444396	3.111976
C	2.715549	-1.012733	4.306579
H	2.507505	-1.362483	5.325779
H	1.986213	-0.227565	4.076702
H	3.717701	-0.569415	4.307868
C	0.817708	-2.985363	2.860719
H	0.707582	-3.867117	2.220213
H	0.395541	-3.243301	3.841020
H	0.225691	-2.159482	2.455802
C	3.698124	-3.894437	3.587911
H	4.732358	-3.577091	3.762614
H	3.333423	-4.362196	4.510652
H	3.709356	-4.667889	2.810793
C	4.795549	-1.775307	-0.185152
C	5.816478	-1.260225	0.635673
C	5.027281	-1.870336	-1.571007
C	7.035171	-0.867636	0.088981
H	5.645360	-1.181359	1.702904
C	6.241498	-1.463363	-2.115219
H	4.255779	-2.250211	-2.232164
C	7.251100	-0.965763	-1.287676
H	7.818319	-0.486062	0.738059
H	6.400152	-1.534055	-3.187179
H	8.201196	-0.656887	-1.714344

TS 233

B3LYP/6-31G(d) = -3573.917009
B3LYP-D3(BJ)/def2-TZVPP/IEFPCM(propanonitrile) = -3575.496939
B3LYP-D3(BJ)/def2-TZVPP/IEFPCM(propanonitrile)//B3LYP-D3(BJ)/6-31G(d) Free Energy (Quasiharmonic) = -3574.780563

Frequencies (Top 3 out of 300)

1. -260.3200 cm^{-1}
2. 7.5596 cm^{-1}
3. 9.3820 cm^{-1}

B3LYP/6-31G(d) Molecular Geometry in Cartesian Coordinates

B	-0.291522	0.233286	0.213463
O	-0.234682	0.289688	1.695455
N	-0.095791	1.759960	-0.122038
C	-0.307198	2.570625	1.092557
S	0.068319	2.491847	-1.577377
C	-0.184769	1.520651	2.195341
O	-0.040533	1.775547	3.374102
H	-1.567733	4.207257	0.480958
H	0.518541	3.280727	1.228130
C	-1.627339	3.392345	1.208247
C	-2.912202	2.641676	1.011918
C	-3.660901	1.875365	1.983421
C	-4.797644	1.345760	1.308957
N	-4.749688	1.798264	0.004850
C	-3.607461	2.551128	-0.169288
H	-3.357424	2.965587	-1.135024
C	-5.529966	0.244457	3.293415
C	-3.488489	1.579081	3.348002
H	-6.591587	0.127319	1.411729
H	-5.305202	1.441308	-0.758368
C	-4.420115	0.768946	3.986227
H	-6.236667	-0.392943	3.818125
H	-2.625172	1.955399	3.889171
C	-5.735856	0.529524	1.948169
H	-4.288021	0.527665	5.037713
C	1.441371	3.644690	-1.364721
O	-1.081700	3.329717	-1.958250
O	0.484492	1.440316	-2.530347
C	3.609039	5.423489	-1.221403
C	1.378829	4.870514	-2.030320
C	2.572933	3.301119	-0.622349
C	3.644951	4.189771	-0.556754
C	2.460913	5.745708	-1.957762
H	0.487494	5.128491	-2.591425
H	2.607001	2.358969	-0.085042
H	4.524616	3.922766	0.024645
H	2.410133	6.698005	-2.480206
C	4.761300	6.394196	-1.119542
H	4.877797	6.975346	-2.040255
H	4.601973	7.109715	-0.301998
H	5.705938	5.877531	-0.920980
H	-1.593837	3.851562	2.204787
C	-1.643343	-0.504964	-0.288237
C	-2.262120	-0.283091	-1.526536
C	-2.241171	-1.445749	0.566572
C	-3.435859	-0.956280	-1.881927
C	-3.398717	-2.138378	0.204982
H	-1.810636	-1.617358	1.547593
C	-4.008944	-1.894312	-1.023874
H	-4.919018	-2.413489	-1.301417
C	-3.971266	-3.178830	1.127181
C	-4.158637	-0.577929	-3.141153
F	-5.305044	-3.332362	0.954455
F	-3.412005	-4.398770	0.911639
F	-3.761505	-2.882813	2.426065
F	-4.905193	-1.590897	-3.634565
F	-5.026441	0.459068	-2.920772
F	-3.325670	-0.171903	-4.119278
O	0.877641	-0.699164	-0.105904
C	1.351795	-1.106224	-1.252631
C	1.462259	-2.573498	-1.468318
C	1.781288	-3.066113	-2.743249
C	1.169918	-3.476530	-0.436717
C	1.820331	-4.437552	-2.979628
H	1.982478	-2.369804	-3.554614
C	1.205140	-4.849430	-0.676826
H	0.884415	-3.089203	0.535516
C	1.534496	-5.332884	-1.944719
H	2.059963	-4.809206	-3.972021
H	0.961127	-5.542818	0.123317
H	1.555032	-6.403167	-2.131143
H	1.081142	-0.520057	-2.132111
C	3.234056	-0.379026	-1.372312
H	3.592594	-0.841396	-2.284787
H	2.919311	0.654031	-1.456704
C	3.796855	-0.806511	-0.166871
C	4.603129	-2.043483	-0.093953
C	5.576912	-2.297931	-1.075918
C	4.397862	-2.991288	0.923121
C	6.341633	-3.460720	-1.027105
H	5.754244	-1.566181	-1.858044
C	5.150464	-4.162389	0.957177
H	3.620749	-2.828777	1.660773
C	6.128562	-4.396128	-0.012243
H	7.103469	-3.636675	-1.780892
H	4.969091	-4.896191	1.737086
H	6.720216	-5.306580	0.021187
O	3.465178	-0.151016	0.920483
Si	3.853064	0.032538	2.613054
C	3.589438	1.859617	2.931585
H	4.012889	2.122468	3.909454
H	4.079678	2.489756	2.181469
H	2.524274	2.110503	2.964901
C	5.659188	-0.424812	2.885893
H	5.940437	-0.144654	3.909163
H	5.863622	-1.491931	2.763791
H	6.318798	0.126241	2.205129
H	-1.841995	0.430487	-2.227594
C	2.639240	-0.991181	3.606417
H	1.614042	-0.775779	3.285379
H	2.809550	-2.071144	3.546502
H	2.716757	-0.705043	4.663206

TS 234

B3LYP/6-31G(d) = -3573.917228
B3LYP-D3(BJ)/def2-TZVPP/IEFPCM(propanonitrile) = -3575.496451
B3LYP-D3(BJ)/def2-TZVPP/IEFPCM(propanonitrile)//B3LYP-D3(BJ)/6-31G(d) Free Energy (Quasiharmonic) = -3574.780502

Frequencies (Top 3 out of 300)

1. -229.9362 cm^{-1}
2. 13.6161 cm^{-1}
3. 14.4256 cm^{-1}

B3LYP/6-31G(d) Molecular Geometry in Cartesian Coordinates

B	-0.067490	-0.630386	0.774798
O	0.219906	-1.398619	2.006951
N	-0.884098	-1.689356	-0.040040
C	-0.746243	-3.017342	0.589533
S	-1.637917	-1.525416	-1.494150
C	-0.163468	-2.674461	1.961227
O	-0.050718	-3.461886	2.873494
H	-0.401444	-4.413827	-1.026826
H	-1.732221	-3.467811	0.762493
C	0.136749	-4.079863	-0.135362
C	1.520126	-3.644255	-0.528789
C	2.725216	-3.645294	0.272438
C	3.770817	-3.132575	-0.547025
N	3.226672	-2.865033	-1.787920
C	1.877548	-3.151497	-1.760577
H	1.254579	-2.976475	-2.625366
C	5.345222	-3.343648	1.231620

C	3.029539	-4.022392	1.593604
H	5.861475	-2.573820	-0.723740
H	3.672280	-2.329817	-2.517823
C	4.330139	-3.866793	2.057973
H	6.351133	-3.227855	1.626371
H	2.253154	-4.402412	2.250627
C	5.080221	-2.974278	-0.082373
H	4.569378	-4.146407	3.080567
C	-3.355658	-2.022140	-1.244936
O	-1.112265	-2.463665	-2.497791
O	-1.647062	-0.078640	-1.784444
C	-6.045133	-2.773446	-0.924635
C	-4.355380	-1.048203	-1.247233
C	-3.692998	-3.374616	-1.130872
C	-5.026886	-3.737201	-0.959612
C	-5.687832	-1.428970	-1.087265
H	-4.089407	-0.010452	-1.418501
H	-2.927866	-4.139422	-1.208511
H	-5.282880	-4.789980	-0.868213
H	-6.465500	-0.668720	-1.105332
C	-7.484802	-3.178849	-0.717479
H	-8.174547	-2.406736	-1.072879
H	-7.697648	-3.345982	0.346688
H	-7.718090	-4.111052	-1.243046
H	0.180516	-4.932454	0.554032
C	1.306507	-0.034215	0.164189
C	2.395624	0.148543	1.032893
C	1.513402	0.274409	-1.188000
C	3.626677	0.630978	0.580363
H	2.289611	-0.124088	2.077721
C	2.753025	0.731478	-1.649285
H	0.703042	0.151751	-1.897996
C	3.818204	0.920367	-0.769396
H	4.778032	1.272966	-1.128173
C	2.967520	0.922019	-3.121427
C	4.734982	0.894857	1.559723
F	1.881707	1.452257	-3.737245
F	3.216725	-0.262119	-3.752696
F	4.015099	1.728320	-3.396628
F	5.956079	0.819516	0.984513
F	4.719040	0.042609	2.601265
F	4.635249	2.151168	2.084706
O	-0.904705	0.555407	1.322088
C	-2.165066	0.817169	1.223681
C	-2.985333	0.800241	2.457054
C	-4.385769	0.870289	2.373629
C	-2.373591	0.642436	3.710833
C	-5.161978	0.801477	3.526612
H	-4.863885	0.961254	1.401135
C	-3.154906	0.575348	4.862523
H	-1.295252	0.538863	3.760853
C	-4.546711	0.660820	4.774561
H	-6.244976	0.848934	3.454937
H	-2.677945	0.442243	5.829313
H	-5.152838	0.604485	5.674556
H	-2.664803	0.548164	0.294047
C	-2.370670	2.929282	0.838684
H	-2.112826	3.229011	1.846571
H	-3.429127	2.919503	0.613745
C	-1.467027	3.198667	-0.184259
C	-1.807887	3.138714	-1.614515
C	-0.788217	2.970481	-2.567809
C	-3.138636	3.265589	-2.057177
C	-1.093364	2.905723	-3.923221
H	0.235901	2.857818	-2.236522
C	-3.437654	3.214624	-3.414592
H	-3.939374	3.439590	-1.345265
C	-2.416523	3.028771	-4.350814
H	-0.295719	2.746004	-4.641792
H	-4.466595	3.326615	-3.744565
H	-2.653567	2.981192	-5.410058
O	-0.190566	3.436122	0.072031
Si	0.621334	4.266017	1.365039
C	0.621188	3.246159	2.940182
H	-0.357353	3.208639	3.430879
H	0.936573	2.218584	2.735959
H	1.332916	3.681616	3.653169
C	-0.286222	5.902682	1.580542
H	-1.335742	5.767720	1.864100
H	0.193589	6.495799	2.368872
H	-0.259989	6.494613	0.658493
C	2.350950	4.523344	0.699500
H	2.871030	5.280715	1.299538
H	2.947780	3.607665	0.742596
H	2.330022	4.876533	-0.337201

TS 235

B3LYP/6-31G(d) = -3573.920164
B3LYP-D3(BJ)/def2-TZVPP/IEFPCM(propanonitrile) = -3575.498331
B3LYP-D3(BJ)/def2-TZVPP/IEFPCM(propanonitrile)//B3LYP-D3(BJ)/6-31G(d) Free Energy (Quasiharmonic) = -3574.780239

Frequencies (Top 3 out of 300)

1. -285.7762 cm^{-1}
2. 11.6855 cm^{-1}
3. 14.6665 cm^{-1}

B3LYP/6-31G(d) Molecular Geometry in Cartesian Coordinates

B	-0.160227	0.999403	0.134005
O	-0.691362	1.842474	1.222768
N	-0.894710	1.588830	-1.106778
C	-1.576943	2.846854	-0.733930
S	-0.402879	1.419399	-2.685994
C	-1.433452	2.869644	0.788901
O	-1.901062	3.716497	1.517929
H	-3.028273	3.337165	-2.235774
H	-1.020977	3.724033	-1.096025
C	-3.049858	3.051507	-1.182895
C	-4.000157	1.901708	-1.012486
C	-4.687813	1.453761	0.179578
C	-5.514618	0.357656	-0.199044
N	-5.332966	0.156657	-1.552232
C	-4.434347	1.091846	-2.033253
H	-4.164793	1.120392	-3.080288
C	-6.328679	0.124426	2.028733
C	-4.709994	1.879434	1.522474
H	-6.950132	-1.157049	0.401046
H	-5.819343	-0.526590	-2.110890
C	-5.526303	1.212523	2.427964
H	-6.950940	-0.380079	2.762982
H	-4.094537	2.713393	1.844840
C	-6.335119	-0.316168	0.711230
H	-5.546651	1.534260	3.465824
C	-0.427481	-0.356482	-2.949269
O	-1.419633	2.054895	-3.539849
O	1.001768	1.845495	-2.879246
C	-0.442309	-3.114620	-3.436254
C	-1.587725	-1.093250	-2.703533
C	0.713919	-0.972792	-3.462010
C	0.698162	-2.345937	-3.701619
C	-1.584864	-2.463864	-2.946794
H	-2.467332	-0.604422	-2.299382
H	1.599844	-0.378608	-3.655955
H	1.591692	-2.830104	-4.087989
H	-2.480809	-3.042818	-2.736008
C	-0.423330	-4.613284	-3.618822
H	-0.055879	-5.104301	-2.708302
H	-1.423794	-5.007702	-3.824373

H	0.237346	-4.910870	-4.439998
H	-3.398943	3.922132	-0.612966
C	-0.417553	-0.570913	0.436437
C	0.518347	-1.567292	0.127146
C	-1.629170	-0.988165	1.010709
C	0.258388	-2.919518	0.366240
C	-1.896224	-2.340570	1.250285
H	-2.379910	-0.250015	1.277706
C	-0.953728	-3.317441	0.929594
H	-1.160988	-4.364240	1.117332
C	-3.236630	-2.752184	1.797871
C	1.316233	-3.941756	0.074081
F	-4.172504	-2.826105	0.818983
F	-3.191465	-3.975188	2.381523
F	-3.696672	-1.883538	2.719435
F	2.049922	-3.634292	-1.022657
F	2.214162	-4.059117	1.098132
F	0.804550	-5.178667	-0.124801
O	1.371445	1.129556	0.054889
C	2.240518	2.032676	-0.295862
C	2.024338	3.501450	-0.209097
C	2.486452	4.282510	-1.281759
C	1.410600	4.132117	0.885204
C	2.315821	5.665152	-1.270867
H	2.935948	3.794962	-2.141589
C	1.242508	5.514603	0.892589
H	1.057133	3.542688	1.722968
C	1.694475	6.283592	-0.183481
H	2.657792	6.257548	-2.114754
H	0.749108	5.988166	1.735896
H	1.558266	7.361560	-0.175824
H	2.876423	1.733281	-1.130152
C	3.808153	1.865288	1.069162
H	4.531717	2.467683	0.534642
H	3.346826	2.347856	1.921205
C	3.964750	0.480545	1.106170
C	4.749984	-0.275207	0.112997
C	4.550872	-1.662801	-0.017309
C	5.687431	0.357159	-0.727338
C	5.252527	-2.390379	-0.973523
H	3.833249	-2.160583	0.621783
C	6.396589	-0.377658	-1.672224
H	5.881828	1.420390	-0.634107
C	6.178103	-1.752078	-1.801749
H	5.065204	-3.454997	-1.074710
H	7.121599	0.121981	-2.308096
H	6.728915	-2.321573	-2.545197
O	3.263780	-0.265938	1.934850
Si	2.690842	-0.155064	3.574132
C	1.665317	1.381585	3.900786
H	1.213797	1.282346	4.897191
H	2.254228	2.305552	3.909814
H	0.844735	1.491786	3.183327
C	4.258196	-0.177136	4.616034
H	4.008515	-0.138139	5.683432
H	4.840657	-1.088750	4.442092
H	4.903095	0.682210	4.397658
H	1.463830	-1.281909	-0.317915
C	1.665248	-1.707227	3.767177
H	2.154449	-2.579238	3.321954
H	1.491611	-1.917830	4.829689
H	0.689410	-1.592033	3.284290

TS 236

B3LYP/6-31G(d) = -3573.92435

B3LYP-D3(BJ)/def2-TZVPP/IEFPCM(propanonitrile) = -3575.496576

B3LYP-D3(BJ)/def2-TZVPP/IEFPCM(propanonitrile)//B3LYP-D3(BJ)/6-31G(d) Free Energy (Quasiharmonic) = -3574.780178

Frequencies (Top 3 out of 300)

1. -252.1301 cm^{-1}
2. 8.9596 cm^{-1}
3. 9.8165 cm^{-1}

B3LYP/6-31G(d) Molecular Geometry in Cartesian Coordinates

B	0.097977	1.138520	-0.071036
O	0.879291	2.328649	-0.453364
N	0.489317	1.003432	1.443429
C	1.378994	2.110198	1.845960
S	-0.252394	0.096665	2.597204
C	1.454536	2.960943	0.576895
O	1.985470	4.044724	0.505776
H	2.722978	1.086627	3.196423
H	0.916192	2.703011	2.642422
C	2.812559	1.720635	2.308236
C	3.680802	1.062392	1.271610
C	4.629582	1.710426	0.390271
C	5.200575	0.697513	-0.427555
N	4.646869	-0.506435	-0.038496
C	3.722535	-0.275571	0.961856
H	3.167899	-1.098385	1.389133
C	6.553111	2.295911	-1.564202
C	5.056393	3.039378	0.213546
H	6.556490	0.183852	-2.041999
H	4.716063	-1.360409	-0.570769
C	6.012562	3.316733	-0.754759
H	7.290632	2.547878	-2.321761
H	4.618250	3.839864	0.801088
C	6.153558	0.973749	-1.413860
H	6.342113	4.341736	-0.902653
C	0.811417	-1.275225	3.078152
O	-1.433409	-0.520185	1.954083
O	-0.441870	0.913277	3.807787
C	2.445359	-3.417978	3.859164
C	1.536877	-1.194981	4.267900
C	0.873255	-2.423017	2.283906
C	1.687986	-3.481762	2.679823
C	2.348950	-2.263163	4.647103
H	1.444609	-0.315076	4.895341
H	0.282838	-2.501804	1.379125
H	1.725718	-4.376538	2.063097
H	2.910218	-2.200293	5.576173
C	3.350003	-4.558062	4.261345
H	4.353885	-4.432038	3.834544
H	3.464428	-4.615006	5.348690
H	2.964707	-5.519920	3.907487
H	3.280240	2.654202	2.642106
C	0.419267	-0.135532	-1.005176
C	-0.372304	-1.291462	-1.003928
C	1.577992	-0.151227	-1.800781
C	-0.005024	-2.430835	-1.731790
C	1.934227	-1.277030	-2.547097
H	2.210080	0.729639	-1.830704
C	1.148707	-2.431396	-2.511137
H	1.430259	-3.309212	-3.081483
C	3.206376	-1.303285	-3.346339
C	-0.814170	-3.685988	-1.584785
F	4.193345	-1.996400	-2.691738
F	3.697819	-0.078211	-3.594892
F	3.046978	-1.933579	-4.532367
F	-2.151046	-3.458794	-1.670946
F	-0.616183	-4.264261	-0.367043
F	-0.511603	-4.618743	-2.511724
O	-1.370205	1.563322	-0.310282
C	-2.209251	2.102992	0.520661
C	-2.465710	3.555374	0.436303
C	-3.214725	4.193992	1.440534
C	-1.909715	4.317385	-0.604783

C	-3.417346	5.569056	1.396002
H	-3.626533	3.608363	2.259339
C	-2.115951	5.695081	-0.643876
H	-1.286144	3.824668	-1.342652
C	-2.872742	6.321295	0.349158
H	-3.989876	6.058675	2.178681
H	-1.670606	6.283314	-1.440952
H	-3.026827	7.396399	0.317906
H	-2.294834	1.649792	1.508597
C	-4.205344	1.429114	0.051064
H	-4.521358	2.295837	-0.511581
H	-4.570663	1.378649	1.068966
C	-4.032396	0.236107	-0.648475
C	-3.762683	0.200819	-2.093835
C	-3.819484	-1.023937	-2.785854
C	-3.450098	1.372100	-2.811974
C	-3.587563	-1.072543	-4.156126
H	-4.040865	-1.930843	-2.239046
C	-3.210732	1.316409	-4.180872
H	-3.369554	2.324601	-2.301514
C	-3.282814	0.096387	-4.857786
H	-3.637684	-2.024467	-4.676687
H	-2.959353	2.225165	-4.719718
H	-3.096361	0.056608	-5.927446
O	-4.004480	-0.942171	-0.055409
Si	-4.690032	-1.712765	1.340168
C	-3.575223	-3.190580	1.598814
H	-4.026071	-3.900928	2.302544
H	-3.377877	-3.716227	0.659287
H	-2.615197	-2.861122	2.008318
C	-6.426287	-2.189918	0.788582
H	-6.401363	-2.848127	-0.087103
H	-6.950913	-2.722823	1.591306
H	-7.026432	-1.309178	0.530974
H	-1.278604	-1.313975	-0.408614
C	-4.741456	-0.606813	2.858178
H	-3.751355	-0.192224	3.071320
H	-5.470825	0.207219	2.782752
H	-5.032567	-1.220156	3.721316

TS 237

B3LYP/6-31G(d) = -3573.915428
B3LYP-D3(BJ)/def2-TZVPP/IEFPCM(propanonitrile) = -3575.497155
B3LYP-D3(BJ)/def2-TZVPP/IEFPCM(propanonitrile)//B3LYP-D3(BJ)/6-31G(d) Free Energy (Quasiharmonic) = -3574.780167

Frequencies (Top 3 out of 300)

1. -260.2702 cm^{-1}
2. 9.8347 cm^{-1}
3. 13.6659 cm^{-1}

B3LYP/6-31G(d) Molecular Geometry in Cartesian Coordinates

B	-0.232296	0.215021	0.444065
O	-0.017988	0.292634	1.913064
N	-0.187906	1.748227	0.087915
C	-0.348913	2.552163	1.313713
S	-0.141641	2.493764	-1.366839
C	-0.030331	1.528887	2.403894
O	0.178299	1.801665	3.569035
H	-1.919540	3.924733	0.753867
H	0.413699	3.340377	1.357915
C	-1.725495	3.232294	1.576961
C	-2.901832	2.313236	1.770154
C	-3.986931	2.058034	0.849349
C	-4.857062	1.124993	1.477639
N	-4.337772	0.853474	2.727672
C	-3.158414	1.553450	2.888003
H	-2.586134	1.464851	3.801502
C	-6.294758	1.096373	-0.422768
C	-4.307801	2.514259	-0.442436
H	-6.644985	-0.096853	1.350690
H	-4.633663	0.083639	3.308121
C	-5.452711	2.029405	-1.062655
H	-7.177034	0.728955	-0.940179
H	-3.651739	3.205826	-0.960212
C	-6.009170	0.632508	0.855883
H	-5.698845	2.364475	-2.066571
C	1.254636	3.637568	-1.270761
O	-1.311846	3.347451	-1.622658
O	0.187005	1.453227	-2.364724
C	3.437610	5.404330	-1.312830
C	1.163031	4.839600	-1.976829
C	2.422243	3.314350	-0.578197
C	3.502006	4.197175	-0.604838
C	2.251974	5.707941	-1.997051
H	0.243069	5.085754	-2.495573
H	2.481048	2.392401	-0.008917
H	4.410189	3.945819	-0.061650
H	2.177625	6.640863	-2.550884
C	4.599199	6.369366	-1.314743
H	5.537511	5.869694	-1.053573
H	4.726191	6.840634	-2.295370
H	4.442874	7.175916	-0.586370
H	-1.571191	3.840406	2.477523
C	-1.582308	-0.601459	0.083215
C	-2.210065	-0.557045	-1.170844
C	-2.160162	-1.427235	1.059921
C	-3.365738	-1.298541	-1.433557
C	-3.319791	-2.162967	0.803833
H	-1.705551	-1.479541	2.043585
C	-3.930477	-2.105874	-0.448654
H	-4.835395	-2.666973	-0.649211
C	-3.967676	-2.948246	1.905678
C	-3.985793	-1.252227	-2.803073
F	-4.655178	-4.015882	1.444345
F	-3.073531	-3.402753	2.811652
F	-4.870027	-2.196890	2.609824
F	-3.866635	-0.037693	-3.376960
F	-3.396551	-2.139887	-3.647228
F	-5.302759	-1.562812	-2.777118
O	0.954835	-0.637857	0.000855
C	1.335337	-1.031196	-1.185044
C	1.469889	-2.494175	-1.414394
C	1.690536	-2.976931	-2.713612
C	1.296014	-3.405354	-0.363305
C	1.750023	-4.346574	-2.955884
H	1.797676	-2.275122	-3.537925
C	1.352084	-4.776531	-0.609004
H	1.083799	-3.026024	0.630654
C	1.583753	-5.249959	-1.902096
H	1.911754	-4.710924	-3.966510
H	1.201028	-5.477181	0.207720
H	1.620396	-6.319137	-2.092396
H	0.971517	-0.452143	-2.035102
C	3.178953	-0.246865	-1.464913
H	3.466714	-0.685594	-2.413433
H	2.830639	0.778210	-1.502831
C	3.860205	-0.673665	-0.322073
C	4.710892	-1.882514	-0.343640
C	4.631191	-2.852887	0.669618
C	5.600867	-2.086210	-1.413169
C	5.424128	-3.996230	0.614403
H	3.918036	-2.730784	1.476309
C	6.406940	-3.221008	-1.454689
H	5.681587	-1.336455	-2.194148
C	6.318746	-4.179195	-0.442587
H	5.339151	-4.748263	1.393415
H	7.103487	-3.357362	-2.276735

H	6.942307	-5.067971	-0.479193
O	3.605900	-0.045268	0.801741
Si	4.114661	0.115993	2.462693
C	3.012368	-0.968992	3.519839
H	3.155499	-0.699580	4.574164
H	1.960746	-0.783211	3.274348
H	3.214049	-2.041074	3.425604
C	3.814065	1.924994	2.844526
H	4.287341	2.177497	3.802056
H	4.239199	2.588352	2.083481
H	2.745989	2.142721	2.947054
H	-1.810082	0.077172	-1.955946
C	5.950232	-0.283407	2.590166
H	6.180453	-1.339744	2.426921
H	6.537957	0.305221	1.875847
H	6.298361	-0.016119	3.596172

TS 238

B3LYP/6-31G(d) = -3573.923652
B3LYP-D3(BJ)/def2-TZVPP/IEFPCM(propanonitrile) = -3575.495951
B3LYP-D3(BJ)/def2-TZVPP/IEFPCM(propanonitrile)//B3LYP-D3(BJ)/6-31G(d) Free Energy (Quasiharmonic) = -3574.780063

Frequencies (Top 3 out of 300)

1. -252.5722 cm^{-1}
2. 7.6802 cm^{-1}
3. 8.8474 cm^{-1}

B3LYP/6-31G(d) Molecular Geometry in Cartesian Coordinates

B	0.092007	1.067051	-0.247029
O	0.849523	2.146495	-0.904391
N	0.528815	1.264939	1.246931
C	1.411864	2.440742	1.371592
S	-0.175629	0.629272	2.591662
C	1.441756	2.993748	-0.053919
O	1.953251	4.040946	-0.376505
H	2.810385	1.758760	2.874440
H	0.962241	3.189907	2.032194
C	2.863806	2.174330	1.863146
C	3.706643	1.300897	0.975361
C	4.618867	1.734397	-0.062258
C	5.175282	0.563765	-0.646293
N	4.648802	-0.521777	0.025930
C	3.754119	-0.072261	0.977819
H	3.222658	-0.778086	1.599662
C	6.471336	1.865948	-2.163274
C	5.023942	2.989597	-0.552097
H	6.484738	-0.299371	-2.145973
H	4.708907	-1.474143	-0.300833
C	5.944948	3.042410	-1.590290
H	7.181094	1.941250	-2.982980
H	4.595609	3.901280	-0.147954
C	6.092685	0.611431	-1.701471
H	6.257393	4.007907	-1.979311
C	0.920065	-0.590193	3.339335
O	-1.356647	-0.132257	2.129465
O	-0.347197	1.692784	3.594770
C	2.613252	-2.482229	4.532569
C	1.708027	-0.223882	4.432022
C	0.944400	-1.899411	2.852432
C	1.790850	-2.831167	3.451025
C	2.549378	-1.168790	5.017086
H	1.640502	0.782896	4.829819
H	0.295293	-2.195083	2.036396
H	1.803687	-3.852115	3.076800
H	3.159438	-0.881952	5.870156
C	3.550193	-3.491789	5.151446
H	3.700150	-3.298171	6.218642
H	4.537863	-3.456154	4.672821
H	3.170838	-4.512722	5.039702
H	3.329868	3.161856	1.960032
C	0.395716	-0.381933	-0.886238
C	-0.396647	-1.502790	-0.605860
C	1.533444	-0.584205	-1.685840
C	-0.051370	-2.779239	-1.067531
H	-1.280964	-1.384046	0.010413
C	1.869478	-1.852553	-2.166832
H	2.167201	0.262125	-1.928088
C	1.082206	-2.963326	-1.855185
H	1.352354	-3.949820	-2.214438
C	3.124954	-2.067880	-2.963685
C	-0.914084	-3.951027	-0.699601
F	3.605204	-0.936271	-3.505422
F	4.129425	-2.587066	-2.186206
F	2.943808	-2.960327	-3.964097
F	-1.280920	-3.905513	0.610912
F	-0.295031	-5.134617	-0.894921
F	-2.071978	-3.999054	-1.410529
O	-1.391368	1.416696	-0.521138
C	-2.267522	2.044629	0.203616
C	-2.451986	3.499091	0.023145
C	-3.209357	4.224996	0.959322
C	-1.832262	4.174350	-1.041959
C	-3.354351	5.601937	0.826729
H	-3.668624	3.705952	1.797601
C	-1.979705	5.554000	-1.168574
H	-1.208399	3.614979	-1.730735
C	-2.743528	6.267731	-0.241558
H	-3.932306	6.159758	1.558119
H	-1.484076	6.075296	-1.982280
H	-2.852054	7.344237	-0.341542
H	-2.458309	1.648089	1.201936
C	-4.221056	1.425730	-0.434805
H	-4.732956	1.550349	0.512446
H	-4.388645	2.215537	-1.152997
C	-4.024954	0.133584	-0.918149
C	-3.534219	-0.144623	-2.272899
C	-3.450361	-1.476701	-2.722124
C	-3.132654	0.890746	-3.141172
C	-2.993100	-1.763127	-4.003228
H	-3.728094	-2.278958	-2.051863
C	-2.674392	0.598421	-4.420936
H	-3.156935	1.923794	-2.815301
C	-2.606739	-0.727275	-4.857323
H	-2.927203	-2.796399	-4.330700
H	-2.359416	1.405066	-5.076133
H	-2.245572	-0.951740	-5.857162
O	-4.166600	-0.937062	-0.153178
Si	-5.126351	-1.348597	1.233208
C	-4.649697	-0.332203	2.735330
H	-3.562626	-0.302739	2.864490
H	-5.020854	0.698158	2.688315
H	-5.086182	-0.790846	3.632153
C	-4.717111	-3.163573	1.445316
H	-3.633876	-3.320053	1.476957
H	-5.145087	-3.557927	2.374618
H	-5.112633	-3.759202	0.614726
C	-6.919163	-1.067363	0.731029
H	-7.120902	-0.015884	0.496875
H	-7.187560	-1.665469	-0.147044
H	-7.591292	-1.356489	1.548511

TS 239

B3LYP/6-31G(d) = -3573.923742
B3LYP-D3(BJ)/def2-TZVPP/IEFPCM(propanonitrile) = -3575.497222

B3LYP-D3(BJ)/def2-TZVPP/IEFPCM(propanonitrile)//B3LYP-D3(BJ)/6-31G(d) Free Energy (Quasiharmonic) = -3574.780051

Frequencies (Top 3 out of 300)

1. -294.5061 cm^{-1}
2. 11.5814 cm^{-1}
3. 12.9071 cm^{-1}

B3LYP/6-31G(d) Molecular Geometry in Cartesian Coordinates

B	-0.392221	0.016632	1.571399
O	-0.834733	-0.403836	2.915654
N	-1.278572	1.308807	1.331683
C	-2.214841	1.453068	2.469960
S	-0.715335	2.687584	0.631716
C	-1.755265	0.384308	3.464198
O	-2.220335	0.233292	4.572625
H	-4.035594	1.958696	1.400723
H	-2.114924	2.433280	2.950290
C	-3.714668	1.205971	2.127223
C	-4.033216	-0.177577	1.618488
C	-4.357450	-0.568493	0.266209
C	-4.512472	-1.983226	0.260830
N	-4.329615	-2.419090	1.556085
C	-4.028099	-1.337567	2.359463
H	-3.823380	-1.475199	3.411904
C	-4.950566	-1.989035	-2.083309
C	-4.538043	0.126944	-0.942150
H	-4.891124	-3.788199	-0.879364
H	-4.202346	-3.384449	1.819697
C	-4.826960	-0.584400	-2.100564
H	-5.169112	-2.518804	-3.006577
H	-4.450106	1.208551	-0.971399
C	-4.800649	-2.705412	-0.901450
H	-4.959593	-0.052194	-3.039022
C	-2.134517	3.563859	-0.031366
O	0.098492	2.256597	-0.531883
O	-0.083742	3.598223	1.604918
C	-4.281632	5.004596	-1.108169
C	-2.634178	3.217430	-1.289233
C	-2.676404	4.631843	0.684686
C	-3.749830	5.339030	0.144124
C	-3.700595	3.941541	-1.816788
H	-2.188756	2.401691	-1.848363
H	-2.246151	4.915227	1.639267
H	-4.172960	6.171464	0.700786
H	-4.086761	3.678859	-2.798714
C	-5.459421	5.757454	-1.678822
H	-6.397102	5.217759	-1.491898
H	-5.368587	5.881198	-2.763288
H	-5.557478	6.750344	-1.228917
H	-4.264126	1.406548	3.055228
C	-0.638227	-1.161329	0.489101
C	-0.752429	-0.925772	-0.888023
C	-0.786526	-2.487106	0.924959
C	-0.978626	-1.965707	-1.792668
H	-0.676301	0.089908	-1.261139
C	-1.053047	-3.525961	0.027361
H	-0.725799	-2.702847	1.986872
C	-1.139054	-3.275091	-1.342251
H	-1.342819	-4.078207	-2.040519
C	-1.352348	-4.901311	0.548241
C	-0.993421	-1.668488	-3.263811
F	-1.073192	-5.868074	-0.353719
F	-2.676410	-5.045016	0.860368
F	-0.666471	-5.187162	1.675311
F	0.273186	-1.635749	-3.772248
F	-1.542175	-0.465289	-3.540090
F	-1.667225	-2.597019	-3.975456
O	1.103738	0.188211	1.790359
C	2.117891	0.878730	1.347801
C	3.369727	0.713886	2.138742
C	3.530755	-0.376136	3.006537
C	4.374975	1.689613	2.066126
C	4.688967	-0.496590	3.772555
H	2.730332	-1.103177	3.091303
C	5.531588	1.566579	2.834377
H	4.242552	2.553726	1.421254
C	5.693787	0.470809	3.685057
H	4.801349	-1.338334	4.450580
H	6.300017	2.332609	2.778207
H	6.592689	0.378679	4.288712
H	1.925692	1.889175	0.985962
C	2.424254	0.156933	-0.493882
H	2.181190	-0.879681	-0.293334
H	1.609066	0.736432	-0.907946
C	3.718055	0.419567	-0.956609
C	4.161758	1.733060	-1.441375
C	5.517267	1.933379	-1.772562
C	3.257870	2.807986	-1.579300
C	5.957014	3.170924	-2.228644
H	6.214202	1.111268	-1.661066
C	3.708295	4.043573	-2.035484
H	2.207066	2.690244	-1.335676
C	5.054057	4.229673	-2.361536
H	7.004351	3.312461	-2.479799
H	3.003019	4.863020	-2.136842
H	5.398387	5.196418	-2.718682
O	4.672410	-0.496787	-0.887044
Si	4.629292	-2.227953	-1.066130
C	3.697519	-2.595196	-2.655459
H	4.187569	-2.125798	-3.516320
H	2.656285	-2.255382	-2.637049
H	3.684447	-3.677796	-2.834408
C	3.868160	-3.057639	0.436331
H	2.778783	-2.956691	0.484321
H	4.094828	-4.131295	0.405413
H	4.286161	-2.655197	1.365084
C	6.456086	-2.631328	-1.197497
H	6.918731	-2.123136	-2.050813
H	6.993225	-2.328004	-0.291725
H	6.607452	-3.709517	-1.328793

TS 240

B3LYP/6-31G(d) = -3573.92108
B3LYP-D3(BJ)/def2-TZVPP/IEFPCM(propanonitrile) = -3575.495889
B3LYP-D3(BJ)/def2-TZVPP/IEFPCM(propanonitrile)//B3LYP-D3(BJ)/6-31G(d) Free Energy (Quasiharmonic) = -3574.779934

Frequencies (Top 3 out of 300)

1. -227.4048 cm^{-1}
2. 10.2846 cm^{-1}
3. 12.8476 cm^{-1}

B3LYP/6-31G(d) Molecular Geometry in Cartesian Coordinates

B	0.411653	-0.002241	-1.321169
O	0.305749	0.375016	-2.745511
N	-0.353322	-1.369325	-1.307523
C	-0.845676	-1.684280	-2.663315
S	-0.084378	-2.609822	-0.248887
C	-0.278541	-0.547954	-3.513981
O	-0.391108	-0.460295	-4.716618
H	-2.786739	-2.551781	-2.233180
H	-0.420580	-2.631056	-3.015794
C	-2.389811	-1.743177	-2.854769
C	-3.140819	-0.466341	-2.570899
C	-4.116113	-0.239490	-1.529462

C	-4.536080	1.116373	-1.629801
N	-3.876263	1.667672	-2.706719
C	-3.037598	0.720697	-3.260654
H	-2.431903	0.963558	-4.122086
C	-6.012630	0.861000	0.223736
C	-4.697504	-1.047769	-0.537065
H	-5.753924	2.728657	-0.839531
H	-3.860251	2.651728	-2.925399
C	-5.635871	-0.494122	0.324831
H	-6.742737	1.267651	0.918640
H	-4.419378	-2.092836	-0.442348
C	-5.469062	1.683424	-0.755553
H	-6.088028	-1.115064	1.094028
C	-1.632386	-3.116370	0.514038
O	0.740229	-2.049761	0.839537
O	0.407654	-3.787645	-0.987387
C	-3.941730	-4.029505	1.813541
C	-2.274917	-4.267139	0.054169
C	-2.124566	-2.416873	1.618301
C	-3.272772	-2.878953	2.256427
C	-3.428207	-4.710110	0.701152
H	-1.859296	-4.817568	-0.782948
H	-1.614741	-1.533807	1.986133
H	-3.656363	-2.333660	3.115081
H	-3.928539	-5.606633	0.343021
C	-5.165332	-4.538713	2.537794
H	-5.767464	-3.714889	2.935356
H	-5.801239	-5.138219	1.878662
H	-4.883495	-5.174270	3.387917
H	-2.537480	-2.051002	-3.897710
C	-0.174522	1.148077	-0.354805
C	-0.215754	1.010494	1.041218
C	-0.728203	2.316252	-0.897092
C	-0.812849	1.978541	1.852908
C	-1.323269	3.291692	-0.088328
H	-0.703303	2.459734	-1.971959
C	-1.376920	3.127017	1.294964
H	-1.846591	3.874512	1.923206
C	-1.958090	4.495236	-0.726018
C	-0.797468	1.802173	3.343303
F	-1.255577	4.931264	-1.795419
F	-2.075285	5.531120	0.132682
F	-3.217116	4.232940	-1.182635
F	-1.766732	2.506567	3.961947
F	0.384657	2.211123	3.894960
F	-0.943374	0.503967	3.707573
O	1.949264	-0.082312	-1.099945
C	2.793874	-1.061181	-1.129437
C	3.653869	-1.246787	-2.314765
C	3.667570	-0.299035	-3.351337
C	4.410546	-2.424817	-2.441214
C	4.441048	-0.524296	-4.487678
H	3.042591	0.582993	-3.268991
C	5.181969	-2.644288	-3.577666
H	4.375623	-3.172852	-1.652508
C	5.202843	-1.690377	-4.600485
H	4.438771	0.203978	-5.293537
H	5.757929	-3.560259	-3.673963
H	5.801610	-1.863723	-5.490481
H	2.557220	-1.953221	-0.549327
C	4.392848	-0.521189	0.261037
H	4.906366	-1.473076	0.274747
H	4.815074	0.226842	-0.396941
C	3.697568	-0.099427	1.385515
C	3.309965	-0.978133	2.502301
C	2.441848	-0.491400	3.496405
C	3.790304	-2.296908	2.611631
C	2.052610	-1.303811	4.556475
H	2.064033	0.519664	3.423728
C	3.406521	-3.102528	3.677439
H	4.469425	-2.700742	1.868441
C	2.533632	-2.610613	4.651891
H	1.363933	-0.914588	5.300460
H	3.785460	-4.118036	3.747132
H	2.230316	-3.245510	5.479847
O	3.237048	1.140511	1.475808
Si	3.799116	2.688374	0.942584
C	5.513348	2.894730	1.693519
H	5.941253	3.863859	1.408975
H	5.474945	2.855407	2.787960
H	6.205748	2.116214	1.353267
C	2.546661	3.871743	1.673560
H	2.313796	3.626429	2.714373
H	2.928654	4.899608	1.641094
H	1.607531	3.845581	1.111060
H	0.212605	0.123603	1.495077
C	3.832294	2.829312	-0.930661
H	4.656650	2.279962	-1.397915
H	2.893819	2.465830	-1.360678
H	3.944318	3.885687	-1.207421

TS 241

B3LYP/6-31G(d) = -3573.928528
B3LYP-D3(BJ)/def2-TZVPP/IEFPCM(propanonitrile) = -3575.497463
B3LYP-D3(BJ)/def2-TZVPP/IEFPCM(propanonitrile)//B3LYP-D3(BJ)/6-31G(d) Free Energy (Quasiharmonic) = -3574.779928

Frequencies (Top 3 out of 300)

1. -283.4775 cm^{-1}
2. 8.9370 cm^{-1}
3. 13.6289 cm^{-1}

B3LYP/6-31G(d) Molecular Geometry in Cartesian Coordinates

B	-0.335080	0.246740	-0.516740
O	-1.381129	-0.754801	-0.865900
N	0.352500	0.458660	-1.912990
C	-0.338629	-0.345510	-2.945100
S	0.941440	1.907370	-2.500410
C	-1.379579	-1.122601	-2.146170
O	-2.117009	-1.976391	-2.602350
H	1.146481	-0.670070	-4.458970
H	-0.897560	0.295929	-3.644280
C	0.534821	-1.294260	-3.805060
C	1.406671	-2.250120	-3.041380
C	2.831071	-2.143310	-2.832530
C	3.233181	-3.273739	-2.068780
N	2.106952	-4.039340	-1.848630
C	1.014001	-3.411790	-2.419890
H	0.030272	-3.857530	-2.360760
C	5.502321	-2.540969	-2.082100
C	3.807951	-1.212219	-3.231630
H	4.848282	-4.350409	-1.093120
H	2.051152	-4.809370	-1.200690
C	5.129601	-1.419429	-2.852570
H	6.543451	-2.674138	-1.800140
H	3.527621	-0.338709	-3.813730
C	4.561541	-3.482619	-1.681910
H	5.892051	-0.706719	-3.156080
C	2.089560	2.452590	-1.240390
O	1.689010	1.610760	-3.734240
O	-0.133040	2.919850	-2.595920
C	3.954539	3.414391	0.611960
C	1.878459	3.689230	-0.631880
C	3.227680	1.693171	-0.953260
C	4.144810	2.177301	-0.025590
C	2.813189	4.159590	0.291280
H	0.998069	4.268250	-0.885760
H	3.389630	0.737231	-1.440100

H	5.024000	1.583261	0.210140
H	2.652899	5.123920	0.767390
C	4.947989	3.910231	1.634400
H	5.978259	3.791391	1.280080
H	4.788959	4.967161	1.869880
H	4.855550	3.337811	2.564290
H	-0.169339	-1.844150	-4.441350
C	0.592671	-0.317740	0.684580
C	1.265180	0.516730	1.589920
C	0.720171	-1.700510	0.881480
C	2.037610	-0.004980	2.631460
C	1.472121	-2.229740	1.934270
H	0.218061	-2.380980	0.203130
C	2.142051	-1.383490	2.816550
H	2.728871	-1.787990	3.633890
C	1.485181	-3.709180	2.188930
C	2.811930	0.906240	3.541380
F	0.502172	-4.081080	3.056980
F	1.277762	-4.437320	1.062050
F	2.647492	-4.130420	2.729660
F	2.815810	0.462030	4.820330
F	4.116390	1.000031	3.167910
F	2.322620	2.165940	3.554390
O	-1.001120	1.504409	0.029900
C	-2.028940	2.092749	-0.523840
C	-2.142621	3.559819	-0.325070
C	-2.866561	4.330009	-1.245060
C	-1.532841	4.185059	0.772150
C	-2.976301	5.708099	-1.074800
H	-3.319801	3.850529	-2.109050
C	-1.649281	5.562949	0.943110
H	-0.965161	3.585669	1.476420
C	-2.371471	6.327099	0.021980
H	-3.525231	6.300159	-1.801520
H	-1.173311	6.042899	1.793880
H	-2.458002	7.401919	0.155650
H	-2.362400	1.711199	-1.489280
C	-3.804540	1.611249	0.412720
H	-3.643510	2.178059	1.321230
H	-4.431200	2.083008	-0.337540
C	-3.938130	0.224199	0.549610
C	-3.564539	-0.464921	1.799920
C	-2.850739	-1.676581	1.779570
C	-3.908440	0.107969	3.039990
C	-2.476909	-2.289821	2.973020
H	-2.533119	-2.083651	0.828950
C	-3.560759	-0.526461	4.228810
H	-4.476600	1.032628	3.067860
C	-2.838279	-1.721991	4.196940
H	-1.873209	-3.191191	2.948520
H	-3.846920	-0.085561	5.179330
H	-2.548199	-2.205631	5.125550
O	-4.341039	-0.458332	-0.497610
Si	-5.353889	-1.817522	-0.883090
C	-6.880609	-1.623642	0.201860
H	-6.631729	-1.692932	1.267020
H	-7.380339	-0.663292	0.031090
H	-7.603869	-2.418362	-0.019070
C	-4.500829	-3.463402	-0.613490
H	-5.112228	-4.254932	-1.066660
H	-3.529949	-3.457631	-1.119390
H	-4.361039	-3.712172	0.442620
H	1.186520	1.593040	1.486510
C	-5.700249	-1.532462	-2.699830
H	-6.172199	-0.559972	-2.878030
H	-4.760109	-1.571122	-3.260700
H	-6.364379	-2.308832	-3.098710

TS 242

B3LYP/6-31G(d) = -3573.918154
B3LYP-D3(BJ)/def2-TZVPP/IEFPCM(propanonitrile) = -3575.495893
B3LYP-D3(BJ)/def2-TZVPP/IEFPCM(propanonitrile)//B3LYP-D3(BJ)/6-31G(d) Free Energy (Quasiharmonic) = -3574.779746

Frequencies (Top 3 out of 300)

1. -259.3106 cm^{-1}
2. 7.6053 cm^{-1}
3. 10.1573 cm^{-1}

B3LYP/6-31G(d) Molecular Geometry in Cartesian Coordinates

B	0.186604	-0.759134	0.511870
O	0.418477	-1.678753	1.641431
N	0.682688	-1.625038	-0.685964
C	0.725673	-3.046144	-0.273156
S	0.653975	-1.191457	-2.278649
C	0.657318	-2.933575	1.258142
O	0.785909	-3.868027	2.017963
H	1.627298	-4.330717	-1.740637
H	-0.200813	-3.562356	-0.572230
C	1.877036	-3.977293	-0.739026
C	3.279047	-3.440824	-0.767119
C	4.182570	-3.148102	0.325865
C	5.409155	-2.710020	-0.252290
N	5.253471	-2.751980	-1.623413
C	3.979093	-3.189996	-1.922114
H	3.645080	-3.273104	-2.946852
C	6.390589	-2.418375	1.900680
C	4.094519	-3.229134	1.729560
H	7.441896	-2.008221	0.054552
H	5.948571	-2.480390	-2.300523
C	5.193139	-2.862864	2.497265
H	7.230244	-2.136024	2.530150
H	3.183101	-3.577793	2.204554
C	6.516306	-2.340021	0.518954
H	5.129855	-2.920660	3.580517
C	1.980649	0.006370	-2.498366
O	-0.584838	-0.430094	-2.570546
O	0.911879	-2.390446	-3.093107
C	3.952319	1.956543	-2.907359
C	1.818716	0.954898	-3.513544
C	3.123337	0.008129	-1.700495
C	4.098589	0.982812	-1.911980
C	2.804303	1.916862	-3.712731
H	0.918123	0.953091	-4.117867
H	3.237294	-0.718608	-0.906260
H	4.977006	0.995056	-1.271558
H	2.670889	2.662417	-4.492993
C	4.971463	3.056665	-3.077997
H	5.934853	2.787541	-2.633609
H	4.624044	3.974553	-2.586385
H	5.135645	3.295389	-4.134666
H	1.809933	-4.838862	-0.062153
C	0.878729	0.671838	0.753869
C	0.632502	1.770633	-0.082992
C	1.761278	0.866260	1.823830
C	1.237904	3.007204	0.136162
H	-0.032191	1.654798	-0.930704
C	2.371877	2.106064	2.050944
H	1.967809	0.040363	2.496720
C	2.113380	3.185340	1.210184
H	2.574116	4.149678	1.394914
C	3.353573	2.261908	3.180788
C	0.970821	4.165555	-0.779137
F	3.440948	3.547296	3.601012
F	4.602629	1.887735	2.815275
F	3.017448	1.510816	4.251278
F	-0.059052	3.930962	-1.628620
F	2.043298	4.489179	-1.539975

F	0.648702	5.289557	-0.082103
O	-1.353796	-0.513423	0.518374
C	-2.242920	-1.243851	-0.095109
C	-3.221244	-2.001319	0.722353
C	-3.403953	-1.704743	2.081345
C	-3.915264	-3.079183	0.151023
C	-4.276210	-2.471420	2.850711
H	-2.832889	-0.895468	2.523397
C	-4.783239	-3.848028	0.924723
H	-3.751168	-3.330334	-0.894912
C	-4.968412	-3.541330	2.276121
H	-4.403558	-2.246534	3.905914
H	-5.299670	-4.694905	0.481154
H	-5.635826	-4.146582	2.883560
H	-1.916967	-1.739001	-1.013249
C	-3.342739	0.034881	-1.254758
H	-3.772986	-0.678973	-1.948853
H	-2.461755	0.546008	-1.618613
C	-4.212612	0.667127	-0.367199
C	-3.847390	1.811051	0.473677
C	-2.798225	2.680091	0.118317
C	-4.573161	2.064681	1.656414
C	-2.483696	3.770976	0.923818
H	-2.246683	2.528293	-0.801350
C	-4.243966	3.145554	2.465104
H	-5.376627	1.393471	1.936479
C	-3.200735	4.002020	2.099306
H	-1.677001	4.436573	0.636179
H	-4.798868	3.322923	3.381934
H	-2.948500	4.850452	2.729326
O	-5.406975	0.144997	-0.106177
Si	-6.797605	-0.298024	-1.019713
C	-7.903407	-1.076327	0.275828
H	-7.407205	-1.932427	0.746008
H	-8.158143	-0.359753	1.064535
H	-8.840536	-1.430385	-0.170285
C	-6.388803	-1.506878	-2.400709
H	-5.884701	-2.401262	-2.020441
H	-7.321422	-1.832467	-2.879529
H	-5.762752	-1.062877	-3.182371
C	-7.497007	1.301781	-1.715817
H	-7.729017	2.017360	-0.919311
H	-6.793810	1.783395	-2.405371
H	-8.422323	1.107944	-2.272301

TS 243

B3LYP/6-31G(d) = -3573.928941
B3LYP-D3(BJ)/def2-TZVPP/IEFPCM(propanonitrile) = -3575.496696
B3LYP-D3(BJ)/def2-TZVPP/IEFPCM(propanonitrile)//B3LYP-D3(BJ)/6-31G(d) Free Energy (Quasiharmonic) = -3574.779733

Frequencies (Top 3 out of 300)

1. -285.5362 cm^{-1}
2. 6.8943 cm^{-1}
3. 14.2960 cm^{-1}

B3LYP/6-31G(d) Molecular Geometry in Cartesian Coordinates

B	0.443947	0.148714	1.113205
O	1.011182	0.355851	2.456334
N	1.121145	-1.203337	0.707242
C	1.732061	-1.827589	1.899983
S	0.676210	-2.137910	-0.573909
C	1.539499	-0.753409	2.977225
O	1.849677	-0.892084	4.138235
H	3.323835	-3.026153	1.056866
H	1.166742	-2.719495	2.196596
C	3.229987	-2.235746	1.808809
C	4.228321	-1.142925	1.532938
C	4.936333	-0.324737	2.496883
C	5.799067	0.536115	1.763440
N	5.638262	0.236731	0.425816
C	4.689016	-0.758610	0.296424
H	4.440045	-1.144396	-0.681018
C	6.620732	1.522050	3.771282
C	4.945152	-0.246995	3.902020
H	7.284670	2.119892	1.800410
H	5.994937	0.786520	-0.340348
C	5.784283	0.669979	4.521395
H	7.260737	2.233753	4.286285
H	4.280534	-0.871321	4.489337
C	6.639336	1.467300	2.383253
H	5.790303	0.740462	5.605807
C	2.097135	-2.430705	-1.636776
O	-0.248391	-1.315854	-1.384682
O	0.227604	-3.462613	-0.106343
C	4.278071	-2.895692	-3.334870
C	2.758810	-3.658110	-1.589179
C	2.496553	-1.438621	-2.535452
C	3.586698	-1.674643	-3.369718
C	3.845675	-3.879380	-2.434868
H	2.415200	-4.429472	-0.908309
H	1.965180	-0.495625	-2.583112
H	3.904274	-0.892417	-4.054279
H	4.363916	-4.834361	-2.395786
C	5.437800	-3.153640	-4.266826
H	6.139584	-3.879138	-3.843185
H	5.088064	-3.558235	-5.225924
H	5.988460	-2.232881	-4.485508
H	3.455472	-2.703243	2.774682
C	0.682071	1.397614	0.122868
C	1.877811	1.548895	-0.591192
C	-0.288231	2.392098	-0.053775
C	2.072594	2.612473	-1.478885
C	-0.105449	3.456451	-0.939145
H	-1.207651	2.335680	0.517169
C	1.077534	3.571375	-1.669405
H	1.224158	4.392039	-2.361516
C	-1.232670	4.421162	-1.168852
C	3.337488	2.663082	-2.287408
F	-1.964265	4.631025	-0.046195
F	-2.107211	3.954052	-2.104647
F	-0.808121	5.623985	-1.606069
F	3.526035	3.853949	-2.890611
F	3.337069	1.721247	-3.273486
F	4.438640	2.414920	-1.534772
O	-1.069418	0.077651	1.421377
C	-2.031658	-0.770618	1.191910
C	-3.070288	-0.860584	2.257478
C	-3.110329	0.087045	3.291105
C	-3.966436	-1.940246	2.279694
C	-4.045423	-0.035441	4.318057
H	-2.384106	0.892572	3.294696
C	-4.898696	-2.060211	3.308396
H	-3.922937	-2.694786	1.499116
C	-4.944180	-1.105194	4.327225
H	-4.060994	0.696068	5.121323
H	-5.581493	-2.905233	3.321256
H	-5.666532	-1.203106	5.133107
H	-1.770490	-1.721001	0.719362
C	-2.908630	-0.011116	-0.449647
H	-2.075113	-0.294406	-1.081390
H	-2.914837	1.028752	-0.144150
C	-4.164098	-0.581073	-0.694734
C	-4.364507	-1.914084	-1.278935
C	-3.275146	-2.684616	-1.736334
C	-5.667515	-2.443563	-1.382270
C	-3.492215	-3.948759	-2.276764
H	-2.260639	-2.305899	-1.676137

C	-5.874502	-3.705730	-1.926796
H	-6.505971	-1.856626	-1.026480
C	-4.787491	-4.462499	-2.374799
H	-2.643271	-4.533036	-2.618483
H	-6.882960	-4.102751	-2.000807
H	-4.950523	-5.449814	-2.798355
O	-5.276174	0.017771	-0.296960
Si	-5.774477	1.682875	-0.209271
C	-7.607496	1.488838	0.136744
H	-8.093312	2.468426	0.219690
H	-8.110681	0.932866	-0.662148
H	-7.775245	0.953306	1.078060
C	-5.425248	2.454142	-1.885864
H	-5.784996	1.824660	-2.707497
H	-5.941814	3.419716	-1.958703
H	-4.358356	2.648135	-2.038591
H	2.670652	0.820841	-0.450399
C	-4.912976	2.610370	1.177344
H	-4.803207	1.994996	2.076061
H	-3.924313	2.982296	0.887605
H	-5.517825	3.486298	1.446076

TS 244

B3LYP/6-31G(d) = -3573.917535
B3LYP-D3(BJ)/def2-TZVPP/IEFPCM(propanonitrile) = -3575.496942
B3LYP-D3(BJ)/def2-TZVPP/IEFPCM(propanonitrile)//B3LYP-D3(BJ)/6-31G(d) Free Energy (Quasiharmonic) = -3574.779597

Frequencies (Top 3 out of 300)

1. -312.3685 cm^{-1}
2. 11.2811 cm^{-1}
3. 13.9768 cm^{-1}

B3LYP/6-31G(d) Molecular Geometry in Cartesian Coordinates

B	0.071596	1.121214	0.378499
O	-0.150562	2.017280	1.531343
N	-0.799490	1.812478	-0.720877
C	-1.287706	3.121393	-0.233525
S	-0.643825	1.601714	-2.360209
C	-0.849863	3.115649	1.233927
O	-1.109021	3.987395	2.035855
H	-3.049165	3.539264	-1.396758
H	-0.759282	3.952641	-0.721265
C	-2.804679	3.424305	-0.339976
C	-3.729668	2.421434	0.295362
C	-4.679507	1.574560	-0.387904
C	-5.333666	0.785137	0.599199
N	-4.827586	1.165023	1.824373
C	-3.860326	2.137904	1.635032
H	-3.348962	2.575989	2.481086
C	-6.644068	-0.306130	-1.067386
C	-5.046619	1.411892	-1.737463
H	-6.782397	-0.770287	1.041540
H	-4.982588	0.672853	2.690982
C	-6.022185	0.477538	-2.062602
H	-7.400021	-1.032609	-1.353918
H	-4.555599	1.991888	-2.514005
C	-6.309045	-0.163935	0.274024
H	-6.309750	0.342483	-3.101901
C	-1.015617	-0.138621	-2.605373
O	0.754522	1.784277	-2.815823
O	-1.669789	2.428936	-3.015262
C	-1.574407	-2.849394	-3.040808
C	-2.188510	-0.692123	-2.090620
C	-0.138482	-0.910764	-3.368267
C	-0.422727	-2.259225	-3.578884
C	-2.457446	-2.040707	-2.311052
H	-2.873085	-0.086357	-1.509654
H	0.755066	-0.454514	-3.778798
H	0.264892	-2.864957	-4.164730
H	-3.365678	-2.470455	-1.896383
C	-1.830380	-4.328967	-3.200018
H	-1.344575	-4.890186	-2.390712
H	-1.430751	-4.708459	-4.146551
H	-2.900079	-4.559090	-3.163126
H	-2.927173	4.407058	0.132294
C	-0.344056	-0.403146	0.741807
C	0.378686	-1.510381	0.284538
C	-1.481860	-0.665564	1.518185
C	-0.019028	-2.819916	0.567952
H	1.266882	-1.344438	-0.312750
C	-1.885275	-1.970988	1.812418
H	-2.066290	0.165487	1.897274
C	-1.157130	-3.063040	1.336669
H	-1.471452	-4.076331	1.558944
C	-3.154527	-2.206832	2.582366
C	0.819520	-3.962477	0.079568
F	-3.394463	-1.223751	3.489171
F	-4.241046	-2.250251	1.773288
F	-3.130590	-3.375850	3.262660
F	1.857778	-4.240879	0.923066
F	1.389709	-3.707716	-1.127253
F	0.116757	-5.110029	-0.049883
O	1.557773	1.032653	0.042309
C	2.455868	1.793450	-0.532703
C	2.511609	3.265320	-0.339329
C	2.893297	4.059550	-1.432704
C	2.216998	3.879460	0.889855
C	2.964699	5.445174	-1.305153
H	3.092224	3.588215	-2.391033
C	2.289403	5.264924	1.012518
H	1.909788	3.272499	1.733990
C	2.664939	6.049661	-0.081978
H	3.241842	6.053213	-2.161672
H	2.038514	5.732566	1.959941
H	2.714817	7.130634	0.016564
H	2.723918	1.475913	-1.539593
C	4.288274	1.258742	0.194634
H	4.861240	1.290061	-0.726446
H	4.477004	2.094554	0.853444
C	4.098292	0.003331	0.793774
C	3.620322	-0.162434	2.169682
C	3.152967	-1.421667	2.599586
C	3.609483	0.912003	3.083080
C	2.687315	-1.594691	3.897767
H	3.130931	-2.249900	1.903052
C	3.145680	0.731497	4.380762
H	3.970842	1.890717	2.789036
C	2.683073	-0.521380	4.791831
H	2.316361	-2.566616	4.208838
H	3.142493	1.568521	5.072277
H	2.316614	-0.658419	5.805320
O	4.202242	-1.123211	0.116771
Si	4.900081	-1.691342	-1.366368
C	6.599368	-0.910888	-1.582128
H	6.562259	0.148117	-1.857670
H	7.201031	-1.003515	-0.670660
H	7.135980	-1.436103	-2.382406
C	3.726207	-1.288073	-2.770539
H	3.592234	-0.211928	-2.924539
H	4.110879	-1.710073	-3.707910
H	2.739503	-1.727052	-2.588533
C	5.020058	-3.533385	-1.054747
H	5.385631	-4.056530	-1.946700
H	4.039445	-3.948006	-0.799563
H	5.707738	-3.755595	-0.231032

TS 245

B3LYP/6-31G(d) = -3573.929438
B3LYP-D3(BJ)/def2-TZVPP/IEFPCM(propanonitrile) = -3575.495507
B3LYP-D3(BJ)/def2-TZVPP/IEFPCM(propanonitrile)//B3LYP-D3(BJ)/6-31G(d) Free Energy (Quasiharmonic) = -3574.779532

Frequencies (Top 3 out of 300)

1. -244.0269 cm^{-1}
2. 8.8871 cm^{-1}
3. 10.9175 cm^{-1}

B3LYP/6-31G(d) Molecular Geometry in Cartesian Coordinates

B	-0.420822	0.028322	0.456707
O	-0.488456	-0.787431	-0.789605
N	0.985684	-0.369886	0.972069
C	1.726106	-1.162542	-0.022299
S	1.736112	0.332228	2.275103
C	0.655247	-1.404852	-1.091990
O	0.795901	-2.086920	-2.087896
H	1.880245	-3.327010	-0.101237
H	2.523424	-0.568896	-0.493479
C	2.339536	-2.515364	0.475897
C	3.832468	-2.613063	0.346332
C	4.586152	-2.827951	-0.867675
C	5.961609	-2.878706	-0.506476
N	6.029004	-2.696879	0.860481
C	4.750508	-2.537545	1.364933
H	4.583156	-2.363417	2.418153
C	6.593582	-3.234059	-2.777786
C	4.231391	-2.982198	-2.220388
H	8.021248	-3.119249	-1.155846
H	6.874117	-2.689080	1.409363
C	5.236819	-3.183405	-3.158711
H	7.356323	-3.393057	-3.535590
H	3.188251	-2.930137	-2.520554
C	6.974839	-3.081790	-1.449061
H	4.975901	-3.301489	-4.207203
C	2.742973	1.674992	1.634609
O	2.659466	-0.646758	2.866855
O	0.666723	0.929288	3.085826
C	4.222586	3.769217	0.504974
C	2.258716	2.983563	1.710012
C	3.974362	1.403470	1.033471
C	4.699893	2.453145	0.467956
C	3.002483	4.018050	1.151548
H	1.311052	3.175359	2.200400
H	4.364872	0.390863	1.017259
H	5.652197	2.242395	-0.012483
H	2.620672	5.034399	1.199526
C	4.978351	4.899546	-0.150823
H	5.185736	5.709366	0.559112
H	5.932664	4.560563	-0.565227
H	4.386521	5.329214	-0.968416
H	2.049878	-2.642335	1.520500
C	-0.717524	1.582495	0.131619
C	0.076900	2.267729	-0.803608
C	-1.783598	2.289879	0.699433
C	-0.181102	3.593739	-1.156445
C	-2.057455	3.615157	0.341363
H	-2.413668	1.799255	1.432899
C	-1.258475	4.276908	-0.588079
H	-1.470960	5.302599	-0.867590
C	-3.275001	4.294850	0.901530
C	0.720078	4.325177	-2.113494
F	-3.508236	3.946253	2.185183
F	-4.394981	3.961244	0.202822
F	-3.179864	5.641684	0.856199
F	0.018723	5.135444	-2.940852
F	1.441970	3.486203	-2.884469
F	1.607084	5.124120	-1.462781
O	-1.490701	-0.436780	1.432705
C	-1.788627	-1.678917	1.652577
C	-2.029067	-2.093124	3.052777
C	-2.186988	-3.454081	3.364131
C	-2.028220	-1.139694	4.081987
C	-2.360815	-3.855368	4.684380
H	-2.167353	-4.195582	2.568025
C	-2.202942	-1.548036	5.403089
H	-1.842890	-0.099508	3.840361
C	-2.375693	-2.900146	5.706395
H	-2.476709	-4.909435	4.920220
H	-2.189180	-0.809106	6.199001
H	-2.507955	-3.213322	6.738432
H	-1.340875	-2.430990	0.999226
C	-3.763544	-1.935758	0.911874
H	-4.152289	-1.035861	1.376122
H	-3.987567	-2.876207	1.397720
C	-3.594286	-1.944741	-0.468571
C	-3.350278	-3.208899	-1.201119
C	-4.144020	-4.339435	-0.935433
C	-2.301119	-3.306640	-2.132001
C	-3.906183	-5.537813	-1.604039
H	-4.966060	-4.266314	-0.229175
C	-2.052809	-4.516928	-2.777211
H	-1.640285	-2.464559	-2.302719
C	-2.858032	-5.629538	-2.523837
H	-4.537694	-6.399614	-1.407525
H	-1.217942	-4.587227	-3.467976
H	-2.666078	-6.568453	-3.035840
O	-3.531561	-0.790166	-1.094195
Si	-3.893795	-0.121317	-2.654760
C	-4.836904	-1.378977	-3.690071
H	-5.694974	-1.795059	-3.149648
H	-5.227294	-0.875665	-4.584112
H	-4.210101	-2.211566	-4.022058
C	-2.285478	0.407183	-3.452343
H	-2.493934	0.871936	-4.424634
H	-1.763235	1.141090	-2.831226
H	-1.600925	-0.430722	-3.615879
H	0.915836	1.761896	-1.272015
C	-4.991306	1.341085	-2.234314
H	-5.939919	1.011895	-1.794067
H	-5.228537	1.914279	-3.139459
H	-4.513074	2.023203	-1.523861

TS 246

B3LYP/6-31G(d) = -3573.920973
B3LYP-D3(BJ)/def2-TZVPP/IEFPCM(propanonitrile) = -3575.495838
B3LYP-D3(BJ)/def2-TZVPP/IEFPCM(propanonitrile)//B3LYP-D3(BJ)/6-31G(d) Free Energy (Quasiharmonic) = -3574.779491

Frequencies (Top 3 out of 300)

1. -291.5507 cm^{-1}
2. 6.0040 cm^{-1}
3. 12.0491 cm^{-1}

B3LYP/6-31G(d) Molecular Geometry in Cartesian Coordinates

B	-0.476092	-0.643207	-0.855068
O	-0.555420	-0.072775	-2.210277
N	-1.649155	-1.668342	-0.859817
C	-2.243765	-1.728254	-2.205511
S	-1.658417	-3.024323	0.093300
C	-1.394023	-0.743409	-3.014002
O	-1.486372	-0.563790	-4.205753
H	-4.320226	-2.038168	-1.689985

H	-2.126419	-2.727947	-2.638783
C	-3.744128	-1.324171	-2.287957
C	-4.047334	0.085412	-1.861183
C	-4.145268	1.254338	-2.709111
C	-4.406900	2.366655	-1.862757
N	-4.489452	1.887641	-0.570176
C	-4.252883	0.525623	-0.576209
H	-4.258852	-0.035666	0.347698
C	-4.438436	3.850920	-3.726523
C	-4.040820	1.472185	-4.095160
H	-4.718007	4.506608	-1.681779
H	-4.501359	2.470139	0.252424
C	-4.192531	2.761709	-4.588222
H	-4.539742	4.850004	-4.142480
H	-3.810534	0.649174	-4.764279
C	-4.545568	3.669827	-2.352979
H	-4.106827	2.939183	-5.657018
C	-3.258686	-3.049132	0.923587
O	-0.654190	-2.811049	1.149659
O	-1.611992	-4.236224	-0.740155
C	-5.743238	-3.106132	2.225555
C	-3.460822	-2.265384	2.062926
C	-4.272885	-3.877771	0.444145
C	-5.506606	-3.898338	1.095205
C	-4.698561	-2.298872	2.701648
H	-2.653625	-1.658126	2.459260
H	-4.084787	-4.511411	-0.416088
H	-6.294723	-4.548120	0.722291
H	-4.853329	-1.696589	3.594011
C	-7.090018	-3.109187	2.908885
H	-6.991514	-2.968049	3.990523
H	-7.725043	-2.296533	2.531598
H	-7.625541	-4.048101	2.735409
H	-4.046936	-1.471564	-3.331203
C	-0.579739	0.514080	0.278633
C	-0.409288	0.272829	1.651954
C	-0.949226	1.819751	-0.097800
C	-0.586272	1.287707	2.601866
C	-1.149276	2.826127	0.850093
H	-1.106423	2.039368	-1.148350
C	-0.957672	2.571005	2.209683
H	-1.104073	3.353836	2.945330
C	-1.645935	4.185105	0.439288
C	-0.315521	0.989420	4.047841
F	-1.441523	4.445776	-0.861842
F	-1.058172	5.172651	1.155303
F	-2.988858	4.310820	0.664530
F	-0.878396	1.891338	4.879807
F	1.024003	0.999293	4.314257
F	-0.764136	-0.232655	4.406956
O	0.839223	-1.425120	-0.859974
C	1.931209	-1.188261	-0.188526
C	2.939975	-2.281891	-0.211258
C	2.916717	-3.254192	-1.220556
C	3.865283	-2.395570	0.836120
C	3.809678	-4.323428	-1.181010
H	2.174511	-3.179395	-2.007876
C	4.752196	-3.471232	0.878299
H	3.869394	-1.658039	1.635827
C	4.727769	-4.435886	-0.132592
H	3.776442	-5.081175	-1.958746
H	5.446090	-3.568391	1.708985
H	5.410881	-5.280099	-0.094985
H	1.820956	-0.699025	0.781173
C	2.680945	0.383439	-1.121783
H	1.873023	1.059241	-0.861474
H	2.623812	-0.026166	-2.125112
C	3.970100	0.758311	-0.705142
C	4.219258	1.616367	0.465441
C	5.401250	2.375501	0.562876
C	3.263939	1.725225	1.495808
C	5.626364	3.201091	1.659832
H	6.122879	2.354126	-0.244815
C	3.504212	2.530450	2.605305
H	2.332606	1.174357	1.445653
C	4.685129	3.270825	2.690513
H	6.534659	3.794655	1.709225
H	2.764951	2.569983	3.398282
H	4.867802	3.907242	3.551663
O	4.981162	0.297457	-1.407028
Si	6.629330	-0.207811	-1.500446
C	7.333437	-0.564463	0.203240
H	8.365677	-0.921818	0.094523
H	7.350701	0.313323	0.855835
H	6.761861	-1.353035	0.703041
C	7.546705	1.170493	-2.393663
H	7.025178	1.453791	-3.314831
H	7.682119	2.075251	-1.791506
H	8.545922	0.816777	-2.677853
H	-0.176476	-0.732639	1.989736
C	6.563161	-1.743537	-2.567754
H	5.963703	-2.532178	-2.101796
H	6.126099	-1.520723	-3.547529
H	7.572810	-2.139338	-2.734407

TS 247

B3LYP/6-31G(d) = -3573.917094
B3LYP-D3(BJ)/def2-TZVPP/IEFPCM(propanonitrile) = -3575.496031
B3LYP-D3(BJ)/def2-TZVPP/IEFPCM(propanonitrile)//B3LYP-D3(BJ)/6-31G(d) Free Energy (Quasiharmonic) = -3574.779476

Frequencies (Top 3 out of 300)

1. -259.5150 cm^{-1}
2. 9.6088 cm^{-1}
3. 12.0136 cm^{-1}

B3LYP/6-31G(d) Molecular Geometry in Cartesian Coordinates

B	-0.299718	0.206596	0.204867
O	-0.259800	0.273414	1.686604
N	-0.109544	1.730148	-0.140977
C	-0.325342	2.550031	1.066826
S	0.044446	2.447770	-1.605402
C	-0.207507	1.507823	2.177595
O	-0.063888	1.771360	3.354482
H	-1.593342	4.168929	0.423512
H	0.500338	3.260717	1.198953
C	-1.645192	3.372734	1.172059
C	-2.930491	2.614731	1.009876
C	-3.673907	1.889543	2.016365
C	-4.814386	1.332626	1.370739
N	-4.772667	1.730332	0.048644
C	-3.632974	2.477203	-0.162540
H	-3.390967	2.855232	-1.145129
C	-5.543203	0.327625	3.406523
C	-3.497068	1.655928	3.392427
H	-6.608481	0.121344	1.535188
H	-5.339030	1.352088	-0.695949
C	-4.429884	0.880717	4.070879
H	-6.249489	-0.283504	3.962103
H	-2.630004	2.053274	3.912021
C	-5.751371	0.548004	2.049856
H	-4.295620	0.689087	5.132323
C	1.374262	3.649929	-1.394951
O	0.506480	1.397938	-2.538768
O	-1.131088	3.237757	-2.011040
C	3.473861	5.508221	-1.251361
C	1.254096	4.883844	-2.036774
C	2.528027	3.338634	-0.672471

C	3.564797	4.267629	-0.604461
C	2.301618	5.800202	-1.961546
H	0.343187	5.117802	-2.576484
H	2.603271	2.392832	-0.145874
H	4.458785	4.028582	-0.032862
H	2.202851	6.762051	-2.458970
C	4.613809	6.497130	-1.197833
H	4.249292	7.529526	-1.210291
H	5.279446	6.377470	-2.063031
H	5.221516	6.361771	-0.297287
O	0.882291	-0.719994	-0.092654
C	1.359888	-1.138797	-1.233452
H	1.071327	-0.577085	-2.123256
H	-1.603895	3.857995	2.155831
C	1.504033	-2.607256	-1.419572
C	1.240375	-3.495125	-0.367240
C	1.826702	-3.118824	-2.686019
C	1.307619	-4.871557	-0.578746
H	0.952156	-3.094270	0.598697
C	1.897493	-4.493645	-2.893924
H	2.005970	-2.435127	-3.513086
C	1.640321	-5.373623	-1.838545
H	1.086466	-5.553584	0.237689
H	2.139801	-4.880230	-3.879940
H	1.685983	-6.446793	-2.002609
C	-1.637266	-0.549320	-0.308456
C	-2.284215	-0.297937	-1.525617
C	-2.187522	-1.547133	0.514360
C	-3.437227	-1.000433	-1.894412
H	-1.902710	0.458147	-2.203366
C	-3.321871	-2.268715	0.138515
H	-1.726353	-1.754729	1.474224
C	-3.960074	-1.995750	-1.070926
H	-4.844152	-2.549299	-1.366173
C	-3.911579	-3.295310	1.065798
C	-4.184091	-0.601623	-3.133284
F	-4.866917	-2.768820	1.865421
F	-4.495679	-4.310904	0.382879
F	-5.061791	0.417850	-2.873859
F	-2.978463	-3.844561	1.877025
F	-4.923700	-1.612100	-3.641253
F	-3.371350	-0.159117	-4.112619
C	3.223252	-0.368577	-1.380189
H	2.878431	0.653395	-1.479441
H	3.586126	-0.836395	-2.288207
C	3.806340	-0.762771	-0.173240
C	4.645428	-1.977220	-0.089115
C	4.472517	-2.916163	0.942046
C	5.618647	-2.219385	-1.074740
C	5.256011	-4.066504	0.986258
H	3.696729	-2.764174	1.683313
C	6.414173	-3.360866	-1.015992
H	5.771005	-1.493978	-1.867962
C	6.233095	-4.287648	0.012883
H	5.099599	-4.794074	1.777336
H	7.175068	-3.526914	-1.772974
H	6.848842	-5.181654	0.054205
O	3.467323	-0.099236	0.907068
Si	3.856787	0.114252	2.595645
C	2.669030	-0.924315	3.605189
H	2.744997	-0.625498	4.658573
H	1.637958	-0.734233	3.286950
H	2.861766	-2.001055	3.555831
C	5.673606	-0.298627	2.868513
H	6.318464	0.260215	2.179996
H	5.950845	-0.000877	3.887904
H	5.902509	-1.361921	2.757227
C	3.553628	1.938667	2.893379
H	2.483277	2.165625	2.930672
H	4.024663	2.570538	2.132526
H	3.977114	2.222954	3.865231

TS 248

B3LYP/6-31G(d) = -3573.920504
B3LYP-D3(BJ)/def2-TZVPP/IEFPCM(propanonitrile) = -3575.495009
B3LYP-D3(BJ)/def2-TZVPP/IEFPCM(propanonitrile)//B3LYP-D3(BJ)/6-31G(d) Free Energy (Quasiharmonic) = -3574.779435

Frequencies (Top 3 out of 300)

1. -194.5628 cm^{-1}
2. 4.2489 cm^{-1}
3. 9.9383 cm^{-1}

B3LYP/6-31G(d) Molecular Geometry in Cartesian Coordinates

B	-0.177889	-1.154429	-0.852652
O	0.123089	-2.159133	-1.892548
N	-0.080286	0.175898	-1.684438
C	0.321863	-0.123908	-3.073326
S	-0.888464	1.576436	-1.370707
C	0.310183	-1.652569	-3.113576
O	0.499957	-2.317920	-4.107288
H	1.727112	1.481356	-3.472493
H	-0.428319	0.251257	-3.778778
C	1.719568	0.388633	-3.532830
C	2.907832	-0.173517	-2.793773
C	3.817774	0.537280	-1.925419
C	4.759363	-0.409151	-1.432906
N	4.458436	-1.623177	-2.013593
C	3.343406	-1.479134	-2.815023
H	2.943198	-2.326950	-3.352611
C	5.863576	1.266241	-0.146725
C	3.947401	1.878932	-1.526621
H	6.460787	-0.814211	-0.148411
H	4.838150	-2.510563	-1.721020
C	4.963772	2.229769	-0.646892
H	6.640234	1.569219	0.550458
H	3.262175	2.634133	-1.898891
C	5.775109	-0.065114	-0.534993
H	5.065944	3.265142	-0.331662
C	0.262092	2.955337	-1.269121
O	-1.479238	1.430909	-0.027068
O	-1.785923	1.886864	-2.505132
C	1.925676	5.202291	-1.070122
C	0.428975	3.791941	-2.374787
C	0.905297	3.232020	-0.060484
C	1.727563	4.352993	0.028053
C	1.262667	4.904284	-2.269037
H	-0.111859	3.587381	-3.292476
H	0.755523	2.596876	0.804950
H	2.223138	4.571349	0.970580
H	1.387894	5.559026	-3.128039
C	2.843669	6.397030	-0.968659
H	2.850847	6.811961	0.044564
H	2.547083	7.191376	-1.661314
H	3.877220	6.119274	-1.214162
H	1.778945	0.135249	-4.598824
C	0.818026	-1.268510	0.405078
C	0.787725	-0.371002	1.483499
C	1.847430	-2.220195	0.389716
C	1.778025	-0.386411	2.468700
C	2.833584	-2.247589	1.380152
H	1.898425	-2.926715	-0.430276
C	2.813148	-1.320910	2.420884
H	3.600684	-1.310646	3.167050
C	3.930038	-3.274316	1.348847
C	1.763132	0.610001	3.593023
F	5.133256	-2.740240	1.669337
F	3.711834	-4.283556	2.224190

F	4.070570	-3.851087	0.125377
F	0.918197	1.642877	3.358086
F	1.378571	0.053011	4.771821
F	2.988978	1.139327	3.812479
O	-1.615727	-1.550120	-0.397424
C	-2.721673	-1.308766	-1.014235
C	-3.440421	-2.409828	-1.672558
C	-4.511158	-2.122531	-2.537369
C	-3.022353	-3.740596	-1.503779
C	-5.160964	-3.150223	-3.212932
H	-4.819042	-1.090174	-2.686396
C	-3.678828	-4.765533	-2.178955
H	-2.168123	-3.947241	-0.868555
C	-4.749323	-4.474214	-3.029524
H	-5.981352	-2.922560	-3.887453
H	-3.347634	-5.792468	-2.054498
H	-5.255171	-5.276717	-3.559244
H	-2.886948	-0.305648	-1.410013
C	-4.332774	-0.960761	0.557570
H	-4.398141	-2.019866	0.758902
H	-5.075903	-0.566220	-0.123569
C	-3.803748	-0.131166	1.532298
C	-2.950776	-0.623522	2.627001
C	-2.520461	-1.963079	2.683766
C	-2.555677	0.256770	3.652724
C	-1.731980	-2.407672	3.740640
H	-2.779488	-2.657548	1.893876
C	-1.768155	-0.192349	4.706325
H	-2.868166	1.292851	3.606309
C	-1.354982	-1.526365	4.755198
H	-1.400068	-3.441486	3.764679
H	-1.459067	0.500552	5.482720
H	-0.728841	-1.871468	5.572516
O	-3.999845	1.178221	1.546783
Si	-4.987124	2.339955	0.749661
C	-6.697116	2.179621	1.523566
H	-6.659857	2.329524	2.608403
H	-7.381668	2.928435	1.106131
H	-7.135352	1.192123	1.337162
C	-5.067381	2.108873	-1.113687
H	-4.071408	2.058151	-1.566441
H	-5.578422	2.979931	-1.545435
H	-5.640524	1.224442	-1.414455
H	-0.005042	0.366255	1.537713
C	-4.137139	3.947307	1.198174
H	-4.639751	4.807188	0.739606
H	-3.100531	3.927644	0.844928
H	-4.122209	4.104629	2.282516

TS 249

B3LYP/6-31G(d) = -3573.917108
B3LYP-D3(BJ)/def2-TZVPP/IEFPCM(propanonitrile) = -3575.496047
B3LYP-D3(BJ)/def2-TZVPP/IEFPCM(propanonitrile)//B3LYP-D3(BJ)/6-31G(d) Free Energy (Quasiharmonic) = -3574.779422

Frequencies (Top 3 out of 300)

1. -259.4195 cm^{-1}
2. 9.6242 cm^{-1}
3. 12.0809 cm^{-1}

B3LYP/6-31G(d) Molecular Geometry in Cartesian Coordinates

B	-0.299662	0.207270	0.204455
O	-0.259169	0.276098	1.686098
N	-0.109837	1.730370	-0.143517
C	-0.325238	2.551872	1.063242
S	0.044306	2.445935	-1.608909
C	-0.207108	1.511189	2.175413
O	-0.063419	1.776383	3.351918
H	-1.593115	4.170075	0.418075
H	0.500525	3.262666	1.194228
C	-1.644994	3.374813	1.167608
C	-2.930329	2.616685	1.006368
C	-3.673743	1.892720	2.013729
C	-4.814251	1.335074	1.368785
N	-4.772543	1.731198	0.046213
C	-3.632814	2.477765	-0.165881
H	-3.390794	2.854626	-1.148919
C	-5.543044	0.332499	3.405775
C	-3.496869	1.660720	3.390060
H	-6.608382	0.124048	1.534715
H	-5.338856	1.351971	-0.697919
C	-4.429688	0.886333	4.069450
H	-6.249329	-0.277962	3.962090
H	-2.629773	2.058646	3.909157
C	-5.751246	0.551290	2.048856
H	-4.295397	0.695937	5.131112
C	1.375147	3.647141	-1.399647
O	-1.130416	3.236946	-2.014903
O	0.504444	1.394289	-2.541161
C	3.479298	5.500689	-1.262956
C	1.260387	4.876586	-2.050877
C	2.528028	3.335853	-0.675620
C	3.568063	4.261444	-0.613078
C	2.311367	5.789510	-1.981339
H	0.353842	5.107226	-2.599317
H	2.603017	2.389647	-0.149729
H	4.464471	4.019518	-0.046445
H	2.220070	6.744861	-2.492523
C	4.597622	6.510526	-1.163621
H	5.565566	6.024093	-1.004594
H	4.667744	7.120797	-2.069981
H	4.434669	7.196472	-0.321782
H	-1.603621	3.861225	2.150811
C	-1.637358	-0.549462	-0.307281
C	-2.284556	-0.300069	-1.524722
C	-2.187446	-1.545920	0.517286
C	-3.437650	-1.003161	-1.892123
C	-3.321871	-2.268119	0.142853
H	-1.726068	-1.751956	1.477384
C	-3.960327	-1.997127	-1.066897
H	-4.844455	-2.551173	-1.361062
C	-3.911343	-3.293262	1.071894
C	-4.184722	-0.606449	-3.131546
F	-4.495468	-4.310003	0.390705
F	-2.978044	-3.841125	1.883847
F	-4.866597	-2.765576	1.870828
F	-5.062421	0.413434	-2.873712
F	-3.372143	-0.165560	-4.111744
F	-4.924377	-1.617798	-3.637703
O	0.882356	-0.719573	-0.092334
C	1.358914	-1.140509	-1.232774
C	1.502716	-2.609287	-1.416430
C	1.824364	-3.123114	-2.682225
C	1.239697	-3.495275	-0.362350
C	1.894778	-4.498307	-2.887777
H	2.003143	-2.440892	-3.510613
C	1.306556	-4.872083	-0.571503
H	0.952262	-3.092686	0.603101
C	1.638242	-5.376399	-1.830672
H	2.136300	-4.886652	-3.873294
H	1.085892	-5.552649	0.246282
H	1.683607	-6.449860	-1.992898
H	1.069855	-0.580252	-2.123330
C	3.222540	-0.370772	-1.382388
H	3.584564	-0.840071	-2.289979
H	2.877712	0.651061	-1.482989
C	3.806476	-0.763251	-0.175323
C	4.645676	-1.977565	-0.090165

C	5.618263	-2.220872	-1.076136
C	4.473553	-2.915230	0.942284
C	6.413944	-3.362200	-1.016489
H	5.770012	-1.496449	-1.870374
C	5.257196	-4.065435	0.987418
H	3.698245	-2.762407	1.683876
C	6.233653	-4.287706	0.013670
H	7.174345	-3.529118	-1.773776
H	5.101385	-4.792026	1.779513
H	6.849518	-5.181597	0.055704
O	3.468349	-0.098099	0.904307
Si	3.858224	0.117055	2.592555
C	5.675215	-0.295152	2.865266
H	5.952642	0.003302	3.884399
H	5.904376	-1.358451	2.754564
H	6.319735	0.263445	2.176234
C	2.670948	-0.920747	3.603485
H	2.746867	-0.620563	4.656484
H	1.639790	-0.731412	3.285064
H	2.864038	-1.997483	3.555497
H	-1.903088	0.454832	-2.203812
C	3.554641	1.941638	2.888885
H	4.024734	2.573140	2.127156
H	2.484223	2.168190	2.926915
H	3.978844	2.226860	3.860154

TS 250

B3LYP/6-31G(d) = -3573.924385
B3LYP-D3(BJ)/def2-TZVPP/IEFPCM(propanonitrile) = -3575.494984
B3LYP-D3(BJ)/def2-TZVPP/IEFPCM(propanonitrile)//B3LYP-D3(BJ)/6-31G(d) Free Energy (Quasiharmonic) = -3574.779386

Frequencies (Top 3 out of 300)

1. -244.3507 cm^{-1}
2. 5.7620 cm^{-1}
3. 7.9645 cm^{-1}

B3LYP/6-31G(d) Molecular Geometry in Cartesian Coordinates

B	0.149731	-1.014691	-0.615240
O	0.278535	-1.920736	-1.776532
N	0.450887	0.372850	-1.295818
C	0.743927	0.191459	-2.731017
S	-0.027618	1.845667	-0.751963
C	0.505834	-1.305717	-2.938227
O	0.567534	-1.867852	-4.009358
H	2.327757	1.635572	-3.049631
H	0.021870	0.747505	-3.340118
C	2.169757	0.567315	-3.229315
C	3.321962	-0.219613	-2.654334
C	4.446055	0.302739	-1.911273
C	5.282225	-0.798608	-1.575457
N	4.710047	-1.925682	-2.125790
C	3.536483	-1.575133	-2.764547
H	2.946497	-2.325965	-3.270288
C	6.823739	0.632970	-0.455642
C	4.848331	1.591455	-1.518899
H	7.068944	-1.513731	-0.573972
H	4.988904	-2.873711	-1.923172
C	6.027811	1.744451	-0.801181
H	7.736347	0.783726	0.114968
H	4.246964	2.459536	-1.771594
C	6.462271	-0.652508	-0.838975
H	6.345802	2.739137	-0.498941
C	1.388293	2.908483	-0.440924
O	-0.649655	1.627411	0.571760
O	-0.821528	2.515618	-1.803930
C	3.458276	4.699739	0.150420
C	1.651114	3.967893	-1.308969
C	2.142610	2.730059	0.723034
C	3.169608	3.624136	1.005512
C	2.687705	4.853423	-1.008406
H	1.035292	4.107485	-2.190749
H	1.924154	1.919545	1.409271
H	3.756180	3.484429	1.909961
H	2.890721	5.682852	-1.681388
C	4.566456	5.668702	0.487130
H	5.512849	5.143589	0.662507
H	4.337127	6.230126	1.401629
H	4.726063	6.392362	-0.317888
H	2.128823	0.441223	-4.318934
C	1.122939	-1.425112	0.598647
C	1.047254	-0.826613	1.861347
C	2.139281	-2.373044	0.398446
C	1.971916	-1.127992	2.868973
H	0.269543	-0.095609	2.060139
C	3.057464	-2.683986	1.401085
H	2.214865	-2.865268	-0.562949
C	2.985030	-2.056305	2.647572
H	3.702724	-2.286432	3.427061
C	4.171938	-3.661612	1.156115
C	1.888640	-0.374919	4.165216
F	4.222631	-4.622185	2.107328
F	4.061573	-4.293161	-0.040785
F	5.385998	-3.055766	1.160865
F	2.704454	-0.877956	5.116556
F	0.632934	-0.372628	4.670479
F	2.233648	0.931052	3.999910
O	-1.290088	-1.218784	-0.125146
C	-2.390091	-0.802488	-0.684730
C	-3.328987	-1.814310	-1.206201
C	-4.359284	-1.426429	-2.078828
C	-3.156185	-3.173523	-0.900611
C	-5.200321	-2.383018	-2.639504
H	-4.488503	-0.374949	-2.324424
C	-4.004850	-4.126534	-1.458732
H	-2.337316	-3.466356	-0.252413
C	-5.026472	-3.734614	-2.327601
H	-5.986483	-2.077870	-3.324142
H	-3.859740	-5.178356	-1.228800
H	-5.680227	-4.481426	-2.769810
H	-2.357442	0.146716	-1.228768
C	-3.324494	0.033739	0.998774
H	-2.563470	0.801800	1.065305
H	-3.157855	-0.840808	1.614098
C	-4.634805	0.406070	0.715122
C	-5.792634	-0.494470	0.850368
C	-5.670750	-1.784801	1.404077
C	-7.065302	-0.062717	0.426065
C	-6.782916	-2.611349	1.524591
H	-4.708728	-2.149025	1.743675
C	-8.174747	-0.891838	0.549558
H	-7.168226	0.926307	-0.003743
C	-8.037587	-2.169407	1.098362
H	-6.670345	-3.602657	1.953534
H	-9.148181	-0.543010	0.216708
H	-8.904654	-2.816828	1.196150
O	-4.917526	1.584214	0.173361
Si	-4.530262	3.255184	0.439848
C	-3.305038	3.446502	1.845750
H	-3.636156	2.923993	2.750944
H	-2.304573	3.088479	1.585026
H	-3.225543	4.512321	2.097058
C	-6.194000	4.006901	0.899883
H	-6.593516	3.571489	1.823141
H	-6.095642	5.087893	1.059697
H	-6.936215	3.857323	0.107193
C	-3.917677	3.904824	-1.203003
H	-3.788599	4.993457	-1.148864

H	-4.640175	3.699350	-2.001696
H	-2.953473	3.467454	-1.483202

TS 251

B3LYP/6-31G(d) = -3573.917282
B3LYP-D3(BJ)/def2-TZVPP/IEFPCM(propanonitrile) = -3575.494862
B3LYP-D3(BJ)/def2-TZVPP/IEFPCM(propanonitrile)//B3LYP-D3(BJ)/6-31G(d) Free Energy (Quasiharmonic) = -3574.77918

Frequencies (Top 3 out of 300)

1. -269.9947 cm^{-1}
2. 6.1003 cm^{-1}
3. 7.9629 cm^{-1}

B3LYP/6-31G(d) Molecular Geometry in Cartesian Coordinates

B	0.272679	-0.017473	-0.499811
O	0.482866	0.053997	-1.970886
N	-0.312248	-1.456368	-0.333847
C	-0.362264	-2.127866	-1.641883
S	-0.088439	-2.390596	1.025030
C	0.269457	-1.110358	-2.588502
O	0.530285	-1.310448	-3.758482
H	-2.215325	-3.228236	-1.450369
H	0.268391	-3.023487	-1.634812
C	-1.767262	-2.538865	-2.172663
C	-2.724592	-1.413774	-2.470107
C	-3.934932	-1.078152	-1.756762
C	-4.502327	0.058995	-2.395839
N	-3.689761	0.372183	-3.464535
C	-2.622556	-0.504515	-3.498260
H	-1.866460	-0.420104	-4.266172
C	-6.315227	0.098205	-0.849390
C	-4.607807	-1.633116	-0.654199
H	-6.082889	1.545776	-2.441198
H	-3.765807	1.209990	-4.019636
C	-5.785701	-1.043757	-0.213431
H	-7.233746	0.542456	-0.474932
H	-4.212272	-2.508621	-0.149248
C	-5.681861	0.663750	-1.949161
H	-6.307345	-1.464710	0.642282
C	-1.711169	-2.967894	1.558650
O	0.672314	-3.601825	0.666936
O	0.389886	-1.506782	2.097289
C	-4.168348	-3.922172	2.517777
C	-2.518655	-2.136509	2.338805
C	-2.109920	-4.272256	1.264543
C	-3.335925	-4.738232	1.740331
C	-3.736303	-2.620098	2.811156
H	-2.197169	-1.132152	2.591055
H	-1.450896	-4.918442	0.694542
H	-3.642535	-5.757249	1.515938
H	-4.360051	-1.973475	3.423511
C	-5.503483	-4.423880	3.015236
H	-5.742859	-4.013680	4.002155
H	-6.314071	-4.128595	2.335763
H	-5.520377	-5.516260	3.087100
H	-1.577318	-3.121640	-3.083135
C	-0.683026	1.156568	0.058370
C	-0.870687	1.358462	1.433655
C	-1.411069	1.978333	-0.812454
C	-1.764079	2.319612	1.914034
C	-2.278147	2.965995	-0.336206
H	-1.298886	1.844581	-1.883788
C	-2.469261	3.139468	1.034000
H	-3.151687	3.892936	1.406799
C	-2.947352	3.883274	-1.318865
C	-1.974226	2.421910	3.397619
F	-3.529077	3.207706	-2.344679
F	-2.055148	4.732427	-1.893215
F	-3.907003	4.645601	-0.753845
F	-2.569179	1.305143	3.893544
F	-2.754657	3.469455	3.743969
F	-0.803947	2.555853	4.065486
O	1.661381	0.006994	0.139488
C	2.563057	0.926256	0.315086
C	2.354097	2.380632	0.035582
C	2.744392	3.290801	1.030120
C	1.802181	2.867253	-1.159943
C	2.568744	4.662137	0.845061
H	3.159770	2.920797	1.964115
C	1.630832	4.237193	-1.342420
H	1.497973	2.171983	-1.935128
C	2.012725	5.137197	-0.343280
H	2.857568	5.354822	1.630540
H	1.184654	4.603869	-2.262248
H	1.871825	6.204425	-0.490220
H	3.165834	0.747837	1.210530
C	4.079641	0.571675	-0.984751
H	3.477918	0.570313	-1.884902
H	4.665044	1.471577	-0.830916
C	4.636413	-0.640564	-0.559084
C	4.153801	-1.953699	-0.979789
C	3.402426	-2.109741	-2.162020
C	4.463719	-3.096120	-0.212060
C	2.973848	-3.371280	-2.562124
H	3.161021	-1.258399	-2.787064
C	4.021010	-4.350115	-0.609555
H	5.030530	-2.980510	0.704112
C	3.278472	-4.491443	-1.785905
H	2.387773	-3.464994	-3.470692
H	4.243269	-5.219348	0.001858
H	2.933420	-5.474636	-2.093317
O	5.578653	-0.671048	0.378502
Si	6.876905	0.363667	0.854600
C	7.934320	-0.775232	1.900598
H	8.809880	-0.244241	2.293228
H	7.370115	-1.163967	2.755826
H	8.294379	-1.630717	1.318606
C	6.218219	1.801170	1.869073
H	5.629705	1.444639	2.722518
H	7.056689	2.384070	2.271100
H	5.592051	2.485507	1.286983
H	-0.325010	0.738867	2.139351
C	7.768510	0.930675	-0.700141
H	8.110233	0.074756	-1.293167
H	7.142154	1.556954	-1.344202
H	8.653457	1.519829	-0.429420

TS 252

B3LYP/6-31G(d) = -3573.912783
B3LYP-D3(BJ)/def2-TZVPP/IEFPCM(propanonitrile) = -3575.494638
B3LYP-D3(BJ)/def2-TZVPP/IEFPCM(propanonitrile)//B3LYP-D3(BJ)/6-31G(d) Free Energy (Quasiharmonic) = -3574.779167

Frequencies (Top 3 out of 300)

1. -281.3477 cm^{-1}
2. 9.8444 cm^{-1}
3. 12.3692 cm^{-1}

B3LYP/6-31G(d) Molecular Geometry in Cartesian Coordinates

B	0.147256	-0.483376	0.206720
O	-0.022065	-0.369344	1.675104
N	-0.044913	-2.024295	0.021481
C	-0.016572	-2.706281	1.325563

S	-0.249427	-2.867935	-1.360215
C	-0.153555	-1.536034	2.303162
O	-0.358593	-1.652588	3.493517
H	1.155488	-4.486526	1.021713
H	-0.903275	-3.341400	1.454963
C	1.231763	-3.585501	1.636753
C	2.564728	-2.935438	1.405826
C	3.315714	-2.081912	2.299086
C	4.499026	-1.694783	1.608129
N	4.474613	-2.310268	0.371779
C	3.301387	-3.027265	0.250202
H	3.063638	-3.554940	-0.662521
C	5.215778	-0.378036	3.463128
C	3.109907	-1.601713	3.605453
H	6.348404	-0.558215	1.628082
H	5.065382	-2.064801	-0.409032
C	4.057100	-0.756587	4.171265
H	5.935107	0.289169	3.930658
H	2.209982	-1.865562	4.153541
C	5.454128	-0.845577	2.175543
H	3.900428	-0.373824	5.176428
C	-1.858856	-3.669089	-1.202599
O	-0.334966	-1.873367	-2.450333
O	0.716683	-3.970210	-1.505165
C	-4.373206	-4.911917	-1.109338
C	-2.932219	-3.014815	-0.596928
C	-2.024922	-4.941464	-1.753811
C	-3.277069	-5.550284	-1.706473
C	-4.177163	-3.641712	-0.550887
H	-2.788055	-2.039409	-0.142311
H	-1.174397	-5.445625	-2.199406
H	-3.402494	-6.542897	-2.132736
H	-5.006062	-3.138199	-0.060100
C	-5.730182	-5.574493	-1.080197
H	-6.371953	-5.142220	-0.305808
H	-5.646889	-6.650020	-0.890045
H	-6.248439	-5.455906	-2.041031
O	-1.007705	0.379374	-0.315574
C	-1.316889	0.818342	-1.514347
H	-1.272030	0.084405	-2.323131
H	1.122092	-3.898054	2.683018
C	-0.905470	2.194671	-1.892542
C	-0.377327	3.080172	-0.941786
C	-0.967925	2.592347	-3.238621
C	0.089660	4.335542	-1.330125
H	-0.293100	2.757091	0.088956
C	-0.517077	3.850823	-3.623690
H	-1.353123	1.901734	-3.985954
C	0.013105	4.725828	-2.668925
H	0.542778	4.991665	-0.592648
H	-0.559317	4.145185	-4.668557
H	0.383648	5.700849	-2.973516
C	1.548627	0.185366	-0.255720
C	2.088128	1.207343	0.539815
C	2.261882	-0.164038	-1.412249
C	3.260994	1.879262	0.185712
H	1.587731	1.476106	1.465132
C	3.461483	0.473127	-1.745106
H	1.882533	-0.941255	-2.067061
C	3.963659	1.511098	-0.958493
H	4.882983	2.016312	-1.229702
C	4.270599	-0.023283	-2.907382
C	3.716379	3.039187	1.023633
F	5.047110	0.942541	-3.447663
F	5.124870	-1.025309	-2.528738
F	2.933574	4.136826	0.811652
F	3.507605	-0.531555	-3.894610
F	4.985634	3.412951	0.749044
F	3.645891	2.775861	2.344633
C	-3.327531	0.895959	-1.617065
H	-3.423255	1.464383	-2.535942
H	-3.547865	-0.164665	-1.678632
C	-3.653013	1.539469	-0.419024
C	-3.934768	0.768044	0.811259
C	-5.034491	-0.107265	0.842812
C	-3.084771	0.856736	1.927097
C	-5.302858	-0.847973	1.992436
H	-5.686843	-0.182572	-0.022860
C	-3.328692	0.071052	3.053363
H	-2.191907	1.467353	1.881726
C	-4.444987	-0.766688	3.093329
H	-6.172597	-1.498261	2.024778
H	-2.621634	0.084218	3.876361
H	-4.635993	-1.370998	3.975438
O	-3.550891	2.848362	-0.376349
Si	-4.108730	4.150459	0.638456
C	-2.930531	4.399767	2.076443
H	-1.890402	4.449483	1.736182
H	-3.159511	5.351742	2.572308
H	-3.009164	3.607054	2.826241
C	-4.048902	5.593574	-0.553984
H	-4.334069	6.527342	-0.054486
H	-3.037582	5.720396	-0.956079
H	-4.729507	5.443317	-1.399311
C	-5.857078	3.750210	1.196822
H	-6.268253	4.601996	1.753115
H	-6.519495	3.565084	0.343418
H	-5.893945	2.875556	1.853762

TS 253

B3LYP/6-31G(d) = -3573.919256
B3LYP-D3(BJ)/def2-TZVPP/IEFPCM(propanonitrile) = -3575.495233
B3LYP-D3(BJ)/def2-TZVPP/IEFPCM(propanonitrile)//B3LYP-D3(BJ)/6-31G(d) Free Energy (Quasiharmonic) = -3574.779162

Frequencies (Top 3 out of 300)

1. -254.9378 cm^{-1}
2. 12.4333 cm^{-1}
3. 13.7688 cm^{-1}

B3LYP/6-31G(d) Molecular Geometry in Cartesian Coordinates

B	0.590190	0.724532	-0.125619
O	0.214102	0.656291	-1.552991
N	1.567684	1.909552	-0.109828
C	1.795101	2.417904	-1.468817
S	2.004673	2.812256	1.202336
C	0.833936	1.574133	-2.311672
O	0.651606	1.692097	-3.502123
H	3.880816	2.950721	-1.420236
H	1.480947	3.468884	-1.561507
C	3.240743	2.304741	-2.025743
C	3.798638	0.909385	-2.073369
C	4.675773	0.279557	-1.112819
C	4.931181	-1.038888	-1.578744
N	4.254405	-1.190309	-2.772855
C	3.567936	-0.024496	-3.056783
H	2.969107	0.059740	-3.953613
C	6.321568	-1.492335	0.302050
C	5.273804	0.701332	0.089235
H	5.912284	-2.947282	-1.247039
H	4.145456	-2.063486	-3.264285
C	6.089500	-0.185565	0.779837
H	6.959337	-2.164295	0.870599
H	5.081094	1.695700	0.479765
C	5.745364	-1.937896	-0.881078
H	6.551170	0.128368	1.712198
C	0.850040	4.195839	1.284223
O	3.318796	3.416252	0.937734

O	1.774913	1.981465	2.388776
C	-0.956131	6.340411	1.396315
C	1.152526	5.391555	0.630843
C	-0.337177	4.065398	2.010380
C	-1.227105	5.134186	2.061731
C	0.245665	6.451360	0.687327
H	2.100855	5.499073	0.115335
H	-0.539423	3.141830	2.540610
H	-2.145256	5.036394	2.636867
H	0.485383	7.384229	0.182700
C	-1.940540	7.484794	1.449933
H	-1.508696	8.404598	1.043796
H	-2.261635	7.687785	2.478218
H	-2.844504	7.257929	0.869792
H	3.202129	2.732148	-3.035235
C	1.087712	-0.721319	0.407114
C	1.084201	-1.063455	1.768881
C	1.418710	-1.731738	-0.505372
C	1.355671	-2.368100	2.189885
H	0.853898	-0.300810	2.505420
C	1.707617	-3.034776	-0.087138
H	1.420671	-1.505341	-1.566386
C	1.667847	-3.366990	1.265331
H	1.863457	-4.380608	1.593158
C	2.014854	-4.077341	-1.124091
C	1.177874	-2.716561	3.640214
F	3.231409	-3.898175	-1.703464
F	1.111635	-4.045679	-2.139701
F	2.005241	-5.329525	-0.621562
F	-0.149038	-2.881850	3.939568
F	1.632087	-1.754112	4.463908
F	1.789452	-3.871505	3.977814
O	-0.679628	1.055222	0.723901
C	-1.950295	0.828928	0.614707
C	-2.827913	1.620456	-0.277956
C	-2.315535	2.604081	-1.137586
C	-4.222857	1.468454	-0.169793
C	-3.177229	3.395584	-1.893670
H	-1.248004	2.765464	-1.187783
C	-5.081899	2.262079	-0.923643
H	-4.632688	0.731375	0.515272
C	-4.559763	3.223671	-1.794670
H	-2.766878	4.153353	-2.554563
H	-6.156828	2.137058	-0.828396
H	-5.229440	3.845058	-2.382986
H	-2.427710	0.524227	1.551700
C	-2.012495	-1.182218	-0.109572
H	-1.544436	-0.972984	-1.063483
H	-1.336217	-1.486626	0.675846
C	-3.310653	-1.664831	-0.085566
C	-3.970477	-2.150838	1.141432
C	-3.242000	-2.399463	2.323195
C	-5.360522	-2.377426	1.143322
C	-3.889753	-2.853670	3.467740
H	-2.165967	-2.269909	2.359240
C	-6.003280	-2.824815	2.293909
H	-5.924505	-2.195759	0.235704
C	-5.271070	-3.064055	3.459420
H	-3.308068	-3.049816	4.363481
H	-7.076811	-2.990595	2.280914
H	-5.773057	-3.418949	4.355186
O	-4.090768	-1.625441	-1.160581
Si	-3.703527	-1.818040	-2.850841
C	-2.938794	-0.266764	-3.571942
H	-3.546629	0.616907	-3.349354
H	-1.917295	-0.065073	-3.233131
H	-2.901284	-0.371936	-4.664523
C	-5.405338	-2.132450	-3.577616
H	-6.073418	-1.281421	-3.402776
H	-5.341610	-2.285977	-4.661584
H	-5.870602	-3.023009	-3.140695
C	-2.588487	-3.324897	-2.998240
H	-2.418331	-3.549143	-4.058916
H	-1.605491	-3.187229	-2.535226
H	-3.053725	-4.208694	-2.546488

TS 254

B3LYP/6-31G(d) = -3573.915355
B3LYP-D3(BJ)/def2-TZVPP/IEFPCM(propanonitrile) = -3575.495151
B3LYP-D3(BJ)/def2-TZVPP/IEFPCM(propanonitrile)//B3LYP-D3(BJ)/6-31G(d) Free Energy (Quasiharmonic) = -3574.779012

Frequencies (Top 3 out of 300)

1. -298.2169 cm^{-1}
2. 7.5199 cm^{-1}
3. 13.2215 cm^{-1}

B3LYP/6-31G(d) Molecular Geometry in Cartesian Coordinates

B	0.265719	-0.730558	-0.405722
O	0.739229	-0.997444	-1.786805
N	-1.068867	-1.581385	-0.351289
C	-1.320707	-2.145968	-1.692299
S	-1.389053	-2.553818	0.949069
C	-0.059571	-1.797449	-2.484254
O	0.171570	-2.173709	-3.616939
H	-3.457806	-1.836598	-1.832281
H	-1.398755	-3.239146	-1.656806
C	-2.572920	-1.582748	-2.424464
C	-2.537614	-0.102076	-2.700479
C	-3.269141	0.939204	-2.017694
C	-2.867729	2.179751	-2.586561
N	-1.964762	1.896870	-3.590391
C	-1.757883	0.531808	-3.640262
H	-1.061260	0.107065	-4.349700
C	-4.285487	3.388954	-1.098651
C	-4.221179	0.953380	-0.983582
H	-3.021335	4.343201	-2.575160
H	-1.372305	2.583796	-4.031522
C	-4.715896	2.171077	-0.532666
H	-4.682452	4.325864	-0.717573
H	-4.559870	0.024282	-0.535266
C	-3.360859	3.408581	-2.136411
H	-5.443890	2.189784	0.274124
C	-3.174910	-2.748219	0.994727
O	-0.829885	-3.910551	0.769097
O	-1.001066	-1.783948	2.146938
C	-5.952135	-3.086129	1.147779
C	-3.751295	-3.935195	0.543844
C	-3.964177	-1.731481	1.540319
C	-5.342989	-1.909576	1.613054
C	-5.135798	-4.092850	0.618174
H	-3.118269	-4.728469	0.160979
H	-3.505215	-0.820057	1.908518
H	-5.958181	-1.122863	2.043398
H	-5.586021	-5.018388	0.267827
C	-7.451919	-3.250411	1.210474
H	-7.852162	-2.905764	2.170504
H	-7.945365	-2.662478	0.425603
H	-7.747252	-4.295350	1.074527
H	-2.647999	-2.144047	-3.364130
C	0.020834	0.844856	-0.177377
C	-0.798736	1.337717	0.848788
C	0.594749	1.782124	-1.046272
C	-1.028022	2.705044	1.004951
C	0.366584	3.154263	-0.896440
H	1.214282	1.432760	-1.865868
C	-0.446377	3.626653	0.133195
H	-0.636696	4.687556	0.245976

C	0.924253	4.110562	-1.907492
C	-1.866191	3.192967	2.152618
F	0.115424	4.229672	-3.003038
F	2.132055	3.713396	-2.380551
F	1.077160	5.358526	-1.415796
F	-1.116387	3.396855	3.267275
F	-2.831393	2.309819	2.492857
F	-2.473741	4.370361	1.877853
O	1.390101	-1.324626	0.429722
C	1.785111	-1.464835	1.669941
C	1.842618	-0.359867	2.660461
C	2.088601	0.979921	2.324868
C	1.632221	-0.699421	4.008278
C	2.103988	1.958751	3.317510
H	2.250939	1.256703	1.291460
C	1.649990	0.279322	4.997124
H	1.420774	-1.732905	4.270536
C	1.884898	1.613295	4.652069
H	2.278502	2.995245	3.044505
H	1.466437	0.006275	6.032505
H	1.889350	2.381421	5.420605
H	1.511669	-2.420617	2.122651
C	3.736822	-2.071989	1.553297
H	4.102165	-1.726651	2.512273
H	3.572849	-3.141863	1.480102
C	4.181079	-1.411922	0.398581
C	4.697382	-0.034468	0.413748
C	4.446898	0.800928	-0.692274
C	5.402882	0.477416	1.517042
C	4.864587	2.127931	-0.676645
H	3.891045	0.405330	-1.534696
C	5.851232	1.795622	1.510974
H	5.622917	-0.160024	2.367368
C	5.574053	2.624662	0.421231
H	4.619929	2.778487	-1.510696
H	6.409198	2.178712	2.360321
H	5.908234	3.658502	0.428101
O	3.983561	-1.899238	-0.798827
Si	3.820627	-3.487891	-1.513369
C	5.510454	-4.277075	-1.242469
H	5.533232	-5.281609	-1.683006
H	6.307090	-3.689656	-1.712504
H	5.750068	-4.381336	-0.177688
C	3.507034	-3.098844	-3.311705
H	2.533720	-2.621181	-3.470398
H	4.287758	-2.438984	-3.707440
H	3.524159	-4.023964	-3.901961
H	-1.261270	0.638660	1.537306
C	2.466358	-4.506472	-0.708664
H	2.794108	-4.973930	0.227229
H	1.560938	-3.930481	-0.497237
H	2.186928	-5.319619	-1.391246

TS 255

B3LYP/6-31G(d) = -3573.924259
B3LYP-D3(BJ)/def2-TZVPP/IEFPCM(propanonitrile) = -3575.496305
B3LYP-D3(BJ)/def2-TZVPP/IEFPCM(propanonitrile)//B3LYP-D3(BJ)/6-31G(d) Free Energy (Quasiharmonic) = -3574.778999

Frequencies (Top 3 out of 300)

1. -274.6292 cm^{-1}
2. 7.8219 cm^{-1}
3. 12.1725 cm^{-1}

B3LYP/6-31G(d) Molecular Geometry in Cartesian Coordinates

B	-0.326707	0.195863	0.400220
O	-0.491340	-0.518540	1.693196
N	0.350480	-0.936191	-0.464031
C	0.369076	-2.207091	0.284159
S	0.528403	-1.008584	-2.100947
C	-0.251499	-1.822046	1.625534
O	-0.487669	-2.608805	2.522399
H	2.051245	-3.301575	-0.503741
H	-0.302159	-2.934755	-0.188947
C	1.730898	-2.922557	0.473095
C	2.861211	-2.143487	1.091933
C	4.267672	-2.420156	0.905902
C	4.995204	-1.474613	1.684603
N	4.068687	-0.681790	2.326240
C	2.799344	-1.085088	1.963340
H	1.944530	-0.572729	2.371027
C	7.072902	-2.410315	0.976962
C	4.985147	-3.376950	0.167460
H	6.929149	-0.722960	2.323392
H	4.260151	0.146061	2.870956
C	6.375113	-3.364807	0.208026
H	8.159518	-2.426079	0.992394
H	4.461504	-4.119556	-0.428816
C	6.393129	-1.454663	1.724182
H	6.935711	-4.108252	-0.353085
C	2.271408	-1.205285	-2.477866
O	0.131076	0.310665	-2.636815
O	-0.158844	-2.212831	-2.611304
C	4.970799	-1.480987	-3.162004
C	2.696206	-2.349009	-3.153043
C	3.169619	-0.182899	-2.160528
C	4.508847	-0.330597	-2.504133
C	4.043778	-2.477499	-3.489923
H	1.975919	-3.116861	-3.413508
H	2.830462	0.720200	-1.665192
H	5.210326	0.460975	-2.253635
H	4.378469	-3.368590	-4.015540
C	6.437484	-1.649032	-3.472944
H	6.973844	-2.004760	-2.583645
H	6.896039	-0.701067	-3.774664
H	6.599292	-2.377771	-4.273786
H	1.501012	-3.811301	1.078534
C	0.550831	1.544222	0.610559
C	0.948799	2.390506	-0.436557
C	1.002813	1.879325	1.896157
C	1.796744	3.480612	-0.215629
H	0.617438	2.183800	-1.448884
C	1.865052	2.956570	2.119857
H	0.683852	1.277831	2.741430
C	2.275206	3.766377	1.062166
H	2.946032	4.600452	1.228769
C	2.426645	3.165657	3.495944
C	2.265667	4.283572	-1.396875
F	2.944529	4.399270	3.667379
F	3.441621	2.283264	3.756025
F	1.506127	2.969926	4.463024
F	2.813666	5.464992	-1.033747
F	3.212415	3.611745	-2.102496
F	1.260921	4.551666	-2.259305
O	-1.759352	0.571159	0.056804
C	-2.376599	0.768158	-1.084036
C	-3.408313	1.845931	-1.086866
C	-3.842934	2.433301	0.108829
C	-3.901858	2.329139	-2.307954
C	-4.763392	3.480021	0.081118
H	-3.430213	2.078804	1.047404
C	-4.822042	3.374666	-2.333759
H	-3.547394	1.896347	-3.241212
C	-5.257895	3.950390	-1.137393
H	-5.087864	3.936924	1.012196
H	-5.189541	3.748631	-3.285427
H	-5.970401	4.770665	-1.156469
H	-1.759871	0.729283	-1.983460

C	-3.295489	-0.880929	-1.621714
H	-2.422941	-1.504032	-1.801427
H	-3.792006	-0.511231	-2.512329
C	-4.119975	-1.247034	-0.546824
C	-5.497987	-0.743238	-0.406044
C	-5.982842	-0.358591	0.857884
C	-6.334673	-0.607077	-1.528197
C	-7.271700	0.148007	0.993532
H	-5.330186	-0.423818	1.721831
C	-7.630287	-0.118194	-1.384855
H	-5.981168	-0.918114	-2.506048
C	-8.100112	0.262896	-0.125909
H	-7.629071	0.458937	1.970915
H	-8.273996	-0.034866	-2.255543
H	-9.109023	0.650897	-0.017487
O	-3.579349	-1.926365	0.438485
Si	-4.031163	-3.237802	1.507437
C	-2.822700	-4.607815	1.097661
H	-2.805543	-4.830422	0.024629
H	-1.813984	-4.334469	1.422620
H	-3.107674	-5.527817	1.623919
C	-5.791902	-3.784109	1.119898
H	-5.926823	-4.003556	0.054309
H	-5.989458	-4.713843	1.669152
H	-6.552075	-3.055193	1.413741
C	-3.812052	-2.594706	3.250177
H	-4.007807	-3.402920	3.966573
H	-2.777283	-2.265670	3.395178
H	-4.488541	-1.767935	3.492753

TS 256

B3LYP/6-31G(d) = -3573.921149
B3LYP-D3(BJ)/def2-TZVPP/IEFPCM(propanonitrile) = -3575.496411
B3LYP-D3(BJ)/def2-TZVPP/IEFPCM(propanonitrile)//B3LYP-D3(BJ)/6-31G(d) Free Energy (Quasiharmonic) = -3574.778911

Frequencies (Top 3 out of 300)

1. -283.6146 cm^{-1}
2. 13.2489 cm^{-1}
3. 15.6427 cm^{-1}

B3LYP/6-31G(d) Molecular Geometry in Cartesian Coordinates

B	-0.374837	0.141216	1.589277
O	-0.654972	-0.500050	2.887804
N	-1.597477	1.118766	1.417400
C	-2.514883	0.921534	2.558199
S	-1.355794	2.676222	0.890706
C	-1.773965	-0.058990	3.473847
O	-2.170328	-0.435550	4.553385
H	-4.417958	1.035147	1.531279
H	-2.663021	1.853272	3.116860
C	-3.906127	0.328153	2.191689
C	-3.868027	-1.040097	1.559922
C	-4.088219	-1.378029	0.173048
C	-3.891963	-2.781471	0.042823
N	-3.601809	-3.270883	1.298757
C	-3.574727	-2.223312	2.198684
H	-3.343636	-2.398682	3.240074
C	-4.324921	-2.686142	-2.300295
C	-4.435368	-0.642382	-0.973443
H	-3.822537	-4.516181	-1.257104
H	-3.256781	-4.200144	1.483840
C	-4.546926	-1.297499	-2.193871
H	-4.412064	-3.169458	-3.269719
H	-4.608453	0.427219	-0.908347
C	-3.999492	-3.446544	-1.182921
H	-4.806375	-0.732201	-3.085187
C	-2.916404	3.187004	0.154332
O	-0.378470	2.604331	-0.220601
O	-1.103279	3.611389	2.000235
C	-5.299668	4.080920	-1.019528
C	-3.232081	2.796052	-1.149770
C	-3.764618	4.031818	0.869928
C	-4.952281	4.466772	0.280941
C	-4.417504	3.247173	-1.724764
H	-2.559705	2.154131	-1.708739
H	-3.482724	4.358508	1.865125
H	-5.613042	5.125870	0.838699
H	-4.661084	2.949541	-2.741991
C	-6.595080	4.536334	-1.647924
H	-7.007152	5.408446	-1.130813
H	-7.351874	3.741896	-1.608757
H	-6.457804	4.798990	-2.702658
H	-4.475806	0.298857	3.128591
C	-0.280308	-0.956624	0.401486
C	-0.478727	-0.597028	-0.940535
C	-0.136099	-2.324591	0.675877
C	-0.514262	-1.549250	-1.960368
H	-0.623627	0.448879	-1.188573
C	-0.181812	-3.285841	-0.340357
H	-0.023905	-2.653603	1.703388
C	-0.366001	-2.904834	-1.669323
H	-0.415605	-3.647823	-2.456324
C	-0.140072	-4.744963	0.010742
C	-0.690711	-1.093206	-3.380409
F	0.603174	-4.987742	1.112257
F	0.359835	-5.500915	-0.991407
F	-1.385814	-5.240876	0.274814
F	0.485845	-0.652629	-3.913742
F	-1.557562	-0.061241	-3.481762
F	-1.133029	-2.078063	-4.189865
O	0.857622	1.009431	1.776475
C	2.148179	1.009987	1.646147
C	3.029225	-0.016671	2.261736
C	2.517683	-1.149841	2.912766
C	4.416471	0.218919	2.285078
C	3.383766	-2.044091	3.541484
H	1.447246	-1.298155	2.978629
C	5.276064	-0.673780	2.918704
H	4.819705	1.112845	1.817885
C	4.761196	-1.815838	3.540367
H	2.976637	-2.913352	4.049978
H	6.344134	-0.475218	2.937453
H	5.429941	-2.512681	4.038314
H	2.559000	2.021964	1.686817
C	2.418312	0.885007	-0.394238
H	2.243390	-0.173182	-0.542487
H	1.554789	1.519638	-0.552406
C	3.676655	1.387185	-0.718037
C	3.993581	2.823962	-0.719845
C	2.993978	3.795518	-0.495552
C	5.319938	3.248031	-0.937840
C	3.323937	5.147787	-0.489792
H	1.958494	3.510101	-0.336552
C	5.639396	4.601450	-0.929728
H	6.089932	2.504951	-1.109712
C	4.642757	5.555205	-0.705086
H	2.544944	5.884189	-0.316924
H	6.665893	4.914401	-1.098429
H	4.893106	6.612509	-0.699607
O	4.715079	0.586081	-0.929713
Si	4.854298	-0.982091	-1.670542
C	3.989205	-0.889064	-3.334031
H	4.174157	-1.813305	-3.896226
H	2.903951	-0.773758	-3.247314
H	4.371496	-0.056430	-3.935650
C	4.165294	-2.343637	-0.577649
H	3.072587	-2.339660	-0.510667

H	4.459783	-3.314080	-0.998792
H	4.565927	-2.288412	0.439759
C	6.716153	-1.140535	-1.841758
H	7.131876	-0.338109	-2.461286
H	7.208680	-1.101816	-0.863555
H	6.982683	-2.095796	-2.309791

TS 257

B3LYP/6-31G(d) = -3573.92334
B3LYP-D3(BJ)/def2-TZVPP/IEFPCM(propanonitrile) = -3575.493974
B3LYP-D3(BJ)/def2-TZVPP/IEFPCM(propanonitrile)//B3LYP-D3(BJ)/6-31G(d) Free Energy (Quasiharmonic) = -3574.778907

Frequencies (Top 3 out of 300)

1. -200.7259 cm^{-1}
2. 8.3758 cm^{-1}
3. 10.9667 cm^{-1}

B3LYP/6-31G(d) Molecular Geometry in Cartesian Coordinates

B	0.033934	-0.339481	-1.029498
O	0.747361	-0.574368	-2.297879
N	1.197230	-0.416045	-0.002858
C	2.492733	-0.569424	-0.693890
S	1.135407	0.161626	1.551713
C	2.070763	-0.727477	-2.161061
O	2.814509	-0.978909	-3.081666
H	3.007335	-2.680694	-0.742440
H	3.092277	0.347663	-0.614325
C	3.366160	-1.776091	-0.237694
C	4.835206	-1.574024	-0.487031
C	5.800648	-1.086778	0.470957
C	7.059908	-1.028789	-0.189918
N	6.856180	-1.460878	-1.484595
C	5.521729	-1.776980	-1.658458
H	5.146642	-2.094755	-2.620132
C	8.117246	-0.215154	1.786867
C	5.724208	-0.693185	1.820230
H	9.178097	-0.562674	-0.066716
H	7.559692	-1.503168	-2.204893
C	6.879946	-0.261341	2.461449
H	9.003096	0.126182	2.316062
H	4.774528	-0.721225	2.347919
C	8.224308	-0.598697	0.454290
H	6.831527	0.046183	3.502825
C	1.479601	1.923510	1.502455
O	2.225949	-0.457348	2.321417
O	-0.260361	-0.023353	1.982170
C	2.003966	4.664275	1.243159
C	2.798011	2.363937	1.351732
C	0.426761	2.838395	1.579724
C	0.698084	4.197666	1.452176
C	3.046836	3.729498	1.216479
H	3.620972	1.656505	1.360390
H	-0.585828	2.487390	1.738165
H	-0.121993	4.909652	1.505556
H	4.070664	4.071648	1.088018
C	2.264313	6.135065	1.026259
H	1.723684	6.751471	1.753604
H	1.924233	6.437485	0.027476
H	3.329773	6.372612	1.103052
O	-0.908897	-1.579853	-0.881723
C	-0.663501	-2.577158	-0.096869
H	-0.350886	-2.352056	0.924133
H	3.196151	-1.914568	0.831251
C	-0.217425	-3.856463	-0.675875
C	0.267174	-4.875593	0.163060
C	-0.192130	-4.034610	-2.069862
C	0.752972	-6.059723	-0.381345
H	0.275787	-4.727623	1.240518
C	0.292703	-5.223660	-2.609380
H	-0.518905	-3.222218	-2.709715
C	0.759402	-6.238110	-1.768943
H	1.135178	-6.839793	0.270682
H	0.321269	-5.354043	-3.687307
H	1.141751	-7.162116	-2.193808
C	-0.838086	1.014651	-1.118972
C	-2.001636	1.256948	-0.379239
C	-0.370973	2.060677	-1.934953
C	-2.658078	2.494142	-0.434961
H	-2.387294	0.483317	0.273825
C	-1.017612	3.295420	-1.990553
H	0.516547	1.907238	-2.539650
C	-2.170634	3.522048	-1.237725
H	-2.676016	4.479577	-1.277538
C	-0.430884	4.426818	-2.788932
C	-3.851799	2.727987	0.443754
F	0.380576	5.204818	-2.025225
F	0.310449	3.992385	-3.828278
F	-4.429802	3.929028	0.238813
F	-1.388547	5.244945	-3.285091
F	-3.523033	2.663418	1.760012
F	-4.820871	1.788448	0.256162
C	-2.612661	-3.386159	0.646556
H	-2.166025	-4.034974	1.386933
H	-2.866543	-3.858287	-0.293356
C	-3.338815	-2.280845	1.057388
O	-4.118868	-1.614717	0.210935
Si	-4.955470	-2.049626	-1.241917
C	-3.752000	-2.200460	-2.674442
H	-4.309223	-2.241853	-3.619032
H	-3.133262	-3.101938	-2.615447
H	-3.077711	-1.339248	-2.715223
C	-5.881630	-3.656512	-0.911575
H	-6.547703	-3.556457	-0.046862
H	-6.504301	-3.907460	-1.779518
H	-5.217405	-4.507200	-0.727431
C	-6.146002	-0.625245	-1.485650
H	-6.870266	-0.567900	-0.665355
H	-5.620823	0.332902	-1.529953
H	-6.706608	-0.751602	-2.419882
C	-3.256727	-1.688296	2.403744
C	-3.949152	-0.495127	2.680608
C	-2.501511	-2.284474	3.432391
C	-3.878121	0.088080	3.941878
H	-4.524970	-0.020971	1.896911
C	-2.440056	-1.703161	4.692702
H	-1.953817	-3.203555	3.254586
C	-3.126603	-0.513704	4.951924
H	-4.401965	1.020657	4.128650
H	-1.848266	-2.172775	5.472774
H	-3.069203	-0.056978	5.936040

TS 258

B3LYP/6-31G(d) = -3573.92168
B3LYP-D3(BJ)/def2-TZVPP/IEFPCM(propanonitrile) = -3575.494764
B3LYP-D3(BJ)/def2-TZVPP/IEFPCM(propanonitrile)//B3LYP-D3(BJ)/6-31G(d) Free Energy (Quasiharmonic) = -3574.778892

Frequencies (Top 3 out of 300)

1. -275.0375 cm^{-1}
2. 7.8042 cm^{-1}
3. 11.6827 cm^{-1}

B3LYP/6-31G(d) Molecular Geometry in Cartesian Coordinates

B	0.126208	-0.143107	-0.064393
O	-0.223071	-0.767451	-1.359426
N	0.567664	1.311366	-0.520669
C	0.605569	1.352299	-1.995960
S	0.022259	2.666572	0.260124
C	0.011473	0.010263	-2.424376
O	-0.225949	-0.315249	-3.565467
H	2.293179	2.617970	-2.449597
H	-0.063198	2.126674	-2.391836
C	2.018534	1.571638	-2.616294
C	3.098666	0.675321	-2.084525
C	3.447783	-0.661250	-2.512292
C	4.492509	-1.114241	-1.657844
N	4.781346	-0.087227	-0.779885
C	3.924927	0.966260	-1.027883
H	3.973766	1.862961	-0.426270
C	4.563652	-3.220277	-2.772879
C	2.981281	-1.521912	-3.523118
H	5.837070	-2.724792	-1.096631
H	5.327141	-0.177996	0.064324
C	3.542111	-2.787571	-3.642796
H	4.972943	-4.220796	-2.883102
H	2.175720	-1.211823	-4.182199
C	5.055328	-2.389245	-1.772756
H	3.181434	-3.461831	-4.415104
C	1.215960	3.954908	-0.129520
O	0.104915	2.420360	1.707136
O	-1.268896	3.130824	-0.307365
C	3.078025	5.973904	-0.687797
C	2.384780	4.046352	0.631385
C	0.953468	4.873368	-1.145027
C	1.888042	5.872604	-1.419507
C	3.304761	5.051761	0.346291
H	2.556114	3.351961	1.447428
H	0.022098	4.814590	-1.697987
H	1.683650	6.589975	-2.210508
H	4.210937	5.128973	0.942432
C	4.099316	7.039622	-1.005806
H	4.545034	7.451928	-0.094063
H	3.655654	7.864856	-1.571405
H	4.919310	6.629838	-1.610063
H	1.899824	1.444682	-3.699531
C	1.268743	-0.960476	0.729224
C	2.049885	-0.334999	1.711435
C	1.570370	-2.294424	0.419589
C	3.106086	-1.004640	2.333098
H	1.841231	0.695883	1.977820
C	2.601776	-2.980787	1.066753
H	1.020118	-2.801807	-0.364290
C	3.383574	-2.336905	2.023785
H	4.205377	-2.855974	2.505425
C	2.886026	-4.423007	0.750018
C	4.049413	-0.251858	3.222562
F	4.217932	-4.668341	0.686773
F	2.347100	-4.812573	-0.422978
F	2.391698	-5.251541	1.705095
F	3.477911	0.818801	3.809720
F	5.115367	0.230031	2.505569
F	4.575520	-1.023160	4.198309
O	-1.103522	-0.016551	0.805259
C	-2.292687	-0.524074	0.925739
C	-2.632464	-1.968872	0.810674
C	-2.049149	-2.865011	-0.100431
C	-3.596250	-2.449212	1.714698
C	-2.410587	-4.211100	-0.082669
H	-1.330868	-2.504080	-0.825681
C	-3.951755	-3.795777	1.730377
H	-4.055221	-1.763969	2.423646
C	-3.355612	-4.681965	0.831710
H	-1.947459	-4.895881	-0.787559
H	-4.687600	-4.152152	2.445688
H	-3.626799	-5.734091	0.842761
H	-2.849820	-0.030931	1.725406
C	-3.327269	0.431904	-0.584622
H	-2.844251	1.387868	-0.405958
H	-2.917504	-0.133250	-1.412226
C	-4.686694	0.313907	-0.302740
C	-5.535634	-0.786506	-0.787833
C	-5.090658	-1.678428	-1.783644
C	-6.831684	-0.954308	-0.260502
C	-5.913989	-2.708312	-2.227655
H	-4.106872	-1.567641	-2.223982
C	-7.650334	-1.985304	-0.707830
H	-7.181888	-0.269904	0.502995
C	-7.193956	-2.866497	-1.691779
H	-5.556281	-3.385674	-2.997320
H	-8.645928	-2.103289	-0.289550
H	-7.835120	-3.670339	-2.042964
O	-5.298714	1.137985	0.537173
Si	-5.003121	2.738657	1.159536
C	-6.677785	3.136133	1.911945
H	-7.468651	3.135124	1.153392
H	-6.951389	2.410282	2.686286
H	-6.665191	4.128584	2.378679
C	-4.591174	3.879011	-0.270924
H	-3.559725	3.752961	-0.612333
H	-5.271578	3.726827	-1.116797
H	-4.703451	4.920114	0.058275
C	-3.670039	2.669691	2.476386
H	-3.899996	1.919605	3.242591
H	-2.670767	2.472343	2.075520
H	-3.621484	3.642206	2.983807

TS 259

B3LYP/6-31G(d) = -3573.92168
B3LYP-D3(BJ)/def2-TZVPP/IEFPCM(propanonitrile) = -3575.494763
B3LYP-D3(BJ)/def2-TZVPP/IEFPCM(propanonitrile)//B3LYP-D3(BJ)/6-31G(d) Free Energy (Quasiharmonic) = -3574.778887

Frequencies (Top 3 out of 300)

1. -275.0384 cm^{-1}
2. 7.8152 cm^{-1}
3. 11.6848 cm^{-1}

B3LYP/6-31G(d) Molecular Geometry in Cartesian Coordinates

B	0.126251	-0.143121	-0.064460
O	-0.222959	-0.767461	-1.359510
N	0.567681	1.311369	-0.520700
C	0.605651	1.352309	-1.995990
S	0.022161	2.666549	0.260070
C	0.011631	0.010249	-2.424440
O	-0.225709	-0.315271	-3.565550
H	2.293241	2.618049	-2.449530
H	-0.063119	2.126659	-2.391900
C	2.018641	1.571709	-2.616250
C	3.098781	0.675429	-2.084430
C	3.447981	-0.661121	-2.512210
C	4.492670	-1.114091	-1.657700
N	4.781401	-0.087081	-0.779690
C	3.924951	0.966369	-1.027720
H	3.973711	1.863049	-0.426070
C	4.563980	-3.220091	-2.772790
C	2.981590	-1.521771	-3.523090
H	5.837270	-2.724591	-1.096440
H	5.327151	-0.177851	0.064550
C	3.542480	-2.787401	-3.642760
H	4.973330	-4.220591	-2.883020
H	2.176050	-1.211701	-4.182210

C	5.055560	-2.389061	-1.772610
H	3.181890	-3.461661	-4.415110
C	1.215771	3.954969	-0.129580
O	0.104801	2.420359	1.707080
O	-1.269029	3.130699	-0.307450
C	3.077691	5.974089	-0.687860
C	0.953221	4.873389	-1.145110
C	2.384561	4.046509	0.631350
C	3.304481	5.051979	0.346250
C	1.887731	5.872689	-1.419590
H	0.021871	4.814529	-1.698080
H	2.555931	3.352149	1.447410
H	4.210641	5.129269	0.942400
H	1.683301	6.590029	-2.210610
C	4.098901	7.039889	-1.005870
H	3.655171	7.865099	-1.571450
H	4.544601	7.452209	-0.094120
H	4.918921	6.630169	-1.610140
H	1.900001	1.444749	-3.699490
C	1.268771	-0.960471	0.729190
C	1.570460	-2.294401	0.419530
C	2.049831	-0.335001	1.711480
C	2.601840	-2.980751	1.066730
C	3.106010	-1.004641	2.333190
H	1.841131	0.695859	1.977890
C	3.383560	-2.336881	2.023840
H	4.205350	-2.855951	2.505520
C	4.049251	-0.251851	3.222740
C	2.886160	-4.422951	0.749960
F	4.575520	-1.023231	4.198340
F	3.477601	0.818619	3.810090
F	5.115101	0.230329	2.505780
F	4.218080	-4.668241	0.686790
F	2.347320	-4.812491	-0.423090
F	2.391800	-5.251541	1.704970
O	-1.103509	-0.016621	0.805170
C	-2.292659	-0.524160	0.925650
C	-2.632400	-1.968960	0.810550
C	-3.596160	-2.449360	1.714580
C	-2.049090	-2.865050	-0.100600
C	-3.951620	-3.795940	1.730220
H	-4.055130	-1.764150	2.423560
C	-2.410490	-4.211150	-0.082880
H	-1.330830	-2.504081	-0.825860
C	-3.355480	-4.682080	0.831510
H	-4.687440	-4.152360	2.445540
H	-1.947360	-4.895900	-0.787800
H	-3.626640	-5.734210	0.842530
H	-2.849789	-0.031050	1.725340
C	-3.327299	0.431840	-0.584660
H	-2.917549	-0.133270	-1.412300
H	-2.844299	1.387810	-0.405970
C	-4.686719	0.313810	-0.302740
C	-5.535659	-0.786600	-0.787860
C	-5.090680	-1.678490	-1.783700
C	-6.831709	-0.954410	-0.260540
C	-5.914010	-2.708350	-2.227750
H	-4.106890	-1.567690	-2.224030
C	-7.650360	-1.985390	-0.707910
H	-7.181909	-0.270040	0.502980
C	-7.193980	-2.866550	-1.691890
H	-5.556300	-3.385690	-2.997440
H	-8.645950	-2.103390	-0.289640
H	-7.835140	-3.670380	-2.043100
O	-5.298729	1.137830	0.537240
Si	-5.003159	2.738480	1.159670
C	-6.677799	3.135860	1.912170
H	-6.665219	4.128290	2.378950
H	-7.468699	3.134860	1.153660
H	-6.951349	2.409970	2.686490
C	-4.591309	3.878910	-0.270750
H	-3.559869	3.752910	-0.612210
H	-5.271739	3.726730	-1.116610
H	-4.703619	4.920000	0.058490
H	1.020270	-2.801761	-0.364410
C	-3.670019	2.669500	2.476470
H	-3.899939	1.919390	3.242660
H	-2.670759	2.472180	2.075560
H	-3.621459	3.642000	2.983910

TS 260

B3LYP/6-31G(d) = -3573.926146
B3LYP-D3(BJ)/def2-TZVPP/IEFPCM(propanonitrile) = -3575.495372
B3LYP-D3(BJ)/def2-TZVPP/IEFPCM(propanonitrile)//B3LYP-D3(BJ)/6-31G(d) Free Energy (Quasiharmonic) = -3574.77886

Frequencies (Top 3 out of 300)

1. -281.9684 cm^{-1}
2. 5.7318 cm^{-1}
3. 14.2733 cm^{-1}

B3LYP/6-31G(d) Molecular Geometry in Cartesian Coordinates

B	-0.426191	-0.544120	1.094690
O	-0.977501	-1.263910	2.254460
N	-1.190400	0.831560	1.201580
C	-1.888000	0.901830	2.505180
S	-0.687680	2.224480	0.482930
C	-1.613621	-0.475270	3.119330
O	-1.962871	-0.803660	4.230360
H	-3.590220	2.182250	2.097510
H	-1.428750	1.661560	3.149360
C	-3.422110	1.166150	2.468030
C	-4.255420	0.191381	1.679950
C	-4.863851	-1.039339	2.143320
C	-5.557641	-1.605699	1.038220
N	-5.402851	-0.746109	-0.031480
C	-4.609400	0.316081	0.358450
H	-4.383200	1.108411	-0.340640
C	-6.283711	-3.448699	2.363200
C	-4.908681	-1.708519	3.380520
H	-6.780681	-3.226629	0.268730
H	-5.608201	-0.976239	-0.991920
C	-5.615801	-2.899959	3.477110
H	-6.822492	-4.386599	2.470230
H	-4.370131	-1.315689	4.236290
C	-6.264081	-2.809649	1.129680
H	-5.647681	-3.426169	4.427390
C	-2.100220	3.068720	-0.242590
O	0.161440	1.817880	-0.658030
O	-0.121440	3.159840	1.476710
C	-4.244739	4.438001	-1.418910
C	-2.551690	2.686920	-1.508350
C	-2.686990	4.141080	0.430520
C	-3.755769	4.814030	-0.160310
C	-3.620010	3.371830	-2.083800
H	-2.069590	1.875210	-2.040810
H	-2.294909	4.453220	1.392580
H	-4.211689	5.650861	0.362970
H	-3.969550	3.073841	-3.069300
C	-5.422779	5.151391	-2.037580
H	-5.360809	5.155001	-3.130700
H	-5.488439	6.189171	-1.695330
H	-6.365559	4.658221	-1.766290
H	-3.737380	1.163150	3.518290
C	-0.622071	-1.363940	-0.280150
C	0.219389	-2.435100	-0.608310
C	-1.652121	-1.073670	-1.182400
C	0.075739	-3.147480	-1.802720

H	1.008519	-2.720511	0.081510
C	-1.814941	-1.789320	-2.371410
H	-2.344071	-0.275110	-0.942310
C	-0.942791	-2.828150	-2.699650
H	-1.053351	-3.373840	-3.628590
C	-2.955141	-1.432110	-3.280780
C	1.078248	-4.219061	-2.119800
F	-3.060901	-0.087490	-3.454540
F	-4.156641	-1.835959	-2.781930
F	-2.840301	-1.983470	-4.505540
F	0.766878	-4.918431	-3.229610
F	2.319629	-3.685411	-2.328770
F	1.219278	-5.100971	-1.106550
O	1.071999	-0.528981	1.432060
C	2.066670	0.314999	1.332170
C	3.198079	0.058299	2.269930
C	4.044430	1.113909	2.638520
C	3.398979	-1.211581	2.827170
C	5.083080	0.900168	3.543810
H	3.873360	2.107559	2.232680
C	4.440159	-1.422862	3.728860
H	2.718439	-2.016031	2.569330
C	5.285569	-0.370032	4.087630
H	5.724600	1.727468	3.835240
H	4.585029	-2.408702	4.162010
H	6.091309	-0.536062	4.797590
H	1.818480	1.370109	1.205760
C	2.715759	0.082169	-0.519590
H	1.959210	0.722949	-0.958770
H	2.510299	-0.975801	-0.613700
C	4.060330	0.461989	-0.634740
C	5.176149	-0.485602	-0.490700
C	6.461979	-0.008132	-0.168950
C	4.994259	-1.869202	-0.690200
C	7.528049	-0.889202	-0.029270
H	6.606210	1.054768	-0.016100
C	6.068729	-2.744082	-0.561320
H	4.030359	-2.266481	-0.988240
C	7.334619	-2.259532	-0.224560
H	8.511529	-0.509242	0.232020
H	5.917139	-3.805902	-0.730870
H	8.169749	-2.946912	-0.121270
O	4.430180	1.722078	-0.759870
Si	3.812030	3.206289	-1.432050
C	2.609190	2.832519	-2.821060
H	2.471880	3.741599	-3.420970
H	2.989060	2.053799	-3.492560
H	1.627290	2.529429	-2.446500
C	5.387810	3.998358	-2.079230
H	6.122400	4.140608	-1.278430
H	5.853950	3.385168	-2.858690
H	5.174891	4.983328	-2.512540
C	3.070810	4.217379	-0.040140
H	2.831281	5.221789	-0.413500
H	2.143840	3.791309	0.356980
H	3.781421	4.341419	0.785560

TS 261

B3LYP/6-31G(d) = -3573.917783
B3LYP-D3(BJ)/def2-TZVPP/IEFPCM(propanonitrile) = -3575.495138
B3LYP-D3(BJ)/def2-TZVPP/IEFPCM(propanonitrile)//B3LYP-D3(BJ)/6-31G(d) Free Energy (Quasiharmonic) = -3574.778822

Frequencies (Top 3 out of 300)

1. -294.8657 cm^{-1}
2. 7.2064 cm^{-1}
3. 10.0155 cm^{-1}

B3LYP/6-31G(d) Molecular Geometry in Cartesian Coordinates

B	-0.918681	-0.716552	0.719932
O	-0.984698	-0.944032	2.180530
N	-1.669469	-1.965205	0.186778
C	-2.002584	-2.882500	1.280554
S	-2.172605	-2.247971	-1.353805
C	-1.589608	-2.086633	2.523276
O	-1.759319	-2.451361	3.665036
H	-1.567390	-4.802564	0.391864
H	-3.086469	-3.059306	1.334212
C	-1.296483	-4.268771	1.304766
C	0.198442	-4.242114	1.477759
C	1.219897	-4.326875	0.459150
C	2.481471	-4.291869	1.120983
N	2.228118	-4.177423	2.471708
C	0.861376	-4.157065	2.678517
H	0.454275	-4.066385	3.675858
C	3.637673	-4.513561	-0.953579
C	1.195810	-4.448914	-0.942043
H	4.647410	-4.367831	0.955765
H	2.924205	-4.150386	3.199789
C	2.400311	-4.543695	-1.631171
H	4.562564	-4.602975	-1.517719
H	0.248703	-4.462556	-1.472868
C	3.695243	-4.382384	0.430223
H	2.387723	-4.655317	-2.712776
C	-3.915592	-1.796863	-1.430830
O	-2.129158	-3.697150	-1.613423
O	-1.441877	-1.320788	-2.229649
C	-6.631271	-1.092596	-1.498421
C	-4.883262	-2.720226	-1.025526
C	-4.291803	-0.534320	-1.893873
C	-5.643010	-0.194716	-1.925196
C	-6.229408	-2.360571	-1.057240
H	-4.585814	-3.718411	-0.722363
H	-3.539246	0.161504	-2.246696
H	-5.935424	0.785138	-2.295530
H	-6.980791	-3.082372	-0.745660
C	-8.089432	-0.699115	-1.503180
H	-8.369249	-0.212280	-0.559340
H	-8.740297	-1.571255	-1.624579
H	-8.311823	0.006759	-2.310381
H	-1.747968	-4.805550	2.147609
C	-1.525742	0.753630	0.375972
C	-1.370656	1.390269	-0.864777
C	-2.250839	1.454600	1.356504
C	-1.903748	2.660333	-1.110932
H	-0.860430	0.868822	-1.666563
C	-2.785481	2.723450	1.115277
H	-2.398319	0.996955	2.329620
C	-2.612747	3.340861	-0.123474
H	-3.028830	4.322220	-0.316079
C	-3.492826	3.455602	2.223049
C	-1.680510	3.275969	-2.461230
F	-2.614224	4.091562	3.039266
F	-4.212740	2.622264	3.003353
F	-4.337209	4.401035	1.750316
F	-2.119689	2.476749	-3.459271
F	-0.351260	3.481348	-2.698065
F	-2.289699	4.471525	-2.601866
O	0.506328	-0.880865	0.215229
C	1.723031	-0.461455	0.448534
C	2.169824	0.166754	1.731953
C	1.362263	0.995042	2.525413
C	3.479719	-0.111031	2.155420
C	1.854886	1.521944	3.718603
H	0.346006	1.213820	2.224822
C	3.970143	0.413416	3.350403
H	4.110194	-0.759016	1.550824
C	3.156622	1.232128	4.136750

H	1.213172	2.149596	4.330468
H	4.981543	0.177357	3.670087
H	3.530886	1.635759	5.073744
H	2.459157	-1.179467	0.074661
C	1.999905	0.855532	-0.949608
H	1.343236	1.644281	-0.603156
H	1.574987	0.249698	-1.741440
C	3.364454	1.179458	-1.068325
C	4.331456	0.329242	-1.774111
C	3.944567	-0.889454	-2.370282
C	5.678673	0.735632	-1.872584
C	4.877613	-1.665542	-3.050709
H	2.923208	-1.247076	-2.301419
C	6.603730	-0.042654	-2.557444
H	5.984220	1.666366	-1.409702
C	6.205287	-1.244375	-3.151033
H	4.563511	-2.602504	-3.498880
H	7.636576	0.285489	-2.630444
H	6.928752	-1.851396	-3.688189
O	3.888758	2.207324	-0.433346
Si	3.490426	3.853840	-0.039208
C	4.120812	4.838362	-1.510427
H	5.189636	4.670841	-1.683682
H	3.583161	4.575112	-2.428661
H	3.974646	5.912386	-1.341158
C	4.490902	4.150160	1.515209
H	4.161345	3.490781	2.325414
H	5.558257	3.971052	1.344769
H	4.375248	5.186637	1.854780
C	1.657697	4.140100	0.226978
H	1.062710	3.988417	-0.679026
H	1.249173	3.510758	1.024020
H	1.518398	5.185356	0.534321

TS 262

B3LYP/6-31G(d) = -3573.923269
B3LYP-D3(BJ)/def2-TZVPP/IEFPCM(propanonitrile) = -3575.495827
B3LYP-D3(BJ)/def2-TZVPP/IEFPCM(propanonitrile)//B3LYP-D3(BJ)/6-31G(d) Free Energy (Quasiharmonic) = -3574.778727

Frequencies (Top 3 out of 300)

1. -270.2019 cm^{-1}
2. 10.7927 cm^{-1}
3. 14.4491 cm^{-1}

B3LYP/6-31G(d) Molecular Geometry in Cartesian Coordinates

B	0.894454	0.473411	-0.853856
O	1.556230	0.202237	-2.143906
N	1.794896	1.597967	-0.277544
C	2.966369	1.819016	-1.137913
S	1.426689	2.669863	0.904950
C	2.678510	0.911392	-2.338376
O	3.365501	0.837221	-3.329713
H	4.559944	2.330693	0.210746
H	2.984817	2.850570	-1.518204
C	4.357515	1.530869	-0.506935
C	4.497517	0.199697	0.171553
C	4.892178	-1.065462	-0.406136
C	4.859969	-2.029805	0.640863
N	4.485366	-1.371456	1.795382
C	4.249880	-0.043020	1.500441
H	3.924323	0.648807	2.264907
C	5.556774	-3.753007	-0.853295
C	5.275091	-1.480407	-1.694883
H	5.153931	-4.094566	1.245292
H	4.203026	-1.819299	2.654008
C	5.601988	-2.814534	-1.904626
H	5.815484	-4.790227	-1.050019
H	5.289419	-0.772634	-2.518435
C	5.186442	-3.373729	0.431940
H	5.893115	-3.144210	-2.898535
C	1.164156	4.256353	0.083246
O	2.573857	2.878339	1.804302
O	0.145488	2.232213	1.499504
C	0.710204	6.755234	-1.107154
C	0.341599	4.347571	-1.043098
C	1.761977	5.397086	0.619768
C	1.527463	6.636256	0.023978
C	0.122685	5.592257	-1.628135
H	-0.108389	3.451850	-1.458453
H	2.403961	5.305013	1.488846
H	1.992879	7.524796	0.443862
H	-0.512256	5.662801	-2.508379
C	0.491386	8.094047	-1.770787
H	0.644365	8.919652	-1.068481
H	-0.520759	8.178259	-2.180935
H	1.191604	8.239247	-2.604053
H	5.084729	1.633108	-1.321665
C	0.769996	-0.890986	0.016723
C	0.912882	-0.920827	1.413823
C	0.540497	-2.120730	-0.620604
C	0.816849	-2.113758	2.135124
H	1.084485	0.001941	1.954000
C	0.403881	-3.312176	0.100567
H	0.468333	-2.162248	-1.701374
C	0.546086	-3.319214	1.486275
H	0.450374	-4.241635	2.047163
C	0.012631	-4.571315	-0.619462
C	1.055246	-2.115474	3.618978
F	-1.342895	-4.629933	-0.800969
F	0.352013	-5.685031	0.062279
F	0.561308	-4.655869	-1.846665
F	0.360633	-3.090955	4.245647
F	0.723368	-0.943777	4.195539
F	2.370161	-2.336015	3.922832
O	-0.479628	1.120562	-1.134060
C	-1.721704	0.809547	-1.355309
C	-2.160392	-0.060655	-2.471840
C	-3.535865	-0.139789	-2.759076
C	-1.246817	-0.698475	-3.328514
C	-3.991933	-0.865256	-3.854466
H	-4.249242	0.375995	-2.122227
C	-1.710004	-1.426188	-4.423933
H	-0.181214	-0.579566	-3.171249
C	-3.078132	-1.519710	-4.685484
H	-5.056832	-0.919073	-4.061644
H	-0.994541	-1.908484	-5.083691
H	-3.431809	-2.087358	-5.541822
H	-2.399231	1.644441	-1.160295
C	-2.282077	-0.094748	0.458419
H	-1.722497	0.653947	1.008522
H	-1.755751	-1.023721	0.299468
C	-3.661367	-0.148543	0.628912
C	-4.489125	-1.299373	0.231924
C	-5.893749	-1.214612	0.313100
C	-3.909922	-2.502939	-0.218808
C	-6.689859	-2.295891	-0.049109
H	-6.347079	-0.294363	0.662404
C	-4.710990	-3.582356	-0.576708
H	-2.835357	-2.617416	-0.286842
C	-6.101680	-3.483388	-0.494675
H	-7.771100	-2.215263	0.018598
H	-4.240587	-4.500832	-0.914222
H	-6.725257	-4.328905	-0.771956
O	-4.350436	0.883156	1.105401
Si	-3.947895	2.269268	2.080254
C	-3.155807	3.574737	0.990483
H	-2.122362	3.316396	0.737327

H	-3.725781	3.739892	0.068610
H	-3.123899	4.530155	1.529794
C	-2.838283	1.738270	3.497449
H	-1.791341	1.636834	3.194716
H	-2.876205	2.502661	4.284250
H	-3.169614	0.793779	3.944127
C	-5.649650	2.797876	2.671297
H	-5.580481	3.700487	3.290418
H	-6.313407	3.023608	1.828849
H	-6.124267	2.016013	3.274821

TS 263

B3LYP/6-31G(d) = -3573.923269
B3LYP-D3(BJ)/def2-TZVPP/IEFPCM(propanonitrile) = -3575.495825
B3LYP-D3(BJ)/def2-TZVPP/IEFPCM(propanonitrile)//B3LYP-D3(BJ)/6-31G(d) Free Energy (Quasiharmonic) = -3574.778725

Frequencies (Top 3 out of 300)

1. -270.1839 cm^{-1}
2. 10.8039 cm^{-1}
3. 14.4457 cm^{-1}

B3LYP/6-31G(d) Molecular Geometry in Cartesian Coordinates

B	0.894359	0.473574	-0.853842
O	1.556192	0.202545	-2.143893
N	1.794548	1.598332	-0.277531
C	2.965941	1.819685	-1.137930
S	1.426082	2.670127	0.904979
C	2.678285	0.911990	-2.338389
O	3.365269	0.838001	-3.329744
H	4.559414	2.331758	0.210701
H	2.984115	2.851244	-1.518220
C	4.357175	1.531890	-0.506987
C	4.497522	0.200745	0.171480
C	4.892483	-1.064309	-0.406232
C	4.860522	-2.028674	0.640754
N	4.485774	-1.370433	1.795288
C	4.249960	-0.042050	1.500367
H	3.924241	0.649688	2.264844
C	5.557720	-3.751688	-0.853437
C	5.275482	-1.479144	-1.694990
H	5.154984	-4.093373	1.245151
H	4.203550	-1.818360	2.653909
C	5.602697	-2.813190	-1.904755
H	5.816677	-4.788843	-1.050179
H	5.289632	-0.771356	-2.518532
C	5.187314	-3.372517	0.431808
H	5.893892	-3.142781	-2.898673
C	1.163158	4.256561	0.083291
O	2.573203	2.878874	1.804330
O	0.144993	2.232154	1.499533
C	0.708590	6.755341	-1.107088
C	0.340606	4.347583	-1.043077
C	1.760670	5.397440	0.619842
C	1.525849	6.636559	0.024061
C	0.121387	5.592216	-1.628103
H	-0.109128	3.451747	-1.458461
H	2.402657	5.305523	1.488934
H	1.991026	7.525213	0.443966
H	-0.513545	5.662606	-2.508368
C	0.489410	8.094102	-1.770705
H	0.642896	8.919774	-1.068592
H	-0.523007	8.178323	-2.180188
H	1.189071	8.239167	-2.604461
H	5.084345	1.634327	-1.321731
C	0.770195	-0.890857	0.016728
C	0.913206	-0.920703	1.413816
C	0.540883	-2.120632	-0.620607
C	0.817482	-2.113674	2.135095
H	1.084680	0.002084	1.954000
C	0.404558	-3.312122	0.100547
H	0.468639	-2.162143	-1.701371
C	0.546891	-3.319166	1.486240
H	0.451416	-4.241620	2.047115
C	0.013503	-4.571323	-0.619479
C	1.056052	-2.115410	3.618921
F	0.353185	-5.684985	0.062198
F	0.562081	-4.655727	-1.846737
F	-1.342028	-4.630223	-0.800859
F	0.724200	-0.943745	4.195557
F	2.371016	-2.335910	3.922608
F	0.361560	-3.090939	4.245647
O	-0.479865	1.120435	-1.134069
C	-1.721881	0.809205	-1.355317
C	-2.160419	-0.061075	-2.471841
C	-3.535880	-0.140464	-2.759065
C	-1.246731	-0.698711	-3.328533
C	-3.991821	-0.866010	-3.854455
H	-4.249347	0.375182	-2.122207
C	-1.709791	-1.426503	-4.423953
H	-0.181148	-0.579597	-3.171279
C	-3.077905	-1.520284	-4.685488
H	-5.056712	-0.920029	-4.061623
H	-0.994243	-1.908657	-5.083723
H	-3.431482	-2.087994	-5.541826
H	-2.399568	1.643967	-1.160280
C	-2.282079	-0.095174	0.458471
H	-1.722665	0.653679	1.008529
H	-1.755546	-1.024038	0.299569
C	-3.661359	-0.149255	0.628911
C	-4.488855	-1.300286	0.231957
C	-3.909376	-2.503726	-0.218756
C	-5.893497	-1.215856	0.313156
C	-4.710192	-3.583346	-0.576602
H	-2.834786	-2.617942	-0.286819
C	-6.689357	-2.297338	-0.049000
H	-6.347040	-0.295706	0.662443
C	-6.100905	-3.484709	-0.494539
H	-4.239577	-4.501720	-0.914099
H	-7.770615	-2.216966	0.018728
H	-6.724285	-4.330385	-0.771778
O	-4.350671	0.882331	1.105307
Si	-3.948494	2.268437	2.080315
C	-3.156685	3.574212	0.990714
H	-3.726803	3.739523	0.068959
H	-3.124778	4.529510	1.530238
H	-2.123249	3.316032	0.737349
C	-2.838776	1.737554	3.497474
H	-1.791760	1.636709	3.194800
H	-3.169687	0.792766	3.943835
H	-2.877143	2.501685	4.284503
C	-5.650392	2.796581	2.671359
H	-6.314218	3.022098	1.828907
H	-6.124784	2.014602	3.274909
H	-5.581476	3.699232	3.290451

TS 264

B3LYP/6-31G(d) = -3573.917073
B3LYP-D3(BJ)/def2-TZVPP/IEFPCM(propanonitrile) = -3575.495031
B3LYP-D3(BJ)/def2-TZVPP/IEFPCM(propanonitrile)//B3LYP-D3(BJ)/6-31G(d) Free Energy (Quasiharmonic) = -3574.778685

Frequencies (Top 3 out of 300)

1. -258.7245 cm^{-1}
2. 7.0297 cm^{-1}

3. 11.5933 cm^{-1}

B3LYP/6-31G(d) Molecular Geometry in Cartesian Coordinates

B	-0.325453	0.162604	0.192625
O	-0.301823	0.266471	1.672419
N	-0.195243	1.680566	-0.192562
C	-0.414795	2.525626	0.997093
S	-0.086623	2.357941	-1.681425
C	-0.273991	1.512841	2.132441
O	-0.131800	1.809450	3.301776
H	-1.720256	4.092520	0.303564
H	0.402578	3.249807	1.105022
C	-1.741311	3.336101	1.094081
C	-3.024829	2.561352	1.015476
C	-3.747254	1.922633	2.093565
C	-4.909678	1.331148	1.522406
N	-4.896892	1.621361	0.172664
C	-3.759029	2.341039	-0.124541
H	-3.545410	2.648344	-1.137908
C	-5.606411	0.514924	3.652067
C	-3.539585	1.799418	3.479515
H	-6.722058	0.176009	1.829229
H	-5.489669	1.198486	-0.525613
C	-4.467254	1.099696	4.241648
H	-6.311084	-0.030785	4.274141
H	-2.653223	2.222279	3.943185
C	-5.843112	0.623668	2.286468
H	-4.309924	0.994377	5.311941
C	1.142625	3.666755	-1.505431
O	-1.319990	3.036877	-2.117742
O	0.460968	1.316841	-2.576879
C	3.088551	5.688055	-1.416979
C	2.340049	3.447661	-0.820268
C	0.903977	4.887303	-2.138572
C	1.875990	5.885718	-2.090129
C	3.299534	4.456918	-0.778916
H	2.507886	2.509655	-0.301061
H	-0.037727	5.046301	-2.652043
H	1.685169	6.837139	-2.580641
H	4.227421	4.289323	-0.236652
C	4.147249	6.764523	-1.393037
H	3.703557	7.764003	-1.444504
H	4.827689	6.665954	-2.249348
H	4.756533	6.708885	-0.484969
O	0.901177	-0.720184	-0.069401
C	1.392226	-1.152894	-1.199870
H	1.060827	-0.643487	-2.106264
H	-1.678552	3.874520	2.048516
C	1.620521	-2.616396	-1.335053
C	1.945325	-3.154402	-2.589902
C	1.436400	-3.478920	-0.245452
C	2.094644	-4.529346	-2.750370
H	2.063893	-2.492201	-3.444940
C	1.581577	-4.855742	-0.409282
H	1.149033	-3.060566	0.713288
C	1.914930	-5.383389	-1.658396
H	2.337417	-4.937019	-3.727746
H	1.420130	-5.519086	0.435943
H	2.020860	-6.457316	-1.785298
C	-1.619814	-0.669356	-0.313027
C	-2.370942	-0.381333	-1.459595
C	-2.021527	-1.778566	0.450864
C	-3.481875	-1.155479	-1.815550
H	-2.112177	0.464018	-2.087284
C	-3.113633	-2.568199	0.087369
H	-1.483181	-2.015896	1.362951
C	-3.858411	-2.257250	-1.050152
H	-4.717401	-2.856549	-1.328270
C	-3.456552	-3.792115	0.890302
C	-4.331967	-0.733751	-2.978570
F	-4.769283	-4.108014	0.802462
F	-2.767690	-4.880922	0.460192
F	-3.607417	-0.206586	-3.984809
F	-3.161787	-3.643307	2.199655
F	-5.235857	0.230401	-2.615566
F	-5.057463	-1.753461	-3.487263
C	3.202294	-0.283236	-1.398767
H	3.581207	-0.761394	-2.294818
H	2.794159	0.711262	-1.530552
C	3.824330	-0.597431	-0.187654
O	3.461326	0.084510	0.873284
Si	3.858146	0.383181	2.546907
C	2.744596	-0.683438	3.610555
H	2.994336	-1.749402	3.592495
H	2.821849	-0.346300	4.652234
H	1.699338	-0.559755	3.306208
C	5.698803	0.085125	2.809824
H	6.302528	0.654353	2.093085
H	5.970196	0.435155	3.814071
H	5.987261	-0.966690	2.733444
C	3.453338	2.196946	2.782319
H	2.372151	2.360917	2.834425
H	3.878183	2.543296	3.733197
H	3.870313	2.825366	1.987849
C	4.735873	-1.755815	-0.073329
C	4.636148	-2.663416	0.994921
C	5.706041	-1.977517	-1.066830
C	5.487445	-3.763008	1.067389
H	3.864871	-2.529462	1.744266
C	6.569023	-3.067022	-0.980474
H	5.802234	-1.275188	-1.889062
C	6.460095	-3.963118	0.085079
H	5.387431	-4.467888	1.887612
H	7.326135	-3.216567	-1.744650
H	7.128534	-4.817113	0.148114

TS 265

B3LYP/6-31G(d) = -3573.922489
B3LYP-D3(BJ)/def2-TZVPP/IEFPCM(propanonitrile) = -3575.495295
B3LYP-D3(BJ)/def2-TZVPP/IEFPCM(propanonitrile)//B3LYP-D3(BJ)/6-31G(d) Free Energy (Quasiharmonic) = -3574.778668

Frequencies (Top 3 out of 300)

1. -257.4336 cm^{-1}
2. 8.2448 cm^{-1}
3. 10.1775 cm^{-1}

B3LYP/6-31G(d) Molecular Geometry in Cartesian Coordinates

B	0.465375	-0.339009	-1.332654
O	0.157236	-0.414382	-2.778120
N	-0.423459	-1.511080	-0.784940
C	-1.057219	-2.244383	-1.899755
S	-0.295612	-2.267894	0.670720
C	-0.582680	-1.472710	-3.133905
O	-0.858693	-1.759974	-4.274604
H	-2.869592	-3.059371	-1.061536
H	-0.645624	-3.259148	-1.967704
C	-2.599105	-2.385540	-1.882320
C	-3.418284	-1.125894	-1.773920
C	-4.739318	-1.031912	-1.194961
C	-5.166213	0.321489	-1.321724
N	-4.158491	1.003549	-1.967849
C	-3.119280	0.133445	-2.231412
H	-2.242676	0.494801	-2.742930
C	-7.241771	-0.176361	-0.255174
C	-5.609872	-1.957294	-0.593645
H	-6.712854	1.800732	-0.956618

H	-4.108269	2.000388	-2.117228
C	-6.847953	-1.524982	-0.131043
H	-8.216668	0.132827	0.112556
H	-5.321737	-3.000295	-0.492446
C	-6.407363	0.762735	-0.851357
H	-7.530716	-2.238518	0.323546
C	-1.868293	-2.139696	1.526384
O	0.689185	-1.489252	1.456453
O	-0.036298	-3.705984	0.472488
C	-4.269042	-1.972116	2.955783
C	-2.535721	-3.309033	1.889733
C	-2.372336	-0.885616	1.881716
C	-3.565269	-0.813445	2.592139
C	-3.731356	-3.215374	2.602716
H	-2.115845	-4.271874	1.620021
H	-1.840957	0.020916	1.615060
H	-3.963196	0.161094	2.863025
H	-4.254614	-4.125449	2.885957
C	-5.590392	-1.869483	3.676577
H	-5.863406	-2.815068	4.155720
H	-6.387608	-1.609509	2.968326
H	-5.569717	-1.089406	4.445657
H	-2.843039	-2.924898	-2.808571
C	0.143470	1.142138	-0.762191
C	0.230336	1.493796	0.593470
C	-0.343030	2.126335	-1.636930
C	-0.195073	2.744614	1.056409
H	0.610626	0.768976	1.304661
C	-0.779947	3.372518	-1.179065
H	-0.394501	1.907258	-2.698562
C	-0.714963	3.692271	0.176523
H	-1.065602	4.651433	0.537283
C	-1.423394	4.322885	-2.146771
C	-0.118452	3.034842	2.528382
F	-2.724456	3.975630	-2.394079
F	-1.451774	5.592195	-1.688543
F	-0.804615	4.334141	-3.344969
F	1.167488	3.095276	2.979345
F	-0.722045	2.066029	3.259432
F	-0.697248	4.207463	2.861222
O	1.985533	-0.593941	-1.288143
C	2.664422	-1.646675	-0.948509
C	3.467038	-2.319930	-1.994645
C	4.042896	-3.575309	-1.736790
C	3.605794	-1.741721	-3.266546
C	4.759642	-4.234800	-2.730458
H	3.915260	-4.035696	-0.759792
C	4.326470	-2.405610	-4.257592
H	3.113743	-0.796678	-3.470395
C	4.908282	-3.647493	-3.991311
H	5.195206	-5.209235	-2.528207
H	4.421272	-1.960798	-5.244120
H	5.464929	-4.164738	-4.767947
H	2.253172	-2.286480	-0.169376
C	4.283405	-1.087182	0.332675
H	4.310767	-1.981727	0.942439
H	5.044420	-1.030972	-0.432256
C	3.893926	0.108693	0.939147
C	4.023765	1.411646	0.269786
C	4.464149	1.514906	-1.065099
C	3.702439	2.590782	0.969295
C	4.591506	2.759867	-1.671492
H	4.692321	0.625557	-1.640760
C	3.835124	3.833275	0.359324
H	3.331805	2.520299	1.983013
C	4.282466	3.922548	-0.961003
H	4.925704	2.823547	-2.702843
H	3.580508	4.732684	0.912290
H	4.383224	4.893635	-1.437866
O	3.295181	0.149352	2.112312
Si	3.287157	-0.760770	3.591195
C	1.737825	-0.160381	4.446375
H	1.681316	0.932649	4.456862
H	0.852044	-0.536823	3.925476
H	1.705061	-0.514964	5.483902
C	4.862497	-0.215385	4.467892
H	4.872894	0.867327	4.635781
H	4.945963	-0.705511	5.445883
H	5.758396	-0.475664	3.891793
C	3.272537	-2.621372	3.329275
H	3.079988	-3.104402	4.296488
H	4.224589	-3.016335	2.957655
H	2.466466	-2.913412	2.649665

TS 266

B3LYP/6-31G(d) = -3573.916469
B3LYP-D3(BJ)/def2-TZVPP/IEFPCM(propanonitrile) = -3575.496011
B3LYP-D3(BJ)/def2-TZVPP/IEFPCM(propanonitrile)//B3LYP-D3(BJ)/6-31G(d) Free Energy (Quasiharmonic) = -3574.778647

Frequencies (Top 3 out of 300)

1. -282.5486 cm^{-1}
2. 10.3842 cm^{-1}
3. 13.4509 cm^{-1}

B3LYP/6-31G(d) Molecular Geometry in Cartesian Coordinates

B	0.059440	-0.653790	0.497260
O	0.329469	-1.086590	1.882060
N	-0.109432	-2.021500	-0.218410
C	0.012157	-3.135360	0.734700
S	-0.631872	-2.266059	-1.762250
C	0.250868	-2.412440	2.062520
O	0.369407	-2.952270	3.139650
H	0.950575	-4.673591	-0.453910
H	-0.934544	-3.688089	0.828710
C	1.145256	-4.173391	0.496320
C	2.548246	-3.630793	0.529850
C	3.393507	-3.277703	-0.589030
C	4.634377	-2.833715	-0.054150
N	4.546977	-2.933055	1.319550
C	3.292427	-3.398183	1.663330
H	3.019696	-3.539313	2.700080
C	5.498238	-2.433286	-2.238340
C	3.230807	-3.300873	-1.986820
H	6.624478	-2.050477	-0.430020
H	5.234927	-2.586865	1.969410
C	4.281567	-2.881844	-2.792950
H	6.297868	-2.104047	-2.896910
H	2.291326	-3.623142	-2.424340
C	5.691348	-2.402006	-0.862630
H	4.162057	-2.890324	-3.873110
C	-2.435592	-2.405547	-1.747060
O	-0.165284	-3.585900	-2.213080
O	-0.329391	-1.052479	-2.527370
C	-5.232703	-2.649834	-1.865480
C	-3.045364	-3.611106	-1.387590
C	-3.209571	-1.326066	-2.175400
C	-4.598601	-1.454745	-2.231720
C	-4.433324	-3.720465	-1.437280
H	-2.437225	-4.464547	-1.107160
H	-2.718510	-0.411247	-2.489640
H	-5.196890	-0.617344	-2.584420
H	-4.903195	-4.661114	-1.159320
C	-6.732153	-2.801592	-1.972990
H	-7.237122	-1.830122	-1.964930
H	-7.136063	-3.402782	-1.150950
H	-7.010553	-3.306122	-2.907540
H	1.018435	-4.917311	1.292290

C	1.213471	0.343309	-0.038230
C	0.996092	1.269269	-1.070300
C	2.488241	0.324148	0.544770
C	2.005393	2.131628	-1.502250
H	0.027012	1.306670	-1.551580
C	3.503692	1.186686	0.114970
H	2.694970	-0.375183	1.348100
C	3.270953	2.094797	-0.915130
H	4.056333	2.762336	-1.251010
C	4.812312	1.237805	0.848520
C	1.708024	3.187848	-2.524530
F	5.831252	1.647554	0.063220
F	5.166250	0.045135	1.380390
F	4.752753	2.114285	1.891880
F	2.781784	3.507667	-3.274660
F	1.306625	4.357649	-1.923480
F	0.706683	2.846539	-3.363110
O	-1.241910	0.179082	0.430230
C	-2.466140	0.164363	0.869260
C	-3.015421	-0.747716	1.917110
C	-2.372731	-1.041837	3.130800
C	-4.284421	-1.298125	1.667420
C	-2.974702	-1.893676	4.053620
H	-1.398310	-0.621768	3.344410
C	-4.883872	-2.149334	2.593820
H	-4.788371	-1.079945	0.729770
C	-4.229312	-2.449405	3.789670
H	-2.454972	-2.128967	4.977350
H	-5.857763	-2.580553	2.379310
H	-4.693103	-3.114495	4.513020
H	-3.195339	0.376724	0.082180
C	-2.856408	1.960393	1.785880
H	-2.405898	1.805563	2.758820
H	-3.936458	1.881985	1.768870
C	-2.227967	2.855553	0.913800
C	-2.818806	3.285553	-0.364240
C	-4.196536	3.150595	-0.628550
C	-1.984196	3.819822	-1.364250
C	-4.717586	3.532935	-1.860760
H	-4.869177	2.770216	0.133270
C	-2.507765	4.183353	-2.600830
H	-0.925375	3.929221	-1.172080
C	-3.874365	4.043214	-2.852480
H	-5.783166	3.435887	-2.047920
H	-1.840945	4.564702	-3.367900
H	-4.283435	4.331575	-3.816780
O	-0.978466	3.222711	1.097080
Si	-0.010306	3.616640	2.496670
C	0.234053	2.138270	3.626830
H	-0.651168	1.904841	4.229050
H	0.525282	1.237690	3.075950
H	1.047583	2.370069	4.327080
C	-0.968104	4.993441	3.352110
H	-1.967995	4.668652	3.662360
H	-0.434884	5.322471	4.252560
H	-1.083493	5.864501	2.697200
C	1.601745	4.222059	1.760720
H	2.006216	5.042248	2.366730
H	2.358004	3.431778	1.725990
H	1.461915	4.593149	0.740380

TS 267

B3LYP/6-31G(d) = -3573.917085
B3LYP-D3(BJ)/def2-TZVPP/IEFPCM(propanonitrile) = -3575.495036
B3LYP-D3(BJ)/def2-TZVPP/IEFPCM(propanonitrile)//B3LYP-D3(BJ)/6-31G(d) Free Energy (Quasiharmonic) = -3574.778625

Frequencies (Top 3 out of 300)

1. -258.6893 cm^{-1}
2. 7.0153 cm^{-1}
3. 11.5933 cm^{-1}

B3LYP/6-31G(d) Molecular Geometry in Cartesian Coordinates

B	-0.325123	0.162553	0.192119
O	-0.300980	0.268307	1.671790
N	-0.195500	1.680047	-0.195040
C	-0.414914	2.526557	0.993602
S	-0.087503	2.355389	-1.684875
C	-0.273543	1.515272	2.130214
O	-0.131215	1.813468	3.299130
H	-1.720818	4.092229	0.298243
H	0.402355	3.251011	1.100414
C	-1.741537	3.336924	1.089839
C	-3.024941	2.561848	1.012651
C	-3.746976	1.924517	2.091821
C	-4.909459	1.332061	1.521793
N	-4.897070	1.620388	0.171643
C	-3.759402	2.339831	-0.126867
H	-3.546087	2.645758	-1.140715
C	-5.605515	0.518711	3.652775
C	-3.538920	1.803259	3.477885
H	-6.721587	0.177085	1.830703
H	-5.489942	1.196417	-0.525889
C	-4.466287	1.104466	4.241239
H	-6.309943	-0.026234	4.275794
H	-2.652486	2.226876	3.940727
C	-5.842589	0.625512	2.287087
H	-4.308653	1.000652	5.311634
C	1.141116	3.664916	-1.510014
O	-1.321054	3.033692	-2.121687
O	0.459692	1.313018	-2.579085
C	3.089096	5.684443	-1.428829
C	0.905684	4.881656	-2.151546
C	2.338664	3.446803	-0.824608
C	3.300197	4.454220	-0.788945
C	1.879929	5.878220	-2.109030
H	-0.032344	5.036781	-2.672885
H	2.508064	2.508148	-0.307094
H	4.231394	4.284733	-0.252922
H	1.694872	6.823934	-2.612609
C	4.124235	6.781566	-1.358120
H	3.963655	7.417683	-0.477570
H	4.081550	7.429777	-2.239282
H	5.137755	6.373676	-1.283815
H	-1.678635	3.876666	2.043521
C	-1.619459	-0.670406	-0.311971
C	-2.020567	-1.778762	0.453479
C	-2.371129	-0.384038	-1.458601
C	-3.112607	-2.569147	0.091422
H	-1.481791	-2.014801	1.365648
C	-3.481982	-1.158948	-1.813145
H	-2.112854	0.460593	-2.087459
C	-3.857916	-2.259848	-1.046201
H	-4.716849	-2.859736	-1.323222
C	-4.332606	-0.738949	-2.976405
C	-3.454880	-3.792132	0.896048
F	-5.236540	0.225529	-2.614360
F	-5.058107	-1.759479	-3.483437
F	-3.608543	-0.213007	-3.983630
F	-3.159739	-3.641532	2.205113
F	-2.765816	-4.881277	0.467128
F	-4.767541	-4.108553	0.809045
O	0.901692	-0.720177	-0.069269
C	1.391905	-1.154742	-1.199386
C	1.620466	-2.618399	-1.332250
C	1.944424	-3.158458	-2.586439
C	1.437409	-3.479113	-0.241037
C	2.093955	-4.533636	-2.744681

H	2.062176	-2.497683	-3.442691
C	1.582798	-4.856175	-0.402638
H	1.150689	-3.059206	0.717219
C	1.915304	-5.385863	-1.651114
H	2.336071	-4.942911	-3.721549
H	1.422176	-5.518121	0.443840
H	2.021401	-6.459977	-1.776272
H	1.059746	-0.646920	-2.106389
C	3.201782	-0.284953	-1.401294
H	2.793189	0.709168	-1.534486
H	3.580065	-0.764558	-2.296836
C	3.824781	-0.597017	-0.190153
C	4.736842	-1.754893	-0.074701
C	5.706439	-1.977637	-1.068529
C	4.638205	-2.660984	0.994928
C	6.569924	-3.066666	-0.981173
H	5.801788	-1.276465	-1.891846
C	5.489999	-3.760125	1.068414
H	3.867375	-2.526277	1.744593
C	6.462073	-3.961264	0.085746
H	7.326582	-3.217006	-1.745642
H	5.390815	-4.463860	1.889722
H	7.130900	-4.814898	0.149568
O	3.462380	0.086552	0.869974
Si	3.859876	0.387469	2.543012
C	3.454019	2.201249	2.776599
H	2.372757	2.364403	2.829808
H	3.879740	2.549047	3.726559
H	3.869499	2.829089	1.980898
C	5.700842	0.090804	2.805325
H	6.303853	0.659629	2.087666
H	5.972549	0.442067	3.809056
H	5.989888	-0.960920	2.729914
C	2.747487	-0.678441	3.608615
H	1.702042	-0.556007	3.304396
H	2.998019	-1.744242	3.592120
H	2.824790	-0.339652	4.649754

TS 268

B3LYP/6-31G(d) = -3573.920475
B3LYP-D3(BJ)/def2-TZVPP/IEFPCM(propanonitrile) = -3575.495797
B3LYP-D3(BJ)/def2-TZVPP/IEFPCM(propanonitrile)//B3LYP-D3(BJ)/6-31G(d) Free Energy (Quasiharmonic) = -3574.778555

Frequencies (Top 3 out of 300)

1. -278.0533 cm^{-1}
2. 9.9373 cm^{-1}
3. 13.1816 cm^{-1}

B3LYP/6-31G(d) Molecular Geometry in Cartesian Coordinates

B	0.540970	-0.138760	0.038480
O	1.210600	0.134781	-1.260890
N	-0.223920	-1.469290	-0.289660
C	0.042921	-1.874370	-1.680080
S	-0.572929	-2.648570	0.821320
C	1.047290	-0.831849	-2.163570
O	1.606240	-0.854569	-3.242540
H	-1.872989	-2.683901	-2.271950
H	0.533971	-2.853210	-1.709670
C	-1.172369	-1.932990	-2.650170
C	-1.900170	-0.635891	-2.892320
C	-3.269180	-0.328761	-2.548390
C	-3.520101	1.008879	-2.961320
N	-2.368641	1.472669	-3.561030
C	-1.399860	0.488799	-3.507890
H	-0.421330	0.660930	-3.933700
C	-5.763441	0.897618	-2.164660
C	-4.316380	-1.054902	-1.954590
H	-4.912711	2.669738	-3.069450
H	-2.200451	2.436379	-3.804570
C	-5.547670	-0.439122	-1.770570
H	-6.735401	1.354817	-1.998760
H	-4.168029	-2.083382	-1.640080
C	-4.753671	1.638748	-2.765750
H	-6.360810	-0.995053	-1.310990
C	-2.333969	-3.019901	0.737600
O	-0.345749	-2.087140	2.161790
O	0.114522	-3.898450	0.432320
C	-5.053939	-3.698742	0.764740
C	-3.248369	-2.183141	1.383020
C	-2.760258	-4.190751	0.110350
C	-4.116858	-4.518242	0.122540
C	-4.596679	-2.528212	1.389230
H	-2.917440	-1.281761	1.886130
H	-2.032758	-4.844781	-0.357980
H	-4.448468	-5.432372	-0.364190
H	-5.306139	-1.874882	1.890880
C	-6.515638	-4.076243	0.812040
H	-6.775878	-4.523673	1.780450
H	-6.767568	-4.804963	0.035180
H	-7.159849	-3.200173	0.678850
H	-0.772159	-2.328660	-3.592630
C	-0.416681	1.075040	0.507260
C	-1.071341	1.055330	1.746980
C	-0.673671	2.168290	-0.331240
C	-1.964511	2.065079	2.117260
H	-0.892100	0.228180	2.428480
C	-1.543292	3.195399	0.045660
H	-0.178241	2.221690	-1.294730
C	-2.205582	3.147199	1.272350
H	-2.885792	3.936819	1.566480
C	-1.768482	4.345779	-0.893610
C	-2.678451	1.940619	3.433110
F	-0.612482	4.757240	-1.469380
F	-2.316053	5.416249	-0.280300
F	-2.602312	4.023769	-1.923260
F	-3.432742	3.023089	3.722800
F	-1.814891	1.765679	4.460870
F	-3.508891	0.864359	3.447200
O	1.622650	-0.442279	1.065340
C	2.409010	0.292721	1.806800
C	2.598159	1.762071	1.659770
C	2.601159	2.431871	0.425490
C	2.800429	2.500521	2.838650
C	2.781048	3.813581	0.383190
H	2.452909	1.874811	-0.491620
C	2.979628	3.880531	2.791820
H	2.797219	1.989001	3.798850
C	2.969038	4.540601	1.560520
H	2.766888	4.324521	-0.575220
H	3.117818	4.440271	3.712590
H	3.103687	5.618081	1.519780
H	2.405850	-0.022859	2.854510
C	4.305490	-0.421618	1.651440
H	4.198960	-1.243778	2.349380
H	4.858530	0.428422	2.030310
C	4.450180	-0.762018	0.297830
C	4.953330	0.165172	-0.723010
C	4.596580	-0.020008	-2.073500
C	5.800189	1.238153	-0.379000
C	5.064869	0.856532	-3.046670
H	3.920870	-0.819008	-2.350210
C	6.281379	2.097523	-1.360980
H	6.107729	1.385523	0.650680
C	5.911759	1.911093	-2.695920
H	4.757499	0.717912	-4.078630
H	6.944749	2.912253	-1.085790
H	6.282919	2.588283	-3.460500

O	3.994501	-1.908618	-0.157010
Si	4.020911	-3.539368	0.486400
C	3.194412	-4.536419	-0.858710
H	2.107262	-4.429079	-0.796710
H	3.532922	-4.231528	-1.854780
H	3.431412	-5.600538	-0.733560
C	3.143231	-3.680479	2.138670
H	2.194701	-3.135319	2.149750
H	2.901132	-4.737399	2.309210
H	3.760511	-3.348918	2.981690
C	5.857942	-3.927387	0.644230
H	5.997472	-4.946837	1.024810
H	6.365821	-3.860437	-0.324390
H	6.363811	-3.246567	1.338970

TS 269

B3LYP/6-31G(d) = -3573.919713
B3LYP-D3(BJ)/def2-TZVPP/IEFPCM(propanonitrile) = -3575.495213
B3LYP-D3(BJ)/def2-TZVPP/IEFPCM(propanonitrile)//B3LYP-D3(BJ)/6-31G(d) Free Energy (Quasiharmonic) = -3574.778511

Frequencies (Top 3 out of 300)

1. -277.3618 cm^{-1}
2. 11.4914 cm^{-1}
3. 12.0925 cm^{-1}

B3LYP/6-31G(d) Molecular Geometry in Cartesian Coordinates

B	0.621118	0.605180	0.880257
O	0.777914	1.250491	2.201247
N	0.669434	1.839850	-0.067016
C	0.763269	3.084444	0.704181
S	0.955938	1.849013	-1.685417
C	0.831111	2.583931	2.151540
O	0.910159	3.301690	3.123663
H	-0.388297	4.462694	-0.486752
H	1.709314	3.606371	0.493196
C	-0.378481	4.122232	0.550512
C	-1.758317	3.676819	0.956862
C	-2.868865	3.352570	0.091419
C	-3.993550	3.086621	0.924377
N	-3.566536	3.205716	2.231331
C	-2.235167	3.584894	2.242446
H	-1.716621	3.755892	3.175517
C	-5.396204	2.771652	-0.979208
C	-3.032610	3.293520	-1.304419
H	-6.110807	2.617199	1.058693
H	-4.159640	3.166941	3.045423
C	-4.291068	3.008698	-1.824368
H	-6.374011	2.582215	-1.416079
H	-2.184323	3.462140	-1.960851
C	-5.260074	2.797062	0.405577
H	-4.430942	2.982445	-2.902049
C	2.719339	2.128987	-1.924771
O	0.282942	3.013564	-2.286137
O	0.655756	0.498279	-2.187530
C	5.470805	2.579448	-2.237135
C	3.201105	3.436165	-2.025850
C	3.595002	1.042311	-1.997617
C	4.958593	1.275981	-2.155555
C	4.570821	3.650936	-2.177481
H	2.507114	4.269746	-2.018812
H	3.208653	0.030447	-1.953388
H	5.637945	0.429399	-2.221418
H	4.943724	4.668943	-2.261082
C	6.955936	2.817089	-2.373149
H	7.424456	2.066744	-3.019237
H	7.454358	2.759498	-1.396435
H	7.167726	3.806371	-2.791148
H	-0.073965	4.973342	1.171510
C	1.748030	-0.543211	0.684776
C	2.945591	-0.478371	1.417145
C	1.591713	-1.634478	-0.182545
C	3.939219	-1.453055	1.290195
H	3.101248	0.344793	2.107149
C	2.579847	-2.617342	-0.307326
H	0.690497	-1.708853	-0.779696
C	3.762395	-2.533915	0.426319
H	4.527868	-3.294599	0.329651
C	2.374741	-3.741417	-1.282374
C	5.176068	-1.378017	2.143703
F	2.585726	-3.346735	-2.561450
F	1.102556	-4.219720	-1.234400
F	3.199416	-4.785910	-1.051983
F	4.985309	-1.958875	3.353675
F	5.552874	-0.102836	2.378961
F	6.225921	-2.011385	1.570035
O	-0.793167	0.044432	0.747936
C	-1.625418	-0.570610	1.544184
C	-1.376639	-0.881033	2.986778
C	-0.158787	-1.359557	3.491081
C	-2.456333	-0.706067	3.866446
C	-0.025061	-1.641417	4.847778
H	0.683680	-1.506500	2.826362
C	-2.319447	-0.983597	5.225639
H	-3.404101	-0.335776	3.482055
C	-1.101660	-1.453660	5.718977
H	0.925496	-2.004848	5.227604
H	-3.160159	-0.831382	5.897003
H	-0.989882	-1.672065	6.777470
H	-2.659190	-0.256091	1.377332
C	-1.849195	-2.426072	0.821491
H	-0.805190	-2.713041	0.750965
H	-2.420181	-2.885557	1.620030
C	-2.536822	-2.168743	-0.372060
C	-4.017023	-2.110225	-0.381557
C	-4.682553	-0.981154	-0.888683
C	-4.765441	-3.167263	0.164566
C	-6.074706	-0.921898	-0.857554
H	-4.115053	-0.134341	-1.261050
C	-6.157085	-3.112364	0.167832
H	-4.253977	-4.042093	0.555297
C	-6.813427	-1.989340	-0.342592
H	-6.575251	-0.032123	-1.225637
H	-6.728830	-3.943639	0.570246
H	-7.898900	-1.943499	-0.330927
O	-1.852149	-1.819748	-1.427930
Si	-2.046181	-1.788092	-3.173939
C	-2.440821	-0.039596	-3.708838
H	-3.435874	0.300642	-3.407592
H	-1.694319	0.650236	-3.302878
H	-2.386092	0.017095	-4.804016
C	-3.388978	-3.028888	-3.628905
H	-4.390399	-2.714171	-3.321015
H	-3.398375	-3.149642	-4.719899
H	-3.191506	-4.016291	-3.195428
C	-0.372485	-2.339743	-3.791905
H	-0.383617	-2.421333	-4.886270
H	0.390756	-1.606518	-3.514009
H	-0.079756	-3.311064	-3.382608

TS 270

B3LYP/6-31G(d) = -3573.922541
B3LYP-D3(BJ)/def2-TZVPP/IEFPCM(propanonitrile) = -3575.494056
B3LYP-D3(BJ)/def2-TZVPP/IEFPCM(propanonitrile)//B3LYP-D3(BJ)/6-31G(d) Free Energy (Quasiharmonic) = -3574.778472

Frequencies (Top 3 out of 300)

1. -217.9463 cm^{-1}
2. 4.3131 cm^{-1}
3. 15.0869 cm^{-1}

B3LYP/6-31G(d) Molecular Geometry in Cartesian Coordinates

B	-0.132545	-0.724358	0.903509
O	-0.619553	-1.233888	2.198727
N	-0.160479	0.817270	1.146429
C	-0.535403	1.101938	2.546774
S	0.775036	1.897224	0.314731
C	-0.737186	-0.295139	3.140469
O	-0.982267	-0.513674	4.305076
H	-1.579211	2.994046	2.428508
H	0.298456	1.577446	3.078037
C	-1.799499	1.980269	2.777775
C	-3.080922	1.497188	2.156864
C	-4.057218	0.586374	2.718646
C	-5.097301	0.445087	1.758591
N	-4.781694	1.253802	0.684484
C	-3.566166	1.864427	0.925305
H	-3.148265	2.546909	0.198624
C	-6.288174	-1.049300	3.182938
C	-4.167577	-0.107628	3.937960
H	-6.991336	-0.469786	1.220079
H	-5.218618	1.202454	-0.223337
C	-5.277278	-0.913859	4.156412
H	-7.140560	-1.693628	3.381945
H	-3.381659	-0.038113	4.682695
C	-6.212522	-0.371815	1.972212
H	-5.365199	-1.458936	5.092444
C	-0.257152	3.259645	-0.247124
O	1.763378	2.503114	1.237425
O	1.241864	1.199484	-0.890523
C	-1.840547	5.383827	-1.164413
C	-0.321385	4.442330	0.491397
C	-0.952873	3.132636	-1.451485
C	-1.743982	4.190473	-1.895663
C	-1.115413	5.491646	0.030702
H	0.254234	4.539623	1.405644
H	-0.872184	2.223159	-2.035230
H	-2.293403	4.084321	-2.827774
H	-1.169348	6.411257	0.608277
C	-2.676087	6.535582	-1.669673
H	-3.527818	6.183623	-2.260615
H	-3.060962	7.143908	-0.844873
H	-2.083305	7.197558	-2.314891
H	-1.909353	2.044142	3.867202
C	-0.966043	-1.315746	-0.335654
C	-0.892886	-2.674827	-0.677536
C	-1.853010	-0.524805	-1.072273
C	-1.658906	-3.212376	-1.714916
C	-2.617222	-1.050757	-2.118683
H	-1.958462	0.521019	-0.813664
C	-2.525812	-2.401154	-2.449337
H	-3.118902	-2.815914	-3.256106
C	-3.576043	-0.159644	-2.853697
C	-1.508125	-4.659393	-2.091011
F	-3.084022	1.095378	-3.015530
F	-3.885792	-0.629667	-4.079278
F	-4.759529	-0.012297	-2.190067
F	-1.148697	-5.427262	-1.040923
F	-2.646031	-5.175136	-2.604425
F	-0.546985	-4.838417	-3.043388
O	1.303928	-1.359638	0.800473
C	2.351242	-0.982795	1.453244
C	2.958383	-1.907143	2.429459
C	2.589289	-3.261298	2.465586
C	3.866100	-1.412416	3.380987
C	3.132982	-4.106322	3.428514
H	1.862639	-3.630796	1.750392
C	4.402525	-2.259190	4.346594
H	4.132497	-0.358336	3.371581
C	4.041347	-3.609301	4.367823
H	2.839018	-5.151589	3.456697
H	5.093999	-1.867464	5.087093
H	4.458359	-4.270491	5.122361
H	2.482126	0.085665	1.642143
C	4.092491	-1.035203	0.053000
H	3.999679	-2.105749	-0.068058
H	4.810134	-0.712568	0.796590
C	3.890974	-0.229407	-1.061477
C	3.180204	-0.679230	-2.267195
C	3.236482	0.096562	-3.439936
C	2.450362	-1.881896	-2.284071
C	2.601898	-0.331709	-4.600276
H	3.779064	1.034082	-3.426800
C	1.802595	-2.299856	-3.442742
H	2.355766	-2.475611	-1.382880
C	1.883609	-1.530491	-4.605346
H	2.660043	0.273105	-5.501019
H	1.219479	-3.214384	-3.431196
H	1.381977	-1.861226	-5.510815
O	4.332136	1.019940	-1.121846
Si	5.486715	1.992607	-0.296850
C	7.180122	1.269062	-0.690266
H	7.971690	1.865191	-0.219537
H	7.278886	0.241158	-0.322652
H	7.368457	1.257717	-1.769698
C	5.170876	2.062770	1.553634
H	5.754129	2.886314	1.985738
H	4.112491	2.260932	1.754712
H	5.470360	1.146909	2.074840
H	-0.235097	-3.331803	-0.117734
C	5.219423	3.672358	-1.081759
H	5.925167	4.410981	-0.682863
H	5.350500	3.634928	-2.168854
H	4.204464	4.029193	-0.875027

TS 271

B3LYP/6-31G(d) = -3573.922553
B3LYP-D3(BJ)/def2-TZVPP/IEFPCM(propanonitrile) = -3575.494026
B3LYP-D3(BJ)/def2-TZVPP/IEFPCM(propanonitrile)//B3LYP-D3(BJ)/6-31G(d) Free Energy (Quasiharmonic) = -3574.778384

Frequencies (Top 3 out of 300)

1. -218.0423 cm^{-1}
2. 4.2694 cm^{-1}
3. 15.0858 cm^{-1}

B3LYP/6-31G(d) Molecular Geometry in Cartesian Coordinates

B	-0.135414	-0.718410	0.905971
O	-0.626638	-1.222712	2.201582
N	-0.160828	0.824329	1.143662
C	-0.541428	1.114185	2.541457
S	0.782923	1.898793	0.314269
C	-0.746685	-0.280515	3.139581
O	-0.996563	-0.494579	4.303968
H	-1.585981	3.005112	2.408634
H	0.290232	1.591456	3.074581
C	-1.806861	1.993153	2.762750
C	-3.085083	1.504682	2.139397
C	-4.064780	0.600034	2.705121
C	-5.099207	0.448378	1.740561
N	-4.777152	1.245346	0.659534
C	-3.562859	1.858240	0.900995

H	-3.139871	2.531739	0.168884
C	-6.298227	-1.030719	3.174030
C	-4.181906	-0.081103	3.930994
H	-6.990223	-0.472233	1.201138
H	-5.207330	1.183107	-0.250878
C	-5.292823	-0.885039	4.151712
H	-7.151756	-1.672909	3.375033
H	-3.399961	-0.003937	4.679180
C	-6.215674	-0.366184	1.956576
H	-5.385996	-1.420326	5.092870
C	-0.240583	3.269787	-0.242706
O	1.244478	1.200024	-0.892431
O	1.776365	2.495489	1.237607
C	-1.802950	5.411394	-1.156075
C	-0.935331	3.152986	-1.448723
C	-0.291279	4.452610	0.496691
C	-1.072694	5.511577	0.036567
C	-1.713957	4.220413	-1.892152
H	-0.857163	2.246932	-2.038191
H	0.290783	4.544858	1.407371
H	-1.109873	6.433984	0.610955
H	-2.256227	4.125286	-2.829611
C	-2.673741	6.550664	-1.628893
H	-3.692038	6.457928	-1.228689
H	-2.753744	6.568092	-2.720618
H	-2.282223	7.518509	-1.299283
H	-1.921396	2.063026	3.851300
C	-0.967660	-1.311867	-0.332998
C	-0.908392	-2.675150	-0.660264
C	-1.839401	-0.518034	-1.084429
C	-1.673177	-3.214181	-1.697882
H	-0.262738	-3.334203	-0.088814
C	-2.603221	-1.045721	-2.130086
H	-1.933341	0.531679	-0.838061
C	-2.525555	-2.400499	-2.446354
H	-3.118082	-2.816570	-3.252880
C	-3.548317	-0.151867	-2.879245
C	-1.535402	-4.666348	-2.058749
F	-3.846775	-0.624734	-4.106516
F	-3.048208	1.099884	-3.039595
F	-4.739013	0.003749	-2.229782
F	-1.198000	-5.428520	-0.997291
F	-0.564767	-4.865544	-2.997384
F	-2.673046	-5.173369	-2.581345
O	1.299283	-1.357802	0.808058
C	2.348318	-0.981248	1.458348
C	2.952304	-1.902468	2.439462
C	3.863081	-1.406007	3.387161
C	2.577352	-3.254755	2.483991
C	4.396725	-2.249342	4.357310
H	4.134100	-0.353179	3.371125
C	3.118224	-4.096371	3.451490
H	1.848456	-3.625286	1.771618
C	4.029633	-3.597705	4.386973
H	5.090647	-1.856248	5.094790
H	2.819719	-5.140153	3.486217
H	4.444455	-4.256183	5.145083
H	2.483610	0.087679	1.641174
C	4.087466	-1.049239	0.056632
H	4.808058	-0.725082	0.796703
H	3.989894	-2.120096	-0.057642
C	3.886401	-0.249275	-1.062102
C	3.169891	-0.702969	-2.262952
C	3.223480	0.067271	-3.439512
C	2.437277	-1.904074	-2.271588
C	2.583599	-0.364923	-4.595459
H	3.768124	1.003653	-3.432695
C	1.784423	-2.326039	-3.425962
H	2.344768	-2.493568	-1.367428
C	1.862772	-1.562206	-4.592371
H	2.639626	0.235645	-5.499170
H	1.199627	-3.239407	-3.408066
H	1.357152	-1.896039	-5.494478
O	4.332849	0.997776	-1.131036
Si	5.494983	1.969283	-0.315332
C	5.184819	2.052190	1.535602
H	4.127957	2.256422	1.738627
H	5.481422	1.138040	2.061466
H	5.773242	2.875503	1.961082
C	5.234225	3.645630	-1.109716
H	4.221510	4.008882	-0.903159
H	5.944476	4.383128	-0.716782
H	5.362815	3.601017	-2.196839
C	7.183478	1.234732	-0.709398
H	7.278112	0.208483	-0.336124
H	7.368529	1.216196	-1.789299
H	7.979459	1.829535	-0.244472

TS 272

B3LYP/6-31G(d) = -3573.912095
B3LYP-D3(BJ)/def2-TZVPP/IEFPCM(propanonitrile) = -3575.494113
B3LYP-D3(BJ)/def2-TZVPP/IEFPCM(propanonitrile)//B3LYP-D3(BJ)/6-31G(d) Free Energy (Quasiharmonic) = -3574.778172

Frequencies (Top 3 out of 300)

1. -272.2660 cm^{-1}
2. 7.8732 cm^{-1}
3. 11.5933 cm^{-1}

B3LYP/6-31G(d) Molecular Geometry in Cartesian Coordinates

B	0.532572	-0.270190	-0.089549
O	0.520081	0.071335	-1.530780
N	1.276348	-1.629387	-0.108406
C	1.585382	-2.044459	-1.481615
S	1.967504	-2.408413	1.173861
C	1.096675	-0.851834	-2.306422
O	1.208346	-0.739837	-3.510322
H	1.307471	-4.177469	-1.377433
H	2.672654	-2.129189	-1.636418
C	0.942976	-3.360654	-2.004267
C	-0.558763	-3.379256	-2.073118
C	-1.486657	-3.910434	-1.100201
C	-2.795529	-3.723845	-1.623245
N	-2.661343	-3.124321	-2.859313
C	-1.321223	-2.909803	-3.117705
H	-1.002783	-2.450692	-4.043675
C	-3.769250	-4.726544	0.305975
C	-1.341096	-4.530932	0.154541
H	-4.937159	-3.952443	-1.343741
H	-3.423702	-2.778908	-3.420419
C	-2.480145	-4.932556	0.840279
H	-4.640345	-5.045907	0.872188
H	-0.354822	-4.675359	0.584386
C	-3.945479	-4.119308	-0.931245
H	-2.378625	-5.406830	1.812713
C	3.711851	-1.952495	1.193147
O	1.949543	-3.855763	0.912128
O	1.367033	-1.851974	2.390086
C	6.423346	-1.233990	1.236521
C	4.634966	-2.686036	0.445667
C	4.132083	-0.878033	1.981045
C	5.479676	-0.530451	2.000352
C	5.980591	-2.316867	0.467151
H	4.308322	-3.555859	-0.114279
H	3.406464	-0.345577	2.586031
H	5.807696	0.299159	2.622481
H	6.699618	-2.891658	-0.111757
C	7.876899	-0.824297	1.245074

H	8.025932	0.102567	0.675658
H	8.514321	-1.593753	0.798597
H	8.234812	-0.637558	2.264009
H	1.360071	-3.505443	-3.008389
C	-0.947610	-0.237165	0.561200
C	-1.154626	-0.125547	1.947479
C	-2.084684	-0.287065	-0.251879
C	-2.438625	-0.074889	2.489514
H	-0.299315	-0.097071	2.611029
C	-3.375593	-0.214027	0.285768
H	-1.967750	-0.376037	-1.325714
C	-3.562476	-0.113333	1.661050
H	-4.560654	-0.069451	2.082226
C	-4.554485	-0.115421	-0.634154
C	-2.645764	0.107836	3.966902
F	-4.437103	-0.896446	-1.734012
F	-4.701328	1.164292	-1.107107
F	-5.720265	-0.432627	-0.036428
F	-3.664258	-0.653332	4.431598
F	-2.972930	1.396938	4.266458
F	-1.549963	-0.192060	4.689933
O	1.319310	0.822719	0.672842
C	2.010891	1.902239	0.536054
C	3.342815	1.976048	-0.123190
C	3.759217	1.110807	-1.144441
C	4.231880	2.963457	0.335432
C	5.033836	1.232566	-1.692257
H	3.088163	0.349677	-1.514508
C	5.507486	3.082532	-0.209378
H	3.922100	3.634316	1.134350
C	5.910904	2.216601	-1.229029
H	5.342724	0.555516	-2.483216
H	6.185600	3.846407	0.160683
H	6.903387	2.309132	-1.661487
H	1.934160	2.551478	1.414375
C	1.095497	3.426915	-0.653082
H	1.522615	3.207007	-1.622144
H	1.600751	4.205596	-0.096308
C	-0.295466	3.332399	-0.535969
C	-1.038247	3.729740	0.671974
C	-0.419490	4.444278	1.717921
C	-2.398578	3.387620	0.799362
C	-1.135876	4.795786	2.856287
H	0.621580	4.740109	1.642513
C	-3.107226	3.733913	1.945180
H	-2.883177	2.822635	0.012842
C	-2.480716	4.435675	2.976546
H	-0.645220	5.347521	3.652830
H	-4.148445	3.442060	2.039953
H	-3.035586	4.696989	3.872876
O	-1.025475	2.772876	-1.479848
Si	-1.099965	2.864659	-3.215985
C	0.568487	2.745596	-4.066746
H	1.254695	3.561189	-3.815393
H	1.048218	1.782366	-3.864379
H	0.391845	2.797805	-5.149913
C	-1.902385	4.540274	-3.527688
H	-1.280862	5.363022	-3.154469
H	-2.050095	4.701112	-4.602746
H	-2.881517	4.610849	-3.041110
C	-2.205862	1.439939	-3.706823
H	-2.516235	1.554450	-4.753119
H	-1.663386	0.492930	-3.620081
H	-3.107680	1.385992	-3.089895

TS 273

B3LYP/6-31G(d) = -3573.921991
B3LYP-D3(BJ)/def2-TZVPP/IEFPCM(propanonitrile) = -3575.492818
B3LYP-D3(BJ)/def2-TZVPP/IEFPCM(propanonitrile)//B3LYP-D3(BJ)/6-31G(d) Free Energy (Quasiharmonic) = -3574.777725

Frequencies (Top 3 out of 300)

1. -189.7431 cm^{-1}
2. 8.8109 cm^{-1}
3. 10.0371 cm^{-1}

B3LYP/6-31G(d) Molecular Geometry in Cartesian Coordinates

B	0.057124	-0.415881	-0.924864
O	0.837201	-0.745001	-2.133286
N	1.125774	-0.601967	0.190258
C	2.457524	-0.831769	-0.404222
S	0.968064	-0.058069	1.749339
C	2.121019	-1.027596	-1.888633
O	2.907034	-1.384132	-2.737088
H	3.420095	-2.756540	-0.644115
H	3.072364	0.078064	-0.345025
C	3.279724	-2.031892	0.166130
C	4.622727	-1.659911	0.728557
C	5.819248	-1.340030	-0.016883
C	6.849104	-1.079929	0.929844
N	6.294310	-1.231026	2.185142
C	4.960482	-1.577026	2.057120
H	4.337277	-1.725403	2.927270
C	8.416085	-0.663971	-0.817907
C	6.116836	-1.249827	-1.389169
H	8.926457	-0.547812	1.281911
H	6.782638	-1.114976	3.058883
C	7.409549	-0.913782	-1.773464
H	9.417366	-0.403602	-1.151235
H	5.340252	-1.426405	-2.128230
C	8.150794	-0.742165	0.544964
H	7.651418	-0.838529	-2.830380
C	1.481337	1.661554	1.779193
O	1.907367	-0.802554	2.601181
O	-0.475570	-0.111637	2.037759
C	2.281169	4.342094	1.617991
C	0.517597	2.672229	1.811068
C	2.842803	1.974037	1.720506
C	3.229533	3.311127	1.633517
C	0.925886	4.000893	1.734408
H	-0.532440	2.417805	1.891826
H	3.588839	1.186342	1.754106
H	4.287241	3.554939	1.572425
H	0.176020	4.788149	1.752543
C	2.694702	5.783559	1.447080
H	3.766253	5.920503	1.622167
H	2.149203	6.443506	2.131135
H	2.476742	6.121492	0.425728
O	-1.023904	-1.539174	-0.842721
C	-0.946630	-2.557225	-0.050264
H	-0.756629	-2.362429	1.006718
H	2.676974	-2.511801	0.939076
C	-0.538604	-3.865560	-0.584367
C	-0.361051	-4.046695	-1.967169
C	-0.238662	-4.918460	0.298751
C	0.094173	-5.269501	-2.453845
H	-0.546500	-3.213156	-2.635662
C	0.216993	-6.136742	-0.193049
H	-0.349988	-4.769963	1.370463
C	0.377931	-6.315537	-1.571391
H	0.243439	-5.402736	-3.521312
H	0.456587	-6.943873	0.493157
H	0.738268	-7.266064	-1.954923
C	-0.649462	1.025461	-1.081044
C	-0.003139	2.014645	-1.843674
C	-1.836265	1.394041	-0.436425
C	-0.501700	3.314345	-1.936731

H	0.908601	1.765240	-2.376412
C	-2.344513	2.696672	-0.531184
H	-2.357280	0.665792	0.173540
C	-1.680816	3.666159	-1.278378
H	-2.071291	4.674354	-1.347751
C	-3.577429	3.056661	0.244669
C	0.272159	4.374501	-2.671108
F	-4.630984	2.247480	-0.059090
F	-3.382088	2.923556	1.582707
F	1.027768	3.863258	-3.664162
F	-3.985320	4.323591	0.028641
F	1.115271	5.040021	-1.838715
F	-0.542233	5.307668	-3.217381
C	-3.052388	-3.201421	0.432812
H	-2.760583	-3.901416	1.203064
H	-3.227029	-3.628234	-0.546035
C	-3.694103	-2.026441	0.779366
O	-4.280658	-1.262729	-0.138466
Si	-4.986239	-1.581257	-1.687101
C	-6.142992	-3.055322	-1.493036
H	-6.893479	-2.867760	-0.716555
H	-6.678985	-3.231241	-2.434147
H	-5.618031	-3.981344	-1.236250
C	-5.942242	-0.010802	-2.041394
H	-5.296116	0.870207	-1.991283
H	-6.388549	-0.051009	-3.042449
H	-6.752856	0.132586	-1.318165
C	-3.655570	-1.887409	-2.975510
H	-3.167661	-2.860957	-2.859772
H	-4.103046	-1.858746	-3.977252
H	-2.876870	-1.119754	-2.929259
C	-3.711327	-1.461396	2.141033
C	-4.264536	-0.185968	2.355862
C	-3.187899	-2.164043	3.243639
C	-4.279825	0.374036	3.629690
H	-4.664360	0.367769	1.516795
C	-3.213688	-1.605103	4.515573
H	-2.757805	-3.151622	3.116114
C	-3.757355	-0.332812	4.713317
H	-4.690406	1.369468	3.769346
H	-2.802599	-2.159132	5.354337
H	-3.767960	0.104907	5.707647

TS 274

B3LYP/6-31G(d) = -3573.922965
B3LYP-D3(BJ)/def2-TZVPP/IEFPCM(propanonitrile) = -3575.493673
B3LYP-D3(BJ)/def2-TZVPP/IEFPCM(propanonitrile)//B3LYP-D3(BJ)/6-31G(d) Free Energy (Quasiharmonic) = -3574.777621

Frequencies (Top 3 out of 300)

1. -277.5011 cm^{-1}
2. 9.3249 cm^{-1}
3. 10.3103 cm^{-1}

B3LYP/6-31G(d) Molecular Geometry in Cartesian Coordinates

B	0.077280	-0.231110	-0.256760
O	-0.359100	-0.686910	-1.596090
N	0.651490	1.212711	-0.578520
C	0.608650	1.422281	-2.040870
S	0.204890	2.505990	0.364860
C	-0.112360	0.186390	-2.580210
O	-0.407110	-0.003350	-3.739200
H	2.462910	2.487271	-2.357420
H	-0.008280	2.291390	-2.300990
C	1.989170	1.577161	-2.737800
C	2.920090	0.404791	-2.581260
C	4.094690	0.324621	-1.745470
C	4.625480	-0.986669	-1.888780
N	3.830220	-1.652869	-2.797830
C	2.802630	-0.820379	-3.196450
H	2.059000	-1.163439	-3.902610
C	6.388370	-0.503619	-0.358970
C	4.761150	1.229861	-0.901090
H	6.129830	-2.434989	-1.301120
H	3.865780	-2.647269	-2.960940
C	5.896920	0.811071	-0.220070
H	7.272260	-0.805829	0.196390
H	4.391690	2.243581	-0.779650
C	5.761100	-1.418669	-1.195410
H	6.415690	1.504291	0.437230
C	1.443490	3.775201	0.071200
O	0.321030	2.103000	1.773210
O	-1.080830	3.086580	-0.102170
C	3.353840	5.791961	-0.289940
C	1.184810	4.818431	-0.817470
C	2.634800	3.734381	0.800130
C	3.577620	4.742191	0.614940
C	2.144390	5.815341	-0.996020
H	0.236670	4.857750	-1.342850
H	2.808740	2.931611	1.508620
H	4.502200	4.716681	1.186750
H	1.944100	6.629881	-1.687760
C	4.399530	6.860771	-0.500530
H	4.853040	7.169071	0.447900
H	5.211350	6.495061	-1.142780
H	3.975530	7.748701	-0.979640
H	1.769400	1.754041	-3.798140
C	1.123740	-1.240599	0.438130
C	1.532560	-2.420879	-0.194650
C	1.665400	-0.966679	1.704230
C	2.451020	-3.291529	0.401590
C	2.580370	-1.834229	2.304700
H	1.377090	-0.054559	2.218680
C	2.981580	-3.003809	1.657660
H	3.694540	-3.673449	2.123580
C	3.093750	-1.529539	3.685200
C	2.903030	-4.514769	-0.341280
F	3.296430	-0.206289	3.869380
F	4.264170	-2.157469	3.945510
F	2.219480	-1.924629	4.644270
F	3.920440	-4.245419	-1.216980
F	1.911020	-5.055259	-1.085910
F	3.364970	-5.479809	0.480960
O	-1.123700	-0.066310	0.650190
C	-2.335180	-0.519430	0.773880
C	-2.746130	-1.937800	0.585630
C	-2.189460	-2.816990	-0.357690
C	-3.743290	-2.414940	1.454570
C	-2.612220	-4.144280	-0.408010
H	-1.443300	-2.458070	-1.054470
C	-4.161220	-3.742250	1.401430
H	-4.179720	-1.743140	2.190120
C	-3.592750	-4.612370	0.469500
H	-2.168220	-4.817120	-1.136350
H	-4.923260	-4.097020	2.089570
H	-3.912190	-5.650100	0.427240
H	-2.844650	-0.050370	1.618550
C	-3.344690	0.576840	-0.636890
H	-2.992930	0.046250	-1.512610
H	-2.800530	1.489410	-0.412200
C	-4.703190	0.526140	-0.326080
C	-5.630300	-0.488970	-0.851790
C	-5.255200	-1.364750	-1.889930
C	-6.931370	-0.589690	-0.319630
C	-6.151530	-2.313850	-2.370800
H	-4.267730	-1.305850	-2.331900
C	-7.823470	-1.539500	-0.804670
H	-7.227610	0.082400	0.476900

C	-7.436350	-2.405490	-1.830870
H	-5.846880	-2.980390	-3.172100
H	-8.822310	-1.605970	-0.382830
H	-8.134650	-3.145970	-2.211200
O	-5.241470	1.334230	0.576170
Si	-4.825630	2.869980	1.291030
C	-6.462410	3.338920	2.084350
H	-6.374530	4.296230	2.612370
H	-7.255890	3.443420	1.335770
H	-6.783370	2.587150	2.814500
C	-4.341970	4.063720	-0.072170
H	-3.318040	3.896460	-0.418410
H	-5.026110	4.001180	-0.926350
H	-4.394180	5.089000	0.316330
H	1.136190	-2.662839	-1.175160
C	-3.490590	2.621780	2.583280
H	-3.765810	1.842490	3.303940
H	-2.513130	2.380900	2.153630
H	-3.366620	3.554160	3.149740

TS 275

B3LYP/6-31G(d) = -3573.922529
B3LYP-D3(BJ)/def2-TZVPP/IEFPCM(propanonitrile) = -3575.494475
B3LYP-D3(BJ)/def2-TZVPP/IEFPCM(propanonitrile)//B3LYP-D3(BJ)/6-31G(d) Free Energy (Quasiharmonic) = -3574.777495

Frequencies (Top 3 out of 300)

1. -274.4930 cm^{-1}
2. 7.1328 cm^{-1}
3. 10.5210 cm^{-1}

B3LYP/6-31G(d) Molecular Geometry in Cartesian Coordinates

B	-0.231925	0.071528	0.392179
O	-0.521025	-0.396317	1.772039
N	0.337438	-1.262941	-0.247679
C	0.297428	-2.352389	0.745600
S	0.350707	-1.662919	-1.844683
C	-0.395572	-1.701179	1.937671
O	-0.744302	-2.301773	2.940012
H	2.160615	-3.386937	0.341141
H	-0.325459	-3.178739	0.384385
C	1.661334	-2.949100	1.210742
C	2.602896	-2.003445	1.914443
C	3.924056	-1.608013	1.482850
C	4.427102	-0.688027	2.444491
N	3.473680	-0.571265	3.434064
C	2.381981	-1.351283	3.106257
H	1.525893	-1.391662	3.764505
C	6.460566	-0.433746	1.228422
C	4.744498	-1.954509	0.395349
H	6.028829	0.641475	3.056619
H	3.470368	0.140544	4.148881
C	5.998997	-1.369121	0.279562
H	7.441792	0.016517	1.103582
H	4.405100	-2.669584	-0.347729
C	5.681984	-0.082218	2.324278
H	6.637655	-1.632831	-0.559653
C	2.005683	-2.148808	-2.351334
O	0.063590	-0.424736	-2.598120
O	-0.510426	-2.843847	-2.064741
C	4.516275	-2.900445	-3.340997
C	2.950411	-1.163924	-2.652981
C	2.296110	-3.500353	-2.539928
C	3.551893	-3.866465	-3.025411
C	4.194565	-1.548834	-3.143784
H	2.719437	-0.112819	-2.521906
H	1.538619	-4.248447	-2.332458
H	3.778758	-4.919361	-3.174345
H	4.929026	-0.782352	-3.377460
C	5.859267	-3.297259	-3.906464
H	6.667112	-2.691788	-3.481132
H	6.083005	-4.350228	-3.708884
H	5.886279	-3.152731	-4.994579
H	1.395823	-3.786412	1.869290
C	0.817431	1.301260	0.391901
C	1.384689	1.736507	1.601538
C	1.260298	1.927368	-0.779076
C	2.377983	2.715002	1.634387
H	1.058661	1.279850	2.527753
C	2.257541	2.909756	-0.752034
H	0.850063	1.626498	-1.738236
C	2.829389	3.307919	0.452118
H	3.614241	4.055632	0.470235
C	2.740438	3.471249	-2.058629
C	3.002080	3.146279	2.931013
F	3.374564	2.523430	-2.801047
F	3.610991	4.491549	-1.899614
F	1.719735	3.927083	-2.820839
F	2.731571	4.439222	3.223935
F	2.575609	2.408679	3.990587
F	4.353282	3.035798	2.901156
O	-1.604239	0.554197	-0.044265
C	-2.210904	0.747274	-1.189435
C	-3.081964	1.956485	-1.268991
C	-3.540393	2.399563	-2.518440
C	-3.394209	2.698009	-0.121750
C	-4.306404	3.558767	-2.619342
H	-3.279175	1.843092	-3.416264
C	-4.159521	3.858800	-0.224757
H	-3.007106	2.368305	0.836657
C	-4.620485	4.289858	-1.470666
H	-4.647678	3.898797	-3.593278
H	-4.388027	4.434475	0.668208
H	-5.211566	5.198371	-1.548908
H	-1.630665	0.540848	-2.089184
C	-3.357668	-0.798375	-1.563573
H	-2.580256	-1.552878	-1.654723
H	-3.799313	-0.468879	-2.497981
C	-4.222506	-0.921355	-0.465600
C	-5.530312	-0.243528	-0.411183
C	-5.975089	0.339702	0.789788
C	-6.338623	-0.144603	-1.557844
C	-7.196789	1.003878	0.840156
H	-5.340189	0.300827	1.668336
C	-7.569049	0.503980	-1.498101
H	-6.017966	-0.607617	-2.485588
C	-7.999078	1.081608	-0.301321
H	-7.521681	1.466229	1.767708
H	-8.193212	0.558062	-2.385205
H	-8.956462	1.593292	-0.258550
O	-3.768784	-1.528862	0.606125
Si	-4.353989	-2.649412	1.812975
C	-3.283276	-4.170269	1.597554
H	-3.683425	-4.999779	2.194466
H	-3.245575	-4.498221	0.552575
H	-2.264756	-3.968773	1.943824
C	-6.158316	-3.057465	1.457070
H	-6.837927	-2.218478	1.630642
H	-6.302900	-3.403208	0.426787
H	-6.463125	-3.877786	2.119747
C	-4.086043	-1.826046	3.471358
H	-4.371224	-2.518021	4.274295
H	-3.024000	-1.589511	3.599545
H	-4.675703	-0.911690	3.599130

TS 276

B3LYP/6-31G(d) = -3573.926601
B3LYP-D3(BJ)/def2-TZVPP/IEFPCM(propanonitrile) = -3575.494033
B3LYP-D3(BJ)/def2-TZVPP/IEFPCM(propanonitrile)//B3LYP-D3(BJ)/6-31G(d) Free Energy (Quasiharmonic) = -3574.777428

Frequencies (Top 3 out of 300)

1. -289.1404 cm^{-1}
2. 9.6367 cm^{-1}
3. 12.9324 cm^{-1}

B3LYP/6-31G(d) Molecular Geometry in Cartesian Coordinates

B	0.085486	-0.260232	0.253438
O	-0.215758	-1.455656	-0.559009
N	0.282021	0.815753	-0.882150
C	0.053114	0.212760	-2.206452
S	0.118615	2.450709	-0.706963
C	-0.411002	-1.199379	-1.858883
O	-0.902886	-1.986974	-2.637386
H	1.577198	1.198750	-3.374562
H	-0.762632	0.724049	-2.729235
C	1.277478	0.166438	-3.166950
C	2.452256	-0.635696	-2.685421
C	2.725799	-2.031064	-2.954134
C	3.928144	-2.359831	-2.269890
N	4.374036	-1.211224	-1.645994
C	3.472635	-0.191731	-1.879773
H	3.633774	0.788714	-1.453912
C	3.819723	-4.611053	-3.042822
C	2.081292	-3.032068	-3.704174
H	5.386792	-3.876132	-1.741355
H	5.089051	-1.195235	-0.934684
C	2.634514	-4.305481	-3.743865
H	4.220229	-5.620548	-3.083878
H	1.149129	-2.820178	-4.219109
C	4.481355	-3.644551	-2.295308
H	2.140042	-5.086098	-4.315979
C	1.688760	3.214139	-1.146327
O	-0.869070	2.936325	-1.694804
O	-0.093836	2.740323	0.719061
C	4.128521	4.422154	-1.816038
C	2.722705	3.248475	-0.205513
C	1.850070	3.794818	-2.404165
C	3.068104	4.391820	-2.730340
C	3.931302	3.849463	-0.549559
H	2.581618	2.835397	0.787614
H	1.023174	3.795135	-3.106298
H	3.191660	4.848458	-3.709293
H	4.732222	3.884862	0.185152
C	5.454671	5.044351	-2.182575
H	6.171670	4.279040	-2.508044
H	5.348338	5.762113	-3.002046
H	5.902415	5.564807	-1.329169
H	0.905697	-0.245613	-4.112953
C	1.334966	-0.454305	1.250894
C	2.238120	-1.518023	1.095722
C	1.593687	0.467310	2.274940
C	3.358356	-1.645100	1.922438
C	2.726203	0.355250	3.088132
H	0.911508	1.297910	2.425572
C	3.615350	-0.703298	2.921522
H	4.486339	-0.797732	3.559786
C	3.004321	1.439205	4.090028
C	4.347970	-2.755218	1.709511
F	3.408504	2.583006	3.472105
F	3.978541	1.103405	4.964067
F	1.907789	1.761983	4.809769
F	4.850146	-3.220738	2.874479
F	5.433641	-2.329766	0.984417
F	3.829113	-3.799562	1.039762
O	-1.111798	0.109975	1.111263
C	-2.366502	-0.176502	1.275621
C	-2.877072	-1.521495	1.650458
C	-2.213593	-2.727591	1.371681
C	-4.071549	-1.555742	2.392857
C	-2.739895	-3.933462	1.832647
H	-1.301752	-2.718816	0.788514
C	-4.591275	-2.762115	2.852583
H	-4.586928	-0.625625	2.621988
C	-3.923941	-3.956738	2.572610
H	-2.217180	-4.860267	1.613450
H	-5.509910	-2.769911	3.432389
H	-4.323957	-4.900881	2.932391
H	-2.904002	0.630123	1.781074
C	-3.197972	0.289247	-0.570967
H	-2.758360	-0.519514	-1.137749
H	-2.679776	1.238341	-0.651676
C	-4.584897	0.308455	-0.445897
C	-5.437579	-0.867274	-0.677718
C	-4.925422	-2.048973	-1.252395
C	-6.802491	-0.818316	-0.330101
C	-5.756266	-3.145310	-1.460625
H	-3.885609	-2.120907	-1.551295
C	-7.626777	-1.917678	-0.541478
H	-7.201680	0.088658	0.108380
C	-7.105854	-3.085304	-1.105754
H	-5.347783	-4.047326	-1.906147
H	-8.676489	-1.865737	-0.266681
H	-7.750740	-3.943655	-1.273176
O	-5.230868	1.375846	0.004575
Si	-4.880774	3.083149	0.077968
C	-3.642014	3.426468	1.444625
H	-2.624723	3.091147	1.214292
H	-3.588213	4.511301	1.606097
H	-3.955061	2.977229	2.394844
C	-6.574283	3.768635	0.511861
H	-6.533588	4.859451	0.617978
H	-7.311167	3.536982	-0.265394
H	-6.941691	3.357480	1.459083
H	2.066990	-2.253178	0.316230
C	-4.300732	3.650404	-1.612138
H	-4.359831	4.745515	-1.659714
H	-3.261889	3.372564	-1.817309
H	-4.939401	3.253133	-2.409684

TS 277

B3LYP/6-31G(d) = -3573.917591
B3LYP-D3(BJ)/def2-TZVPP/IEFPCM(propanonitrile) = -3575.493327
B3LYP-D3(BJ)/def2-TZVPP/IEFPCM(propanonitrile)//B3LYP-D3(BJ)/6-31G(d) Free Energy (Quasiharmonic) = -3574.777161

Frequencies (Top 3 out of 300)

1. -282.1601 cm^{-1}
2. 11.0644 cm^{-1}
3. 12.9175 cm^{-1}

B3LYP/6-31G(d) Molecular Geometry in Cartesian Coordinates

B	0.412794	-0.062978	0.584128
O	0.623197	-0.660076	1.914620
N	-0.417377	-1.187113	-0.116277
C	-0.353679	-2.425523	0.688550
S	-1.048142	-1.201252	-1.634412
C	0.180539	-1.911283	2.026571
O	0.195813	-2.556309	3.052273
H	0.014440	-3.973473	-0.767557
H	-1.364375	-2.811075	0.869904
C	0.494910	-3.613830	0.148373

C	1.952579	-3.358509	-0.111733
C	3.056188	-3.473389	0.817174
C	4.241499	-3.151537	0.097096
N	3.870173	-2.880257	-1.204771
C	2.500446	-2.990281	-1.316612
H	2.003597	-2.793906	-2.255251
C	5.582726	-3.510844	2.035755
C	3.167520	-3.827215	2.174250
H	6.398679	-2.908698	0.124107
H	4.456693	-2.455775	-1.907388
C	4.424120	-3.842455	2.767146
H	6.550870	-3.526724	2.529543
H	2.280785	-4.056720	2.757145
C	5.507005	-3.162177	0.692217
H	4.516775	-4.107658	3.817117
C	-2.533196	-2.211063	-1.500215
O	-0.197697	-1.882241	-2.628807
O	-1.446141	0.190609	-1.942258
C	-4.883554	-3.738122	-1.407381
C	-3.547802	-1.864500	-0.605462
C	-2.681815	-3.303985	-2.354230
C	-3.855374	-4.056517	-2.303170
C	-4.710142	-2.630278	-0.562979
H	-3.429272	-1.013634	0.055138
H	-1.886273	-3.549906	-3.048630
H	-3.971147	-4.906379	-2.971295
H	-5.494154	-2.366372	0.142797
C	-6.134260	-4.580633	-1.329283
H	-7.011633	-3.975482	-1.077297
H	-6.039274	-5.353356	-0.554922
H	-6.333249	-5.091746	-2.276537
O	-0.352692	1.242336	0.944745
C	-0.861081	2.189103	0.212264
H	-1.328589	1.891152	-0.725826
H	0.377764	-4.409782	0.894800
C	-0.189200	3.514126	0.221356
C	0.712717	3.863086	1.237869
C	-0.406738	4.404922	-0.841479
C	1.388160	5.079414	1.188588
H	0.895802	3.163607	2.046172
C	0.265639	5.624091	-0.886420
H	-1.079432	4.124799	-1.648473
C	1.162539	5.963814	0.129831
H	2.105550	5.328422	1.964570
H	0.102735	6.301805	-1.719743
H	1.694715	6.910212	0.090083
C	1.812144	0.431425	-0.063866
C	2.737810	1.044476	0.796522
C	2.175252	0.320402	-1.412213
C	3.953141	1.552019	0.334202
H	2.508531	1.114885	1.855307
C	3.409509	0.793380	-1.874003
H	1.509556	-0.157762	-2.122252
C	4.302681	1.424088	-1.009406
H	5.251140	1.801852	-1.371963
C	3.807194	0.538515	-3.298860
C	4.847807	2.297935	1.283408
F	4.770605	1.382925	-3.727739
F	4.310546	-0.725410	-3.461185
F	6.118702	2.388141	0.832198
F	2.768292	0.638901	-4.151368
F	4.889093	1.722363	2.504056
F	4.412718	3.573692	1.476733
C	-2.621372	2.783411	1.163345
H	-2.126170	3.110384	2.069087
H	-2.887233	3.581290	0.479403
C	-3.512433	1.712961	1.280727
O	-4.457285	1.472261	0.381360
Si	-5.187200	2.323067	-0.940806
C	-6.488791	1.098422	-1.503622
H	-7.035267	1.482321	-2.373406
H	-7.220140	0.897868	-0.712421
H	-6.029118	0.145912	-1.789655
C	-5.952304	3.896971	-0.249054
H	-5.204309	4.585197	0.159506
H	-6.490090	4.430987	-1.042439
H	-6.672777	3.673559	0.545910
C	-3.923582	2.661473	-2.284402
H	-3.279552	1.792300	-2.460249
H	-4.442859	2.890796	-3.223651
H	-3.285623	3.520288	-2.049279
C	-3.451635	0.722273	2.360918
C	-2.302476	0.580722	3.164737
C	-4.555998	-0.125904	2.592926
C	-2.249541	-0.405842	4.147158
H	-1.429500	1.194468	2.986831
C	-4.509260	-1.081615	3.599861
H	-5.445121	-0.016947	1.982177
C	-3.351690	-1.231515	4.371951
H	-1.336078	-0.545983	4.715118
H	-5.370134	-1.719475	3.779534
H	-3.307099	-1.995901	5.142375

TS 278

B3LYP/6-31G(d) = -3573.922211
B3LYP-D3(BJ)/def2-TZVPP/IEFPCM(propanonitrile) = -3575.492155
B3LYP-D3(BJ)/def2-TZVPP/IEFPCM(propanonitrile)//B3LYP-D3(BJ)/6-31G(d) Free Energy (Quasiharmonic) = -3574.777098

Frequencies (Top 3 out of 300)

1. -208.1711 cm^{-1}
2. 6.9195 cm^{-1}
3. 10.7980 cm^{-1}

B3LYP/6-31G(d) Molecular Geometry in Cartesian Coordinates

B	0.009074	-0.203748	-1.059350
O	0.799831	-0.430043	-2.285255
N	1.070253	-0.443002	0.042748
C	2.387082	-0.738785	-0.551757
S	0.967248	0.083386	1.612859
C	2.067692	-0.784348	-2.052334
O	2.853313	-1.097903	-2.918021
H	3.171847	-2.727899	-0.923664
H	3.076179	0.104656	-0.402854
C	3.088599	-2.049750	-0.066190
C	4.449941	-1.849305	0.537113
C	5.682954	-1.569023	-0.162956
C	6.712846	-1.484761	0.815055
N	6.123492	-1.696685	2.045753
C	4.767828	-1.914872	1.871361
H	4.117579	-2.085577	2.717622
C	8.342661	-1.052887	-0.869933
C	6.012751	-1.381645	-1.517993
H	8.821264	-1.169703	1.237310
H	6.605080	-1.709897	2.930766
C	7.336611	-1.126737	-1.855398
H	9.368766	-0.852306	-1.167118
H	5.237666	-1.420768	-2.278316
C	8.045952	-1.229016	0.477133
H	7.603996	-0.977807	-2.898217
C	1.763352	1.691675	1.677676
O	1.761815	-0.822172	2.457761
O	-0.465751	0.265103	1.882704
C	3.017154	4.196986	1.622892
C	3.144846	1.775906	1.864789
C	0.997012	2.844985	1.484348
C	1.628015	4.084543	1.456504
C	3.759260	3.028715	1.834084

H	3.727317	0.877013	2.037834
H	-0.076619	2.764338	1.360012
H	1.034728	4.978723	1.285105
H	4.835601	3.095370	1.971824
C	3.683324	5.551348	1.577074
H	3.379555	6.173106	2.428919
H	3.405761	6.095072	0.666432
H	4.773776	5.463748	1.600128
O	-1.056097	-1.359873	-1.076153
C	-0.862512	-2.504242	-0.507715
H	-0.308174	-2.507182	0.433211
H	2.434013	-2.522355	0.669275
C	-0.750149	-3.709770	-1.351558
C	-0.299981	-4.918453	-0.792808
C	-1.011275	-3.639779	-2.729870
C	-0.128016	-6.041849	-1.595109
H	-0.071622	-4.969428	0.269447
C	-0.841606	-4.767970	-3.528345
H	-1.309339	-2.690757	-3.161861
C	-0.405590	-5.969784	-2.963948
H	0.229832	-6.970050	-1.159019
H	-1.033968	-4.706689	-4.595587
H	-0.268096	-6.846090	-3.591302
C	-0.760391	1.207798	-1.129789
C	-0.138122	2.288755	-1.774785
C	-2.014775	1.443527	-0.553453
C	-0.728712	3.553750	-1.823289
H	0.825262	2.142436	-2.252183
C	-2.622417	2.702560	-0.619616
H	-2.520581	0.638683	-0.034377
C	-1.981517	3.768743	-1.249760
H	-2.448837	4.745479	-1.295914
C	-4.005368	2.891237	-0.068526
C	0.013506	4.715886	-2.423006
F	-4.957893	2.450419	-0.942460
F	-4.206991	2.197018	1.078531
F	-0.826147	5.623951	-2.972494
F	-4.293588	4.183199	0.190083
F	0.880801	4.331840	-3.381280
F	0.736784	5.381843	-1.483504
C	-2.709558	-3.172696	0.519433
H	-2.202682	-3.949105	1.075631
H	-3.192616	-3.497873	-0.391784
C	-3.265047	-2.103846	1.209557
O	-4.148279	-1.297467	0.630253
Si	-5.332091	-1.457839	-0.625149
C	-5.917252	-3.247874	-0.732260
H	-6.881663	-3.266390	-1.255928
H	-6.078263	-3.683672	0.260514
H	-5.236734	-3.904010	-1.284190
C	-6.725798	-0.357666	-0.026125
H	-6.379372	0.669030	0.121647
H	-7.538475	-0.333621	-0.762794
H	-7.142579	-0.721279	0.919999
C	-4.566082	-0.856250	-2.226684
H	-4.417675	0.227648	-2.200892
H	-3.591188	-1.320384	-2.409175
H	-5.221278	-1.086801	-3.076028
C	-2.900255	-1.720176	2.582018
C	-3.410521	-0.528847	3.130355
C	-2.058698	-2.523637	3.375258
C	-3.086663	-0.155016	4.429930
H	-4.038046	0.108478	2.520574
C	-1.737638	-2.145810	4.673103
H	-1.650641	-3.449879	2.985214
C	-2.251058	-0.960320	5.205373
H	-3.479353	0.773050	4.835028
H	-1.082797	-2.773783	5.269802
H	-1.995540	-0.664776	6.219182

TS 279

B3LYP/6-31G(d) = -3573.923586
B3LYP-D3(BJ)/def2-TZVPP/IEFPCM(propanonitrile) = -3575.492983
B3LYP-D3(BJ)/def2-TZVPP/IEFPCM(propanonitrile)//B3LYP-D3(BJ)/6-31G(d) Free Energy (Quasiharmonic) = -3574.777094

Frequencies (Top 3 out of 300)

1. -293.6489 cm^{-1}
2. 7.2242 cm^{-1}
3. 8.8487 cm^{-1}

B3LYP/6-31G(d) Molecular Geometry in Cartesian Coordinates

B	0.438431	0.142759	0.441688
O	1.098606	1.388639	-0.046848
N	0.130560	-0.567112	-0.927617
C	0.561561	0.272962	-2.056006
S	-0.044241	-2.197395	-1.161178
C	1.240819	1.448655	-1.360380
O	1.824034	2.350009	-1.942422
H	-1.023211	-0.070971	-3.487399
H	1.312092	-0.253340	-2.658837
C	-0.534528	0.795831	-3.032015
C	-1.576839	1.720818	-2.454239
C	-3.004061	1.498594	-2.403180
C	-3.588614	2.635138	-1.778775
N	-2.566966	3.520258	-1.504725
C	-1.366322	2.964126	-1.902318
H	-0.444474	3.513915	-1.777569
C	-5.771812	1.704025	-1.988114
C	-3.851285	0.466909	-2.843993
H	-5.382038	3.613863	-1.048237
H	-2.651067	4.329394	-0.908227
C	-5.219892	0.579610	-2.635789
H	-6.845724	1.757919	-1.830212
H	-3.445123	-0.409970	-3.338747
C	-4.964317	2.746872	-1.552005
H	-5.879999	-0.214438	-2.975253
C	-1.663327	-2.550865	-1.860221
O	0.943359	-2.632608	-2.168491
O	-0.040267	-2.811783	0.175989
C	-4.146600	-3.325449	-2.901605
C	-1.762673	-2.926726	-3.199757
C	-2.787409	-2.560982	-1.029117
C	-4.016155	-2.943899	-1.557024
C	-3.004765	-3.305355	-3.712115
H	-0.872858	-2.946150	-3.819652
H	-2.706846	-2.291865	0.017910
H	-4.889277	-2.949904	-0.909442
H	-3.082291	-3.603232	-4.755005
C	-5.483474	-3.768382	-3.447141
H	-5.757073	-4.759903	-3.063637
H	-6.282956	-3.078870	-3.152789
H	-5.471849	-3.827865	-4.539826
H	0.015622	1.298372	-3.838299
C	-0.851273	0.468393	1.353499
C	-1.534049	-0.538909	2.046157
C	-1.373020	1.768747	1.434226
C	-2.712498	-0.268462	2.752372
C	-2.540100	2.046186	2.146128
H	-0.863152	2.574117	0.920605
C	-3.226271	1.023976	2.807179
H	-4.141231	1.231302	3.350717
C	-3.108051	3.435749	2.197171
C	-3.440230	-1.413454	3.397066
F	-4.332594	3.505063	1.617928
F	-2.327026	4.346325	1.557100
F	-3.262841	3.876281	3.466893
F	-3.948205	-2.263392	2.464543

F	-4.476378	-1.008435	4.163823
F	-2.624307	-2.154335	4.181305
O	1.420250	-0.581300	1.351386
C	2.350321	-1.440678	1.028454
C	2.524021	-2.601097	1.946506
C	2.072104	-2.539675	3.270880
C	3.127621	-3.774043	1.472380
C	2.229883	-3.639231	4.111915
H	1.581745	-1.637557	3.621690
C	3.280460	-4.872620	2.314856
H	3.451405	-3.831484	0.435745
C	2.836616	-4.805435	3.638210
H	1.870730	-3.590154	5.136143
H	3.733929	-5.784722	1.936903
H	2.953647	-5.663206	4.294923
H	2.491961	-1.662683	-0.031564
C	4.164557	-0.594950	1.365779
H	4.741787	-1.510050	1.367409
H	3.930109	-0.180701	2.341116
C	4.434623	0.322478	0.341825
C	5.088157	-0.071931	-0.921798
C	4.727039	0.555440	-2.130019
C	6.066400	-1.085522	-0.941764
C	5.325468	0.165405	-3.325739
H	3.950418	1.312539	-2.133895
C	6.671898	-1.456398	-2.138210
H	6.378617	-1.558147	-0.015955
C	6.299828	-0.835032	-3.333417
H	5.024400	0.641207	-4.254448
H	7.436097	-2.228171	-2.138857
H	6.768057	-1.131716	-4.267805
O	3.967099	1.539563	0.457663
Si	4.457093	3.194568	0.551341
C	3.013412	4.024831	1.398803
H	2.885394	3.659570	2.423560
H	3.150349	5.112201	1.437791
H	2.093270	3.808391	0.847744
C	4.783054	3.905834	-1.151596
H	5.609652	3.405211	-1.666966
H	3.883676	3.826594	-1.770131
H	5.043368	4.968299	-1.058248
H	-1.152802	-1.555746	2.018980
C	6.020335	3.177259	1.602402
H	5.841734	2.728212	2.585973
H	6.830764	2.620721	1.116879
H	6.378205	4.201397	1.765571

TS 280

B3LYP/6-31G(d) = -3573.915788
B3LYP-D3(BJ)/def2-TZVPP/IEFPCM(propanonitrile) = -3575.492201
B3LYP-D3(BJ)/def2-TZVPP/IEFPCM(propanonitrile)//B3LYP-D3(BJ)/6-31G(d) Free Energy (Quasiharmonic) = -3574.777077

Frequencies (Top 3 out of 300)

1. -250.9916 cm^{-1}
2. 12.4033 cm^{-1}
3. 12.8047 cm^{-1}

B3LYP/6-31G(d) Molecular Geometry in Cartesian Coordinates

B	0.035729	-0.509797	0.541794
O	-0.014852	-0.888848	1.962678
N	0.512384	0.999976	0.640063
C	0.541716	1.400615	2.060053
S	1.495461	1.779012	-0.418844
C	0.315414	0.084537	2.805629
O	0.403124	-0.039685	4.008708
H	-0.206603	3.433210	2.098561
H	1.532037	1.776673	2.341606
C	-0.517275	2.461108	2.494370
C	-1.932737	2.184347	2.074413
C	-2.912253	1.326679	2.707345
C	-4.086055	1.370843	1.903644
N	-3.839927	2.239923	0.858273
C	-2.541391	2.698779	0.956573
H	-2.147206	3.380023	0.215578
C	-5.205261	-0.155294	3.353339
C	-2.919355	0.527667	3.865941
H	-6.118054	0.679413	1.577840
H	-4.395399	2.304876	0.018153
C	-4.060287	-0.202922	4.174256
H	-6.078786	-0.745243	3.617609
H	-2.036797	0.459806	4.494473
C	-5.235381	0.635848	2.211004
H	-4.068923	-0.830543	5.061389
C	0.840726	3.435291	-0.698198
O	1.389893	1.072363	-1.708662
O	2.834708	1.997308	0.176381
C	-0.145024	6.023130	-1.156647
C	-0.101029	3.648686	-1.708015
C	1.311912	4.503175	0.067415
C	0.811739	5.784388	-0.161036
C	-0.587385	4.936542	-1.925812
H	-0.440723	2.825803	-2.326864
H	2.072010	4.331998	0.822043
H	1.176714	6.613194	0.440655
H	-1.320121	5.100074	-2.712426
C	-0.659543	7.417813	-1.421283
H	-1.706026	7.404305	-1.743309
H	-0.585459	8.048332	-0.529610
H	-0.081061	7.905915	-2.216857
H	-0.437995	2.526785	3.586805
C	-1.390356	-0.735526	-0.179972
C	-1.833723	0.046775	-1.252992
C	-2.253789	-1.740743	0.276511
C	-3.086686	-0.152658	-1.835916
C	-3.492012	-1.975083	-0.329980
H	-1.964765	-2.339187	1.135213
C	-3.922456	-1.177901	-1.387938
H	-4.889381	-1.345677	-1.847753
C	-4.330352	-3.127694	0.147355
C	-3.585227	0.804511	-2.876088
F	-4.391694	-3.188143	1.493557
F	-5.599601	-3.070539	-0.315952
F	-3.816029	-4.317015	-0.268411
F	-2.581966	1.396529	-3.564521
F	-4.403708	0.219053	-3.775110
F	-4.308612	1.824577	-2.315239
O	1.052987	-1.508935	-0.030519
C	1.415451	-1.845891	-1.233531
C	0.766982	-3.006150	-1.879668
C	0.015219	-3.925090	-1.130301
C	0.855387	-3.157379	-3.273354
C	-0.650425	-4.967879	-1.768319
H	-0.051469	-3.798007	-0.055332
C	0.194317	-4.205409	-3.908064
H	1.427232	-2.438752	-3.856300
C	-0.559996	-5.111275	-3.156181
H	-1.254393	-5.657768	-1.186529
H	0.255916	-4.310863	-4.987559
H	-1.083896	-5.923418	-3.652777
H	1.758798	-1.045402	-1.890053
C	3.371348	-2.682615	-1.107108
H	3.080896	-3.633379	-0.679445
H	3.581383	-2.664399	-2.171035
C	4.018225	-1.761764	-0.292826
C	4.023471	-1.901300	1.170966
C	4.200319	-3.166097	1.763621
C	3.791965	-0.781329	1.991440

C	4.159825	-3.304329	3.147164
H	4.397541	-4.031673	1.138262
C	3.723941	-0.933333	3.374406
H	3.613365	0.185152	1.532598
C	3.912361	-2.190037	3.953576
H	4.312199	-4.281097	3.597229
H	3.494269	-0.077393	4.000957
H	3.855085	-2.302682	5.032404
O	4.479969	-0.656828	-0.852812
Si	5.914947	0.300140	-0.851409
C	7.328346	-0.918159	-1.111789
H	8.284393	-0.385603	-1.188005
H	7.412059	-1.623082	-0.276070
H	7.196642	-1.498970	-2.031764
C	6.154893	1.280993	0.729594
H	7.049923	1.908585	0.624597
H	5.298285	1.938176	0.907308
H	6.295568	0.644439	1.608779
H	-1.193584	0.832113	-1.634176
C	5.644533	1.429275	-2.319374
H	5.541758	0.862337	-3.251059
H	4.728979	2.012132	-2.173546
H	6.480422	2.129312	-2.438132

TS 281

B3LYP/6-31G(d) = -3573.915788
B3LYP-D3(BJ)/def2-TZVPP/IEFPCM(propanonitrile) = -3575.492202
B3LYP-D3(BJ)/def2-TZVPP/IEFPCM(propanonitrile)//B3LYP-D3(BJ)/6-31G(d) Free Energy (Quasiharmonic) = -3574.777077

Frequencies (Top 3 out of 300)

1. -250.9905 cm^{-1}
2. 12.4020 cm^{-1}
3. 12.8055 cm^{-1}

B3LYP/6-31G(d) Molecular Geometry in Cartesian Coordinates

B	0.035731	-0.509816	0.541757
O	-0.014841	-0.888930	1.962625
N	0.512383	0.999954	0.640091
C	0.541728	1.400529	2.060099
S	1.495465	1.779027	-0.418785
C	0.315429	0.084417	2.805618
O	0.403147	-0.039858	4.008691
H	-0.206586	3.433123	2.098694
H	1.532052	1.776571	2.341661
C	-0.517257	2.461006	2.494468
C	-1.932723	2.184264	2.074515
C	-2.912235	1.326578	2.707431
C	-4.086046	1.370771	1.903744
N	-3.839926	2.239884	0.858398
C	-2.541388	2.698733	0.956698
H	-2.147208	3.379998	0.215720
C	-5.205241	-0.155405	3.353405
C	-2.919328	0.527531	3.866002
H	-6.118051	0.679359	1.577941
H	-4.395408	2.304868	0.018286
C	-4.060259	-0.203063	4.174308
H	-6.078766	-0.745359	3.617666
H	-2.036761	0.459647	4.494521
C	-5.235371	0.635771	2.211094
H	-4.068887	-0.830712	5.061421
C	0.840752	3.435330	-0.698048
O	2.834721	1.997270	0.176438
O	1.389873	1.072445	-1.708638
C	-0.144963	6.023207	-1.156357
C	1.311969	4.503169	0.067607
C	-0.101019	3.648788	-1.707838
C	-0.587356	4.936663	-1.925565
C	0.811813	5.784401	-0.160774
H	2.072077	4.331944	0.822215
H	-0.440738	2.825940	-2.326720
H	-1.320103	5.100245	-2.712158
H	1.176813	6.613172	0.440950
C	-0.659465	7.417911	-1.420918
H	-0.081014	7.906026	-2.216507
H	-0.585319	8.048397	-0.529228
H	-1.705965	7.404436	-1.742889
O	1.052991	-1.508921	-0.030608
C	1.415424	-1.845835	-1.233642
H	1.758747	-1.045321	-1.890145
H	-0.437966	2.526641	3.586906
C	0.766944	-3.006076	-1.879800
C	0.855306	-3.157253	-3.273494
C	0.015216	-3.925052	-1.130442
C	0.194227	-4.205267	-3.908221
H	1.427125	-2.438599	-3.856433
C	-0.650437	-4.967825	-1.768477
H	-0.051437	-3.798010	-0.055466
C	-0.560053	-5.111169	-3.156347
H	0.255792	-4.310680	-4.987723
H	-1.254377	-5.657743	-1.186693
H	-1.083960	-5.923299	-3.652956
C	-1.390358	-0.735512	-0.180009
C	-1.833730	0.046838	-1.252993
C	-2.253791	-1.740745	0.276436
C	-3.086699	-0.152564	-1.835915
H	-1.193590	0.832190	-1.634146
C	-3.492021	-1.975054	-0.330056
H	-1.964764	-2.339228	1.135110
C	-3.922469	-1.177823	-1.387975
H	-4.889399	-1.345575	-1.847789
C	-4.330362	-3.127682	0.147235
C	-3.585245	0.804655	-2.876039
F	-5.599617	-3.070495	-0.316052
F	-3.816053	-4.316987	-0.268595
F	-4.308616	1.824700	-2.315135
F	-4.391687	-3.188194	1.493435
F	-4.403739	0.219243	-3.775078
F	-2.581987	1.396697	-3.564455
C	3.371331	-2.682554	-1.107306
H	3.080890	-3.633349	-0.679705
H	3.581354	-2.664264	-2.171234
C	4.018215	-1.761759	-0.292967
O	4.479962	-0.656787	-0.852880
Si	5.914942	0.300179	-0.851397
C	6.154888	1.280905	0.729685
H	7.049924	1.908498	0.624741
H	6.295554	0.644280	1.608820
H	5.298285	1.938081	0.907447
C	7.328338	-0.918104	-1.111870
H	8.284387	-0.385545	-1.188039
H	7.412045	-1.623095	-0.276207
H	7.196634	-1.498840	-2.031892
C	5.644536	1.429431	-2.319274
H	5.541751	0.862567	-3.251001
H	6.480434	2.129466	-2.437980
H	4.728991	2.012288	-2.173396
C	4.023466	-1.901395	1.170816
C	4.200331	-3.166229	1.763385
C	3.791944	-0.781483	1.991365
C	4.159839	-3.304556	3.146918
H	4.397563	-4.031761	1.137967
C	3.723921	-0.933581	3.374321
H	3.613331	0.185027	1.532588
C	3.912360	-2.190322	3.953406
H	4.312227	-4.281352	3.596917
H	3.494234	-0.077687	4.000929
H	3.855085	-2.303040	5.032226

TS 282

B3LYP/6-31G(d) = -3573.92181
B3LYP-D3(BJ)/def2-TZVPP/IEFPCM(propanonitrile) = -3575.492808
B3LYP-D3(BJ)/def2-TZVPP/IEFPCM(propanonitrile)//B3LYP-D3(BJ)/6-31G(d) Free Energy (Quasiharmonic) = -3574.777012

Frequencies (Top 3 out of 300)

1. -238.8448 cm^{-1}
2. 6.1836 cm^{-1}
3. 9.1359 cm^{-1}

B3LYP/6-31G(d) Molecular Geometry in Cartesian Coordinates

B	-0.332507	0.504386	0.744991
O	-0.404265	0.740725	2.201539
N	-0.251147	-1.059842	0.700362
C	-0.260762	-1.609548	2.067636
S	-0.692508	-2.042364	-0.547016
C	-0.491327	-0.372178	2.927539
O	-0.691149	-0.386449	4.125628
H	1.191584	-3.206269	1.889149
H	-1.108316	-2.290781	2.198323
C	1.022774	-2.349737	2.549489
C	2.280997	-1.522981	2.646958
C	3.502936	-1.696819	1.895176
C	4.408610	-0.684101	2.317833
N	3.776696	0.040306	3.306577
C	2.502882	-0.459870	3.493143
H	1.848454	-0.022639	4.233856
C	6.082365	-1.464949	0.812300
C	3.935283	-2.618040	0.925117
H	6.353177	0.254050	2.098597
H	4.107506	0.917031	3.679412
C	5.214485	-2.496005	0.397643
H	7.073356	-1.389228	0.372589
H	3.280085	-3.415368	0.588080
C	5.691749	-0.546066	1.778351
H	5.553928	-3.206077	-0.352071
C	0.731237	-2.966219	-1.149826
O	-1.636811	-3.057245	-0.044324
O	-1.086575	-1.157218	-1.660797
C	2.832059	-4.469595	-2.240956
C	1.671907	-2.344131	-1.974883
C	0.825610	-4.329054	-0.866665
C	1.877395	-5.068853	-1.409822
C	2.712177	-3.097213	-2.509178
H	1.593822	-1.290621	-2.212105
H	0.070546	-4.803008	-0.249248
H	1.948104	-6.131791	-1.191896
H	3.441593	-2.606110	-3.148335
C	3.950783	-5.277534	-2.854552
H	4.915020	-4.766931	-2.751299
H	4.037252	-6.263418	-2.387522
H	3.782672	-5.432479	-3.928259
H	0.762627	-2.763915	3.532141
C	0.910648	1.277746	0.060345
C	1.134374	1.293630	-1.320885
C	1.856134	1.931143	0.868773
C	2.272417	1.895049	-1.872149
C	2.988694	2.536294	0.325133
H	1.708246	1.946649	1.941380
C	3.210628	2.517170	-1.054482
H	4.096561	2.974690	-1.480223
C	4.003707	3.216991	1.200213
C	2.487926	1.798650	-3.355579
F	5.246372	2.707556	1.020811
F	3.719327	3.100156	2.522812
F	4.092274	4.541499	0.935523
F	1.385957	2.164492	-4.050170
F	2.768619	0.521622	-3.734465
F	3.510438	2.567781	-3.785788
O	-1.642727	1.176894	0.273315
C	-2.242801	1.401027	-0.851258
C	-2.243118	2.781191	-1.391345
C	-2.571511	2.992074	-2.740146
C	-1.871407	3.871606	-0.590076
C	-2.531800	4.275096	-3.279642
H	-2.841829	2.144275	-3.365841
C	-1.837281	5.153793	-1.132080
H	-1.597232	3.696133	0.445004
C	-2.168732	5.358610	-2.474782
H	-2.774418	4.430464	-4.327031
H	-1.543059	5.994966	-0.510694
H	-2.135479	6.359725	-2.895763
H	-2.275644	0.588615	-1.577338
C	-4.328086	1.346755	-0.392955
H	-4.709217	1.643913	-1.360640
H	-4.259413	2.134264	0.347008
C	-4.519709	0.038730	0.036423
C	-4.919416	-1.058601	-0.861846
C	-5.579382	-0.803233	-2.080534
C	-4.666729	-2.391771	-0.492135
C	-5.968574	-1.852571	-2.905514
H	-5.819171	0.214024	-2.372385
C	-5.047273	-3.437295	-1.328110
H	-4.132215	-2.595459	0.425918
C	-5.700550	-3.173023	-2.532907
H	-6.486471	-1.642136	-3.836898
H	-4.821975	-4.459817	-1.040822
H	-6.000587	-3.991505	-3.181589
O	-4.238039	-0.322153	1.279819
Si	-4.614987	0.393590	2.820687
C	-4.177244	-0.959236	4.035982
H	-3.093115	-1.074227	4.138793
H	-4.573914	-0.710509	5.028480
H	-4.608767	-1.920529	3.735748
C	-6.468314	0.728921	2.766018
H	-6.735181	1.430999	1.967654
H	-7.037232	-0.194133	2.607225
H	-6.802294	1.167755	3.714280
H	0.424682	0.811303	-1.987570
C	-3.639504	1.965584	3.134072
H	-2.570346	1.816845	2.953650
H	-3.983178	2.810776	2.526966
H	-3.762417	2.249865	4.187429

TS 283

B3LYP/6-31G(d) = -3573.918996
B3LYP-D3(BJ)/def2-TZVPP/IEFPCM(propanonitrile) = -3575.49391
B3LYP-D3(BJ)/def2-TZVPP/IEFPCM(propanonitrile)//B3LYP-D3(BJ)/6-31G(d) Free Energy (Quasiharmonic) = -3574.776942

Frequencies (Top 3 out of 300)

1. -279.8256 cm^{-1}
2. 5.4275 cm^{-1}
3. 12.5868 cm^{-1}

B3LYP/6-31G(d) Molecular Geometry in Cartesian Coordinates

B	0.323090	-0.639100	0.697410
O	0.776520	-1.055640	2.035880
N	1.044129	-1.683340	-0.202740
C	1.273289	-2.895860	0.624230
S	1.145349	-1.698210	-1.845940
C	1.191189	-2.323220	2.049310

O	1.453789	-2.946520	3.053870
H	2.334009	-4.440060	-0.426990
H	0.413149	-3.576590	0.528940
C	2.526719	-3.787480	0.425810
C	3.869219	-3.140880	0.230090
C	4.738359	-2.493880	1.191890
C	5.921139	-2.111831	0.494390
N	5.776119	-2.524601	-0.814390
C	4.548169	-3.135460	-0.964190
H	4.228769	-3.514930	-1.924440
C	6.867440	-1.186631	2.477530
C	4.655769	-2.216220	2.570490
H	7.879710	-1.175571	0.564720
H	6.436299	-2.358061	-1.557240
C	5.714809	-1.567601	3.193920
H	7.677180	-0.678731	2.994900
H	3.776509	-2.504750	3.136980
C	6.987519	-1.456481	1.119420
H	5.654329	-1.349221	4.256760
C	2.422930	-0.509730	-2.295390
O	-0.096400	-1.124780	-2.426310
O	1.531699	-3.045800	-2.292230
C	4.326440	1.378530	-3.113290
C	3.397280	-0.081160	-1.397020
C	2.393000	-0.015440	-3.604190
C	3.344080	0.917930	-4.003150
C	4.341130	0.858450	-1.814020
H	3.415120	-0.464380	-0.384300
H	1.619460	-0.341540	-4.291360
H	3.313410	1.308970	-5.017380
H	5.093580	1.197000	-1.106150
C	5.311590	2.440029	-3.540120
H	6.182070	2.471739	-2.877310
H	5.666430	2.273079	-4.563350
H	4.843200	3.432760	-3.517620
H	2.537999	-4.419450	1.323120
C	0.586360	0.940680	0.474530
C	0.386940	1.627840	-0.735040
C	0.997000	1.707170	1.578870
C	0.593880	3.007830	-0.837620
C	1.186890	3.088800	1.484090
H	1.169890	1.209670	2.527220
C	0.991000	3.751100	0.272310
H	1.142600	4.821250	0.195890
C	1.526310	3.877470	2.718210
C	0.437480	3.678620	-2.174620
F	0.403640	4.199080	3.424710
F	2.139990	5.045620	2.428110
F	2.324850	3.193620	3.560380
F	-0.590680	3.152310	-2.881850
F	1.545390	3.544430	-2.942140
F	0.203680	5.005960	-2.055030
O	-1.211560	-0.913820	0.804120
C	-2.021861	-1.380870	-0.112070
C	-3.050031	-2.354680	0.350010
C	-3.412181	-2.440809	1.702030
C	-3.616121	-3.245449	-0.573300
C	-4.340101	-3.394179	2.116470
H	-2.940161	-1.775530	2.417250
C	-4.541811	-4.202079	-0.155080
H	-3.304471	-3.207790	-1.614910
C	-4.909721	-4.273549	1.190960
H	-4.609771	-3.461839	3.166840
H	-4.959621	-4.901969	-0.873810
H	-5.623811	-5.023439	1.520520
H	-1.579161	-1.600140	-1.085750
C	-2.996640	0.165820	-0.905500
H	-2.150440	0.840450	-0.896020
H	-3.237270	-0.227560	-1.886950
C	-4.080560	0.446721	-0.059830
C	-3.970230	1.169721	1.208390
C	-5.073180	1.212261	2.088670
C	-2.779080	1.825440	1.580130
C	-4.982660	1.888341	3.298350
H	-5.987340	0.698701	1.816180
C	-2.694610	2.503070	2.793020
H	-1.917070	1.813760	0.927190
C	-3.794230	2.536211	3.652720
H	-5.836290	1.910001	3.969770
H	-1.770410	3.004050	3.062290
H	-3.726330	3.066361	4.598690
O	-5.272190	-0.081579	-0.295420
Si	-6.340220	-0.277599	-1.637480
C	-7.008570	1.439991	-2.004480
H	-7.746530	1.400381	-2.815210
H	-7.498550	1.876661	-1.127270
H	-6.211060	2.123261	-2.319080
C	-7.636711	-1.438449	-0.945800
H	-8.143540	-1.000579	-0.078760
H	-8.399601	-1.667379	-1.699550
H	-7.180641	-2.382309	-0.627350
H	0.075740	1.083460	-1.620700
C	-5.498570	-1.006779	-3.151990
H	-4.777830	-0.322669	-3.612560
H	-4.986091	-1.946769	-2.924210
H	-6.264230	-1.225359	-3.907910

TS 284

B3LYP/6-31G(d) = -3573.92066
B3LYP-D3(BJ)/def2-TZVPP/IEFPCM(propanonitrile) = -3575.492989
B3LYP-D3(BJ)/def2-TZVPP/IEFPCM(propanonitrile)//B3LYP-D3(BJ)/6-31G(d) Free Energy (Quasiharmonic) = -3574.776794

Frequencies (Top 3 out of 300)

1. -270.3487 cm^{-1}
2. 8.8129 cm^{-1}
3. 11.6557 cm^{-1}

B3LYP/6-31G(d) Molecular Geometry in Cartesian Coordinates

B	-0.422277	-0.400795	-0.445306
O	-0.629228	-0.849793	-1.847067
N	-0.126216	1.126377	-0.649198
C	-0.232468	1.468652	-2.076515
S	-0.537286	2.281133	0.470794
C	-0.656451	0.152349	-2.723285
O	-0.959782	0.017887	-3.893575
H	1.338635	2.941965	-2.280796
H	-1.032322	2.200938	-2.238610
C	1.054479	2.004825	-2.769369
C	2.232620	1.065200	-2.794286
C	3.495856	1.231498	-2.113752
C	4.291038	0.090042	-2.410551
N	3.556160	-0.708197	-3.262516
C	2.321637	-0.125470	-3.478543
H	1.596936	-0.603243	-4.122929
C	6.086045	0.920962	-1.081681
C	4.044210	2.237796	-1.299514
H	6.155887	-0.980076	-2.112720
H	3.791364	-1.658266	-3.505369
C	5.328055	2.075074	-0.795107
H	7.083638	0.817690	-0.662991
H	3.473567	3.130679	-1.063272
C	5.579103	-0.085834	-1.893976
H	5.756772	2.848430	-0.162976
C	0.903112	3.329336	0.737562
O	-0.777395	1.597775	1.750542
O	-1.577500	3.168975	-0.091792
C	3.069375	5.024599	1.278135

C	0.969220	4.582223	0.127338
C	1.903114	2.911662	1.619449
C	2.975114	3.760941	1.880525
C	2.054461	5.417211	0.395498
H	0.168104	4.902910	-0.529971
H	1.843437	1.944179	2.105603
H	3.752917	3.434626	2.566435
H	2.105523	6.394066	-0.079640
C	4.219913	5.948845	1.599858
H	4.374820	6.688091	0.807514
H	5.154285	5.393536	1.734940
H	4.033767	6.500042	2.531274
H	0.750305	2.265296	-3.791311
C	0.767363	-1.203488	0.290710
C	1.059340	-1.021923	1.647574
C	1.627696	-2.035119	-0.444837
C	2.179514	-1.617730	2.239099
H	0.422668	-0.378835	2.248417
C	2.739751	-2.638002	0.141903
H	1.432298	-2.195404	-1.498213
C	3.029262	-2.428523	1.493139
H	3.901836	-2.881525	1.950103
C	3.655220	-3.524542	-0.653849
C	2.468967	-1.316022	3.681806
F	3.642166	-4.801207	-0.201736
F	4.943473	-3.111035	-0.590337
F	3.324000	-3.575202	-1.970705
F	2.763769	-0.001992	3.864402
F	1.403320	-1.578886	4.474734
F	3.512550	-2.024702	4.164834
O	-1.769448	-0.525919	0.248980
C	-2.370922	-1.388186	1.010539
C	-2.048319	-2.837000	1.025609
C	-1.601271	-3.518610	-0.118437
C	-2.221924	-3.543131	2.226288
C	-1.324374	-4.881311	-0.050294
H	-1.468318	-2.977370	-1.049547
C	-1.937011	-4.905325	2.291444
H	-2.563352	-3.016075	3.114505
C	-1.489541	-5.576692	1.151519
H	-0.973686	-5.403616	-0.935891
H	-2.058696	-5.440257	3.229007
H	-1.267368	-6.639183	1.199071
H	-2.682586	-0.981750	1.977844
C	-4.331286	-1.508225	0.370942
H	-4.222669	-2.165762	-0.483195
H	-4.736727	-1.966672	1.265102
C	-4.579498	-0.153955	0.135646
C	-4.928055	0.787631	1.209086
C	-5.683705	0.372347	2.323036
C	-4.488032	2.122427	1.131455
C	-6.000421	1.276486	3.331675
H	-6.056227	-0.645973	2.381361
C	-4.791180	3.014041	2.156312
H	-3.865118	2.437136	0.302447
C	-5.550963	2.597922	3.251836
H	-6.599310	0.953098	4.178235
H	-4.419974	4.032856	2.101952
H	-5.790618	3.299897	4.045902
O	-4.320546	0.390435	-1.036767
Si	-4.794848	-0.007407	-2.665452
C	-6.673708	-0.115527	-2.593555
H	-7.011200	-0.911792	-1.919642
H	-7.114846	0.826774	-2.249734
H	-7.081637	-0.332426	-3.588380
C	-4.037760	-1.615181	-3.268009
H	-2.944040	-1.573332	-3.253674
H	-4.374801	-2.495603	-2.710090
H	-4.339957	-1.761314	-4.313888
C	-4.194078	1.456558	-3.660891
H	-4.683516	1.467689	-4.642920
H	-4.422187	2.403680	-3.160475
H	-3.114671	1.396525	-3.833824

TS 285

B3LYP/6-31G(d) = -3573.913045
B3LYP-D3(BJ)/def2-TZVPP/IEFPCM(propanonitrile) = -3575.493109
B3LYP-D3(BJ)/def2-TZVPP/IEFPCM(propanonitrile)//B3LYP-D3(BJ)/6-31G(d) Free Energy (Quasiharmonic) = -3574.776773

Frequencies (Top 3 out of 300)

1. -273.0628 cm^{-1}
2. 9.2056 cm^{-1}
3. 12.7165 cm^{-1}

B3LYP/6-31G(d) Molecular Geometry in Cartesian Coordinates

B	-0.447587	0.264781	0.592339
O	-0.320938	0.411472	2.060119
N	-0.402885	1.773433	0.163794
C	-0.663786	2.632499	1.334500
S	-0.272485	2.432696	-1.327837
C	-0.394125	1.670494	2.493833
O	-0.277517	2.000412	3.654772
H	-2.227659	3.928622	0.600680
H	0.074295	3.442545	1.388452
C	-2.072151	3.283234	1.469239
C	-3.233779	2.339113	1.628991
C	-4.246024	2.006920	0.651595
C	-5.131024	1.078324	1.265804
N	-4.690573	0.882628	2.559414
C	-3.545035	1.625847	2.763576
H	-3.033491	1.597666	3.716088
C	-6.433909	0.914409	-0.722978
C	-4.490049	2.390458	-0.679984
H	-6.871987	-0.202457	1.080511
H	-5.006016	0.134993	3.158320
C	-5.576031	1.841309	-1.350677
H	-7.268344	0.495726	-1.279335
H	-3.819318	3.076248	-1.186613
C	-6.223892	0.521409	0.593545
H	-5.762034	2.120231	-2.384276
C	1.083120	3.620881	-1.203279
O	-1.442839	3.232131	-1.720471
O	0.151995	1.338768	-2.228602
C	3.211689	5.453587	-1.178395
C	2.186356	3.394673	-0.380112
C	1.026417	4.763058	-2.006532
C	2.087896	5.664402	-1.991166
C	3.238716	4.310279	-0.369911
H	2.214931	2.527605	0.270900
H	0.151281	4.941995	-2.621527
H	2.038532	6.553381	-2.615642
H	4.088348	4.133937	0.285190
C	4.362043	6.432000	-1.184983
H	5.007407	6.298749	-0.311151
H	4.985643	6.301692	-2.079238
H	4.006075	7.468127	-1.189301
H	-1.997000	3.939366	2.345809
C	-1.739121	-0.615052	0.180255
C	-2.286721	-0.646949	-1.111292
C	-2.351323	-1.415958	1.156841
C	-3.398528	-1.439796	-1.410971
C	-3.469268	-2.200374	0.863460
H	-1.957727	-1.409047	2.167770
C	-3.999532	-2.220736	-0.426234
H	-4.871739	-2.821055	-0.656235
C	-4.162884	-2.955244	1.959064
C	-3.929635	-1.479500	-2.817485

F	-4.792775	-4.061169	1.505407
F	-3.314765	-3.343688	2.936363
F	-5.125532	-2.199086	2.571395
F	-3.271023	-2.399711	-3.571870
F	-5.239443	-1.816209	-2.858632
F	-3.794193	-0.296137	-3.449322
O	0.806392	-0.576437	0.267089
C	1.227728	-1.085600	-0.856940
C	1.490188	-2.546076	-0.884986
C	1.485001	-3.299288	0.299392
C	1.626626	-3.201023	-2.118350
C	1.616863	-4.685870	0.246039
H	1.336029	-2.790712	1.246227
C	1.751615	-4.587758	-2.169342
H	1.608011	-2.623857	-3.039899
C	1.748887	-5.333030	-0.986522
H	1.592250	-5.265752	1.164581
H	1.836580	-5.088183	-3.129746
H	1.833965	-6.415548	-1.026231
H	0.819014	-0.653793	-1.771046
C	3.012128	-0.196730	-1.315722
H	2.620925	0.808512	-1.224032
H	3.082419	-0.578214	-2.329968
C	4.072720	-0.562140	-0.479825
C	4.378256	0.120326	0.797205
C	3.351141	0.476136	1.687874
C	5.708423	0.453251	1.113438
C	3.652558	1.124675	2.885459
H	2.324360	0.210885	1.463525
C	6.000144	1.126255	2.297586
H	6.503564	0.225344	0.411706
C	4.974897	1.455815	3.189650
H	2.845808	1.376036	3.568687
H	7.027534	1.398696	2.522634
H	5.206718	1.976191	4.114737
O	4.849749	-1.552529	-0.882640
Si	5.859372	-2.800597	-0.214750
C	7.649639	-2.227643	-0.313156
H	8.309840	-3.101141	-0.239657
H	7.861405	-1.741339	-1.272509
H	7.927600	-1.539053	0.490526
C	5.587098	-4.224727	-1.399619
H	6.203118	-5.085538	-1.111136
H	4.540768	-4.545052	-1.411298
H	5.866364	-3.943585	-2.421464
H	-1.859723	-0.032610	-1.897789
C	5.323016	-3.209544	1.535887
H	5.480040	-2.378892	2.230330
H	4.264890	-3.490272	1.567958
H	5.904766	-4.066001	1.900351

TS 286

B3LYP/6-31G(d) = -3573.922885
B3LYP-D3(BJ)/def2-TZVPP/IEFPCM(propanonitrile) = -3575.494168
B3LYP-D3(BJ)/def2-TZVPP/IEFPCM(propanonitrile)//B3LYP-D3(BJ)/6-31G(d) Free Energy (Quasiharmonic) = -3574.776762

Frequencies (Top 3 out of 300)

1. -274.8938 cm^{-1}
2. -1.8175 cm^{-1}
3. 8.9888 cm^{-1}

B3LYP/6-31G(d) Molecular Geometry in Cartesian Coordinates

B	-0.196486	0.098501	0.408238
O	-0.492580	-0.146553	1.840919
N	0.402298	-1.310218	-0.010509
C	0.381277	-2.221485	1.150445
S	0.354663	-1.980233	-1.514132
C	-0.339282	-1.404669	2.217861
O	-0.685249	-1.843102	3.301048
H	2.264502	-3.274517	0.933290
H	-0.216589	-3.112968	0.929934
C	1.759619	-2.689400	1.707851
C	2.677882	-1.600111	2.200635
C	3.976924	-1.253472	1.672381
C	4.455937	-0.143063	2.421440
N	3.512643	0.134987	3.387402
C	2.446879	-0.731013	3.242522
H	1.600194	-0.667955	3.911314
C	6.461524	-0.063523	1.136103
C	4.795711	-1.780589	0.658758
H	6.014352	1.334385	2.727727
H	3.495077	0.980241	3.937447
C	6.024100	-1.184836	0.401298
H	7.422731	0.387381	0.903904
H	4.474441	-2.641926	0.081036
C	5.684630	0.470397	2.157080
H	6.660844	-1.586200	-0.383145
C	1.978618	-2.621573	-1.940725
O	0.101422	-0.878362	-2.465639
O	-0.560063	-3.142069	-1.521642
C	4.443278	-3.649556	-2.787418
C	2.212764	-3.995522	-1.873396
C	2.955512	-1.751636	-2.432557
C	4.175090	-2.273893	-2.853904
C	3.445320	-4.498280	-2.290506
H	1.428091	-4.659629	-1.527416
H	2.767353	-0.686186	-2.501326
H	4.932408	-1.597670	-3.242161
H	3.626439	-5.569374	-2.244289
C	5.780910	-4.195417	-3.227369
H	5.726672	-5.267626	-3.440587
H	6.143814	-3.686661	-4.127030
H	6.540033	-4.053314	-2.446979
H	1.518412	-3.389357	2.518566
C	0.819742	1.341417	0.219288
C	1.372381	1.693600	-1.019586
C	1.218705	2.098459	1.331228
C	2.306798	2.726744	-1.138173
C	2.149830	3.134731	1.218101
H	0.801698	1.863224	2.304516
C	2.707502	3.455137	-0.020279
H	3.435458	4.251930	-0.111460
C	2.599506	3.861982	2.452424
C	2.870392	3.026882	-2.498088
F	3.102385	5.084425	2.179504
F	3.587008	3.185671	3.116639
F	1.597287	4.023507	3.344535
F	3.392839	1.913542	-3.077080
F	3.852831	3.952963	-2.463009
F	1.921056	3.484124	-3.348778
O	-1.577532	0.478603	-0.105930
C	-2.144341	0.576799	-1.280761
C	-3.000197	1.776970	-1.502488
C	-3.339263	2.627589	-0.441568
C	-3.418152	2.099470	-2.802232
C	-4.090708	3.777523	-0.679116
H	-2.983910	2.389420	0.555475
C	-4.170265	3.248142	-3.037285
H	-3.136510	1.456315	-3.633469
C	-4.510948	4.088746	-1.974205
H	-4.340473	4.438122	0.146811
H	-4.480309	3.494459	-4.049109
H	-5.091423	4.988958	-2.157356
H	-1.549397	0.266677	-2.140328
C	-3.319406	-0.998172	-1.525725
H	-3.747359	-0.756966	-2.492614
H	-2.542390	-1.758078	-1.527546

C	-4.184850	-0.988992	-0.424426
C	-5.493871	-0.311771	-0.449376
C	-6.291612	-0.328079	-1.607667
C	-5.952935	0.383213	0.684916
C	-7.523789	0.319664	-1.623629
H	-5.961332	-0.878787	-2.482577
C	-7.177276	1.044030	0.658971
H	-5.327056	0.431132	1.569304
C	-7.967807	1.008794	-0.492728
H	-8.138981	0.285279	-2.517981
H	-7.513342	1.591769	1.534623
H	-8.927034	1.518577	-0.509135
O	-3.730207	-1.461307	0.714496
Si	-4.292258	-2.472066	2.019846
C	-3.146260	-3.953565	1.999275
H	-3.560225	-4.762831	2.614048
H	-3.000787	-4.341664	0.984920
H	-2.170799	-3.681893	2.415163
C	-6.067289	-2.992729	1.664244
H	-6.162646	-3.466883	0.680308
H	-6.369729	-3.735846	2.413252
H	-6.779454	-2.163799	1.710796
H	1.087121	1.139477	-1.908732
C	-4.109154	-1.472249	3.591300
H	-3.064508	-1.168520	3.721818
H	-4.747331	-0.582234	3.614957
H	-4.380747	-2.095642	4.453092

TS 287

B3LYP/6-31G(d) = -3573.913052
B3LYP-D3(BJ)/def2-TZVPP/IEFPCM(propanonitrile) = -3575.493132
B3LYP-D3(BJ)/def2-TZVPP/IEFPCM(propanonitrile)//B3LYP-D3(BJ)/6-31G(d) Free Energy (Quasiharmonic) = -3574.77674

Frequencies (Top 3 out of 300)

1. -272.9234 cm^{-1}
2. 9.0198 cm^{-1}
3. 12.7458 cm^{-1}

B3LYP/6-31G(d) Molecular Geometry in Cartesian Coordinates

B	-0.447344	0.265744	0.592156
O	-0.320580	0.413932	2.059762
N	-0.403264	1.773936	0.162063
C	-0.664252	2.634121	1.331900
S	-0.272452	2.431622	-1.330222
C	-0.394434	1.673351	2.492235
O	-0.278254	2.004474	3.652865
H	-2.228214	3.929307	0.596684
H	0.073730	3.444309	1.385066
C	-2.072698	3.284789	1.465894
C	-3.234177	2.340625	1.626431
C	-4.246144	2.007179	0.649177
C	-5.131098	1.079079	1.264203
N	-4.690885	0.884865	2.558121
C	-3.545522	1.628513	2.761714
H	-3.034151	1.601442	3.714352
C	-6.433512	0.912766	-0.724688
C	-4.489930	2.389218	-0.682875
H	-6.871790	-0.202217	1.079918
H	-5.006266	0.137772	3.157737
C	-5.575663	1.839136	-1.353208
H	-7.267744	0.493322	-1.280775
H	-3.819200	3.074568	-1.190106
C	-6.223716	0.521236	0.592310
H	-5.761483	2.116888	-2.387156
C	1.082723	3.620240	-1.205748
O	0.151986	1.336653	-2.229735
O	-1.442590	3.230842	-1.723958
C	3.213053	5.450780	-1.184293
C	1.029188	4.758497	-2.014527
C	2.185795	3.394505	-0.381983
C	3.239874	4.307890	-0.374952
C	2.092744	5.657662	-2.002560
H	0.157695	4.933361	-2.635853
H	2.215405	2.526216	0.267355
H	4.092072	4.128981	0.276189
H	2.048994	6.540269	-2.636401
C	4.342644	6.452694	-1.155470
H	4.565363	6.833580	-2.158180
H	5.258394	6.013760	-0.747012
H	4.085973	7.318314	-0.530764
O	0.806925	-0.575312	0.267472
C	1.227215	-1.086597	-0.855976
H	0.818012	-0.656222	-1.770534
H	-1.997756	3.941771	2.341847
C	1.489432	-2.547124	-0.881728
C	1.484896	-3.298341	0.303927
C	1.624907	-3.204174	-2.114084
C	1.616453	-4.685034	0.252819
H	1.336657	-2.788150	1.250007
C	1.749583	-4.591016	-2.162821
H	1.605796	-2.628554	-3.036588
C	1.747513	-5.334291	-0.978742
H	1.592344	-5.263365	1.172351
H	1.833796	-5.093077	-3.122436
H	1.832348	-6.416892	-1.016685
C	-1.738674	-0.614891	0.181102
C	-2.350715	-1.414806	1.158601
C	-2.286254	-0.648393	-1.110415
C	-3.468474	-2.199819	0.866100
H	-1.957138	-1.406638	2.169527
C	-3.397850	-1.441868	-1.409212
H	-1.859408	-0.034819	-1.897594
C	-3.998690	-2.221814	-0.423586
H	-4.870739	-2.822619	-0.652918
C	-3.928889	-1.483349	-2.815701
C	-4.161955	-2.953577	1.962553
F	-3.269959	-2.404229	-3.568993
F	-5.238592	-1.820509	-2.856516
F	-5.124897	-2.196973	2.573872
F	-3.793780	-0.300676	-3.448897
F	-3.313813	-3.340578	2.940401
F	-4.791467	-4.060243	1.510175
C	3.011674	-0.198668	-1.317726
H	3.080817	-0.581499	-2.331542
H	2.620700	0.806739	-1.226904
C	4.072808	-0.563412	-0.482308
C	4.379694	0.120561	0.793633
C	5.710184	0.454013	1.107923
C	3.353583	0.477193	1.685119
C	6.003224	1.128356	2.290992
H	6.504519	0.225385	0.405509
C	3.656351	1.127103	2.881623
H	2.326546	0.211648	1.462238
C	4.978993	1.458762	3.183905
H	7.030848	1.401154	2.514543
H	2.850392	1.379116	3.565540
H	5.211835	1.980195	4.108138
O	4.849138	-1.554616	-0.884480
Si	5.859266	-2.801822	-0.215763
C	5.585885	-4.227544	-1.398459
H	5.864232	-3.947788	-2.420935
H	4.539535	-4.547852	-1.408757
H	6.202143	-5.087982	-1.109372
C	7.649490	-2.229136	-0.316420
H	7.928150	-1.539508	0.486129
H	8.309691	-3.102583	-0.242315
H	7.860492	-1.744110	-1.276588

C	5.324351	-3.208372	1.535882
H	5.906328	-4.064390	1.901012
H	5.482034	-2.376801	2.229079
H	4.266225	-3.488956	1.569216

TS 288

B3LYP/6-31G(d) = -3573.922886
B3LYP-D3(BJ)/def2-TZVPP/IEFPCM(propanonitrile) = -3575.494015
B3LYP-D3(BJ)/def2-TZVPP/IEFPCM(propanonitrile)//B3LYP-D3(BJ)/6-31G(d) Free Energy (Quasiharmonic) = -3574.776676

Frequencies (Top 3 out of 300)

1. -274.9246 cm^{-1}
2. -2.5759 cm^{-1}
3. 7.8643 cm^{-1}

B3LYP/6-31G(d) Molecular Geometry in Cartesian Coordinates

B	-0.198867	0.101527	0.406767
O	-0.494837	-0.146872	1.838935
N	0.402695	-1.305167	-0.014340
C	0.380995	-2.219620	1.144088
S	0.360529	-1.970999	-1.519953
C	-0.340129	-1.405617	2.213232
O	-0.685414	-1.846500	3.295631
H	2.262518	-3.274614	0.924776
H	-0.217188	-3.110237	0.920807
C	1.758679	-2.690149	1.700516
C	2.679500	-1.603560	2.194675
C	3.985565	-1.269666	1.675425
C	4.467080	-0.159300	2.422973
N	3.518153	0.130922	3.379801
C	2.446902	-0.727562	3.230381
H	1.596163	-0.655185	3.892999
C	6.484603	-0.103053	1.155177
C	4.808845	-1.808792	0.671767
H	6.035338	1.306321	2.736093
H	3.501376	0.980070	3.923915
C	6.044427	-1.224580	0.422366
H	7.451757	0.338413	0.929585
H	4.486303	-2.670678	0.095532
C	5.703093	0.442628	2.166387
H	6.685117	-1.636419	-0.353329
C	1.986910	-2.605474	-1.947466
O	0.105114	-0.867223	-2.468707
O	-0.550100	-3.135989	-1.532286
C	4.455851	-3.621576	-2.795558
C	2.964295	-1.729327	-2.427062
C	2.224550	-3.979168	-1.888034
C	3.460040	-4.475844	-2.303736
C	4.186948	-2.245417	-2.847015
H	2.776483	-0.663025	-2.482481
H	1.442509	-4.647036	-1.543345
H	3.646189	-5.546120	-2.258039
H	4.947179	-1.563603	-3.219386
C	5.775636	-4.167382	-3.286480
H	6.607370	-3.508757	-3.014100
H	5.978220	-5.160409	-2.873192
H	5.780666	-4.258848	-4.380716
H	1.516605	-3.391431	2.509889
C	0.814916	1.347021	0.221077
C	1.212758	2.101307	1.335339
C	1.366619	1.704298	-1.016731
C	2.142007	3.139563	1.225439
H	0.796101	1.862462	2.307889
C	2.299393	2.739363	-1.132064
H	1.081927	1.152877	-1.907761
C	2.699114	3.464818	-0.011961
H	3.425595	4.263226	-0.100724
C	2.864365	3.042303	-2.490765
C	2.590570	3.863550	2.462074
F	1.912042	3.481761	-3.347328
F	3.405604	1.934203	-3.062713
F	3.833126	3.982639	-2.455481
F	3.579695	3.186782	3.123480
F	3.091057	5.087837	2.193166
F	1.588408	4.020061	3.355072
O	-1.580355	0.480116	-0.107166
C	-2.148618	0.574379	-1.281708
C	-3.007943	1.771959	-1.504559
C	-3.427949	2.091342	-2.804406
C	-3.348232	2.623383	-0.444687
C	-4.183288	3.237676	-3.040562
H	-3.145370	1.447659	-3.634917
C	-4.102906	3.770979	-0.683325
H	-2.991289	2.387755	0.552390
C	-4.525183	4.079067	-1.978497
H	-4.494905	3.481589	-4.052487
H	-4.353606	4.432222	0.141804
H	-5.108176	4.977473	-2.162518
H	-1.553136	0.265173	-2.141243
C	-3.318705	-1.003818	-1.523605
H	-2.539864	-1.761898	-1.525094
H	-3.747755	-0.764948	-2.490599
C	-4.184255	-0.996319	-0.422249
C	-5.494627	-0.321709	-0.447607
C	-5.954435	0.374228	0.685794
C	-6.292811	-0.341265	-1.605532
C	-7.179986	1.032780	0.659350
H	-5.328246	0.424740	1.569824
C	-7.526212	0.304140	-1.621938
H	-5.961849	-0.892665	-2.479752
C	-7.970980	0.994238	-0.491924
H	-7.516636	1.581328	1.534268
H	-8.141740	0.267222	-2.515955
H	-8.931146	1.502236	-0.508702
O	-3.728811	-1.467715	0.716685
Si	-4.290878	-2.476345	2.023982
C	-6.066338	-2.997064	1.670545
H	-6.162942	-3.471336	0.686791
H	-6.367564	-3.740217	2.420018
H	-6.778644	-2.168328	1.717997
C	-4.106071	-1.474325	3.593791
H	-3.061111	-1.171144	3.723007
H	-4.743607	-0.583826	3.616495
H	-4.377527	-2.096212	4.456708
C	-3.145889	-3.958580	2.004407
H	-3.557598	-4.764673	2.624857
H	-3.005815	-4.351591	0.991180
H	-2.168256	-3.685898	2.414462

TS 289

B3LYP/6-31G(d) = -3573.925705
B3LYP-D3(BJ)/def2-TZVPP/IEFPCM(propanonitrile) = -3575.493073
B3LYP-D3(BJ)/def2-TZVPP/IEFPCM(propanonitrile)//B3LYP-D3(BJ)/6-31G(d) Free Energy (Quasiharmonic) = -3574.776651

Frequencies (Top 3 out of 300)

1. -253.1442 cm^{-1}
2. 9.3854 cm^{-1}
3. 11.9498 cm^{-1}

B3LYP/6-31G(d) Molecular Geometry in Cartesian Coordinates

B	0.136584	0.111681	1.036832
O	0.736550	-0.106633	2.360387

N	1.392210	0.011336	0.111395
C	2.616265	-0.145743	0.928049
S	1.498054	-0.212667	-1.520161
C	2.063462	-0.262338	2.351669
O	2.726024	-0.432359	3.349789
H	3.305166	1.833599	1.516315
H	3.132200	-1.081805	0.681233
C	3.641060	1.029973	0.850418
C	5.047563	0.613618	1.180968
C	6.076689	0.262964	0.229409
C	7.237807	-0.090696	0.972966
N	6.917302	0.042342	2.308434
C	5.603690	0.455728	2.426348
H	5.145405	0.575613	3.396930
C	8.453484	-0.524506	-1.032634
C	6.131622	0.213413	-1.176236
H	9.307957	-0.748071	0.942644
H	7.529823	-0.166224	3.081018
C	7.315388	-0.180326	-1.790458
H	9.364656	-0.827057	-1.542065
H	5.259454	0.474675	-1.769491
C	8.430637	-0.483058	0.357371
H	7.367563	-0.222512	-2.875336
C	1.706995	-1.969048	-1.841865
O	0.197223	0.203243	-2.085210
O	2.720227	0.435227	-2.018236
C	2.064712	-4.713251	-2.294599
C	2.989995	-2.522644	-1.767362
C	0.605980	-2.765318	-2.161967
C	0.794685	-4.128142	-2.384733
C	3.155282	-3.888605	-1.985042
H	3.850309	-1.892792	-1.566516
H	-0.379910	-2.326953	-2.254280
H	-0.063142	-4.747062	-2.636504
H	4.152245	-4.317787	-1.921473
C	2.261186	-6.186677	-2.560934
H	1.346678	-6.755084	-2.363631
H	3.061649	-6.602299	-1.939846
H	2.537625	-6.364270	-3.608807
O	-0.304662	1.614910	1.122913
C	-0.241711	2.482594	0.160789
H	-0.190671	2.102077	-0.860579
H	3.608524	1.425377	-0.165612
C	0.484585	3.748993	0.427549
C	0.890822	4.558208	-0.644608
C	0.820412	4.118502	1.739338
C	1.613486	5.725333	-0.408947
H	0.659053	4.257703	-1.663831
C	1.538170	5.289432	1.971273
H	0.534276	3.467976	2.559645
C	1.932630	6.096364	0.899926
H	1.936515	6.339772	-1.244484
H	1.801421	5.568474	2.987713
H	2.498121	7.005685	1.083759
C	-1.134324	-0.886251	0.868809
C	-1.890074	-1.073477	-0.299738
C	-1.521332	-1.640545	1.991501
C	-2.933392	-2.003035	-0.359321
H	-1.640834	-0.512109	-1.190281
C	-2.586134	-2.544504	1.946426
H	-0.969454	-1.524913	2.917161
C	-3.292628	-2.746926	0.763171
H	-4.095757	-3.473468	0.715185
C	-3.017899	-3.258119	3.197468
C	-3.713299	-2.174638	-1.629604
F	-3.611794	-4.444026	2.927056
F	-1.987548	-3.501528	4.032639
F	-4.802505	-1.358162	-1.685605
F	-3.924176	-2.526125	3.899512
F	-4.177976	-3.433557	-1.782420
F	-2.970062	-1.885620	-2.730157
C	-2.144814	3.387911	-0.113701
H	-1.905254	3.925893	-1.023061
H	-2.103227	3.984289	0.787284
C	-3.109660	2.382189	-0.179923
C	-3.701364	1.766537	1.012675
C	-3.229158	2.069526	2.306377
C	-4.762286	0.848999	0.875191
C	-3.801714	1.471001	3.423542
H	-2.394970	2.746044	2.443074
C	-5.334744	0.261467	1.997014
H	-5.109736	0.585744	-0.115490
C	-4.854996	0.566325	3.273819
H	-3.416172	1.700828	4.412287
H	-6.147367	-0.448850	1.876645
H	-5.291757	0.090365	4.146607
O	-3.506686	1.862971	-1.334208
Si	-3.529194	2.449378	-2.969756
C	-1.783457	2.628847	-3.638799
H	-1.153318	1.770715	-3.379202
H	-1.823900	2.690821	-4.733988
H	-1.284370	3.538139	-3.284573
C	-4.482934	1.121586	-3.879467
H	-3.967812	0.157930	-3.835386
H	-5.483204	0.982625	-3.455435
H	-4.599298	1.397665	-4.934761
C	-4.457428	4.087493	-2.945852
H	-4.539257	4.486677	-3.964496
H	-5.474440	3.960692	-2.557708
H	-3.956820	4.846468	-2.334830

TS 290

B3LYP/6-31G(d) = -3573.914455
B3LYP-D3(BJ)/def2-TZVPP/IEFPCM(propanonitrile) = -3575.492528
B3LYP-D3(BJ)/def2-TZVPP/IEFPCM(propanonitrile)//B3LYP-D3(BJ)/6-31G(d) Free Energy (Quasiharmonic) = -3574.77664

Frequencies (Top 3 out of 300)

1. -274.2146 cm^{-1}
2. 9.3878 cm^{-1}
3. 10.8617 cm^{-1}

B3LYP/6-31G(d) Molecular Geometry in Cartesian Coordinates

B	-0.498079	0.268815	0.338499
O	-0.505195	0.379107	1.813030
N	-0.333713	1.780873	-0.049371
C	-0.634239	2.631620	1.119087
S	-0.130386	2.443001	-1.534121
C	-0.521298	1.631207	2.269982
O	-0.445027	1.935447	3.441312
H	-1.930885	4.184996	0.379852
H	0.154053	3.381051	1.261899
C	-1.991869	3.397555	1.136944
C	-3.232743	2.583772	0.910059
C	-3.992277	1.816359	1.872506
C	-5.073828	1.217292	1.166276
N	-4.985693	1.630616	-0.148800
C	-3.869228	2.426542	-0.297052
H	-3.592925	2.821125	-1.263967
C	-5.849689	0.147890	3.151546
C	-3.870344	1.570597	3.252523
H	-6.819021	-0.072555	1.229655
H	-5.490948	1.225918	-0.923011
C	-4.795438	0.741082	3.875076
H	-6.552483	-0.502602	3.665316
H	-3.049347	2.000402	3.818984
C	-6.005664	0.381848	1.789877
H	-4.701281	0.538398	4.938675

C	1.180407	3.665544	-1.321611
O	-1.294575	3.205778	-2.015699
O	0.372207	1.355708	-2.402088
C	3.253194	5.553393	-1.161488
C	2.265773	3.436413	-0.474356
C	1.114485	4.835380	-2.082051
C	2.148739	5.765139	-1.998943
C	3.289438	4.379626	-0.396827
H	2.300020	2.545887	0.144140
H	0.253709	5.012271	-2.717405
H	2.092204	6.676316	-2.589731
H	4.124279	4.201992	0.276693
C	4.377916	6.558936	-1.096639
H	4.939932	6.473028	-0.161253
H	5.087567	6.409342	-1.921196
H	4.003025	7.584924	-1.175445
H	-2.027555	3.897430	2.113666
C	-1.779247	-0.558902	-0.199720
C	-2.353377	-0.410309	-1.470171
C	-2.363399	-1.502435	0.661616
C	-3.472699	-1.157785	-1.851626
C	-3.466129	-2.267401	0.275318
H	-1.967046	-1.618913	1.664950
C	-4.033035	-2.096782	-0.986219
H	-4.900932	-2.673654	-1.283911
C	-4.022112	-3.306344	1.209698
C	-4.154131	-0.863877	-3.155781
F	-3.917687	-2.938677	2.502685
F	-5.327687	-3.565171	0.963383
F	-3.364467	-4.489340	1.087461
F	-5.081245	0.134341	-3.017015
F	-3.296450	-0.452948	-4.110653
F	-4.824876	-1.931993	-3.641763
O	0.743288	-0.626582	0.114496
C	1.256751	-1.115643	-0.979661
C	1.499563	-2.579894	-1.017037
C	1.368684	-3.358604	0.143324
C	1.744572	-3.210461	-2.246376
C	1.483036	-4.745966	0.070613
H	1.137753	-2.868298	1.083292
C	1.853313	-4.597640	-2.317281
H	1.824905	-2.613178	-3.151737
C	1.724059	-5.368403	-1.158007
H	1.359641	-5.344923	0.968629
H	2.024687	-5.078552	-3.276183
H	1.795856	-6.451117	-1.214479
H	0.933535	-0.659060	-1.915984
C	3.084017	-0.248891	-1.265357
H	2.697067	0.760605	-1.212281
H	3.245840	-0.631714	-2.268522
C	4.059531	-0.623777	-0.334382
C	4.253988	0.058997	0.963063
C	3.151864	0.445425	1.744852
C	5.553774	0.361257	1.410347
C	3.346537	1.092404	2.965225
H	2.147668	0.199841	1.420226
C	5.741084	1.033699	2.615539
H	6.409584	0.110259	0.793017
C	4.639710	1.392668	3.399142
H	2.481308	1.364994	3.563572
H	6.747000	1.282507	2.942136
H	4.790416	1.911778	4.341551
O	4.859521	-1.623355	-0.663050
Si	5.768450	-2.895479	0.097946
C	5.595389	-4.306891	-1.120457
H	6.158359	-5.182520	-0.773986
H	4.549991	-4.604428	-1.247574
H	5.989484	-4.026224	-2.103910
C	5.040653	-3.300613	1.778719
H	5.566754	-4.166531	2.200800
H	5.134556	-2.473785	2.488995
H	3.981051	-3.564917	1.696032
H	-1.941592	0.302429	-2.176763
C	7.570583	-2.360457	0.194371
H	7.892430	-1.870184	-0.731793
H	7.776409	-1.685517	1.030665
H	8.201089	-3.248807	0.326931

TS 291

B3LYP/6-31G(d) = -3573.917657
B3LYP-D3(BJ)/def2-TZVPP/IEFPCM(propanonitrile) = -3575.492809
B3LYP-D3(BJ)/def2-TZVPP/IEFPCM(propanonitrile)//B3LYP-D3(BJ)/6-31G(d) Free Energy (Quasiharmonic) = -3574.776626

Frequencies (Top 3 out of 300)

1. -269.4275 cm^{-1}
2. 10.4532 cm^{-1}
3. 12.0709 cm^{-1}

B3LYP/6-31G(d) Molecular Geometry in Cartesian Coordinates

B	0.124210	-0.409824	-0.647024
O	0.186223	-1.041402	-1.971123
N	0.448062	1.100210	-0.985672
C	0.859352	1.187122	-2.400697
S	-0.346253	2.374737	-0.312680
C	0.587794	-0.220537	-2.945225
O	0.723595	-0.541554	-4.103896
H	2.420701	2.680947	-2.432410
H	0.211901	1.875341	-2.958719
C	2.336845	1.612153	-2.654505
C	3.378964	0.857459	-1.879715
C	3.979470	-0.423850	-2.184936
C	4.876809	-0.726982	-1.122632
N	4.847986	0.335497	-0.239839
C	3.928956	1.262685	-0.689183
H	3.743240	2.165067	-0.123626
C	5.453859	-2.809760	-2.128741
C	3.850917	-1.344755	-3.241463
H	6.286576	-2.129699	-0.251175
H	5.201990	0.310761	0.705026
C	4.585179	-2.523627	-3.201381
H	6.006399	-3.745428	-2.121794
H	3.165947	-1.151081	-4.060957
C	5.615627	-1.913522	-1.078686
H	4.482010	-3.244709	-4.007812
C	0.827770	3.732492	-0.197282
O	-0.685167	1.988931	1.072400
O	-1.430784	2.853160	-1.199742
C	2.626640	5.873313	0.006155
C	1.700363	3.800060	0.892557
C	0.825731	4.731556	-1.170682
C	1.727229	5.790737	-1.064478
C	2.590343	4.867545	0.984627
H	1.671948	3.040329	1.666551
H	0.112675	4.686993	-1.986857
H	1.723908	6.570254	-1.822314
H	3.263584	4.925884	1.836567
C	3.619435	7.006749	0.102429
H	3.776725	7.314651	1.141634
H	3.286797	7.881007	-0.465848
H	4.596702	6.707151	-0.298511
H	2.495590	1.501249	-3.734461
C	1.157555	-1.095766	0.388484
C	1.677912	-0.393776	1.483698
C	1.604135	-2.410104	0.196151
C	2.623232	-0.967775	2.335376
H	1.346514	0.621814	1.665380
C	2.529377	-3.001605	1.063260

H	1.250776	-2.972056	-0.662617
C	3.050837	-2.282254	2.136508
H	3.786700	-2.730633	2.794704
C	2.943953	-4.432692	0.857408
C	3.280526	-0.136865	3.395251
F	2.096529	-5.291820	1.481003
F	4.178248	-4.680313	1.356812
F	2.956869	-4.778805	-0.445443
F	4.481223	0.366915	2.965350
F	3.554817	-0.840068	4.514905
F	2.537404	0.932804	3.758463
O	-1.256015	-0.635579	-0.023254
C	-2.464968	-0.915949	-0.424767
C	-2.899983	-0.997422	-1.844128
C	-2.621902	-0.004755	-2.798259
C	-3.638033	-2.127696	-2.224976
C	-3.044623	-0.165508	-4.113697
H	-2.102438	0.900452	-2.499000
C	-4.050661	-2.290767	-3.547904
H	-3.869606	-2.892923	-1.488177
C	-3.751544	-1.311656	-4.494259
H	-2.816856	0.601735	-4.847800
H	-4.603826	-3.180450	-3.835055
H	-4.069884	-1.435688	-5.525630
H	-2.943073	-1.661721	0.215089
C	-3.568156	0.690748	0.308848
H	-2.763135	1.062167	0.931037
H	-3.711958	1.257658	-0.600658
C	-4.679759	0.124711	0.931150
C	-5.950948	-0.164461	0.246165
C	-6.162343	0.193282	-1.100392
C	-6.990074	-0.811366	0.944603
C	-7.373991	-0.089530	-1.723669
H	-5.382799	0.686995	-1.668225
C	-8.200000	-1.088167	0.317574
H	-6.834175	-1.090746	1.979701
C	-8.396359	-0.728125	-1.018394
H	-7.519328	0.190482	-2.762757
H	-8.991868	-1.585698	0.870214
H	-9.342539	-0.943442	-1.507228
O	-4.633568	-0.304650	2.184356
Si	-3.566482	-0.043760	3.539925
C	-2.077698	-1.162092	3.342617
H	-2.375110	-2.215381	3.278434
H	-1.486955	-0.914519	2.454749
H	-1.420283	-1.059836	4.215427
C	-4.662891	-0.585320	4.965202
H	-4.997714	-1.621300	4.839967
H	-4.117108	-0.526294	5.914737
H	-5.551889	0.049520	5.052728
C	-3.126692	1.776925	3.663389
H	-2.365188	2.083614	2.939626
H	-4.009030	2.414397	3.532644
H	-2.727673	1.974367	4.666906

TS 292

B3LYP/6-31G(d) = -3573.923954
B3LYP-D3(BJ)/def2-TZVPP/IEFPCM(propanonitrile) = -3575.491101
B3LYP-D3(BJ)/def2-TZVPP/IEFPCM(propanonitrile)//B3LYP-D3(BJ)/6-31G(d) Free Energy (Quasiharmonic) = -3574.776441

Frequencies (Top 3 out of 300)

1. -213.2241 cm^{-1}
2. 8.0862 cm^{-1}
3. 9.3895 cm^{-1}

B3LYP/6-31G(d) Molecular Geometry in Cartesian Coordinates

B	-0.035050	0.270430	-1.045520
O	-0.692800	0.390749	-2.359720
N	-1.266460	0.190869	-0.099620
C	-2.525050	0.200159	-0.873830
S	-1.276500	-0.263801	1.492120
C	-2.028520	0.341779	-2.316970
O	-2.734100	0.434109	-3.295770
H	-3.224771	2.249399	-1.080810
H	-3.046370	-0.762131	-0.784380
C	-3.528530	1.346778	-0.537490
C	-4.958100	0.994618	-0.844570
C	-5.943660	0.539258	0.108480
C	-7.148880	0.300697	-0.610380
N	-6.894660	0.598907	-1.933350
C	-5.580480	1.005548	-2.068600
H	-5.168810	1.244128	-3.038150
C	-8.267400	-0.370833	1.386260
C	-5.926190	0.308948	1.497010
H	-9.227970	-0.326003	-0.552000
H	-7.551360	0.502507	-2.691680
C	-7.084350	-0.143963	2.119100
H	-9.156980	-0.723643	1.901450
H	-5.018480	0.477328	2.070590
C	-8.316050	-0.150873	0.013840
H	-7.080710	-0.325943	3.190680
C	-1.567259	-2.035181	1.578240
O	-2.426370	0.368169	2.158110
O	0.081620	0.008790	1.992140
C	-2.039088	-4.796921	1.678940
C	-2.876769	-2.518431	1.479760
C	-0.495149	-2.912511	1.749730
C	-0.740718	-4.283761	1.797920
C	-3.099179	-3.892921	1.521500
H	-3.713229	-1.832212	1.396740
H	0.512021	-2.529350	1.859560
H	0.094352	-4.966940	1.933210
H	-4.116668	-4.267392	1.439440
C	-2.296488	-6.283051	1.755840
H	-2.552338	-6.586311	2.779850
H	-3.131998	-6.578331	1.112380
H	-1.415118	-6.858551	1.455300
H	-3.431420	1.565868	0.526470
C	1.064360	-0.917330	-1.102220
C	1.990391	-1.198360	-0.085490
C	1.101181	-1.745760	-2.237860
C	2.875521	-2.278889	-0.181370
C	1.999751	-2.810410	-2.348030
H	0.409061	-1.555920	-3.050840
C	2.890691	-3.091529	-1.314230
H	3.569151	-3.933249	-1.383540
C	2.039531	-3.628200	-3.610490
C	3.818251	-2.559509	0.952610
F	0.815721	-3.768940	-4.162690
F	2.531852	-4.870530	-3.393660
F	2.829641	-3.055709	-4.551650
F	4.359191	-3.793589	0.888500
F	3.207251	-2.449989	2.158870
F	4.864051	-1.682299	0.984180
O	0.696470	1.645620	-0.876540
C	0.353099	2.589830	-0.065970
C	-0.161731	3.855780	-0.625370
C	-0.762971	4.803469	0.220360
C	-0.126561	4.086120	-2.010690
C	-1.305792	5.970439	-0.308170
H	-0.817081	4.613289	1.289950
C	-0.668152	5.257629	-2.534510
H	0.294789	3.325770	-2.659790
C	-1.252552	6.202409	-1.686420
H	-1.778222	6.694739	0.349100
H	-0.647542	5.428439	-3.607040
H	-1.678802	7.112609	-2.099130

H	0.004499	2.307020	0.928840
C	2.202149	3.485630	0.747570
H	1.704509	4.157930	1.432440
H	2.494069	3.921350	-0.199290
C	2.950589	2.433471	1.253570
C	2.822929	1.911711	2.625780
C	1.983549	2.522060	3.577860
C	3.557880	0.773691	3.005840
C	1.882489	2.007880	4.864710
H	1.400719	3.399400	3.319120
C	3.447680	0.257491	4.293290
H	4.198690	0.288821	2.281320
C	2.612840	0.872620	5.227030
H	1.226689	2.487680	5.585190
H	4.007710	-0.633529	4.560820
H	2.525350	0.468320	6.231670
O	3.798920	1.758861	0.487420
Si	4.723619	2.141211	-0.927330
C	5.484539	3.847991	-0.683090
H	6.201139	4.044112	-1.490730
H	4.749569	4.659271	-0.693960
H	6.034449	3.904072	0.263490
C	3.645099	2.054981	-2.458730
H	4.270389	2.160761	-3.354580
H	3.122840	1.095201	-2.521010
H	2.886329	2.843321	-2.485080
H	1.991280	-0.593910	0.813210
C	6.046610	0.815372	-0.924790
H	5.609310	-0.183499	-0.839550
H	6.633160	0.853992	-1.850690
H	6.737870	0.946452	-0.084490

TS 293

B3LYP/6-31G(d) = -3573.925749
B3LYP-D3(BJ)/def2-TZVPP/IEFPCM(propanonitrile) = -3575.49311
B3LYP-D3(BJ)/def2-TZVPP/IEFPCM(propanonitrile)//B3LYP-D3(BJ)/6-31G(d) Free Energy (Quasiharmonic) = -3574.776436

Frequencies (Top 3 out of 300)

1. -253.0746 cm^{-1}
2. 9.4949 cm^{-1}
3. 11.9746 cm^{-1}

B3LYP/6-31G(d) Molecular Geometry in Cartesian Coordinates

B	0.138360	0.109263	1.035740
O	0.739376	-0.113443	2.358079
N	1.393119	0.011797	0.108911
C	2.617837	-0.148168	0.923961
S	1.497435	-0.208814	-1.523218
C	2.066288	-0.269352	2.347687
O	2.729664	-0.442837	3.344663
H	3.307051	1.829510	1.517380
H	3.133496	-1.083446	0.673578
C	3.642602	1.027732	0.849085
C	5.049271	0.610486	1.177729
C	6.077458	0.260970	0.224735
C	7.239122	-0.094127	0.966737
N	6.919842	0.037017	2.302693
C	5.606454	0.450593	2.422375
H	5.149096	0.569130	3.393560
C	8.452920	-0.525248	-1.040575
C	6.131139	0.213537	-1.181030
H	9.309063	-0.752031	0.933614
H	7.532981	-0.172905	3.074419
C	7.314259	-0.179597	-1.796881
H	9.363560	-0.827284	-1.551262
H	5.258533	0.475969	-1.773118
C	8.431306	-0.485892	0.349509
H	7.365476	-0.220140	-2.881867
C	1.703097	-1.965217	-1.846833
O	2.720310	0.438226	-2.020587
O	0.196879	0.210299	-2.086504
C	2.056306	-4.708728	-2.307786
C	2.985300	-2.520614	-1.776430
C	0.601084	-2.758303	-2.172332
C	0.787857	-4.120077	-2.401602
C	3.148730	-3.885987	-2.000708
H	3.847361	-1.891576	-1.580595
H	-0.382740	-2.316540	-2.270362
H	-0.069485	-4.734932	-2.664764
H	4.146196	-4.315253	-1.947575
C	2.239933	-6.192312	-2.520501
H	2.150685	-6.738510	-1.572014
H	1.483699	-6.596878	-3.201124
H	3.228112	-6.419130	-2.934001
H	3.609474	1.425920	-0.165852
C	-1.132774	-0.887961	0.865314
C	-1.889566	-1.070838	-0.303253
C	-1.518767	-1.646425	1.985528
C	-2.932933	-2.000149	-0.365387
C	-2.583618	-2.550218	1.938020
H	-0.966071	-1.534217	2.911128
C	-3.291162	-2.748233	0.754645
H	-4.094331	-3.474596	0.704668
C	-3.014232	-3.268450	3.186805
C	-3.713978	-2.167102	-1.635599
F	-3.919321	-2.538803	3.892763
F	-1.982954	-3.515531	4.019800
F	-3.608920	-4.453084	2.912548
F	-2.972028	-1.873187	-2.735756
F	-4.803732	-1.351088	-1.687238
F	-4.178019	-3.425679	-1.793026
O	-0.302533	1.612287	1.126947
C	-0.240460	2.482751	0.167286
C	0.486243	3.748290	0.436955
C	0.823479	4.113969	1.749457
C	0.891437	4.560569	-0.633278
C	1.541601	5.284149	1.984029
H	0.538120	3.461108	2.568179
C	1.614471	5.726933	-0.394997
H	0.658557	4.263050	-1.653125
C	1.935026	6.094137	0.914609
H	1.805939	5.560225	3.000996
H	1.936685	6.343758	-1.229090
H	2.500805	7.002864	1.100482
H	-0.190535	2.105203	-0.855243
C	-2.143819	3.389175	-0.102393
H	-2.101196	3.982513	0.800550
H	-1.905023	3.930136	-1.010184
C	-3.108934	2.383885	-0.171033
C	-3.699593	1.764330	1.020070
C	-4.760903	0.847553	0.880538
C	-3.225994	2.062787	2.314315
C	-5.332396	0.256367	2.000934
H	-5.109430	0.587771	-0.110680
C	-3.797575	1.460604	3.430016
H	-2.391478	2.738602	2.452465
C	-4.851259	0.556730	3.278283
H	-6.145358	-0.453290	1.878969
H	-3.410967	1.686959	4.419145
H	-5.287280	0.077936	4.149892
O	-3.507261	1.868675	-1.326670
Si	-3.531196	2.460644	-2.960200
C	-4.458028	4.099444	-2.929780
H	-4.540795	4.501986	-3.947026
H	-5.474667	3.972202	-2.540801
H	-3.956065	4.856039	-2.316926
C	-4.487159	1.136769	-3.873282

H	-4.604339	1.416428	-4.927543
H	-3.972938	0.172475	-3.832899
H	-5.487139	0.997426	-3.448681
H	-1.641105	-0.506180	-1.191919
C	-1.786030	2.640879	-3.630531
H	-1.285904	3.548671	-3.273930
H	-1.156210	1.781491	-3.374338
H	-1.827555	2.706369	-4.725473

TS 294

B3LYP/6-31G(d) = -3573.921929
B3LYP-D3(BJ)/def2-TZVPP/IEFPCM(propanonitrile) = -3575.492208
B3LYP-D3(BJ)/def2-TZVPP/IEFPCM(propanonitrile)//B3LYP-D3(BJ)/6-31G(d) Free Energy (Quasiharmonic) = -3574.776406

Frequencies (Top 3 out of 300)

1. -235.3490 cm^{-1}
2. 5.8793 cm^{-1}
3. 9.8922 cm^{-1}

B3LYP/6-31G(d) Molecular Geometry in Cartesian Coordinates

B	0.308740	-0.513880	0.735420
O	0.384240	-0.796560	2.182420
N	0.217310	1.050150	0.737660
C	0.232099	1.556310	2.122130
S	0.682889	2.067380	-0.473330
C	0.462810	0.293270	2.944050
O	0.655420	0.270150	4.142890
H	-1.206911	3.170240	2.007430
H	1.081729	2.231110	2.272570
C	-1.049811	2.281660	2.627060
C	-2.312491	1.457860	2.666770
C	-3.527571	1.687240	1.919290
C	-4.440790	0.653440	2.267660
N	-3.820060	-0.137370	3.210380
C	-2.545840	0.342680	3.439770
H	-1.900600	-0.144270	4.157190
C	-6.101221	1.543010	0.807880
C	-3.948831	2.673670	1.010400
H	-6.390030	-0.254550	1.978330
H	-4.165360	-1.031400	3.523560
C	-5.225141	2.594360	0.467880
H	-7.090261	1.503190	0.359060
H	-3.287351	3.488350	0.731700
C	-5.721110	0.559350	1.712440
H	-5.556331	3.355100	-0.234370
C	-0.717691	3.055860	-1.025960
O	1.043450	1.214710	-1.623420
O	1.661279	3.033950	0.058760
C	-2.777481	4.666250	-2.040070
C	-0.766831	4.408940	-0.689390
C	-1.684391	2.496000	-1.865490
C	-2.703531	3.302200	-2.361780
C	-1.798611	5.202120	-1.194040
H	0.007499	4.834460	-0.060700
H	-1.644441	1.449790	-2.141760
H	-3.453761	2.860010	-3.012410
H	-1.833991	6.257350	-0.934130
C	-3.874271	5.532350	-2.612480
H	-3.707901	5.725390	-3.680270
H	-3.927261	6.500730	-2.105530
H	-4.853221	5.047890	-2.522770
H	-0.797411	2.645520	3.631500
C	-0.922030	-1.286090	0.027890
C	-1.189270	-1.199220	-1.345030
C	-1.795870	-2.068060	0.797870
C	-2.300300	-1.826770	-1.916440
H	-0.527280	-0.621560	-1.984530
C	-2.902140	-2.706670	0.230540
H	-1.607820	-2.174730	1.860580
C	-3.169140	-2.584060	-1.133210
H	-4.027680	-3.073440	-1.576250
C	-3.831740	-3.491530	1.112720
C	-2.553990	-1.635620	-3.384800
F	-4.712330	-2.687830	1.776860
F	-3.163890	-4.179860	2.066300
F	-4.576360	-4.378230	0.418560
F	-1.466400	-1.945370	-4.128420
F	-3.580080	-2.385530	-3.839650
F	-2.853100	-0.339790	-3.672800
O	1.628600	-1.156600	0.240540
C	2.211540	-1.363800	-0.895620
C	2.189980	-2.728910	-1.469760
C	2.501840	-2.908749	-2.827070
C	1.813720	-3.835320	-0.692620
C	2.441030	-4.176810	-3.398850
H	2.775730	-2.048229	-3.433500
C	1.758460	-5.102490	-1.266990
H	1.552910	-3.683730	0.349640
C	2.073320	-5.276320	-2.618100
H	2.670860	-4.308049	-4.452400
H	1.460890	-5.955970	-0.664280
H	2.023710	-6.265810	-3.064240
H	2.253300	-0.533960	-1.601460
C	4.309430	-1.350549	-0.449010
H	4.230500	-2.155809	0.270390
H	4.679260	-1.627499	-1.427010
C	4.516110	-0.056349	0.011630
C	4.926440	1.058141	-0.860930
C	4.682709	2.384551	-0.462160
C	5.587820	0.824821	-2.083130
C	5.073569	3.445401	-1.273850
H	4.146749	2.571581	0.458610
C	5.987149	1.889241	-2.883750
H	5.821090	-0.187629	-2.396360
C	5.728089	3.203061	-2.482630
H	4.855769	4.463081	-0.964330
H	6.506179	1.695541	-3.818150
H	6.036219	4.033461	-3.112100
O	4.240830	0.278131	1.264000
Si	4.607040	-0.476099	2.787980
C	4.161250	0.845101	4.034960
H	4.585819	1.816071	3.756840
H	3.076590	0.950811	4.142090
H	4.560700	0.576041	5.021020
C	6.460550	-0.810819	2.736780
H	6.731880	-1.491359	1.921410
H	7.030820	0.115801	2.606150
H	6.788510	-1.275219	3.674910
C	3.629780	-2.055319	3.057370
H	3.746050	-2.364649	4.104400
H	2.561750	-1.901779	2.874220
H	3.977160	-2.885989	2.432590

TS 295

B3LYP/6-31G(d) = -3573.909944
B3LYP-D3(BJ)/def2-TZVPP/IEFPCM(propanonitrile) = -3575.491785
B3LYP-D3(BJ)/def2-TZVPP/IEFPCM(propanonitrile)//B3LYP-D3(BJ)/6-31G(d) Free Energy (Quasiharmonic) = -3574.775931

Frequencies (Top 3 out of 300)

1. -305.7232 cm^{-1}
2. 9.6040 cm^{-1}
3. 10.9779 cm^{-1}

B3LYP/6-31G(d) Molecular Geometry in Cartesian Coordinates

B	-0.463363	0.624072	0.191148
O	-0.047939	0.534849	1.618516
N	-1.019003	2.072075	0.134240
C	-1.028245	2.681799	1.469835
S	-1.149674	3.042768	-1.190678
C	-0.303664	1.640918	2.322655
O	0.003702	1.787357	3.488298
H	-2.865535	3.802109	1.458042
H	-0.421004	3.598290	1.497169
C	-2.420558	3.015005	2.071931
C	-3.357300	1.845079	2.182433
C	-4.414968	1.479196	1.268744
C	-5.014783	0.294245	1.775111
N	-4.365185	-0.025665	2.951125
C	-3.361744	0.897212	3.178600
H	-2.725222	0.816296	4.049611
C	-6.542721	0.223205	-0.051694
C	-4.912280	2.034597	0.075534
H	-6.501234	-1.266434	1.519202
H	-4.462868	-0.905388	3.433459
C	-5.969447	1.403992	-0.568196
H	-7.362723	-0.250685	-0.585138
H	-4.458660	2.925867	-0.347273
C	-6.073185	-0.349022	1.124451
H	-6.356308	1.820409	-1.494467
C	0.359014	4.031845	-1.283066
O	-2.228176	4.014734	-0.956009
O	-1.158360	2.175096	-2.375448
C	2.710891	5.561946	-1.425854
C	1.431474	3.587356	-2.059434
C	0.442582	5.244827	-0.596061
C	1.618011	5.993445	-0.663844
C	2.594911	4.351301	-2.125651
H	1.332004	2.666263	-2.622189
H	-0.414494	5.610745	-0.041005
H	1.679867	6.936203	-0.125370
H	3.424086	4.010445	-2.742200
C	3.964266	6.398577	-1.529331
H	3.921423	7.067495	-2.399165
H	4.101758	7.026254	-0.642919
H	4.856274	5.773573	-1.645425
H	-2.223072	3.442501	3.062860
C	-1.478461	-0.570688	-0.203394
C	-1.988670	-0.737655	-1.500396
C	-1.895124	-1.489313	0.770469
C	-2.861868	-1.784445	-1.808773
H	-1.721988	-0.024545	-2.273907
C	-2.772539	-2.534187	0.467606
H	-1.523904	-1.389291	1.784373
C	-3.260456	-2.692246	-0.828398
H	-3.937247	-3.502891	-1.070673
C	-3.212979	-3.472543	1.552789
C	-3.316620	-1.974778	-3.229575
F	-2.234875	-3.691455	2.464107
F	-3.590337	-4.677239	1.071520
F	-4.281668	-2.996059	2.257626
F	-3.526146	-0.803071	-3.861673
F	-2.387214	-2.648318	-3.960432
F	-4.460999	-2.692393	-3.309660
O	0.820134	0.596080	-0.663479
C	1.507654	-0.316245	-1.299193
C	1.521951	-1.748933	-0.908954
C	1.355095	-2.178780	0.417997
C	1.650808	-2.704899	-1.929228
C	1.304279	-3.541122	0.707543
H	1.232183	-1.450569	1.211912
C	1.593631	-4.066924	-1.636701
H	1.763886	-2.377757	-2.960293
C	1.420530	-4.487204	-0.315844
H	1.145130	-3.865214	1.732089
H	1.664356	-4.796229	-2.438789
H	1.361774	-5.547412	-0.085593
H	1.527349	-0.168704	-2.383278
C	3.417337	0.402036	-1.239477
H	3.679799	0.209230	-2.275778
H	3.109088	1.417669	-1.020421
C	4.285852	-0.219854	-0.323257
C	4.378079	0.162041	1.090729
C	3.265182	0.728101	1.740167
C	5.575850	-0.009107	1.812262
C	3.327428	1.059026	3.091977
H	2.337019	0.873319	1.201059
C	5.648184	0.362313	3.150409
H	6.459418	-0.389067	1.312685
C	4.520636	0.880836	3.796107
H	2.437890	1.442529	3.583343
H	6.582936	0.247581	3.691867
H	4.575750	1.152720	4.846559
O	5.043027	-1.188764	-0.797746
Si	5.871080	-2.667053	-0.434891
C	5.241407	-3.432424	1.156605
H	5.706103	-4.418725	1.284574
H	5.475503	-2.833568	2.041332
H	4.156730	-3.579705	1.121540
C	7.706746	-2.256187	-0.387465
H	8.000114	-1.664853	-1.262289
H	8.015128	-1.709370	0.509521
H	8.285155	-3.188431	-0.414188
C	5.500370	-3.741076	-1.924558
H	6.029985	-4.698810	-1.848264
H	4.429542	-3.952866	-2.004148
H	5.822512	-3.255333	-2.852520

TS 296

B3LYP/6-31G(d) = -3573.925128
B3LYP-D3(BJ)/def2-TZVPP/IEFPCM(propanonitrile) = -3575.491185
B3LYP-D3(BJ)/def2-TZVPP/IEFPCM(propanonitrile)//B3LYP-D3(BJ)/6-31G(d) Free Energy (Quasiharmonic) = -3574.775719

Frequencies (Top 3 out of 300)

1. -246.3861 cm^{-1}
2. 6.1884 cm^{-1}
3. 8.5178 cm^{-1}

B3LYP/6-31G(d) Molecular Geometry in Cartesian Coordinates

B	-0.029651	0.175429	-1.159247
O	-0.715371	0.059804	-2.459262
N	-1.243501	0.117520	-0.176013
C	-2.511706	-0.033623	-0.922289
S	-1.230023	-0.181169	1.448354
C	-2.043280	-0.061282	-2.381424
O	-2.768308	-0.145582	-3.346855
H	-3.309418	1.939290	-1.374038
H	-2.983807	-0.998728	-0.696429
C	-3.563886	1.099983	-0.715987
C	-4.979210	0.648020	-0.950176
C	-5.931888	0.274767	0.069693
C	-7.132960	-0.106296	-0.592519
N	-6.907682	0.031798	-1.946714
C	-5.615387	0.475504	-2.154890
H	-5.226120	0.606145	-3.153779
C	-8.197540	-0.566374	1.491713
C	-5.888840	0.224305	1.475747
H	-9.180382	-0.811181	-0.420207
H	-7.567718	-0.189453	-2.675442
C	-7.018048	-0.196077	2.169767

H	-9.064303	-0.889331	2.062369
H	-4.983760	0.504252	2.008227
C	-8.271241	-0.525507	0.103500
H	-6.994228	-0.239392	3.255578
C	-1.437103	-1.945864	1.713226
O	-2.399209	0.464554	2.065592
O	0.115217	0.206061	1.920594
C	-1.788557	-4.702204	2.084061
C	-2.729164	-2.481456	1.738328
C	-0.321585	-2.765943	1.894289
C	-0.507156	-4.133769	2.081129
C	-2.891865	-3.853876	1.917734
H	-3.595665	-1.834539	1.649088
H	0.675085	-2.344198	1.910534
H	0.363471	-4.767404	2.231558
H	-3.896456	-4.268932	1.939672
C	-1.974008	-6.191622	2.248604
H	-2.941231	-6.427964	2.703879
H	-1.186721	-6.627369	2.872563
H	-1.937701	-6.700715	1.276299
H	-3.464584	1.456989	0.309897
C	1.160516	-0.920671	-1.081335
C	2.088526	-1.029197	-0.034159
C	1.275862	-1.855714	-2.125086
C	3.043514	-2.051787	-0.004608
H	2.047899	-0.329311	0.790686
C	2.250482	-2.856894	-2.117145
H	0.586934	-1.799679	-2.960444
C	3.135346	-2.972503	-1.046806
H	3.873977	-3.764715	-1.023108
C	2.378369	-3.786760	-3.292673
C	3.900921	-2.220642	1.215730
F	3.160047	-3.259101	-4.266645
F	1.182788	-4.058768	-3.858158
F	2.939319	-4.969274	-2.944324
F	4.372329	-1.038786	1.690446
F	4.971292	-3.014231	0.998139
F	3.201934	-2.780090	2.241788
O	0.558366	1.620259	-1.195677
C	0.214473	2.599353	-0.420221
C	-0.374784	3.807357	-1.040567
C	-0.387454	3.953536	-2.436999
C	-0.986580	4.783088	-0.236230
C	-0.987375	5.070500	-3.014506
H	0.045133	3.171047	-3.051755
C	-1.588416	5.894925	-0.818035
H	-1.001280	4.659340	0.844195
C	-1.583383	6.044080	-2.208444
H	-1.002516	5.175493	-4.095634
H	-2.068605	6.640865	-0.191181
H	-2.055067	6.911264	-2.662283
H	-0.130266	2.347981	0.582720
C	1.964948	3.587706	0.366466
H	1.458453	4.039563	1.209653
H	2.134214	4.242591	-0.477034
C	2.926396	2.612227	0.619993
C	3.859847	2.141896	-0.414892
C	5.021949	1.440411	-0.041757
C	3.623766	2.391089	-1.781094
C	5.929537	1.017000	-1.006334
H	5.202643	1.233027	1.004831
C	4.528389	1.951771	-2.742476
H	2.718382	2.895170	-2.096060
C	5.685119	1.269454	-2.358799
H	6.825940	0.483082	-0.704426
H	4.326056	2.134359	-3.793671
H	6.390845	0.930145	-3.111969
O	3.030964	1.992261	1.781248
Si	2.646648	2.290247	3.448795
C	4.020402	3.431951	4.044489
H	4.023038	4.383487	3.499766
H	3.892783	3.662148	5.109471
H	5.006102	2.969917	3.919623
C	2.722632	0.584121	4.207007
H	3.631182	0.049718	3.913584
H	2.694500	0.643576	5.301854
H	1.862425	-0.005258	3.873132
C	0.954239	3.069026	3.697769
H	0.891721	4.111163	3.365648
H	0.176718	2.478200	3.203170
H	0.730624	3.061570	4.772910

TS 297

B3LYP/6-31G(d) = -3573.914565
B3LYP-D3(BJ)/def2-TZVPP/IEFPCM(propanonitrile) = -3575.491624
B3LYP-D3(BJ)/def2-TZVPP/IEFPCM(propanonitrile)//B3LYP-D3(BJ)/6-31G(d) Free Energy (Quasiharmonic) = -3574.775577

Frequencies (Top 3 out of 300)

1. -274.0361 cm^{-1}
2. 10.8289 cm^{-1}
3. 12.2224 cm^{-1}

B3LYP/6-31G(d) Molecular Geometry in Cartesian Coordinates

B	-0.499160	0.243514	0.327091
O	-0.519009	0.356626	1.801042
N	-0.338762	1.753895	-0.064139
C	-0.639941	2.607845	1.102168
S	-0.149783	2.408538	-1.555110
C	-0.528740	1.609686	2.255216
O	-0.448229	1.916710	3.425573
H	-1.944335	4.146135	0.344385
H	0.148888	3.356951	1.242870
C	-1.997049	3.374054	1.117934
C	-3.239835	2.554789	0.923833
C	-3.993426	1.821652	1.917185
C	-5.080041	1.199356	1.239307
N	-4.999735	1.566987	-0.089801
C	-3.885316	2.358244	-0.272749
H	-3.618176	2.722926	-1.253890
C	-5.850210	0.210622	3.267731
C	-3.865508	1.628776	3.304964
H	-6.825009	-0.086645	1.359614
H	-5.516940	1.145237	-0.846678
C	-4.791497	0.828584	3.963405
H	-6.551906	-0.418496	3.808835
H	-3.039774	2.076438	3.850186
C	-6.009995	0.389723	1.898512
H	-4.693988	0.667965	5.033925
C	1.120177	3.673672	-1.347571
O	-1.336161	3.128225	-2.049611
O	0.389229	1.328856	-2.410313
C	3.129698	5.629166	-1.197194
C	1.002215	4.848987	-2.092535
C	2.226860	3.471297	-0.520765
C	3.218462	4.447977	-0.447743
C	2.005475	5.812918	-2.014111
H	0.126081	5.002804	-2.712655
H	2.301829	2.575201	0.086067
H	4.069940	4.291390	0.209990
H	1.908395	6.728457	-2.592682
C	4.221652	6.670491	-1.137740
H	4.772955	6.620774	-0.193279
H	3.816547	7.682380	-1.242749
H	4.947599	6.525889	-1.948949
O	0.751722	-0.645291	0.117246
C	1.273387	-1.134577	-0.972661
H	0.943167	-0.691030	-1.912890

H	-2.022835	3.893960	2.084413
C	1.540067	-2.595004	-0.997774
C	1.430183	-3.364835	0.170674
C	1.787593	-3.232861	-2.222781
C	1.567433	-4.750709	0.110053
H	1.197986	-2.869644	1.107784
C	1.918974	-4.618697	-2.281623
H	1.852077	-2.642724	-3.134080
C	1.810395	-5.380613	-1.114437
H	1.460866	-5.343093	1.014591
H	2.092149	-5.105618	-3.237162
H	1.900243	-6.462446	-1.161340
C	-1.767433	-0.597330	-0.221006
C	-2.372703	-0.416480	-1.471554
C	-2.302555	-1.595072	0.612398
C	-3.473072	-1.187599	-1.863865
H	-2.000317	0.336615	-2.157396
C	-3.384149	-2.382532	0.214848
H	-1.872439	-1.748862	1.596412
C	-3.982002	-2.180257	-1.028882
H	-4.825015	-2.786151	-1.341156
C	-3.966081	-3.406731	1.150044
C	-4.181531	-0.869038	-3.147857
F	-4.448001	-4.481305	0.477045
F	-3.053026	-3.866410	2.035092
F	-5.116538	0.114861	-2.966026
F	-4.997335	-2.908775	1.869471
F	-4.848603	-1.931888	-3.649396
F	-3.346131	-0.422979	-4.106631
C	3.087728	-0.242013	-1.266855
H	2.682750	0.760766	-1.223867
H	3.255313	-0.631739	-2.266411
C	4.069671	-0.591997	-0.332974
O	4.886701	-1.580507	-0.653748
Si	5.817092	-2.833245	0.112374
C	7.611897	-2.271459	0.193190
H	8.256070	-3.149481	0.328804
H	7.813661	-1.586783	1.022559
H	7.920603	-1.784185	-0.739004
C	5.105507	-3.235326	1.800868
H	5.646747	-4.090144	2.226434
H	5.191715	-2.401582	2.503985
H	4.049375	-3.515649	1.727073
C	5.657893	-4.257408	-1.093044
H	6.043070	-3.979586	-2.080827
H	6.234901	-5.122036	-0.742060
H	4.616200	-4.570730	-1.212338
C	4.252570	0.104768	0.958372
C	3.143343	0.488834	1.731383
C	5.547445	0.422515	1.409347
C	3.326243	1.147880	2.947103
H	2.143125	0.231008	1.404216
C	5.722948	1.107046	2.609403
H	6.408464	0.173777	0.798454
C	4.614640	1.463004	3.384607
H	2.455737	1.417539	3.539093
H	6.725034	1.367547	2.938651
H	4.756224	1.991361	4.323287

TS 298

B3LYP/6-31G(d) = -3573.924319
B3LYP-D3(BJ)/def2-TZVPP/IEFPCM(propanonitrile) = -3575.491895
B3LYP-D3(BJ)/def2-TZVPP/IEFPCM(propanonitrile)//B3LYP-D3(BJ)/6-31G(d) Free Energy (Quasiharmonic) = -3574.775359

Frequencies (Top 3 out of 300)

1. -250.9978 cm^{-1}
2. 9.4612 cm^{-1}
3. 10.8187 cm^{-1}

B3LYP/6-31G(d) Molecular Geometry in Cartesian Coordinates

B	0.167160	0.183290	0.986530
O	0.857390	-0.015500	2.270340
N	1.379110	0.282020	0.000400
C	2.655180	0.173250	0.742010
S	1.402130	0.232790	-1.648450
C	2.187480	-0.000880	2.190190
O	2.924220	-0.102030	3.145590
H	3.789110	1.796271	1.624600
H	3.180700	-0.750890	0.466200
C	3.650160	1.377801	0.620990
C	4.996230	1.028041	0.051800
C	6.055900	0.297801	0.711310
C	7.152850	0.237351	-0.192890
N	6.767530	0.896041	-1.343720
C	5.475380	1.367591	-1.189450
H	4.979530	1.901721	-1.987060
C	8.451200	-0.985259	1.388940
C	6.181440	-0.306449	1.975960
H	9.184570	-0.434578	-0.571330
H	7.341140	1.030931	-2.161100
C	7.375600	-0.939769	2.299690
H	9.372100	-1.487848	1.673250
H	5.350800	-0.284199	2.676090
C	8.355390	-0.399289	0.131370
H	7.484440	-1.411419	3.272780
C	1.836480	-1.436910	-2.149470
O	2.472460	1.106420	-2.148370
O	0.016820	0.495910	-2.094380
C	2.546411	-4.050040	-2.875030
C	3.184770	-1.809070	-2.165260
C	0.840910	-2.347760	-2.508570
C	1.204941	-3.643780	-2.868580
C	3.525941	-3.113090	-2.518520
H	3.954760	-1.085089	-1.919280
H	-0.199550	-2.047230	-2.521040
H	0.429831	-4.352300	-3.150720
H	4.573461	-3.404389	-2.521480
C	2.929491	-5.451030	-3.288650
H	3.841921	-5.782449	-2.782280
H	2.133771	-6.167720	-3.060890
H	3.118121	-5.505400	-4.369220
H	3.176250	2.145840	0.007560
C	-0.967840	-0.967900	0.821720
C	-1.749180	-1.197371	-0.322170
C	-1.191860	-1.825280	1.914440
C	-2.659690	-2.256621	-0.390410
C	-2.124350	-2.864901	1.862040
H	-0.615880	-1.682260	2.821380
C	-2.856569	-3.100961	0.700710
H	-3.555479	-3.927871	0.645310
C	-2.392559	-3.697371	3.085150
C	-3.477950	-2.462241	-1.631010
F	-3.373849	-3.151001	3.852820
F	-1.306219	-3.818470	3.874320
F	-2.807389	-4.946251	2.768190
F	-3.781399	-3.761581	-1.839190
F	-2.841280	-2.016091	-2.746190
F	-4.666180	-1.796821	-1.584940
O	-0.457140	1.605940	1.178220
C	-0.559231	2.538460	0.282980
C	0.014419	3.866730	0.612080
C	0.393349	4.174920	1.927910
C	0.234189	4.804500	-0.408990
C	0.967279	5.410740	2.216340
H	0.255909	3.428920	2.704030
C	0.813179	6.037090	-0.117300
H	-0.030511	4.555990	-1.434220

C	1.174459	6.344920	1.197240
H	1.265579	5.642200	3.235000
H	0.991269	6.753620	-0.914180
H	1.627749	7.305800	1.424700
H	-0.522870	2.239620	-0.765490
C	-2.578461	3.200339	0.166970
H	-2.560841	3.744929	1.100800
H	-2.466991	3.815609	-0.717720
C	-3.399370	2.075419	0.087040
C	-3.827520	1.316319	1.266580
C	-4.747270	0.257879	1.123830
C	-3.334790	1.618489	2.552850
C	-5.163500	-0.467521	2.233700
H	-5.105470	-0.003051	0.136300
C	-3.750660	0.883269	3.657610
H	-2.604691	2.405909	2.691750
C	-4.665200	-0.160721	3.502930
H	-5.866400	-1.285831	2.108860
H	-3.350000	1.116849	4.639440
H	-4.977970	-0.742471	4.364730
O	-3.788980	1.570589	-1.076400
Si	-3.993320	2.238639	-2.666570
C	-5.144611	3.717999	-2.489010
H	-5.341991	4.160459	-3.473350
H	-6.108501	3.420409	-2.060790
H	-4.723341	4.504669	-1.853580
C	-4.796980	0.837899	-3.611170
H	-5.014480	1.152649	-4.639290
H	-4.146240	-0.039971	-3.655280
H	-5.739540	0.528369	-3.147090
H	-1.623940	-0.560561	-1.187480
C	-2.335231	2.711549	-3.411920
H	-1.950251	3.661729	-3.024190
H	-1.575450	1.940389	-3.241940
H	-2.450621	2.830609	-4.497020

TS 299

B3LYP/6-31G(d) = -3573.924352
B3LYP-D3(BJ)/def2-TZVPP/IEFPCM(propanonitrile) = -3575.491905
B3LYP-D3(BJ)/def2-TZVPP/IEFPCM(propanonitrile)//B3LYP-D3(BJ)/6-31G(d) Free Energy (Quasiharmonic) = -3574.775256

Frequencies (Top 3 out of 300)

1. -250.9618 cm^{-1}
2. 9.4463 cm^{-1}
3. 10.7958 cm^{-1}

B3LYP/6-31G(d) Molecular Geometry in Cartesian Coordinates

B	0.168946	0.182495	0.985293
O	0.860511	-0.018900	2.267975
N	1.379792	0.283529	-0.001885
C	2.656667	0.172850	0.738051
S	1.401005	0.235730	-1.650825
C	2.190534	-0.004008	2.186414
O	2.928266	-0.106951	3.140862
H	3.791258	1.794715	1.622065
H	3.181620	-0.750890	0.459813
C	3.651828	1.377341	0.618061
C	4.997569	1.028038	0.047813
C	6.057850	0.297617	0.706135
C	7.154126	0.237824	-0.198929
N	6.767807	0.897018	-1.349137
C	5.475711	1.368285	-1.193641
H	4.979183	1.902784	-1.990574
C	8.453941	-0.985323	1.381274
C	6.184467	-0.307308	1.970356
H	9.185683	-0.433492	-0.579273
H	7.340795	1.032469	-2.166859
C	7.379015	-0.940544	2.292850
H	9.375168	-1.487866	1.664626
H	5.354313	-0.285713	2.671066
C	8.357047	-0.398728	0.124090
H	7.488680	-1.412700	3.265604
C	1.831894	-1.434779	-2.152122
O	2.472361	1.107959	-2.150911
O	0.015697	0.501556	-2.095126
C	2.536882	-4.047392	-2.884702
C	3.179634	-1.808783	-2.171293
C	0.834882	-2.341910	-2.516557
C	1.196644	-3.636929	-2.882426
C	3.518563	-3.111778	-2.530510
H	3.951276	-1.084867	-1.930414
H	-0.204250	-2.037142	-2.535283
H	0.421022	-4.340232	-3.175924
H	4.566201	-3.402298	-2.543428
C	2.912145	-5.465075	-3.244520
H	3.906655	-5.513832	-3.700029
H	2.931467	-6.104136	-2.351738
H	2.193958	-5.905254	-3.944040
O	-0.455301	1.604642	1.180537
C	-0.558729	2.538714	0.287058
H	-0.523660	2.241717	-0.761980
H	3.177755	2.146121	0.005689
C	0.015091	3.866512	0.617718
C	0.395656	4.172514	1.933594
C	0.233361	4.806087	-0.402013
C	0.969739	5.407940	2.223405
H	0.259341	3.425160	2.708608
C	0.812517	6.038275	-0.108959
H	-0.032619	4.559288	-1.427323
C	1.175439	6.343904	1.205640
H	1.269309	5.637700	3.242072
H	0.989449	6.756205	-0.904831
H	1.628859	7.304470	1.434167
C	-0.965987	-0.968555	0.819021
C	-1.748638	-1.195305	-0.324528
C	-1.188467	-1.828772	1.909806
C	-2.658966	-2.254584	-0.394391
H	-1.624617	-0.556245	-1.188333
C	-2.120740	-2.868523	1.855848
H	-0.611459	-1.687882	2.816437
C	-2.854293	-3.101787	0.694802
H	-3.553053	-3.928736	0.638125
C	-2.387274	-3.704104	3.077199
C	-3.478806	-2.457280	-1.634429
F	-1.299715	-3.827509	3.864365
F	-3.367226	-3.159537	3.847823
F	-4.667728	-1.793383	-1.584514
F	-2.802839	-4.952090	2.757637
F	-3.781068	-3.756301	-1.846195
F	-2.844363	-2.006931	-2.749222
C	-2.578292	3.200420	0.175071
H	-2.559452	3.743009	1.110042
H	-2.468085	3.817600	-0.708446
C	-3.399199	2.075589	0.093840
O	-3.790411	1.573277	-1.070163
Si	-3.997000	2.244930	-2.658539
C	-2.339791	2.718618	-3.405352
H	-1.580024	1.946866	-3.238045
H	-2.456670	2.839960	-4.490039
H	-1.953891	3.667854	-3.016212
C	-4.802966	0.846718	-3.604909
H	-4.152909	-0.031498	-3.651819
H	-5.021748	1.163777	-4.632052
H	-5.745086	0.536893	-3.140119
C	-5.147275	3.724476	-2.476015
H	-6.110507	3.426500	-2.046574
H	-4.724508	4.509810	-1.839929

H	-5.346300	4.168830	-3.459174
C	-3.825638	1.313872	1.272313
C	-3.331216	1.613332	2.558569
C	-4.745431	0.255608	1.128519
C	-3.745520	0.875692	3.662307
H	-2.601032	2.400548	2.698181
C	-5.160077	-0.472229	2.237381
H	-5.104918	-0.003246	0.140916
C	-4.660128	-0.168084	3.506607
H	-3.343585	1.107219	4.644095
H	-5.863059	-1.290347	2.111716
H	-4.971699	-0.751708	4.367581

TS 300

B3LYP/6-31G(d) = -3573.923902
B3LYP-D3(BJ)/def2-TZVPP/IEFPCM(propanonitrile) = -3575.49169
B3LYP-D3(BJ)/def2-TZVPP/IEFPCM(propanonitrile)//B3LYP-D3(BJ)/6-31G(d) Free Energy (Quasiharmonic) = -3574.775208

Frequencies (Top 3 out of 300)

1. -230.8234 cm^{-1}
2. 5.7407 cm^{-1}
3. 11.4835 cm^{-1}

B3LYP/6-31G(d) Molecular Geometry in Cartesian Coordinates

B	-0.050391	0.640750	0.474765
O	-0.085357	0.957681	1.908693
N	-1.343787	1.328007	-0.059675
C	-1.903294	2.183121	1.005992
S	-1.399770	1.860981	-1.635424
C	-1.008543	1.882396	2.214311
O	-1.118511	2.400977	3.300256
H	-4.001125	2.279936	0.499317
H	-1.771722	3.244798	0.760293
C	-3.401620	1.957979	1.356995
C	-3.792880	0.561770	1.756489
C	-3.795627	-0.016518	3.084178
C	-4.247613	-1.359271	2.955024
N	-4.531897	-1.572753	1.620794
C	-4.239983	-0.423923	0.910500
H	-4.401668	-0.389923	-0.157933
C	-4.044971	-1.714130	5.303286
C	-3.479098	0.466948	4.367291
H	-4.712487	-3.244637	3.926727
H	-4.675932	-2.477670	1.198944
C	-3.607324	-0.382744	5.458531
H	-4.128918	-2.355653	6.176666
H	-3.110799	1.479323	4.496631
C	-4.371137	-2.219943	4.050869
H	-3.357856	-0.018349	6.451552
C	-3.076464	1.574305	-2.224712
O	-0.524826	0.965647	-2.410677
O	-1.197724	3.322742	-1.702578
C	-5.668713	1.134599	-3.199546
C	-3.436319	0.304602	-2.682947
C	-3.985197	2.631686	-2.272741
C	-5.273528	2.403653	-2.756174
C	-4.727615	0.094250	-3.162009
H	-2.713805	-0.503451	-2.677571
H	-3.675886	3.621191	-1.953674
H	-5.980447	3.228762	-2.796550
H	-5.005487	-0.894226	-3.519926
C	-7.074506	0.887285	-3.692109
H	-7.529655	1.803021	-4.082715
H	-7.717117	0.523999	-2.879172
H	-7.094850	0.131244	-4.484030
H	-3.621795	2.657967	2.171974
C	0.052637	-0.943775	0.202633
C	-1.084030	-1.634905	-0.240965
C	1.190748	-1.714939	0.476641
C	-1.089064	-3.018497	-0.425936
H	-1.987982	-1.071064	-0.428970
C	1.194154	-3.103818	0.297403
H	2.096070	-1.239430	0.839839
C	0.055663	-3.766973	-0.159453
H	0.062636	-4.841257	-0.301492
C	2.453123	-3.890126	0.518554
C	-2.350206	-3.706806	-0.862721
F	2.211513	-5.187234	0.801577
F	3.206091	-3.389199	1.522551
F	3.251222	-3.886974	-0.593851
F	-2.107906	-4.867956	-1.505610
F	-3.094345	-2.932678	-1.692607
F	-3.162019	-4.013720	0.189280
O	1.169892	1.465060	-0.100974
C	2.123035	1.959615	0.621787
C	2.207750	3.417960	0.845763
C	2.995668	3.897377	1.906647
C	1.479658	4.320260	0.053124
C	3.061413	5.261925	2.169849
H	3.538132	3.193671	2.534348
C	1.553356	5.685454	0.321780
H	0.842987	3.949941	-0.744376
C	2.343459	6.159069	1.372910
H	3.660150	5.626449	2.999695
H	0.983251	6.381478	-0.286939
H	2.391691	7.224822	1.579317
H	2.529270	1.342818	1.429390
C	3.865823	1.882524	-0.675990
H	3.302929	2.262556	-1.517511
H	4.489719	2.599935	-0.163316
C	4.217403	0.541828	-0.652426
C	5.154122	-0.033985	0.336888
C	5.851249	0.772324	1.260130
C	5.370488	-1.423283	0.361447
C	6.730915	0.203588	2.174297
H	5.714586	1.848272	1.267785
C	6.247846	-1.989085	1.283134
H	4.831740	-2.056038	-0.330743
C	6.932510	-1.179958	2.190071
H	7.261099	0.840039	2.876965
H	6.387914	-3.065800	1.295326
H	7.617915	-1.621675	2.908009
O	3.633135	-0.339174	-1.449188
Si	3.226923	-0.421181	-3.138625
C	1.904117	-1.736718	-3.223339
H	2.191697	-2.631425	-2.662781
H	0.962550	-1.355747	-2.815167
H	1.728632	-2.027757	-4.266574
C	4.836039	-0.958887	-3.957521
H	5.189121	-1.915318	-3.556001
H	4.693885	-1.082504	-5.038351
H	5.631828	-0.218450	-3.812968
C	2.628869	1.216245	-3.831147
H	1.724251	1.556680	-3.316203
H	3.388468	2.005193	-3.806260
H	2.360408	1.060124	-4.884721

TS 301

B3LYP/6-31G(d) = -3573.921355
B3LYP-D3(BJ)/def2-TZVPP/IEFPCM(propanonitrile) = -3575.490484
B3LYP-D3(BJ)/def2-TZVPP/IEFPCM(propanonitrile)//B3LYP-D3(BJ)/6-31G(d) Free Energy (Quasiharmonic) = -3574.775041

Frequencies (Top 3 out of 300)

1. -270.1861 cm^{-1}
2. 6.5203 cm^{-1}
3. 9.1991 cm^{-1}

B3LYP/6-31G(d) Molecular Geometry in Cartesian Coordinates

B	-0.297546	-0.040359	0.780048
O	-0.787390	-0.576202	2.070883
N	-1.410181	-0.507932	-0.187934
C	-2.443247	-1.279505	0.525826
S	-1.570111	-0.139065	-1.788713
C	-1.934188	-1.257108	1.971609
O	-2.480338	-1.794333	2.908557
H	-2.472589	-3.414876	0.906971
H	-3.403817	-0.745974	0.516047
C	-2.682439	-2.756623	0.055797
C	-4.071026	-3.041210	-0.441696
C	-5.267318	-3.174363	0.358974
C	-6.338133	-3.476214	-0.528026
N	-5.809574	-3.517340	-1.802922
C	-4.451129	-3.255955	-1.743529
H	-3.849920	-3.224034	-2.640691
C	-7.879183	-3.568007	1.288705
C	-5.532670	-3.068789	1.736736
H	-8.454322	-3.907551	-0.769829
H	-6.325695	-3.718731	-2.644548
C	-6.833317	-3.266703	2.185305
H	-8.885520	-3.717508	1.671463
H	-4.731821	-2.824412	2.429466
C	-7.647501	-3.676047	-0.078396
H	-7.051626	-3.186203	3.246973
C	-2.863415	1.108092	-1.896920
O	-2.070124	-1.315167	-2.516034
O	-0.308792	0.485288	-2.213930
C	-4.885575	3.048685	-2.011262
C	-4.204514	0.713860	-1.873921
C	-2.521132	2.458298	-1.997230
C	-3.533192	3.414484	-2.056774
C	-5.201977	1.686151	-1.925404
H	-4.462842	-0.339485	-1.836404
H	-1.479520	2.754140	-2.047232
H	-3.266321	4.465145	-2.144563
H	-6.244724	1.378186	-1.906160
C	-5.972771	4.096756	-2.036740
H	-5.660859	4.985339	-2.595654
H	-6.227258	4.424387	-1.019924
H	-6.890911	3.711926	-2.492847
H	-1.954153	-2.980960	-0.724782
C	0.022466	1.544725	0.965259
C	-0.267325	2.167742	2.192065
C	0.583717	2.356791	-0.034565
C	-0.023614	3.528142	2.407643
H	-0.690198	1.575224	2.996539
C	0.815716	3.720985	0.172373
H	0.805691	1.918161	-0.999841
C	0.516782	4.319419	1.396083
H	0.698093	5.375281	1.557095
C	1.425723	4.532261	-0.933126
C	-0.402461	4.148823	3.725379
F	2.773091	4.322698	-1.037501
F	1.259944	5.859104	-0.763403
F	0.909503	4.210058	-2.145141
F	-1.724380	4.431034	3.787304
F	0.257340	5.310047	3.947904
F	-0.130335	3.326636	4.763584
O	0.969852	-0.839120	0.411169
C	1.839274	-1.180184	1.328496
C	2.373771	-2.568548	1.262309
C	2.135364	-3.365931	0.133862
C	3.067319	-3.108449	2.354627
C	2.590101	-4.683889	0.101657
H	1.565330	-2.950462	-0.690611
C	3.519159	-4.426225	2.321131
H	3.239355	-2.499194	3.239155
C	3.283562	-5.216555	1.192168
H	2.385855	-5.302963	-0.767759
H	4.040345	-4.841720	3.179221
H	3.622820	-6.248785	1.170853
H	1.635013	-0.829800	2.344786
C	3.411976	0.037564	1.183056
H	3.858542	-0.087125	2.165691
H	2.828886	0.939325	1.035867
C	4.166143	-0.440617	0.103491
C	3.879427	-0.081255	-1.288838
C	3.926242	-1.062181	-2.299112
C	3.523507	1.236332	-1.625996
C	3.607536	-0.728933	-3.611120
H	4.153338	-2.089915	-2.037498
C	3.225435	1.567940	-2.944244
H	3.502605	2.007124	-0.865046
C	3.260457	0.585868	-3.935902
H	3.613073	-1.496206	-4.379700
H	2.948179	2.588500	-3.185990
H	3.007102	0.841331	-4.960645
O	5.053101	-1.383087	0.362084
Si	6.639508	-1.863996	-0.131076
C	7.402047	-0.520846	-1.199757
H	6.881756	-0.400610	-2.154805
H	7.404423	0.449767	-0.690550
H	8.446227	-0.779260	-1.417178
C	6.485053	-3.511759	-1.016015
H	7.465763	-4.000314	-1.072412
H	5.807663	-4.182326	-0.475451
H	6.111480	-3.404016	-2.039668
C	7.545155	-2.056632	1.500300
H	8.571296	-2.409117	1.339481
H	7.600140	-1.106641	2.043784
H	7.038021	-2.783536	2.144412

TS 302

B3LYP/6-31G(d) = -3573.922426
B3LYP-D3(BJ)/def2-TZVPP/IEFPCM(propanonitrile) = -3575.490328
B3LYP-D3(BJ)/def2-TZVPP/IEFPCM(propanonitrile)//B3LYP-D3(BJ)/6-31G(d) Free Energy (Quasiharmonic) = -3574.774929

Frequencies (Top 3 out of 300)

1. -248.4081 cm^{-1}
2. 6.3999 cm^{-1}
3. 11.0787 cm^{-1}

B3LYP/6-31G(d) Molecular Geometry in Cartesian Coordinates

B	-0.514489	-0.300862	1.323169
O	0.258391	-0.673026	2.530783
N	0.614650	-0.236806	0.253027
C	1.937896	-0.430772	0.877910
S	0.467460	-0.474880	-1.378801
C	1.579858	-0.662506	2.353200
O	2.390471	-0.812638	3.240366
H	3.175086	1.161844	1.687970
H	2.400562	-1.359931	0.519730
C	2.963287	0.737878	0.698795
C	4.257671	0.351481	0.041070
C	5.339098	-0.415532	0.616735
C	6.360962	-0.507349	-0.368575
N	5.910507	0.165783	-1.487385
C	4.650700	0.679783	-1.232848
H	4.113204	1.233360	-1.989996
C	7.736141	-1.775252	1.108237

C	5.539092	-1.030265	1.866727
H	8.336409	-1.240792	-0.901039
H	6.427841	0.289521	-2.343200
C	6.733827	-1.701314	2.097597
H	8.659558	-2.307429	1.321382
H	4.762401	-0.989303	2.625313
C	7.564453	-1.182033	-0.137480
H	6.900389	-2.182002	3.057883
C	1.041386	-2.140958	-1.736762
O	1.393650	0.443155	-2.075551
O	-0.963734	-0.409775	-1.705291
C	1.925536	-4.755402	-2.221378
C	2.383864	-2.368599	-2.046628
C	0.131938	-3.201425	-1.677165
C	0.580655	-4.497315	-1.914096
C	2.813479	-3.674433	-2.285330
H	3.078562	-1.537721	-2.101678
H	-0.910768	-3.012591	-1.452032
H	-0.126400	-5.319146	-1.845485
H	3.859911	-3.853436	-2.519358
C	2.393196	-6.165066	-2.493612
H	2.090577	-6.494174	-3.496418
H	3.483050	-6.244866	-2.434757
H	1.960544	-6.873173	-1.778328
H	2.479034	1.514322	0.100894
C	-1.779151	-1.270786	1.116580
C	-1.624545	-2.660346	1.204628
C	-3.073329	-0.783365	0.888201
C	-2.707067	-3.528988	1.029426
C	-4.163739	-1.642281	0.741256
H	-3.230141	0.286443	0.819344
C	-3.986064	-3.025402	0.799130
H	-4.827764	-3.696748	0.670798
C	-5.554483	-1.094582	0.582437
C	-2.478339	-5.013294	0.995245
F	-6.229866	-1.085671	1.756606
F	-6.292540	-1.828290	-0.281628
F	-5.558573	0.184151	0.128196
F	-3.570221	-5.711271	1.377142
F	-1.452977	-5.395397	1.783976
F	-2.175851	-5.435755	-0.266408
O	-1.103207	1.098342	1.645378
C	-0.458999	2.222750	1.621290
C	-0.316918	2.983604	2.884169
C	0.613665	4.032760	2.964698
C	-1.062796	2.627490	4.017258
C	0.792029	4.719112	4.162139
H	1.198907	4.302596	2.088335
C	-0.882799	3.319632	5.212513
H	-1.756623	1.796491	3.951952
C	0.040475	4.365813	5.287278
H	1.520842	5.522345	4.222988
H	-1.455422	3.035863	6.090851
H	0.182506	4.899170	6.223138
H	0.374941	2.304697	0.918641
C	-1.771525	3.578838	0.639819
H	-1.473755	4.484508	1.150143
H	-2.674163	3.090073	0.989788
C	-1.423809	3.447680	-0.700332
C	-0.379021	4.305468	-1.307263
C	0.625421	3.737912	-2.112343
C	-0.360635	5.689051	-1.052190
C	1.626411	4.548451	-2.646287
H	0.651492	2.665187	-2.276694
C	0.631390	6.493427	-1.607503
H	-1.142881	6.135248	-0.445164
C	1.628539	5.923786	-2.403575
H	2.409638	4.100007	-3.250788
H	0.625625	7.563544	-1.420983
H	2.406468	6.550887	-2.830363
O	-1.936387	2.454902	-1.393307
Si	-2.633745	2.278006	-2.981776
C	-3.779242	0.812458	-2.818343
H	-4.491607	0.933656	-1.996815
H	-4.349291	0.671561	-3.745430
H	-3.196206	-0.092837	-2.627690
C	-1.323356	2.000255	-4.295609
H	-1.816172	1.685697	-5.224977
H	-0.745051	2.903574	-4.513801
H	-0.633114	1.205973	-3.996422
H	-0.645132	-3.080395	1.412649
C	-3.568047	3.888285	-3.280711
H	-4.328569	4.061476	-2.510559
H	-4.082497	3.848659	-4.248920
H	-2.898410	4.755814	-3.300411

TS 303

B3LYP/6-31G(d) = -3573.919294
B3LYP-D3(BJ)/def2-TZVPP/IEFPCM(propanonitrile) = -3575.491237
B3LYP-D3(BJ)/def2-TZVPP/IEFPCM(propanonitrile)//B3LYP-D3(BJ)/6-31G(d) Free Energy (Quasiharmonic) = -3574.774828

Frequencies (Top 3 out of 300)

1. -285.8442 cm^{-1}
2. 9.8963 cm^{-1}
3. 11.9663 cm^{-1}

B3LYP/6-31G(d) Molecular Geometry in Cartesian Coordinates

B	0.291021	0.521474	-0.514313
O	0.429259	0.784835	-1.967868
N	0.186889	-1.049351	-0.490382
C	0.324142	-1.573719	-1.859031
S	0.757003	-1.963217	0.768655
C	0.566099	-0.319380	-2.699820
O	0.807956	-0.322019	-3.889986
H	-1.039468	-3.254562	-1.818758
H	1.209925	-2.214712	-1.941754
C	-0.887406	-2.363290	-2.435438
C	-2.180970	-1.598883	-2.552179
C	-3.402593	-1.835983	-1.818920
C	-4.347301	-0.860095	-2.241463
N	-3.736405	-0.096251	-3.214229
C	-2.437094	-0.534883	-3.386459
H	-1.789999	-0.058058	-4.109275
C	-5.998226	-1.730066	-0.759288
C	-3.801446	-2.785358	-0.862056
H	-6.334726	-0.013229	-2.032502
H	-4.098007	0.774660	-3.571749
C	-5.089025	-2.725018	-0.344540
H	-6.995211	-1.701943	-0.327583
H	-3.113068	-3.555190	-0.527192
C	-5.640826	-0.786064	-1.714122
H	-5.402537	-3.453547	0.398905
C	-0.504173	-3.178372	1.185932
O	0.881219	-1.093580	1.946579
O	1.936134	-2.746384	0.328092
C	-2.387768	-5.097143	1.977180
C	-0.375491	-4.495430	0.744641
C	-1.556488	-2.806216	2.026842
C	-2.485263	-3.767754	2.415589
C	-1.320386	-5.443644	1.138411
H	0.470094	-4.775804	0.125758
H	-1.641505	-1.786852	2.387587
H	-3.300254	-3.479362	3.074772
H	-1.216654	-6.471813	0.799898
C	-3.417905	-6.122165	2.388918
H	-4.318658	-6.048205	1.765368
H	-3.032744	-7.141799	2.287415

H	-3.732105	-5.978345	3.428385
H	-0.561612	-2.717181	-3.421934
C	-0.989137	1.264302	0.128570
C	-1.959634	1.860722	-0.692996
C	-1.239964	1.247470	1.505537
C	-3.135512	2.394047	-0.166263
H	-1.798071	1.888862	-1.763748
C	-2.424076	1.770953	2.038020
H	-0.516714	0.787945	2.172660
C	-3.381343	2.347342	1.208954
H	-4.302008	2.744370	1.621542
C	-2.657850	1.642466	3.516065
C	-4.169178	3.027436	-1.053389
F	-2.771315	0.340280	3.888338
F	-3.783636	2.268185	3.923925
F	-1.631162	2.153927	4.235469
F	-4.329258	4.345043	-0.784525
F	-3.857529	2.935095	-2.373117
F	-5.388433	2.456752	-0.899774
O	1.600762	0.892216	0.150334
C	2.167263	1.981711	0.582020
C	1.634374	3.352145	0.342994
C	1.050374	3.741434	-0.873506
C	1.755306	4.289496	1.380452
C	0.583989	5.043662	-1.033805
H	0.961357	3.023856	-1.682621
C	1.280244	5.589597	1.217903
H	2.206520	3.991253	2.324238
C	0.694890	5.968774	0.008545
H	0.129256	5.338438	-1.975314
H	1.363481	6.302479	2.033364
H	0.325307	6.982099	-0.122456
H	2.622803	1.857876	1.569367
C	3.955100	2.159841	-0.339305
H	3.648493	2.513393	-1.318224
H	4.378918	2.900764	0.329572
C	4.446836	0.853200	-0.267851
C	5.113805	0.331455	0.938578
C	6.077912	1.100455	1.616636
C	4.741131	-0.926731	1.449682
C	6.680296	0.607538	2.770559
H	6.376994	2.065556	1.217806
C	5.333089	-1.399522	2.619236
H	3.953724	-1.498730	0.968585
C	6.306576	-0.641902	3.274333
H	7.439487	1.196188	3.277495
H	5.021306	-2.357668	3.024043
H	6.769792	-1.020128	4.181493
O	4.126049	0.027488	-1.238616
Si	5.013268	-1.142438	-2.185939
C	4.285592	-0.937396	-3.897102
H	4.698379	-1.691974	-4.578629
H	3.194420	-1.032972	-3.906045
H	4.527526	0.049502	-4.308136
C	4.773789	-2.865771	-1.491771
H	5.410370	-3.042477	-0.618162
H	3.737052	-3.040225	-1.186651
H	5.039967	-3.607458	-2.255732
C	6.818251	-0.608314	-2.130220
H	7.416501	-1.270605	-2.768547
H	6.950353	0.413975	-2.503263
H	7.235987	-0.660586	-1.119065

TS 304

B3LYP/6-31G(d) = -3573.920132
B3LYP-D3(BJ)/def2-TZVPP/IEFPCM(propanonitrile) = -3575.490513
B3LYP-D3(BJ)/def2-TZVPP/IEFPCM(propanonitrile)//B3LYP-D3(BJ)/6-31G(d) Free Energy (Quasiharmonic) = -3574.774803

Frequencies (Top 3 out of 300)

1. -289.7128 cm^{-1}
2. 6.5598 cm^{-1}
3. 9.0768 cm^{-1}

B3LYP/6-31G(d) Molecular Geometry in Cartesian Coordinates

B	0.502361	-0.013228	-1.280443
O	0.860212	-0.889618	-2.406654
N	1.701979	0.997475	-1.234702
C	2.601259	0.713108	-2.371603
S	1.414069	2.589662	-0.845709
C	1.945450	-0.479200	-3.076369
O	2.358558	-0.996157	-4.088031
H	4.538981	1.299223	-1.617632
H	2.606185	1.545534	-3.086573
C	4.073024	0.382392	-1.992637
C	4.252436	-0.725408	-0.992726
C	4.397931	-2.142973	-1.245726
C	4.526911	-2.783376	0.018820
N	4.484246	-1.798278	0.985235
C	4.301698	-0.574596	0.371105
H	4.243501	0.329049	0.961672
C	4.723312	-4.919691	-1.021019
C	4.443707	-2.932824	-2.409328
H	4.778778	-4.635994	1.123564
H	4.371724	-1.955006	1.975055
C	4.605905	-4.306933	-2.285506
H	4.844981	-5.997932	-0.957639
H	4.323742	-2.477074	-3.387093
C	4.685598	-4.167274	0.146936
H	4.635792	-4.924287	-3.179432
C	2.927433	3.188495	-0.072855
O	0.386325	2.585453	0.218687
O	1.212524	3.423232	-2.042729
C	5.267242	4.163720	1.128783
C	3.150524	2.965992	1.288524
C	3.843398	3.917049	-0.832068
C	5.006516	4.394587	-0.228244
C	4.316643	3.452158	1.876287
H	2.412619	2.441753	1.886389
H	3.633834	4.121124	-1.876724
H	5.718686	4.963579	-0.820921
H	4.486505	3.284714	2.937257
C	6.542976	4.655864	1.769326
H	7.318138	3.878405	1.747799
H	6.386141	4.928350	2.818318
H	6.943519	5.529902	1.245920
H	4.579138	0.133847	-2.933116
C	0.288609	-0.871550	0.076882
C	0.920206	-0.493801	1.267975
C	-0.459601	-2.060471	0.107456
C	0.813868	-1.252669	2.436631
H	1.519565	0.405457	1.274191
C	-0.593761	-2.811757	1.278935
H	-0.940801	-2.420471	-0.795452
C	0.047451	-2.416029	2.454600
H	-0.044770	-3.006457	3.359679
C	-1.479092	-4.025298	1.319350
C	1.585025	-0.843844	3.658667
F	-0.912545	-5.050194	1.990876
F	-1.808476	-4.470972	0.089758
F	-2.653339	-3.753493	1.961298
F	1.010798	-1.278802	4.799655
F	1.717270	0.498992	3.756761
F	2.854612	-1.348911	3.654683
O	-0.718913	0.799267	-1.696773
C	-2.011550	0.875778	-1.708287
C	-2.857937	-0.114144	-2.426004
C	-2.317632	-1.281424	-2.989905

C	-4.211071	0.195588	-2.651527
C	-3.130112	-2.134471	-3.736459
H	-1.257109	-1.488476	-2.894386
C	-5.014794	-0.655334	-3.405434
H	-4.628188	1.114967	-2.249794
C	-4.477222	-1.829185	-3.942019
H	-2.701259	-3.030046	-4.176773
H	-6.055659	-0.399292	-3.583693
H	-5.102177	-2.492863	-4.533363
H	-2.362845	1.906614	-1.806011
C	-2.526463	0.795761	0.287147
H	-2.464116	-0.270249	0.469940
H	-1.637153	1.357563	0.547978
C	-3.768435	1.409032	0.451470
C	-3.962796	2.866395	0.394979
C	-2.869816	3.748002	0.248899
C	-5.264649	3.401222	0.476733
C	-3.085802	5.121887	0.186222
H	-1.850403	3.378496	0.191076
C	-5.469301	4.775031	0.413656
H	-6.106137	2.727358	0.587496
C	-4.380603	5.639512	0.267485
H	-2.235686	5.787783	0.072826
H	-6.477810	5.173683	0.477685
H	-4.541500	6.712984	0.218048
O	-4.888543	0.703512	0.554278
Si	-5.269009	-0.828459	1.288298
C	-4.917385	-2.235064	0.101861
H	-5.470529	-2.111940	-0.834556
H	-3.857491	-2.335650	-0.146664
H	-5.229483	-3.181455	0.560879
C	-7.112934	-0.646596	1.589729
H	-7.651067	-0.455911	0.654212
H	-7.525507	-1.563271	2.028279
H	-7.328727	0.178539	2.277713
C	-4.311374	-0.957538	2.899472
H	-3.248722	-1.167471	2.748523
H	-4.403338	-0.042199	3.495161
H	-4.720674	-1.783113	3.495036

TS 305

B3LYP/6-31G(d) = -3573.918813
B3LYP-D3(BJ)/def2-TZVPP/IEFPCM(propanonitrile) = -3575.490321
B3LYP-D3(BJ)/def2-TZVPP/IEFPCM(propanonitrile)//B3LYP-D3(BJ)/6-31G(d) Free Energy (Quasiharmonic) = -3574.774449

Frequencies (Top 3 out of 300)

1. -258.2166 cm^{-1}
2. 7.5521 cm^{-1}
3. 11.3131 cm^{-1}

B3LYP/6-31G(d) Molecular Geometry in Cartesian Coordinates

B	0.110134	0.527511	-1.027308
O	0.007287	0.515359	-2.497242
N	0.243335	-1.014118	-0.725803
C	0.197657	-1.779153	-1.984481
S	0.957945	-1.690697	0.587246
C	0.181405	-0.682756	-3.047941
O	0.263561	-0.884200	-4.242098
H	-1.005726	-3.483611	-1.400203
H	1.109585	-2.373181	-2.105529
C	-1.017182	-2.731009	-2.195242
C	-2.376369	-2.080967	-2.263118
C	-3.479100	-2.260318	-1.346822
C	-4.547870	-1.439714	-1.804219
N	-4.123161	-0.827479	-2.964652
C	-2.821621	-1.206699	-3.228658
H	-2.308933	-0.828655	-4.101836
C	-5.936673	-2.133352	0.003785
C	-3.683386	-3.046353	-0.199265
H	-6.562286	-0.696861	-1.488980
H	-4.600403	-0.065712	-3.421958
C	-4.903989	-2.977906	0.460417
H	-6.876432	-2.093195	0.548209
H	-2.898701	-3.698888	0.171428
C	-5.773085	-1.354197	-1.134936
H	-5.067301	-3.583678	1.348110
C	-0.226772	-2.706976	1.485383
O	2.016499	-2.626136	0.146205
O	1.315207	-0.588649	1.502287
C	-1.955302	-4.304429	3.009289
C	-1.170026	-2.103293	2.321016
C	-0.135739	-4.096826	1.402935
C	-1.003680	-4.884406	2.160823
C	-2.024188	-2.904164	3.072236
H	-1.237246	-1.025264	2.397576
H	0.619672	-4.549863	0.770452
H	-0.930790	-5.967489	2.098103
H	-2.757233	-2.428260	3.718609
C	-2.870291	-5.158204	3.854610
H	-3.899684	-4.783370	3.832288
H	-2.879792	-6.197590	3.512222
H	-2.550646	-5.158397	4.904941
H	-0.803465	-3.264570	-3.130305
C	-1.162745	1.243127	-0.332101
C	-2.249338	1.655940	-1.121656
C	-1.271593	1.435227	1.049576
C	-3.402587	2.195565	-0.552819
C	-2.427785	1.974199	1.626949
H	-0.452666	1.141323	1.700539
C	-3.504582	2.353659	0.832045
H	-4.405019	2.759190	1.279371
C	-2.499542	2.077558	3.123475
C	-4.566125	2.618620	-1.405012
F	-1.413200	2.699640	3.638377
F	-2.542009	0.847505	3.705740
F	-3.589319	2.749959	3.549851
F	-4.399655	2.302698	-2.715226
F	-4.775530	3.954646	-1.350002
F	-5.721671	2.033840	-1.005609
O	1.362733	1.383561	-0.808048
C	1.940871	1.969594	0.193071
C	1.760084	3.433749	0.355000
C	1.255911	4.216619	-0.694275
C	2.053850	4.033248	1.589770
C	1.053978	5.581833	-0.508043
H	1.013947	3.739490	-1.638582
C	1.846483	5.397999	1.773357
H	2.430965	3.422916	2.407509
C	1.349799	6.174934	0.722964
H	0.657622	6.184592	-1.320351
H	2.063132	5.854609	2.734973
H	1.186041	7.239502	0.866233
H	2.072483	1.391298	1.108393
C	3.960024	2.032842	-0.356136
H	4.329164	2.720598	0.393995
H	3.736885	2.446546	-1.333035
C	4.373520	0.705964	-0.293830
C	5.061378	0.142610	0.884107
C	5.991161	0.918976	1.602363
C	4.793316	-1.173478	1.303859
C	6.645245	0.387501	2.709938
H	6.226445	1.925469	1.269811
C	5.440946	-1.691544	2.423167
H	4.041407	-1.760334	0.787941
C	6.369161	-0.918402	3.124170
H	7.373128	0.988968	3.247012
H	5.210011	-2.699890	2.754250

H	6.875525	-1.330345	3.992867
O	4.028661	-0.097438	-1.284152
Si	4.885622	-1.120779	-2.390415
C	4.808524	-2.902868	-1.812612
H	3.798051	-3.166980	-1.484864
H	5.092756	-3.572586	-2.634168
H	5.491005	-3.089657	-0.976486
C	6.656157	-0.480316	-2.444938
H	7.243880	-1.067861	-3.161152
H	6.698726	0.567252	-2.764882
H	7.150539	-0.559110	-1.470250
H	-2.193722	1.531508	-2.196008
C	4.007328	-0.861168	-4.023170
H	4.472432	-1.475171	-4.804985
H	2.945011	-1.124862	-3.984423
H	4.071433	0.185627	-4.341244

TS 306

B3LYP/6-31G(d) = -3573.914751
B3LYP-D3(BJ)/def2-TZVPP/IEFPCM(propanonitrile) = -3575.490924
B3LYP-D3(BJ)/def2-TZVPP/IEFPCM(propanonitrile)//B3LYP-D3(BJ)/6-31G(d) Free Energy (Quasiharmonic) = -3574.774435

Frequencies (Top 3 out of 300)

1. -297.3513 cm^{-1}
2. 10.4465 cm^{-1}
3. 13.2293 cm^{-1}

B3LYP/6-31G(d) Molecular Geometry in Cartesian Coordinates

B	-0.030049	-0.782860	0.100778
O	-0.633877	-1.057355	1.421303
N	1.142802	-1.815019	0.060326
C	1.214134	-2.531382	1.345761
S	1.640138	-2.597152	-1.320262
C	-0.013640	-2.026861	2.107110
O	-0.370047	-2.414303	3.197283
H	3.355895	-2.656695	1.635670
H	1.100082	-3.611450	1.190961
C	2.490614	-2.310295	2.208836
C	2.725135	-0.903477	2.693452
C	3.758294	0.010469	2.265598
C	3.573561	1.224677	2.983160
N	2.502411	1.037213	3.830870
C	1.991871	-0.232945	3.645859
H	1.140172	-0.572015	4.218587
C	5.439861	2.224411	1.888560
C	4.833115	-0.079260	1.364082
H	4.218587	3.265565	3.341056
H	2.050902	1.770021	4.355223
C	5.660366	1.022041	1.185144
H	6.098857	3.072091	1.719959
H	5.016236	-0.995518	0.812062
C	4.395494	2.341323	2.797699
H	6.491048	0.959688	0.486902
C	3.437608	-2.504069	-1.389215
O	1.149351	-1.811698	-2.460571
O	1.313575	-4.030981	-1.205181
C	6.229737	-2.415055	-1.630576
C	4.204695	-3.584687	-0.951016
C	4.046179	-1.382238	-1.956661
C	5.434057	-1.347894	-2.072625
C	5.593461	-3.528684	-1.065049
H	3.714206	-4.464330	-0.547883
H	3.445473	-0.552116	-2.311983
H	5.907440	-0.475149	-2.515779
H	6.190786	-4.370034	-0.721719
C	7.730933	-2.380249	-1.793144
H	8.231269	-2.983236	-1.028453
H	8.117849	-1.358004	-1.727895
H	8.030056	-2.779480	-2.771516
H	2.386316	-2.992325	3.062014
C	0.416254	0.766304	-0.058245
C	0.937197	1.239711	-1.275132
C	0.289181	1.690896	0.985155
C	1.334296	2.568033	-1.429892
H	1.032040	0.550398	-2.109084
C	0.673204	3.030089	0.829104
H	-0.116279	1.361223	1.936974
C	1.204761	3.477660	-0.377613
H	1.509367	4.509941	-0.499222
C	0.482416	3.979966	1.976252
C	1.831506	3.034775	-2.768591
F	0.705202	5.265421	1.633278
F	1.306204	3.709044	3.025650
F	-0.781575	3.908777	2.468154
F	0.802414	3.331894	-3.609415
F	2.573983	2.091977	-3.391307
F	2.586709	4.151534	-2.677551
O	-1.079760	-1.009891	-0.995225
C	-2.361991	-1.258898	-1.007473
C	-2.912525	-2.587131	-0.622329
C	-2.093620	-3.607806	-0.118018
C	-4.261047	-2.872753	-0.900596
C	-2.622729	-4.870888	0.139418
H	-1.033687	-3.436742	0.006514
C	-4.787438	-4.135202	-0.643455
H	-4.896926	-2.106075	-1.333928
C	-3.970308	-5.135657	-0.110346
H	-1.972129	-5.653907	0.517257
H	-5.831139	-4.340243	-0.864471
H	-4.378172	-6.122915	0.088886
H	-2.852784	-0.838434	-1.890453
C	-3.121302	0.114089	0.276196
H	-2.359177	0.845314	0.031479
H	-2.930836	-0.409466	1.203046
C	-4.435611	0.465947	-0.051835
C	-5.628323	-0.147123	0.549350
C	-5.527779	-1.040490	1.635357
C	-6.907673	0.170519	0.048892
C	-6.672124	-1.596627	2.198435
H	-4.560000	-1.300365	2.047719
C	-8.047062	-0.389755	0.614961
H	-6.991737	0.856827	-0.785372
C	-7.933075	-1.274265	1.691722
H	-6.578048	-2.281200	3.035969
H	-9.026224	-0.136803	0.218617
H	-8.824632	-1.708130	2.135968
O	-4.697622	1.346605	-1.007198
Si	-3.791787	2.636157	-1.760505
C	-5.127045	3.476907	-2.774743
H	-5.575023	2.783769	-3.495761
H	-5.928389	3.870957	-2.139833
H	-4.706784	4.316698	-3.341317
C	-2.448483	1.922925	-2.856772
H	-1.769959	1.243901	-2.331648
H	-2.880727	1.382899	-3.707408
H	-1.824923	2.730308	-3.260563
C	-3.159501	3.760875	-0.396140
H	-3.984388	4.098855	0.241945
H	-2.400202	3.305509	0.246777
H	-2.707171	4.653475	-0.846698

TS 307

B3LYP/6-31G(d) = -3573.922438
B3LYP-D3(BJ)/def2-TZVPP/IEFPCM(propanonitrile) = -3575.491328

B3LYP-D3(BJ)/def2-TZVPP/IEFPCM(propanonitrile)//B3LYP-D3(BJ)/6-31G(d) Free Energy (Quasiharmonic) = -3574.774396

Frequencies (Top 3 out of 300)

1. -287.1140 cm^{-1}
2. 8.7592 cm^{-1}
3. 9.7052 cm^{-1}

B3LYP/6-31G(d) Molecular Geometry in Cartesian Coordinates

B	0.127300	0.337730	-0.365640
O	0.036440	0.374030	-1.843240
N	0.206110	-1.211140	-0.104140
C	0.218130	-1.932900	-1.387200
S	0.936780	-1.915890	1.200450
C	0.253530	-0.811740	-2.422720
O	0.434410	-0.964830	-3.611310
H	-0.961760	-3.668550	-0.885000
H	1.134780	-2.520170	-1.487780
C	-0.986640	-2.883140	-1.647770
C	-2.337870	-2.228160	-1.678560
C	-3.038860	-1.720960	-2.838470
C	-4.265290	-1.168540	-2.376980
N	-4.315130	-1.361380	-1.010340
C	-3.147310	-1.973440	-0.598430
H	-2.983520	-2.191550	0.447710
C	-4.882440	-0.542090	-4.591620
C	-2.757130	-1.680270	-4.216470
H	-6.108190	-0.128520	-2.854800
H	-4.969710	-0.899180	-0.397910
C	-3.679650	-1.097340	-5.075880
H	-5.577900	-0.081780	-5.288600
H	-1.817830	-2.069330	-4.597350
C	-5.190830	-0.567620	-3.236940
H	-3.466950	-1.056050	-6.140920
C	-0.289820	-2.989860	1.968970
O	1.240910	-0.871970	2.189330
O	2.024580	-2.806910	0.738360
C	-2.194430	-4.652650	3.183210
C	-0.250100	-4.365560	1.745280
C	-1.256780	-2.432450	2.811410
C	-2.198950	-3.266830	3.408140
C	-1.202510	-5.185980	2.351320
H	0.530440	-4.785270	1.119750
H	-1.261260	-1.367300	3.017820
H	-2.945120	-2.834400	4.070700
H	-1.167780	-6.259100	2.179390
C	-3.240780	-5.539360	3.815260
H	-3.478000	-5.215320	4.834420
H	-2.910630	-6.582090	3.857720
H	-4.176760	-5.514730	3.241620
H	-0.787030	-3.373810	-2.608180
C	-1.102100	1.078220	0.375620
C	-2.290280	1.400830	-0.300090
C	-1.049460	1.352690	1.749010
C	-3.377080	1.967490	0.371770
H	-2.368600	1.200660	-1.363640
C	-2.143140	1.899810	2.428510
H	-0.148180	1.110290	2.303980
C	-3.313810	2.216530	1.745140
H	-4.157200	2.653080	2.267660
C	-2.053290	2.071640	3.917640
C	-4.660910	2.260650	-0.351260
F	-0.899460	2.668450	4.292440
F	-2.086370	0.869050	4.553350
F	-3.069690	2.806820	4.420280
F	-5.590550	1.271350	-0.150110
F	-4.501350	2.369190	-1.682140
F	-5.243160	3.399500	0.087290
O	1.452900	0.933350	0.057690
C	2.021390	2.103920	-0.041670
C	1.344450	3.353730	-0.507980
C	1.623600	4.526930	0.212300
C	0.484480	3.418750	-1.616300
C	1.037680	5.738820	-0.147990
H	2.290310	4.484440	1.070810
C	-0.094420	4.634070	-1.975870
H	0.270580	2.522800	-2.187690
C	0.176720	5.794060	-1.245420
H	1.250310	6.635110	0.428000
H	-0.761950	4.674180	-2.831960
H	-0.280830	6.737220	-1.531470
H	2.695870	2.296150	0.798210
C	3.484970	1.958990	-1.379030
H	2.931720	1.855110	-2.307580
H	3.941630	2.925320	-1.193820
C	4.164180	0.827710	-0.912940
C	5.093980	0.911340	0.231410
C	6.098470	1.894310	0.277960
C	4.917260	0.041800	1.324350
C	6.937170	1.983620	1.386010
H	6.237750	2.563770	-0.566230
C	5.743960	0.158160	2.440230
H	4.105740	-0.678880	1.314890
C	6.758810	1.117700	2.468660
H	7.726670	2.729290	1.407240
H	5.588020	-0.498120	3.291220
H	7.406440	1.196850	3.337420
O	3.800650	-0.349820	-1.365670
Si	4.725590	-1.747700	-1.884700
C	6.186470	-1.017340	-2.822390
H	5.854170	-0.401190	-3.665770
H	6.822480	-0.400440	-2.177290
H	6.812220	-1.822470	-3.226790
C	3.558920	-2.679490	-3.014390
H	2.845730	-2.020020	-3.520090
H	4.132470	-3.213650	-3.782450
H	2.986270	-3.425140	-2.453060
C	5.268370	-2.805540	-0.437380
H	5.690430	-3.741190	-0.828320
H	6.031630	-2.327830	0.184150
H	4.407700	-3.060480	0.190100

TS 308

B3LYP/6-31G(d) = -3573.914743
B3LYP-D3(BJ)/def2-TZVPP/IEFPCM(propanonitrile) = -3575.490319
B3LYP-D3(BJ)/def2-TZVPP/IEFPCM(propanonitrile)//B3LYP-D3(BJ)/6-31G(d) Free Energy (Quasiharmonic) = -3574.774282

Frequencies (Top 3 out of 300)

1. -294.5359 cm^{-1}
2. 9.8699 cm^{-1}
3. 13.6774 cm^{-1}

B3LYP/6-31G(d) Molecular Geometry in Cartesian Coordinates

B	-0.802756	-0.862493	0.489025
O	-0.900720	-1.900921	1.532806
N	-1.136925	-1.691411	-0.788502
C	-1.340179	-3.105964	-0.452758
S	-1.514449	-1.138984	-2.290785
C	-1.232560	-3.111888	1.074195
O	-1.399386	-4.085055	1.774027
H	-0.379016	-4.061313	-2.133546
H	-2.355406	-3.432052	-0.721403
C	-0.342803	-4.141422	-1.045139
C	1.076728	-4.051211	-0.553504
C	2.196128	-3.369270	-1.160796

C	3.337202	-3.617068	-0.342668
N	2.917336	-4.398356	0.712819
C	1.566729	-4.658873	0.577290
H	1.041228	-5.251247	1.313004
C	4.734112	-2.371469	-1.823038
C	2.353517	-2.592896	-2.322899
H	5.473528	-3.352772	-0.038915
H	3.502334	-4.749630	1.454309
C	3.616060	-2.103970	-2.641801
H	5.713206	-1.999755	-2.114259
H	1.498105	-2.382443	-2.958280
C	4.609346	-3.127210	-0.659963
H	3.748670	-1.515839	-3.546202
C	-3.309866	-1.086299	-2.417685
O	-1.069571	-2.126790	-3.288244
O	-1.033670	0.250352	-2.377947
C	-6.110776	-1.037997	-2.569971
C	-4.003970	-2.235485	-2.805473
C	-3.997838	0.094939	-2.128622
C	-5.388477	0.108606	-2.209383
C	-5.396032	-2.204029	-2.873864
H	-3.457318	-3.130464	-3.082678
H	-3.453164	0.995453	-1.869238
H	-5.922314	1.031176	-1.994001
H	-5.934097	-3.097945	-3.180200
C	-7.620050	-1.017452	-2.617617
H	-8.010737	-1.778077	-3.301313
H	-8.047482	-1.218125	-1.626173
H	-7.997767	-0.041727	-2.941291
H	-0.751574	-5.121775	-0.773634
C	-1.760721	0.391414	0.875265
C	-1.680886	1.650304	0.259834
C	-2.734255	0.241098	1.878208
C	-2.537058	2.697752	0.613585
H	-0.966293	1.809977	-0.538627
C	-3.584263	1.288838	2.244844
H	-2.827094	-0.714112	2.384936
C	-3.495230	2.527786	1.611051
H	-4.158436	3.339215	1.885648
C	-4.559924	1.094455	3.373663
C	-2.458734	3.984175	-0.157810
F	-3.972513	1.289674	4.580351
F	-5.072977	-0.154709	3.389667
F	-5.598905	1.958310	3.302304
F	-3.023943	3.859580	-1.385732
F	-3.092274	5.002503	0.464775
F	-1.177234	4.371136	-0.361887
O	0.645276	-0.406746	0.295899
C	1.693864	-0.216906	1.058862
C	1.761361	-0.608376	2.502862
C	2.926828	-1.255415	2.938076
C	0.749127	-0.326543	3.432093
C	3.073147	-1.632504	4.273220
H	3.712143	-1.479914	2.220702
C	0.899728	-0.696893	4.764909
H	-0.153805	0.181504	3.113370
C	2.059771	-1.351851	5.189800
H	3.976742	-2.142861	4.596114
H	0.107148	-0.478331	5.474747
H	2.170506	-1.641159	6.231303
H	2.620362	-0.454394	0.531034
C	1.960394	1.751054	1.184764
H	2.289740	1.823891	2.215032
H	0.944441	2.080638	0.996933
C	2.937909	2.144793	0.251681
C	2.634391	2.566012	-1.122596
C	3.338998	3.643506	-1.694908
C	1.610257	1.940747	-1.855130
C	3.017777	4.086851	-2.973702
H	4.099435	4.160334	-1.116987
C	1.315143	2.371818	-3.146757
H	1.066683	1.097884	-1.442765
C	2.011621	3.445960	-3.704478
H	3.545687	4.935762	-3.399011
H	0.529853	1.863188	-3.695969
H	1.766444	3.792174	-4.704783
O	4.190681	2.134547	0.665064
Si	5.797325	1.823550	0.082863
C	5.832068	1.356336	-1.732409
H	6.841061	0.997601	-1.976240
H	5.134078	0.540393	-1.949260
H	5.599626	2.190223	-2.399693
C	6.374314	0.390942	1.148619
H	6.196197	0.583284	2.212108
H	5.853414	-0.533449	0.873270
H	7.449266	0.218533	1.012457
C	6.745627	3.398753	0.462827
H	7.813216	3.257709	0.253615
H	6.401978	4.246705	-0.139935
H	6.647552	3.674176	1.518764

TS 309

B3LYP/6-31G(d) = -3573.915528
B3LYP-D3(BJ)/def2-TZVPP/IEFPCM(propanonitrile) = -3575.490331
B3LYP-D3(BJ)/def2-TZVPP/IEFPCM(propanonitrile)//B3LYP-D3(BJ)/6-31G(d) Free Energy (Quasiharmonic) = -3574.774084

Frequencies (Top 3 out of 300)

1. -297.1334 cm^{-1}
2. 4.3001 cm^{-1}
3. 9.8040 cm^{-1}

B3LYP/6-31G(d) Molecular Geometry in Cartesian Coordinates

B	-0.113019	-0.777838	-0.614364
O	0.113815	-1.115852	-2.029284
N	-1.338808	-1.680966	-0.231366
C	-1.775643	-2.405978	-1.440765
S	-1.342080	-2.527894	1.208612
C	-0.730173	-2.045436	-2.497452
O	-0.679140	-2.492886	-3.621313
H	-3.923703	-2.294461	-1.199939
H	-1.734967	-3.492032	-1.293804
C	-3.190046	-2.022477	-1.964842
C	-3.351522	-0.574740	-2.349798
C	-4.042396	0.460349	-1.616770
C	-3.880238	1.672984	-2.342656
N	-3.155133	1.375669	-3.477322
C	-2.830038	0.033045	-3.468844
H	-2.245052	-0.396095	-4.270513
C	-5.128605	2.885609	-0.713264
C	-4.790115	0.487273	-0.426582
H	-4.246861	3.807576	-2.462606
H	-2.754068	2.064417	-4.094792
C	-5.323729	1.692378	0.012830
H	-5.551287	3.814663	-0.339919
H	-4.945862	-0.422102	0.145308
C	-4.406935	2.891361	-1.900975
H	-5.899496	1.720524	0.934432
C	-3.074698	-2.882599	1.541756
O	-0.892098	-1.595292	2.255814
O	-0.677627	-3.835804	1.063224
C	-5.743207	-3.467700	2.168114
C	-3.595336	-4.140522	1.238644
C	-3.861661	-1.918745	2.176916
C	-5.186403	-2.218784	2.484997
C	-4.926726	-4.421248	1.546976
H	-2.956051	-4.893318	0.789776
H	-3.435613	-0.955062	2.434843

H	-5.798283	-1.472355	2.986212
H	-5.332778	-5.402119	1.311855
C	-7.189987	-3.766828	2.481151
H	-7.854895	-3.329650	1.724593
H	-7.484818	-3.348798	3.449894
H	-7.381445	-4.844256	2.502954
H	-3.377173	-2.670881	-2.829805
C	-0.373286	0.808926	-0.424022
C	-0.845632	1.324357	0.793467
C	-0.191534	1.716049	-1.477291
C	-1.123837	2.684014	0.950116
H	-1.014010	0.643254	1.622238
C	-0.483053	3.077072	-1.329248
H	0.161471	1.349553	-2.436064
C	-0.950125	3.572347	-0.111859
H	-1.180611	4.624445	0.005051
C	-0.364297	3.994824	-2.511693
C	-1.571794	3.185441	2.293356
F	0.659378	3.650011	-3.324523
F	-0.178577	5.282450	-2.149337
F	-1.487208	3.977558	-3.290729
F	-2.457240	2.354452	2.880083
F	-2.137039	4.409373	2.230304
F	-0.520001	3.292985	3.163212
O	1.071487	-1.170254	0.252256
C	2.345177	-1.425776	0.252851
C	3.038926	-2.382203	-0.663397
C	2.789117	-2.508357	-2.040139
C	4.005783	-3.210023	-0.069102
C	3.481458	-3.456923	-2.790050
H	2.046252	-1.880844	-2.516145
C	4.691427	-4.162242	-0.822151
H	4.208020	-3.121056	0.995509
C	4.429822	-4.287958	-2.186363
H	3.268220	-3.553092	-3.850898
H	5.424219	-4.804355	-0.341696
H	4.958856	-5.030914	-2.777178
H	2.722369	-1.504957	1.275924
C	3.198911	0.371568	-0.171897
H	2.342413	0.955801	0.141261
H	3.299714	0.278801	-1.245898
C	4.364166	0.493081	0.594976
C	5.696106	0.057546	0.146227
C	5.913421	-0.445919	-1.151848
C	6.792907	0.153389	1.026671
C	7.186254	-0.840347	-1.551780
H	5.091951	-0.543056	-1.851438
C	8.063323	-0.238551	0.620229
H	6.633225	0.538158	2.026734
C	8.264466	-0.736104	-0.670277
H	7.335463	-1.229895	-2.554258
H	8.898787	-0.156794	1.309529
H	9.257939	-1.040716	-0.987664
O	4.329700	0.927462	1.842929
Si	3.177791	1.811911	2.820632
C	1.817292	0.649976	3.369853
H	1.317617	0.116697	2.555385
H	2.203127	-0.096734	4.073559
H	1.042855	1.228050	3.888807
C	2.601485	3.308931	1.844167
H	3.447459	3.956668	1.586734
H	2.068865	3.059660	0.921997
H	1.904864	3.887692	2.461336
C	4.254865	2.333478	4.266347
H	4.682825	1.464769	4.779363
H	5.079556	2.979424	3.944879
H	3.661519	2.891547	5.000905

TS 310

B3LYP/6-31G(d) = -3573.918972

B3LYP-D3(BJ)/def2-TZVPP/IEFPCM(propanonitrile) = -3575.48982

B3LYP-D3(BJ)/def2-TZVPP/IEFPCM(propanonitrile)//B3LYP-D3(BJ)/6-31G(d) Free Energy (Quasiharmonic) = -3574.773887

Frequencies (Top 3 out of 300)

1. -254.1444 cm^{-1}
2. 9.3544 cm^{-1}
3. 11.6135 cm^{-1}

B3LYP/6-31G(d) Molecular Geometry in Cartesian Coordinates

B	0.085335	0.552265	-1.003232
O	-0.013725	0.607037	-2.471463
N	0.224497	-0.999987	-0.768391
C	0.188873	-1.707177	-2.061492
S	0.966573	-1.724135	0.504392
C	0.162134	-0.565117	-3.075600
O	0.238372	-0.712831	-4.277716
H	-0.985638	-3.458904	-1.566806
H	1.107574	-2.284857	-2.208682
C	-1.016282	-2.659471	-2.314208
C	-2.381434	-2.019758	-2.319759
C	-3.469219	-2.276708	-1.404475
C	-4.549918	-1.432522	-1.783706
N	-4.146296	-0.731104	-2.899348
C	-2.846823	-1.079064	-3.210545
H	-2.350732	-0.633943	-4.061442
C	-5.909518	-2.276112	-0.017318
C	-3.651799	-3.150177	-0.318126
H	-6.567075	-0.737353	-1.390178
H	-4.643617	0.053219	-3.291956
C	-4.863961	-3.143639	0.360420
H	-6.842812	-2.287334	0.539541
H	-2.857043	-3.822419	-0.008980
C	-5.766737	-1.410063	-1.094422
H	-5.010891	-3.817545	1.200633
C	-0.182576	-2.828413	1.343272
O	2.054634	-2.598787	0.013086
O	1.290058	-0.663138	1.478723
C	-1.848506	-4.570175	2.776503
C	-1.142743	-2.306894	2.214397
C	-0.044622	-4.207299	1.180423
C	-0.881701	-5.066947	1.893292
C	-1.965178	-3.179092	2.920273
H	-1.248865	-1.238074	2.352505
H	0.723239	-4.596852	0.521256
H	-0.771992	-6.141404	1.767603
H	-2.711902	-2.767450	3.594483
C	-2.730465	-5.502042	3.573000
H	-3.774370	-5.168982	3.563569
H	-2.696063	-6.522405	3.178960
H	-2.414948	-5.541696	4.623794
H	-0.810156	-3.132207	-3.283167
C	-1.186157	1.244926	-0.282972
C	-2.220752	1.792038	-1.056612
C	-1.330088	1.313873	1.108968
C	-3.358366	2.352612	-0.469180
C	-2.469961	1.864407	1.702743
H	-0.544812	0.921060	1.749199
C	-3.496678	2.386316	0.918847
H	-4.378407	2.816158	1.377888
C	-2.570475	1.853470	3.201363
C	-4.458615	2.879136	-1.346946
F	-2.607880	0.583280	3.688571
F	-3.674836	2.481462	3.656693
F	-1.500626	2.446733	3.781999
F	-3.976774	3.494491	-2.450073
F	-5.253889	3.760955	-0.704461
F	-5.277705	1.884490	-1.799023

O	1.339637	1.398068	-0.739987
C	1.891872	1.961128	0.287790
C	1.677579	3.412664	0.504100
C	1.945014	3.969042	1.764876
C	1.167624	4.225470	-0.519554
C	1.705830	5.320644	1.999535
H	2.326368	3.334885	2.562224
C	0.933820	5.577482	-0.282109
H	0.946809	3.781229	-1.484854
C	1.203450	6.127547	0.974720
H	1.902194	5.743533	2.980716
H	0.533197	6.203319	-1.074615
H	1.015013	7.181893	1.157759
H	2.034493	1.351356	1.180944
C	3.922044	2.095492	-0.245143
H	4.270614	2.753402	0.540735
H	3.692504	2.551089	-1.201597
C	4.356266	0.774705	-0.243084
C	5.047430	0.167203	0.911010
C	5.962452	0.922985	1.668918
C	4.797026	-1.170563	1.268063
C	6.619367	0.350521	2.754217
H	6.184027	1.947143	1.384274
C	5.447328	-1.730367	2.365571
H	4.056374	-1.743524	0.721166
C	6.360837	-0.977020	3.106311
H	7.335838	0.937092	3.322237
H	5.230077	-2.756279	2.648496
H	6.869404	-1.421276	3.957628
O	4.028111	0.013466	-1.271913
Si	4.900383	-0.943196	-2.423371
C	4.865696	-2.750016	-1.923471
H	3.859856	-3.053217	-1.616071
H	5.172780	-3.375871	-2.771014
H	5.546857	-2.955709	-1.090728
C	6.656507	-0.262716	-2.458290
H	7.253524	-0.806274	-3.201043
H	6.674845	0.798232	-2.733265
H	7.157099	-0.372061	-1.489684
H	-2.133245	1.774487	-2.137438
C	4.007366	-0.633640	-4.039518
H	4.480657	-1.203166	-4.849534
H	2.951363	-0.922307	-4.006368
H	4.046326	0.426983	-4.312607

TS 311

B3LYP/6-31G(d) = -3573.91917
B3LYP-D3(BJ)/def2-TZVPP/IEFPCM(propanonitrile) = -3575.489342
B3LYP-D3(BJ)/def2-TZVPP/IEFPCM(propanonitrile)//B3LYP-D3(BJ)/6-31G(d) Free Energy (Quasiharmonic) = -3574.773486

Frequencies (Top 3 out of 300)

1. -207.8112 cm^{-1}
2. 7.6088 cm^{-1}
3. 11.3436 cm^{-1}

B3LYP/6-31G(d) Molecular Geometry in Cartesian Coordinates

B	0.117919	0.926350	-0.704750
O	0.843528	2.031020	-1.361930
N	0.577049	1.117010	0.778370
C	1.290088	2.400720	0.932360
S	-0.181261	0.434820	2.074520
C	1.359428	2.919790	-0.508140
O	1.824118	3.990521	-0.826060
H	2.594688	2.039871	2.617600
H	0.685588	3.105840	1.517380
C	2.705008	2.371811	1.579830
C	3.767899	1.557372	0.892080
C	4.676448	1.981392	-0.154870
C	5.510929	0.870682	-0.459710
N	5.148069	-0.161618	0.382250
C	4.095609	0.252942	1.175240
H	3.676260	-0.417419	1.910510
C	6.686048	2.128743	-2.107240
C	4.884678	3.188562	-0.847770
H	7.128879	0.058583	-1.658240
H	5.427650	-1.123968	0.268840
C	5.883378	3.249472	-1.810900
H	7.453678	2.206843	-2.872730
H	4.251768	4.047762	-0.654170
C	6.511339	0.925123	-1.435980
H	6.044347	4.176373	-2.354940
C	1.020070	-0.504250	3.030150
O	-1.120220	-0.563481	1.533510
O	-0.685021	1.495310	2.971090
C	2.837040	-2.013859	4.538730
C	1.492419	-0.003539	4.243160
C	1.432090	-1.755750	2.564990
C	2.339830	-2.496549	3.317600
C	2.399670	-0.759889	4.985770
H	1.141989	0.958860	4.600470
H	1.053940	-2.139780	1.624270
H	2.671051	-3.462859	2.945520
H	2.770809	-0.368919	5.929960
C	3.796261	-2.840068	5.361840
H	4.463781	-3.430898	4.725880
H	4.412520	-2.210688	6.011620
H	3.254431	-3.544949	6.006380
H	3.016838	3.422291	1.624750
C	0.418520	-0.504770	-1.369900
C	-0.437650	-1.138400	-2.283820
C	1.625150	-1.149249	-1.071960
C	-0.101370	-2.363350	-2.865290
C	1.958020	-2.386749	-1.633860
H	2.316210	-0.677149	-0.381050
C	1.098311	-3.001590	-2.539580
H	1.353891	-3.956570	-2.984750
C	3.259601	-3.031119	-1.251750
C	-1.031319	-3.053171	-3.823470
F	4.332980	-2.372218	-1.753880
F	3.434401	-3.040569	0.102200
F	3.351611	-4.311189	-1.668000
F	-1.604059	-4.155281	-3.262620
F	-2.039240	-2.258471	-4.238900
F	-0.381379	-3.492830	-4.926230
O	-1.387951	1.257669	-0.965920
C	-2.036182	2.231899	-0.410610
C	-2.238172	3.487729	-1.152730
C	-1.948872	3.562419	-2.525280
C	-2.659163	4.641159	-0.467340
C	-2.089473	4.771899	-3.199190
H	-1.591642	2.676309	-3.037470
C	-2.792983	5.848469	-1.144350
H	-2.865553	4.587789	0.599430
C	-2.513223	5.913619	-2.513240
H	-1.854753	4.829659	-4.258020
H	-3.106274	6.739718	-0.608320
H	-2.614854	6.857399	-3.041890
H	-2.000042	2.307019	0.681340
C	-4.186911	1.725928	-0.294720
H	-4.387861	1.798458	-1.355800
H	-4.407912	2.592968	0.316180
C	-4.207831	0.479488	0.306960
C	-4.292890	-0.762072	-0.487280
C	-5.203520	-0.838562	-1.557820
C	-3.457980	-1.859722	-0.213200
C	-5.285800	-1.993532	-2.330700
H	-5.861351	0.000627	-1.763770

C	-3.532589	-3.003592	-1.004690
H	-2.710910	-1.775741	0.565760
C	-4.444189	-3.075022	-2.060450
H	-5.998910	-2.046643	-3.148390
H	-2.854999	-3.830851	-0.819360
H	-4.483429	-3.964802	-2.681510
O	-4.054671	0.429458	1.618290
Si	-4.604890	-0.452772	2.991650
C	-6.466890	-0.645873	2.784540
H	-6.713180	-1.245733	1.900720
H	-6.965011	0.325227	2.684600
H	-6.897800	-1.152663	3.656960
C	-4.135701	0.701118	4.390980
H	-3.070251	0.950468	4.326520
H	-4.323751	0.241478	5.368750
H	-4.702511	1.637878	4.343910
H	-1.380920	-0.671181	-2.542210
C	-3.762440	-2.119392	3.177920
H	-4.098539	-2.846122	2.431740
H	-2.676190	-2.015041	3.093930
H	-3.991650	-2.527792	4.170910

TS 312

B3LYP/6-31G(d) = -3573.921147
B3LYP-D3(BJ)/def2-TZVPP/IEFPCM(propanonitrile) = -3575.49011
B3LYP-D3(BJ)/def2-TZVPP/IEFPCM(propanonitrile)//B3LYP-D3(BJ)/6-31G(d) Free Energy (Quasiharmonic) = -3574.772992

Frequencies (Top 3 out of 300)

1. -283.7490 cm^{-1}
2. 9.4670 cm^{-1}
3. 12.6574 cm^{-1}

B3LYP/6-31G(d) Molecular Geometry in Cartesian Coordinates

B	0.449274	-0.177500	-1.263916
O	0.817949	-1.114385	-2.337265
N	1.654509	0.832230	-1.270018
C	2.560022	0.483850	-2.384360
S	1.381608	2.442380	-0.963954
C	1.902693	-0.740508	-3.028052
O	2.315390	-1.309899	-4.011197
H	4.498481	1.093958	-1.648931
H	2.575694	1.276777	-3.142774
C	4.026846	0.162413	-1.977308
C	4.189649	-0.891752	-0.918305
C	4.321411	-2.322414	-1.093156
C	4.436278	-2.894358	0.205106
N	4.399252	-1.857782	1.116509
C	4.233140	-0.667742	0.435620
H	4.181904	0.267265	0.975703
C	4.615815	-5.085969	-0.715834
C	4.365843	-3.174818	-2.211830
H	4.661979	-4.686600	1.410435
H	4.276276	-1.959279	2.112384
C	4.512579	-4.541740	-2.012473
H	4.725461	-6.160364	-0.593196
H	4.256934	-2.771916	-3.213802
C	4.579391	-4.270792	0.409223
H	4.541206	-5.207064	-2.871321
C	2.913589	3.084942	-0.267630
O	0.380431	2.499095	0.123405
O	1.142195	3.212509	-2.197215
C	5.280004	4.130826	0.815184
C	3.802694	3.778257	-1.089091
C	3.176796	2.931631	1.096243
C	4.355791	3.453048	1.624786
C	4.979434	4.291429	-0.543746
H	3.562140	3.927653	-2.136293
H	2.460678	2.432516	1.740520
H	4.557461	3.339944	2.687351
H	5.670995	4.832872	-1.184620
C	6.570184	4.660930	1.393333
H	6.439985	4.990869	2.429393
H	6.953484	5.505415	0.811797
H	7.347270	3.885140	1.394531
H	4.536567	-0.140373	-2.899797
C	0.228028	-0.958550	0.139896
C	-0.521382	-2.141402	0.241483
C	0.854495	-0.511666	1.311824
C	-0.668871	-2.817995	1.458387
H	-0.999654	-2.558884	-0.637667
C	0.739248	-1.197310	2.522461
H	1.454443	0.385897	1.268103
C	-0.036184	-2.353666	2.610425
H	-0.140000	-2.883390	3.550202
C	1.510773	-0.722940	3.719940
C	-1.574154	-4.015157	1.531634
F	1.682688	0.619263	3.723497
F	2.765394	-1.263898	3.765908
F	0.915084	-1.058021	4.883196
F	-1.287180	-4.817618	2.577720
F	-1.526131	-4.764994	0.412861
F	-2.880437	-3.638325	1.684373
O	-0.768983	0.618103	-1.741093
C	-2.067898	0.614418	-1.747918
C	-2.873233	-0.513272	-2.287195
C	-4.260375	-0.337513	-2.440439
C	-2.278466	-1.687751	-2.778061
C	-5.041659	-1.325482	-3.030770
H	-4.727339	0.583174	-2.102394
C	-3.066366	-2.676018	-3.368002
H	-1.200925	-1.803067	-2.751185
C	-4.446337	-2.504732	-3.487399
H	-6.112088	-1.175036	-3.138264
H	-2.593147	-3.575598	-3.751018
H	-5.054294	-3.277836	-3.949324
H	-2.478916	1.594885	-1.999561
C	-2.506763	0.884630	0.252737
H	-1.711032	1.617943	0.335943
H	-2.239925	-0.102457	0.602511
C	-3.820089	1.310003	0.444469
C	-4.940013	0.402967	0.745201
C	-6.257593	0.902274	0.784661
C	-4.727997	-0.964529	1.011186
C	-7.325011	0.059389	1.074842
H	-6.429347	1.953430	0.584883
C	-5.798959	-1.803000	1.302550
H	-3.731770	-1.387913	1.005101
C	-7.099815	-1.295844	1.334301
H	-8.334984	0.458703	1.100896
H	-5.608553	-2.851949	1.507918
H	-7.934825	-1.951962	1.564348
O	-4.175130	2.578606	0.285023
Si	-3.352824	4.115700	0.304670
C	-2.479934	4.386767	-1.332450
H	-3.142813	4.192869	-2.184022
H	-2.167188	5.437035	-1.400630
H	-1.574686	3.780495	-1.439344
C	-2.194919	4.175958	1.779649
H	-2.662806	3.764755	2.681581
H	-1.258162	3.643556	1.589466
H	-1.941038	5.222938	1.990142
C	-4.807630	5.286734	0.504647
H	-5.522210	5.180361	-0.319583
H	-5.344043	5.103663	1.442547
H	-4.466002	6.328883	0.514110

TS 313

B3LYP/6-31G(d) = -3573.917304
B3LYP-D3(BJ)/def2-TZVPP/IEFPCM(propanonitrile) = -3575.489342
B3LYP-D3(BJ)/def2-TZVPP/IEFPCM(propanonitrile)//B3LYP-D3(BJ)/6-31G(d) Free Energy (Quasiharmonic) = -3574.772941

Frequencies (Top 3 out of 300)

1. -292.9472 cm^{-1}
2. 6.1483 cm^{-1}
3. 10.4722 cm^{-1}

B3LYP/6-31G(d) Molecular Geometry in Cartesian Coordinates

B	-0.777729	-0.913248	0.743278
O	-0.896555	-1.553442	2.071374
N	-1.410412	-2.013488	-0.169359
C	-1.678739	-3.236294	0.599318
S	-1.632779	-1.935612	-1.790301
C	-1.402757	-2.789147	2.038973
O	-1.607254	-3.470120	3.018187
H	-1.208060	-4.910856	-0.679623
H	-2.740750	-3.514634	0.541557
C	-0.847716	-4.514595	0.271458
C	0.644888	-4.349976	0.209566
C	1.603594	-4.440940	1.289719
C	2.894464	-4.234893	0.725867
N	2.717185	-4.011127	-0.625811
C	1.369922	-4.097719	-0.928350
H	1.011686	-3.962061	-1.938695
C	3.935168	-4.537925	2.849603
C	1.506537	-4.693406	2.670606
H	5.041948	-4.126645	1.035789
H	3.456680	-3.899332	-1.301149
C	2.667814	-4.740280	3.432443
H	4.824969	-4.582251	3.472362
H	0.535378	-4.827080	3.136894
C	4.065351	-4.280985	1.489299
H	2.598878	-4.931899	4.499950
C	-3.389495	-1.649996	-2.070114
O	-1.344557	-3.239298	-2.411589
O	-0.911189	-0.739425	-2.276571
C	-6.141977	-1.244894	-2.440757
C	-3.876675	-0.359034	-2.280207
C	-4.260271	-2.743714	-2.065053
C	-5.625586	-2.533332	-2.245222
C	-5.245087	-0.168642	-2.465689
H	-3.197788	0.484133	-2.319234
H	-3.868234	-3.748906	-1.952595
H	-6.299769	-3.386565	-2.244997
H	-5.620792	0.837060	-2.638695
C	-7.626420	-1.021863	-2.606076
H	-7.833615	-0.135624	-3.214806
H	-8.112108	-0.869636	-1.632979
H	-8.111390	-1.881795	-3.079869
H	-1.118028	-5.236870	1.051523
C	-1.511913	0.545244	0.811854
C	-1.563489	1.474532	-0.237774
C	-2.156462	0.925238	2.004760
C	-2.184467	2.720079	-0.097341
H	-1.131049	1.218213	-1.196103
C	-2.790935	2.162464	2.147511
H	-2.152697	0.238327	2.844521
C	-2.801933	3.080312	1.097818
H	-3.278704	4.046517	1.209854
C	-3.503914	2.486902	3.432987
C	-2.112721	3.680846	-1.246249
F	-2.839444	2.012655	4.510202
F	-4.744159	1.947218	3.469598
F	-3.652002	3.821637	3.606650
F	-2.912365	4.753682	-1.090063
F	-2.440356	3.096394	-2.422781
F	-0.841123	4.162378	-1.410433
O	0.685624	-0.893712	0.357999
C	1.797716	-0.205678	0.405589
C	2.284842	0.481619	1.644059
C	1.465959	0.731573	2.752952
C	3.646186	0.827242	1.711307
C	1.994142	1.340253	3.891935
H	0.430706	0.419673	2.744796
C	4.172363	1.431358	2.850877
H	4.300284	0.602163	0.872815
C	3.342622	1.698274	3.943543
H	1.349370	1.520077	4.747449
H	5.229664	1.679142	2.893135
H	3.749777	2.165299	4.836164
H	2.602297	-0.756849	-0.090477
C	1.587423	1.161159	-0.938803
H	1.100335	1.962737	-0.396429
H	0.915934	0.569935	-1.550099
C	2.837353	1.462214	-1.512688
C	3.501609	0.565839	-2.468660
C	4.834380	0.815599	-2.855317
C	2.830099	-0.551600	-3.012059
C	5.479108	-0.026861	-3.753679
H	5.353313	1.670773	-2.438120
C	3.482127	-1.385843	-3.915755
H	1.798576	-0.766765	-2.752221
C	4.804654	-1.129070	-4.287303
H	6.506885	0.175061	-4.041055
H	2.949390	-2.232131	-4.339518
H	5.306836	-1.782466	-4.995460
O	3.553225	2.497567	-1.121761
Si	3.196260	4.139974	-0.641245
C	4.901175	4.746687	-0.152669
H	5.285016	4.186474	0.707258
H	5.618606	4.642497	-0.974061
H	4.867210	5.805858	0.129341
C	1.981923	4.255001	0.782766
H	2.203158	3.546457	1.586804
H	2.047432	5.265906	1.207160
H	0.946987	4.110190	0.457834
C	2.528594	4.967690	-2.188303
H	3.193932	4.827245	-3.047192
H	1.537144	4.581711	-2.449840
H	2.422585	6.046751	-2.020102

TS 314

B3LYP/6-31G(d) = -3573.915622
B3LYP-D3(BJ)/def2-TZVPP/IEFPCM(propanonitrile) = -3575.487602
B3LYP-D3(BJ)/def2-TZVPP/IEFPCM(propanonitrile)//B3LYP-D3(BJ)/6-31G(d) Free Energy (Quasiharmonic) = -3574.772373

Frequencies (Top 3 out of 300)

1. -205.8367 cm^{-1}
2. 12.3309 cm^{-1}
3. 12.6778 cm^{-1}

B3LYP/6-31G(d) Molecular Geometry in Cartesian Coordinates

B	-0.261629	-0.393710	0.981350
O	-0.969059	-0.670220	2.246160
N	0.041900	1.153741	1.122760
C	-0.593390	1.656800	2.359280
S	1.446460	1.856261	0.622440
C	-1.125289	0.391910	3.037600
O	-1.638249	0.366670	4.133420
H	-1.309361	3.631260	1.832780

H	0.142650	2.104391	3.038670
C	-1.751100	2.679230	2.142130
C	-2.799130	2.262310	1.149670
C	-3.964760	1.431769	1.365650
C	-4.614020	1.287419	0.107200
N	-3.895260	2.023509	-0.815070
C	-2.799920	2.579850	-0.185830
H	-2.098860	3.183270	-0.744660
C	-6.297059	-0.098012	1.071290
C	-4.526350	0.798969	2.490180
H	-6.246259	0.416158	-1.029220
H	-3.990490	1.953849	-1.817130
C	-5.681929	0.044169	2.331670
H	-7.193229	-0.705402	0.977920
H	-4.047230	0.874769	3.461330
C	-5.773999	0.523718	-0.056090
H	-6.116079	-0.455332	3.193690
C	1.095909	3.603541	0.384690
O	1.759300	1.297291	-0.707900
O	2.493880	1.798931	1.667930
C	0.598328	6.332081	-0.019370
C	0.581369	4.038091	-0.839100
C	1.393849	4.516441	1.396730
C	1.137559	5.871431	1.189350
C	0.336269	5.395701	-1.031130
H	0.397420	3.323151	-1.634070
H	1.836659	4.169021	2.324020
H	1.369398	6.583431	1.977550
H	-0.057711	5.736041	-1.985720
C	0.294418	7.796431	-0.226000
H	0.460668	8.097801	-1.265570
H	0.914178	8.429721	0.416570
H	-0.754822	8.015690	0.011900
H	-2.193780	2.843400	3.132260
C	-1.145859	-0.828760	-0.291200
C	-2.068989	-1.879950	-0.170550
C	-1.082319	-0.173040	-1.524860
C	-2.882048	-2.268360	-1.237350
H	-2.168958	-2.392110	0.780880
C	-1.907539	-0.545070	-2.591900
H	-0.379550	0.642810	-1.654510
C	-2.810359	-1.598250	-2.459040
H	-3.443649	-1.893341	-3.287660
C	-1.870389	0.262110	-3.853130
C	-3.902008	-3.359081	-1.058980
F	-2.516680	1.459330	-3.704340
F	-2.454809	-0.359530	-4.897340
F	-0.604090	0.575950	-4.228630
F	-3.537068	-4.247351	-0.107490
F	-4.095288	-4.056331	-2.206180
F	-5.110208	-2.868911	-0.698140
O	0.989521	-1.296579	0.979560
C	1.912491	-1.400609	1.889420
C	1.722262	-2.279629	3.049950
C	0.711422	-3.255709	3.067970
C	2.544611	-2.109329	4.178050
C	0.530342	-4.046329	4.197650
H	0.074062	-3.371609	2.198740
C	2.354932	-2.897569	5.308350
H	3.318591	-1.345158	4.166750
C	1.349542	-3.869429	5.317340
H	-0.256768	-4.794470	4.212920
H	2.982402	-2.753268	6.183130
H	1.199922	-4.484739	6.200300
H	2.588521	-0.550339	2.015470
C	3.593842	-2.550208	1.001360
H	4.266802	-2.302888	1.814060
H	3.132432	-3.529318	1.012610
C	3.726851	-1.882668	-0.201550
C	3.087782	-2.373468	-1.437790
C	3.175182	-3.736128	-1.777650
C	2.361781	-1.500299	-2.266310
C	2.569432	-4.210019	-2.937930
H	3.742242	-4.412348	-1.144410
C	1.738011	-1.986539	-3.413970
H	2.241691	-0.465199	-1.970730
C	1.848122	-3.335959	-3.756190
H	2.656543	-5.259969	-3.202800
H	1.146171	-1.311509	-4.024240
H	1.364342	-3.709839	-4.654510
O	4.342401	-0.710828	-0.190100
Si	5.445571	0.208282	-1.140700
C	4.629500	1.031852	-2.615220
H	4.323991	0.312892	-3.381850
H	3.746340	1.596802	-2.301020
H	5.339930	1.731452	-3.075020
C	6.797411	-0.985857	-1.683300
H	6.401001	-1.771877	-2.336620
H	7.575681	-0.453607	-2.244210
H	7.277811	-1.469107	-0.824890
C	6.042040	1.476883	0.103180
H	5.181800	1.966942	0.573450
H	6.635430	1.009903	0.897440
H	6.660510	2.247203	-0.372740

TS 315

B3LYP/6-31G(d) = -3573.908874
B3LYP-D3(BJ)/def2-TZVPP/IEFPCM(propanonitrile) = -3575.48805
B3LYP-D3(BJ)/def2-TZVPP/IEFPCM(propanonitrile)//B3LYP-D3(BJ)/6-31G(d) Free Energy (Quasiharmonic) = -3574.771899

Frequencies (Top 3 out of 300)

1. -299.2246 cm^{-1}
2. 10.2992 cm^{-1}
3. 15.2539 cm^{-1}

B3LYP/6-31G(d) Molecular Geometry in Cartesian Coordinates

B	0.428461	0.930829	-0.106243
O	0.003091	0.438318	-1.439336
N	1.271185	2.179799	-0.504612
C	1.416825	2.260747	-1.963429
S	1.564493	3.520332	0.403573
C	0.516822	1.130393	-2.460785
O	0.269446	0.885722	-3.624215
H	3.359870	3.101784	-2.316933
H	0.989312	3.192607	-2.361653
C	2.871245	2.143916	-2.510228
C	3.679934	1.034761	-1.907107
C	3.776849	-0.340955	-2.335194
C	4.616981	-1.011449	-1.400585
N	5.024701	-0.076429	-0.469596
C	4.438030	1.138094	-0.766975
H	4.602796	2.002887	-0.139154
C	4.361853	-3.073005	-2.574013
C	3.238968	-1.074577	-3.408270
H	5.557744	-2.868049	-0.782839
H	5.452782	-0.291316	0.419128
C	3.534431	-2.428425	-3.516251
H	4.571752	-4.133763	-2.682614
H	2.585324	-0.591791	-4.129862
C	4.916627	-2.373151	-1.508043
H	3.121720	-3.003836	-4.341289
C	0.200814	4.671175	0.134976
O	2.760523	4.187476	-0.134815
O	1.502722	3.119170	1.815717
C	-1.931533	6.448103	-0.286613
C	0.259330	5.588901	-0.915721
C	-0.901108	4.648111	0.993316

C	-1.954114	5.534801	0.778500
C	-0.807588	6.463845	-1.122288
H	1.146070	5.640436	-1.538459
H	-0.906848	3.961031	1.831968
H	-2.801842	5.532070	1.460555
H	-0.756439	7.182011	-1.937297
C	-3.091069	7.385752	-0.527638
H	-2.764487	8.310171	-1.014944
H	-3.591293	7.654161	0.408928
H	-3.845574	6.924323	-1.179136
H	2.787012	2.029746	-3.598012
C	1.227744	-0.236200	0.685583
C	2.095255	0.024249	1.761053
C	1.131392	-1.568370	0.253369
C	2.831581	-0.999295	2.361779
H	2.209174	1.043098	2.116546
C	1.837045	-2.601850	0.881385
H	0.509114	-1.805489	-0.603087
C	2.698735	-2.323366	1.938042
H	3.265318	-3.115338	2.413356
C	1.632728	-4.019118	0.427737
C	3.868258	-0.666688	3.395717
F	1.597312	-4.129909	-0.918843
F	0.447676	-4.521319	0.880642
F	2.597340	-4.852114	0.875839
F	3.557678	0.426667	4.115279
F	5.082317	-0.415775	2.813387
F	4.075051	-1.683526	4.263731
O	-0.827781	1.432610	0.603446
C	-2.087715	1.208938	0.855233
C	-2.547492	0.235719	1.890744
C	-3.762027	0.507236	2.541334
C	-1.806966	-0.890596	2.275255
C	-4.226800	-0.323647	3.559337
H	-4.337929	1.385108	2.256669
C	-2.275011	-1.723574	3.290997
H	-0.859518	-1.102373	1.796912
C	-3.482592	-1.444491	3.936332
H	-5.158763	-0.089161	4.066716
H	-1.685285	-2.586889	3.586292
H	-3.835168	-2.089190	4.736939
H	-2.646535	2.148643	0.921072
C	-3.045097	0.775053	-0.870649
H	-3.838822	1.511278	-0.784195
H	-2.223039	1.026437	-1.527191
C	-3.485101	-0.559349	-0.875104
C	-2.703466	-1.679703	-1.404739
C	-2.718124	-2.925826	-0.742431
C	-1.913922	-1.523875	-2.560156
C	-1.957049	-3.984602	-1.222831
H	-3.273388	-3.032984	0.182825
C	-1.173651	-2.596817	-3.048248
H	-1.867259	-0.573841	-3.077920
C	-1.189924	-3.823636	-2.382084
H	-1.932957	-4.924559	-0.680995
H	-0.559976	-2.457866	-3.932358
H	-0.584142	-4.647338	-2.746793
O	-4.620934	-0.807556	-0.246872
Si	-6.039130	-1.777833	-0.454490
C	-6.054649	-2.514905	-2.181411
H	-5.957761	-1.742394	-2.952888
H	-5.253775	-3.245061	-2.331251
H	-7.011003	-3.026772	-2.348146
C	-7.428599	-0.538543	-0.217752
H	-7.392720	0.253294	-0.974476
H	-8.406473	-1.029378	-0.295083
H	-7.370634	-0.064165	0.768265
C	-6.056554	-3.073962	0.903242
H	-5.391720	-3.916360	0.686528
H	-5.752617	-2.639820	1.862363
H	-7.070279	-3.475797	1.023836

TS 316

B3LYP/6-31G(d) = -3573.915375

B3LYP-D3(BJ)/def2-TZVPP/IEFPCM(propanonitrile) = -3575.486944

B3LYP-D3(BJ)/def2-TZVPP/IEFPCM(propanonitrile)//B3LYP-D3(BJ)/6-31G(d) Free Energy (Quasiharmonic) = -3574.770476

Frequencies (Top 3 out of 300)

1. -288.2074 cm^{-1}
2. 10.0994 cm^{-1}
3. 12.1915 cm^{-1}

B3LYP/6-31G(d) Molecular Geometry in Cartesian Coordinates

B	0.255432	-0.737092	-0.007464
O	-0.165390	-1.125560	1.363347
N	1.589546	-1.525910	-0.182710
C	1.851271	-2.307924	1.040252
S	1.996276	-2.202581	-1.648829
C	0.622861	-2.052681	1.913281
O	0.386832	-2.600187	2.969389
H	4.004186	-2.268915	1.200088
H	1.876894	-3.383039	0.824058
C	3.156136	-1.925926	1.800975
C	3.311913	-0.464664	2.112436
C	2.844648	0.256734	3.275786
C	3.205370	1.622127	3.095084
N	3.877391	1.714111	1.892278
C	3.918147	0.465403	1.304657
H	4.397707	0.328383	0.345662
C	2.230640	2.212609	5.189572
C	2.168015	-0.112194	4.452934
H	3.185764	3.646392	3.880511
H	4.098416	2.571563	1.408590
C	1.869257	0.865191	5.394037
H	1.979303	2.954576	5.942929
H	1.861234	-1.141816	4.610935
C	2.903324	2.608610	4.039438
H	1.342035	0.589098	6.303448
C	3.797305	-2.174861	-1.715805
O	1.523403	-1.261430	-2.677678
O	1.613301	-3.622869	-1.705610
C	6.599437	-2.155305	-1.863469
C	4.459326	-1.027846	-2.161800
C	4.517087	-3.319986	-1.374036
C	5.910038	-3.300715	-1.444890
C	5.851031	-1.026294	-2.229588
H	3.894071	-0.156873	-2.476499
H	3.987091	-4.218925	-1.077559
H	6.468996	-4.194994	-1.180137
H	6.365105	-0.136118	-2.584465
C	8.108492	-2.129812	-1.910376
H	8.523355	-3.136163	-2.026146
H	8.473607	-1.513066	-2.738433
H	8.523356	-1.709270	-0.984699
H	3.161121	-2.521174	2.722330
C	0.372365	0.872661	-0.102148
C	-0.468642	1.712452	0.637481
C	1.376663	1.498737	-0.855845
C	-0.380675	3.106804	0.557545
H	-1.200524	1.281492	1.311818
C	1.518219	2.886383	-0.884981
H	2.062882	0.883994	-1.424132
C	0.619410	3.708654	-0.199805
H	0.704704	4.787769	-0.248728
C	2.700443	3.494557	-1.580249
C	-1.417047	3.926770	1.270940
F	3.768509	3.617493	-0.732725

F	2.447256	4.734815	-2.047664
F	3.132645	2.746693	-2.619655
F	-1.547769	3.570112	2.565824
F	-1.154437	5.249381	1.234923
F	-2.648414	3.754648	0.704833
O	-0.800110	-1.397244	-0.912909
C	-1.998406	-1.163517	-1.372404
C	-2.250733	-0.210274	-2.484402
C	-1.204931	0.508391	-3.083073
C	-3.542851	-0.123622	-3.033788
C	-1.461157	1.328823	-4.180775
H	-0.192902	0.373200	-2.728593
C	-3.793416	0.696422	-4.128942
H	-4.352875	-0.705760	-2.605673
C	-2.752548	1.435666	-4.698877
H	-0.641962	1.873238	-4.641797
H	-4.796957	0.755851	-4.540717
H	-2.945995	2.076523	-5.554942
H	-2.599852	-2.074483	-1.436338
C	-3.039082	-0.443101	0.263444
H	-2.343087	-0.906996	0.953963
H	-2.887188	0.615094	0.103760
C	-4.355788	-0.907561	0.277009
C	-5.504121	-0.120226	-0.204534
C	-5.420825	1.278066	-0.362689
C	-6.722575	-0.766067	-0.494162
C	-6.523363	2.000565	-0.810029
H	-4.512434	1.813367	-0.108580
C	-7.817199	-0.038788	-0.948750
H	-6.792987	-1.839936	-0.365069
C	-7.720673	1.346582	-1.108703
H	-6.448161	3.078424	-0.918211
H	-8.747919	-0.550518	-1.176367
H	-8.578317	1.915052	-1.457670
O	-4.661241	-2.135281	0.661889
Si	-3.873108	-3.365598	1.627234
C	-2.572199	-4.221737	0.583241
H	-2.114481	-5.027865	1.170881
H	-3.008503	-4.676925	-0.313458
H	-1.763171	-3.552657	0.273186
C	-5.322813	-4.504452	1.981795
H	-6.099471	-3.994634	2.562892
H	-5.779369	-4.873389	1.056301
H	-4.993087	-5.376528	2.559486
C	-3.198148	-2.579032	3.191039
H	-3.204369	-3.331559	3.990109
H	-2.164375	-2.231676	3.091381
H	-3.824140	-1.744449	3.528031

TS 317

B3LYP/6-31G(d) = -3573.918444
B3LYP-D3(BJ)/def2-TZVPP/IEFPCM(propanonitrile) = -3575.486574
B3LYP-D3(BJ)/def2-TZVPP/IEFPCM(propanonitrile)//B3LYP-D3(BJ)/6-31G(d) Free Energy (Quasiharmonic) = -3574.770368

Frequencies (Top 3 out of 300)

1. -170.4963 cm^{-1}
2. 8.8144 cm^{-1}
3. 11.1109 cm^{-1}

B3LYP/6-31G(d) Molecular Geometry in Cartesian Coordinates

B	-0.226157	1.404008	-0.520755
O	-0.692697	2.509776	-1.379138
N	-0.088523	0.241538	-1.568507
C	-0.548724	0.703587	-2.894219
S	1.023359	-0.970661	-1.493564
C	-0.814141	2.193490	-2.671157
O	-1.141890	2.973198	-3.537073
H	-1.634995	-1.031713	-3.612424
H	0.251295	0.600843	-3.636546
C	-1.834342	0.033812	-3.461363
C	-3.080341	0.209760	-2.631943
C	-3.804519	-0.812946	-1.914062
C	-4.881477	-0.173440	-1.239189
N	-4.834129	1.163436	-1.571324
C	-3.749516	1.389214	-2.394636
H	-3.536894	2.384093	-2.759587
C	-5.605004	-2.243359	-0.304760
C	-3.668782	-2.206401	-1.788314
H	-6.575658	-0.351522	0.103532
H	-5.369581	1.884152	-1.112345
C	-4.565420	-2.905549	-0.990032
H	-6.284108	-2.816324	0.321056
H	-2.874650	-2.733487	-2.308085
C	-5.778222	-0.869570	-0.422158
H	-4.464384	-3.983087	-0.887849
C	0.213461	-2.554843	-1.752696
O	1.538767	-0.984977	-0.110477
O	1.989634	-0.826307	-2.605253
C	-0.916935	-5.091117	-2.131360
C	-0.387840	-3.209377	-0.674940
C	0.260391	-3.150789	-3.014574
C	-0.312517	-4.408723	-3.196467
C	-0.944178	-4.470696	-0.873808
H	-0.419711	-2.749614	0.306188
H	0.757986	-2.642361	-3.833353
H	-0.278042	-4.872053	-4.179479
H	-1.411977	-4.979710	-0.034975
C	-1.496351	-6.472115	-2.324817
H	-0.754259	-7.246199	-2.088032
H	-2.359139	-6.639621	-1.671900
H	-1.815735	-6.631729	-3.359594
H	-1.970138	0.471250	-4.458492
C	-1.225251	1.139052	0.712777
C	-2.329976	1.978632	0.910293
C	-1.089879	0.043226	1.579068
C	-3.276301	1.722613	1.908435
C	-2.035635	-0.220611	2.572716
H	-0.239906	-0.620553	1.464398
C	-3.140196	0.615439	2.742934
H	-3.880245	0.404088	3.506351
C	-1.859063	-1.392105	3.495660
C	-4.485530	2.605054	2.034614
F	-1.086382	-2.362901	2.954208
F	-3.037653	-1.956299	3.836617
F	-1.259869	-1.031404	4.665781
F	-4.997723	2.600038	3.283613
F	-5.500996	2.206711	1.208391
F	-4.218975	3.887386	1.707226
O	1.120580	1.926553	0.064747
C	2.194316	2.155828	-0.618994
C	2.544192	3.535514	-0.973807
C	1.864178	4.620811	-0.396011
C	3.521304	3.770567	-1.958276
C	2.166310	5.919640	-0.793124
H	1.093362	4.426786	0.341428
C	3.813946	5.070001	-2.356805
H	4.037946	2.928546	-2.413369
C	3.139925	6.146567	-1.770411
H	1.635309	6.757202	-0.350270
H	4.560179	5.247106	-3.125813
H	3.368342	7.162010	-2.082194
H	2.577351	1.363290	-1.266638
C	3.962023	1.896342	0.812585
H	3.505689	2.551803	1.543532
H	4.627214	2.347643	0.088437
C	4.238380	0.587163	1.169395
C	3.697770	-0.033889	2.393137

C	2.412297	0.293376	2.856066
C	4.500411	-0.901967	3.153423
C	1.932663	-0.261453	4.039762
H	1.780966	0.949137	2.266312
C	4.025070	-1.435892	4.348481
H	5.510855	-1.125296	2.826581
C	2.736974	-1.124322	4.788775
H	0.924953	-0.036749	4.370922
H	4.660174	-2.092856	4.936340
H	2.359639	-1.550069	5.714384
O	5.112951	-0.081900	0.429601
Si	5.466634	-1.658276	-0.179987
C	4.278164	-2.972068	0.435274
H	3.239678	-2.646242	0.321279
H	4.417267	-3.880113	-0.166264
H	4.440890	-3.235097	1.484712
C	7.234269	-1.976998	0.386274
H	7.610718	-2.907072	-0.057858
H	7.902716	-1.166671	0.074080
H	7.314659	-2.078213	1.474646
H	-2.460002	2.838831	0.262593
C	5.392034	-1.464531	-2.042876
H	4.372183	-1.249160	-2.379613
H	6.049197	-0.655392	-2.382215
H	5.721585	-2.388370	-2.535434

TS 318

B3LYP/6-31G(d) = -3573.915428
B3LYP-D3(BJ)/def2-TZVPP/IEFPCM(propanonitrile) = -3575.485605
B3LYP-D3(BJ)/def2-TZVPP/IEFPCM(propanonitrile)//B3LYP-D3(BJ)/6-31G(d) Free Energy (Quasiharmonic) = -3574.769668

Frequencies (Top 3 out of 300)

1. -275.8339 cm^{-1}
2. 7.8066 cm^{-1}
3. 8.4983 cm^{-1}

B3LYP/6-31G(d) Molecular Geometry in Cartesian Coordinates

B	-0.044707	-0.670528	-0.955935
O	0.312907	-1.340879	-2.213669
N	0.509246	0.782817	-1.203864
C	0.894132	0.916152	-2.622662
S	-0.218808	2.070781	-0.459397
C	0.697449	-0.497562	-3.177537
O	0.913121	-0.826530	-4.322365
H	2.463985	2.408136	-2.546797
H	0.205390	1.585518	-3.154744
C	2.347409	1.385032	-2.916473
C	3.450973	0.520366	-2.359465
C	4.476337	0.927299	-1.426852
C	5.289376	-0.209994	-1.163539
N	4.802507	-1.243947	-1.934438
C	3.699309	-0.803493	-2.643554
H	3.184827	-1.471330	-3.319682
C	6.665790	1.044284	0.324272
C	4.807447	2.145585	-0.808257
H	6.967507	-1.059373	-0.080449
H	5.075255	-2.210786	-1.842910
C	5.893580	2.193620	0.056201
H	7.504482	1.110799	1.012238
H	4.223660	3.040169	-1.002549
C	6.375793	-0.170957	-0.283289
H	6.155812	3.132975	0.536607
C	1.053877	3.292663	-0.108593
O	-1.182682	2.740881	-1.361741
O	-0.692888	1.592872	0.850789
C	2.901363	5.282984	0.578047
C	1.164291	4.433542	-0.903302
C	1.851328	3.131519	1.026723
C	2.765545	4.126171	1.361137
C	2.093771	5.415889	-0.559298
H	0.515601	4.555394	-1.764169
H	1.746403	2.243868	1.640930
H	3.388335	4.000154	2.243172
H	2.182465	6.305616	-1.178066
C	3.876189	6.367880	0.970542
H	4.813835	5.945886	1.348353
H	4.113577	7.019769	0.124039
H	3.462424	7.000233	1.767228
H	2.415501	1.438624	-4.010617
C	0.555467	-1.427099	0.332712
C	-0.104191	-1.502833	1.566620
C	1.805590	-2.056917	0.233377
C	0.456453	-2.189368	2.650214
C	2.374219	-2.735519	1.312942
H	2.339022	-2.022805	-0.709518
C	1.699900	-2.809976	2.532484
H	2.126846	-3.352524	3.367770
C	3.740405	-3.348026	1.174049
C	-0.271438	-2.220895	3.964442
F	3.967316	-3.807747	-0.085930
F	3.920225	-4.392418	2.013861
F	4.728097	-2.460465	1.436968
F	-1.616487	-2.315853	3.795261
F	-0.058968	-1.097585	4.692712
F	0.105320	-3.263654	4.737788
O	-1.587997	-0.581556	-0.898546
C	-2.541388	-1.254000	-1.474396
C	-2.423113	-2.657432	-1.947527
C	-1.630090	-3.621572	-1.306530
C	-3.170542	-3.025063	-3.077767
C	-1.579726	-4.922106	-1.798645
H	-1.052054	-3.349351	-0.430928
C	-3.112384	-4.325797	-3.572988
H	-3.786513	-2.281580	-3.578955
C	-2.317258	-5.277585	-2.931973
H	-0.959470	-5.661168	-1.299644
H	-3.681814	-4.594446	-4.458194
H	-2.270331	-6.293533	-3.314365
H	-3.204533	-0.645491	-2.097171
C	-4.015281	-1.513120	-0.039814
H	-4.742827	-1.985367	-0.689347
H	-3.494310	-2.154747	0.663713
C	-4.230079	-0.184877	0.322344
C	-5.097277	0.691674	-0.500312
C	-4.572040	1.877857	-1.044455
C	-6.415583	0.310011	-0.802226
C	-5.369259	2.669348	-1.871029
H	-3.535829	2.149974	-0.865073
C	-7.211739	1.121735	-1.607404
H	-6.819213	-0.607734	-0.383507
C	-6.688564	2.301234	-2.143946
H	-4.951633	3.572323	-2.306728
H	-8.237216	0.832659	-1.819238
H	-7.306975	2.927743	-2.780858
O	-3.509285	0.323309	1.291011
Si	-3.744871	1.545068	2.519188
C	-5.465631	1.203364	3.208117
H	-5.546774	0.180871	3.594432
H	-5.680320	1.888299	4.037878
H	-6.247152	1.346945	2.453270
C	-3.611899	3.290807	1.847647
H	-2.692136	3.413484	1.268768
H	-4.463413	3.575109	1.222451
H	-3.570882	3.986902	2.696028
H	-1.069438	-1.021336	1.680931
C	-2.393593	1.165539	3.749143
H	-2.450740	0.136272	4.114745

H	-1.414102	1.305739	3.281939
H	-2.461337	1.837761	4.613655

TS 319

B3LYP/6-31G(d) = -3573.914901
B3LYP-D3(BJ)/def2-TZVPP/IEFPCM(propanonitrile) = -3575.485046
B3LYP-D3(BJ)/def2-TZVPP/IEFPCM(propanonitrile)//B3LYP-D3(BJ)/6-31G(d) Free Energy (Quasiharmonic) = -3574.769425

Frequencies (Top 3 out of 300)

1. -276.2389 cm^{-1}
2. 6.9818 cm^{-1}
3. 8.8438 cm^{-1}

B3LYP/6-31G(d) Molecular Geometry in Cartesian Coordinates

B	0.001066	-0.665546	-0.925851
O	0.378257	-1.336385	-2.176971
N	0.522681	0.797837	-1.180966
C	0.880805	0.935077	-2.606617
S	-0.223407	2.073946	-0.432456
C	0.715999	-0.486139	-3.152114
O	0.915562	-0.812608	-4.300762
H	2.419268	2.459874	-2.536075
H	0.165083	1.583155	-3.129120
C	2.315324	1.442720	-2.925360
C	3.452379	0.589617	-2.420860
C	4.471276	0.977009	-1.473245
C	5.333998	-0.140193	-1.294372
N	4.876263	-1.141256	-2.122916
C	3.748271	-0.703291	-2.790794
H	3.248644	-1.347220	-3.500264
C	6.677475	1.069300	0.259407
C	4.760105	2.163912	-0.777509
H	7.063295	-0.986924	-0.291953
H	5.215347	-2.090633	-2.126000
C	5.854172	2.199835	0.077586
H	7.522015	1.125359	0.941132
H	4.138012	3.044204	-0.905763
C	6.429786	-0.113973	-0.425467
H	6.082246	3.114942	0.618345
C	1.031106	3.313413	-0.076219
O	-0.691620	1.587584	0.876368
O	-1.195145	2.733640	-1.334353
C	2.851101	5.327660	0.615418
C	1.816270	3.169814	1.069702
C	1.139538	4.449261	-0.878796
C	2.055452	5.443120	-0.532573
C	2.716776	4.176556	1.406714
H	1.712179	2.286497	1.690239
H	0.499676	4.558243	-1.747984
H	2.143006	6.328329	-1.157970
H	3.329201	4.064724	2.297922
C	3.808621	6.427095	1.009969
H	4.092890	7.039959	0.148763
H	4.723032	6.021151	1.455573
H	3.355315	7.096003	1.753632
H	2.354794	1.521825	-4.019246
C	0.594283	-1.404333	0.375337
C	1.830982	-2.063928	0.297664
C	-0.072965	-1.432994	1.607849
C	2.377349	-2.726103	1.400134
H	2.375175	-2.058994	-0.641063
C	0.469922	-2.094857	2.714540
H	-1.028223	-0.928318	1.702574
C	1.698727	-2.746909	2.618998
H	2.117414	-3.263203	3.474593
C	-0.260897	-2.061172	4.026865
C	3.662644	-3.492078	1.251743
F	-0.064855	-0.896039	4.690882
F	0.124257	-3.054428	4.858792
F	-1.604392	-2.181540	3.858571
F	4.528241	-2.882936	0.405150
F	3.451197	-4.735313	0.750741
F	4.302654	-3.652464	2.431353
O	-1.547853	-0.603407	-0.885228
C	-2.481040	-1.305732	-1.458521
C	-2.331373	-2.717415	-1.897529
C	-1.535346	-3.653969	-1.220424
C	-3.050580	-3.122083	-3.033441
C	-1.453342	-4.963750	-1.683052
H	-0.979703	-3.353379	-0.339750
C	-2.961081	-4.432026	-3.498821
H	-3.668768	-2.400196	-3.562660
C	-2.162606	-5.356036	-2.822246
H	-0.830497	-5.680917	-1.156120
H	-3.508695	-4.729395	-4.388611
H	-2.091264	-6.379011	-3.181453
H	-3.145282	-0.724177	-2.105589
C	-3.970077	-1.555755	-0.038955
H	-3.445603	-2.165082	0.690213
H	-4.677297	-2.062346	-0.685043
C	-4.219310	-0.222912	0.281857
C	-5.092541	0.610789	-0.578166
C	-6.395934	0.189580	-0.892039
C	-4.586699	1.795211	-1.144254
C	-7.197278	0.960542	-1.731392
H	-6.784751	-0.726519	-0.456097
C	-5.388382	2.545612	-2.004196
H	-3.560613	2.097278	-0.954333
C	-6.693479	2.138390	-2.289602
H	-8.211657	0.641049	-1.952744
H	-4.985542	3.447423	-2.455979
H	-7.315644	2.733099	-2.952797
O	-3.525226	0.328565	1.246394
Si	-3.808986	1.579047	2.434874
C	-5.543668	1.235894	3.087420
H	-5.787800	1.941374	3.891458
H	-6.306670	1.348543	2.308838
H	-5.623316	0.223895	3.500796
C	-3.679314	3.306387	1.716012
H	-4.530333	3.568414	1.080304
H	-2.759062	3.419317	1.135908
H	-3.644943	4.026200	2.544610
C	-2.489598	1.250268	3.713161
H	-2.539994	0.227577	4.097864
H	-1.498228	1.398113	3.274569
H	-2.597117	1.938510	4.560919

TS 320

B3LYP/6-31G(d) = -3573.915481
B3LYP-D3(BJ)/def2-TZVPP/IEFPCM(propanonitrile) = -3575.485897
B3LYP-D3(BJ)/def2-TZVPP/IEFPCM(propanonitrile)//B3LYP-D3(BJ)/6-31G(d) Free Energy (Quasiharmonic) = -3574.769001

Frequencies (Top 3 out of 300)

1. -313.7166 cm^{-1}
2. 10.9270 cm^{-1}
3. 15.1711 cm^{-1}

B3LYP/6-31G(d) Molecular Geometry in Cartesian Coordinates

B	0.285348	-0.500173	-0.137174
O	0.210690	0.613791	-1.120478
N	0.581311	-1.700865	-1.068006
C	0.703824	-1.261994	-2.466492

S	1.191001	-3.164783	-0.619511
C	0.512919	0.250707	-2.361030
O	0.622763	1.036153	-3.285246
H	0.014453	-2.938334	-3.622116
H	1.721066	-1.428303	-2.850846
C	-0.293217	-1.899963	-3.476975
C	-1.733769	-1.848435	-3.061365
C	-2.713258	-0.828540	-3.356803
C	-3.922481	-1.210116	-2.708798
N	-3.686941	-2.413418	-2.074603
C	-2.369752	-2.777416	-2.274741
H	-1.973266	-3.688942	-1.849270
C	-5.036349	0.732779	-3.530291
C	-2.695975	0.359853	-4.109198
H	-5.999393	-0.746498	-2.278842
H	-4.292070	-2.834428	-1.385373
C	-3.852896	1.126010	-4.188723
H	-5.924751	1.353989	-3.609287
H	-1.784247	0.682761	-4.603913
C	-5.086992	-0.439054	-2.783747
H	-3.847959	2.046067	-4.767634
C	2.985830	-3.083622	-0.760559
O	0.759175	-4.174065	-1.599158
O	0.874198	-3.333049	0.807195
C	5.778481	-2.942161	-0.987554
C	3.750875	-2.689662	0.339765
C	3.601432	-3.422717	-1.967143
C	4.989966	-3.341825	-2.073737
C	5.136362	-2.622252	0.218272
H	3.261738	-2.461681	1.279861
H	3.001190	-3.772219	-2.800348
H	5.467771	-3.605833	-3.014204
H	5.732709	-2.324736	1.078036
C	7.284284	-2.895001	-1.096409
H	7.607874	-2.771080	-2.134846
H	7.736112	-3.823045	-0.721669
H	7.703663	-2.071366	-0.508378
H	-0.142036	-1.375656	-4.428415
C	-1.079552	-0.523518	0.730381
C	-1.630641	0.691277	1.157216
C	-1.818515	-1.684505	1.012380
C	-2.838900	0.753918	1.857456
H	-1.116826	1.616582	0.924914
C	-3.039638	-1.626086	1.690507
H	-1.429425	-2.649707	0.712899
C	-3.559219	-0.405646	2.127576
H	-4.498698	-0.362940	2.665684
C	-3.841198	-2.880551	1.894499
C	-3.346692	2.101363	2.277889
F	-4.621536	-2.816529	2.995523
F	-3.067569	-3.977120	2.003995
F	-4.691470	-3.113428	0.845962
F	-4.426631	2.032598	3.080833
F	-3.692842	2.868693	1.209165
F	-2.393150	2.808909	2.944261
O	1.573873	-0.200301	0.649654
C	2.081975	0.373310	1.697186
C	1.888912	-0.163104	3.076074
C	2.714069	0.315319	4.111069
C	0.983782	-1.197490	3.353744
C	2.613160	-0.200030	5.398132
H	3.449043	1.090404	3.903453
C	0.885891	-1.713744	4.646359
H	0.392841	-1.625878	2.556738
C	1.688138	-1.213273	5.671547
H	3.257283	0.180047	6.186173
H	0.185529	-2.519791	4.844788
H	1.606890	-1.618545	6.676408
H	3.103089	0.721557	1.511073
C	1.495194	2.354161	1.990776
H	0.546858	2.296772	2.515951
H	2.365479	2.521509	2.612362
C	1.492975	3.008773	0.750671
C	2.746751	3.289077	0.019542
C	2.823970	3.010050	-1.357928
C	3.882908	3.775125	0.691566
C	4.021172	3.213984	-2.040520
H	1.966627	2.596132	-1.880085
C	5.068255	3.998628	-0.004611
H	3.825837	4.014166	1.749714
C	5.140374	3.713920	-1.370670
H	4.077788	2.976134	-3.098702
H	5.934004	4.396012	0.517425
H	6.068351	3.880118	-1.910821
O	0.347226	3.252951	0.170149
Si	-0.493660	4.489750	-0.727236
C	-1.330242	5.503589	0.616060
H	-1.967819	4.876434	1.246194
H	-0.598304	6.005433	1.260160
H	-1.961915	6.280026	0.166399
C	-1.678104	3.545295	-1.812151
H	-2.399985	2.977840	-1.216575
H	-2.241371	4.237739	-2.450233
H	-1.142932	2.840526	-2.456683
C	0.697762	5.567863	-1.704546
H	1.492254	5.993051	-1.081782
H	1.165813	5.032379	-2.535488
H	0.128809	6.406437	-2.127736

TS 321

B3LYP/6-31G(d) = -3573.913764
B3LYP-D3(BJ)/def2-TZVPP/IEFPCM(propanonitrile) = -3575.485068
B3LYP-D3(BJ)/def2-TZVPP/IEFPCM(propanonitrile)//B3LYP-D3(BJ)/6-31G(d) Free Energy (Quasiharmonic) = -3574.768814

Frequencies (Top 3 out of 300)

1. -193.7803 cm^{-1}
2. 5.7411 cm^{-1}
3. 8.6709 cm^{-1}

B3LYP/6-31G(d) Molecular Geometry in Cartesian Coordinates

B	0.276494	-1.176028	-0.255343
O	0.275592	-2.601811	0.120035
N	0.940956	-1.220767	-1.665814
C	1.313833	-2.616834	-2.004945
S	0.700592	-0.201063	-2.957560
C	0.825440	-3.409675	-0.794173
O	0.938497	-4.606767	-0.657102
H	3.074786	-2.402922	-3.213524
H	0.754765	-2.974452	-2.879129
C	2.810560	-2.911087	-2.284013
C	3.778504	-2.531115	-1.195519
C	4.876926	-1.600359	-1.310949
C	5.514643	-1.535452	-0.039635
N	4.857883	-2.417945	0.790949
C	3.814067	-3.001095	0.096564
H	3.186094	-3.738896	0.576042
C	7.089086	0.062585	-0.851257
C	5.394112	-0.824049	-2.365071
H	7.080063	-0.664371	1.186577
H	4.959348	-2.449937	1.794510
C	6.488511	-0.000695	-2.126203
H	7.943461	0.715673	-0.694363
H	4.939232	-0.860854	-3.351781
C	6.613076	-0.704826	0.205829
H	6.893405	0.601328	-2.935397
C	1.381500	1.380521	-2.461140
O	1.486747	-0.734665	-4.080049

O	-0.742481	0.030364	-3.194979
C	2.480546	3.888060	-1.868264
C	2.690912	1.463826	-1.982540
C	0.619691	2.532380	-2.663036
C	1.174402	3.776255	-2.361351
C	3.227632	2.713219	-1.687943
H	3.279298	0.567569	-1.826054
H	-0.388628	2.449199	-3.052525
H	0.581111	4.674551	-2.513755
H	4.244741	2.772392	-1.309384
C	3.064266	5.231681	-1.505877
H	3.057975	5.366823	-0.417906
H	4.104357	5.318794	-1.839132
H	2.493160	6.052503	-1.950920
H	2.857234	-3.989426	-2.484155
C	0.975126	-0.290134	0.907108
C	1.601421	-0.958103	1.969826
C	1.003191	1.113769	0.939178
C	2.245041	-0.268948	3.002520
C	1.639458	1.809085	1.971682
H	0.529485	1.683886	0.147643
C	2.269867	1.122566	3.010992
H	2.756795	1.663253	3.814748
C	1.719275	3.309024	1.964150
C	2.952361	-1.039699	4.081000
F	1.528034	3.833176	3.196729
F	2.932292	3.750331	1.545241
F	0.800091	3.877686	1.143221
F	4.215123	-1.404587	3.700496
F	3.096303	-0.323956	5.215987
F	2.310910	-2.183814	4.396699
O	-1.239359	-0.751124	-0.230842
C	-2.138567	-1.432786	-0.875967
C	-3.050259	-2.312011	-0.132544
C	-3.778909	-3.294681	-0.823581
C	-3.092251	-2.276893	1.271940
C	-4.526935	-4.236326	-0.122292
H	-3.734518	-3.333860	-1.909312
C	-3.850774	-3.213124	1.968840
H	-2.495727	-1.538810	1.797195
C	-4.565524	-4.195608	1.274968
H	-5.069098	-5.007944	-0.661120
H	-3.866072	-3.195534	3.054912
H	-5.140895	-4.936665	1.822961
H	-1.894754	-1.722477	-1.900518
C	-3.491137	0.007193	-1.873294
H	-2.626288	0.547858	-2.233769
H	-3.896816	-0.756333	-2.528741
C	-4.396405	0.629401	-1.031026
C	-4.093443	1.885633	-0.314671
C	-2.808009	2.125462	0.199962
C	-5.077291	2.883380	-0.192134
C	-2.527132	3.325222	0.851395
H	-2.043047	1.361079	0.113278
C	-4.785788	4.087864	0.442531
H	-6.054389	2.732319	-0.636446
C	-3.512486	4.307572	0.973342
H	-1.533384	3.498768	1.249215
H	-5.548672	4.858133	0.514136
H	-3.284958	5.246235	1.470914
O	-5.608708	0.090228	-0.916033
Si	-6.896637	-0.062760	0.222388
C	-6.264202	0.166327	1.975601
H	-5.460450	-0.542860	2.201041
H	-5.889294	1.178352	2.156122
H	-7.081024	-0.020365	2.684592
C	-7.532885	-1.799456	-0.082222
H	-8.397733	-2.009122	0.559510
H	-7.854651	-1.920258	-1.123066
H	-6.766917	-2.552938	0.123921
H	1.573742	-2.041162	1.995987
C	-8.251474	1.169877	-0.220253
H	-8.057579	2.182516	0.146496
H	-8.404580	1.221102	-1.304566
H	-9.197407	0.836621	0.225299

TS 322

B3LYP/6-31G(d) = -3573.915485
B3LYP-D3(BJ)/def2-TZVPP/IEFPCM(propanonitrile) = -3575.485776
B3LYP-D3(BJ)/def2-TZVPP/IEFPCM(propanonitrile)//B3LYP-D3(BJ)/6-31G(d) Free Energy (Quasiharmonic) = -3574.768394

Frequencies (Top 3 out of 300)

1. -307.5219 cm^{-1}
2. 10.5049 cm^{-1}
3. 15.2078 cm^{-1}

B3LYP/6-31G(d) Molecular Geometry in Cartesian Coordinates

B	0.158249	-0.489890	-0.197085
O	0.162529	0.596528	-1.217616
N	0.336755	-1.738621	-1.094338
C	0.502122	-1.351804	-2.503262
S	0.806390	-3.244304	-0.612487
C	0.473504	0.173131	-2.437340
O	0.701618	0.913767	-3.376788
H	-0.373052	-2.968682	-3.618368
H	1.492099	-1.638560	-2.886043
C	-0.566004	-1.900358	-3.495001
C	-1.988582	-1.687660	-3.069620
C	-2.839610	-0.548058	-3.323979
C	-4.081533	-0.801311	-2.675006
N	-3.989246	-2.043319	-2.079575
C	-2.726279	-2.556197	-2.303253
H	-2.437358	-3.518957	-1.904347
C	-4.957951	1.288088	-3.420872
C	-2.683367	0.654790	-4.037208
H	-6.083904	-0.105239	-2.207632
H	-4.635070	-2.404625	-1.393363
C	-3.739675	1.557775	-4.078160
H	-5.764937	2.014504	-3.469589
H	-1.743404	0.881677	-4.532237
C	-5.144931	0.106350	-2.712950
H	-3.627608	2.489053	-4.627638
C	2.600627	-3.334302	-0.759416
O	0.277918	-4.227086	-1.571744
O	0.479879	-3.351615	0.816936
C	5.393742	-3.464889	-0.992411
C	3.177621	-3.754448	-1.959736
C	3.403112	-2.999928	0.333829
C	4.788476	-3.069785	0.210092
C	4.567107	-3.810961	-2.068789
H	2.544379	-4.066647	-2.783275
H	2.941063	-2.718710	1.273294
H	5.412526	-2.825722	1.066980
H	5.014357	-4.144535	-3.002145
C	6.897906	-3.508079	-1.122697
H	7.300259	-2.521253	-1.388565
H	7.213152	-4.208879	-1.902343
H	7.373314	-3.808821	-0.182898
H	-0.366786	-1.415769	-4.458798
C	-1.176041	-0.380510	0.708222
C	-2.043418	-1.456600	0.951539
C	-1.545584	0.860248	1.246842
C	-3.209438	-1.299130	1.707958
H	-1.799040	-2.436758	0.563543
C	-2.687358	1.016356	2.036752
H	-0.932706	1.729077	1.042837
C	-3.534924	-0.064718	2.270803

H	-4.421922	0.048174	2.883336
C	-3.014311	2.369720	2.599848
C	-4.152837	-2.457330	1.872587
F	-1.891109	3.067904	2.915794
F	-3.766450	2.293909	3.718145
F	-3.702235	3.143329	1.720710
F	-3.518503	-3.645126	1.873406
F	-5.067237	-2.511683	0.854114
F	-4.872699	-2.374741	3.012512
O	1.486054	-0.274373	0.558202
C	2.087196	0.208265	1.604268
C	1.894816	-0.344867	2.978696
C	0.859648	-1.233546	3.300639
C	2.847337	-0.022913	3.963538
C	0.768108	-1.762956	4.588530
H	0.155179	-1.541533	2.541447
C	2.752026	-0.550063	5.246404
H	3.676142	0.638087	3.716925
C	1.702752	-1.418556	5.564820
H	-0.033969	-2.457225	4.821382
H	3.496193	-0.292951	5.995084
H	1.625395	-1.833978	6.565840
H	3.135327	0.443273	1.391547
C	1.705139	2.200544	1.964264
H	0.740329	2.208088	2.460779
H	2.566671	2.297399	2.613429
C	1.795055	2.851637	0.725294
C	3.109127	3.078053	0.082061
C	3.312523	2.682432	-1.252279
C	4.177650	3.628148	0.812232
C	4.566539	2.841356	-1.839273
H	2.503625	2.223675	-1.810884
C	5.420858	3.805598	0.209696
H	4.022188	3.947286	1.838918
C	5.617728	3.408901	-1.115467
H	4.720810	2.517840	-2.864485
H	6.234737	4.252913	0.773076
H	6.590262	3.539763	-1.581841
O	0.695258	3.129982	0.080083
Si	0.031485	4.333914	-1.006713
C	0.287681	5.948953	-0.073530
H	1.351490	6.173114	0.068580
H	-0.151995	6.783315	-0.633765
H	-0.190778	5.920903	0.911798
C	-1.762552	3.842335	-1.151210
H	-2.273727	3.803810	-0.185201
H	-2.291776	4.569745	-1.780077
H	-1.860683	2.861930	-1.629462
C	0.854619	4.351090	-2.688920
H	1.896784	4.683172	-2.663527
H	0.804343	3.360218	-3.154157
H	0.297538	5.050149	-3.327518

TS 323

B3LYP/6-31G(d) = -3573.909947
B3LYP-D3(BJ)/def2-TZVPP/IEFPCM(propanonitrile) = -3575.483543
B3LYP-D3(BJ)/def2-TZVPP/IEFPCM(propanonitrile)//B3LYP-D3(BJ)/6-31G(d) Free Energy (Quasiharmonic) = -3574.767799

Frequencies (Top 3 out of 300)

1. -284.3199 cm^{-1}
2. 6.0964 cm^{-1}
3. 10.1252 cm^{-1}

B3LYP/6-31G(d) Molecular Geometry in Cartesian Coordinates

B	0.113883	-0.444643	-1.041049
O	0.185795	-1.536302	-2.021144
N	1.088937	0.621546	-1.660732
C	1.609449	0.109417	-2.943709
S	0.869958	2.259786	-1.514884
C	0.904825	-1.237633	-3.109024
O	1.024502	-1.971161	-4.064473
H	3.642283	0.862380	-2.920218
H	1.304714	0.759045	-3.772690
C	3.148304	-0.107679	-3.031206
C	3.727194	-1.089813	-2.044478
C	4.638431	-0.802578	-0.961300
C	4.884624	-2.025708	-0.277482
N	4.186702	-3.010598	-0.942737
C	3.489244	-2.444541	-1.992790
H	2.877443	-3.058656	-2.638334
C	6.332178	-0.937512	1.272450
C	5.290929	0.358742	-0.512470
H	5.867830	-3.049696	1.363708
H	4.053375	-3.949041	-0.599557
C	6.127920	0.281590	0.593566
H	6.985669	-0.964434	2.140536
H	5.144496	1.306242	-1.021509
C	5.715312	-2.107074	0.844994
H	6.633670	1.176961	0.946034
C	2.472764	3.000805	-1.159551
O	0.063311	2.488619	-0.299173
O	0.429122	2.838281	-2.795937
C	4.881880	4.311485	-0.578948
C	2.947501	3.022423	0.154131
C	3.182136	3.629139	-2.183780
C	4.385162	4.270590	-1.888740
C	4.144592	3.677527	0.432247
H	2.388597	2.543698	0.950777
H	2.781737	3.631737	-3.192002
H	4.938739	4.758532	-2.687472
H	4.513920	3.693793	1.454659
C	6.161327	5.046830	-0.256849
H	6.711721	4.557554	0.553690
H	5.953686	6.075551	0.066800
H	6.820615	5.105787	-1.128791
H	3.337087	-0.440029	-4.059788
C	0.523041	-0.935345	0.445910
C	0.950705	-2.252195	0.674548
C	0.547614	-0.060999	1.543129
C	1.386697	-2.676377	1.933782
H	0.956778	-2.953616	-0.153492
C	0.988488	-0.478168	2.802268
H	0.247611	0.973239	1.401393
C	1.410933	-1.789932	3.010670
H	1.752666	-2.112252	3.986544
C	0.989498	0.522484	3.923368
C	1.877121	-4.086291	2.106177
F	1.669088	1.647046	3.586595
F	1.549108	0.039895	5.053304
F	-0.268462	0.919111	4.239199
F	1.861163	-4.484369	3.395843
F	3.159758	-4.244108	1.670693
F	1.132033	-4.968743	1.399934
O	-1.300232	0.144863	-1.087022
C	-2.513659	-0.335723	-1.087060
C	-2.882352	-1.720867	-1.520807
C	-3.876853	-1.831655	-2.503104
C	-2.293824	-2.888517	-1.013633
C	-4.265724	-3.082892	-2.985858
H	-4.327781	-0.931680	-2.915584
C	-2.691546	-4.135405	-1.484750
H	-1.528596	-2.820537	-0.248920
C	-3.675484	-4.237517	-2.473374
H	-5.021884	-3.151558	-3.762961
H	-2.227213	-5.032179	-1.084761
H	-3.976162	-5.213810	-2.843145
H	-3.221270	0.407634	-1.461721

C	-3.098172	-0.309488	0.857945
H	-3.300035	-1.358535	1.041216
H	-2.169813	0.065767	1.269992
C	-4.218859	0.531088	0.923561
C	-4.121625	1.992444	1.011774
C	-2.953199	2.655153	0.585296
C	-5.177726	2.749071	1.558798
C	-2.861233	4.040919	0.680151
H	-2.115372	2.114618	0.158590
C	-5.070460	4.131246	1.666678
H	-6.057222	2.245905	1.943668
C	-3.915335	4.781418	1.219190
H	-1.954688	4.526222	0.333147
H	-5.882150	4.702493	2.108166
H	-3.836174	5.861976	1.301242
O	-5.410317	-0.044749	0.880701
Si	-6.942587	0.052734	0.089329
C	-6.835670	1.251693	-1.352913
H	-6.610293	2.273199	-1.031200
H	-6.070534	0.949233	-2.076991
H	-7.796471	1.272046	-1.882764
C	-8.219488	0.570481	1.368869
H	-8.183828	1.637314	1.611745
H	-9.225188	0.355966	0.985893
H	-8.097764	0.004424	2.299504
C	-7.272190	-1.706923	-0.464449
H	-8.235743	-1.774269	-0.984875
H	-6.493764	-2.072351	-1.142209
H	-7.311159	-2.385115	0.395658

TS 324

B3LYP/6-31G(d) = -3573.905388
B3LYP-D3(BJ)/def2-TZVPP/IEFPCM(propanonitrile) = -3575.482981
B3LYP-D3(BJ)/def2-TZVPP/IEFPCM(propanonitrile)//B3LYP-D3(BJ)/6-31G(d) Free Energy (Quasiharmonic) = -3574.767712

Frequencies (Top 3 out of 300)

1. -316.7456 cm^{-1}
2. 9.2185 cm^{-1}
3. 11.0821 cm^{-1}

B3LYP/6-31G(d) Molecular Geometry in Cartesian Coordinates

B	0.707177	0.488215	0.318629
O	0.459224	0.741077	1.769066
N	1.370869	1.825701	-0.094652
C	1.286093	2.810447	0.993352
S	2.430090	2.091593	-1.333499
C	0.682494	2.002170	2.143436
O	0.418799	2.438025	3.245117
H	0.889090	4.603649	-0.127478
H	2.291197	3.131775	1.304860
C	0.449759	4.093467	0.733262
C	-1.027275	3.893912	0.534134
C	-1.741100	3.695578	-0.706063
C	-3.122135	3.570268	-0.384400
N	-3.231471	3.675164	0.988452
C	-1.974591	3.883684	1.528537
H	-1.842715	3.994749	2.595712
C	-3.687932	3.313116	-2.687998
C	-1.351338	3.625373	-2.055398
H	-5.158439	3.303311	-1.098683
H	-4.095700	3.707232	1.505721
C	-2.325234	3.435507	-3.029048
H	-4.425449	3.170562	-3.473564
H	-0.303247	3.714761	-2.326770
C	-4.105689	3.379498	-1.362446
H	-2.033196	3.378260	-4.073972
C	4.085544	1.908067	-0.651648
O	2.311917	3.498229	-1.750736
O	2.224534	1.010529	-2.308562
C	6.609235	1.607883	0.532812
C	4.770101	3.032885	-0.188481
C	4.656523	0.635841	-0.549705
C	5.905883	0.494664	0.046027
C	6.026126	2.873527	0.399229
H	4.331826	4.017863	-0.308282
H	4.129352	-0.226977	-0.939252
H	6.335850	-0.498544	0.145642
H	6.560198	3.749801	0.758666
C	7.969260	1.436413	1.166516
H	7.959791	0.637330	1.916697
H	8.303485	2.356754	1.655276
H	8.723926	1.165275	0.416975
H	0.611818	4.731007	1.610412
C	1.531088	-0.899818	0.178132
C	2.348151	-1.298068	1.251930
C	1.522470	-1.735637	-0.948964
C	3.131105	-2.453526	1.194536
H	2.375896	-0.692286	2.150836
C	2.283119	-2.908086	-1.001834
H	0.933554	-1.469675	-1.816375
C	3.098760	-3.273869	0.067039
H	3.696626	-4.176740	0.022257
C	2.166593	-3.812155	-2.198969
C	4.079694	-2.777643	2.315027
F	3.252092	-4.605921	-2.345196
F	2.012745	-3.119251	-3.346735
F	1.092554	-4.635889	-2.094889
F	4.281129	-4.109568	2.441193
F	3.641972	-2.319318	3.506739
F	5.302444	-2.221085	2.107817
O	-0.685354	0.488560	-0.319068
C	-1.697869	-0.171247	-0.793815
C	-1.676320	-0.657740	-2.216943
C	-2.691855	-1.485143	-2.722934
C	-0.701163	-0.162539	-3.093883
C	-2.717841	-1.830848	-4.070813
H	-3.452984	-1.877979	-2.057031
C	-0.730217	-0.514200	-4.445027
H	0.075414	0.499790	-2.729471
C	-1.732552	-1.348075	-4.937791
H	-3.503278	-2.482048	-4.445069
H	0.038845	-0.127920	-5.107598
H	-1.749840	-1.622146	-5.989103
H	-2.654475	0.286721	-0.523839
C	-1.949729	-1.853415	0.331652
H	-1.957311	-2.577136	-0.474414
H	-1.033988	-1.818689	0.909909
C	-3.152875	-1.726090	1.043190
C	-3.225064	-1.187529	2.408608
C	-2.292297	-0.230533	2.844563
C	-4.191986	-1.664716	3.316500
C	-2.339391	0.260986	4.146768
H	-1.530686	0.144674	2.174121
C	-4.223518	-1.184739	4.622018
H	-4.877095	-2.448779	3.013831
C	-3.304454	-0.214243	5.037267
H	-1.604937	1.003477	4.446052
H	-4.956741	-1.576048	5.321702
H	-3.334841	0.157567	6.057859
O	-4.271162	-2.113170	0.440094
Si	-5.910121	-1.554453	0.296490
C	-6.268817	-1.668536	-1.541351
H	-5.650830	-0.980821	-2.127725
H	-6.100108	-2.682985	-1.920372
H	-7.319513	-1.415746	-1.731493
C	-6.050369	0.216906	0.905989
H	-5.336410	0.873496	0.394439

H	-7.058109	0.589836	0.681528
H	-5.888864	0.308376	1.983821
C	-7.014888	-2.757279	1.227952
H	-8.053320	-2.625952	0.898832
H	-6.734350	-3.795207	1.014543
H	-6.994964	-2.614707	2.312741

TS 325

B3LYP/6-31G(d) = -3573.904272
B3LYP-D3(BJ)/def2-TZVPP/IEFPCM(propanonitrile) = -3575.482883
B3LYP-D3(BJ)/def2-TZVPP/IEFPCM(propanonitrile)//B3LYP-D3(BJ)/6-31G(d) Free Energy (Quasiharmonic) = -3574.767611

Frequencies (Top 3 out of 300)

1. -289.9521 cm^{-1}
2. 5.3139 cm^{-1}
3. 10.3071 cm^{-1}

B3LYP/6-31G(d) Molecular Geometry in Cartesian Coordinates

B	-0.065927	-0.901965	-0.584310
O	0.490714	-0.284895	-1.815118
N	-1.431615	-1.472675	-1.111025
C	-1.535499	-1.239827	-2.562214
S	-2.066516	-2.891234	-0.509124
C	-0.225150	-0.538993	-2.911311
O	0.130452	-0.213079	-4.027684
H	-3.658370	-0.935729	-2.821273
H	-1.559635	-2.191313	-3.108146
C	-2.740799	-0.382071	-3.041825
C	-2.838354	1.005027	-2.459349
C	-3.929516	1.539412	-1.677161
C	-3.592597	2.878770	-1.336081
N	-2.372171	3.153877	-1.917887
C	-1.921078	2.022621	-2.574405
H	-0.977676	2.041883	-3.100910
C	-5.639484	3.150714	-0.143991
C	-5.165341	1.027007	-1.246102
H	-4.149104	4.710284	-0.310788
H	-1.784087	3.936594	-1.674520
C	-6.004679	1.832791	-0.486897
H	-6.315347	3.755383	0.455008
H	-5.461258	0.012469	-1.495923
C	-4.430572	3.691572	-0.565708
H	-6.960855	1.441875	-0.148631
C	-3.838874	-2.628272	-0.317336
O	-1.920849	-3.972992	-1.499882
O	-1.532075	-3.055960	0.850598
C	-6.603277	-2.366605	0.069515
C	-4.317945	-1.939180	0.799968
C	-4.722016	-3.191557	-1.238183
C	-6.096849	-3.050015	-1.043278
C	-5.692128	-1.813626	0.982534
H	-3.630098	-1.515754	1.523739
H	-4.332017	-3.751343	-2.081602
H	-6.785227	-3.490710	-1.760654
H	-6.064049	-1.276028	1.851275
C	-8.091530	-2.251597	0.301482
H	-8.434419	-2.991222	1.037160
H	-8.361461	-1.263074	0.689229
H	-8.655990	-2.420707	-0.621022
H	-2.658253	-0.338669	-4.135823
C	-0.235105	0.197239	0.587855
C	-0.792052	-0.117908	1.837350
C	0.095709	1.540721	0.357272
C	-1.037579	0.869992	2.794935
C	-0.132988	2.530143	1.319119
H	0.520874	1.821226	-0.599850
C	-0.709621	2.202851	2.546605
H	-0.913013	2.967779	3.286124
C	0.288062	3.943448	1.042242
C	-1.660240	0.465603	4.102655
F	-0.393468	4.845390	1.777621
F	0.116901	4.287863	-0.267649
F	1.607355	4.146519	1.302354
F	-2.768945	-0.291043	3.913671
F	-2.026069	1.529760	4.852495
F	-0.813068	-0.276092	4.854636
O	0.844861	-2.065377	-0.268086
C	2.087274	-2.430641	-0.076245
C	2.846643	-2.100309	1.176625
C	2.720834	-0.889158	1.870736
C	3.705828	-3.086151	1.684474
C	3.429022	-0.680310	3.053923
H	2.055901	-0.120100	1.499066
C	4.416479	-2.876640	2.866017
H	3.802664	-4.034069	1.159518
C	4.277289	-1.670870	3.556564
H	3.305383	0.255024	3.592925
H	5.062630	-3.659119	3.254710
H	4.814988	-1.508821	4.486978
H	2.204767	-3.492099	-0.318267
C	3.179776	-1.898774	-1.595704
H	2.382586	-1.704304	-2.302351
H	3.714165	-2.834482	-1.738049
C	4.028008	-0.813493	-1.281199
C	3.698461	0.594251	-1.520819
C	4.084082	1.577562	-0.585007
C	2.987935	0.981825	-2.672428
C	3.758733	2.912439	-0.793434
H	4.579420	1.276525	0.331535
C	2.698239	2.325921	-2.891958
H	2.656981	0.246333	-3.395256
C	3.074702	3.289904	-1.954612
H	4.014686	3.656621	-0.046391
H	2.157185	2.610801	-3.788987
H	2.821227	4.333819	-2.115667
O	5.128506	-1.107469	-0.621716
Si	6.806595	-0.683681	-0.559088
C	7.124372	0.114401	1.108509
H	6.615156	-0.436325	1.907246
H	8.199334	0.105812	1.327657
H	6.788991	1.156061	1.146799
C	7.226246	0.432117	-2.010172
H	8.310180	0.602309	-2.033617
H	6.946099	-0.024153	-2.966566
H	6.735951	1.408016	-1.944080
H	-1.056009	-1.149708	2.050250
C	7.662807	-2.347394	-0.688811
H	8.751622	-2.232906	-0.621658
H	7.345526	-3.014357	0.120418
H	7.438026	-2.842173	-1.640419

TS 326

B3LYP/6-31G(d) = -3573.911443
B3LYP-D3(BJ)/def2-TZVPP/IEFPCM(propanonitrile) = -3575.483865
B3LYP-D3(BJ)/def2-TZVPP/IEFPCM(propanonitrile)//B3LYP-D3(BJ)/6-31G(d) Free Energy (Quasiharmonic) = -3574.767287

Frequencies (Top 3 out of 300)

1. -315.3706 cm^{-1}
2. 9.0125 cm^{-1}
3. 11.2609 cm^{-1}

B3LYP/6-31G(d) Molecular Geometry in Cartesian Coordinates

B	0.051280	-0.422450	-0.257299
O	0.485630	0.487360	-1.353869
N	-0.055709	-1.770880	-1.013429
C	0.404230	-1.618840	-2.404719
S	-0.019429	-3.281730	-0.342989
C	0.804700	-0.147280	-2.473979
O	1.328270	0.392540	-3.432969
H	-0.892259	-3.007100	-3.415399
H	1.307641	-2.216230	-2.594279
C	-0.627229	-1.951550	-3.516689
C	-1.863180	-1.096421	-3.529729
C	-3.129450	-1.361441	-2.886959
C	-3.992740	-0.273051	-3.191809
N	-3.277390	0.606339	-3.979919
C	-2.004630	0.106319	-4.180489
H	-1.285050	0.648800	-4.778559
C	-5.766450	-1.262051	-1.944669
C	-3.621149	-2.414941	-2.094229
H	-5.955260	0.633429	-2.970959
H	-3.628470	1.472839	-4.355569
C	-4.929669	-2.353541	-1.631749
H	-6.784510	-1.243021	-1.564739
H	-2.981239	-3.254971	-1.840089
C	-5.311400	-0.208741	-2.728869
H	-5.315769	-3.155841	-1.009689
C	1.702181	-3.806300	-0.217839
O	-0.644779	-4.219460	-1.288639
O	-0.529409	-3.152920	1.029781
C	4.377371	-4.631029	-0.023999
C	2.422921	-3.550880	0.951101
C	2.297881	-4.485720	-1.282689
C	3.630981	-4.884120	-1.181539
C	3.750091	-3.963230	1.038631
H	1.937561	-3.055640	1.784071
H	1.713851	-4.725210	-2.164789
H	4.092901	-5.412570	-2.012159
H	4.307581	-3.772779	1.953121
C	5.807691	-5.101429	0.096461
H	6.273071	-5.227089	-0.886459
H	5.862171	-6.070429	0.610171
H	6.415241	-4.395679	0.673411
H	-0.087759	-1.837470	-4.464919
C	-1.254660	0.179430	0.474931
C	-1.343410	1.566770	0.653401
C	-2.315040	-0.591751	0.978051
C	-2.402400	2.160099	1.345981
H	-0.565410	2.200820	0.247571
C	-3.373860	-0.004811	1.676941
H	-2.301929	-1.667851	0.852981
C	-3.425960	1.375609	1.873061
H	-4.243290	1.825119	2.424501
C	-4.408200	-0.883551	2.325341
C	-2.426810	3.653049	1.492821
F	-3.988440	-1.308291	3.548891
F	-4.674599	-1.989131	1.603761
F	-5.578220	-0.233701	2.525651
F	-1.183080	4.153960	1.736421
F	-3.225960	4.066689	2.497241
F	-2.861571	4.277079	0.365341
O	1.301530	-0.506040	0.652061
C	1.901220	-0.001730	1.690771
C	1.426340	-0.249590	3.085801
C	0.182510	-0.834810	3.357221
C	2.289550	0.037500	4.159731
C	-0.209060	-1.082750	4.673021
H	-0.462750	-1.127600	2.543371
C	1.902040	-0.217380	5.470571
H	3.275680	0.455390	3.965721
C	0.642130	-0.767970	5.731871
H	-1.184460	-1.523820	4.857301
H	2.579930	0.008550	6.289081
H	0.335450	-0.961030	6.756231
H	2.990860	-0.060830	1.597391
C	2.002470	2.058330	1.705111
H	1.015270	2.381560	2.018841
H	2.760820	2.044570	2.477841
C	2.436250	2.473100	0.437491
C	3.832660	2.247870	0.000281
C	4.086200	1.637200	-1.241239
C	4.909620	2.580301	0.841181
C	5.398130	1.368921	-1.627269
H	3.259380	1.345420	-1.880589
C	6.218780	2.330631	0.436291
H	4.720860	3.065521	1.794601
C	6.464440	1.720861	-0.796519
H	5.585350	0.880861	-2.579279
H	7.046080	2.611631	1.081661
H	7.485570	1.518121	-1.107399
O	1.559780	2.942580	-0.408229
Si	1.361570	4.112570	-1.684539
C	-0.421490	3.905130	-2.202449
H	-0.603990	2.874000	-2.522419
H	-1.127080	4.145120	-1.402149
H	-0.635891	4.567850	-3.050709
C	2.504660	3.819010	-3.140799
H	2.315640	2.832550	-3.578109
H	2.282769	4.574550	-3.906599
H	3.566520	3.901000	-2.890789
C	1.693719	5.753120	-0.825849
H	1.029569	5.883750	0.035871
H	2.729579	5.829160	-0.474669
H	1.516729	6.588310	-1.514659

TS 327

B3LYP/6-31G(d) = -3573.91171
B3LYP-D3(BJ)/def2-TZVPP/IEFPCM(propanonitrile) = -3575.481313
B3LYP-D3(BJ)/def2-TZVPP/IEFPCM(propanonitrile)//B3LYP-D3(BJ)/6-31G(d) Free Energy (Quasiharmonic) = -3574.764942

Frequencies (Top 3 out of 300)

1. -321.5383 cm^{-1}
2. 11.4040 cm^{-1}
3. 13.7672 cm^{-1}

B3LYP/6-31G(d) Molecular Geometry in Cartesian Coordinates

B	0.077166	-0.684781	-0.201730
O	0.825855	0.084600	-1.234553
N	-1.173381	-1.164179	-1.013354
C	-0.995889	-0.807876	-2.434890
S	-1.879804	-2.633889	-0.660028
C	0.344473	-0.075877	-2.459595
O	0.907385	0.325078	-3.463116
H	-3.029784	-0.518277	-3.102772
H	-0.891898	-1.707837	-3.053871
C	-2.115947	0.081571	-3.049779
C	-2.389205	1.368689	-2.323988
C	-1.790868	2.665038	-2.555442
C	-2.349390	3.561870	-1.601142
N	-3.258058	2.846410	-0.847984
C	-3.261503	1.533412	-1.276477
H	-3.913103	0.808595	-0.809008
C	-1.054850	5.370333	-2.462003
C	-0.852587	3.161552	-3.478772
H	-2.424543	5.578683	-0.800068
H	-3.676280	3.157460	0.015805
C	-0.495727	4.503522	-3.423629
H	-0.753710	6.414517	-2.442545
H	-0.396467	2.498163	-4.207294

C	-1.988158	4.911842	-1.539578
H	0.229199	4.894418	-4.133019
C	-3.646303	-2.419093	-0.953701
O	-1.720482	-2.826177	0.789034
O	-1.439336	-3.671321	-1.607787
C	-6.402457	-2.126872	-1.394766
C	-4.203131	-2.840099	-2.161219
C	-4.455260	-1.878131	0.049378
C	-5.821574	-1.730734	-0.180306
C	-5.572941	-2.684882	-2.375800
H	-3.569346	-3.296449	-2.914232
H	-4.025154	-1.595023	1.004117
H	-6.449336	-1.308855	0.601195
H	-6.004358	-3.009888	-3.319528
C	-7.889347	-1.988178	-1.619754
H	-8.134077	-1.976865	-2.686525
H	-8.278195	-1.067491	-1.171527
H	-8.436221	-2.825413	-1.166167
H	-1.812603	0.284129	-4.084034
C	-0.223992	0.281951	1.055238
C	-1.519175	0.479247	1.552624
C	0.795288	1.048275	1.633651
C	-1.782953	1.385770	2.582408
H	-2.335422	-0.078626	1.114148
C	0.542980	1.952985	2.669204
H	1.808501	0.954782	1.259393
C	-0.750256	2.131098	3.155511
H	-0.949134	2.830655	3.958801
C	1.690113	2.761570	3.201314
C	-3.200205	1.614443	3.021488
F	2.772004	1.983834	3.467085
F	1.385370	3.427972	4.332661
F	2.112816	3.686039	2.296342
F	-3.284005	2.034133	4.300622
F	-3.819161	2.573734	2.266634
F	-3.961058	0.502202	2.906633
O	0.962343	-1.889436	0.055781
C	1.852724	-2.492052	0.789382
C	1.588049	-2.947643	2.189404
C	2.416223	-3.954942	2.715184
C	0.534069	-2.459758	2.971877
C	2.211733	-4.449767	3.998781
H	3.218824	-4.364938	2.104611
C	0.330406	-2.956940	4.259022
H	-0.143033	-1.723682	2.564145
C	1.167853	-3.944096	4.779176
H	2.856636	-5.233101	4.387358
H	-0.496266	-2.576583	4.852336
H	1.002333	-4.328346	5.782049
H	2.390217	-3.248130	0.207469
C	3.536687	-1.433716	1.083825
H	3.318813	-0.786261	1.928218
H	4.115336	-2.318260	1.322778
C	3.874441	-0.787396	-0.120439
C	4.376988	-1.537397	-1.287370
C	3.845217	-1.276255	-2.564567
C	5.333880	-2.557368	-1.130817
C	4.267280	-2.029329	-3.657738
H	3.071487	-0.525695	-2.693985
C	5.768453	-3.286421	-2.234307
H	5.763031	-2.752793	-0.152309
C	5.231909	-3.026834	-3.498375
H	3.832243	-1.838415	-4.634235
H	6.523388	-4.057180	-2.108700
H	5.562690	-3.605419	-4.356435
O	3.588867	0.481073	-0.242837
Si	4.227269	1.973752	-0.885288
C	2.726510	2.950898	-1.401082
H	2.134988	2.409890	-2.146085
H	2.077761	3.164747	-0.545693
H	3.034205	3.910471	-1.835293
C	5.426386	1.659026	-2.298647
H	6.220731	0.954258	-2.029941
H	4.929074	1.284623	-3.197807
H	5.906974	2.612259	-2.556151
C	5.135977	2.707730	0.587212
H	5.549409	3.689980	0.325782
H	4.465253	2.844849	1.440914
H	5.970897	2.072266	0.905654

TS 328

B3LYP/6-31G(d) = -3573.912419
B3LYP-D3(BJ)/def2-TZVPP/IEFPCM(propanonitrile) = -3575.481693
B3LYP-D3(BJ)/def2-TZVPP/IEFPCM(propanonitrile)//B3LYP-D3(BJ)/6-31G(d) Free Energy (Quasiharmonic) = -3574.764578

Frequencies (Top 3 out of 300)

1. -309.5803 cm^{-1}
2. 10.5587 cm^{-1}
3. 12.7936 cm^{-1}

B3LYP/6-31G(d) Molecular Geometry in Cartesian Coordinates

B	0.126931	-0.533439	-0.287285
O	0.794183	0.349874	-1.291559
N	-1.074586	-1.092316	-1.124967
C	-0.924506	-0.675164	-2.532315
S	-1.660947	-2.626666	-0.834233
C	0.360301	0.146760	-2.528670
O	0.913720	0.577240	-3.525373
H	-2.970533	-0.502183	-3.205192
H	-0.762822	-1.542098	-3.183937
C	-2.103790	0.160101	-3.115758
C	-2.480192	1.384542	-2.330503
C	-1.927455	2.717935	-2.431972
C	-2.587000	3.520374	-1.458335
N	-3.511488	2.717830	-0.820724
C	-3.425848	1.439052	-1.336630
H	-4.070268	0.655233	-0.963048
C	-1.320447	5.436967	-2.097988
C	-0.954238	3.318304	-3.252354
H	-2.805203	5.468249	-0.524535
H	-3.986181	2.948156	0.039297
C	-0.661582	4.665727	-3.077608
H	-1.067678	6.487890	-1.984002
H	-0.424275	2.730505	-3.995422
C	-2.291594	4.875516	-1.277351
H	0.089508	5.136109	-3.707046
C	-3.439977	-2.533878	-1.114109
O	-1.146419	-3.582541	-1.829660
O	-1.476436	-2.872467	0.603009
C	-6.213290	-2.432199	-1.530965
C	-4.278619	-2.087860	-0.089396
C	-3.973418	-2.958616	-2.331029
C	-5.352713	-2.901107	-2.532144
C	-5.654247	-2.036778	-0.306136
H	-3.862770	-1.808092	0.872390
H	-3.313731	-3.348537	-3.098985
H	-5.767030	-3.235829	-3.480150
H	-6.305594	-1.694955	0.494858
C	-7.702573	-2.343952	-1.765010
H	-8.032326	-3.059912	-2.524659
H	-8.265193	-2.538840	-0.845873
H	-7.987564	-1.342544	-2.114176
H	-1.809145	0.432676	-4.136690
C	-0.261270	0.322919	1.023993
C	-1.587362	0.446858	1.458630
C	0.708545	1.020506	1.756313
C	-1.927045	1.206916	2.581112

C	0.382913	1.766431	2.892572
H	1.742769	0.986090	1.435168
C	-0.941128	1.866119	3.317292
H	-1.197265	2.435794	4.202857
C	1.478355	2.482365	3.630372
C	-3.371032	1.358099	2.963858
F	1.835396	3.641480	3.017230
F	2.606876	1.731397	3.701555
F	1.127689	2.803394	4.893225
F	-4.101471	0.259152	2.663133
F	-3.967607	2.398108	2.305237
F	-3.534232	1.606014	4.279477
O	1.135273	-1.651730	-0.085937
C	1.990066	-2.299730	0.658115
C	1.666270	-2.892668	1.995614
C	0.613821	-2.463670	2.813502
C	2.467051	-3.965221	2.425970
C	0.381275	-3.087562	4.038876
H	-0.038917	-1.670178	2.481697
C	2.234222	-4.586504	3.648643
H	3.271808	-4.324029	1.786609
C	1.189380	-4.142665	4.463704
H	-0.444725	-2.751275	4.659159
H	2.857922	-5.419421	3.961349
H	1.000717	-4.625092	5.418950
H	2.576055	-2.997307	0.050412
C	3.574785	-1.215820	1.133017
H	4.200652	-2.055056	1.415284
H	3.240012	-0.594868	1.958667
C	3.940456	-0.546663	-0.051155
C	4.639237	-1.262190	-1.139216
C	5.770911	-2.050407	-0.862772
C	4.128894	-1.215157	-2.449442
C	6.400800	-2.754050	-1.886023
H	6.172677	-2.083260	0.145927
C	4.750360	-1.944299	-3.461377
H	3.230595	-0.645795	-2.662429
C	5.888792	-2.704940	-3.185326
H	7.287787	-3.342517	-1.669947
H	4.338640	-1.920795	-4.465960
H	6.373863	-3.264349	-3.980375
O	3.500158	0.665391	-0.235419
Si	3.961423	2.166067	-1.026129
C	2.652072	3.365418	-0.452048
H	2.594874	3.445171	0.637476
H	2.866717	4.364872	-0.851105
H	1.668652	3.063176	-0.826816
C	3.997772	2.052193	-2.893996
H	4.178121	3.063033	-3.284900
H	4.791266	1.402682	-3.275478
H	3.032916	1.709392	-3.284777
H	-2.365436	-0.056187	0.900574
C	5.665048	2.520062	-0.306000
H	6.040476	3.480104	-0.681180
H	5.634463	2.579562	0.787682
H	6.393564	1.750336	-0.586797

TS Me 1

B3LYP/6-31G(d) = -2708.11832
B3LYP-D3(BJ)/def2-TZVPP/IEFPCM(propanonitrile) = -2709.277536
B3LYP-D3(BJ)/def2-TZVPP/IEFPCM(propanonitrile)//B3LYP-D3(BJ)/6-31G(d) Free Energy (Quasiharmonic) = -2708.616475

Frequencies (Top 3 out of 261)

1. -282.1669 cm^{-1}
2. 10.5476 cm^{-1}
3. 15.9424 cm^{-1}

B3LYP/6-31G(d) Molecular Geometry in Cartesian Coordinates

B	1.407841	1.256010	-0.349681
O	1.054591	1.233680	-1.820061
N	2.147620	-0.127950	-0.245231
C	2.496580	-0.622410	-1.585641
S	3.075930	-0.575120	1.053869
C	1.673260	0.281070	-2.510921
O	1.582150	0.135560	-3.712961
H	2.832670	-2.720160	-1.220431
H	3.558280	-0.427470	-1.810941
C	2.240840	-2.116710	-1.911521
C	0.812160	-2.596150	-1.908991
C	0.128529	-3.354659	-0.882741
C	-1.166551	-3.673959	-1.382781
N	-1.270691	-3.103959	-2.637141
C	-0.073030	-2.484099	-2.954491
H	0.063400	-1.993519	-3.907921
C	-1.692361	-4.889389	0.602109
C	0.491269	-3.813400	0.398279
H	-3.062131	-4.687859	-1.062331
H	-2.023191	-3.265109	-3.288461
C	-0.419491	-4.574819	1.122699
H	-2.376901	-5.496639	1.188849
H	1.461839	-3.559800	0.813399
C	-2.084501	-4.440519	-0.655571
H	-0.146021	-4.935609	2.110559
C	4.733200	0.105889	0.835499
O	3.257480	-2.037780	1.008839
O	2.484520	0.061170	2.235059
C	7.313041	1.161169	0.499579
C	5.674250	-0.587941	0.069949
C	5.077341	1.308279	1.456099
C	6.360081	1.824209	1.285999
C	6.951270	-0.053591	-0.097331
H	5.420310	-1.552201	-0.356911
H	4.350571	1.816619	2.078899
H	6.628441	2.755689	1.779179
H	7.682620	-0.597761	-0.690371
C	8.689501	1.747838	0.293659
H	9.428051	0.970778	0.071139
H	8.695391	2.454338	-0.547201
H	9.028411	2.295968	1.179249
H	2.658890	-2.261730	-2.914391
O	0.114311	1.258531	0.449199
C	-0.676710	0.215521	0.534919
C	-1.471620	0.088941	1.787399
C	-1.963680	-1.167069	2.170219
C	-1.687279	1.198381	2.617119
C	-2.667310	-1.309539	3.366859
H	-1.763470	-2.039289	1.552969
C	-2.393549	1.052821	3.809029
H	-1.270909	2.158201	2.330449
C	-2.887220	-0.199609	4.186679
H	-3.024280	-2.291019	3.667359
H	-2.547209	1.914251	4.453529
H	-3.425340	-0.312829	5.124269
H	-0.269640	-0.727599	0.159469
C	-1.966490	0.305091	-0.945101
H	-1.226760	0.495661	-1.712741
H	-2.356300	-0.707019	-0.934851
C	-2.876609	1.329621	-0.638521
C	-2.604579	2.756131	-0.825591
C	-3.492909	3.712082	-0.285761
C	-1.472759	3.203091	-1.538471
C	-3.255958	5.069881	-0.452881
H	-4.356769	3.374032	0.273879
C	-1.245358	4.567401	-1.702501
H	-0.764149	2.502171	-1.962161
C	-2.131098	5.501821	-1.164221
H	-3.944268	5.794952	-0.027891

H	-0.369518	4.896671	-2.253261
H	-1.947758	6.564731	-1.296701
O	-4.005219	1.059942	0.000519
Si	-5.332860	-0.031388	-0.108551
C	-6.100020	0.063932	1.596009
H	-6.978530	-0.589148	1.664389
H	-6.422749	1.084452	1.830399
H	-5.383210	-0.247348	2.363379
C	-4.809280	-1.778918	-0.555661
H	-5.697400	-2.424688	-0.548661
H	-4.371570	-1.838588	-1.558251
H	-4.090180	-2.198198	0.154919
C	-6.448330	0.683012	-1.444221
H	-7.358520	0.079653	-1.550031
H	-6.753539	1.708072	-1.206471
H	-5.949110	0.700372	-2.420171
C	2.174571	2.608960	0.039469
H	1.565851	3.478190	-0.243301
H	2.355171	2.682460	1.117359
H	3.139101	2.708000	-0.474151

TS Me 2

B3LYP/6-31G(d) = -2708.114017
B3LYP-D3(BJ)/def2-TZVPP/IEFPCM(propanonitrile) = -2709.277697
B3LYP-D3(BJ)/def2-TZVPP/IEFPCM(propanonitrile)//B3LYP-D3(BJ)/6-31G(d) Free Energy (Quasiharmonic) = -2708.616253

Frequencies (Top 3 out of 261)

1. -277.7385 cm^{-1}
2. 17.4347 cm^{-1}
3. 19.1669 cm^{-1}

B3LYP/6-31G(d) Molecular Geometry in Cartesian Coordinates

B	-1.304920	-1.269469	-1.680060
O	-0.591270	-0.704520	-2.879090
N	-2.369520	-0.138159	-1.422300
C	-2.460050	0.711181	-2.634730
S	-3.876510	-0.459969	-0.750130
C	-1.193680	0.355091	-3.414200
O	-0.775650	0.960320	-4.380130
H	-3.520209	2.439401	-1.921700
H	-3.312500	0.402591	-3.260680
C	-2.579609	2.241461	-2.438650
C	-1.446139	2.928941	-1.725700
C	-1.400539	3.349881	-0.342690
C	-0.164449	4.027830	-0.142890
N	0.520791	3.986600	-1.342890
C	-0.269449	3.354320	-2.292760
H	0.072161	3.239080	-3.311840
C	-0.720869	4.476820	2.134410
C	-2.297309	3.241321	0.737060
H	1.129781	5.125160	1.216190
H	1.347201	4.524500	-1.554110
C	-1.948629	3.804221	1.960390
H	-0.483289	4.917210	3.099310
H	-3.244199	2.724551	0.607280
C	0.188121	4.596710	1.086740
H	-2.637549	3.733261	2.797820
C	-3.485470	-1.250569	0.814130
O	-4.653730	-1.418529	-1.555350
O	-4.505110	0.840791	-0.445810
C	-3.048700	-2.447029	3.307230
C	-2.870640	-0.518029	1.832580
C	-3.905640	-2.560919	1.031450
C	-3.681121	-3.150909	2.276250
C	-2.649480	-1.122999	3.065490
H	-2.569840	0.510381	1.662920
H	-4.402160	-3.100569	0.232600
H	-4.004741	-4.174909	2.446600
H	-2.154990	-0.559539	3.853110
C	-2.806090	-3.083539	4.655380
H	-1.754190	-2.993069	4.952100
H	-3.403770	-2.598539	5.437760
H	-3.065931	-4.146599	4.650140
H	-2.667169	2.653741	-3.450330
O	-0.280790	-1.311460	-0.530710
C	0.234120	-0.226750	-0.008060
C	0.705750	-0.331830	1.401250
C	1.026650	-1.576810	1.961240
C	0.774600	0.821360	2.196040
C	1.419880	-1.663210	3.295110
H	0.938330	-2.467800	1.348610
C	1.162450	0.730310	3.534100
H	0.493581	1.785490	1.779540
C	1.489980	-0.511270	4.085510
H	1.662710	-2.631920	3.723920
H	1.192061	1.627330	4.146860
H	1.786380	-0.583350	5.128810
H	-0.273570	0.711200	-0.251690
C	1.812830	0.316930	-1.076740
H	1.929361	1.341490	-0.741000
H	1.320070	0.206410	-2.034440
C	2.853670	-0.568970	-0.763050
C	2.977390	-1.919010	-1.315780
C	2.160820	-2.354720	-2.380350
C	3.934030	-2.808601	-0.779580
C	2.305419	-3.643680	-2.887570
H	1.413990	-1.701880	-2.815720
C	4.068379	-4.092491	-1.291030
H	4.555300	-2.482151	0.045870
C	3.254669	-4.513381	-2.348690
H	1.670449	-3.965980	-3.707140
H	4.804939	-4.768991	-0.866870
H	3.362409	-5.517381	-2.750200
O	3.724870	-0.278261	0.193870
Si	4.715610	1.045769	0.674240
C	6.170460	1.040019	-0.516630
H	5.847441	1.225379	-1.547780
H	6.699420	0.080739	-0.499190
H	6.890551	1.823799	-0.250670
C	5.203120	0.583169	2.421770
H	5.856421	1.347199	2.860240
H	4.317240	0.487079	3.058960
H	5.741080	-0.371011	2.446190
C	3.821731	2.699009	0.616650
H	4.488881	3.472049	1.020380
H	3.557051	2.996859	-0.403770
H	2.909841	2.698300	1.222140
C	-1.764020	-2.782579	-1.932260
H	-2.169611	-3.263349	-1.035600
H	-2.529210	-2.850389	-2.713240
H	-0.902111	-3.383180	-2.252740

TS Me 3

B3LYP/6-31G(d) = -2708.119908
B3LYP-D3(BJ)/def2-TZVPP/IEFPCM(propanonitrile) = -2709.277025
B3LYP-D3(BJ)/def2-TZVPP/IEFPCM(propanonitrile)//B3LYP-D3(BJ)/6-31G(d) Free Energy (Quasiharmonic) = -2708.616165

Frequencies (Top 3 out of 261)

1. -280.5688 cm^{-1}
2. 11.5074 cm^{-1}
3. 14.2036 cm^{-1}

B3LYP/6-31G(d) Molecular Geometry in Cartesian Coordinates

B	-1.481465	-1.030851	-0.580040
O	-1.015431	-0.534476	-1.928216
N	-2.215403	0.261644	-0.069255
C	-2.426865	1.203901	-1.176045
S	-3.219897	0.298450	1.246917
C	-1.525356	0.641457	-2.280820
O	-1.306849	1.193889	-3.339608
H	-2.981172	3.098318	-0.321914
H	-3.458195	1.134791	-1.561985
C	-2.157267	2.710521	-0.924415
C	-0.861613	3.093111	-0.261582
C	0.411956	3.397255	-0.880280
C	1.295397	3.816941	0.156364
N	0.596232	3.735337	1.345186
C	-0.700078	3.323078	1.082872
H	-1.422756	3.200257	1.877036
C	3.049777	4.197126	-1.417314
C	0.888904	3.376197	-2.204495
H	3.260112	4.564914	0.706501
H	0.918637	4.081284	2.235820
C	2.197649	3.773223	-2.458003
H	4.061559	4.519310	-1.649508
H	0.244347	3.037585	-3.009707
C	2.610520	4.223157	-0.096038
H	2.569842	3.765436	-3.479038
C	-4.873513	-0.186632	0.711710
O	-3.355887	1.699077	1.688192
O	-2.734657	-0.724628	2.178865
C	-7.442325	-0.942892	-0.131339
C	-5.734472	0.767469	0.164026
C	-5.290815	-1.511218	0.860045
C	-6.566477	-1.878324	0.439099
C	-7.005949	0.381642	-0.260188
H	-5.423570	1.804627	0.098831
H	-4.623241	-2.235557	1.311977
H	-6.889855	-2.909887	0.558990
H	-7.673069	1.125936	-0.688660
C	-8.831805	-1.350116	-0.561626
H	-9.258899	-0.632760	-1.269578
H	-8.830489	-2.337112	-1.037217
H	-9.511536	-1.406957	0.298954
H	-2.222342	3.183393	-1.911573
O	-0.242353	-1.343075	0.250417
C	0.574944	-0.422849	0.700503
C	1.304857	-0.756524	1.954613
C	1.796111	0.273268	2.767374
C	1.455981	-2.089019	2.364154
C	2.434104	-0.023399	3.972141
H	1.656929	1.307686	2.462007
C	2.100509	-2.382967	3.563636
H	1.039815	-2.878439	1.747703
C	2.592529	-1.352717	4.369990
H	2.795266	0.781995	4.606547
H	2.206979	-3.417491	3.878855
H	3.084441	-1.584522	5.310953
H	0.218524	0.609695	0.637737
C	1.939155	-0.076568	-0.693798
H	2.341487	0.882367	-0.379651
H	1.217146	-0.015390	-1.498572
C	2.828602	-1.161647	-0.687890
C	2.529009	-2.474961	-1.260738
C	3.375903	-3.568323	-0.974511
C	1.414607	-2.675655	-2.101364
C	3.112345	-4.822943	-1.507869
H	4.227689	-3.418785	-0.321751
C	1.163028	-3.936865	-2.636356
H	0.745834	-1.859844	-2.346616
C	2.005115	-5.010275	-2.342772
H	3.767069	-5.657849	-1.274611
H	0.303722	-4.076675	-3.285161
H	1.802400	-5.991537	-2.763487
O	3.975204	-1.096191	-0.022706
Si	5.267668	0.053330	0.024225
C	5.438295	0.779341	-1.699835
H	6.334130	1.411014	-1.750067
H	5.542978	-0.003746	-2.459424
H	4.580133	1.405337	-1.968040
C	6.729736	-1.021478	0.502454
H	7.642216	-0.420173	0.595624
H	6.556690	-1.514292	1.466005
H	6.917425	-1.799820	-0.245576
C	4.951069	1.373019	1.317950
H	5.865950	1.960941	1.468642
H	4.160234	2.066193	1.012343
H	4.670174	0.930867	2.278987
C	-2.290409	-2.407965	-0.708546
H	-2.550188	-2.821481	0.272050
H	-3.216633	-2.296494	-1.286082
H	-1.676412	-3.160552	-1.220686

TS Me 4

B3LYP/6-31G(d) = -2708.119935
B3LYP-D3(BJ)/def2-TZVPP/IEFPCM(propanonitrile) = -2709.277033
B3LYP-D3(BJ)/def2-TZVPP/IEFPCM(propanonitrile)//B3LYP-D3(BJ)/6-31G(d) Free Energy (Quasiharmonic) = -2708.615943

Frequencies (Top 3 out of 261)

1. -280.5937 cm^{-1}
2. 11.4730 cm^{-1}
3. 14.2046 cm^{-1}

B3LYP/6-31G(d) Molecular Geometry in Cartesian Coordinates

B	-1.483600	-1.027780	-0.578250
O	-1.018050	-0.532590	-1.927060
N	-2.215320	0.265920	-0.067380
C	-2.427290	1.207500	-1.174660
S	-3.218450	0.304141	1.249780
C	-1.527310	0.643540	-2.279940
O	-1.309400	1.195080	-3.339320
H	-2.979440	3.103101	-0.321810
H	-3.459050	1.138911	-1.559550
C	-2.156170	2.714070	-0.924360
C	-0.859850	3.095720	-0.262310
C	0.413881	3.397790	-0.881670
C	1.298241	3.817050	0.154370
N	0.599401	3.737170	1.343490
C	-0.697530	3.326400	1.081920
H	-1.420090	3.205090	1.876440
C	3.052521	4.193929	-1.420220
C	0.890321	3.375220	-2.206050
H	3.264101	4.562899	0.703270
H	0.922581	4.083290	2.233780
C	2.199481	3.770400	-2.460310
H	4.064631	4.514669	-1.653000
H	0.245030	3.036910	-3.010800
C	2.613781	4.221419	-0.098800
H	2.571291	3.761449	-3.481470
C	-4.871440	-0.184569	0.716040
O	-3.355710	1.705501	1.688300
O	-2.730890	-0.716439	2.183260
C	-7.441580	-0.945369	-0.119040
C	-5.734360	0.767351	0.166560
C	-5.289390	-1.507289	0.874670
C	-6.567011	-1.876169	0.459750
C	-7.006950	0.379951	-0.251380
H	-5.425950	1.805301	0.102070
H	-4.623781	-2.227509	1.336030

H	-6.893621	-2.904809	0.594280
H	-7.677800	1.123832	-0.674830
C	-8.811590	-1.364598	-0.597090
H	-9.229121	-2.159708	0.029830
H	-9.513120	-0.523988	-0.592670
H	-8.769931	-1.749738	-1.624760
H	-2.221160	3.186250	-1.911860
O	-0.244310	-1.341390	0.251310
C	0.574160	-0.421970	0.700910
C	1.304400	-0.756220	1.954650
C	1.454559	-2.088820	2.364220
C	1.796950	0.273210	2.767090
C	2.099419	-2.383230	3.563410
H	1.037409	-2.877930	1.748040
C	2.435260	-0.023920	3.971570
H	1.658500	1.307730	2.461720
C	2.592730	-1.353341	4.369440
H	2.205149	-3.417820	3.878660
H	2.797420	0.781209	4.605740
H	3.084890	-1.585501	5.310180
H	0.218780	0.610930	0.638160
C	1.938040	-0.077320	-0.694270
H	2.341570	0.881220	-0.380470
H	1.215620	-0.015470	-1.498630
C	2.826270	-1.163381	-0.688670
C	2.524929	-2.476441	-1.261200
C	1.409789	-2.676060	-2.101100
C	3.370819	-3.570681	-0.975360
C	1.156519	-3.937070	-2.635760
H	0.741719	-1.859570	-2.346040
C	3.105589	-4.825081	-1.508390
H	4.223179	-3.421971	-0.323150
C	1.997639	-5.011340	-2.342570
H	0.296649	-4.076050	-3.284000
H	3.759579	-5.660661	-1.275440
H	1.793609	-5.992450	-2.763030
O	3.973280	-1.099131	-0.024060
Si	5.267010	0.048979	0.022270
C	4.952090	1.369539	1.315510
H	5.867660	1.956489	1.465840
H	4.162000	2.063499	1.009750
H	4.670840	0.928089	2.276770
C	5.438140	0.774129	-1.702100
H	4.580530	1.400839	-1.970430
H	6.334570	1.404929	-1.752710
H	5.541960	-0.009361	-2.461390
C	6.727940	-1.027281	0.500690
H	7.641100	-0.426962	0.593530
H	6.554470	-1.519551	1.464440
H	6.914669	-1.806101	-0.247080
C	-2.294811	-2.403670	-0.705530
H	-2.555691	-2.815839	0.275330
H	-3.220721	-2.291159	-1.283360
H	-1.681981	-3.157820	-1.216790

TS Me 5

B3LYP/6-31G(d) = -2708.119687
B3LYP-D3(BJ)/def2-TZVPP/IEFPCM(propanonitrile) = -2709.276809
B3LYP-D3(BJ)/def2-TZVPP/IEFPCM(propanonitrile)//B3LYP-D3(BJ)/6-31G(d) Free Energy (Quasiharmonic) = -2708.615642

Frequencies (Top 3 out of 261)

1. -264.4255 cm^{-1}
2. 14.6949 cm^{-1}
3. 18.7278 cm^{-1}

B3LYP/6-31G(d) Molecular Geometry in Cartesian Coordinates

B	-1.583848	0.468684	1.583603
O	-1.686896	-0.509650	2.705831
N	-2.043943	-0.447391	0.376260
C	-2.633572	-1.695691	0.896552
S	-2.587998	0.117609	-1.075984
C	-2.250854	-1.663148	2.382603
O	-2.446021	-2.583693	3.150566
H	-2.534233	-3.015586	-0.806059
H	-3.734066	-1.646171	0.856366
C	-2.225193	-3.036091	0.241023
C	-0.777268	-3.441444	0.333836
C	0.215482	-3.423483	-0.717096
C	1.402517	-4.011345	-0.189807
N	1.139362	-4.345907	1.122148
C	-0.167331	-4.012031	1.426534
H	-0.575933	-4.199764	2.409348
C	2.525071	-3.748469	-2.279929
C	0.214784	-2.985578	-2.055269
H	3.450276	-4.650242	-0.542672
H	1.780421	-4.801547	1.752685
C	1.364641	-3.153553	-2.820146
H	3.401838	-3.881828	-2.908300
H	-0.668559	-2.512893	-2.473773
C	2.561130	-4.181063	-0.957297
H	1.367228	-2.832669	-3.858770
C	-4.337387	0.533277	-0.942539
O	-2.523477	-0.971728	-2.065485
O	-1.860886	1.373571	-1.341395
C	-7.065379	1.159709	-0.734320
C	-4.727518	1.837627	-0.632155
C	-5.297179	-0.456087	-1.172958
C	-6.649956	-0.137476	-1.061918
C	-6.083373	2.140451	-0.533253
H	-3.976098	2.604531	-0.487909
H	-4.987802	-1.452190	-1.470044
H	-7.394566	-0.908377	-1.245196
H	-6.384810	3.159191	-0.301086
C	-8.531243	1.491888	-0.588529
H	-8.742030	2.521126	-0.897733
H	-9.156353	0.821365	-1.187033
H	-8.854343	1.393945	0.456436
H	-2.837910	-3.791455	0.746630
O	-0.098833	0.849672	1.491432
C	0.837103	0.027226	1.102067
[truncated: 765,839 more chars]
